# Divergently expressed RNA identification and interaction prediction of long non-coding RNA and mRNA involved in Hu sheep hair follicle

**DOI:** 10.1038/s41598-019-43854-8

**Published:** 2019-05-13

**Authors:** Xiaoyang Lv, Wen Gao, Chengyan Jin, Yue Wang, Weihao Chen, Lihong Wang, Shuangxia Zou, Shuixing Sheng, Ling Chen, Wei Sun

**Affiliations:** 1 College of Animal Science and Technology, Yangzhou University, Yangzhou, 225009 Jiangsu P.R. China; 2 Animal Science and Veterinary Medicine Bureau of Suzhou City, Suzhou, 215200 Jiangsu P.R. China; 3 Joint international research laboratory of agriculture and agri - product safety of Ministry of Education of China, Yangzhou, 225009 Jiangsu P.R. China

**Keywords:** Animal breeding, Long non-coding RNAs

## Abstract

Hair follicles are the basis of the formation of Hu sheep pattern. This study was to employ whole transcriptome sequencing to screen differentially expressed long non-coding RNAs (lncRNAs) between three wave patterns in lambskin. In this study, three groups of 2-day-old Hu sheep were selected from full-sib individuals that included small, medium, and large waves, and hair follicle tissues were collected from dorsal side of Hu sheep. LncRNA and mRNA expression profiles were analyzed by whole transcriptome sequencing technology. 33, 31, and 41 differentially expressed lncRNAs were selected between large and medium, medium and small, and large and small, respectively. 458, 481, and 498 differentially expressed mRNAs were found between large and medium, medium and small, and large and small, respectively, by RNA-seq analysis. qRT-PCR results of 16 randomly selected lncRNAs and mRNAs were similar to the sequencing results. Correlation analysis of lncRNA and mRNA expression showed that, several lncRNAs may be enriched for hair follicle such as Wnt, mTOR, Notch signaling pathways. Our results aid in excavation of mRNAs and lncRNAs in hair follicle, and providing a basis for future study on pattern formation mechanisms.

## Introduction

Hu sheep is famous for its white lambskin in the word, and the lambskin from 2-day-old Hu lambs is called soft gem in China. The quality of lambskin is affected by many factors, especially the type of pattern. Wave patterns in lambskin have three types, including small waves, medium waves, and large waves. The quality of small waves is excellent, while large wave is poor. The type of wave pattern was determined by fineness, density, and curvature of the hair follicles^1–4^. Therefore, wool is the basic element to form different wave pattern, and hair follicles are determined to hair growth. Hair follicle growth and development is regulated by various types of cell, and hair follicle cyclical development is affected by several molecules and regulatory pathways^5,6^, such as Wnt^7^, TGF-β^8,9^, MAPK^10^, and Notch^11^ are generally involved in the periodic growth and morphogenesis of hair follicles.

The long non-coding RNA(lncRNA) is a non-translated RNA longer than 200 bp. Although, litter is known regarding how hair follicle development is regulated by lncRNA. Yue *et al*.^12^ identified 15 significant differentially lncRNAs, and he found that XLOC005698 might be involved in hair follicle development, which could be compete for oar-miR-3955-5p. Si *et al*.^13^ found that LncRNA PlncRNA-1could regulate proliferation and differentiation of hair follicle stem cells through TGF-β1-mediated Wnt/β-catenin signal pathway. Furthermore, Zhu *et al*.^14^ successfully constructed the regulatory network of lncRNA-H19 in secondary hair follicle of Liaoning cashmere goat. In addition, they found that lncRNA-H19 has the highest level of expression at the anagen phase, which speculates that lncRNA-H19 is closely related to hair follicle growth and development of Liaoning cashmere goat.

However, there are currently very few reports on lncRNA in hair follicle growth and development, and no relevant lncRNA studies on wave pattern of lambskin. Therefore, the molecular mechanisms of wave patterns formation are unknown. Whole transcriptome sequencing was used To study the molecular mechanism of waves pattern formation in Hu sheep and screen the differentially expressed lncRNAs between three wave patterns, which participated in the hair follicle cell biological process of growth and apoptosis, proliferation and differentiation. This study can provide basis for the mechanism of hair follicle growth and development between different wave pattern.

## Results

### Whole transcriptome sequencing results

**Figure 1:**
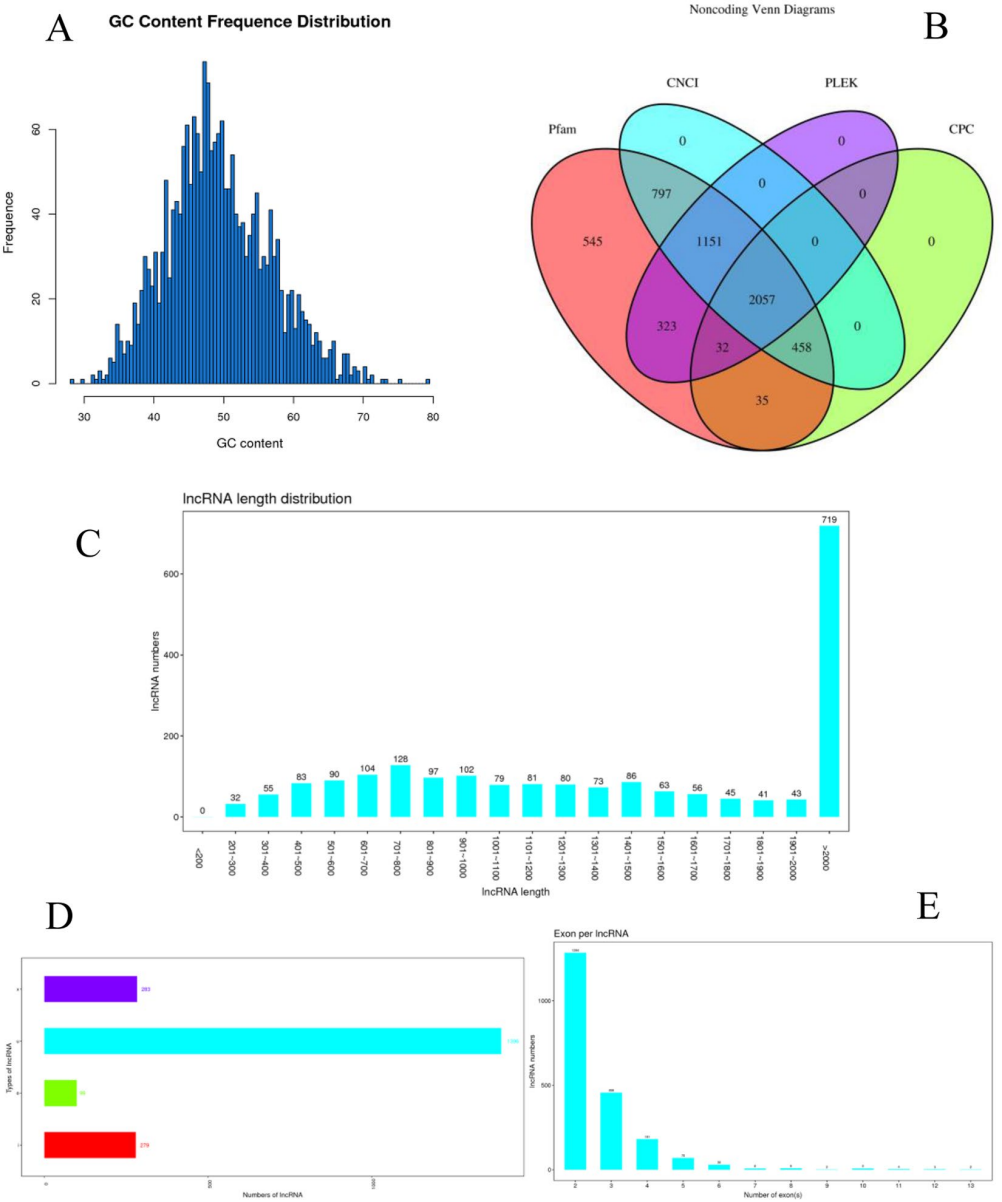
Summary of the predicted lncRNAs. (**A**) GC content frequence distribution. (**B**) Venn diagrams of coding ability prediction of candidate noncoding RNA. (**C**) lncRNA length distribution. (**D**) Number of different types of lncRNA E, number of exon per lncRNA.

Firstly, we extracted the total RNA from small waves, medium waves, and large waves, and then constructed the cDNA libraries for sequencing using the Illumina sequencer. Three pairs of full-sib individuals were divided to three groups, including Group 1(L1, M1, S1), Group 2(L2, M2,S2), and Group 3(L3, M3, S3). From the several cDNA libraries, the number of clean reads in Group 1(L1, M1, S1) were 96,961,910, 97,159,054, and 97,402,430 (Table 1), with GC contents of 52.00%, 52.50% and 52.00% (Fig. 1A), respectively. The number of clean reads in Group 2(L2, M2,S2) were 115,775,290, 97,345,832, and 96,934,824, with GC contents of 52.00%, 51.50% and 51.50%, respectively. The number of clean reads in Group 3(L3, M3, S3) were 98,295,984, 97,460,938, and 97,228,364, with GC contents of 51.50%, 52.00% and 52.50%, respectively. In addition, the valid reads in the clean reads were mapped to the Ovis aries genome.

**Table 1:** Summary of whole transcriptome sequencing.

Sample	Raw read	Raw bases	Clean reads	Clean bases	Valid bases	Q30	GC
L1	99343220	14901483000	96961910	14286474282	95.87%	93.90%	52.00%
L2	118892094	17833814100	115775290	16955209001	95.07%	93.35%	52.00%
L3	100483136	15072470400	98295984	14423345087	95.69%	93.93%	51.50%
M1	99281802	14892270300	97159054	14324796521	96.18%	93.99%	52.50%
M2	99632082	14944812300	97345832	14321674435	95.83%	93.75%	51.50%
M3	100087240	15013086000	97460938	14258549503	94.97%	93.44%	52.00%
S1	100693232	15103984800	97402430	14335981667	94.91%	92.95%	52.00%
S2	99413876	14912081400	96934824	14226115215	95.39%	93.46%	51.50%
S3	99730304	14959545600	97228364	14287606030	95.50%	93.49%	52.50%

In order to Identify the transcripts in sheep hair follicle, non-coding RNA candidates from the unknown transcripts were categorized using four coding potential analysis methods, namely, CPC^15^, CNCI^16^, Pfam^17^, and PLEK^18^ (Fig. 1B). After mapping the reference sequence, we identified 2,057 lncRNAs from transcripts. The length of the lncRNAs was mainly distributed within the range of 200 bp-5,000 bp, and the average length was 2,109.99 bp (Fig. 1C). Additionally, the lncRNA types mainly include intergenic lncRNAs (character u) and intronic lncRNAs (character i) (Fig. 1D), containing 2 to 3 exons (Fig. 1E).

#### Profiling and verification of differentially expressed lncRNA and mRNA of different wave patterns

**Figure 2:**
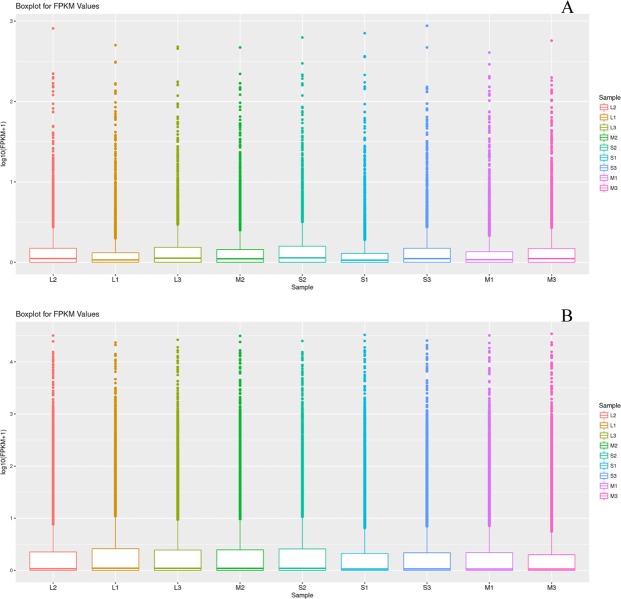
RPKM value of lncRNAs and mRNAs in sheep hair follicle. The boxplots in (**A**,**B**) show the expression levels of lncRNAs and mRNAs in the hair follicle between three groups, respectively.

**Figure 3:**
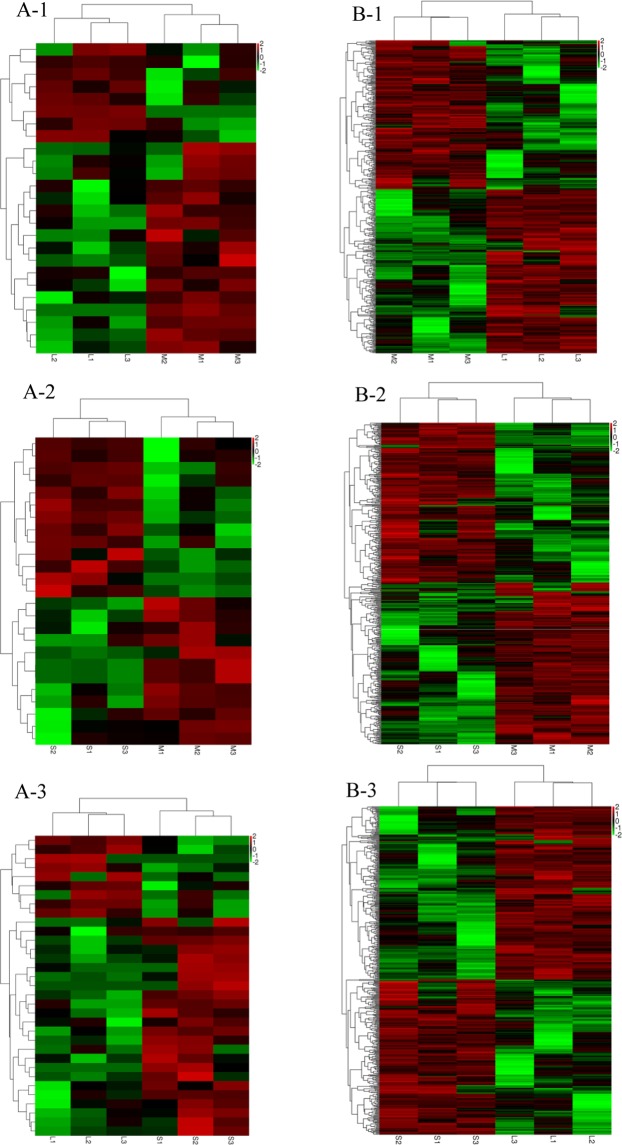
Differentially expressed lncRNAs and mRNAs between three wave patterns of Hu sheep lambskin. (**A-1–A-3**) Show 33, 31, and 41 differentially expressed lncRNAs between large and medium, medium and small, and large and small, respectively. (**B-1–B-3**) Show 458, 481, and 498 differentially expressed mRNAs between large and medium, medium and small, and large and small, respectively.

The expression levels of lncRNA and mRNA transcripts were estimated using the FPKM values (Reads Per Kilobase per Million mapped reads). We found that the expression level of the lncRNAs transcripts was relatively lower than those of the mRNA (Fig. 2A,B). Based on adjusted P-value threshold of <0.001 and |log2(fold change)| > 1, a total of 21, 16, and 29 upregulated and 12, 15, and 12 downregulated lncRNA, and 222, 239, and 236 upregulated and 236, 242, and 262 downregulated mRNA between large and medium waves, small and medium waves, and large and small waves, respectively (Fig. 3A,B).

**Figure 4:**
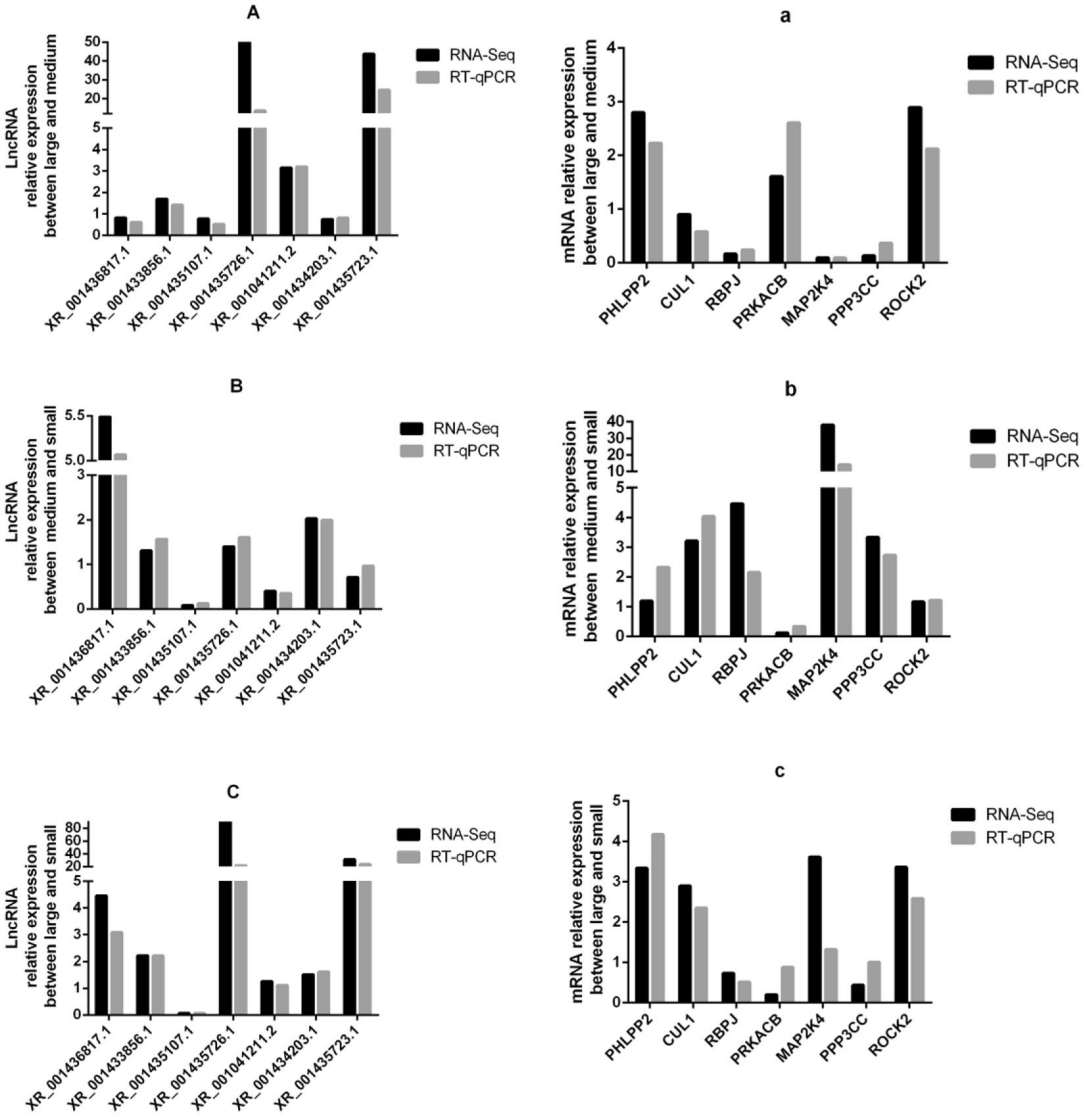
The comparisons on the results of sequencing and RT-qPCR analyses of differentially expressed lncRNAs and mRNAs. (**A**–**C**) Show differentially expressed lncRNAs between large and medium, medium and small, and large and small, respectively. (a–c) Show differentially expressed mRNAs between large and medium, medium and small, and large and small, respectively.

To further verify the reliability of whole transcriptome sequencing results, 7 differentially expressed lncRNAs and mRNAs were randomly selected for RT-PCR and their relative expression levels in the three wave patterns were confirmed. Compared with RNA-seq data, the expression trends were the same (Fig. 4), thus indicating that our RNA-seq data was reliable.

#### GO and pathway analysis

**Figure 5:**
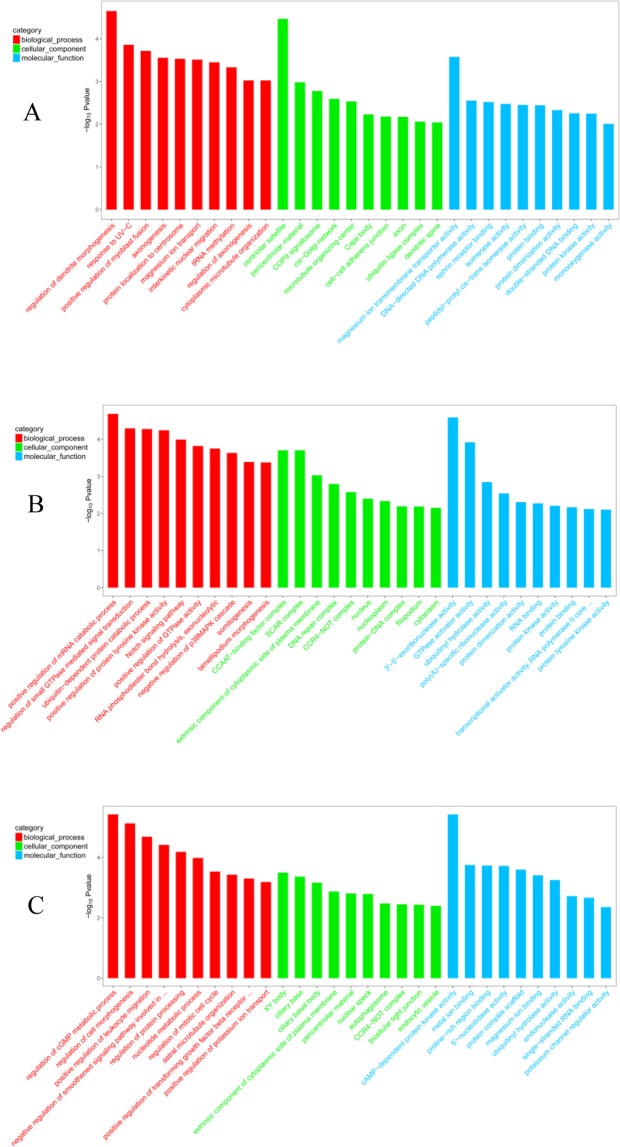
Gene Ontology enrichment analyses of differentially expressed mRNAs. (**A**) Large and medium waves. (**B**) Medium and small waves. (**C**) Large and small waves.

After obtaining differentially expressed mRNA, we performed gene ontology (GO) enrichment analysis on these mRNAs and described its function^19^. GO analysis showed that significant enrichment of 658, 704, and 682 GO items with these mRNAs were in medium and large waves, small and medium waves, and large and small waves, respectively. Between large and medium waves, a total of 87, 136, and 435 GO were enriched in cellular component(CC), molecular function(MF), and biological processes(BP), respectively. Between small and medium waves, 70, 129, and 505, GO were enriched in CC, MF, and BP, respectively. Between large and small waves, 74, 152, and 456 GO were enriched in CC, MF, and BP, respectively Fig. 5 showed part of GO items.

**Figure 6:**
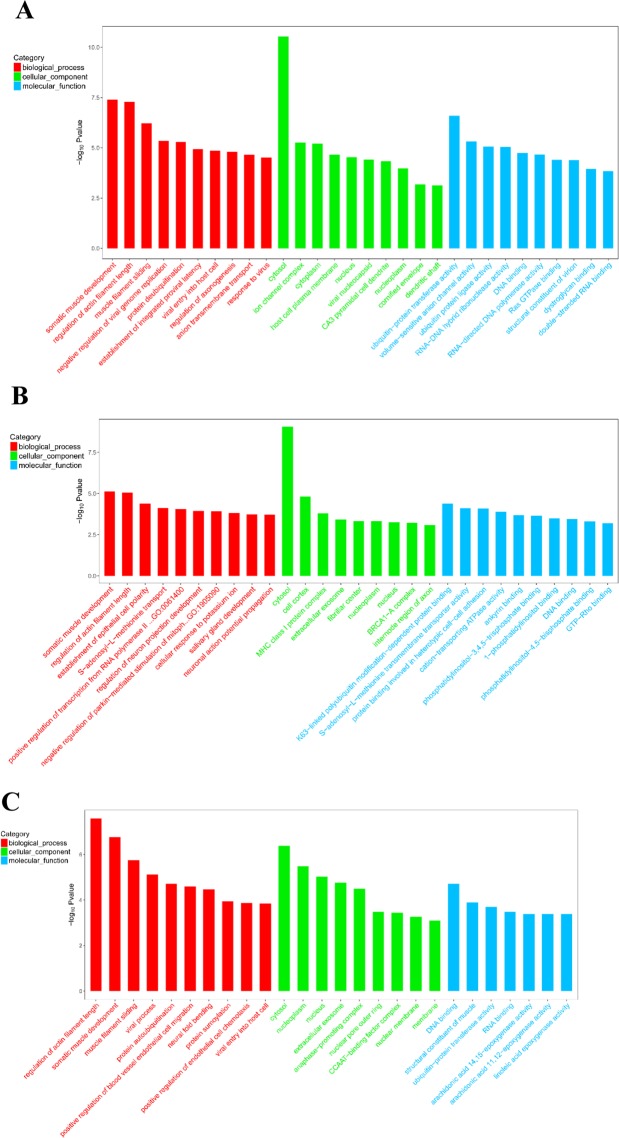
Gene Ontology enrichment analyses of differentially expressed lncRNAs. (**A**) Large and medium waves. (**B**) Medium and small waves. (**C**) Large and small waves.

GO function analysis of DE lncRNA showed that significant enrichment of 68, 73, and 119 GO items with these mRNAs were in small and medium waves, large and small waves and medium and large waves, respectively. Between large and medium waves, a total of 26, 29, and 63 GO were enriched in cellular component(CC), molecular function(MF), and biological processes(BP), respectively. Between small and medium waves, 18, 12, and 26, GO were enriched in CC, MF, and BP, respectively. Between large and small waves, 25, 13, and 34 GO were enriched in CC, MF, and BP, respectively. Figure 6 showed part of GO items.

**Figure 7:**
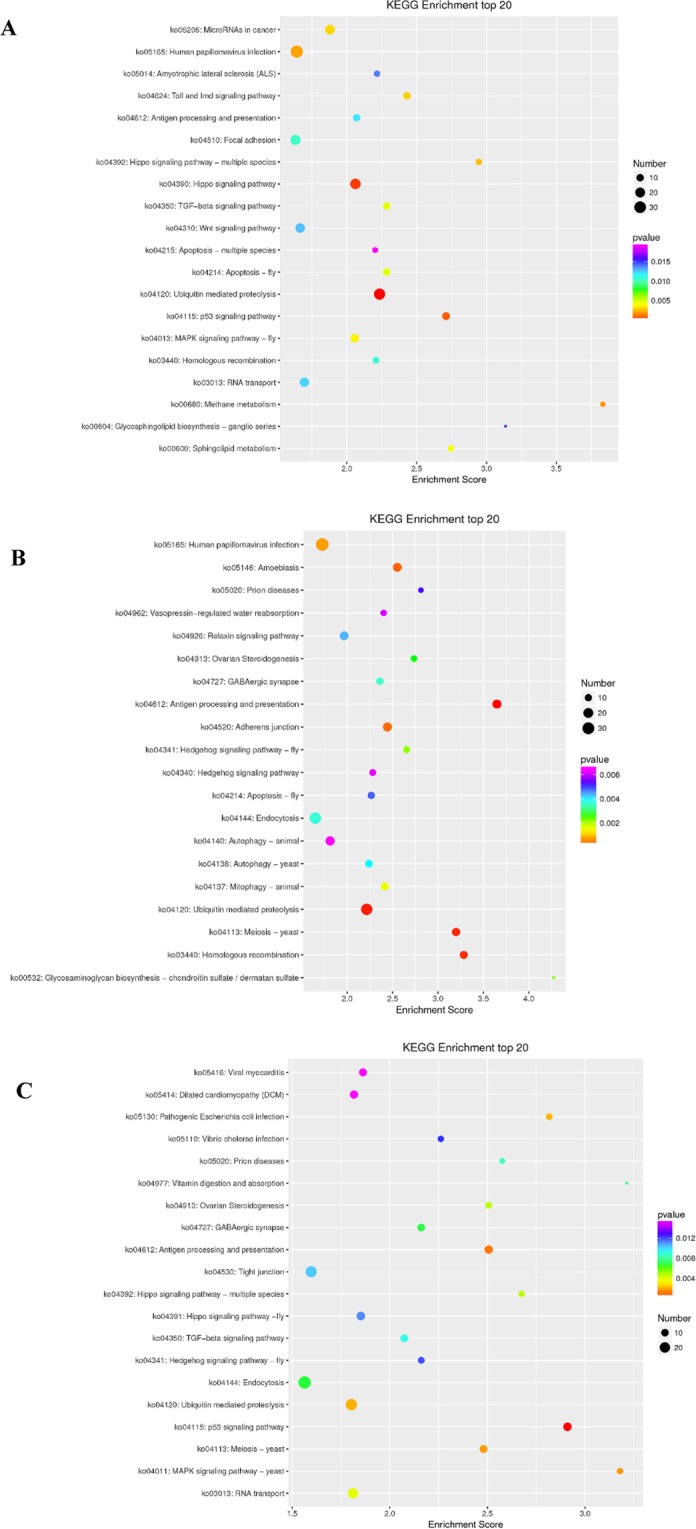
KEGG enrichment analyses of differentially expressed mRNAs. (**A**) Large and medium waves. (**B**) Medium and small waves. (**C**) Large and small waves.

Comparison between the DE mRNAs with the KEGG Pathway database showed that 287, 290, and 294 pathways in medium and large waves, small and medium waves, and large and small waves, respectively^20^. We found Wnt, Hippo, TGF-β, and Notch signaling pathway, which were related to hair follicle growth and development. In addition, some differentially expressed mRNAs were enriched in these pathways. Figure 7 showed part of KEGG items.

Comparison between the DE lncRNAs with the KEGG Pathway database indicated that a total of 4, 15, 3 KEGG pathways in medium and large waves, small and medium waves, and large and small waves, respectively (Table 2).

**Table 2:** KEGG enrichment analyses of differentially expressed lncRNAs.

	Term ID	Term_description	P_value
Large and medium waves	ko04120	Ubiquitin mediated proteolysis	2.33E-05
	ko04390	Hippo signaling pathway	0.017
	ko04115	p53 signaling pathway	0.031
	ko03013	RNA transport	0.040
Medium and small waves	ko04612	Antigen processing and presentation	0.002
	ko04144	Endocytosis	0.005
	ko05165	Human papillomavirus infection	0.005
	ko04138	Autophagy - yeast	0.009
	ko03440	Homologous recombination	0.012
	ko05146	Amoebiasis	0.020
	ko04520	Adherens junction	0.020
	ko04137	Mitophagy - animal	0.024
	ko04341	Hedgehog signaling pathway - fly	0.025
	ko05110	Vibrio cholerae infection	0.039
	ko00532	Glycosaminoglycan biosynthesis - chondroitin sulfate/dermatan sulfate	0.045
	ko04140	Autophagy - animal	0.045
	ko05020	Prion diseases	0.046
	ko04962	Vasopressin-regulated water reabsorption	0.048
Large and small waves	ko04120	Ubiquitin mediated proteolysis	0.003
	ko04113	Meiosis - yeast	0.029
	ko03013	RNA transport	0.046

#### Co-expression analysis of differentially expressed lncRNA-mRNA

**Figure 8:**
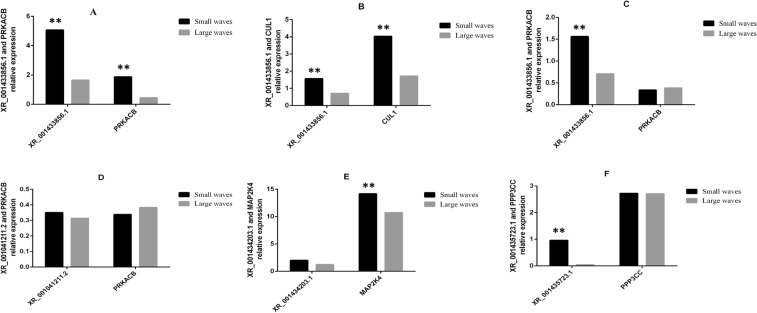
The relative expression on the co-expression analysis of lncRNAs and mRNAs.

In general, lncRNAs play a part of transcriptional or post-transcriptional regulation by silencing or suppressing some genes. In order to preferentially filter lncRNAs that regulate hair follicle development, correlation analysis of lncRNAs and mRNAs was done. Between large and medium waves, we found 25 differentially expressed lncRNAs were associated with 377 differentially expressed mRNAs. Between medium and small waves, we found 29 differentially expressed lncRNAs were associated with 435 differentially expressed mRNAs. Between large and small waves, we found 27 differentially expressed lncRNAs were associated with 333 differentially expressed mRNAs (Supplementary Table S1). The network pattern suggested that there were not one-to-one correspondence between lncRNAs and mRNAs. We chose some mRNAs that enriched in the pathways related to hair follicleand and co-expression lncRNAs to verify their expression. From the Fig. 8, XR_001436817.1 and *PHLPP2*, XR_001433856.1 and *CUL1*, and XR_001434203.1 and *MAP2K4* have the same expression trend. In contrast, XR_001433856.1 and *PRKACB*, XR_001041211.2 and *PRKACB* have opposite trend. Then, the expression of XR_001435723.1 in small waves higher than large waves, and *PPP3CC* has no changes. Therefore, we speculate that XR_001435723.1 and *PPP3CC* may not have relationship, and others may have some correlations. These results indicated that some lncRNAs and mRNAs might be have a close relationship, and they were screened from different patterns. So they could remaine the topics for future investigations.

## Discussion

Although there were many studies on the morphological changes of hair follicles, the specific molecular mechanisms were poorly understood. Nowadays, studies on the hair follicles mainly focused on human, mouse, and other models. More importantly, the molecular mechanisms of different types of hair follicle development are not exactly the same. For example, mouse acicular substrate formation is dependent on the Eda-A1/Edar/NF-kB pathway^21^, but conic hair substrate formation is not required for Eda -A1/Edar/NF-kB pathway but the Noggin/Lef-1 pathway^22^. Therefore, based on the molecular mechanism of hair follicle development in human, mouse, and other models, it is necessary to deeply study the molecular mechanism of hair follicle morphogenesis in Hu sheep due to the economic value of its lambskin. In order to screened differentially expressed mRNAs between three different patterns and investigate the possible role of lncRNA in hair follicle development., we collected hair follicle tissue from 9 two-day-old Hu lambs included three pairs of full-sib individuals with small, medium, large waves for high-throughput sequencing.

Due to the rapid development of transcriptome analysis, lncRNAs have received widespread attention over the past several years as novel regulators of cell development^23^. At present, the research on LncRNA mainly focuses on cancer in human^24–26^. The research on lncRNA mainly focuses on muscle growth, reproductive function, and so on in animal^27^. In the NONCODE database, there are only a few lncRNAs in sheep (antiPeg11, MEG3, MEG9, Rian, Xist)^28,29^. In our study, a total of 33, 31, 41 significant differentially expressed lncRNAs between medium and large waves, medium and small waves, and small and large medium. Although many lncRNAs have been screened in hair follicles by high-throughput sequencing, whether they are related to hair follicle development requires further analysis. At the same time, a total of 458, 481, 498 significant differentially expressed mRNAs between medium and large waves, medium and small waves, and small and large medium. In order to verify the RNA-seq results, we selected 7 lncRNA and mRNA for qRT-PCR, and the results were consistent with RNA-seq.

To further understand the function of differential expression mRNAs, GO and KEGG database were used to annotate their function. GO analysis indicated that some mRNAs such as *RBPJ* were enriched with regulation of hair cycle (GO:0042634), hair follicle development (GO:0001942), and hair follicle maturation (GO:0048820). Moreover, KEGG Pathway analysis showed that *PHLPP2*, *CUL1*, *MAP2K4*, *PPP3CC* enriched in Wnt(path:oas04310), Hippo(path:oas04390), MAPK(path:oas04010), mTOR(path:oas04150), Notch(path:oas04330) signaling pathways, and these mRNAs may be potentially involved in hair follicle growth and development process. Interestingly, we found that *RPBJ* found that it is involved in the biological process of hair follicle development, and *RPBJ* is in the Notch signaling pathway, which is one of the classic hair follicle development pathways. However, the mechanisms of these genes regulation in hair follicle growth and development are still unclear. The two *PHLPP*, *PHLPP1* and *PHLPP2*, have emerged as critical regulators of cellular homeostasis, and were identified in a search for phosphatases that dephosphorylate Akt, and thus suppress growth factor signaling^30^. *CUL1* is a scaffold protein of the ubiquitin E3 ligase Skp1/Cullin1/Rbx1/F-box protein complex, which increased in renal cell carcinoma and promotes cancer cell proliferation, migration, and invasion^31^. *MAP2K4* is a negative regulator of the TGF-β1 signaling associated with atrial remodeling and arrhythmogenesis^32^. So we speculate whether *MAP2K4* could regulate hair follicle growth and development by regulating the TGF-β1 pathway. PPP3CC decrease is responsible for activation of NF-κB and contributes to invasion and growth in glioma cells^33^. Subsequently, further researches will focus on the relationship between these genes and hair follicle growth and development.

Next, co-expression analysis of differentially expressed lncRNA-mRNA was performed to predict the correlation of lncRNAs and mRNAs by calculating the Pearson correlation coefficients. Analyzing the expression level of some lncRNA-mRNA, we found that XR_001436817.1 and PHLPP2, XR_001433856.1 and CUL1, and XR_001434203.1 and MAP2K4 have the same expression trend. These mRNAs were enriched in some pathways related to hair follicle growth and development, so we speculated that these co-expression lncRNAs might have some relationship with hair follicle development.

In conclusion, we screened significant differentially expressed lncRNAs and mRNAs in hair follicles of different waves of Hu sheep lambskin. Further research on these lncRNAs can provide a useful basis for the hair follicle development of lambskin, and our research may provide a certain basis to research the mechanism of the pattern formation in Hu sheep.

## Methods

### Ethics description

Animal research proposals were approved by the Chinese Ministry of Agriculture (License Number: 39) and the Jiangsu Provincial Government Animal Care and Use Committee (IACUC) (License Number: 45). All experimental procedures were carried out in strict accordance with the guidelines for the care and use of laboratory animals in Jiangsu Province and the recommendations of the Animal Protection and Use Committee of the Ministry of Agriculture of China. All efforts are made to minimize the suffering of animals.

### Sample collection

The experiment Hu sheep were selected from the Suzhou Stud Farm in Jiangsu Province, China. A total of 9 two-day-old healthy lambs showing approximately similar weight included three pairs of full-sib individuals with small, medium, large waves. 1 cm of hair root was cut off and placed in the freezing tube with Drikold until about 1/3 volume was collected from the dorsal side of the Hu sheep.

### RNA library construction

Total RNA was isolated for whole transcriptome sequencing,. The constructed RNA library was qualified by Agilent 2100 Bioanalyzer. A NanoDrop 2000 Ultra Microscope were utilized in determining the quality control of the extracted total RNAs. Ribosomal RNA was removed using a Ribo-Zero (TM) kit (Epicenter, Madison, WI, USA). Short fragments (approximately 200 bp in length) were obtained and used as templates for first-stand cDNA synthesis. Second-strand cDNA synthesis was performed using a buffer, dNTPs, RNase H, and DNA polymerase I. After the PCR amplification and purification using the Qubit® dsDNA HS Assay Kit, the cDNA library was constructed using an NEBNext® Ultra™ RNA Library Preparation Kit. The cDNA libraries were sequenced on the Illumina HiSeq 2500 platform in Shanghai OE Biomedical Technology Co (sequencing read length: 150 bp).

### Reads processing

The raw data was filtered to eliminate low-quality reads. Clean reads mapped to the reference genome (Ovis aries v4.0) were selected for de novo assembly. Coding RNA and non-coding RNA candidates from the unknown transcripts were categorized using four coding potential analysis methods, namely, CPC^15^, CNCI^16^, Pfam^17^, and PLEK^18^. The minimum length and the number of exons were set as thresholds, thereby filtering putative encoded RNAs, and transcripts containing two exons and longer than 200 nt were selected as candidate lncRNAs. Different types of lncRNAs were classified by cuffcompare, including intergenic lncRNAs (character u), intronic lncRNAs (character i), anti-sense lncRNAs (character x), and sense-overlapping lncRNAs (character o). After that, we used a reference transcript as a library and software bowtie2^34^ and eXpress^35^ to determine the abundance of each transcript in each sample by means of sequence similarity alignment. Then, we used FPKM method (Fragments Per kb Per Million Reads) to calculate mRNA and lncRNA expression. The number of counts of each lncRNA was normalized by DESeq. In addition, the difference multiple was calculated, and differential significance test was analyzed by NB (negative binomial distribution test) to screen differentially expressed lncRNAs.

### GO and KEGG pathway analyses

Functional annotation was performed using GO enrichment analysis and KEGG pathway analysis. We performed functional annotation on differentially expressed transcripts to cellular component(CC), molecular function(MF), and biological processes(BP) by the gene ontology database (http://geneontology.org/). Enrichment analysis employed counting the number of transcripts in each GO term was counted, followed by Fisher’s exact test to assess statistical significance (p < 0.05). KEGG (http://www.genome.jp/kegg/) is the main public database used in pathway analysis, which was followed by Fisher’s exact test to assess statistical significance (p < 0.05).

### Co-expression analysis of differentially expressed lncRNA-mRNA

Based on the expression values of mRNAs and lncRNAs, the correlation of lncRNAs were predicted by calculating the Pearson correlation coefficients and P values of the lncRNA-mRNA. The |correlation| ≥ 0.7 and *P* ≤ 0.05 were considered to be relevant^36^. We selected these lncRNA-mRNAs for follow-up studies.

### Sequencing results verification

We randomly selected 7 differentially expressed lncRNAs and mRNAs with GAPDH as a reference genes. In order to verify the reliability of the sequencing results, the SYBR Green I method was used. Relevant information on primers used in RT-PCR is shown in Table 3. Each sample was tested 3 times using a Fast 7500 PCR instrument to establish standard curve. To calculate the relative expression of the target genes, we used the following method^37^: Relative expression = 2^−ΔΔCt,^ ΔΔC t = (C, target gene—C, housekeeping gene) _large waves_ − (Ct, target gene—C, housekeeping gene)_small waves_. SPSS16.0 and one-way ANOVA were used to compute for relative expression and analysis of significance.

**Table 3:** Primers used in real-time RT-qPCR analysis.

lncRNA/Gene	Forward primer (5′-3′)	Reverse primer (5′-3′)
XR_001436817.1	GGAGGAAAGACCCGACCTTG	CTGGGACCACCGCCTAAAAT
XR_001433856.1	ACAACCCACCCAGACAATGG	TCTGACAACCCCTCGGGTAT
XR_001435107.1	TAGAAGAGGCCACGGAGCTA	ACCCCTCTGTGTTGTGACTC
XR_001435726.1	ACGAAGCAAGCCTCAGTTGT	GCTTATGGAGCCAGCCTTCT
XR_001041211.2	TGATGTGCAATACCCGGAGG	GCTGTGATTCCGGGCAGTTA
XR_001434203.1	AGGTGGGGAGAGATGACCAA	GCTAGATCACCACGGGACAG
XR_001435723.1	CCACCTCAACCCAGAACACA	GATTAGAGGCGAGCCACCAG
GAPDH	GTTCCACGGCACAGTCAAGG	ACTCAGCACCAGCATCACCC
PHLPP2	TTATGGCCGGAAACCGAGTG	GAGAGTTCGAAGCGAGAAGC
CUL1	AGTTTGTGGGCCTGGAATTGT	CCTTCATCACATTCGCCGTG
RBPJ	CTTACACTGACTTGAGTGCGG	GCACTTGCAGGTAATGGGGTT
PRKACB	TTCCCGTCATCCGTGTGTG	AATGAAACAGGACGGAATGGATG
MAP2K4	CCGTGAACGTGGATGTCAGTA	AAGCCTGACGAAACACCCTT
PPP3CC	GCCATCAGAGGGTTTTCGCT	AAGTACTGGCTATCTTTTCGGGG
ROCK2	TACGCCTTGGAAGAAATGGAGTA	AGTTCAGGTACCACAGGAGCA

## Supplementary information

Co-expression analysis of lncRNAs and mRNAs

## Data Availability

Raw sequencing data used as part of my study should be deposited in SRA. SRA Accession: PRJNA531734.
