# Supplementary material for: Divergently expressed RNA identification and interaction prediction of long non-coding RNA and mRNA involved in Hu sheep hair follicle
Source: Sci Rep. 2019 May 13;9:7283. doi: 10.1038/s41598-019-43854-8 (PMC6514014; doi:10.1038/s41598-019-43854-8)
Supplement: Supplementary file 1 — Co-expression analysis of lncRNAs and mRNAs [file 41598_2019_43854_MOESM1_ESM.pdf]

1) The title of the manuscript

Divergently expressed RNA identification and interaction prediction of long non-coding RNA and mRNA involved in Hu sheep hair follicle

2) The author list

Corresponding Author: **Wei Sun**
Company/ Institution: College of Animal Science and Technology, Yangzhou University
Address: 48 Wenhui East Road, Hanjiang District, Yangzhou 225009, Jiangsu, P. R. China.
Joint international research laboratory of agriculture and agri - product safety of Ministry of Education of China
Address: 460 Wenhcang Middle Road, Guangling District, Yangzhou 225009, Jiangsu, P. R. China.
Contributing Author: **Xiaoyang Lv**
Company/ Institution: College of Animal Science and Technology, Yangzhou University
Address: 48 Wenhui East Road, Hanjiang District, Yangzhou 225009, Jiangsu, P. R. China.
Contributing Author: **Wen Gao**
Company/ Institution: College of Animal Science and Technology, Yangzhou University
Address: 48 Wenhui East Road, Hanjiang District, Yangzhou 225009, Jiangsu, P. R. China.
Contributing Author: **Chengyan Jin**
Company/ Institution: College of Animal Science and Technology, Yangzhou University
Address: 48 Wenhui East Road, Hanjiang District, Yangzhou 225009, Jiangsu, P. R. China.
Contributing Author: **Yue Wang**
Company/ Institution: College of Animal Science and Technology, Yangzhou University
Address: 48 Wenhui East Road, Hanjiang District, Yangzhou 225009, Jiangsu, P. R. China.
Contributing Author: **Weihao Chen**
Company/ Institution: College of Animal Science and Technology, Yangzhou University
Address: 48 Wenhui East Road, Hanjiang District, Yangzhou 225009, Jiangsu, P. R. China.
Contributing Author: **Lihong Wang**
Company/ Institution: College of Animal Science and Technology, Yangzhou University
Address: 48 Wenhui East Road, Hanjiang District, Yangzhou 225009, Jiangsu, P. R. China.
Contributing Author: **Shuangxia Zou**
Company/ Institution: College of Animal Science and Technology, Yangzhou University
Address: 48 Wenhui East Road, Hanjiang District, Yangzhou 225009, Jiangsu, P. R. China.
Contributing Author: **Shuixing Sheng**
Company/ Institution: Animal Science and Veterinary Medicine Bureau of Suzhou City
Address: Qingyang Road, Wujiang District, Suzhou 215200, Jiangsu, P. R. China.
Contributing Author: **Ling Chen**
Company/ Institution: Animal Science and Veterinary Medicine Bureau of Suzhou City
Address: Qingyang Road, Wujiang District, Suzhou 215200, Jiangsu, P. R. China.

Supplementary S1 Table Co-expression analysis of lncRNAs and mRNAs

Medium and small waves

mRNA	Genesymbol	type	lncRNA	type
XM_015103461.1	TACC2	mRNA	XR_001436817.1	lncRNA
XM_015103461.1	TACC2	mRNA	XR_001436817.1	lncRNA
XM_015103461.1	TACC2	mRNA	XR_001436817.1	lncRNA
XM_015103461.1	TACC2	mRNA	XR_001436817.1	lncRNA
XM_015103461.1	TACC2	mRNA	XR_001436817.1	lncRNA
XM_015103461.1	TACC2	mRNA	XR_001436817.1	lncRNA
XM_015103461.1	TACC2	mRNA	XR_001436817.1	lncRNA
XM_015103461.1	TACC2	mRNA	XR_001436817.1	lncRNA
XM_015103461.1	TACC2	mRNA	XR_001436817.1	lncRNA
XM_015103461.1	TACC2	mRNA	XR_001436817.1	lncRNA
XM_015103461.1	TACC2	mRNA	XR_001436817.1	lncRNA
XM_015103461.1	TACC2	mRNA	XR_001436817.1	lncRNA
XM_015103461.1	TACC2	mRNA	XR_001436817.1	lncRNA
XM_015103461.1	TACC2	mRNA	XR_001436817.1	lncRNA
XM_015103461.1	TACC2	mRNA	XR_001436817.1	lncRNA
XM_015103461.1	TACC2	mRNA	XR_001436817.1	lncRNA
XM_015103461.1	TACC2	mRNA	XR_001436817.1	lncRNA
XM_015103461.1	TACC2	mRNA	XR_001436817.1	lncRNA
XM_015103461.1	TACC2	mRNA	XR_001436817.1	lncRNA
XM_015103461.1	TACC2	mRNA	XR_001434203.1	lncRNA
XM_015103461.1	TACC2	mRNA	XR_001434203.1	lncRNA
XM_015103461.1	TACC2	mRNA	XR_001434203.1	lncRNA
XM_015103461.1	TACC2	mRNA	XR_001434203.1	lncRNA
XM_015103461.1	TACC2	mRNA	XR_001434203.1	lncRNA
XM_015103461.1	TACC2	mRNA	XR_001434203.1	lncRNA
XM_015103461.1	TACC2	mRNA	XR_001434203.1	lncRNA
XM_015103461.1	TACC2	mRNA	XR_001434203.1	lncRNA
XM_015103461.1	TACC2	mRNA	XR_001434203.1	lncRNA
XM_015103461.1	TACC2	mRNA	XR_001434203.1	lncRNA
XM_015103461.1	TACC2	mRNA	XR_001434203.1	lncRNA
XM_015103461.1	TACC2	mRNA	XR_001434203.1	lncRNA
XM_015103461.1	TACC2	mRNA	XR_001434203.1	lncRNA
XM_015103461.1	TACC2	mRNA	XR_001434203.1	lncRNA
XM_015103461.1	TACC2	mRNA	XR_001434203.1	lncRNA
XM_015103461.1	TACC2	mRNA	XR_001434203.1	lncRNA
XM_015103461.1	TACC2	mRNA	XR_001434203.1	lncRNA
XM_015103461.1	TACC2	mRNA	XR_001434203.1	lncRNA
XM_015103461.1	TACC2	mRNA	XR_001434203.1	lncRNA
XM_012187378.2	CAMTA1	mRNA	XR_001436817.1	lncRNA
XM_012187378.2	CAMTA1	mRNA	XR_001436817.1	lncRNA
XM_012187378.2	CAMTA1	mRNA	XR_001436817.1	lncRNA
XM_012187378.2	CAMTA1	mRNA	XR_001436817.1	lncRNA
XM_012187378.2	CAMTA1	mRNA	XR_001436817.1	lncRNA
XM_012187378.2	CAMTA1	mRNA	XR_001436817.1	lncRNA
XM_012187378.2	CAMTA1	mRNA	XR_001436817.1	lncRNA
XM_012187378.2	CAMTA1	mRNA	XR_001436817.1	lncRNA
XM_012187378.2	CAMTA1	mRNA	XR_001436817.1	lncRNA
XM_012187378.2	CAMTA1	mRNA	XR_001436817.1	lncRNA
XM_012187378.2	CAMTA1	mRNA	XR_001436817.1	lncRNA
XM_012187378.2	CAMTA1	mRNA	XR_001436817.1	lncRNA
XM_012187378.2	CAMTA1	mRNA	XR_001436817.1	lncRNA

XM_012187378.2	CAMTA1	mRNA	XR_001436817.1	lncRNA
XM_012187378.2	CAMTA1	mRNA	XR_001436817.1	lncRNA
XM_012187378.2	CAMTA1	mRNA	XR_001436817.1	lncRNA
XM_012187378.2	CAMTA1	mRNA	XR_001436817.1	lncRNA
XM_012187378.2	CAMTA1	mRNA	XR_001436817.1	lncRNA
XM_012187378.2	CAMTA1	mRNA	XR_001436817.1	lncRNA
XM_012187378.2	CAMTA1	mRNA	XR_001434203.1	lncRNA
XM_012187378.2	CAMTA1	mRNA	XR_001434203.1	lncRNA
XM_012187378.2	CAMTA1	mRNA	XR_001434203.1	lncRNA
XM_012187378.2	CAMTA1	mRNA	XR_001434203.1	lncRNA
XM_012187378.2	CAMTA1	mRNA	XR_001434203.1	lncRNA
XM_012187378.2	CAMTA1	mRNA	XR_001434203.1	lncRNA
XM_012187378.2	CAMTA1	mRNA	XR_001434203.1	lncRNA
XM_012187378.2	CAMTA1	mRNA	XR_001434203.1	lncRNA
XM_012187378.2	CAMTA1	mRNA	XR_001434203.1	lncRNA
XM_012187378.2	CAMTA1	mRNA	XR_001434203.1	lncRNA
XM_012187378.2	CAMTA1	mRNA	XR_001434203.1	lncRNA
XM_012187378.2	CAMTA1	mRNA	XR_001434203.1	lncRNA
XM_012187378.2	CAMTA1	mRNA	XR_001434203.1	lncRNA
XM_012187378.2	CAMTA1	mRNA	XR_001434203.1	lncRNA
XM_012187378.2	CAMTA1	mRNA	XR_001434203.1	lncRNA
XM_012187378.2	CAMTA1	mRNA	XR_001434203.1	lncRNA
XM_012187378.2	CAMTA1	mRNA	XR_001434203.1	lncRNA
XM_012187378.2	CAMTA1	mRNA	XR_001434203.1	lncRNA
XM_012187378.2	CAMTA1	mRNA	XR_001434203.1	lncRNA
XM_004005724.3	NCOA1	mRNA	XR_001041211.2	lncRNA
XM_004005724.3	NCOA1	mRNA	XR_001041211.2	lncRNA
XM_004005724.3	NCOA1	mRNA	XR_001041211.2	lncRNA
XM_004005724.3	NCOA1	mRNA	XR_001041211.2	lncRNA
XM_004005724.3	NCOA1	mRNA	XR_001041211.2	lncRNA
XM_004005724.3	NCOA1	mRNA	XR_001041211.2	lncRNA
XM_004005724.3	NCOA1	mRNA	XR_001041211.2	lncRNA
XM_004005724.3	NCOA1	mRNA	XR_001041211.2	lncRNA
XM_004005724.3	NCOA1	mRNA	XR_001041211.2	lncRNA
XM_004005724.3	NCOA1	mRNA	XR_001041211.2	lncRNA
XM_004005724.3	NCOA1	mRNA	XR_001041211.2	lncRNA
XM_004005724.3	NCOA1	mRNA	XR_001041211.2	lncRNA
XM_004005724.3	NCOA1	mRNA	XR_001041211.2	lncRNA
XM_004005724.3	NCOA1	mRNA	XR_001041211.2	lncRNA
XM_004005724.3	NCOA1	mRNA	XR_001041211.2	lncRNA
XM_004005724.3	NCOA1	mRNA	XR_001041211.2	lncRNA
XM_004005724.3	NCOA1	mRNA	XR_001041211.2	lncRNA
XM_012185285.2	SYNRG	mRNA	XR_001041211.2	lncRNA
XM_012185285.2	SYNRG	mRNA	XR_001041211.2	lncRNA
XM_012185285.2	SYNRG	mRNA	XR_001041211.2	lncRNA
XM_012185285.2	SYNRG	mRNA	XR_001041211.2	lncRNA
XM_012185285.2	SYNRG	mRNA	XR_001041211.2	lncRNA
XM_012185285.2	SYNRG	mRNA	XR_001041211.2	lncRNA
XM_012185285.2	SYNRG	mRNA	XR_001041211.2	lncRNA
XM_012185285.2	SYNRG	mRNA	XR_001041211.2	lncRNA
XM_012185285.2	SYNRG	mRNA	XR_001041211.2	lncRNA
XM_012185285.2	SYNRG	mRNA	XR_001041211.2	lncRNA
XM_012185285.2	SYNRG	mRNA	XR_001041211.2	lncRNA
XM_012185285.2	SYNRG	mRNA	XR_001041211.2	lncRNA

XM_012185285.2	SYNRG	mRNA	XR_001041211.2	lncRNA
XM_012185285.2	SYNRG	mRNA	XR_001041211.2	lncRNA
XM_012185285.2	SYNRG	mRNA	XR_001041211.2	lncRNA
XM_012185285.2	SYNRG	mRNA	XR_001041211.2	lncRNA
XM_012185285.2	SYNRG	mRNA	XR_001041211.2	lncRNA
XM_012180879.2	MYO9A	mRNA	XR_001436817.1	lncRNA
XM_012180879.2	MYO9A	mRNA	XR_001436817.1	lncRNA
XM_012180879.2	MYO9A	mRNA	XR_001436817.1	lncRNA
XM_012180879.2	MYO9A	mRNA	XR_001436817.1	lncRNA
XM_012180879.2	MYO9A	mRNA	XR_001436817.1	lncRNA
XM_012180879.2	MYO9A	mRNA	XR_001436817.1	lncRNA
XM_012180879.2	MYO9A	mRNA	XR_001436817.1	lncRNA
XM_012180879.2	MYO9A	mRNA	XR_001436817.1	lncRNA
XM_012180879.2	MYO9A	mRNA	XR_001436817.1	lncRNA
XM_012180879.2	MYO9A	mRNA	XR_001436817.1	lncRNA
XM_012180879.2	MYO9A	mRNA	XR_001436817.1	lncRNA
XM_012180879.2	MYO9A	mRNA	XR_001436817.1	lncRNA
XM_012180879.2	MYO9A	mRNA	XR_001436817.1	lncRNA
XM_012180879.2	MYO9A	mRNA	XR_001436817.1	lncRNA
XM_012180879.2	MYO9A	mRNA	XR_001436817.1	lncRNA
XM_012180879.2	MYO9A	mRNA	XR_001436817.1	lncRNA
XM_012180879.2	MYO9A	mRNA	XR_001436817.1	lncRNA
XM_012180879.2	MYO9A	mRNA	XR_001434203.1	lncRNA
XM_012180879.2	MYO9A	mRNA	XR_001434203.1	lncRNA
XM_012180879.2	MYO9A	mRNA	XR_001434203.1	lncRNA
XM_012180879.2	MYO9A	mRNA	XR_001434203.1	lncRNA
XM_012180879.2	MYO9A	mRNA	XR_001434203.1	lncRNA
XM_012180879.2	MYO9A	mRNA	XR_001434203.1	lncRNA
XM_012180879.2	MYO9A	mRNA	XR_001434203.1	lncRNA
XM_012180879.2	MYO9A	mRNA	XR_001434203.1	lncRNA
XM_012180879.2	MYO9A	mRNA	XR_001434203.1	lncRNA
XM_012180879.2	MYO9A	mRNA	XR_001434203.1	lncRNA
XM_012180879.2	MYO9A	mRNA	XR_001434203.1	lncRNA
XM_012180879.2	MYO9A	mRNA	XR_001434203.1	lncRNA
XM_012180879.2	MYO9A	mRNA	XR_001434203.1	lncRNA
XM_012180879.2	MYO9A	mRNA	XR_001434203.1	lncRNA
XM_012180879.2	MYO9A	mRNA	XR_001434203.1	lncRNA
XM_012180879.2	MYO9A	mRNA	XR_001434203.1	lncRNA
XM_012180879.2	MYO9A	mRNA	XR_001434203.1	lncRNA
XM_015103461.1	TACC2	mRNA	XR_001042381.2	lncRNA
XM_015103461.1	TACC2	mRNA	XR_001042381.2	lncRNA
XM_015103461.1	TACC2	mRNA	XR_001042381.2	lncRNA
XM_015103461.1	TACC2	mRNA	XR_001042381.2	lncRNA
XM_015103461.1	TACC2	mRNA	XR_001042381.2	lncRNA
XM_015103461.1	TACC2	mRNA	XR_001042381.2	lncRNA
XM_015103461.1	TACC2	mRNA	XR_001042381.2	lncRNA
XM_015103461.1	TACC2	mRNA	XR_001042381.2	lncRNA
XM_015103461.1	TACC2	mRNA	XR_001042381.2	lncRNA
XM_015103461.1	TACC2	mRNA	XR_001042381.2	lncRNA
XM_015103461.1	TACC2	mRNA	XR_001042381.2	lncRNA
XM_015103461.1	TACC2	mRNA	XR_001042381.2	lncRNA
XM_015103461.1	TACC2	mRNA	XR_001042381.2	lncRNA
XM_015103461.1	TACC2	mRNA	XR_001042381.2	lncRNA
XM_015103461.1	TACC2	mRNA	XR_001042381.2	lncRNA

XM_015103461.1	TACC2	mRNA	XR_001042381.2	lncRNA
XM_015103461.1	TACC2	mRNA	XR_001042381.2	lncRNA
XM_015091996.1	LOC101121639	mRNA	XR_001436532.1	lncRNA
XM_015091996.1	LOC101121639	mRNA	XR_001436532.1	lncRNA
XM_015091996.1	LOC101121639	mRNA	XR_001436532.1	lncRNA
XM_015091996.1	LOC101121639	mRNA	XR_001436532.1	lncRNA
XM_015091996.1	LOC101121639	mRNA	XR_001436532.1	lncRNA
XM_015091996.1	LOC101121639	mRNA	XR_001436532.1	lncRNA
XM_015091996.1	LOC101121639	mRNA	XR_001436532.1	lncRNA
XM_015091996.1	LOC101121639	mRNA	XR_001436532.1	lncRNA
XM_015091996.1	LOC101121639	mRNA	XR_001436532.1	lncRNA
XM_015091996.1	LOC101121639	mRNA	XR_001436532.1	lncRNA
XM_015091996.1	LOC101121639	mRNA	XR_001436532.1	lncRNA
XM_015091996.1	LOC101121639	mRNA	XR_001436532.1	lncRNA
XM_015091996.1	LOC101121639	mRNA	XR_001436532.1	lncRNA
XM_015091996.1	LOC101121639	mRNA	XR_001436532.1	lncRNA
XM_015091996.1	LOC101121639	mRNA	XR_001436532.1	lncRNA
XM_015091996.1	LOC101121639	mRNA	XR_001436532.1	lncRNA
XM_015091996.1	LOC101121639	mRNA	XR_001436532.1	lncRNA
XM_012137425.2	NEB	mRNA	XR_001436817.1	lncRNA
XM_012137425.2	NEB	mRNA	XR_001436817.1	lncRNA
XM_012137425.2	NEB	mRNA	XR_001436817.1	lncRNA
XM_012137425.2	NEB	mRNA	XR_001436817.1	lncRNA
XM_012137425.2	NEB	mRNA	XR_001436817.1	lncRNA
XM_012137425.2	NEB	mRNA	XR_001436817.1	lncRNA
XM_012137425.2	NEB	mRNA	XR_001436817.1	lncRNA
XM_012137425.2	NEB	mRNA	XR_001436817.1	lncRNA
XM_012137425.2	NEB	mRNA	XR_001436817.1	lncRNA
XM_012137425.2	NEB	mRNA	XR_001436817.1	lncRNA
XM_012137425.2	NEB	mRNA	XR_001436817.1	lncRNA
XM_012137425.2	NEB	mRNA	XR_001436817.1	lncRNA
XM_012137425.2	NEB	mRNA	XR_001436817.1	lncRNA
XM_012137425.2	NEB	mRNA	XR_001436817.1	lncRNA
XM_012137425.2	NEB	mRNA	XR_001436817.1	lncRNA
XM_012137425.2	NEB	mRNA	XR_001436817.1	lncRNA
XM_012137425.2	NEB	mRNA	XR_001436817.1	lncRNA
XM_012137434.2	NEB	mRNA	XR_001436817.1	lncRNA
XM_012137434.2	NEB	mRNA	XR_001436817.1	lncRNA
XM_012137434.2	NEB	mRNA	XR_001436817.1	lncRNA
XM_012137434.2	NEB	mRNA	XR_001436817.1	lncRNA
XM_012137434.2	NEB	mRNA	XR_001436817.1	lncRNA
XM_012137434.2	NEB	mRNA	XR_001436817.1	lncRNA
XM_012137434.2	NEB	mRNA	XR_001436817.1	lncRNA
XM_012137434.2	NEB	mRNA	XR_001436817.1	lncRNA
XM_012137434.2	NEB	mRNA	XR_001436817.1	lncRNA
XM_012137434.2	NEB	mRNA	XR_001436817.1	lncRNA
XM_012137434.2	NEB	mRNA	XR_001436817.1	lncRNA
XM_012137434.2	NEB	mRNA	XR_001436817.1	lncRNA
XM_012137434.2	NEB	mRNA	XR_001436817.1	lncRNA
XM_012137434.2	NEB	mRNA	XR_001436817.1	lncRNA
XM_012137434.2	NEB	mRNA	XR_001436817.1	lncRNA
XM_012137434.2	NEB	mRNA	XR_001436817.1	lncRNA
XM_012137434.2	NEB	mRNA	XR_001436817.1	lncRNA
XM_012137434.2	NEB	mRNA	XR_001434203.1	lncRNA

XM_012137434.2	NEB	mRNA	XR_001434203.1	lncRNA
XM_012137434.2	NEB	mRNA	XR_001434203.1	lncRNA
XM_012137434.2	NEB	mRNA	XR_001434203.1	lncRNA
XM_012137434.2	NEB	mRNA	XR_001434203.1	lncRNA
XM_012137434.2	NEB	mRNA	XR_001434203.1	lncRNA
XM_012137434.2	NEB	mRNA	XR_001434203.1	lncRNA
XM_012137434.2	NEB	mRNA	XR_001434203.1	lncRNA
XM_012137434.2	NEB	mRNA	XR_001434203.1	lncRNA
XM_012137434.2	NEB	mRNA	XR_001434203.1	lncRNA
XM_012137434.2	NEB	mRNA	XR_001434203.1	lncRNA
XM_012137434.2	NEB	mRNA	XR_001434203.1	lncRNA
XM_012137434.2	NEB	mRNA	XR_001434203.1	lncRNA
XM_012137434.2	NEB	mRNA	XR_001434203.1	lncRNA
XM_012137434.2	NEB	mRNA	XR_001434203.1	lncRNA
XM_012137434.2	NEB	mRNA	XR_001434203.1	lncRNA
XM_012137434.2	NEB	mRNA	XR_001434203.1	lncRNA
XM_012178306.2	FAM193B	mRNA	XR_001041211.2	lncRNA
XM_012178306.2	FAM193B	mRNA	XR_001041211.2	lncRNA
XM_012178306.2	FAM193B	mRNA	XR_001041211.2	lncRNA
XM_012178306.2	FAM193B	mRNA	XR_001041211.2	lncRNA
XM_012178306.2	FAM193B	mRNA	XR_001041211.2	lncRNA
XM_012178306.2	FAM193B	mRNA	XR_001041211.2	lncRNA
XM_012178306.2	FAM193B	mRNA	XR_001041211.2	lncRNA
XM_012178306.2	FAM193B	mRNA	XR_001041211.2	lncRNA
XM_012178306.2	FAM193B	mRNA	XR_001041211.2	lncRNA
XM_012178306.2	FAM193B	mRNA	XR_001041211.2	lncRNA
XM_012178306.2	FAM193B	mRNA	XR_001041211.2	lncRNA
XM_012178306.2	FAM193B	mRNA	XR_001041211.2	lncRNA
XM_012178306.2	FAM193B	mRNA	XR_001041211.2	lncRNA
XM_012178306.2	FAM193B	mRNA	XR_001041211.2	lncRNA
XM_012178306.2	FAM193B	mRNA	XR_001041211.2	lncRNA
XM_012178306.2	FAM193B	mRNA	XR_001041211.2	lncRNA
XM_012177947.2	INSR	mRNA	XR_001434203.1	lncRNA
XM_012177947.2	INSR	mRNA	XR_001434203.1	lncRNA
XM_012177947.2	INSR	mRNA	XR_001434203.1	lncRNA
XM_012177947.2	INSR	mRNA	XR_001434203.1	lncRNA
XM_012177947.2	INSR	mRNA	XR_001434203.1	lncRNA
XM_012177947.2	INSR	mRNA	XR_001434203.1	lncRNA
XM_012177947.2	INSR	mRNA	XR_001434203.1	lncRNA
XM_012177947.2	INSR	mRNA	XR_001434203.1	lncRNA
XM_012177947.2	INSR	mRNA	XR_001434203.1	lncRNA
XM_012177947.2	INSR	mRNA	XR_001434203.1	lncRNA
XM_012177947.2	INSR	mRNA	XR_001434203.1	lncRNA
XM_012177947.2	INSR	mRNA	XR_001434203.1	lncRNA
XM_012177947.2	INSR	mRNA	XR_001434203.1	lncRNA
XM_012177947.2	INSR	mRNA	XR_001434203.1	lncRNA
XM_012177947.2	INSR	mRNA	XR_001434203.1	lncRNA
XM_012177947.2	INSR	mRNA	XR_001434203.1	lncRNA
XM_012177947.2	INSR	mRNA	XR_001434203.1	lncRNA
XM_012177947.2	INSR	mRNA	XR_001436817.1	lncRNA
XM_012177947.2	INSR	mRNA	XR_001436817.1	lncRNA
XM_012177947.2	INSR	mRNA	XR_001436817.1	lncRNA
XM_012177947.2	INSR	mRNA	XR_001436817.1	lncRNA
XM_012177947.2	INSR	mRNA	XR_001436817.1	lncRNA

XM_012177947.2	INSR	mRNA	XR_001436817.1	lncRNA
XM_012177947.2	INSR	mRNA	XR_001436817.1	lncRNA
XM_012177947.2	INSR	mRNA	XR_001436817.1	lncRNA
XM_012177947.2	INSR	mRNA	XR_001436817.1	lncRNA
XM_012177947.2	INSR	mRNA	XR_001436817.1	lncRNA
XM_012177947.2	INSR	mRNA	XR_001436817.1	lncRNA
XM_012177947.2	INSR	mRNA	XR_001436817.1	lncRNA
XM_012177947.2	INSR	mRNA	XR_001436817.1	lncRNA
XM_012177947.2	INSR	mRNA	XR_001436817.1	lncRNA
XM_012177947.2	INSR	mRNA	XR_001436817.1	lncRNA
XM_012177947.2	INSR	mRNA	XR_001436817.1	lncRNA
XM_012177947.2	INSR	mRNA	XR_001436817.1	lncRNA
XM_012185799.2	EPN2	mRNA	XR_001434203.1	lncRNA
XM_012185799.2	EPN2	mRNA	XR_001434203.1	lncRNA
XM_012185799.2	EPN2	mRNA	XR_001434203.1	lncRNA
XM_012185799.2	EPN2	mRNA	XR_001434203.1	lncRNA
XM_012185799.2	EPN2	mRNA	XR_001434203.1	lncRNA
XM_012185799.2	EPN2	mRNA	XR_001434203.1	lncRNA
XM_012185799.2	EPN2	mRNA	XR_001434203.1	lncRNA
XM_012185799.2	EPN2	mRNA	XR_001434203.1	lncRNA
XM_012185799.2	EPN2	mRNA	XR_001434203.1	lncRNA
XM_012185799.2	EPN2	mRNA	XR_001434203.1	lncRNA
XM_012185799.2	EPN2	mRNA	XR_001434203.1	lncRNA
XM_012185799.2	EPN2	mRNA	XR_001434203.1	lncRNA
XM_012185799.2	EPN2	mRNA	XR_001434203.1	lncRNA
XM_012185799.2	EPN2	mRNA	XR_001434203.1	lncRNA
XM_012185799.2	EPN2	mRNA	XR_001434203.1	lncRNA
XM_012185799.2	EPN2	mRNA	XR_001434203.1	lncRNA
XM_012185799.2	EPN2	mRNA	XR_001434203.1	lncRNA
XM_012185799.2	EPN2	mRNA	XR_001436817.1	lncRNA
XM_012185799.2	EPN2	mRNA	XR_001436817.1	lncRNA
XM_012185799.2	EPN2	mRNA	XR_001436817.1	lncRNA
XM_012185799.2	EPN2	mRNA	XR_001436817.1	lncRNA
XM_012185799.2	EPN2	mRNA	XR_001436817.1	lncRNA
XM_012185799.2	EPN2	mRNA	XR_001436817.1	lncRNA
XM_012185799.2	EPN2	mRNA	XR_001436817.1	lncRNA
XM_012185799.2	EPN2	mRNA	XR_001436817.1	lncRNA
XM_012185799.2	EPN2	mRNA	XR_001436817.1	lncRNA
XM_012185799.2	EPN2	mRNA	XR_001436817.1	lncRNA
XM_012185799.2	EPN2	mRNA	XR_001436817.1	lncRNA
XM_012185799.2	EPN2	mRNA	XR_001436817.1	lncRNA
XM_012185799.2	EPN2	mRNA	XR_001436817.1	lncRNA
XM_012185799.2	EPN2	mRNA	XR_001436817.1	lncRNA
XM_012185799.2	EPN2	mRNA	XR_001436817.1	lncRNA
XM_012185799.2	EPN2	mRNA	XR_001436817.1	lncRNA
XM_012185799.2	EPN2	mRNA	XR_001436817.1	lncRNA
XM_012187914.1	GIGYF2	mRNA	XR_001436817.1	lncRNA
XM_012187914.1	GIGYF2	mRNA	XR_001436817.1	lncRNA
XM_012187914.1	GIGYF2	mRNA	XR_001436817.1	lncRNA
XM_012187914.1	GIGYF2	mRNA	XR_001436817.1	lncRNA
XM_012187914.1	GIGYF2	mRNA	XR_001436817.1	lncRNA
XM_012187914.1	GIGYF2	mRNA	XR_001436817.1	lncRNA
XM_012187914.1	GIGYF2	mRNA	XR_001436817.1	lncRNA
XM_012187914.1	GIGYF2	mRNA	XR_001436817.1	lncRNA

XM_012187914.1	GIGYF2	mRNA	XR_001436817.1	lncRNA
XM_012187914.1	GIGYF2	mRNA	XR_001436817.1	lncRNA
XM_012187914.1	GIGYF2	mRNA	XR_001436817.1	lncRNA
XM_012187914.1	GIGYF2	mRNA	XR_001436817.1	lncRNA
XM_012187914.1	GIGYF2	mRNA	XR_001436817.1	lncRNA
XM_012187914.1	GIGYF2	mRNA	XR_001436817.1	lncRNA
XM_012187914.1	GIGYF2	mRNA	XR_001436817.1	lncRNA
XM_012187914.1	GIGYF2	mRNA	XR_001436817.1	lncRNA
XM_012152455.2	LUZP1	mRNA	XR_001436817.1	lncRNA
XM_012152455.2	LUZP1	mRNA	XR_001436817.1	lncRNA
XM_012152455.2	LUZP1	mRNA	XR_001436817.1	lncRNA
XM_012152455.2	LUZP1	mRNA	XR_001436817.1	lncRNA
XM_012152455.2	LUZP1	mRNA	XR_001436817.1	lncRNA
XM_012152455.2	LUZP1	mRNA	XR_001436817.1	lncRNA
XM_012152455.2	LUZP1	mRNA	XR_001436817.1	lncRNA
XM_012152455.2	LUZP1	mRNA	XR_001436817.1	lncRNA
XM_012152455.2	LUZP1	mRNA	XR_001436817.1	lncRNA
XM_012152455.2	LUZP1	mRNA	XR_001436817.1	lncRNA
XM_012152455.2	LUZP1	mRNA	XR_001436817.1	lncRNA
XM_012152455.2	LUZP1	mRNA	XR_001436817.1	lncRNA
XM_012152455.2	LUZP1	mRNA	XR_001436817.1	lncRNA
XM_012152455.2	LUZP1	mRNA	XR_001436817.1	lncRNA
XM_012152455.2	LUZP1	mRNA	XR_001436817.1	lncRNA
XM_012152455.2	LUZP1	mRNA	XR_001436817.1	lncRNA
XM_004005724.3	NCOA1	mRNA	XR_001436532.1	lncRNA
XM_004005724.3	NCOA1	mRNA	XR_001436532.1	lncRNA
XM_004005724.3	NCOA1	mRNA	XR_001436532.1	lncRNA
XM_004005724.3	NCOA1	mRNA	XR_001436532.1	lncRNA
XM_004005724.3	NCOA1	mRNA	XR_001436532.1	lncRNA
XM_004005724.3	NCOA1	mRNA	XR_001436532.1	lncRNA
XM_004005724.3	NCOA1	mRNA	XR_001436532.1	lncRNA
XM_004005724.3	NCOA1	mRNA	XR_001436532.1	lncRNA
XM_004005724.3	NCOA1	mRNA	XR_001436532.1	lncRNA
XM_004005724.3	NCOA1	mRNA	XR_001436532.1	lncRNA
XM_004005724.3	NCOA1	mRNA	XR_001436532.1	lncRNA
XM_004005724.3	NCOA1	mRNA	XR_001436532.1	lncRNA
XM_004005724.3	NCOA1	mRNA	XR_001436532.1	lncRNA
XM_004005724.3	NCOA1	mRNA	XR_001436532.1	lncRNA
XM_004005724.3	NCOA1	mRNA	XR_001436532.1	lncRNA
XM_004005724.3	NCOA1	mRNA	XR_001436532.1	lncRNA
XM_012186944.1	DSTYK	mRNA	XR_001436532.1	lncRNA
XM_012186944.1	DSTYK	mRNA	XR_001436532.1	lncRNA
XM_012186944.1	DSTYK	mRNA	XR_001436532.1	lncRNA
XM_012186944.1	DSTYK	mRNA	XR_001436532.1	lncRNA
XM_012186944.1	DSTYK	mRNA	XR_001436532.1	lncRNA
XM_012186944.1	DSTYK	mRNA	XR_001436532.1	lncRNA
XM_012186944.1	DSTYK	mRNA	XR_001436532.1	lncRNA
XM_012186944.1	DSTYK	mRNA	XR_001436532.1	lncRNA
XM_012186944.1	DSTYK	mRNA	XR_001436532.1	lncRNA
XM_012186944.1	DSTYK	mRNA	XR_001436532.1	lncRNA
XM_012186944.1	DSTYK	mRNA	XR_001436532.1	lncRNA
XM_012186944.1	DSTYK	mRNA	XR_001436532.1	lncRNA
XM_012186944.1	DSTYK	mRNA	XR_001436532.1	lncRNA
XM_012186944.1	DSTYK	mRNA	XR_001436532.1	lncRNA

XM_012186944.1	DSTYK	mRNA	XR_001436532.1	lncRNA
XM_012186944.1	DSTYK	mRNA	XR_001436532.1	lncRNA
XM_012185285.2	SYNRG	mRNA	XR_001436532.1	lncRNA
XM_012185285.2	SYNRG	mRNA	XR_001436532.1	lncRNA
XM_012185285.2	SYNRG	mRNA	XR_001436532.1	lncRNA
XM_012185285.2	SYNRG	mRNA	XR_001436532.1	lncRNA
XM_012185285.2	SYNRG	mRNA	XR_001436532.1	lncRNA
XM_012185285.2	SYNRG	mRNA	XR_001436532.1	lncRNA
XM_012185285.2	SYNRG	mRNA	XR_001436532.1	lncRNA
XM_012185285.2	SYNRG	mRNA	XR_001436532.1	lncRNA
XM_012185285.2	SYNRG	mRNA	XR_001436532.1	lncRNA
XM_012185285.2	SYNRG	mRNA	XR_001436532.1	lncRNA
XM_012185285.2	SYNRG	mRNA	XR_001436532.1	lncRNA
XM_012185285.2	SYNRG	mRNA	XR_001436532.1	lncRNA
XM_012185285.2	SYNRG	mRNA	XR_001436532.1	lncRNA
XM_012185285.2	SYNRG	mRNA	XR_001436532.1	lncRNA
XM_012185285.2	SYNRG	mRNA	XR_001436532.1	lncRNA
XM_012185285.2	SYNRG	mRNA	XR_001436532.1	lncRNA
XM_012105688.2	WDR17	mRNA	XR_001436532.1	lncRNA
XM_012105688.2	WDR17	mRNA	XR_001436532.1	lncRNA
XM_012105688.2	WDR17	mRNA	XR_001436532.1	lncRNA
XM_012105688.2	WDR17	mRNA	XR_001436532.1	lncRNA
XM_012105688.2	WDR17	mRNA	XR_001436532.1	lncRNA
XM_012105688.2	WDR17	mRNA	XR_001436532.1	lncRNA
XM_012105688.2	WDR17	mRNA	XR_001436532.1	lncRNA
XM_012105688.2	WDR17	mRNA	XR_001436532.1	lncRNA
XM_012105688.2	WDR17	mRNA	XR_001436532.1	lncRNA
XM_012105688.2	WDR17	mRNA	XR_001436532.1	lncRNA
XM_012105688.2	WDR17	mRNA	XR_001436532.1	lncRNA
XM_012105688.2	WDR17	mRNA	XR_001436532.1	lncRNA
XM_012105688.2	WDR17	mRNA	XR_001436532.1	lncRNA
XM_012105688.2	WDR17	mRNA	XR_001436532.1	lncRNA
XM_012105688.2	WDR17	mRNA	XR_001436532.1	lncRNA
XM_012105688.2	WDR17	mRNA	XR_001436532.1	lncRNA
XM_012137425.2	NEB	mRNA	XR_001436532.1	lncRNA
XM_012137425.2	NEB	mRNA	XR_001436532.1	lncRNA
XM_012137425.2	NEB	mRNA	XR_001436532.1	lncRNA
XM_012137425.2	NEB	mRNA	XR_001436532.1	lncRNA
XM_012137425.2	NEB	mRNA	XR_001436532.1	lncRNA
XM_012137425.2	NEB	mRNA	XR_001436532.1	lncRNA
XM_012137425.2	NEB	mRNA	XR_001436532.1	lncRNA
XM_012137425.2	NEB	mRNA	XR_001436532.1	lncRNA
XM_012137425.2	NEB	mRNA	XR_001436532.1	lncRNA
XM_012137425.2	NEB	mRNA	XR_001436532.1	lncRNA
XM_012137425.2	NEB	mRNA	XR_001436532.1	lncRNA
XM_012137425.2	NEB	mRNA	XR_001436532.1	lncRNA
XM_012137425.2	NEB	mRNA	XR_001436532.1	lncRNA
XM_012137425.2	NEB	mRNA	XR_001436532.1	lncRNA
XM_012137425.2	NEB	mRNA	XR_001436532.1	lncRNA
XM_012137425.2	NEB	mRNA	XR_001436532.1	lncRNA
XM_015092323.1	KIRREL	mRNA	XR_001434203.1	lncRNA
XM_015092323.1	KIRREL	mRNA	XR_001434203.1	lncRNA
XM_015092323.1	KIRREL	mRNA	XR_001434203.1	lncRNA
XM_015092323.1	KIRREL	mRNA	XR_001434203.1	lncRNA

XM_015092323.1	KIRREL	mRNA	XR_001434203.1	lncRNA
XM_015092323.1	KIRREL	mRNA	XR_001434203.1	lncRNA
XM_015092323.1	KIRREL	mRNA	XR_001434203.1	lncRNA
XM_015092323.1	KIRREL	mRNA	XR_001434203.1	lncRNA
XM_015092323.1	KIRREL	mRNA	XR_001434203.1	lncRNA
XM_015092323.1	KIRREL	mRNA	XR_001434203.1	lncRNA
XM_015092323.1	KIRREL	mRNA	XR_001434203.1	lncRNA
XM_015092323.1	KIRREL	mRNA	XR_001434203.1	lncRNA
XM_015092323.1	KIRREL	mRNA	XR_001434203.1	lncRNA
XM_015092323.1	KIRREL	mRNA	XR_001434203.1	lncRNA
XM_015092323.1	KIRREL	mRNA	XR_001434203.1	lncRNA
XM_015092323.1	KIRREL	mRNA	XR_001434203.1	lncRNA
XM_015098446.1	VEZF1	mRNA	XR_001436817.1	lncRNA
XM_015098446.1	VEZF1	mRNA	XR_001436817.1	lncRNA
XM_015098446.1	VEZF1	mRNA	XR_001436817.1	lncRNA
XM_015098446.1	VEZF1	mRNA	XR_001436817.1	lncRNA
XM_015098446.1	VEZF1	mRNA	XR_001436817.1	lncRNA
XM_015098446.1	VEZF1	mRNA	XR_001436817.1	lncRNA
XM_015098446.1	VEZF1	mRNA	XR_001436817.1	lncRNA
XM_015098446.1	VEZF1	mRNA	XR_001436817.1	lncRNA
XM_015098446.1	VEZF1	mRNA	XR_001436817.1	lncRNA
XM_015098446.1	VEZF1	mRNA	XR_001436817.1	lncRNA
XM_015098446.1	VEZF1	mRNA	XR_001436817.1	lncRNA
XM_015098446.1	VEZF1	mRNA	XR_001436817.1	lncRNA
XM_015098446.1	VEZF1	mRNA	XR_001436817.1	lncRNA
XM_015098446.1	VEZF1	mRNA	XR_001436817.1	lncRNA
XM_015098446.1	VEZF1	mRNA	XR_001436817.1	lncRNA
XM_015098446.1	VEZF1	mRNA	XR_001436817.1	lncRNA
XM_015092323.1	KIRREL	mRNA	XR_001436817.1	lncRNA
XM_015092323.1	KIRREL	mRNA	XR_001436817.1	lncRNA
XM_015092323.1	KIRREL	mRNA	XR_001436817.1	lncRNA
XM_015092323.1	KIRREL	mRNA	XR_001436817.1	lncRNA
XM_015092323.1	KIRREL	mRNA	XR_001436817.1	lncRNA
XM_015092323.1	KIRREL	mRNA	XR_001436817.1	lncRNA
XM_015092323.1	KIRREL	mRNA	XR_001436817.1	lncRNA
XM_015092323.1	KIRREL	mRNA	XR_001436817.1	lncRNA
XM_015092323.1	KIRREL	mRNA	XR_001436817.1	lncRNA
XM_015092323.1	KIRREL	mRNA	XR_001436817.1	lncRNA
XM_015092323.1	KIRREL	mRNA	XR_001436817.1	lncRNA
XM_015092323.1	KIRREL	mRNA	XR_001436817.1	lncRNA
XM_015092323.1	KIRREL	mRNA	XR_001436817.1	lncRNA
XM_015092323.1	KIRREL	mRNA	XR_001436817.1	lncRNA
XM_015092323.1	KIRREL	mRNA	XR_001436817.1	lncRNA
XM_015092323.1	KIRREL	mRNA	XR_001436817.1	lncRNA
XM_012096323.1	PHF21A	mRNA	XR_001436817.1	lncRNA
XM_012096323.1	PHF21A	mRNA	XR_001436817.1	lncRNA
XM_012096323.1	PHF21A	mRNA	XR_001436817.1	lncRNA
XM_012096323.1	PHF21A	mRNA	XR_001436817.1	lncRNA
XM_012096323.1	PHF21A	mRNA	XR_001436817.1	lncRNA
XM_012096323.1	PHF21A	mRNA	XR_001436817.1	lncRNA
XM_012096323.1	PHF21A	mRNA	XR_001436817.1	lncRNA
XM_012096323.1	PHF21A	mRNA	XR_001436817.1	lncRNA
XM_012096323.1	PHF21A	mRNA	XR_001436817.1	lncRNA
XM_012096323.1	PHF21A	mRNA	XR_001436817.1	lncRNA

XM_012096323.1	PHF21A	mRNA	XR_001436817.1	lncRNA
XM_012096323.1	PHF21A	mRNA	XR_001436817.1	lncRNA
XM_012096323.1	PHF21A	mRNA	XR_001436817.1	lncRNA
XM_012096323.1	PHF21A	mRNA	XR_001436817.1	lncRNA
XM_012096323.1	PHF21A	mRNA	XR_001436817.1	lncRNA
XM_012096323.1	PHF21A	mRNA	XR_001436817.1	lncRNA
XM_012096323.1	PHF21A	mRNA	XR_001434203.1	lncRNA
XM_012096323.1	PHF21A	mRNA	XR_001434203.1	lncRNA
XM_012096323.1	PHF21A	mRNA	XR_001434203.1	lncRNA
XM_012096323.1	PHF21A	mRNA	XR_001434203.1	lncRNA
XM_012096323.1	PHF21A	mRNA	XR_001434203.1	lncRNA
XM_012096323.1	PHF21A	mRNA	XR_001434203.1	lncRNA
XM_012096323.1	PHF21A	mRNA	XR_001434203.1	lncRNA
XM_012096323.1	PHF21A	mRNA	XR_001434203.1	lncRNA
XM_012096323.1	PHF21A	mRNA	XR_001434203.1	lncRNA
XM_012096323.1	PHF21A	mRNA	XR_001434203.1	lncRNA
XM_012096323.1	PHF21A	mRNA	XR_001434203.1	lncRNA
XM_012096323.1	PHF21A	mRNA	XR_001434203.1	lncRNA
XM_012096323.1	PHF21A	mRNA	XR_001434203.1	lncRNA
XM_012096323.1	PHF21A	mRNA	XR_001434203.1	lncRNA
XM_012096323.1	PHF21A	mRNA	XR_001434203.1	lncRNA
XM_012096323.1	PHF21A	mRNA	XR_001434203.1	lncRNA
XM_012178306.2	FAM193B	mRNA	XR_001434892.1	lncRNA
XM_012178306.2	FAM193B	mRNA	XR_001434892.1	lncRNA
XM_012178306.2	FAM193B	mRNA	XR_001434892.1	lncRNA
XM_012178306.2	FAM193B	mRNA	XR_001434892.1	lncRNA
XM_012178306.2	FAM193B	mRNA	XR_001434892.1	lncRNA
XM_012178306.2	FAM193B	mRNA	XR_001434892.1	lncRNA
XM_012178306.2	FAM193B	mRNA	XR_001434892.1	lncRNA
XM_012178306.2	FAM193B	mRNA	XR_001434892.1	lncRNA
XM_012178306.2	FAM193B	mRNA	XR_001434892.1	lncRNA
XM_012178306.2	FAM193B	mRNA	XR_001434892.1	lncRNA
XM_012178306.2	FAM193B	mRNA	XR_001434892.1	lncRNA
XM_012178306.2	FAM193B	mRNA	XR_001434892.1	lncRNA
XM_012178306.2	FAM193B	mRNA	XR_001434892.1	lncRNA
XM_012178306.2	FAM193B	mRNA	XR_001434892.1	lncRNA
XM_012178306.2	FAM193B	mRNA	XR_001434892.1	lncRNA
XM_012144682.2	MAP2	mRNA	XR_001434761.1	lncRNA
XM_012144682.2	MAP2	mRNA	XR_001434761.1	lncRNA
XM_012144682.2	MAP2	mRNA	XR_001434761.1	lncRNA
XM_012144682.2	MAP2	mRNA	XR_001434761.1	lncRNA
XM_012144682.2	MAP2	mRNA	XR_001434761.1	lncRNA
XM_012144682.2	MAP2	mRNA	XR_001434761.1	lncRNA
XM_012144682.2	MAP2	mRNA	XR_001434761.1	lncRNA
XM_012144682.2	MAP2	mRNA	XR_001434761.1	lncRNA
XM_012144682.2	MAP2	mRNA	XR_001434761.1	lncRNA
XM_012144682.2	MAP2	mRNA	XR_001434761.1	lncRNA
XM_012144682.2	MAP2	mRNA	XR_001434761.1	lncRNA
XM_012144682.2	MAP2	mRNA	XR_001434761.1	lncRNA
XM_012144682.2	MAP2	mRNA	XR_001434761.1	lncRNA
XM_012144682.2	MAP2	mRNA	XR_001434761.1	lncRNA
XM_012144682.2	MAP2	mRNA	XR_001434761.1	lncRNA
XM_012185799.2	EPN2	mRNA	XR_001042381.2	lncRNA
XM_012185799.2	EPN2	mRNA	XR_001042381.2	lncRNA

XM_012185799.2	EPN2	mRNA	XR_001042381.2	lncRNA
XM_012185799.2	EPN2	mRNA	XR_001042381.2	lncRNA
XM_012185799.2	EPN2	mRNA	XR_001042381.2	lncRNA
XM_012185799.2	EPN2	mRNA	XR_001042381.2	lncRNA
XM_012185799.2	EPN2	mRNA	XR_001042381.2	lncRNA
XM_012185799.2	EPN2	mRNA	XR_001042381.2	lncRNA
XM_012185799.2	EPN2	mRNA	XR_001042381.2	lncRNA
XM_012185799.2	EPN2	mRNA	XR_001042381.2	lncRNA
XM_012185799.2	EPN2	mRNA	XR_001042381.2	lncRNA
XM_012185799.2	EPN2	mRNA	XR_001042381.2	lncRNA
XM_012185799.2	EPN2	mRNA	XR_001042381.2	lncRNA
XM_012185799.2	EPN2	mRNA	XR_001042381.2	lncRNA
XM_012185799.2	EPN2	mRNA	XR_001042381.2	lncRNA
XM_012185799.2	EPN2	mRNA	XR_001042381.2	lncRNA
XM_012177947.2	INSR	mRNA	XR_001436532.1	lncRNA
XM_012177947.2	INSR	mRNA	XR_001436532.1	lncRNA
XM_012177947.2	INSR	mRNA	XR_001436532.1	lncRNA
XM_012177947.2	INSR	mRNA	XR_001436532.1	lncRNA
XM_012177947.2	INSR	mRNA	XR_001436532.1	lncRNA
XM_012177947.2	INSR	mRNA	XR_001436532.1	lncRNA
XM_012177947.2	INSR	mRNA	XR_001436532.1	lncRNA
XM_012177947.2	INSR	mRNA	XR_001436532.1	lncRNA
XM_012177947.2	INSR	mRNA	XR_001436532.1	lncRNA
XM_012177947.2	INSR	mRNA	XR_001436532.1	lncRNA
XM_012177947.2	INSR	mRNA	XR_001436532.1	lncRNA
XM_012177947.2	INSR	mRNA	XR_001436532.1	lncRNA
XM_012177947.2	INSR	mRNA	XR_001436532.1	lncRNA
XM_012177947.2	INSR	mRNA	XR_001436532.1	lncRNA
XM_012177947.2	INSR	mRNA	XR_001436532.1	lncRNA
XM_012177947.2	INSR	mRNA	XR_001436532.1	lncRNA
XM_012174704.2	ZBTB39	mRNA	XR_001436817.1	lncRNA
XM_012174704.2	ZBTB39	mRNA	XR_001436817.1	lncRNA
XM_012174704.2	ZBTB39	mRNA	XR_001436817.1	lncRNA
XM_012174704.2	ZBTB39	mRNA	XR_001436817.1	lncRNA
XM_012174704.2	ZBTB39	mRNA	XR_001436817.1	lncRNA
XM_012174704.2	ZBTB39	mRNA	XR_001436817.1	lncRNA
XM_012174704.2	ZBTB39	mRNA	XR_001436817.1	lncRNA
XM_012174704.2	ZBTB39	mRNA	XR_001436817.1	lncRNA
XM_012174704.2	ZBTB39	mRNA	XR_001436817.1	lncRNA
XM_012174704.2	ZBTB39	mRNA	XR_001436817.1	lncRNA
XM_012174704.2	ZBTB39	mRNA	XR_001436817.1	lncRNA
XM_012174704.2	ZBTB39	mRNA	XR_001436817.1	lncRNA
XM_012174704.2	ZBTB39	mRNA	XR_001436817.1	lncRNA
XM_012174704.2	ZBTB39	mRNA	XR_001436817.1	lncRNA
XM_012174704.2	ZBTB39	mRNA	XR_001436817.1	lncRNA
XM_012174704.2	ZBTB39	mRNA	XR_001436817.1	lncRNA
XM_012184887.2	DOCK9	mRNA	XR_001436817.1	lncRNA
XM_012184887.2	DOCK9	mRNA	XR_001436817.1	lncRNA
XM_012184887.2	DOCK9	mRNA	XR_001436817.1	lncRNA
XM_012184887.2	DOCK9	mRNA	XR_001436817.1	lncRNA
XM_012184887.2	DOCK9	mRNA	XR_001436817.1	lncRNA
XM_012184887.2	DOCK9	mRNA	XR_001436817.1	lncRNA
XM_012184887.2	DOCK9	mRNA	XR_001436817.1	lncRNA
XM_012184887.2	DOCK9	mRNA	XR_001436817.1	lncRNA

XM_012184887.2	DOCK9	mRNA	XR_001436817.1	lncRNA
XM_012184887.2	DOCK9	mRNA	XR_001436817.1	lncRNA
XM_012184887.2	DOCK9	mRNA	XR_001436817.1	lncRNA
XM_012184887.2	DOCK9	mRNA	XR_001436817.1	lncRNA
XM_012184887.2	DOCK9	mRNA	XR_001436817.1	lncRNA
XM_012184887.2	DOCK9	mRNA	XR_001436817.1	lncRNA
XM_012184887.2	DOCK9	mRNA	XR_001436817.1	lncRNA
XM_012152472.1	LUZP1	mRNA	XR_001434203.1	lncRNA
XM_012152472.1	LUZP1	mRNA	XR_001434203.1	lncRNA
XM_012152472.1	LUZP1	mRNA	XR_001434203.1	lncRNA
XM_012152472.1	LUZP1	mRNA	XR_001434203.1	lncRNA
XM_012152472.1	LUZP1	mRNA	XR_001434203.1	lncRNA
XM_012152472.1	LUZP1	mRNA	XR_001434203.1	lncRNA
XM_012152472.1	LUZP1	mRNA	XR_001434203.1	lncRNA
XM_012152472.1	LUZP1	mRNA	XR_001434203.1	lncRNA
XM_012152472.1	LUZP1	mRNA	XR_001434203.1	lncRNA
XM_012152472.1	LUZP1	mRNA	XR_001434203.1	lncRNA
XM_012152472.1	LUZP1	mRNA	XR_001434203.1	lncRNA
XM_012152472.1	LUZP1	mRNA	XR_001434203.1	lncRNA
XM_012152472.1	LUZP1	mRNA	XR_001434203.1	lncRNA
XM_012152472.1	LUZP1	mRNA	XR_001434203.1	lncRNA
XM_012152472.1	LUZP1	mRNA	XR_001434203.1	lncRNA
XM_015098618.1	DLG4	mRNA	XR_001436817.1	lncRNA
XM_015098618.1	DLG4	mRNA	XR_001436817.1	lncRNA
XM_015098618.1	DLG4	mRNA	XR_001436817.1	lncRNA
XM_015098618.1	DLG4	mRNA	XR_001436817.1	lncRNA
XM_015098618.1	DLG4	mRNA	XR_001436817.1	lncRNA
XM_015098618.1	DLG4	mRNA	XR_001436817.1	lncRNA
XM_015098618.1	DLG4	mRNA	XR_001436817.1	lncRNA
XM_015098618.1	DLG4	mRNA	XR_001436817.1	lncRNA
XM_015098618.1	DLG4	mRNA	XR_001436817.1	lncRNA
XM_015098618.1	DLG4	mRNA	XR_001436817.1	lncRNA
XM_015098618.1	DLG4	mRNA	XR_001436817.1	lncRNA
XM_015098618.1	DLG4	mRNA	XR_001436817.1	lncRNA
XM_015098618.1	DLG4	mRNA	XR_001436817.1	lncRNA
XM_015098618.1	DLG4	mRNA	XR_001436817.1	lncRNA
XM_015098618.1	DLG4	mRNA	XR_001436817.1	lncRNA
XM_012184887.2	DOCK9	mRNA	XR_001434203.1	lncRNA
XM_012184887.2	DOCK9	mRNA	XR_001434203.1	lncRNA
XM_012184887.2	DOCK9	mRNA	XR_001434203.1	lncRNA
XM_012184887.2	DOCK9	mRNA	XR_001434203.1	lncRNA
XM_012184887.2	DOCK9	mRNA	XR_001434203.1	lncRNA
XM_012184887.2	DOCK9	mRNA	XR_001434203.1	lncRNA
XM_012184887.2	DOCK9	mRNA	XR_001434203.1	lncRNA
XM_012184887.2	DOCK9	mRNA	XR_001434203.1	lncRNA
XM_012184887.2	DOCK9	mRNA	XR_001434203.1	lncRNA
XM_012184887.2	DOCK9	mRNA	XR_001434203.1	lncRNA
XM_012184887.2	DOCK9	mRNA	XR_001434203.1	lncRNA
XM_012184887.2	DOCK9	mRNA	XR_001434203.1	lncRNA
XM_012184887.2	DOCK9	mRNA	XR_001434203.1	lncRNA
XM_012184887.2	DOCK9	mRNA	XR_001434203.1	lncRNA
XM_012184887.2	DOCK9	mRNA	XR_001434203.1	lncRNA
XM_012152472.1	LUZP1	mRNA	XR_001436817.1	lncRNA
XM_012152472.1	LUZP1	mRNA	XR_001436817.1	lncRNA

XM_012152472.1	LUZP1	mRNA	XR_001436817.1	lncRNA
XM_012152472.1	LUZP1	mRNA	XR_001436817.1	lncRNA
XM_012152472.1	LUZP1	mRNA	XR_001436817.1	lncRNA
XM_012152472.1	LUZP1	mRNA	XR_001436817.1	lncRNA
XM_012152472.1	LUZP1	mRNA	XR_001436817.1	lncRNA
XM_012152472.1	LUZP1	mRNA	XR_001436817.1	lncRNA
XM_012152472.1	LUZP1	mRNA	XR_001436817.1	lncRNA
XM_012152472.1	LUZP1	mRNA	XR_001436817.1	lncRNA
XM_012152472.1	LUZP1	mRNA	XR_001436817.1	lncRNA
XM_012152472.1	LUZP1	mRNA	XR_001436817.1	lncRNA
XM_012152472.1	LUZP1	mRNA	XR_001436817.1	lncRNA
XM_012152472.1	LUZP1	mRNA	XR_001436817.1	lncRNA
XM_012152472.1	LUZP1	mRNA	XR_001436817.1	lncRNA
XM_015098618.1	DLG4	mRNA	XR_001434203.1	lncRNA
XM_015098618.1	DLG4	mRNA	XR_001434203.1	lncRNA
XM_015098618.1	DLG4	mRNA	XR_001434203.1	lncRNA
XM_015098618.1	DLG4	mRNA	XR_001434203.1	lncRNA
XM_015098618.1	DLG4	mRNA	XR_001434203.1	lncRNA
XM_015098618.1	DLG4	mRNA	XR_001434203.1	lncRNA
XM_015098618.1	DLG4	mRNA	XR_001434203.1	lncRNA
XM_015098618.1	DLG4	mRNA	XR_001434203.1	lncRNA
XM_015098618.1	DLG4	mRNA	XR_001434203.1	lncRNA
XM_015098618.1	DLG4	mRNA	XR_001434203.1	lncRNA
XM_015098618.1	DLG4	mRNA	XR_001434203.1	lncRNA
XM_015098618.1	DLG4	mRNA	XR_001434203.1	lncRNA
XM_015098618.1	DLG4	mRNA	XR_001434203.1	lncRNA
XM_015098618.1	DLG4	mRNA	XR_001434203.1	lncRNA
XM_015098618.1	DLG4	mRNA	XR_001434203.1	lncRNA
XM_012178667.2	ARHGAP26	mRNA	XR_001042381.2	lncRNA
XM_012178667.2	ARHGAP26	mRNA	XR_001042381.2	lncRNA
XM_012178667.2	ARHGAP26	mRNA	XR_001042381.2	lncRNA
XM_012178667.2	ARHGAP26	mRNA	XR_001042381.2	lncRNA
XM_012178667.2	ARHGAP26	mRNA	XR_001042381.2	lncRNA
XM_012178667.2	ARHGAP26	mRNA	XR_001042381.2	lncRNA
XM_012178667.2	ARHGAP26	mRNA	XR_001042381.2	lncRNA
XM_012178667.2	ARHGAP26	mRNA	XR_001042381.2	lncRNA
XM_012178667.2	ARHGAP26	mRNA	XR_001042381.2	lncRNA
XM_012178667.2	ARHGAP26	mRNA	XR_001042381.2	lncRNA
XM_012178667.2	ARHGAP26	mRNA	XR_001042381.2	lncRNA
XM_012178667.2	ARHGAP26	mRNA	XR_001042381.2	lncRNA
XM_012178667.2	ARHGAP26	mRNA	XR_001042381.2	lncRNA
XM_012178667.2	ARHGAP26	mRNA	XR_001042381.2	lncRNA
XM_012178667.2	ARHGAP26	mRNA	XR_001042381.2	lncRNA
XM_012144682.2	MAP2	mRNA	XR_001436102.1	lncRNA
XM_012144682.2	MAP2	mRNA	XR_001436102.1	lncRNA
XM_012144682.2	MAP2	mRNA	XR_001436102.1	lncRNA
XM_012144682.2	MAP2	mRNA	XR_001436102.1	lncRNA
XM_012144682.2	MAP2	mRNA	XR_001436102.1	lncRNA
XM_012144682.2	MAP2	mRNA	XR_001436102.1	lncRNA
XM_012144682.2	MAP2	mRNA	XR_001436102.1	lncRNA
XM_012144682.2	MAP2	mRNA	XR_001436102.1	lncRNA
XM_012144682.2	MAP2	mRNA	XR_001436102.1	lncRNA
XM_012144682.2	MAP2	mRNA	XR_001436102.1	lncRNA
XM_012144682.2	MAP2	mRNA	XR_001436102.1	lncRNA

XM_012144682.2	MAP2	mRNA	XR_001436102.1	lncRNA
XM_012144682.2	MAP2	mRNA	XR_001436102.1	lncRNA
XM_012144682.2	MAP2	mRNA	XR_001436102.1	lncRNA
XM_012100690.1	KLC4	mRNA	XR_001434761.1	lncRNA
XM_012100690.1	KLC4	mRNA	XR_001434761.1	lncRNA
XM_012100690.1	KLC4	mRNA	XR_001434761.1	lncRNA
XM_012100690.1	KLC4	mRNA	XR_001434761.1	lncRNA
XM_012100690.1	KLC4	mRNA	XR_001434761.1	lncRNA
XM_012100690.1	KLC4	mRNA	XR_001434761.1	lncRNA
XM_012100690.1	KLC4	mRNA	XR_001434761.1	lncRNA
XM_012100690.1	KLC4	mRNA	XR_001434761.1	lncRNA
XM_012100690.1	KLC4	mRNA	XR_001434761.1	lncRNA
XM_012100690.1	KLC4	mRNA	XR_001434761.1	lncRNA
XM_012100690.1	KLC4	mRNA	XR_001434761.1	lncRNA
XM_012100690.1	KLC4	mRNA	XR_001434761.1	lncRNA
XM_012100690.1	KLC4	mRNA	XR_001434761.1	lncRNA
XM_012100690.1	KLC4	mRNA	XR_001434761.1	lncRNA
XM_012096323.1	PHF21A	mRNA	XR_001042381.2	lncRNA
XM_012096323.1	PHF21A	mRNA	XR_001042381.2	lncRNA
XM_012096323.1	PHF21A	mRNA	XR_001042381.2	lncRNA
XM_012096323.1	PHF21A	mRNA	XR_001042381.2	lncRNA
XM_012096323.1	PHF21A	mRNA	XR_001042381.2	lncRNA
XM_012096323.1	PHF21A	mRNA	XR_001042381.2	lncRNA
XM_012096323.1	PHF21A	mRNA	XR_001042381.2	lncRNA
XM_012096323.1	PHF21A	mRNA	XR_001042381.2	lncRNA
XM_012096323.1	PHF21A	mRNA	XR_001042381.2	lncRNA
XM_012096323.1	PHF21A	mRNA	XR_001042381.2	lncRNA
XM_012096323.1	PHF21A	mRNA	XR_001042381.2	lncRNA
XM_012096323.1	PHF21A	mRNA	XR_001042381.2	lncRNA
XM_012096323.1	PHF21A	mRNA	XR_001042381.2	lncRNA
XM_012096323.1	PHF21A	mRNA	XR_001042381.2	lncRNA
XM_012096323.1	PHF21A	mRNA	XR_001042381.2	lncRNA
XM_012180879.2	MYO9A	mRNA	XR_001042381.2	lncRNA
XM_012180879.2	MYO9A	mRNA	XR_001042381.2	lncRNA
XM_012180879.2	MYO9A	mRNA	XR_001042381.2	lncRNA
XM_012180879.2	MYO9A	mRNA	XR_001042381.2	lncRNA
XM_012180879.2	MYO9A	mRNA	XR_001042381.2	lncRNA
XM_012180879.2	MYO9A	mRNA	XR_001042381.2	lncRNA
XM_012180879.2	MYO9A	mRNA	XR_001042381.2	lncRNA
XM_012180879.2	MYO9A	mRNA	XR_001042381.2	lncRNA
XM_012180879.2	MYO9A	mRNA	XR_001042381.2	lncRNA
XM_012180879.2	MYO9A	mRNA	XR_001042381.2	lncRNA
XM_012180879.2	MYO9A	mRNA	XR_001042381.2	lncRNA
XM_012180879.2	MYO9A	mRNA	XR_001042381.2	lncRNA
XM_012180879.2	MYO9A	mRNA	XR_001042381.2	lncRNA
XM_012180879.2	MYO9A	mRNA	XR_001042381.2	lncRNA
XM_012180879.2	MYO9A	mRNA	XR_001042381.2	lncRNA
XM_012144682.2	MAP2	mRNA	XR_001436817.1	lncRNA
XM_012144682.2	MAP2	mRNA	XR_001436817.1	lncRNA
XM_012144682.2	MAP2	mRNA	XR_001436817.1	lncRNA
XM_012144682.2	MAP2	mRNA	XR_001436817.1	lncRNA
XM_012144682.2	MAP2	mRNA	XR_001436817.1	lncRNA
XM_012144682.2	MAP2	mRNA	XR_001436817.1	lncRNA
XM_012144682.2	MAP2	mRNA	XR_001436817.1	lncRNA

XM_012144682.2	MAP2	mRNA	XR_001436817.1	lncRNA
XM_012144682.2	MAP2	mRNA	XR_001436817.1	lncRNA
XM_012144682.2	MAP2	mRNA	XR_001436817.1	lncRNA
XM_012144682.2	MAP2	mRNA	XR_001436817.1	lncRNA
XM_012144682.2	MAP2	mRNA	XR_001436817.1	lncRNA
XM_012144682.2	MAP2	mRNA	XR_001436817.1	lncRNA
XM_012144682.2	MAP2	mRNA	XR_001436817.1	lncRNA
XM_012144682.2	MAP2	mRNA	XR_001436817.1	lncRNA
XM_015091997.1	LOC10112163S	mRNA	XR_001436817.1	lncRNA
XM_015091997.1	LOC10112163S	mRNA	XR_001436817.1	lncRNA
XM_015091997.1	LOC10112163S	mRNA	XR_001436817.1	lncRNA
XM_015091997.1	LOC10112163S	mRNA	XR_001436817.1	lncRNA
XM_015091997.1	LOC10112163S	mRNA	XR_001436817.1	lncRNA
XM_015091997.1	LOC10112163S	mRNA	XR_001436817.1	lncRNA
XM_015091997.1	LOC10112163S	mRNA	XR_001436817.1	lncRNA
XM_015091997.1	LOC10112163S	mRNA	XR_001436817.1	lncRNA
XM_015091997.1	LOC10112163S	mRNA	XR_001436817.1	lncRNA
XM_015091997.1	LOC10112163S	mRNA	XR_001436817.1	lncRNA
XM_015091997.1	LOC10112163S	mRNA	XR_001436817.1	lncRNA
XM_015091997.1	LOC10112163S	mRNA	XR_001436817.1	lncRNA
XM_015091997.1	LOC10112163S	mRNA	XR_001436817.1	lncRNA
XM_015091997.1	LOC10112163S	mRNA	XR_001436817.1	lncRNA
XM_015091997.1	LOC10112163S	mRNA	XR_001436817.1	lncRNA
XM_012178667.2	ARHGAP26	mRNA	XR_001436817.1	lncRNA
XM_012178667.2	ARHGAP26	mRNA	XR_001436817.1	lncRNA
XM_012178667.2	ARHGAP26	mRNA	XR_001436817.1	lncRNA
XM_012178667.2	ARHGAP26	mRNA	XR_001436817.1	lncRNA
XM_012178667.2	ARHGAP26	mRNA	XR_001436817.1	lncRNA
XM_012178667.2	ARHGAP26	mRNA	XR_001436817.1	lncRNA
XM_012178667.2	ARHGAP26	mRNA	XR_001436817.1	lncRNA
XM_012178667.2	ARHGAP26	mRNA	XR_001436817.1	lncRNA
XM_012178667.2	ARHGAP26	mRNA	XR_001436817.1	lncRNA
XM_012178667.2	ARHGAP26	mRNA	XR_001436817.1	lncRNA
XM_012178667.2	ARHGAP26	mRNA	XR_001436817.1	lncRNA
XM_012178667.2	ARHGAP26	mRNA	XR_001436817.1	lncRNA
XM_012178667.2	ARHGAP26	mRNA	XR_001436817.1	lncRNA
XM_012178667.2	ARHGAP26	mRNA	XR_001436817.1	lncRNA
XM_012178667.2	ARHGAP26	mRNA	XR_001436817.1	lncRNA
XM_015091997.1	LOC10112163S	mRNA	XR_001434203.1	lncRNA
XM_015091997.1	LOC10112163S	mRNA	XR_001434203.1	lncRNA
XM_015091997.1	LOC10112163S	mRNA	XR_001434203.1	lncRNA
XM_015091997.1	LOC10112163S	mRNA	XR_001434203.1	lncRNA
XM_015091997.1	LOC10112163S	mRNA	XR_001434203.1	lncRNA
XM_015091997.1	LOC10112163S	mRNA	XR_001434203.1	lncRNA
XM_015091997.1	LOC10112163S	mRNA	XR_001434203.1	lncRNA
XM_015091997.1	LOC10112163S	mRNA	XR_001434203.1	lncRNA
XM_015091997.1	LOC10112163S	mRNA	XR_001434203.1	lncRNA
XM_015091997.1	LOC10112163S	mRNA	XR_001434203.1	lncRNA
XM_015091997.1	LOC10112163S	mRNA	XR_001434203.1	lncRNA
XM_015091997.1	LOC10112163S	mRNA	XR_001434203.1	lncRNA
XM_015091997.1	LOC10112163S	mRNA	XR_001434203.1	lncRNA
XM_015091997.1	LOC10112163S	mRNA	XR_001434203.1	lncRNA
XM_015091997.1	LOC10112163S	mRNA	XR_001434203.1	lncRNA
XM_015103448.1	TACC2	mRNA	XR_001434761.1	lncRNA

XM_015103448.1	TACC2	mRNA	XR_001434761.1	lncRNA
XM_015103448.1	TACC2	mRNA	XR_001434761.1	lncRNA
XM_015103448.1	TACC2	mRNA	XR_001434761.1	lncRNA
XM_015103448.1	TACC2	mRNA	XR_001434761.1	lncRNA
XM_015103448.1	TACC2	mRNA	XR_001434761.1	lncRNA
XM_015103448.1	TACC2	mRNA	XR_001434761.1	lncRNA
XM_015103448.1	TACC2	mRNA	XR_001434761.1	lncRNA
XM_015103448.1	TACC2	mRNA	XR_001434761.1	lncRNA
XM_015103448.1	TACC2	mRNA	XR_001434761.1	lncRNA
XM_015103448.1	TACC2	mRNA	XR_001434761.1	lncRNA
XM_015103448.1	TACC2	mRNA	XR_001434761.1	lncRNA
XM_015103448.1	TACC2	mRNA	XR_001434761.1	lncRNA
XM_015103448.1	TACC2	mRNA	XR_001434761.1	lncRNA
XM_015103448.1	TACC2	mRNA	XR_001434761.1	lncRNA
XM_012183728.2	LOC101111337	mRNA	XR_001436532.1	lncRNA
XM_012183728.2	LOC101111337	mRNA	XR_001436532.1	lncRNA
XM_012183728.2	LOC101111337	mRNA	XR_001436532.1	lncRNA
XM_012183728.2	LOC101111337	mRNA	XR_001436532.1	lncRNA
XM_012183728.2	LOC101111337	mRNA	XR_001436532.1	lncRNA
XM_012183728.2	LOC101111337	mRNA	XR_001436532.1	lncRNA
XM_012183728.2	LOC101111337	mRNA	XR_001436532.1	lncRNA
XM_012183728.2	LOC101111337	mRNA	XR_001436532.1	lncRNA
XM_012183728.2	LOC101111337	mRNA	XR_001436532.1	lncRNA
XM_012183728.2	LOC101111337	mRNA	XR_001436532.1	lncRNA
XM_012183728.2	LOC101111337	mRNA	XR_001436532.1	lncRNA
XM_012183728.2	LOC101111337	mRNA	XR_001436532.1	lncRNA
XM_012183728.2	LOC101111337	mRNA	XR_001436532.1	lncRNA
XM_012183728.2	LOC101111337	mRNA	XR_001436532.1	lncRNA
XM_012137425.2	NEB	mRNA	XR_001021689.2	lncRNA
XM_012137425.2	NEB	mRNA	XR_001021689.2	lncRNA
XM_012137425.2	NEB	mRNA	XR_001021689.2	lncRNA
XM_012137425.2	NEB	mRNA	XR_001021689.2	lncRNA
XM_012137425.2	NEB	mRNA	XR_001021689.2	lncRNA
XM_012137425.2	NEB	mRNA	XR_001021689.2	lncRNA
XM_012137425.2	NEB	mRNA	XR_001021689.2	lncRNA
XM_012137425.2	NEB	mRNA	XR_001021689.2	lncRNA
XM_012137425.2	NEB	mRNA	XR_001021689.2	lncRNA
XM_012137425.2	NEB	mRNA	XR_001021689.2	lncRNA
XM_012137425.2	NEB	mRNA	XR_001021689.2	lncRNA
XM_012137425.2	NEB	mRNA	XR_001021689.2	lncRNA
XM_012137425.2	NEB	mRNA	XR_001021689.2	lncRNA
XM_012137425.2	NEB	mRNA	XR_001021689.2	lncRNA
XM_012137434.2	NEB	mRNA	XR_001021689.2	lncRNA
XM_012137434.2	NEB	mRNA	XR_001021689.2	lncRNA
XM_012137434.2	NEB	mRNA	XR_001021689.2	lncRNA
XM_012137434.2	NEB	mRNA	XR_001021689.2	lncRNA
XM_012137434.2	NEB	mRNA	XR_001021689.2	lncRNA
XM_012137434.2	NEB	mRNA	XR_001021689.2	lncRNA
XM_012137434.2	NEB	mRNA	XR_001021689.2	lncRNA
XM_012137434.2	NEB	mRNA	XR_001021689.2	lncRNA
XM_012137434.2	NEB	mRNA	XR_001021689.2	lncRNA
XM_012137434.2	NEB	mRNA	XR_001021689.2	lncRNA
XM_012137434.2	NEB	mRNA	XR_001021689.2	lncRNA
XM_012137434.2	NEB	mRNA	XR_001021689.2	lncRNA

XM_012137434.2	NEB	mRNA	XR_001021689.2	lncRNA
XM_012137434.2	NEB	mRNA	XR_001021689.2	lncRNA
XM_012137425.2	NEB	mRNA	XR_001436102.1	lncRNA
XM_012137425.2	NEB	mRNA	XR_001436102.1	lncRNA
XM_012137425.2	NEB	mRNA	XR_001436102.1	lncRNA
XM_012137425.2	NEB	mRNA	XR_001436102.1	lncRNA
XM_012137425.2	NEB	mRNA	XR_001436102.1	lncRNA
XM_012137425.2	NEB	mRNA	XR_001436102.1	lncRNA
XM_012137425.2	NEB	mRNA	XR_001436102.1	lncRNA
XM_012137425.2	NEB	mRNA	XR_001436102.1	lncRNA
XM_012137425.2	NEB	mRNA	XR_001436102.1	lncRNA
XM_012137425.2	NEB	mRNA	XR_001436102.1	lncRNA
XM_012137425.2	NEB	mRNA	XR_001436102.1	lncRNA
XM_012137425.2	NEB	mRNA	XR_001436102.1	lncRNA
XM_012137425.2	NEB	mRNA	XR_001436102.1	lncRNA
XM_012137425.2	NEB	mRNA	XR_001436102.1	lncRNA
XM_012174704.2	ZBTB39	mRNA	XR_001042381.2	lncRNA
XM_012174704.2	ZBTB39	mRNA	XR_001042381.2	lncRNA
XM_012174704.2	ZBTB39	mRNA	XR_001042381.2	lncRNA
XM_012174704.2	ZBTB39	mRNA	XR_001042381.2	lncRNA
XM_012174704.2	ZBTB39	mRNA	XR_001042381.2	lncRNA
XM_012174704.2	ZBTB39	mRNA	XR_001042381.2	lncRNA
XM_012174704.2	ZBTB39	mRNA	XR_001042381.2	lncRNA
XM_012174704.2	ZBTB39	mRNA	XR_001042381.2	lncRNA
XM_012174704.2	ZBTB39	mRNA	XR_001042381.2	lncRNA
XM_012174704.2	ZBTB39	mRNA	XR_001042381.2	lncRNA
XM_012174704.2	ZBTB39	mRNA	XR_001042381.2	lncRNA
XM_012174704.2	ZBTB39	mRNA	XR_001042381.2	lncRNA
XM_012174704.2	ZBTB39	mRNA	XR_001042381.2	lncRNA
XM_012174704.2	ZBTB39	mRNA	XR_001042381.2	lncRNA
XM_012174704.2	ZBTB39	mRNA	XR_001042381.2	lncRNA
XM_012143523.2	INO80D	mRNA	XR_001434761.1	lncRNA
XM_012143523.2	INO80D	mRNA	XR_001434761.1	lncRNA
XM_012143523.2	INO80D	mRNA	XR_001434761.1	lncRNA
XM_012143523.2	INO80D	mRNA	XR_001434761.1	lncRNA
XM_012143523.2	INO80D	mRNA	XR_001434761.1	lncRNA
XM_012143523.2	INO80D	mRNA	XR_001434761.1	lncRNA
XM_012143523.2	INO80D	mRNA	XR_001434761.1	lncRNA
XM_012143523.2	INO80D	mRNA	XR_001434761.1	lncRNA
XM_012143523.2	INO80D	mRNA	XR_001434761.1	lncRNA
XM_012143523.2	INO80D	mRNA	XR_001434761.1	lncRNA
XM_012143523.2	INO80D	mRNA	XR_001434761.1	lncRNA
XM_012143523.2	INO80D	mRNA	XR_001434761.1	lncRNA
XM_012143523.2	INO80D	mRNA	XR_001434761.1	lncRNA
XM_012143523.2	INO80D	mRNA	XR_001434761.1	lncRNA
XM_012143523.2	INO80D	mRNA	XR_001434761.1	lncRNA
XM_012178308.2	FAM193B	mRNA	XR_001041211.2	lncRNA
XM_012178308.2	FAM193B	mRNA	XR_001041211.2	lncRNA
XM_012178308.2	FAM193B	mRNA	XR_001041211.2	lncRNA
XM_012178308.2	FAM193B	mRNA	XR_001041211.2	lncRNA
XM_012178308.2	FAM193B	mRNA	XR_001041211.2	lncRNA
XM_012178308.2	FAM193B	mRNA	XR_001041211.2	lncRNA
XM_012178308.2	FAM193B	mRNA	XR_001041211.2	lncRNA
XM_012178308.2	FAM193B	mRNA	XR_001041211.2	lncRNA

XM_012178308.2	FAM193B	mRNA	XR_001041211.2	lncRNA
XM_012178308.2	FAM193B	mRNA	XR_001041211.2	lncRNA
XM_012178308.2	FAM193B	mRNA	XR_001041211.2	lncRNA
XM_012178308.2	FAM193B	mRNA	XR_001041211.2	lncRNA
XM_012178308.2	FAM193B	mRNA	XR_001041211.2	lncRNA
XM_012178308.2	FAM193B	mRNA	XR_001041211.2	lncRNA
XM_012175045.1	FGD4	mRNA	XR_001041211.2	lncRNA
XM_012175045.1	FGD4	mRNA	XR_001041211.2	lncRNA
XM_012175045.1	FGD4	mRNA	XR_001041211.2	lncRNA
XM_012175045.1	FGD4	mRNA	XR_001041211.2	lncRNA
XM_012175045.1	FGD4	mRNA	XR_001041211.2	lncRNA
XM_012175045.1	FGD4	mRNA	XR_001041211.2	lncRNA
XM_012175045.1	FGD4	mRNA	XR_001041211.2	lncRNA
XM_012175045.1	FGD4	mRNA	XR_001041211.2	lncRNA
XM_012175045.1	FGD4	mRNA	XR_001041211.2	lncRNA
XM_012175045.1	FGD4	mRNA	XR_001041211.2	lncRNA
XM_012175045.1	FGD4	mRNA	XR_001041211.2	lncRNA
XM_012175045.1	FGD4	mRNA	XR_001041211.2	lncRNA
XM_012175045.1	FGD4	mRNA	XR_001041211.2	lncRNA
XM_012175045.1	FGD4	mRNA	XR_001041211.2	lncRNA
XM_015099551.1	PARD3	mRNA	XR_001041211.2	lncRNA
XM_015099551.1	PARD3	mRNA	XR_001041211.2	lncRNA
XM_015099551.1	PARD3	mRNA	XR_001041211.2	lncRNA
XM_015099551.1	PARD3	mRNA	XR_001041211.2	lncRNA
XM_015099551.1	PARD3	mRNA	XR_001041211.2	lncRNA
XM_015099551.1	PARD3	mRNA	XR_001041211.2	lncRNA
XM_015099551.1	PARD3	mRNA	XR_001041211.2	lncRNA
XM_015099551.1	PARD3	mRNA	XR_001041211.2	lncRNA
XM_015099551.1	PARD3	mRNA	XR_001041211.2	lncRNA
XM_015099551.1	PARD3	mRNA	XR_001041211.2	lncRNA
XM_015099551.1	PARD3	mRNA	XR_001041211.2	lncRNA
XM_015099551.1	PARD3	mRNA	XR_001041211.2	lncRNA
XM_015099551.1	PARD3	mRNA	XR_001041211.2	lncRNA
XM_015099551.1	PARD3	mRNA	XR_001041211.2	lncRNA
XM_015091836.1	USP40	mRNA	XR_001434203.1	lncRNA
XM_015091836.1	USP40	mRNA	XR_001434203.1	lncRNA
XM_015091836.1	USP40	mRNA	XR_001434203.1	lncRNA
XM_015091836.1	USP40	mRNA	XR_001434203.1	lncRNA
XM_015091836.1	USP40	mRNA	XR_001434203.1	lncRNA
XM_015091836.1	USP40	mRNA	XR_001434203.1	lncRNA
XM_015091836.1	USP40	mRNA	XR_001434203.1	lncRNA
XM_015091836.1	USP40	mRNA	XR_001434203.1	lncRNA
XM_015091836.1	USP40	mRNA	XR_001434203.1	lncRNA
XM_015091836.1	USP40	mRNA	XR_001434203.1	lncRNA
XM_015091836.1	USP40	mRNA	XR_001434203.1	lncRNA
XM_015091836.1	USP40	mRNA	XR_001434203.1	lncRNA
XM_015091836.1	USP40	mRNA	XR_001434203.1	lncRNA
XM_015091836.1	USP40	mRNA	XR_001434203.1	lncRNA
XM_015091836.1	USP40	mRNA	XR_001434203.1	lncRNA
XM_015091836.1	USP40	mRNA	XR_001436817.1	lncRNA
XM_015091836.1	USP40	mRNA	XR_001436817.1	lncRNA
XM_015091836.1	USP40	mRNA	XR_001436817.1	lncRNA
XM_015091836.1	USP40	mRNA	XR_001436817.1	lncRNA
XM_015091836.1	USP40	mRNA	XR_001436817.1	lncRNA

XM_015091836.1	USP40	mRNA	XR_001436817.1	lncRNA
XM_015091836.1	USP40	mRNA	XR_001436817.1	lncRNA
XM_015091836.1	USP40	mRNA	XR_001436817.1	lncRNA
XM_015091836.1	USP40	mRNA	XR_001436817.1	lncRNA
XM_015091836.1	USP40	mRNA	XR_001436817.1	lncRNA
XM_015091836.1	USP40	mRNA	XR_001436817.1	lncRNA
XM_015091836.1	USP40	mRNA	XR_001436817.1	lncRNA
XM_015091836.1	USP40	mRNA	XR_001436817.1	lncRNA
XM_015091836.1	USP40	mRNA	XR_001436817.1	lncRNA
XM_015091836.1	USP40	mRNA	XR_001436817.1	lncRNA
XM_015102486.1	NR2C2	mRNA	XR_001434203.1	lncRNA
XM_015102486.1	NR2C2	mRNA	XR_001434203.1	lncRNA
XM_015102486.1	NR2C2	mRNA	XR_001434203.1	lncRNA
XM_015102486.1	NR2C2	mRNA	XR_001434203.1	lncRNA
XM_015102486.1	NR2C2	mRNA	XR_001434203.1	lncRNA
XM_015102486.1	NR2C2	mRNA	XR_001434203.1	lncRNA
XM_015102486.1	NR2C2	mRNA	XR_001434203.1	lncRNA
XM_015102486.1	NR2C2	mRNA	XR_001434203.1	lncRNA
XM_015102486.1	NR2C2	mRNA	XR_001434203.1	lncRNA
XM_015102486.1	NR2C2	mRNA	XR_001434203.1	lncRNA
XM_015102486.1	NR2C2	mRNA	XR_001434203.1	lncRNA
XM_015102486.1	NR2C2	mRNA	XR_001434203.1	lncRNA
XM_015102486.1	NR2C2	mRNA	XR_001434203.1	lncRNA
XM_015102486.1	NR2C2	mRNA	XR_001434203.1	lncRNA
XM_015102486.1	NR2C2	mRNA	XR_001434203.1	lncRNA
XM_012177309.2	LOC10111622(	mRNA	XR_001436817.1	lncRNA
XM_012177309.2	LOC10111622(	mRNA	XR_001436817.1	lncRNA
XM_012177309.2	LOC10111622(	mRNA	XR_001436817.1	lncRNA
XM_012177309.2	LOC10111622(	mRNA	XR_001436817.1	lncRNA
XM_012177309.2	LOC10111622(	mRNA	XR_001436817.1	lncRNA
XM_012177309.2	LOC10111622(	mRNA	XR_001436817.1	lncRNA
XM_012177309.2	LOC10111622(	mRNA	XR_001436817.1	lncRNA
XM_012177309.2	LOC10111622(	mRNA	XR_001436817.1	lncRNA
XM_012177309.2	LOC10111622(	mRNA	XR_001436817.1	lncRNA
XM_012177309.2	LOC10111622(	mRNA	XR_001436817.1	lncRNA
XM_012177309.2	LOC10111622(	mRNA	XR_001436817.1	lncRNA
XM_012177309.2	LOC10111622(	mRNA	XR_001436817.1	lncRNA
XM_012177309.2	LOC10111622(	mRNA	XR_001436817.1	lncRNA
XM_012177309.2	LOC10111622(	mRNA	XR_001436817.1	lncRNA
XM_012177309.2	LOC10111622(	mRNA	XR_001436817.1	lncRNA
XM_012105688.2	WDR17	mRNA	XR_001041211.2	lncRNA
XM_012105688.2	WDR17	mRNA	XR_001041211.2	lncRNA
XM_012105688.2	WDR17	mRNA	XR_001041211.2	lncRNA
XM_012105688.2	WDR17	mRNA	XR_001041211.2	lncRNA
XM_012105688.2	WDR17	mRNA	XR_001041211.2	lncRNA
XM_012105688.2	WDR17	mRNA	XR_001041211.2	lncRNA
XM_012105688.2	WDR17	mRNA	XR_001041211.2	lncRNA
XM_012105688.2	WDR17	mRNA	XR_001041211.2	lncRNA
XM_012105688.2	WDR17	mRNA	XR_001041211.2	lncRNA
XM_012105688.2	WDR17	mRNA	XR_001041211.2	lncRNA
XM_012105688.2	WDR17	mRNA	XR_001041211.2	lncRNA
XM_012105688.2	WDR17	mRNA	XR_001041211.2	lncRNA
XM_012105688.2	WDR17	mRNA	XR_001041211.2	lncRNA
XM_012105688.2	WDR17	mRNA	XR_001041211.2	lncRNA

XM_012105688.2	WDR17	mRNA	XR_001041211.2	lncRNA
XM_012102845.2	GBF1	mRNA	XR_001021689.2	lncRNA
XM_012102845.2	GBF1	mRNA	XR_001021689.2	lncRNA
XM_012102845.2	GBF1	mRNA	XR_001021689.2	lncRNA
XM_012102845.2	GBF1	mRNA	XR_001021689.2	lncRNA
XM_012102845.2	GBF1	mRNA	XR_001021689.2	lncRNA
XM_012102845.2	GBF1	mRNA	XR_001021689.2	lncRNA
XM_012102845.2	GBF1	mRNA	XR_001021689.2	lncRNA
XM_012102845.2	GBF1	mRNA	XR_001021689.2	lncRNA
XM_012102845.2	GBF1	mRNA	XR_001021689.2	lncRNA
XM_012102845.2	GBF1	mRNA	XR_001021689.2	lncRNA
XM_012102845.2	GBF1	mRNA	XR_001021689.2	lncRNA
XM_012102845.2	GBF1	mRNA	XR_001021689.2	lncRNA
XM_012102845.2	GBF1	mRNA	XR_001021689.2	lncRNA
XM_015102318.1	SETD5	mRNA	XR_001436817.1	lncRNA
XM_015102318.1	SETD5	mRNA	XR_001436817.1	lncRNA
XM_015102318.1	SETD5	mRNA	XR_001436817.1	lncRNA
XM_015102318.1	SETD5	mRNA	XR_001436817.1	lncRNA
XM_015102318.1	SETD5	mRNA	XR_001436817.1	lncRNA
XM_015102318.1	SETD5	mRNA	XR_001436817.1	lncRNA
XM_015102318.1	SETD5	mRNA	XR_001436817.1	lncRNA
XM_015102318.1	SETD5	mRNA	XR_001436817.1	lncRNA
XM_015102318.1	SETD5	mRNA	XR_001436817.1	lncRNA
XM_015102318.1	SETD5	mRNA	XR_001436817.1	lncRNA
XM_015102318.1	SETD5	mRNA	XR_001436817.1	lncRNA
XM_015102318.1	SETD5	mRNA	XR_001436817.1	lncRNA
XM_015102318.1	SETD5	mRNA	XR_001436817.1	lncRNA
XM_015102318.1	SETD5	mRNA	XR_001436817.1	lncRNA
XM_012184884.2	DOCK9	mRNA	XR_001436817.1	lncRNA
XM_012184884.2	DOCK9	mRNA	XR_001436817.1	lncRNA
XM_012184884.2	DOCK9	mRNA	XR_001436817.1	lncRNA
XM_012184884.2	DOCK9	mRNA	XR_001436817.1	lncRNA
XM_012184884.2	DOCK9	mRNA	XR_001436817.1	lncRNA
XM_012184884.2	DOCK9	mRNA	XR_001436817.1	lncRNA
XM_012184884.2	DOCK9	mRNA	XR_001436817.1	lncRNA
XM_012184884.2	DOCK9	mRNA	XR_001436817.1	lncRNA
XM_012184884.2	DOCK9	mRNA	XR_001436817.1	lncRNA
XM_012184884.2	DOCK9	mRNA	XR_001436817.1	lncRNA
XM_012184884.2	DOCK9	mRNA	XR_001436817.1	lncRNA
XM_012184884.2	DOCK9	mRNA	XR_001436817.1	lncRNA
XM_012184884.2	DOCK9	mRNA	XR_001436817.1	lncRNA
XM_012184884.2	DOCK9	mRNA	XR_001436817.1	lncRNA
XM_012183412.2	ZNF7	mRNA	XR_001436817.1	lncRNA
XM_012183412.2	ZNF7	mRNA	XR_001436817.1	lncRNA
XM_012183412.2	ZNF7	mRNA	XR_001436817.1	lncRNA
XM_012183412.2	ZNF7	mRNA	XR_001436817.1	lncRNA
XM_012183412.2	ZNF7	mRNA	XR_001436817.1	lncRNA
XM_012183412.2	ZNF7	mRNA	XR_001436817.1	lncRNA
XM_012183412.2	ZNF7	mRNA	XR_001436817.1	lncRNA
XM_012183412.2	ZNF7	mRNA	XR_001436817.1	lncRNA
XM_012183412.2	ZNF7	mRNA	XR_001436817.1	lncRNA
XM_012183412.2	ZNF7	mRNA	XR_001436817.1	lncRNA
XM_012183412.2	ZNF7	mRNA	XR_001436817.1	lncRNA
XM_012183412.2	ZNF7	mRNA	XR_001436817.1	lncRNA

XM_012183412.2	ZNF7	mRNA	XR_001436817.1	lncRNA
XM_012183412.2	ZNF7	mRNA	XR_001436817.1	lncRNA
XM_012187378.2	CAMTA1	mRNA	XR_001021689.2	lncRNA
XM_012187378.2	CAMTA1	mRNA	XR_001021689.2	lncRNA
XM_012187378.2	CAMTA1	mRNA	XR_001021689.2	lncRNA
XM_012187378.2	CAMTA1	mRNA	XR_001021689.2	lncRNA
XM_012187378.2	CAMTA1	mRNA	XR_001021689.2	lncRNA
XM_012187378.2	CAMTA1	mRNA	XR_001021689.2	lncRNA
XM_012187378.2	CAMTA1	mRNA	XR_001021689.2	lncRNA
XM_012187378.2	CAMTA1	mRNA	XR_001021689.2	lncRNA
XM_012187378.2	CAMTA1	mRNA	XR_001021689.2	lncRNA
XM_012187378.2	CAMTA1	mRNA	XR_001021689.2	lncRNA
XM_012187378.2	CAMTA1	mRNA	XR_001021689.2	lncRNA
XM_012187378.2	CAMTA1	mRNA	XR_001021689.2	lncRNA
XM_012187378.2	CAMTA1	mRNA	XR_001021689.2	lncRNA
XM_012187378.2	CAMTA1	mRNA	XR_001021689.2	lncRNA
XM_015105096.1	PAK3	mRNA	XR_001041211.2	lncRNA
XM_015105096.1	PAK3	mRNA	XR_001041211.2	lncRNA
XM_015105096.1	PAK3	mRNA	XR_001041211.2	lncRNA
XM_015105096.1	PAK3	mRNA	XR_001041211.2	lncRNA
XM_015105096.1	PAK3	mRNA	XR_001041211.2	lncRNA
XM_015105096.1	PAK3	mRNA	XR_001041211.2	lncRNA
XM_015105096.1	PAK3	mRNA	XR_001041211.2	lncRNA
XM_015105096.1	PAK3	mRNA	XR_001041211.2	lncRNA
XM_015105096.1	PAK3	mRNA	XR_001041211.2	lncRNA
XM_015105096.1	PAK3	mRNA	XR_001041211.2	lncRNA
XM_015105096.1	PAK3	mRNA	XR_001041211.2	lncRNA
XM_015105096.1	PAK3	mRNA	XR_001041211.2	lncRNA
XM_015105096.1	PAK3	mRNA	XR_001041211.2	lncRNA
XM_015105096.1	PAK3	mRNA	XR_001041211.2	lncRNA
XM_004005724.3	NCOA1	mRNA	XR_001434892.1	lncRNA
XM_004005724.3	NCOA1	mRNA	XR_001434892.1	lncRNA
XM_004005724.3	NCOA1	mRNA	XR_001434892.1	lncRNA
XM_004005724.3	NCOA1	mRNA	XR_001434892.1	lncRNA
XM_004005724.3	NCOA1	mRNA	XR_001434892.1	lncRNA
XM_004005724.3	NCOA1	mRNA	XR_001434892.1	lncRNA
XM_004005724.3	NCOA1	mRNA	XR_001434892.1	lncRNA
XM_004005724.3	NCOA1	mRNA	XR_001434892.1	lncRNA
XM_004005724.3	NCOA1	mRNA	XR_001434892.1	lncRNA
XM_004005724.3	NCOA1	mRNA	XR_001434892.1	lncRNA
XM_004005724.3	NCOA1	mRNA	XR_001434892.1	lncRNA
XM_004005724.3	NCOA1	mRNA	XR_001434892.1	lncRNA
XM_004005724.3	NCOA1	mRNA	XR_001434892.1	lncRNA
XM_004005724.3	NCOA1	mRNA	XR_001434892.1	lncRNA
XM_015098446.1	VEZF1	mRNA	XR_001434761.1	lncRNA
XM_015098446.1	VEZF1	mRNA	XR_001434761.1	lncRNA
XM_015098446.1	VEZF1	mRNA	XR_001434761.1	lncRNA
XM_015098446.1	VEZF1	mRNA	XR_001434761.1	lncRNA
XM_015098446.1	VEZF1	mRNA	XR_001434761.1	lncRNA
XM_015098446.1	VEZF1	mRNA	XR_001434761.1	lncRNA
XM_015098446.1	VEZF1	mRNA	XR_001434761.1	lncRNA
XM_015098446.1	VEZF1	mRNA	XR_001434761.1	lncRNA
XM_015098446.1	VEZF1	mRNA	XR_001434761.1	lncRNA
XM_015098446.1	VEZF1	mRNA	XR_001434761.1	lncRNA

XM_015098446.1	VEZF1	mRNA	XR_001434761.1	lncRNA
XM_015098446.1	VEZF1	mRNA	XR_001434761.1	lncRNA
XM_015098446.1	VEZF1	mRNA	XR_001434761.1	lncRNA
XM_015098446.1	VEZF1	mRNA	XR_001434761.1	lncRNA
XM_012174394.2	RAPGEF3	mRNA	XR_001436532.1	lncRNA
XM_012174394.2	RAPGEF3	mRNA	XR_001436532.1	lncRNA
XM_012174394.2	RAPGEF3	mRNA	XR_001436532.1	lncRNA
XM_012174394.2	RAPGEF3	mRNA	XR_001436532.1	lncRNA
XM_012174394.2	RAPGEF3	mRNA	XR_001436532.1	lncRNA
XM_012174394.2	RAPGEF3	mRNA	XR_001436532.1	lncRNA
XM_012174394.2	RAPGEF3	mRNA	XR_001436532.1	lncRNA
XM_012174394.2	RAPGEF3	mRNA	XR_001436532.1	lncRNA
XM_012174394.2	RAPGEF3	mRNA	XR_001436532.1	lncRNA
XM_012174394.2	RAPGEF3	mRNA	XR_001436532.1	lncRNA
XM_012174394.2	RAPGEF3	mRNA	XR_001436532.1	lncRNA
XM_012174394.2	RAPGEF3	mRNA	XR_001436532.1	lncRNA
XM_012174394.2	RAPGEF3	mRNA	XR_001436532.1	lncRNA
XM_012174394.2	RAPGEF3	mRNA	XR_001436532.1	lncRNA
XM_012178306.2	FAM193B	mRNA	XR_001436532.1	lncRNA
XM_012178306.2	FAM193B	mRNA	XR_001436532.1	lncRNA
XM_012178306.2	FAM193B	mRNA	XR_001436532.1	lncRNA
XM_012178306.2	FAM193B	mRNA	XR_001436532.1	lncRNA
XM_012178306.2	FAM193B	mRNA	XR_001436532.1	lncRNA
XM_012178306.2	FAM193B	mRNA	XR_001436532.1	lncRNA
XM_012178306.2	FAM193B	mRNA	XR_001436532.1	lncRNA
XM_012178306.2	FAM193B	mRNA	XR_001436532.1	lncRNA
XM_012178306.2	FAM193B	mRNA	XR_001436532.1	lncRNA
XM_012178306.2	FAM193B	mRNA	XR_001436532.1	lncRNA
XM_012178306.2	FAM193B	mRNA	XR_001436532.1	lncRNA
XM_012178306.2	FAM193B	mRNA	XR_001436532.1	lncRNA
XM_012178306.2	FAM193B	mRNA	XR_001436532.1	lncRNA
XM_012178306.2	FAM193B	mRNA	XR_001436532.1	lncRNA
XM_015098529.1	NF1	mRNA	XR_001436817.1	lncRNA
XM_015098529.1	NF1	mRNA	XR_001436817.1	lncRNA
XM_015098529.1	NF1	mRNA	XR_001436817.1	lncRNA
XM_015098529.1	NF1	mRNA	XR_001436817.1	lncRNA
XM_015098529.1	NF1	mRNA	XR_001436817.1	lncRNA
XM_015098529.1	NF1	mRNA	XR_001436817.1	lncRNA
XM_015098529.1	NF1	mRNA	XR_001436817.1	lncRNA
XM_015098529.1	NF1	mRNA	XR_001436817.1	lncRNA
XM_015098529.1	NF1	mRNA	XR_001436817.1	lncRNA
XM_015098529.1	NF1	mRNA	XR_001436817.1	lncRNA
XM_015098529.1	NF1	mRNA	XR_001436817.1	lncRNA
XM_015098529.1	NF1	mRNA	XR_001436817.1	lncRNA
XM_015098529.1	NF1	mRNA	XR_001436817.1	lncRNA
XM_015098529.1	NF1	mRNA	XR_001436817.1	lncRNA
XM_012105688.2	WDR17	mRNA	XR_001434892.1	lncRNA
XM_012105688.2	WDR17	mRNA	XR_001434892.1	lncRNA
XM_012105688.2	WDR17	mRNA	XR_001434892.1	lncRNA
XM_012105688.2	WDR17	mRNA	XR_001434892.1	lncRNA
XM_012105688.2	WDR17	mRNA	XR_001434892.1	lncRNA
XM_012105688.2	WDR17	mRNA	XR_001434892.1	lncRNA
XM_012105688.2	WDR17	mRNA	XR_001434892.1	lncRNA
XM_012105688.2	WDR17	mRNA	XR_001434892.1	lncRNA

XM_012105688.2	WDR17	mRNA	XR_001434892.1	lncRNA
XM_012105688.2	WDR17	mRNA	XR_001434892.1	lncRNA
XM_012105688.2	WDR17	mRNA	XR_001434892.1	lncRNA
XM_012105688.2	WDR17	mRNA	XR_001434892.1	lncRNA
XM_012105688.2	WDR17	mRNA	XR_001434892.1	lncRNA
XM_012105688.2	WDR17	mRNA	XR_001434892.1	lncRNA
XM_012189186.2	RALGAPB	mRNA	XR_001041211.2	lncRNA
XM_012189186.2	RALGAPB	mRNA	XR_001041211.2	lncRNA
XM_012189186.2	RALGAPB	mRNA	XR_001041211.2	lncRNA
XM_012189186.2	RALGAPB	mRNA	XR_001041211.2	lncRNA
XM_012189186.2	RALGAPB	mRNA	XR_001041211.2	lncRNA
XM_012189186.2	RALGAPB	mRNA	XR_001041211.2	lncRNA
XM_012189186.2	RALGAPB	mRNA	XR_001041211.2	lncRNA
XM_012189186.2	RALGAPB	mRNA	XR_001041211.2	lncRNA
XM_012189186.2	RALGAPB	mRNA	XR_001041211.2	lncRNA
XM_012189186.2	RALGAPB	mRNA	XR_001041211.2	lncRNA
XM_012189186.2	RALGAPB	mRNA	XR_001041211.2	lncRNA
XM_012189186.2	RALGAPB	mRNA	XR_001041211.2	lncRNA
XM_012189186.2	RALGAPB	mRNA	XR_001041211.2	lncRNA
XM_012189186.2	RALGAPB	mRNA	XR_001041211.2	lncRNA
XM_012143277.2	ABI2	mRNA	XR_001041211.2	lncRNA
XM_012143277.2	ABI2	mRNA	XR_001041211.2	lncRNA
XM_012143277.2	ABI2	mRNA	XR_001041211.2	lncRNA
XM_012143277.2	ABI2	mRNA	XR_001041211.2	lncRNA
XM_012143277.2	ABI2	mRNA	XR_001041211.2	lncRNA
XM_012143277.2	ABI2	mRNA	XR_001041211.2	lncRNA
XM_012143277.2	ABI2	mRNA	XR_001041211.2	lncRNA
XM_012143277.2	ABI2	mRNA	XR_001041211.2	lncRNA
XM_012143277.2	ABI2	mRNA	XR_001041211.2	lncRNA
XM_012143277.2	ABI2	mRNA	XR_001041211.2	lncRNA
XM_012143277.2	ABI2	mRNA	XR_001041211.2	lncRNA
XM_012143277.2	ABI2	mRNA	XR_001041211.2	lncRNA
XM_012143277.2	ABI2	mRNA	XR_001041211.2	lncRNA
XM_012143277.2	ABI2	mRNA	XR_001041211.2	lncRNA
XM_015100519.1	NFAT5	mRNA	XR_001024290.1	lncRNA
XM_015100519.1	NFAT5	mRNA	XR_001024290.1	lncRNA
XM_015100519.1	NFAT5	mRNA	XR_001024290.1	lncRNA
XM_015100519.1	NFAT5	mRNA	XR_001024290.1	lncRNA
XM_015100519.1	NFAT5	mRNA	XR_001024290.1	lncRNA
XM_015100519.1	NFAT5	mRNA	XR_001024290.1	lncRNA
XM_015100519.1	NFAT5	mRNA	XR_001024290.1	lncRNA
XM_015100519.1	NFAT5	mRNA	XR_001024290.1	lncRNA
XM_015100519.1	NFAT5	mRNA	XR_001024290.1	lncRNA
XM_015100519.1	NFAT5	mRNA	XR_001024290.1	lncRNA
XM_015100519.1	NFAT5	mRNA	XR_001024290.1	lncRNA
XM_015100519.1	NFAT5	mRNA	XR_001024290.1	lncRNA
XM_012182538.2	TRAPPC3L	mRNA	XR_001434761.1	lncRNA
XM_012182538.2	TRAPPC3L	mRNA	XR_001434761.1	lncRNA
XM_012182538.2	TRAPPC3L	mRNA	XR_001434761.1	lncRNA
XM_012182538.2	TRAPPC3L	mRNA	XR_001434761.1	lncRNA
XM_012182538.2	TRAPPC3L	mRNA	XR_001434761.1	lncRNA
XM_012182538.2	TRAPPC3L	mRNA	XR_001434761.1	lncRNA
XM_012182538.2	TRAPPC3L	mRNA	XR_001434761.1	lncRNA
XM_012182538.2	TRAPPC3L	mRNA	XR_001434761.1	lncRNA

XM_012182538.2	TRAPPC3L	mRNA	XR_001434761.1	lncRNA
XM_012182538.2	TRAPPC3L	mRNA	XR_001434761.1	lncRNA
XM_012182538.2	TRAPPC3L	mRNA	XR_001434761.1	lncRNA
XM_012182538.2	TRAPPC3L	mRNA	XR_001434761.1	lncRNA
XM_012182538.2	TRAPPC3L	mRNA	XR_001434761.1	lncRNA
XM_012178308.2	FAM193B	mRNA	XR_001434892.1	lncRNA
XM_012178308.2	FAM193B	mRNA	XR_001434892.1	lncRNA
XM_012178308.2	FAM193B	mRNA	XR_001434892.1	lncRNA
XM_012178308.2	FAM193B	mRNA	XR_001434892.1	lncRNA
XM_012178308.2	FAM193B	mRNA	XR_001434892.1	lncRNA
XM_012178308.2	FAM193B	mRNA	XR_001434892.1	lncRNA
XM_012178308.2	FAM193B	mRNA	XR_001434892.1	lncRNA
XM_012178308.2	FAM193B	mRNA	XR_001434892.1	lncRNA
XM_012178308.2	FAM193B	mRNA	XR_001434892.1	lncRNA
XM_012178308.2	FAM193B	mRNA	XR_001434892.1	lncRNA
XM_012178308.2	FAM193B	mRNA	XR_001434892.1	lncRNA
XM_012178308.2	FAM193B	mRNA	XR_001434892.1	lncRNA
XM_012178308.2	FAM193B	mRNA	XR_001434892.1	lncRNA
XM_015091836.1	USP40	mRNA	XR_001042381.2	lncRNA
XM_015091836.1	USP40	mRNA	XR_001042381.2	lncRNA
XM_015091836.1	USP40	mRNA	XR_001042381.2	lncRNA
XM_015091836.1	USP40	mRNA	XR_001042381.2	lncRNA
XM_015091836.1	USP40	mRNA	XR_001042381.2	lncRNA
XM_015091836.1	USP40	mRNA	XR_001042381.2	lncRNA
XM_015091836.1	USP40	mRNA	XR_001042381.2	lncRNA
XM_015091836.1	USP40	mRNA	XR_001042381.2	lncRNA
XM_015091836.1	USP40	mRNA	XR_001042381.2	lncRNA
XM_015091836.1	USP40	mRNA	XR_001042381.2	lncRNA
XM_015091836.1	USP40	mRNA	XR_001042381.2	lncRNA
XM_015091836.1	USP40	mRNA	XR_001042381.2	lncRNA
XM_015091836.1	USP40	mRNA	XR_001042381.2	lncRNA
XM_015091836.1	USP40	mRNA	XR_001042381.2	lncRNA
XM_012177309.2	LOC10111622(	mRNA	XR_001042381.2	lncRNA
XM_012177309.2	LOC10111622(	mRNA	XR_001042381.2	lncRNA
XM_012177309.2	LOC10111622(	mRNA	XR_001042381.2	lncRNA
XM_012177309.2	LOC10111622(	mRNA	XR_001042381.2	lncRNA
XM_012177309.2	LOC10111622(	mRNA	XR_001042381.2	lncRNA
XM_012177309.2	LOC10111622(	mRNA	XR_001042381.2	lncRNA
XM_012177309.2	LOC10111622(	mRNA	XR_001042381.2	lncRNA
XM_012177309.2	LOC10111622(	mRNA	XR_001042381.2	lncRNA
XM_012177309.2	LOC10111622(	mRNA	XR_001042381.2	lncRNA
XM_012177309.2	LOC10111622(	mRNA	XR_001042381.2	lncRNA
XM_012177309.2	LOC10111622(	mRNA	XR_001042381.2	lncRNA
XM_012177309.2	LOC10111622(	mRNA	XR_001042381.2	lncRNA
XM_012177309.2	LOC10111622(	mRNA	XR_001042381.2	lncRNA
XM_012177309.2	LOC10111622(	mRNA	XR_001042381.2	lncRNA
XM_015100041.1	ZNF473	mRNA	XR_001041211.2	lncRNA
XM_015100041.1	ZNF473	mRNA	XR_001041211.2	lncRNA
XM_015100041.1	ZNF473	mRNA	XR_001041211.2	lncRNA
XM_015100041.1	ZNF473	mRNA	XR_001041211.2	lncRNA
XM_015100041.1	ZNF473	mRNA	XR_001041211.2	lncRNA
XM_015100041.1	ZNF473	mRNA	XR_001041211.2	lncRNA
XM_015100041.1	ZNF473	mRNA	XR_001041211.2	lncRNA
XM_015100041.1	ZNF473	mRNA	XR_001041211.2	lncRNA

XM_015100041.1	ZNF473	mRNA	XR_001041211.2	lncRNA
XM_015100041.1	ZNF473	mRNA	XR_001041211.2	lncRNA
XM_015100041.1	ZNF473	mRNA	XR_001041211.2	lncRNA
XM_015100041.1	ZNF473	mRNA	XR_001041211.2	lncRNA
XM_015100041.1	ZNF473	mRNA	XR_001041211.2	lncRNA
XM_004003942.3	COL6A6	mRNA	XR_001434203.1	lncRNA
XM_004003942.3	COL6A6	mRNA	XR_001434203.1	lncRNA
XM_004003942.3	COL6A6	mRNA	XR_001434203.1	lncRNA
XM_004003942.3	COL6A6	mRNA	XR_001434203.1	lncRNA
XM_004003942.3	COL6A6	mRNA	XR_001434203.1	lncRNA
XM_004003942.3	COL6A6	mRNA	XR_001434203.1	lncRNA
XM_004003942.3	COL6A6	mRNA	XR_001434203.1	lncRNA
XM_004003942.3	COL6A6	mRNA	XR_001434203.1	lncRNA
XM_004003942.3	COL6A6	mRNA	XR_001434203.1	lncRNA
XM_004003942.3	COL6A6	mRNA	XR_001434203.1	lncRNA
XM_004003942.3	COL6A6	mRNA	XR_001434203.1	lncRNA
XM_004003942.3	COL6A6	mRNA	XR_001434203.1	lncRNA
XM_004003942.3	COL6A6	mRNA	XR_001434203.1	lncRNA
XM_004003942.3	COL6A6	mRNA	XR_001434203.1	lncRNA
XM_012185318.2	RAD51D	mRNA	XR_001436817.1	lncRNA
XM_012185318.2	RAD51D	mRNA	XR_001436817.1	lncRNA
XM_012185318.2	RAD51D	mRNA	XR_001436817.1	lncRNA
XM_012185318.2	RAD51D	mRNA	XR_001436817.1	lncRNA
XM_012185318.2	RAD51D	mRNA	XR_001436817.1	lncRNA
XM_012185318.2	RAD51D	mRNA	XR_001436817.1	lncRNA
XM_012185318.2	RAD51D	mRNA	XR_001436817.1	lncRNA
XM_012185318.2	RAD51D	mRNA	XR_001436817.1	lncRNA
XM_012185318.2	RAD51D	mRNA	XR_001436817.1	lncRNA
XM_012185318.2	RAD51D	mRNA	XR_001436817.1	lncRNA
XM_012185318.2	RAD51D	mRNA	XR_001436817.1	lncRNA
XM_012185318.2	RAD51D	mRNA	XR_001436817.1	lncRNA
XM_012185318.2	RAD51D	mRNA	XR_001436817.1	lncRNA
XM_012185318.2	RAD51D	mRNA	XR_001436817.1	lncRNA
XM_015092323.1	KIRREL	mRNA	XR_001436102.1	lncRNA
XM_015092323.1	KIRREL	mRNA	XR_001436102.1	lncRNA
XM_015092323.1	KIRREL	mRNA	XR_001436102.1	lncRNA
XM_015092323.1	KIRREL	mRNA	XR_001436102.1	lncRNA
XM_015092323.1	KIRREL	mRNA	XR_001436102.1	lncRNA
XM_015092323.1	KIRREL	mRNA	XR_001436102.1	lncRNA
XM_015092323.1	KIRREL	mRNA	XR_001436102.1	lncRNA
XM_015092323.1	KIRREL	mRNA	XR_001436102.1	lncRNA
XM_015092323.1	KIRREL	mRNA	XR_001436102.1	lncRNA
XM_015092323.1	KIRREL	mRNA	XR_001436102.1	lncRNA
XM_015092323.1	KIRREL	mRNA	XR_001436102.1	lncRNA
XM_015092323.1	KIRREL	mRNA	XR_001436102.1	lncRNA
XM_015092323.1	KIRREL	mRNA	XR_001436102.1	lncRNA
XM_012180879.2	MYO9A	mRNA	XR_001436102.1	lncRNA
XM_012180879.2	MYO9A	mRNA	XR_001436102.1	lncRNA
XM_012180879.2	MYO9A	mRNA	XR_001436102.1	lncRNA
XM_012180879.2	MYO9A	mRNA	XR_001436102.1	lncRNA
XM_012180879.2	MYO9A	mRNA	XR_001436102.1	lncRNA
XM_012180879.2	MYO9A	mRNA	XR_001436102.1	lncRNA
XM_012180879.2	MYO9A	mRNA	XR_001436102.1	lncRNA
XM_012180879.2	MYO9A	mRNA	XR_001436102.1	lncRNA

XM_012180879.2	MYO9A	mRNA	XR_001436102.1	lncRNA
XM_012180879.2	MYO9A	mRNA	XR_001436102.1	lncRNA
XM_012180879.2	MYO9A	mRNA	XR_001436102.1	lncRNA
XM_012180879.2	MYO9A	mRNA	XR_001436102.1	lncRNA
XM_012180879.2	MYO9A	mRNA	XR_001436102.1	lncRNA
XM_012181966.2	SIPA1L1	mRNA	XR_001436102.1	lncRNA
XM_012181966.2	SIPA1L1	mRNA	XR_001436102.1	lncRNA
XM_012181966.2	SIPA1L1	mRNA	XR_001436102.1	lncRNA
XM_012181966.2	SIPA1L1	mRNA	XR_001436102.1	lncRNA
XM_012181966.2	SIPA1L1	mRNA	XR_001436102.1	lncRNA
XM_012181966.2	SIPA1L1	mRNA	XR_001436102.1	lncRNA
XM_012181966.2	SIPA1L1	mRNA	XR_001436102.1	lncRNA
XM_012181966.2	SIPA1L1	mRNA	XR_001436102.1	lncRNA
XM_012181966.2	SIPA1L1	mRNA	XR_001436102.1	lncRNA
XM_012181966.2	SIPA1L1	mRNA	XR_001436102.1	lncRNA
XM_012181966.2	SIPA1L1	mRNA	XR_001436102.1	lncRNA
XM_012181966.2	SIPA1L1	mRNA	XR_001436102.1	lncRNA
XM_012181966.2	SIPA1L1	mRNA	XR_001436102.1	lncRNA
XM_012185318.2	RAD51D	mRNA	XR_001034847.2	lncRNA
XM_012185318.2	RAD51D	mRNA	XR_001034847.2	lncRNA
XM_012185318.2	RAD51D	mRNA	XR_001034847.2	lncRNA
XM_012185318.2	RAD51D	mRNA	XR_001034847.2	lncRNA
XM_012185318.2	RAD51D	mRNA	XR_001034847.2	lncRNA
XM_012185318.2	RAD51D	mRNA	XR_001034847.2	lncRNA
XM_012185318.2	RAD51D	mRNA	XR_001034847.2	lncRNA
XM_012185318.2	RAD51D	mRNA	XR_001034847.2	lncRNA
XM_012185318.2	RAD51D	mRNA	XR_001034847.2	lncRNA
XM_012185318.2	RAD51D	mRNA	XR_001034847.2	lncRNA
XM_012185318.2	RAD51D	mRNA	XR_001034847.2	lncRNA
XM_012185318.2	RAD51D	mRNA	XR_001034847.2	lncRNA
XM_012185318.2	RAD51D	mRNA	XR_001034847.2	lncRNA
XM_012185318.2	RAD51D	mRNA	XR_001434202.1	lncRNA
XM_012185318.2	RAD51D	mRNA	XR_001434202.1	lncRNA
XM_012185318.2	RAD51D	mRNA	XR_001434202.1	lncRNA
XM_012185318.2	RAD51D	mRNA	XR_001434202.1	lncRNA
XM_012185318.2	RAD51D	mRNA	XR_001434202.1	lncRNA
XM_012185318.2	RAD51D	mRNA	XR_001434202.1	lncRNA
XM_012185318.2	RAD51D	mRNA	XR_001434202.1	lncRNA
XM_012185318.2	RAD51D	mRNA	XR_001434202.1	lncRNA
XM_012185318.2	RAD51D	mRNA	XR_001434202.1	lncRNA
XM_012185318.2	RAD51D	mRNA	XR_001434202.1	lncRNA
XM_012185318.2	RAD51D	mRNA	XR_001434202.1	lncRNA
XM_012185318.2	RAD51D	mRNA	XR_001434202.1	lncRNA
XM_012185318.2	RAD51D	mRNA	XR_001434202.1	lncRNA
XM_004010552.3	VPS13C	mRNA	XR_001436817.1	lncRNA
XM_004010552.3	VPS13C	mRNA	XR_001436817.1	lncRNA
XM_004010552.3	VPS13C	mRNA	XR_001436817.1	lncRNA
XM_004010552.3	VPS13C	mRNA	XR_001436817.1	lncRNA
XM_004010552.3	VPS13C	mRNA	XR_001436817.1	lncRNA
XM_004010552.3	VPS13C	mRNA	XR_001436817.1	lncRNA
XM_004010552.3	VPS13C	mRNA	XR_001436817.1	lncRNA
XM_004010552.3	VPS13C	mRNA	XR_001436817.1	lncRNA
XM_004010552.3	VPS13C	mRNA	XR_001436817.1	lncRNA
XM_004010552.3	VPS13C	mRNA	XR_001436817.1	lncRNA

XM_004010552.3	VPS13C	mRNA	XR_001436817.1	lncRNA
XM_004010552.3	VPS13C	mRNA	XR_001436817.1	lncRNA
XM_004010552.3	VPS13C	mRNA	XR_001436817.1	lncRNA
XM_012137434.2	NEB	mRNA	XR_001042381.2	lncRNA
XM_012137434.2	NEB	mRNA	XR_001042381.2	lncRNA
XM_012137434.2	NEB	mRNA	XR_001042381.2	lncRNA
XM_012137434.2	NEB	mRNA	XR_001042381.2	lncRNA
XM_012137434.2	NEB	mRNA	XR_001042381.2	lncRNA
XM_012137434.2	NEB	mRNA	XR_001042381.2	lncRNA
XM_012137434.2	NEB	mRNA	XR_001042381.2	lncRNA
XM_012137434.2	NEB	mRNA	XR_001042381.2	lncRNA
XM_012137434.2	NEB	mRNA	XR_001042381.2	lncRNA
XM_012137434.2	NEB	mRNA	XR_001042381.2	lncRNA
XM_012137434.2	NEB	mRNA	XR_001042381.2	lncRNA
XM_012137434.2	NEB	mRNA	XR_001042381.2	lncRNA
XM_012137434.2	NEB	mRNA	XR_001042381.2	lncRNA
XM_012137434.2	NEB	mRNA	XR_001042381.2	lncRNA
XM_012095465.2	GOLGB1	mRNA	XR_001434761.1	lncRNA
XM_012095465.2	GOLGB1	mRNA	XR_001434761.1	lncRNA
XM_012095465.2	GOLGB1	mRNA	XR_001434761.1	lncRNA
XM_012095465.2	GOLGB1	mRNA	XR_001434761.1	lncRNA
XM_012095465.2	GOLGB1	mRNA	XR_001434761.1	lncRNA
XM_012095465.2	GOLGB1	mRNA	XR_001434761.1	lncRNA
XM_012095465.2	GOLGB1	mRNA	XR_001434761.1	lncRNA
XM_012095465.2	GOLGB1	mRNA	XR_001434761.1	lncRNA
XM_012095465.2	GOLGB1	mRNA	XR_001434761.1	lncRNA
XM_012095465.2	GOLGB1	mRNA	XR_001434761.1	lncRNA
XM_012095465.2	GOLGB1	mRNA	XR_001434761.1	lncRNA
XM_012095465.2	GOLGB1	mRNA	XR_001434761.1	lncRNA
XM_012095465.2	GOLGB1	mRNA	XR_001434761.1	lncRNA
XM_012178308.2	FAM193B	mRNA	XR_001436532.1	lncRNA
XM_012178308.2	FAM193B	mRNA	XR_001436532.1	lncRNA
XM_012178308.2	FAM193B	mRNA	XR_001436532.1	lncRNA
XM_012178308.2	FAM193B	mRNA	XR_001436532.1	lncRNA
XM_012178308.2	FAM193B	mRNA	XR_001436532.1	lncRNA
XM_012178308.2	FAM193B	mRNA	XR_001436532.1	lncRNA
XM_012178308.2	FAM193B	mRNA	XR_001436532.1	lncRNA
XM_012178308.2	FAM193B	mRNA	XR_001436532.1	lncRNA
XM_012178308.2	FAM193B	mRNA	XR_001436532.1	lncRNA
XM_012178308.2	FAM193B	mRNA	XR_001436532.1	lncRNA
XM_012178308.2	FAM193B	mRNA	XR_001436532.1	lncRNA
XM_012178308.2	FAM193B	mRNA	XR_001436532.1	lncRNA
XM_012178308.2	FAM193B	mRNA	XR_001436532.1	lncRNA
XM_015103466.1	TACC2	mRNA	XR_001436102.1	lncRNA
XM_015103466.1	TACC2	mRNA	XR_001436102.1	lncRNA
XM_015103466.1	TACC2	mRNA	XR_001436102.1	lncRNA
XM_015103466.1	TACC2	mRNA	XR_001436102.1	lncRNA
XM_015103466.1	TACC2	mRNA	XR_001436102.1	lncRNA
XM_015103466.1	TACC2	mRNA	XR_001436102.1	lncRNA
XM_015103466.1	TACC2	mRNA	XR_001436102.1	lncRNA
XM_015103466.1	TACC2	mRNA	XR_001436102.1	lncRNA
XM_015103466.1	TACC2	mRNA	XR_001436102.1	lncRNA
XM_015103466.1	TACC2	mRNA	XR_001436102.1	lncRNA
XM_015103466.1	TACC2	mRNA	XR_001436102.1	lncRNA

XM_015103466.1	TACC2	mRNA	XR_001436102.1	lncRNA
XM_015103466.1	TACC2	mRNA	XR_001436102.1	lncRNA
XM_012177947.2	INSR	mRNA	XR_001021689.2	lncRNA
XM_012177947.2	INSR	mRNA	XR_001021689.2	lncRNA
XM_012177947.2	INSR	mRNA	XR_001021689.2	lncRNA
XM_012177947.2	INSR	mRNA	XR_001021689.2	lncRNA
XM_012177947.2	INSR	mRNA	XR_001021689.2	lncRNA
XM_012177947.2	INSR	mRNA	XR_001021689.2	lncRNA
XM_012177947.2	INSR	mRNA	XR_001021689.2	lncRNA
XM_012177947.2	INSR	mRNA	XR_001021689.2	lncRNA
XM_012177947.2	INSR	mRNA	XR_001021689.2	lncRNA
XM_012177947.2	INSR	mRNA	XR_001021689.2	lncRNA
XM_012177947.2	INSR	mRNA	XR_001021689.2	lncRNA
XM_012177947.2	INSR	mRNA	XR_001021689.2	lncRNA
XM_015103448.1	TACC2	mRNA	XR_001436102.1	lncRNA
XM_015103448.1	TACC2	mRNA	XR_001436102.1	lncRNA
XM_015103448.1	TACC2	mRNA	XR_001436102.1	lncRNA
XM_015103448.1	TACC2	mRNA	XR_001436102.1	lncRNA
XM_015103448.1	TACC2	mRNA	XR_001436102.1	lncRNA
XM_015103448.1	TACC2	mRNA	XR_001436102.1	lncRNA
XM_015103448.1	TACC2	mRNA	XR_001436102.1	lncRNA
XM_015103448.1	TACC2	mRNA	XR_001436102.1	lncRNA
XM_015103448.1	TACC2	mRNA	XR_001436102.1	lncRNA
XM_015103448.1	TACC2	mRNA	XR_001436102.1	lncRNA
XM_015103448.1	TACC2	mRNA	XR_001436102.1	lncRNA
XM_015103448.1	TACC2	mRNA	XR_001436102.1	lncRNA
XM_015103448.1	TACC2	mRNA	XR_001436102.1	lncRNA
XM_012176498.2	RAPGEF5	mRNA	XR_001434761.1	lncRNA
XM_012176498.2	RAPGEF5	mRNA	XR_001434761.1	lncRNA
XM_012176498.2	RAPGEF5	mRNA	XR_001434761.1	lncRNA
XM_012176498.2	RAPGEF5	mRNA	XR_001434761.1	lncRNA
XM_012176498.2	RAPGEF5	mRNA	XR_001434761.1	lncRNA
XM_012176498.2	RAPGEF5	mRNA	XR_001434761.1	lncRNA
XM_012176498.2	RAPGEF5	mRNA	XR_001434761.1	lncRNA
XM_012176498.2	RAPGEF5	mRNA	XR_001434761.1	lncRNA
XM_012176498.2	RAPGEF5	mRNA	XR_001434761.1	lncRNA
XM_012176498.2	RAPGEF5	mRNA	XR_001434761.1	lncRNA
XM_012176498.2	RAPGEF5	mRNA	XR_001434761.1	lncRNA
XM_012176498.2	RAPGEF5	mRNA	XR_001434761.1	lncRNA
XM_012176498.2	RAPGEF5	mRNA	XR_001434761.1	lncRNA
XM_012098820.2	RALGAPA1	mRNA	XR_001436817.1	lncRNA
XM_012098820.2	RALGAPA1	mRNA	XR_001436817.1	lncRNA
XM_012098820.2	RALGAPA1	mRNA	XR_001436817.1	lncRNA
XM_012098820.2	RALGAPA1	mRNA	XR_001436817.1	lncRNA
XM_012098820.2	RALGAPA1	mRNA	XR_001436817.1	lncRNA
XM_012098820.2	RALGAPA1	mRNA	XR_001436817.1	lncRNA
XM_012098820.2	RALGAPA1	mRNA	XR_001436817.1	lncRNA
XM_012098820.2	RALGAPA1	mRNA	XR_001436817.1	lncRNA
XM_012098820.2	RALGAPA1	mRNA	XR_001436817.1	lncRNA
XM_012098820.2	RALGAPA1	mRNA	XR_001436817.1	lncRNA
XM_012098820.2	RALGAPA1	mRNA	XR_001436817.1	lncRNA
XM_012098820.2	RALGAPA1	mRNA	XR_001436817.1	lncRNA
XM_012098820.2	RALGAPA1	mRNA	XR_001436817.1	lncRNA

XM_004020074.3	PIK3AP1	mRNA	XR_001436817.1	lncRNA
XM_004020074.3	PIK3AP1	mRNA	XR_001436817.1	lncRNA
XM_004020074.3	PIK3AP1	mRNA	XR_001436817.1	lncRNA
XM_004020074.3	PIK3AP1	mRNA	XR_001436817.1	lncRNA
XM_004020074.3	PIK3AP1	mRNA	XR_001436817.1	lncRNA
XM_004020074.3	PIK3AP1	mRNA	XR_001436817.1	lncRNA
XM_004020074.3	PIK3AP1	mRNA	XR_001436817.1	lncRNA
XM_004020074.3	PIK3AP1	mRNA	XR_001436817.1	lncRNA
XM_004020074.3	PIK3AP1	mRNA	XR_001436817.1	lncRNA
XM_004020074.3	PIK3AP1	mRNA	XR_001436817.1	lncRNA
XM_004020074.3	PIK3AP1	mRNA	XR_001436817.1	lncRNA
XM_004020074.3	PIK3AP1	mRNA	XR_001436817.1	lncRNA
XM_004020074.3	PIK3AP1	mRNA	XR_001436817.1	lncRNA
XM_012098820.2	RALGAPA1	mRNA	XR_001434203.1	lncRNA
XM_012098820.2	RALGAPA1	mRNA	XR_001434203.1	lncRNA
XM_012098820.2	RALGAPA1	mRNA	XR_001434203.1	lncRNA
XM_012098820.2	RALGAPA1	mRNA	XR_001434203.1	lncRNA
XM_012098820.2	RALGAPA1	mRNA	XR_001434203.1	lncRNA
XM_012098820.2	RALGAPA1	mRNA	XR_001434203.1	lncRNA
XM_012098820.2	RALGAPA1	mRNA	XR_001434203.1	lncRNA
XM_012098820.2	RALGAPA1	mRNA	XR_001434203.1	lncRNA
XM_012098820.2	RALGAPA1	mRNA	XR_001434203.1	lncRNA
XM_012098820.2	RALGAPA1	mRNA	XR_001434203.1	lncRNA
XM_012098820.2	RALGAPA1	mRNA	XR_001434203.1	lncRNA
XM_012098820.2	RALGAPA1	mRNA	XR_001434203.1	lncRNA
XM_012098820.2	RALGAPA1	mRNA	XR_001434203.1	lncRNA
XM_015098618.1	DLG4	mRNA	XR_001042381.2	lncRNA
XM_015098618.1	DLG4	mRNA	XR_001042381.2	lncRNA
XM_015098618.1	DLG4	mRNA	XR_001042381.2	lncRNA
XM_015098618.1	DLG4	mRNA	XR_001042381.2	lncRNA
XM_015098618.1	DLG4	mRNA	XR_001042381.2	lncRNA
XM_015098618.1	DLG4	mRNA	XR_001042381.2	lncRNA
XM_015098618.1	DLG4	mRNA	XR_001042381.2	lncRNA
XM_015098618.1	DLG4	mRNA	XR_001042381.2	lncRNA
XM_015098618.1	DLG4	mRNA	XR_001042381.2	lncRNA
XM_015098618.1	DLG4	mRNA	XR_001042381.2	lncRNA
XM_015098618.1	DLG4	mRNA	XR_001042381.2	lncRNA
XM_015098618.1	DLG4	mRNA	XR_001042381.2	lncRNA
XM_015098618.1	DLG4	mRNA	XR_001042381.2	lncRNA
XM_015098529.1	NF1	mRNA	XR_001042381.2	lncRNA
XM_015098529.1	NF1	mRNA	XR_001042381.2	lncRNA
XM_015098529.1	NF1	mRNA	XR_001042381.2	lncRNA
XM_015098529.1	NF1	mRNA	XR_001042381.2	lncRNA
XM_015098529.1	NF1	mRNA	XR_001042381.2	lncRNA
XM_015098529.1	NF1	mRNA	XR_001042381.2	lncRNA
XM_015098529.1	NF1	mRNA	XR_001042381.2	lncRNA
XM_015098529.1	NF1	mRNA	XR_001042381.2	lncRNA
XM_015098529.1	NF1	mRNA	XR_001042381.2	lncRNA
XM_015098529.1	NF1	mRNA	XR_001042381.2	lncRNA
XM_015098529.1	NF1	mRNA	XR_001042381.2	lncRNA
XM_015098529.1	NF1	mRNA	XR_001042381.2	lncRNA
XM_015098529.1	NF1	mRNA	XR_001042381.2	lncRNA
XM_012105690.2	WDR17	mRNA	XR_001436532.1	lncRNA
XM_012105690.2	WDR17	mRNA	XR_001436532.1	lncRNA

XM_012105690.2	WDR17	mRNA	XR_001436532.1	lncRNA
XM_012105690.2	WDR17	mRNA	XR_001436532.1	lncRNA
XM_012105690.2	WDR17	mRNA	XR_001436532.1	lncRNA
XM_012105690.2	WDR17	mRNA	XR_001436532.1	lncRNA
XM_012105690.2	WDR17	mRNA	XR_001436532.1	lncRNA
XM_012105690.2	WDR17	mRNA	XR_001436532.1	lncRNA
XM_012105690.2	WDR17	mRNA	XR_001436532.1	lncRNA
XM_012105690.2	WDR17	mRNA	XR_001436532.1	lncRNA
XM_012105690.2	WDR17	mRNA	XR_001436532.1	lncRNA
XM_012105690.2	WDR17	mRNA	XR_001436532.1	lncRNA
XM_012105690.2	WDR17	mRNA	XR_001436532.1	lncRNA
XM_012187378.2	CAMTA1	mRNA	XR_001436102.1	lncRNA
XM_012187378.2	CAMTA1	mRNA	XR_001436102.1	lncRNA
XM_012187378.2	CAMTA1	mRNA	XR_001436102.1	lncRNA
XM_012187378.2	CAMTA1	mRNA	XR_001436102.1	lncRNA
XM_012187378.2	CAMTA1	mRNA	XR_001436102.1	lncRNA
XM_012187378.2	CAMTA1	mRNA	XR_001436102.1	lncRNA
XM_012187378.2	CAMTA1	mRNA	XR_001436102.1	lncRNA
XM_012187378.2	CAMTA1	mRNA	XR_001436102.1	lncRNA
XM_012187378.2	CAMTA1	mRNA	XR_001436102.1	lncRNA
XM_012187378.2	CAMTA1	mRNA	XR_001436102.1	lncRNA
XM_012187378.2	CAMTA1	mRNA	XR_001436102.1	lncRNA
XM_012187378.2	CAMTA1	mRNA	XR_001436102.1	lncRNA
XM_012187378.2	CAMTA1	mRNA	XR_001436102.1	lncRNA
XM_012184884.2	DOCK9	mRNA	XR_001436102.1	lncRNA
XM_012184884.2	DOCK9	mRNA	XR_001436102.1	lncRNA
XM_012184884.2	DOCK9	mRNA	XR_001436102.1	lncRNA
XM_012184884.2	DOCK9	mRNA	XR_001436102.1	lncRNA
XM_012184884.2	DOCK9	mRNA	XR_001436102.1	lncRNA
XM_012184884.2	DOCK9	mRNA	XR_001436102.1	lncRNA
XM_012184884.2	DOCK9	mRNA	XR_001436102.1	lncRNA
XM_012184884.2	DOCK9	mRNA	XR_001436102.1	lncRNA
XM_012184884.2	DOCK9	mRNA	XR_001436102.1	lncRNA
XM_012184884.2	DOCK9	mRNA	XR_001436102.1	lncRNA
XM_012184884.2	DOCK9	mRNA	XR_001436102.1	lncRNA
XM_012184884.2	DOCK9	mRNA	XR_001436102.1	lncRNA
XM_012184885.2	DOCK9	mRNA	XR_001436102.1	lncRNA
XM_012184885.2	DOCK9	mRNA	XR_001436102.1	lncRNA
XM_012184885.2	DOCK9	mRNA	XR_001436102.1	lncRNA
XM_012184885.2	DOCK9	mRNA	XR_001436102.1	lncRNA
XM_012184885.2	DOCK9	mRNA	XR_001436102.1	lncRNA
XM_012184885.2	DOCK9	mRNA	XR_001436102.1	lncRNA
XM_012184885.2	DOCK9	mRNA	XR_001436102.1	lncRNA
XM_012184885.2	DOCK9	mRNA	XR_001436102.1	lncRNA
XM_012184885.2	DOCK9	mRNA	XR_001436102.1	lncRNA
XM_012184885.2	DOCK9	mRNA	XR_001436102.1	lncRNA
XM_012184885.2	DOCK9	mRNA	XR_001436102.1	lncRNA
XM_012184885.2	DOCK9	mRNA	XR_001436102.1	lncRNA
XM_015103461.1	TACC2	mRNA	XR_001034847.2	lncRNA
XM_015103461.1	TACC2	mRNA	XR_001034847.2	lncRNA
XM_015103461.1	TACC2	mRNA	XR_001034847.2	lncRNA
XM_015103461.1	TACC2	mRNA	XR_001034847.2	lncRNA
XM_015103461.1	TACC2	mRNA	XR_001034847.2	lncRNA
XM_015103461.1	TACC2	mRNA	XR_001034847.2	lncRNA

XM_015103461.1	TACC2	mRNA	XR_001034847.2	lncRNA
XM_015103461.1	TACC2	mRNA	XR_001034847.2	lncRNA
XM_015103461.1	TACC2	mRNA	XR_001034847.2	lncRNA
XM_015103461.1	TACC2	mRNA	XR_001034847.2	lncRNA
XM_015103461.1	TACC2	mRNA	XR_001034847.2	lncRNA
XM_015103461.1	TACC2	mRNA	XR_001034847.2	lncRNA
XM_015103461.1	TACC2	mRNA	XR_001034847.2	lncRNA
XM_015103461.1	TACC2	mRNA	XR_001434202.1	lncRNA
XM_015103461.1	TACC2	mRNA	XR_001434202.1	lncRNA
XM_015103461.1	TACC2	mRNA	XR_001434202.1	lncRNA
XM_015103461.1	TACC2	mRNA	XR_001434202.1	lncRNA
XM_015103461.1	TACC2	mRNA	XR_001434202.1	lncRNA
XM_015103461.1	TACC2	mRNA	XR_001434202.1	lncRNA
XM_015103461.1	TACC2	mRNA	XR_001434202.1	lncRNA
XM_015103461.1	TACC2	mRNA	XR_001434202.1	lncRNA
XM_015103461.1	TACC2	mRNA	XR_001434202.1	lncRNA
XM_015103461.1	TACC2	mRNA	XR_001434202.1	lncRNA
XM_015103461.1	TACC2	mRNA	XR_001434202.1	lncRNA
XM_015103461.1	TACC2	mRNA	XR_001434202.1	lncRNA
XM_015103461.1	TACC2	mRNA	XR_001434202.1	lncRNA
XM_012181966.2	SIPA1L1	mRNA	XR_001434761.1	lncRNA
XM_012181966.2	SIPA1L1	mRNA	XR_001434761.1	lncRNA
XM_012181966.2	SIPA1L1	mRNA	XR_001434761.1	lncRNA
XM_012181966.2	SIPA1L1	mRNA	XR_001434761.1	lncRNA
XM_012181966.2	SIPA1L1	mRNA	XR_001434761.1	lncRNA
XM_012181966.2	SIPA1L1	mRNA	XR_001434761.1	lncRNA
XM_012181966.2	SIPA1L1	mRNA	XR_001434761.1	lncRNA
XM_012181966.2	SIPA1L1	mRNA	XR_001434761.1	lncRNA
XM_012181966.2	SIPA1L1	mRNA	XR_001434761.1	lncRNA
XM_012181966.2	SIPA1L1	mRNA	XR_001434761.1	lncRNA
XM_012181966.2	SIPA1L1	mRNA	XR_001434761.1	lncRNA
XM_012181966.2	SIPA1L1	mRNA	XR_001434761.1	lncRNA
XM_012181966.2	SIPA1L1	mRNA	XR_001434761.1	lncRNA
XM_012181247.2	MGA	mRNA	XR_001041211.2	lncRNA
XM_012181247.2	MGA	mRNA	XR_001041211.2	lncRNA
XM_012181247.2	MGA	mRNA	XR_001041211.2	lncRNA
XM_012181247.2	MGA	mRNA	XR_001041211.2	lncRNA
XM_012181247.2	MGA	mRNA	XR_001041211.2	lncRNA
XM_012181247.2	MGA	mRNA	XR_001041211.2	lncRNA
XM_012181247.2	MGA	mRNA	XR_001041211.2	lncRNA
XM_012181247.2	MGA	mRNA	XR_001041211.2	lncRNA
XM_012181247.2	MGA	mRNA	XR_001041211.2	lncRNA
XM_012181247.2	MGA	mRNA	XR_001041211.2	lncRNA
XM_012181247.2	MGA	mRNA	XR_001041211.2	lncRNA
XM_012181247.2	MGA	mRNA	XR_001041211.2	lncRNA
XM_012181247.2	MGA	mRNA	XR_001041211.2	lncRNA
XM_015096638.1	CC2L1A	mRNA	XR_001042381.2	lncRNA
XM_015096638.1	CC2L1A	mRNA	XR_001042381.2	lncRNA
XM_015096638.1	CC2L1A	mRNA	XR_001042381.2	lncRNA
XM_015096638.1	CC2L1A	mRNA	XR_001042381.2	lncRNA
XM_015096638.1	CC2L1A	mRNA	XR_001042381.2	lncRNA
XM_015096638.1	CC2L1A	mRNA	XR_001042381.2	lncRNA
XM_015096638.1	CC2L1A	mRNA	XR_001042381.2	lncRNA
XM_015096638.1	CC2L1A	mRNA	XR_001042381.2	lncRNA

XM_015096638.1	CC2L1A	mRNA	XR_001042381.2	lncRNA
XM_015096638.1	CC2L1A	mRNA	XR_001042381.2	lncRNA
XM_015096638.1	CC2L1A	mRNA	XR_001042381.2	lncRNA
XM_015096638.1	CC2L1A	mRNA	XR_001042381.2	lncRNA
XM_012119517.2	SORBS3	mRNA	XR_001034847.2	lncRNA
XM_012119517.2	SORBS3	mRNA	XR_001034847.2	lncRNA
XM_012119517.2	SORBS3	mRNA	XR_001034847.2	lncRNA
XM_012119517.2	SORBS3	mRNA	XR_001034847.2	lncRNA
XM_012119517.2	SORBS3	mRNA	XR_001034847.2	lncRNA
XM_012119517.2	SORBS3	mRNA	XR_001034847.2	lncRNA
XM_012119517.2	SORBS3	mRNA	XR_001034847.2	lncRNA
XM_012119517.2	SORBS3	mRNA	XR_001034847.2	lncRNA
XM_012119517.2	SORBS3	mRNA	XR_001034847.2	lncRNA
XM_012119517.2	SORBS3	mRNA	XR_001034847.2	lncRNA
XM_012119517.2	SORBS3	mRNA	XR_001034847.2	lncRNA
XM_012119517.2	SORBS3	mRNA	XR_001034847.2	lncRNA
XM_012119517.2	SORBS3	mRNA	XR_001434202.1	lncRNA
XM_012119517.2	SORBS3	mRNA	XR_001434202.1	lncRNA
XM_012119517.2	SORBS3	mRNA	XR_001434202.1	lncRNA
XM_012119517.2	SORBS3	mRNA	XR_001434202.1	lncRNA
XM_012119517.2	SORBS3	mRNA	XR_001434202.1	lncRNA
XM_012119517.2	SORBS3	mRNA	XR_001434202.1	lncRNA
XM_012119517.2	SORBS3	mRNA	XR_001434202.1	lncRNA
XM_012119517.2	SORBS3	mRNA	XR_001434202.1	lncRNA
XM_012119517.2	SORBS3	mRNA	XR_001434202.1	lncRNA
XM_012119517.2	SORBS3	mRNA	XR_001434202.1	lncRNA
XM_012119517.2	SORBS3	mRNA	XR_001434202.1	lncRNA
XM_012119517.2	SORBS3	mRNA	XR_001434202.1	lncRNA
XM_012184887.2	DOCK9	mRNA	XR_001436102.1	lncRNA
XM_012184887.2	DOCK9	mRNA	XR_001436102.1	lncRNA
XM_012184887.2	DOCK9	mRNA	XR_001436102.1	lncRNA
XM_012184887.2	DOCK9	mRNA	XR_001436102.1	lncRNA
XM_012184887.2	DOCK9	mRNA	XR_001436102.1	lncRNA
XM_012184887.2	DOCK9	mRNA	XR_001436102.1	lncRNA
XM_012184887.2	DOCK9	mRNA	XR_001436102.1	lncRNA
XM_012184887.2	DOCK9	mRNA	XR_001436102.1	lncRNA
XM_012184887.2	DOCK9	mRNA	XR_001436102.1	lncRNA
XM_012184887.2	DOCK9	mRNA	XR_001436102.1	lncRNA
XM_012184887.2	DOCK9	mRNA	XR_001436102.1	lncRNA
XM_012184887.2	DOCK9	mRNA	XR_001436102.1	lncRNA
XM_012095465.2	GOLGB1	mRNA	XR_001436102.1	lncRNA
XM_012095465.2	GOLGB1	mRNA	XR_001436102.1	lncRNA
XM_012095465.2	GOLGB1	mRNA	XR_001436102.1	lncRNA
XM_012095465.2	GOLGB1	mRNA	XR_001436102.1	lncRNA
XM_012095465.2	GOLGB1	mRNA	XR_001436102.1	lncRNA
XM_012095465.2	GOLGB1	mRNA	XR_001436102.1	lncRNA
XM_012095465.2	GOLGB1	mRNA	XR_001436102.1	lncRNA
XM_012095465.2	GOLGB1	mRNA	XR_001436102.1	lncRNA
XM_012095465.2	GOLGB1	mRNA	XR_001436102.1	lncRNA
XM_012095465.2	GOLGB1	mRNA	XR_001436102.1	lncRNA
XM_012095465.2	GOLGB1	mRNA	XR_001436102.1	lncRNA
XM_012095465.2	GOLGB1	mRNA	XR_001436102.1	lncRNA
XM_012189779.2	RBL2	mRNA	XR_001434892.1	lncRNA
XM_012189779.2	RBL2	mRNA	XR_001434892.1	lncRNA

XM_012189779.2	RBL2	mRNA	XR_001434892.1	lncRNA
XM_012189779.2	RBL2	mRNA	XR_001434892.1	lncRNA
XM_012189779.2	RBL2	mRNA	XR_001434892.1	lncRNA
XM_012189779.2	RBL2	mRNA	XR_001434892.1	lncRNA
XM_012189779.2	RBL2	mRNA	XR_001434892.1	lncRNA
XM_012189779.2	RBL2	mRNA	XR_001434892.1	lncRNA
XM_012189779.2	RBL2	mRNA	XR_001434892.1	lncRNA
XM_012189779.2	RBL2	mRNA	XR_001434892.1	lncRNA
XM_012189779.2	RBL2	mRNA	XR_001434892.1	lncRNA
XM_012189779.2	RBL2	mRNA	XR_001434892.1	lncRNA
XM_012105690.2	WDR17	mRNA	XR_001436817.1	lncRNA
XM_012105690.2	WDR17	mRNA	XR_001436817.1	lncRNA
XM_012105690.2	WDR17	mRNA	XR_001436817.1	lncRNA
XM_012105690.2	WDR17	mRNA	XR_001436817.1	lncRNA
XM_012105690.2	WDR17	mRNA	XR_001436817.1	lncRNA
XM_012105690.2	WDR17	mRNA	XR_001436817.1	lncRNA
XM_012105690.2	WDR17	mRNA	XR_001436817.1	lncRNA
XM_012105690.2	WDR17	mRNA	XR_001436817.1	lncRNA
XM_012105690.2	WDR17	mRNA	XR_001436817.1	lncRNA
XM_012105690.2	WDR17	mRNA	XR_001436817.1	lncRNA
XM_012105690.2	WDR17	mRNA	XR_001436817.1	lncRNA
XM_012105690.2	WDR17	mRNA	XR_001436817.1	lncRNA
XM_012105690.2	WDR17	mRNA	XR_001436817.1	lncRNA
XM_015096937.1	BMF	mRNA	XR_001434761.1	lncRNA
XM_015096937.1	BMF	mRNA	XR_001434761.1	lncRNA
XM_015096937.1	BMF	mRNA	XR_001434761.1	lncRNA
XM_015096937.1	BMF	mRNA	XR_001434761.1	lncRNA
XM_015096937.1	BMF	mRNA	XR_001434761.1	lncRNA
XM_015096937.1	BMF	mRNA	XR_001434761.1	lncRNA
XM_015096937.1	BMF	mRNA	XR_001434761.1	lncRNA
XM_015096937.1	BMF	mRNA	XR_001434761.1	lncRNA
XM_015096937.1	BMF	mRNA	XR_001434761.1	lncRNA
XM_015096937.1	BMF	mRNA	XR_001434761.1	lncRNA
XM_015096937.1	BMF	mRNA	XR_001434761.1	lncRNA
XM_015096937.1	BMF	mRNA	XR_001434761.1	lncRNA
XM_015096937.1	BMF	mRNA	XR_001434761.1	lncRNA
XM_012179034.2	FER	mRNA	XR_001434761.1	lncRNA
XM_012179034.2	FER	mRNA	XR_001434761.1	lncRNA
XM_012179034.2	FER	mRNA	XR_001434761.1	lncRNA
XM_012179034.2	FER	mRNA	XR_001434761.1	lncRNA
XM_012179034.2	FER	mRNA	XR_001434761.1	lncRNA
XM_012179034.2	FER	mRNA	XR_001434761.1	lncRNA
XM_012179034.2	FER	mRNA	XR_001434761.1	lncRNA
XM_012179034.2	FER	mRNA	XR_001434761.1	lncRNA
XM_012179034.2	FER	mRNA	XR_001434761.1	lncRNA
XM_012179034.2	FER	mRNA	XR_001434761.1	lncRNA
XM_012179034.2	FER	mRNA	XR_001434761.1	lncRNA
XM_012179034.2	FER	mRNA	XR_001434761.1	lncRNA
XM_012179034.2	FER	mRNA	XR_001434761.1	lncRNA
XM_012174703.2	ZBTB39	mRNA	XR_001434761.1	lncRNA
XM_012174703.2	ZBTB39	mRNA	XR_001434761.1	lncRNA
XM_012174703.2	ZBTB39	mRNA	XR_001434761.1	lncRNA
XM_012174703.2	ZBTB39	mRNA	XR_001434761.1	lncRNA
XM_012174703.2	ZBTB39	mRNA	XR_001434761.1	lncRNA

XM_012174703.2	ZBTB39	mRNA	XR_001434761.1	lncRNA
XM_012174703.2	ZBTB39	mRNA	XR_001434761.1	lncRNA
XM_012174703.2	ZBTB39	mRNA	XR_001434761.1	lncRNA
XM_012174703.2	ZBTB39	mRNA	XR_001434761.1	lncRNA
XM_012174703.2	ZBTB39	mRNA	XR_001434761.1	lncRNA
XM_012174703.2	ZBTB39	mRNA	XR_001434761.1	lncRNA
XM_012174703.2	ZBTB39	mRNA	XR_001434761.1	lncRNA
XM_012174703.2	ZBTB39	mRNA	XR_001434761.1	lncRNA
XM_012105331.2	USP54	mRNA	XR_001436817.1	lncRNA
XM_012105331.2	USP54	mRNA	XR_001436817.1	lncRNA
XM_012105331.2	USP54	mRNA	XR_001436817.1	lncRNA
XM_012105331.2	USP54	mRNA	XR_001436817.1	lncRNA
XM_012105331.2	USP54	mRNA	XR_001436817.1	lncRNA
XM_012105331.2	USP54	mRNA	XR_001436817.1	lncRNA
XM_012105331.2	USP54	mRNA	XR_001436817.1	lncRNA
XM_012105331.2	USP54	mRNA	XR_001436817.1	lncRNA
XM_012105331.2	USP54	mRNA	XR_001436817.1	lncRNA
XM_012105331.2	USP54	mRNA	XR_001436817.1	lncRNA
XM_012105331.2	USP54	mRNA	XR_001436817.1	lncRNA
XM_012105331.2	USP54	mRNA	XR_001436817.1	lncRNA
XM_015103117.1	ZBTB44	mRNA	XR_001436817.1	lncRNA
XM_015103117.1	ZBTB44	mRNA	XR_001436817.1	lncRNA
XM_015103117.1	ZBTB44	mRNA	XR_001436817.1	lncRNA
XM_015103117.1	ZBTB44	mRNA	XR_001436817.1	lncRNA
XM_015103117.1	ZBTB44	mRNA	XR_001436817.1	lncRNA
XM_015103117.1	ZBTB44	mRNA	XR_001436817.1	lncRNA
XM_015103117.1	ZBTB44	mRNA	XR_001436817.1	lncRNA
XM_015103117.1	ZBTB44	mRNA	XR_001436817.1	lncRNA
XM_015103117.1	ZBTB44	mRNA	XR_001436817.1	lncRNA
XM_015103117.1	ZBTB44	mRNA	XR_001436817.1	lncRNA
XM_015103117.1	ZBTB44	mRNA	XR_001436817.1	lncRNA
XM_015103117.1	ZBTB44	mRNA	XR_001436817.1	lncRNA
XM_004006548.3	R3HDM2	mRNA	XR_001436817.1	lncRNA
XM_004006548.3	R3HDM2	mRNA	XR_001436817.1	lncRNA
XM_004006548.3	R3HDM2	mRNA	XR_001436817.1	lncRNA
XM_004006548.3	R3HDM2	mRNA	XR_001436817.1	lncRNA
XM_004006548.3	R3HDM2	mRNA	XR_001436817.1	lncRNA
XM_004006548.3	R3HDM2	mRNA	XR_001436817.1	lncRNA
XM_004006548.3	R3HDM2	mRNA	XR_001436817.1	lncRNA
XM_004006548.3	R3HDM2	mRNA	XR_001436817.1	lncRNA
XM_004006548.3	R3HDM2	mRNA	XR_001436817.1	lncRNA
XM_004006548.3	R3HDM2	mRNA	XR_001436817.1	lncRNA
XM_004006548.3	R3HDM2	mRNA	XR_001436817.1	lncRNA
XM_004006548.3	R3HDM2	mRNA	XR_001436817.1	lncRNA
XM_015096638.1	CC2L1A	mRNA	XR_001436817.1	lncRNA
XM_015096638.1	CC2L1A	mRNA	XR_001436817.1	lncRNA
XM_015096638.1	CC2L1A	mRNA	XR_001436817.1	lncRNA
XM_015096638.1	CC2L1A	mRNA	XR_001436817.1	lncRNA
XM_015096638.1	CC2L1A	mRNA	XR_001436817.1	lncRNA
XM_015096638.1	CC2L1A	mRNA	XR_001436817.1	lncRNA
XM_015096638.1	CC2L1A	mRNA	XR_001436817.1	lncRNA
XM_015096638.1	CC2L1A	mRNA	XR_001436817.1	lncRNA
XM_015096638.1	CC2L1A	mRNA	XR_001436817.1	lncRNA
XM_015096638.1	CC2L1A	mRNA	XR_001436817.1	lncRNA

XM_015096638.1	CC2L1A	mRNA	XR_001436817.1	lncRNA
XM_015096638.1	CC2L1A	mRNA	XR_001436817.1	lncRNA
XM_012096925.2	SLC38A9	mRNA	XR_001436817.1	lncRNA
XM_012096925.2	SLC38A9	mRNA	XR_001436817.1	lncRNA
XM_012096925.2	SLC38A9	mRNA	XR_001436817.1	lncRNA
XM_012096925.2	SLC38A9	mRNA	XR_001436817.1	lncRNA
XM_012096925.2	SLC38A9	mRNA	XR_001436817.1	lncRNA
XM_012096925.2	SLC38A9	mRNA	XR_001436817.1	lncRNA
XM_012096925.2	SLC38A9	mRNA	XR_001436817.1	lncRNA
XM_012096925.2	SLC38A9	mRNA	XR_001436817.1	lncRNA
XM_012096925.2	SLC38A9	mRNA	XR_001436817.1	lncRNA
XM_012096925.2	SLC38A9	mRNA	XR_001436817.1	lncRNA
XM_012096925.2	SLC38A9	mRNA	XR_001436817.1	lncRNA
XM_012096925.2	SLC38A9	mRNA	XR_001436817.1	lncRNA
XM_015092421.1	BBX	mRNA	XR_001436817.1	lncRNA
XM_015092421.1	BBX	mRNA	XR_001436817.1	lncRNA
XM_015092421.1	BBX	mRNA	XR_001436817.1	lncRNA
XM_015092421.1	BBX	mRNA	XR_001436817.1	lncRNA
XM_015092421.1	BBX	mRNA	XR_001436817.1	lncRNA
XM_015092421.1	BBX	mRNA	XR_001436817.1	lncRNA
XM_015092421.1	BBX	mRNA	XR_001436817.1	lncRNA
XM_015092421.1	BBX	mRNA	XR_001436817.1	lncRNA
XM_015092421.1	BBX	mRNA	XR_001436817.1	lncRNA
XM_015092421.1	BBX	mRNA	XR_001436817.1	lncRNA
XM_015092421.1	BBX	mRNA	XR_001436817.1	lncRNA
XM_015092421.1	BBX	mRNA	XR_001436817.1	lncRNA
XM_015098660.1	MAP2K4	mRNA	XR_001436817.1	lncRNA
XM_015098660.1	MAP2K4	mRNA	XR_001436817.1	lncRNA
XM_015098660.1	MAP2K4	mRNA	XR_001436817.1	lncRNA
XM_015098660.1	MAP2K4	mRNA	XR_001436817.1	lncRNA
XM_015098660.1	MAP2K4	mRNA	XR_001436817.1	lncRNA
XM_015098660.1	MAP2K4	mRNA	XR_001436817.1	lncRNA
XM_015098660.1	MAP2K4	mRNA	XR_001436817.1	lncRNA
XM_015098660.1	MAP2K4	mRNA	XR_001436817.1	lncRNA
XM_015098660.1	MAP2K4	mRNA	XR_001436817.1	lncRNA
XM_015098660.1	MAP2K4	mRNA	XR_001436817.1	lncRNA
XM_015098660.1	MAP2K4	mRNA	XR_001436817.1	lncRNA
XM_015098660.1	MAP2K4	mRNA	XR_001436817.1	lncRNA
XM_004005653.3	ZBTB26	mRNA	XR_001436817.1	lncRNA
XM_004005653.3	ZBTB26	mRNA	XR_001436817.1	lncRNA
XM_004005653.3	ZBTB26	mRNA	XR_001436817.1	lncRNA
XM_004005653.3	ZBTB26	mRNA	XR_001436817.1	lncRNA
XM_004005653.3	ZBTB26	mRNA	XR_001436817.1	lncRNA
XM_004005653.3	ZBTB26	mRNA	XR_001436817.1	lncRNA
XM_004005653.3	ZBTB26	mRNA	XR_001436817.1	lncRNA
XM_004005653.3	ZBTB26	mRNA	XR_001436817.1	lncRNA
XM_004005653.3	ZBTB26	mRNA	XR_001436817.1	lncRNA
XM_004005653.3	ZBTB26	mRNA	XR_001436817.1	lncRNA
XM_004005653.3	ZBTB26	mRNA	XR_001436817.1	lncRNA
XM_004005653.3	ZBTB26	mRNA	XR_001436817.1	lncRNA
XM_012098709.2	PML	mRNA	XR_001436817.1	lncRNA
XM_012098709.2	PML	mRNA	XR_001436817.1	lncRNA
XM_012098709.2	PML	mRNA	XR_001436817.1	lncRNA
XM_012098709.2	PML	mRNA	XR_001436817.1	lncRNA

XM_012098709.2	PML	mRNA	XR_001436817.1	lncRNA
XM_012098709.2	PML	mRNA	XR_001436817.1	lncRNA
XM_012098709.2	PML	mRNA	XR_001436817.1	lncRNA
XM_012098709.2	PML	mRNA	XR_001436817.1	lncRNA
XM_012098709.2	PML	mRNA	XR_001436817.1	lncRNA
XM_012098709.2	PML	mRNA	XR_001436817.1	lncRNA
XM_012098709.2	PML	mRNA	XR_001436817.1	lncRNA
XM_012098709.2	PML	mRNA	XR_001436817.1	lncRNA
XM_015098660.1	MAP2K4	mRNA	XR_001434203.1	lncRNA
XM_015098660.1	MAP2K4	mRNA	XR_001434203.1	lncRNA
XM_015098660.1	MAP2K4	mRNA	XR_001434203.1	lncRNA
XM_015098660.1	MAP2K4	mRNA	XR_001434203.1	lncRNA
XM_015098660.1	MAP2K4	mRNA	XR_001434203.1	lncRNA
XM_015098660.1	MAP2K4	mRNA	XR_001434203.1	lncRNA
XM_015098660.1	MAP2K4	mRNA	XR_001434203.1	lncRNA
XM_015098660.1	MAP2K4	mRNA	XR_001434203.1	lncRNA
XM_015098660.1	MAP2K4	mRNA	XR_001434203.1	lncRNA
XM_015098660.1	MAP2K4	mRNA	XR_001434203.1	lncRNA
XM_015098660.1	MAP2K4	mRNA	XR_001434203.1	lncRNA
XM_015098660.1	MAP2K4	mRNA	XR_001434203.1	lncRNA
XM_015103117.1	ZBTB44	mRNA	XR_001434203.1	lncRNA
XM_015103117.1	ZBTB44	mRNA	XR_001434203.1	lncRNA
XM_015103117.1	ZBTB44	mRNA	XR_001434203.1	lncRNA
XM_015103117.1	ZBTB44	mRNA	XR_001434203.1	lncRNA
XM_015103117.1	ZBTB44	mRNA	XR_001434203.1	lncRNA
XM_015103117.1	ZBTB44	mRNA	XR_001434203.1	lncRNA
XM_015103117.1	ZBTB44	mRNA	XR_001434203.1	lncRNA
XM_015103117.1	ZBTB44	mRNA	XR_001434203.1	lncRNA
XM_015103117.1	ZBTB44	mRNA	XR_001434203.1	lncRNA
XM_015103117.1	ZBTB44	mRNA	XR_001434203.1	lncRNA
XM_015103117.1	ZBTB44	mRNA	XR_001434203.1	lncRNA
XM_015103117.1	ZBTB44	mRNA	XR_001434203.1	lncRNA
XM_012106817.2	JADE3	mRNA	XR_001436817.1	lncRNA
XM_012106817.2	JADE3	mRNA	XR_001436817.1	lncRNA
XM_012106817.2	JADE3	mRNA	XR_001436817.1	lncRNA
XM_012106817.2	JADE3	mRNA	XR_001436817.1	lncRNA
XM_012106817.2	JADE3	mRNA	XR_001436817.1	lncRNA
XM_012106817.2	JADE3	mRNA	XR_001436817.1	lncRNA
XM_012106817.2	JADE3	mRNA	XR_001436817.1	lncRNA
XM_012106817.2	JADE3	mRNA	XR_001436817.1	lncRNA
XM_012106817.2	JADE3	mRNA	XR_001436817.1	lncRNA
XM_012106817.2	JADE3	mRNA	XR_001436817.1	lncRNA
XM_012106817.2	JADE3	mRNA	XR_001436817.1	lncRNA
XM_012106817.2	JADE3	mRNA	XR_001436817.1	lncRNA
XM_012096925.2	SLC38A9	mRNA	XR_001434203.1	lncRNA
XM_012096925.2	SLC38A9	mRNA	XR_001434203.1	lncRNA
XM_012096925.2	SLC38A9	mRNA	XR_001434203.1	lncRNA
XM_012096925.2	SLC38A9	mRNA	XR_001434203.1	lncRNA
XM_012096925.2	SLC38A9	mRNA	XR_001434203.1	lncRNA
XM_012096925.2	SLC38A9	mRNA	XR_001434203.1	lncRNA
XM_012096925.2	SLC38A9	mRNA	XR_001434203.1	lncRNA
XM_012096925.2	SLC38A9	mRNA	XR_001434203.1	lncRNA
XM_012096925.2	SLC38A9	mRNA	XR_001434203.1	lncRNA
XM_012096925.2	SLC38A9	mRNA	XR_001434203.1	lncRNA

XM_012096925.2	SLC38A9	mRNA	XR_001434203.1	lncRNA
XM_012096925.2	SLC38A9	mRNA	XR_001434203.1	lncRNA
XM_015099256.1	CEP104	mRNA	XR_001034847.2	lncRNA
XM_015099256.1	CEP104	mRNA	XR_001034847.2	lncRNA
XM_015099256.1	CEP104	mRNA	XR_001034847.2	lncRNA
XM_015099256.1	CEP104	mRNA	XR_001034847.2	lncRNA
XM_015099256.1	CEP104	mRNA	XR_001034847.2	lncRNA
XM_015099256.1	CEP104	mRNA	XR_001034847.2	lncRNA
XM_015099256.1	CEP104	mRNA	XR_001034847.2	lncRNA
XM_015099256.1	CEP104	mRNA	XR_001034847.2	lncRNA
XM_015099256.1	CEP104	mRNA	XR_001034847.2	lncRNA
XM_015099256.1	CEP104	mRNA	XR_001034847.2	lncRNA
XM_015099256.1	CEP104	mRNA	XR_001034847.2	lncRNA
XM_015099256.1	CEP104	mRNA	XR_001034847.2	lncRNA
XM_015099256.1	CEP104	mRNA	XR_001434202.1	lncRNA
XM_015099256.1	CEP104	mRNA	XR_001434202.1	lncRNA
XM_015099256.1	CEP104	mRNA	XR_001434202.1	lncRNA
XM_015099256.1	CEP104	mRNA	XR_001434202.1	lncRNA
XM_015099256.1	CEP104	mRNA	XR_001434202.1	lncRNA
XM_015099256.1	CEP104	mRNA	XR_001434202.1	lncRNA
XM_015099256.1	CEP104	mRNA	XR_001434202.1	lncRNA
XM_015099256.1	CEP104	mRNA	XR_001434202.1	lncRNA
XM_015099256.1	CEP104	mRNA	XR_001434202.1	lncRNA
XM_015099256.1	CEP104	mRNA	XR_001434202.1	lncRNA
XM_015099256.1	CEP104	mRNA	XR_001434202.1	lncRNA
XM_015099256.1	CEP104	mRNA	XR_001434202.1	lncRNA
XM_012185318.2	RAD51D	mRNA	XR_001021689.2	lncRNA
XM_012185318.2	RAD51D	mRNA	XR_001021689.2	lncRNA
XM_012185318.2	RAD51D	mRNA	XR_001021689.2	lncRNA
XM_012185318.2	RAD51D	mRNA	XR_001021689.2	lncRNA
XM_012185318.2	RAD51D	mRNA	XR_001021689.2	lncRNA
XM_012185318.2	RAD51D	mRNA	XR_001021689.2	lncRNA
XM_012185318.2	RAD51D	mRNA	XR_001021689.2	lncRNA
XM_012185318.2	RAD51D	mRNA	XR_001021689.2	lncRNA
XM_012185318.2	RAD51D	mRNA	XR_001021689.2	lncRNA
XM_012185318.2	RAD51D	mRNA	XR_001021689.2	lncRNA
XM_012185318.2	RAD51D	mRNA	XR_001021689.2	lncRNA
XM_012185318.2	RAD51D	mRNA	XR_001021689.2	lncRNA
XM_015091997.1	LOC10112163[illegible]	mRNA	XR_001021689.2	lncRNA
XM_015091997.1	LOC10112163[illegible]	mRNA	XR_001021689.2	lncRNA
XM_015091997.1	LOC10112163[illegible]	mRNA	XR_001021689.2	lncRNA
XM_015091997.1	LOC10112163[illegible]	mRNA	XR_001021689.2	lncRNA
XM_015091997.1	LOC10112163[illegible]	mRNA	XR_001021689.2	lncRNA
XM_015091997.1	LOC10112163[illegible]	mRNA	XR_001021689.2	lncRNA
XM_015091997.1	LOC10112163[illegible]	mRNA	XR_001021689.2	lncRNA
XM_015091997.1	LOC10112163[illegible]	mRNA	XR_001021689.2	lncRNA
XM_015091997.1	LOC10112163[illegible]	mRNA	XR_001021689.2	lncRNA
XM_015091997.1	LOC10112163[illegible]	mRNA	XR_001021689.2	lncRNA
XM_015091997.1	LOC10112163[illegible]	mRNA	XR_001021689.2	lncRNA
XM_015091997.1	LOC10112163[illegible]	mRNA	XR_001021689.2	lncRNA
XM_012178308.2	FAM193B	mRNA	XR_001436127.1	lncRNA
XM_012178308.2	FAM193B	mRNA	XR_001436127.1	lncRNA
XM_012178308.2	FAM193B	mRNA	XR_001436127.1	lncRNA
XM_012178308.2	FAM193B	mRNA	XR_001436127.1	lncRNA

XM_012178308.2	FAM193B	mRNA	XR_001436127.1	lncRNA
XM_012178308.2	FAM193B	mRNA	XR_001436127.1	lncRNA
XM_012178308.2	FAM193B	mRNA	XR_001436127.1	lncRNA
XM_012178308.2	FAM193B	mRNA	XR_001436127.1	lncRNA
XM_012178308.2	FAM193B	mRNA	XR_001436127.1	lncRNA
XM_012178308.2	FAM193B	mRNA	XR_001436127.1	lncRNA
XM_012178308.2	FAM193B	mRNA	XR_001436127.1	lncRNA
XM_004016103.2	USP2	mRNA	XR_001034847.2	lncRNA
XM_004016103.2	USP2	mRNA	XR_001034847.2	lncRNA
XM_004016103.2	USP2	mRNA	XR_001034847.2	lncRNA
XM_004016103.2	USP2	mRNA	XR_001034847.2	lncRNA
XM_004016103.2	USP2	mRNA	XR_001034847.2	lncRNA
XM_004016103.2	USP2	mRNA	XR_001034847.2	lncRNA
XM_004016103.2	USP2	mRNA	XR_001034847.2	lncRNA
XM_004016103.2	USP2	mRNA	XR_001034847.2	lncRNA
XM_004016103.2	USP2	mRNA	XR_001034847.2	lncRNA
XM_004016103.2	USP2	mRNA	XR_001034847.2	lncRNA
XM_004016103.2	USP2	mRNA	XR_001034847.2	lncRNA
XM_012190630.2	PLEKHA4	mRNA	XR_001434202.1	lncRNA
XM_012190630.2	PLEKHA4	mRNA	XR_001434202.1	lncRNA
XM_012190630.2	PLEKHA4	mRNA	XR_001434202.1	lncRNA
XM_012190630.2	PLEKHA4	mRNA	XR_001434202.1	lncRNA
XM_012190630.2	PLEKHA4	mRNA	XR_001434202.1	lncRNA
XM_012190630.2	PLEKHA4	mRNA	XR_001434202.1	lncRNA
XM_012190630.2	PLEKHA4	mRNA	XR_001434202.1	lncRNA
XM_012190630.2	PLEKHA4	mRNA	XR_001434202.1	lncRNA
XM_012190630.2	PLEKHA4	mRNA	XR_001434202.1	lncRNA
XM_012190630.2	PLEKHA4	mRNA	XR_001434202.1	lncRNA
XM_012190630.2	PLEKHA4	mRNA	XR_001434202.1	lncRNA
XM_012190630.2	PLEKHA4	mRNA	XR_001034847.2	lncRNA
XM_012190630.2	PLEKHA4	mRNA	XR_001034847.2	lncRNA
XM_012190630.2	PLEKHA4	mRNA	XR_001034847.2	lncRNA
XM_012190630.2	PLEKHA4	mRNA	XR_001034847.2	lncRNA
XM_012190630.2	PLEKHA4	mRNA	XR_001034847.2	lncRNA
XM_012190630.2	PLEKHA4	mRNA	XR_001034847.2	lncRNA
XM_012190630.2	PLEKHA4	mRNA	XR_001034847.2	lncRNA
XM_012190630.2	PLEKHA4	mRNA	XR_001034847.2	lncRNA
XM_012190630.2	PLEKHA4	mRNA	XR_001034847.2	lncRNA
XM_012190630.2	PLEKHA4	mRNA	XR_001034847.2	lncRNA
XM_012190630.2	PLEKHA4	mRNA	XR_001034847.2	lncRNA
XM_004016103.2	USP2	mRNA	XR_001434202.1	lncRNA
XM_004016103.2	USP2	mRNA	XR_001434202.1	lncRNA
XM_004016103.2	USP2	mRNA	XR_001434202.1	lncRNA
XM_004016103.2	USP2	mRNA	XR_001434202.1	lncRNA
XM_004016103.2	USP2	mRNA	XR_001434202.1	lncRNA
XM_004016103.2	USP2	mRNA	XR_001434202.1	lncRNA
XM_004016103.2	USP2	mRNA	XR_001434202.1	lncRNA
XM_004016103.2	USP2	mRNA	XR_001434202.1	lncRNA
XM_004016103.2	USP2	mRNA	XR_001434202.1	lncRNA
XM_004016103.2	USP2	mRNA	XR_001434202.1	lncRNA
XM_004016103.2	USP2	mRNA	XR_001434202.1	lncRNA
XM_012189779.2	RBL2	mRNA	XR_001436532.1	lncRNA
XM_012189779.2	RBL2	mRNA	XR_001436532.1	lncRNA
XM_012189779.2	RBL2	mRNA	XR_001436532.1	lncRNA

XM_012189779.2	RBL2	mRNA	XR_001436532.1	lncRNA
XM_012189779.2	RBL2	mRNA	XR_001436532.1	lncRNA
XM_012189779.2	RBL2	mRNA	XR_001436532.1	lncRNA
XM_012189779.2	RBL2	mRNA	XR_001436532.1	lncRNA
XM_012189779.2	RBL2	mRNA	XR_001436532.1	lncRNA
XM_012189779.2	RBL2	mRNA	XR_001436532.1	lncRNA
XM_012189779.2	RBL2	mRNA	XR_001436532.1	lncRNA
XM_012189779.2	RBL2	mRNA	XR_001436532.1	lncRNA
XM_012189779.2	RBL2	mRNA	XR_001436532.1	lncRNA
XM_012165515.2	ATAL1B	mRNA	XR_001436532.1	lncRNA
XM_012165515.2	ATAL1B	mRNA	XR_001436532.1	lncRNA
XM_012165515.2	ATAL1B	mRNA	XR_001436532.1	lncRNA
XM_012165515.2	ATAL1B	mRNA	XR_001436532.1	lncRNA
XM_012165515.2	ATAL1B	mRNA	XR_001436532.1	lncRNA
XM_012165515.2	ATAL1B	mRNA	XR_001436532.1	lncRNA
XM_012165515.2	ATAL1B	mRNA	XR_001436532.1	lncRNA
XM_012165515.2	ATAL1B	mRNA	XR_001436532.1	lncRNA
XM_012165515.2	ATAL1B	mRNA	XR_001436532.1	lncRNA
XM_012165515.2	ATAL1B	mRNA	XR_001436532.1	lncRNA
XM_012165515.2	ATAL1B	mRNA	XR_001436532.1	lncRNA
XM_012165515.2	ATAL1B	mRNA	XR_001436532.1	lncRNA
XM_015103495.1	UROS	mRNA	XR_001042381.2	lncRNA
XM_015103495.1	UROS	mRNA	XR_001042381.2	lncRNA
XM_015103495.1	UROS	mRNA	XR_001042381.2	lncRNA
XM_015103495.1	UROS	mRNA	XR_001042381.2	lncRNA
XM_015103495.1	UROS	mRNA	XR_001042381.2	lncRNA
XM_015103495.1	UROS	mRNA	XR_001042381.2	lncRNA
XM_015103495.1	UROS	mRNA	XR_001042381.2	lncRNA
XM_015103495.1	UROS	mRNA	XR_001042381.2	lncRNA
XM_015103495.1	UROS	mRNA	XR_001042381.2	lncRNA
XM_015103495.1	UROS	mRNA	XR_001042381.2	lncRNA
XM_015103495.1	UROS	mRNA	XR_001042381.2	lncRNA
XM_015103495.1	UROS	mRNA	XR_001042381.2	lncRNA
XM_015099551.1	PARD3	mRNA	XR_001434892.1	lncRNA
XM_015099551.1	PARD3	mRNA	XR_001434892.1	lncRNA
XM_015099551.1	PARD3	mRNA	XR_001434892.1	lncRNA
XM_015099551.1	PARD3	mRNA	XR_001434892.1	lncRNA
XM_015099551.1	PARD3	mRNA	XR_001434892.1	lncRNA
XM_015099551.1	PARD3	mRNA	XR_001434892.1	lncRNA
XM_015099551.1	PARD3	mRNA	XR_001434892.1	lncRNA
XM_015099551.1	PARD3	mRNA	XR_001434892.1	lncRNA
XM_015099551.1	PARD3	mRNA	XR_001434892.1	lncRNA
XM_015099551.1	PARD3	mRNA	XR_001434892.1	lncRNA
XM_015099551.1	PARD3	mRNA	XR_001434892.1	lncRNA
XM_015099551.1	PARD3	mRNA	XR_001434892.1	lncRNA
XM_015096356.1	SEC24D	mRNA	XR_001434761.1	lncRNA
XM_015096356.1	SEC24D	mRNA	XR_001434761.1	lncRNA
XM_015096356.1	SEC24D	mRNA	XR_001434761.1	lncRNA
XM_015096356.1	SEC24D	mRNA	XR_001434761.1	lncRNA
XM_015096356.1	SEC24D	mRNA	XR_001434761.1	lncRNA
XM_015096356.1	SEC24D	mRNA	XR_001434761.1	lncRNA
XM_015096356.1	SEC24D	mRNA	XR_001434761.1	lncRNA
XM_015096356.1	SEC24D	mRNA	XR_001434761.1	lncRNA
XM_015096356.1	SEC24D	mRNA	XR_001434761.1	lncRNA

XM_015096356.1	SEC24D	mRNA	XR_001434761.1	lncRNA
XM_015096356.1	SEC24D	mRNA	XR_001434761.1	lncRNA
XM_015096356.1	SEC24D	mRNA	XR_001434761.1	lncRNA
XM_012134416.2	PPIG	mRNA	XR_001434761.1	lncRNA
XM_012134416.2	PPIG	mRNA	XR_001434761.1	lncRNA
XM_012134416.2	PPIG	mRNA	XR_001434761.1	lncRNA
XM_012134416.2	PPIG	mRNA	XR_001434761.1	lncRNA
XM_012134416.2	PPIG	mRNA	XR_001434761.1	lncRNA
XM_012134416.2	PPIG	mRNA	XR_001434761.1	lncRNA
XM_012134416.2	PPIG	mRNA	XR_001434761.1	lncRNA
XM_012134416.2	PPIG	mRNA	XR_001434761.1	lncRNA
XM_012134416.2	PPIG	mRNA	XR_001434761.1	lncRNA
XM_012134416.2	PPIG	mRNA	XR_001434761.1	lncRNA
XM_012134416.2	PPIG	mRNA	XR_001434761.1	lncRNA
XM_012134416.2	PPIG	mRNA	XR_001434761.1	lncRNA
XM_012173296.2	CDKL4	mRNA	XR_001434761.1	lncRNA
XM_012173296.2	CDKL4	mRNA	XR_001434761.1	lncRNA
XM_012173296.2	CDKL4	mRNA	XR_001434761.1	lncRNA
XM_012173296.2	CDKL4	mRNA	XR_001434761.1	lncRNA
XM_012173296.2	CDKL4	mRNA	XR_001434761.1	lncRNA
XM_012173296.2	CDKL4	mRNA	XR_001434761.1	lncRNA
XM_012173296.2	CDKL4	mRNA	XR_001434761.1	lncRNA
XM_012173296.2	CDKL4	mRNA	XR_001434761.1	lncRNA
XM_012173296.2	CDKL4	mRNA	XR_001434761.1	lncRNA
XM_012173296.2	CDKL4	mRNA	XR_001434761.1	lncRNA
XM_012173296.2	CDKL4	mRNA	XR_001434761.1	lncRNA
XM_012173296.2	CDKL4	mRNA	XR_001434761.1	lncRNA
XM_012183728.2	LOC101111337	mRNA	XR_001434892.1	lncRNA
XM_012183728.2	LOC101111337	mRNA	XR_001434892.1	lncRNA
XM_012183728.2	LOC101111337	mRNA	XR_001434892.1	lncRNA
XM_012183728.2	LOC101111337	mRNA	XR_001434892.1	lncRNA
XM_012183728.2	LOC101111337	mRNA	XR_001434892.1	lncRNA
XM_012183728.2	LOC101111337	mRNA	XR_001434892.1	lncRNA
XM_012183728.2	LOC101111337	mRNA	XR_001434892.1	lncRNA
XM_012183728.2	LOC101111337	mRNA	XR_001434892.1	lncRNA
XM_012183728.2	LOC101111337	mRNA	XR_001434892.1	lncRNA
XM_012183728.2	LOC101111337	mRNA	XR_001434892.1	lncRNA
XM_012183728.2	LOC101111337	mRNA	XR_001434892.1	lncRNA
XM_012183728.2	LOC101111337	mRNA	XR_001434892.1	lncRNA
XM_012096323.1	PHF21A	mRNA	XR_001021689.2	lncRNA
XM_012096323.1	PHF21A	mRNA	XR_001021689.2	lncRNA
XM_012096323.1	PHF21A	mRNA	XR_001021689.2	lncRNA
XM_012096323.1	PHF21A	mRNA	XR_001021689.2	lncRNA
XM_012096323.1	PHF21A	mRNA	XR_001021689.2	lncRNA
XM_012096323.1	PHF21A	mRNA	XR_001021689.2	lncRNA
XM_012096323.1	PHF21A	mRNA	XR_001021689.2	lncRNA
XM_012096323.1	PHF21A	mRNA	XR_001021689.2	lncRNA
XM_012096323.1	PHF21A	mRNA	XR_001021689.2	lncRNA
XM_012096323.1	PHF21A	mRNA	XR_001021689.2	lncRNA
XM_012096323.1	PHF21A	mRNA	XR_001021689.2	lncRNA
XM_012096323.1	PHF21A	mRNA	XR_001021689.2	lncRNA
XM_015091836.1	USP40	mRNA	XR_001021689.2	lncRNA
XM_015091836.1	USP40	mRNA	XR_001021689.2	lncRNA
XM_015091836.1	USP40	mRNA	XR_001021689.2	lncRNA

XM_015091836.1	USP40	mRNA	XR_001021689.2	lncRNA
XM_015091836.1	USP40	mRNA	XR_001021689.2	lncRNA
XM_015091836.1	USP40	mRNA	XR_001021689.2	lncRNA
XM_015091836.1	USP40	mRNA	XR_001021689.2	lncRNA
XM_015091836.1	USP40	mRNA	XR_001021689.2	lncRNA
XM_015091836.1	USP40	mRNA	XR_001021689.2	lncRNA
XM_015091836.1	USP40	mRNA	XR_001021689.2	lncRNA
XM_015091836.1	USP40	mRNA	XR_001021689.2	lncRNA
XM_015091836.1	USP40	mRNA	XR_001021689.2	lncRNA
XM_015102486.1	NR2C2	mRNA	XR_001436102.1	lncRNA
XM_015102486.1	NR2C2	mRNA	XR_001436102.1	lncRNA
XM_015102486.1	NR2C2	mRNA	XR_001436102.1	lncRNA
XM_015102486.1	NR2C2	mRNA	XR_001436102.1	lncRNA
XM_015102486.1	NR2C2	mRNA	XR_001436102.1	lncRNA
XM_015102486.1	NR2C2	mRNA	XR_001436102.1	lncRNA
XM_015102486.1	NR2C2	mRNA	XR_001436102.1	lncRNA
XM_015102486.1	NR2C2	mRNA	XR_001436102.1	lncRNA
XM_015102486.1	NR2C2	mRNA	XR_001436102.1	lncRNA
XM_015102486.1	NR2C2	mRNA	XR_001436102.1	lncRNA
XM_015102486.1	NR2C2	mRNA	XR_001436102.1	lncRNA
XM_015102486.1	NR2C2	mRNA	XR_001436102.1	lncRNA
XM_012186944.1	DSTYK	mRNA	XR_001436102.1	lncRNA
XM_012186944.1	DSTYK	mRNA	XR_001436102.1	lncRNA
XM_012186944.1	DSTYK	mRNA	XR_001436102.1	lncRNA
XM_012186944.1	DSTYK	mRNA	XR_001436102.1	lncRNA
XM_012186944.1	DSTYK	mRNA	XR_001436102.1	lncRNA
XM_012186944.1	DSTYK	mRNA	XR_001436102.1	lncRNA
XM_012186944.1	DSTYK	mRNA	XR_001436102.1	lncRNA
XM_012186944.1	DSTYK	mRNA	XR_001436102.1	lncRNA
XM_012186944.1	DSTYK	mRNA	XR_001436102.1	lncRNA
XM_012186944.1	DSTYK	mRNA	XR_001436102.1	lncRNA
XM_012186944.1	DSTYK	mRNA	XR_001436102.1	lncRNA
XM_012186944.1	DSTYK	mRNA	XR_001436102.1	lncRNA
XM_012180879.2	MYO9A	mRNA	XR_001021689.2	lncRNA
XM_012180879.2	MYO9A	mRNA	XR_001021689.2	lncRNA
XM_012180879.2	MYO9A	mRNA	XR_001021689.2	lncRNA
XM_012180879.2	MYO9A	mRNA	XR_001021689.2	lncRNA
XM_012180879.2	MYO9A	mRNA	XR_001021689.2	lncRNA
XM_012180879.2	MYO9A	mRNA	XR_001021689.2	lncRNA
XM_012180879.2	MYO9A	mRNA	XR_001021689.2	lncRNA
XM_012180879.2	MYO9A	mRNA	XR_001021689.2	lncRNA
XM_012180879.2	MYO9A	mRNA	XR_001021689.2	lncRNA
XM_012180879.2	MYO9A	mRNA	XR_001021689.2	lncRNA
XM_012180879.2	MYO9A	mRNA	XR_001021689.2	lncRNA
XM_012180879.2	MYO9A	mRNA	XR_001021689.2	lncRNA
XM_012177309.2	LOC10111622(	mRNA	XR_001021689.2	lncRNA
XM_012177309.2	LOC10111622(	mRNA	XR_001021689.2	lncRNA
XM_012177309.2	LOC10111622(	mRNA	XR_001021689.2	lncRNA
XM_012177309.2	LOC10111622(	mRNA	XR_001021689.2	lncRNA
XM_012177309.2	LOC10111622(	mRNA	XR_001021689.2	lncRNA
XM_012177309.2	LOC10111622(	mRNA	XR_001021689.2	lncRNA
XM_012177309.2	LOC10111622(	mRNA	XR_001021689.2	lncRNA
XM_012177309.2	LOC10111622(	mRNA	XR_001021689.2	lncRNA
XM_012177309.2	LOC10111622(	mRNA	XR_001021689.2	lncRNA

XM_012177309.2	LOC10111622€	mRNA	XR_001021689.2	lncRNA
XM_012177309.2	LOC10111622€	mRNA	XR_001021689.2	lncRNA
XM_012177309.2	LOC10111622€	mRNA	XR_001021689.2	lncRNA
XM_015092323.1	KIRREL	mRNA	XR_001021689.2	lncRNA
XM_015092323.1	KIRREL	mRNA	XR_001021689.2	lncRNA
XM_015092323.1	KIRREL	mRNA	XR_001021689.2	lncRNA
XM_015092323.1	KIRREL	mRNA	XR_001021689.2	lncRNA
XM_015092323.1	KIRREL	mRNA	XR_001021689.2	lncRNA
XM_015092323.1	KIRREL	mRNA	XR_001021689.2	lncRNA
XM_015092323.1	KIRREL	mRNA	XR_001021689.2	lncRNA
XM_015092323.1	KIRREL	mRNA	XR_001021689.2	lncRNA
XM_015092323.1	KIRREL	mRNA	XR_001021689.2	lncRNA
XM_015092323.1	KIRREL	mRNA	XR_001021689.2	lncRNA
XM_015092323.1	KIRREL	mRNA	XR_001021689.2	lncRNA
XM_015092323.1	KIRREL	mRNA	XR_001021689.2	lncRNA
XM_012144682.2	MAP2	mRNA	XR_001034847.2	lncRNA
XM_012144682.2	MAP2	mRNA	XR_001034847.2	lncRNA
XM_012144682.2	MAP2	mRNA	XR_001034847.2	lncRNA
XM_012144682.2	MAP2	mRNA	XR_001034847.2	lncRNA
XM_012144682.2	MAP2	mRNA	XR_001034847.2	lncRNA
XM_012144682.2	MAP2	mRNA	XR_001034847.2	lncRNA
XM_012144682.2	MAP2	mRNA	XR_001034847.2	lncRNA
XM_012144682.2	MAP2	mRNA	XR_001034847.2	lncRNA
XM_012144682.2	MAP2	mRNA	XR_001034847.2	lncRNA
XM_012144682.2	MAP2	mRNA	XR_001034847.2	lncRNA
XM_012144682.2	MAP2	mRNA	XR_001034847.2	lncRNA
XM_012144682.2	MAP2	mRNA	XR_001034847.2	lncRNA
XM_012174394.2	RAPGEF3	mRNA	XR_001034847.2	lncRNA
XM_012174394.2	RAPGEF3	mRNA	XR_001034847.2	lncRNA
XM_012174394.2	RAPGEF3	mRNA	XR_001034847.2	lncRNA
XM_012174394.2	RAPGEF3	mRNA	XR_001034847.2	lncRNA
XM_012174394.2	RAPGEF3	mRNA	XR_001034847.2	lncRNA
XM_012174394.2	RAPGEF3	mRNA	XR_001034847.2	lncRNA
XM_012174394.2	RAPGEF3	mRNA	XR_001034847.2	lncRNA
XM_012174394.2	RAPGEF3	mRNA	XR_001034847.2	lncRNA
XM_012174394.2	RAPGEF3	mRNA	XR_001034847.2	lncRNA
XM_012174394.2	RAPGEF3	mRNA	XR_001034847.2	lncRNA
XM_012174394.2	RAPGEF3	mRNA	XR_001034847.2	lncRNA
XM_012174394.2	RAPGEF3	mRNA	XR_001034847.2	lncRNA
XM_012174394.2	RAPGEF3	mRNA	XR_001434202.1	lncRNA
XM_012174394.2	RAPGEF3	mRNA	XR_001434202.1	lncRNA
XM_012174394.2	RAPGEF3	mRNA	XR_001434202.1	lncRNA
XM_012174394.2	RAPGEF3	mRNA	XR_001434202.1	lncRNA
XM_012174394.2	RAPGEF3	mRNA	XR_001434202.1	lncRNA
XM_012174394.2	RAPGEF3	mRNA	XR_001434202.1	lncRNA
XM_012174394.2	RAPGEF3	mRNA	XR_001434202.1	lncRNA
XM_012174394.2	RAPGEF3	mRNA	XR_001434202.1	lncRNA
XM_012174394.2	RAPGEF3	mRNA	XR_001434202.1	lncRNA
XM_012174394.2	RAPGEF3	mRNA	XR_001434202.1	lncRNA
XM_012174394.2	RAPGEF3	mRNA	XR_001434202.1	lncRNA
XM_012174394.2	RAPGEF3	mRNA	XR_001434202.1	lncRNA
XM_012144682.2	MAP2	mRNA	XR_001434202.1	lncRNA
XM_012144682.2	MAP2	mRNA	XR_001434202.1	lncRNA
XM_012144682.2	MAP2	mRNA	XR_001434202.1	lncRNA

XM_012144682.2	MAP2	mRNA	XR_001434202.1	lncRNA
XM_012144682.2	MAP2	mRNA	XR_001434202.1	lncRNA
XM_012144682.2	MAP2	mRNA	XR_001434202.1	lncRNA
XM_012144682.2	MAP2	mRNA	XR_001434202.1	lncRNA
XM_012144682.2	MAP2	mRNA	XR_001434202.1	lncRNA
XM_012144682.2	MAP2	mRNA	XR_001434202.1	lncRNA
XM_012144682.2	MAP2	mRNA	XR_001434202.1	lncRNA
XM_012144682.2	MAP2	mRNA	XR_001434202.1	lncRNA
XM_012144682.2	MAP2	mRNA	XR_001434202.1	lncRNA
XM_012096925.2	SLC38A9	mRNA	XR_001021689.2	lncRNA
XM_012096925.2	SLC38A9	mRNA	XR_001021689.2	lncRNA
XM_012096925.2	SLC38A9	mRNA	XR_001021689.2	lncRNA
XM_012096925.2	SLC38A9	mRNA	XR_001021689.2	lncRNA
XM_012096925.2	SLC38A9	mRNA	XR_001021689.2	lncRNA
XM_012096925.2	SLC38A9	mRNA	XR_001021689.2	lncRNA
XM_012096925.2	SLC38A9	mRNA	XR_001021689.2	lncRNA
XM_012096925.2	SLC38A9	mRNA	XR_001021689.2	lncRNA
XM_012096925.2	SLC38A9	mRNA	XR_001021689.2	lncRNA
XM_012096925.2	SLC38A9	mRNA	XR_001021689.2	lncRNA
XM_012096925.2	SLC38A9	mRNA	XR_001021689.2	lncRNA
XM_015092421.1	BBX	mRNA	XR_001021689.2	lncRNA
XM_015092421.1	BBX	mRNA	XR_001021689.2	lncRNA
XM_015092421.1	BBX	mRNA	XR_001021689.2	lncRNA
XM_015092421.1	BBX	mRNA	XR_001021689.2	lncRNA
XM_015092421.1	BBX	mRNA	XR_001021689.2	lncRNA
XM_015092421.1	BBX	mRNA	XR_001021689.2	lncRNA
XM_015092421.1	BBX	mRNA	XR_001021689.2	lncRNA
XM_015092421.1	BBX	mRNA	XR_001021689.2	lncRNA
XM_015092421.1	BBX	mRNA	XR_001021689.2	lncRNA
XM_015092421.1	BBX	mRNA	XR_001021689.2	lncRNA
XM_015092421.1	BBX	mRNA	XR_001021689.2	lncRNA
XM_004010552.3	VPS13C	mRNA	XR_001041211.2	lncRNA
XM_004010552.3	VPS13C	mRNA	XR_001041211.2	lncRNA
XM_004010552.3	VPS13C	mRNA	XR_001041211.2	lncRNA
XM_004010552.3	VPS13C	mRNA	XR_001041211.2	lncRNA
XM_004010552.3	VPS13C	mRNA	XR_001041211.2	lncRNA
XM_004010552.3	VPS13C	mRNA	XR_001041211.2	lncRNA
XM_004010552.3	VPS13C	mRNA	XR_001041211.2	lncRNA
XM_004010552.3	VPS13C	mRNA	XR_001041211.2	lncRNA
XM_004010552.3	VPS13C	mRNA	XR_001041211.2	lncRNA
XM_004010552.3	VPS13C	mRNA	XR_001041211.2	lncRNA
XM_004010552.3	VPS13C	mRNA	XR_001041211.2	lncRNA
XM_004010552.3	VPS13C	mRNA	XR_001041211.2	lncRNA
XM_015094471.1	CCDC142	mRNA	XR_001041211.2	lncRNA
XM_015094471.1	CCDC142	mRNA	XR_001041211.2	lncRNA
XM_015094471.1	CCDC142	mRNA	XR_001041211.2	lncRNA
XM_015094471.1	CCDC142	mRNA	XR_001041211.2	lncRNA
XM_015094471.1	CCDC142	mRNA	XR_001041211.2	lncRNA
XM_015094471.1	CCDC142	mRNA	XR_001041211.2	lncRNA
XM_015094471.1	CCDC142	mRNA	XR_001041211.2	lncRNA
XM_015094471.1	CCDC142	mRNA	XR_001041211.2	lncRNA
XM_015094471.1	CCDC142	mRNA	XR_001041211.2	lncRNA
XM_015094471.1	CCDC142	mRNA	XR_001041211.2	lncRNA
XM_015094471.1	CCDC142	mRNA	XR_001041211.2	lncRNA

XM_015094471.1	CCDC142	mRNA	XR_001041211.2	lncRNA
XM_012165515.2	ATAL1B	mRNA	XR_001041211.2	lncRNA
XM_012165515.2	ATAL1B	mRNA	XR_001041211.2	lncRNA
XM_012165515.2	ATAL1B	mRNA	XR_001041211.2	lncRNA
XM_012165515.2	ATAL1B	mRNA	XR_001041211.2	lncRNA
XM_012165515.2	ATAL1B	mRNA	XR_001041211.2	lncRNA
XM_012165515.2	ATAL1B	mRNA	XR_001041211.2	lncRNA
XM_012165515.2	ATAL1B	mRNA	XR_001041211.2	lncRNA
XM_012165515.2	ATAL1B	mRNA	XR_001041211.2	lncRNA
XM_012165515.2	ATAL1B	mRNA	XR_001041211.2	lncRNA
XM_012165515.2	ATAL1B	mRNA	XR_001041211.2	lncRNA
XM_012165515.2	ATAL1B	mRNA	XR_001041211.2	lncRNA
XM_012165515.2	ATAL1B	mRNA	XR_001041211.2	lncRNA
XM_015092486.1	PIGZ	mRNA	XR_001434203.1	lncRNA
XM_015092486.1	PIGZ	mRNA	XR_001434203.1	lncRNA
XM_015092486.1	PIGZ	mRNA	XR_001434203.1	lncRNA
XM_015092486.1	PIGZ	mRNA	XR_001434203.1	lncRNA
XM_015092486.1	PIGZ	mRNA	XR_001434203.1	lncRNA
XM_015092486.1	PIGZ	mRNA	XR_001434203.1	lncRNA
XM_015092486.1	PIGZ	mRNA	XR_001434203.1	lncRNA
XM_015092486.1	PIGZ	mRNA	XR_001434203.1	lncRNA
XM_015092486.1	PIGZ	mRNA	XR_001434203.1	lncRNA
XM_015092486.1	PIGZ	mRNA	XR_001434203.1	lncRNA
XM_015092486.1	PIGZ	mRNA	XR_001434203.1	lncRNA
XM_015092486.1	PIGZ	mRNA	XR_001434203.1	lncRNA
XM_015097502.1	SNX9	mRNA	XR_001436817.1	lncRNA
XM_015097502.1	SNX9	mRNA	XR_001436817.1	lncRNA
XM_015097502.1	SNX9	mRNA	XR_001436817.1	lncRNA
XM_015097502.1	SNX9	mRNA	XR_001436817.1	lncRNA
XM_015097502.1	SNX9	mRNA	XR_001436817.1	lncRNA
XM_015097502.1	SNX9	mRNA	XR_001436817.1	lncRNA
XM_015097502.1	SNX9	mRNA	XR_001436817.1	lncRNA
XM_015097502.1	SNX9	mRNA	XR_001436817.1	lncRNA
XM_015097502.1	SNX9	mRNA	XR_001436817.1	lncRNA
XM_015097502.1	SNX9	mRNA	XR_001436817.1	lncRNA
XM_015097502.1	SNX9	mRNA	XR_001436817.1	lncRNA
XM_015097502.1	SNX9	mRNA	XR_001436817.1	lncRNA
XM_015092486.1	PIGZ	mRNA	XR_001436817.1	lncRNA
XM_015092486.1	PIGZ	mRNA	XR_001436817.1	lncRNA
XM_015092486.1	PIGZ	mRNA	XR_001436817.1	lncRNA
XM_015092486.1	PIGZ	mRNA	XR_001436817.1	lncRNA
XM_015092486.1	PIGZ	mRNA	XR_001436817.1	lncRNA
XM_015092486.1	PIGZ	mRNA	XR_001436817.1	lncRNA
XM_015092486.1	PIGZ	mRNA	XR_001436817.1	lncRNA
XM_015092486.1	PIGZ	mRNA	XR_001436817.1	lncRNA
XM_015092486.1	PIGZ	mRNA	XR_001436817.1	lncRNA
XM_015092486.1	PIGZ	mRNA	XR_001436817.1	lncRNA
XM_015092486.1	PIGZ	mRNA	XR_001436817.1	lncRNA
XM_015092486.1	PIGZ	mRNA	XR_001436817.1	lncRNA
XM_015097502.1	SNX9	mRNA	XR_001434203.1	lncRNA
XM_015097502.1	SNX9	mRNA	XR_001434203.1	lncRNA
XM_015097502.1	SNX9	mRNA	XR_001434203.1	lncRNA
XM_015097502.1	SNX9	mRNA	XR_001434203.1	lncRNA
XM_015097502.1	SNX9	mRNA	XR_001434203.1	lncRNA

XM_015097502.1	SNX9	mRNA	XR_001434203.1	lncRNA
XM_015097502.1	SNX9	mRNA	XR_001434203.1	lncRNA
XM_015097502.1	SNX9	mRNA	XR_001434203.1	lncRNA
XM_015097502.1	SNX9	mRNA	XR_001434203.1	lncRNA
XM_015097502.1	SNX9	mRNA	XR_001434203.1	lncRNA
XM_015097502.1	SNX9	mRNA	XR_001434203.1	lncRNA
XM_015097502.1	SNX9	mRNA	XR_001434203.1	lncRNA
XM_012143416.2	NRP2	mRNA	XR_001436817.1	lncRNA
XM_012143416.2	NRP2	mRNA	XR_001436817.1	lncRNA
XM_012143416.2	NRP2	mRNA	XR_001436817.1	lncRNA
XM_012143416.2	NRP2	mRNA	XR_001436817.1	lncRNA
XM_012143416.2	NRP2	mRNA	XR_001436817.1	lncRNA
XM_012143416.2	NRP2	mRNA	XR_001436817.1	lncRNA
XM_012143416.2	NRP2	mRNA	XR_001436817.1	lncRNA
XM_012143416.2	NRP2	mRNA	XR_001436817.1	lncRNA
XM_012143416.2	NRP2	mRNA	XR_001436817.1	lncRNA
XM_012143416.2	NRP2	mRNA	XR_001436817.1	lncRNA
XM_012143416.2	NRP2	mRNA	XR_001436817.1	lncRNA
XM_012143416.2	NRP2	mRNA	XR_001436817.1	lncRNA
XM_015103495.1	UROS	mRNA	XR_001436817.1	lncRNA
XM_015103495.1	UROS	mRNA	XR_001436817.1	lncRNA
XM_015103495.1	UROS	mRNA	XR_001436817.1	lncRNA
XM_015103495.1	UROS	mRNA	XR_001436817.1	lncRNA
XM_015103495.1	UROS	mRNA	XR_001436817.1	lncRNA
XM_015103495.1	UROS	mRNA	XR_001436817.1	lncRNA
XM_015103495.1	UROS	mRNA	XR_001436817.1	lncRNA
XM_015103495.1	UROS	mRNA	XR_001436817.1	lncRNA
XM_015103495.1	UROS	mRNA	XR_001436817.1	lncRNA
XM_015103495.1	UROS	mRNA	XR_001436817.1	lncRNA
XM_015103495.1	UROS	mRNA	XR_001436817.1	lncRNA
XM_015103495.1	UROS	mRNA	XR_001436817.1	lncRNA
XM_015095790.1	CC2D1A	mRNA	XR_001436817.1	lncRNA
XM_015095790.1	CC2D1A	mRNA	XR_001436817.1	lncRNA
XM_015095790.1	CC2D1A	mRNA	XR_001436817.1	lncRNA
XM_015095790.1	CC2D1A	mRNA	XR_001436817.1	lncRNA
XM_015095790.1	CC2D1A	mRNA	XR_001436817.1	lncRNA
XM_015095790.1	CC2D1A	mRNA	XR_001436817.1	lncRNA
XM_015095790.1	CC2D1A	mRNA	XR_001436817.1	lncRNA
XM_015095790.1	CC2D1A	mRNA	XR_001436817.1	lncRNA
XM_015095790.1	CC2D1A	mRNA	XR_001436817.1	lncRNA
XM_015095790.1	CC2D1A	mRNA	XR_001436817.1	lncRNA
XM_015095790.1	CC2D1A	mRNA	XR_001436817.1	lncRNA
XM_015095790.1	CC2D1A	mRNA	XR_001436817.1	lncRNA
XM_012185926.2	DLX4	mRNA	XR_001436102.1	lncRNA
XM_012185926.2	DLX4	mRNA	XR_001436102.1	lncRNA
XM_012185926.2	DLX4	mRNA	XR_001436102.1	lncRNA
XM_012185926.2	DLX4	mRNA	XR_001436102.1	lncRNA
XM_012185926.2	DLX4	mRNA	XR_001436102.1	lncRNA
XM_012185926.2	DLX4	mRNA	XR_001436102.1	lncRNA
XM_012185926.2	DLX4	mRNA	XR_001436102.1	lncRNA
XM_012185926.2	DLX4	mRNA	XR_001436102.1	lncRNA
XM_012185926.2	DLX4	mRNA	XR_001436102.1	lncRNA
XM_012185926.2	DLX4	mRNA	XR_001436102.1	lncRNA
XM_012185926.2	DLX4	mRNA	XR_001436102.1	lncRNA

XM_012185926.2	DLX4	mRNA	XR_001436102.1	lncRNA
XM_015096937.1	BMF	mRNA	XR_001436102.1	lncRNA
XM_015096937.1	BMF	mRNA	XR_001436102.1	lncRNA
XM_015096937.1	BMF	mRNA	XR_001436102.1	lncRNA
XM_015096937.1	BMF	mRNA	XR_001436102.1	lncRNA
XM_015096937.1	BMF	mRNA	XR_001436102.1	lncRNA
XM_015096937.1	BMF	mRNA	XR_001436102.1	lncRNA
XM_015096937.1	BMF	mRNA	XR_001436102.1	lncRNA
XM_015096937.1	BMF	mRNA	XR_001436102.1	lncRNA
XM_015096937.1	BMF	mRNA	XR_001436102.1	lncRNA
XM_015096937.1	BMF	mRNA	XR_001436102.1	lncRNA
XM_015096937.1	BMF	mRNA	XR_001436102.1	lncRNA
XM_015096937.1	BMF	mRNA	XR_001436102.1	lncRNA
XM_015096598.1	LIN54	mRNA	XR_001434761.1	lncRNA
XM_015096598.1	LIN54	mRNA	XR_001434761.1	lncRNA
XM_015096598.1	LIN54	mRNA	XR_001434761.1	lncRNA
XM_015096598.1	LIN54	mRNA	XR_001434761.1	lncRNA
XM_015096598.1	LIN54	mRNA	XR_001434761.1	lncRNA
XM_015096598.1	LIN54	mRNA	XR_001434761.1	lncRNA
XM_015096598.1	LIN54	mRNA	XR_001434761.1	lncRNA
XM_015096598.1	LIN54	mRNA	XR_001434761.1	lncRNA
XM_015096598.1	LIN54	mRNA	XR_001434761.1	lncRNA
XM_015096598.1	LIN54	mRNA	XR_001434761.1	lncRNA
XM_015096598.1	LIN54	mRNA	XR_001434761.1	lncRNA
XM_015096598.1	LIN54	mRNA	XR_001434761.1	lncRNA
XM_012182026.2	ENTPD5	mRNA	XR_001434761.1	lncRNA
XM_012182026.2	ENTPD5	mRNA	XR_001434761.1	lncRNA
XM_012182026.2	ENTPD5	mRNA	XR_001434761.1	lncRNA
XM_012182026.2	ENTPD5	mRNA	XR_001434761.1	lncRNA
XM_012182026.2	ENTPD5	mRNA	XR_001434761.1	lncRNA
XM_012182026.2	ENTPD5	mRNA	XR_001434761.1	lncRNA
XM_012182026.2	ENTPD5	mRNA	XR_001434761.1	lncRNA
XM_012182026.2	ENTPD5	mRNA	XR_001434761.1	lncRNA
XM_012182026.2	ENTPD5	mRNA	XR_001434761.1	lncRNA
XM_012182026.2	ENTPD5	mRNA	XR_001434761.1	lncRNA
XM_012182026.2	ENTPD5	mRNA	XR_001434761.1	lncRNA
XM_012182026.2	ENTPD5	mRNA	XR_001434761.1	lncRNA
XM_012097680.2	SCARB1	mRNA	XR_001434761.1	lncRNA
XM_012097680.2	SCARB1	mRNA	XR_001434761.1	lncRNA
XM_012097680.2	SCARB1	mRNA	XR_001434761.1	lncRNA
XM_012097680.2	SCARB1	mRNA	XR_001434761.1	lncRNA
XM_012097680.2	SCARB1	mRNA	XR_001434761.1	lncRNA
XM_012097680.2	SCARB1	mRNA	XR_001434761.1	lncRNA
XM_012097680.2	SCARB1	mRNA	XR_001434761.1	lncRNA
XM_012097680.2	SCARB1	mRNA	XR_001434761.1	lncRNA
XM_012097680.2	SCARB1	mRNA	XR_001434761.1	lncRNA
XM_012097680.2	SCARB1	mRNA	XR_001434761.1	lncRNA
XM_012097680.2	SCARB1	mRNA	XR_001434761.1	lncRNA
XM_012097680.2	SCARB1	mRNA	XR_001434761.1	lncRNA
XM_012186944.1	DSTYK	mRNA	XR_001434202.1	lncRNA
XM_012186944.1	DSTYK	mRNA	XR_001434202.1	lncRNA
XM_012186944.1	DSTYK	mRNA	XR_001434202.1	lncRNA
XM_012186944.1	DSTYK	mRNA	XR_001434202.1	lncRNA
XM_012186944.1	DSTYK	mRNA	XR_001434202.1	lncRNA

XM_012186944.1	DSTYK	mRNA	XR_001434202.1	lncRNA
XM_012186944.1	DSTYK	mRNA	XR_001434202.1	lncRNA
XM_012186944.1	DSTYK	mRNA	XR_001434202.1	lncRNA
XM_012186944.1	DSTYK	mRNA	XR_001434202.1	lncRNA
XM_012186944.1	DSTYK	mRNA	XR_001434202.1	lncRNA
XM_012186944.1	DSTYK	mRNA	XR_001434202.1	lncRNA
XM_012186944.1	DSTYK	mRNA	XR_001434202.1	lncRNA
XM_012186944.1	DSTYK	mRNA	XR_001034847.2	lncRNA
XM_012186944.1	DSTYK	mRNA	XR_001034847.2	lncRNA
XM_012186944.1	DSTYK	mRNA	XR_001034847.2	lncRNA
XM_012186944.1	DSTYK	mRNA	XR_001034847.2	lncRNA
XM_012186944.1	DSTYK	mRNA	XR_001034847.2	lncRNA
XM_012186944.1	DSTYK	mRNA	XR_001034847.2	lncRNA
XM_012186944.1	DSTYK	mRNA	XR_001034847.2	lncRNA
XM_012186944.1	DSTYK	mRNA	XR_001034847.2	lncRNA
XM_012186944.1	DSTYK	mRNA	XR_001034847.2	lncRNA
XM_012186944.1	DSTYK	mRNA	XR_001034847.2	lncRNA
XM_012186944.1	DSTYK	mRNA	XR_001034847.2	lncRNA
XM_012186944.1	DSTYK	mRNA	XR_001034847.2	lncRNA
XM_012176498.2	RAPGEF5	mRNA	XR_001024290.1	lncRNA
XM_012176498.2	RAPGEF5	mRNA	XR_001024290.1	lncRNA
XM_012176498.2	RAPGEF5	mRNA	XR_001024290.1	lncRNA
XM_012176498.2	RAPGEF5	mRNA	XR_001024290.1	lncRNA
XM_012176498.2	RAPGEF5	mRNA	XR_001024290.1	lncRNA
XM_012176498.2	RAPGEF5	mRNA	XR_001024290.1	lncRNA
XM_012176498.2	RAPGEF5	mRNA	XR_001024290.1	lncRNA
XM_012176498.2	RAPGEF5	mRNA	XR_001024290.1	lncRNA
XM_012176498.2	RAPGEF5	mRNA	XR_001024290.1	lncRNA
XM_012176498.2	RAPGEF5	mRNA	XR_001024290.1	lncRNA
XM_012176498.2	RAPGEF5	mRNA	XR_001024290.1	lncRNA
XM_012178306.2	FAM193B	mRNA	XR_001436127.1	lncRNA
XM_012178306.2	FAM193B	mRNA	XR_001436127.1	lncRNA
XM_012178306.2	FAM193B	mRNA	XR_001436127.1	lncRNA
XM_012178306.2	FAM193B	mRNA	XR_001436127.1	lncRNA
XM_012178306.2	FAM193B	mRNA	XR_001436127.1	lncRNA
XM_012178306.2	FAM193B	mRNA	XR_001436127.1	lncRNA
XM_012178306.2	FAM193B	mRNA	XR_001436127.1	lncRNA
XM_012178306.2	FAM193B	mRNA	XR_001436127.1	lncRNA
XM_012178306.2	FAM193B	mRNA	XR_001436127.1	lncRNA
XM_012178306.2	FAM193B	mRNA	XR_001436127.1	lncRNA
XM_012178306.2	FAM193B	mRNA	XR_001436127.1	lncRNA
XM_015099551.1	PARD3	mRNA	XR_001436532.1	lncRNA
XM_015099551.1	PARD3	mRNA	XR_001436532.1	lncRNA
XM_015099551.1	PARD3	mRNA	XR_001436532.1	lncRNA
XM_015099551.1	PARD3	mRNA	XR_001436532.1	lncRNA
XM_015099551.1	PARD3	mRNA	XR_001436532.1	lncRNA
XM_015099551.1	PARD3	mRNA	XR_001436532.1	lncRNA
XM_015099551.1	PARD3	mRNA	XR_001436532.1	lncRNA
XM_015099551.1	PARD3	mRNA	XR_001436532.1	lncRNA
XM_015099551.1	PARD3	mRNA	XR_001436532.1	lncRNA
XM_015099551.1	PARD3	mRNA	XR_001436532.1	lncRNA
XM_015099551.1	PARD3	mRNA	XR_001436532.1	lncRNA
XM_015099551.1	PARD3	mRNA	XR_001436532.1	lncRNA
XM_012183412.2	ZNF7	mRNA	XR_001042381.2	lncRNA

XM_012183412.2	ZNF7	mRNA	XR_001042381.2	lncRNA
XM_012183412.2	ZNF7	mRNA	XR_001042381.2	lncRNA
XM_012183412.2	ZNF7	mRNA	XR_001042381.2	lncRNA
XM_012183412.2	ZNF7	mRNA	XR_001042381.2	lncRNA
XM_012183412.2	ZNF7	mRNA	XR_001042381.2	lncRNA
XM_012183412.2	ZNF7	mRNA	XR_001042381.2	lncRNA
XM_012183412.2	ZNF7	mRNA	XR_001042381.2	lncRNA
XM_012183412.2	ZNF7	mRNA	XR_001042381.2	lncRNA
XM_012183412.2	ZNF7	mRNA	XR_001042381.2	lncRNA
XM_012183412.2	ZNF7	mRNA	XR_001042381.2	lncRNA
XM_012183412.2	ZNF7	mRNA	XR_001042381.2	lncRNA
XM_012185799.2	EPN2	mRNA	XR_001021689.2	lncRNA
XM_012185799.2	EPN2	mRNA	XR_001021689.2	lncRNA
XM_012185799.2	EPN2	mRNA	XR_001021689.2	lncRNA
XM_012185799.2	EPN2	mRNA	XR_001021689.2	lncRNA
XM_012185799.2	EPN2	mRNA	XR_001021689.2	lncRNA
XM_012185799.2	EPN2	mRNA	XR_001021689.2	lncRNA
XM_012185799.2	EPN2	mRNA	XR_001021689.2	lncRNA
XM_012185799.2	EPN2	mRNA	XR_001021689.2	lncRNA
XM_012185799.2	EPN2	mRNA	XR_001021689.2	lncRNA
XM_012185799.2	EPN2	mRNA	XR_001021689.2	lncRNA
XM_012185799.2	EPN2	mRNA	XR_001021689.2	lncRNA
XM_012185799.2	EPN2	mRNA	XR_001021689.2	lncRNA
XM_015103461.1	TACC2	mRNA	XR_001021689.2	lncRNA
XM_015103461.1	TACC2	mRNA	XR_001021689.2	lncRNA
XM_015103461.1	TACC2	mRNA	XR_001021689.2	lncRNA
XM_015103461.1	TACC2	mRNA	XR_001021689.2	lncRNA
XM_015103461.1	TACC2	mRNA	XR_001021689.2	lncRNA
XM_015103461.1	TACC2	mRNA	XR_001021689.2	lncRNA
XM_015103461.1	TACC2	mRNA	XR_001021689.2	lncRNA
XM_015103461.1	TACC2	mRNA	XR_001021689.2	lncRNA
XM_015103461.1	TACC2	mRNA	XR_001021689.2	lncRNA
XM_015103461.1	TACC2	mRNA	XR_001021689.2	lncRNA
XM_015103461.1	TACC2	mRNA	XR_001021689.2	lncRNA
XM_015103461.1	TACC2	mRNA	XR_001021689.2	lncRNA
XM_015103475.1	PLEKHA1	mRNA	XR_001436102.1	lncRNA
XM_015103475.1	PLEKHA1	mRNA	XR_001436102.1	lncRNA
XM_015103475.1	PLEKHA1	mRNA	XR_001436102.1	lncRNA
XM_015103475.1	PLEKHA1	mRNA	XR_001436102.1	lncRNA
XM_015103475.1	PLEKHA1	mRNA	XR_001436102.1	lncRNA
XM_015103475.1	PLEKHA1	mRNA	XR_001436102.1	lncRNA
XM_015103475.1	PLEKHA1	mRNA	XR_001436102.1	lncRNA
XM_015103475.1	PLEKHA1	mRNA	XR_001436102.1	lncRNA
XM_015103475.1	PLEKHA1	mRNA	XR_001436102.1	lncRNA
XM_015103475.1	PLEKHA1	mRNA	XR_001436102.1	lncRNA
XM_015103475.1	PLEKHA1	mRNA	XR_001436102.1	lncRNA
XM_012181966.2	SIPA1L1	mRNA	XR_001024290.1	lncRNA
XM_012181966.2	SIPA1L1	mRNA	XR_001024290.1	lncRNA
XM_012181966.2	SIPA1L1	mRNA	XR_001024290.1	lncRNA
XM_012181966.2	SIPA1L1	mRNA	XR_001024290.1	lncRNA
XM_012181966.2	SIPA1L1	mRNA	XR_001024290.1	lncRNA
XM_012181966.2	SIPA1L1	mRNA	XR_001024290.1	lncRNA
XM_012181966.2	SIPA1L1	mRNA	XR_001024290.1	lncRNA
XM_012181966.2	SIPA1L1	mRNA	XR_001024290.1	lncRNA

XM_012181966.2	SIPA1L1	mRNA	XR_001024290.1	lncRNA
XM_012181966.2	SIPA1L1	mRNA	XR_001024290.1	lncRNA
XM_012181966.2	SIPA1L1	mRNA	XR_001024290.1	lncRNA
XM_012137434.2	NEB	mRNA	XR_001034847.2	lncRNA
XM_012137434.2	NEB	mRNA	XR_001034847.2	lncRNA
XM_012137434.2	NEB	mRNA	XR_001034847.2	lncRNA
XM_012137434.2	NEB	mRNA	XR_001034847.2	lncRNA
XM_012137434.2	NEB	mRNA	XR_001034847.2	lncRNA
XM_012137434.2	NEB	mRNA	XR_001034847.2	lncRNA
XM_012137434.2	NEB	mRNA	XR_001034847.2	lncRNA
XM_012137434.2	NEB	mRNA	XR_001034847.2	lncRNA
XM_012137434.2	NEB	mRNA	XR_001034847.2	lncRNA
XM_012137434.2	NEB	mRNA	XR_001034847.2	lncRNA
XM_012137434.2	NEB	mRNA	XR_001034847.2	lncRNA
XM_012137434.2	NEB	mRNA	XR_001034847.2	lncRNA
XM_012137434.2	NEB	mRNA	XR_001434202.1	lncRNA
XM_012137434.2	NEB	mRNA	XR_001434202.1	lncRNA
XM_012137434.2	NEB	mRNA	XR_001434202.1	lncRNA
XM_012137434.2	NEB	mRNA	XR_001434202.1	lncRNA
XM_012137434.2	NEB	mRNA	XR_001434202.1	lncRNA
XM_012137434.2	NEB	mRNA	XR_001434202.1	lncRNA
XM_012137434.2	NEB	mRNA	XR_001434202.1	lncRNA
XM_012137434.2	NEB	mRNA	XR_001434202.1	lncRNA
XM_012137434.2	NEB	mRNA	XR_001434202.1	lncRNA
XM_012137434.2	NEB	mRNA	XR_001434202.1	lncRNA
XM_012137434.2	NEB	mRNA	XR_001434202.1	lncRNA
XM_012137434.2	NEB	mRNA	XR_001434202.1	lncRNA
XM_012137425.2	NEB	mRNA	XR_001034847.2	lncRNA
XM_012137425.2	NEB	mRNA	XR_001034847.2	lncRNA
XM_012137425.2	NEB	mRNA	XR_001034847.2	lncRNA
XM_012137425.2	NEB	mRNA	XR_001034847.2	lncRNA
XM_012137425.2	NEB	mRNA	XR_001034847.2	lncRNA
XM_012137425.2	NEB	mRNA	XR_001034847.2	lncRNA
XM_012137425.2	NEB	mRNA	XR_001034847.2	lncRNA
XM_012137425.2	NEB	mRNA	XR_001034847.2	lncRNA
XM_012137425.2	NEB	mRNA	XR_001034847.2	lncRNA
XM_012137425.2	NEB	mRNA	XR_001034847.2	lncRNA
XM_012137425.2	NEB	mRNA	XR_001034847.2	lncRNA
XM_012137425.2	NEB	mRNA	XR_001034847.2	lncRNA
XM_012137425.2	NEB	mRNA	XR_001434202.1	lncRNA
XM_012137425.2	NEB	mRNA	XR_001434202.1	lncRNA
XM_012137425.2	NEB	mRNA	XR_001434202.1	lncRNA
XM_012137425.2	NEB	mRNA	XR_001434202.1	lncRNA
XM_012137425.2	NEB	mRNA	XR_001434202.1	lncRNA
XM_012137425.2	NEB	mRNA	XR_001434202.1	lncRNA
XM_012137425.2	NEB	mRNA	XR_001434202.1	lncRNA
XM_012137425.2	NEB	mRNA	XR_001434202.1	lncRNA
XM_012137425.2	NEB	mRNA	XR_001434202.1	lncRNA
XM_012137425.2	NEB	mRNA	XR_001434202.1	lncRNA
XM_012137425.2	NEB	mRNA	XR_001434202.1	lncRNA
XM_012137425.2	NEB	mRNA	XR_001434202.1	lncRNA
XM_012174704.2	ZBTB39	mRNA	XR_001434202.1	lncRNA
XM_012174704.2	ZBTB39	mRNA	XR_001434202.1	lncRNA
XM_012174704.2	ZBTB39	mRNA	XR_001434202.1	lncRNA

XM_012174704.2	ZBTB39	mRNA	XR_001434202.1	lncRNA
XM_012174704.2	ZBTB39	mRNA	XR_001434202.1	lncRNA
XM_012174704.2	ZBTB39	mRNA	XR_001434202.1	lncRNA
XM_012174704.2	ZBTB39	mRNA	XR_001434202.1	lncRNA
XM_012174704.2	ZBTB39	mRNA	XR_001434202.1	lncRNA
XM_012174704.2	ZBTB39	mRNA	XR_001434202.1	lncRNA
XM_012174704.2	ZBTB39	mRNA	XR_001434202.1	lncRNA
XM_012174704.2	ZBTB39	mRNA	XR_001434202.1	lncRNA
XM_012174704.2	ZBTB39	mRNA	XR_001434202.1	lncRNA
XM_012174704.2	ZBTB39	mRNA	XR_001034847.2	lncRNA
XM_012174704.2	ZBTB39	mRNA	XR_001034847.2	lncRNA
XM_012174704.2	ZBTB39	mRNA	XR_001034847.2	lncRNA
XM_012174704.2	ZBTB39	mRNA	XR_001034847.2	lncRNA
XM_012174704.2	ZBTB39	mRNA	XR_001034847.2	lncRNA
XM_012174704.2	ZBTB39	mRNA	XR_001034847.2	lncRNA
XM_012174704.2	ZBTB39	mRNA	XR_001034847.2	lncRNA
XM_012174704.2	ZBTB39	mRNA	XR_001034847.2	lncRNA
XM_012174704.2	ZBTB39	mRNA	XR_001034847.2	lncRNA
XM_012174704.2	ZBTB39	mRNA	XR_001034847.2	lncRNA
XM_012174704.2	ZBTB39	mRNA	XR_001034847.2	lncRNA
XM_012174704.2	ZBTB39	mRNA	XR_001034847.2	lncRNA
XM_015097018.1	ZNF280D	mRNA	XR_001042381.2	lncRNA
XM_015097018.1	ZNF280D	mRNA	XR_001042381.2	lncRNA
XM_015097018.1	ZNF280D	mRNA	XR_001042381.2	lncRNA
XM_015097018.1	ZNF280D	mRNA	XR_001042381.2	lncRNA
XM_015097018.1	ZNF280D	mRNA	XR_001042381.2	lncRNA
XM_015097018.1	ZNF280D	mRNA	XR_001042381.2	lncRNA
XM_015097018.1	ZNF280D	mRNA	XR_001042381.2	lncRNA
XM_015097018.1	ZNF280D	mRNA	XR_001042381.2	lncRNA
XM_015097018.1	ZNF280D	mRNA	XR_001042381.2	lncRNA
XM_015097018.1	ZNF280D	mRNA	XR_001042381.2	lncRNA
XM_015097018.1	ZNF280D	mRNA	XR_001042381.2	lncRNA
XM_012175416.2	PKN2	mRNA	XR_001436532.1	lncRNA
XM_012175416.2	PKN2	mRNA	XR_001436532.1	lncRNA
XM_012175416.2	PKN2	mRNA	XR_001436532.1	lncRNA
XM_012175416.2	PKN2	mRNA	XR_001436532.1	lncRNA
XM_012175416.2	PKN2	mRNA	XR_001436532.1	lncRNA
XM_012175416.2	PKN2	mRNA	XR_001436532.1	lncRNA
XM_012175416.2	PKN2	mRNA	XR_001436532.1	lncRNA
XM_012175416.2	PKN2	mRNA	XR_001436532.1	lncRNA
XM_012175416.2	PKN2	mRNA	XR_001436532.1	lncRNA
XM_012175416.2	PKN2	mRNA	XR_001436532.1	lncRNA
XM_012175416.2	PKN2	mRNA	XR_001436532.1	lncRNA
XM_004003552.3	LOC10112182(	mRNA	XR_001436532.1	lncRNA
XM_004003552.3	LOC10112182(	mRNA	XR_001436532.1	lncRNA
XM_004003552.3	LOC10112182(	mRNA	XR_001436532.1	lncRNA
XM_004003552.3	LOC10112182(	mRNA	XR_001436532.1	lncRNA
XM_004003552.3	LOC10112182(	mRNA	XR_001436532.1	lncRNA
XM_004003552.3	LOC10112182(	mRNA	XR_001436532.1	lncRNA
XM_004003552.3	LOC10112182(	mRNA	XR_001436532.1	lncRNA
XM_004003552.3	LOC10112182(	mRNA	XR_001436532.1	lncRNA
XM_004003552.3	LOC10112182(	mRNA	XR_001436532.1	lncRNA
XM_004003552.3	LOC10112182(	mRNA	XR_001436532.1	lncRNA
XM_004003552.3	LOC10112182(	mRNA	XR_001436532.1	lncRNA

XM_004003552.3	LOC10112182(	mRNA	XR_001436127.1	lncRNA
XM_004003552.3	LOC10112182(	mRNA	XR_001436127.1	lncRNA
XM_004003552.3	LOC10112182(	mRNA	XR_001436127.1	lncRNA
XM_004003552.3	LOC10112182(	mRNA	XR_001436127.1	lncRNA
XM_004003552.3	LOC10112182(	mRNA	XR_001436127.1	lncRNA
XM_004003552.3	LOC10112182(	mRNA	XR_001436127.1	lncRNA
XM_004003552.3	LOC10112182(	mRNA	XR_001436127.1	lncRNA
XM_004003552.3	LOC10112182(	mRNA	XR_001436127.1	lncRNA
XM_004003552.3	LOC10112182(	mRNA	XR_001436127.1	lncRNA
XM_004003552.3	LOC10112182(	mRNA	XR_001436127.1	lncRNA
XM_012181247.2	MGA	mRNA	XR_001436532.1	lncRNA
XM_012181247.2	MGA	mRNA	XR_001436532.1	lncRNA
XM_012181247.2	MGA	mRNA	XR_001436532.1	lncRNA
XM_012181247.2	MGA	mRNA	XR_001436532.1	lncRNA
XM_012181247.2	MGA	mRNA	XR_001436532.1	lncRNA
XM_012181247.2	MGA	mRNA	XR_001436532.1	lncRNA
XM_012181247.2	MGA	mRNA	XR_001436532.1	lncRNA
XM_012181247.2	MGA	mRNA	XR_001436532.1	lncRNA
XM_012181247.2	MGA	mRNA	XR_001436532.1	lncRNA
XM_012181247.2	MGA	mRNA	XR_001436532.1	lncRNA
XM_012181247.2	MGA	mRNA	XR_001436532.1	lncRNA
XM_012181247.2	MGA	mRNA	XR_001436532.1	lncRNA
XM_015094531.1	ANAPC1	mRNA	XR_001436102.1	lncRNA
XM_015094531.1	ANAPC1	mRNA	XR_001436102.1	lncRNA
XM_015094531.1	ANAPC1	mRNA	XR_001436102.1	lncRNA
XM_015094531.1	ANAPC1	mRNA	XR_001436102.1	lncRNA
XM_015094531.1	ANAPC1	mRNA	XR_001436102.1	lncRNA
XM_015094531.1	ANAPC1	mRNA	XR_001436102.1	lncRNA
XM_015094531.1	ANAPC1	mRNA	XR_001436102.1	lncRNA
XM_015094531.1	ANAPC1	mRNA	XR_001436102.1	lncRNA
XM_015094531.1	ANAPC1	mRNA	XR_001436102.1	lncRNA
XM_015094531.1	ANAPC1	mRNA	XR_001436102.1	lncRNA
XM_015094531.1	ANAPC1	mRNA	XR_001436102.1	lncRNA
XM_015094531.1	ANAPC1	mRNA	XR_001436102.1	lncRNA
XM_012179910.2	ATP10D	mRNA	XR_001041211.2	lncRNA
XM_012179910.2	ATP10D	mRNA	XR_001041211.2	lncRNA
XM_012179910.2	ATP10D	mRNA	XR_001041211.2	lncRNA
XM_012179910.2	ATP10D	mRNA	XR_001041211.2	lncRNA
XM_012179910.2	ATP10D	mRNA	XR_001041211.2	lncRNA
XM_012179910.2	ATP10D	mRNA	XR_001041211.2	lncRNA
XM_012179910.2	ATP10D	mRNA	XR_001041211.2	lncRNA
XM_012179910.2	ATP10D	mRNA	XR_001041211.2	lncRNA
XM_012179910.2	ATP10D	mRNA	XR_001041211.2	lncRNA
XM_012179910.2	ATP10D	mRNA	XR_001041211.2	lncRNA
XM_012179910.2	ATP10D	mRNA	XR_001041211.2	lncRNA
XM_012096185.2	TCP11L1	mRNA	XR_001041211.2	lncRNA
XM_012096185.2	TCP11L1	mRNA	XR_001041211.2	lncRNA
XM_012096185.2	TCP11L1	mRNA	XR_001041211.2	lncRNA
XM_012096185.2	TCP11L1	mRNA	XR_001041211.2	lncRNA
XM_012096185.2	TCP11L1	mRNA	XR_001041211.2	lncRNA
XM_012096185.2	TCP11L1	mRNA	XR_001041211.2	lncRNA
XM_012096185.2	TCP11L1	mRNA	XR_001041211.2	lncRNA
XM_012096185.2	TCP11L1	mRNA	XR_001041211.2	lncRNA
XM_012096185.2	TCP11L1	mRNA	XR_001041211.2	lncRNA

XM_012096185.2	TCP11L1	mRNA	XR_001041211.2	lncRNA
XM_012096185.2	TCP11L1	mRNA	XR_001041211.2	lncRNA
XM_004003552.3	LOC10112182(	mRNA	XR_001041211.2	lncRNA
XM_004003552.3	LOC10112182(	mRNA	XR_001041211.2	lncRNA
XM_004003552.3	LOC10112182(	mRNA	XR_001041211.2	lncRNA
XM_004003552.3	LOC10112182(	mRNA	XR_001041211.2	lncRNA
XM_004003552.3	LOC10112182(	mRNA	XR_001041211.2	lncRNA
XM_004003552.3	LOC10112182(	mRNA	XR_001041211.2	lncRNA
XM_004003552.3	LOC10112182(	mRNA	XR_001041211.2	lncRNA
XM_004003552.3	LOC10112182(	mRNA	XR_001041211.2	lncRNA
XM_004003552.3	LOC10112182(	mRNA	XR_001041211.2	lncRNA
XM_004003552.3	LOC10112182(	mRNA	XR_001041211.2	lncRNA
XM_004003552.3	LOC10112182(	mRNA	XR_001041211.2	lncRNA
XM_012175416.2	PKN2	mRNA	XR_001041211.2	lncRNA
XM_012175416.2	PKN2	mRNA	XR_001041211.2	lncRNA
XM_012175416.2	PKN2	mRNA	XR_001041211.2	lncRNA
XM_012175416.2	PKN2	mRNA	XR_001041211.2	lncRNA
XM_012175416.2	PKN2	mRNA	XR_001041211.2	lncRNA
XM_012175416.2	PKN2	mRNA	XR_001041211.2	lncRNA
XM_012175416.2	PKN2	mRNA	XR_001041211.2	lncRNA
XM_012175416.2	PKN2	mRNA	XR_001041211.2	lncRNA
XM_012175416.2	PKN2	mRNA	XR_001041211.2	lncRNA
XM_012175416.2	PKN2	mRNA	XR_001041211.2	lncRNA
XM_012175416.2	PKN2	mRNA	XR_001041211.2	lncRNA
XM_015096356.1	SEC24D	mRNA	XR_001436102.1	lncRNA
XM_015096356.1	SEC24D	mRNA	XR_001436102.1	lncRNA
XM_015096356.1	SEC24D	mRNA	XR_001436102.1	lncRNA
XM_015096356.1	SEC24D	mRNA	XR_001436102.1	lncRNA
XM_015096356.1	SEC24D	mRNA	XR_001436102.1	lncRNA
XM_015096356.1	SEC24D	mRNA	XR_001436102.1	lncRNA
XM_015096356.1	SEC24D	mRNA	XR_001436102.1	lncRNA
XM_015096356.1	SEC24D	mRNA	XR_001436102.1	lncRNA
XM_015096356.1	SEC24D	mRNA	XR_001436102.1	lncRNA
XM_015096356.1	SEC24D	mRNA	XR_001436102.1	lncRNA
XM_015096356.1	SEC24D	mRNA	XR_001436102.1	lncRNA
XM_012100690.1	KLC4	mRNA	XR_001436102.1	lncRNA
XM_012100690.1	KLC4	mRNA	XR_001436102.1	lncRNA
XM_012100690.1	KLC4	mRNA	XR_001436102.1	lncRNA
XM_012100690.1	KLC4	mRNA	XR_001436102.1	lncRNA
XM_012100690.1	KLC4	mRNA	XR_001436102.1	lncRNA
XM_012100690.1	KLC4	mRNA	XR_001436102.1	lncRNA
XM_012100690.1	KLC4	mRNA	XR_001436102.1	lncRNA
XM_012100690.1	KLC4	mRNA	XR_001436102.1	lncRNA
XM_012100690.1	KLC4	mRNA	XR_001436102.1	lncRNA
XM_012100690.1	KLC4	mRNA	XR_001436102.1	lncRNA
XM_012100690.1	KLC4	mRNA	XR_001436102.1	lncRNA
XM_015099550.1	PARD3	mRNA	XR_001436102.1	lncRNA
XM_015099550.1	PARD3	mRNA	XR_001436102.1	lncRNA
XM_015099550.1	PARD3	mRNA	XR_001436102.1	lncRNA
XM_015099550.1	PARD3	mRNA	XR_001436102.1	lncRNA
XM_015099550.1	PARD3	mRNA	XR_001436102.1	lncRNA
XM_015099550.1	PARD3	mRNA	XR_001436102.1	lncRNA
XM_015099550.1	PARD3	mRNA	XR_001436102.1	lncRNA
XM_015099550.1	PARD3	mRNA	XR_001436102.1	lncRNA

XM_015099550.1	PARD3	mRNA	XR_001436102.1	lncRNA
XM_015099550.1	PARD3	mRNA	XR_001436102.1	lncRNA
XM_015099550.1	PARD3	mRNA	XR_001436102.1	lncRNA
XM_012146543.1	ATG9A	mRNA	XR_001436102.1	lncRNA
XM_012146543.1	ATG9A	mRNA	XR_001436102.1	lncRNA
XM_012146543.1	ATG9A	mRNA	XR_001436102.1	lncRNA
XM_012146543.1	ATG9A	mRNA	XR_001436102.1	lncRNA
XM_012146543.1	ATG9A	mRNA	XR_001436102.1	lncRNA
XM_012146543.1	ATG9A	mRNA	XR_001436102.1	lncRNA
XM_012146543.1	ATG9A	mRNA	XR_001436102.1	lncRNA
XM_012146543.1	ATG9A	mRNA	XR_001436102.1	lncRNA
XM_012146543.1	ATG9A	mRNA	XR_001436102.1	lncRNA
XM_012146543.1	ATG9A	mRNA	XR_001436102.1	lncRNA
XM_012146543.1	ATG9A	mRNA	XR_001436102.1	lncRNA
XM_012105690.2	WDR17	mRNA	XR_001041211.2	lncRNA
XM_012105690.2	WDR17	mRNA	XR_001041211.2	lncRNA
XM_012105690.2	WDR17	mRNA	XR_001041211.2	lncRNA
XM_012105690.2	WDR17	mRNA	XR_001041211.2	lncRNA
XM_012105690.2	WDR17	mRNA	XR_001041211.2	lncRNA
XM_012105690.2	WDR17	mRNA	XR_001041211.2	lncRNA
XM_012105690.2	WDR17	mRNA	XR_001041211.2	lncRNA
XM_012105690.2	WDR17	mRNA	XR_001041211.2	lncRNA
XM_012105690.2	WDR17	mRNA	XR_001041211.2	lncRNA
XM_012105690.2	WDR17	mRNA	XR_001041211.2	lncRNA
XM_012105690.2	WDR17	mRNA	XR_001041211.2	lncRNA
XM_012105690.2	WDR17	mRNA	XR_001041211.2	lncRNA
XM_015097018.1	ZNF280D	mRNA	XR_001436817.1	lncRNA
XM_015097018.1	ZNF280D	mRNA	XR_001436817.1	lncRNA
XM_015097018.1	ZNF280D	mRNA	XR_001436817.1	lncRNA
XM_015097018.1	ZNF280D	mRNA	XR_001436817.1	lncRNA
XM_015097018.1	ZNF280D	mRNA	XR_001436817.1	lncRNA
XM_015097018.1	ZNF280D	mRNA	XR_001436817.1	lncRNA
XM_015097018.1	ZNF280D	mRNA	XR_001436817.1	lncRNA
XM_015097018.1	ZNF280D	mRNA	XR_001436817.1	lncRNA
XM_015097018.1	ZNF280D	mRNA	XR_001436817.1	lncRNA
XM_015097018.1	ZNF280D	mRNA	XR_001436817.1	lncRNA
XM_015097018.1	ZNF280D	mRNA	XR_001436817.1	lncRNA
XM_015105040.1	THOC2	mRNA	XR_001434203.1	lncRNA
XM_015105040.1	THOC2	mRNA	XR_001434203.1	lncRNA
XM_015105040.1	THOC2	mRNA	XR_001434203.1	lncRNA
XM_015105040.1	THOC2	mRNA	XR_001434203.1	lncRNA
XM_015105040.1	THOC2	mRNA	XR_001434203.1	lncRNA
XM_015105040.1	THOC2	mRNA	XR_001434203.1	lncRNA
XM_015105040.1	THOC2	mRNA	XR_001434203.1	lncRNA
XM_015105040.1	THOC2	mRNA	XR_001434203.1	lncRNA
XM_015105040.1	THOC2	mRNA	XR_001434203.1	lncRNA
XM_015105040.1	THOC2	mRNA	XR_001434203.1	lncRNA
XM_015105040.1	THOC2	mRNA	XR_001434203.1	lncRNA
XM_012140422.2	CLASP1	mRNA	XR_001434203.1	lncRNA
XM_012140422.2	CLASP1	mRNA	XR_001434203.1	lncRNA
XM_012140422.2	CLASP1	mRNA	XR_001434203.1	lncRNA
XM_012140422.2	CLASP1	mRNA	XR_001434203.1	lncRNA
XM_012140422.2	CLASP1	mRNA	XR_001434203.1	lncRNA
XM_012140422.2	CLASP1	mRNA	XR_001434203.1	lncRNA

XM_012140422.2	CLASP1	mRNA	XR_001434203.1	lncRNA
XM_012140422.2	CLASP1	mRNA	XR_001434203.1	lncRNA
XM_012140422.2	CLASP1	mRNA	XR_001434203.1	lncRNA
XM_012140422.2	CLASP1	mRNA	XR_001434203.1	lncRNA
XM_012140422.2	CLASP1	mRNA	XR_001434203.1	lncRNA
XM_012095858.2	ZNF214	mRNA	XR_001436817.1	lncRNA
XM_012095858.2	ZNF214	mRNA	XR_001436817.1	lncRNA
XM_012095858.2	ZNF214	mRNA	XR_001436817.1	lncRNA
XM_012095858.2	ZNF214	mRNA	XR_001436817.1	lncRNA
XM_012095858.2	ZNF214	mRNA	XR_001436817.1	lncRNA
XM_012095858.2	ZNF214	mRNA	XR_001436817.1	lncRNA
XM_012095858.2	ZNF214	mRNA	XR_001436817.1	lncRNA
XM_012095858.2	ZNF214	mRNA	XR_001436817.1	lncRNA
XM_012095858.2	ZNF214	mRNA	XR_001436817.1	lncRNA
XM_012095858.2	ZNF214	mRNA	XR_001436817.1	lncRNA
XM_012095858.2	ZNF214	mRNA	XR_001436817.1	lncRNA
XM_004008647.3	RAPGEF6	mRNA	XR_001434203.1	lncRNA
XM_004008647.3	RAPGEF6	mRNA	XR_001434203.1	lncRNA
XM_004008647.3	RAPGEF6	mRNA	XR_001434203.1	lncRNA
XM_004008647.3	RAPGEF6	mRNA	XR_001434203.1	lncRNA
XM_004008647.3	RAPGEF6	mRNA	XR_001434203.1	lncRNA
XM_004008647.3	RAPGEF6	mRNA	XR_001434203.1	lncRNA
XM_004008647.3	RAPGEF6	mRNA	XR_001434203.1	lncRNA
XM_004008647.3	RAPGEF6	mRNA	XR_001434203.1	lncRNA
XM_004008647.3	RAPGEF6	mRNA	XR_001434203.1	lncRNA
XM_004008647.3	RAPGEF6	mRNA	XR_001434203.1	lncRNA
XM_004008647.3	RAPGEF6	mRNA	XR_001434203.1	lncRNA
XM_015097018.1	ZNF280D	mRNA	XR_001434203.1	lncRNA
XM_015097018.1	ZNF280D	mRNA	XR_001434203.1	lncRNA
XM_015097018.1	ZNF280D	mRNA	XR_001434203.1	lncRNA
XM_015097018.1	ZNF280D	mRNA	XR_001434203.1	lncRNA
XM_015097018.1	ZNF280D	mRNA	XR_001434203.1	lncRNA
XM_015097018.1	ZNF280D	mRNA	XR_001434203.1	lncRNA
XM_015097018.1	ZNF280D	mRNA	XR_001434203.1	lncRNA
XM_015097018.1	ZNF280D	mRNA	XR_001434203.1	lncRNA
XM_015097018.1	ZNF280D	mRNA	XR_001434203.1	lncRNA
XM_015097018.1	ZNF280D	mRNA	XR_001434203.1	lncRNA
XM_015097018.1	ZNF280D	mRNA	XR_001434203.1	lncRNA
XM_004008647.3	RAPGEF6	mRNA	XR_001436817.1	lncRNA
XM_004008647.3	RAPGEF6	mRNA	XR_001436817.1	lncRNA
XM_004008647.3	RAPGEF6	mRNA	XR_001436817.1	lncRNA
XM_004008647.3	RAPGEF6	mRNA	XR_001436817.1	lncRNA
XM_004008647.3	RAPGEF6	mRNA	XR_001436817.1	lncRNA
XM_004008647.3	RAPGEF6	mRNA	XR_001436817.1	lncRNA
XM_004008647.3	RAPGEF6	mRNA	XR_001436817.1	lncRNA
XM_004008647.3	RAPGEF6	mRNA	XR_001436817.1	lncRNA
XM_004008647.3	RAPGEF6	mRNA	XR_001436817.1	lncRNA
XM_004008647.3	RAPGEF6	mRNA	XR_001436817.1	lncRNA
XM_004008647.3	RAPGEF6	mRNA	XR_001436817.1	lncRNA
XM_012174768.2	RAB5B	mRNA	XR_001436817.1	lncRNA
XM_012174768.2	RAB5B	mRNA	XR_001436817.1	lncRNA
XM_012174768.2	RAB5B	mRNA	XR_001436817.1	lncRNA
XM_012174768.2	RAB5B	mRNA	XR_001436817.1	lncRNA
XM_012174768.2	RAB5B	mRNA	XR_001436817.1	lncRNA

XM_012174768.2	RAB5B	mRNA	XR_001436817.1	lncRNA
XM_012174768.2	RAB5B	mRNA	XR_001436817.1	lncRNA
XM_012174768.2	RAB5B	mRNA	XR_001436817.1	lncRNA
XM_012174768.2	RAB5B	mRNA	XR_001436817.1	lncRNA
XM_012174768.2	RAB5B	mRNA	XR_001436817.1	lncRNA
XM_012174768.2	RAB5B	mRNA	XR_001436817.1	lncRNA
XM_012174160.2	FUBP1	mRNA	XR_001436817.1	lncRNA
XM_012174160.2	FUBP1	mRNA	XR_001436817.1	lncRNA
XM_012174160.2	FUBP1	mRNA	XR_001436817.1	lncRNA
XM_012174160.2	FUBP1	mRNA	XR_001436817.1	lncRNA
XM_012174160.2	FUBP1	mRNA	XR_001436817.1	lncRNA
XM_012174160.2	FUBP1	mRNA	XR_001436817.1	lncRNA
XM_012174160.2	FUBP1	mRNA	XR_001436817.1	lncRNA
XM_012174160.2	FUBP1	mRNA	XR_001436817.1	lncRNA
XM_012174160.2	FUBP1	mRNA	XR_001436817.1	lncRNA
XM_012174160.2	FUBP1	mRNA	XR_001436817.1	lncRNA
XM_012174160.2	FUBP1	mRNA	XR_001436817.1	lncRNA
XM_012176217.2	EVI5	mRNA	XR_001434203.1	lncRNA
XM_012176217.2	EVI5	mRNA	XR_001434203.1	lncRNA
XM_012176217.2	EVI5	mRNA	XR_001434203.1	lncRNA
XM_012176217.2	EVI5	mRNA	XR_001434203.1	lncRNA
XM_012176217.2	EVI5	mRNA	XR_001434203.1	lncRNA
XM_012176217.2	EVI5	mRNA	XR_001434203.1	lncRNA
XM_012176217.2	EVI5	mRNA	XR_001434203.1	lncRNA
XM_012176217.2	EVI5	mRNA	XR_001434203.1	lncRNA
XM_012176217.2	EVI5	mRNA	XR_001434203.1	lncRNA
XM_012176217.2	EVI5	mRNA	XR_001434203.1	lncRNA
XM_012176217.2	EVI5	mRNA	XR_001434203.1	lncRNA
XM_012183305.1	FAM120B	mRNA	XR_001436817.1	lncRNA
XM_012183305.1	FAM120B	mRNA	XR_001436817.1	lncRNA
XM_012183305.1	FAM120B	mRNA	XR_001436817.1	lncRNA
XM_012183305.1	FAM120B	mRNA	XR_001436817.1	lncRNA
XM_012183305.1	FAM120B	mRNA	XR_001436817.1	lncRNA
XM_012183305.1	FAM120B	mRNA	XR_001436817.1	lncRNA
XM_012183305.1	FAM120B	mRNA	XR_001436817.1	lncRNA
XM_012183305.1	FAM120B	mRNA	XR_001436817.1	lncRNA
XM_012183305.1	FAM120B	mRNA	XR_001436817.1	lncRNA
XM_012183305.1	FAM120B	mRNA	XR_001436817.1	lncRNA
XM_012183305.1	FAM120B	mRNA	XR_001436817.1	lncRNA
XM_012098903.1	PPIP5K1	mRNA	XR_001434203.1	lncRNA
XM_012098903.1	PPIP5K1	mRNA	XR_001434203.1	lncRNA
XM_012098903.1	PPIP5K1	mRNA	XR_001434203.1	lncRNA
XM_012098903.1	PPIP5K1	mRNA	XR_001434203.1	lncRNA
XM_012098903.1	PPIP5K1	mRNA	XR_001434203.1	lncRNA
XM_012098903.1	PPIP5K1	mRNA	XR_001434203.1	lncRNA
XM_012098903.1	PPIP5K1	mRNA	XR_001434203.1	lncRNA
XM_012098903.1	PPIP5K1	mRNA	XR_001434203.1	lncRNA
XM_012098903.1	PPIP5K1	mRNA	XR_001434203.1	lncRNA
XM_012098903.1	PPIP5K1	mRNA	XR_001434203.1	lncRNA
XM_012098903.1	PPIP5K1	mRNA	XR_001434203.1	lncRNA
XM_012174768.2	RAB5B	mRNA	XR_001434203.1	lncRNA
XM_012174768.2	RAB5B	mRNA	XR_001434203.1	lncRNA
XM_012174768.2	RAB5B	mRNA	XR_001434203.1	lncRNA
XM_012174768.2	RAB5B	mRNA	XR_001434203.1	lncRNA

XM_012174768.2	RAB5B	mRNA	XR_001434203.1	lncRNA
XM_012174768.2	RAB5B	mRNA	XR_001434203.1	lncRNA
XM_012174768.2	RAB5B	mRNA	XR_001434203.1	lncRNA
XM_012174768.2	RAB5B	mRNA	XR_001434203.1	lncRNA
XM_012174768.2	RAB5B	mRNA	XR_001434203.1	lncRNA
XM_012174768.2	RAB5B	mRNA	XR_001434203.1	lncRNA
XM_012174768.2	RAB5B	mRNA	XR_001434203.1	lncRNA
XM_012176217.2	EVI5	mRNA	XR_001436817.1	lncRNA
XM_012176217.2	EVI5	mRNA	XR_001436817.1	lncRNA
XM_012176217.2	EVI5	mRNA	XR_001436817.1	lncRNA
XM_012176217.2	EVI5	mRNA	XR_001436817.1	lncRNA
XM_012176217.2	EVI5	mRNA	XR_001436817.1	lncRNA
XM_012176217.2	EVI5	mRNA	XR_001436817.1	lncRNA
XM_012176217.2	EVI5	mRNA	XR_001436817.1	lncRNA
XM_012176217.2	EVI5	mRNA	XR_001436817.1	lncRNA
XM_012176217.2	EVI5	mRNA	XR_001436817.1	lncRNA
XM_012176217.2	EVI5	mRNA	XR_001436817.1	lncRNA
XM_012176217.2	EVI5	mRNA	XR_001436817.1	lncRNA
XM_015103448.1	TACC2	mRNA	XR_001024290.1	lncRNA
XM_015103448.1	TACC2	mRNA	XR_001024290.1	lncRNA
XM_015103448.1	TACC2	mRNA	XR_001024290.1	lncRNA
XM_015103448.1	TACC2	mRNA	XR_001024290.1	lncRNA
XM_015103448.1	TACC2	mRNA	XR_001024290.1	lncRNA
XM_015103448.1	TACC2	mRNA	XR_001024290.1	lncRNA
XM_015103448.1	TACC2	mRNA	XR_001024290.1	lncRNA
XM_015103448.1	TACC2	mRNA	XR_001024290.1	lncRNA
XM_015103448.1	TACC2	mRNA	XR_001024290.1	lncRNA
XM_015103448.1	TACC2	mRNA	XR_001024290.1	lncRNA
XM_015103448.1	TACC2	mRNA	XR_001024290.1	lncRNA
XM_015091997.1	LOC101121639	mRNA	XR_001042381.2	lncRNA
XM_015091997.1	LOC101121639	mRNA	XR_001042381.2	lncRNA
XM_015091997.1	LOC101121639	mRNA	XR_001042381.2	lncRNA
XM_015091997.1	LOC101121639	mRNA	XR_001042381.2	lncRNA
XM_015091997.1	LOC101121639	mRNA	XR_001042381.2	lncRNA
XM_015091997.1	LOC101121639	mRNA	XR_001042381.2	lncRNA
XM_015091997.1	LOC101121639	mRNA	XR_001042381.2	lncRNA
XM_015091997.1	LOC101121639	mRNA	XR_001042381.2	lncRNA
XM_015091997.1	LOC101121639	mRNA	XR_001042381.2	lncRNA
XM_015091997.1	LOC101121639	mRNA	XR_001042381.2	lncRNA
XM_015091997.1	LOC101121639	mRNA	XR_001042381.2	lncRNA
XM_015091997.1	LOC101121639	mRNA	XR_001042381.2	lncRNA
XM_012177920.2	KANK2	mRNA	XR_001436532.1	lncRNA
XM_012177920.2	KANK2	mRNA	XR_001436532.1	lncRNA
XM_012177920.2	KANK2	mRNA	XR_001436532.1	lncRNA
XM_012177920.2	KANK2	mRNA	XR_001436532.1	lncRNA
XM_012177920.2	KANK2	mRNA	XR_001436532.1	lncRNA
XM_012177920.2	KANK2	mRNA	XR_001436532.1	lncRNA
XM_012177920.2	KANK2	mRNA	XR_001436532.1	lncRNA
XM_012177920.2	KANK2	mRNA	XR_001436532.1	lncRNA
XM_012177920.2	KANK2	mRNA	XR_001436532.1	lncRNA
XM_012177920.2	KANK2	mRNA	XR_001436532.1	lncRNA
XM_012177920.2	KANK2	mRNA	XR_001436532.1	lncRNA
XM_012177920.2	KANK2	mRNA	XR_001436532.1	lncRNA
XM_015102318.1	SETD5	mRNA	XR_001434202.1	lncRNA

XM_015102318.1	SETD5	mRNA	XR_001434202.1	lncRNA
XM_015102318.1	SETD5	mRNA	XR_001434202.1	lncRNA
XM_015102318.1	SETD5	mRNA	XR_001434202.1	lncRNA
XM_015102318.1	SETD5	mRNA	XR_001434202.1	lncRNA
XM_015102318.1	SETD5	mRNA	XR_001434202.1	lncRNA
XM_015102318.1	SETD5	mRNA	XR_001434202.1	lncRNA
XM_015102318.1	SETD5	mRNA	XR_001434202.1	lncRNA
XM_015102318.1	SETD5	mRNA	XR_001434202.1	lncRNA
XM_015102318.1	SETD5	mRNA	XR_001434202.1	lncRNA
XM_015102318.1	SETD5	mRNA	XR_001434202.1	lncRNA
XM_012102845.2	GBF1	mRNA	XR_001034847.2	lncRNA
XM_012102845.2	GBF1	mRNA	XR_001034847.2	lncRNA
XM_012102845.2	GBF1	mRNA	XR_001034847.2	lncRNA
XM_012102845.2	GBF1	mRNA	XR_001034847.2	lncRNA
XM_012102845.2	GBF1	mRNA	XR_001034847.2	lncRNA
XM_012102845.2	GBF1	mRNA	XR_001034847.2	lncRNA
XM_012102845.2	GBF1	mRNA	XR_001034847.2	lncRNA
XM_012102845.2	GBF1	mRNA	XR_001034847.2	lncRNA
XM_012102845.2	GBF1	mRNA	XR_001034847.2	lncRNA
XM_012102845.2	GBF1	mRNA	XR_001034847.2	lncRNA
XM_012102845.2	GBF1	mRNA	XR_001034847.2	lncRNA
XM_012102845.2	GBF1	mRNA	XR_001434202.1	lncRNA
XM_012102845.2	GBF1	mRNA	XR_001434202.1	lncRNA
XM_012102845.2	GBF1	mRNA	XR_001434202.1	lncRNA
XM_012102845.2	GBF1	mRNA	XR_001434202.1	lncRNA
XM_012102845.2	GBF1	mRNA	XR_001434202.1	lncRNA
XM_012102845.2	GBF1	mRNA	XR_001434202.1	lncRNA
XM_012102845.2	GBF1	mRNA	XR_001434202.1	lncRNA
XM_012102845.2	GBF1	mRNA	XR_001434202.1	lncRNA
XM_012102845.2	GBF1	mRNA	XR_001434202.1	lncRNA
XM_012102845.2	GBF1	mRNA	XR_001434202.1	lncRNA
XM_012102845.2	GBF1	mRNA	XR_001434202.1	lncRNA
XM_015102318.1	SETD5	mRNA	XR_001034847.2	lncRNA
XM_015102318.1	SETD5	mRNA	XR_001034847.2	lncRNA
XM_015102318.1	SETD5	mRNA	XR_001034847.2	lncRNA
XM_015102318.1	SETD5	mRNA	XR_001034847.2	lncRNA
XM_015102318.1	SETD5	mRNA	XR_001034847.2	lncRNA
XM_015102318.1	SETD5	mRNA	XR_001034847.2	lncRNA
XM_015102318.1	SETD5	mRNA	XR_001034847.2	lncRNA
XM_015102318.1	SETD5	mRNA	XR_001034847.2	lncRNA
XM_015102318.1	SETD5	mRNA	XR_001034847.2	lncRNA
XM_015102318.1	SETD5	mRNA	XR_001034847.2	lncRNA
XM_015102318.1	SETD5	mRNA	XR_001034847.2	lncRNA
XM_015098529.1	NF1	mRNA	XR_001021689.2	lncRNA
XM_015098529.1	NF1	mRNA	XR_001021689.2	lncRNA
XM_015098529.1	NF1	mRNA	XR_001021689.2	lncRNA
XM_015098529.1	NF1	mRNA	XR_001021689.2	lncRNA
XM_015098529.1	NF1	mRNA	XR_001021689.2	lncRNA
XM_015098529.1	NF1	mRNA	XR_001021689.2	lncRNA
XM_015098529.1	NF1	mRNA	XR_001021689.2	lncRNA
XM_015098529.1	NF1	mRNA	XR_001021689.2	lncRNA
XM_015098529.1	NF1	mRNA	XR_001021689.2	lncRNA
XM_015098529.1	NF1	mRNA	XR_001021689.2	lncRNA
XM_015098529.1	NF1	mRNA	XR_001021689.2	lncRNA

XM_012151052.2	WASF2	mRNA	XR_001042381.2	lncRNA
XM_012151052.2	WASF2	mRNA	XR_001042381.2	lncRNA
XM_012151052.2	WASF2	mRNA	XR_001042381.2	lncRNA
XM_012151052.2	WASF2	mRNA	XR_001042381.2	lncRNA
XM_012151052.2	WASF2	mRNA	XR_001042381.2	lncRNA
XM_012151052.2	WASF2	mRNA	XR_001042381.2	lncRNA
XM_012151052.2	WASF2	mRNA	XR_001042381.2	lncRNA
XM_012151052.2	WASF2	mRNA	XR_001042381.2	lncRNA
XM_012151052.2	WASF2	mRNA	XR_001042381.2	lncRNA
XM_012151052.2	WASF2	mRNA	XR_001042381.2	lncRNA
XM_012151052.2	WASF2	mRNA	XR_001042381.2	lncRNA
XM_012106817.2	JADE3	mRNA	XR_001042381.2	lncRNA
XM_012106817.2	JADE3	mRNA	XR_001042381.2	lncRNA
XM_012106817.2	JADE3	mRNA	XR_001042381.2	lncRNA
XM_012106817.2	JADE3	mRNA	XR_001042381.2	lncRNA
XM_012106817.2	JADE3	mRNA	XR_001042381.2	lncRNA
XM_012106817.2	JADE3	mRNA	XR_001042381.2	lncRNA
XM_012106817.2	JADE3	mRNA	XR_001042381.2	lncRNA
XM_012106817.2	JADE3	mRNA	XR_001042381.2	lncRNA
XM_012106817.2	JADE3	mRNA	XR_001042381.2	lncRNA
XM_012106817.2	JADE3	mRNA	XR_001042381.2	lncRNA
XM_012106817.2	JADE3	mRNA	XR_001042381.2	lncRNA
XM_015091902.1	SCMH1	mRNA	XR_001436532.1	lncRNA
XM_015091902.1	SCMH1	mRNA	XR_001436532.1	lncRNA
XM_015091902.1	SCMH1	mRNA	XR_001436532.1	lncRNA
XM_015091902.1	SCMH1	mRNA	XR_001436532.1	lncRNA
XM_015091902.1	SCMH1	mRNA	XR_001436532.1	lncRNA
XM_015091902.1	SCMH1	mRNA	XR_001436532.1	lncRNA
XM_015091902.1	SCMH1	mRNA	XR_001436532.1	lncRNA
XM_015091902.1	SCMH1	mRNA	XR_001436532.1	lncRNA
XM_015091902.1	SCMH1	mRNA	XR_001436532.1	lncRNA
XM_015091902.1	SCMH1	mRNA	XR_001436532.1	lncRNA
XM_015091902.1	SCMH1	mRNA	XR_001436532.1	lncRNA
XM_012105318.2	USP54	mRNA	XR_001436532.1	lncRNA
XM_012105318.2	USP54	mRNA	XR_001436532.1	lncRNA
XM_012105318.2	USP54	mRNA	XR_001436532.1	lncRNA
XM_012105318.2	USP54	mRNA	XR_001436532.1	lncRNA
XM_012105318.2	USP54	mRNA	XR_001436532.1	lncRNA
XM_012105318.2	USP54	mRNA	XR_001436532.1	lncRNA
XM_012105318.2	USP54	mRNA	XR_001436532.1	lncRNA
XM_012105318.2	USP54	mRNA	XR_001436532.1	lncRNA
XM_012105318.2	USP54	mRNA	XR_001436532.1	lncRNA
XM_012105318.2	USP54	mRNA	XR_001436532.1	lncRNA
XM_012105318.2	USP54	mRNA	XR_001436532.1	lncRNA
XM_012097610.2	ZNF605	mRNA	XR_001042381.2	lncRNA
XM_012097610.2	ZNF605	mRNA	XR_001042381.2	lncRNA
XM_012097610.2	ZNF605	mRNA	XR_001042381.2	lncRNA
XM_012097610.2	ZNF605	mRNA	XR_001042381.2	lncRNA
XM_012097610.2	ZNF605	mRNA	XR_001042381.2	lncRNA
XM_012097610.2	ZNF605	mRNA	XR_001042381.2	lncRNA
XM_012097610.2	ZNF605	mRNA	XR_001042381.2	lncRNA
XM_012097610.2	ZNF605	mRNA	XR_001042381.2	lncRNA
XM_012097610.2	ZNF605	mRNA	XR_001042381.2	lncRNA
XM_012097610.2	ZNF605	mRNA	XR_001042381.2	lncRNA

XM_012097610.2	ZNF605	mRNA	XR_001042381.2	lncRNA
XM_012187378.2	CAMTA1	mRNA	XR_001044126.1	lncRNA
XM_012187378.2	CAMTA1	mRNA	XR_001044126.1	lncRNA
XM_012187378.2	CAMTA1	mRNA	XR_001044126.1	lncRNA
XM_012187378.2	CAMTA1	mRNA	XR_001044126.1	lncRNA
XM_012187378.2	CAMTA1	mRNA	XR_001044126.1	lncRNA
XM_012187378.2	CAMTA1	mRNA	XR_001044126.1	lncRNA
XM_012187378.2	CAMTA1	mRNA	XR_001044126.1	lncRNA
XM_012187378.2	CAMTA1	mRNA	XR_001044126.1	lncRNA
XM_012187378.2	CAMTA1	mRNA	XR_001044126.1	lncRNA
XM_012187378.2	CAMTA1	mRNA	XR_001044126.1	lncRNA
XM_012187378.2	CAMTA1	mRNA	XR_001044126.1	lncRNA
XM_012143523.2	INO80D	mRNA	XR_001024290.1	lncRNA
XM_012143523.2	INO80D	mRNA	XR_001024290.1	lncRNA
XM_012143523.2	INO80D	mRNA	XR_001024290.1	lncRNA
XM_012143523.2	INO80D	mRNA	XR_001024290.1	lncRNA
XM_012143523.2	INO80D	mRNA	XR_001024290.1	lncRNA
XM_012143523.2	INO80D	mRNA	XR_001024290.1	lncRNA
XM_012143523.2	INO80D	mRNA	XR_001024290.1	lncRNA
XM_012143523.2	INO80D	mRNA	XR_001024290.1	lncRNA
XM_012143523.2	INO80D	mRNA	XR_001024290.1	lncRNA
XM_012143523.2	INO80D	mRNA	XR_001024290.1	lncRNA
XM_012143523.2	INO80D	mRNA	XR_001024290.1	lncRNA
XM_015103475.1	PLEKHA1	mRNA	XR_001434761.1	lncRNA
XM_015103475.1	PLEKHA1	mRNA	XR_001434761.1	lncRNA
XM_015103475.1	PLEKHA1	mRNA	XR_001434761.1	lncRNA
XM_015103475.1	PLEKHA1	mRNA	XR_001434761.1	lncRNA
XM_015103475.1	PLEKHA1	mRNA	XR_001434761.1	lncRNA
XM_015103475.1	PLEKHA1	mRNA	XR_001434761.1	lncRNA
XM_015103475.1	PLEKHA1	mRNA	XR_001434761.1	lncRNA
XM_015103475.1	PLEKHA1	mRNA	XR_001434761.1	lncRNA
XM_015103475.1	PLEKHA1	mRNA	XR_001434761.1	lncRNA
XM_015103475.1	PLEKHA1	mRNA	XR_001434761.1	lncRNA
XM_015103475.1	PLEKHA1	mRNA	XR_001434761.1	lncRNA
XM_015091902.1	SCMH1	mRNA	XR_001041211.2	lncRNA
XM_015091902.1	SCMH1	mRNA	XR_001041211.2	lncRNA
XM_015091902.1	SCMH1	mRNA	XR_001041211.2	lncRNA
XM_015091902.1	SCMH1	mRNA	XR_001041211.2	lncRNA
XM_015091902.1	SCMH1	mRNA	XR_001041211.2	lncRNA
XM_015091902.1	SCMH1	mRNA	XR_001041211.2	lncRNA
XM_015091902.1	SCMH1	mRNA	XR_001041211.2	lncRNA
XM_015091902.1	SCMH1	mRNA	XR_001041211.2	lncRNA
XM_015091902.1	SCMH1	mRNA	XR_001041211.2	lncRNA
XM_015091902.1	SCMH1	mRNA	XR_001041211.2	lncRNA
XM_015091902.1	SCMH1	mRNA	XR_001041211.2	lncRNA
XM_012105318.2	USP54	mRNA	XR_001041211.2	lncRNA
XM_012105318.2	USP54	mRNA	XR_001041211.2	lncRNA
XM_012105318.2	USP54	mRNA	XR_001041211.2	lncRNA
XM_012105318.2	USP54	mRNA	XR_001041211.2	lncRNA
XM_012105318.2	USP54	mRNA	XR_001041211.2	lncRNA
XM_012105318.2	USP54	mRNA	XR_001041211.2	lncRNA
XM_012105318.2	USP54	mRNA	XR_001041211.2	lncRNA
XM_012105318.2	USP54	mRNA	XR_001041211.2	lncRNA
XM_012105318.2	USP54	mRNA	XR_001041211.2	lncRNA

XM_012105318.2	USP54	mRNA	XR_001041211.2	lncRNA
XM_012105318.2	USP54	mRNA	XR_001041211.2	lncRNA
XM_012176510.2	FNBP1L	mRNA	XR_001025491.1	lncRNA
XM_012176510.2	FNBP1L	mRNA	XR_001025491.1	lncRNA
XM_012176510.2	FNBP1L	mRNA	XR_001025491.1	lncRNA
XM_012176510.2	FNBP1L	mRNA	XR_001025491.1	lncRNA
XM_012176510.2	FNBP1L	mRNA	XR_001025491.1	lncRNA
XM_012176510.2	FNBP1L	mRNA	XR_001025491.1	lncRNA
XM_012176510.2	FNBP1L	mRNA	XR_001025491.1	lncRNA
XM_012176510.2	FNBP1L	mRNA	XR_001025491.1	lncRNA
XM_012176510.2	FNBP1L	mRNA	XR_001025491.1	lncRNA
XM_012176510.2	FNBP1L	mRNA	XR_001025491.1	lncRNA
XM_015098529.1	NF1	mRNA	XR_001434202.1	lncRNA
XM_015098529.1	NF1	mRNA	XR_001434202.1	lncRNA
XM_015098529.1	NF1	mRNA	XR_001434202.1	lncRNA
XM_015098529.1	NF1	mRNA	XR_001434202.1	lncRNA
XM_015098529.1	NF1	mRNA	XR_001434202.1	lncRNA
XM_015098529.1	NF1	mRNA	XR_001434202.1	lncRNA
XM_015098529.1	NF1	mRNA	XR_001434202.1	lncRNA
XM_015098529.1	NF1	mRNA	XR_001434202.1	lncRNA
XM_015098529.1	NF1	mRNA	XR_001434202.1	lncRNA
XM_015098529.1	NF1	mRNA	XR_001434202.1	lncRNA
XM_015098529.1	NF1	mRNA	XR_001434202.1	lncRNA
XM_015098611.1	DLG4	mRNA	XR_001434202.1	lncRNA
XM_015098611.1	DLG4	mRNA	XR_001434202.1	lncRNA
XM_015098611.1	DLG4	mRNA	XR_001434202.1	lncRNA
XM_015098611.1	DLG4	mRNA	XR_001434202.1	lncRNA
XM_015098611.1	DLG4	mRNA	XR_001434202.1	lncRNA
XM_015098611.1	DLG4	mRNA	XR_001434202.1	lncRNA
XM_015098611.1	DLG4	mRNA	XR_001434202.1	lncRNA
XM_015098611.1	DLG4	mRNA	XR_001434202.1	lncRNA
XM_015098611.1	DLG4	mRNA	XR_001434202.1	lncRNA
XM_015098611.1	DLG4	mRNA	XR_001434202.1	lncRNA
XM_015098611.1	DLG4	mRNA	XR_001434202.1	lncRNA
XM_015098611.1	DLG4	mRNA	XR_001034847.2	lncRNA
XM_015098611.1	DLG4	mRNA	XR_001034847.2	lncRNA
XM_015098611.1	DLG4	mRNA	XR_001034847.2	lncRNA
XM_015098611.1	DLG4	mRNA	XR_001034847.2	lncRNA
XM_015098611.1	DLG4	mRNA	XR_001034847.2	lncRNA
XM_015098611.1	DLG4	mRNA	XR_001034847.2	lncRNA
XM_015098611.1	DLG4	mRNA	XR_001034847.2	lncRNA
XM_015098611.1	DLG4	mRNA	XR_001034847.2	lncRNA
XM_015098611.1	DLG4	mRNA	XR_001034847.2	lncRNA
XM_015098611.1	DLG4	mRNA	XR_001034847.2	lncRNA
XM_015098611.1	DLG4	mRNA	XR_001034847.2	lncRNA
XM_015098529.1	NF1	mRNA	XR_001034847.2	lncRNA
XM_015098529.1	NF1	mRNA	XR_001034847.2	lncRNA
XM_015098529.1	NF1	mRNA	XR_001034847.2	lncRNA
XM_015098529.1	NF1	mRNA	XR_001034847.2	lncRNA
XM_015098529.1	NF1	mRNA	XR_001034847.2	lncRNA
XM_015098529.1	NF1	mRNA	XR_001034847.2	lncRNA
XM_015098529.1	NF1	mRNA	XR_001034847.2	lncRNA
XM_015098529.1	NF1	mRNA	XR_001034847.2	lncRNA
XM_015098529.1	NF1	mRNA	XR_001034847.2	lncRNA

XM_015098529.1	NF1	mRNA	XR_001034847.2	lncRNA
XM_015098529.1	NF1	mRNA	XR_001034847.2	lncRNA
XM_012176498.2	RAPGEF5	mRNA	XR_001436102.1	lncRNA
XM_012176498.2	RAPGEF5	mRNA	XR_001436102.1	lncRNA
XM_012176498.2	RAPGEF5	mRNA	XR_001436102.1	lncRNA
XM_012176498.2	RAPGEF5	mRNA	XR_001436102.1	lncRNA
XM_012176498.2	RAPGEF5	mRNA	XR_001436102.1	lncRNA
XM_012176498.2	RAPGEF5	mRNA	XR_001436102.1	lncRNA
XM_012176498.2	RAPGEF5	mRNA	XR_001436102.1	lncRNA
XM_012176498.2	RAPGEF5	mRNA	XR_001436102.1	lncRNA
XM_012176498.2	RAPGEF5	mRNA	XR_001436102.1	lncRNA
XM_012176498.2	RAPGEF5	mRNA	XR_001436102.1	lncRNA
XM_012176498.2	RAPGEF5	mRNA	XR_001436102.1	lncRNA
XM_012098812.2	RALGAPA1	mRNA	XR_001436102.1	lncRNA
XM_012098812.2	RALGAPA1	mRNA	XR_001436102.1	lncRNA
XM_012098812.2	RALGAPA1	mRNA	XR_001436102.1	lncRNA
XM_012098812.2	RALGAPA1	mRNA	XR_001436102.1	lncRNA
XM_012098812.2	RALGAPA1	mRNA	XR_001436102.1	lncRNA
XM_012098812.2	RALGAPA1	mRNA	XR_001436102.1	lncRNA
XM_012098812.2	RALGAPA1	mRNA	XR_001436102.1	lncRNA
XM_012098812.2	RALGAPA1	mRNA	XR_001436102.1	lncRNA
XM_012098812.2	RALGAPA1	mRNA	XR_001436102.1	lncRNA
XM_012098812.2	RALGAPA1	mRNA	XR_001436102.1	lncRNA
XM_012098812.2	RALGAPA1	mRNA	XR_001436102.1	lncRNA
XM_012152455.2	LUZP1	mRNA	XR_001021689.2	lncRNA
XM_012152455.2	LUZP1	mRNA	XR_001021689.2	lncRNA
XM_012152455.2	LUZP1	mRNA	XR_001021689.2	lncRNA
XM_012152455.2	LUZP1	mRNA	XR_001021689.2	lncRNA
XM_012152455.2	LUZP1	mRNA	XR_001021689.2	lncRNA
XM_012152455.2	LUZP1	mRNA	XR_001021689.2	lncRNA
XM_012152455.2	LUZP1	mRNA	XR_001021689.2	lncRNA
XM_012152455.2	LUZP1	mRNA	XR_001021689.2	lncRNA
XM_012152455.2	LUZP1	mRNA	XR_001021689.2	lncRNA
XM_012152455.2	LUZP1	mRNA	XR_001021689.2	lncRNA
XM_012152455.2	LUZP1	mRNA	XR_001021689.2	lncRNA
XM_012178667.2	ARHGAP26	mRNA	XR_001021689.2	lncRNA
XM_012178667.2	ARHGAP26	mRNA	XR_001021689.2	lncRNA
XM_012178667.2	ARHGAP26	mRNA	XR_001021689.2	lncRNA
XM_012178667.2	ARHGAP26	mRNA	XR_001021689.2	lncRNA
XM_012178667.2	ARHGAP26	mRNA	XR_001021689.2	lncRNA
XM_012178667.2	ARHGAP26	mRNA	XR_001021689.2	lncRNA
XM_012178667.2	ARHGAP26	mRNA	XR_001021689.2	lncRNA
XM_012178667.2	ARHGAP26	mRNA	XR_001021689.2	lncRNA
XM_012178667.2	ARHGAP26	mRNA	XR_001021689.2	lncRNA
XM_012178667.2	ARHGAP26	mRNA	XR_001021689.2	lncRNA
XM_012178667.2	ARHGAP26	mRNA	XR_001021689.2	lncRNA
XM_004008647.3	RAPGEF6	mRNA	XR_001436102.1	lncRNA
XM_004008647.3	RAPGEF6	mRNA	XR_001436102.1	lncRNA
XM_004008647.3	RAPGEF6	mRNA	XR_001436102.1	lncRNA
XM_004008647.3	RAPGEF6	mRNA	XR_001436102.1	lncRNA
XM_004008647.3	RAPGEF6	mRNA	XR_001436102.1	lncRNA
XM_004008647.3	RAPGEF6	mRNA	XR_001436102.1	lncRNA
XM_004008647.3	RAPGEF6	mRNA	XR_001436102.1	lncRNA
XM_004008647.3	RAPGEF6	mRNA	XR_001436102.1	lncRNA

XM_004008647.3	RAPGEF6	mRNA	XR_001436102.1	lncRNA
XM_004008647.3	RAPGEF6	mRNA	XR_001436102.1	lncRNA
XM_012168955.2	DNAJC6	mRNA	XR_001436102.1	lncRNA
XM_012168955.2	DNAJC6	mRNA	XR_001436102.1	lncRNA
XM_012168955.2	DNAJC6	mRNA	XR_001436102.1	lncRNA
XM_012168955.2	DNAJC6	mRNA	XR_001436102.1	lncRNA
XM_012168955.2	DNAJC6	mRNA	XR_001436102.1	lncRNA
XM_012168955.2	DNAJC6	mRNA	XR_001436102.1	lncRNA
XM_012168955.2	DNAJC6	mRNA	XR_001436102.1	lncRNA
XM_012168955.2	DNAJC6	mRNA	XR_001436102.1	lncRNA
XM_012168955.2	DNAJC6	mRNA	XR_001436102.1	lncRNA
XM_012168955.2	DNAJC6	mRNA	XR_001436102.1	lncRNA
XM_012151052.2	WASF2	mRNA	XR_001436817.1	lncRNA
XM_012151052.2	WASF2	mRNA	XR_001436817.1	lncRNA
XM_012151052.2	WASF2	mRNA	XR_001436817.1	lncRNA
XM_012151052.2	WASF2	mRNA	XR_001436817.1	lncRNA
XM_012151052.2	WASF2	mRNA	XR_001436817.1	lncRNA
XM_012151052.2	WASF2	mRNA	XR_001436817.1	lncRNA
XM_012151052.2	WASF2	mRNA	XR_001436817.1	lncRNA
XM_012151052.2	WASF2	mRNA	XR_001436817.1	lncRNA
XM_012151052.2	WASF2	mRNA	XR_001436817.1	lncRNA
XM_012151052.2	WASF2	mRNA	XR_001436817.1	lncRNA
XM_012151052.2	WASF2	mRNA	XR_001436817.1	lncRNA
XM_015101485.1	LOC106991694	mRNA	XR_001436817.1	lncRNA
XM_015101485.1	LOC106991694	mRNA	XR_001436817.1	lncRNA
XM_015101485.1	LOC106991694	mRNA	XR_001436817.1	lncRNA
XM_015101485.1	LOC106991694	mRNA	XR_001436817.1	lncRNA
XM_015101485.1	LOC106991694	mRNA	XR_001436817.1	lncRNA
XM_015101485.1	LOC106991694	mRNA	XR_001436817.1	lncRNA
XM_015101485.1	LOC106991694	mRNA	XR_001436817.1	lncRNA
XM_015101485.1	LOC106991694	mRNA	XR_001436817.1	lncRNA
XM_015101485.1	LOC106991694	mRNA	XR_001436817.1	lncRNA
XM_015101485.1	LOC106991694	mRNA	XR_001436817.1	lncRNA
XM_015101485.1	LOC106991694	mRNA	XR_001436817.1	lncRNA
XM_012097610.2	ZNF605	mRNA	XR_001436817.1	lncRNA
XM_012097610.2	ZNF605	mRNA	XR_001436817.1	lncRNA
XM_012097610.2	ZNF605	mRNA	XR_001436817.1	lncRNA
XM_012097610.2	ZNF605	mRNA	XR_001436817.1	lncRNA
XM_012097610.2	ZNF605	mRNA	XR_001436817.1	lncRNA
XM_012097610.2	ZNF605	mRNA	XR_001436817.1	lncRNA
XM_012097610.2	ZNF605	mRNA	XR_001436817.1	lncRNA
XM_012097610.2	ZNF605	mRNA	XR_001436817.1	lncRNA
XM_012097610.2	ZNF605	mRNA	XR_001436817.1	lncRNA
XM_012097610.2	ZNF605	mRNA	XR_001436817.1	lncRNA
XM_012097610.2	ZNF605	mRNA	XR_001436817.1	lncRNA
XM_012151052.2	WASF2	mRNA	XR_001434203.1	lncRNA
XM_012151052.2	WASF2	mRNA	XR_001434203.1	lncRNA
XM_012151052.2	WASF2	mRNA	XR_001434203.1	lncRNA
XM_012151052.2	WASF2	mRNA	XR_001434203.1	lncRNA
XM_012151052.2	WASF2	mRNA	XR_001434203.1	lncRNA
XM_012151052.2	WASF2	mRNA	XR_001434203.1	lncRNA
XM_012151052.2	WASF2	mRNA	XR_001434203.1	lncRNA
XM_012151052.2	WASF2	mRNA	XR_001434203.1	lncRNA
XM_012151052.2	WASF2	mRNA	XR_001434203.1	lncRNA

XM_012151052.2	WASF2	mRNA	XR_001434203.1	lncRNA
XM_012151052.2	WASF2	mRNA	XR_001434203.1	lncRNA
XM_015101485.1	LOC106991694	mRNA	XR_001434203.1	lncRNA
XM_015101485.1	LOC106991694	mRNA	XR_001434203.1	lncRNA
XM_015101485.1	LOC106991694	mRNA	XR_001434203.1	lncRNA
XM_015101485.1	LOC106991694	mRNA	XR_001434203.1	lncRNA
XM_015101485.1	LOC106991694	mRNA	XR_001434203.1	lncRNA
XM_015101485.1	LOC106991694	mRNA	XR_001434203.1	lncRNA
XM_015101485.1	LOC106991694	mRNA	XR_001434203.1	lncRNA
XM_015101485.1	LOC106991694	mRNA	XR_001434203.1	lncRNA
XM_015101485.1	LOC106991694	mRNA	XR_001434203.1	lncRNA
XM_015101485.1	LOC106991694	mRNA	XR_001434203.1	lncRNA
XM_015101485.1	LOC106991694	mRNA	XR_001434203.1	lncRNA
XM_012102512.2	HEYL	mRNA	XR_001434761.1	lncRNA
XM_012102512.2	HEYL	mRNA	XR_001434761.1	lncRNA
XM_012102512.2	HEYL	mRNA	XR_001434761.1	lncRNA
XM_012102512.2	HEYL	mRNA	XR_001434761.1	lncRNA
XM_012102512.2	HEYL	mRNA	XR_001434761.1	lncRNA
XM_012102512.2	HEYL	mRNA	XR_001434761.1	lncRNA
XM_012102512.2	HEYL	mRNA	XR_001434761.1	lncRNA
XM_012102512.2	HEYL	mRNA	XR_001434761.1	lncRNA
XM_012102512.2	HEYL	mRNA	XR_001434761.1	lncRNA
XM_012102512.2	HEYL	mRNA	XR_001434761.1	lncRNA
XM_012183229.2	QKI	mRNA	XR_001434892.1	lncRNA
XM_012183229.2	QKI	mRNA	XR_001434892.1	lncRNA
XM_012183229.2	QKI	mRNA	XR_001434892.1	lncRNA
XM_012183229.2	QKI	mRNA	XR_001434892.1	lncRNA
XM_012183229.2	QKI	mRNA	XR_001434892.1	lncRNA
XM_012183229.2	QKI	mRNA	XR_001434892.1	lncRNA
XM_012183229.2	QKI	mRNA	XR_001434892.1	lncRNA
XM_012183229.2	QKI	mRNA	XR_001434892.1	lncRNA
XM_012183229.2	QKI	mRNA	XR_001434892.1	lncRNA
XM_012183229.2	QKI	mRNA	XR_001434892.1	lncRNA
XM_012175045.1	FGD4	mRNA	XR_001434892.1	lncRNA
XM_012175045.1	FGD4	mRNA	XR_001434892.1	lncRNA
XM_012175045.1	FGD4	mRNA	XR_001434892.1	lncRNA
XM_012175045.1	FGD4	mRNA	XR_001434892.1	lncRNA
XM_012175045.1	FGD4	mRNA	XR_001434892.1	lncRNA
XM_012175045.1	FGD4	mRNA	XR_001434892.1	lncRNA
XM_012175045.1	FGD4	mRNA	XR_001434892.1	lncRNA
XM_012175045.1	FGD4	mRNA	XR_001434892.1	lncRNA
XM_012175045.1	FGD4	mRNA	XR_001434892.1	lncRNA
XM_012175045.1	FGD4	mRNA	XR_001434892.1	lncRNA
XM_012175045.1	FGD4	mRNA	XR_001434892.1	lncRNA
XM_012119517.2	SORBS3	mRNA	XR_001434761.1	lncRNA
XM_012119517.2	SORBS3	mRNA	XR_001434761.1	lncRNA
XM_012119517.2	SORBS3	mRNA	XR_001434761.1	lncRNA
XM_012119517.2	SORBS3	mRNA	XR_001434761.1	lncRNA
XM_012119517.2	SORBS3	mRNA	XR_001434761.1	lncRNA
XM_012119517.2	SORBS3	mRNA	XR_001434761.1	lncRNA
XM_012119517.2	SORBS3	mRNA	XR_001434761.1	lncRNA
XM_012119517.2	SORBS3	mRNA	XR_001434761.1	lncRNA
XM_012119517.2	SORBS3	mRNA	XR_001434761.1	lncRNA
XM_012119517.2	SORBS3	mRNA	XR_001434761.1	lncRNA

XM_012119517.2	SORBS3	mRNA	XR_001434761.1	lncRNA
XM_015096594.1	SEC31A	mRNA	XR_001434761.1	lncRNA
XM_015096594.1	SEC31A	mRNA	XR_001434761.1	lncRNA
XM_015096594.1	SEC31A	mRNA	XR_001434761.1	lncRNA
XM_015096594.1	SEC31A	mRNA	XR_001434761.1	lncRNA
XM_015096594.1	SEC31A	mRNA	XR_001434761.1	lncRNA
XM_015096594.1	SEC31A	mRNA	XR_001434761.1	lncRNA
XM_015096594.1	SEC31A	mRNA	XR_001434761.1	lncRNA
XM_015096594.1	SEC31A	mRNA	XR_001434761.1	lncRNA
XM_015096594.1	SEC31A	mRNA	XR_001434761.1	lncRNA
XM_015096594.1	SEC31A	mRNA	XR_001434761.1	lncRNA
XM_015096594.1	SEC31A	mRNA	XR_001434761.1	lncRNA
XM_012178788.2	HMGXB3	mRNA	XR_001434761.1	lncRNA
XM_012178788.2	HMGXB3	mRNA	XR_001434761.1	lncRNA
XM_012178788.2	HMGXB3	mRNA	XR_001434761.1	lncRNA
XM_012178788.2	HMGXB3	mRNA	XR_001434761.1	lncRNA
XM_012178788.2	HMGXB3	mRNA	XR_001434761.1	lncRNA
XM_012178788.2	HMGXB3	mRNA	XR_001434761.1	lncRNA
XM_012178788.2	HMGXB3	mRNA	XR_001434761.1	lncRNA
XM_012178788.2	HMGXB3	mRNA	XR_001434761.1	lncRNA
XM_012178788.2	HMGXB3	mRNA	XR_001434761.1	lncRNA
XM_012178788.2	HMGXB3	mRNA	XR_001434761.1	lncRNA
XM_012178788.2	HMGXB3	mRNA	XR_001434761.1	lncRNA
XM_015095790.1	CC2D1A	mRNA	XR_001436532.1	lncRNA
XM_015095790.1	CC2D1A	mRNA	XR_001436532.1	lncRNA
XM_015095790.1	CC2D1A	mRNA	XR_001436532.1	lncRNA
XM_015095790.1	CC2D1A	mRNA	XR_001436532.1	lncRNA
XM_015095790.1	CC2D1A	mRNA	XR_001436532.1	lncRNA
XM_015095790.1	CC2D1A	mRNA	XR_001436532.1	lncRNA
XM_015095790.1	CC2D1A	mRNA	XR_001436532.1	lncRNA
XM_015095790.1	CC2D1A	mRNA	XR_001436532.1	lncRNA
XM_015095790.1	CC2D1A	mRNA	XR_001436532.1	lncRNA
XM_015095790.1	CC2D1A	mRNA	XR_001436532.1	lncRNA
XM_015095790.1	CC2D1A	mRNA	XR_001436532.1	lncRNA
XM_015100041.1	ZNF473	mRNA	XR_001436532.1	lncRNA
XM_015100041.1	ZNF473	mRNA	XR_001436532.1	lncRNA
XM_015100041.1	ZNF473	mRNA	XR_001436532.1	lncRNA
XM_015100041.1	ZNF473	mRNA	XR_001436532.1	lncRNA
XM_015100041.1	ZNF473	mRNA	XR_001436532.1	lncRNA
XM_015100041.1	ZNF473	mRNA	XR_001436532.1	lncRNA
XM_015100041.1	ZNF473	mRNA	XR_001436532.1	lncRNA
XM_015100041.1	ZNF473	mRNA	XR_001436532.1	lncRNA
XM_015100041.1	ZNF473	mRNA	XR_001436532.1	lncRNA
XM_015100041.1	ZNF473	mRNA	XR_001436532.1	lncRNA
XM_015100041.1	ZNF473	mRNA	XR_001436532.1	lncRNA
XM_015094471.1	CCDC142	mRNA	XR_001436532.1	lncRNA
XM_015094471.1	CCDC142	mRNA	XR_001436532.1	lncRNA
XM_015094471.1	CCDC142	mRNA	XR_001436532.1	lncRNA
XM_015094471.1	CCDC142	mRNA	XR_001436532.1	lncRNA
XM_015094471.1	CCDC142	mRNA	XR_001436532.1	lncRNA
XM_015094471.1	CCDC142	mRNA	XR_001436532.1	lncRNA
XM_015094471.1	CCDC142	mRNA	XR_001436532.1	lncRNA
XM_015094471.1	CCDC142	mRNA	XR_001436532.1	lncRNA
XM_015094471.1	CCDC142	mRNA	XR_001436532.1	lncRNA

XM_015094471.1	CCDC142	mRNA	XR_001436532.1	lncRNA
XM_015094471.1	CCDC142	mRNA	XR_001436532.1	lncRNA
XM_004010552.3	VPS13C	mRNA	XR_001436532.1	lncRNA
XM_004010552.3	VPS13C	mRNA	XR_001436532.1	lncRNA
XM_004010552.3	VPS13C	mRNA	XR_001436532.1	lncRNA
XM_004010552.3	VPS13C	mRNA	XR_001436532.1	lncRNA
XM_004010552.3	VPS13C	mRNA	XR_001436532.1	lncRNA
XM_004010552.3	VPS13C	mRNA	XR_001436532.1	lncRNA
XM_004010552.3	VPS13C	mRNA	XR_001436532.1	lncRNA
XM_004010552.3	VPS13C	mRNA	XR_001436532.1	lncRNA
XM_004010552.3	VPS13C	mRNA	XR_001436532.1	lncRNA
XM_004010552.3	VPS13C	mRNA	XR_001436532.1	lncRNA
XM_004010552.3	VPS13C	mRNA	XR_001436532.1	lncRNA
XM_015097502.1	SNX9	mRNA	XR_001042381.2	lncRNA
XM_015097502.1	SNX9	mRNA	XR_001042381.2	lncRNA
XM_015097502.1	SNX9	mRNA	XR_001042381.2	lncRNA
XM_015097502.1	SNX9	mRNA	XR_001042381.2	lncRNA
XM_015097502.1	SNX9	mRNA	XR_001042381.2	lncRNA
XM_015097502.1	SNX9	mRNA	XR_001042381.2	lncRNA
XM_015097502.1	SNX9	mRNA	XR_001042381.2	lncRNA
XM_015097502.1	SNX9	mRNA	XR_001042381.2	lncRNA
XM_015097502.1	SNX9	mRNA	XR_001042381.2	lncRNA
XM_015097502.1	SNX9	mRNA	XR_001042381.2	lncRNA
XM_015097502.1	SNX9	mRNA	XR_001042381.2	lncRNA
XM_012184885.2	DOCK9	mRNA	XR_001025491.1	lncRNA
XM_012184885.2	DOCK9	mRNA	XR_001025491.1	lncRNA
XM_012184885.2	DOCK9	mRNA	XR_001025491.1	lncRNA
XM_012184885.2	DOCK9	mRNA	XR_001025491.1	lncRNA
XM_012184885.2	DOCK9	mRNA	XR_001025491.1	lncRNA
XM_012184885.2	DOCK9	mRNA	XR_001025491.1	lncRNA
XM_012184885.2	DOCK9	mRNA	XR_001025491.1	lncRNA
XM_012184885.2	DOCK9	mRNA	XR_001025491.1	lncRNA
XM_012184885.2	DOCK9	mRNA	XR_001025491.1	lncRNA
XM_012184885.2	DOCK9	mRNA	XR_001025491.1	lncRNA
XM_012095502.1	SIK3	mRNA	XR_001436102.1	lncRNA
XM_012095502.1	SIK3	mRNA	XR_001436102.1	lncRNA
XM_012095502.1	SIK3	mRNA	XR_001436102.1	lncRNA
XM_012095502.1	SIK3	mRNA	XR_001436102.1	lncRNA
XM_012095502.1	SIK3	mRNA	XR_001436102.1	lncRNA
XM_012095502.1	SIK3	mRNA	XR_001436102.1	lncRNA
XM_012095502.1	SIK3	mRNA	XR_001436102.1	lncRNA
XM_012095502.1	SIK3	mRNA	XR_001436102.1	lncRNA
XM_012095502.1	SIK3	mRNA	XR_001436102.1	lncRNA
XM_012095502.1	SIK3	mRNA	XR_001436102.1	lncRNA
XM_012095502.1	SIK3	mRNA	XR_001436102.1	lncRNA
XM_015098446.1	VEZF1	mRNA	XR_001021689.2	lncRNA
XM_015098446.1	VEZF1	mRNA	XR_001021689.2	lncRNA
XM_015098446.1	VEZF1	mRNA	XR_001021689.2	lncRNA
XM_015098446.1	VEZF1	mRNA	XR_001021689.2	lncRNA
XM_015098446.1	VEZF1	mRNA	XR_001021689.2	lncRNA
XM_015098446.1	VEZF1	mRNA	XR_001021689.2	lncRNA
XM_015098446.1	VEZF1	mRNA	XR_001021689.2	lncRNA
XM_015098446.1	VEZF1	mRNA	XR_001021689.2	lncRNA
XM_015098446.1	VEZF1	mRNA	XR_001021689.2	lncRNA

XM_015098446.1	VEZF1	mRNA	XR_001021689.2	lncRNA
XM_015098446.1	VEZF1	mRNA	XR_001021689.2	lncRNA
XM_015105096.1	PAK3	mRNA	XR_001434892.1	lncRNA
XM_015105096.1	PAK3	mRNA	XR_001434892.1	lncRNA
XM_015105096.1	PAK3	mRNA	XR_001434892.1	lncRNA
XM_015105096.1	PAK3	mRNA	XR_001434892.1	lncRNA
XM_015105096.1	PAK3	mRNA	XR_001434892.1	lncRNA
XM_015105096.1	PAK3	mRNA	XR_001434892.1	lncRNA
XM_015105096.1	PAK3	mRNA	XR_001434892.1	lncRNA
XM_015105096.1	PAK3	mRNA	XR_001434892.1	lncRNA
XM_015105096.1	PAK3	mRNA	XR_001434892.1	lncRNA
XM_015105096.1	PAK3	mRNA	XR_001434892.1	lncRNA
XM_015105096.1	PAK3	mRNA	XR_001434892.1	lncRNA
XM_012099038.2	SLC25A29	mRNA	XR_001434761.1	lncRNA
XM_012099038.2	SLC25A29	mRNA	XR_001434761.1	lncRNA
XM_012099038.2	SLC25A29	mRNA	XR_001434761.1	lncRNA
XM_012099038.2	SLC25A29	mRNA	XR_001434761.1	lncRNA
XM_012099038.2	SLC25A29	mRNA	XR_001434761.1	lncRNA
XM_012099038.2	SLC25A29	mRNA	XR_001434761.1	lncRNA
XM_012099038.2	SLC25A29	mRNA	XR_001434761.1	lncRNA
XM_012099038.2	SLC25A29	mRNA	XR_001434761.1	lncRNA
XM_012099038.2	SLC25A29	mRNA	XR_001434761.1	lncRNA
XM_012099038.2	SLC25A29	mRNA	XR_001434761.1	lncRNA
XM_012099038.2	SLC25A29	mRNA	XR_001434761.1	lncRNA
XM_015101107.1	TCP11L1	mRNA	XR_001042381.2	lncRNA
XM_015101107.1	TCP11L1	mRNA	XR_001042381.2	lncRNA
XM_015101107.1	TCP11L1	mRNA	XR_001042381.2	lncRNA
XM_015101107.1	TCP11L1	mRNA	XR_001042381.2	lncRNA
XM_015101107.1	TCP11L1	mRNA	XR_001042381.2	lncRNA
XM_015101107.1	TCP11L1	mRNA	XR_001042381.2	lncRNA
XM_015101107.1	TCP11L1	mRNA	XR_001042381.2	lncRNA
XM_015101107.1	TCP11L1	mRNA	XR_001042381.2	lncRNA
XM_015101107.1	TCP11L1	mRNA	XR_001042381.2	lncRNA
XM_015101107.1	TCP11L1	mRNA	XR_001042381.2	lncRNA
XM_012183229.2	QKI	mRNA	XR_001436532.1	lncRNA
XM_012183229.2	QKI	mRNA	XR_001436532.1	lncRNA
XM_012183229.2	QKI	mRNA	XR_001436532.1	lncRNA
XM_012183229.2	QKI	mRNA	XR_001436532.1	lncRNA
XM_012183229.2	QKI	mRNA	XR_001436532.1	lncRNA
XM_012183229.2	QKI	mRNA	XR_001436532.1	lncRNA
XM_012183229.2	QKI	mRNA	XR_001436532.1	lncRNA
XM_012183229.2	QKI	mRNA	XR_001436532.1	lncRNA
XM_012183229.2	QKI	mRNA	XR_001436532.1	lncRNA
XM_012183229.2	QKI	mRNA	XR_001436532.1	lncRNA
XM_012107698.1	NFYC	mRNA	XR_001436532.1	lncRNA
XM_012107698.1	NFYC	mRNA	XR_001436532.1	lncRNA
XM_012107698.1	NFYC	mRNA	XR_001436532.1	lncRNA
XM_012107698.1	NFYC	mRNA	XR_001436532.1	lncRNA
XM_012107698.1	NFYC	mRNA	XR_001436532.1	lncRNA
XM_012107698.1	NFYC	mRNA	XR_001436532.1	lncRNA
XM_012107698.1	NFYC	mRNA	XR_001436532.1	lncRNA
XM_012107698.1	NFYC	mRNA	XR_001436532.1	lncRNA
XM_012107698.1	NFYC	mRNA	XR_001436532.1	lncRNA
XM_012107698.1	NFYC	mRNA	XR_001436532.1	lncRNA

XM_015092421.1	BBX	mRNA	XR_001436102.1	lncRNA
XM_015092421.1	BBX	mRNA	XR_001436102.1	lncRNA
XM_015092421.1	BBX	mRNA	XR_001436102.1	lncRNA
XM_015092421.1	BBX	mRNA	XR_001436102.1	lncRNA
XM_015092421.1	BBX	mRNA	XR_001436102.1	lncRNA
XM_015092421.1	BBX	mRNA	XR_001436102.1	lncRNA
XM_015092421.1	BBX	mRNA	XR_001436102.1	lncRNA
XM_015092421.1	BBX	mRNA	XR_001436102.1	lncRNA
XM_015092421.1	BBX	mRNA	XR_001436102.1	lncRNA
XM_015092421.1	BBX	mRNA	XR_001436102.1	lncRNA
XM_012188768.2	UBOX5	mRNA	XR_001436102.1	lncRNA
XM_012188768.2	UBOX5	mRNA	XR_001436102.1	lncRNA
XM_012188768.2	UBOX5	mRNA	XR_001436102.1	lncRNA
XM_012188768.2	UBOX5	mRNA	XR_001436102.1	lncRNA
XM_012188768.2	UBOX5	mRNA	XR_001436102.1	lncRNA
XM_012188768.2	UBOX5	mRNA	XR_001436102.1	lncRNA
XM_012188768.2	UBOX5	mRNA	XR_001436102.1	lncRNA
XM_012188768.2	UBOX5	mRNA	XR_001436102.1	lncRNA
XM_012188768.2	UBOX5	mRNA	XR_001436102.1	lncRNA
XM_012188768.2	UBOX5	mRNA	XR_001436102.1	lncRNA
XM_012106817.2	JADE3	mRNA	XR_001021689.2	lncRNA
XM_012106817.2	JADE3	mRNA	XR_001021689.2	lncRNA
XM_012106817.2	JADE3	mRNA	XR_001021689.2	lncRNA
XM_012106817.2	JADE3	mRNA	XR_001021689.2	lncRNA
XM_012106817.2	JADE3	mRNA	XR_001021689.2	lncRNA
XM_012106817.2	JADE3	mRNA	XR_001021689.2	lncRNA
XM_012106817.2	JADE3	mRNA	XR_001021689.2	lncRNA
XM_012106817.2	JADE3	mRNA	XR_001021689.2	lncRNA
XM_012106817.2	JADE3	mRNA	XR_001021689.2	lncRNA
XM_012106817.2	JADE3	mRNA	XR_001021689.2	lncRNA
XM_015098660.1	MAP2K4	mRNA	XR_001021689.2	lncRNA
XM_015098660.1	MAP2K4	mRNA	XR_001021689.2	lncRNA
XM_015098660.1	MAP2K4	mRNA	XR_001021689.2	lncRNA
XM_015098660.1	MAP2K4	mRNA	XR_001021689.2	lncRNA
XM_015098660.1	MAP2K4	mRNA	XR_001021689.2	lncRNA
XM_015098660.1	MAP2K4	mRNA	XR_001021689.2	lncRNA
XM_015098660.1	MAP2K4	mRNA	XR_001021689.2	lncRNA
XM_015098660.1	MAP2K4	mRNA	XR_001021689.2	lncRNA
XM_015098660.1	MAP2K4	mRNA	XR_001021689.2	lncRNA
XM_015098660.1	MAP2K4	mRNA	XR_001021689.2	lncRNA
XM_015092323.1	KIRREL	mRNA	XR_001034847.2	lncRNA
XM_015092323.1	KIRREL	mRNA	XR_001034847.2	lncRNA
XM_015092323.1	KIRREL	mRNA	XR_001034847.2	lncRNA
XM_015092323.1	KIRREL	mRNA	XR_001034847.2	lncRNA
XM_015092323.1	KIRREL	mRNA	XR_001034847.2	lncRNA
XM_015092323.1	KIRREL	mRNA	XR_001034847.2	lncRNA
XM_015092323.1	KIRREL	mRNA	XR_001034847.2	lncRNA
XM_015092323.1	KIRREL	mRNA	XR_001034847.2	lncRNA
XM_015092323.1	KIRREL	mRNA	XR_001034847.2	lncRNA
XM_015092323.1	KIRREL	mRNA	XR_001034847.2	lncRNA
XM_015092323.1	KIRREL	mRNA	XR_001034847.2	lncRNA
XM_015098446.1	VEZF1	mRNA	XR_001434202.1	lncRNA
XM_015098446.1	VEZF1	mRNA	XR_001434202.1	lncRNA
XM_015098446.1	VEZF1	mRNA	XR_001434202.1	lncRNA

XM_015098446.1	VEZF1	mRNA	XR_001434202.1	lncRNA
XM_015098446.1	VEZF1	mRNA	XR_001434202.1	lncRNA
XM_015098446.1	VEZF1	mRNA	XR_001434202.1	lncRNA
XM_015098446.1	VEZF1	mRNA	XR_001434202.1	lncRNA
XM_015098446.1	VEZF1	mRNA	XR_001434202.1	lncRNA
XM_015098446.1	VEZF1	mRNA	XR_001434202.1	lncRNA
XM_015098446.1	VEZF1	mRNA	XR_001434202.1	lncRNA
XM_015098446.1	VEZF1	mRNA	XR_001434202.1	lncRNA
XM_012181966.2	SIPA1L1	mRNA	XR_001034847.2	lncRNA
XM_012181966.2	SIPA1L1	mRNA	XR_001034847.2	lncRNA
XM_012181966.2	SIPA1L1	mRNA	XR_001034847.2	lncRNA
XM_012181966.2	SIPA1L1	mRNA	XR_001034847.2	lncRNA
XM_012181966.2	SIPA1L1	mRNA	XR_001034847.2	lncRNA
XM_012181966.2	SIPA1L1	mRNA	XR_001034847.2	lncRNA
XM_012181966.2	SIPA1L1	mRNA	XR_001034847.2	lncRNA
XM_012181966.2	SIPA1L1	mRNA	XR_001034847.2	lncRNA
XM_012181966.2	SIPA1L1	mRNA	XR_001034847.2	lncRNA
XM_012181966.2	SIPA1L1	mRNA	XR_001034847.2	lncRNA
XM_012181966.2	SIPA1L1	mRNA	XR_001034847.2	lncRNA
XM_012181966.2	SIPA1L1	mRNA	XR_001434202.1	lncRNA
XM_012181966.2	SIPA1L1	mRNA	XR_001434202.1	lncRNA
XM_012181966.2	SIPA1L1	mRNA	XR_001434202.1	lncRNA
XM_012181966.2	SIPA1L1	mRNA	XR_001434202.1	lncRNA
XM_012181966.2	SIPA1L1	mRNA	XR_001434202.1	lncRNA
XM_012181966.2	SIPA1L1	mRNA	XR_001434202.1	lncRNA
XM_012181966.2	SIPA1L1	mRNA	XR_001434202.1	lncRNA
XM_012181966.2	SIPA1L1	mRNA	XR_001434202.1	lncRNA
XM_012181966.2	SIPA1L1	mRNA	XR_001434202.1	lncRNA
XM_012181966.2	SIPA1L1	mRNA	XR_001434202.1	lncRNA
XM_012181966.2	SIPA1L1	mRNA	XR_001434202.1	lncRNA
XM_015098446.1	VEZF1	mRNA	XR_001034847.2	lncRNA
XM_015098446.1	VEZF1	mRNA	XR_001034847.2	lncRNA
XM_015098446.1	VEZF1	mRNA	XR_001034847.2	lncRNA
XM_015098446.1	VEZF1	mRNA	XR_001034847.2	lncRNA
XM_015098446.1	VEZF1	mRNA	XR_001034847.2	lncRNA
XM_015098446.1	VEZF1	mRNA	XR_001034847.2	lncRNA
XM_015098446.1	VEZF1	mRNA	XR_001034847.2	lncRNA
XM_015098446.1	VEZF1	mRNA	XR_001034847.2	lncRNA
XM_015098446.1	VEZF1	mRNA	XR_001034847.2	lncRNA
XM_015098446.1	VEZF1	mRNA	XR_001034847.2	lncRNA
XM_015098446.1	VEZF1	mRNA	XR_001034847.2	lncRNA
XM_015092323.1	KIRREL	mRNA	XR_001434202.1	lncRNA
XM_015092323.1	KIRREL	mRNA	XR_001434202.1	lncRNA
XM_015092323.1	KIRREL	mRNA	XR_001434202.1	lncRNA
XM_015092323.1	KIRREL	mRNA	XR_001434202.1	lncRNA
XM_015092323.1	KIRREL	mRNA	XR_001434202.1	lncRNA
XM_015092323.1	KIRREL	mRNA	XR_001434202.1	lncRNA
XM_015092323.1	KIRREL	mRNA	XR_001434202.1	lncRNA
XM_015092323.1	KIRREL	mRNA	XR_001434202.1	lncRNA
XM_015092323.1	KIRREL	mRNA	XR_001434202.1	lncRNA
XM_015092323.1	KIRREL	mRNA	XR_001434202.1	lncRNA
XM_015092323.1	KIRREL	mRNA	XR_001434202.1	lncRNA
XM_004011402.3	STXBP5	mRNA	XR_001436817.1	lncRNA
XM_004011402.3	STXBP5	mRNA	XR_001436817.1	lncRNA

XM_004011402.3	STXBP5	mRNA	XR_001436817.1	lncRNA
XM_004011402.3	STXBP5	mRNA	XR_001436817.1	lncRNA
XM_004011402.3	STXBP5	mRNA	XR_001436817.1	lncRNA
XM_004011402.3	STXBP5	mRNA	XR_001436817.1	lncRNA
XM_004011402.3	STXBP5	mRNA	XR_001436817.1	lncRNA
XM_004011402.3	STXBP5	mRNA	XR_001436817.1	lncRNA
XM_004011402.3	STXBP5	mRNA	XR_001436817.1	lncRNA
XM_004011402.3	STXBP5	mRNA	XR_001436817.1	lncRNA
XM_004011402.3	STXBP5	mRNA	XR_001436817.1	lncRNA
XM_015101031.1	MICAL2	mRNA	XR_001436532.1	lncRNA
XM_015101031.1	MICAL2	mRNA	XR_001436532.1	lncRNA
XM_015101031.1	MICAL2	mRNA	XR_001436532.1	lncRNA
XM_015101031.1	MICAL2	mRNA	XR_001436532.1	lncRNA
XM_015101031.1	MICAL2	mRNA	XR_001436532.1	lncRNA
XM_015101031.1	MICAL2	mRNA	XR_001436532.1	lncRNA
XM_015101031.1	MICAL2	mRNA	XR_001436532.1	lncRNA
XM_015101031.1	MICAL2	mRNA	XR_001436532.1	lncRNA
XM_015101031.1	MICAL2	mRNA	XR_001436532.1	lncRNA
XM_015101031.1	MICAL2	mRNA	XR_001436532.1	lncRNA
XM_015101031.1	MICAL2	mRNA	XR_001436532.1	lncRNA
XM_012124921.1	KIAA2026	mRNA	XR_001041211.2	lncRNA
XM_012124921.1	KIAA2026	mRNA	XR_001041211.2	lncRNA
XM_012124921.1	KIAA2026	mRNA	XR_001041211.2	lncRNA
XM_012124921.1	KIAA2026	mRNA	XR_001041211.2	lncRNA
XM_012124921.1	KIAA2026	mRNA	XR_001041211.2	lncRNA
XM_012124921.1	KIAA2026	mRNA	XR_001041211.2	lncRNA
XM_012124921.1	KIAA2026	mRNA	XR_001041211.2	lncRNA
XM_012124921.1	KIAA2026	mRNA	XR_001041211.2	lncRNA
XM_012124921.1	KIAA2026	mRNA	XR_001041211.2	lncRNA
XM_012124921.1	KIAA2026	mRNA	XR_001041211.2	lncRNA
XM_012107698.1	NFYC	mRNA	XR_001041211.2	lncRNA
XM_012107698.1	NFYC	mRNA	XR_001041211.2	lncRNA
XM_012107698.1	NFYC	mRNA	XR_001041211.2	lncRNA
XM_012107698.1	NFYC	mRNA	XR_001041211.2	lncRNA
XM_012107698.1	NFYC	mRNA	XR_001041211.2	lncRNA
XM_012107698.1	NFYC	mRNA	XR_001041211.2	lncRNA
XM_012107698.1	NFYC	mRNA	XR_001041211.2	lncRNA
XM_012107698.1	NFYC	mRNA	XR_001041211.2	lncRNA
XM_012107698.1	NFYC	mRNA	XR_001041211.2	lncRNA
XM_012107698.1	NFYC	mRNA	XR_001041211.2	lncRNA
XM_012183229.2	QKI	mRNA	XR_001041211.2	lncRNA
XM_012183229.2	QKI	mRNA	XR_001041211.2	lncRNA
XM_012183229.2	QKI	mRNA	XR_001041211.2	lncRNA
XM_012183229.2	QKI	mRNA	XR_001041211.2	lncRNA
XM_012183229.2	QKI	mRNA	XR_001041211.2	lncRNA
XM_012183229.2	QKI	mRNA	XR_001041211.2	lncRNA
XM_012183229.2	QKI	mRNA	XR_001041211.2	lncRNA
XM_012183229.2	QKI	mRNA	XR_001041211.2	lncRNA
XM_012183229.2	QKI	mRNA	XR_001041211.2	lncRNA
XM_012183229.2	QKI	mRNA	XR_001041211.2	lncRNA
XM_004016103.2	USP2	mRNA	XR_001434761.1	lncRNA
XM_004016103.2	USP2	mRNA	XR_001434761.1	lncRNA
XM_004016103.2	USP2	mRNA	XR_001434761.1	lncRNA
XM_004016103.2	USP2	mRNA	XR_001434761.1	lncRNA

XM_004016103.2	USP2	mRNA	XR_001434761.1	lncRNA
XM_004016103.2	USP2	mRNA	XR_001434761.1	lncRNA
XM_004016103.2	USP2	mRNA	XR_001434761.1	lncRNA
XM_004016103.2	USP2	mRNA	XR_001434761.1	lncRNA
XM_004016103.2	USP2	mRNA	XR_001434761.1	lncRNA
XM_004016103.2	USP2	mRNA	XR_001434761.1	lncRNA
XM_012185846.2	MBTD1	mRNA	XR_001434892.1	lncRNA
XM_012185846.2	MBTD1	mRNA	XR_001434892.1	lncRNA
XM_012185846.2	MBTD1	mRNA	XR_001434892.1	lncRNA
XM_012185846.2	MBTD1	mRNA	XR_001434892.1	lncRNA
XM_012185846.2	MBTD1	mRNA	XR_001434892.1	lncRNA
XM_012185846.2	MBTD1	mRNA	XR_001434892.1	lncRNA
XM_012185846.2	MBTD1	mRNA	XR_001434892.1	lncRNA
XM_012185846.2	MBTD1	mRNA	XR_001434892.1	lncRNA
XM_012185846.2	MBTD1	mRNA	XR_001434892.1	lncRNA
XM_012185846.2	MBTD1	mRNA	XR_001434892.1	lncRNA
XM_012098306.1	UBE3A	mRNA	XR_001434761.1	lncRNA
XM_012098306.1	UBE3A	mRNA	XR_001434761.1	lncRNA
XM_012098306.1	UBE3A	mRNA	XR_001434761.1	lncRNA
XM_012098306.1	UBE3A	mRNA	XR_001434761.1	lncRNA
XM_012098306.1	UBE3A	mRNA	XR_001434761.1	lncRNA
XM_012098306.1	UBE3A	mRNA	XR_001434761.1	lncRNA
XM_012098306.1	UBE3A	mRNA	XR_001434761.1	lncRNA
XM_012098306.1	UBE3A	mRNA	XR_001434761.1	lncRNA
XM_012098306.1	UBE3A	mRNA	XR_001434761.1	lncRNA
XM_012098306.1	UBE3A	mRNA	XR_001434761.1	lncRNA
XM_012178109.2	SGTA	mRNA	XR_001434761.1	lncRNA
XM_012178109.2	SGTA	mRNA	XR_001434761.1	lncRNA
XM_012178109.2	SGTA	mRNA	XR_001434761.1	lncRNA
XM_012178109.2	SGTA	mRNA	XR_001434761.1	lncRNA
XM_012178109.2	SGTA	mRNA	XR_001434761.1	lncRNA
XM_012178109.2	SGTA	mRNA	XR_001434761.1	lncRNA
XM_012178109.2	SGTA	mRNA	XR_001434761.1	lncRNA
XM_012178109.2	SGTA	mRNA	XR_001434761.1	lncRNA
XM_012178109.2	SGTA	mRNA	XR_001434761.1	lncRNA
XM_012178109.2	SGTA	mRNA	XR_001434761.1	lncRNA
XM_012175416.2	PKN2	mRNA	XR_001434892.1	lncRNA
XM_012175416.2	PKN2	mRNA	XR_001434892.1	lncRNA
XM_012175416.2	PKN2	mRNA	XR_001434892.1	lncRNA
XM_012175416.2	PKN2	mRNA	XR_001434892.1	lncRNA
XM_012175416.2	PKN2	mRNA	XR_001434892.1	lncRNA
XM_012175416.2	PKN2	mRNA	XR_001434892.1	lncRNA
XM_012175416.2	PKN2	mRNA	XR_001434892.1	lncRNA
XM_012175416.2	PKN2	mRNA	XR_001434892.1	lncRNA
XM_012175416.2	PKN2	mRNA	XR_001434892.1	lncRNA
XM_012175416.2	PKN2	mRNA	XR_001434892.1	lncRNA
XM_012168955.2	DNAJC6	mRNA	XR_001434761.1	lncRNA
XM_012168955.2	DNAJC6	mRNA	XR_001434761.1	lncRNA
XM_012168955.2	DNAJC6	mRNA	XR_001434761.1	lncRNA
XM_012168955.2	DNAJC6	mRNA	XR_001434761.1	lncRNA
XM_012168955.2	DNAJC6	mRNA	XR_001434761.1	lncRNA
XM_012168955.2	DNAJC6	mRNA	XR_001434761.1	lncRNA
XM_012168955.2	DNAJC6	mRNA	XR_001434761.1	lncRNA
XM_012168955.2	DNAJC6	mRNA	XR_001434761.1	lncRNA

XM_012168955.2	DNAJC6	mRNA	XR_001434761.1	lncRNA
XM_012168955.2	DNAJC6	mRNA	XR_001434761.1	lncRNA
XM_012190071.1	ATXN1L	mRNA	XR_001033284.2	lncRNA
XM_012190071.1	ATXN1L	mRNA	XR_001033284.2	lncRNA
XM_012190071.1	ATXN1L	mRNA	XR_001033284.2	lncRNA
XM_012190071.1	ATXN1L	mRNA	XR_001033284.2	lncRNA
XM_012190071.1	ATXN1L	mRNA	XR_001033284.2	lncRNA
XM_012190071.1	ATXN1L	mRNA	XR_001033284.2	lncRNA
XM_012190071.1	ATXN1L	mRNA	XR_001033284.2	lncRNA
XM_012190071.1	ATXN1L	mRNA	XR_001033284.2	lncRNA
XM_012190071.1	ATXN1L	mRNA	XR_001033284.2	lncRNA
XM_012190071.1	ATXN1L	mRNA	XR_001033284.2	lncRNA
XM_004006548.3	R3HDM2	mRNA	XR_001434202.1	lncRNA
XM_004006548.3	R3HDM2	mRNA	XR_001434202.1	lncRNA
XM_004006548.3	R3HDM2	mRNA	XR_001434202.1	lncRNA
XM_004006548.3	R3HDM2	mRNA	XR_001434202.1	lncRNA
XM_004006548.3	R3HDM2	mRNA	XR_001434202.1	lncRNA
XM_004006548.3	R3HDM2	mRNA	XR_001434202.1	lncRNA
XM_004006548.3	R3HDM2	mRNA	XR_001434202.1	lncRNA
XM_004006548.3	R3HDM2	mRNA	XR_001434202.1	lncRNA
XM_004006548.3	R3HDM2	mRNA	XR_001434202.1	lncRNA
XM_004006548.3	R3HDM2	mRNA	XR_001434202.1	lncRNA
XM_004006548.3	R3HDM2	mRNA	XR_001034847.2	lncRNA
XM_004006548.3	R3HDM2	mRNA	XR_001034847.2	lncRNA
XM_004006548.3	R3HDM2	mRNA	XR_001034847.2	lncRNA
XM_004006548.3	R3HDM2	mRNA	XR_001034847.2	lncRNA
XM_004006548.3	R3HDM2	mRNA	XR_001034847.2	lncRNA
XM_004006548.3	R3HDM2	mRNA	XR_001034847.2	lncRNA
XM_004006548.3	R3HDM2	mRNA	XR_001034847.2	lncRNA
XM_004006548.3	R3HDM2	mRNA	XR_001034847.2	lncRNA
XM_004006548.3	R3HDM2	mRNA	XR_001034847.2	lncRNA
XM_004006548.3	R3HDM2	mRNA	XR_001034847.2	lncRNA
XM_012174394.2	RAPGEF3	mRNA	XR_001436127.1	lncRNA
XM_012174394.2	RAPGEF3	mRNA	XR_001436127.1	lncRNA
XM_012174394.2	RAPGEF3	mRNA	XR_001436127.1	lncRNA
XM_012174394.2	RAPGEF3	mRNA	XR_001436127.1	lncRNA
XM_012174394.2	RAPGEF3	mRNA	XR_001436127.1	lncRNA
XM_012174394.2	RAPGEF3	mRNA	XR_001436127.1	lncRNA
XM_012174394.2	RAPGEF3	mRNA	XR_001436127.1	lncRNA
XM_012174394.2	RAPGEF3	mRNA	XR_001436127.1	lncRNA
XM_012174394.2	RAPGEF3	mRNA	XR_001436127.1	lncRNA
XM_012174394.2	RAPGEF3	mRNA	XR_001436127.1	lncRNA
XM_012185926.2	DLX4	mRNA	XR_001434202.1	lncRNA
XM_012185926.2	DLX4	mRNA	XR_001434202.1	lncRNA
XM_012185926.2	DLX4	mRNA	XR_001434202.1	lncRNA
XM_012185926.2	DLX4	mRNA	XR_001434202.1	lncRNA
XM_012185926.2	DLX4	mRNA	XR_001434202.1	lncRNA
XM_012185926.2	DLX4	mRNA	XR_001434202.1	lncRNA
XM_012185926.2	DLX4	mRNA	XR_001434202.1	lncRNA
XM_012185926.2	DLX4	mRNA	XR_001434202.1	lncRNA
XM_012185926.2	DLX4	mRNA	XR_001434202.1	lncRNA
XM_012185926.2	DLX4	mRNA	XR_001434202.1	lncRNA
XM_012185926.2	DLX4	mRNA	XR_001434202.1	lncRNA
XM_012185926.2	DLX4	mRNA	XR_001034847.2	lncRNA

XM_012185926.2	DLX4	mRNA	XR_001034847.2	lncRNA
XM_012185926.2	DLX4	mRNA	XR_001034847.2	lncRNA
XM_012185926.2	DLX4	mRNA	XR_001034847.2	lncRNA
XM_012185926.2	DLX4	mRNA	XR_001034847.2	lncRNA
XM_012185926.2	DLX4	mRNA	XR_001034847.2	lncRNA
XM_012185926.2	DLX4	mRNA	XR_001034847.2	lncRNA
XM_012185926.2	DLX4	mRNA	XR_001034847.2	lncRNA
XM_012185926.2	DLX4	mRNA	XR_001034847.2	lncRNA
XM_012185926.2	DLX4	mRNA	XR_001034847.2	lncRNA
XM_012185926.2	DLX4	mRNA	XR_001034847.2	lncRNA
XM_012181456.2	ADAM10	mRNA	XR_001434761.1	lncRNA
XM_012181456.2	ADAM10	mRNA	XR_001434761.1	lncRNA
XM_012181456.2	ADAM10	mRNA	XR_001434761.1	lncRNA
XM_012181456.2	ADAM10	mRNA	XR_001434761.1	lncRNA
XM_012181456.2	ADAM10	mRNA	XR_001434761.1	lncRNA
XM_012181456.2	ADAM10	mRNA	XR_001434761.1	lncRNA
XM_012181456.2	ADAM10	mRNA	XR_001434761.1	lncRNA
XM_012181456.2	ADAM10	mRNA	XR_001434761.1	lncRNA
XM_012181456.2	ADAM10	mRNA	XR_001434761.1	lncRNA
XM_012181456.2	ADAM10	mRNA	XR_001434761.1	lncRNA
XM_012181456.2	ADAM10	mRNA	XR_001434761.1	lncRNA
XM_012143277.2	ABI2	mRNA	XR_001434892.1	lncRNA
XM_012143277.2	ABI2	mRNA	XR_001434892.1	lncRNA
XM_012143277.2	ABI2	mRNA	XR_001434892.1	lncRNA
XM_012143277.2	ABI2	mRNA	XR_001434892.1	lncRNA
XM_012143277.2	ABI2	mRNA	XR_001434892.1	lncRNA
XM_012143277.2	ABI2	mRNA	XR_001434892.1	lncRNA
XM_012143277.2	ABI2	mRNA	XR_001434892.1	lncRNA
XM_012143277.2	ABI2	mRNA	XR_001434892.1	lncRNA
XM_012143277.2	ABI2	mRNA	XR_001434892.1	lncRNA
XM_012143277.2	ABI2	mRNA	XR_001434892.1	lncRNA
XM_012143277.2	ABI2	mRNA	XR_001434892.1	lncRNA
XM_015101107.1	TCP11L1	mRNA	XR_001436817.1	lncRNA
XM_015101107.1	TCP11L1	mRNA	XR_001436817.1	lncRNA
XM_015101107.1	TCP11L1	mRNA	XR_001436817.1	lncRNA
XM_015101107.1	TCP11L1	mRNA	XR_001436817.1	lncRNA
XM_015101107.1	TCP11L1	mRNA	XR_001436817.1	lncRNA
XM_015101107.1	TCP11L1	mRNA	XR_001436817.1	lncRNA
XM_015101107.1	TCP11L1	mRNA	XR_001436817.1	lncRNA
XM_015101107.1	TCP11L1	mRNA	XR_001436817.1	lncRNA
XM_015101107.1	TCP11L1	mRNA	XR_001436817.1	lncRNA
XM_015101107.1	TCP11L1	mRNA	XR_001436817.1	lncRNA
XM_004007700.3	CLDN12	mRNA	XR_001436817.1	lncRNA
XM_004007700.3	CLDN12	mRNA	XR_001436817.1	lncRNA
XM_004007700.3	CLDN12	mRNA	XR_001436817.1	lncRNA
XM_004007700.3	CLDN12	mRNA	XR_001436817.1	lncRNA
XM_004007700.3	CLDN12	mRNA	XR_001436817.1	lncRNA
XM_004007700.3	CLDN12	mRNA	XR_001436817.1	lncRNA
XM_004007700.3	CLDN12	mRNA	XR_001436817.1	lncRNA
XM_004007700.3	CLDN12	mRNA	XR_001436817.1	lncRNA
XM_004007700.3	CLDN12	mRNA	XR_001436817.1	lncRNA
XM_004007700.3	CLDN12	mRNA	XR_001436817.1	lncRNA
XM_012184823.2	RBM26	mRNA	XR_001436817.1	lncRNA
XM_012184823.2	RBM26	mRNA	XR_001436817.1	lncRNA

XM_012184823.2	RBM26	mRNA	XR_001436817.1	lncRNA
XM_012184823.2	RBM26	mRNA	XR_001436817.1	lncRNA
XM_012184823.2	RBM26	mRNA	XR_001436817.1	lncRNA
XM_012184823.2	RBM26	mRNA	XR_001436817.1	lncRNA
XM_012184823.2	RBM26	mRNA	XR_001436817.1	lncRNA
XM_012184823.2	RBM26	mRNA	XR_001436817.1	lncRNA
XM_012184823.2	RBM26	mRNA	XR_001436817.1	lncRNA
XM_012184823.2	RBM26	mRNA	XR_001436817.1	lncRNA
XM_012187546.2	ANKRD45	mRNA	XR_001434203.1	lncRNA
XM_012187546.2	ANKRD45	mRNA	XR_001434203.1	lncRNA
XM_012187546.2	ANKRD45	mRNA	XR_001434203.1	lncRNA
XM_012187546.2	ANKRD45	mRNA	XR_001434203.1	lncRNA
XM_012187546.2	ANKRD45	mRNA	XR_001434203.1	lncRNA
XM_012187546.2	ANKRD45	mRNA	XR_001434203.1	lncRNA
XM_012187546.2	ANKRD45	mRNA	XR_001434203.1	lncRNA
XM_012187546.2	ANKRD45	mRNA	XR_001434203.1	lncRNA
XM_012187546.2	ANKRD45	mRNA	XR_001434203.1	lncRNA
XM_012187546.2	ANKRD45	mRNA	XR_001434203.1	lncRNA
XM_015092486.1	PIGZ	mRNA	XR_001436102.1	lncRNA
XM_015092486.1	PIGZ	mRNA	XR_001436102.1	lncRNA
XM_015092486.1	PIGZ	mRNA	XR_001436102.1	lncRNA
XM_015092486.1	PIGZ	mRNA	XR_001436102.1	lncRNA
XM_015092486.1	PIGZ	mRNA	XR_001436102.1	lncRNA
XM_015092486.1	PIGZ	mRNA	XR_001436102.1	lncRNA
XM_015092486.1	PIGZ	mRNA	XR_001436102.1	lncRNA
XM_015092486.1	PIGZ	mRNA	XR_001436102.1	lncRNA
XM_015092486.1	PIGZ	mRNA	XR_001436102.1	lncRNA
XM_015092486.1	PIGZ	mRNA	XR_001436102.1	lncRNA
XM_012174536.2	KIF21A	mRNA	XR_001436102.1	lncRNA
XM_012174536.2	KIF21A	mRNA	XR_001436102.1	lncRNA
XM_012174536.2	KIF21A	mRNA	XR_001436102.1	lncRNA
XM_012174536.2	KIF21A	mRNA	XR_001436102.1	lncRNA
XM_012174536.2	KIF21A	mRNA	XR_001436102.1	lncRNA
XM_012174536.2	KIF21A	mRNA	XR_001436102.1	lncRNA
XM_012174536.2	KIF21A	mRNA	XR_001436102.1	lncRNA
XM_012174536.2	KIF21A	mRNA	XR_001436102.1	lncRNA
XM_012174536.2	KIF21A	mRNA	XR_001436102.1	lncRNA
XM_012174536.2	KIF21A	mRNA	XR_001436102.1	lncRNA
XM_004011402.3	STXBP5	mRNA	XR_001436102.1	lncRNA
XM_004011402.3	STXBP5	mRNA	XR_001436102.1	lncRNA
XM_004011402.3	STXBP5	mRNA	XR_001436102.1	lncRNA
XM_004011402.3	STXBP5	mRNA	XR_001436102.1	lncRNA
XM_004011402.3	STXBP5	mRNA	XR_001436102.1	lncRNA
XM_004011402.3	STXBP5	mRNA	XR_001436102.1	lncRNA
XM_004011402.3	STXBP5	mRNA	XR_001436102.1	lncRNA
XM_004011402.3	STXBP5	mRNA	XR_001436102.1	lncRNA
XM_004011402.3	STXBP5	mRNA	XR_001436102.1	lncRNA
XM_004011402.3	STXBP5	mRNA	XR_001436102.1	lncRNA
XM_015099256.1	CEP104	mRNA	XR_001436532.1	lncRNA
XM_015099256.1	CEP104	mRNA	XR_001436532.1	lncRNA
XM_015099256.1	CEP104	mRNA	XR_001436532.1	lncRNA
XM_015099256.1	CEP104	mRNA	XR_001436532.1	lncRNA
XM_015099256.1	CEP104	mRNA	XR_001436532.1	lncRNA
XM_015099256.1	CEP104	mRNA	XR_001436532.1	lncRNA

XM_015099256.1	CEP104	mRNA	XR_001436532.1	lncRNA
XM_015099256.1	CEP104	mRNA	XR_001436532.1	lncRNA
XM_015099256.1	CEP104	mRNA	XR_001436532.1	lncRNA
XM_015099256.1	CEP104	mRNA	XR_001436532.1	lncRNA
XM_015099256.1	CEP104	mRNA	XR_001436532.1	lncRNA
XM_015103466.1	TACC2	mRNA	XR_001033284.2	lncRNA
XM_015103466.1	TACC2	mRNA	XR_001033284.2	lncRNA
XM_015103466.1	TACC2	mRNA	XR_001033284.2	lncRNA
XM_015103466.1	TACC2	mRNA	XR_001033284.2	lncRNA
XM_015103466.1	TACC2	mRNA	XR_001033284.2	lncRNA
XM_015103466.1	TACC2	mRNA	XR_001033284.2	lncRNA
XM_015103466.1	TACC2	mRNA	XR_001033284.2	lncRNA
XM_015103466.1	TACC2	mRNA	XR_001033284.2	lncRNA
XM_015103466.1	TACC2	mRNA	XR_001033284.2	lncRNA
XM_015103466.1	TACC2	mRNA	XR_001033284.2	lncRNA
XM_012185285.2	SYNRG	mRNA	XR_001436127.1	lncRNA
XM_012185285.2	SYNRG	mRNA	XR_001436127.1	lncRNA
XM_012185285.2	SYNRG	mRNA	XR_001436127.1	lncRNA
XM_012185285.2	SYNRG	mRNA	XR_001436127.1	lncRNA
XM_012185285.2	SYNRG	mRNA	XR_001436127.1	lncRNA
XM_012185285.2	SYNRG	mRNA	XR_001436127.1	lncRNA
XM_012185285.2	SYNRG	mRNA	XR_001436127.1	lncRNA
XM_012185285.2	SYNRG	mRNA	XR_001436127.1	lncRNA
XM_012185285.2	SYNRG	mRNA	XR_001436127.1	lncRNA
XM_012185285.2	SYNRG	mRNA	XR_001436127.1	lncRNA
XM_012096615.2	DLG1	mRNA	XR_001436102.1	lncRNA
XM_012096615.2	DLG1	mRNA	XR_001436102.1	lncRNA
XM_012096615.2	DLG1	mRNA	XR_001436102.1	lncRNA
XM_012096615.2	DLG1	mRNA	XR_001436102.1	lncRNA
XM_012096615.2	DLG1	mRNA	XR_001436102.1	lncRNA
XM_012096615.2	DLG1	mRNA	XR_001436102.1	lncRNA
XM_012096615.2	DLG1	mRNA	XR_001436102.1	lncRNA
XM_012096615.2	DLG1	mRNA	XR_001436102.1	lncRNA
XM_012096615.2	DLG1	mRNA	XR_001436102.1	lncRNA
XM_015102111.1	EIF4E3	mRNA	XR_001021689.2	lncRNA
XM_015102111.1	EIF4E3	mRNA	XR_001021689.2	lncRNA
XM_015102111.1	EIF4E3	mRNA	XR_001021689.2	lncRNA
XM_015102111.1	EIF4E3	mRNA	XR_001021689.2	lncRNA
XM_015102111.1	EIF4E3	mRNA	XR_001021689.2	lncRNA
XM_015102111.1	EIF4E3	mRNA	XR_001021689.2	lncRNA
XM_015102111.1	EIF4E3	mRNA	XR_001021689.2	lncRNA
XM_015102111.1	EIF4E3	mRNA	XR_001021689.2	lncRNA
XM_015102111.1	EIF4E3	mRNA	XR_001021689.2	lncRNA
XM_015098990.1	GNA13	mRNA	XR_001042381.2	lncRNA
XM_015098990.1	GNA13	mRNA	XR_001042381.2	lncRNA
XM_015098990.1	GNA13	mRNA	XR_001042381.2	lncRNA
XM_015098990.1	GNA13	mRNA	XR_001042381.2	lncRNA
XM_015098990.1	GNA13	mRNA	XR_001042381.2	lncRNA
XM_015098990.1	GNA13	mRNA	XR_001042381.2	lncRNA
XM_015098990.1	GNA13	mRNA	XR_001042381.2	lncRNA
XM_015098990.1	GNA13	mRNA	XR_001042381.2	lncRNA
XM_015098990.1	GNA13	mRNA	XR_001042381.2	lncRNA
XM_015098990.1	GNA13	mRNA	XR_001042381.2	lncRNA
XM_015102039.1	SYNE3	mRNA	XR_001436532.1	lncRNA

XM_015102039.1	SYNE3	mRNA	XR_001436532.1	lncRNA
XM_015102039.1	SYNE3	mRNA	XR_001436532.1	lncRNA
XM_015102039.1	SYNE3	mRNA	XR_001436532.1	lncRNA
XM_015102039.1	SYNE3	mRNA	XR_001436532.1	lncRNA
XM_015102039.1	SYNE3	mRNA	XR_001436532.1	lncRNA
XM_015102039.1	SYNE3	mRNA	XR_001436532.1	lncRNA
XM_015102039.1	SYNE3	mRNA	XR_001436532.1	lncRNA
XM_015102039.1	SYNE3	mRNA	XR_001436532.1	lncRNA
XM_015102039.1	SYNE3	mRNA	XR_001436532.1	lncRNA
XM_012183305.1	FAM120B	mRNA	XR_001042381.2	lncRNA
XM_012183305.1	FAM120B	mRNA	XR_001042381.2	lncRNA
XM_012183305.1	FAM120B	mRNA	XR_001042381.2	lncRNA
XM_012183305.1	FAM120B	mRNA	XR_001042381.2	lncRNA
XM_012183305.1	FAM120B	mRNA	XR_001042381.2	lncRNA
XM_012183305.1	FAM120B	mRNA	XR_001042381.2	lncRNA
XM_012183305.1	FAM120B	mRNA	XR_001042381.2	lncRNA
XM_012183305.1	FAM120B	mRNA	XR_001042381.2	lncRNA
XM_012183305.1	FAM120B	mRNA	XR_001042381.2	lncRNA
XM_012183305.1	FAM120B	mRNA	XR_001042381.2	lncRNA
XM_012103119.1	EDRF1	mRNA	XR_001436532.1	lncRNA
XM_012103119.1	EDRF1	mRNA	XR_001436532.1	lncRNA
XM_012103119.1	EDRF1	mRNA	XR_001436532.1	lncRNA
XM_012103119.1	EDRF1	mRNA	XR_001436532.1	lncRNA
XM_012103119.1	EDRF1	mRNA	XR_001436532.1	lncRNA
XM_012103119.1	EDRF1	mRNA	XR_001436532.1	lncRNA
XM_012103119.1	EDRF1	mRNA	XR_001436532.1	lncRNA
XM_012103119.1	EDRF1	mRNA	XR_001436532.1	lncRNA
XM_012103119.1	EDRF1	mRNA	XR_001436532.1	lncRNA
XM_012103119.1	EDRF1	mRNA	XR_001436532.1	lncRNA
XM_012096185.2	TCP11L1	mRNA	XR_001436532.1	lncRNA
XM_012096185.2	TCP11L1	mRNA	XR_001436532.1	lncRNA
XM_012096185.2	TCP11L1	mRNA	XR_001436532.1	lncRNA
XM_012096185.2	TCP11L1	mRNA	XR_001436532.1	lncRNA
XM_012096185.2	TCP11L1	mRNA	XR_001436532.1	lncRNA
XM_012096185.2	TCP11L1	mRNA	XR_001436532.1	lncRNA
XM_012096185.2	TCP11L1	mRNA	XR_001436532.1	lncRNA
XM_012096185.2	TCP11L1	mRNA	XR_001436532.1	lncRNA
XM_012096185.2	TCP11L1	mRNA	XR_001436532.1	lncRNA
XM_012096185.2	TCP11L1	mRNA	XR_001436532.1	lncRNA
XM_012182238.1	SPG11	mRNA	XR_001434761.1	lncRNA
XM_012182238.1	SPG11	mRNA	XR_001434761.1	lncRNA
XM_012182238.1	SPG11	mRNA	XR_001434761.1	lncRNA
XM_012182238.1	SPG11	mRNA	XR_001434761.1	lncRNA
XM_012182238.1	SPG11	mRNA	XR_001434761.1	lncRNA
XM_012182238.1	SPG11	mRNA	XR_001434761.1	lncRNA
XM_012182238.1	SPG11	mRNA	XR_001434761.1	lncRNA
XM_012182238.1	SPG11	mRNA	XR_001434761.1	lncRNA
XM_012182238.1	SPG11	mRNA	XR_001434761.1	lncRNA
XM_012182238.1	SPG11	mRNA	XR_001434761.1	lncRNA
XM_015091902.1	SCMH1	mRNA	XR_001434892.1	lncRNA
XM_015091902.1	SCMH1	mRNA	XR_001434892.1	lncRNA
XM_015091902.1	SCMH1	mRNA	XR_001434892.1	lncRNA
XM_015091902.1	SCMH1	mRNA	XR_001434892.1	lncRNA
XM_015091902.1	SCMH1	mRNA	XR_001434892.1	lncRNA

XM_015091902.1	SCMH1	mRNA	XR_001434892.1	lncRNA
XM_015091902.1	SCMH1	mRNA	XR_001434892.1	lncRNA
XM_015091902.1	SCMH1	mRNA	XR_001434892.1	lncRNA
XM_015091902.1	SCMH1	mRNA	XR_001434892.1	lncRNA
XM_015091902.1	SCMH1	mRNA	XR_001434892.1	lncRNA
XM_015092341.1	USP21	mRNA	XR_001434761.1	lncRNA
XM_015092341.1	USP21	mRNA	XR_001434761.1	lncRNA
XM_015092341.1	USP21	mRNA	XR_001434761.1	lncRNA
XM_015092341.1	USP21	mRNA	XR_001434761.1	lncRNA
XM_015092341.1	USP21	mRNA	XR_001434761.1	lncRNA
XM_015092341.1	USP21	mRNA	XR_001434761.1	lncRNA
XM_015092341.1	USP21	mRNA	XR_001434761.1	lncRNA
XM_015092341.1	USP21	mRNA	XR_001434761.1	lncRNA
XM_015092341.1	USP21	mRNA	XR_001434761.1	lncRNA
XM_015092341.1	USP21	mRNA	XR_001434761.1	lncRNA
XM_004010813.3	GSTM1	mRNA	XR_001434761.1	lncRNA
XM_004010813.3	GSTM1	mRNA	XR_001434761.1	lncRNA
XM_004010813.3	GSTM1	mRNA	XR_001434761.1	lncRNA
XM_004010813.3	GSTM1	mRNA	XR_001434761.1	lncRNA
XM_004010813.3	GSTM1	mRNA	XR_001434761.1	lncRNA
XM_004010813.3	GSTM1	mRNA	XR_001434761.1	lncRNA
XM_004010813.3	GSTM1	mRNA	XR_001434761.1	lncRNA
XM_004010813.3	GSTM1	mRNA	XR_001434761.1	lncRNA
XM_004010813.3	GSTM1	mRNA	XR_001434761.1	lncRNA
XM_004010813.3	GSTM1	mRNA	XR_001434761.1	lncRNA
XM_012137434.2	NEB	mRNA	XR_001044126.1	lncRNA
XM_012137434.2	NEB	mRNA	XR_001044126.1	lncRNA
XM_012137434.2	NEB	mRNA	XR_001044126.1	lncRNA
XM_012137434.2	NEB	mRNA	XR_001044126.1	lncRNA
XM_012137434.2	NEB	mRNA	XR_001044126.1	lncRNA
XM_012137434.2	NEB	mRNA	XR_001044126.1	lncRNA
XM_012137434.2	NEB	mRNA	XR_001044126.1	lncRNA
XM_012137434.2	NEB	mRNA	XR_001044126.1	lncRNA
XM_012137434.2	NEB	mRNA	XR_001044126.1	lncRNA
XM_012137434.2	NEB	mRNA	XR_001044126.1	lncRNA
XM_012137425.2	NEB	mRNA	XR_001436127.1	lncRNA
XM_012137425.2	NEB	mRNA	XR_001436127.1	lncRNA
XM_012137425.2	NEB	mRNA	XR_001436127.1	lncRNA
XM_012137425.2	NEB	mRNA	XR_001436127.1	lncRNA
XM_012137425.2	NEB	mRNA	XR_001436127.1	lncRNA
XM_012137425.2	NEB	mRNA	XR_001436127.1	lncRNA
XM_012137425.2	NEB	mRNA	XR_001436127.1	lncRNA
XM_012137425.2	NEB	mRNA	XR_001436127.1	lncRNA
XM_012137425.2	NEB	mRNA	XR_001436127.1	lncRNA
XM_012137425.2	NEB	mRNA	XR_001436127.1	lncRNA
XM_012185318.2	RAD51D	mRNA	TCONS_00022613	lncRNA
XM_012185318.2	RAD51D	mRNA	TCONS_00022613	lncRNA
XM_012185318.2	RAD51D	mRNA	TCONS_00022613	lncRNA
XM_012185318.2	RAD51D	mRNA	TCONS_00022613	lncRNA
XM_012185318.2	RAD51D	mRNA	TCONS_00022613	lncRNA
XM_012185318.2	RAD51D	mRNA	TCONS_00022613	lncRNA
XM_012185318.2	RAD51D	mRNA	TCONS_00022613	lncRNA
XM_012185318.2	RAD51D	mRNA	TCONS_00022613	lncRNA
XM_012185318.2	RAD51D	mRNA	TCONS_00022613	lncRNA

XM_012176498.2	RAPGEF5	mRNA	TCONS_00022613	lncRNA
XM_012176498.2	RAPGEF5	mRNA	TCONS_00022613	lncRNA
XM_012176498.2	RAPGEF5	mRNA	TCONS_00022613	lncRNA
XM_012176498.2	RAPGEF5	mRNA	TCONS_00022613	lncRNA
XM_012176498.2	RAPGEF5	mRNA	TCONS_00022613	lncRNA
XM_012176498.2	RAPGEF5	mRNA	TCONS_00022613	lncRNA
XM_012176498.2	RAPGEF5	mRNA	TCONS_00022613	lncRNA
XM_012176498.2	RAPGEF5	mRNA	TCONS_00022613	lncRNA
XM_012176498.2	RAPGEF5	mRNA	TCONS_00022613	lncRNA
XM_012183412.2	ZNF7	mRNA	XR_001021689.2	lncRNA
XM_012183412.2	ZNF7	mRNA	XR_001021689.2	lncRNA
XM_012183412.2	ZNF7	mRNA	XR_001021689.2	lncRNA
XM_012183412.2	ZNF7	mRNA	XR_001021689.2	lncRNA
XM_012183412.2	ZNF7	mRNA	XR_001021689.2	lncRNA
XM_012183412.2	ZNF7	mRNA	XR_001021689.2	lncRNA
XM_012183412.2	ZNF7	mRNA	XR_001021689.2	lncRNA
XM_012183412.2	ZNF7	mRNA	XR_001021689.2	lncRNA
XM_012183412.2	ZNF7	mRNA	XR_001021689.2	lncRNA
XM_012183412.2	ZNF7	mRNA	XR_001021689.2	lncRNA
XM_015102318.1	SETD5	mRNA	XR_001021689.2	lncRNA
XM_015102318.1	SETD5	mRNA	XR_001021689.2	lncRNA
XM_015102318.1	SETD5	mRNA	XR_001021689.2	lncRNA
XM_015102318.1	SETD5	mRNA	XR_001021689.2	lncRNA
XM_015102318.1	SETD5	mRNA	XR_001021689.2	lncRNA
XM_015102318.1	SETD5	mRNA	XR_001021689.2	lncRNA
XM_015102318.1	SETD5	mRNA	XR_001021689.2	lncRNA
XM_015102318.1	SETD5	mRNA	XR_001021689.2	lncRNA
XM_015102318.1	SETD5	mRNA	XR_001021689.2	lncRNA
XM_015102318.1	SETD5	mRNA	XR_001021689.2	lncRNA
XM_012134416.2	PPIG	mRNA	XR_001436102.1	lncRNA
XM_012134416.2	PPIG	mRNA	XR_001436102.1	lncRNA
XM_012134416.2	PPIG	mRNA	XR_001436102.1	lncRNA
XM_012134416.2	PPIG	mRNA	XR_001436102.1	lncRNA
XM_012134416.2	PPIG	mRNA	XR_001436102.1	lncRNA
XM_012134416.2	PPIG	mRNA	XR_001436102.1	lncRNA
XM_012134416.2	PPIG	mRNA	XR_001436102.1	lncRNA
XM_012134416.2	PPIG	mRNA	XR_001436102.1	lncRNA
XM_012134416.2	PPIG	mRNA	XR_001436102.1	lncRNA
XM_012134416.2	PPIG	mRNA	XR_001436102.1	lncRNA
XM_012182538.2	TRAPPC3L	mRNA	XR_001436102.1	lncRNA
XM_012182538.2	TRAPPC3L	mRNA	XR_001436102.1	lncRNA
XM_012182538.2	TRAPPC3L	mRNA	XR_001436102.1	lncRNA
XM_012182538.2	TRAPPC3L	mRNA	XR_001436102.1	lncRNA
XM_012182538.2	TRAPPC3L	mRNA	XR_001436102.1	lncRNA
XM_012182538.2	TRAPPC3L	mRNA	XR_001436102.1	lncRNA
XM_012182538.2	TRAPPC3L	mRNA	XR_001436102.1	lncRNA
XM_012182538.2	TRAPPC3L	mRNA	XR_001436102.1	lncRNA
XM_012182538.2	TRAPPC3L	mRNA	XR_001436102.1	lncRNA
XM_012182538.2	TRAPPC3L	mRNA	XR_001436102.1	lncRNA
XM_012178109.2	SGTA	mRNA	XR_001024290.1	lncRNA
XM_012178109.2	SGTA	mRNA	XR_001024290.1	lncRNA
XM_012178109.2	SGTA	mRNA	XR_001024290.1	lncRNA
XM_012178109.2	SGTA	mRNA	XR_001024290.1	lncRNA
XM_012178109.2	SGTA	mRNA	XR_001024290.1	lncRNA

XM_012178109.2	SGTA	mRNA	XR_001024290.1	lncRNA
XM_012178109.2	SGTA	mRNA	XR_001024290.1	lncRNA
XM_012178109.2	SGTA	mRNA	XR_001024290.1	lncRNA
XM_012178109.2	SGTA	mRNA	XR_001024290.1	lncRNA
XM_015102039.1	SYNE3	mRNA	XR_001041211.2	lncRNA
XM_015102039.1	SYNE3	mRNA	XR_001041211.2	lncRNA
XM_015102039.1	SYNE3	mRNA	XR_001041211.2	lncRNA
XM_015102039.1	SYNE3	mRNA	XR_001041211.2	lncRNA
XM_015102039.1	SYNE3	mRNA	XR_001041211.2	lncRNA
XM_015102039.1	SYNE3	mRNA	XR_001041211.2	lncRNA
XM_015102039.1	SYNE3	mRNA	XR_001041211.2	lncRNA
XM_015102039.1	SYNE3	mRNA	XR_001041211.2	lncRNA
XM_015102039.1	SYNE3	mRNA	XR_001041211.2	lncRNA
XM_015102039.1	SYNE3	mRNA	XR_001041211.2	lncRNA
XM_012103119.1	EDRF1	mRNA	XR_001041211.2	lncRNA
XM_012103119.1	EDRF1	mRNA	XR_001041211.2	lncRNA
XM_012103119.1	EDRF1	mRNA	XR_001041211.2	lncRNA
XM_012103119.1	EDRF1	mRNA	XR_001041211.2	lncRNA
XM_012103119.1	EDRF1	mRNA	XR_001041211.2	lncRNA
XM_012103119.1	EDRF1	mRNA	XR_001041211.2	lncRNA
XM_012103119.1	EDRF1	mRNA	XR_001041211.2	lncRNA
XM_012103119.1	EDRF1	mRNA	XR_001041211.2	lncRNA
XM_012103119.1	EDRF1	mRNA	XR_001041211.2	lncRNA
XM_012103119.1	EDRF1	mRNA	XR_001041211.2	lncRNA
XM_012185846.2	MBTD1	mRNA	XR_001041211.2	lncRNA
XM_012185846.2	MBTD1	mRNA	XR_001041211.2	lncRNA
XM_012185846.2	MBTD1	mRNA	XR_001041211.2	lncRNA
XM_012185846.2	MBTD1	mRNA	XR_001041211.2	lncRNA
XM_012185846.2	MBTD1	mRNA	XR_001041211.2	lncRNA
XM_012185846.2	MBTD1	mRNA	XR_001041211.2	lncRNA
XM_012185846.2	MBTD1	mRNA	XR_001041211.2	lncRNA
XM_012185846.2	MBTD1	mRNA	XR_001041211.2	lncRNA
XM_012185846.2	MBTD1	mRNA	XR_001041211.2	lncRNA
XM_012185846.2	MBTD1	mRNA	XR_001041211.2	lncRNA
XM_015091996.1	LOC101121639	mRNA	XR_001436127.1	lncRNA
XM_015091996.1	LOC101121639	mRNA	XR_001436127.1	lncRNA
XM_015091996.1	LOC101121639	mRNA	XR_001436127.1	lncRNA
XM_015091996.1	LOC101121639	mRNA	XR_001436127.1	lncRNA
XM_015091996.1	LOC101121639	mRNA	XR_001436127.1	lncRNA
XM_015091996.1	LOC101121639	mRNA	XR_001436127.1	lncRNA
XM_015091996.1	LOC101121639	mRNA	XR_001436127.1	lncRNA
XM_015091996.1	LOC101121639	mRNA	XR_001436127.1	lncRNA
XM_015091996.1	LOC101121639	mRNA	XR_001436127.1	lncRNA
XM_015091996.1	LOC101121639	mRNA	XR_001436127.1	lncRNA
XM_015103461.1	TACC2	mRNA	XR_001044126.1	lncRNA
XM_015103461.1	TACC2	mRNA	XR_001044126.1	lncRNA
XM_015103461.1	TACC2	mRNA	XR_001044126.1	lncRNA
XM_015103461.1	TACC2	mRNA	XR_001044126.1	lncRNA
XM_015103461.1	TACC2	mRNA	XR_001044126.1	lncRNA
XM_015103461.1	TACC2	mRNA	XR_001044126.1	lncRNA
XM_015103461.1	TACC2	mRNA	XR_001044126.1	lncRNA
XM_015103461.1	TACC2	mRNA	XR_001044126.1	lncRNA
XM_015103461.1	TACC2	mRNA	XR_001044126.1	lncRNA
XM_015103461.1	TACC2	mRNA	XR_001044126.1	lncRNA

XM_015103448.1	TACC2	mRNA	XR_001025491.1	lncRNA
XM_015103448.1	TACC2	mRNA	XR_001025491.1	lncRNA
XM_015103448.1	TACC2	mRNA	XR_001025491.1	lncRNA
XM_015103448.1	TACC2	mRNA	XR_001025491.1	lncRNA
XM_015103448.1	TACC2	mRNA	XR_001025491.1	lncRNA
XM_015103448.1	TACC2	mRNA	XR_001025491.1	lncRNA
XM_015103448.1	TACC2	mRNA	XR_001025491.1	lncRNA
XM_015103448.1	TACC2	mRNA	XR_001025491.1	lncRNA
XM_015103448.1	TACC2	mRNA	XR_001025491.1	lncRNA
XM_015103448.1	TACC2	mRNA	XR_001025491.1	lncRNA
XM_012103119.1	EDRF1	mRNA	XR_001436817.1	lncRNA
XM_012103119.1	EDRF1	mRNA	XR_001436817.1	lncRNA
XM_012103119.1	EDRF1	mRNA	XR_001436817.1	lncRNA
XM_012103119.1	EDRF1	mRNA	XR_001436817.1	lncRNA
XM_012103119.1	EDRF1	mRNA	XR_001436817.1	lncRNA
XM_012103119.1	EDRF1	mRNA	XR_001436817.1	lncRNA
XM_012103119.1	EDRF1	mRNA	XR_001436817.1	lncRNA
XM_012103119.1	EDRF1	mRNA	XR_001436817.1	lncRNA
XM_012103119.1	EDRF1	mRNA	XR_001436817.1	lncRNA
XM_012103119.1	EDRF1	mRNA	XR_001436817.1	lncRNA
XM_012100690.1	KLC4	mRNA	XR_001034847.2	lncRNA
XM_012100690.1	KLC4	mRNA	XR_001034847.2	lncRNA
XM_012100690.1	KLC4	mRNA	XR_001034847.2	lncRNA
XM_012100690.1	KLC4	mRNA	XR_001034847.2	lncRNA
XM_012100690.1	KLC4	mRNA	XR_001034847.2	lncRNA
XM_012100690.1	KLC4	mRNA	XR_001034847.2	lncRNA
XM_012100690.1	KLC4	mRNA	XR_001034847.2	lncRNA
XM_012100690.1	KLC4	mRNA	XR_001034847.2	lncRNA
XM_012100690.1	KLC4	mRNA	XR_001034847.2	lncRNA
XM_012100690.1	KLC4	mRNA	XR_001034847.2	lncRNA
XM_015094203.1	RC3H2	mRNA	XR_001434202.1	lncRNA
XM_015094203.1	RC3H2	mRNA	XR_001434202.1	lncRNA
XM_015094203.1	RC3H2	mRNA	XR_001434202.1	lncRNA
XM_015094203.1	RC3H2	mRNA	XR_001434202.1	lncRNA
XM_015094203.1	RC3H2	mRNA	XR_001434202.1	lncRNA
XM_015094203.1	RC3H2	mRNA	XR_001434202.1	lncRNA
XM_015094203.1	RC3H2	mRNA	XR_001434202.1	lncRNA
XM_015094203.1	RC3H2	mRNA	XR_001434202.1	lncRNA
XM_015094203.1	RC3H2	mRNA	XR_001434202.1	lncRNA
XM_015094203.1	RC3H2	mRNA	XR_001434202.1	lncRNA
XM_012100690.1	KLC4	mRNA	XR_001434202.1	lncRNA
XM_012100690.1	KLC4	mRNA	XR_001434202.1	lncRNA
XM_012100690.1	KLC4	mRNA	XR_001434202.1	lncRNA
XM_012100690.1	KLC4	mRNA	XR_001434202.1	lncRNA
XM_012100690.1	KLC4	mRNA	XR_001434202.1	lncRNA
XM_012100690.1	KLC4	mRNA	XR_001434202.1	lncRNA
XM_012100690.1	KLC4	mRNA	XR_001434202.1	lncRNA
XM_012100690.1	KLC4	mRNA	XR_001434202.1	lncRNA
XM_012100690.1	KLC4	mRNA	XR_001434202.1	lncRNA
XM_012100690.1	KLC4	mRNA	XR_001434202.1	lncRNA
XM_015094203.1	RC3H2	mRNA	XR_001034847.2	lncRNA
XM_015094203.1	RC3H2	mRNA	XR_001034847.2	lncRNA
XM_015094203.1	RC3H2	mRNA	XR_001034847.2	lncRNA
XM_015094203.1	RC3H2	mRNA	XR_001034847.2	lncRNA

XM_015094203.1	RC3H2	mRNA	XR_001034847.2	lncRNA
XM_015094203.1	RC3H2	mRNA	XR_001034847.2	lncRNA
XM_015094203.1	RC3H2	mRNA	XR_001034847.2	lncRNA
XM_015094203.1	RC3H2	mRNA	XR_001034847.2	lncRNA
XM_015094203.1	RC3H2	mRNA	XR_001034847.2	lncRNA
XM_015094203.1	RC3H2	mRNA	XR_001034847.2	lncRNA
XM_015097170.1	NUMB	mRNA	XR_001436102.1	lncRNA
XM_015097170.1	NUMB	mRNA	XR_001436102.1	lncRNA
XM_015097170.1	NUMB	mRNA	XR_001436102.1	lncRNA
XM_015097170.1	NUMB	mRNA	XR_001436102.1	lncRNA
XM_015097170.1	NUMB	mRNA	XR_001436102.1	lncRNA
XM_015097170.1	NUMB	mRNA	XR_001436102.1	lncRNA
XM_015097170.1	NUMB	mRNA	XR_001436102.1	lncRNA
XM_015097170.1	NUMB	mRNA	XR_001436102.1	lncRNA
XM_015097170.1	NUMB	mRNA	XR_001436102.1	lncRNA
XM_015097170.1	NUMB	mRNA	XR_001436102.1	lncRNA
XM_015098618.1	DLG4	mRNA	XR_001021689.2	lncRNA
XM_015098618.1	DLG4	mRNA	XR_001021689.2	lncRNA
XM_015098618.1	DLG4	mRNA	XR_001021689.2	lncRNA
XM_015098618.1	DLG4	mRNA	XR_001021689.2	lncRNA
XM_015098618.1	DLG4	mRNA	XR_001021689.2	lncRNA
XM_015098618.1	DLG4	mRNA	XR_001021689.2	lncRNA
XM_015098618.1	DLG4	mRNA	XR_001021689.2	lncRNA
XM_015098618.1	DLG4	mRNA	XR_001021689.2	lncRNA
XM_015098618.1	DLG4	mRNA	XR_001021689.2	lncRNA
XM_015098618.1	DLG4	mRNA	XR_001021689.2	lncRNA
XM_012152472.1	LUZP1	mRNA	XR_001436102.1	lncRNA
XM_012152472.1	LUZP1	mRNA	XR_001436102.1	lncRNA
XM_012152472.1	LUZP1	mRNA	XR_001436102.1	lncRNA
XM_012152472.1	LUZP1	mRNA	XR_001436102.1	lncRNA
XM_012152472.1	LUZP1	mRNA	XR_001436102.1	lncRNA
XM_012152472.1	LUZP1	mRNA	XR_001436102.1	lncRNA
XM_012152472.1	LUZP1	mRNA	XR_001436102.1	lncRNA
XM_012152472.1	LUZP1	mRNA	XR_001436102.1	lncRNA
XM_012152472.1	LUZP1	mRNA	XR_001436102.1	lncRNA
XM_012152472.1	LUZP1	mRNA	XR_001436102.1	lncRNA
XM_012105690.2	WDR17	mRNA	XR_001021689.2	lncRNA
XM_012105690.2	WDR17	mRNA	XR_001021689.2	lncRNA
XM_012105690.2	WDR17	mRNA	XR_001021689.2	lncRNA
XM_012105690.2	WDR17	mRNA	XR_001021689.2	lncRNA
XM_012105690.2	WDR17	mRNA	XR_001021689.2	lncRNA
XM_012105690.2	WDR17	mRNA	XR_001021689.2	lncRNA
XM_012105690.2	WDR17	mRNA	XR_001021689.2	lncRNA
XM_012105690.2	WDR17	mRNA	XR_001021689.2	lncRNA
XM_012105690.2	WDR17	mRNA	XR_001021689.2	lncRNA
XM_012105690.2	WDR17	mRNA	XR_001021689.2	lncRNA
XM_012165732.1	FAM228B	mRNA	XR_001020707.2	lncRNA
XM_012165732.1	FAM228B	mRNA	XR_001020707.2	lncRNA
XM_012165732.1	FAM228B	mRNA	XR_001020707.2	lncRNA
XM_012165732.1	FAM228B	mRNA	XR_001020707.2	lncRNA
XM_012165732.1	FAM228B	mRNA	XR_001020707.2	lncRNA
XM_012165732.1	FAM228B	mRNA	XR_001020707.2	lncRNA
XM_012165732.1	FAM228B	mRNA	XR_001020707.2	lncRNA
XM_012165732.1	FAM228B	mRNA	XR_001020707.2	lncRNA

XM_012165732.1	FAM228B	mRNA	XR_001020707.2	lncRNA
XM_015103117.1	ZBTB44	mRNA	XR_001042381.2	lncRNA
XM_015103117.1	ZBTB44	mRNA	XR_001042381.2	lncRNA
XM_015103117.1	ZBTB44	mRNA	XR_001042381.2	lncRNA
XM_015103117.1	ZBTB44	mRNA	XR_001042381.2	lncRNA
XM_015103117.1	ZBTB44	mRNA	XR_001042381.2	lncRNA
XM_015103117.1	ZBTB44	mRNA	XR_001042381.2	lncRNA
XM_015103117.1	ZBTB44	mRNA	XR_001042381.2	lncRNA
XM_015103117.1	ZBTB44	mRNA	XR_001042381.2	lncRNA
XM_015103117.1	ZBTB44	mRNA	XR_001042381.2	lncRNA
XM_015103117.1	ZBTB44	mRNA	XR_001042381.2	lncRNA
XM_012143523.2	INO80D	mRNA	XR_001025491.1	lncRNA
XM_012143523.2	INO80D	mRNA	XR_001025491.1	lncRNA
XM_012143523.2	INO80D	mRNA	XR_001025491.1	lncRNA
XM_012143523.2	INO80D	mRNA	XR_001025491.1	lncRNA
XM_012143523.2	INO80D	mRNA	XR_001025491.1	lncRNA
XM_012143523.2	INO80D	mRNA	XR_001025491.1	lncRNA
XM_012143523.2	INO80D	mRNA	XR_001025491.1	lncRNA
XM_012143523.2	INO80D	mRNA	XR_001025491.1	lncRNA
XM_012143523.2	INO80D	mRNA	XR_001025491.1	lncRNA
XM_012143523.2	INO80D	mRNA	XR_001025491.1	lncRNA
XM_015094471.1	CCDC142	mRNA	XR_001434892.1	lncRNA
XM_015094471.1	CCDC142	mRNA	XR_001434892.1	lncRNA
XM_015094471.1	CCDC142	mRNA	XR_001434892.1	lncRNA
XM_015094471.1	CCDC142	mRNA	XR_001434892.1	lncRNA
XM_015094471.1	CCDC142	mRNA	XR_001434892.1	lncRNA
XM_015094471.1	CCDC142	mRNA	XR_001434892.1	lncRNA
XM_015094471.1	CCDC142	mRNA	XR_001434892.1	lncRNA
XM_015094471.1	CCDC142	mRNA	XR_001434892.1	lncRNA
XM_015094471.1	CCDC142	mRNA	XR_001434892.1	lncRNA
XM_015094471.1	CCDC142	mRNA	XR_001434892.1	lncRNA
XM_012176510.2	FNBP1L	mRNA	XR_001434761.1	lncRNA
XM_012176510.2	FNBP1L	mRNA	XR_001434761.1	lncRNA
XM_012176510.2	FNBP1L	mRNA	XR_001434761.1	lncRNA
XM_012176510.2	FNBP1L	mRNA	XR_001434761.1	lncRNA
XM_012176510.2	FNBP1L	mRNA	XR_001434761.1	lncRNA
XM_012176510.2	FNBP1L	mRNA	XR_001434761.1	lncRNA
XM_012176510.2	FNBP1L	mRNA	XR_001434761.1	lncRNA
XM_012176510.2	FNBP1L	mRNA	XR_001434761.1	lncRNA
XM_012176510.2	FNBP1L	mRNA	XR_001434761.1	lncRNA
XM_012176510.2	FNBP1L	mRNA	XR_001434761.1	lncRNA
XM_004010552.3	VPS13C	mRNA	XR_001434892.1	lncRNA
XM_004010552.3	VPS13C	mRNA	XR_001434892.1	lncRNA
XM_004010552.3	VPS13C	mRNA	XR_001434892.1	lncRNA
XM_004010552.3	VPS13C	mRNA	XR_001434892.1	lncRNA
XM_004010552.3	VPS13C	mRNA	XR_001434892.1	lncRNA
XM_004010552.3	VPS13C	mRNA	XR_001434892.1	lncRNA
XM_004010552.3	VPS13C	mRNA	XR_001434892.1	lncRNA
XM_004010552.3	VPS13C	mRNA	XR_001434892.1	lncRNA
XM_004010552.3	VPS13C	mRNA	XR_001434892.1	lncRNA
XM_004010552.3	VPS13C	mRNA	XR_001434892.1	lncRNA
XM_004006548.3	R3HDM2	mRNA	XR_001436127.1	lncRNA
XM_004006548.3	R3HDM2	mRNA	XR_001436127.1	lncRNA
XM_004006548.3	R3HDM2	mRNA	XR_001436127.1	lncRNA

XM_004006548.3	R3HDM2	mRNA	XR_001436127.1	lncRNA
XM_004006548.3	R3HDM2	mRNA	XR_001436127.1	lncRNA
XM_004006548.3	R3HDM2	mRNA	XR_001436127.1	lncRNA
XM_004006548.3	R3HDM2	mRNA	XR_001436127.1	lncRNA
XM_004006548.3	R3HDM2	mRNA	XR_001436127.1	lncRNA
XM_004006548.3	R3HDM2	mRNA	XR_001436127.1	lncRNA
XM_012096615.2	DLG1	mRNA	XR_001434761.1	lncRNA
XM_012096615.2	DLG1	mRNA	XR_001434761.1	lncRNA
XM_012096615.2	DLG1	mRNA	XR_001434761.1	lncRNA
XM_012096615.2	DLG1	mRNA	XR_001434761.1	lncRNA
XM_012096615.2	DLG1	mRNA	XR_001434761.1	lncRNA
XM_012096615.2	DLG1	mRNA	XR_001434761.1	lncRNA
XM_012096615.2	DLG1	mRNA	XR_001434761.1	lncRNA
XM_012096615.2	DLG1	mRNA	XR_001434761.1	lncRNA
XM_012096615.2	DLG1	mRNA	XR_001434761.1	lncRNA
XM_004007700.3	CLDN12	mRNA	XR_001021689.2	lncRNA
XM_004007700.3	CLDN12	mRNA	XR_001021689.2	lncRNA
XM_004007700.3	CLDN12	mRNA	XR_001021689.2	lncRNA
XM_004007700.3	CLDN12	mRNA	XR_001021689.2	lncRNA
XM_004007700.3	CLDN12	mRNA	XR_001021689.2	lncRNA
XM_004007700.3	CLDN12	mRNA	XR_001021689.2	lncRNA
XM_004007700.3	CLDN12	mRNA	XR_001021689.2	lncRNA
XM_004007700.3	CLDN12	mRNA	XR_001021689.2	lncRNA
XM_004007700.3	CLDN12	mRNA	XR_001021689.2	lncRNA
XM_015101107.1	TCP11L1	mRNA	XR_001021689.2	lncRNA
XM_015101107.1	TCP11L1	mRNA	XR_001021689.2	lncRNA
XM_015101107.1	TCP11L1	mRNA	XR_001021689.2	lncRNA
XM_015101107.1	TCP11L1	mRNA	XR_001021689.2	lncRNA
XM_015101107.1	TCP11L1	mRNA	XR_001021689.2	lncRNA
XM_015101107.1	TCP11L1	mRNA	XR_001021689.2	lncRNA
XM_015101107.1	TCP11L1	mRNA	XR_001021689.2	lncRNA
XM_015101107.1	TCP11L1	mRNA	XR_001021689.2	lncRNA
XM_015101107.1	TCP11L1	mRNA	XR_001021689.2	lncRNA
XM_015092063.1	FUBP1	mRNA	XR_001436102.1	lncRNA
XM_015092063.1	FUBP1	mRNA	XR_001436102.1	lncRNA
XM_015092063.1	FUBP1	mRNA	XR_001436102.1	lncRNA
XM_015092063.1	FUBP1	mRNA	XR_001436102.1	lncRNA
XM_015092063.1	FUBP1	mRNA	XR_001436102.1	lncRNA
XM_015092063.1	FUBP1	mRNA	XR_001436102.1	lncRNA
XM_015092063.1	FUBP1	mRNA	XR_001436102.1	lncRNA
XM_015092063.1	FUBP1	mRNA	XR_001436102.1	lncRNA
XM_015092063.1	FUBP1	mRNA	XR_001436102.1	lncRNA
XM_012181326.2	MAP4K5	mRNA	XR_001021689.2	lncRNA
XM_012181326.2	MAP4K5	mRNA	XR_001021689.2	lncRNA
XM_012181326.2	MAP4K5	mRNA	XR_001021689.2	lncRNA
XM_012181326.2	MAP4K5	mRNA	XR_001021689.2	lncRNA
XM_012181326.2	MAP4K5	mRNA	XR_001021689.2	lncRNA
XM_012181326.2	MAP4K5	mRNA	XR_001021689.2	lncRNA
XM_012181326.2	MAP4K5	mRNA	XR_001021689.2	lncRNA
XM_012181326.2	MAP4K5	mRNA	XR_001021689.2	lncRNA
XM_012181326.2	MAP4K5	mRNA	XR_001021689.2	lncRNA
XM_012190075.2	PHLPP2	mRNA	XR_001033284.2	lncRNA
XM_012190075.2	PHLPP2	mRNA	XR_001033284.2	lncRNA
XM_012190075.2	PHLPP2	mRNA	XR_001033284.2	lncRNA

XM_012190075.2	PHLPP2	mRNA	XR_001033284.2	lncRNA
XM_012190075.2	PHLPP2	mRNA	XR_001033284.2	lncRNA
XM_012190075.2	PHLPP2	mRNA	XR_001033284.2	lncRNA
XM_012190075.2	PHLPP2	mRNA	XR_001033284.2	lncRNA
XM_012190075.2	PHLPP2	mRNA	XR_001033284.2	lncRNA
XM_012190075.2	PHLPP2	mRNA	XR_001033284.2	lncRNA
XM_012175045.1	FGD4	mRNA	XR_001033284.2	lncRNA
XM_012175045.1	FGD4	mRNA	XR_001033284.2	lncRNA
XM_012175045.1	FGD4	mRNA	XR_001033284.2	lncRNA
XM_012175045.1	FGD4	mRNA	XR_001033284.2	lncRNA
XM_012175045.1	FGD4	mRNA	XR_001033284.2	lncRNA
XM_012175045.1	FGD4	mRNA	XR_001033284.2	lncRNA
XM_012175045.1	FGD4	mRNA	XR_001033284.2	lncRNA
XM_012175045.1	FGD4	mRNA	XR_001033284.2	lncRNA
XM_012175045.1	FGD4	mRNA	XR_001033284.2	lncRNA
XM_015103401.1	CALHM2	mRNA	XR_001034847.2	lncRNA
XM_015103401.1	CALHM2	mRNA	XR_001034847.2	lncRNA
XM_015103401.1	CALHM2	mRNA	XR_001034847.2	lncRNA
XM_015103401.1	CALHM2	mRNA	XR_001034847.2	lncRNA
XM_015103401.1	CALHM2	mRNA	XR_001034847.2	lncRNA
XM_015103401.1	CALHM2	mRNA	XR_001034847.2	lncRNA
XM_015103401.1	CALHM2	mRNA	XR_001034847.2	lncRNA
XM_015103401.1	CALHM2	mRNA	XR_001034847.2	lncRNA
XM_015103401.1	CALHM2	mRNA	XR_001034847.2	lncRNA
XM_015103401.1	CALHM2	mRNA	XR_001434202.1	lncRNA
XM_015103401.1	CALHM2	mRNA	XR_001434202.1	lncRNA
XM_015103401.1	CALHM2	mRNA	XR_001434202.1	lncRNA
XM_015103401.1	CALHM2	mRNA	XR_001434202.1	lncRNA
XM_015103401.1	CALHM2	mRNA	XR_001434202.1	lncRNA
XM_015103401.1	CALHM2	mRNA	XR_001434202.1	lncRNA
XM_015103401.1	CALHM2	mRNA	XR_001434202.1	lncRNA
XM_015103401.1	CALHM2	mRNA	XR_001434202.1	lncRNA
XM_015103401.1	CALHM2	mRNA	XR_001434202.1	lncRNA
XM_012181456.2	ADAM10	mRNA	TCONS_00075453	lncRNA
XM_012181456.2	ADAM10	mRNA	TCONS_00075453	lncRNA
XM_012181456.2	ADAM10	mRNA	TCONS_00075453	lncRNA
XM_012181456.2	ADAM10	mRNA	TCONS_00075453	lncRNA
XM_012181456.2	ADAM10	mRNA	TCONS_00075453	lncRNA
XM_012181456.2	ADAM10	mRNA	TCONS_00075453	lncRNA
XM_012181456.2	ADAM10	mRNA	TCONS_00075453	lncRNA
XM_012181456.2	ADAM10	mRNA	TCONS_00075453	lncRNA
XM_012181456.2	ADAM10	mRNA	TCONS_00075453	lncRNA
XM_012189779.2	RBL2	mRNA	XR_001436127.1	lncRNA
XM_012189779.2	RBL2	mRNA	XR_001436127.1	lncRNA
XM_012189779.2	RBL2	mRNA	XR_001436127.1	lncRNA
XM_012189779.2	RBL2	mRNA	XR_001436127.1	lncRNA
XM_012189779.2	RBL2	mRNA	XR_001436127.1	lncRNA
XM_012189779.2	RBL2	mRNA	XR_001436127.1	lncRNA
XM_012189779.2	RBL2	mRNA	XR_001436127.1	lncRNA
XM_012189779.2	RBL2	mRNA	XR_001436127.1	lncRNA
XM_012189779.2	RBL2	mRNA	XR_001436127.1	lncRNA
XM_012104950.1	TTC13	mRNA	XR_001434203.1	lncRNA
XM_012104950.1	TTC13	mRNA	XR_001434203.1	lncRNA
XM_012104950.1	TTC13	mRNA	XR_001434203.1	lncRNA

XM_012104950.1	TTC13	mRNA	XR_001434203.1	lncRNA
XM_012104950.1	TTC13	mRNA	XR_001434203.1	lncRNA
XM_012104950.1	TTC13	mRNA	XR_001434203.1	lncRNA
XM_012104950.1	TTC13	mRNA	XR_001434203.1	lncRNA
XM_012104950.1	TTC13	mRNA	XR_001434203.1	lncRNA
XM_012104950.1	TTC13	mRNA	XR_001434203.1	lncRNA
XM_012104950.1	TTC13	mRNA	XR_001434203.1	lncRNA
XM_015095233.1	KIAA1324L	mRNA	XR_001436532.1	lncRNA
XM_015095233.1	KIAA1324L	mRNA	XR_001436532.1	lncRNA
XM_015095233.1	KIAA1324L	mRNA	XR_001436532.1	lncRNA
XM_015095233.1	KIAA1324L	mRNA	XR_001436532.1	lncRNA
XM_015095233.1	KIAA1324L	mRNA	XR_001436532.1	lncRNA
XM_015095233.1	KIAA1324L	mRNA	XR_001436532.1	lncRNA
XM_015095233.1	KIAA1324L	mRNA	XR_001436532.1	lncRNA
XM_015095233.1	KIAA1324L	mRNA	XR_001436532.1	lncRNA
XM_015095233.1	KIAA1324L	mRNA	XR_001436532.1	lncRNA
XM_012181827.2	ZBTB1	mRNA	XR_001042381.2	lncRNA
XM_012181827.2	ZBTB1	mRNA	XR_001042381.2	lncRNA
XM_012181827.2	ZBTB1	mRNA	XR_001042381.2	lncRNA
XM_012181827.2	ZBTB1	mRNA	XR_001042381.2	lncRNA
XM_012181827.2	ZBTB1	mRNA	XR_001042381.2	lncRNA
XM_012181827.2	ZBTB1	mRNA	XR_001042381.2	lncRNA
XM_012181827.2	ZBTB1	mRNA	XR_001042381.2	lncRNA
XM_012181827.2	ZBTB1	mRNA	XR_001042381.2	lncRNA
XM_012181827.2	ZBTB1	mRNA	XR_001042381.2	lncRNA
XM_015102111.1	EIF4E3	mRNA	XR_001042381.2	lncRNA
XM_015102111.1	EIF4E3	mRNA	XR_001042381.2	lncRNA
XM_015102111.1	EIF4E3	mRNA	XR_001042381.2	lncRNA
XM_015102111.1	EIF4E3	mRNA	XR_001042381.2	lncRNA
XM_015102111.1	EIF4E3	mRNA	XR_001042381.2	lncRNA
XM_015102111.1	EIF4E3	mRNA	XR_001042381.2	lncRNA
XM_015102111.1	EIF4E3	mRNA	XR_001042381.2	lncRNA
XM_015102111.1	EIF4E3	mRNA	XR_001042381.2	lncRNA
XM_015102111.1	EIF4E3	mRNA	XR_001042381.2	lncRNA
XM_012175276.1	PLCM1	mRNA	XR_001436532.1	lncRNA
XM_012175276.1	PLCM1	mRNA	XR_001436532.1	lncRNA
XM_012175276.1	PLCM1	mRNA	XR_001436532.1	lncRNA
XM_012175276.1	PLCM1	mRNA	XR_001436532.1	lncRNA
XM_012175276.1	PLCM1	mRNA	XR_001436532.1	lncRNA
XM_012175276.1	PLCM1	mRNA	XR_001436532.1	lncRNA
XM_012175276.1	PLCM1	mRNA	XR_001436532.1	lncRNA
XM_012175276.1	PLCM1	mRNA	XR_001436532.1	lncRNA
XM_012175276.1	PLCM1	mRNA	XR_001436532.1	lncRNA
XM_015104296.1	ADAMTS14	mRNA	TCONS_00022613	lncRNA
XM_015104296.1	ADAMTS14	mRNA	TCONS_00022613	lncRNA
XM_015104296.1	ADAMTS14	mRNA	TCONS_00022613	lncRNA
XM_015104296.1	ADAMTS14	mRNA	TCONS_00022613	lncRNA
XM_015104296.1	ADAMTS14	mRNA	TCONS_00022613	lncRNA
XM_015104296.1	ADAMTS14	mRNA	TCONS_00022613	lncRNA
XM_015104296.1	ADAMTS14	mRNA	TCONS_00022613	lncRNA
XM_015104296.1	ADAMTS14	mRNA	TCONS_00022613	lncRNA
XM_015104296.1	ADAMTS14	mRNA	TCONS_00022613	lncRNA
XM_012187378.2	CAMTA1	mRNA	TCONS_00022613	lncRNA
XM_012187378.2	CAMTA1	mRNA	TCONS_00022613	lncRNA

XM_012187378.2	CAMTA1	mRNA	TCONS_00022613	lncRNA
XM_012187378.2	CAMTA1	mRNA	TCONS_00022613	lncRNA
XM_012187378.2	CAMTA1	mRNA	TCONS_00022613	lncRNA
XM_012187378.2	CAMTA1	mRNA	TCONS_00022613	lncRNA
XM_012187378.2	CAMTA1	mRNA	TCONS_00022613	lncRNA
XM_012187378.2	CAMTA1	mRNA	TCONS_00022613	lncRNA
XM_012187378.2	CAMTA1	mRNA	TCONS_00022613	lncRNA
XM_015098446.1	VEZF1	mRNA	XR_001436102.1	lncRNA
XM_015098446.1	VEZF1	mRNA	XR_001436102.1	lncRNA
XM_015098446.1	VEZF1	mRNA	XR_001436102.1	lncRNA
XM_015098446.1	VEZF1	mRNA	XR_001436102.1	lncRNA
XM_015098446.1	VEZF1	mRNA	XR_001436102.1	lncRNA
XM_015098446.1	VEZF1	mRNA	XR_001436102.1	lncRNA
XM_015098446.1	VEZF1	mRNA	XR_001436102.1	lncRNA
XM_015098446.1	VEZF1	mRNA	XR_001436102.1	lncRNA
XM_015098446.1	VEZF1	mRNA	XR_001436102.1	lncRNA
XM_015098446.1	VEZF1	mRNA	XR_001436102.1	lncRNA
XM_012098812.2	RALGAPA1	mRNA	XR_001034847.2	lncRNA
XM_012098812.2	RALGAPA1	mRNA	XR_001034847.2	lncRNA
XM_012098812.2	RALGAPA1	mRNA	XR_001034847.2	lncRNA
XM_012098812.2	RALGAPA1	mRNA	XR_001034847.2	lncRNA
XM_012098812.2	RALGAPA1	mRNA	XR_001034847.2	lncRNA
XM_012098812.2	RALGAPA1	mRNA	XR_001034847.2	lncRNA
XM_012098812.2	RALGAPA1	mRNA	XR_001034847.2	lncRNA
XM_012098812.2	RALGAPA1	mRNA	XR_001034847.2	lncRNA
XM_012098812.2	RALGAPA1	mRNA	XR_001034847.2	lncRNA
XM_012098812.2	RALGAPA1	mRNA	XR_001034847.2	lncRNA
XM_012098812.2	RALGAPA1	mRNA	XR_001434202.1	lncRNA
XM_012098812.2	RALGAPA1	mRNA	XR_001434202.1	lncRNA
XM_012098812.2	RALGAPA1	mRNA	XR_001434202.1	lncRNA
XM_012098812.2	RALGAPA1	mRNA	XR_001434202.1	lncRNA
XM_012098812.2	RALGAPA1	mRNA	XR_001434202.1	lncRNA
XM_012098812.2	RALGAPA1	mRNA	XR_001434202.1	lncRNA
XM_012098812.2	RALGAPA1	mRNA	XR_001434202.1	lncRNA
XM_012098812.2	RALGAPA1	mRNA	XR_001434202.1	lncRNA
XM_012098812.2	RALGAPA1	mRNA	XR_001434202.1	lncRNA
XM_012098812.2	RALGAPA1	mRNA	XR_001434202.1	lncRNA
XM_012183305.1	FAM120B	mRNA	XR_001021689.2	lncRNA
XM_012183305.1	FAM120B	mRNA	XR_001021689.2	lncRNA
XM_012183305.1	FAM120B	mRNA	XR_001021689.2	lncRNA
XM_012183305.1	FAM120B	mRNA	XR_001021689.2	lncRNA
XM_012183305.1	FAM120B	mRNA	XR_001021689.2	lncRNA
XM_012183305.1	FAM120B	mRNA	XR_001021689.2	lncRNA
XM_012183305.1	FAM120B	mRNA	XR_001021689.2	lncRNA
XM_012183305.1	FAM120B	mRNA	XR_001021689.2	lncRNA
XM_012183305.1	FAM120B	mRNA	XR_001021689.2	lncRNA
XM_012102658.2	ZNF518A	mRNA	XR_001436102.1	lncRNA
XM_012102658.2	ZNF518A	mRNA	XR_001436102.1	lncRNA
XM_012102658.2	ZNF518A	mRNA	XR_001436102.1	lncRNA
XM_012102658.2	ZNF518A	mRNA	XR_001436102.1	lncRNA
XM_012102658.2	ZNF518A	mRNA	XR_001436102.1	lncRNA
XM_012102658.2	ZNF518A	mRNA	XR_001436102.1	lncRNA
XM_012102658.2	ZNF518A	mRNA	XR_001436102.1	lncRNA
XM_012102658.2	ZNF518A	mRNA	XR_001436102.1	lncRNA

XM_012102658.2	ZNF518A	mRNA	XR_001436102.1	lncRNA
XM_012095858.2	ZNF214	mRNA	XR_001021689.2	lncRNA
XM_012095858.2	ZNF214	mRNA	XR_001021689.2	lncRNA
XM_012095858.2	ZNF214	mRNA	XR_001021689.2	lncRNA
XM_012095858.2	ZNF214	mRNA	XR_001021689.2	lncRNA
XM_012095858.2	ZNF214	mRNA	XR_001021689.2	lncRNA
XM_012095858.2	ZNF214	mRNA	XR_001021689.2	lncRNA
XM_012095858.2	ZNF214	mRNA	XR_001021689.2	lncRNA
XM_012095858.2	ZNF214	mRNA	XR_001021689.2	lncRNA
XM_012095858.2	ZNF214	mRNA	XR_001021689.2	lncRNA
XM_012178109.2	SGTA	mRNA	XR_001436102.1	lncRNA
XM_012178109.2	SGTA	mRNA	XR_001436102.1	lncRNA
XM_012178109.2	SGTA	mRNA	XR_001436102.1	lncRNA
XM_012178109.2	SGTA	mRNA	XR_001436102.1	lncRNA
XM_012178109.2	SGTA	mRNA	XR_001436102.1	lncRNA
XM_012178109.2	SGTA	mRNA	XR_001436102.1	lncRNA
XM_012178109.2	SGTA	mRNA	XR_001436102.1	lncRNA
XM_012178109.2	SGTA	mRNA	XR_001436102.1	lncRNA
XM_012178109.2	SGTA	mRNA	XR_001436102.1	lncRNA
XM_012102669.1	ZNF518A	mRNA	XR_001436102.1	lncRNA
XM_012102669.1	ZNF518A	mRNA	XR_001436102.1	lncRNA
XM_012102669.1	ZNF518A	mRNA	XR_001436102.1	lncRNA
XM_012102669.1	ZNF518A	mRNA	XR_001436102.1	lncRNA
XM_012102669.1	ZNF518A	mRNA	XR_001436102.1	lncRNA
XM_012102669.1	ZNF518A	mRNA	XR_001436102.1	lncRNA
XM_012102669.1	ZNF518A	mRNA	XR_001436102.1	lncRNA
XM_012102669.1	ZNF518A	mRNA	XR_001436102.1	lncRNA
XM_012102669.1	ZNF518A	mRNA	XR_001436102.1	lncRNA
XM_015098611.1	DLG4	mRNA	XR_001020707.2	lncRNA
XM_015098611.1	DLG4	mRNA	XR_001020707.2	lncRNA
XM_015098611.1	DLG4	mRNA	XR_001020707.2	lncRNA
XM_015098611.1	DLG4	mRNA	XR_001020707.2	lncRNA
XM_015098611.1	DLG4	mRNA	XR_001020707.2	lncRNA
XM_015098611.1	DLG4	mRNA	XR_001020707.2	lncRNA
XM_015098611.1	DLG4	mRNA	XR_001020707.2	lncRNA
XM_015098611.1	DLG4	mRNA	XR_001020707.2	lncRNA
XM_015098611.1	DLG4	mRNA	XR_001020707.2	lncRNA
XM_015095233.1	KIAA1324L	mRNA	XR_001041211.2	lncRNA
XM_015095233.1	KIAA1324L	mRNA	XR_001041211.2	lncRNA
XM_015095233.1	KIAA1324L	mRNA	XR_001041211.2	lncRNA
XM_015095233.1	KIAA1324L	mRNA	XR_001041211.2	lncRNA
XM_015095233.1	KIAA1324L	mRNA	XR_001041211.2	lncRNA
XM_015095233.1	KIAA1324L	mRNA	XR_001041211.2	lncRNA
XM_015095233.1	KIAA1324L	mRNA	XR_001041211.2	lncRNA
XM_015095233.1	KIAA1324L	mRNA	XR_001041211.2	lncRNA
XM_015095233.1	KIAA1324L	mRNA	XR_001041211.2	lncRNA
XM_012183566.1	FAM49B	mRNA	XR_001041211.2	lncRNA
XM_012183566.1	FAM49B	mRNA	XR_001041211.2	lncRNA
XM_012183566.1	FAM49B	mRNA	XR_001041211.2	lncRNA
XM_012183566.1	FAM49B	mRNA	XR_001041211.2	lncRNA
XM_012183566.1	FAM49B	mRNA	XR_001041211.2	lncRNA
XM_012183566.1	FAM49B	mRNA	XR_001041211.2	lncRNA
XM_012183566.1	FAM49B	mRNA	XR_001041211.2	lncRNA
XM_012183566.1	FAM49B	mRNA	XR_001041211.2	lncRNA

XM_012183566.1	FAM49B	mRNA	XR_001041211.2	lncRNA
XM_012178109.2	SGTA	mRNA	TCONS_00022613	lncRNA
XM_012178109.2	SGTA	mRNA	TCONS_00022613	lncRNA
XM_012178109.2	SGTA	mRNA	TCONS_00022613	lncRNA
XM_012178109.2	SGTA	mRNA	TCONS_00022613	lncRNA
XM_012178109.2	SGTA	mRNA	TCONS_00022613	lncRNA
XM_012178109.2	SGTA	mRNA	TCONS_00022613	lncRNA
XM_012178109.2	SGTA	mRNA	TCONS_00022613	lncRNA
XM_012178109.2	SGTA	mRNA	TCONS_00022613	lncRNA
XM_012182538.2	TRAPPC3L	mRNA	XR_001024290.1	lncRNA
XM_012182538.2	TRAPPC3L	mRNA	XR_001024290.1	lncRNA
XM_012182538.2	TRAPPC3L	mRNA	XR_001024290.1	lncRNA
XM_012182538.2	TRAPPC3L	mRNA	XR_001024290.1	lncRNA
XM_012182538.2	TRAPPC3L	mRNA	XR_001024290.1	lncRNA
XM_012182538.2	TRAPPC3L	mRNA	XR_001024290.1	lncRNA
XM_012182538.2	TRAPPC3L	mRNA	XR_001024290.1	lncRNA
XM_012182538.2	TRAPPC3L	mRNA	XR_001024290.1	lncRNA
XM_012182538.2	TRAPPC3L	mRNA	XR_001024290.1	lncRNA
XM_012097367.2	SH3D19	mRNA	XR_001434892.1	lncRNA
XM_012097367.2	SH3D19	mRNA	XR_001434892.1	lncRNA
XM_012097367.2	SH3D19	mRNA	XR_001434892.1	lncRNA
XM_012097367.2	SH3D19	mRNA	XR_001434892.1	lncRNA
XM_012097367.2	SH3D19	mRNA	XR_001434892.1	lncRNA
XM_012097367.2	SH3D19	mRNA	XR_001434892.1	lncRNA
XM_012097367.2	SH3D19	mRNA	XR_001434892.1	lncRNA
XM_012097367.2	SH3D19	mRNA	XR_001434892.1	lncRNA
XM_012097367.2	SH3D19	mRNA	XR_001434892.1	lncRNA
XM_015092063.1	FUBP1	mRNA	XR_001434761.1	lncRNA
XM_015092063.1	FUBP1	mRNA	XR_001434761.1	lncRNA
XM_015092063.1	FUBP1	mRNA	XR_001434761.1	lncRNA
XM_015092063.1	FUBP1	mRNA	XR_001434761.1	lncRNA
XM_015092063.1	FUBP1	mRNA	XR_001434761.1	lncRNA
XM_015092063.1	FUBP1	mRNA	XR_001434761.1	lncRNA
XM_015092063.1	FUBP1	mRNA	XR_001434761.1	lncRNA
XM_015092063.1	FUBP1	mRNA	XR_001434761.1	lncRNA
XM_015092063.1	FUBP1	mRNA	XR_001434761.1	lncRNA
XM_015102111.1	EIF4E3	mRNA	XR_001436817.1	lncRNA
XM_015102111.1	EIF4E3	mRNA	XR_001436817.1	lncRNA
XM_015102111.1	EIF4E3	mRNA	XR_001436817.1	lncRNA
XM_015102111.1	EIF4E3	mRNA	XR_001436817.1	lncRNA
XM_015102111.1	EIF4E3	mRNA	XR_001436817.1	lncRNA
XM_015102111.1	EIF4E3	mRNA	XR_001436817.1	lncRNA
XM_015102111.1	EIF4E3	mRNA	XR_001436817.1	lncRNA
XM_015102111.1	EIF4E3	mRNA	XR_001436817.1	lncRNA
XM_015102111.1	EIF4E3	mRNA	XR_001436817.1	lncRNA
XM_015094913.1	PLCM1	mRNA	XR_001434203.1	lncRNA
XM_015094913.1	PLCM1	mRNA	XR_001434203.1	lncRNA
XM_015094913.1	PLCM1	mRNA	XR_001434203.1	lncRNA
XM_015094913.1	PLCM1	mRNA	XR_001434203.1	lncRNA
XM_015094913.1	PLCM1	mRNA	XR_001434203.1	lncRNA
XM_015094913.1	PLCM1	mRNA	XR_001434203.1	lncRNA
XM_015094913.1	PLCM1	mRNA	XR_001434203.1	lncRNA
XM_015094913.1	PLCM1	mRNA	XR_001434203.1	lncRNA
XM_015094913.1	PLCM1	mRNA	XR_001434203.1	lncRNA

XM_015098136.1	PDS5B	mRNA	XR_001436817.1	lncRNA
XM_015098136.1	PDS5B	mRNA	XR_001436817.1	lncRNA
XM_015098136.1	PDS5B	mRNA	XR_001436817.1	lncRNA
XM_015098136.1	PDS5B	mRNA	XR_001436817.1	lncRNA
XM_015098136.1	PDS5B	mRNA	XR_001436817.1	lncRNA
XM_015098136.1	PDS5B	mRNA	XR_001436817.1	lncRNA
XM_015098136.1	PDS5B	mRNA	XR_001436817.1	lncRNA
XM_015098136.1	PDS5B	mRNA	XR_001436817.1	lncRNA
XM_015098136.1	PDS5B	mRNA	XR_001436817.1	lncRNA
XM_012180705.1	PAPD4	mRNA	XR_001436817.1	lncRNA
XM_012180705.1	PAPD4	mRNA	XR_001436817.1	lncRNA
XM_012180705.1	PAPD4	mRNA	XR_001436817.1	lncRNA
XM_012180705.1	PAPD4	mRNA	XR_001436817.1	lncRNA
XM_012180705.1	PAPD4	mRNA	XR_001436817.1	lncRNA
XM_012180705.1	PAPD4	mRNA	XR_001436817.1	lncRNA
XM_012180705.1	PAPD4	mRNA	XR_001436817.1	lncRNA
XM_012180705.1	PAPD4	mRNA	XR_001436817.1	lncRNA
XM_012180705.1	PAPD4	mRNA	XR_001436817.1	lncRNA
XM_012104566.2	ZKSCAN1	mRNA	XR_001436817.1	lncRNA
XM_012104566.2	ZKSCAN1	mRNA	XR_001436817.1	lncRNA
XM_012104566.2	ZKSCAN1	mRNA	XR_001436817.1	lncRNA
XM_012104566.2	ZKSCAN1	mRNA	XR_001436817.1	lncRNA
XM_012104566.2	ZKSCAN1	mRNA	XR_001436817.1	lncRNA
XM_012104566.2	ZKSCAN1	mRNA	XR_001436817.1	lncRNA
XM_012104566.2	ZKSCAN1	mRNA	XR_001436817.1	lncRNA
XM_012104566.2	ZKSCAN1	mRNA	XR_001436817.1	lncRNA
XM_012104566.2	ZKSCAN1	mRNA	XR_001436817.1	lncRNA
XM_012104566.2	ZKSCAN1	mRNA	XR_001434203.1	lncRNA
XM_012104566.2	ZKSCAN1	mRNA	XR_001434203.1	lncRNA
XM_012104566.2	ZKSCAN1	mRNA	XR_001434203.1	lncRNA
XM_012104566.2	ZKSCAN1	mRNA	XR_001434203.1	lncRNA
XM_012104566.2	ZKSCAN1	mRNA	XR_001434203.1	lncRNA
XM_012104566.2	ZKSCAN1	mRNA	XR_001434203.1	lncRNA
XM_012104566.2	ZKSCAN1	mRNA	XR_001434203.1	lncRNA
XM_012104566.2	ZKSCAN1	mRNA	XR_001434203.1	lncRNA
XM_012104566.2	ZKSCAN1	mRNA	XR_001434203.1	lncRNA
XM_012176829.1	BBS9	mRNA	XR_001436817.1	lncRNA
XM_012176829.1	BBS9	mRNA	XR_001436817.1	lncRNA
XM_012176829.1	BBS9	mRNA	XR_001436817.1	lncRNA
XM_012176829.1	BBS9	mRNA	XR_001436817.1	lncRNA
XM_012176829.1	BBS9	mRNA	XR_001436817.1	lncRNA
XM_012176829.1	BBS9	mRNA	XR_001436817.1	lncRNA
XM_012176829.1	BBS9	mRNA	XR_001436817.1	lncRNA
XM_012176829.1	BBS9	mRNA	XR_001436817.1	lncRNA
XM_012176829.1	BBS9	mRNA	XR_001436817.1	lncRNA
XM_015102111.1	EIF4E3	mRNA	XR_001434203.1	lncRNA
XM_015102111.1	EIF4E3	mRNA	XR_001434203.1	lncRNA
XM_015102111.1	EIF4E3	mRNA	XR_001434203.1	lncRNA
XM_015102111.1	EIF4E3	mRNA	XR_001434203.1	lncRNA
XM_015102111.1	EIF4E3	mRNA	XR_001434203.1	lncRNA
XM_015102111.1	EIF4E3	mRNA	XR_001434203.1	lncRNA
XM_015102111.1	EIF4E3	mRNA	XR_001434203.1	lncRNA
XM_015102111.1	EIF4E3	mRNA	XR_001434203.1	lncRNA
XM_015102111.1	EIF4E3	mRNA	XR_001434203.1	lncRNA

XM_012174704.2	ZBTB39	mRNA	XR_001436102.1	lncRNA
XM_012174704.2	ZBTB39	mRNA	XR_001436102.1	lncRNA
XM_012174704.2	ZBTB39	mRNA	XR_001436102.1	lncRNA
XM_012174704.2	ZBTB39	mRNA	XR_001436102.1	lncRNA
XM_012174704.2	ZBTB39	mRNA	XR_001436102.1	lncRNA
XM_012174704.2	ZBTB39	mRNA	XR_001436102.1	lncRNA
XM_012174704.2	ZBTB39	mRNA	XR_001436102.1	lncRNA
XM_012174704.2	ZBTB39	mRNA	XR_001436102.1	lncRNA
XM_012174704.2	ZBTB39	mRNA	XR_001436102.1	lncRNA
XM_012174704.2	ZBTB39	mRNA	XR_001436102.1	lncRNA
XM_012174704.2	ZBTB39	mRNA	XR_001021689.2	lncRNA
XM_012174704.2	ZBTB39	mRNA	XR_001021689.2	lncRNA
XM_012174704.2	ZBTB39	mRNA	XR_001021689.2	lncRNA
XM_012174704.2	ZBTB39	mRNA	XR_001021689.2	lncRNA
XM_012174704.2	ZBTB39	mRNA	XR_001021689.2	lncRNA
XM_012174704.2	ZBTB39	mRNA	XR_001021689.2	lncRNA
XM_012174704.2	ZBTB39	mRNA	XR_001021689.2	lncRNA
XM_012174704.2	ZBTB39	mRNA	XR_001021689.2	lncRNA
XM_012174704.2	ZBTB39	mRNA	XR_001021689.2	lncRNA
XM_012174704.2	ZBTB39	mRNA	XR_001021689.2	lncRNA
XM_012105690.2	WDR17	mRNA	XR_001434892.1	lncRNA
XM_012105690.2	WDR17	mRNA	XR_001434892.1	lncRNA
XM_012105690.2	WDR17	mRNA	XR_001434892.1	lncRNA
XM_012105690.2	WDR17	mRNA	XR_001434892.1	lncRNA
XM_012105690.2	WDR17	mRNA	XR_001434892.1	lncRNA
XM_012105690.2	WDR17	mRNA	XR_001434892.1	lncRNA
XM_012105690.2	WDR17	mRNA	XR_001434892.1	lncRNA
XM_012105690.2	WDR17	mRNA	XR_001434892.1	lncRNA
XM_012105690.2	WDR17	mRNA	XR_001434892.1	lncRNA
XM_012105690.2	WDR17	mRNA	XR_001434892.1	lncRNA
XM_012178109.2	SGTA	mRNA	XR_001434202.1	lncRNA
XM_012178109.2	SGTA	mRNA	XR_001434202.1	lncRNA
XM_012178109.2	SGTA	mRNA	XR_001434202.1	lncRNA
XM_012178109.2	SGTA	mRNA	XR_001434202.1	lncRNA
XM_012178109.2	SGTA	mRNA	XR_001434202.1	lncRNA
XM_012178109.2	SGTA	mRNA	XR_001434202.1	lncRNA
XM_012178109.2	SGTA	mRNA	XR_001434202.1	lncRNA
XM_012178109.2	SGTA	mRNA	XR_001434202.1	lncRNA
XM_012178109.2	SGTA	mRNA	XR_001434202.1	lncRNA
XM_012168955.2	DNAJC6	mRNA	XR_001434202.1	lncRNA
XM_012168955.2	DNAJC6	mRNA	XR_001434202.1	lncRNA
XM_012168955.2	DNAJC6	mRNA	XR_001434202.1	lncRNA
XM_012168955.2	DNAJC6	mRNA	XR_001434202.1	lncRNA
XM_012168955.2	DNAJC6	mRNA	XR_001434202.1	lncRNA
XM_012168955.2	DNAJC6	mRNA	XR_001434202.1	lncRNA
XM_012168955.2	DNAJC6	mRNA	XR_001434202.1	lncRNA
XM_012168955.2	DNAJC6	mRNA	XR_001434202.1	lncRNA
XM_012168955.2	DNAJC6	mRNA	XR_001434202.1	lncRNA
XM_012168955.2	DNAJC6	mRNA	XR_001034847.2	lncRNA
XM_012168955.2	DNAJC6	mRNA	XR_001034847.2	lncRNA
XM_012168955.2	DNAJC6	mRNA	XR_001034847.2	lncRNA
XM_012168955.2	DNAJC6	mRNA	XR_001034847.2	lncRNA
XM_012168955.2	DNAJC6	mRNA	XR_001034847.2	lncRNA
XM_012168955.2	DNAJC6	mRNA	XR_001034847.2	lncRNA

XM_012168955.2	DNAJC6	mRNA	XR_001034847.2	lncRNA
XM_012168955.2	DNAJC6	mRNA	XR_001034847.2	lncRNA
XM_012168955.2	DNAJC6	mRNA	XR_001034847.2	lncRNA
XM_012178109.2	SGTA	mRNA	XR_001034847.2	lncRNA
XM_012178109.2	SGTA	mRNA	XR_001034847.2	lncRNA
XM_012178109.2	SGTA	mRNA	XR_001034847.2	lncRNA
XM_012178109.2	SGTA	mRNA	XR_001034847.2	lncRNA
XM_012178109.2	SGTA	mRNA	XR_001034847.2	lncRNA
XM_012178109.2	SGTA	mRNA	XR_001034847.2	lncRNA
XM_012178109.2	SGTA	mRNA	XR_001034847.2	lncRNA
XM_012178109.2	SGTA	mRNA	XR_001034847.2	lncRNA
XM_012178109.2	SGTA	mRNA	XR_001034847.2	lncRNA
XM_012102834.2	PPRC1	mRNA	XR_001434202.1	lncRNA
XM_012102834.2	PPRC1	mRNA	XR_001434202.1	lncRNA
XM_012102834.2	PPRC1	mRNA	XR_001434202.1	lncRNA
XM_012102834.2	PPRC1	mRNA	XR_001434202.1	lncRNA
XM_012102834.2	PPRC1	mRNA	XR_001434202.1	lncRNA
XM_012102834.2	PPRC1	mRNA	XR_001434202.1	lncRNA
XM_012102834.2	PPRC1	mRNA	XR_001434202.1	lncRNA
XM_012102834.2	PPRC1	mRNA	XR_001434202.1	lncRNA
XM_012102834.2	PPRC1	mRNA	XR_001434202.1	lncRNA
XM_012102834.2	PPRC1	mRNA	XR_001034847.2	lncRNA
XM_012102834.2	PPRC1	mRNA	XR_001034847.2	lncRNA
XM_012102834.2	PPRC1	mRNA	XR_001034847.2	lncRNA
XM_012102834.2	PPRC1	mRNA	XR_001034847.2	lncRNA
XM_012102834.2	PPRC1	mRNA	XR_001034847.2	lncRNA
XM_012102834.2	PPRC1	mRNA	XR_001034847.2	lncRNA
XM_012102834.2	PPRC1	mRNA	XR_001034847.2	lncRNA
XM_012102834.2	PPRC1	mRNA	XR_001034847.2	lncRNA
XM_012102834.2	PPRC1	mRNA	XR_001034847.2	lncRNA
XM_015103466.1	TACC2	mRNA	TCONS_00075453	lncRNA
XM_015103466.1	TACC2	mRNA	TCONS_00075453	lncRNA
XM_015103466.1	TACC2	mRNA	TCONS_00075453	lncRNA
XM_015103466.1	TACC2	mRNA	TCONS_00075453	lncRNA
XM_015103466.1	TACC2	mRNA	TCONS_00075453	lncRNA
XM_015103466.1	TACC2	mRNA	TCONS_00075453	lncRNA
XM_015103466.1	TACC2	mRNA	TCONS_00075453	lncRNA
XM_015103466.1	TACC2	mRNA	TCONS_00075453	lncRNA
XM_015103466.1	TACC2	mRNA	TCONS_00075453	lncRNA
XM_012177947.2	INSR	mRNA	XR_001436102.1	lncRNA
XM_012177947.2	INSR	mRNA	XR_001436102.1	lncRNA
XM_012177947.2	INSR	mRNA	XR_001436102.1	lncRNA
XM_012177947.2	INSR	mRNA	XR_001436102.1	lncRNA
XM_012177947.2	INSR	mRNA	XR_001436102.1	lncRNA
XM_012177947.2	INSR	mRNA	XR_001436102.1	lncRNA
XM_012177947.2	INSR	mRNA	XR_001436102.1	lncRNA
XM_012177947.2	INSR	mRNA	XR_001436102.1	lncRNA
XM_012177947.2	INSR	mRNA	XR_001436102.1	lncRNA
XM_012177947.2	INSR	mRNA	XR_001436102.1	lncRNA
XM_012184887.2	DOCK9	mRNA	XR_001044126.1	lncRNA
XM_012184887.2	DOCK9	mRNA	XR_001044126.1	lncRNA
XM_012184887.2	DOCK9	mRNA	XR_001044126.1	lncRNA
XM_012184887.2	DOCK9	mRNA	XR_001044126.1	lncRNA
XM_012184887.2	DOCK9	mRNA	XR_001044126.1	lncRNA

XM_012184887.2	DOCK9	mRNA	XR_001044126.1	lncRNA
XM_012184887.2	DOCK9	mRNA	XR_001044126.1	lncRNA
XM_012184887.2	DOCK9	mRNA	XR_001044126.1	lncRNA
XM_012184887.2	DOCK9	mRNA	XR_001044126.1	lncRNA
XM_004006548.3	R3HDM2	mRNA	XR_001021689.2	lncRNA
XM_004006548.3	R3HDM2	mRNA	XR_001021689.2	lncRNA
XM_004006548.3	R3HDM2	mRNA	XR_001021689.2	lncRNA
XM_004006548.3	R3HDM2	mRNA	XR_001021689.2	lncRNA
XM_004006548.3	R3HDM2	mRNA	XR_001021689.2	lncRNA
XM_004006548.3	R3HDM2	mRNA	XR_001021689.2	lncRNA
XM_004006548.3	R3HDM2	mRNA	XR_001021689.2	lncRNA
XM_004006548.3	R3HDM2	mRNA	XR_001021689.2	lncRNA
XM_004006548.3	R3HDM2	mRNA	XR_001021689.2	lncRNA
XM_015096638.1	CC2L1A	mRNA	XR_001021689.2	lncRNA
XM_015096638.1	CC2L1A	mRNA	XR_001021689.2	lncRNA
XM_015096638.1	CC2L1A	mRNA	XR_001021689.2	lncRNA
XM_015096638.1	CC2L1A	mRNA	XR_001021689.2	lncRNA
XM_015096638.1	CC2L1A	mRNA	XR_001021689.2	lncRNA
XM_015096638.1	CC2L1A	mRNA	XR_001021689.2	lncRNA
XM_015096638.1	CC2L1A	mRNA	XR_001021689.2	lncRNA
XM_015096638.1	CC2L1A	mRNA	XR_001021689.2	lncRNA
XM_015096638.1	CC2L1A	mRNA	XR_001021689.2	lncRNA
XM_012097610.2	ZNF605	mRNA	XR_001436102.1	lncRNA
XM_012097610.2	ZNF605	mRNA	XR_001436102.1	lncRNA
XM_012097610.2	ZNF605	mRNA	XR_001436102.1	lncRNA
XM_012097610.2	ZNF605	mRNA	XR_001436102.1	lncRNA
XM_012097610.2	ZNF605	mRNA	XR_001436102.1	lncRNA
XM_012097610.2	ZNF605	mRNA	XR_001436102.1	lncRNA
XM_012097610.2	ZNF605	mRNA	XR_001436102.1	lncRNA
XM_012097610.2	ZNF605	mRNA	XR_001436102.1	lncRNA
XM_012097610.2	ZNF605	mRNA	XR_001436102.1	lncRNA
XM_012096925.2	SLC38A9	mRNA	XR_001436102.1	lncRNA
XM_012096925.2	SLC38A9	mRNA	XR_001436102.1	lncRNA
XM_012096925.2	SLC38A9	mRNA	XR_001436102.1	lncRNA
XM_012096925.2	SLC38A9	mRNA	XR_001436102.1	lncRNA
XM_012096925.2	SLC38A9	mRNA	XR_001436102.1	lncRNA
XM_012096925.2	SLC38A9	mRNA	XR_001436102.1	lncRNA
XM_012096925.2	SLC38A9	mRNA	XR_001436102.1	lncRNA
XM_012096925.2	SLC38A9	mRNA	XR_001436102.1	lncRNA
XM_012096925.2	SLC38A9	mRNA	XR_001436102.1	lncRNA
XM_012151052.2	WASF2	mRNA	XR_001021689.2	lncRNA
XM_012151052.2	WASF2	mRNA	XR_001021689.2	lncRNA
XM_012151052.2	WASF2	mRNA	XR_001021689.2	lncRNA
XM_012151052.2	WASF2	mRNA	XR_001021689.2	lncRNA
XM_012151052.2	WASF2	mRNA	XR_001021689.2	lncRNA
XM_012151052.2	WASF2	mRNA	XR_001021689.2	lncRNA
XM_012151052.2	WASF2	mRNA	XR_001021689.2	lncRNA
XM_012151052.2	WASF2	mRNA	XR_001021689.2	lncRNA
XM_012151052.2	WASF2	mRNA	XR_001021689.2	lncRNA
XM_015098660.1	MAP2K4	mRNA	XR_001436102.1	lncRNA
XM_015098660.1	MAP2K4	mRNA	XR_001436102.1	lncRNA
XM_015098660.1	MAP2K4	mRNA	XR_001436102.1	lncRNA
XM_015098660.1	MAP2K4	mRNA	XR_001436102.1	lncRNA
XM_015098660.1	MAP2K4	mRNA	XR_001436102.1	lncRNA

XM_015098660.1	MAP2K4	mRNA	XR_001436102.1	lncRNA
XM_015098660.1	MAP2K4	mRNA	XR_001436102.1	lncRNA
XM_015098660.1	MAP2K4	mRNA	XR_001436102.1	lncRNA
XM_015098660.1	MAP2K4	mRNA	XR_001436102.1	lncRNA
XM_012182238.1	SPG11	mRNA	XR_001436102.1	lncRNA
XM_012182238.1	SPG11	mRNA	XR_001436102.1	lncRNA
XM_012182238.1	SPG11	mRNA	XR_001436102.1	lncRNA
XM_012182238.1	SPG11	mRNA	XR_001436102.1	lncRNA
XM_012182238.1	SPG11	mRNA	XR_001436102.1	lncRNA
XM_012182238.1	SPG11	mRNA	XR_001436102.1	lncRNA
XM_012182238.1	SPG11	mRNA	XR_001436102.1	lncRNA
XM_012182238.1	SPG11	mRNA	XR_001436102.1	lncRNA
XM_012182238.1	SPG11	mRNA	XR_001436102.1	lncRNA
XM_015098660.1	MAP2K4	mRNA	TCONS_00022613	lncRNA
XM_015098660.1	MAP2K4	mRNA	TCONS_00022613	lncRNA
XM_015098660.1	MAP2K4	mRNA	TCONS_00022613	lncRNA
XM_015098660.1	MAP2K4	mRNA	TCONS_00022613	lncRNA
XM_015098660.1	MAP2K4	mRNA	TCONS_00022613	lncRNA
XM_015098660.1	MAP2K4	mRNA	TCONS_00022613	lncRNA
XM_015098660.1	MAP2K4	mRNA	TCONS_00022613	lncRNA
XM_015098660.1	MAP2K4	mRNA	TCONS_00022613	lncRNA
XM_012182450.2	IBTK	mRNA	XR_001436532.1	lncRNA
XM_012182450.2	IBTK	mRNA	XR_001436532.1	lncRNA
XM_012182450.2	IBTK	mRNA	XR_001436532.1	lncRNA
XM_012182450.2	IBTK	mRNA	XR_001436532.1	lncRNA
XM_012182450.2	IBTK	mRNA	XR_001436532.1	lncRNA
XM_012182450.2	IBTK	mRNA	XR_001436532.1	lncRNA
XM_012182450.2	IBTK	mRNA	XR_001436532.1	lncRNA
XM_012182450.2	IBTK	mRNA	XR_001436532.1	lncRNA
XM_012182450.2	IBTK	mRNA	XR_001436532.1	lncRNA
XM_012181326.2	MAP4K5	mRNA	XR_001042381.2	lncRNA
XM_012181326.2	MAP4K5	mRNA	XR_001042381.2	lncRNA
XM_012181326.2	MAP4K5	mRNA	XR_001042381.2	lncRNA
XM_012181326.2	MAP4K5	mRNA	XR_001042381.2	lncRNA
XM_012181326.2	MAP4K5	mRNA	XR_001042381.2	lncRNA
XM_012181326.2	MAP4K5	mRNA	XR_001042381.2	lncRNA
XM_012181326.2	MAP4K5	mRNA	XR_001042381.2	lncRNA
XM_012181326.2	MAP4K5	mRNA	XR_001042381.2	lncRNA
XM_012181326.2	MAP4K5	mRNA	XR_001042381.2	lncRNA
XM_012104225.2	DCUN1D3	mRNA	XR_001021689.2	lncRNA
XM_012104225.2	DCUN1D3	mRNA	XR_001021689.2	lncRNA
XM_012104225.2	DCUN1D3	mRNA	XR_001021689.2	lncRNA
XM_012104225.2	DCUN1D3	mRNA	XR_001021689.2	lncRNA
XM_012104225.2	DCUN1D3	mRNA	XR_001021689.2	lncRNA
XM_012104225.2	DCUN1D3	mRNA	XR_001021689.2	lncRNA
XM_012104225.2	DCUN1D3	mRNA	XR_001021689.2	lncRNA
XM_012104225.2	DCUN1D3	mRNA	XR_001021689.2	lncRNA
XM_012184456.2	FNDC3A	mRNA	XR_001021689.2	lncRNA
XM_012184456.2	FNDC3A	mRNA	XR_001021689.2	lncRNA
XM_012184456.2	FNDC3A	mRNA	XR_001021689.2	lncRNA
XM_012184456.2	FNDC3A	mRNA	XR_001021689.2	lncRNA
XM_012184456.2	FNDC3A	mRNA	XR_001021689.2	lncRNA
XM_012184456.2	FNDC3A	mRNA	XR_001021689.2	lncRNA
XM_012184456.2	FNDC3A	mRNA	XR_001021689.2	lncRNA

XM_012184456.2	FNDC3A	mRNA	XR_001021689.2	lncRNA
XM_012184401.1	ZC3H13	mRNA	XR_001020707.2	lncRNA
XM_012184401.1	ZC3H13	mRNA	XR_001020707.2	lncRNA
XM_012184401.1	ZC3H13	mRNA	XR_001020707.2	lncRNA
XM_012184401.1	ZC3H13	mRNA	XR_001020707.2	lncRNA
XM_012184401.1	ZC3H13	mRNA	XR_001020707.2	lncRNA
XM_012184401.1	ZC3H13	mRNA	XR_001020707.2	lncRNA
XM_012184401.1	ZC3H13	mRNA	XR_001020707.2	lncRNA
XM_012184401.1	ZC3H13	mRNA	XR_001020707.2	lncRNA
XM_012177920.2	KANK2	mRNA	XR_001436127.1	lncRNA
XM_012177920.2	KANK2	mRNA	XR_001436127.1	lncRNA
XM_012177920.2	KANK2	mRNA	XR_001436127.1	lncRNA
XM_012177920.2	KANK2	mRNA	XR_001436127.1	lncRNA
XM_012177920.2	KANK2	mRNA	XR_001436127.1	lncRNA
XM_012177920.2	KANK2	mRNA	XR_001436127.1	lncRNA
XM_012177920.2	KANK2	mRNA	XR_001436127.1	lncRNA
XM_012177920.2	KANK2	mRNA	XR_001436127.1	lncRNA
XM_012177920.2	KANK2	mRNA	XR_001436127.1	lncRNA
XM_012097254.2	RHOF	mRNA	XR_001434202.1	lncRNA
XM_012097254.2	RHOF	mRNA	XR_001434202.1	lncRNA
XM_012097254.2	RHOF	mRNA	XR_001434202.1	lncRNA
XM_012097254.2	RHOF	mRNA	XR_001434202.1	lncRNA
XM_012097254.2	RHOF	mRNA	XR_001434202.1	lncRNA
XM_012097254.2	RHOF	mRNA	XR_001434202.1	lncRNA
XM_012097254.2	RHOF	mRNA	XR_001434202.1	lncRNA
XM_012097254.2	RHOF	mRNA	XR_001434202.1	lncRNA
XM_012097254.2	RHOF	mRNA	XR_001434202.1	lncRNA
XM_012097254.2	RHOF	mRNA	XR_001034847.2	lncRNA
XM_012097254.2	RHOF	mRNA	XR_001034847.2	lncRNA
XM_012097254.2	RHOF	mRNA	XR_001034847.2	lncRNA
XM_012097254.2	RHOF	mRNA	XR_001034847.2	lncRNA
XM_012097254.2	RHOF	mRNA	XR_001034847.2	lncRNA
XM_012097254.2	RHOF	mRNA	XR_001034847.2	lncRNA
XM_012097254.2	RHOF	mRNA	XR_001034847.2	lncRNA
XM_012097254.2	RHOF	mRNA	XR_001034847.2	lncRNA
XM_012097254.2	RHOF	mRNA	XR_001034847.2	lncRNA
XM_012190630.2	PLEKHA4	mRNA	XR_001434761.1	lncRNA
XM_012190630.2	PLEKHA4	mRNA	XR_001434761.1	lncRNA
XM_012190630.2	PLEKHA4	mRNA	XR_001434761.1	lncRNA
XM_012190630.2	PLEKHA4	mRNA	XR_001434761.1	lncRNA
XM_012190630.2	PLEKHA4	mRNA	XR_001434761.1	lncRNA
XM_012190630.2	PLEKHA4	mRNA	XR_001434761.1	lncRNA
XM_012190630.2	PLEKHA4	mRNA	XR_001434761.1	lncRNA
XM_012190630.2	PLEKHA4	mRNA	XR_001434761.1	lncRNA
XM_012190630.2	PLEKHA4	mRNA	XR_001434761.1	lncRNA
XM_012187432.1	PRKCZ	mRNA	XR_001434892.1	lncRNA
XM_012187432.1	PRKCZ	mRNA	XR_001434892.1	lncRNA
XM_012187432.1	PRKCZ	mRNA	XR_001434892.1	lncRNA
XM_012187432.1	PRKCZ	mRNA	XR_001434892.1	lncRNA
XM_012187432.1	PRKCZ	mRNA	XR_001434892.1	lncRNA
XM_012187432.1	PRKCZ	mRNA	XR_001434892.1	lncRNA
XM_012187432.1	PRKCZ	mRNA	XR_001434892.1	lncRNA
XM_012187432.1	PRKCZ	mRNA	XR_001434892.1	lncRNA
XM_012187432.1	PRKCZ	mRNA	XR_001434892.1	lncRNA

XM_012102658.2	ZNF518A	mRNA	XR_001434761.1	lncRNA
XM_012102658.2	ZNF518A	mRNA	XR_001434761.1	lncRNA
XM_012102658.2	ZNF518A	mRNA	XR_001434761.1	lncRNA
XM_012102658.2	ZNF518A	mRNA	XR_001434761.1	lncRNA
XM_012102658.2	ZNF518A	mRNA	XR_001434761.1	lncRNA
XM_012102658.2	ZNF518A	mRNA	XR_001434761.1	lncRNA
XM_012102658.2	ZNF518A	mRNA	XR_001434761.1	lncRNA
XM_012102658.2	ZNF518A	mRNA	XR_001434761.1	lncRNA
XM_012102658.2	ZNF518A	mRNA	XR_001434761.1	lncRNA
XM_012179910.2	ATP10D	mRNA	XR_001434892.1	lncRNA
XM_012179910.2	ATP10D	mRNA	XR_001434892.1	lncRNA
XM_012179910.2	ATP10D	mRNA	XR_001434892.1	lncRNA
XM_012179910.2	ATP10D	mRNA	XR_001434892.1	lncRNA
XM_012179910.2	ATP10D	mRNA	XR_001434892.1	lncRNA
XM_012179910.2	ATP10D	mRNA	XR_001434892.1	lncRNA
XM_012179910.2	ATP10D	mRNA	XR_001434892.1	lncRNA
XM_012179910.2	ATP10D	mRNA	XR_001434892.1	lncRNA
XM_012179910.2	ATP10D	mRNA	XR_001434892.1	lncRNA
XM_015096937.1	BMF	mRNA	XR_001033284.2	lncRNA
XM_015096937.1	BMF	mRNA	XR_001033284.2	lncRNA
XM_015096937.1	BMF	mRNA	XR_001033284.2	lncRNA
XM_015096937.1	BMF	mRNA	XR_001033284.2	lncRNA
XM_015096937.1	BMF	mRNA	XR_001033284.2	lncRNA
XM_015096937.1	BMF	mRNA	XR_001033284.2	lncRNA
XM_015096937.1	BMF	mRNA	XR_001033284.2	lncRNA
XM_015096937.1	BMF	mRNA	XR_001033284.2	lncRNA
XM_015096937.1	BMF	mRNA	XR_001033284.2	lncRNA
XM_004023170.3	LOC10110814	mRNA	XR_001041211.2	lncRNA
XM_004023170.3	LOC10110814	mRNA	XR_001041211.2	lncRNA
XM_004023170.3	LOC10110814	mRNA	XR_001041211.2	lncRNA
XM_004023170.3	LOC10110814	mRNA	XR_001041211.2	lncRNA
XM_004023170.3	LOC10110814	mRNA	XR_001041211.2	lncRNA
XM_004023170.3	LOC10110814	mRNA	XR_001041211.2	lncRNA
XM_004023170.3	LOC10110814	mRNA	XR_001041211.2	lncRNA
XM_004023170.3	LOC10110814	mRNA	XR_001041211.2	lncRNA
XM_004023170.3	LOC10110814	mRNA	XR_001041211.2	lncRNA
XM_012165732.1	FAM228B	mRNA	XR_001436102.1	lncRNA
XM_012165732.1	FAM228B	mRNA	XR_001436102.1	lncRNA
XM_012165732.1	FAM228B	mRNA	XR_001436102.1	lncRNA
XM_012165732.1	FAM228B	mRNA	XR_001436102.1	lncRNA
XM_012165732.1	FAM228B	mRNA	XR_001436102.1	lncRNA
XM_012165732.1	FAM228B	mRNA	XR_001436102.1	lncRNA
XM_012165732.1	FAM228B	mRNA	XR_001436102.1	lncRNA
XM_012165732.1	FAM228B	mRNA	XR_001436102.1	lncRNA
XM_012165732.1	FAM228B	mRNA	XR_001436102.1	lncRNA
XM_004010552.3	VPS13C	mRNA	XR_001021689.2	lncRNA
XM_004010552.3	VPS13C	mRNA	XR_001021689.2	lncRNA
XM_004010552.3	VPS13C	mRNA	XR_001021689.2	lncRNA
XM_004010552.3	VPS13C	mRNA	XR_001021689.2	lncRNA
XM_004010552.3	VPS13C	mRNA	XR_001021689.2	lncRNA
XM_004010552.3	VPS13C	mRNA	XR_001021689.2	lncRNA
XM_004010552.3	VPS13C	mRNA	XR_001021689.2	lncRNA
XM_004010552.3	VPS13C	mRNA	XR_001021689.2	lncRNA
XM_004010552.3	VPS13C	mRNA	XR_001021689.2	lncRNA

XM_012105332.2	USP54	mRNA	XR_001436102.1	lncRNA
XM_012105332.2	USP54	mRNA	XR_001436102.1	lncRNA
XM_012105332.2	USP54	mRNA	XR_001436102.1	lncRNA
XM_012105332.2	USP54	mRNA	XR_001436102.1	lncRNA
XM_012105332.2	USP54	mRNA	XR_001436102.1	lncRNA
XM_012105332.2	USP54	mRNA	XR_001436102.1	lncRNA
XM_012105332.2	USP54	mRNA	XR_001436102.1	lncRNA
XM_012105332.2	USP54	mRNA	XR_001436102.1	lncRNA
XM_012105332.2	USP54	mRNA	XR_001436102.1	lncRNA
XM_004011865.3	ENPP2	mRNA	XR_001436102.1	lncRNA
XM_004011865.3	ENPP2	mRNA	XR_001436102.1	lncRNA
XM_004011865.3	ENPP2	mRNA	XR_001436102.1	lncRNA
XM_004011865.3	ENPP2	mRNA	XR_001436102.1	lncRNA
XM_004011865.3	ENPP2	mRNA	XR_001436102.1	lncRNA
XM_004011865.3	ENPP2	mRNA	XR_001436102.1	lncRNA
XM_004011865.3	ENPP2	mRNA	XR_001436102.1	lncRNA
XM_004011865.3	ENPP2	mRNA	XR_001436102.1	lncRNA
XM_004011865.3	ENPP2	mRNA	XR_001436102.1	lncRNA
XM_012181326.2	MAP4K5	mRNA	XR_001434203.1	lncRNA
XM_012181326.2	MAP4K5	mRNA	XR_001434203.1	lncRNA
XM_012181326.2	MAP4K5	mRNA	XR_001434203.1	lncRNA
XM_012181326.2	MAP4K5	mRNA	XR_001434203.1	lncRNA
XM_012181326.2	MAP4K5	mRNA	XR_001434203.1	lncRNA
XM_012181326.2	MAP4K5	mRNA	XR_001434203.1	lncRNA
XM_012181326.2	MAP4K5	mRNA	XR_001434203.1	lncRNA
XM_012181326.2	MAP4K5	mRNA	XR_001434203.1	lncRNA
XM_012181326.2	MAP4K5	mRNA	XR_001434203.1	lncRNA
XM_004009100.3	PAM	mRNA	XR_001434203.1	lncRNA
XM_004009100.3	PAM	mRNA	XR_001434203.1	lncRNA
XM_004009100.3	PAM	mRNA	XR_001434203.1	lncRNA
XM_004009100.3	PAM	mRNA	XR_001434203.1	lncRNA
XM_004009100.3	PAM	mRNA	XR_001434203.1	lncRNA
XM_004009100.3	PAM	mRNA	XR_001434203.1	lncRNA
XM_004009100.3	PAM	mRNA	XR_001434203.1	lncRNA
XM_004009100.3	PAM	mRNA	XR_001434203.1	lncRNA
XM_004009100.3	PAM	mRNA	XR_001434203.1	lncRNA
XM_012095439.2	SIK2	mRNA	XR_001436817.1	lncRNA
XM_012095439.2	SIK2	mRNA	XR_001436817.1	lncRNA
XM_012095439.2	SIK2	mRNA	XR_001436817.1	lncRNA
XM_012095439.2	SIK2	mRNA	XR_001436817.1	lncRNA
XM_012095439.2	SIK2	mRNA	XR_001436817.1	lncRNA
XM_012095439.2	SIK2	mRNA	XR_001436817.1	lncRNA
XM_012095439.2	SIK2	mRNA	XR_001436817.1	lncRNA
XM_012095439.2	SIK2	mRNA	XR_001436817.1	lncRNA
XM_012095439.2	SIK2	mRNA	XR_001436817.1	lncRNA
XM_012097367.2	SH3D19	mRNA	XR_001436817.1	lncRNA
XM_012097367.2	SH3D19	mRNA	XR_001436817.1	lncRNA
XM_012097367.2	SH3D19	mRNA	XR_001436817.1	lncRNA
XM_012097367.2	SH3D19	mRNA	XR_001436817.1	lncRNA
XM_012097367.2	SH3D19	mRNA	XR_001436817.1	lncRNA
XM_012097367.2	SH3D19	mRNA	XR_001436817.1	lncRNA
XM_012097367.2	SH3D19	mRNA	XR_001436817.1	lncRNA
XM_012097367.2	SH3D19	mRNA	XR_001436817.1	lncRNA
XM_012097367.2	SH3D19	mRNA	XR_001436817.1	lncRNA

XM_012097367.2	SH3D19	mRNA	XR_001434203.1	lncRNA
XM_012097367.2	SH3D19	mRNA	XR_001434203.1	lncRNA
XM_012097367.2	SH3D19	mRNA	XR_001434203.1	lncRNA
XM_012097367.2	SH3D19	mRNA	XR_001434203.1	lncRNA
XM_012097367.2	SH3D19	mRNA	XR_001434203.1	lncRNA
XM_012097367.2	SH3D19	mRNA	XR_001434203.1	lncRNA
XM_012097367.2	SH3D19	mRNA	XR_001434203.1	lncRNA
XM_012097367.2	SH3D19	mRNA	XR_001434203.1	lncRNA
XM_012097367.2	SH3D19	mRNA	XR_001434203.1	lncRNA
XM_012181326.2	MAP4K5	mRNA	XR_001436817.1	lncRNA
XM_012181326.2	MAP4K5	mRNA	XR_001436817.1	lncRNA
XM_012181326.2	MAP4K5	mRNA	XR_001436817.1	lncRNA
XM_012181326.2	MAP4K5	mRNA	XR_001436817.1	lncRNA
XM_012181326.2	MAP4K5	mRNA	XR_001436817.1	lncRNA
XM_012181326.2	MAP4K5	mRNA	XR_001436817.1	lncRNA
XM_012181326.2	MAP4K5	mRNA	XR_001436817.1	lncRNA
XM_012181326.2	MAP4K5	mRNA	XR_001436817.1	lncRNA
XM_012181326.2	MAP4K5	mRNA	XR_001436817.1	lncRNA
XM_012177947.2	INSR	mRNA	XR_001033284.2	lncRNA
XM_012177947.2	INSR	mRNA	XR_001033284.2	lncRNA
XM_012177947.2	INSR	mRNA	XR_001033284.2	lncRNA
XM_012177947.2	INSR	mRNA	XR_001033284.2	lncRNA
XM_012177947.2	INSR	mRNA	XR_001033284.2	lncRNA
XM_012177947.2	INSR	mRNA	XR_001033284.2	lncRNA
XM_012177947.2	INSR	mRNA	XR_001033284.2	lncRNA
XM_012177947.2	INSR	mRNA	XR_001033284.2	lncRNA
XM_012177947.2	INSR	mRNA	XR_001033284.2	lncRNA
XM_012180879.2	MYO9A	mRNA	XR_001044126.1	lncRNA
XM_012180879.2	MYO9A	mRNA	XR_001044126.1	lncRNA
XM_012180879.2	MYO9A	mRNA	XR_001044126.1	lncRNA
XM_012180879.2	MYO9A	mRNA	XR_001044126.1	lncRNA
XM_012180879.2	MYO9A	mRNA	XR_001044126.1	lncRNA
XM_012180879.2	MYO9A	mRNA	XR_001044126.1	lncRNA
XM_012180879.2	MYO9A	mRNA	XR_001044126.1	lncRNA
XM_012180879.2	MYO9A	mRNA	XR_001044126.1	lncRNA
XM_012180879.2	MYO9A	mRNA	XR_001044126.1	lncRNA
XM_012181966.2	SIPA1L1	mRNA	XR_001025491.1	lncRNA
XM_012181966.2	SIPA1L1	mRNA	XR_001025491.1	lncRNA
XM_012181966.2	SIPA1L1	mRNA	XR_001025491.1	lncRNA
XM_012181966.2	SIPA1L1	mRNA	XR_001025491.1	lncRNA
XM_012181966.2	SIPA1L1	mRNA	XR_001025491.1	lncRNA
XM_012181966.2	SIPA1L1	mRNA	XR_001025491.1	lncRNA
XM_012181966.2	SIPA1L1	mRNA	XR_001025491.1	lncRNA
XM_012181966.2	SIPA1L1	mRNA	XR_001025491.1	lncRNA
XM_012181966.2	SIPA1L1	mRNA	XR_001025491.1	lncRNA
XM_012181960.2	SIPA1L1	mRNA	XR_001025491.1	lncRNA
XM_012181960.2	SIPA1L1	mRNA	XR_001025491.1	lncRNA
XM_012181960.2	SIPA1L1	mRNA	XR_001025491.1	lncRNA
XM_012181960.2	SIPA1L1	mRNA	XR_001025491.1	lncRNA
XM_012181960.2	SIPA1L1	mRNA	XR_001025491.1	lncRNA
XM_012181960.2	SIPA1L1	mRNA	XR_001025491.1	lncRNA
XM_012181960.2	SIPA1L1	mRNA	XR_001025491.1	lncRNA
XM_012181960.2	SIPA1L1	mRNA	XR_001025491.1	lncRNA
XM_012181960.2	SIPA1L1	mRNA	XR_001025491.1	lncRNA

XM_012186944.1	DSTYK	mRNA	XR_001436127.1	lncRNA
XM_012186944.1	DSTYK	mRNA	XR_001436127.1	lncRNA
XM_012186944.1	DSTYK	mRNA	XR_001436127.1	lncRNA
XM_012186944.1	DSTYK	mRNA	XR_001436127.1	lncRNA
XM_012186944.1	DSTYK	mRNA	XR_001436127.1	lncRNA
XM_012186944.1	DSTYK	mRNA	XR_001436127.1	lncRNA
XM_012186944.1	DSTYK	mRNA	XR_001436127.1	lncRNA
XM_012186944.1	DSTYK	mRNA	XR_001436127.1	lncRNA
XM_012186944.1	DSTYK	mRNA	XR_001436127.1	lncRNA
XM_012124921.1	KIAA2026	mRNA	XR_001024290.1	lncRNA
XM_012124921.1	KIAA2026	mRNA	XR_001024290.1	lncRNA
XM_012124921.1	KIAA2026	mRNA	XR_001024290.1	lncRNA
XM_012124921.1	KIAA2026	mRNA	XR_001024290.1	lncRNA
XM_012124921.1	KIAA2026	mRNA	XR_001024290.1	lncRNA
XM_012124921.1	KIAA2026	mRNA	XR_001024290.1	lncRNA
XM_012124921.1	KIAA2026	mRNA	XR_001024290.1	lncRNA
XM_012124921.1	KIAA2026	mRNA	XR_001024290.1	lncRNA
XM_015092063.1	FUBP1	mRNA	XR_001024290.1	lncRNA
XM_015092063.1	FUBP1	mRNA	XR_001024290.1	lncRNA
XM_015092063.1	FUBP1	mRNA	XR_001024290.1	lncRNA
XM_015092063.1	FUBP1	mRNA	XR_001024290.1	lncRNA
XM_015092063.1	FUBP1	mRNA	XR_001024290.1	lncRNA
XM_015092063.1	FUBP1	mRNA	XR_001024290.1	lncRNA
XM_015092063.1	FUBP1	mRNA	XR_001024290.1	lncRNA
XM_015092063.1	FUBP1	mRNA	XR_001024290.1	lncRNA
XM_012182450.2	IBTK	mRNA	XR_001025491.1	lncRNA
XM_012182450.2	IBTK	mRNA	XR_001025491.1	lncRNA
XM_012182450.2	IBTK	mRNA	XR_001025491.1	lncRNA
XM_012182450.2	IBTK	mRNA	XR_001025491.1	lncRNA
XM_012182450.2	IBTK	mRNA	XR_001025491.1	lncRNA
XM_012182450.2	IBTK	mRNA	XR_001025491.1	lncRNA
XM_012182450.2	IBTK	mRNA	XR_001025491.1	lncRNA
XM_012182450.2	IBTK	mRNA	XR_001025491.1	lncRNA
XM_012134956.1	STK39	mRNA	XR_001024290.1	lncRNA
XM_012134956.1	STK39	mRNA	XR_001024290.1	lncRNA
XM_012134956.1	STK39	mRNA	XR_001024290.1	lncRNA
XM_012134956.1	STK39	mRNA	XR_001024290.1	lncRNA
XM_012134956.1	STK39	mRNA	XR_001024290.1	lncRNA
XM_012134956.1	STK39	mRNA	XR_001024290.1	lncRNA
XM_012134956.1	STK39	mRNA	XR_001024290.1	lncRNA
XM_012134956.1	STK39	mRNA	XR_001024290.1	lncRNA
XM_012173780.2	REV1	mRNA	TCONS_00022613	lncRNA
XM_012173780.2	REV1	mRNA	TCONS_00022613	lncRNA
XM_012173780.2	REV1	mRNA	TCONS_00022613	lncRNA
XM_012173780.2	REV1	mRNA	TCONS_00022613	lncRNA
XM_012173780.2	REV1	mRNA	TCONS_00022613	lncRNA
XM_012173780.2	REV1	mRNA	TCONS_00022613	lncRNA
XM_012173780.2	REV1	mRNA	TCONS_00022613	lncRNA
XM_012173780.2	REV1	mRNA	TCONS_00022613	lncRNA
XM_012119517.2	SORBS3	mRNA	TCONS_00022613	lncRNA
XM_012119517.2	SORBS3	mRNA	TCONS_00022613	lncRNA
XM_012119517.2	SORBS3	mRNA	TCONS_00022613	lncRNA
XM_012119517.2	SORBS3	mRNA	TCONS_00022613	lncRNA
XM_012119517.2	SORBS3	mRNA	TCONS_00022613	lncRNA

XM_012119517.2	SORBS3	mRNA	TCONS_00022613	lncRNA
XM_012119517.2	SORBS3	mRNA	TCONS_00022613	lncRNA
XM_012119517.2	SORBS3	mRNA	TCONS_00022613	lncRNA
XM_012182538.2	TRAPPC3L	mRNA	TCONS_00022613	lncRNA
XM_012182538.2	TRAPPC3L	mRNA	TCONS_00022613	lncRNA
XM_012182538.2	TRAPPC3L	mRNA	TCONS_00022613	lncRNA
XM_012182538.2	TRAPPC3L	mRNA	TCONS_00022613	lncRNA
XM_012182538.2	TRAPPC3L	mRNA	TCONS_00022613	lncRNA
XM_012182538.2	TRAPPC3L	mRNA	TCONS_00022613	lncRNA
XM_012182538.2	TRAPPC3L	mRNA	TCONS_00022613	lncRNA
XM_012182538.2	TRAPPC3L	mRNA	TCONS_00022613	lncRNA
XM_015103495.1	UROS	mRNA	XR_001434202.1	lncRNA
XM_015103495.1	UROS	mRNA	XR_001434202.1	lncRNA
XM_015103495.1	UROS	mRNA	XR_001434202.1	lncRNA
XM_015103495.1	UROS	mRNA	XR_001434202.1	lncRNA
XM_015103495.1	UROS	mRNA	XR_001434202.1	lncRNA
XM_015103495.1	UROS	mRNA	XR_001434202.1	lncRNA
XM_015103495.1	UROS	mRNA	XR_001434202.1	lncRNA
XM_015103495.1	UROS	mRNA	XR_001434202.1	lncRNA
XM_015103495.1	UROS	mRNA	XR_001434202.1	lncRNA
XM_015103495.1	UROS	mRNA	XR_001034847.2	lncRNA
XM_015103495.1	UROS	mRNA	XR_001034847.2	lncRNA
XM_015103495.1	UROS	mRNA	XR_001034847.2	lncRNA
XM_015103495.1	UROS	mRNA	XR_001034847.2	lncRNA
XM_015103495.1	UROS	mRNA	XR_001034847.2	lncRNA
XM_015103495.1	UROS	mRNA	XR_001034847.2	lncRNA
XM_015103495.1	UROS	mRNA	XR_001034847.2	lncRNA
XM_015103495.1	UROS	mRNA	XR_001034847.2	lncRNA
XM_015103495.1	UROS	mRNA	XR_001034847.2	lncRNA
XM_015095790.1	CC2D1A	mRNA	XR_001034847.2	lncRNA
XM_015095790.1	CC2D1A	mRNA	XR_001034847.2	lncRNA
XM_015095790.1	CC2D1A	mRNA	XR_001034847.2	lncRNA
XM_015095790.1	CC2D1A	mRNA	XR_001034847.2	lncRNA
XM_015095790.1	CC2D1A	mRNA	XR_001034847.2	lncRNA
XM_015095790.1	CC2D1A	mRNA	XR_001034847.2	lncRNA
XM_015095790.1	CC2D1A	mRNA	XR_001034847.2	lncRNA
XM_015095790.1	CC2D1A	mRNA	XR_001034847.2	lncRNA
XM_015095790.1	CC2D1A	mRNA	XR_001034847.2	lncRNA
XM_015095790.1	CC2D1A	mRNA	XR_001434202.1	lncRNA
XM_015095790.1	CC2D1A	mRNA	XR_001434202.1	lncRNA
XM_015095790.1	CC2D1A	mRNA	XR_001434202.1	lncRNA
XM_015095790.1	CC2D1A	mRNA	XR_001434202.1	lncRNA
XM_015095790.1	CC2D1A	mRNA	XR_001434202.1	lncRNA
XM_015095790.1	CC2D1A	mRNA	XR_001434202.1	lncRNA
XM_015095790.1	CC2D1A	mRNA	XR_001434202.1	lncRNA
XM_015095790.1	CC2D1A	mRNA	XR_001434202.1	lncRNA
XM_015095790.1	CC2D1A	mRNA	XR_001434202.1	lncRNA
XM_012185846.2	MBTD1	mRNA	XR_001436532.1	lncRNA
XM_012185846.2	MBTD1	mRNA	XR_001436532.1	lncRNA
XM_012185846.2	MBTD1	mRNA	XR_001436532.1	lncRNA
XM_012185846.2	MBTD1	mRNA	XR_001436532.1	lncRNA
XM_012185846.2	MBTD1	mRNA	XR_001436532.1	lncRNA
XM_012185846.2	MBTD1	mRNA	XR_001436532.1	lncRNA
XM_012185846.2	MBTD1	mRNA	XR_001436532.1	lncRNA

XM_012185846.2	MBTD1	mRNA	XR_001436532.1	lncRNA
XM_012185846.2	MBTD1	mRNA	XR_001436532.1	lncRNA
XM_015094531.1	ANAPC1	mRNA	XR_001033284.2	lncRNA
XM_015094531.1	ANAPC1	mRNA	XR_001033284.2	lncRNA
XM_015094531.1	ANAPC1	mRNA	XR_001033284.2	lncRNA
XM_015094531.1	ANAPC1	mRNA	XR_001033284.2	lncRNA
XM_015094531.1	ANAPC1	mRNA	XR_001033284.2	lncRNA
XM_015094531.1	ANAPC1	mRNA	XR_001033284.2	lncRNA
XM_015094531.1	ANAPC1	mRNA	XR_001033284.2	lncRNA
XM_015094531.1	ANAPC1	mRNA	XR_001033284.2	lncRNA
XM_015094531.1	ANAPC1	mRNA	XR_001033284.2	lncRNA
XM_012119517.2	SORBS3	mRNA	XR_001436102.1	lncRNA
XM_012119517.2	SORBS3	mRNA	XR_001436102.1	lncRNA
XM_012119517.2	SORBS3	mRNA	XR_001436102.1	lncRNA
XM_012119517.2	SORBS3	mRNA	XR_001436102.1	lncRNA
XM_012119517.2	SORBS3	mRNA	XR_001436102.1	lncRNA
XM_012119517.2	SORBS3	mRNA	XR_001436102.1	lncRNA
XM_012119517.2	SORBS3	mRNA	XR_001436102.1	lncRNA
XM_012119517.2	SORBS3	mRNA	XR_001436102.1	lncRNA
XM_012119517.2	SORBS3	mRNA	XR_001436102.1	lncRNA
XM_004020074.3	PIK3AP1	mRNA	XR_001021689.2	lncRNA
XM_004020074.3	PIK3AP1	mRNA	XR_001021689.2	lncRNA
XM_004020074.3	PIK3AP1	mRNA	XR_001021689.2	lncRNA
XM_004020074.3	PIK3AP1	mRNA	XR_001021689.2	lncRNA
XM_004020074.3	PIK3AP1	mRNA	XR_001021689.2	lncRNA
XM_004020074.3	PIK3AP1	mRNA	XR_001021689.2	lncRNA
XM_004020074.3	PIK3AP1	mRNA	XR_001021689.2	lncRNA
XM_004020074.3	PIK3AP1	mRNA	XR_001021689.2	lncRNA
XM_004020074.3	PIK3AP1	mRNA	XR_001021689.2	lncRNA
XM_012102845.2	GBF1	mRNA	XR_001436102.1	lncRNA
XM_012102845.2	GBF1	mRNA	XR_001436102.1	lncRNA
XM_012102845.2	GBF1	mRNA	XR_001436102.1	lncRNA
XM_012102845.2	GBF1	mRNA	XR_001436102.1	lncRNA
XM_012102845.2	GBF1	mRNA	XR_001436102.1	lncRNA
XM_012102845.2	GBF1	mRNA	XR_001436102.1	lncRNA
XM_012102845.2	GBF1	mRNA	XR_001436102.1	lncRNA
XM_012102845.2	GBF1	mRNA	XR_001436102.1	lncRNA
XM_012102845.2	GBF1	mRNA	XR_001436102.1	lncRNA
XM_012178788.2	HMGXB3	mRNA	XR_001436102.1	lncRNA
XM_012178788.2	HMGXB3	mRNA	XR_001436102.1	lncRNA
XM_012178788.2	HMGXB3	mRNA	XR_001436102.1	lncRNA
XM_012178788.2	HMGXB3	mRNA	XR_001436102.1	lncRNA
XM_012178788.2	HMGXB3	mRNA	XR_001436102.1	lncRNA
XM_012178788.2	HMGXB3	mRNA	XR_001436102.1	lncRNA
XM_012178788.2	HMGXB3	mRNA	XR_001436102.1	lncRNA
XM_012178788.2	HMGXB3	mRNA	XR_001436102.1	lncRNA
XM_012178788.2	HMGXB3	mRNA	XR_001436102.1	lncRNA
XM_012173296.2	CDKL4	mRNA	XR_001436102.1	lncRNA
XM_012173296.2	CDKL4	mRNA	XR_001436102.1	lncRNA
XM_012173296.2	CDKL4	mRNA	XR_001436102.1	lncRNA
XM_012173296.2	CDKL4	mRNA	XR_001436102.1	lncRNA
XM_012173296.2	CDKL4	mRNA	XR_001436102.1	lncRNA
XM_012173296.2	CDKL4	mRNA	XR_001436102.1	lncRNA
XM_012173296.2	CDKL4	mRNA	XR_001436102.1	lncRNA

XM_012173296.2	CDKL4	mRNA	XR_001436102.1	lncRNA
XM_012173296.2	CDKL4	mRNA	XR_001436102.1	lncRNA
XM_015102318.1	SETD5	mRNA	XR_001436102.1	lncRNA
XM_015102318.1	SETD5	mRNA	XR_001436102.1	lncRNA
XM_015102318.1	SETD5	mRNA	XR_001436102.1	lncRNA
XM_015102318.1	SETD5	mRNA	XR_001436102.1	lncRNA
XM_015102318.1	SETD5	mRNA	XR_001436102.1	lncRNA
XM_015102318.1	SETD5	mRNA	XR_001436102.1	lncRNA
XM_015102318.1	SETD5	mRNA	XR_001436102.1	lncRNA
XM_015102318.1	SETD5	mRNA	XR_001436102.1	lncRNA
XM_015102318.1	SETD5	mRNA	XR_001436102.1	lncRNA
XM_012098820.2	RALGAPA1	mRNA	XR_001436102.1	lncRNA
XM_012098820.2	RALGAPA1	mRNA	XR_001436102.1	lncRNA
XM_012098820.2	RALGAPA1	mRNA	XR_001436102.1	lncRNA
XM_012098820.2	RALGAPA1	mRNA	XR_001436102.1	lncRNA
XM_012098820.2	RALGAPA1	mRNA	XR_001436102.1	lncRNA
XM_012098820.2	RALGAPA1	mRNA	XR_001436102.1	lncRNA
XM_012098820.2	RALGAPA1	mRNA	XR_001436102.1	lncRNA
XM_012098820.2	RALGAPA1	mRNA	XR_001436102.1	lncRNA
XM_012098820.2	RALGAPA1	mRNA	XR_001436102.1	lncRNA
XM_012184884.2	DOCK9	mRNA	XR_001021689.2	lncRNA
XM_012184884.2	DOCK9	mRNA	XR_001021689.2	lncRNA
XM_012184884.2	DOCK9	mRNA	XR_001021689.2	lncRNA
XM_012184884.2	DOCK9	mRNA	XR_001021689.2	lncRNA
XM_012184884.2	DOCK9	mRNA	XR_001021689.2	lncRNA
XM_012184884.2	DOCK9	mRNA	XR_001021689.2	lncRNA
XM_012184884.2	DOCK9	mRNA	XR_001021689.2	lncRNA
XM_012184884.2	DOCK9	mRNA	XR_001021689.2	lncRNA
XM_012184884.2	DOCK9	mRNA	XR_001021689.2	lncRNA
XM_012179034.2	FER	mRNA	XR_001025491.1	lncRNA
XM_012179034.2	FER	mRNA	XR_001025491.1	lncRNA
XM_012179034.2	FER	mRNA	XR_001025491.1	lncRNA
XM_012179034.2	FER	mRNA	XR_001025491.1	lncRNA
XM_012179034.2	FER	mRNA	XR_001025491.1	lncRNA
XM_012179034.2	FER	mRNA	XR_001025491.1	lncRNA
XM_012179034.2	FER	mRNA	XR_001025491.1	lncRNA
XM_012179034.2	FER	mRNA	XR_001025491.1	lncRNA
XM_012179034.2	FER	mRNA	XR_001025491.1	lncRNA
XM_012179034.2	FER	mRNA	XR_001024290.1	lncRNA
XM_012179034.2	FER	mRNA	XR_001024290.1	lncRNA
XM_012179034.2	FER	mRNA	XR_001024290.1	lncRNA
XM_012179034.2	FER	mRNA	XR_001024290.1	lncRNA
XM_012179034.2	FER	mRNA	XR_001024290.1	lncRNA
XM_012179034.2	FER	mRNA	XR_001024290.1	lncRNA
XM_012179034.2	FER	mRNA	XR_001024290.1	lncRNA
XM_012179034.2	FER	mRNA	XR_001024290.1	lncRNA
XM_012179034.2	FER	mRNA	XR_001024290.1	lncRNA
XM_012102629.2	SORBS1	mRNA	XR_001434761.1	lncRNA
XM_012102629.2	SORBS1	mRNA	XR_001434761.1	lncRNA
XM_012102629.2	SORBS1	mRNA	XR_001434761.1	lncRNA
XM_012102629.2	SORBS1	mRNA	XR_001434761.1	lncRNA
XM_012102629.2	SORBS1	mRNA	XR_001434761.1	lncRNA
XM_012102629.2	SORBS1	mRNA	XR_001434761.1	lncRNA
XM_012102629.2	SORBS1	mRNA	XR_001434761.1	lncRNA

XM_012102629.2	SORBS1	mRNA	XR_001434761.1	lncRNA
XM_012102629.2	SORBS1	mRNA	XR_001434761.1	lncRNA
XM_004022347.2	TMEM255A	mRNA	XR_001434761.1	lncRNA
XM_004022347.2	TMEM255A	mRNA	XR_001434761.1	lncRNA
XM_004022347.2	TMEM255A	mRNA	XR_001434761.1	lncRNA
XM_004022347.2	TMEM255A	mRNA	XR_001434761.1	lncRNA
XM_004022347.2	TMEM255A	mRNA	XR_001434761.1	lncRNA
XM_004022347.2	TMEM255A	mRNA	XR_001434761.1	lncRNA
XM_004022347.2	TMEM255A	mRNA	XR_001434761.1	lncRNA
XM_004022347.2	TMEM255A	mRNA	XR_001434761.1	lncRNA
XM_012187432.1	PRKCZ	mRNA	XR_001041211.2	lncRNA
XM_012187432.1	PRKCZ	mRNA	XR_001041211.2	lncRNA
XM_012187432.1	PRKCZ	mRNA	XR_001041211.2	lncRNA
XM_012187432.1	PRKCZ	mRNA	XR_001041211.2	lncRNA
XM_012187432.1	PRKCZ	mRNA	XR_001041211.2	lncRNA
XM_012187432.1	PRKCZ	mRNA	XR_001041211.2	lncRNA
XM_012187432.1	PRKCZ	mRNA	XR_001041211.2	lncRNA
XM_012187432.1	PRKCZ	mRNA	XR_001041211.2	lncRNA
XM_012187432.1	PRKCZ	mRNA	XR_001041211.2	lncRNA
XM_012097035.1	C16H5orf42	mRNA	TCONS_00022613	lncRNA
XM_012097035.1	C16H5orf42	mRNA	TCONS_00022613	lncRNA
XM_012097035.1	C16H5orf42	mRNA	TCONS_00022613	lncRNA
XM_012097035.1	C16H5orf42	mRNA	TCONS_00022613	lncRNA
XM_012097035.1	C16H5orf42	mRNA	TCONS_00022613	lncRNA
XM_012097035.1	C16H5orf42	mRNA	TCONS_00022613	lncRNA
XM_012097035.1	C16H5orf42	mRNA	TCONS_00022613	lncRNA
XM_012097035.1	C16H5orf42	mRNA	TCONS_00022613	lncRNA
XM_012152472.1	LUZP1	mRNA	TCONS_00022613	lncRNA
XM_012152472.1	LUZP1	mRNA	TCONS_00022613	lncRNA
XM_012152472.1	LUZP1	mRNA	TCONS_00022613	lncRNA
XM_012152472.1	LUZP1	mRNA	TCONS_00022613	lncRNA
XM_012152472.1	LUZP1	mRNA	TCONS_00022613	lncRNA
XM_012152472.1	LUZP1	mRNA	TCONS_00022613	lncRNA
XM_012152472.1	LUZP1	mRNA	TCONS_00022613	lncRNA
XM_012152472.1	LUZP1	mRNA	TCONS_00022613	lncRNA
XM_004005653.3	ZBTB26	mRNA	XR_001033284.2	lncRNA
XM_004005653.3	ZBTB26	mRNA	XR_001033284.2	lncRNA
XM_004005653.3	ZBTB26	mRNA	XR_001033284.2	lncRNA
XM_004005653.3	ZBTB26	mRNA	XR_001033284.2	lncRNA
XM_004005653.3	ZBTB26	mRNA	XR_001033284.2	lncRNA
XM_004005653.3	ZBTB26	mRNA	XR_001033284.2	lncRNA
XM_004005653.3	ZBTB26	mRNA	XR_001033284.2	lncRNA
XM_004005653.3	ZBTB26	mRNA	XR_001033284.2	lncRNA
XM_015092421.1	BBX	mRNA	XR_001033284.2	lncRNA
XM_015092421.1	BBX	mRNA	XR_001033284.2	lncRNA
XM_015092421.1	BBX	mRNA	XR_001033284.2	lncRNA
XM_015092421.1	BBX	mRNA	XR_001033284.2	lncRNA
XM_015092421.1	BBX	mRNA	XR_001033284.2	lncRNA
XM_015092421.1	BBX	mRNA	XR_001033284.2	lncRNA
XM_015092421.1	BBX	mRNA	XR_001033284.2	lncRNA
XM_015092421.1	BBX	mRNA	XR_001033284.2	lncRNA
XM_004006548.3	R3HDM2	mRNA	XR_001020707.2	lncRNA
XM_004006548.3	R3HDM2	mRNA	XR_001020707.2	lncRNA
XM_004006548.3	R3HDM2	mRNA	XR_001020707.2	lncRNA

XM_004006548.3	R3HDM2	mRNA	XR_001020707.2	lncRNA
XM_004006548.3	R3HDM2	mRNA	XR_001020707.2	lncRNA
XM_004006548.3	R3HDM2	mRNA	XR_001020707.2	lncRNA
XM_004006548.3	R3HDM2	mRNA	XR_001020707.2	lncRNA
XM_004006548.3	R3HDM2	mRNA	XR_001020707.2	lncRNA
XM_015099256.1	CEP104	mRNA	XR_001436102.1	lncRNA
XM_015099256.1	CEP104	mRNA	XR_001436102.1	lncRNA
XM_015099256.1	CEP104	mRNA	XR_001436102.1	lncRNA
XM_015099256.1	CEP104	mRNA	XR_001436102.1	lncRNA
XM_015099256.1	CEP104	mRNA	XR_001436102.1	lncRNA
XM_015099256.1	CEP104	mRNA	XR_001436102.1	lncRNA
XM_015099256.1	CEP104	mRNA	XR_001436102.1	lncRNA
XM_015099256.1	CEP104	mRNA	XR_001436102.1	lncRNA
XM_015099256.1	CEP104	mRNA	XR_001436102.1	lncRNA
XM_012184887.2	DOCK9	mRNA	XR_001021689.2	lncRNA
XM_012184887.2	DOCK9	mRNA	XR_001021689.2	lncRNA
XM_012184887.2	DOCK9	mRNA	XR_001021689.2	lncRNA
XM_012184887.2	DOCK9	mRNA	XR_001021689.2	lncRNA
XM_012184887.2	DOCK9	mRNA	XR_001021689.2	lncRNA
XM_012184887.2	DOCK9	mRNA	XR_001021689.2	lncRNA
XM_012184887.2	DOCK9	mRNA	XR_001021689.2	lncRNA
XM_012184887.2	DOCK9	mRNA	XR_001021689.2	lncRNA
XM_012184887.2	DOCK9	mRNA	XR_001021689.2	lncRNA
XM_012105690.2	WDR17	mRNA	XR_001436102.1	lncRNA
XM_012105690.2	WDR17	mRNA	XR_001436102.1	lncRNA
XM_012105690.2	WDR17	mRNA	XR_001436102.1	lncRNA
XM_012105690.2	WDR17	mRNA	XR_001436102.1	lncRNA
XM_012105690.2	WDR17	mRNA	XR_001436102.1	lncRNA
XM_012105690.2	WDR17	mRNA	XR_001436102.1	lncRNA
XM_012105690.2	WDR17	mRNA	XR_001436102.1	lncRNA
XM_012105690.2	WDR17	mRNA	XR_001436102.1	lncRNA
XM_012105690.2	WDR17	mRNA	XR_001436102.1	lncRNA
XM_015098529.1	NF1	mRNA	XR_001436102.1	lncRNA
XM_015098529.1	NF1	mRNA	XR_001436102.1	lncRNA
XM_015098529.1	NF1	mRNA	XR_001436102.1	lncRNA
XM_015098529.1	NF1	mRNA	XR_001436102.1	lncRNA
XM_015098529.1	NF1	mRNA	XR_001436102.1	lncRNA
XM_015098529.1	NF1	mRNA	XR_001436102.1	lncRNA
XM_015098529.1	NF1	mRNA	XR_001436102.1	lncRNA
XM_015098529.1	NF1	mRNA	XR_001436102.1	lncRNA
XM_015098529.1	NF1	mRNA	XR_001436102.1	lncRNA
XM_015098990.1	GNA13	mRNA	XR_001436817.1	lncRNA
XM_015098990.1	GNA13	mRNA	XR_001436817.1	lncRNA
XM_015098990.1	GNA13	mRNA	XR_001436817.1	lncRNA
XM_015098990.1	GNA13	mRNA	XR_001436817.1	lncRNA
XM_015098990.1	GNA13	mRNA	XR_001436817.1	lncRNA
XM_015098990.1	GNA13	mRNA	XR_001436817.1	lncRNA
XM_015098990.1	GNA13	mRNA	XR_001436817.1	lncRNA
XM_015098990.1	GNA13	mRNA	XR_001436817.1	lncRNA
XM_015098990.1	GNA13	mRNA	XR_001436817.1	lncRNA
XM_015092883.1	PADI1	mRNA	XR_001436817.1	lncRNA
XM_015092883.1	PADI1	mRNA	XR_001436817.1	lncRNA
XM_015092883.1	PADI1	mRNA	XR_001436817.1	lncRNA
XM_015092883.1	PADI1	mRNA	XR_001436817.1	lncRNA

XM_015092883.1	PADI1	mRNA	XR_001436817.1	lncRNA
XM_015092883.1	PADI1	mRNA	XR_001436817.1	lncRNA
XM_015092883.1	PADI1	mRNA	XR_001436817.1	lncRNA
XM_015092883.1	PADI1	mRNA	XR_001436817.1	lncRNA
XM_015092883.1	PADI1	mRNA	XR_001436817.1	lncRNA
XM_015098990.1	GNA13	mRNA	XR_001434203.1	lncRNA
XM_015098990.1	GNA13	mRNA	XR_001434203.1	lncRNA
XM_015098990.1	GNA13	mRNA	XR_001434203.1	lncRNA
XM_015098990.1	GNA13	mRNA	XR_001434203.1	lncRNA
XM_015098990.1	GNA13	mRNA	XR_001434203.1	lncRNA
XM_015098990.1	GNA13	mRNA	XR_001434203.1	lncRNA
XM_015098990.1	GNA13	mRNA	XR_001434203.1	lncRNA
XM_015098990.1	GNA13	mRNA	XR_001434203.1	lncRNA
XM_015098990.1	GNA13	mRNA	XR_001434203.1	lncRNA
XM_015092883.1	PADI1	mRNA	XR_001434203.1	lncRNA
XM_015092883.1	PADI1	mRNA	XR_001434203.1	lncRNA
XM_015092883.1	PADI1	mRNA	XR_001434203.1	lncRNA
XM_015092883.1	PADI1	mRNA	XR_001434203.1	lncRNA
XM_015092883.1	PADI1	mRNA	XR_001434203.1	lncRNA
XM_015092883.1	PADI1	mRNA	XR_001434203.1	lncRNA
XM_015092883.1	PADI1	mRNA	XR_001434203.1	lncRNA
XM_015092883.1	PADI1	mRNA	XR_001434203.1	lncRNA
XM_015092883.1	PADI1	mRNA	XR_001434203.1	lncRNA
XM_012144682.2	MAP2	mRNA	TCONS_00022613	lncRNA
XM_012144682.2	MAP2	mRNA	TCONS_00022613	lncRNA
XM_012144682.2	MAP2	mRNA	TCONS_00022613	lncRNA
XM_012144682.2	MAP2	mRNA	TCONS_00022613	lncRNA
XM_012144682.2	MAP2	mRNA	TCONS_00022613	lncRNA
XM_012144682.2	MAP2	mRNA	TCONS_00022613	lncRNA
XM_012144682.2	MAP2	mRNA	TCONS_00022613	lncRNA
XM_012144682.2	MAP2	mRNA	TCONS_00022613	lncRNA
XM_012105332.2	USP54	mRNA	XR_001434761.1	lncRNA
XM_012105332.2	USP54	mRNA	XR_001434761.1	lncRNA
XM_012105332.2	USP54	mRNA	XR_001434761.1	lncRNA
XM_012105332.2	USP54	mRNA	XR_001434761.1	lncRNA
XM_012105332.2	USP54	mRNA	XR_001434761.1	lncRNA
XM_012105332.2	USP54	mRNA	XR_001434761.1	lncRNA
XM_012105332.2	USP54	mRNA	XR_001434761.1	lncRNA
XM_012105332.2	USP54	mRNA	XR_001434761.1	lncRNA
XM_012105332.2	USP54	mRNA	XR_001434761.1	lncRNA
XM_004011865.3	ENPP2	mRNA	XR_001434761.1	lncRNA
XM_004011865.3	ENPP2	mRNA	XR_001434761.1	lncRNA
XM_004011865.3	ENPP2	mRNA	XR_001434761.1	lncRNA
XM_004011865.3	ENPP2	mRNA	XR_001434761.1	lncRNA
XM_004011865.3	ENPP2	mRNA	XR_001434761.1	lncRNA
XM_004011865.3	ENPP2	mRNA	XR_001434761.1	lncRNA
XM_004011865.3	ENPP2	mRNA	XR_001434761.1	lncRNA
XM_004011865.3	ENPP2	mRNA	XR_001434761.1	lncRNA
XM_004011865.3	ENPP2	mRNA	XR_001434761.1	lncRNA
XM_012188265.2	CCDC7	mRNA	XR_001042381.2	lncRNA
XM_012188265.2	CCDC7	mRNA	XR_001042381.2	lncRNA
XM_012188265.2	CCDC7	mRNA	XR_001042381.2	lncRNA
XM_012188265.2	CCDC7	mRNA	XR_001042381.2	lncRNA
XM_012188265.2	CCDC7	mRNA	XR_001042381.2	lncRNA

XM_012188265.2	CCDC7	mRNA	XR_001042381.2	lncRNA
XM_012188265.2	CCDC7	mRNA	XR_001042381.2	lncRNA
XM_012188265.2	CCDC7	mRNA	XR_001042381.2	lncRNA
XM_015098766.1	WIPF2	mRNA	XR_001042381.2	lncRNA
XM_015098766.1	WIPF2	mRNA	XR_001042381.2	lncRNA
XM_015098766.1	WIPF2	mRNA	XR_001042381.2	lncRNA
XM_015098766.1	WIPF2	mRNA	XR_001042381.2	lncRNA
XM_015098766.1	WIPF2	mRNA	XR_001042381.2	lncRNA
XM_015098766.1	WIPF2	mRNA	XR_001042381.2	lncRNA
XM_015098766.1	WIPF2	mRNA	XR_001042381.2	lncRNA
XM_015098766.1	WIPF2	mRNA	XR_001042381.2	lncRNA
XM_015104323.1	SPRTN	mRNA	XR_001436532.1	lncRNA
XM_015104323.1	SPRTN	mRNA	XR_001436532.1	lncRNA
XM_015104323.1	SPRTN	mRNA	XR_001436532.1	lncRNA
XM_015104323.1	SPRTN	mRNA	XR_001436532.1	lncRNA
XM_015104323.1	SPRTN	mRNA	XR_001436532.1	lncRNA
XM_015104323.1	SPRTN	mRNA	XR_001436532.1	lncRNA
XM_015104323.1	SPRTN	mRNA	XR_001436532.1	lncRNA
XM_015104323.1	SPRTN	mRNA	XR_001436532.1	lncRNA
XM_015096972.1	L2HGDH	mRNA	XR_001436532.1	lncRNA
XM_015096972.1	L2HGDH	mRNA	XR_001436532.1	lncRNA
XM_015096972.1	L2HGDH	mRNA	XR_001436532.1	lncRNA
XM_015096972.1	L2HGDH	mRNA	XR_001436532.1	lncRNA
XM_015096972.1	L2HGDH	mRNA	XR_001436532.1	lncRNA
XM_015096972.1	L2HGDH	mRNA	XR_001436532.1	lncRNA
XM_015096972.1	L2HGDH	mRNA	XR_001436532.1	lncRNA
XM_015096972.1	L2HGDH	mRNA	XR_001436532.1	lncRNA
XM_012094704.2	LOC10560198	mRNA	XR_001436532.1	lncRNA
XM_012094704.2	LOC10560198	mRNA	XR_001436532.1	lncRNA
XM_012094704.2	LOC10560198	mRNA	XR_001436532.1	lncRNA
XM_012094704.2	LOC10560198	mRNA	XR_001436532.1	lncRNA
XM_012094704.2	LOC10560198	mRNA	XR_001436532.1	lncRNA
XM_012094704.2	LOC10560198	mRNA	XR_001436532.1	lncRNA
XM_012094704.2	LOC10560198	mRNA	XR_001436532.1	lncRNA
XM_012094704.2	LOC10560198	mRNA	XR_001436532.1	lncRNA
XM_015104471.1	CCSER2	mRNA	XR_001436532.1	lncRNA
XM_015104471.1	CCSER2	mRNA	XR_001436532.1	lncRNA
XM_015104471.1	CCSER2	mRNA	XR_001436532.1	lncRNA
XM_015104471.1	CCSER2	mRNA	XR_001436532.1	lncRNA
XM_015104471.1	CCSER2	mRNA	XR_001436532.1	lncRNA
XM_015104471.1	CCSER2	mRNA	XR_001436532.1	lncRNA
XM_015104471.1	CCSER2	mRNA	XR_001436532.1	lncRNA
XM_015104471.1	CCSER2	mRNA	XR_001436532.1	lncRNA
XM_015103641.1	YES1	mRNA	XR_001436532.1	lncRNA
XM_015103641.1	YES1	mRNA	XR_001436532.1	lncRNA
XM_015103641.1	YES1	mRNA	XR_001436532.1	lncRNA
XM_015103641.1	YES1	mRNA	XR_001436532.1	lncRNA
XM_015103641.1	YES1	mRNA	XR_001436532.1	lncRNA
XM_015103641.1	YES1	mRNA	XR_001436532.1	lncRNA
XM_015103641.1	YES1	mRNA	XR_001436532.1	lncRNA
XM_015103641.1	YES1	mRNA	XR_001436532.1	lncRNA
XM_012184456.2	FNDC3A	mRNA	XR_001042381.2	lncRNA
XM_012184456.2	FNDC3A	mRNA	XR_001042381.2	lncRNA
XM_012184456.2	FNDC3A	mRNA	XR_001042381.2	lncRNA

XM_012184456.2	FNDC3A	mRNA	XR_001042381.2	lncRNA
XM_012184456.2	FNDC3A	mRNA	XR_001042381.2	lncRNA
XM_012184456.2	FNDC3A	mRNA	XR_001042381.2	lncRNA
XM_012184456.2	FNDC3A	mRNA	XR_001042381.2	lncRNA
XM_012184456.2	FNDC3A	mRNA	XR_001042381.2	lncRNA
XM_012187417.2	PANK4	mRNA	XR_001042381.2	lncRNA
XM_012187417.2	PANK4	mRNA	XR_001042381.2	lncRNA
XM_012187417.2	PANK4	mRNA	XR_001042381.2	lncRNA
XM_012187417.2	PANK4	mRNA	XR_001042381.2	lncRNA
XM_012187417.2	PANK4	mRNA	XR_001042381.2	lncRNA
XM_012187417.2	PANK4	mRNA	XR_001042381.2	lncRNA
XM_012187417.2	PANK4	mRNA	XR_001042381.2	lncRNA
XM_012187417.2	PANK4	mRNA	XR_001042381.2	lncRNA
XM_004022347.2	TMEM255A	mRNA	XR_001436532.1	lncRNA
XM_004022347.2	TMEM255A	mRNA	XR_001436532.1	lncRNA
XM_004022347.2	TMEM255A	mRNA	XR_001436532.1	lncRNA
XM_004022347.2	TMEM255A	mRNA	XR_001436532.1	lncRNA
XM_004022347.2	TMEM255A	mRNA	XR_001436532.1	lncRNA
XM_004022347.2	TMEM255A	mRNA	XR_001436532.1	lncRNA
XM_004022347.2	TMEM255A	mRNA	XR_001436532.1	lncRNA
XM_004022347.2	TMEM255A	mRNA	XR_001436532.1	lncRNA
XM_015095875.1	SAFB	mRNA	XR_001436532.1	lncRNA
XM_015095875.1	SAFB	mRNA	XR_001436532.1	lncRNA
XM_015095875.1	SAFB	mRNA	XR_001436532.1	lncRNA
XM_015095875.1	SAFB	mRNA	XR_001436532.1	lncRNA
XM_015095875.1	SAFB	mRNA	XR_001436532.1	lncRNA
XM_015095875.1	SAFB	mRNA	XR_001436532.1	lncRNA
XM_015095875.1	SAFB	mRNA	XR_001436532.1	lncRNA
XM_015095875.1	SAFB	mRNA	XR_001436532.1	lncRNA
XM_015104296.1	ADAMTS14	mRNA	XR_001025491.1	lncRNA
XM_015104296.1	ADAMTS14	mRNA	XR_001025491.1	lncRNA
XM_015104296.1	ADAMTS14	mRNA	XR_001025491.1	lncRNA
XM_015104296.1	ADAMTS14	mRNA	XR_001025491.1	lncRNA
XM_015104296.1	ADAMTS14	mRNA	XR_001025491.1	lncRNA
XM_015104296.1	ADAMTS14	mRNA	XR_001025491.1	lncRNA
XM_015104296.1	ADAMTS14	mRNA	XR_001025491.1	lncRNA
XM_015104296.1	ADAMTS14	mRNA	XR_001025491.1	lncRNA
XM_015104296.1	ADAMTS14	mRNA	XR_001025491.1	lncRNA
XM_012184884.2	DOCK9	mRNA	TCONS_00075453	lncRNA
XM_012184884.2	DOCK9	mRNA	TCONS_00075453	lncRNA
XM_012184884.2	DOCK9	mRNA	TCONS_00075453	lncRNA
XM_012184884.2	DOCK9	mRNA	TCONS_00075453	lncRNA
XM_012184884.2	DOCK9	mRNA	TCONS_00075453	lncRNA
XM_012184884.2	DOCK9	mRNA	TCONS_00075453	lncRNA
XM_012184884.2	DOCK9	mRNA	TCONS_00075453	lncRNA
XM_012184884.2	DOCK9	mRNA	TCONS_00075453	lncRNA
XM_012184885.2	DOCK9	mRNA	TCONS_00075453	lncRNA
XM_012184885.2	DOCK9	mRNA	TCONS_00075453	lncRNA
XM_012184885.2	DOCK9	mRNA	TCONS_00075453	lncRNA
XM_012184885.2	DOCK9	mRNA	TCONS_00075453	lncRNA
XM_012184885.2	DOCK9	mRNA	TCONS_00075453	lncRNA
XM_012184885.2	DOCK9	mRNA	TCONS_00075453	lncRNA
XM_012184885.2	DOCK9	mRNA	TCONS_00075453	lncRNA
XM_012184885.2	DOCK9	mRNA	TCONS_00075453	lncRNA

XM_012185318.2	RAD51D	mRNA	XR_001436102.1	lncRNA
XM_012185318.2	RAD51D	mRNA	XR_001436102.1	lncRNA
XM_012185318.2	RAD51D	mRNA	XR_001436102.1	lncRNA
XM_012185318.2	RAD51D	mRNA	XR_001436102.1	lncRNA
XM_012185318.2	RAD51D	mRNA	XR_001436102.1	lncRNA
XM_012185318.2	RAD51D	mRNA	XR_001436102.1	lncRNA
XM_012185318.2	RAD51D	mRNA	XR_001436102.1	lncRNA
XM_012185318.2	RAD51D	mRNA	XR_001436102.1	lncRNA
XM_012185318.2	RAD51D	mRNA	XR_001436102.1	lncRNA
XM_012181456.2	ADAM10	mRNA	XR_001436102.1	lncRNA
XM_012181456.2	ADAM10	mRNA	XR_001436102.1	lncRNA
XM_012181456.2	ADAM10	mRNA	XR_001436102.1	lncRNA
XM_012181456.2	ADAM10	mRNA	XR_001436102.1	lncRNA
XM_012181456.2	ADAM10	mRNA	XR_001436102.1	lncRNA
XM_012181456.2	ADAM10	mRNA	XR_001436102.1	lncRNA
XM_012181456.2	ADAM10	mRNA	XR_001436102.1	lncRNA
XM_012181456.2	ADAM10	mRNA	XR_001436102.1	lncRNA
XM_012181456.2	ADAM10	mRNA	XR_001436102.1	lncRNA
XM_012099283.2	NEK10	mRNA	XR_001041211.2	lncRNA
XM_012099283.2	NEK10	mRNA	XR_001041211.2	lncRNA
XM_012099283.2	NEK10	mRNA	XR_001041211.2	lncRNA
XM_012099283.2	NEK10	mRNA	XR_001041211.2	lncRNA
XM_012099283.2	NEK10	mRNA	XR_001041211.2	lncRNA
XM_012099283.2	NEK10	mRNA	XR_001041211.2	lncRNA
XM_012099283.2	NEK10	mRNA	XR_001041211.2	lncRNA
XM_012099283.2	NEK10	mRNA	XR_001041211.2	lncRNA
XM_015092228.1	CIART	mRNA	XR_001041211.2	lncRNA
XM_015092228.1	CIART	mRNA	XR_001041211.2	lncRNA
XM_015092228.1	CIART	mRNA	XR_001041211.2	lncRNA
XM_015092228.1	CIART	mRNA	XR_001041211.2	lncRNA
XM_015092228.1	CIART	mRNA	XR_001041211.2	lncRNA
XM_015092228.1	CIART	mRNA	XR_001041211.2	lncRNA
XM_015092228.1	CIART	mRNA	XR_001041211.2	lncRNA
XM_015092228.1	CIART	mRNA	XR_001041211.2	lncRNA
XM_015104323.1	SPRTN	mRNA	XR_001041211.2	lncRNA
XM_015104323.1	SPRTN	mRNA	XR_001041211.2	lncRNA
XM_015104323.1	SPRTN	mRNA	XR_001041211.2	lncRNA
XM_015104323.1	SPRTN	mRNA	XR_001041211.2	lncRNA
XM_015104323.1	SPRTN	mRNA	XR_001041211.2	lncRNA
XM_015104323.1	SPRTN	mRNA	XR_001041211.2	lncRNA
XM_015104323.1	SPRTN	mRNA	XR_001041211.2	lncRNA
XM_015104323.1	SPRTN	mRNA	XR_001041211.2	lncRNA
XM_004022347.2	TMEM255A	mRNA	XR_001041211.2	lncRNA
XM_004022347.2	TMEM255A	mRNA	XR_001041211.2	lncRNA
XM_004022347.2	TMEM255A	mRNA	XR_001041211.2	lncRNA
XM_004022347.2	TMEM255A	mRNA	XR_001041211.2	lncRNA
XM_004022347.2	TMEM255A	mRNA	XR_001041211.2	lncRNA
XM_004022347.2	TMEM255A	mRNA	XR_001041211.2	lncRNA
XM_004022347.2	TMEM255A	mRNA	XR_001041211.2	lncRNA
XM_004022347.2	TMEM255A	mRNA	XR_001041211.2	lncRNA
XM_015098592.1	RABEP1	mRNA	XR_001033284.2	lncRNA
XM_015098592.1	RABEP1	mRNA	XR_001033284.2	lncRNA
XM_015098592.1	RABEP1	mRNA	XR_001033284.2	lncRNA
XM_015098592.1	RABEP1	mRNA	XR_001033284.2	lncRNA

XM_015098592.1	RABEP1	mRNA	XR_001033284.2	lncRNA
XM_015098592.1	RABEP1	mRNA	XR_001033284.2	lncRNA
XM_015098592.1	RABEP1	mRNA	XR_001033284.2	lncRNA
XM_015098592.1	RABEP1	mRNA	XR_001033284.2	lncRNA
XM_015092323.1	KIRREL	mRNA	TCONS_00022613	lncRNA
XM_015092323.1	KIRREL	mRNA	TCONS_00022613	lncRNA
XM_015092323.1	KIRREL	mRNA	TCONS_00022613	lncRNA
XM_015092323.1	KIRREL	mRNA	TCONS_00022613	lncRNA
XM_015092323.1	KIRREL	mRNA	TCONS_00022613	lncRNA
XM_015092323.1	KIRREL	mRNA	TCONS_00022613	lncRNA
XM_015092323.1	KIRREL	mRNA	TCONS_00022613	lncRNA
XM_015092323.1	KIRREL	mRNA	TCONS_00022613	lncRNA
XM_015098660.1	MAP2K4	mRNA	XR_001044126.1	lncRNA
XM_015098660.1	MAP2K4	mRNA	XR_001044126.1	lncRNA
XM_015098660.1	MAP2K4	mRNA	XR_001044126.1	lncRNA
XM_015098660.1	MAP2K4	mRNA	XR_001044126.1	lncRNA
XM_015098660.1	MAP2K4	mRNA	XR_001044126.1	lncRNA
XM_015098660.1	MAP2K4	mRNA	XR_001044126.1	lncRNA
XM_015098660.1	MAP2K4	mRNA	XR_001044126.1	lncRNA
XM_015098660.1	MAP2K4	mRNA	XR_001044126.1	lncRNA
XM_004017713.3	LINS1	mRNA	XR_001024290.1	lncRNA
XM_004017713.3	LINS1	mRNA	XR_001024290.1	lncRNA
XM_004017713.3	LINS1	mRNA	XR_001024290.1	lncRNA
XM_004017713.3	LINS1	mRNA	XR_001024290.1	lncRNA
XM_004017713.3	LINS1	mRNA	XR_001024290.1	lncRNA
XM_004017713.3	LINS1	mRNA	XR_001024290.1	lncRNA
XM_004017713.3	LINS1	mRNA	XR_001024290.1	lncRNA
XM_012184823.2	RBM26	mRNA	XR_001436102.1	lncRNA
XM_012184823.2	RBM26	mRNA	XR_001436102.1	lncRNA
XM_012184823.2	RBM26	mRNA	XR_001436102.1	lncRNA
XM_012184823.2	RBM26	mRNA	XR_001436102.1	lncRNA
XM_012184823.2	RBM26	mRNA	XR_001436102.1	lncRNA
XM_012184823.2	RBM26	mRNA	XR_001436102.1	lncRNA
XM_012184823.2	RBM26	mRNA	XR_001436102.1	lncRNA
XM_012184823.2	RBM26	mRNA	XR_001436102.1	lncRNA
XM_012134956.1	STK39	mRNA	XR_001436102.1	lncRNA
XM_012134956.1	STK39	mRNA	XR_001436102.1	lncRNA
XM_012134956.1	STK39	mRNA	XR_001436102.1	lncRNA
XM_012134956.1	STK39	mRNA	XR_001436102.1	lncRNA
XM_012134956.1	STK39	mRNA	XR_001436102.1	lncRNA
XM_012134956.1	STK39	mRNA	XR_001436102.1	lncRNA
XM_012134956.1	STK39	mRNA	XR_001436102.1	lncRNA
XM_012134956.1	STK39	mRNA	XR_001436102.1	lncRNA
XM_012184401.1	ZC3H13	mRNA	XR_001436102.1	lncRNA
XM_012184401.1	ZC3H13	mRNA	XR_001436102.1	lncRNA
XM_012184401.1	ZC3H13	mRNA	XR_001436102.1	lncRNA
XM_012184401.1	ZC3H13	mRNA	XR_001436102.1	lncRNA
XM_012184401.1	ZC3H13	mRNA	XR_001436102.1	lncRNA
XM_012184401.1	ZC3H13	mRNA	XR_001436102.1	lncRNA
XM_012184401.1	ZC3H13	mRNA	XR_001436102.1	lncRNA
XM_012184401.1	ZC3H13	mRNA	XR_001436102.1	lncRNA
XM_012095439.2	SIK2	mRNA	XR_001436102.1	lncRNA
XM_012095439.2	SIK2	mRNA	XR_001436102.1	lncRNA
XM_012095439.2	SIK2	mRNA	XR_001436102.1	lncRNA

XM_012095439.2	SIK2	mRNA	XR_001436102.1	lncRNA
XM_012095439.2	SIK2	mRNA	XR_001436102.1	lncRNA
XM_012095439.2	SIK2	mRNA	XR_001436102.1	lncRNA
XM_012095439.2	SIK2	mRNA	XR_001436102.1	lncRNA
XM_012095439.2	SIK2	mRNA	XR_001436102.1	lncRNA
XM_012097367.2	SH3D19	mRNA	XR_001021689.2	lncRNA
XM_012097367.2	SH3D19	mRNA	XR_001021689.2	lncRNA
XM_012097367.2	SH3D19	mRNA	XR_001021689.2	lncRNA
XM_012097367.2	SH3D19	mRNA	XR_001021689.2	lncRNA
XM_012097367.2	SH3D19	mRNA	XR_001021689.2	lncRNA
XM_012097367.2	SH3D19	mRNA	XR_001021689.2	lncRNA
XM_012097367.2	SH3D19	mRNA	XR_001021689.2	lncRNA
XM_012097367.2	SH3D19	mRNA	XR_001021689.2	lncRNA
XM_012127663.2	IFT74	mRNA	XR_001434203.1	lncRNA
XM_012127663.2	IFT74	mRNA	XR_001434203.1	lncRNA
XM_012127663.2	IFT74	mRNA	XR_001434203.1	lncRNA
XM_012127663.2	IFT74	mRNA	XR_001434203.1	lncRNA
XM_012127663.2	IFT74	mRNA	XR_001434203.1	lncRNA
XM_012127663.2	IFT74	mRNA	XR_001434203.1	lncRNA
XM_012127663.2	IFT74	mRNA	XR_001434203.1	lncRNA
XM_012127663.2	IFT74	mRNA	XR_001434203.1	lncRNA
XM_012105261.2	MCU	mRNA	XR_001436817.1	lncRNA
XM_012105261.2	MCU	mRNA	XR_001436817.1	lncRNA
XM_012105261.2	MCU	mRNA	XR_001436817.1	lncRNA
XM_012105261.2	MCU	mRNA	XR_001436817.1	lncRNA
XM_012105261.2	MCU	mRNA	XR_001436817.1	lncRNA
XM_012105261.2	MCU	mRNA	XR_001436817.1	lncRNA
XM_012105261.2	MCU	mRNA	XR_001436817.1	lncRNA
XM_012105261.2	MCU	mRNA	XR_001436817.1	lncRNA
XM_015099971.1	ZNF181	mRNA	XR_001436817.1	lncRNA
XM_015099971.1	ZNF181	mRNA	XR_001436817.1	lncRNA
XM_015099971.1	ZNF181	mRNA	XR_001436817.1	lncRNA
XM_015099971.1	ZNF181	mRNA	XR_001436817.1	lncRNA
XM_015099971.1	ZNF181	mRNA	XR_001436817.1	lncRNA
XM_015099971.1	ZNF181	mRNA	XR_001436817.1	lncRNA
XM_015099971.1	ZNF181	mRNA	XR_001436817.1	lncRNA
XM_015099971.1	ZNF181	mRNA	XR_001436817.1	lncRNA
XM_012104225.2	DCUN1D3	mRNA	XR_001434203.1	lncRNA
XM_012104225.2	DCUN1D3	mRNA	XR_001434203.1	lncRNA
XM_012104225.2	DCUN1D3	mRNA	XR_001434203.1	lncRNA
XM_012104225.2	DCUN1D3	mRNA	XR_001434203.1	lncRNA
XM_012104225.2	DCUN1D3	mRNA	XR_001434203.1	lncRNA
XM_012104225.2	DCUN1D3	mRNA	XR_001434203.1	lncRNA
XM_012104225.2	DCUN1D3	mRNA	XR_001434203.1	lncRNA
XM_012104225.2	DCUN1D3	mRNA	XR_001434203.1	lncRNA
XM_012149251.2	S100PBP	mRNA	XR_001436817.1	lncRNA
XM_012149251.2	S100PBP	mRNA	XR_001436817.1	lncRNA
XM_012149251.2	S100PBP	mRNA	XR_001436817.1	lncRNA
XM_012149251.2	S100PBP	mRNA	XR_001436817.1	lncRNA
XM_012149251.2	S100PBP	mRNA	XR_001436817.1	lncRNA
XM_012149251.2	S100PBP	mRNA	XR_001436817.1	lncRNA
XM_012149251.2	S100PBP	mRNA	XR_001436817.1	lncRNA
XM_012149251.2	S100PBP	mRNA	XR_001436817.1	lncRNA
XM_012094704.2	LOC10560198[illegible]	mRNA	XR_001436817.1	lncRNA

XM_012094704.2	LOC10560198[illegible]	mRNA	XR_001436817.1	lncRNA
XM_012094704.2	LOC10560198[illegible]	mRNA	XR_001436817.1	lncRNA
XM_012094704.2	LOC10560198[illegible]	mRNA	XR_001436817.1	lncRNA
XM_012094704.2	LOC10560198[illegible]	mRNA	XR_001436817.1	lncRNA
XM_012094704.2	LOC10560198[illegible]	mRNA	XR_001436817.1	lncRNA
XM_012094704.2	LOC10560198[illegible]	mRNA	XR_001436817.1	lncRNA
XM_012094704.2	LOC10560198[illegible]	mRNA	XR_001436817.1	lncRNA
XM_012184148.1	OTUL3B	mRNA	XR_001436817.1	lncRNA
XM_012184148.1	OTUL3B	mRNA	XR_001436817.1	lncRNA
XM_012184148.1	OTUL3B	mRNA	XR_001436817.1	lncRNA
XM_012184148.1	OTUL3B	mRNA	XR_001436817.1	lncRNA
XM_012184148.1	OTUL3B	mRNA	XR_001436817.1	lncRNA
XM_012184148.1	OTUL3B	mRNA	XR_001436817.1	lncRNA
XM_012184148.1	OTUL3B	mRNA	XR_001436817.1	lncRNA
XM_012184148.1	OTUL3B	mRNA	XR_001436817.1	lncRNA
XM_015095875.1	SAFB	mRNA	XR_001436817.1	lncRNA
XM_015095875.1	SAFB	mRNA	XR_001436817.1	lncRNA
XM_015095875.1	SAFB	mRNA	XR_001436817.1	lncRNA
XM_015095875.1	SAFB	mRNA	XR_001436817.1	lncRNA
XM_015095875.1	SAFB	mRNA	XR_001436817.1	lncRNA
XM_015095875.1	SAFB	mRNA	XR_001436817.1	lncRNA
XM_015095875.1	SAFB	mRNA	XR_001436817.1	lncRNA
XM_015095875.1	SAFB	mRNA	XR_001436817.1	lncRNA
XM_004010212.3	MTX3	mRNA	XR_001436817.1	lncRNA
XM_004010212.3	MTX3	mRNA	XR_001436817.1	lncRNA
XM_004010212.3	MTX3	mRNA	XR_001436817.1	lncRNA
XM_004010212.3	MTX3	mRNA	XR_001436817.1	lncRNA
XM_004010212.3	MTX3	mRNA	XR_001436817.1	lncRNA
XM_004010212.3	MTX3	mRNA	XR_001436817.1	lncRNA
XM_004010212.3	MTX3	mRNA	XR_001436817.1	lncRNA
XM_004010212.3	MTX3	mRNA	XR_001436817.1	lncRNA
XM_012187417.2	PANK4	mRNA	XR_001436817.1	lncRNA
XM_012187417.2	PANK4	mRNA	XR_001436817.1	lncRNA
XM_012187417.2	PANK4	mRNA	XR_001436817.1	lncRNA
XM_012187417.2	PANK4	mRNA	XR_001436817.1	lncRNA
XM_012187417.2	PANK4	mRNA	XR_001436817.1	lncRNA
XM_012187417.2	PANK4	mRNA	XR_001436817.1	lncRNA
XM_012187417.2	PANK4	mRNA	XR_001436817.1	lncRNA
XM_012187417.2	PANK4	mRNA	XR_001436817.1	lncRNA
XM_012184456.2	FNDC3A	mRNA	XR_001436817.1	lncRNA
XM_012184456.2	FNDC3A	mRNA	XR_001436817.1	lncRNA
XM_012184456.2	FNDC3A	mRNA	XR_001436817.1	lncRNA
XM_012184456.2	FNDC3A	mRNA	XR_001436817.1	lncRNA
XM_012184456.2	FNDC3A	mRNA	XR_001436817.1	lncRNA
XM_012184456.2	FNDC3A	mRNA	XR_001436817.1	lncRNA
XM_012184456.2	FNDC3A	mRNA	XR_001436817.1	lncRNA
XM_012184456.2	FNDC3A	mRNA	XR_001436817.1	lncRNA
XM_012105261.2	MCU	mRNA	XR_001434203.1	lncRNA
XM_012105261.2	MCU	mRNA	XR_001434203.1	lncRNA
XM_012105261.2	MCU	mRNA	XR_001434203.1	lncRNA
XM_012105261.2	MCU	mRNA	XR_001434203.1	lncRNA
XM_012105261.2	MCU	mRNA	XR_001434203.1	lncRNA
XM_012105261.2	MCU	mRNA	XR_001434203.1	lncRNA
XM_012105261.2	MCU	mRNA	XR_001434203.1	lncRNA

XM_012105261.2	MCU	mRNA	XR_001434203.1	lncRNA
XM_012104225.2	DCUN1D3	mRNA	XR_001436817.1	lncRNA
XM_012104225.2	DCUN1D3	mRNA	XR_001436817.1	lncRNA
XM_012104225.2	DCUN1D3	mRNA	XR_001436817.1	lncRNA
XM_012104225.2	DCUN1D3	mRNA	XR_001436817.1	lncRNA
XM_012104225.2	DCUN1D3	mRNA	XR_001436817.1	lncRNA
XM_012104225.2	DCUN1D3	mRNA	XR_001436817.1	lncRNA
XM_012104225.2	DCUN1D3	mRNA	XR_001436817.1	lncRNA
XM_012104225.2	DCUN1D3	mRNA	XR_001436817.1	lncRNA
XM_012188945.2	CSTF1	mRNA	XR_001436817.1	lncRNA
XM_012188945.2	CSTF1	mRNA	XR_001436817.1	lncRNA
XM_012188945.2	CSTF1	mRNA	XR_001436817.1	lncRNA
XM_012188945.2	CSTF1	mRNA	XR_001436817.1	lncRNA
XM_012188945.2	CSTF1	mRNA	XR_001436817.1	lncRNA
XM_012188945.2	CSTF1	mRNA	XR_001436817.1	lncRNA
XM_012188945.2	CSTF1	mRNA	XR_001436817.1	lncRNA
XM_012188945.2	CSTF1	mRNA	XR_001436817.1	lncRNA
XM_012127663.2	IFT74	mRNA	XR_001436817.1	lncRNA
XM_012127663.2	IFT74	mRNA	XR_001436817.1	lncRNA
XM_012127663.2	IFT74	mRNA	XR_001436817.1	lncRNA
XM_012127663.2	IFT74	mRNA	XR_001436817.1	lncRNA
XM_012127663.2	IFT74	mRNA	XR_001436817.1	lncRNA
XM_012127663.2	IFT74	mRNA	XR_001436817.1	lncRNA
XM_012127663.2	IFT74	mRNA	XR_001436817.1	lncRNA
XM_012127663.2	IFT74	mRNA	XR_001436817.1	lncRNA
XM_004004866.2	METTL21A	mRNA	XR_001436817.1	lncRNA
XM_004004866.2	METTL21A	mRNA	XR_001436817.1	lncRNA
XM_004004866.2	METTL21A	mRNA	XR_001436817.1	lncRNA
XM_004004866.2	METTL21A	mRNA	XR_001436817.1	lncRNA
XM_004004866.2	METTL21A	mRNA	XR_001436817.1	lncRNA
XM_004004866.2	METTL21A	mRNA	XR_001436817.1	lncRNA
XM_004004866.2	METTL21A	mRNA	XR_001436817.1	lncRNA
XM_004004866.2	METTL21A	mRNA	XR_001436817.1	lncRNA
XM_004015967.3	DYNC2H1	mRNA	XR_001434761.1	lncRNA
XM_004015967.3	DYNC2H1	mRNA	XR_001434761.1	lncRNA
XM_004015967.3	DYNC2H1	mRNA	XR_001434761.1	lncRNA
XM_004015967.3	DYNC2H1	mRNA	XR_001434761.1	lncRNA
XM_004015967.3	DYNC2H1	mRNA	XR_001434761.1	lncRNA
XM_004015967.3	DYNC2H1	mRNA	XR_001434761.1	lncRNA
XM_004015967.3	DYNC2H1	mRNA	XR_001434761.1	lncRNA
XM_004015967.3	DYNC2H1	mRNA	XR_001434761.1	lncRNA
XM_012175276.1	PLCM1	mRNA	XR_001434892.1	lncRNA
XM_012175276.1	PLCM1	mRNA	XR_001434892.1	lncRNA
XM_012175276.1	PLCM1	mRNA	XR_001434892.1	lncRNA
XM_012175276.1	PLCM1	mRNA	XR_001434892.1	lncRNA
XM_012175276.1	PLCM1	mRNA	XR_001434892.1	lncRNA
XM_012175276.1	PLCM1	mRNA	XR_001434892.1	lncRNA
XM_012175276.1	PLCM1	mRNA	XR_001434892.1	lncRNA
XM_012175276.1	PLCM1	mRNA	XR_001434892.1	lncRNA
XM_015094913.1	PLCM1	mRNA	TCONS_00022613	lncRNA
XM_015094913.1	PLCM1	mRNA	TCONS_00022613	lncRNA
XM_015094913.1	PLCM1	mRNA	TCONS_00022613	lncRNA
XM_015094913.1	PLCM1	mRNA	TCONS_00022613	lncRNA
XM_015094913.1	PLCM1	mRNA	TCONS_00022613	lncRNA

XM_015094913.1	PLCM1	mRNA	TCONS_00022613	lncRNA
XM_015094913.1	PLCM1	mRNA	TCONS_00022613	lncRNA
XM_012102845.2	GBF1	mRNA	XR_001020707.2	lncRNA
XM_012102845.2	GBF1	mRNA	XR_001020707.2	lncRNA
XM_012102845.2	GBF1	mRNA	XR_001020707.2	lncRNA
XM_012102845.2	GBF1	mRNA	XR_001020707.2	lncRNA
XM_012102845.2	GBF1	mRNA	XR_001020707.2	lncRNA
XM_012102845.2	GBF1	mRNA	XR_001020707.2	lncRNA
XM_012102845.2	GBF1	mRNA	XR_001020707.2	lncRNA
XM_012102845.2	GBF1	mRNA	XR_001020707.2	lncRNA
XM_012105080.2	BICC1	mRNA	XR_001020707.2	lncRNA
XM_012105080.2	BICC1	mRNA	XR_001020707.2	lncRNA
XM_012105080.2	BICC1	mRNA	XR_001020707.2	lncRNA
XM_012105080.2	BICC1	mRNA	XR_001020707.2	lncRNA
XM_012105080.2	BICC1	mRNA	XR_001020707.2	lncRNA
XM_012105080.2	BICC1	mRNA	XR_001020707.2	lncRNA
XM_012105080.2	BICC1	mRNA	XR_001020707.2	lncRNA
XM_012105080.2	BICC1	mRNA	XR_001020707.2	lncRNA
XM_012100690.1	KLC4	mRNA	XR_001020707.2	lncRNA
XM_012100690.1	KLC4	mRNA	XR_001020707.2	lncRNA
XM_012100690.1	KLC4	mRNA	XR_001020707.2	lncRNA
XM_012100690.1	KLC4	mRNA	XR_001020707.2	lncRNA
XM_012100690.1	KLC4	mRNA	XR_001020707.2	lncRNA
XM_012100690.1	KLC4	mRNA	XR_001020707.2	lncRNA
XM_012100690.1	KLC4	mRNA	XR_001020707.2	lncRNA
XM_012100690.1	KLC4	mRNA	XR_001020707.2	lncRNA
XM_012107472.1	NFYC	mRNA	XR_001434202.1	lncRNA
XM_012107472.1	NFYC	mRNA	XR_001434202.1	lncRNA
XM_012107472.1	NFYC	mRNA	XR_001434202.1	lncRNA
XM_012107472.1	NFYC	mRNA	XR_001434202.1	lncRNA
XM_012107472.1	NFYC	mRNA	XR_001434202.1	lncRNA
XM_012107472.1	NFYC	mRNA	XR_001434202.1	lncRNA
XM_012107472.1	NFYC	mRNA	XR_001434202.1	lncRNA
XM_012107472.1	NFYC	mRNA	XR_001434202.1	lncRNA
XM_004007700.3	CLDN12	mRNA	XR_001034847.2	lncRNA
XM_004007700.3	CLDN12	mRNA	XR_001034847.2	lncRNA
XM_004007700.3	CLDN12	mRNA	XR_001034847.2	lncRNA
XM_004007700.3	CLDN12	mRNA	XR_001034847.2	lncRNA
XM_004007700.3	CLDN12	mRNA	XR_001034847.2	lncRNA
XM_004007700.3	CLDN12	mRNA	XR_001034847.2	lncRNA
XM_004007700.3	CLDN12	mRNA	XR_001034847.2	lncRNA
XM_004007700.3	CLDN12	mRNA	XR_001034847.2	lncRNA
XM_004007700.3	CLDN12	mRNA	XR_001434202.1	lncRNA
XM_004007700.3	CLDN12	mRNA	XR_001434202.1	lncRNA
XM_004007700.3	CLDN12	mRNA	XR_001434202.1	lncRNA
XM_004007700.3	CLDN12	mRNA	XR_001434202.1	lncRNA
XM_004007700.3	CLDN12	mRNA	XR_001434202.1	lncRNA
XM_004007700.3	CLDN12	mRNA	XR_001434202.1	lncRNA
XM_004007700.3	CLDN12	mRNA	XR_001434202.1	lncRNA
XM_004007700.3	CLDN12	mRNA	XR_001434202.1	lncRNA
XM_012107472.1	NFYC	mRNA	XR_001034847.2	lncRNA
XM_012107472.1	NFYC	mRNA	XR_001034847.2	lncRNA
XM_012107472.1	NFYC	mRNA	XR_001034847.2	lncRNA
XM_012107472.1	NFYC	mRNA	XR_001034847.2	lncRNA

XM_012107472.1	NFYC	mRNA	XR_001034847.2	lncRNA
XM_012107472.1	NFYC	mRNA	XR_001034847.2	lncRNA
XM_012107472.1	NFYC	mRNA	XR_001034847.2	lncRNA
XM_012107472.1	NFYC	mRNA	XR_001034847.2	lncRNA
XM_004010577.2	CGNL1	mRNA	TCONS_00075453	lncRNA
XM_004010577.2	CGNL1	mRNA	TCONS_00075453	lncRNA
XM_004010577.2	CGNL1	mRNA	TCONS_00075453	lncRNA
XM_004010577.2	CGNL1	mRNA	TCONS_00075453	lncRNA
XM_004010577.2	CGNL1	mRNA	TCONS_00075453	lncRNA
XM_004010577.2	CGNL1	mRNA	TCONS_00075453	lncRNA
XM_004010577.2	CGNL1	mRNA	TCONS_00075453	lncRNA
XM_004010577.2	CGNL1	mRNA	TCONS_00075453	lncRNA
XM_015103475.1	PLEKHA1	mRNA	XR_001024290.1	lncRNA
XM_015103475.1	PLEKHA1	mRNA	XR_001024290.1	lncRNA
XM_015103475.1	PLEKHA1	mRNA	XR_001024290.1	lncRNA
XM_015103475.1	PLEKHA1	mRNA	XR_001024290.1	lncRNA
XM_015103475.1	PLEKHA1	mRNA	XR_001024290.1	lncRNA
XM_015103475.1	PLEKHA1	mRNA	XR_001024290.1	lncRNA
XM_015103475.1	PLEKHA1	mRNA	XR_001024290.1	lncRNA
XM_015103475.1	PLEKHA1	mRNA	XR_001024290.1	lncRNA
XM_004011865.3	ENPP2	mRNA	XR_001025491.1	lncRNA
XM_004011865.3	ENPP2	mRNA	XR_001025491.1	lncRNA
XM_004011865.3	ENPP2	mRNA	XR_001025491.1	lncRNA
XM_004011865.3	ENPP2	mRNA	XR_001025491.1	lncRNA
XM_004011865.3	ENPP2	mRNA	XR_001025491.1	lncRNA
XM_004011865.3	ENPP2	mRNA	XR_001025491.1	lncRNA
XM_004011865.3	ENPP2	mRNA	XR_001025491.1	lncRNA
XM_004011865.3	ENPP2	mRNA	XR_001025491.1	lncRNA
XM_012105319.2	USP54	mRNA	XR_001024290.1	lncRNA
XM_012105319.2	USP54	mRNA	XR_001024290.1	lncRNA
XM_012105319.2	USP54	mRNA	XR_001024290.1	lncRNA
XM_012105319.2	USP54	mRNA	XR_001024290.1	lncRNA
XM_012105319.2	USP54	mRNA	XR_001024290.1	lncRNA
XM_012105319.2	USP54	mRNA	XR_001024290.1	lncRNA
XM_012105319.2	USP54	mRNA	XR_001024290.1	lncRNA
XM_012105319.2	USP54	mRNA	XR_001024290.1	lncRNA
XM_012104181.2	CCP110	mRNA	XR_001044126.1	lncRNA
XM_012104181.2	CCP110	mRNA	XR_001044126.1	lncRNA
XM_012104181.2	CCP110	mRNA	XR_001044126.1	lncRNA
XM_012104181.2	CCP110	mRNA	XR_001044126.1	lncRNA
XM_012104181.2	CCP110	mRNA	XR_001044126.1	lncRNA
XM_012104181.2	CCP110	mRNA	XR_001044126.1	lncRNA
XM_012104181.2	CCP110	mRNA	XR_001044126.1	lncRNA
XM_012104181.2	CCP110	mRNA	XR_001044126.1	lncRNA
XM_015103448.1	TACC2	mRNA	TCONS_00022613	lncRNA
XM_015103448.1	TACC2	mRNA	TCONS_00022613	lncRNA
XM_015103448.1	TACC2	mRNA	TCONS_00022613	lncRNA
XM_015103448.1	TACC2	mRNA	TCONS_00022613	lncRNA
XM_015103448.1	TACC2	mRNA	TCONS_00022613	lncRNA
XM_015103448.1	TACC2	mRNA	TCONS_00022613	lncRNA
XM_015103448.1	TACC2	mRNA	TCONS_00022613	lncRNA
XM_015103448.1	TACC2	mRNA	TCONS_00022613	lncRNA
XM_012181247.2	MGA	mRNA	XR_001434892.1	lncRNA
XM_012181247.2	MGA	mRNA	XR_001434892.1	lncRNA

XM_012181247.2	MGA	mRNA	XR_001434892.1	lncRNA
XM_012181247.2	MGA	mRNA	XR_001434892.1	lncRNA
XM_012181247.2	MGA	mRNA	XR_001434892.1	lncRNA
XM_012181247.2	MGA	mRNA	XR_001434892.1	lncRNA
XM_012181247.2	MGA	mRNA	XR_001434892.1	lncRNA
XM_012181247.2	MGA	mRNA	XR_001434892.1	lncRNA
XM_012181247.2	MGA	mRNA	XR_001434892.1	lncRNA
XM_012176680.2	ARHGAP29	mRNA	XR_001020707.2	lncRNA
XM_012176680.2	ARHGAP29	mRNA	XR_001020707.2	lncRNA
XM_012176680.2	ARHGAP29	mRNA	XR_001020707.2	lncRNA
XM_012176680.2	ARHGAP29	mRNA	XR_001020707.2	lncRNA
XM_012176680.2	ARHGAP29	mRNA	XR_001020707.2	lncRNA
XM_012176680.2	ARHGAP29	mRNA	XR_001020707.2	lncRNA
XM_012176680.2	ARHGAP29	mRNA	XR_001020707.2	lncRNA
XM_012179696.2	LOC10111286	mRNA	XR_001033284.2	lncRNA
XM_012179696.2	LOC10111286	mRNA	XR_001033284.2	lncRNA
XM_012179696.2	LOC10111286	mRNA	XR_001033284.2	lncRNA
XM_012179696.2	LOC10111286	mRNA	XR_001033284.2	lncRNA
XM_012179696.2	LOC10111286	mRNA	XR_001033284.2	lncRNA
XM_012179696.2	LOC10111286	mRNA	XR_001033284.2	lncRNA
XM_012179696.2	LOC10111286	mRNA	XR_001033284.2	lncRNA
XM_012179696.2	LOC10111286	mRNA	XR_001033284.2	lncRNA
XM_015098990.1	GNA13	mRNA	XR_001021689.2	lncRNA
XM_015098990.1	GNA13	mRNA	XR_001021689.2	lncRNA
XM_015098990.1	GNA13	mRNA	XR_001021689.2	lncRNA
XM_015098990.1	GNA13	mRNA	XR_001021689.2	lncRNA
XM_015098990.1	GNA13	mRNA	XR_001021689.2	lncRNA
XM_015098990.1	GNA13	mRNA	XR_001021689.2	lncRNA
XM_015098990.1	GNA13	mRNA	XR_001021689.2	lncRNA
XM_015098990.1	GNA13	mRNA	XR_001021689.2	lncRNA
XM_012185217.2	CLTC	mRNA	XR_001436102.1	lncRNA
XM_012185217.2	CLTC	mRNA	XR_001436102.1	lncRNA
XM_012185217.2	CLTC	mRNA	XR_001436102.1	lncRNA
XM_012185217.2	CLTC	mRNA	XR_001436102.1	lncRNA
XM_012185217.2	CLTC	mRNA	XR_001436102.1	lncRNA
XM_012185217.2	CLTC	mRNA	XR_001436102.1	lncRNA
XM_012185217.2	CLTC	mRNA	XR_001436102.1	lncRNA
XM_012185217.2	CLTC	mRNA	XR_001436102.1	lncRNA
XM_012176217.2	EVI5	mRNA	XR_001436102.1	lncRNA
XM_012176217.2	EVI5	mRNA	XR_001436102.1	lncRNA
XM_012176217.2	EVI5	mRNA	XR_001436102.1	lncRNA
XM_012176217.2	EVI5	mRNA	XR_001436102.1	lncRNA
XM_012176217.2	EVI5	mRNA	XR_001436102.1	lncRNA
XM_012176217.2	EVI5	mRNA	XR_001436102.1	lncRNA
XM_012176217.2	EVI5	mRNA	XR_001436102.1	lncRNA
XM_012176217.2	EVI5	mRNA	XR_001436102.1	lncRNA
XM_012102834.2	PPRC1	mRNA	XR_001436102.1	lncRNA
XM_012102834.2	PPRC1	mRNA	XR_001436102.1	lncRNA
XM_012102834.2	PPRC1	mRNA	XR_001436102.1	lncRNA
XM_012102834.2	PPRC1	mRNA	XR_001436102.1	lncRNA
XM_012102834.2	PPRC1	mRNA	XR_001436102.1	lncRNA
XM_012102834.2	PPRC1	mRNA	XR_001436102.1	lncRNA
XM_012102834.2	PPRC1	mRNA	XR_001436102.1	lncRNA
XM_012102834.2	PPRC1	mRNA	XR_001436102.1	lncRNA

XM_012174768.2	RAB5B	mRNA	XR_001436102.1	lncRNA
XM_012174768.2	RAB5B	mRNA	XR_001436102.1	lncRNA
XM_012174768.2	RAB5B	mRNA	XR_001436102.1	lncRNA
XM_012174768.2	RAB5B	mRNA	XR_001436102.1	lncRNA
XM_012174768.2	RAB5B	mRNA	XR_001436102.1	lncRNA
XM_012174768.2	RAB5B	mRNA	XR_001436102.1	lncRNA
XM_012174768.2	RAB5B	mRNA	XR_001436102.1	lncRNA
XM_012174768.2	RAB5B	mRNA	XR_001436102.1	lncRNA
XM_012140422.2	CLASP1	mRNA	XR_001436102.1	lncRNA
XM_012140422.2	CLASP1	mRNA	XR_001436102.1	lncRNA
XM_012140422.2	CLASP1	mRNA	XR_001436102.1	lncRNA
XM_012140422.2	CLASP1	mRNA	XR_001436102.1	lncRNA
XM_012140422.2	CLASP1	mRNA	XR_001436102.1	lncRNA
XM_012140422.2	CLASP1	mRNA	XR_001436102.1	lncRNA
XM_012140422.2	CLASP1	mRNA	XR_001436102.1	lncRNA
XM_012140422.2	CLASP1	mRNA	XR_001436102.1	lncRNA
XM_004008647.3	RAPGEF6	mRNA	XR_001021689.2	lncRNA
XM_004008647.3	RAPGEF6	mRNA	XR_001021689.2	lncRNA
XM_004008647.3	RAPGEF6	mRNA	XR_001021689.2	lncRNA
XM_004008647.3	RAPGEF6	mRNA	XR_001021689.2	lncRNA
XM_004008647.3	RAPGEF6	mRNA	XR_001021689.2	lncRNA
XM_004008647.3	RAPGEF6	mRNA	XR_001021689.2	lncRNA
XM_004008647.3	RAPGEF6	mRNA	XR_001021689.2	lncRNA
XM_004008647.3	RAPGEF6	mRNA	XR_001021689.2	lncRNA
XM_012181966.2	SIPA1L1	mRNA	TCONS_00075453	lncRNA
XM_012181966.2	SIPA1L1	mRNA	TCONS_00075453	lncRNA
XM_012181966.2	SIPA1L1	mRNA	TCONS_00075453	lncRNA
XM_012181966.2	SIPA1L1	mRNA	TCONS_00075453	lncRNA
XM_012181966.2	SIPA1L1	mRNA	TCONS_00075453	lncRNA
XM_012181966.2	SIPA1L1	mRNA	TCONS_00075453	lncRNA
XM_012181966.2	SIPA1L1	mRNA	TCONS_00075453	lncRNA
XM_012181966.2	SIPA1L1	mRNA	TCONS_00075453	lncRNA
XM_015098136.1	PDS5B	mRNA	XR_001042381.2	lncRNA
XM_015098136.1	PDS5B	mRNA	XR_001042381.2	lncRNA
XM_015098136.1	PDS5B	mRNA	XR_001042381.2	lncRNA
XM_015098136.1	PDS5B	mRNA	XR_001042381.2	lncRNA
XM_015098136.1	PDS5B	mRNA	XR_001042381.2	lncRNA
XM_015098136.1	PDS5B	mRNA	XR_001042381.2	lncRNA
XM_015098136.1	PDS5B	mRNA	XR_001042381.2	lncRNA
XM_015098136.1	PDS5B	mRNA	XR_001042381.2	lncRNA
XM_015099551.1	PARD3	mRNA	XR_001436127.1	lncRNA
XM_015099551.1	PARD3	mRNA	XR_001436127.1	lncRNA
XM_015099551.1	PARD3	mRNA	XR_001436127.1	lncRNA
XM_015099551.1	PARD3	mRNA	XR_001436127.1	lncRNA
XM_015099551.1	PARD3	mRNA	XR_001436127.1	lncRNA
XM_015099551.1	PARD3	mRNA	XR_001436127.1	lncRNA
XM_015099551.1	PARD3	mRNA	XR_001436127.1	lncRNA
XM_015099551.1	PARD3	mRNA	XR_001436127.1	lncRNA
XM_012134416.2	PPIG	mRNA	XR_001025491.1	lncRNA
XM_012134416.2	PPIG	mRNA	XR_001025491.1	lncRNA
XM_012134416.2	PPIG	mRNA	XR_001025491.1	lncRNA
XM_012134416.2	PPIG	mRNA	XR_001025491.1	lncRNA
XM_012134416.2	PPIG	mRNA	XR_001025491.1	lncRNA
XM_012134416.2	PPIG	mRNA	XR_001025491.1	lncRNA

XM_012134416.2	PPIG	mRNA	XR_001025491.1	lncRNA
XM_012134416.2	PPIG	mRNA	XR_001025491.1	lncRNA
XM_012173296.2	CDKL4	mRNA	XR_001024290.1	lncRNA
XM_012173296.2	CDKL4	mRNA	XR_001024290.1	lncRNA
XM_012173296.2	CDKL4	mRNA	XR_001024290.1	lncRNA
XM_012173296.2	CDKL4	mRNA	XR_001024290.1	lncRNA
XM_012173296.2	CDKL4	mRNA	XR_001024290.1	lncRNA
XM_012173296.2	CDKL4	mRNA	XR_001024290.1	lncRNA
XM_012173296.2	CDKL4	mRNA	XR_001024290.1	lncRNA
XM_012173296.2	CDKL4	mRNA	XR_001024290.1	lncRNA
XM_012097219.1	AGO3	mRNA	XR_001025491.1	lncRNA
XM_012097219.1	AGO3	mRNA	XR_001025491.1	lncRNA
XM_012097219.1	AGO3	mRNA	XR_001025491.1	lncRNA
XM_012097219.1	AGO3	mRNA	XR_001025491.1	lncRNA
XM_012097219.1	AGO3	mRNA	XR_001025491.1	lncRNA
XM_012097219.1	AGO3	mRNA	XR_001025491.1	lncRNA
XM_012097219.1	AGO3	mRNA	XR_001025491.1	lncRNA
XM_012097219.1	AGO3	mRNA	XR_001025491.1	lncRNA
XM_012182301.2	LRRFIP1	mRNA	XR_001041211.2	lncRNA
XM_012182301.2	LRRFIP1	mRNA	XR_001041211.2	lncRNA
XM_012182301.2	LRRFIP1	mRNA	XR_001041211.2	lncRNA
XM_012182301.2	LRRFIP1	mRNA	XR_001041211.2	lncRNA
XM_012182301.2	LRRFIP1	mRNA	XR_001041211.2	lncRNA
XM_012182301.2	LRRFIP1	mRNA	XR_001041211.2	lncRNA
XM_012182301.2	LRRFIP1	mRNA	XR_001041211.2	lncRNA
XM_012182301.2	LRRFIP1	mRNA	XR_001041211.2	lncRNA
XM_012096480.2	STX3	mRNA	XR_001041211.2	lncRNA
XM_012096480.2	STX3	mRNA	XR_001041211.2	lncRNA
XM_012096480.2	STX3	mRNA	XR_001041211.2	lncRNA
XM_012096480.2	STX3	mRNA	XR_001041211.2	lncRNA
XM_012096480.2	STX3	mRNA	XR_001041211.2	lncRNA
XM_012096480.2	STX3	mRNA	XR_001041211.2	lncRNA
XM_012096480.2	STX3	mRNA	XR_001041211.2	lncRNA
XM_012096480.2	STX3	mRNA	XR_001041211.2	lncRNA
XM_012102216.2	TFDP2	mRNA	XR_001041211.2	lncRNA
XM_012102216.2	TFDP2	mRNA	XR_001041211.2	lncRNA
XM_012102216.2	TFDP2	mRNA	XR_001041211.2	lncRNA
XM_012102216.2	TFDP2	mRNA	XR_001041211.2	lncRNA
XM_012102216.2	TFDP2	mRNA	XR_001041211.2	lncRNA
XM_012102216.2	TFDP2	mRNA	XR_001041211.2	lncRNA
XM_012102216.2	TFDP2	mRNA	XR_001041211.2	lncRNA
XM_012102216.2	TFDP2	mRNA	XR_001041211.2	lncRNA
XM_012124921.1	KIAA2026	mRNA	XR_001434761.1	lncRNA
XM_012124921.1	KIAA2026	mRNA	XR_001434761.1	lncRNA
XM_012124921.1	KIAA2026	mRNA	XR_001434761.1	lncRNA
XM_012124921.1	KIAA2026	mRNA	XR_001434761.1	lncRNA
XM_012124921.1	KIAA2026	mRNA	XR_001434761.1	lncRNA
XM_012124921.1	KIAA2026	mRNA	XR_001434761.1	lncRNA
XM_012124921.1	KIAA2026	mRNA	XR_001434761.1	lncRNA
XM_012124921.1	KIAA2026	mRNA	XR_001434761.1	lncRNA
XM_012184401.1	ZC3H13	mRNA	XR_001434761.1	lncRNA
XM_012184401.1	ZC3H13	mRNA	XR_001434761.1	lncRNA
XM_012184401.1	ZC3H13	mRNA	XR_001434761.1	lncRNA
XM_012184401.1	ZC3H13	mRNA	XR_001434761.1	lncRNA

XM_012184401.1	ZC3H13	mRNA	XR_001434761.1	lncRNA
XM_012184401.1	ZC3H13	mRNA	XR_001434761.1	lncRNA
XM_012184401.1	ZC3H13	mRNA	XR_001434761.1	lncRNA
XM_012184401.1	ZC3H13	mRNA	XR_001434761.1	lncRNA
XM_004018249.3	TRAK1	mRNA	XR_001434761.1	lncRNA
XM_004018249.3	TRAK1	mRNA	XR_001434761.1	lncRNA
XM_004018249.3	TRAK1	mRNA	XR_001434761.1	lncRNA
XM_004018249.3	TRAK1	mRNA	XR_001434761.1	lncRNA
XM_004018249.3	TRAK1	mRNA	XR_001434761.1	lncRNA
XM_004018249.3	TRAK1	mRNA	XR_001434761.1	lncRNA
XM_004018249.3	TRAK1	mRNA	XR_001434761.1	lncRNA
XM_004018249.3	TRAK1	mRNA	XR_001434761.1	lncRNA
XM_012134956.1	STK39	mRNA	XR_001434761.1	lncRNA
XM_012134956.1	STK39	mRNA	XR_001434761.1	lncRNA
XM_012134956.1	STK39	mRNA	XR_001434761.1	lncRNA
XM_012134956.1	STK39	mRNA	XR_001434761.1	lncRNA
XM_012134956.1	STK39	mRNA	XR_001434761.1	lncRNA
XM_012134956.1	STK39	mRNA	XR_001434761.1	lncRNA
XM_012134956.1	STK39	mRNA	XR_001434761.1	lncRNA
XM_012134956.1	STK39	mRNA	XR_001434761.1	lncRNA
XM_004016880.3	FAM169A	mRNA	XR_001434761.1	lncRNA
XM_004016880.3	FAM169A	mRNA	XR_001434761.1	lncRNA
XM_004016880.3	FAM169A	mRNA	XR_001434761.1	lncRNA
XM_004016880.3	FAM169A	mRNA	XR_001434761.1	lncRNA
XM_004016880.3	FAM169A	mRNA	XR_001434761.1	lncRNA
XM_004016880.3	FAM169A	mRNA	XR_001434761.1	lncRNA
XM_004016880.3	FAM169A	mRNA	XR_001434761.1	lncRNA
XM_004016880.3	FAM169A	mRNA	XR_001434761.1	lncRNA
XM_012107698.1	NFYC	mRNA	XR_001434892.1	lncRNA
XM_012107698.1	NFYC	mRNA	XR_001434892.1	lncRNA
XM_012107698.1	NFYC	mRNA	XR_001434892.1	lncRNA
XM_012107698.1	NFYC	mRNA	XR_001434892.1	lncRNA
XM_012107698.1	NFYC	mRNA	XR_001434892.1	lncRNA
XM_012107698.1	NFYC	mRNA	XR_001434892.1	lncRNA
XM_012107698.1	NFYC	mRNA	XR_001434892.1	lncRNA
XM_012107698.1	NFYC	mRNA	XR_001434892.1	lncRNA
XM_015096937.1	BMF	mRNA	TCONS_00075453	lncRNA
XM_015096937.1	BMF	mRNA	TCONS_00075453	lncRNA
XM_015096937.1	BMF	mRNA	TCONS_00075453	lncRNA
XM_015096937.1	BMF	mRNA	TCONS_00075453	lncRNA
XM_015096937.1	BMF	mRNA	TCONS_00075453	lncRNA
XM_015096937.1	BMF	mRNA	TCONS_00075453	lncRNA
XM_015096937.1	BMF	mRNA	TCONS_00075453	lncRNA
XM_015096937.1	BMF	mRNA	TCONS_00075453	lncRNA
XM_015092119.1	MTF2	mRNA	XR_001024290.1	lncRNA
XM_015092119.1	MTF2	mRNA	XR_001024290.1	lncRNA
XM_015092119.1	MTF2	mRNA	XR_001024290.1	lncRNA
XM_015092119.1	MTF2	mRNA	XR_001024290.1	lncRNA
XM_015092119.1	MTF2	mRNA	XR_001024290.1	lncRNA
XM_015092119.1	MTF2	mRNA	XR_001024290.1	lncRNA
XM_015092119.1	MTF2	mRNA	XR_001024290.1	lncRNA
XM_015095875.1	SAFB	mRNA	XR_001436127.1	lncRNA
XM_015095875.1	SAFB	mRNA	XR_001436127.1	lncRNA
XM_015095875.1	SAFB	mRNA	XR_001436127.1	lncRNA

XM_015095875.1	SAFB	mRNA	XR_001436127.1	lncRNA
XM_015095875.1	SAFB	mRNA	XR_001436127.1	lncRNA
XM_015095875.1	SAFB	mRNA	XR_001436127.1	lncRNA
XM_015095875.1	SAFB	mRNA	XR_001436127.1	lncRNA
XM_012095771.2	CTR9	mRNA	XR_001436102.1	lncRNA
XM_012095771.2	CTR9	mRNA	XR_001436102.1	lncRNA
XM_012095771.2	CTR9	mRNA	XR_001436102.1	lncRNA
XM_012095771.2	CTR9	mRNA	XR_001436102.1	lncRNA
XM_012095771.2	CTR9	mRNA	XR_001436102.1	lncRNA
XM_012095771.2	CTR9	mRNA	XR_001436102.1	lncRNA
XM_012095771.2	CTR9	mRNA	XR_001436102.1	lncRNA
XM_012186914.2	ZC3H11A	mRNA	XR_001436102.1	lncRNA
XM_012186914.2	ZC3H11A	mRNA	XR_001436102.1	lncRNA
XM_012186914.2	ZC3H11A	mRNA	XR_001436102.1	lncRNA
XM_012186914.2	ZC3H11A	mRNA	XR_001436102.1	lncRNA
XM_012186914.2	ZC3H11A	mRNA	XR_001436102.1	lncRNA
XM_012186914.2	ZC3H11A	mRNA	XR_001436102.1	lncRNA
XM_012186914.2	ZC3H11A	mRNA	XR_001436102.1	lncRNA
XM_012094768.2	ZMYM1	mRNA	XR_001021689.2	lncRNA
XM_012094768.2	ZMYM1	mRNA	XR_001021689.2	lncRNA
XM_012094768.2	ZMYM1	mRNA	XR_001021689.2	lncRNA
XM_012094768.2	ZMYM1	mRNA	XR_001021689.2	lncRNA
XM_012094768.2	ZMYM1	mRNA	XR_001021689.2	lncRNA
XM_012094768.2	ZMYM1	mRNA	XR_001021689.2	lncRNA
XM_012094768.2	ZMYM1	mRNA	XR_001021689.2	lncRNA
XM_012176451.2	AHR	mRNA	XR_001021689.2	lncRNA
XM_012176451.2	AHR	mRNA	XR_001021689.2	lncRNA
XM_012176451.2	AHR	mRNA	XR_001021689.2	lncRNA
XM_012176451.2	AHR	mRNA	XR_001021689.2	lncRNA
XM_012176451.2	AHR	mRNA	XR_001021689.2	lncRNA
XM_012176451.2	AHR	mRNA	XR_001021689.2	lncRNA
XM_012176451.2	AHR	mRNA	XR_001021689.2	lncRNA
XM_015104177.1	PRSS8	mRNA	XR_001436102.1	lncRNA
XM_015104177.1	PRSS8	mRNA	XR_001436102.1	lncRNA
XM_015104177.1	PRSS8	mRNA	XR_001436102.1	lncRNA
XM_015104177.1	PRSS8	mRNA	XR_001436102.1	lncRNA
XM_015104177.1	PRSS8	mRNA	XR_001436102.1	lncRNA
XM_015104177.1	PRSS8	mRNA	XR_001436102.1	lncRNA
XM_015104177.1	PRSS8	mRNA	XR_001436102.1	lncRNA
XM_012102669.1	ZNF518A	mRNA	XR_001434202.1	lncRNA
XM_012102669.1	ZNF518A	mRNA	XR_001434202.1	lncRNA
XM_012102669.1	ZNF518A	mRNA	XR_001434202.1	lncRNA
XM_012102669.1	ZNF518A	mRNA	XR_001434202.1	lncRNA
XM_012102669.1	ZNF518A	mRNA	XR_001434202.1	lncRNA
XM_012102669.1	ZNF518A	mRNA	XR_001434202.1	lncRNA
XM_012102669.1	ZNF518A	mRNA	XR_001434202.1	lncRNA
XM_012102669.1	ZNF518A	mRNA	XR_001434202.1	lncRNA
XM_012102669.1	ZNF518A	mRNA	XR_001034847.2	lncRNA
XM_012102669.1	ZNF518A	mRNA	XR_001034847.2	lncRNA
XM_012102669.1	ZNF518A	mRNA	XR_001034847.2	lncRNA
XM_012102669.1	ZNF518A	mRNA	XR_001034847.2	lncRNA
XM_012102669.1	ZNF518A	mRNA	XR_001034847.2	lncRNA
XM_012102669.1	ZNF518A	mRNA	XR_001034847.2	lncRNA
XM_012102669.1	ZNF518A	mRNA	XR_001034847.2	lncRNA

XM_012102669.1	ZNF518A	mRNA	XR_001034847.2	lncRNA
XM_012098812.2	RALGAPA1	mRNA	XR_001020707.2	lncRNA
XM_012098812.2	RALGAPA1	mRNA	XR_001020707.2	lncRNA
XM_012098812.2	RALGAPA1	mRNA	XR_001020707.2	lncRNA
XM_012098812.2	RALGAPA1	mRNA	XR_001020707.2	lncRNA
XM_012098812.2	RALGAPA1	mRNA	XR_001020707.2	lncRNA
XM_012098812.2	RALGAPA1	mRNA	XR_001020707.2	lncRNA
XM_012098812.2	RALGAPA1	mRNA	XR_001020707.2	lncRNA
XM_012098812.2	RALGAPA1	mRNA	XR_001020707.2	lncRNA
XM_012189186.2	RALGAPB	mRNA	XR_001033284.2	lncRNA
XM_012189186.2	RALGAPB	mRNA	XR_001033284.2	lncRNA
XM_012189186.2	RALGAPB	mRNA	XR_001033284.2	lncRNA
XM_012189186.2	RALGAPB	mRNA	XR_001033284.2	lncRNA
XM_012189186.2	RALGAPB	mRNA	XR_001033284.2	lncRNA
XM_012189186.2	RALGAPB	mRNA	XR_001033284.2	lncRNA
XM_012189186.2	RALGAPB	mRNA	XR_001033284.2	lncRNA
XM_012189186.2	RALGAPB	mRNA	XR_001033284.2	lncRNA
XM_012101765.1	PRMT3	mRNA	XR_001436817.1	lncRNA
XM_012101765.1	PRMT3	mRNA	XR_001436817.1	lncRNA
XM_012101765.1	PRMT3	mRNA	XR_001436817.1	lncRNA
XM_012101765.1	PRMT3	mRNA	XR_001436817.1	lncRNA
XM_012101765.1	PRMT3	mRNA	XR_001436817.1	lncRNA
XM_012101765.1	PRMT3	mRNA	XR_001436817.1	lncRNA
XM_012101765.1	PRMT3	mRNA	XR_001436817.1	lncRNA
XM_012101765.1	PRMT3	mRNA	XR_001436817.1	lncRNA
XM_012182353.2	LRRFIP1	mRNA	XR_001436817.1	lncRNA
XM_012182353.2	LRRFIP1	mRNA	XR_001436817.1	lncRNA
XM_012182353.2	LRRFIP1	mRNA	XR_001436817.1	lncRNA
XM_012182353.2	LRRFIP1	mRNA	XR_001436817.1	lncRNA
XM_012182353.2	LRRFIP1	mRNA	XR_001436817.1	lncRNA
XM_012182353.2	LRRFIP1	mRNA	XR_001436817.1	lncRNA
XM_012182353.2	LRRFIP1	mRNA	XR_001436817.1	lncRNA
XM_012182353.2	LRRFIP1	mRNA	XR_001436817.1	lncRNA
XM_012181827.2	ZBTB1	mRNA	XR_001436817.1	lncRNA
XM_012181827.2	ZBTB1	mRNA	XR_001436817.1	lncRNA
XM_012181827.2	ZBTB1	mRNA	XR_001436817.1	lncRNA
XM_012181827.2	ZBTB1	mRNA	XR_001436817.1	lncRNA
XM_012181827.2	ZBTB1	mRNA	XR_001436817.1	lncRNA
XM_012181827.2	ZBTB1	mRNA	XR_001436817.1	lncRNA
XM_012181827.2	ZBTB1	mRNA	XR_001436817.1	lncRNA
XM_012181827.2	ZBTB1	mRNA	XR_001436817.1	lncRNA
XM_012100080.2	ARIH2	mRNA	XR_001436817.1	lncRNA
XM_012100080.2	ARIH2	mRNA	XR_001436817.1	lncRNA
XM_012100080.2	ARIH2	mRNA	XR_001436817.1	lncRNA
XM_012100080.2	ARIH2	mRNA	XR_001436817.1	lncRNA
XM_012100080.2	ARIH2	mRNA	XR_001436817.1	lncRNA
XM_012100080.2	ARIH2	mRNA	XR_001436817.1	lncRNA
XM_012100080.2	ARIH2	mRNA	XR_001436817.1	lncRNA
XM_012100080.2	ARIH2	mRNA	XR_001436817.1	lncRNA
XM_012143452.2	NRP2	mRNA	XR_001436817.1	lncRNA
XM_012143452.2	NRP2	mRNA	XR_001436817.1	lncRNA
XM_012143452.2	NRP2	mRNA	XR_001436817.1	lncRNA
XM_012143452.2	NRP2	mRNA	XR_001436817.1	lncRNA
XM_012143452.2	NRP2	mRNA	XR_001436817.1	lncRNA

XM_012143452.2	NRP2	mRNA	XR_001436817.1	lncRNA
XM_012143452.2	NRP2	mRNA	XR_001436817.1	lncRNA
XM_012143452.2	NRP2	mRNA	XR_001436817.1	lncRNA
XM_004010813.3	GSTM1	mRNA	XR_001436102.1	lncRNA
XM_004010813.3	GSTM1	mRNA	XR_001436102.1	lncRNA
XM_004010813.3	GSTM1	mRNA	XR_001436102.1	lncRNA
XM_004010813.3	GSTM1	mRNA	XR_001436102.1	lncRNA
XM_004010813.3	GSTM1	mRNA	XR_001436102.1	lncRNA
XM_004010813.3	GSTM1	mRNA	XR_001436102.1	lncRNA
XM_004010813.3	GSTM1	mRNA	XR_001436102.1	lncRNA
XM_004010813.3	GSTM1	mRNA	XR_001436102.1	lncRNA
XM_012105331.2	USP54	mRNA	XR_001436102.1	lncRNA
XM_012105331.2	USP54	mRNA	XR_001436102.1	lncRNA
XM_012105331.2	USP54	mRNA	XR_001436102.1	lncRNA
XM_012105331.2	USP54	mRNA	XR_001436102.1	lncRNA
XM_012105331.2	USP54	mRNA	XR_001436102.1	lncRNA
XM_012105331.2	USP54	mRNA	XR_001436102.1	lncRNA
XM_012105331.2	USP54	mRNA	XR_001436102.1	lncRNA
XM_012105331.2	USP54	mRNA	XR_001436102.1	lncRNA
XM_015103117.1	ZBTB44	mRNA	XR_001021689.2	lncRNA
XM_015103117.1	ZBTB44	mRNA	XR_001021689.2	lncRNA
XM_015103117.1	ZBTB44	mRNA	XR_001021689.2	lncRNA
XM_015103117.1	ZBTB44	mRNA	XR_001021689.2	lncRNA
XM_015103117.1	ZBTB44	mRNA	XR_001021689.2	lncRNA
XM_015103117.1	ZBTB44	mRNA	XR_001021689.2	lncRNA
XM_015103117.1	ZBTB44	mRNA	XR_001021689.2	lncRNA
XM_015103117.1	ZBTB44	mRNA	XR_001021689.2	lncRNA
XM_004005653.3	ZBTB26	mRNA	XR_001436102.1	lncRNA
XM_004005653.3	ZBTB26	mRNA	XR_001436102.1	lncRNA
XM_004005653.3	ZBTB26	mRNA	XR_001436102.1	lncRNA
XM_004005653.3	ZBTB26	mRNA	XR_001436102.1	lncRNA
XM_004005653.3	ZBTB26	mRNA	XR_001436102.1	lncRNA
XM_004005653.3	ZBTB26	mRNA	XR_001436102.1	lncRNA
XM_004005653.3	ZBTB26	mRNA	XR_001436102.1	lncRNA
XM_004005653.3	ZBTB26	mRNA	XR_001436102.1	lncRNA
XM_012097610.2	ZNF605	mRNA	XR_001021689.2	lncRNA
XM_012097610.2	ZNF605	mRNA	XR_001021689.2	lncRNA
XM_012097610.2	ZNF605	mRNA	XR_001021689.2	lncRNA
XM_012097610.2	ZNF605	mRNA	XR_001021689.2	lncRNA
XM_012097610.2	ZNF605	mRNA	XR_001021689.2	lncRNA
XM_012097610.2	ZNF605	mRNA	XR_001021689.2	lncRNA
XM_012097610.2	ZNF605	mRNA	XR_001021689.2	lncRNA
XM_012097610.2	ZNF605	mRNA	XR_001021689.2	lncRNA
XM_015100519.1	NFAT5	mRNA	XR_001025491.1	lncRNA
XM_015100519.1	NFAT5	mRNA	XR_001025491.1	lncRNA
XM_015100519.1	NFAT5	mRNA	XR_001025491.1	lncRNA
XM_015100519.1	NFAT5	mRNA	XR_001025491.1	lncRNA
XM_015100519.1	NFAT5	mRNA	XR_001025491.1	lncRNA
XM_015100519.1	NFAT5	mRNA	XR_001025491.1	lncRNA
XM_015100519.1	NFAT5	mRNA	XR_001025491.1	lncRNA
XM_015100519.1	NFAT5	mRNA	XR_001025491.1	lncRNA
XM_015096598.1	LIN54	mRNA	XR_001024290.1	lncRNA
XM_015096598.1	LIN54	mRNA	XR_001024290.1	lncRNA
XM_015096598.1	LIN54	mRNA	XR_001024290.1	lncRNA

XM_015096598.1	LIN54	mRNA	XR_001024290.1	lncRNA
XM_015096598.1	LIN54	mRNA	XR_001024290.1	lncRNA
XM_015096598.1	LIN54	mRNA	XR_001024290.1	lncRNA
XM_015096598.1	LIN54	mRNA	XR_001024290.1	lncRNA
XM_015096598.1	LIN54	mRNA	XR_001024290.1	lncRNA
XM_015098618.1	DLG4	mRNA	XR_001044126.1	lncRNA
XM_015098618.1	DLG4	mRNA	XR_001044126.1	lncRNA
XM_015098618.1	DLG4	mRNA	XR_001044126.1	lncRNA
XM_015098618.1	DLG4	mRNA	XR_001044126.1	lncRNA
XM_015098618.1	DLG4	mRNA	XR_001044126.1	lncRNA
XM_015098618.1	DLG4	mRNA	XR_001044126.1	lncRNA
XM_015098618.1	DLG4	mRNA	XR_001044126.1	lncRNA
XM_015098618.1	DLG4	mRNA	XR_001044126.1	lncRNA
XM_015096598.1	LIN54	mRNA	XR_001025491.1	lncRNA
XM_015096598.1	LIN54	mRNA	XR_001025491.1	lncRNA
XM_015096598.1	LIN54	mRNA	XR_001025491.1	lncRNA
XM_015096598.1	LIN54	mRNA	XR_001025491.1	lncRNA
XM_015096598.1	LIN54	mRNA	XR_001025491.1	lncRNA
XM_015096598.1	LIN54	mRNA	XR_001025491.1	lncRNA
XM_015096598.1	LIN54	mRNA	XR_001025491.1	lncRNA
XM_015096598.1	LIN54	mRNA	XR_001025491.1	lncRNA
XM_012097680.2	SCARB1	mRNA	XR_001024290.1	lncRNA
XM_012097680.2	SCARB1	mRNA	XR_001024290.1	lncRNA
XM_012097680.2	SCARB1	mRNA	XR_001024290.1	lncRNA
XM_012097680.2	SCARB1	mRNA	XR_001024290.1	lncRNA
XM_012097680.2	SCARB1	mRNA	XR_001024290.1	lncRNA
XM_012097680.2	SCARB1	mRNA	XR_001024290.1	lncRNA
XM_012097680.2	SCARB1	mRNA	XR_001024290.1	lncRNA
XM_012097680.2	SCARB1	mRNA	XR_001024290.1	lncRNA
XM_015099256.1	CEP104	mRNA	XR_001436127.1	lncRNA
XM_015099256.1	CEP104	mRNA	XR_001436127.1	lncRNA
XM_015099256.1	CEP104	mRNA	XR_001436127.1	lncRNA
XM_015099256.1	CEP104	mRNA	XR_001436127.1	lncRNA
XM_015099256.1	CEP104	mRNA	XR_001436127.1	lncRNA
XM_015099256.1	CEP104	mRNA	XR_001436127.1	lncRNA
XM_015099256.1	CEP104	mRNA	XR_001436127.1	lncRNA
XM_015099256.1	CEP104	mRNA	XR_001436127.1	lncRNA
XM_012152455.2	LUZP1	mRNA	XR_001433755.1	lncRNA
XM_012152455.2	LUZP1	mRNA	XR_001433755.1	lncRNA
XM_012152455.2	LUZP1	mRNA	XR_001433755.1	lncRNA
XM_012152455.2	LUZP1	mRNA	XR_001433755.1	lncRNA
XM_012152455.2	LUZP1	mRNA	XR_001433755.1	lncRNA
XM_012152455.2	LUZP1	mRNA	XR_001433755.1	lncRNA
XM_012152455.2	LUZP1	mRNA	XR_001433755.1	lncRNA
XM_015104177.1	PRSS8	mRNA	XR_001434202.1	lncRNA
XM_015104177.1	PRSS8	mRNA	XR_001434202.1	lncRNA
XM_015104177.1	PRSS8	mRNA	XR_001434202.1	lncRNA
XM_015104177.1	PRSS8	mRNA	XR_001434202.1	lncRNA
XM_015104177.1	PRSS8	mRNA	XR_001434202.1	lncRNA
XM_015104177.1	PRSS8	mRNA	XR_001434202.1	lncRNA
XM_015104177.1	PRSS8	mRNA	XR_001434202.1	lncRNA
XM_015104177.1	PRSS8	mRNA	XR_001034847.2	lncRNA
XM_015104177.1	PRSS8	mRNA	XR_001034847.2	lncRNA
XM_015104177.1	PRSS8	mRNA	XR_001034847.2	lncRNA

XM_015104177.1	PRSS8	mRNA	XR_001034847.2	lncRNA
XM_015104177.1	PRSS8	mRNA	XR_001034847.2	lncRNA
XM_015104177.1	PRSS8	mRNA	XR_001034847.2	lncRNA
XM_015104177.1	PRSS8	mRNA	XR_001034847.2	lncRNA
XM_004016103.2	USP2	mRNA	TCONS_00022613	lncRNA
XM_004016103.2	USP2	mRNA	TCONS_00022613	lncRNA
XM_004016103.2	USP2	mRNA	TCONS_00022613	lncRNA
XM_004016103.2	USP2	mRNA	TCONS_00022613	lncRNA
XM_004016103.2	USP2	mRNA	TCONS_00022613	lncRNA
XM_004016103.2	USP2	mRNA	TCONS_00022613	lncRNA
XM_004016103.2	USP2	mRNA	TCONS_00022613	lncRNA
XM_012095502.1	SIK3	mRNA	XR_001033284.2	lncRNA
XM_012095502.1	SIK3	mRNA	XR_001033284.2	lncRNA
XM_012095502.1	SIK3	mRNA	XR_001033284.2	lncRNA
XM_012095502.1	SIK3	mRNA	XR_001033284.2	lncRNA
XM_012095502.1	SIK3	mRNA	XR_001033284.2	lncRNA
XM_012095502.1	SIK3	mRNA	XR_001033284.2	lncRNA
XM_012095502.1	SIK3	mRNA	XR_001033284.2	lncRNA
XM_012095502.1	SIK3	mRNA	XR_001033284.2	lncRNA
XM_015098446.1	VEZF1	mRNA	XR_001020707.2	lncRNA
XM_015098446.1	VEZF1	mRNA	XR_001020707.2	lncRNA
XM_015098446.1	VEZF1	mRNA	XR_001020707.2	lncRNA
XM_015098446.1	VEZF1	mRNA	XR_001020707.2	lncRNA
XM_015098446.1	VEZF1	mRNA	XR_001020707.2	lncRNA
XM_015098446.1	VEZF1	mRNA	XR_001020707.2	lncRNA
XM_015098446.1	VEZF1	mRNA	XR_001020707.2	lncRNA
XM_015098446.1	VEZF1	mRNA	XR_001020707.2	lncRNA
XM_012097367.2	SH3D19	mRNA	XR_001042381.2	lncRNA
XM_012097367.2	SH3D19	mRNA	XR_001042381.2	lncRNA
XM_012097367.2	SH3D19	mRNA	XR_001042381.2	lncRNA
XM_012097367.2	SH3D19	mRNA	XR_001042381.2	lncRNA
XM_012097367.2	SH3D19	mRNA	XR_001042381.2	lncRNA
XM_012097367.2	SH3D19	mRNA	XR_001042381.2	lncRNA
XM_012097367.2	SH3D19	mRNA	XR_001042381.2	lncRNA
XM_012097367.2	SH3D19	mRNA	XR_001042381.2	lncRNA
XM_015094531.1	ANAPC1	mRNA	TCONS_00075453	lncRNA
XM_015094531.1	ANAPC1	mRNA	TCONS_00075453	lncRNA
XM_015094531.1	ANAPC1	mRNA	TCONS_00075453	lncRNA
XM_015094531.1	ANAPC1	mRNA	TCONS_00075453	lncRNA
XM_015094531.1	ANAPC1	mRNA	TCONS_00075453	lncRNA
XM_015094531.1	ANAPC1	mRNA	TCONS_00075453	lncRNA
XM_015094531.1	ANAPC1	mRNA	TCONS_00075453	lncRNA
XM_015094531.1	ANAPC1	mRNA	TCONS_00075453	lncRNA
XM_004005724.3	NCOA1	mRNA	XR_001433755.1	lncRNA
XM_004005724.3	NCOA1	mRNA	XR_001433755.1	lncRNA
XM_004005724.3	NCOA1	mRNA	XR_001433755.1	lncRNA
XM_004005724.3	NCOA1	mRNA	XR_001433755.1	lncRNA
XM_004005724.3	NCOA1	mRNA	XR_001433755.1	lncRNA
XM_004005724.3	NCOA1	mRNA	XR_001433755.1	lncRNA
XM_004005724.3	NCOA1	mRNA	XR_001433755.1	lncRNA
XM_012097610.2	ZNF605	mRNA	XR_001434202.1	lncRNA
XM_012097610.2	ZNF605	mRNA	XR_001434202.1	lncRNA
XM_012097610.2	ZNF605	mRNA	XR_001434202.1	lncRNA
XM_012097610.2	ZNF605	mRNA	XR_001434202.1	lncRNA

XM_012097610.2	ZNF605	mRNA	XR_001434202.1	lncRNA
XM_012097610.2	ZNF605	mRNA	XR_001434202.1	lncRNA
XM_012097610.2	ZNF605	mRNA	XR_001434202.1	lncRNA
XM_012097610.2	ZNF605	mRNA	XR_001434202.1	lncRNA
XM_012097610.2	ZNF605	mRNA	XR_001034847.2	lncRNA
XM_012097610.2	ZNF605	mRNA	XR_001034847.2	lncRNA
XM_012097610.2	ZNF605	mRNA	XR_001034847.2	lncRNA
XM_012097610.2	ZNF605	mRNA	XR_001034847.2	lncRNA
XM_012097610.2	ZNF605	mRNA	XR_001034847.2	lncRNA
XM_012097610.2	ZNF605	mRNA	XR_001034847.2	lncRNA
XM_012097610.2	ZNF605	mRNA	XR_001034847.2	lncRNA
XM_012097610.2	ZNF605	mRNA	XR_001034847.2	lncRNA
XM_012106817.2	JADE3	mRNA	XR_001034847.2	lncRNA
XM_012106817.2	JADE3	mRNA	XR_001034847.2	lncRNA
XM_012106817.2	JADE3	mRNA	XR_001034847.2	lncRNA
XM_012106817.2	JADE3	mRNA	XR_001034847.2	lncRNA
XM_012106817.2	JADE3	mRNA	XR_001034847.2	lncRNA
XM_012106817.2	JADE3	mRNA	XR_001034847.2	lncRNA
XM_012106817.2	JADE3	mRNA	XR_001034847.2	lncRNA
XM_012106817.2	JADE3	mRNA	XR_001034847.2	lncRNA
XM_012106817.2	JADE3	mRNA	XR_001434202.1	lncRNA
XM_012106817.2	JADE3	mRNA	XR_001434202.1	lncRNA
XM_012106817.2	JADE3	mRNA	XR_001434202.1	lncRNA
XM_012106817.2	JADE3	mRNA	XR_001434202.1	lncRNA
XM_012106817.2	JADE3	mRNA	XR_001434202.1	lncRNA
XM_012106817.2	JADE3	mRNA	XR_001434202.1	lncRNA
XM_012106817.2	JADE3	mRNA	XR_001434202.1	lncRNA
XM_012106817.2	JADE3	mRNA	XR_001434202.1	lncRNA
XM_012176498.2	RAPGEF5	mRNA	XR_001025491.1	lncRNA
XM_012176498.2	RAPGEF5	mRNA	XR_001025491.1	lncRNA
XM_012176498.2	RAPGEF5	mRNA	XR_001025491.1	lncRNA
XM_012176498.2	RAPGEF5	mRNA	XR_001025491.1	lncRNA
XM_012176498.2	RAPGEF5	mRNA	XR_001025491.1	lncRNA
XM_012176498.2	RAPGEF5	mRNA	XR_001025491.1	lncRNA
XM_012176498.2	RAPGEF5	mRNA	XR_001025491.1	lncRNA
XM_012176498.2	RAPGEF5	mRNA	XR_001025491.1	lncRNA
XM_015091997.1	LOC10112163[illegible]	mRNA	XR_001044126.1	lncRNA
XM_015091997.1	LOC10112163[illegible]	mRNA	XR_001044126.1	lncRNA
XM_015091997.1	LOC10112163[illegible]	mRNA	XR_001044126.1	lncRNA
XM_015091997.1	LOC10112163[illegible]	mRNA	XR_001044126.1	lncRNA
XM_015091997.1	LOC10112163[illegible]	mRNA	XR_001044126.1	lncRNA
XM_015091997.1	LOC10112163[illegible]	mRNA	XR_001044126.1	lncRNA
XM_015091997.1	LOC10112163[illegible]	mRNA	XR_001044126.1	lncRNA
XM_015091997.1	LOC10112163[illegible]	mRNA	XR_001044126.1	lncRNA
XM_015104788.1	TBL1X	mRNA	XR_001434761.1	lncRNA
XM_015104788.1	TBL1X	mRNA	XR_001434761.1	lncRNA
XM_015104788.1	TBL1X	mRNA	XR_001434761.1	lncRNA
XM_015104788.1	TBL1X	mRNA	XR_001434761.1	lncRNA
XM_015104788.1	TBL1X	mRNA	XR_001434761.1	lncRNA
XM_015104788.1	TBL1X	mRNA	XR_001434761.1	lncRNA
XM_015104788.1	TBL1X	mRNA	XR_001434761.1	lncRNA
XM_015104788.1	TBL1X	mRNA	XR_001434761.1	lncRNA
XM_012174704.2	ZBTB39	mRNA	XR_001033284.2	lncRNA
XM_012174704.2	ZBTB39	mRNA	XR_001033284.2	lncRNA

XM_012174704.2	ZBTB39	mRNA	XR_001033284.2	lncRNA
XM_012174704.2	ZBTB39	mRNA	XR_001033284.2	lncRNA
XM_012174704.2	ZBTB39	mRNA	XR_001033284.2	lncRNA
XM_012174704.2	ZBTB39	mRNA	XR_001033284.2	lncRNA
XM_012174704.2	ZBTB39	mRNA	XR_001033284.2	lncRNA
XM_012174704.2	ZBTB39	mRNA	XR_001033284.2	lncRNA
XM_012185926.2	DLX4	mRNA	XR_001020707.2	lncRNA
XM_012185926.2	DLX4	mRNA	XR_001020707.2	lncRNA
XM_012185926.2	DLX4	mRNA	XR_001020707.2	lncRNA
XM_012185926.2	DLX4	mRNA	XR_001020707.2	lncRNA
XM_012185926.2	DLX4	mRNA	XR_001020707.2	lncRNA
XM_012185926.2	DLX4	mRNA	XR_001020707.2	lncRNA
XM_012185926.2	DLX4	mRNA	XR_001020707.2	lncRNA
XM_012185926.2	DLX4	mRNA	XR_001020707.2	lncRNA
XM_004015967.3	DYNC2H1	mRNA	XR_001024290.1	lncRNA
XM_004015967.3	DYNC2H1	mRNA	XR_001024290.1	lncRNA
XM_004015967.3	DYNC2H1	mRNA	XR_001024290.1	lncRNA
XM_004015967.3	DYNC2H1	mRNA	XR_001024290.1	lncRNA
XM_004015967.3	DYNC2H1	mRNA	XR_001024290.1	lncRNA
XM_004015967.3	DYNC2H1	mRNA	XR_001024290.1	lncRNA
XM_004015967.3	DYNC2H1	mRNA	XR_001024290.1	lncRNA
XM_012118246.2	DCAF12	mRNA	XR_001025491.1	lncRNA
XM_012118246.2	DCAF12	mRNA	XR_001025491.1	lncRNA
XM_012118246.2	DCAF12	mRNA	XR_001025491.1	lncRNA
XM_012118246.2	DCAF12	mRNA	XR_001025491.1	lncRNA
XM_012118246.2	DCAF12	mRNA	XR_001025491.1	lncRNA
XM_012118246.2	DCAF12	mRNA	XR_001025491.1	lncRNA
XM_012118246.2	DCAF12	mRNA	XR_001025491.1	lncRNA
XM_015096551.1	MOB1B	mRNA	XR_001024290.1	lncRNA
XM_015096551.1	MOB1B	mRNA	XR_001024290.1	lncRNA
XM_015096551.1	MOB1B	mRNA	XR_001024290.1	lncRNA
XM_015096551.1	MOB1B	mRNA	XR_001024290.1	lncRNA
XM_015096551.1	MOB1B	mRNA	XR_001024290.1	lncRNA
XM_015096551.1	MOB1B	mRNA	XR_001024290.1	lncRNA
XM_015096551.1	MOB1B	mRNA	XR_001024290.1	lncRNA
XM_004010813.3	GSTM1	mRNA	TCONS_00022613	lncRNA
XM_004010813.3	GSTM1	mRNA	TCONS_00022613	lncRNA
XM_004010813.3	GSTM1	mRNA	TCONS_00022613	lncRNA
XM_004010813.3	GSTM1	mRNA	TCONS_00022613	lncRNA
XM_004010813.3	GSTM1	mRNA	TCONS_00022613	lncRNA
XM_004010813.3	GSTM1	mRNA	TCONS_00022613	lncRNA
XM_004010813.3	GSTM1	mRNA	TCONS_00022613	lncRNA
XM_012102599.2	CEP70	mRNA	XR_001434892.1	lncRNA
XM_012102599.2	CEP70	mRNA	XR_001434892.1	lncRNA
XM_012102599.2	CEP70	mRNA	XR_001434892.1	lncRNA
XM_012102599.2	CEP70	mRNA	XR_001434892.1	lncRNA
XM_012102599.2	CEP70	mRNA	XR_001434892.1	lncRNA
XM_012102599.2	CEP70	mRNA	XR_001434892.1	lncRNA
XM_012102599.2	CEP70	mRNA	XR_001434892.1	lncRNA
XM_012099798.2	KCTL3	mRNA	XR_001434892.1	lncRNA
XM_012099798.2	KCTL3	mRNA	XR_001434892.1	lncRNA
XM_012099798.2	KCTL3	mRNA	XR_001434892.1	lncRNA
XM_012099798.2	KCTL3	mRNA	XR_001434892.1	lncRNA
XM_012099798.2	KCTL3	mRNA	XR_001434892.1	lncRNA

XM_012099798.2	KCTL3	mRNA	XR_001434892.1	lncRNA
XM_012099798.2	KCTL3	mRNA	XR_001434892.1	lncRNA
XM_012190563.2	ZMYM1	mRNA	XR_001434761.1	lncRNA
XM_012190563.2	ZMYM1	mRNA	XR_001434761.1	lncRNA
XM_012190563.2	ZMYM1	mRNA	XR_001434761.1	lncRNA
XM_012190563.2	ZMYM1	mRNA	XR_001434761.1	lncRNA
XM_012190563.2	ZMYM1	mRNA	XR_001434761.1	lncRNA
XM_012190563.2	ZMYM1	mRNA	XR_001434761.1	lncRNA
XM_012190563.2	ZMYM1	mRNA	XR_001434761.1	lncRNA
XM_012096323.1	PHF21A	mRNA	XR_001044126.1	lncRNA
XM_012096323.1	PHF21A	mRNA	XR_001044126.1	lncRNA
XM_012096323.1	PHF21A	mRNA	XR_001044126.1	lncRNA
XM_012096323.1	PHF21A	mRNA	XR_001044126.1	lncRNA
XM_012096323.1	PHF21A	mRNA	XR_001044126.1	lncRNA
XM_012096323.1	PHF21A	mRNA	XR_001044126.1	lncRNA
XM_012096323.1	PHF21A	mRNA	XR_001044126.1	lncRNA
XM_012096323.1	PHF21A	mRNA	XR_001044126.1	lncRNA
XM_015098446.1	VEZF1	mRNA	XR_001044126.1	lncRNA
XM_015098446.1	VEZF1	mRNA	XR_001044126.1	lncRNA
XM_015098446.1	VEZF1	mRNA	XR_001044126.1	lncRNA
XM_015098446.1	VEZF1	mRNA	XR_001044126.1	lncRNA
XM_015098446.1	VEZF1	mRNA	XR_001044126.1	lncRNA
XM_015098446.1	VEZF1	mRNA	XR_001044126.1	lncRNA
XM_015098446.1	VEZF1	mRNA	XR_001044126.1	lncRNA
XM_015098446.1	VEZF1	mRNA	XR_001044126.1	lncRNA
XM_015096573.1	SOWAHB	mRNA	XR_001024290.1	lncRNA
XM_015096573.1	SOWAHB	mRNA	XR_001024290.1	lncRNA
XM_015096573.1	SOWAHB	mRNA	XR_001024290.1	lncRNA
XM_015096573.1	SOWAHB	mRNA	XR_001024290.1	lncRNA
XM_015096573.1	SOWAHB	mRNA	XR_001024290.1	lncRNA
XM_015096573.1	SOWAHB	mRNA	XR_001024290.1	lncRNA
XM_015096573.1	SOWAHB	mRNA	XR_001024290.1	lncRNA
XM_015096573.1	SOWAHB	mRNA	XR_001024290.1	lncRNA
XM_015102486.1	NR2C2	mRNA	XR_001044126.1	lncRNA
XM_015102486.1	NR2C2	mRNA	XR_001044126.1	lncRNA
XM_015102486.1	NR2C2	mRNA	XR_001044126.1	lncRNA
XM_015102486.1	NR2C2	mRNA	XR_001044126.1	lncRNA
XM_015102486.1	NR2C2	mRNA	XR_001044126.1	lncRNA
XM_015102486.1	NR2C2	mRNA	XR_001044126.1	lncRNA
XM_015102486.1	NR2C2	mRNA	XR_001044126.1	lncRNA
XM_015102486.1	NR2C2	mRNA	XR_001044126.1	lncRNA
XM_015092323.1	KIRREL	mRNA	XR_001044126.1	lncRNA
XM_015092323.1	KIRREL	mRNA	XR_001044126.1	lncRNA
XM_015092323.1	KIRREL	mRNA	XR_001044126.1	lncRNA
XM_015092323.1	KIRREL	mRNA	XR_001044126.1	lncRNA
XM_015092323.1	KIRREL	mRNA	XR_001044126.1	lncRNA
XM_015092323.1	KIRREL	mRNA	XR_001044126.1	lncRNA
XM_015092323.1	KIRREL	mRNA	XR_001044126.1	lncRNA
XM_015092323.1	KIRREL	mRNA	XR_001044126.1	lncRNA
XM_015099609.1	ARHGAP12	mRNA	XR_001020707.2	lncRNA
XM_015099609.1	ARHGAP12	mRNA	XR_001020707.2	lncRNA
XM_015099609.1	ARHGAP12	mRNA	XR_001020707.2	lncRNA
XM_015099609.1	ARHGAP12	mRNA	XR_001020707.2	lncRNA
XM_015099609.1	ARHGAP12	mRNA	XR_001020707.2	lncRNA

XM_015099609.1	ARHGAP12	mRNA	XR_001020707.2	lncRNA
XM_015099609.1	ARHGAP12	mRNA	XR_001020707.2	lncRNA
XM_012176510.2	FNBP1L	mRNA	XR_001434202.1	lncRNA
XM_012176510.2	FNBP1L	mRNA	XR_001434202.1	lncRNA
XM_012176510.2	FNBP1L	mRNA	XR_001434202.1	lncRNA
XM_012176510.2	FNBP1L	mRNA	XR_001434202.1	lncRNA
XM_012176510.2	FNBP1L	mRNA	XR_001434202.1	lncRNA
XM_012176510.2	FNBP1L	mRNA	XR_001434202.1	lncRNA
XM_012176510.2	FNBP1L	mRNA	XR_001434202.1	lncRNA
XM_012176510.2	FNBP1L	mRNA	XR_001434202.1	lncRNA
XM_012174536.2	KIF21A	mRNA	XR_001034847.2	lncRNA
XM_012174536.2	KIF21A	mRNA	XR_001034847.2	lncRNA
XM_012174536.2	KIF21A	mRNA	XR_001034847.2	lncRNA
XM_012174536.2	KIF21A	mRNA	XR_001034847.2	lncRNA
XM_012174536.2	KIF21A	mRNA	XR_001034847.2	lncRNA
XM_012174536.2	KIF21A	mRNA	XR_001034847.2	lncRNA
XM_012174536.2	KIF21A	mRNA	XR_001034847.2	lncRNA
XM_012174536.2	KIF21A	mRNA	XR_001034847.2	lncRNA
XM_012174536.2	KIF21A	mRNA	XR_001434202.1	lncRNA
XM_012174536.2	KIF21A	mRNA	XR_001434202.1	lncRNA
XM_012174536.2	KIF21A	mRNA	XR_001434202.1	lncRNA
XM_012174536.2	KIF21A	mRNA	XR_001434202.1	lncRNA
XM_012174536.2	KIF21A	mRNA	XR_001434202.1	lncRNA
XM_012174536.2	KIF21A	mRNA	XR_001434202.1	lncRNA
XM_012174536.2	KIF21A	mRNA	XR_001434202.1	lncRNA
XM_012174536.2	KIF21A	mRNA	XR_001434202.1	lncRNA
XM_012176510.2	FNBP1L	mRNA	XR_001034847.2	lncRNA
XM_012176510.2	FNBP1L	mRNA	XR_001034847.2	lncRNA
XM_012176510.2	FNBP1L	mRNA	XR_001034847.2	lncRNA
XM_012176510.2	FNBP1L	mRNA	XR_001034847.2	lncRNA
XM_012176510.2	FNBP1L	mRNA	XR_001034847.2	lncRNA
XM_012176510.2	FNBP1L	mRNA	XR_001034847.2	lncRNA
XM_012176510.2	FNBP1L	mRNA	XR_001034847.2	lncRNA
XM_012176510.2	FNBP1L	mRNA	XR_001034847.2	lncRNA
XM_012188945.2	CSTF1	mRNA	XR_001436102.1	lncRNA
XM_012188945.2	CSTF1	mRNA	XR_001436102.1	lncRNA
XM_012188945.2	CSTF1	mRNA	XR_001436102.1	lncRNA
XM_012188945.2	CSTF1	mRNA	XR_001436102.1	lncRNA
XM_012188945.2	CSTF1	mRNA	XR_001436102.1	lncRNA
XM_012188945.2	CSTF1	mRNA	XR_001436102.1	lncRNA
XM_012188945.2	CSTF1	mRNA	XR_001436102.1	lncRNA
XM_012095401.2	ATM	mRNA	XR_001436102.1	lncRNA
XM_012095401.2	ATM	mRNA	XR_001436102.1	lncRNA
XM_012095401.2	ATM	mRNA	XR_001436102.1	lncRNA
XM_012095401.2	ATM	mRNA	XR_001436102.1	lncRNA
XM_012095401.2	ATM	mRNA	XR_001436102.1	lncRNA
XM_012095401.2	ATM	mRNA	XR_001436102.1	lncRNA
XM_012095401.2	ATM	mRNA	XR_001436102.1	lncRNA
XM_015095875.1	SAFB	mRNA	XR_001021689.2	lncRNA
XM_015095875.1	SAFB	mRNA	XR_001021689.2	lncRNA
XM_015095875.1	SAFB	mRNA	XR_001021689.2	lncRNA
XM_015095875.1	SAFB	mRNA	XR_001021689.2	lncRNA
XM_015095875.1	SAFB	mRNA	XR_001021689.2	lncRNA
XM_015095875.1	SAFB	mRNA	XR_001021689.2	lncRNA

XM_015095875.1	SAFB	mRNA	XR_001021689.2	lncRNA
XM_004022347.2	TMEM255A	mRNA	XR_001436102.1	lncRNA
XM_004022347.2	TMEM255A	mRNA	XR_001436102.1	lncRNA
XM_004022347.2	TMEM255A	mRNA	XR_001436102.1	lncRNA
XM_004022347.2	TMEM255A	mRNA	XR_001436102.1	lncRNA
XM_004022347.2	TMEM255A	mRNA	XR_001436102.1	lncRNA
XM_004022347.2	TMEM255A	mRNA	XR_001436102.1	lncRNA
XM_004022347.2	TMEM255A	mRNA	XR_001436102.1	lncRNA
XM_012187417.2	PANK4	mRNA	XR_001436102.1	lncRNA
XM_012187417.2	PANK4	mRNA	XR_001436102.1	lncRNA
XM_012187417.2	PANK4	mRNA	XR_001436102.1	lncRNA
XM_012187417.2	PANK4	mRNA	XR_001436102.1	lncRNA
XM_012187417.2	PANK4	mRNA	XR_001436102.1	lncRNA
XM_012187417.2	PANK4	mRNA	XR_001436102.1	lncRNA
XM_012187417.2	PANK4	mRNA	XR_001436102.1	lncRNA
XM_015099971.1	ZNF181	mRNA	XR_001436102.1	lncRNA
XM_015099971.1	ZNF181	mRNA	XR_001436102.1	lncRNA
XM_015099971.1	ZNF181	mRNA	XR_001436102.1	lncRNA
XM_015099971.1	ZNF181	mRNA	XR_001436102.1	lncRNA
XM_015099971.1	ZNF181	mRNA	XR_001436102.1	lncRNA
XM_015099971.1	ZNF181	mRNA	XR_001436102.1	lncRNA
XM_015099971.1	ZNF181	mRNA	XR_001436102.1	lncRNA
XM_012188945.2	CSTF1	mRNA	XR_001021689.2	lncRNA
XM_012188945.2	CSTF1	mRNA	XR_001021689.2	lncRNA
XM_012188945.2	CSTF1	mRNA	XR_001021689.2	lncRNA
XM_012188945.2	CSTF1	mRNA	XR_001021689.2	lncRNA
XM_012188945.2	CSTF1	mRNA	XR_001021689.2	lncRNA
XM_012188945.2	CSTF1	mRNA	XR_001021689.2	lncRNA
XM_012188945.2	CSTF1	mRNA	XR_001021689.2	lncRNA
XM_015095875.1	SAFB	mRNA	XR_001436102.1	lncRNA
XM_015095875.1	SAFB	mRNA	XR_001436102.1	lncRNA
XM_015095875.1	SAFB	mRNA	XR_001436102.1	lncRNA
XM_015095875.1	SAFB	mRNA	XR_001436102.1	lncRNA
XM_015095875.1	SAFB	mRNA	XR_001436102.1	lncRNA
XM_015095875.1	SAFB	mRNA	XR_001436102.1	lncRNA
XM_015095875.1	SAFB	mRNA	XR_001436102.1	lncRNA
XM_012098820.2	RALGAPA1	mRNA	XR_001021689.2	lncRNA
XM_012098820.2	RALGAPA1	mRNA	XR_001021689.2	lncRNA
XM_012098820.2	RALGAPA1	mRNA	XR_001021689.2	lncRNA
XM_012098820.2	RALGAPA1	mRNA	XR_001021689.2	lncRNA
XM_012098820.2	RALGAPA1	mRNA	XR_001021689.2	lncRNA
XM_012098820.2	RALGAPA1	mRNA	XR_001021689.2	lncRNA
XM_012098820.2	RALGAPA1	mRNA	XR_001021689.2	lncRNA
XM_012098820.2	RALGAPA1	mRNA	XR_001021689.2	lncRNA
XM_012188301.2	NEBL	mRNA	XR_001436102.1	lncRNA
XM_012188301.2	NEBL	mRNA	XR_001436102.1	lncRNA
XM_012188301.2	NEBL	mRNA	XR_001436102.1	lncRNA
XM_012188301.2	NEBL	mRNA	XR_001436102.1	lncRNA
XM_012188301.2	NEBL	mRNA	XR_001436102.1	lncRNA
XM_012188301.2	NEBL	mRNA	XR_001436102.1	lncRNA
XM_012188301.2	NEBL	mRNA	XR_001436102.1	lncRNA
XM_012188301.2	NEBL	mRNA	XR_001436102.1	lncRNA
XM_012105656.2	ZNF596	mRNA	XR_001436102.1	lncRNA
XM_012105656.2	ZNF596	mRNA	XR_001436102.1	lncRNA

XM_012105656.2	ZNF596	mRNA	XR_001436102.1	lncRNA
XM_012105656.2	ZNF596	mRNA	XR_001436102.1	lncRNA
XM_012105656.2	ZNF596	mRNA	XR_001436102.1	lncRNA
XM_012105656.2	ZNF596	mRNA	XR_001436102.1	lncRNA
XM_012105656.2	ZNF596	mRNA	XR_001436102.1	lncRNA
XM_012105656.2	ZNF596	mRNA	XR_001436102.1	lncRNA
XM_012104181.2	CCP110	mRNA	TCONS_00022613	lncRNA
XM_012104181.2	CCP110	mRNA	TCONS_00022613	lncRNA
XM_012104181.2	CCP110	mRNA	TCONS_00022613	lncRNA
XM_012104181.2	CCP110	mRNA	TCONS_00022613	lncRNA
XM_012104181.2	CCP110	mRNA	TCONS_00022613	lncRNA
XM_012104181.2	CCP110	mRNA	TCONS_00022613	lncRNA
XM_012104181.2	CCP110	mRNA	TCONS_00022613	lncRNA
XM_012105318.2	USP54	mRNA	XR_001434892.1	lncRNA
XM_012105318.2	USP54	mRNA	XR_001434892.1	lncRNA
XM_012105318.2	USP54	mRNA	XR_001434892.1	lncRNA
XM_012105318.2	USP54	mRNA	XR_001434892.1	lncRNA
XM_012105318.2	USP54	mRNA	XR_001434892.1	lncRNA
XM_012105318.2	USP54	mRNA	XR_001434892.1	lncRNA
XM_012105318.2	USP54	mRNA	XR_001434892.1	lncRNA
XM_012105318.2	USP54	mRNA	XR_001434892.1	lncRNA
XM_012174703.2	ZBTB39	mRNA	XR_001025491.1	lncRNA
XM_012174703.2	ZBTB39	mRNA	XR_001025491.1	lncRNA
XM_012174703.2	ZBTB39	mRNA	XR_001025491.1	lncRNA
XM_012174703.2	ZBTB39	mRNA	XR_001025491.1	lncRNA
XM_012174703.2	ZBTB39	mRNA	XR_001025491.1	lncRNA
XM_012174703.2	ZBTB39	mRNA	XR_001025491.1	lncRNA
XM_012174703.2	ZBTB39	mRNA	XR_001025491.1	lncRNA
XM_012174703.2	ZBTB39	mRNA	XR_001025491.1	lncRNA
XM_012174703.2	ZBTB39	mRNA	XR_001024290.1	lncRNA
XM_012174703.2	ZBTB39	mRNA	XR_001024290.1	lncRNA
XM_012174703.2	ZBTB39	mRNA	XR_001024290.1	lncRNA
XM_012174703.2	ZBTB39	mRNA	XR_001024290.1	lncRNA
XM_012174703.2	ZBTB39	mRNA	XR_001024290.1	lncRNA
XM_012174703.2	ZBTB39	mRNA	XR_001024290.1	lncRNA
XM_012174703.2	ZBTB39	mRNA	XR_001024290.1	lncRNA
XM_012174703.2	ZBTB39	mRNA	XR_001024290.1	lncRNA
XM_012186312.2	DDX42	mRNA	XR_001436532.1	lncRNA
XM_012186312.2	DDX42	mRNA	XR_001436532.1	lncRNA
XM_012186312.2	DDX42	mRNA	XR_001436532.1	lncRNA
XM_012186312.2	DDX42	mRNA	XR_001436532.1	lncRNA
XM_012186312.2	DDX42	mRNA	XR_001436532.1	lncRNA
XM_012186312.2	DDX42	mRNA	XR_001436532.1	lncRNA
XM_012186312.2	DDX42	mRNA	XR_001436532.1	lncRNA
XM_015091991.1	YIPF1	mRNA	XR_001436532.1	lncRNA
XM_015091991.1	YIPF1	mRNA	XR_001436532.1	lncRNA
XM_015091991.1	YIPF1	mRNA	XR_001436532.1	lncRNA
XM_015091991.1	YIPF1	mRNA	XR_001436532.1	lncRNA
XM_015091991.1	YIPF1	mRNA	XR_001436532.1	lncRNA
XM_015091991.1	YIPF1	mRNA	XR_001436532.1	lncRNA
XM_015091991.1	YIPF1	mRNA	XR_001436532.1	lncRNA
XM_015102007.1	PPP2R3C	mRNA	XR_001436532.1	lncRNA
XM_015102007.1	PPP2R3C	mRNA	XR_001436532.1	lncRNA
XM_015102007.1	PPP2R3C	mRNA	XR_001436532.1	lncRNA

XM_015102007.1	PPP2R3C	mRNA	XR_001436532.1	lncRNA
XM_015102007.1	PPP2R3C	mRNA	XR_001436532.1	lncRNA
XM_015102007.1	PPP2R3C	mRNA	XR_001436532.1	lncRNA
XM_015102007.1	PPP2R3C	mRNA	XR_001436532.1	lncRNA
XM_012181234.2	NDUFAF1	mRNA	XR_001436532.1	lncRNA
XM_012181234.2	NDUFAF1	mRNA	XR_001436532.1	lncRNA
XM_012181234.2	NDUFAF1	mRNA	XR_001436532.1	lncRNA
XM_012181234.2	NDUFAF1	mRNA	XR_001436532.1	lncRNA
XM_012181234.2	NDUFAF1	mRNA	XR_001436532.1	lncRNA
XM_012181234.2	NDUFAF1	mRNA	XR_001436532.1	lncRNA
XM_012181234.2	NDUFAF1	mRNA	XR_001436532.1	lncRNA
XM_015098766.1	WIPF2	mRNA	XR_001434202.1	lncRNA
XM_015098766.1	WIPF2	mRNA	XR_001434202.1	lncRNA
XM_015098766.1	WIPF2	mRNA	XR_001434202.1	lncRNA
XM_015098766.1	WIPF2	mRNA	XR_001434202.1	lncRNA
XM_015098766.1	WIPF2	mRNA	XR_001434202.1	lncRNA
XM_015098766.1	WIPF2	mRNA	XR_001434202.1	lncRNA
XM_015098766.1	WIPF2	mRNA	XR_001434202.1	lncRNA
XM_015098766.1	WIPF2	mRNA	XR_001034847.2	lncRNA
XM_015098766.1	WIPF2	mRNA	XR_001034847.2	lncRNA
XM_015098766.1	WIPF2	mRNA	XR_001034847.2	lncRNA
XM_015098766.1	WIPF2	mRNA	XR_001034847.2	lncRNA
XM_015098766.1	WIPF2	mRNA	XR_001034847.2	lncRNA
XM_015098766.1	WIPF2	mRNA	XR_001034847.2	lncRNA
XM_015098766.1	WIPF2	mRNA	XR_001034847.2	lncRNA
XM_004018249.3	TRAK1	mRNA	XR_001024290.1	lncRNA
XM_004018249.3	TRAK1	mRNA	XR_001024290.1	lncRNA
XM_004018249.3	TRAK1	mRNA	XR_001024290.1	lncRNA
XM_004018249.3	TRAK1	mRNA	XR_001024290.1	lncRNA
XM_004018249.3	TRAK1	mRNA	XR_001024290.1	lncRNA
XM_004018249.3	TRAK1	mRNA	XR_001024290.1	lncRNA
XM_004018249.3	TRAK1	mRNA	XR_001024290.1	lncRNA
XM_012181326.2	MAP4K5	mRNA	XR_001044126.1	lncRNA
XM_012181326.2	MAP4K5	mRNA	XR_001044126.1	lncRNA
XM_012181326.2	MAP4K5	mRNA	XR_001044126.1	lncRNA
XM_012181326.2	MAP4K5	mRNA	XR_001044126.1	lncRNA
XM_012181326.2	MAP4K5	mRNA	XR_001044126.1	lncRNA
XM_012181326.2	MAP4K5	mRNA	XR_001044126.1	lncRNA
XM_012181326.2	MAP4K5	mRNA	XR_001044126.1	lncRNA
XM_012107472.1	NFYC	mRNA	XR_001436127.1	lncRNA
XM_012107472.1	NFYC	mRNA	XR_001436127.1	lncRNA
XM_012107472.1	NFYC	mRNA	XR_001436127.1	lncRNA
XM_012107472.1	NFYC	mRNA	XR_001436127.1	lncRNA
XM_012107472.1	NFYC	mRNA	XR_001436127.1	lncRNA
XM_012107472.1	NFYC	mRNA	XR_001436127.1	lncRNA
XM_012107472.1	NFYC	mRNA	XR_001436127.1	lncRNA
XM_015098987.1	ABCA5	mRNA	XR_001436127.1	lncRNA
XM_015098987.1	ABCA5	mRNA	XR_001436127.1	lncRNA
XM_015098987.1	ABCA5	mRNA	XR_001436127.1	lncRNA
XM_015098987.1	ABCA5	mRNA	XR_001436127.1	lncRNA
XM_015098987.1	ABCA5	mRNA	XR_001436127.1	lncRNA
XM_015098987.1	ABCA5	mRNA	XR_001436127.1	lncRNA
XM_015098987.1	ABCA5	mRNA	XR_001436127.1	lncRNA
XM_015092063.1	FUBP1	mRNA	XR_001025491.1	lncRNA

XM_015092063.1	FUBP1	mRNA	XR_001025491.1	lncRNA
XM_015092063.1	FUBP1	mRNA	XR_001025491.1	lncRNA
XM_015092063.1	FUBP1	mRNA	XR_001025491.1	lncRNA
XM_015092063.1	FUBP1	mRNA	XR_001025491.1	lncRNA
XM_015092063.1	FUBP1	mRNA	XR_001025491.1	lncRNA
XM_015092063.1	FUBP1	mRNA	XR_001025491.1	lncRNA
XM_004007700.3	CLDN12	mRNA	XR_001436127.1	lncRNA
XM_004007700.3	CLDN12	mRNA	XR_001436127.1	lncRNA
XM_004007700.3	CLDN12	mRNA	XR_001436127.1	lncRNA
XM_004007700.3	CLDN12	mRNA	XR_001436127.1	lncRNA
XM_004007700.3	CLDN12	mRNA	XR_001436127.1	lncRNA
XM_004007700.3	CLDN12	mRNA	XR_001436127.1	lncRNA
XM_004007700.3	CLDN12	mRNA	XR_001436127.1	lncRNA
XM_015092421.1	BBX	mRNA	TCONS_00075453	lncRNA
XM_015092421.1	BBX	mRNA	TCONS_00075453	lncRNA
XM_015092421.1	BBX	mRNA	TCONS_00075453	lncRNA
XM_015092421.1	BBX	mRNA	TCONS_00075453	lncRNA
XM_015092421.1	BBX	mRNA	TCONS_00075453	lncRNA
XM_015092421.1	BBX	mRNA	TCONS_00075453	lncRNA
XM_015092421.1	BBX	mRNA	TCONS_00075453	lncRNA
XM_012102599.2	CEP70	mRNA	XR_001041211.2	lncRNA
XM_012102599.2	CEP70	mRNA	XR_001041211.2	lncRNA
XM_012102599.2	CEP70	mRNA	XR_001041211.2	lncRNA
XM_012102599.2	CEP70	mRNA	XR_001041211.2	lncRNA
XM_012102599.2	CEP70	mRNA	XR_001041211.2	lncRNA
XM_012102599.2	CEP70	mRNA	XR_001041211.2	lncRNA
XM_012102599.2	CEP70	mRNA	XR_001041211.2	lncRNA
XM_012099798.2	KCTL3	mRNA	XR_001041211.2	lncRNA
XM_012099798.2	KCTL3	mRNA	XR_001041211.2	lncRNA
XM_012099798.2	KCTL3	mRNA	XR_001041211.2	lncRNA
XM_012099798.2	KCTL3	mRNA	XR_001041211.2	lncRNA
XM_012099798.2	KCTL3	mRNA	XR_001041211.2	lncRNA
XM_012099798.2	KCTL3	mRNA	XR_001041211.2	lncRNA
XM_012099798.2	KCTL3	mRNA	XR_001041211.2	lncRNA
XM_012181234.2	NDUFAF1	mRNA	XR_001041211.2	lncRNA
XM_012181234.2	NDUFAF1	mRNA	XR_001041211.2	lncRNA
XM_012181234.2	NDUFAF1	mRNA	XR_001041211.2	lncRNA
XM_012181234.2	NDUFAF1	mRNA	XR_001041211.2	lncRNA
XM_012181234.2	NDUFAF1	mRNA	XR_001041211.2	lncRNA
XM_012181234.2	NDUFAF1	mRNA	XR_001041211.2	lncRNA
XM_012181234.2	NDUFAF1	mRNA	XR_001041211.2	lncRNA
XM_012105080.2	BICC1	mRNA	XR_001434202.1	lncRNA
XM_012105080.2	BICC1	mRNA	XR_001434202.1	lncRNA
XM_012105080.2	BICC1	mRNA	XR_001434202.1	lncRNA
XM_012105080.2	BICC1	mRNA	XR_001434202.1	lncRNA
XM_012105080.2	BICC1	mRNA	XR_001434202.1	lncRNA
XM_012105080.2	BICC1	mRNA	XR_001434202.1	lncRNA
XM_012105080.2	BICC1	mRNA	XR_001434202.1	lncRNA
XM_012105080.2	BICC1	mRNA	XR_001434202.1	lncRNA
XM_012105080.2	BICC1	mRNA	XR_001034847.2	lncRNA
XM_012105080.2	BICC1	mRNA	XR_001034847.2	lncRNA
XM_012105080.2	BICC1	mRNA	XR_001034847.2	lncRNA
XM_012105080.2	BICC1	mRNA	XR_001034847.2	lncRNA
XM_012105080.2	BICC1	mRNA	XR_001034847.2	lncRNA

XM_012105080.2	BICC1	mRNA	XR_001034847.2	lncRNA
XM_012105080.2	BICC1	mRNA	XR_001034847.2	lncRNA
XM_012105080.2	BICC1	mRNA	XR_001034847.2	lncRNA
XM_012105656.2	ZNF596	mRNA	XR_001434202.1	lncRNA
XM_012105656.2	ZNF596	mRNA	XR_001434202.1	lncRNA
XM_012105656.2	ZNF596	mRNA	XR_001434202.1	lncRNA
XM_012105656.2	ZNF596	mRNA	XR_001434202.1	lncRNA
XM_012105656.2	ZNF596	mRNA	XR_001434202.1	lncRNA
XM_012105656.2	ZNF596	mRNA	XR_001434202.1	lncRNA
XM_012105656.2	ZNF596	mRNA	XR_001434202.1	lncRNA
XM_012105656.2	ZNF596	mRNA	XR_001434202.1	lncRNA
XM_012098820.2	RALGAPA1	mRNA	XR_001434202.1	lncRNA
XM_012098820.2	RALGAPA1	mRNA	XR_001434202.1	lncRNA
XM_012098820.2	RALGAPA1	mRNA	XR_001434202.1	lncRNA
XM_012098820.2	RALGAPA1	mRNA	XR_001434202.1	lncRNA
XM_012098820.2	RALGAPA1	mRNA	XR_001434202.1	lncRNA
XM_012098820.2	RALGAPA1	mRNA	XR_001434202.1	lncRNA
XM_012098820.2	RALGAPA1	mRNA	XR_001434202.1	lncRNA
XM_012098820.2	RALGAPA1	mRNA	XR_001434202.1	lncRNA
XM_012105656.2	ZNF596	mRNA	XR_001034847.2	lncRNA
XM_012105656.2	ZNF596	mRNA	XR_001034847.2	lncRNA
XM_012105656.2	ZNF596	mRNA	XR_001034847.2	lncRNA
XM_012105656.2	ZNF596	mRNA	XR_001034847.2	lncRNA
XM_012105656.2	ZNF596	mRNA	XR_001034847.2	lncRNA
XM_012105656.2	ZNF596	mRNA	XR_001034847.2	lncRNA
XM_012105656.2	ZNF596	mRNA	XR_001034847.2	lncRNA
XM_012105656.2	ZNF596	mRNA	XR_001034847.2	lncRNA
XM_012098820.2	RALGAPA1	mRNA	XR_001034847.2	lncRNA
XM_012098820.2	RALGAPA1	mRNA	XR_001034847.2	lncRNA
XM_012098820.2	RALGAPA1	mRNA	XR_001034847.2	lncRNA
XM_012098820.2	RALGAPA1	mRNA	XR_001034847.2	lncRNA
XM_012098820.2	RALGAPA1	mRNA	XR_001034847.2	lncRNA
XM_012098820.2	RALGAPA1	mRNA	XR_001034847.2	lncRNA
XM_012098820.2	RALGAPA1	mRNA	XR_001034847.2	lncRNA
XM_012098820.2	RALGAPA1	mRNA	XR_001034847.2	lncRNA
XM_012100690.1	KLC4	mRNA	TCONS_00022613	lncRNA
XM_012100690.1	KLC4	mRNA	TCONS_00022613	lncRNA
XM_012100690.1	KLC4	mRNA	TCONS_00022613	lncRNA
XM_012100690.1	KLC4	mRNA	TCONS_00022613	lncRNA
XM_012100690.1	KLC4	mRNA	TCONS_00022613	lncRNA
XM_012100690.1	KLC4	mRNA	TCONS_00022613	lncRNA
XM_012100690.1	KLC4	mRNA	TCONS_00022613	lncRNA
XM_012173296.2	CDKL4	mRNA	TCONS_00022613	lncRNA
XM_012173296.2	CDKL4	mRNA	TCONS_00022613	lncRNA
XM_012173296.2	CDKL4	mRNA	TCONS_00022613	lncRNA
XM_012173296.2	CDKL4	mRNA	TCONS_00022613	lncRNA
XM_012173296.2	CDKL4	mRNA	TCONS_00022613	lncRNA
XM_012173296.2	CDKL4	mRNA	TCONS_00022613	lncRNA
XM_012173296.2	CDKL4	mRNA	TCONS_00022613	lncRNA
XM_012099038.2	SLC25A29	mRNA	XR_001436102.1	lncRNA
XM_012099038.2	SLC25A29	mRNA	XR_001436102.1	lncRNA
XM_012099038.2	SLC25A29	mRNA	XR_001436102.1	lncRNA
XM_012099038.2	SLC25A29	mRNA	XR_001436102.1	lncRNA
XM_012099038.2	SLC25A29	mRNA	XR_001436102.1	lncRNA

XM_012099038.2	SLC25A29	mRNA	XR_001436102.1	lncRNA
XM_012099038.2	SLC25A29	mRNA	XR_001436102.1	lncRNA
XM_012099038.2	SLC25A29	mRNA	XR_001436102.1	lncRNA
XM_012186312.2	DDX42	mRNA	XR_001436817.1	lncRNA
XM_012186312.2	DDX42	mRNA	XR_001436817.1	lncRNA
XM_012186312.2	DDX42	mRNA	XR_001436817.1	lncRNA
XM_012186312.2	DDX42	mRNA	XR_001436817.1	lncRNA
XM_012186312.2	DDX42	mRNA	XR_001436817.1	lncRNA
XM_012186312.2	DDX42	mRNA	XR_001436817.1	lncRNA
XM_012186312.2	DDX42	mRNA	XR_001436817.1	lncRNA
XM_012186914.2	ZC3H11A	mRNA	XR_001436817.1	lncRNA
XM_012186914.2	ZC3H11A	mRNA	XR_001436817.1	lncRNA
XM_012186914.2	ZC3H11A	mRNA	XR_001436817.1	lncRNA
XM_012186914.2	ZC3H11A	mRNA	XR_001436817.1	lncRNA
XM_012186914.2	ZC3H11A	mRNA	XR_001436817.1	lncRNA
XM_012186914.2	ZC3H11A	mRNA	XR_001436817.1	lncRNA
XM_012186914.2	ZC3H11A	mRNA	XR_001436817.1	lncRNA
XM_012179693.1	PIGY	mRNA	XR_001436817.1	lncRNA
XM_012179693.1	PIGY	mRNA	XR_001436817.1	lncRNA
XM_012179693.1	PIGY	mRNA	XR_001436817.1	lncRNA
XM_012179693.1	PIGY	mRNA	XR_001436817.1	lncRNA
XM_012179693.1	PIGY	mRNA	XR_001436817.1	lncRNA
XM_012179693.1	PIGY	mRNA	XR_001436817.1	lncRNA
XM_012179693.1	PIGY	mRNA	XR_001436817.1	lncRNA
XM_015104177.1	PRSS8	mRNA	XR_001436817.1	lncRNA
XM_015104177.1	PRSS8	mRNA	XR_001436817.1	lncRNA
XM_015104177.1	PRSS8	mRNA	XR_001436817.1	lncRNA
XM_015104177.1	PRSS8	mRNA	XR_001436817.1	lncRNA
XM_015104177.1	PRSS8	mRNA	XR_001436817.1	lncRNA
XM_015104177.1	PRSS8	mRNA	XR_001436817.1	lncRNA
XM_015104177.1	PRSS8	mRNA	XR_001436817.1	lncRNA
XM_015104177.1	PRSS8	mRNA	XR_001434203.1	lncRNA
XM_015104177.1	PRSS8	mRNA	XR_001434203.1	lncRNA
XM_015104177.1	PRSS8	mRNA	XR_001434203.1	lncRNA
XM_015104177.1	PRSS8	mRNA	XR_001434203.1	lncRNA
XM_015104177.1	PRSS8	mRNA	XR_001434203.1	lncRNA
XM_015104177.1	PRSS8	mRNA	XR_001434203.1	lncRNA
XM_015104177.1	PRSS8	mRNA	XR_001434203.1	lncRNA
XM_012179693.1	PIGY	mRNA	XR_001434203.1	lncRNA
XM_012179693.1	PIGY	mRNA	XR_001434203.1	lncRNA
XM_012179693.1	PIGY	mRNA	XR_001434203.1	lncRNA
XM_012179693.1	PIGY	mRNA	XR_001434203.1	lncRNA
XM_012179693.1	PIGY	mRNA	XR_001434203.1	lncRNA
XM_012179693.1	PIGY	mRNA	XR_001434203.1	lncRNA
XM_012179693.1	PIGY	mRNA	XR_001434203.1	lncRNA
XM_015104948.1	RPS6KA6	mRNA	XR_001436817.1	lncRNA
XM_015104948.1	RPS6KA6	mRNA	XR_001436817.1	lncRNA
XM_015104948.1	RPS6KA6	mRNA	XR_001436817.1	lncRNA
XM_015104948.1	RPS6KA6	mRNA	XR_001436817.1	lncRNA
XM_015104948.1	RPS6KA6	mRNA	XR_001436817.1	lncRNA
XM_015104948.1	RPS6KA6	mRNA	XR_001436817.1	lncRNA
XM_015104948.1	RPS6KA6	mRNA	XR_001436817.1	lncRNA
XM_012181234.2	NDUFAF1	mRNA	XR_001436817.1	lncRNA
XM_012181234.2	NDUFAF1	mRNA	XR_001436817.1	lncRNA

XM_012181234.2	NDUFAF1	mRNA	XR_001436817.1	lncRNA
XM_012181234.2	NDUFAF1	mRNA	XR_001436817.1	lncRNA
XM_012181234.2	NDUFAF1	mRNA	XR_001436817.1	lncRNA
XM_012181234.2	NDUFAF1	mRNA	XR_001436817.1	lncRNA
XM_012181234.2	NDUFAF1	mRNA	XR_001436817.1	lncRNA
XM_004004723.3	SPOPL	mRNA	XR_001436817.1	lncRNA
XM_004004723.3	SPOPL	mRNA	XR_001436817.1	lncRNA
XM_004004723.3	SPOPL	mRNA	XR_001436817.1	lncRNA
XM_004004723.3	SPOPL	mRNA	XR_001436817.1	lncRNA
XM_004004723.3	SPOPL	mRNA	XR_001436817.1	lncRNA
XM_004004723.3	SPOPL	mRNA	XR_001436817.1	lncRNA
XM_004004723.3	SPOPL	mRNA	XR_001436817.1	lncRNA
XM_012102549.2	CEP70	mRNA	XR_001436817.1	lncRNA
XM_012102549.2	CEP70	mRNA	XR_001436817.1	lncRNA
XM_012102549.2	CEP70	mRNA	XR_001436817.1	lncRNA
XM_012102549.2	CEP70	mRNA	XR_001436817.1	lncRNA
XM_012102549.2	CEP70	mRNA	XR_001436817.1	lncRNA
XM_012102549.2	CEP70	mRNA	XR_001436817.1	lncRNA
XM_012102549.2	CEP70	mRNA	XR_001436817.1	lncRNA
XM_012186312.2	DDX42	mRNA	XR_001434203.1	lncRNA
XM_012186312.2	DDX42	mRNA	XR_001434203.1	lncRNA
XM_012186312.2	DDX42	mRNA	XR_001434203.1	lncRNA
XM_012186312.2	DDX42	mRNA	XR_001434203.1	lncRNA
XM_012186312.2	DDX42	mRNA	XR_001434203.1	lncRNA
XM_012186312.2	DDX42	mRNA	XR_001434203.1	lncRNA
XM_012186312.2	DDX42	mRNA	XR_001434203.1	lncRNA
XM_012104594.2	ZNF789	mRNA	XR_001436817.1	lncRNA
XM_012104594.2	ZNF789	mRNA	XR_001436817.1	lncRNA
XM_012104594.2	ZNF789	mRNA	XR_001436817.1	lncRNA
XM_012104594.2	ZNF789	mRNA	XR_001436817.1	lncRNA
XM_012104594.2	ZNF789	mRNA	XR_001436817.1	lncRNA
XM_012104594.2	ZNF789	mRNA	XR_001436817.1	lncRNA
XM_012104594.2	ZNF789	mRNA	XR_001436817.1	lncRNA
XM_012094768.2	ZMYM1	mRNA	XR_001436817.1	lncRNA
XM_012094768.2	ZMYM1	mRNA	XR_001436817.1	lncRNA
XM_012094768.2	ZMYM1	mRNA	XR_001436817.1	lncRNA
XM_012094768.2	ZMYM1	mRNA	XR_001436817.1	lncRNA
XM_012094768.2	ZMYM1	mRNA	XR_001436817.1	lncRNA
XM_012094768.2	ZMYM1	mRNA	XR_001436817.1	lncRNA
XM_012094768.2	ZMYM1	mRNA	XR_001436817.1	lncRNA
XM_012187413.1	NRIP1	mRNA	XR_001433755.1	lncRNA
XM_012187413.1	NRIP1	mRNA	XR_001433755.1	lncRNA
XM_012187413.1	NRIP1	mRNA	XR_001433755.1	lncRNA
XM_012187413.1	NRIP1	mRNA	XR_001433755.1	lncRNA
XM_012187413.1	NRIP1	mRNA	XR_001433755.1	lncRNA
XM_012187413.1	NRIP1	mRNA	XR_001433755.1	lncRNA
XM_012176829.1	BBS9	mRNA	XR_001021689.2	lncRNA
XM_012176829.1	BBS9	mRNA	XR_001021689.2	lncRNA
XM_012176829.1	BBS9	mRNA	XR_001021689.2	lncRNA
XM_012176829.1	BBS9	mRNA	XR_001021689.2	lncRNA
XM_012176829.1	BBS9	mRNA	XR_001021689.2	lncRNA
XM_012176829.1	BBS9	mRNA	XR_001021689.2	lncRNA
XM_012176829.1	BBS9	mRNA	XR_001021689.2	lncRNA
XM_012101765.1	PRMT3	mRNA	XR_001021689.2	lncRNA

XM_012101765.1	PRMT3	mRNA	XR_001021689.2	lncRNA
XM_012101765.1	PRMT3	mRNA	XR_001021689.2	lncRNA
XM_012101765.1	PRMT3	mRNA	XR_001021689.2	lncRNA
XM_012101765.1	PRMT3	mRNA	XR_001021689.2	lncRNA
XM_012101765.1	PRMT3	mRNA	XR_001021689.2	lncRNA
XM_012101765.1	PRMT3	mRNA	XR_001021689.2	lncRNA
XM_012104566.2	ZKSCAN1	mRNA	XR_001021689.2	lncRNA
XM_012104566.2	ZKSCAN1	mRNA	XR_001021689.2	lncRNA
XM_012104566.2	ZKSCAN1	mRNA	XR_001021689.2	lncRNA
XM_012104566.2	ZKSCAN1	mRNA	XR_001021689.2	lncRNA
XM_012104566.2	ZKSCAN1	mRNA	XR_001021689.2	lncRNA
XM_012104566.2	ZKSCAN1	mRNA	XR_001021689.2	lncRNA
XM_012104566.2	ZKSCAN1	mRNA	XR_001021689.2	lncRNA
XM_012143452.2	NRP2	mRNA	XR_001436102.1	lncRNA
XM_012143452.2	NRP2	mRNA	XR_001436102.1	lncRNA
XM_012143452.2	NRP2	mRNA	XR_001436102.1	lncRNA
XM_012143452.2	NRP2	mRNA	XR_001436102.1	lncRNA
XM_012143452.2	NRP2	mRNA	XR_001436102.1	lncRNA
XM_012143452.2	NRP2	mRNA	XR_001436102.1	lncRNA
XM_012143452.2	NRP2	mRNA	XR_001436102.1	lncRNA
XM_012097680.2	SCARB1	mRNA	TCONS_00022613	lncRNA
XM_012097680.2	SCARB1	mRNA	TCONS_00022613	lncRNA
XM_012097680.2	SCARB1	mRNA	TCONS_00022613	lncRNA
XM_012097680.2	SCARB1	mRNA	TCONS_00022613	lncRNA
XM_012097680.2	SCARB1	mRNA	TCONS_00022613	lncRNA
XM_012097680.2	SCARB1	mRNA	TCONS_00022613	lncRNA
XM_012097680.2	SCARB1	mRNA	TCONS_00022613	lncRNA
XM_015097170.1	NUMB	mRNA	TCONS_00022613	lncRNA
XM_015097170.1	NUMB	mRNA	TCONS_00022613	lncRNA
XM_015097170.1	NUMB	mRNA	TCONS_00022613	lncRNA
XM_015097170.1	NUMB	mRNA	TCONS_00022613	lncRNA
XM_015097170.1	NUMB	mRNA	TCONS_00022613	lncRNA
XM_015097170.1	NUMB	mRNA	TCONS_00022613	lncRNA
XM_015097170.1	NUMB	mRNA	TCONS_00022613	lncRNA
XM_012184887.2	DOCK9	mRNA	TCONS_00022613	lncRNA
XM_012184887.2	DOCK9	mRNA	TCONS_00022613	lncRNA
XM_012184887.2	DOCK9	mRNA	TCONS_00022613	lncRNA
XM_012184887.2	DOCK9	mRNA	TCONS_00022613	lncRNA
XM_012184887.2	DOCK9	mRNA	TCONS_00022613	lncRNA
XM_012184887.2	DOCK9	mRNA	TCONS_00022613	lncRNA
XM_012184887.2	DOCK9	mRNA	TCONS_00022613	lncRNA
XM_015103641.1	YES1	mRNA	XR_001434892.1	lncRNA
XM_015103641.1	YES1	mRNA	XR_001434892.1	lncRNA
XM_015103641.1	YES1	mRNA	XR_001434892.1	lncRNA
XM_015103641.1	YES1	mRNA	XR_001434892.1	lncRNA
XM_015103641.1	YES1	mRNA	XR_001434892.1	lncRNA
XM_015103641.1	YES1	mRNA	XR_001434892.1	lncRNA
XM_015103641.1	YES1	mRNA	XR_001434892.1	lncRNA
XM_015099971.1	ZNF181	mRNA	XR_001434761.1	lncRNA
XM_015099971.1	ZNF181	mRNA	XR_001434761.1	lncRNA
XM_015099971.1	ZNF181	mRNA	XR_001434761.1	lncRNA
XM_015099971.1	ZNF181	mRNA	XR_001434761.1	lncRNA
XM_015099971.1	ZNF181	mRNA	XR_001434761.1	lncRNA
XM_015099971.1	ZNF181	mRNA	XR_001434761.1	lncRNA

XM_015099971.1	ZNF181	mRNA	XR_001434761.1	lncRNA
XM_012146477.2	ABCB6	mRNA	XR_001434761.1	lncRNA
XM_012146477.2	ABCB6	mRNA	XR_001434761.1	lncRNA
XM_012146477.2	ABCB6	mRNA	XR_001434761.1	lncRNA
XM_012146477.2	ABCB6	mRNA	XR_001434761.1	lncRNA
XM_012146477.2	ABCB6	mRNA	XR_001434761.1	lncRNA
XM_012146477.2	ABCB6	mRNA	XR_001434761.1	lncRNA
XM_012146477.2	ABCB6	mRNA	XR_001434761.1	lncRNA
XM_015095425.1	CUL1	mRNA	XR_001434761.1	lncRNA
XM_015095425.1	CUL1	mRNA	XR_001434761.1	lncRNA
XM_015095425.1	CUL1	mRNA	XR_001434761.1	lncRNA
XM_015095425.1	CUL1	mRNA	XR_001434761.1	lncRNA
XM_015095425.1	CUL1	mRNA	XR_001434761.1	lncRNA
XM_015095425.1	CUL1	mRNA	XR_001434761.1	lncRNA
XM_015095425.1	CUL1	mRNA	XR_001434761.1	lncRNA
XM_015092119.1	MTF2	mRNA	XR_001434761.1	lncRNA
XM_015092119.1	MTF2	mRNA	XR_001434761.1	lncRNA
XM_015092119.1	MTF2	mRNA	XR_001434761.1	lncRNA
XM_015092119.1	MTF2	mRNA	XR_001434761.1	lncRNA
XM_015092119.1	MTF2	mRNA	XR_001434761.1	lncRNA
XM_015092119.1	MTF2	mRNA	XR_001434761.1	lncRNA
XM_015092119.1	MTF2	mRNA	XR_001434761.1	lncRNA
XM_015104323.1	SPRTN	mRNA	XR_001434892.1	lncRNA
XM_015104323.1	SPRTN	mRNA	XR_001434892.1	lncRNA
XM_015104323.1	SPRTN	mRNA	XR_001434892.1	lncRNA
XM_015104323.1	SPRTN	mRNA	XR_001434892.1	lncRNA
XM_015104323.1	SPRTN	mRNA	XR_001434892.1	lncRNA
XM_015104323.1	SPRTN	mRNA	XR_001434892.1	lncRNA
XM_015104323.1	SPRTN	mRNA	XR_001434892.1	lncRNA
XM_012098306.1	UBE3A	mRNA	XR_001024290.1	lncRNA
XM_012098306.1	UBE3A	mRNA	XR_001024290.1	lncRNA
XM_012098306.1	UBE3A	mRNA	XR_001024290.1	lncRNA
XM_012098306.1	UBE3A	mRNA	XR_001024290.1	lncRNA
XM_012098306.1	UBE3A	mRNA	XR_001024290.1	lncRNA
XM_012098306.1	UBE3A	mRNA	XR_001024290.1	lncRNA
XM_012098306.1	UBE3A	mRNA	XR_001024290.1	lncRNA
XM_012098306.1	UBE3A	mRNA	XR_001025491.1	lncRNA
XM_012098306.1	UBE3A	mRNA	XR_001025491.1	lncRNA
XM_012098306.1	UBE3A	mRNA	XR_001025491.1	lncRNA
XM_012098306.1	UBE3A	mRNA	XR_001025491.1	lncRNA
XM_012098306.1	UBE3A	mRNA	XR_001025491.1	lncRNA
XM_012098306.1	UBE3A	mRNA	XR_001025491.1	lncRNA
XM_012098306.1	UBE3A	mRNA	XR_001025491.1	lncRNA
XM_015101001.1	RRAS2	mRNA	XR_001025491.1	lncRNA
XM_015101001.1	RRAS2	mRNA	XR_001025491.1	lncRNA
XM_015101001.1	RRAS2	mRNA	XR_001025491.1	lncRNA
XM_015101001.1	RRAS2	mRNA	XR_001025491.1	lncRNA
XM_015101001.1	RRAS2	mRNA	XR_001025491.1	lncRNA
XM_015101001.1	RRAS2	mRNA	XR_001025491.1	lncRNA
XM_015101001.1	RRAS2	mRNA	XR_001025491.1	lncRNA
XM_012176217.2	EVI5	mRNA	XR_001025491.1	lncRNA
XM_012176217.2	EVI5	mRNA	XR_001025491.1	lncRNA
XM_012176217.2	EVI5	mRNA	XR_001025491.1	lncRNA
XM_012176217.2	EVI5	mRNA	XR_001025491.1	lncRNA

XM_012176217.2	EVI5	mRNA	XR_001025491.1	lncRNA
XM_012176217.2	EVI5	mRNA	XR_001025491.1	lncRNA
XM_012176217.2	EVI5	mRNA	XR_001025491.1	lncRNA
XM_012112310.2	C2H9orf91	mRNA	XR_001033284.2	lncRNA
XM_012112310.2	C2H9orf91	mRNA	XR_001033284.2	lncRNA
XM_012112310.2	C2H9orf91	mRNA	XR_001033284.2	lncRNA
XM_012112310.2	C2H9orf91	mRNA	XR_001033284.2	lncRNA
XM_012112310.2	C2H9orf91	mRNA	XR_001033284.2	lncRNA
XM_012112310.2	C2H9orf91	mRNA	XR_001033284.2	lncRNA
XM_012112310.2	C2H9orf91	mRNA	XR_001033284.2	lncRNA
XM_012103501.2	MIB1	mRNA	XR_001024743.2	lncRNA
XM_012103501.2	MIB1	mRNA	XR_001024743.2	lncRNA
XM_012103501.2	MIB1	mRNA	XR_001024743.2	lncRNA
XM_012103501.2	MIB1	mRNA	XR_001024743.2	lncRNA
XM_012103501.2	MIB1	mRNA	XR_001024743.2	lncRNA
XM_012103501.2	MIB1	mRNA	XR_001024743.2	lncRNA
XM_015099550.1	PARD3	mRNA	TCONS_00075453	lncRNA
XM_015099550.1	PARD3	mRNA	TCONS_00075453	lncRNA
XM_015099550.1	PARD3	mRNA	TCONS_00075453	lncRNA
XM_015099550.1	PARD3	mRNA	TCONS_00075453	lncRNA
XM_015099550.1	PARD3	mRNA	TCONS_00075453	lncRNA
XM_015099550.1	PARD3	mRNA	TCONS_00075453	lncRNA
XM_015099550.1	PARD3	mRNA	TCONS_00075453	lncRNA
XM_004020074.3	PIK3AP1	mRNA	TCONS_00075453	lncRNA
XM_004020074.3	PIK3AP1	mRNA	TCONS_00075453	lncRNA
XM_004020074.3	PIK3AP1	mRNA	TCONS_00075453	lncRNA
XM_004020074.3	PIK3AP1	mRNA	TCONS_00075453	lncRNA
XM_004020074.3	PIK3AP1	mRNA	TCONS_00075453	lncRNA
XM_004020074.3	PIK3AP1	mRNA	TCONS_00075453	lncRNA
XM_004020074.3	PIK3AP1	mRNA	TCONS_00075453	lncRNA
XM_012188301.2	NEBL	mRNA	TCONS_00075453	lncRNA
XM_012188301.2	NEBL	mRNA	TCONS_00075453	lncRNA
XM_012188301.2	NEBL	mRNA	TCONS_00075453	lncRNA
XM_012188301.2	NEBL	mRNA	TCONS_00075453	lncRNA
XM_012188301.2	NEBL	mRNA	TCONS_00075453	lncRNA
XM_012188301.2	NEBL	mRNA	TCONS_00075453	lncRNA
XM_012188301.2	NEBL	mRNA	TCONS_00075453	lncRNA
XM_012104566.2	ZKSCAN1	mRNA	XR_001034847.2	lncRNA
XM_012104566.2	ZKSCAN1	mRNA	XR_001034847.2	lncRNA
XM_012104566.2	ZKSCAN1	mRNA	XR_001034847.2	lncRNA
XM_012104566.2	ZKSCAN1	mRNA	XR_001034847.2	lncRNA
XM_012104566.2	ZKSCAN1	mRNA	XR_001034847.2	lncRNA
XM_012104566.2	ZKSCAN1	mRNA	XR_001034847.2	lncRNA
XM_012104566.2	ZKSCAN1	mRNA	XR_001034847.2	lncRNA
XM_012130346.2	UGGT1	mRNA	XR_001034847.2	lncRNA
XM_012130346.2	UGGT1	mRNA	XR_001034847.2	lncRNA
XM_012130346.2	UGGT1	mRNA	XR_001034847.2	lncRNA
XM_012130346.2	UGGT1	mRNA	XR_001034847.2	lncRNA
XM_012130346.2	UGGT1	mRNA	XR_001034847.2	lncRNA
XM_012130346.2	UGGT1	mRNA	XR_001034847.2	lncRNA
XM_012130346.2	UGGT1	mRNA	XR_001034847.2	lncRNA
XM_012104566.2	ZKSCAN1	mRNA	XR_001434202.1	lncRNA
XM_012104566.2	ZKSCAN1	mRNA	XR_001434202.1	lncRNA
XM_012104566.2	ZKSCAN1	mRNA	XR_001434202.1	lncRNA

XM_012104566.2	ZKSCAN1	mRNA	XR_001434202.1	lncRNA
XM_012104566.2	ZKSCAN1	mRNA	XR_001434202.1	lncRNA
XM_012104566.2	ZKSCAN1	mRNA	XR_001434202.1	lncRNA
XM_012104566.2	ZKSCAN1	mRNA	XR_001434202.1	lncRNA
XM_012130346.2	UGGT1	mRNA	XR_001434202.1	lncRNA
XM_012130346.2	UGGT1	mRNA	XR_001434202.1	lncRNA
XM_012130346.2	UGGT1	mRNA	XR_001434202.1	lncRNA
XM_012130346.2	UGGT1	mRNA	XR_001434202.1	lncRNA
XM_012130346.2	UGGT1	mRNA	XR_001434202.1	lncRNA
XM_012130346.2	UGGT1	mRNA	XR_001434202.1	lncRNA
XM_012130346.2	UGGT1	mRNA	XR_001434202.1	lncRNA
XM_012176829.1	BBS9	mRNA	XR_001434202.1	lncRNA
XM_012176829.1	BBS9	mRNA	XR_001434202.1	lncRNA
XM_012176829.1	BBS9	mRNA	XR_001434202.1	lncRNA
XM_012176829.1	BBS9	mRNA	XR_001434202.1	lncRNA
XM_012176829.1	BBS9	mRNA	XR_001434202.1	lncRNA
XM_012176829.1	BBS9	mRNA	XR_001434202.1	lncRNA
XM_012176829.1	BBS9	mRNA	XR_001434202.1	lncRNA
XM_012094663.2	GRAMD1C	mRNA	XR_001034847.2	lncRNA
XM_012094663.2	GRAMD1C	mRNA	XR_001034847.2	lncRNA
XM_012094663.2	GRAMD1C	mRNA	XR_001034847.2	lncRNA
XM_012094663.2	GRAMD1C	mRNA	XR_001034847.2	lncRNA
XM_012094663.2	GRAMD1C	mRNA	XR_001034847.2	lncRNA
XM_012094663.2	GRAMD1C	mRNA	XR_001034847.2	lncRNA
XM_012094663.2	GRAMD1C	mRNA	XR_001034847.2	lncRNA
XM_012094663.2	GRAMD1C	mRNA	XR_001434202.1	lncRNA
XM_012094663.2	GRAMD1C	mRNA	XR_001434202.1	lncRNA
XM_012094663.2	GRAMD1C	mRNA	XR_001434202.1	lncRNA
XM_012094663.2	GRAMD1C	mRNA	XR_001434202.1	lncRNA
XM_012094663.2	GRAMD1C	mRNA	XR_001434202.1	lncRNA
XM_012094663.2	GRAMD1C	mRNA	XR_001434202.1	lncRNA
XM_012094663.2	GRAMD1C	mRNA	XR_001434202.1	lncRNA
XM_012176829.1	BBS9	mRNA	XR_001034847.2	lncRNA
XM_012176829.1	BBS9	mRNA	XR_001034847.2	lncRNA
XM_012176829.1	BBS9	mRNA	XR_001034847.2	lncRNA
XM_012176829.1	BBS9	mRNA	XR_001034847.2	lncRNA
XM_012176829.1	BBS9	mRNA	XR_001034847.2	lncRNA
XM_012176829.1	BBS9	mRNA	XR_001034847.2	lncRNA
XM_012176829.1	BBS9	mRNA	XR_001034847.2	lncRNA
XM_004003942.3	COL6A6	mRNA	TCONS_00022613	lncRNA
XM_004003942.3	COL6A6	mRNA	TCONS_00022613	lncRNA
XM_004003942.3	COL6A6	mRNA	TCONS_00022613	lncRNA
XM_004003942.3	COL6A6	mRNA	TCONS_00022613	lncRNA
XM_004003942.3	COL6A6	mRNA	TCONS_00022613	lncRNA
XM_004003942.3	COL6A6	mRNA	TCONS_00022613	lncRNA
XM_004003942.3	COL6A6	mRNA	TCONS_00022613	lncRNA
XM_012104225.2	DCUN1D3	mRNA	XR_001042381.2	lncRNA
XM_012104225.2	DCUN1D3	mRNA	XR_001042381.2	lncRNA
XM_012104225.2	DCUN1D3	mRNA	XR_001042381.2	lncRNA
XM_012104225.2	DCUN1D3	mRNA	XR_001042381.2	lncRNA
XM_012104225.2	DCUN1D3	mRNA	XR_001042381.2	lncRNA
XM_012104225.2	DCUN1D3	mRNA	XR_001042381.2	lncRNA
XM_012104225.2	DCUN1D3	mRNA	XR_001042381.2	lncRNA
XM_012184148.1	OTUL3B	mRNA	XR_001042381.2	lncRNA

XM_012184148.1	OTUL3B	mRNA	XR_001042381.2	lncRNA
XM_012184148.1	OTUL3B	mRNA	XR_001042381.2	lncRNA
XM_012184148.1	OTUL3B	mRNA	XR_001042381.2	lncRNA
XM_012184148.1	OTUL3B	mRNA	XR_001042381.2	lncRNA
XM_012184148.1	OTUL3B	mRNA	XR_001042381.2	lncRNA
XM_012184148.1	OTUL3B	mRNA	XR_001042381.2	lncRNA
XM_004004866.2	METTL21A	mRNA	XR_001436532.1	lncRNA
XM_004004866.2	METTL21A	mRNA	XR_001436532.1	lncRNA
XM_004004866.2	METTL21A	mRNA	XR_001436532.1	lncRNA
XM_004004866.2	METTL21A	mRNA	XR_001436532.1	lncRNA
XM_004004866.2	METTL21A	mRNA	XR_001436532.1	lncRNA
XM_004004866.2	METTL21A	mRNA	XR_001436532.1	lncRNA
XM_004004866.2	METTL21A	mRNA	XR_001436532.1	lncRNA
XM_012097254.2	RHOF	mRNA	XR_001436127.1	lncRNA
XM_012097254.2	RHOF	mRNA	XR_001436127.1	lncRNA
XM_012097254.2	RHOF	mRNA	XR_001436127.1	lncRNA
XM_012097254.2	RHOF	mRNA	XR_001436127.1	lncRNA
XM_012097254.2	RHOF	mRNA	XR_001436127.1	lncRNA
XM_012097254.2	RHOF	mRNA	XR_001436127.1	lncRNA
XM_012097254.2	RHOF	mRNA	XR_001436127.1	lncRNA
XM_012097610.2	ZNF605	mRNA	XR_001044126.1	lncRNA
XM_012097610.2	ZNF605	mRNA	XR_001044126.1	lncRNA
XM_012097610.2	ZNF605	mRNA	XR_001044126.1	lncRNA
XM_012097610.2	ZNF605	mRNA	XR_001044126.1	lncRNA
XM_012097610.2	ZNF605	mRNA	XR_001044126.1	lncRNA
XM_012097610.2	ZNF605	mRNA	XR_001044126.1	lncRNA
XM_012097610.2	ZNF605	mRNA	XR_001044126.1	lncRNA
XM_004010813.3	GSTM1	mRNA	XR_001024290.1	lncRNA
XM_004010813.3	GSTM1	mRNA	XR_001024290.1	lncRNA
XM_004010813.3	GSTM1	mRNA	XR_001024290.1	lncRNA
XM_004010813.3	GSTM1	mRNA	XR_001024290.1	lncRNA
XM_004010813.3	GSTM1	mRNA	XR_001024290.1	lncRNA
XM_004010813.3	GSTM1	mRNA	XR_001024290.1	lncRNA
XM_004010813.3	GSTM1	mRNA	XR_001024290.1	lncRNA
XM_012096925.2	SLC38A9	mRNA	XR_001044126.1	lncRNA
XM_012096925.2	SLC38A9	mRNA	XR_001044126.1	lncRNA
XM_012096925.2	SLC38A9	mRNA	XR_001044126.1	lncRNA
XM_012096925.2	SLC38A9	mRNA	XR_001044126.1	lncRNA
XM_012096925.2	SLC38A9	mRNA	XR_001044126.1	lncRNA
XM_012096925.2	SLC38A9	mRNA	XR_001044126.1	lncRNA
XM_012096925.2	SLC38A9	mRNA	XR_001044126.1	lncRNA
XM_012189779.2	RBL2	mRNA	XR_001024743.2	lncRNA
XM_012189779.2	RBL2	mRNA	XR_001024743.2	lncRNA
XM_012189779.2	RBL2	mRNA	XR_001024743.2	lncRNA
XM_012189779.2	RBL2	mRNA	XR_001024743.2	lncRNA
XM_012189779.2	RBL2	mRNA	XR_001024743.2	lncRNA
XM_012189779.2	RBL2	mRNA	XR_001024743.2	lncRNA
XM_015096573.1	SOWAHB	mRNA	TCONS_00022613	lncRNA
XM_015096573.1	SOWAHB	mRNA	TCONS_00022613	lncRNA
XM_015096573.1	SOWAHB	mRNA	TCONS_00022613	lncRNA
XM_015096573.1	SOWAHB	mRNA	TCONS_00022613	lncRNA
XM_015096573.1	SOWAHB	mRNA	TCONS_00022613	lncRNA
XM_015096573.1	SOWAHB	mRNA	TCONS_00022613	lncRNA
XM_015096573.1	SOWAHB	mRNA	TCONS_00022613	lncRNA

XM_015098446.1	VEZF1	mRNA	TCONS_00022613	lncRNA
XM_015098446.1	VEZF1	mRNA	TCONS_00022613	lncRNA
XM_015098446.1	VEZF1	mRNA	TCONS_00022613	lncRNA
XM_015098446.1	VEZF1	mRNA	TCONS_00022613	lncRNA
XM_015098446.1	VEZF1	mRNA	TCONS_00022613	lncRNA
XM_015098446.1	VEZF1	mRNA	TCONS_00022613	lncRNA
XM_015098446.1	VEZF1	mRNA	TCONS_00022613	lncRNA
XM_012181966.2	SIPA1L1	mRNA	TCONS_00022613	lncRNA
XM_012181966.2	SIPA1L1	mRNA	TCONS_00022613	lncRNA
XM_012181966.2	SIPA1L1	mRNA	TCONS_00022613	lncRNA
XM_012181966.2	SIPA1L1	mRNA	TCONS_00022613	lncRNA
XM_012181966.2	SIPA1L1	mRNA	TCONS_00022613	lncRNA
XM_012181966.2	SIPA1L1	mRNA	TCONS_00022613	lncRNA
XM_012181966.2	SIPA1L1	mRNA	TCONS_00022613	lncRNA
XM_015102486.1	NR2C2	mRNA	TCONS_00022613	lncRNA
XM_015102486.1	NR2C2	mRNA	TCONS_00022613	lncRNA
XM_015102486.1	NR2C2	mRNA	TCONS_00022613	lncRNA
XM_015102486.1	NR2C2	mRNA	TCONS_00022613	lncRNA
XM_015102486.1	NR2C2	mRNA	TCONS_00022613	lncRNA
XM_015102486.1	NR2C2	mRNA	TCONS_00022613	lncRNA
XM_015102486.1	NR2C2	mRNA	TCONS_00022613	lncRNA
XM_012181960.2	SIPA1L1	mRNA	TCONS_00022613	lncRNA
XM_012181960.2	SIPA1L1	mRNA	TCONS_00022613	lncRNA
XM_012181960.2	SIPA1L1	mRNA	TCONS_00022613	lncRNA
XM_012181960.2	SIPA1L1	mRNA	TCONS_00022613	lncRNA
XM_012181960.2	SIPA1L1	mRNA	TCONS_00022613	lncRNA
XM_012181960.2	SIPA1L1	mRNA	TCONS_00022613	lncRNA
XM_012181960.2	SIPA1L1	mRNA	TCONS_00022613	lncRNA
XM_012095439.2	SIK2	mRNA	XR_001021689.2	lncRNA
XM_012095439.2	SIK2	mRNA	XR_001021689.2	lncRNA
XM_012095439.2	SIK2	mRNA	XR_001021689.2	lncRNA
XM_012095439.2	SIK2	mRNA	XR_001021689.2	lncRNA
XM_012095439.2	SIK2	mRNA	XR_001021689.2	lncRNA
XM_012095439.2	SIK2	mRNA	XR_001021689.2	lncRNA
XM_012095439.2	SIK2	mRNA	XR_001021689.2	lncRNA
NM_001308600.1	KRT17	mRNA	XR_001436102.1	lncRNA
NM_001308600.1	KRT17	mRNA	XR_001436102.1	lncRNA
NM_001308600.1	KRT17	mRNA	XR_001436102.1	lncRNA
NM_001308600.1	KRT17	mRNA	XR_001436102.1	lncRNA
NM_001308600.1	KRT17	mRNA	XR_001436102.1	lncRNA
NM_001308600.1	KRT17	mRNA	XR_001436102.1	lncRNA
NM_001308600.1	KRT17	mRNA	XR_001436102.1	lncRNA
XM_012181326.2	MAP4K5	mRNA	XR_001436102.1	lncRNA
XM_012181326.2	MAP4K5	mRNA	XR_001436102.1	lncRNA
XM_012181326.2	MAP4K5	mRNA	XR_001436102.1	lncRNA
XM_012181326.2	MAP4K5	mRNA	XR_001436102.1	lncRNA
XM_012181326.2	MAP4K5	mRNA	XR_001436102.1	lncRNA
XM_012181326.2	MAP4K5	mRNA	XR_001436102.1	lncRNA
XM_012181326.2	MAP4K5	mRNA	XR_001436102.1	lncRNA
XM_015100438.1	CYLD	mRNA	XR_001436102.1	lncRNA
XM_015100438.1	CYLD	mRNA	XR_001436102.1	lncRNA
XM_015100438.1	CYLD	mRNA	XR_001436102.1	lncRNA
XM_015100438.1	CYLD	mRNA	XR_001436102.1	lncRNA
XM_015100438.1	CYLD	mRNA	XR_001436102.1	lncRNA

XM_015100438.1	CYLD	mRNA	XR_001436102.1	lncRNA
XM_015100438.1	CYLD	mRNA	XR_001436102.1	lncRNA
XM_004004866.2	METTL21A	mRNA	XR_001041211.2	lncRNA
XM_004004866.2	METTL21A	mRNA	XR_001041211.2	lncRNA
XM_004004866.2	METTL21A	mRNA	XR_001041211.2	lncRNA
XM_004004866.2	METTL21A	mRNA	XR_001041211.2	lncRNA
XM_004004866.2	METTL21A	mRNA	XR_001041211.2	lncRNA
XM_004004866.2	METTL21A	mRNA	XR_001041211.2	lncRNA
XM_004004866.2	METTL21A	mRNA	XR_001041211.2	lncRNA
XM_015092786.1	LOC106990898	mRNA	XR_001041211.2	lncRNA
XM_015092786.1	LOC106990898	mRNA	XR_001041211.2	lncRNA
XM_015092786.1	LOC106990898	mRNA	XR_001041211.2	lncRNA
XM_015092786.1	LOC106990898	mRNA	XR_001041211.2	lncRNA
XM_015092786.1	LOC106990898	mRNA	XR_001041211.2	lncRNA
XM_015092786.1	LOC106990898	mRNA	XR_001041211.2	lncRNA
XM_015092786.1	LOC106990898	mRNA	XR_001041211.2	lncRNA
XM_012183728.2	LOC101111337	mRNA	XR_001024743.2	lncRNA
XM_012183728.2	LOC101111337	mRNA	XR_001024743.2	lncRNA
XM_012183728.2	LOC101111337	mRNA	XR_001024743.2	lncRNA
XM_012183728.2	LOC101111337	mRNA	XR_001024743.2	lncRNA
XM_012183728.2	LOC101111337	mRNA	XR_001024743.2	lncRNA
XM_012183728.2	LOC101111337	mRNA	XR_001024743.2	lncRNA
XM_015096356.1	SEC24D	mRNA	XR_001020707.2	lncRNA
XM_015096356.1	SEC24D	mRNA	XR_001020707.2	lncRNA
XM_015096356.1	SEC24D	mRNA	XR_001020707.2	lncRNA
XM_015096356.1	SEC24D	mRNA	XR_001020707.2	lncRNA
XM_015096356.1	SEC24D	mRNA	XR_001020707.2	lncRNA
XM_015096356.1	SEC24D	mRNA	XR_001020707.2	lncRNA
XM_015096356.1	SEC24D	mRNA	XR_001020707.2	lncRNA
XM_015091978.1	ZCCHC11	mRNA	XR_001033284.2	lncRNA
XM_015091978.1	ZCCHC11	mRNA	XR_001033284.2	lncRNA
XM_015091978.1	ZCCHC11	mRNA	XR_001033284.2	lncRNA
XM_015091978.1	ZCCHC11	mRNA	XR_001033284.2	lncRNA
XM_015091978.1	ZCCHC11	mRNA	XR_001033284.2	lncRNA
XM_015091978.1	ZCCHC11	mRNA	XR_001033284.2	lncRNA
XM_015091978.1	ZCCHC11	mRNA	XR_001033284.2	lncRNA
XM_004020074.3	PIK3AP1	mRNA	XR_001033284.2	lncRNA
XM_004020074.3	PIK3AP1	mRNA	XR_001033284.2	lncRNA
XM_004020074.3	PIK3AP1	mRNA	XR_001033284.2	lncRNA
XM_004020074.3	PIK3AP1	mRNA	XR_001033284.2	lncRNA
XM_004020074.3	PIK3AP1	mRNA	XR_001033284.2	lncRNA
XM_004020074.3	PIK3AP1	mRNA	XR_001033284.2	lncRNA
XM_004020074.3	PIK3AP1	mRNA	XR_001033284.2	lncRNA
XM_012188301.2	NEBL	mRNA	XR_001033284.2	lncRNA
XM_012188301.2	NEBL	mRNA	XR_001033284.2	lncRNA
XM_012188301.2	NEBL	mRNA	XR_001033284.2	lncRNA
XM_012188301.2	NEBL	mRNA	XR_001033284.2	lncRNA
XM_012188301.2	NEBL	mRNA	XR_001033284.2	lncRNA
XM_012188301.2	NEBL	mRNA	XR_001033284.2	lncRNA
XM_012188301.2	NEBL	mRNA	XR_001033284.2	lncRNA
XM_015102318.1	SETD5	mRNA	XR_001020707.2	lncRNA
XM_015102318.1	SETD5	mRNA	XR_001020707.2	lncRNA
XM_015102318.1	SETD5	mRNA	XR_001020707.2	lncRNA
XM_015102318.1	SETD5	mRNA	XR_001020707.2	lncRNA

XM_015102318.1	SETD5	mRNA	XR_001020707.2	lncRNA
XM_015102318.1	SETD5	mRNA	XR_001020707.2	lncRNA
XM_015102318.1	SETD5	mRNA	XR_001020707.2	lncRNA
XM_012105656.2	ZNF596	mRNA	XR_001020707.2	lncRNA
XM_012105656.2	ZNF596	mRNA	XR_001020707.2	lncRNA
XM_012105656.2	ZNF596	mRNA	XR_001020707.2	lncRNA
XM_012105656.2	ZNF596	mRNA	XR_001020707.2	lncRNA
XM_012105656.2	ZNF596	mRNA	XR_001020707.2	lncRNA
XM_012105656.2	ZNF596	mRNA	XR_001020707.2	lncRNA
XM_012105656.2	ZNF596	mRNA	XR_001020707.2	lncRNA
XM_012119517.2	SORBS3	mRNA	XR_001020707.2	lncRNA
XM_012119517.2	SORBS3	mRNA	XR_001020707.2	lncRNA
XM_012119517.2	SORBS3	mRNA	XR_001020707.2	lncRNA
XM_012119517.2	SORBS3	mRNA	XR_001020707.2	lncRNA
XM_012119517.2	SORBS3	mRNA	XR_001020707.2	lncRNA
XM_012119517.2	SORBS3	mRNA	XR_001020707.2	lncRNA
XM_012119517.2	SORBS3	mRNA	XR_001020707.2	lncRNA
XM_012100690.1	KLC4	mRNA	XR_001033284.2	lncRNA
XM_012100690.1	KLC4	mRNA	XR_001033284.2	lncRNA
XM_012100690.1	KLC4	mRNA	XR_001033284.2	lncRNA
XM_012100690.1	KLC4	mRNA	XR_001033284.2	lncRNA
XM_012100690.1	KLC4	mRNA	XR_001033284.2	lncRNA
XM_012100690.1	KLC4	mRNA	XR_001033284.2	lncRNA
XM_012100690.1	KLC4	mRNA	XR_001033284.2	lncRNA
XM_012174703.2	ZBTB39	mRNA	TCONS_00022613	lncRNA
XM_012174703.2	ZBTB39	mRNA	TCONS_00022613	lncRNA
XM_012174703.2	ZBTB39	mRNA	TCONS_00022613	lncRNA
XM_012174703.2	ZBTB39	mRNA	TCONS_00022613	lncRNA
XM_012174703.2	ZBTB39	mRNA	TCONS_00022613	lncRNA
XM_012174703.2	ZBTB39	mRNA	TCONS_00022613	lncRNA
XM_012174703.2	ZBTB39	mRNA	TCONS_00022613	lncRNA
XM_015096937.1	BMF	mRNA	TCONS_00022613	lncRNA
XM_015096937.1	BMF	mRNA	TCONS_00022613	lncRNA
XM_015096937.1	BMF	mRNA	TCONS_00022613	lncRNA
XM_015096937.1	BMF	mRNA	TCONS_00022613	lncRNA
XM_015096937.1	BMF	mRNA	TCONS_00022613	lncRNA
XM_015096937.1	BMF	mRNA	TCONS_00022613	lncRNA
XM_015096937.1	BMF	mRNA	TCONS_00022613	lncRNA
XM_015103753.1	MBD1	mRNA	XR_001434761.1	lncRNA
XM_015103753.1	MBD1	mRNA	XR_001434761.1	lncRNA
XM_015103753.1	MBD1	mRNA	XR_001434761.1	lncRNA
XM_015103753.1	MBD1	mRNA	XR_001434761.1	lncRNA
XM_015103753.1	MBD1	mRNA	XR_001434761.1	lncRNA
XM_015103753.1	MBD1	mRNA	XR_001434761.1	lncRNA
XM_015103753.1	MBD1	mRNA	XR_001434761.1	lncRNA
XM_015096551.1	MOB1B	mRNA	XR_001434761.1	lncRNA
XM_015096551.1	MOB1B	mRNA	XR_001434761.1	lncRNA
XM_015096551.1	MOB1B	mRNA	XR_001434761.1	lncRNA
XM_015096551.1	MOB1B	mRNA	XR_001434761.1	lncRNA
XM_015096551.1	MOB1B	mRNA	XR_001434761.1	lncRNA
XM_015096551.1	MOB1B	mRNA	XR_001434761.1	lncRNA
XM_015096551.1	MOB1B	mRNA	XR_001434761.1	lncRNA
XM_012096185.2	TCP11L1	mRNA	XR_001433755.1	lncRNA
XM_012096185.2	TCP11L1	mRNA	XR_001433755.1	lncRNA

XM_012096185.2	TCP11L1	mRNA	XR_001433755.1	lncRNA
XM_012096185.2	TCP11L1	mRNA	XR_001433755.1	lncRNA
XM_012096185.2	TCP11L1	mRNA	XR_001433755.1	lncRNA
XM_012096185.2	TCP11L1	mRNA	XR_001433755.1	lncRNA
XM_012175416.2	PKN2	mRNA	XR_001433755.1	lncRNA
XM_012175416.2	PKN2	mRNA	XR_001433755.1	lncRNA
XM_012175416.2	PKN2	mRNA	XR_001433755.1	lncRNA
XM_012175416.2	PKN2	mRNA	XR_001433755.1	lncRNA
XM_012175416.2	PKN2	mRNA	XR_001433755.1	lncRNA
XM_012175416.2	PKN2	mRNA	XR_001433755.1	lncRNA
XM_015100041.1	ZNF473	mRNA	XR_001436127.1	lncRNA
XM_015100041.1	ZNF473	mRNA	XR_001436127.1	lncRNA
XM_015100041.1	ZNF473	mRNA	XR_001436127.1	lncRNA
XM_015100041.1	ZNF473	mRNA	XR_001436127.1	lncRNA
XM_015100041.1	ZNF473	mRNA	XR_001436127.1	lncRNA
XM_015100041.1	ZNF473	mRNA	XR_001436127.1	lncRNA
XM_015100041.1	ZNF473	mRNA	XR_001436127.1	lncRNA
XM_015094471.1	CCDC142	mRNA	XR_001436127.1	lncRNA
XM_015094471.1	CCDC142	mRNA	XR_001436127.1	lncRNA
XM_015094471.1	CCDC142	mRNA	XR_001436127.1	lncRNA
XM_015094471.1	CCDC142	mRNA	XR_001436127.1	lncRNA
XM_015094471.1	CCDC142	mRNA	XR_001436127.1	lncRNA
XM_015094471.1	CCDC142	mRNA	XR_001436127.1	lncRNA
XM_015094471.1	CCDC142	mRNA	XR_001436127.1	lncRNA
XM_004011865.3	ENPP2	mRNA	XR_001024290.1	lncRNA
XM_004011865.3	ENPP2	mRNA	XR_001024290.1	lncRNA
XM_004011865.3	ENPP2	mRNA	XR_001024290.1	lncRNA
XM_004011865.3	ENPP2	mRNA	XR_001024290.1	lncRNA
XM_004011865.3	ENPP2	mRNA	XR_001024290.1	lncRNA
XM_004011865.3	ENPP2	mRNA	XR_001024290.1	lncRNA
XM_004011865.3	ENPP2	mRNA	XR_001024290.1	lncRNA
XM_012105332.2	USP54	mRNA	XR_001025491.1	lncRNA
XM_012105332.2	USP54	mRNA	XR_001025491.1	lncRNA
XM_012105332.2	USP54	mRNA	XR_001025491.1	lncRNA
XM_012105332.2	USP54	mRNA	XR_001025491.1	lncRNA
XM_012105332.2	USP54	mRNA	XR_001025491.1	lncRNA
XM_012105332.2	USP54	mRNA	XR_001025491.1	lncRNA
XM_012105332.2	USP54	mRNA	XR_001025491.1	lncRNA
XM_012105319.2	USP54	mRNA	XR_001025491.1	lncRNA
XM_012105319.2	USP54	mRNA	XR_001025491.1	lncRNA
XM_012105319.2	USP54	mRNA	XR_001025491.1	lncRNA
XM_012105319.2	USP54	mRNA	XR_001025491.1	lncRNA
XM_012105319.2	USP54	mRNA	XR_001025491.1	lncRNA
XM_012105319.2	USP54	mRNA	XR_001025491.1	lncRNA
XM_012105319.2	USP54	mRNA	XR_001025491.1	lncRNA
XM_012104181.2	CCP110	mRNA	XR_001025491.1	lncRNA
XM_012104181.2	CCP110	mRNA	XR_001025491.1	lncRNA
XM_012104181.2	CCP110	mRNA	XR_001025491.1	lncRNA
XM_012104181.2	CCP110	mRNA	XR_001025491.1	lncRNA
XM_012104181.2	CCP110	mRNA	XR_001025491.1	lncRNA
XM_012104181.2	CCP110	mRNA	XR_001025491.1	lncRNA
XM_012104181.2	CCP110	mRNA	XR_001025491.1	lncRNA
XM_012188265.2	CCDC7	mRNA	XR_001434203.1	lncRNA
XM_012188265.2	CCDC7	mRNA	XR_001434203.1	lncRNA

XM_012188265.2	CCDC7	mRNA	XR_001434203.1	lncRNA
XM_012188265.2	CCDC7	mRNA	XR_001434203.1	lncRNA
XM_012188265.2	CCDC7	mRNA	XR_001434203.1	lncRNA
XM_012188265.2	CCDC7	mRNA	XR_001434203.1	lncRNA
XM_012188265.2	CCDC7	mRNA	XR_001434203.1	lncRNA
XM_012188265.2	CCDC7	mRNA	XR_001436817.1	lncRNA
XM_012188265.2	CCDC7	mRNA	XR_001436817.1	lncRNA
XM_012188265.2	CCDC7	mRNA	XR_001436817.1	lncRNA
XM_012188265.2	CCDC7	mRNA	XR_001436817.1	lncRNA
XM_012188265.2	CCDC7	mRNA	XR_001436817.1	lncRNA
XM_012188265.2	CCDC7	mRNA	XR_001436817.1	lncRNA
XM_012188265.2	CCDC7	mRNA	XR_001436817.1	lncRNA
XM_015098766.1	WIPF2	mRNA	XR_001436817.1	lncRNA
XM_015098766.1	WIPF2	mRNA	XR_001436817.1	lncRNA
XM_015098766.1	WIPF2	mRNA	XR_001436817.1	lncRNA
XM_015098766.1	WIPF2	mRNA	XR_001436817.1	lncRNA
XM_015098766.1	WIPF2	mRNA	XR_001436817.1	lncRNA
XM_015098766.1	WIPF2	mRNA	XR_001436817.1	lncRNA
XM_015098766.1	WIPF2	mRNA	XR_001436817.1	lncRNA
XM_012188276.2	CCDC7	mRNA	XR_001436817.1	lncRNA
XM_012188276.2	CCDC7	mRNA	XR_001436817.1	lncRNA
XM_012188276.2	CCDC7	mRNA	XR_001436817.1	lncRNA
XM_012188276.2	CCDC7	mRNA	XR_001436817.1	lncRNA
XM_012188276.2	CCDC7	mRNA	XR_001436817.1	lncRNA
XM_012188276.2	CCDC7	mRNA	XR_001436817.1	lncRNA
XM_012188276.2	CCDC7	mRNA	XR_001436817.1	lncRNA
XM_015098766.1	WIPF2	mRNA	XR_001434203.1	lncRNA
XM_015098766.1	WIPF2	mRNA	XR_001434203.1	lncRNA
XM_015098766.1	WIPF2	mRNA	XR_001434203.1	lncRNA
XM_015098766.1	WIPF2	mRNA	XR_001434203.1	lncRNA
XM_015098766.1	WIPF2	mRNA	XR_001434203.1	lncRNA
XM_015098766.1	WIPF2	mRNA	XR_001434203.1	lncRNA
XM_015098766.1	WIPF2	mRNA	XR_001434203.1	lncRNA
XM_012188214.2	ABI1	mRNA	XR_001436817.1	lncRNA
XM_012188214.2	ABI1	mRNA	XR_001436817.1	lncRNA
XM_012188214.2	ABI1	mRNA	XR_001436817.1	lncRNA
XM_012188214.2	ABI1	mRNA	XR_001436817.1	lncRNA
XM_012188214.2	ABI1	mRNA	XR_001436817.1	lncRNA
XM_012188214.2	ABI1	mRNA	XR_001436817.1	lncRNA
XM_012188214.2	ABI1	mRNA	XR_001436817.1	lncRNA
XM_012176498.2	RAPGEF5	mRNA	TCONS_00075453	lncRNA
XM_012176498.2	RAPGEF5	mRNA	TCONS_00075453	lncRNA
XM_012176498.2	RAPGEF5	mRNA	TCONS_00075453	lncRNA
XM_012176498.2	RAPGEF5	mRNA	TCONS_00075453	lncRNA
XM_012176498.2	RAPGEF5	mRNA	TCONS_00075453	lncRNA
XM_012176498.2	RAPGEF5	mRNA	TCONS_00075453	lncRNA
XM_012176498.2	RAPGEF5	mRNA	TCONS_00075453	lncRNA
XM_015098987.1	ABCA5	mRNA	XR_001434202.1	lncRNA
XM_015098987.1	ABCA5	mRNA	XR_001434202.1	lncRNA
XM_015098987.1	ABCA5	mRNA	XR_001434202.1	lncRNA
XM_015098987.1	ABCA5	mRNA	XR_001434202.1	lncRNA
XM_015098987.1	ABCA5	mRNA	XR_001434202.1	lncRNA
XM_015098987.1	ABCA5	mRNA	XR_001434202.1	lncRNA
XM_015098987.1	ABCA5	mRNA	XR_001434202.1	lncRNA

XM_004002178.3	LRRC8B	mRNA	XR_001434202.1	lncRNA
XM_004002178.3	LRRC8B	mRNA	XR_001434202.1	lncRNA
XM_004002178.3	LRRC8B	mRNA	XR_001434202.1	lncRNA
XM_004002178.3	LRRC8B	mRNA	XR_001434202.1	lncRNA
XM_004002178.3	LRRC8B	mRNA	XR_001434202.1	lncRNA
XM_004002178.3	LRRC8B	mRNA	XR_001434202.1	lncRNA
XM_004002178.3	LRRC8B	mRNA	XR_001434202.1	lncRNA
XM_004002178.3	LRRC8B	mRNA	XR_001034847.2	lncRNA
XM_004002178.3	LRRC8B	mRNA	XR_001034847.2	lncRNA
XM_004002178.3	LRRC8B	mRNA	XR_001034847.2	lncRNA
XM_004002178.3	LRRC8B	mRNA	XR_001034847.2	lncRNA
XM_004002178.3	LRRC8B	mRNA	XR_001034847.2	lncRNA
XM_004002178.3	LRRC8B	mRNA	XR_001034847.2	lncRNA
XM_004002178.3	LRRC8B	mRNA	XR_001034847.2	lncRNA
XM_015098987.1	ABCA5	mRNA	XR_001034847.2	lncRNA
XM_015098987.1	ABCA5	mRNA	XR_001034847.2	lncRNA
XM_015098987.1	ABCA5	mRNA	XR_001034847.2	lncRNA
XM_015098987.1	ABCA5	mRNA	XR_001034847.2	lncRNA
XM_015098987.1	ABCA5	mRNA	XR_001034847.2	lncRNA
XM_015098987.1	ABCA5	mRNA	XR_001034847.2	lncRNA
XM_015098987.1	ABCA5	mRNA	XR_001034847.2	lncRNA
XM_012178924.2	PHTF1	mRNA	XR_001021689.2	lncRNA
XM_012178924.2	PHTF1	mRNA	XR_001021689.2	lncRNA
XM_012178924.2	PHTF1	mRNA	XR_001021689.2	lncRNA
XM_012178924.2	PHTF1	mRNA	XR_001021689.2	lncRNA
XM_012178924.2	PHTF1	mRNA	XR_001021689.2	lncRNA
XM_012178924.2	PHTF1	mRNA	XR_001021689.2	lncRNA
XM_012178924.2	PHTF1	mRNA	XR_001436102.1	lncRNA
XM_012178924.2	PHTF1	mRNA	XR_001436102.1	lncRNA
XM_012178924.2	PHTF1	mRNA	XR_001436102.1	lncRNA
XM_012178924.2	PHTF1	mRNA	XR_001436102.1	lncRNA
XM_012178924.2	PHTF1	mRNA	XR_001436102.1	lncRNA
XM_012178924.2	PHTF1	mRNA	XR_001436102.1	lncRNA
XM_004002018.3	OMA1	mRNA	XR_001021689.2	lncRNA
XM_004002018.3	OMA1	mRNA	XR_001021689.2	lncRNA
XM_004002018.3	OMA1	mRNA	XR_001021689.2	lncRNA
XM_004002018.3	OMA1	mRNA	XR_001021689.2	lncRNA
XM_004002018.3	OMA1	mRNA	XR_001021689.2	lncRNA
XM_004002018.3	OMA1	mRNA	XR_001021689.2	lncRNA
XM_012106834.1	OPHN1	mRNA	XR_001436102.1	lncRNA
XM_012106834.1	OPHN1	mRNA	XR_001436102.1	lncRNA
XM_012106834.1	OPHN1	mRNA	XR_001436102.1	lncRNA
XM_012106834.1	OPHN1	mRNA	XR_001436102.1	lncRNA
XM_012106834.1	OPHN1	mRNA	XR_001436102.1	lncRNA
XM_012106834.1	OPHN1	mRNA	XR_001436102.1	lncRNA
XM_015096552.1	MOB1B	mRNA	XR_001436102.1	lncRNA
XM_015096552.1	MOB1B	mRNA	XR_001436102.1	lncRNA
XM_015096552.1	MOB1B	mRNA	XR_001436102.1	lncRNA
XM_015096552.1	MOB1B	mRNA	XR_001436102.1	lncRNA
XM_015096552.1	MOB1B	mRNA	XR_001436102.1	lncRNA
XM_015096552.1	MOB1B	mRNA	XR_001436102.1	lncRNA
XM_012190630.2	PLEKHA4	mRNA	XR_001436102.1	lncRNA
XM_012190630.2	PLEKHA4	mRNA	XR_001436102.1	lncRNA
XM_012190630.2	PLEKHA4	mRNA	XR_001436102.1	lncRNA

XM_012190630.2	PLEKHA4	mRNA	XR_001436102.1	lncRNA
XM_012190630.2	PLEKHA4	mRNA	XR_001436102.1	lncRNA
XM_012190630.2	PLEKHA4	mRNA	XR_001436102.1	lncRNA
XM_012190630.2	PLEKHA4	mRNA	XR_001436102.1	lncRNA
XM_004016103.2	USP2	mRNA	XR_001436102.1	lncRNA
XM_004016103.2	USP2	mRNA	XR_001436102.1	lncRNA
XM_004016103.2	USP2	mRNA	XR_001436102.1	lncRNA
XM_004016103.2	USP2	mRNA	XR_001436102.1	lncRNA
XM_004016103.2	USP2	mRNA	XR_001436102.1	lncRNA
XM_004016103.2	USP2	mRNA	XR_001436102.1	lncRNA
XM_004016103.2	USP2	mRNA	XR_001436102.1	lncRNA
XM_012181190.2	EIF2AK4	mRNA	XR_001436102.1	lncRNA
XM_012181190.2	EIF2AK4	mRNA	XR_001436102.1	lncRNA
XM_012181190.2	EIF2AK4	mRNA	XR_001436102.1	lncRNA
XM_012181190.2	EIF2AK4	mRNA	XR_001436102.1	lncRNA
XM_012181190.2	EIF2AK4	mRNA	XR_001436102.1	lncRNA
XM_012181190.2	EIF2AK4	mRNA	XR_001436102.1	lncRNA
XM_012181190.2	EIF2AK4	mRNA	XR_001436102.1	lncRNA
XM_015097018.1	ZNF280D	mRNA	XR_001021689.2	lncRNA
XM_015097018.1	ZNF280D	mRNA	XR_001021689.2	lncRNA
XM_015097018.1	ZNF280D	mRNA	XR_001021689.2	lncRNA
XM_015097018.1	ZNF280D	mRNA	XR_001021689.2	lncRNA
XM_015097018.1	ZNF280D	mRNA	XR_001021689.2	lncRNA
XM_015097018.1	ZNF280D	mRNA	XR_001021689.2	lncRNA
XM_015097018.1	ZNF280D	mRNA	XR_001021689.2	lncRNA
XM_012103119.1	EDRF1	mRNA	XR_001021689.2	lncRNA
XM_012103119.1	EDRF1	mRNA	XR_001021689.2	lncRNA
XM_012103119.1	EDRF1	mRNA	XR_001021689.2	lncRNA
XM_012103119.1	EDRF1	mRNA	XR_001021689.2	lncRNA
XM_012103119.1	EDRF1	mRNA	XR_001021689.2	lncRNA
XM_012103119.1	EDRF1	mRNA	XR_001021689.2	lncRNA
XM_012103119.1	EDRF1	mRNA	XR_001021689.2	lncRNA
XM_012095465.2	GOLGB1	mRNA	XR_001033284.2	lncRNA
XM_012095465.2	GOLGB1	mRNA	XR_001033284.2	lncRNA
XM_012095465.2	GOLGB1	mRNA	XR_001033284.2	lncRNA
XM_012095465.2	GOLGB1	mRNA	XR_001033284.2	lncRNA
XM_012095465.2	GOLGB1	mRNA	XR_001033284.2	lncRNA
XM_012095465.2	GOLGB1	mRNA	XR_001033284.2	lncRNA
XM_012095465.2	GOLGB1	mRNA	XR_001033284.2	lncRNA
XM_012127663.2	IFT74	mRNA	TCONS_00075453	lncRNA
XM_012127663.2	IFT74	mRNA	TCONS_00075453	lncRNA
XM_012127663.2	IFT74	mRNA	TCONS_00075453	lncRNA
XM_012127663.2	IFT74	mRNA	TCONS_00075453	lncRNA
XM_012127663.2	IFT74	mRNA	TCONS_00075453	lncRNA
XM_012127663.2	IFT74	mRNA	TCONS_00075453	lncRNA
XM_012127655.2	IFT74	mRNA	TCONS_00075453	lncRNA
XM_012127655.2	IFT74	mRNA	TCONS_00075453	lncRNA
XM_012127655.2	IFT74	mRNA	TCONS_00075453	lncRNA
XM_012127655.2	IFT74	mRNA	TCONS_00075453	lncRNA
XM_012127655.2	IFT74	mRNA	TCONS_00075453	lncRNA
XM_012127655.2	IFT74	mRNA	TCONS_00075453	lncRNA
XM_015102486.1	NR2C2	mRNA	TCONS_00075453	lncRNA
XM_015102486.1	NR2C2	mRNA	TCONS_00075453	lncRNA
XM_015102486.1	NR2C2	mRNA	TCONS_00075453	lncRNA

XM_015102486.1	NR2C2	mRNA	TCONS_00075453	lncRNA
XM_015102486.1	NR2C2	mRNA	TCONS_00075453	lncRNA
XM_015102486.1	NR2C2	mRNA	TCONS_00075453	lncRNA
XM_015102486.1	NR2C2	mRNA	TCONS_00075453	lncRNA
XM_015098446.1	VEZF1	mRNA	TCONS_00075453	lncRNA
XM_015098446.1	VEZF1	mRNA	TCONS_00075453	lncRNA
XM_015098446.1	VEZF1	mRNA	TCONS_00075453	lncRNA
XM_015098446.1	VEZF1	mRNA	TCONS_00075453	lncRNA
XM_015098446.1	VEZF1	mRNA	TCONS_00075453	lncRNA
XM_015098446.1	VEZF1	mRNA	TCONS_00075453	lncRNA
XM_015098446.1	VEZF1	mRNA	TCONS_00075453	lncRNA
XM_012190563.2	ZMYM1	mRNA	XR_001024290.1	lncRNA
XM_012190563.2	ZMYM1	mRNA	XR_001024290.1	lncRNA
XM_012190563.2	ZMYM1	mRNA	XR_001024290.1	lncRNA
XM_012190563.2	ZMYM1	mRNA	XR_001024290.1	lncRNA
XM_012190563.2	ZMYM1	mRNA	XR_001024290.1	lncRNA
XM_012190563.2	ZMYM1	mRNA	XR_001024290.1	lncRNA
XM_012112310.2	C2H9orf91	mRNA	TCONS_00078440	lncRNA
XM_012112310.2	C2H9orf91	mRNA	TCONS_00078440	lncRNA
XM_012112310.2	C2H9orf91	mRNA	TCONS_00078440	lncRNA
XM_012112310.2	C2H9orf91	mRNA	TCONS_00078440	lncRNA
XM_012112310.2	C2H9orf91	mRNA	TCONS_00078440	lncRNA
XM_012112310.2	C2H9orf91	mRNA	TCONS_00078440	lncRNA
XM_012188768.2	UBOX5	mRNA	TCONS_00078440	lncRNA
XM_012188768.2	UBOX5	mRNA	TCONS_00078440	lncRNA
XM_012188768.2	UBOX5	mRNA	TCONS_00078440	lncRNA
XM_012188768.2	UBOX5	mRNA	TCONS_00078440	lncRNA
XM_012188768.2	UBOX5	mRNA	TCONS_00078440	lncRNA
XM_012188768.2	UBOX5	mRNA	TCONS_00078440	lncRNA
XM_012182538.2	TRAPPC3L	mRNA	XR_001025491.1	lncRNA
XM_012182538.2	TRAPPC3L	mRNA	XR_001025491.1	lncRNA
XM_012182538.2	TRAPPC3L	mRNA	XR_001025491.1	lncRNA
XM_012182538.2	TRAPPC3L	mRNA	XR_001025491.1	lncRNA
XM_012182538.2	TRAPPC3L	mRNA	XR_001025491.1	lncRNA
XM_012182538.2	TRAPPC3L	mRNA	XR_001025491.1	lncRNA
XM_012182538.2	TRAPPC3L	mRNA	XR_001025491.1	lncRNA
XM_012178788.2	HMGXB3	mRNA	XR_001024290.1	lncRNA
XM_012178788.2	HMGXB3	mRNA	XR_001024290.1	lncRNA
XM_012178788.2	HMGXB3	mRNA	XR_001024290.1	lncRNA
XM_012178788.2	HMGXB3	mRNA	XR_001024290.1	lncRNA
XM_012178788.2	HMGXB3	mRNA	XR_001024290.1	lncRNA
XM_012178788.2	HMGXB3	mRNA	XR_001024290.1	lncRNA
XM_012178788.2	HMGXB3	mRNA	XR_001024290.1	lncRNA
XM_012183728.2	LOC10111133	mRNA	XR_001436127.1	lncRNA
XM_012183728.2	LOC10111133	mRNA	XR_001436127.1	lncRNA
XM_012183728.2	LOC10111133	mRNA	XR_001436127.1	lncRNA
XM_012183728.2	LOC10111133	mRNA	XR_001436127.1	lncRNA
XM_012183728.2	LOC10111133	mRNA	XR_001436127.1	lncRNA
XM_012183728.2	LOC10111133	mRNA	XR_001436127.1	lncRNA
XM_012183728.2	LOC10111133	mRNA	XR_001436127.1	lncRNA
XM_012101635.2	SYTL2	mRNA	XR_001044126.1	lncRNA
XM_012101635.2	SYTL2	mRNA	XR_001044126.1	lncRNA
XM_012101635.2	SYTL2	mRNA	XR_001044126.1	lncRNA
XM_012101635.2	SYTL2	mRNA	XR_001044126.1	lncRNA

XM_012101635.2	SYTL2	mRNA	XR_001044126.1	lncRNA
XM_012101635.2	SYTL2	mRNA	XR_001044126.1	lncRNA
XM_012101635.2	SYTL2	mRNA	XR_001044126.1	lncRNA
XM_012134416.2	PPIG	mRNA	XR_001024290.1	lncRNA
XM_012134416.2	PPIG	mRNA	XR_001024290.1	lncRNA
XM_012134416.2	PPIG	mRNA	XR_001024290.1	lncRNA
XM_012134416.2	PPIG	mRNA	XR_001024290.1	lncRNA
XM_012134416.2	PPIG	mRNA	XR_001024290.1	lncRNA
XM_012134416.2	PPIG	mRNA	XR_001024290.1	lncRNA
XM_012134416.2	PPIG	mRNA	XR_001024290.1	lncRNA
XM_012100080.2	ARIH2	mRNA	XR_001436532.1	lncRNA
XM_012100080.2	ARIH2	mRNA	XR_001436532.1	lncRNA
XM_012100080.2	ARIH2	mRNA	XR_001436532.1	lncRNA
XM_012100080.2	ARIH2	mRNA	XR_001436532.1	lncRNA
XM_012100080.2	ARIH2	mRNA	XR_001436532.1	lncRNA
XM_012100080.2	ARIH2	mRNA	XR_001436532.1	lncRNA
XM_012100080.2	ARIH2	mRNA	XR_001436532.1	lncRNA
XM_012179641.2	LOC10112286	mRNA	XR_001034847.2	lncRNA
XM_012179641.2	LOC10112286	mRNA	XR_001034847.2	lncRNA
XM_012179641.2	LOC10112286	mRNA	XR_001034847.2	lncRNA
XM_012179641.2	LOC10112286	mRNA	XR_001034847.2	lncRNA
XM_012179641.2	LOC10112286	mRNA	XR_001034847.2	lncRNA
XM_012179641.2	LOC10112286	mRNA	XR_001034847.2	lncRNA
XM_012179641.2	LOC10112286	mRNA	XR_001034847.2	lncRNA
XM_012178138.2	P4HA2	mRNA	XR_001434202.1	lncRNA
XM_012178138.2	P4HA2	mRNA	XR_001434202.1	lncRNA
XM_012178138.2	P4HA2	mRNA	XR_001434202.1	lncRNA
XM_012178138.2	P4HA2	mRNA	XR_001434202.1	lncRNA
XM_012178138.2	P4HA2	mRNA	XR_001434202.1	lncRNA
XM_012178138.2	P4HA2	mRNA	XR_001434202.1	lncRNA
XM_012178138.2	P4HA2	mRNA	XR_001434202.1	lncRNA
XM_012102658.2	ZNF518A	mRNA	XR_001034847.2	lncRNA
XM_012102658.2	ZNF518A	mRNA	XR_001034847.2	lncRNA
XM_012102658.2	ZNF518A	mRNA	XR_001034847.2	lncRNA
XM_012102658.2	ZNF518A	mRNA	XR_001034847.2	lncRNA
XM_012102658.2	ZNF518A	mRNA	XR_001034847.2	lncRNA
XM_012102658.2	ZNF518A	mRNA	XR_001034847.2	lncRNA
XM_012102658.2	ZNF518A	mRNA	XR_001034847.2	lncRNA
XM_012179641.2	LOC10112286	mRNA	XR_001434202.1	lncRNA
XM_012179641.2	LOC10112286	mRNA	XR_001434202.1	lncRNA
XM_012179641.2	LOC10112286	mRNA	XR_001434202.1	lncRNA
XM_012179641.2	LOC10112286	mRNA	XR_001434202.1	lncRNA
XM_012179641.2	LOC10112286	mRNA	XR_001434202.1	lncRNA
XM_012179641.2	LOC10112286	mRNA	XR_001434202.1	lncRNA
XM_012179641.2	LOC10112286	mRNA	XR_001434202.1	lncRNA
XM_012174768.2	RAB5B	mRNA	XR_001434202.1	lncRNA
XM_012174768.2	RAB5B	mRNA	XR_001434202.1	lncRNA
XM_012174768.2	RAB5B	mRNA	XR_001434202.1	lncRNA
XM_012174768.2	RAB5B	mRNA	XR_001434202.1	lncRNA
XM_012174768.2	RAB5B	mRNA	XR_001434202.1	lncRNA
XM_012174768.2	RAB5B	mRNA	XR_001434202.1	lncRNA
XM_012174768.2	RAB5B	mRNA	XR_001434202.1	lncRNA
XM_012178138.2	P4HA2	mRNA	XR_001034847.2	lncRNA
XM_012178138.2	P4HA2	mRNA	XR_001034847.2	lncRNA

XM_012178138.2	P4HA2	mRNA	XR_001034847.2	lncRNA
XM_012178138.2	P4HA2	mRNA	XR_001034847.2	lncRNA
XM_012178138.2	P4HA2	mRNA	XR_001034847.2	lncRNA
XM_012178138.2	P4HA2	mRNA	XR_001034847.2	lncRNA
XM_012178138.2	P4HA2	mRNA	XR_001034847.2	lncRNA
XM_012174768.2	RAB5B	mRNA	XR_001034847.2	lncRNA
XM_012174768.2	RAB5B	mRNA	XR_001034847.2	lncRNA
XM_012174768.2	RAB5B	mRNA	XR_001034847.2	lncRNA
XM_012174768.2	RAB5B	mRNA	XR_001034847.2	lncRNA
XM_012174768.2	RAB5B	mRNA	XR_001034847.2	lncRNA
XM_012174768.2	RAB5B	mRNA	XR_001034847.2	lncRNA
XM_012174768.2	RAB5B	mRNA	XR_001034847.2	lncRNA
XM_012102658.2	ZNF518A	mRNA	XR_001434202.1	lncRNA
XM_012102658.2	ZNF518A	mRNA	XR_001434202.1	lncRNA
XM_012102658.2	ZNF518A	mRNA	XR_001434202.1	lncRNA
XM_012102658.2	ZNF518A	mRNA	XR_001434202.1	lncRNA
XM_012102658.2	ZNF518A	mRNA	XR_001434202.1	lncRNA
XM_012102658.2	ZNF518A	mRNA	XR_001434202.1	lncRNA
XM_012102658.2	ZNF518A	mRNA	XR_001434202.1	lncRNA
XM_004010577.2	CGNL1	mRNA	XR_001033284.2	lncRNA
XM_004010577.2	CGNL1	mRNA	XR_001033284.2	lncRNA
XM_004010577.2	CGNL1	mRNA	XR_001033284.2	lncRNA
XM_004010577.2	CGNL1	mRNA	XR_001033284.2	lncRNA
XM_004010577.2	CGNL1	mRNA	XR_001033284.2	lncRNA
XM_004010577.2	CGNL1	mRNA	XR_001033284.2	lncRNA
XM_004010577.2	CGNL1	mRNA	XR_001033284.2	lncRNA
XM_012181456.2	ADAM10	mRNA	XR_001033284.2	lncRNA
XM_012181456.2	ADAM10	mRNA	XR_001033284.2	lncRNA
XM_012181456.2	ADAM10	mRNA	XR_001033284.2	lncRNA
XM_012181456.2	ADAM10	mRNA	XR_001033284.2	lncRNA
XM_012181456.2	ADAM10	mRNA	XR_001033284.2	lncRNA
XM_012181456.2	ADAM10	mRNA	XR_001033284.2	lncRNA
XM_012181456.2	ADAM10	mRNA	XR_001033284.2	lncRNA
NM_001308600.1	KRT17	mRNA	XR_001434761.1	lncRNA
NM_001308600.1	KRT17	mRNA	XR_001434761.1	lncRNA
NM_001308600.1	KRT17	mRNA	XR_001434761.1	lncRNA
NM_001308600.1	KRT17	mRNA	XR_001434761.1	lncRNA
NM_001308600.1	KRT17	mRNA	XR_001434761.1	lncRNA
NM_001308600.1	KRT17	mRNA	XR_001434761.1	lncRNA
NM_001308600.1	KRT17	mRNA	XR_001434761.1	lncRNA
XM_012100080.2	ARIH2	mRNA	XR_001041211.2	lncRNA
XM_012100080.2	ARIH2	mRNA	XR_001041211.2	lncRNA
XM_012100080.2	ARIH2	mRNA	XR_001041211.2	lncRNA
XM_012100080.2	ARIH2	mRNA	XR_001041211.2	lncRNA
XM_012100080.2	ARIH2	mRNA	XR_001041211.2	lncRNA
XM_012100080.2	ARIH2	mRNA	XR_001041211.2	lncRNA
XM_012100080.2	ARIH2	mRNA	XR_001041211.2	lncRNA
XM_012127663.2	IFT74	mRNA	XR_001033284.2	lncRNA
XM_012127663.2	IFT74	mRNA	XR_001033284.2	lncRNA
XM_012127663.2	IFT74	mRNA	XR_001033284.2	lncRNA
XM_012127663.2	IFT74	mRNA	XR_001033284.2	lncRNA
XM_012127663.2	IFT74	mRNA	XR_001033284.2	lncRNA
XM_012127663.2	IFT74	mRNA	XR_001033284.2	lncRNA
XM_012127655.2	IFT74	mRNA	XR_001033284.2	lncRNA

XM_012127655.2	IFT74	mRNA	XR_001033284.2	lncRNA
XM_012127655.2	IFT74	mRNA	XR_001033284.2	lncRNA
XM_012127655.2	IFT74	mRNA	XR_001033284.2	lncRNA
XM_012127655.2	IFT74	mRNA	XR_001033284.2	lncRNA
XM_012127655.2	IFT74	mRNA	XR_001033284.2	lncRNA
XM_012105690.2	WDR17	mRNA	XR_001436127.1	lncRNA
XM_012105690.2	WDR17	mRNA	XR_001436127.1	lncRNA
XM_012105690.2	WDR17	mRNA	XR_001436127.1	lncRNA
XM_012105690.2	WDR17	mRNA	XR_001436127.1	lncRNA
XM_012105690.2	WDR17	mRNA	XR_001436127.1	lncRNA
XM_012105690.2	WDR17	mRNA	XR_001436127.1	lncRNA
XM_012105690.2	WDR17	mRNA	XR_001436127.1	lncRNA
XM_012097035.1	C16H5orf42	mRNA	XR_001025491.1	lncRNA
XM_012097035.1	C16H5orf42	mRNA	XR_001025491.1	lncRNA
XM_012097035.1	C16H5orf42	mRNA	XR_001025491.1	lncRNA
XM_012097035.1	C16H5orf42	mRNA	XR_001025491.1	lncRNA
XM_012097035.1	C16H5orf42	mRNA	XR_001025491.1	lncRNA
XM_012097035.1	C16H5orf42	mRNA	XR_001025491.1	lncRNA
XM_012097035.1	C16H5orf42	mRNA	XR_001025491.1	lncRNA
XM_012099038.2	SLC25A29	mRNA	XR_001025491.1	lncRNA
XM_012099038.2	SLC25A29	mRNA	XR_001025491.1	lncRNA
XM_012099038.2	SLC25A29	mRNA	XR_001025491.1	lncRNA
XM_012099038.2	SLC25A29	mRNA	XR_001025491.1	lncRNA
XM_012099038.2	SLC25A29	mRNA	XR_001025491.1	lncRNA
XM_012099038.2	SLC25A29	mRNA	XR_001025491.1	lncRNA
XM_012099038.2	SLC25A29	mRNA	XR_001025491.1	lncRNA
XM_012152472.1	LUZP1	mRNA	XR_001044126.1	lncRNA
XM_012152472.1	LUZP1	mRNA	XR_001044126.1	lncRNA
XM_012152472.1	LUZP1	mRNA	XR_001044126.1	lncRNA
XM_012152472.1	LUZP1	mRNA	XR_001044126.1	lncRNA
XM_012152472.1	LUZP1	mRNA	XR_001044126.1	lncRNA
XM_012152472.1	LUZP1	mRNA	XR_001044126.1	lncRNA
XM_012152472.1	LUZP1	mRNA	XR_001044126.1	lncRNA
XM_012105331.2	USP54	mRNA	XR_001021689.2	lncRNA
XM_012105331.2	USP54	mRNA	XR_001021689.2	lncRNA
XM_012105331.2	USP54	mRNA	XR_001021689.2	lncRNA
XM_012105331.2	USP54	mRNA	XR_001021689.2	lncRNA
XM_012105331.2	USP54	mRNA	XR_001021689.2	lncRNA
XM_012105331.2	USP54	mRNA	XR_001021689.2	lncRNA
XM_012105331.2	USP54	mRNA	XR_001021689.2	lncRNA
XM_012101919.2	ZBTB44	mRNA	XR_001436102.1	lncRNA
XM_012101919.2	ZBTB44	mRNA	XR_001436102.1	lncRNA
XM_012101919.2	ZBTB44	mRNA	XR_001436102.1	lncRNA
XM_012101919.2	ZBTB44	mRNA	XR_001436102.1	lncRNA
XM_012101919.2	ZBTB44	mRNA	XR_001436102.1	lncRNA
XM_012101919.2	ZBTB44	mRNA	XR_001436102.1	lncRNA
XM_012101919.2	ZBTB44	mRNA	XR_001436102.1	lncRNA
XM_015103117.1	ZBTB44	mRNA	XR_001436102.1	lncRNA
XM_015103117.1	ZBTB44	mRNA	XR_001436102.1	lncRNA
XM_015103117.1	ZBTB44	mRNA	XR_001436102.1	lncRNA
XM_015103117.1	ZBTB44	mRNA	XR_001436102.1	lncRNA
XM_015103117.1	ZBTB44	mRNA	XR_001436102.1	lncRNA
XM_015103117.1	ZBTB44	mRNA	XR_001436102.1	lncRNA
XM_015103117.1	ZBTB44	mRNA	XR_001436102.1	lncRNA

XM_015101485.1	LOC106991694	mRNA	XR_001021689.2	lncRNA
XM_015101485.1	LOC106991694	mRNA	XR_001021689.2	lncRNA
XM_015101485.1	LOC106991694	mRNA	XR_001021689.2	lncRNA
XM_015101485.1	LOC106991694	mRNA	XR_001021689.2	lncRNA
XM_015101485.1	LOC106991694	mRNA	XR_001021689.2	lncRNA
XM_015101485.1	LOC106991694	mRNA	XR_001021689.2	lncRNA
XM_015101485.1	LOC106991694	mRNA	XR_001021689.2	lncRNA
XM_012187546.2	ANKRD45	mRNA	TCONS_00022613	lncRNA
XM_012187546.2	ANKRD45	mRNA	TCONS_00022613	lncRNA
XM_012187546.2	ANKRD45	mRNA	TCONS_00022613	lncRNA
XM_012187546.2	ANKRD45	mRNA	TCONS_00022613	lncRNA
XM_012187546.2	ANKRD45	mRNA	TCONS_00022613	lncRNA
XM_012187546.2	ANKRD45	mRNA	TCONS_00022613	lncRNA
XM_012131583.2	FAM171B	mRNA	TCONS_00022613	lncRNA
XM_012131583.2	FAM171B	mRNA	TCONS_00022613	lncRNA
XM_012131583.2	FAM171B	mRNA	TCONS_00022613	lncRNA
XM_012131583.2	FAM171B	mRNA	TCONS_00022613	lncRNA
XM_012131583.2	FAM171B	mRNA	TCONS_00022613	lncRNA
XM_012131583.2	FAM171B	mRNA	TCONS_00022613	lncRNA
XM_004002178.3	LRRC8B	mRNA	TCONS_00022613	lncRNA
XM_004002178.3	LRRC8B	mRNA	TCONS_00022613	lncRNA
XM_004002178.3	LRRC8B	mRNA	TCONS_00022613	lncRNA
XM_004002178.3	LRRC8B	mRNA	TCONS_00022613	lncRNA
XM_004002178.3	LRRC8B	mRNA	TCONS_00022613	lncRNA
XM_004002178.3	LRRC8B	mRNA	TCONS_00022613	lncRNA
XM_012105396.2	C25H10orf11	mRNA	TCONS_00022613	lncRNA
XM_012105396.2	C25H10orf11	mRNA	TCONS_00022613	lncRNA
XM_012105396.2	C25H10orf11	mRNA	TCONS_00022613	lncRNA
XM_012105396.2	C25H10orf11	mRNA	TCONS_00022613	lncRNA
XM_012105396.2	C25H10orf11	mRNA	TCONS_00022613	lncRNA
XM_012105396.2	C25H10orf11	mRNA	TCONS_00022613	lncRNA
NM_001308600.1	KRT17	mRNA	TCONS_00022613	lncRNA
NM_001308600.1	KRT17	mRNA	TCONS_00022613	lncRNA
NM_001308600.1	KRT17	mRNA	TCONS_00022613	lncRNA
NM_001308600.1	KRT17	mRNA	TCONS_00022613	lncRNA
NM_001308600.1	KRT17	mRNA	TCONS_00022613	lncRNA
NM_001308600.1	KRT17	mRNA	TCONS_00022613	lncRNA
XM_012105688.2	WDR17	mRNA	XR_001024743.2	lncRNA
XM_012105688.2	WDR17	mRNA	XR_001024743.2	lncRNA
XM_012105688.2	WDR17	mRNA	XR_001024743.2	lncRNA
XM_012105688.2	WDR17	mRNA	XR_001024743.2	lncRNA
XM_012105688.2	WDR17	mRNA	XR_001024743.2	lncRNA
XM_012105688.2	WDR17	mRNA	XR_001024743.2	lncRNA
XM_015103387.1	C22H10orf76	mRNA	XR_001434761.1	lncRNA
XM_015103387.1	C22H10orf76	mRNA	XR_001434761.1	lncRNA
XM_015103387.1	C22H10orf76	mRNA	XR_001434761.1	lncRNA
XM_015103387.1	C22H10orf76	mRNA	XR_001434761.1	lncRNA
XM_015103387.1	C22H10orf76	mRNA	XR_001434761.1	lncRNA
XM_015103387.1	C22H10orf76	mRNA	XR_001434761.1	lncRNA
XM_012184446.2	RCBTB2	mRNA	XR_001434892.1	lncRNA
XM_012184446.2	RCBTB2	mRNA	XR_001434892.1	lncRNA
XM_012184446.2	RCBTB2	mRNA	XR_001434892.1	lncRNA
XM_012184446.2	RCBTB2	mRNA	XR_001434892.1	lncRNA
XM_012184446.2	RCBTB2	mRNA	XR_001434892.1	lncRNA

XM_012184446.2	RCBTB2	mRNA	XR_001434892.1	lncRNA
XM_012178848.2	CNOT8	mRNA	XR_001434892.1	lncRNA
XM_012178848.2	CNOT8	mRNA	XR_001434892.1	lncRNA
XM_012178848.2	CNOT8	mRNA	XR_001434892.1	lncRNA
XM_012178848.2	CNOT8	mRNA	XR_001434892.1	lncRNA
XM_012178848.2	CNOT8	mRNA	XR_001434892.1	lncRNA
XM_012178848.2	CNOT8	mRNA	XR_001434892.1	lncRNA
XM_012175045.1	FGD4	mRNA	XR_001433755.1	lncRNA
XM_012175045.1	FGD4	mRNA	XR_001433755.1	lncRNA
XM_012175045.1	FGD4	mRNA	XR_001433755.1	lncRNA
XM_012175045.1	FGD4	mRNA	XR_001433755.1	lncRNA
XM_012175045.1	FGD4	mRNA	XR_001433755.1	lncRNA
XM_012175045.1	FGD4	mRNA	XR_001433755.1	lncRNA
XM_012094704.2	LOC10560198	mRNA	TCONS_00054422	lncRNA
XM_012094704.2	LOC10560198	mRNA	TCONS_00054422	lncRNA
XM_012094704.2	LOC10560198	mRNA	TCONS_00054422	lncRNA
XM_012094704.2	LOC10560198	mRNA	TCONS_00054422	lncRNA
XM_012094704.2	LOC10560198	mRNA	TCONS_00054422	lncRNA
XM_004014209.3	CUL2	mRNA	XR_001436931.1	lncRNA
XM_004014209.3	CUL2	mRNA	XR_001436931.1	lncRNA
XM_004014209.3	CUL2	mRNA	XR_001436931.1	lncRNA
XM_004014209.3	CUL2	mRNA	XR_001436931.1	lncRNA
XM_004014209.3	CUL2	mRNA	XR_001436931.1	lncRNA
XM_012146477.2	ABCB6	mRNA	XR_001024290.1	lncRNA
XM_012146477.2	ABCB6	mRNA	XR_001024290.1	lncRNA
XM_012146477.2	ABCB6	mRNA	XR_001024290.1	lncRNA
XM_012146477.2	ABCB6	mRNA	XR_001024290.1	lncRNA
XM_012146477.2	ABCB6	mRNA	XR_001024290.1	lncRNA
XM_012146477.2	ABCB6	mRNA	XR_001024290.1	lncRNA
XM_015103641.1	YES1	mRNA	XR_001436127.1	lncRNA
XM_015103641.1	YES1	mRNA	XR_001436127.1	lncRNA
XM_015103641.1	YES1	mRNA	XR_001436127.1	lncRNA
XM_015103641.1	YES1	mRNA	XR_001436127.1	lncRNA
XM_015103641.1	YES1	mRNA	XR_001436127.1	lncRNA
XM_015103641.1	YES1	mRNA	XR_001436127.1	lncRNA
XM_015091996.1	LOC10112163	mRNA	XR_001024743.2	lncRNA
XM_015091996.1	LOC10112163	mRNA	XR_001024743.2	lncRNA
XM_015091996.1	LOC10112163	mRNA	XR_001024743.2	lncRNA
XM_015091996.1	LOC10112163	mRNA	XR_001024743.2	lncRNA
XM_015091996.1	LOC10112163	mRNA	XR_001024743.2	lncRNA
XM_015091996.1	LOC10112163	mRNA	XR_001024743.2	lncRNA
XM_012096185.2	TCP11L1	mRNA	XR_001434892.1	lncRNA
XM_012096185.2	TCP11L1	mRNA	XR_001434892.1	lncRNA
XM_012096185.2	TCP11L1	mRNA	XR_001434892.1	lncRNA
XM_012096185.2	TCP11L1	mRNA	XR_001434892.1	lncRNA
XM_012096185.2	TCP11L1	mRNA	XR_001434892.1	lncRNA
XM_012096185.2	TCP11L1	mRNA	XR_001434892.1	lncRNA
XM_012096185.2	TCP11L1	mRNA	XR_001434892.1	lncRNA
XM_012122817.1	RNF220	mRNA	XR_001033284.2	lncRNA
XM_012122817.1	RNF220	mRNA	XR_001033284.2	lncRNA
XM_012122817.1	RNF220	mRNA	XR_001033284.2	lncRNA
XM_012122817.1	RNF220	mRNA	XR_001033284.2	lncRNA
XM_012122817.1	RNF220	mRNA	XR_001033284.2	lncRNA
XM_012122817.1	RNF220	mRNA	XR_001033284.2	lncRNA

XM_012122817.1	RNF220	mRNA	XR_001033284.2	lncRNA
XM_015103495.1	UROS	mRNA	XR_001021689.2	lncRNA
XM_015103495.1	UROS	mRNA	XR_001021689.2	lncRNA
XM_015103495.1	UROS	mRNA	XR_001021689.2	lncRNA
XM_015103495.1	UROS	mRNA	XR_001021689.2	lncRNA
XM_015103495.1	UROS	mRNA	XR_001021689.2	lncRNA
XM_015103495.1	UROS	mRNA	XR_001021689.2	lncRNA
XM_015103495.1	UROS	mRNA	XR_001021689.2	lncRNA
XM_012176510.2	FNBP1L	mRNA	XR_001436102.1	lncRNA
XM_012176510.2	FNBP1L	mRNA	XR_001436102.1	lncRNA
XM_012176510.2	FNBP1L	mRNA	XR_001436102.1	lncRNA
XM_012176510.2	FNBP1L	mRNA	XR_001436102.1	lncRNA
XM_012176510.2	FNBP1L	mRNA	XR_001436102.1	lncRNA
XM_012176510.2	FNBP1L	mRNA	XR_001436102.1	lncRNA
XM_012176510.2	FNBP1L	mRNA	XR_001436102.1	lncRNA
XM_015095790.1	CC2D1A	mRNA	XR_001436102.1	lncRNA
XM_015095790.1	CC2D1A	mRNA	XR_001436102.1	lncRNA
XM_015095790.1	CC2D1A	mRNA	XR_001436102.1	lncRNA
XM_015095790.1	CC2D1A	mRNA	XR_001436102.1	lncRNA
XM_015095790.1	CC2D1A	mRNA	XR_001436102.1	lncRNA
XM_015095790.1	CC2D1A	mRNA	XR_001436102.1	lncRNA
XM_015095790.1	CC2D1A	mRNA	XR_001436102.1	lncRNA
XM_015097502.1	SNX9	mRNA	XR_001021689.2	lncRNA
XM_015097502.1	SNX9	mRNA	XR_001021689.2	lncRNA
XM_015097502.1	SNX9	mRNA	XR_001021689.2	lncRNA
XM_015097502.1	SNX9	mRNA	XR_001021689.2	lncRNA
XM_015097502.1	SNX9	mRNA	XR_001021689.2	lncRNA
XM_015097502.1	SNX9	mRNA	XR_001021689.2	lncRNA
XM_015097502.1	SNX9	mRNA	XR_001021689.2	lncRNA
XM_015104177.1	PRSS8	mRNA	XR_001021689.2	lncRNA
XM_015104177.1	PRSS8	mRNA	XR_001021689.2	lncRNA
XM_015104177.1	PRSS8	mRNA	XR_001021689.2	lncRNA
XM_015104177.1	PRSS8	mRNA	XR_001021689.2	lncRNA
XM_015104177.1	PRSS8	mRNA	XR_001021689.2	lncRNA
XM_015104177.1	PRSS8	mRNA	XR_001021689.2	lncRNA
XM_015093666.1	PIGV	mRNA	XR_001021689.2	lncRNA
XM_015093666.1	PIGV	mRNA	XR_001021689.2	lncRNA
XM_015093666.1	PIGV	mRNA	XR_001021689.2	lncRNA
XM_015093666.1	PIGV	mRNA	XR_001021689.2	lncRNA
XM_015093666.1	PIGV	mRNA	XR_001021689.2	lncRNA
XM_015093666.1	PIGV	mRNA	XR_001021689.2	lncRNA
XM_012102549.2	CEP70	mRNA	XR_001436102.1	lncRNA
XM_012102549.2	CEP70	mRNA	XR_001436102.1	lncRNA
XM_012102549.2	CEP70	mRNA	XR_001436102.1	lncRNA
XM_012102549.2	CEP70	mRNA	XR_001436102.1	lncRNA
XM_012102549.2	CEP70	mRNA	XR_001436102.1	lncRNA
XM_012102549.2	CEP70	mRNA	XR_001436102.1	lncRNA
XM_012186312.2	DDX42	mRNA	XR_001436102.1	lncRNA
XM_012186312.2	DDX42	mRNA	XR_001436102.1	lncRNA
XM_012186312.2	DDX42	mRNA	XR_001436102.1	lncRNA
XM_012186312.2	DDX42	mRNA	XR_001436102.1	lncRNA
XM_012186312.2	DDX42	mRNA	XR_001436102.1	lncRNA
XM_012186312.2	DDX42	mRNA	XR_001436102.1	lncRNA
XM_012094768.2	ZMYM1	mRNA	XR_001436102.1	lncRNA

XM_012094768.2	ZMYM1	mRNA	XR_001436102.1	lncRNA
XM_012094768.2	ZMYM1	mRNA	XR_001436102.1	lncRNA
XM_012094768.2	ZMYM1	mRNA	XR_001436102.1	lncRNA
XM_012094768.2	ZMYM1	mRNA	XR_001436102.1	lncRNA
XM_012094768.2	ZMYM1	mRNA	XR_001436102.1	lncRNA
XM_015105096.1	PAK3	mRNA	XR_001433755.1	lncRNA
XM_015105096.1	PAK3	mRNA	XR_001433755.1	lncRNA
XM_015105096.1	PAK3	mRNA	XR_001433755.1	lncRNA
XM_015105096.1	PAK3	mRNA	XR_001433755.1	lncRNA
XM_015105096.1	PAK3	mRNA	XR_001433755.1	lncRNA
XM_015105096.1	PAK3	mRNA	XR_001433755.1	lncRNA
XM_015100519.1	NFAT5	mRNA	XR_001433755.1	lncRNA
XM_015100519.1	NFAT5	mRNA	XR_001433755.1	lncRNA
XM_015100519.1	NFAT5	mRNA	XR_001433755.1	lncRNA
XM_015100519.1	NFAT5	mRNA	XR_001433755.1	lncRNA
XM_015100519.1	NFAT5	mRNA	XR_001433755.1	lncRNA
XM_015100519.1	NFAT5	mRNA	XR_001433755.1	lncRNA
XM_012190139.2	ZMYM6	mRNA	XR_001033284.2	lncRNA
XM_012190139.2	ZMYM6	mRNA	XR_001033284.2	lncRNA
XM_012190139.2	ZMYM6	mRNA	XR_001033284.2	lncRNA
XM_012190139.2	ZMYM6	mRNA	XR_001033284.2	lncRNA
XM_012190139.2	ZMYM6	mRNA	XR_001033284.2	lncRNA
XM_012190139.2	ZMYM6	mRNA	XR_001033284.2	lncRNA
XM_012102658.2	ZNF518A	mRNA	TCONS_00022613	lncRNA
XM_012102658.2	ZNF518A	mRNA	TCONS_00022613	lncRNA
XM_012102658.2	ZNF518A	mRNA	TCONS_00022613	lncRNA
XM_012102658.2	ZNF518A	mRNA	TCONS_00022613	lncRNA
XM_012102658.2	ZNF518A	mRNA	TCONS_00022613	lncRNA
XM_012102658.2	ZNF518A	mRNA	TCONS_00022613	lncRNA
XM_012102669.1	ZNF518A	mRNA	TCONS_00022613	lncRNA
XM_012102669.1	ZNF518A	mRNA	TCONS_00022613	lncRNA
XM_012102669.1	ZNF518A	mRNA	TCONS_00022613	lncRNA
XM_012102669.1	ZNF518A	mRNA	TCONS_00022613	lncRNA
XM_012102669.1	ZNF518A	mRNA	TCONS_00022613	lncRNA
XM_012102669.1	ZNF518A	mRNA	TCONS_00022613	lncRNA
XM_004008647.3	RAPGEF6	mRNA	TCONS_00022613	lncRNA
XM_004008647.3	RAPGEF6	mRNA	TCONS_00022613	lncRNA
XM_004008647.3	RAPGEF6	mRNA	TCONS_00022613	lncRNA
XM_004008647.3	RAPGEF6	mRNA	TCONS_00022613	lncRNA
XM_004008647.3	RAPGEF6	mRNA	TCONS_00022613	lncRNA
XM_004008647.3	RAPGEF6	mRNA	TCONS_00022613	lncRNA
XM_012174768.2	RAB5B	mRNA	TCONS_00022613	lncRNA
XM_012174768.2	RAB5B	mRNA	TCONS_00022613	lncRNA
XM_012174768.2	RAB5B	mRNA	TCONS_00022613	lncRNA
XM_012174768.2	RAB5B	mRNA	TCONS_00022613	lncRNA
XM_012174768.2	RAB5B	mRNA	TCONS_00022613	lncRNA
XM_012174768.2	RAB5B	mRNA	TCONS_00022613	lncRNA
XM_012178848.2	CNOT8	mRNA	XR_001436532.1	lncRNA
XM_012178848.2	CNOT8	mRNA	XR_001436532.1	lncRNA
XM_012178848.2	CNOT8	mRNA	XR_001436532.1	lncRNA
XM_012178848.2	CNOT8	mRNA	XR_001436532.1	lncRNA
XM_012178848.2	CNOT8	mRNA	XR_001436532.1	lncRNA
XM_012178848.2	CNOT8	mRNA	XR_001436532.1	lncRNA
XM_012185094.2	STRADA	mRNA	XR_001042381.2	lncRNA

XM_012185094.2	STRADA	mRNA	XR_001042381.2	lncRNA
XM_012185094.2	STRADA	mRNA	XR_001042381.2	lncRNA
XM_012185094.2	STRADA	mRNA	XR_001042381.2	lncRNA
XM_012185094.2	STRADA	mRNA	XR_001042381.2	lncRNA
XM_012185094.2	STRADA	mRNA	XR_001042381.2	lncRNA
XM_004002018.3	OMA1	mRNA	XR_001436532.1	lncRNA
XM_004002018.3	OMA1	mRNA	XR_001436532.1	lncRNA
XM_004002018.3	OMA1	mRNA	XR_001436532.1	lncRNA
XM_004002018.3	OMA1	mRNA	XR_001436532.1	lncRNA
XM_004002018.3	OMA1	mRNA	XR_001436532.1	lncRNA
XM_004002018.3	OMA1	mRNA	XR_001436532.1	lncRNA
XM_012099693.2	EOGT	mRNA	XR_001436532.1	lncRNA
XM_012099693.2	EOGT	mRNA	XR_001436532.1	lncRNA
XM_012099693.2	EOGT	mRNA	XR_001436532.1	lncRNA
XM_012099693.2	EOGT	mRNA	XR_001436532.1	lncRNA
XM_012099693.2	EOGT	mRNA	XR_001436532.1	lncRNA
XM_012099693.2	EOGT	mRNA	XR_001436532.1	lncRNA
XM_004010651.3	SORD	mRNA	XR_001436532.1	lncRNA
XM_004010651.3	SORD	mRNA	XR_001436532.1	lncRNA
XM_004010651.3	SORD	mRNA	XR_001436532.1	lncRNA
XM_004010651.3	SORD	mRNA	XR_001436532.1	lncRNA
XM_004010651.3	SORD	mRNA	XR_001436532.1	lncRNA
XM_004010651.3	SORD	mRNA	XR_001436532.1	lncRNA
XM_015099539.1	KIN	mRNA	XR_001436532.1	lncRNA
XM_015099539.1	KIN	mRNA	XR_001436532.1	lncRNA
XM_015099539.1	KIN	mRNA	XR_001436532.1	lncRNA
XM_015099539.1	KIN	mRNA	XR_001436532.1	lncRNA
XM_015099539.1	KIN	mRNA	XR_001436532.1	lncRNA
XM_015099539.1	KIN	mRNA	XR_001436532.1	lncRNA
XM_015094624.1	PCBP2	mRNA	XR_001436532.1	lncRNA
XM_015094624.1	PCBP2	mRNA	XR_001436532.1	lncRNA
XM_015094624.1	PCBP2	mRNA	XR_001436532.1	lncRNA
XM_015094624.1	PCBP2	mRNA	XR_001436532.1	lncRNA
XM_015094624.1	PCBP2	mRNA	XR_001436532.1	lncRNA
XM_015094624.1	PCBP2	mRNA	XR_001436532.1	lncRNA
XM_004011402.3	STXBP5	mRNA	XR_001034847.2	lncRNA
XM_004011402.3	STXBP5	mRNA	XR_001034847.2	lncRNA
XM_004011402.3	STXBP5	mRNA	XR_001034847.2	lncRNA
XM_004011402.3	STXBP5	mRNA	XR_001034847.2	lncRNA
XM_004011402.3	STXBP5	mRNA	XR_001034847.2	lncRNA
XM_004011402.3	STXBP5	mRNA	XR_001034847.2	lncRNA
XM_004011402.3	STXBP5	mRNA	XR_001034847.2	lncRNA
XM_004011402.3	STXBP5	mRNA	XR_001434202.1	lncRNA
XM_004011402.3	STXBP5	mRNA	XR_001434202.1	lncRNA
XM_004011402.3	STXBP5	mRNA	XR_001434202.1	lncRNA
XM_004011402.3	STXBP5	mRNA	XR_001434202.1	lncRNA
XM_004011402.3	STXBP5	mRNA	XR_001434202.1	lncRNA
XM_004011402.3	STXBP5	mRNA	XR_001434202.1	lncRNA
XM_004011402.3	STXBP5	mRNA	XR_001434202.1	lncRNA
XM_012180705.1	PAPD4	mRNA	TCONS_00075051	lncRNA
XM_012180705.1	PAPD4	mRNA	TCONS_00075051	lncRNA
XM_012180705.1	PAPD4	mRNA	TCONS_00075051	lncRNA
XM_012180705.1	PAPD4	mRNA	TCONS_00075051	lncRNA
XM_012180705.1	PAPD4	mRNA	TCONS_00075051	lncRNA

XM_012102216.2	TFDP2	mRNA	TCONS_00075051	lncRNA
XM_012102216.2	TFDP2	mRNA	TCONS_00075051	lncRNA
XM_012102216.2	TFDP2	mRNA	TCONS_00075051	lncRNA
XM_012102216.2	TFDP2	mRNA	TCONS_00075051	lncRNA
XM_012102216.2	TFDP2	mRNA	TCONS_00075051	lncRNA
XM_012101765.1	PRMT3	mRNA	TCONS_00075051	lncRNA
XM_012101765.1	PRMT3	mRNA	TCONS_00075051	lncRNA
XM_012101765.1	PRMT3	mRNA	TCONS_00075051	lncRNA
XM_012101765.1	PRMT3	mRNA	TCONS_00075051	lncRNA
XM_012101765.1	PRMT3	mRNA	TCONS_00075051	lncRNA
XM_012184946.1	LIG4	mRNA	XR_001041211.2	lncRNA
XM_012184946.1	LIG4	mRNA	XR_001041211.2	lncRNA
XM_012184946.1	LIG4	mRNA	XR_001041211.2	lncRNA
XM_012184946.1	LIG4	mRNA	XR_001041211.2	lncRNA
XM_012184946.1	LIG4	mRNA	XR_001041211.2	lncRNA
XM_012184946.1	LIG4	mRNA	XR_001041211.2	lncRNA
XM_012180244.2	BMP2K	mRNA	XR_001041211.2	lncRNA
XM_012180244.2	BMP2K	mRNA	XR_001041211.2	lncRNA
XM_012180244.2	BMP2K	mRNA	XR_001041211.2	lncRNA
XM_012180244.2	BMP2K	mRNA	XR_001041211.2	lncRNA
XM_012180244.2	BMP2K	mRNA	XR_001041211.2	lncRNA
XM_012180244.2	BMP2K	mRNA	XR_001041211.2	lncRNA
XM_012099693.2	EOGT	mRNA	XR_001041211.2	lncRNA
XM_012099693.2	EOGT	mRNA	XR_001041211.2	lncRNA
XM_012099693.2	EOGT	mRNA	XR_001041211.2	lncRNA
XM_012099693.2	EOGT	mRNA	XR_001041211.2	lncRNA
XM_012099693.2	EOGT	mRNA	XR_001041211.2	lncRNA
XM_012099693.2	EOGT	mRNA	XR_001041211.2	lncRNA
XM_012117587.2	KIF27	mRNA	XR_001041211.2	lncRNA
XM_012117587.2	KIF27	mRNA	XR_001041211.2	lncRNA
XM_012117587.2	KIF27	mRNA	XR_001041211.2	lncRNA
XM_012117587.2	KIF27	mRNA	XR_001041211.2	lncRNA
XM_012117587.2	KIF27	mRNA	XR_001041211.2	lncRNA
XM_012117587.2	KIF27	mRNA	XR_001041211.2	lncRNA
XM_012178848.2	CNOT8	mRNA	XR_001041211.2	lncRNA
XM_012178848.2	CNOT8	mRNA	XR_001041211.2	lncRNA
XM_012178848.2	CNOT8	mRNA	XR_001041211.2	lncRNA
XM_012178848.2	CNOT8	mRNA	XR_001041211.2	lncRNA
XM_012178848.2	CNOT8	mRNA	XR_001041211.2	lncRNA
XM_012178848.2	CNOT8	mRNA	XR_001041211.2	lncRNA
XM_012184446.2	RCBTB2	mRNA	XR_001041211.2	lncRNA
XM_012184446.2	RCBTB2	mRNA	XR_001041211.2	lncRNA
XM_012184446.2	RCBTB2	mRNA	XR_001041211.2	lncRNA
XM_012184446.2	RCBTB2	mRNA	XR_001041211.2	lncRNA
XM_012184446.2	RCBTB2	mRNA	XR_001041211.2	lncRNA
XM_012184446.2	RCBTB2	mRNA	XR_001041211.2	lncRNA
XM_012178847.2	CNOT8	mRNA	XR_001034847.2	lncRNA
XM_012178847.2	CNOT8	mRNA	XR_001034847.2	lncRNA
XM_012178847.2	CNOT8	mRNA	XR_001034847.2	lncRNA
XM_012178847.2	CNOT8	mRNA	XR_001034847.2	lncRNA
XM_012178847.2	CNOT8	mRNA	XR_001034847.2	lncRNA
XM_012178847.2	CNOT8	mRNA	XR_001034847.2	lncRNA
XM_012178847.2	CNOT8	mRNA	XR_001434202.1	lncRNA
XM_012178847.2	CNOT8	mRNA	XR_001434202.1	lncRNA

XM_012178847.2	CNOT8	mRNA	XR_001434202.1	lncRNA
XM_012178847.2	CNOT8	mRNA	XR_001434202.1	lncRNA
XM_012178847.2	CNOT8	mRNA	XR_001434202.1	lncRNA
XM_012178847.2	CNOT8	mRNA	XR_001434202.1	lncRNA
XM_015097132.1	TMEM229B	mRNA	XR_001436102.1	lncRNA
XM_015097132.1	TMEM229B	mRNA	XR_001436102.1	lncRNA
XM_015097132.1	TMEM229B	mRNA	XR_001436102.1	lncRNA
XM_015097132.1	TMEM229B	mRNA	XR_001436102.1	lncRNA
XM_015097132.1	TMEM229B	mRNA	XR_001436102.1	lncRNA
XM_015097132.1	TMEM229B	mRNA	XR_001436102.1	lncRNA
XM_015097132.1	TMEM229B	mRNA	XR_001436102.1	lncRNA
XM_004021989.3	DMD	mRNA	XR_001436817.1	lncRNA
XM_004021989.3	DMD	mRNA	XR_001436817.1	lncRNA
XM_004021989.3	DMD	mRNA	XR_001436817.1	lncRNA
XM_004021989.3	DMD	mRNA	XR_001436817.1	lncRNA
XM_004021989.3	DMD	mRNA	XR_001436817.1	lncRNA
XM_004021989.3	DMD	mRNA	XR_001436817.1	lncRNA
XM_004002018.3	OMA1	mRNA	XR_001436817.1	lncRNA
XM_004002018.3	OMA1	mRNA	XR_001436817.1	lncRNA
XM_004002018.3	OMA1	mRNA	XR_001436817.1	lncRNA
XM_004002018.3	OMA1	mRNA	XR_001436817.1	lncRNA
XM_004002018.3	OMA1	mRNA	XR_001436817.1	lncRNA
XM_004002018.3	OMA1	mRNA	XR_001436817.1	lncRNA
XM_012103465.2	OSBPL1A	mRNA	XR_001436817.1	lncRNA
XM_012103465.2	OSBPL1A	mRNA	XR_001436817.1	lncRNA
XM_012103465.2	OSBPL1A	mRNA	XR_001436817.1	lncRNA
XM_012103465.2	OSBPL1A	mRNA	XR_001436817.1	lncRNA
XM_012103465.2	OSBPL1A	mRNA	XR_001436817.1	lncRNA
XM_012103465.2	OSBPL1A	mRNA	XR_001436817.1	lncRNA
XM_012096298.1	TP53I11	mRNA	XR_001436817.1	lncRNA
XM_012096298.1	TP53I11	mRNA	XR_001436817.1	lncRNA
XM_012096298.1	TP53I11	mRNA	XR_001436817.1	lncRNA
XM_012096298.1	TP53I11	mRNA	XR_001436817.1	lncRNA
XM_012096298.1	TP53I11	mRNA	XR_001436817.1	lncRNA
XM_012096298.1	TP53I11	mRNA	XR_001436817.1	lncRNA
XM_015096552.1	MOB1B	mRNA	XR_001434203.1	lncRNA
XM_015096552.1	MOB1B	mRNA	XR_001434203.1	lncRNA
XM_015096552.1	MOB1B	mRNA	XR_001434203.1	lncRNA
XM_015096552.1	MOB1B	mRNA	XR_001434203.1	lncRNA
XM_015096552.1	MOB1B	mRNA	XR_001434203.1	lncRNA
XM_015096552.1	MOB1B	mRNA	XR_001434203.1	lncRNA
XM_004002018.3	OMA1	mRNA	XR_001434203.1	lncRNA
XM_004002018.3	OMA1	mRNA	XR_001434203.1	lncRNA
XM_004002018.3	OMA1	mRNA	XR_001434203.1	lncRNA
XM_004002018.3	OMA1	mRNA	XR_001434203.1	lncRNA
XM_004002018.3	OMA1	mRNA	XR_001434203.1	lncRNA
XM_004002018.3	OMA1	mRNA	XR_001434203.1	lncRNA
XM_012099693.2	EOGT	mRNA	XR_001436817.1	lncRNA
XM_012099693.2	EOGT	mRNA	XR_001436817.1	lncRNA
XM_012099693.2	EOGT	mRNA	XR_001436817.1	lncRNA
XM_012099693.2	EOGT	mRNA	XR_001436817.1	lncRNA
XM_012099693.2	EOGT	mRNA	XR_001436817.1	lncRNA
XM_012099693.2	EOGT	mRNA	XR_001436817.1	lncRNA
XM_012106834.1	OPHN1	mRNA	XR_001434203.1	lncRNA

XM_012106834.1	OPHN1	mRNA	XR_001434203.1	lncRNA
XM_012106834.1	OPHN1	mRNA	XR_001434203.1	lncRNA
XM_012106834.1	OPHN1	mRNA	XR_001434203.1	lncRNA
XM_012106834.1	OPHN1	mRNA	XR_001434203.1	lncRNA
XM_012106834.1	OPHN1	mRNA	XR_001434203.1	lncRNA
XM_015092341.1	USP21	mRNA	TCONS_00022613	lncRNA
XM_015092341.1	USP21	mRNA	TCONS_00022613	lncRNA
XM_015092341.1	USP21	mRNA	TCONS_00022613	lncRNA
XM_015092341.1	USP21	mRNA	TCONS_00022613	lncRNA
XM_015092341.1	USP21	mRNA	TCONS_00022613	lncRNA
XM_015092341.1	USP21	mRNA	TCONS_00022613	lncRNA
XM_015093216.1	TTC39B	mRNA	TCONS_00022613	lncRNA
XM_015093216.1	TTC39B	mRNA	TCONS_00022613	lncRNA
XM_015093216.1	TTC39B	mRNA	TCONS_00022613	lncRNA
XM_015093216.1	TTC39B	mRNA	TCONS_00022613	lncRNA
XM_015093216.1	TTC39B	mRNA	TCONS_00022613	lncRNA
XM_015093216.1	TTC39B	mRNA	TCONS_00022613	lncRNA
XM_004015967.3	DYNC2H1	mRNA	XR_001025491.1	lncRNA
XM_004015967.3	DYNC2H1	mRNA	XR_001025491.1	lncRNA
XM_004015967.3	DYNC2H1	mRNA	XR_001025491.1	lncRNA
XM_004015967.3	DYNC2H1	mRNA	XR_001025491.1	lncRNA
XM_004015967.3	DYNC2H1	mRNA	XR_001025491.1	lncRNA
XM_004015967.3	DYNC2H1	mRNA	XR_001025491.1	lncRNA
XM_012175276.1	PLCM1	mRNA	XR_001436127.1	lncRNA
XM_012175276.1	PLCM1	mRNA	XR_001436127.1	lncRNA
XM_012175276.1	PLCM1	mRNA	XR_001436127.1	lncRNA
XM_012175276.1	PLCM1	mRNA	XR_001436127.1	lncRNA
XM_012175276.1	PLCM1	mRNA	XR_001436127.1	lncRNA
XM_012175276.1	PLCM1	mRNA	XR_001436127.1	lncRNA
XM_012099341.2	CLASP2	mRNA	XR_001025491.1	lncRNA
XM_012099341.2	CLASP2	mRNA	XR_001025491.1	lncRNA
XM_012099341.2	CLASP2	mRNA	XR_001025491.1	lncRNA
XM_012099341.2	CLASP2	mRNA	XR_001025491.1	lncRNA
XM_012099341.2	CLASP2	mRNA	XR_001025491.1	lncRNA
XM_012099341.2	CLASP2	mRNA	XR_001025491.1	lncRNA
XM_012096480.2	STX3	mRNA	XR_001024290.1	lncRNA
XM_012096480.2	STX3	mRNA	XR_001024290.1	lncRNA
XM_012096480.2	STX3	mRNA	XR_001024290.1	lncRNA
XM_012096480.2	STX3	mRNA	XR_001024290.1	lncRNA
XM_012096480.2	STX3	mRNA	XR_001024290.1	lncRNA
XM_012096480.2	STX3	mRNA	XR_001024290.1	lncRNA
XM_004006510.3	SLC16A7	mRNA	XR_001020707.2	lncRNA
XM_004006510.3	SLC16A7	mRNA	XR_001020707.2	lncRNA
XM_004006510.3	SLC16A7	mRNA	XR_001020707.2	lncRNA
XM_004006510.3	SLC16A7	mRNA	XR_001020707.2	lncRNA
XM_004006510.3	SLC16A7	mRNA	XR_001020707.2	lncRNA
XM_004006510.3	SLC16A7	mRNA	XR_001020707.2	lncRNA
XM_015104846.1	TAB3	mRNA	XR_001033284.2	lncRNA
XM_015104846.1	TAB3	mRNA	XR_001033284.2	lncRNA
XM_015104846.1	TAB3	mRNA	XR_001033284.2	lncRNA
XM_015104846.1	TAB3	mRNA	XR_001033284.2	lncRNA
XM_015104846.1	TAB3	mRNA	XR_001033284.2	lncRNA
XM_015104846.1	TAB3	mRNA	XR_001033284.2	lncRNA
XM_012176217.2	EVI5	mRNA	TCONS_00075453	lncRNA

XM_012176217.2	EVI5	mRNA	TCONS_00075453	lncRNA
XM_012176217.2	EVI5	mRNA	TCONS_00075453	lncRNA
XM_012176217.2	EVI5	mRNA	TCONS_00075453	lncRNA
XM_012176217.2	EVI5	mRNA	TCONS_00075453	lncRNA
XM_012176217.2	EVI5	mRNA	TCONS_00075453	lncRNA
XM_004016880.3	FAM169A	mRNA	TCONS_00075051	lncRNA
XM_004016880.3	FAM169A	mRNA	TCONS_00075051	lncRNA
XM_004016880.3	FAM169A	mRNA	TCONS_00075051	lncRNA
XM_004016880.3	FAM169A	mRNA	TCONS_00075051	lncRNA
XM_004016880.3	FAM169A	mRNA	TCONS_00075051	lncRNA
XM_012105396.2	C25H10orf11	mRNA	TCONS_00084305	lncRNA
XM_012105396.2	C25H10orf11	mRNA	TCONS_00084305	lncRNA
XM_012105396.2	C25H10orf11	mRNA	TCONS_00084305	lncRNA
XM_012105396.2	C25H10orf11	mRNA	TCONS_00084305	lncRNA
XM_012105396.2	C25H10orf11	mRNA	TCONS_00084305	lncRNA
XM_012095771.2	CTR9	mRNA	XR_001434761.1	lncRNA
XM_012095771.2	CTR9	mRNA	XR_001434761.1	lncRNA
XM_012095771.2	CTR9	mRNA	XR_001434761.1	lncRNA
XM_012095771.2	CTR9	mRNA	XR_001434761.1	lncRNA
XM_012095771.2	CTR9	mRNA	XR_001434761.1	lncRNA
XM_012095771.2	CTR9	mRNA	XR_001434761.1	lncRNA
XM_004017713.3	LINS1	mRNA	XR_001434761.1	lncRNA
XM_004017713.3	LINS1	mRNA	XR_001434761.1	lncRNA
XM_004017713.3	LINS1	mRNA	XR_001434761.1	lncRNA
XM_004017713.3	LINS1	mRNA	XR_001434761.1	lncRNA
XM_004017713.3	LINS1	mRNA	XR_001434761.1	lncRNA
XM_004017713.3	LINS1	mRNA	XR_001434761.1	lncRNA
XM_012104594.2	ZNF789	mRNA	XR_001434761.1	lncRNA
XM_012104594.2	ZNF789	mRNA	XR_001434761.1	lncRNA
XM_012104594.2	ZNF789	mRNA	XR_001434761.1	lncRNA
XM_012104594.2	ZNF789	mRNA	XR_001434761.1	lncRNA
XM_012104594.2	ZNF789	mRNA	XR_001434761.1	lncRNA
XM_012104594.2	ZNF789	mRNA	XR_001434761.1	lncRNA
XM_012105688.2	WDR17	mRNA	XR_001433755.1	lncRNA
XM_012105688.2	WDR17	mRNA	XR_001433755.1	lncRNA
XM_012105688.2	WDR17	mRNA	XR_001433755.1	lncRNA
XM_012105688.2	WDR17	mRNA	XR_001433755.1	lncRNA
XM_012105688.2	WDR17	mRNA	XR_001433755.1	lncRNA
XM_012105688.2	WDR17	mRNA	XR_001433755.1	lncRNA
XM_012122817.1	RNF220	mRNA	TCONS_00078440	lncRNA
XM_012122817.1	RNF220	mRNA	TCONS_00078440	lncRNA
XM_012122817.1	RNF220	mRNA	TCONS_00078440	lncRNA
XM_012122817.1	RNF220	mRNA	TCONS_00078440	lncRNA
XM_012122817.1	RNF220	mRNA	TCONS_00078440	lncRNA
XM_012122817.1	RNF220	mRNA	TCONS_00078440	lncRNA
XM_015104327.1	LOC101115427	mRNA	XR_001021689.2	lncRNA
XM_015104327.1	LOC101115427	mRNA	XR_001021689.2	lncRNA
XM_015104327.1	LOC101115427	mRNA	XR_001021689.2	lncRNA
XM_015104327.1	LOC101115427	mRNA	XR_001021689.2	lncRNA
XM_015104327.1	LOC101115427	mRNA	XR_001021689.2	lncRNA
XM_015104327.1	LOC101115427	mRNA	XR_001021689.2	lncRNA
XM_012187417.2	PANK4	mRNA	XR_001021689.2	lncRNA
XM_012187417.2	PANK4	mRNA	XR_001021689.2	lncRNA
XM_012187417.2	PANK4	mRNA	XR_001021689.2	lncRNA

XM_012187417.2	PANK4	mRNA	XR_001021689.2	lncRNA
XM_012187417.2	PANK4	mRNA	XR_001021689.2	lncRNA
XM_012187417.2	PANK4	mRNA	XR_001021689.2	lncRNA
XM_004010212.3	MTX3	mRNA	XR_001021689.2	lncRNA
XM_004010212.3	MTX3	mRNA	XR_001021689.2	lncRNA
XM_004010212.3	MTX3	mRNA	XR_001021689.2	lncRNA
XM_004010212.3	MTX3	mRNA	XR_001021689.2	lncRNA
XM_004010212.3	MTX3	mRNA	XR_001021689.2	lncRNA
XM_004010212.3	MTX3	mRNA	XR_001021689.2	lncRNA
XM_012188265.2	CCDC7	mRNA	XR_001021689.2	lncRNA
XM_012188265.2	CCDC7	mRNA	XR_001021689.2	lncRNA
XM_012188265.2	CCDC7	mRNA	XR_001021689.2	lncRNA
XM_012188265.2	CCDC7	mRNA	XR_001021689.2	lncRNA
XM_012188265.2	CCDC7	mRNA	XR_001021689.2	lncRNA
XM_012188265.2	CCDC7	mRNA	XR_001021689.2	lncRNA
XM_015099971.1	ZNF181	mRNA	XR_001021689.2	lncRNA
XM_015099971.1	ZNF181	mRNA	XR_001021689.2	lncRNA
XM_015099971.1	ZNF181	mRNA	XR_001021689.2	lncRNA
XM_015099971.1	ZNF181	mRNA	XR_001021689.2	lncRNA
XM_015099971.1	ZNF181	mRNA	XR_001021689.2	lncRNA
XM_015099971.1	ZNF181	mRNA	XR_001021689.2	lncRNA
XM_012188276.2	CCDC7	mRNA	XR_001021689.2	lncRNA
XM_012188276.2	CCDC7	mRNA	XR_001021689.2	lncRNA
XM_012188276.2	CCDC7	mRNA	XR_001021689.2	lncRNA
XM_012188276.2	CCDC7	mRNA	XR_001021689.2	lncRNA
XM_012188276.2	CCDC7	mRNA	XR_001021689.2	lncRNA
XM_012188276.2	CCDC7	mRNA	XR_001021689.2	lncRNA
XM_012186029.1	FBXL20	mRNA	XR_001436102.1	lncRNA
XM_012186029.1	FBXL20	mRNA	XR_001436102.1	lncRNA
XM_012186029.1	FBXL20	mRNA	XR_001436102.1	lncRNA
XM_012186029.1	FBXL20	mRNA	XR_001436102.1	lncRNA
XM_012186029.1	FBXL20	mRNA	XR_001436102.1	lncRNA
XM_012186029.1	FBXL20	mRNA	XR_001436102.1	lncRNA
XM_004010212.3	MTX3	mRNA	XR_001436102.1	lncRNA
XM_004010212.3	MTX3	mRNA	XR_001436102.1	lncRNA
XM_004010212.3	MTX3	mRNA	XR_001436102.1	lncRNA
XM_004010212.3	MTX3	mRNA	XR_001436102.1	lncRNA
XM_004010212.3	MTX3	mRNA	XR_001436102.1	lncRNA
XM_004010212.3	MTX3	mRNA	XR_001436102.1	lncRNA
XM_012099283.2	NEK10	mRNA	XR_001436102.1	lncRNA
XM_012099283.2	NEK10	mRNA	XR_001436102.1	lncRNA
XM_012099283.2	NEK10	mRNA	XR_001436102.1	lncRNA
XM_012099283.2	NEK10	mRNA	XR_001436102.1	lncRNA
XM_012099283.2	NEK10	mRNA	XR_001436102.1	lncRNA
XM_012099283.2	NEK10	mRNA	XR_001436102.1	lncRNA
XM_015095425.1	CUL1	mRNA	XR_001436102.1	lncRNA
XM_015095425.1	CUL1	mRNA	XR_001436102.1	lncRNA
XM_015095425.1	CUL1	mRNA	XR_001436102.1	lncRNA
XM_015095425.1	CUL1	mRNA	XR_001436102.1	lncRNA
XM_015095425.1	CUL1	mRNA	XR_001436102.1	lncRNA
XM_015095425.1	CUL1	mRNA	XR_001436102.1	lncRNA
XM_012176680.2	ARHGAP29	mRNA	XR_001436102.1	lncRNA
XM_012176680.2	ARHGAP29	mRNA	XR_001436102.1	lncRNA
XM_012176680.2	ARHGAP29	mRNA	XR_001436102.1	lncRNA

XM_012176680.2	ARHGAP29	mRNA	XR_001436102.1	lncRNA
XM_012176680.2	ARHGAP29	mRNA	XR_001436102.1	lncRNA
XM_012176680.2	ARHGAP29	mRNA	XR_001436102.1	lncRNA
XM_012188276.2	CCDC7	mRNA	XR_001436102.1	lncRNA
XM_012188276.2	CCDC7	mRNA	XR_001436102.1	lncRNA
XM_012188276.2	CCDC7	mRNA	XR_001436102.1	lncRNA
XM_012188276.2	CCDC7	mRNA	XR_001436102.1	lncRNA
XM_012188276.2	CCDC7	mRNA	XR_001436102.1	lncRNA
XM_012188276.2	CCDC7	mRNA	XR_001436102.1	lncRNA
XM_012188265.2	CCDC7	mRNA	XR_001436102.1	lncRNA
XM_012188265.2	CCDC7	mRNA	XR_001436102.1	lncRNA
XM_012188265.2	CCDC7	mRNA	XR_001436102.1	lncRNA
XM_012188265.2	CCDC7	mRNA	XR_001436102.1	lncRNA
XM_012188265.2	CCDC7	mRNA	XR_001436102.1	lncRNA
XM_012188265.2	CCDC7	mRNA	XR_001436102.1	lncRNA
XM_012127663.2	IFT74	mRNA	XR_001021689.2	lncRNA
XM_012127663.2	IFT74	mRNA	XR_001021689.2	lncRNA
XM_012127663.2	IFT74	mRNA	XR_001021689.2	lncRNA
XM_012127663.2	IFT74	mRNA	XR_001021689.2	lncRNA
XM_012127663.2	IFT74	mRNA	XR_001021689.2	lncRNA
XM_012127663.2	IFT74	mRNA	XR_001021689.2	lncRNA
XM_012094704.2	LOC10560198	mRNA	XR_001021689.2	lncRNA
XM_012094704.2	LOC10560198	mRNA	XR_001021689.2	lncRNA
XM_012094704.2	LOC10560198	mRNA	XR_001021689.2	lncRNA
XM_012094704.2	LOC10560198	mRNA	XR_001021689.2	lncRNA
XM_012094704.2	LOC10560198	mRNA	XR_001021689.2	lncRNA
XM_012094704.2	LOC10560198	mRNA	XR_001021689.2	lncRNA
XM_012105261.2	MCU	mRNA	XR_001021689.2	lncRNA
XM_012105261.2	MCU	mRNA	XR_001021689.2	lncRNA
XM_012105261.2	MCU	mRNA	XR_001021689.2	lncRNA
XM_012105261.2	MCU	mRNA	XR_001021689.2	lncRNA
XM_012105261.2	MCU	mRNA	XR_001021689.2	lncRNA
XM_012105261.2	MCU	mRNA	XR_001021689.2	lncRNA
XM_012149251.2	S100PBP	mRNA	XR_001436102.1	lncRNA
XM_012149251.2	S100PBP	mRNA	XR_001436102.1	lncRNA
XM_012149251.2	S100PBP	mRNA	XR_001436102.1	lncRNA
XM_012149251.2	S100PBP	mRNA	XR_001436102.1	lncRNA
XM_012149251.2	S100PBP	mRNA	XR_001436102.1	lncRNA
XM_012149251.2	S100PBP	mRNA	XR_001436102.1	lncRNA
XM_004011402.3	STXBP5	mRNA	TCONS_00022613	lncRNA
XM_004011402.3	STXBP5	mRNA	TCONS_00022613	lncRNA
XM_004011402.3	STXBP5	mRNA	TCONS_00022613	lncRNA
XM_004011402.3	STXBP5	mRNA	TCONS_00022613	lncRNA
XM_004011402.3	STXBP5	mRNA	TCONS_00022613	lncRNA
XM_004011402.3	STXBP5	mRNA	TCONS_00022613	lncRNA
XM_015103475.1	PLEKHA1	mRNA	TCONS_00022613	lncRNA
XM_015103475.1	PLEKHA1	mRNA	TCONS_00022613	lncRNA
XM_015103475.1	PLEKHA1	mRNA	TCONS_00022613	lncRNA
XM_015103475.1	PLEKHA1	mRNA	TCONS_00022613	lncRNA
XM_015103475.1	PLEKHA1	mRNA	TCONS_00022613	lncRNA
XM_015103475.1	PLEKHA1	mRNA	TCONS_00022613	lncRNA
XM_015095875.1	SAFB	mRNA	XR_001024743.2	lncRNA
XM_015095875.1	SAFB	mRNA	XR_001024743.2	lncRNA
XM_015095875.1	SAFB	mRNA	XR_001024743.2	lncRNA

XM_015095875.1	SAFB	mRNA	XR_001024743.2	lncRNA
XM_015095875.1	SAFB	mRNA	XR_001024743.2	lncRNA
XM_015104471.1	CCSER2	mRNA	XR_001024743.2	lncRNA
XM_015104471.1	CCSER2	mRNA	XR_001024743.2	lncRNA
XM_015104471.1	CCSER2	mRNA	XR_001024743.2	lncRNA
XM_015104471.1	CCSER2	mRNA	XR_001024743.2	lncRNA
XM_015104471.1	CCSER2	mRNA	XR_001024743.2	lncRNA
XM_015103641.1	YES1	mRNA	XR_001024743.2	lncRNA
XM_015103641.1	YES1	mRNA	XR_001024743.2	lncRNA
XM_015103641.1	YES1	mRNA	XR_001024743.2	lncRNA
XM_015103641.1	YES1	mRNA	XR_001024743.2	lncRNA
XM_015103641.1	YES1	mRNA	XR_001024743.2	lncRNA
XM_015103466.1	TACC2	mRNA	TCONS_00078440	lncRNA
XM_015103466.1	TACC2	mRNA	TCONS_00078440	lncRNA
XM_015103466.1	TACC2	mRNA	TCONS_00078440	lncRNA
XM_015103466.1	TACC2	mRNA	TCONS_00078440	lncRNA
XM_015103466.1	TACC2	mRNA	TCONS_00078440	lncRNA
XM_015103466.1	TACC2	mRNA	TCONS_00078440	lncRNA
XM_015095024.1	JOSD1	mRNA	XR_001025491.1	lncRNA
XM_015095024.1	JOSD1	mRNA	XR_001025491.1	lncRNA
XM_015095024.1	JOSD1	mRNA	XR_001025491.1	lncRNA
XM_015095024.1	JOSD1	mRNA	XR_001025491.1	lncRNA
XM_015095024.1	JOSD1	mRNA	XR_001025491.1	lncRNA
XM_015095024.1	JOSD1	mRNA	XR_001025491.1	lncRNA
XM_015095024.1	JOSD1	mRNA	XR_001024290.1	lncRNA
XM_015095024.1	JOSD1	mRNA	XR_001024290.1	lncRNA
XM_015095024.1	JOSD1	mRNA	XR_001024290.1	lncRNA
XM_015095024.1	JOSD1	mRNA	XR_001024290.1	lncRNA
XM_015095024.1	JOSD1	mRNA	XR_001024290.1	lncRNA
XM_015095024.1	JOSD1	mRNA	XR_001024290.1	lncRNA
XM_012131583.2	FAM171B	mRNA	XR_001044126.1	lncRNA
XM_012131583.2	FAM171B	mRNA	XR_001044126.1	lncRNA
XM_012131583.2	FAM171B	mRNA	XR_001044126.1	lncRNA
XM_012131583.2	FAM171B	mRNA	XR_001044126.1	lncRNA
XM_012131583.2	FAM171B	mRNA	XR_001044126.1	lncRNA
XM_012131583.2	FAM171B	mRNA	XR_001044126.1	lncRNA
NM_001308600.1	KRT17	mRNA	XR_001024290.1	lncRNA
NM_001308600.1	KRT17	mRNA	XR_001024290.1	lncRNA
NM_001308600.1	KRT17	mRNA	XR_001024290.1	lncRNA
NM_001308600.1	KRT17	mRNA	XR_001024290.1	lncRNA
NM_001308600.1	KRT17	mRNA	XR_001024290.1	lncRNA
NM_001308600.1	KRT17	mRNA	XR_001024290.1	lncRNA
XM_004009100.3	PAM	mRNA	XR_001044126.1	lncRNA
XM_004009100.3	PAM	mRNA	XR_001044126.1	lncRNA
XM_004009100.3	PAM	mRNA	XR_001044126.1	lncRNA
XM_004009100.3	PAM	mRNA	XR_001044126.1	lncRNA
XM_004009100.3	PAM	mRNA	XR_001044126.1	lncRNA
XM_004009100.3	PAM	mRNA	XR_001044126.1	lncRNA
XM_012134956.1	STK39	mRNA	XR_001025491.1	lncRNA
XM_012134956.1	STK39	mRNA	XR_001025491.1	lncRNA
XM_012134956.1	STK39	mRNA	XR_001025491.1	lncRNA
XM_012134956.1	STK39	mRNA	XR_001025491.1	lncRNA
XM_012134956.1	STK39	mRNA	XR_001025491.1	lncRNA
XM_012134956.1	STK39	mRNA	XR_001025491.1	lncRNA

XM_015097748.1	PCMTD1	mRNA	TCONS_00075453	lncRNA
XM_015097748.1	PCMTD1	mRNA	TCONS_00075453	lncRNA
XM_015097748.1	PCMTD1	mRNA	TCONS_00075453	lncRNA
XM_015097748.1	PCMTD1	mRNA	TCONS_00075453	lncRNA
XM_015097748.1	PCMTD1	mRNA	TCONS_00075453	lncRNA
XM_015097748.1	PCMTD1	mRNA	TCONS_00075453	lncRNA
XM_012151052.2	WASF2	mRNA	TCONS_00075453	lncRNA
XM_012151052.2	WASF2	mRNA	TCONS_00075453	lncRNA
XM_012151052.2	WASF2	mRNA	TCONS_00075453	lncRNA
XM_012151052.2	WASF2	mRNA	TCONS_00075453	lncRNA
XM_012151052.2	WASF2	mRNA	TCONS_00075453	lncRNA
XM_012151052.2	WASF2	mRNA	TCONS_00075453	lncRNA
XM_015094761.1	SLC35E3	mRNA	XR_001033284.2	lncRNA
XM_015094761.1	SLC35E3	mRNA	XR_001033284.2	lncRNA
XM_015094761.1	SLC35E3	mRNA	XR_001033284.2	lncRNA
XM_015094761.1	SLC35E3	mRNA	XR_001033284.2	lncRNA
XM_015094761.1	SLC35E3	mRNA	XR_001033284.2	lncRNA
XM_012184607.2	BRCA2	mRNA	TCONS_00075051	lncRNA
XM_012184607.2	BRCA2	mRNA	TCONS_00075051	lncRNA
XM_012184607.2	BRCA2	mRNA	TCONS_00075051	lncRNA
XM_012184607.2	BRCA2	mRNA	TCONS_00075051	lncRNA
XM_012184607.2	BRCA2	mRNA	TCONS_00075051	lncRNA
XM_015102039.1	SYNE3	mRNA	TCONS_00054422	lncRNA
XM_015102039.1	SYNE3	mRNA	TCONS_00054422	lncRNA
XM_015102039.1	SYNE3	mRNA	TCONS_00054422	lncRNA
XM_015102039.1	SYNE3	mRNA	TCONS_00054422	lncRNA
XM_015102039.1	SYNE3	mRNA	TCONS_00054422	lncRNA
XM_012185217.2	CLTC	mRNA	XR_001033284.2	lncRNA
XM_012185217.2	CLTC	mRNA	XR_001033284.2	lncRNA
XM_012185217.2	CLTC	mRNA	XR_001033284.2	lncRNA
XM_012185217.2	CLTC	mRNA	XR_001033284.2	lncRNA
XM_012185217.2	CLTC	mRNA	XR_001033284.2	lncRNA
XM_012185217.2	CLTC	mRNA	XR_001033284.2	lncRNA
XM_012095858.2	ZNF214	mRNA	XR_001033284.2	lncRNA
XM_012095858.2	ZNF214	mRNA	XR_001033284.2	lncRNA
XM_012095858.2	ZNF214	mRNA	XR_001033284.2	lncRNA
XM_012095858.2	ZNF214	mRNA	XR_001033284.2	lncRNA
XM_012095858.2	ZNF214	mRNA	XR_001033284.2	lncRNA
XM_012095858.2	ZNF214	mRNA	XR_001033284.2	lncRNA
XM_012102834.2	PPRC1	mRNA	XR_001020707.2	lncRNA
XM_012102834.2	PPRC1	mRNA	XR_001020707.2	lncRNA
XM_012102834.2	PPRC1	mRNA	XR_001020707.2	lncRNA
XM_012102834.2	PPRC1	mRNA	XR_001020707.2	lncRNA
XM_012102834.2	PPRC1	mRNA	XR_001020707.2	lncRNA
XM_012102834.2	PPRC1	mRNA	XR_001020707.2	lncRNA
XM_012168955.2	DNAJC6	mRNA	XR_001020707.2	lncRNA
XM_012168955.2	DNAJC6	mRNA	XR_001020707.2	lncRNA
XM_012168955.2	DNAJC6	mRNA	XR_001020707.2	lncRNA
XM_012168955.2	DNAJC6	mRNA	XR_001020707.2	lncRNA
XM_012168955.2	DNAJC6	mRNA	XR_001020707.2	lncRNA
XM_012168955.2	DNAJC6	mRNA	XR_001020707.2	lncRNA
XM_015095425.1	CUL1	mRNA	XR_001434202.1	lncRNA
XM_015095425.1	CUL1	mRNA	XR_001434202.1	lncRNA
XM_015095425.1	CUL1	mRNA	XR_001434202.1	lncRNA

XM_015095425.1	CUL1	mRNA	XR_001434202.1	lncRNA
XM_015095425.1	CUL1	mRNA	XR_001434202.1	lncRNA
XM_015095425.1	CUL1	mRNA	XR_001434202.1	lncRNA
XM_004010212.3	MTX3	mRNA	XR_001034847.2	lncRNA
XM_004010212.3	MTX3	mRNA	XR_001034847.2	lncRNA
XM_004010212.3	MTX3	mRNA	XR_001034847.2	lncRNA
XM_004010212.3	MTX3	mRNA	XR_001034847.2	lncRNA
XM_004010212.3	MTX3	mRNA	XR_001034847.2	lncRNA
XM_004010212.3	MTX3	mRNA	XR_001034847.2	lncRNA
XM_012187417.2	PANK4	mRNA	XR_001034847.2	lncRNA
XM_012187417.2	PANK4	mRNA	XR_001034847.2	lncRNA
XM_012187417.2	PANK4	mRNA	XR_001034847.2	lncRNA
XM_012187417.2	PANK4	mRNA	XR_001034847.2	lncRNA
XM_012187417.2	PANK4	mRNA	XR_001034847.2	lncRNA
XM_012187417.2	PANK4	mRNA	XR_001034847.2	lncRNA
XM_015095425.1	CUL1	mRNA	XR_001034847.2	lncRNA
XM_015095425.1	CUL1	mRNA	XR_001034847.2	lncRNA
XM_015095425.1	CUL1	mRNA	XR_001034847.2	lncRNA
XM_015095425.1	CUL1	mRNA	XR_001034847.2	lncRNA
XM_015095425.1	CUL1	mRNA	XR_001034847.2	lncRNA
XM_015095425.1	CUL1	mRNA	XR_001034847.2	lncRNA
XM_012187417.2	PANK4	mRNA	XR_001434202.1	lncRNA
XM_012187417.2	PANK4	mRNA	XR_001434202.1	lncRNA
XM_012187417.2	PANK4	mRNA	XR_001434202.1	lncRNA
XM_012187417.2	PANK4	mRNA	XR_001434202.1	lncRNA
XM_012187417.2	PANK4	mRNA	XR_001434202.1	lncRNA
XM_012187417.2	PANK4	mRNA	XR_001434202.1	lncRNA
XM_004010212.3	MTX3	mRNA	XR_001434202.1	lncRNA
XM_004010212.3	MTX3	mRNA	XR_001434202.1	lncRNA
XM_004010212.3	MTX3	mRNA	XR_001434202.1	lncRNA
XM_004010212.3	MTX3	mRNA	XR_001434202.1	lncRNA
XM_004010212.3	MTX3	mRNA	XR_001434202.1	lncRNA
XM_004010212.3	MTX3	mRNA	XR_001434202.1	lncRNA
XM_012178788.2	HMGXB3	mRNA	TCONS_00022613	lncRNA
XM_012178788.2	HMGXB3	mRNA	TCONS_00022613	lncRNA
XM_012178788.2	HMGXB3	mRNA	TCONS_00022613	lncRNA
XM_012178788.2	HMGXB3	mRNA	TCONS_00022613	lncRNA
XM_012178788.2	HMGXB3	mRNA	TCONS_00022613	lncRNA
XM_012178788.2	HMGXB3	mRNA	TCONS_00022613	lncRNA
XM_012097219.1	AGO3	mRNA	TCONS_00022613	lncRNA
XM_012097219.1	AGO3	mRNA	TCONS_00022613	lncRNA
XM_012097219.1	AGO3	mRNA	TCONS_00022613	lncRNA
XM_012097219.1	AGO3	mRNA	TCONS_00022613	lncRNA
XM_012097219.1	AGO3	mRNA	TCONS_00022613	lncRNA
XM_012097219.1	AGO3	mRNA	TCONS_00022613	lncRNA
XM_015093666.1	PIGV	mRNA	XR_001042381.2	lncRNA
XM_015093666.1	PIGV	mRNA	XR_001042381.2	lncRNA
XM_015093666.1	PIGV	mRNA	XR_001042381.2	lncRNA
XM_015093666.1	PIGV	mRNA	XR_001042381.2	lncRNA
XM_015093666.1	PIGV	mRNA	XR_001042381.2	lncRNA
XM_015093666.1	PIGV	mRNA	XR_001042381.2	lncRNA
XM_012176451.2	AHR	mRNA	XR_001042381.2	lncRNA
XM_012176451.2	AHR	mRNA	XR_001042381.2	lncRNA
XM_012176451.2	AHR	mRNA	XR_001042381.2	lncRNA

XM_012176451.2	AHR	mRNA	XR_001042381.2	lncRNA
XM_012176451.2	AHR	mRNA	XR_001042381.2	lncRNA
XM_012176451.2	AHR	mRNA	XR_001042381.2	lncRNA
XM_012099798.2	KCTL3	mRNA	XR_001436532.1	lncRNA
XM_012099798.2	KCTL3	mRNA	XR_001436532.1	lncRNA
XM_012099798.2	KCTL3	mRNA	XR_001436532.1	lncRNA
XM_012099798.2	KCTL3	mRNA	XR_001436532.1	lncRNA
XM_012099798.2	KCTL3	mRNA	XR_001436532.1	lncRNA
XM_012099798.2	KCTL3	mRNA	XR_001436532.1	lncRNA
XM_012179693.1	PIGY	mRNA	XR_001042381.2	lncRNA
XM_012179693.1	PIGY	mRNA	XR_001042381.2	lncRNA
XM_012179693.1	PIGY	mRNA	XR_001042381.2	lncRNA
XM_012179693.1	PIGY	mRNA	XR_001042381.2	lncRNA
XM_012179693.1	PIGY	mRNA	XR_001042381.2	lncRNA
XM_012179693.1	PIGY	mRNA	XR_001042381.2	lncRNA
XM_015094531.1	ANAPC1	mRNA	TCONS_00078440	lncRNA
XM_015094531.1	ANAPC1	mRNA	TCONS_00078440	lncRNA
XM_015094531.1	ANAPC1	mRNA	TCONS_00078440	lncRNA
XM_015094531.1	ANAPC1	mRNA	TCONS_00078440	lncRNA
XM_015094531.1	ANAPC1	mRNA	TCONS_00078440	lncRNA
XM_015094531.1	ANAPC1	mRNA	TCONS_00078440	lncRNA
XM_012180475.2	CIART	mRNA	XR_001024290.1	lncRNA
XM_012180475.2	CIART	mRNA	XR_001024290.1	lncRNA
XM_012180475.2	CIART	mRNA	XR_001024290.1	lncRNA
XM_012180475.2	CIART	mRNA	XR_001024290.1	lncRNA
XM_012180475.2	CIART	mRNA	XR_001024290.1	lncRNA
XM_015093911.1	LOC10110405	mRNA	XR_001024290.1	lncRNA
XM_015093911.1	LOC10110405	mRNA	XR_001024290.1	lncRNA
XM_015093911.1	LOC10110405	mRNA	XR_001024290.1	lncRNA
XM_015093911.1	LOC10110405	mRNA	XR_001024290.1	lncRNA
XM_015093911.1	LOC10110405	mRNA	XR_001024290.1	lncRNA
XM_012182428.1	C1H1orf43	mRNA	XR_001024290.1	lncRNA
XM_012182428.1	C1H1orf43	mRNA	XR_001024290.1	lncRNA
XM_012182428.1	C1H1orf43	mRNA	XR_001024290.1	lncRNA
XM_012182428.1	C1H1orf43	mRNA	XR_001024290.1	lncRNA
XM_012182428.1	C1H1orf43	mRNA	XR_001024290.1	lncRNA
XM_012179768.2	RBPJ	mRNA	TCONS_00075051	lncRNA
XM_012179768.2	RBPJ	mRNA	TCONS_00075051	lncRNA
XM_012179768.2	RBPJ	mRNA	TCONS_00075051	lncRNA
XM_012179768.2	RBPJ	mRNA	TCONS_00075051	lncRNA
XM_012179768.2	RBPJ	mRNA	TCONS_00075051	lncRNA
XM_015092421.1	BBX	mRNA	XR_001436931.1	lncRNA
XM_015092421.1	BBX	mRNA	XR_001436931.1	lncRNA
XM_015092421.1	BBX	mRNA	XR_001436931.1	lncRNA
XM_015092421.1	BBX	mRNA	XR_001436931.1	lncRNA
XM_015092421.1	BBX	mRNA	XR_001436931.1	lncRNA
XM_012112310.2	C2H9orf91	mRNA	XR_001436931.1	lncRNA
XM_012112310.2	C2H9orf91	mRNA	XR_001436931.1	lncRNA
XM_012112310.2	C2H9orf91	mRNA	XR_001436931.1	lncRNA
XM_012112310.2	C2H9orf91	mRNA	XR_001436931.1	lncRNA
XM_012112310.2	C2H9orf91	mRNA	XR_001436931.1	lncRNA
XM_012188768.2	UBOX5	mRNA	XR_001436931.1	lncRNA
XM_012188768.2	UBOX5	mRNA	XR_001436931.1	lncRNA
XM_012188768.2	UBOX5	mRNA	XR_001436931.1	lncRNA

XM_012188768.2	UBOX5	mRNA	XR_001436931.1	lncRNA
XM_012188768.2	UBOX5	mRNA	XR_001436931.1	lncRNA
XM_012104597.2	ZKSCAN5	mRNA	TCONS_00075051	lncRNA
XM_012104597.2	ZKSCAN5	mRNA	TCONS_00075051	lncRNA
XM_012104597.2	ZKSCAN5	mRNA	TCONS_00075051	lncRNA
XM_012104597.2	ZKSCAN5	mRNA	TCONS_00075051	lncRNA
XM_012104597.2	ZKSCAN5	mRNA	TCONS_00075051	lncRNA
XM_015092421.1	BBX	mRNA	TCONS_00075051	lncRNA
XM_015092421.1	BBX	mRNA	TCONS_00075051	lncRNA
XM_015092421.1	BBX	mRNA	TCONS_00075051	lncRNA
XM_015092421.1	BBX	mRNA	TCONS_00075051	lncRNA
XM_015092421.1	BBX	mRNA	TCONS_00075051	lncRNA
XM_012100212.2	ATG7	mRNA	TCONS_00075453	lncRNA
XM_012100212.2	ATG7	mRNA	TCONS_00075453	lncRNA
XM_012100212.2	ATG7	mRNA	TCONS_00075453	lncRNA
XM_012100212.2	ATG7	mRNA	TCONS_00075453	lncRNA
XM_012100212.2	ATG7	mRNA	TCONS_00075453	lncRNA
XM_012100212.2	ATG7	mRNA	TCONS_00075453	lncRNA
XM_012101765.1	PRMT3	mRNA	XR_001436102.1	lncRNA
XM_012101765.1	PRMT3	mRNA	XR_001436102.1	lncRNA
XM_012101765.1	PRMT3	mRNA	XR_001436102.1	lncRNA
XM_012101765.1	PRMT3	mRNA	XR_001436102.1	lncRNA
XM_012101765.1	PRMT3	mRNA	XR_001436102.1	lncRNA
XM_012101765.1	PRMT3	mRNA	XR_001436102.1	lncRNA
XM_012118246.2	DCAF12	mRNA	XR_001436102.1	lncRNA
XM_012118246.2	DCAF12	mRNA	XR_001436102.1	lncRNA
XM_012118246.2	DCAF12	mRNA	XR_001436102.1	lncRNA
XM_012118246.2	DCAF12	mRNA	XR_001436102.1	lncRNA
XM_012118246.2	DCAF12	mRNA	XR_001436102.1	lncRNA
XM_012118246.2	DCAF12	mRNA	XR_001436102.1	lncRNA
XM_015097748.1	PCMTD1	mRNA	XR_001033284.2	lncRNA
XM_015097748.1	PCMTD1	mRNA	XR_001033284.2	lncRNA
XM_015097748.1	PCMTD1	mRNA	XR_001033284.2	lncRNA
XM_015097748.1	PCMTD1	mRNA	XR_001033284.2	lncRNA
XM_015097748.1	PCMTD1	mRNA	XR_001033284.2	lncRNA
XM_015097748.1	PCMTD1	mRNA	XR_001033284.2	lncRNA
XM_012098709.2	PML	mRNA	XR_001033284.2	lncRNA
XM_012098709.2	PML	mRNA	XR_001033284.2	lncRNA
XM_012098709.2	PML	mRNA	XR_001033284.2	lncRNA
XM_012098709.2	PML	mRNA	XR_001033284.2	lncRNA
XM_012098709.2	PML	mRNA	XR_001033284.2	lncRNA
XM_012098709.2	PML	mRNA	XR_001033284.2	lncRNA
XM_015105040.1	THOC2	mRNA	XR_001044126.1	lncRNA
XM_015105040.1	THOC2	mRNA	XR_001044126.1	lncRNA
XM_015105040.1	THOC2	mRNA	XR_001044126.1	lncRNA
XM_015105040.1	THOC2	mRNA	XR_001044126.1	lncRNA
XM_015105040.1	THOC2	mRNA	XR_001044126.1	lncRNA
XM_015105040.1	THOC2	mRNA	XR_001044126.1	lncRNA
XM_004008647.3	RAPGEF6	mRNA	XR_001044126.1	lncRNA
XM_004008647.3	RAPGEF6	mRNA	XR_001044126.1	lncRNA
XM_004008647.3	RAPGEF6	mRNA	XR_001044126.1	lncRNA
XM_004008647.3	RAPGEF6	mRNA	XR_001044126.1	lncRNA
XM_004008647.3	RAPGEF6	mRNA	XR_001044126.1	lncRNA
XM_004008647.3	RAPGEF6	mRNA	XR_001044126.1	lncRNA

XM_004016103.2	USP2	mRNA	XR_001024290.1	lncRNA
XM_004016103.2	USP2	mRNA	XR_001024290.1	lncRNA
XM_004016103.2	USP2	mRNA	XR_001024290.1	lncRNA
XM_004016103.2	USP2	mRNA	XR_001024290.1	lncRNA
XM_004016103.2	USP2	mRNA	XR_001024290.1	lncRNA
XM_004016103.2	USP2	mRNA	XR_001024290.1	lncRNA
XM_015101001.1	RRAS2	mRNA	XR_001044126.1	lncRNA
XM_015101001.1	RRAS2	mRNA	XR_001044126.1	lncRNA
XM_015101001.1	RRAS2	mRNA	XR_001044126.1	lncRNA
XM_015101001.1	RRAS2	mRNA	XR_001044126.1	lncRNA
XM_015101001.1	RRAS2	mRNA	XR_001044126.1	lncRNA
XM_015101001.1	RRAS2	mRNA	XR_001044126.1	lncRNA
XM_015092883.1	PADI1	mRNA	XR_001044126.1	lncRNA
XM_015092883.1	PADI1	mRNA	XR_001044126.1	lncRNA
XM_015092883.1	PADI1	mRNA	XR_001044126.1	lncRNA
XM_015092883.1	PADI1	mRNA	XR_001044126.1	lncRNA
XM_015092883.1	PADI1	mRNA	XR_001044126.1	lncRNA
XM_015092883.1	PADI1	mRNA	XR_001044126.1	lncRNA
XM_015102039.1	SYNE3	mRNA	XR_001436127.1	lncRNA
XM_015102039.1	SYNE3	mRNA	XR_001436127.1	lncRNA
XM_015102039.1	SYNE3	mRNA	XR_001436127.1	lncRNA
XM_015102039.1	SYNE3	mRNA	XR_001436127.1	lncRNA
XM_015102039.1	SYNE3	mRNA	XR_001436127.1	lncRNA
XM_015102039.1	SYNE3	mRNA	XR_001436127.1	lncRNA
XM_012096185.2	TCP11L1	mRNA	XR_001436127.1	lncRNA
XM_012096185.2	TCP11L1	mRNA	XR_001436127.1	lncRNA
XM_012096185.2	TCP11L1	mRNA	XR_001436127.1	lncRNA
XM_012096185.2	TCP11L1	mRNA	XR_001436127.1	lncRNA
XM_012096185.2	TCP11L1	mRNA	XR_001436127.1	lncRNA
XM_012096185.2	TCP11L1	mRNA	XR_001436127.1	lncRNA
XM_012176217.2	EVI5	mRNA	XR_001044126.1	lncRNA
XM_012176217.2	EVI5	mRNA	XR_001044126.1	lncRNA
XM_012176217.2	EVI5	mRNA	XR_001044126.1	lncRNA
XM_012176217.2	EVI5	mRNA	XR_001044126.1	lncRNA
XM_012176217.2	EVI5	mRNA	XR_001044126.1	lncRNA
XM_012176217.2	EVI5	mRNA	XR_001044126.1	lncRNA
XM_012176680.2	ARHGAP29	mRNA	XR_001434761.1	lncRNA
XM_012176680.2	ARHGAP29	mRNA	XR_001434761.1	lncRNA
XM_012176680.2	ARHGAP29	mRNA	XR_001434761.1	lncRNA
XM_012176680.2	ARHGAP29	mRNA	XR_001434761.1	lncRNA
XM_012176680.2	ARHGAP29	mRNA	XR_001434761.1	lncRNA
XM_012176680.2	ARHGAP29	mRNA	XR_001434761.1	lncRNA
XM_012170864.2	PAPOLG	mRNA	XR_001434761.1	lncRNA
XM_012170864.2	PAPOLG	mRNA	XR_001434761.1	lncRNA
XM_012170864.2	PAPOLG	mRNA	XR_001434761.1	lncRNA
XM_012170864.2	PAPOLG	mRNA	XR_001434761.1	lncRNA
XM_012170864.2	PAPOLG	mRNA	XR_001434761.1	lncRNA
XM_012170864.2	PAPOLG	mRNA	XR_001434761.1	lncRNA
XM_012190370.2	FAM98C	mRNA	XR_001434892.1	lncRNA
XM_012190370.2	FAM98C	mRNA	XR_001434892.1	lncRNA
XM_012190370.2	FAM98C	mRNA	XR_001434892.1	lncRNA
XM_012190370.2	FAM98C	mRNA	XR_001434892.1	lncRNA
XM_012190370.2	FAM98C	mRNA	XR_001434892.1	lncRNA
XM_012190370.2	FAM98C	mRNA	XR_001434892.1	lncRNA

XM_012176451.2	AHR	mRNA	XR_001436817.1	lncRNA
XM_012176451.2	AHR	mRNA	XR_001436817.1	lncRNA
XM_012176451.2	AHR	mRNA	XR_001436817.1	lncRNA
XM_012176451.2	AHR	mRNA	XR_001436817.1	lncRNA
XM_012176451.2	AHR	mRNA	XR_001436817.1	lncRNA
XM_012176451.2	AHR	mRNA	XR_001436817.1	lncRNA
XM_012188283.2	CCDC7	mRNA	XR_001436817.1	lncRNA
XM_012188283.2	CCDC7	mRNA	XR_001436817.1	lncRNA
XM_012188283.2	CCDC7	mRNA	XR_001436817.1	lncRNA
XM_012188283.2	CCDC7	mRNA	XR_001436817.1	lncRNA
XM_012188283.2	CCDC7	mRNA	XR_001436817.1	lncRNA
XM_012188283.2	CCDC7	mRNA	XR_001436817.1	lncRNA
XM_015093666.1	PIGV	mRNA	XR_001436817.1	lncRNA
XM_015093666.1	PIGV	mRNA	XR_001436817.1	lncRNA
XM_015093666.1	PIGV	mRNA	XR_001436817.1	lncRNA
XM_015093666.1	PIGV	mRNA	XR_001436817.1	lncRNA
XM_015093666.1	PIGV	mRNA	XR_001436817.1	lncRNA
XM_015093666.1	PIGV	mRNA	XR_001436817.1	lncRNA
XM_012176510.2	FNBP1L	mRNA	TCONS_00084305	lncRNA
XM_012176510.2	FNBP1L	mRNA	TCONS_00084305	lncRNA
XM_012176510.2	FNBP1L	mRNA	TCONS_00084305	lncRNA
XM_012176510.2	FNBP1L	mRNA	TCONS_00084305	lncRNA
XM_012176510.2	FNBP1L	mRNA	TCONS_00084305	lncRNA
XM_012176510.2	FNBP1L	mRNA	TCONS_00078442	lncRNA
XM_012176510.2	FNBP1L	mRNA	TCONS_00078442	lncRNA
XM_012176510.2	FNBP1L	mRNA	TCONS_00078442	lncRNA
XM_012176510.2	FNBP1L	mRNA	TCONS_00078442	lncRNA
XM_012176510.2	FNBP1L	mRNA	TCONS_00078442	lncRNA
XM_012182538.2	TRAPPC3L	mRNA	TCONS_00075453	lncRNA
XM_012182538.2	TRAPPC3L	mRNA	TCONS_00075453	lncRNA
XM_012182538.2	TRAPPC3L	mRNA	TCONS_00075453	lncRNA
XM_012182538.2	TRAPPC3L	mRNA	TCONS_00075453	lncRNA
XM_012182538.2	TRAPPC3L	mRNA	TCONS_00075453	lncRNA
XM_012182538.2	TRAPPC3L	mRNA	TCONS_00075453	lncRNA
XM_012107472.1	NFYC	mRNA	XR_001024743.2	lncRNA
XM_012107472.1	NFYC	mRNA	XR_001024743.2	lncRNA
XM_012107472.1	NFYC	mRNA	XR_001024743.2	lncRNA
XM_012107472.1	NFYC	mRNA	XR_001024743.2	lncRNA
XM_012107472.1	NFYC	mRNA	XR_001024743.2	lncRNA
XM_012181456.2	ADAM10	mRNA	TCONS_00022613	lncRNA
XM_012181456.2	ADAM10	mRNA	TCONS_00022613	lncRNA
XM_012181456.2	ADAM10	mRNA	TCONS_00022613	lncRNA
XM_012181456.2	ADAM10	mRNA	TCONS_00022613	lncRNA
XM_012181456.2	ADAM10	mRNA	TCONS_00022613	lncRNA
XM_012181456.2	ADAM10	mRNA	TCONS_00022613	lncRNA
XM_012143416.2	NRP2	mRNA	XR_001033284.2	lncRNA
XM_012143416.2	NRP2	mRNA	XR_001033284.2	lncRNA
XM_012143416.2	NRP2	mRNA	XR_001033284.2	lncRNA
XM_012143416.2	NRP2	mRNA	XR_001033284.2	lncRNA
XM_012143416.2	NRP2	mRNA	XR_001033284.2	lncRNA
XM_012143416.2	NRP2	mRNA	XR_001033284.2	lncRNA
XM_012100212.2	ATG7	mRNA	XR_001033284.2	lncRNA
XM_012100212.2	ATG7	mRNA	XR_001033284.2	lncRNA
XM_012100212.2	ATG7	mRNA	XR_001033284.2	lncRNA

XM_012100212.2	ATG7	mRNA	XR_001033284.2	lncRNA
XM_012100212.2	ATG7	mRNA	XR_001033284.2	lncRNA
XM_012100212.2	ATG7	mRNA	XR_001033284.2	lncRNA
XM_015092341.1	USP21	mRNA	XR_001024290.1	lncRNA
XM_015092341.1	USP21	mRNA	XR_001024290.1	lncRNA
XM_015092341.1	USP21	mRNA	XR_001024290.1	lncRNA
XM_015092341.1	USP21	mRNA	XR_001024290.1	lncRNA
XM_015092341.1	USP21	mRNA	XR_001024290.1	lncRNA
XM_015092341.1	USP21	mRNA	XR_001024290.1	lncRNA
XM_015092341.1	USP21	mRNA	XR_001025491.1	lncRNA
XM_015092341.1	USP21	mRNA	XR_001025491.1	lncRNA
XM_015092341.1	USP21	mRNA	XR_001025491.1	lncRNA
XM_015092341.1	USP21	mRNA	XR_001025491.1	lncRNA
XM_015092341.1	USP21	mRNA	XR_001025491.1	lncRNA
XM_015092341.1	USP21	mRNA	XR_001025491.1	lncRNA
XM_012180554.2	TARS2	mRNA	XR_001024290.1	lncRNA
XM_012180554.2	TARS2	mRNA	XR_001024290.1	lncRNA
XM_012180554.2	TARS2	mRNA	XR_001024290.1	lncRNA
XM_012180554.2	TARS2	mRNA	XR_001024290.1	lncRNA
XM_012180554.2	TARS2	mRNA	XR_001024290.1	lncRNA
XM_012180554.2	TARS2	mRNA	XR_001024290.1	lncRNA
XM_012102629.2	SORBS1	mRNA	XR_001024290.1	lncRNA
XM_012102629.2	SORBS1	mRNA	XR_001024290.1	lncRNA
XM_012102629.2	SORBS1	mRNA	XR_001024290.1	lncRNA
XM_012102629.2	SORBS1	mRNA	XR_001024290.1	lncRNA
XM_012102629.2	SORBS1	mRNA	XR_001024290.1	lncRNA
XM_012102629.2	SORBS1	mRNA	XR_001024290.1	lncRNA
XM_015103117.1	ZBTB44	mRNA	XR_001044126.1	lncRNA
XM_015103117.1	ZBTB44	mRNA	XR_001044126.1	lncRNA
XM_015103117.1	ZBTB44	mRNA	XR_001044126.1	lncRNA
XM_015103117.1	ZBTB44	mRNA	XR_001044126.1	lncRNA
XM_015103117.1	ZBTB44	mRNA	XR_001044126.1	lncRNA
XM_015103117.1	ZBTB44	mRNA	XR_001044126.1	lncRNA
XM_012103501.2	MIB1	mRNA	XR_001044126.1	lncRNA
XM_012103501.2	MIB1	mRNA	XR_001044126.1	lncRNA
XM_012103501.2	MIB1	mRNA	XR_001044126.1	lncRNA
XM_012103501.2	MIB1	mRNA	XR_001044126.1	lncRNA
XM_012103501.2	MIB1	mRNA	XR_001044126.1	lncRNA
XM_012103501.2	MIB1	mRNA	XR_001044126.1	lncRNA
XM_012151052.2	WASF2	mRNA	XR_001044126.1	lncRNA
XM_012151052.2	WASF2	mRNA	XR_001044126.1	lncRNA
XM_012151052.2	WASF2	mRNA	XR_001044126.1	lncRNA
XM_012151052.2	WASF2	mRNA	XR_001044126.1	lncRNA
XM_012151052.2	WASF2	mRNA	XR_001044126.1	lncRNA
XM_012151052.2	WASF2	mRNA	XR_001044126.1	lncRNA
XM_012183728.2	LOC10111133?	mRNA	TCONS_00054422	lncRNA
XM_012183728.2	LOC10111133?	mRNA	TCONS_00054422	lncRNA
XM_012183728.2	LOC10111133?	mRNA	TCONS_00054422	lncRNA
XM_012183728.2	LOC10111133?	mRNA	TCONS_00054422	lncRNA
XM_012183728.2	LOC10111133?	mRNA	TCONS_00054422	lncRNA
XM_015099550.1	PARD3	mRNA	XR_001436931.1	lncRNA
XM_015099550.1	PARD3	mRNA	XR_001436931.1	lncRNA
XM_015099550.1	PARD3	mRNA	XR_001436931.1	lncRNA
XM_015099550.1	PARD3	mRNA	XR_001436931.1	lncRNA

XM_015099550.1	PARD3	mRNA	XR_001436931.1	lncRNA
XM_015096594.1	SEC31A	mRNA	TCONS_00075051	lncRNA
XM_015096594.1	SEC31A	mRNA	TCONS_00075051	lncRNA
XM_015096594.1	SEC31A	mRNA	TCONS_00075051	lncRNA
XM_015096594.1	SEC31A	mRNA	TCONS_00075051	lncRNA
XM_015096594.1	SEC31A	mRNA	TCONS_00075051	lncRNA
XM_015102318.1	SETD5	mRNA	TCONS_00078442	lncRNA
XM_015102318.1	SETD5	mRNA	TCONS_00078442	lncRNA
XM_015102318.1	SETD5	mRNA	TCONS_00078442	lncRNA
XM_015102318.1	SETD5	mRNA	TCONS_00078442	lncRNA
XM_015102318.1	SETD5	mRNA	TCONS_00078442	lncRNA
XM_015102318.1	SETD5	mRNA	TCONS_00075051	lncRNA
XM_015102318.1	SETD5	mRNA	TCONS_00075051	lncRNA
XM_015102318.1	SETD5	mRNA	TCONS_00075051	lncRNA
XM_015102318.1	SETD5	mRNA	TCONS_00075051	lncRNA
XM_015102318.1	SETD5	mRNA	TCONS_00075051	lncRNA
XM_004002178.3	LRRC8B	mRNA	XR_001436102.1	lncRNA
XM_004002178.3	LRRC8B	mRNA	XR_001436102.1	lncRNA
XM_004002178.3	LRRC8B	mRNA	XR_001436102.1	lncRNA
XM_004002178.3	LRRC8B	mRNA	XR_001436102.1	lncRNA
XM_004002178.3	LRRC8B	mRNA	XR_001436102.1	lncRNA
XM_004002178.3	LRRC8B	mRNA	XR_001436102.1	lncRNA
XM_012152472.1	LUZP1	mRNA	TCONS_00075453	lncRNA
XM_012152472.1	LUZP1	mRNA	TCONS_00075453	lncRNA
XM_012152472.1	LUZP1	mRNA	TCONS_00075453	lncRNA
XM_012152472.1	LUZP1	mRNA	TCONS_00075453	lncRNA
XM_012152472.1	LUZP1	mRNA	TCONS_00075453	lncRNA
XM_012152472.1	LUZP1	mRNA	TCONS_00075453	lncRNA
XM_012184887.2	DOCK9	mRNA	TCONS_00075453	lncRNA
XM_012184887.2	DOCK9	mRNA	TCONS_00075453	lncRNA
XM_012184887.2	DOCK9	mRNA	TCONS_00075453	lncRNA
XM_012184887.2	DOCK9	mRNA	TCONS_00075453	lncRNA
XM_012184887.2	DOCK9	mRNA	TCONS_00075453	lncRNA
XM_012184887.2	DOCK9	mRNA	TCONS_00075453	lncRNA
XM_012188214.2	ABI1	mRNA	XR_001042381.2	lncRNA
XM_012188214.2	ABI1	mRNA	XR_001042381.2	lncRNA
XM_012188214.2	ABI1	mRNA	XR_001042381.2	lncRNA
XM_012188214.2	ABI1	mRNA	XR_001042381.2	lncRNA
XM_012188214.2	ABI1	mRNA	XR_001042381.2	lncRNA
XM_012188214.2	ABI1	mRNA	XR_001042381.2	lncRNA
XM_012127663.2	IFT74	mRNA	XR_001042381.2	lncRNA
XM_012127663.2	IFT74	mRNA	XR_001042381.2	lncRNA
XM_012127663.2	IFT74	mRNA	XR_001042381.2	lncRNA
XM_012127663.2	IFT74	mRNA	XR_001042381.2	lncRNA
XM_012127663.2	IFT74	mRNA	XR_001042381.2	lncRNA
XM_012127663.2	IFT74	mRNA	XR_001042381.2	lncRNA
XM_015104177.1	PRSS8	mRNA	TCONS_00022613	lncRNA
XM_015104177.1	PRSS8	mRNA	TCONS_00022613	lncRNA
XM_015104177.1	PRSS8	mRNA	TCONS_00022613	lncRNA
XM_015104177.1	PRSS8	mRNA	TCONS_00022613	lncRNA
XM_015104177.1	PRSS8	mRNA	TCONS_00022613	lncRNA
XM_012094768.2	ZMYM1	mRNA	TCONS_00022613	lncRNA
XM_012094768.2	ZMYM1	mRNA	TCONS_00022613	lncRNA
XM_012094768.2	ZMYM1	mRNA	TCONS_00022613	lncRNA

XM_012094768.2	ZMYM1	mRNA	TCONS_00022613	lncRNA
XM_012094768.2	ZMYM1	mRNA	TCONS_00022613	lncRNA
XM_012143452.2	NRP2	mRNA	TCONS_00078440	lncRNA
XM_012143452.2	NRP2	mRNA	TCONS_00078440	lncRNA
XM_012143452.2	NRP2	mRNA	TCONS_00078440	lncRNA
XM_012143452.2	NRP2	mRNA	TCONS_00078440	lncRNA
XM_012143452.2	NRP2	mRNA	TCONS_00078440	lncRNA
XM_012175416.2	PKN2	mRNA	XR_001024743.2	lncRNA
XM_012175416.2	PKN2	mRNA	XR_001024743.2	lncRNA
XM_012175416.2	PKN2	mRNA	XR_001024743.2	lncRNA
XM_012175416.2	PKN2	mRNA	XR_001024743.2	lncRNA
XM_012175416.2	PKN2	mRNA	XR_001024743.2	lncRNA
XM_015098575.1	METTL16	mRNA	XR_001033284.2	lncRNA
XM_015098575.1	METTL16	mRNA	XR_001033284.2	lncRNA
XM_015098575.1	METTL16	mRNA	XR_001033284.2	lncRNA
XM_015098575.1	METTL16	mRNA	XR_001033284.2	lncRNA
XM_015098575.1	METTL16	mRNA	XR_001033284.2	lncRNA
XM_004021989.3	DMD	mRNA	XR_001020707.2	lncRNA
XM_004021989.3	DMD	mRNA	XR_001020707.2	lncRNA
XM_004021989.3	DMD	mRNA	XR_001020707.2	lncRNA
XM_004021989.3	DMD	mRNA	XR_001020707.2	lncRNA
XM_004021989.3	DMD	mRNA	XR_001020707.2	lncRNA
XM_012184629.2	USPL1	mRNA	XR_001436102.1	lncRNA
XM_012184629.2	USPL1	mRNA	XR_001436102.1	lncRNA
XM_012184629.2	USPL1	mRNA	XR_001436102.1	lncRNA
XM_012184629.2	USPL1	mRNA	XR_001436102.1	lncRNA
XM_012184629.2	USPL1	mRNA	XR_001436102.1	lncRNA
XM_015101231.1	FBXW11	mRNA	TCONS_00075051	lncRNA
XM_015101231.1	FBXW11	mRNA	TCONS_00075051	lncRNA
XM_015101231.1	FBXW11	mRNA	TCONS_00075051	lncRNA
XM_015101231.1	FBXW11	mRNA	TCONS_00075051	lncRNA
XM_015101231.1	FBXW11	mRNA	TCONS_00075051	lncRNA
XM_012095465.2	GOLGB1	mRNA	TCONS_00075051	lncRNA
XM_012095465.2	GOLGB1	mRNA	TCONS_00075051	lncRNA
XM_012095465.2	GOLGB1	mRNA	TCONS_00075051	lncRNA
XM_012095465.2	GOLGB1	mRNA	TCONS_00075051	lncRNA
XM_012095465.2	GOLGB1	mRNA	TCONS_00075051	lncRNA
XM_012182026.2	ENTPD5	mRNA	TCONS_00075051	lncRNA
XM_012182026.2	ENTPD5	mRNA	TCONS_00075051	lncRNA
XM_012182026.2	ENTPD5	mRNA	TCONS_00075051	lncRNA
XM_012182026.2	ENTPD5	mRNA	TCONS_00075051	lncRNA
XM_012182026.2	ENTPD5	mRNA	TCONS_00075051	lncRNA
XM_012098820.2	RALGAPA1	mRNA	XR_001020707.2	lncRNA
XM_012098820.2	RALGAPA1	mRNA	XR_001020707.2	lncRNA
XM_012098820.2	RALGAPA1	mRNA	XR_001020707.2	lncRNA
XM_012098820.2	RALGAPA1	mRNA	XR_001020707.2	lncRNA
XM_012098820.2	RALGAPA1	mRNA	XR_001020707.2	lncRNA
XM_012098820.2	RALGAPA1	mRNA	XR_001020707.2	lncRNA
XM_015099550.1	PARD3	mRNA	XR_001033284.2	lncRNA
XM_015099550.1	PARD3	mRNA	XR_001033284.2	lncRNA
XM_015099550.1	PARD3	mRNA	XR_001033284.2	lncRNA
XM_015099550.1	PARD3	mRNA	XR_001033284.2	lncRNA
XM_015099550.1	PARD3	mRNA	XR_001033284.2	lncRNA
XM_015099550.1	PARD3	mRNA	XR_001033284.2	lncRNA

XM_012184884.2	DOCK9	mRNA	XR_001033284.2	lncRNA
XM_012184884.2	DOCK9	mRNA	XR_001033284.2	lncRNA
XM_012184884.2	DOCK9	mRNA	XR_001033284.2	lncRNA
XM_012184884.2	DOCK9	mRNA	XR_001033284.2	lncRNA
XM_012184884.2	DOCK9	mRNA	XR_001033284.2	lncRNA
XM_012184884.2	DOCK9	mRNA	XR_001033284.2	lncRNA
XM_012184885.2	DOCK9	mRNA	XR_001033284.2	lncRNA
XM_012184885.2	DOCK9	mRNA	XR_001033284.2	lncRNA
XM_012184885.2	DOCK9	mRNA	XR_001033284.2	lncRNA
XM_012184885.2	DOCK9	mRNA	XR_001033284.2	lncRNA
XM_012184885.2	DOCK9	mRNA	XR_001033284.2	lncRNA
XM_012184885.2	DOCK9	mRNA	XR_001033284.2	lncRNA
XM_012190370.2	FAM98C	mRNA	XR_001041211.2	lncRNA
XM_012190370.2	FAM98C	mRNA	XR_001041211.2	lncRNA
XM_012190370.2	FAM98C	mRNA	XR_001041211.2	lncRNA
XM_012190370.2	FAM98C	mRNA	XR_001041211.2	lncRNA
XM_012190370.2	FAM98C	mRNA	XR_001041211.2	lncRNA
XM_012190370.2	FAM98C	mRNA	XR_001041211.2	lncRNA
XM_004014196.3	ABI1	mRNA	XR_001041211.2	lncRNA
XM_004014196.3	ABI1	mRNA	XR_001041211.2	lncRNA
XM_004014196.3	ABI1	mRNA	XR_001041211.2	lncRNA
XM_004014196.3	ABI1	mRNA	XR_001041211.2	lncRNA
XM_004014196.3	ABI1	mRNA	XR_001041211.2	lncRNA
XM_004014196.3	ABI1	mRNA	XR_001041211.2	lncRNA
XM_012104141.2	PDXDC1	mRNA	XR_001434761.1	lncRNA
XM_012104141.2	PDXDC1	mRNA	XR_001434761.1	lncRNA
XM_012104141.2	PDXDC1	mRNA	XR_001434761.1	lncRNA
XM_012104141.2	PDXDC1	mRNA	XR_001434761.1	lncRNA
XM_012104141.2	PDXDC1	mRNA	XR_001434761.1	lncRNA
XM_012104141.2	PDXDC1	mRNA	XR_001434761.1	lncRNA
XM_015095233.1	KIAA1324L	mRNA	XR_001434892.1	lncRNA
XM_015095233.1	KIAA1324L	mRNA	XR_001434892.1	lncRNA
XM_015095233.1	KIAA1324L	mRNA	XR_001434892.1	lncRNA
XM_015095233.1	KIAA1324L	mRNA	XR_001434892.1	lncRNA
XM_015095233.1	KIAA1324L	mRNA	XR_001434892.1	lncRNA
XM_015095233.1	KIAA1324L	mRNA	XR_001434892.1	lncRNA
XM_015103475.1	PLEKHA1	mRNA	XR_001025491.1	lncRNA
XM_015103475.1	PLEKHA1	mRNA	XR_001025491.1	lncRNA
XM_015103475.1	PLEKHA1	mRNA	XR_001025491.1	lncRNA
XM_015103475.1	PLEKHA1	mRNA	XR_001025491.1	lncRNA
XM_015103475.1	PLEKHA1	mRNA	XR_001025491.1	lncRNA
XM_015103475.1	PLEKHA1	mRNA	XR_001025491.1	lncRNA
XM_012176510.2	FNBP1L	mRNA	XR_001024290.1	lncRNA
XM_012176510.2	FNBP1L	mRNA	XR_001024290.1	lncRNA
XM_012176510.2	FNBP1L	mRNA	XR_001024290.1	lncRNA
XM_012176510.2	FNBP1L	mRNA	XR_001024290.1	lncRNA
XM_012176510.2	FNBP1L	mRNA	XR_001024290.1	lncRNA
XM_012176510.2	FNBP1L	mRNA	XR_001024290.1	lncRNA
XM_004010552.3	VPS13C	mRNA	XR_001436127.1	lncRNA
XM_004010552.3	VPS13C	mRNA	XR_001436127.1	lncRNA
XM_004010552.3	VPS13C	mRNA	XR_001436127.1	lncRNA
XM_004010552.3	VPS13C	mRNA	XR_001436127.1	lncRNA
XM_004010552.3	VPS13C	mRNA	XR_001436127.1	lncRNA
XM_004010552.3	VPS13C	mRNA	XR_001436127.1	lncRNA

XM_004006510.3	SLC16A7	mRNA	XR_001034847.2	lncRNA
XM_004006510.3	SLC16A7	mRNA	XR_001034847.2	lncRNA
XM_004006510.3	SLC16A7	mRNA	XR_001034847.2	lncRNA
XM_004006510.3	SLC16A7	mRNA	XR_001034847.2	lncRNA
XM_004006510.3	SLC16A7	mRNA	XR_001034847.2	lncRNA
XM_004006510.3	SLC16A7	mRNA	XR_001034847.2	lncRNA
XM_004006510.3	SLC16A7	mRNA	XR_001434202.1	lncRNA
XM_004006510.3	SLC16A7	mRNA	XR_001434202.1	lncRNA
XM_004006510.3	SLC16A7	mRNA	XR_001434202.1	lncRNA
XM_004006510.3	SLC16A7	mRNA	XR_001434202.1	lncRNA
XM_004006510.3	SLC16A7	mRNA	XR_001434202.1	lncRNA
XM_004006510.3	SLC16A7	mRNA	XR_001434202.1	lncRNA
XM_012134956.1	STK39	mRNA	XR_001034847.2	lncRNA
XM_012134956.1	STK39	mRNA	XR_001034847.2	lncRNA
XM_012134956.1	STK39	mRNA	XR_001034847.2	lncRNA
XM_012134956.1	STK39	mRNA	XR_001034847.2	lncRNA
XM_012134956.1	STK39	mRNA	XR_001034847.2	lncRNA
XM_012134956.1	STK39	mRNA	XR_001034847.2	lncRNA
XM_012134956.1	STK39	mRNA	XR_001434202.1	lncRNA
XM_012134956.1	STK39	mRNA	XR_001434202.1	lncRNA
XM_012134956.1	STK39	mRNA	XR_001434202.1	lncRNA
XM_012134956.1	STK39	mRNA	XR_001434202.1	lncRNA
XM_012134956.1	STK39	mRNA	XR_001434202.1	lncRNA
XM_012134956.1	STK39	mRNA	XR_001434202.1	lncRNA
XM_004010577.2	CGNL1	mRNA	XR_001436931.1	lncRNA
XM_004010577.2	CGNL1	mRNA	XR_001436931.1	lncRNA
XM_004010577.2	CGNL1	mRNA	XR_001436931.1	lncRNA
XM_004010577.2	CGNL1	mRNA	XR_001436931.1	lncRNA
XM_004010577.2	CGNL1	mRNA	XR_001436931.1	lncRNA
XM_012181456.2	ADAM10	mRNA	XR_001436931.1	lncRNA
XM_012181456.2	ADAM10	mRNA	XR_001436931.1	lncRNA
XM_012181456.2	ADAM10	mRNA	XR_001436931.1	lncRNA
XM_012181456.2	ADAM10	mRNA	XR_001436931.1	lncRNA
XM_012181456.2	ADAM10	mRNA	XR_001436931.1	lncRNA
XM_012174394.2	RAPGEF3	mRNA	TCONS_00054422	lncRNA
XM_012174394.2	RAPGEF3	mRNA	TCONS_00054422	lncRNA
XM_012174394.2	RAPGEF3	mRNA	TCONS_00054422	lncRNA
XM_012174394.2	RAPGEF3	mRNA	TCONS_00054422	lncRNA
XM_012174394.2	RAPGEF3	mRNA	TCONS_00054422	lncRNA
XM_004002018.3	OMA1	mRNA	XR_001436127.1	lncRNA
XM_004002018.3	OMA1	mRNA	XR_001436127.1	lncRNA
XM_004002018.3	OMA1	mRNA	XR_001436127.1	lncRNA
XM_004002018.3	OMA1	mRNA	XR_001436127.1	lncRNA
XM_004002018.3	OMA1	mRNA	XR_001436127.1	lncRNA
XM_012184946.1	LIG4	mRNA	XR_001024290.1	lncRNA
XM_012184946.1	LIG4	mRNA	XR_001024290.1	lncRNA
XM_012184946.1	LIG4	mRNA	XR_001024290.1	lncRNA
XM_012184946.1	LIG4	mRNA	XR_001024290.1	lncRNA
XM_012184946.1	LIG4	mRNA	XR_001024290.1	lncRNA
XM_015104327.1	LOC101115427	mRNA	XR_001436817.1	lncRNA
XM_015104327.1	LOC101115427	mRNA	XR_001436817.1	lncRNA
XM_015104327.1	LOC101115427	mRNA	XR_001436817.1	lncRNA
XM_015104327.1	LOC101115427	mRNA	XR_001436817.1	lncRNA
XM_015104327.1	LOC101115427	mRNA	XR_001436817.1	lncRNA

XM_015104327.1	LOC101115427	mRNA	XR_001436817.1	lncRNA
XM_012104594.2	ZNF789	mRNA	TCONS_00075453	lncRNA
XM_012104594.2	ZNF789	mRNA	TCONS_00075453	lncRNA
XM_012104594.2	ZNF789	mRNA	TCONS_00075453	lncRNA
XM_012104594.2	ZNF789	mRNA	TCONS_00075453	lncRNA
XM_012104594.2	ZNF789	mRNA	TCONS_00075453	lncRNA
XM_012174768.2	RAB5B	mRNA	XR_001021689.2	lncRNA
XM_012174768.2	RAB5B	mRNA	XR_001021689.2	lncRNA
XM_012174768.2	RAB5B	mRNA	XR_001021689.2	lncRNA
XM_012174768.2	RAB5B	mRNA	XR_001021689.2	lncRNA
XM_012174768.2	RAB5B	mRNA	XR_001021689.2	lncRNA
XM_012174768.2	RAB5B	mRNA	XR_001021689.2	lncRNA
XM_015092323.1	KIRREL	mRNA	TCONS_00075453	lncRNA
XM_015092323.1	KIRREL	mRNA	TCONS_00075453	lncRNA
XM_015092323.1	KIRREL	mRNA	TCONS_00075453	lncRNA
XM_015092323.1	KIRREL	mRNA	TCONS_00075453	lncRNA
XM_015092323.1	KIRREL	mRNA	TCONS_00075453	lncRNA
XM_015092323.1	KIRREL	mRNA	TCONS_00075453	lncRNA
XM_012185285.2	SYNRG	mRNA	TCONS_00075051	lncRNA
XM_012185285.2	SYNRG	mRNA	TCONS_00075051	lncRNA
XM_012185285.2	SYNRG	mRNA	TCONS_00075051	lncRNA
XM_012185285.2	SYNRG	mRNA	TCONS_00075051	lncRNA
XM_012185285.2	SYNRG	mRNA	TCONS_00075051	lncRNA
XM_015098446.1	VEZF1	mRNA	TCONS_00078442	lncRNA
XM_015098446.1	VEZF1	mRNA	TCONS_00078442	lncRNA
XM_015098446.1	VEZF1	mRNA	TCONS_00078442	lncRNA
XM_015098446.1	VEZF1	mRNA	TCONS_00078442	lncRNA
XM_015098446.1	VEZF1	mRNA	TCONS_00078442	lncRNA
XM_012096314.1	PHF21A	mRNA	TCONS_00075051	lncRNA
XM_012096314.1	PHF21A	mRNA	TCONS_00075051	lncRNA
XM_012096314.1	PHF21A	mRNA	TCONS_00075051	lncRNA
XM_012096314.1	PHF21A	mRNA	TCONS_00075051	lncRNA
XM_012096314.1	PHF21A	mRNA	TCONS_00075051	lncRNA
XM_015102486.1	NR2C2	mRNA	TCONS_00082638	lncRNA
XM_015102486.1	NR2C2	mRNA	TCONS_00082638	lncRNA
XM_015102486.1	NR2C2	mRNA	TCONS_00082638	lncRNA
XM_015102486.1	NR2C2	mRNA	TCONS_00082638	lncRNA
XM_015102486.1	NR2C2	mRNA	TCONS_00082638	lncRNA
XM_012095502.1	SIK3	mRNA	TCONS_00075051	lncRNA
XM_012095502.1	SIK3	mRNA	TCONS_00075051	lncRNA
XM_012095502.1	SIK3	mRNA	TCONS_00075051	lncRNA
XM_012095502.1	SIK3	mRNA	TCONS_00075051	lncRNA
XM_012095502.1	SIK3	mRNA	TCONS_00075051	lncRNA
XM_015098446.1	VEZF1	mRNA	TCONS_00084305	lncRNA
XM_015098446.1	VEZF1	mRNA	TCONS_00084305	lncRNA
XM_015098446.1	VEZF1	mRNA	TCONS_00084305	lncRNA
XM_015098446.1	VEZF1	mRNA	TCONS_00084305	lncRNA
XM_015098446.1	VEZF1	mRNA	TCONS_00084305	lncRNA
XM_012173780.2	REV1	mRNA	XR_001025491.1	lncRNA
XM_012173780.2	REV1	mRNA	XR_001025491.1	lncRNA
XM_012173780.2	REV1	mRNA	XR_001025491.1	lncRNA
XM_012173780.2	REV1	mRNA	XR_001025491.1	lncRNA
XM_012173780.2	REV1	mRNA	XR_001025491.1	lncRNA
XM_012173780.2	REV1	mRNA	XR_001025491.1	lncRNA

XM_015101031.1	MICAL2	mRNA	XR_001436127.1	lncRNA
XM_015101031.1	MICAL2	mRNA	XR_001436127.1	lncRNA
XM_015101031.1	MICAL2	mRNA	XR_001436127.1	lncRNA
XM_015101031.1	MICAL2	mRNA	XR_001436127.1	lncRNA
XM_015101031.1	MICAL2	mRNA	XR_001436127.1	lncRNA
XM_015101031.1	MICAL2	mRNA	XR_001436127.1	lncRNA
XM_012098820.2	RALGAPA1	mRNA	XR_001044126.1	lncRNA
XM_012098820.2	RALGAPA1	mRNA	XR_001044126.1	lncRNA
XM_012098820.2	RALGAPA1	mRNA	XR_001044126.1	lncRNA
XM_012098820.2	RALGAPA1	mRNA	XR_001044126.1	lncRNA
XM_012098820.2	RALGAPA1	mRNA	XR_001044126.1	lncRNA
XM_012098820.2	RALGAPA1	mRNA	XR_001044126.1	lncRNA
XM_012173296.2	CDKL4	mRNA	XR_001025491.1	lncRNA
XM_012173296.2	CDKL4	mRNA	XR_001025491.1	lncRNA
XM_012173296.2	CDKL4	mRNA	XR_001025491.1	lncRNA
XM_012173296.2	CDKL4	mRNA	XR_001025491.1	lncRNA
XM_012173296.2	CDKL4	mRNA	XR_001025491.1	lncRNA
XM_012173296.2	CDKL4	mRNA	XR_001025491.1	lncRNA
XM_015095425.1	CUL1	mRNA	TCONS_00022613	lncRNA
XM_015095425.1	CUL1	mRNA	TCONS_00022613	lncRNA
XM_015095425.1	CUL1	mRNA	TCONS_00022613	lncRNA
XM_015095425.1	CUL1	mRNA	TCONS_00022613	lncRNA
XM_015095425.1	CUL1	mRNA	TCONS_00022613	lncRNA
XM_015099971.1	ZNF181	mRNA	TCONS_00022613	lncRNA
XM_015099971.1	ZNF181	mRNA	TCONS_00022613	lncRNA
XM_015099971.1	ZNF181	mRNA	TCONS_00022613	lncRNA
XM_015099971.1	ZNF181	mRNA	TCONS_00022613	lncRNA
XM_015099971.1	ZNF181	mRNA	TCONS_00022613	lncRNA
XM_012095401.2	ATM	mRNA	TCONS_00022613	lncRNA
XM_012095401.2	ATM	mRNA	TCONS_00022613	lncRNA
XM_012095401.2	ATM	mRNA	TCONS_00022613	lncRNA
XM_012095401.2	ATM	mRNA	TCONS_00022613	lncRNA
XM_012095401.2	ATM	mRNA	TCONS_00022613	lncRNA
XM_015094471.1	CCDC142	mRNA	XR_001024743.2	lncRNA
XM_015094471.1	CCDC142	mRNA	XR_001024743.2	lncRNA
XM_015094471.1	CCDC142	mRNA	XR_001024743.2	lncRNA
XM_015094471.1	CCDC142	mRNA	XR_001024743.2	lncRNA
XM_015094471.1	CCDC142	mRNA	XR_001024743.2	lncRNA
XM_012152455.2	LUZP1	mRNA	XR_001033284.2	lncRNA
XM_012152455.2	LUZP1	mRNA	XR_001033284.2	lncRNA
XM_012152455.2	LUZP1	mRNA	XR_001033284.2	lncRNA
XM_012152455.2	LUZP1	mRNA	XR_001033284.2	lncRNA
XM_012152455.2	LUZP1	mRNA	XR_001033284.2	lncRNA
XM_012152455.2	LUZP1	mRNA	XR_001033284.2	lncRNA
XM_004008647.3	RAPGEF6	mRNA	XR_001034847.2	lncRNA
XM_004008647.3	RAPGEF6	mRNA	XR_001034847.2	lncRNA
XM_004008647.3	RAPGEF6	mRNA	XR_001034847.2	lncRNA
XM_004008647.3	RAPGEF6	mRNA	XR_001034847.2	lncRNA
XM_004008647.3	RAPGEF6	mRNA	XR_001034847.2	lncRNA
XM_004008647.3	RAPGEF6	mRNA	XR_001034847.2	lncRNA
XM_012181190.2	EIF2AK4	mRNA	XR_001034847.2	lncRNA
XM_012181190.2	EIF2AK4	mRNA	XR_001034847.2	lncRNA
XM_012181190.2	EIF2AK4	mRNA	XR_001034847.2	lncRNA
XM_012181190.2	EIF2AK4	mRNA	XR_001034847.2	lncRNA

XM_012181190.2	EIF2AK4	mRNA	XR_001034847.2	lncRNA
XM_012181190.2	EIF2AK4	mRNA	XR_001034847.2	lncRNA
XM_012176217.2	EVI5	mRNA	XR_001034847.2	lncRNA
XM_012176217.2	EVI5	mRNA	XR_001034847.2	lncRNA
XM_012176217.2	EVI5	mRNA	XR_001034847.2	lncRNA
XM_012176217.2	EVI5	mRNA	XR_001034847.2	lncRNA
XM_012176217.2	EVI5	mRNA	XR_001034847.2	lncRNA
XM_012176217.2	EVI5	mRNA	XR_001034847.2	lncRNA
XM_012176217.2	EVI5	mRNA	XR_001434202.1	lncRNA
XM_012176217.2	EVI5	mRNA	XR_001434202.1	lncRNA
XM_012176217.2	EVI5	mRNA	XR_001434202.1	lncRNA
XM_012176217.2	EVI5	mRNA	XR_001434202.1	lncRNA
XM_012176217.2	EVI5	mRNA	XR_001434202.1	lncRNA
XM_012176217.2	EVI5	mRNA	XR_001434202.1	lncRNA
XM_004008647.3	RAPGEF6	mRNA	XR_001434202.1	lncRNA
XM_004008647.3	RAPGEF6	mRNA	XR_001434202.1	lncRNA
XM_004008647.3	RAPGEF6	mRNA	XR_001434202.1	lncRNA
XM_004008647.3	RAPGEF6	mRNA	XR_001434202.1	lncRNA
XM_004008647.3	RAPGEF6	mRNA	XR_001434202.1	lncRNA
XM_004008647.3	RAPGEF6	mRNA	XR_001434202.1	lncRNA
XM_012181190.2	EIF2AK4	mRNA	XR_001434202.1	lncRNA
XM_012181190.2	EIF2AK4	mRNA	XR_001434202.1	lncRNA
XM_012181190.2	EIF2AK4	mRNA	XR_001434202.1	lncRNA
XM_012181190.2	EIF2AK4	mRNA	XR_001434202.1	lncRNA
XM_012181190.2	EIF2AK4	mRNA	XR_001434202.1	lncRNA
XM_012181190.2	EIF2AK4	mRNA	XR_001434202.1	lncRNA
XM_012182400.2	SENP6	mRNA	XR_001434761.1	lncRNA
XM_012182400.2	SENP6	mRNA	XR_001434761.1	lncRNA
XM_012182400.2	SENP6	mRNA	XR_001434761.1	lncRNA
XM_012182400.2	SENP6	mRNA	XR_001434761.1	lncRNA
XM_012182400.2	SENP6	mRNA	XR_001434761.1	lncRNA
XM_015098513.1	RHOT1	mRNA	XR_001434761.1	lncRNA
XM_015098513.1	RHOT1	mRNA	XR_001434761.1	lncRNA
XM_015098513.1	RHOT1	mRNA	XR_001434761.1	lncRNA
XM_015098513.1	RHOT1	mRNA	XR_001434761.1	lncRNA
XM_015098513.1	RHOT1	mRNA	XR_001434761.1	lncRNA
XM_012180475.2	CIART	mRNA	XR_001434761.1	lncRNA
XM_012180475.2	CIART	mRNA	XR_001434761.1	lncRNA
XM_012180475.2	CIART	mRNA	XR_001434761.1	lncRNA
XM_012180475.2	CIART	mRNA	XR_001434761.1	lncRNA
XM_012180475.2	CIART	mRNA	XR_001434761.1	lncRNA
XM_015098003.1	LOC10110962(	mRNA	XR_001434761.1	lncRNA
XM_015098003.1	LOC10110962(	mRNA	XR_001434761.1	lncRNA
XM_015098003.1	LOC10110962(	mRNA	XR_001434761.1	lncRNA
XM_015098003.1	LOC10110962(	mRNA	XR_001434761.1	lncRNA
XM_015098003.1	LOC10110962(	mRNA	XR_001434761.1	lncRNA
XM_015093911.1	LOC10110405	mRNA	XR_001434761.1	lncRNA
XM_015093911.1	LOC10110405	mRNA	XR_001434761.1	lncRNA
XM_015093911.1	LOC10110405	mRNA	XR_001434761.1	lncRNA
XM_015093911.1	LOC10110405	mRNA	XR_001434761.1	lncRNA
XM_015093911.1	LOC10110405	mRNA	XR_001434761.1	lncRNA
XM_012182428.1	C1H1orf43	mRNA	XR_001434761.1	lncRNA
XM_012182428.1	C1H1orf43	mRNA	XR_001434761.1	lncRNA
XM_012182428.1	C1H1orf43	mRNA	XR_001434761.1	lncRNA

XM_012182428.1	C1H1orf43	mRNA	XR_001434761.1	lncRNA
XM_012182428.1	C1H1orf43	mRNA	XR_001434761.1	lncRNA
XM_012122747.1	RNF220	mRNA	TCONS_00075051	lncRNA
XM_012122747.1	RNF220	mRNA	TCONS_00075051	lncRNA
XM_012122747.1	RNF220	mRNA	TCONS_00075051	lncRNA
XM_012122747.1	RNF220	mRNA	TCONS_00075051	lncRNA
XM_012122747.1	RNF220	mRNA	TCONS_00075051	lncRNA
XM_012190071.1	ATXN1L	mRNA	TCONS_00075051	lncRNA
XM_012190071.1	ATXN1L	mRNA	TCONS_00075051	lncRNA
XM_012190071.1	ATXN1L	mRNA	TCONS_00075051	lncRNA
XM_012190071.1	ATXN1L	mRNA	TCONS_00075051	lncRNA
XM_012190071.1	ATXN1L	mRNA	TCONS_00075051	lncRNA
XM_015104846.1	TAB3	mRNA	XR_001434761.1	lncRNA
XM_015104846.1	TAB3	mRNA	XR_001434761.1	lncRNA
XM_015104846.1	TAB3	mRNA	XR_001434761.1	lncRNA
XM_015104846.1	TAB3	mRNA	XR_001434761.1	lncRNA
XM_015104846.1	TAB3	mRNA	XR_001434761.1	lncRNA
XM_015104846.1	TAB3	mRNA	XR_001434761.1	lncRNA
XM_015098434.1	STXBP4	mRNA	XR_001434761.1	lncRNA
XM_015098434.1	STXBP4	mRNA	XR_001434761.1	lncRNA
XM_015098434.1	STXBP4	mRNA	XR_001434761.1	lncRNA
XM_015098434.1	STXBP4	mRNA	XR_001434761.1	lncRNA
XM_015098434.1	STXBP4	mRNA	XR_001434761.1	lncRNA
XM_015098434.1	STXBP4	mRNA	XR_001434761.1	lncRNA
XM_012104594.2	ZNF789	mRNA	XR_001033284.2	lncRNA
XM_012104594.2	ZNF789	mRNA	XR_001033284.2	lncRNA
XM_012104594.2	ZNF789	mRNA	XR_001033284.2	lncRNA
XM_012104594.2	ZNF789	mRNA	XR_001033284.2	lncRNA
XM_012104594.2	ZNF789	mRNA	XR_001033284.2	lncRNA
XM_012189374.1	NXPE3	mRNA	XR_001033284.2	lncRNA
XM_012189374.1	NXPE3	mRNA	XR_001033284.2	lncRNA
XM_012189374.1	NXPE3	mRNA	XR_001033284.2	lncRNA
XM_012189374.1	NXPE3	mRNA	XR_001033284.2	lncRNA
XM_012189374.1	NXPE3	mRNA	XR_001033284.2	lncRNA
XM_012151052.2	WASF2	mRNA	XR_001436102.1	lncRNA
XM_012151052.2	WASF2	mRNA	XR_001436102.1	lncRNA
XM_012151052.2	WASF2	mRNA	XR_001436102.1	lncRNA
XM_012151052.2	WASF2	mRNA	XR_001436102.1	lncRNA
XM_012151052.2	WASF2	mRNA	XR_001436102.1	lncRNA
XM_012151052.2	WASF2	mRNA	XR_001436102.1	lncRNA
XM_012098709.2	PML	mRNA	XR_001436102.1	lncRNA
XM_012098709.2	PML	mRNA	XR_001436102.1	lncRNA
XM_012098709.2	PML	mRNA	XR_001436102.1	lncRNA
XM_012098709.2	PML	mRNA	XR_001436102.1	lncRNA
XM_012098709.2	PML	mRNA	XR_001436102.1	lncRNA
XM_012098709.2	PML	mRNA	XR_001436102.1	lncRNA
XM_012102629.2	SORBS1	mRNA	XR_001436102.1	lncRNA
XM_012102629.2	SORBS1	mRNA	XR_001436102.1	lncRNA
XM_012102629.2	SORBS1	mRNA	XR_001436102.1	lncRNA
XM_012102629.2	SORBS1	mRNA	XR_001436102.1	lncRNA
XM_012102629.2	SORBS1	mRNA	XR_001436102.1	lncRNA
XM_012102629.2	SORBS1	mRNA	XR_001436102.1	lncRNA
XM_015092341.1	USP21	mRNA	XR_001436102.1	lncRNA
XM_015092341.1	USP21	mRNA	XR_001436102.1	lncRNA

XM_015092341.1	USP21	mRNA	XR_001436102.1	lncRNA
XM_015092341.1	USP21	mRNA	XR_001436102.1	lncRNA
XM_015092341.1	USP21	mRNA	XR_001436102.1	lncRNA
XM_015092341.1	USP21	mRNA	XR_001436102.1	lncRNA
XM_012104950.1	TTC13	mRNA	XR_001436102.1	lncRNA
XM_012104950.1	TTC13	mRNA	XR_001436102.1	lncRNA
XM_012104950.1	TTC13	mRNA	XR_001436102.1	lncRNA
XM_012104950.1	TTC13	mRNA	XR_001436102.1	lncRNA
XM_012104950.1	TTC13	mRNA	XR_001436102.1	lncRNA
XM_012104950.1	TTC13	mRNA	XR_001436102.1	lncRNA
XM_012097680.2	SCARB1	mRNA	XR_001025491.1	lncRNA
XM_012097680.2	SCARB1	mRNA	XR_001025491.1	lncRNA
XM_012097680.2	SCARB1	mRNA	XR_001025491.1	lncRNA
XM_012097680.2	SCARB1	mRNA	XR_001025491.1	lncRNA
XM_012097680.2	SCARB1	mRNA	XR_001025491.1	lncRNA
XM_012097680.2	SCARB1	mRNA	XR_001025491.1	lncRNA
XM_012099038.2	SLC25A29	mRNA	XR_001024290.1	lncRNA
XM_012099038.2	SLC25A29	mRNA	XR_001024290.1	lncRNA
XM_012099038.2	SLC25A29	mRNA	XR_001024290.1	lncRNA
XM_012099038.2	SLC25A29	mRNA	XR_001024290.1	lncRNA
XM_012099038.2	SLC25A29	mRNA	XR_001024290.1	lncRNA
XM_012099038.2	SLC25A29	mRNA	XR_001024290.1	lncRNA
XM_012185799.2	EPN2	mRNA	TCONS_00082638	lncRNA
XM_012185799.2	EPN2	mRNA	TCONS_00082638	lncRNA
XM_012185799.2	EPN2	mRNA	TCONS_00082638	lncRNA
XM_012185799.2	EPN2	mRNA	TCONS_00082638	lncRNA
XM_012185799.2	EPN2	mRNA	TCONS_00082638	lncRNA
XM_015103466.1	TACC2	mRNA	XR_001436931.1	lncRNA
XM_015103466.1	TACC2	mRNA	XR_001436931.1	lncRNA
XM_015103466.1	TACC2	mRNA	XR_001436931.1	lncRNA
XM_015103466.1	TACC2	mRNA	XR_001436931.1	lncRNA
XM_015103466.1	TACC2	mRNA	XR_001436931.1	lncRNA
XM_015103461.1	TACC2	mRNA	TCONS_00082638	lncRNA
XM_015103461.1	TACC2	mRNA	TCONS_00082638	lncRNA
XM_015103461.1	TACC2	mRNA	TCONS_00082638	lncRNA
XM_015103461.1	TACC2	mRNA	TCONS_00082638	lncRNA
XM_015103461.1	TACC2	mRNA	TCONS_00082638	lncRNA
XM_012178308.2	FAM193B	mRNA	XR_001024743.2	lncRNA
XM_012178308.2	FAM193B	mRNA	XR_001024743.2	lncRNA
XM_012178308.2	FAM193B	mRNA	XR_001024743.2	lncRNA
XM_012178308.2	FAM193B	mRNA	XR_001024743.2	lncRNA
XM_012178308.2	FAM193B	mRNA	XR_001024743.2	lncRNA
XM_015099551.1	PARD3	mRNA	XR_001024743.2	lncRNA
XM_015099551.1	PARD3	mRNA	XR_001024743.2	lncRNA
XM_015099551.1	PARD3	mRNA	XR_001024743.2	lncRNA
XM_015099551.1	PARD3	mRNA	XR_001024743.2	lncRNA
XM_015099551.1	PARD3	mRNA	XR_001024743.2	lncRNA
XM_012182435.2	C1H1orf43	mRNA	TCONS_00054422	lncRNA
XM_012182435.2	C1H1orf43	mRNA	TCONS_00054422	lncRNA
XM_012182435.2	C1H1orf43	mRNA	TCONS_00054422	lncRNA
XM_012182435.2	C1H1orf43	mRNA	TCONS_00054422	lncRNA
XM_012184629.2	USPL1	mRNA	XR_001436931.1	lncRNA
XM_012184629.2	USPL1	mRNA	XR_001436931.1	lncRNA
XM_012184629.2	USPL1	mRNA	XR_001436931.1	lncRNA

XM_012184629.2	USPL1	mRNA	XR_001436931.1	lncRNA
XM_012149251.2	S100PBP	mRNA	TCONS_00075453	lncRNA
XM_012149251.2	S100PBP	mRNA	TCONS_00075453	lncRNA
XM_012149251.2	S100PBP	mRNA	TCONS_00075453	lncRNA
XM_012149251.2	S100PBP	mRNA	TCONS_00075453	lncRNA
XM_012149251.2	S100PBP	mRNA	TCONS_00075453	lncRNA
XM_015094531.1	ANAPC1	mRNA	XR_001436931.1	lncRNA
XM_015094531.1	ANAPC1	mRNA	XR_001436931.1	lncRNA
XM_015094531.1	ANAPC1	mRNA	XR_001436931.1	lncRNA
XM_015094531.1	ANAPC1	mRNA	XR_001436931.1	lncRNA
XM_015094531.1	ANAPC1	mRNA	XR_001436931.1	lncRNA
XM_015104296.1	ADAMTS14	mRNA	TCONS_00084305	lncRNA
XM_015104296.1	ADAMTS14	mRNA	TCONS_00084305	lncRNA
XM_015104296.1	ADAMTS14	mRNA	TCONS_00084305	lncRNA
XM_015104296.1	ADAMTS14	mRNA	TCONS_00084305	lncRNA
XM_015104296.1	ADAMTS14	mRNA	TCONS_00084305	lncRNA
XM_012143523.2	INO80D	mRNA	TCONS_00084305	lncRNA
XM_012143523.2	INO80D	mRNA	TCONS_00084305	lncRNA
XM_012143523.2	INO80D	mRNA	TCONS_00084305	lncRNA
XM_012143523.2	INO80D	mRNA	TCONS_00084305	lncRNA
XM_012143523.2	INO80D	mRNA	TCONS_00084305	lncRNA
XM_012143523.2	INO80D	mRNA	TCONS_00078442	lncRNA
XM_012143523.2	INO80D	mRNA	TCONS_00078442	lncRNA
XM_012143523.2	INO80D	mRNA	TCONS_00078442	lncRNA
XM_012143523.2	INO80D	mRNA	TCONS_00078442	lncRNA
XM_012143523.2	INO80D	mRNA	TCONS_00078442	lncRNA
XM_012187378.2	CAMTA1	mRNA	TCONS_00082638	lncRNA
XM_012187378.2	CAMTA1	mRNA	TCONS_00082638	lncRNA
XM_012187378.2	CAMTA1	mRNA	TCONS_00082638	lncRNA
XM_012187378.2	CAMTA1	mRNA	TCONS_00082638	lncRNA
XM_012187378.2	CAMTA1	mRNA	TCONS_00082638	lncRNA
XM_012105643.1	ZFP69	mRNA	XR_001436532.1	lncRNA
XM_012105643.1	ZFP69	mRNA	XR_001436532.1	lncRNA
XM_012105643.1	ZFP69	mRNA	XR_001436532.1	lncRNA
XM_012105643.1	ZFP69	mRNA	XR_001436532.1	lncRNA
XM_012105643.1	ZFP69	mRNA	XR_001436532.1	lncRNA
XM_012182435.2	C1H1orf43	mRNA	XR_001436532.1	lncRNA
XM_012182435.2	C1H1orf43	mRNA	XR_001436532.1	lncRNA
XM_012182435.2	C1H1orf43	mRNA	XR_001436532.1	lncRNA
XM_012182435.2	C1H1orf43	mRNA	XR_001436532.1	lncRNA
XM_012182435.2	C1H1orf43	mRNA	XR_001436532.1	lncRNA
XM_012125926.2	ZDHHC21	mRNA	XR_001042381.2	lncRNA
XM_012125926.2	ZDHHC21	mRNA	XR_001042381.2	lncRNA
XM_012125926.2	ZDHHC21	mRNA	XR_001042381.2	lncRNA
XM_012125926.2	ZDHHC21	mRNA	XR_001042381.2	lncRNA
XM_012125926.2	ZDHHC21	mRNA	XR_001042381.2	lncRNA
XM_004011187.3	LOC10110720(	mRNA	XR_001436532.1	lncRNA
XM_004011187.3	LOC10110720(	mRNA	XR_001436532.1	lncRNA
XM_004011187.3	LOC10110720(	mRNA	XR_001436532.1	lncRNA
XM_004011187.3	LOC10110720(	mRNA	XR_001436532.1	lncRNA
XM_004011187.3	LOC10110720(	mRNA	XR_001436532.1	lncRNA
XM_012104566.2	ZKSCAN1	mRNA	TCONS_00022613	lncRNA
XM_012104566.2	ZKSCAN1	mRNA	TCONS_00022613	lncRNA
XM_012104566.2	ZKSCAN1	mRNA	TCONS_00022613	lncRNA

XM_012104566.2	ZKSCAN1	mRNA	TCONS_00022613	lncRNA
XM_012104566.2	ZKSCAN1	mRNA	TCONS_00022613	lncRNA
XM_004004723.3	SPOPL	mRNA	XR_001436127.1	lncRNA
XM_004004723.3	SPOPL	mRNA	XR_001436127.1	lncRNA
XM_004004723.3	SPOPL	mRNA	XR_001436127.1	lncRNA
XM_004004723.3	SPOPL	mRNA	XR_001436127.1	lncRNA
XM_004004723.3	SPOPL	mRNA	XR_001436127.1	lncRNA
XM_015102007.1	PPP2R3C	mRNA	XR_001436127.1	lncRNA
XM_015102007.1	PPP2R3C	mRNA	XR_001436127.1	lncRNA
XM_015102007.1	PPP2R3C	mRNA	XR_001436127.1	lncRNA
XM_015102007.1	PPP2R3C	mRNA	XR_001436127.1	lncRNA
XM_015102007.1	PPP2R3C	mRNA	XR_001436127.1	lncRNA
XM_012095345.1	CCDC82	mRNA	XR_001436127.1	lncRNA
XM_012095345.1	CCDC82	mRNA	XR_001436127.1	lncRNA
XM_012095345.1	CCDC82	mRNA	XR_001436127.1	lncRNA
XM_012095345.1	CCDC82	mRNA	XR_001436127.1	lncRNA
XM_012095345.1	CCDC82	mRNA	XR_001436127.1	lncRNA
XM_012098709.2	PML	mRNA	TCONS_00078440	lncRNA
XM_012098709.2	PML	mRNA	TCONS_00078440	lncRNA
XM_012098709.2	PML	mRNA	TCONS_00078440	lncRNA
XM_012098709.2	PML	mRNA	TCONS_00078440	lncRNA
XM_012098709.2	PML	mRNA	TCONS_00078440	lncRNA
XM_012098709.2	PML	mRNA	XR_001433755.1	lncRNA
XM_012098709.2	PML	mRNA	XR_001433755.1	lncRNA
XM_012098709.2	PML	mRNA	XR_001433755.1	lncRNA
XM_012098709.2	PML	mRNA	XR_001433755.1	lncRNA
XM_012098709.2	PML	mRNA	XR_001433755.1	lncRNA
XM_004003942.3	COL6A6	mRNA	XR_001044126.1	lncRNA
XM_004003942.3	COL6A6	mRNA	XR_001044126.1	lncRNA
XM_004003942.3	COL6A6	mRNA	XR_001044126.1	lncRNA
XM_004003942.3	COL6A6	mRNA	XR_001044126.1	lncRNA
XM_004003942.3	COL6A6	mRNA	XR_001044126.1	lncRNA
XM_004003942.3	COL6A6	mRNA	XR_001044126.1	lncRNA
XM_012103465.2	OSBPL1A	mRNA	XR_001021689.2	lncRNA
XM_012103465.2	OSBPL1A	mRNA	XR_001021689.2	lncRNA
XM_012103465.2	OSBPL1A	mRNA	XR_001021689.2	lncRNA
XM_012103465.2	OSBPL1A	mRNA	XR_001021689.2	lncRNA
XM_012103465.2	OSBPL1A	mRNA	XR_001021689.2	lncRNA
XM_012096744.2	GFM2	mRNA	XR_001436102.1	lncRNA
XM_012096744.2	GFM2	mRNA	XR_001436102.1	lncRNA
XM_012096744.2	GFM2	mRNA	XR_001436102.1	lncRNA
XM_012096744.2	GFM2	mRNA	XR_001436102.1	lncRNA
XM_012096744.2	GFM2	mRNA	XR_001436102.1	lncRNA
XM_004021989.3	DMD	mRNA	XR_001021689.2	lncRNA
XM_004021989.3	DMD	mRNA	XR_001021689.2	lncRNA
XM_004021989.3	DMD	mRNA	XR_001021689.2	lncRNA
XM_004021989.3	DMD	mRNA	XR_001021689.2	lncRNA
XM_004021989.3	DMD	mRNA	XR_001021689.2	lncRNA
XM_012103465.2	OSBPL1A	mRNA	XR_001436102.1	lncRNA
XM_012103465.2	OSBPL1A	mRNA	XR_001436102.1	lncRNA
XM_012103465.2	OSBPL1A	mRNA	XR_001436102.1	lncRNA
XM_012103465.2	OSBPL1A	mRNA	XR_001436102.1	lncRNA
XM_012103465.2	OSBPL1A	mRNA	XR_001436102.1	lncRNA
XM_012099693.2	EOGT	mRNA	XR_001436102.1	lncRNA

XM_012099693.2	EOGT	mRNA	XR_001436102.1	lncRNA
XM_012099693.2	EOGT	mRNA	XR_001436102.1	lncRNA
XM_012099693.2	EOGT	mRNA	XR_001436102.1	lncRNA
XM_012099693.2	EOGT	mRNA	XR_001436102.1	lncRNA
XM_004002018.3	OMA1	mRNA	XR_001436102.1	lncRNA
XM_004002018.3	OMA1	mRNA	XR_001436102.1	lncRNA
XM_004002018.3	OMA1	mRNA	XR_001436102.1	lncRNA
XM_004002018.3	OMA1	mRNA	XR_001436102.1	lncRNA
XM_004002018.3	OMA1	mRNA	XR_001436102.1	lncRNA
XM_015103387.1	C22H10orf76	mRNA	XR_001436102.1	lncRNA
XM_015103387.1	C22H10orf76	mRNA	XR_001436102.1	lncRNA
XM_015103387.1	C22H10orf76	mRNA	XR_001436102.1	lncRNA
XM_015103387.1	C22H10orf76	mRNA	XR_001436102.1	lncRNA
XM_015103387.1	C22H10orf76	mRNA	XR_001436102.1	lncRNA
XM_012105690.2	WDR17	mRNA	XR_001024743.2	lncRNA
XM_012105690.2	WDR17	mRNA	XR_001024743.2	lncRNA
XM_012105690.2	WDR17	mRNA	XR_001024743.2	lncRNA
XM_012105690.2	WDR17	mRNA	XR_001024743.2	lncRNA
XM_012105690.2	WDR17	mRNA	XR_001024743.2	lncRNA
XM_012182435.2	C1H1orf43	mRNA	XR_001041211.2	lncRNA
XM_012182435.2	C1H1orf43	mRNA	XR_001041211.2	lncRNA
XM_012182435.2	C1H1orf43	mRNA	XR_001041211.2	lncRNA
XM_012182435.2	C1H1orf43	mRNA	XR_001041211.2	lncRNA
XM_012182435.2	C1H1orf43	mRNA	XR_001041211.2	lncRNA
XM_012107644.2	FAM199X	mRNA	XR_001041211.2	lncRNA
XM_012107644.2	FAM199X	mRNA	XR_001041211.2	lncRNA
XM_012107644.2	FAM199X	mRNA	XR_001041211.2	lncRNA
XM_012107644.2	FAM199X	mRNA	XR_001041211.2	lncRNA
XM_012107644.2	FAM199X	mRNA	XR_001041211.2	lncRNA
XM_015104327.1	LOC10111542	mRNA	XR_001033284.2	lncRNA
XM_015104327.1	LOC10111542	mRNA	XR_001033284.2	lncRNA
XM_015104327.1	LOC10111542	mRNA	XR_001033284.2	lncRNA
XM_015104327.1	LOC10111542	mRNA	XR_001033284.2	lncRNA
XM_015104327.1	LOC10111542	mRNA	XR_001033284.2	lncRNA
XM_004004348.2	RFX3	mRNA	XR_001033284.2	lncRNA
XM_004004348.2	RFX3	mRNA	XR_001033284.2	lncRNA
XM_004004348.2	RFX3	mRNA	XR_001033284.2	lncRNA
XM_004004348.2	RFX3	mRNA	XR_001033284.2	lncRNA
XM_004004348.2	RFX3	mRNA	XR_001033284.2	lncRNA
XM_015096573.1	SOWAHB	mRNA	XR_001025491.1	lncRNA
XM_015096573.1	SOWAHB	mRNA	XR_001025491.1	lncRNA
XM_015096573.1	SOWAHB	mRNA	XR_001025491.1	lncRNA
XM_015096573.1	SOWAHB	mRNA	XR_001025491.1	lncRNA
XM_015096573.1	SOWAHB	mRNA	XR_001025491.1	lncRNA
XM_015096573.1	SOWAHB	mRNA	XR_001025491.1	lncRNA
XM_012174394.2	RAPGEF3	mRNA	XR_001024743.2	lncRNA
XM_012174394.2	RAPGEF3	mRNA	XR_001024743.2	lncRNA
XM_012174394.2	RAPGEF3	mRNA	XR_001024743.2	lncRNA
XM_012174394.2	RAPGEF3	mRNA	XR_001024743.2	lncRNA
XM_012174394.2	RAPGEF3	mRNA	XR_001024743.2	lncRNA
XM_012178306.2	FAM193B	mRNA	XR_001024743.2	lncRNA
XM_012178306.2	FAM193B	mRNA	XR_001024743.2	lncRNA
XM_012178306.2	FAM193B	mRNA	XR_001024743.2	lncRNA
XM_012178306.2	FAM193B	mRNA	XR_001024743.2	lncRNA

XM_012178306.2	FAM193B	mRNA	XR_001024743.2	lncRNA
XM_015094303.1	ACTR2	mRNA	XR_001434203.1	lncRNA
XM_015094303.1	ACTR2	mRNA	XR_001434203.1	lncRNA
XM_015094303.1	ACTR2	mRNA	XR_001434203.1	lncRNA
XM_015094303.1	ACTR2	mRNA	XR_001434203.1	lncRNA
XM_015094303.1	ACTR2	mRNA	XR_001434203.1	lncRNA
XM_012173958.2	OSBPL8	mRNA	XR_001434203.1	lncRNA
XM_012173958.2	OSBPL8	mRNA	XR_001434203.1	lncRNA
XM_012173958.2	OSBPL8	mRNA	XR_001434203.1	lncRNA
XM_012173958.2	OSBPL8	mRNA	XR_001434203.1	lncRNA
XM_012173958.2	OSBPL8	mRNA	XR_001434203.1	lncRNA
XM_004014443.3	RAE1	mRNA	XR_001436817.1	lncRNA
XM_004014443.3	RAE1	mRNA	XR_001436817.1	lncRNA
XM_004014443.3	RAE1	mRNA	XR_001436817.1	lncRNA
XM_004014443.3	RAE1	mRNA	XR_001436817.1	lncRNA
XM_004014443.3	RAE1	mRNA	XR_001436817.1	lncRNA
XM_012184629.2	USPL1	mRNA	XR_001436817.1	lncRNA
XM_012184629.2	USPL1	mRNA	XR_001436817.1	lncRNA
XM_012184629.2	USPL1	mRNA	XR_001436817.1	lncRNA
XM_012184629.2	USPL1	mRNA	XR_001436817.1	lncRNA
XM_012184629.2	USPL1	mRNA	XR_001436817.1	lncRNA
XM_012165515.2	ATAL1B	mRNA	XR_001433755.1	lncRNA
XM_012165515.2	ATAL1B	mRNA	XR_001433755.1	lncRNA
XM_012165515.2	ATAL1B	mRNA	XR_001433755.1	lncRNA
XM_012165515.2	ATAL1B	mRNA	XR_001433755.1	lncRNA
XM_012165515.2	ATAL1B	mRNA	XR_001433755.1	lncRNA
XM_015092486.1	PIGZ	mRNA	TCONS_00078440	lncRNA
XM_015092486.1	PIGZ	mRNA	TCONS_00078440	lncRNA
XM_015092486.1	PIGZ	mRNA	TCONS_00078440	lncRNA
XM_015092486.1	PIGZ	mRNA	TCONS_00078440	lncRNA
XM_015092486.1	PIGZ	mRNA	TCONS_00078440	lncRNA
XM_012101211.2	MBNL1	mRNA	XR_001434202.1	lncRNA
XM_012101211.2	MBNL1	mRNA	XR_001434202.1	lncRNA
XM_012101211.2	MBNL1	mRNA	XR_001434202.1	lncRNA
XM_012101211.2	MBNL1	mRNA	XR_001434202.1	lncRNA
XM_012101211.2	MBNL1	mRNA	XR_001434202.1	lncRNA
XM_012101211.2	MBNL1	mRNA	XR_001434202.1	lncRNA
XM_012101211.2	MBNL1	mRNA	XR_001034847.2	lncRNA
XM_012101211.2	MBNL1	mRNA	XR_001034847.2	lncRNA
XM_012101211.2	MBNL1	mRNA	XR_001034847.2	lncRNA
XM_012101211.2	MBNL1	mRNA	XR_001034847.2	lncRNA
XM_012101211.2	MBNL1	mRNA	XR_001034847.2	lncRNA
XM_012101211.2	MBNL1	mRNA	XR_001034847.2	lncRNA
XM_012134956.1	STK39	mRNA	TCONS_00022613	lncRNA
XM_012134956.1	STK39	mRNA	TCONS_00022613	lncRNA
XM_012134956.1	STK39	mRNA	TCONS_00022613	lncRNA
XM_012134956.1	STK39	mRNA	TCONS_00022613	lncRNA
XM_012134956.1	STK39	mRNA	TCONS_00022613	lncRNA
XM_012102512.2	HEYL	mRNA	TCONS_00022613	lncRNA
XM_012102512.2	HEYL	mRNA	TCONS_00022613	lncRNA
XM_012102512.2	HEYL	mRNA	TCONS_00022613	lncRNA
XM_012102512.2	HEYL	mRNA	TCONS_00022613	lncRNA
XM_012102512.2	HEYL	mRNA	TCONS_00022613	lncRNA
XM_015092063.1	FUBP1	mRNA	TCONS_00022613	lncRNA

XM_015092063.1	FUBP1	mRNA	TCONS_00022613	lncRNA
XM_015092063.1	FUBP1	mRNA	TCONS_00022613	lncRNA
XM_015092063.1	FUBP1	mRNA	TCONS_00022613	lncRNA
XM_015092063.1	FUBP1	mRNA	TCONS_00022613	lncRNA
XM_004020074.3	PIK3AP1	mRNA	XR_001436102.1	lncRNA
XM_004020074.3	PIK3AP1	mRNA	XR_001436102.1	lncRNA
XM_004020074.3	PIK3AP1	mRNA	XR_001436102.1	lncRNA
XM_004020074.3	PIK3AP1	mRNA	XR_001436102.1	lncRNA
XM_004020074.3	PIK3AP1	mRNA	XR_001436102.1	lncRNA
XM_004020074.3	PIK3AP1	mRNA	XR_001436102.1	lncRNA
XM_012186944.1	DSTYK	mRNA	XR_001024743.2	lncRNA
XM_012186944.1	DSTYK	mRNA	XR_001024743.2	lncRNA
XM_012186944.1	DSTYK	mRNA	XR_001024743.2	lncRNA
XM_012186944.1	DSTYK	mRNA	XR_001024743.2	lncRNA
XM_012186944.1	DSTYK	mRNA	XR_001024743.2	lncRNA
XM_012106834.1	OPHN1	mRNA	XR_001436931.1	lncRNA
XM_012106834.1	OPHN1	mRNA	XR_001436931.1	lncRNA
XM_012106834.1	OPHN1	mRNA	XR_001436931.1	lncRNA
XM_012106834.1	OPHN1	mRNA	XR_001436931.1	lncRNA
XM_015099539.1	KIN	mRNA	TCONS_00054422	lncRNA
XM_015099539.1	KIN	mRNA	TCONS_00054422	lncRNA
XM_015099539.1	KIN	mRNA	TCONS_00054422	lncRNA
XM_015099539.1	KIN	mRNA	TCONS_00054422	lncRNA
XM_012178924.2	PHTF1	mRNA	TCONS_00075051	lncRNA
XM_012178924.2	PHTF1	mRNA	TCONS_00075051	lncRNA
XM_012178924.2	PHTF1	mRNA	TCONS_00075051	lncRNA
XM_012178924.2	PHTF1	mRNA	TCONS_00075051	lncRNA
XM_012178924.2	PHTF1	mRNA	XR_001436931.1	lncRNA
XM_012178924.2	PHTF1	mRNA	XR_001436931.1	lncRNA
XM_012178924.2	PHTF1	mRNA	XR_001436931.1	lncRNA
XM_012178924.2	PHTF1	mRNA	XR_001436931.1	lncRNA
XM_012182435.2	C1H1orf43	mRNA	XR_001024743.2	lncRNA
XM_012182435.2	C1H1orf43	mRNA	XR_001024743.2	lncRNA
XM_012182435.2	C1H1orf43	mRNA	XR_001024743.2	lncRNA
XM_012182435.2	C1H1orf43	mRNA	XR_001024743.2	lncRNA
XM_012100690.1	KLC4	mRNA	TCONS_00078440	lncRNA
XM_012100690.1	KLC4	mRNA	TCONS_00078440	lncRNA
XM_012100690.1	KLC4	mRNA	TCONS_00078440	lncRNA
XM_012100690.1	KLC4	mRNA	TCONS_00078440	lncRNA
XM_012100690.1	KLC4	mRNA	TCONS_00078440	lncRNA
XM_012095401.2	ATM	mRNA	XR_001044126.1	lncRNA
XM_012095401.2	ATM	mRNA	XR_001044126.1	lncRNA
XM_012095401.2	ATM	mRNA	XR_001044126.1	lncRNA
XM_012095401.2	ATM	mRNA	XR_001044126.1	lncRNA
XM_012095401.2	ATM	mRNA	XR_001044126.1	lncRNA
XM_012186029.1	FBXL20	mRNA	XR_001044126.1	lncRNA
XM_012186029.1	FBXL20	mRNA	XR_001044126.1	lncRNA
XM_012186029.1	FBXL20	mRNA	XR_001044126.1	lncRNA
XM_012186029.1	FBXL20	mRNA	XR_001044126.1	lncRNA
XM_012186029.1	FBXL20	mRNA	XR_001044126.1	lncRNA
XM_012094704.2	LOC10560198[illegible]	mRNA	XR_001436127.1	lncRNA
XM_012094704.2	LOC10560198[illegible]	mRNA	XR_001436127.1	lncRNA
XM_012094704.2	LOC10560198[illegible]	mRNA	XR_001436127.1	lncRNA
XM_012094704.2	LOC10560198[illegible]	mRNA	XR_001436127.1	lncRNA

XM_012094704.2	LOC10560198{illegible}	mRNA	XR_001436127.1	lncRNA
XM_015095425.1	CUL1	mRNA	XR_001025491.1	lncRNA
XM_015095425.1	CUL1	mRNA	XR_001025491.1	lncRNA
XM_015095425.1	CUL1	mRNA	XR_001025491.1	lncRNA
XM_015095425.1	CUL1	mRNA	XR_001025491.1	lncRNA
XM_015095425.1	CUL1	mRNA	XR_001025491.1	lncRNA
XM_004004866.2	METTL21A	mRNA	XR_001436127.1	lncRNA
XM_004004866.2	METTL21A	mRNA	XR_001436127.1	lncRNA
XM_004004866.2	METTL21A	mRNA	XR_001436127.1	lncRNA
XM_004004866.2	METTL21A	mRNA	XR_001436127.1	lncRNA
XM_004004866.2	METTL21A	mRNA	XR_001436127.1	lncRNA
XM_015092119.1	MTF2	mRNA	XR_001025491.1	lncRNA
XM_015092119.1	MTF2	mRNA	XR_001025491.1	lncRNA
XM_015092119.1	MTF2	mRNA	XR_001025491.1	lncRNA
XM_015092119.1	MTF2	mRNA	XR_001025491.1	lncRNA
XM_015092119.1	MTF2	mRNA	XR_001025491.1	lncRNA
XM_004022347.2	TMEM255A	mRNA	XR_001024290.1	lncRNA
XM_004022347.2	TMEM255A	mRNA	XR_001024290.1	lncRNA
XM_004022347.2	TMEM255A	mRNA	XR_001024290.1	lncRNA
XM_004022347.2	TMEM255A	mRNA	XR_001024290.1	lncRNA
XM_004022347.2	TMEM255A	mRNA	XR_001024290.1	lncRNA
XM_012104225.2	DCUN1D3	mRNA	XR_001044126.1	lncRNA
XM_012104225.2	DCUN1D3	mRNA	XR_001044126.1	lncRNA
XM_012104225.2	DCUN1D3	mRNA	XR_001044126.1	lncRNA
XM_012104225.2	DCUN1D3	mRNA	XR_001044126.1	lncRNA
XM_012104225.2	DCUN1D3	mRNA	XR_001044126.1	lncRNA
XM_015095425.1	CUL1	mRNA	XR_001024290.1	lncRNA
XM_015095425.1	CUL1	mRNA	XR_001024290.1	lncRNA
XM_015095425.1	CUL1	mRNA	XR_001024290.1	lncRNA
XM_015095425.1	CUL1	mRNA	XR_001024290.1	lncRNA
XM_015095425.1	CUL1	mRNA	XR_001024290.1	lncRNA
XM_015100879.1	ARHGAP42	mRNA	XR_001434761.1	lncRNA
XM_015100879.1	ARHGAP42	mRNA	XR_001434761.1	lncRNA
XM_015100879.1	ARHGAP42	mRNA	XR_001434761.1	lncRNA
XM_015100879.1	ARHGAP42	mRNA	XR_001434761.1	lncRNA
XM_015100879.1	ARHGAP42	mRNA	XR_001434761.1	lncRNA
XM_012184946.1	LIG4	mRNA	XR_001434761.1	lncRNA
XM_012184946.1	LIG4	mRNA	XR_001434761.1	lncRNA
XM_012184946.1	LIG4	mRNA	XR_001434761.1	lncRNA
XM_012184946.1	LIG4	mRNA	XR_001434761.1	lncRNA
XM_012184946.1	LIG4	mRNA	XR_001434761.1	lncRNA
XM_015103591.1	KIAA1328	mRNA	XR_001434761.1	lncRNA
XM_015103591.1	KIAA1328	mRNA	XR_001434761.1	lncRNA
XM_015103591.1	KIAA1328	mRNA	XR_001434761.1	lncRNA
XM_015103591.1	KIAA1328	mRNA	XR_001434761.1	lncRNA
XM_015103591.1	KIAA1328	mRNA	XR_001434761.1	lncRNA
XM_012176647.2	NAPEPLD	mRNA	XR_001434892.1	lncRNA
XM_012176647.2	NAPEPLD	mRNA	XR_001434892.1	lncRNA
XM_012176647.2	NAPEPLD	mRNA	XR_001434892.1	lncRNA
XM_012176647.2	NAPEPLD	mRNA	XR_001434892.1	lncRNA
XM_012176647.2	NAPEPLD	mRNA	XR_001434892.1	lncRNA
XM_015099539.1	KIN	mRNA	XR_001434892.1	lncRNA
XM_015099539.1	KIN	mRNA	XR_001434892.1	lncRNA
XM_015099539.1	KIN	mRNA	XR_001434892.1	lncRNA

XM_015099539.1	KIN	mRNA	XR_001434892.1	lncRNA
XM_015099539.1	KIN	mRNA	XR_001434892.1	lncRNA
XM_012117587.2	KIF27	mRNA	XR_001434892.1	lncRNA
XM_012117587.2	KIF27	mRNA	XR_001434892.1	lncRNA
XM_012117587.2	KIF27	mRNA	XR_001434892.1	lncRNA
XM_012117587.2	KIF27	mRNA	XR_001434892.1	lncRNA
XM_012117587.2	KIF27	mRNA	XR_001434892.1	lncRNA
XM_012182353.2	LRRFIP1	mRNA	XR_001033284.2	lncRNA
XM_012182353.2	LRRFIP1	mRNA	XR_001033284.2	lncRNA
XM_012182353.2	LRRFIP1	mRNA	XR_001033284.2	lncRNA
XM_012182353.2	LRRFIP1	mRNA	XR_001033284.2	lncRNA
XM_012182353.2	LRRFIP1	mRNA	XR_001033284.2	lncRNA
XM_012143452.2	NRP2	mRNA	XR_001033284.2	lncRNA
XM_012143452.2	NRP2	mRNA	XR_001033284.2	lncRNA
XM_012143452.2	NRP2	mRNA	XR_001033284.2	lncRNA
XM_012143452.2	NRP2	mRNA	XR_001033284.2	lncRNA
XM_012143452.2	NRP2	mRNA	XR_001033284.2	lncRNA
XM_012140422.2	CLASP1	mRNA	TCONS_00022613	lncRNA
XM_012140422.2	CLASP1	mRNA	TCONS_00022613	lncRNA
XM_012140422.2	CLASP1	mRNA	TCONS_00022613	lncRNA
XM_012140422.2	CLASP1	mRNA	TCONS_00022613	lncRNA
XM_012140422.2	CLASP1	mRNA	TCONS_00022613	lncRNA
XM_015101001.1	RRAS2	mRNA	TCONS_00022613	lncRNA
XM_015101001.1	RRAS2	mRNA	TCONS_00022613	lncRNA
XM_015101001.1	RRAS2	mRNA	TCONS_00022613	lncRNA
XM_015101001.1	RRAS2	mRNA	TCONS_00022613	lncRNA
XM_015101001.1	RRAS2	mRNA	TCONS_00022613	lncRNA
XM_012110487.2	UBQLN1	mRNA	TCONS_00022613	lncRNA
XM_012110487.2	UBQLN1	mRNA	TCONS_00022613	lncRNA
XM_012110487.2	UBQLN1	mRNA	TCONS_00022613	lncRNA
XM_012110487.2	UBQLN1	mRNA	TCONS_00022613	lncRNA
XM_012110487.2	UBQLN1	mRNA	TCONS_00022613	lncRNA
XM_015092883.1	PADI1	mRNA	TCONS_00022613	lncRNA
XM_015092883.1	PADI1	mRNA	TCONS_00022613	lncRNA
XM_015092883.1	PADI1	mRNA	TCONS_00022613	lncRNA
XM_015092883.1	PADI1	mRNA	TCONS_00022613	lncRNA
XM_015092883.1	PADI1	mRNA	TCONS_00022613	lncRNA
XM_012176217.2	EVI5	mRNA	TCONS_00022613	lncRNA
XM_012176217.2	EVI5	mRNA	TCONS_00022613	lncRNA
XM_012176217.2	EVI5	mRNA	TCONS_00022613	lncRNA
XM_012176217.2	EVI5	mRNA	TCONS_00022613	lncRNA
XM_012176217.2	EVI5	mRNA	TCONS_00022613	lncRNA
XM_012181234.2	NDUFAF1	mRNA	XR_001021689.2	lncRNA
XM_012181234.2	NDUFAF1	mRNA	XR_001021689.2	lncRNA
XM_012181234.2	NDUFAF1	mRNA	XR_001021689.2	lncRNA
XM_012181234.2	NDUFAF1	mRNA	XR_001021689.2	lncRNA
XM_012181234.2	NDUFAF1	mRNA	XR_001021689.2	lncRNA
XM_012179693.1	PIGY	mRNA	XR_001021689.2	lncRNA
XM_012179693.1	PIGY	mRNA	XR_001021689.2	lncRNA
XM_012179693.1	PIGY	mRNA	XR_001021689.2	lncRNA
XM_012179693.1	PIGY	mRNA	XR_001021689.2	lncRNA
XM_012179693.1	PIGY	mRNA	XR_001021689.2	lncRNA
XM_012188283.2	CCDC7	mRNA	XR_001436102.1	lncRNA
XM_012188283.2	CCDC7	mRNA	XR_001436102.1	lncRNA

XM_012188283.2	CCDC7	mRNA	XR_001436102.1	lncRNA
XM_012188283.2	CCDC7	mRNA	XR_001436102.1	lncRNA
XM_012188283.2	CCDC7	mRNA	XR_001436102.1	lncRNA
XM_012104594.2	ZNF789	mRNA	XR_001436102.1	lncRNA
XM_012104594.2	ZNF789	mRNA	XR_001436102.1	lncRNA
XM_012104594.2	ZNF789	mRNA	XR_001436102.1	lncRNA
XM_012104594.2	ZNF789	mRNA	XR_001436102.1	lncRNA
XM_012104594.2	ZNF789	mRNA	XR_001436102.1	lncRNA
XM_004004723.3	SPOPL	mRNA	XR_001436102.1	lncRNA
XM_004004723.3	SPOPL	mRNA	XR_001436102.1	lncRNA
XM_004004723.3	SPOPL	mRNA	XR_001436102.1	lncRNA
XM_004004723.3	SPOPL	mRNA	XR_001436102.1	lncRNA
XM_004004723.3	SPOPL	mRNA	XR_001436102.1	lncRNA
XM_012186914.2	ZC3H11A	mRNA	XR_001021689.2	lncRNA
XM_012186914.2	ZC3H11A	mRNA	XR_001021689.2	lncRNA
XM_012186914.2	ZC3H11A	mRNA	XR_001021689.2	lncRNA
XM_012186914.2	ZC3H11A	mRNA	XR_001021689.2	lncRNA
XM_012186914.2	ZC3H11A	mRNA	XR_001021689.2	lncRNA
XM_012178847.2	CNOT8	mRNA	XR_001436102.1	lncRNA
XM_012178847.2	CNOT8	mRNA	XR_001436102.1	lncRNA
XM_012178847.2	CNOT8	mRNA	XR_001436102.1	lncRNA
XM_012178847.2	CNOT8	mRNA	XR_001436102.1	lncRNA
XM_012178847.2	CNOT8	mRNA	XR_001436102.1	lncRNA
XM_004004723.3	SPOPL	mRNA	XR_001021689.2	lncRNA
XM_004004723.3	SPOPL	mRNA	XR_001021689.2	lncRNA
XM_004004723.3	SPOPL	mRNA	XR_001021689.2	lncRNA
XM_004004723.3	SPOPL	mRNA	XR_001021689.2	lncRNA
XM_004004723.3	SPOPL	mRNA	XR_001021689.2	lncRNA
XM_012188283.2	CCDC7	mRNA	XR_001021689.2	lncRNA
XM_012188283.2	CCDC7	mRNA	XR_001021689.2	lncRNA
XM_012188283.2	CCDC7	mRNA	XR_001021689.2	lncRNA
XM_012188283.2	CCDC7	mRNA	XR_001021689.2	lncRNA
XM_012188283.2	CCDC7	mRNA	XR_001021689.2	lncRNA
XM_012102549.2	CEP70	mRNA	XR_001021689.2	lncRNA
XM_012102549.2	CEP70	mRNA	XR_001021689.2	lncRNA
XM_012102549.2	CEP70	mRNA	XR_001021689.2	lncRNA
XM_012102549.2	CEP70	mRNA	XR_001021689.2	lncRNA
XM_012102549.2	CEP70	mRNA	XR_001021689.2	lncRNA
XM_012099258.2	LOC10112214(	mRNA	XR_001433755.1	lncRNA
XM_012099258.2	LOC10112214(	mRNA	XR_001433755.1	lncRNA
XM_012099258.2	LOC10112214(	mRNA	XR_001433755.1	lncRNA
XM_012099258.2	LOC10112214(	mRNA	XR_001433755.1	lncRNA
XM_012099258.2	LOC10112214(	mRNA	XR_001433755.1	lncRNA
XM_012179696.2	LOC10111286	mRNA	TCONS_00078440	lncRNA
XM_012179696.2	LOC10111286	mRNA	TCONS_00078440	lncRNA
XM_012179696.2	LOC10111286	mRNA	TCONS_00078440	lncRNA
XM_012179696.2	LOC10111286	mRNA	TCONS_00078440	lncRNA
XM_012179696.2	LOC10111286	mRNA	TCONS_00078440	lncRNA
XM_015100438.1	CYLD	mRNA	TCONS_00075453	lncRNA
XM_015100438.1	CYLD	mRNA	TCONS_00075453	lncRNA
XM_015100438.1	CYLD	mRNA	TCONS_00075453	lncRNA
XM_015100438.1	CYLD	mRNA	TCONS_00075453	lncRNA
XM_015100438.1	CYLD	mRNA	TCONS_00075453	lncRNA
XM_015092063.1	FUBP1	mRNA	TCONS_00075453	lncRNA

XM_015092063.1	FUBP1	mRNA	TCONS_00075453	lncRNA
XM_015092063.1	FUBP1	mRNA	TCONS_00075453	lncRNA
XM_015092063.1	FUBP1	mRNA	TCONS_00075453	lncRNA
XM_015092063.1	FUBP1	mRNA	TCONS_00075453	lncRNA
XM_015098145.1	HSPH1	mRNA	TCONS_00022613	lncRNA
XM_015098145.1	HSPH1	mRNA	TCONS_00022613	lncRNA
XM_015098145.1	HSPH1	mRNA	TCONS_00022613	lncRNA
XM_015098145.1	HSPH1	mRNA	TCONS_00022613	lncRNA
XM_012101717.1	PAK1	mRNA	TCONS_00075051	lncRNA
XM_012101717.1	PAK1	mRNA	TCONS_00075051	lncRNA
XM_012101717.1	PAK1	mRNA	TCONS_00075051	lncRNA
XM_012101717.1	PAK1	mRNA	TCONS_00075051	lncRNA
XM_004004723.3	SPOPL	mRNA	TCONS_00054422	lncRNA
XM_004004723.3	SPOPL	mRNA	TCONS_00054422	lncRNA
XM_004004723.3	SPOPL	mRNA	TCONS_00054422	lncRNA
XM_004004723.3	SPOPL	mRNA	TCONS_00054422	lncRNA
XM_012178847.2	CNOT8	mRNA	TCONS_00075051	lncRNA
XM_012178847.2	CNOT8	mRNA	TCONS_00075051	lncRNA
XM_012178847.2	CNOT8	mRNA	TCONS_00075051	lncRNA
XM_012178847.2	CNOT8	mRNA	TCONS_00075051	lncRNA
XM_012094768.2	ZMYM1	mRNA	XR_001434202.1	lncRNA
XM_012094768.2	ZMYM1	mRNA	XR_001434202.1	lncRNA
XM_012094768.2	ZMYM1	mRNA	XR_001434202.1	lncRNA
XM_012094768.2	ZMYM1	mRNA	XR_001434202.1	lncRNA
XM_012094768.2	ZMYM1	mRNA	XR_001434202.1	lncRNA
XM_012094768.2	ZMYM1	mRNA	XR_001034847.2	lncRNA
XM_012094768.2	ZMYM1	mRNA	XR_001034847.2	lncRNA
XM_012094768.2	ZMYM1	mRNA	XR_001034847.2	lncRNA
XM_012094768.2	ZMYM1	mRNA	XR_001034847.2	lncRNA
XM_012094768.2	ZMYM1	mRNA	XR_001034847.2	lncRNA
XM_012095345.1	CCDC82	mRNA	XR_001434202.1	lncRNA
XM_012095345.1	CCDC82	mRNA	XR_001434202.1	lncRNA
XM_012095345.1	CCDC82	mRNA	XR_001434202.1	lncRNA
XM_012095345.1	CCDC82	mRNA	XR_001434202.1	lncRNA
XM_012095345.1	CCDC82	mRNA	XR_001434202.1	lncRNA
XM_004004723.3	SPOPL	mRNA	XR_001034847.2	lncRNA
XM_004004723.3	SPOPL	mRNA	XR_001034847.2	lncRNA
XM_004004723.3	SPOPL	mRNA	XR_001034847.2	lncRNA
XM_004004723.3	SPOPL	mRNA	XR_001034847.2	lncRNA
XM_004004723.3	SPOPL	mRNA	XR_001034847.2	lncRNA
XM_004004723.3	SPOPL	mRNA	XR_001434202.1	lncRNA
XM_004004723.3	SPOPL	mRNA	XR_001434202.1	lncRNA
XM_004004723.3	SPOPL	mRNA	XR_001434202.1	lncRNA
XM_004004723.3	SPOPL	mRNA	XR_001434202.1	lncRNA
XM_004004723.3	SPOPL	mRNA	XR_001434202.1	lncRNA
XM_012095771.2	CTR9	mRNA	XR_001034847.2	lncRNA
XM_012095771.2	CTR9	mRNA	XR_001034847.2	lncRNA
XM_012095771.2	CTR9	mRNA	XR_001034847.2	lncRNA
XM_012095771.2	CTR9	mRNA	XR_001034847.2	lncRNA
XM_012095771.2	CTR9	mRNA	XR_001034847.2	lncRNA
XM_012095345.1	CCDC82	mRNA	XR_001034847.2	lncRNA
XM_012095345.1	CCDC82	mRNA	XR_001034847.2	lncRNA
XM_012095345.1	CCDC82	mRNA	XR_001034847.2	lncRNA
XM_012095345.1	CCDC82	mRNA	XR_001034847.2	lncRNA

XM_012095345.1	CCDC82	mRNA	XR_001034847.2	lncRNA
XM_012095771.2	CTR9	mRNA	XR_001434202.1	lncRNA
XM_012095771.2	CTR9	mRNA	XR_001434202.1	lncRNA
XM_012095771.2	CTR9	mRNA	XR_001434202.1	lncRNA
XM_012095771.2	CTR9	mRNA	XR_001434202.1	lncRNA
XM_012095771.2	CTR9	mRNA	XR_001434202.1	lncRNA
XM_012152455.2	LUZP1	mRNA	TCONS_00078440	lncRNA
XM_012152455.2	LUZP1	mRNA	TCONS_00078440	lncRNA
XM_012152455.2	LUZP1	mRNA	TCONS_00078440	lncRNA
XM_012152455.2	LUZP1	mRNA	TCONS_00078440	lncRNA
XM_012152455.2	LUZP1	mRNA	TCONS_00078440	lncRNA
XM_012178306.2	FAM193B	mRNA	XR_001433755.1	lncRNA
XM_012178306.2	FAM193B	mRNA	XR_001433755.1	lncRNA
XM_012178306.2	FAM193B	mRNA	XR_001433755.1	lncRNA
XM_012178306.2	FAM193B	mRNA	XR_001433755.1	lncRNA
XM_012178306.2	FAM193B	mRNA	XR_001433755.1	lncRNA
XM_004010577.2	CGNL1	mRNA	TCONS_00078440	lncRNA
XM_004010577.2	CGNL1	mRNA	TCONS_00078440	lncRNA
XM_004010577.2	CGNL1	mRNA	TCONS_00078440	lncRNA
XM_004010577.2	CGNL1	mRNA	TCONS_00078440	lncRNA
XM_004010577.2	CGNL1	mRNA	TCONS_00078440	lncRNA
XM_012096298.1	TP53I11	mRNA	XR_001042381.2	lncRNA
XM_012096298.1	TP53I11	mRNA	XR_001042381.2	lncRNA
XM_012096298.1	TP53I11	mRNA	XR_001042381.2	lncRNA
XM_012096298.1	TP53I11	mRNA	XR_001042381.2	lncRNA
XM_012096298.1	TP53I11	mRNA	XR_001042381.2	lncRNA
XM_012176829.1	BBS9	mRNA	XR_001436127.1	lncRNA
XM_012176829.1	BBS9	mRNA	XR_001436127.1	lncRNA
XM_012176829.1	BBS9	mRNA	XR_001436127.1	lncRNA
XM_012176829.1	BBS9	mRNA	XR_001436127.1	lncRNA
XM_012176829.1	BBS9	mRNA	XR_001436127.1	lncRNA
XM_015102111.1	EIF4E3	mRNA	XR_001044126.1	lncRNA
XM_015102111.1	EIF4E3	mRNA	XR_001044126.1	lncRNA
XM_015102111.1	EIF4E3	mRNA	XR_001044126.1	lncRNA
XM_015102111.1	EIF4E3	mRNA	XR_001044126.1	lncRNA
XM_015102111.1	EIF4E3	mRNA	XR_001044126.1	lncRNA
XM_012096615.2	DLG1	mRNA	XR_001436127.1	lncRNA
XM_012096615.2	DLG1	mRNA	XR_001436127.1	lncRNA
XM_012096615.2	DLG1	mRNA	XR_001436127.1	lncRNA
XM_012096615.2	DLG1	mRNA	XR_001436127.1	lncRNA
XM_012096615.2	DLG1	mRNA	XR_001436127.1	lncRNA
XM_015094913.1	PLCM1	mRNA	XR_001044126.1	lncRNA
XM_015094913.1	PLCM1	mRNA	XR_001044126.1	lncRNA
XM_015094913.1	PLCM1	mRNA	XR_001044126.1	lncRNA
XM_015094913.1	PLCM1	mRNA	XR_001044126.1	lncRNA
XM_015094913.1	PLCM1	mRNA	XR_001044126.1	lncRNA
XM_012180554.2	TARS2	mRNA	TCONS_00022613	lncRNA
XM_012180554.2	TARS2	mRNA	TCONS_00022613	lncRNA
XM_012180554.2	TARS2	mRNA	TCONS_00022613	lncRNA
XM_012180554.2	TARS2	mRNA	TCONS_00022613	lncRNA
XM_012180554.2	TARS2	mRNA	TCONS_00022613	lncRNA
XM_015103117.1	ZBTB44	mRNA	TCONS_00022613	lncRNA
XM_015103117.1	ZBTB44	mRNA	TCONS_00022613	lncRNA
XM_015103117.1	ZBTB44	mRNA	TCONS_00022613	lncRNA

XM_015103117.1	ZBTB44	mRNA	TCONS_00022613	lncRNA
XM_015103117.1	ZBTB44	mRNA	TCONS_00022613	lncRNA
XM_012096925.2	SLC38A9	mRNA	TCONS_00022613	lncRNA
XM_012096925.2	SLC38A9	mRNA	TCONS_00022613	lncRNA
XM_012096925.2	SLC38A9	mRNA	TCONS_00022613	lncRNA
XM_012096925.2	SLC38A9	mRNA	TCONS_00022613	lncRNA
XM_012096925.2	SLC38A9	mRNA	TCONS_00022613	lncRNA
XM_015093714.1	EIF4G3	mRNA	XR_001033284.2	lncRNA
XM_015093714.1	EIF4G3	mRNA	XR_001033284.2	lncRNA
XM_015093714.1	EIF4G3	mRNA	XR_001033284.2	lncRNA
XM_015093714.1	EIF4G3	mRNA	XR_001033284.2	lncRNA
XM_015093714.1	EIF4G3	mRNA	XR_001033284.2	lncRNA
XM_012107472.1	NFYC	mRNA	XR_001020707.2	lncRNA
XM_012107472.1	NFYC	mRNA	XR_001020707.2	lncRNA
XM_012107472.1	NFYC	mRNA	XR_001020707.2	lncRNA
XM_012107472.1	NFYC	mRNA	XR_001020707.2	lncRNA
XM_012107472.1	NFYC	mRNA	XR_001020707.2	lncRNA
XM_015103401.1	CALHM2	mRNA	XR_001020707.2	lncRNA
XM_015103401.1	CALHM2	mRNA	XR_001020707.2	lncRNA
XM_015103401.1	CALHM2	mRNA	XR_001020707.2	lncRNA
XM_015103401.1	CALHM2	mRNA	XR_001020707.2	lncRNA
XM_015103401.1	CALHM2	mRNA	XR_001020707.2	lncRNA
XM_012184823.2	RBM26	mRNA	XR_001033284.2	lncRNA
XM_012184823.2	RBM26	mRNA	XR_001033284.2	lncRNA
XM_012184823.2	RBM26	mRNA	XR_001033284.2	lncRNA
XM_012184823.2	RBM26	mRNA	XR_001033284.2	lncRNA
XM_012184823.2	RBM26	mRNA	XR_001033284.2	lncRNA
XM_012185217.2	CLTC	mRNA	TCONS_00075453	lncRNA
XM_012185217.2	CLTC	mRNA	TCONS_00075453	lncRNA
XM_012185217.2	CLTC	mRNA	TCONS_00075453	lncRNA
XM_012185217.2	CLTC	mRNA	TCONS_00075453	lncRNA
XM_012185217.2	CLTC	mRNA	TCONS_00075453	lncRNA
XM_012174768.2	RAB5B	mRNA	TCONS_00075453	lncRNA
XM_012174768.2	RAB5B	mRNA	TCONS_00075453	lncRNA
XM_012174768.2	RAB5B	mRNA	TCONS_00075453	lncRNA
XM_012174768.2	RAB5B	mRNA	TCONS_00075453	lncRNA
XM_012174768.2	RAB5B	mRNA	TCONS_00075453	lncRNA
XM_012181190.2	EIF2AK4	mRNA	TCONS_00075453	lncRNA
XM_012181190.2	EIF2AK4	mRNA	TCONS_00075453	lncRNA
XM_012181190.2	EIF2AK4	mRNA	TCONS_00075453	lncRNA
XM_012181190.2	EIF2AK4	mRNA	TCONS_00075453	lncRNA
XM_012181190.2	EIF2AK4	mRNA	TCONS_00075453	lncRNA
XM_015099539.1	KIN	mRNA	XR_001024743.2	lncRNA
XM_015099539.1	KIN	mRNA	XR_001024743.2	lncRNA
XM_015099539.1	KIN	mRNA	XR_001024743.2	lncRNA
XM_015099539.1	KIN	mRNA	XR_001024743.2	lncRNA
XM_015099539.1	KIN	mRNA	XR_001041211.2	lncRNA
XM_015099539.1	KIN	mRNA	XR_001041211.2	lncRNA
XM_015099539.1	KIN	mRNA	XR_001041211.2	lncRNA
XM_015099539.1	KIN	mRNA	XR_001041211.2	lncRNA
XM_015099539.1	KIN	mRNA	XR_001041211.2	lncRNA
XM_004006434.3	PUS7L	mRNA	XR_001434203.1	lncRNA
XM_004006434.3	PUS7L	mRNA	XR_001434203.1	lncRNA
XM_004006434.3	PUS7L	mRNA	XR_001434203.1	lncRNA

XM_004006434.3	PUS7L	mRNA	XR_001434203.1	lncRNA
XM_004006434.3	PUS7L	mRNA	XR_001434203.1	lncRNA
XM_004006434.3	PUS7L	mRNA	XR_001436817.1	lncRNA
XM_004006434.3	PUS7L	mRNA	XR_001436817.1	lncRNA
XM_004006434.3	PUS7L	mRNA	XR_001436817.1	lncRNA
XM_004006434.3	PUS7L	mRNA	XR_001436817.1	lncRNA
XM_004006434.3	PUS7L	mRNA	XR_001436817.1	lncRNA
XM_012105319.2	USP54	mRNA	TCONS_00022613	lncRNA
XM_012105319.2	USP54	mRNA	TCONS_00022613	lncRNA
XM_012105319.2	USP54	mRNA	TCONS_00022613	lncRNA
XM_012105319.2	USP54	mRNA	TCONS_00022613	lncRNA
XM_012105319.2	USP54	mRNA	TCONS_00022613	lncRNA
XM_004011865.3	ENPP2	mRNA	TCONS_00022613	lncRNA
XM_004011865.3	ENPP2	mRNA	TCONS_00022613	lncRNA
XM_004011865.3	ENPP2	mRNA	TCONS_00022613	lncRNA
XM_004011865.3	ENPP2	mRNA	TCONS_00022613	lncRNA
XM_004011865.3	ENPP2	mRNA	TCONS_00022613	lncRNA
XM_012105332.2	USP54	mRNA	TCONS_00022613	lncRNA
XM_012105332.2	USP54	mRNA	TCONS_00022613	lncRNA
XM_012105332.2	USP54	mRNA	TCONS_00022613	lncRNA
XM_012105332.2	USP54	mRNA	TCONS_00022613	lncRNA
XM_012105332.2	USP54	mRNA	TCONS_00022613	lncRNA
XM_012165732.1	FAM228B	mRNA	TCONS_00022613	lncRNA
XM_012165732.1	FAM228B	mRNA	TCONS_00022613	lncRNA
XM_012165732.1	FAM228B	mRNA	TCONS_00022613	lncRNA
XM_012165732.1	FAM228B	mRNA	TCONS_00022613	lncRNA
XM_012165732.1	FAM228B	mRNA	TCONS_00022613	lncRNA
XM_012174536.2	KIF21A	mRNA	TCONS_00022613	lncRNA
XM_012174536.2	KIF21A	mRNA	TCONS_00022613	lncRNA
XM_012174536.2	KIF21A	mRNA	TCONS_00022613	lncRNA
XM_012174536.2	KIF21A	mRNA	TCONS_00022613	lncRNA
XM_012174536.2	KIF21A	mRNA	TCONS_00022613	lncRNA
XM_012184148.1	OTUL3B	mRNA	XR_001021689.2	lncRNA
XM_012184148.1	OTUL3B	mRNA	XR_001021689.2	lncRNA
XM_012184148.1	OTUL3B	mRNA	XR_001021689.2	lncRNA
XM_012184148.1	OTUL3B	mRNA	XR_001021689.2	lncRNA
XM_012184148.1	OTUL3B	mRNA	XR_001021689.2	lncRNA
XM_015098766.1	WIPF2	mRNA	XR_001021689.2	lncRNA
XM_015098766.1	WIPF2	mRNA	XR_001021689.2	lncRNA
XM_015098766.1	WIPF2	mRNA	XR_001021689.2	lncRNA
XM_015098766.1	WIPF2	mRNA	XR_001021689.2	lncRNA
XM_015098766.1	WIPF2	mRNA	XR_001021689.2	lncRNA
XM_004014209.3	CUL2	mRNA	XR_001436102.1	lncRNA
XM_004014209.3	CUL2	mRNA	XR_001436102.1	lncRNA
XM_004014209.3	CUL2	mRNA	XR_001436102.1	lncRNA
XM_004014209.3	CUL2	mRNA	XR_001436102.1	lncRNA
XM_004014209.3	CUL2	mRNA	XR_001436102.1	lncRNA
XM_004004866.2	METTL21A	mRNA	XR_001021689.2	lncRNA
XM_004004866.2	METTL21A	mRNA	XR_001021689.2	lncRNA
XM_004004866.2	METTL21A	mRNA	XR_001021689.2	lncRNA
XM_004004866.2	METTL21A	mRNA	XR_001021689.2	lncRNA
XM_004004866.2	METTL21A	mRNA	XR_001021689.2	lncRNA
XM_012170864.2	PAPOLG	mRNA	XR_001436102.1	lncRNA
XM_012170864.2	PAPOLG	mRNA	XR_001436102.1	lncRNA

XM_012170864.2	PAPOLG	mRNA	XR_001436102.1	lncRNA
XM_012170864.2	PAPOLG	mRNA	XR_001436102.1	lncRNA
XM_012170864.2	PAPOLG	mRNA	XR_001436102.1	lncRNA
XM_012105261.2	MCU	mRNA	XR_001436102.1	lncRNA
XM_012105261.2	MCU	mRNA	XR_001436102.1	lncRNA
XM_012105261.2	MCU	mRNA	XR_001436102.1	lncRNA
XM_012105261.2	MCU	mRNA	XR_001436102.1	lncRNA
XM_012105261.2	MCU	mRNA	XR_001436102.1	lncRNA
XM_004002402.3	CHD1L	mRNA	XR_001033284.2	lncRNA
XM_004002402.3	CHD1L	mRNA	XR_001033284.2	lncRNA
XM_004002402.3	CHD1L	mRNA	XR_001033284.2	lncRNA
XM_004002402.3	CHD1L	mRNA	XR_001033284.2	lncRNA
XM_012177629.2	CLCC1	mRNA	XR_001033284.2	lncRNA
XM_012177629.2	CLCC1	mRNA	XR_001033284.2	lncRNA
XM_012177629.2	CLCC1	mRNA	XR_001033284.2	lncRNA
XM_012177629.2	CLCC1	mRNA	XR_001033284.2	lncRNA
XM_012186312.2	DDX42	mRNA	XR_001434892.1	lncRNA
XM_012186312.2	DDX42	mRNA	XR_001434892.1	lncRNA
XM_012186312.2	DDX42	mRNA	XR_001434892.1	lncRNA
XM_012186312.2	DDX42	mRNA	XR_001434892.1	lncRNA
XM_012186312.2	DDX42	mRNA	XR_001434892.1	lncRNA
XM_012181234.2	NDUFAF1	mRNA	XR_001434892.1	lncRNA
XM_012181234.2	NDUFAF1	mRNA	XR_001434892.1	lncRNA
XM_012181234.2	NDUFAF1	mRNA	XR_001434892.1	lncRNA
XM_012181234.2	NDUFAF1	mRNA	XR_001434892.1	lncRNA
XM_012181234.2	NDUFAF1	mRNA	XR_001434892.1	lncRNA
XM_012178847.2	CNOT8	mRNA	XR_001434761.1	lncRNA
XM_012178847.2	CNOT8	mRNA	XR_001434761.1	lncRNA
XM_012178847.2	CNOT8	mRNA	XR_001434761.1	lncRNA
XM_012178847.2	CNOT8	mRNA	XR_001434761.1	lncRNA
XM_012178847.2	CNOT8	mRNA	XR_001434761.1	lncRNA
XM_012122817.1	RNF220	mRNA	XR_001433755.1	lncRNA
XM_012122817.1	RNF220	mRNA	XR_001433755.1	lncRNA
XM_012122817.1	RNF220	mRNA	XR_001433755.1	lncRNA
XM_012122817.1	RNF220	mRNA	XR_001433755.1	lncRNA
XM_012122817.1	RNF220	mRNA	XR_001433755.1	lncRNA
XM_015096937.1	BMF	mRNA	TCONS_00078440	lncRNA
XM_015096937.1	BMF	mRNA	TCONS_00078440	lncRNA
XM_015096937.1	BMF	mRNA	TCONS_00078440	lncRNA
XM_015096937.1	BMF	mRNA	TCONS_00078440	lncRNA
XM_015096937.1	BMF	mRNA	TCONS_00078440	lncRNA
XM_015095425.1	CUL1	mRNA	TCONS_00084305	lncRNA
XM_015095425.1	CUL1	mRNA	TCONS_00084305	lncRNA
XM_015095425.1	CUL1	mRNA	TCONS_00084305	lncRNA
XM_015095425.1	CUL1	mRNA	TCONS_00084305	lncRNA
XM_015096972.1	L2HGDH	mRNA	TCONS_00054422	lncRNA
XM_015096972.1	L2HGDH	mRNA	TCONS_00054422	lncRNA
XM_015096972.1	L2HGDH	mRNA	TCONS_00054422	lncRNA
XM_015096972.1	L2HGDH	mRNA	TCONS_00054422	lncRNA
XM_015091968.1	EPS15	mRNA	XR_001436931.1	lncRNA
XM_015091968.1	EPS15	mRNA	XR_001436931.1	lncRNA
XM_015091968.1	EPS15	mRNA	XR_001436931.1	lncRNA
XM_015091968.1	EPS15	mRNA	XR_001436931.1	lncRNA
XM_015098434.1	STXBP4	mRNA	XR_001024290.1	lncRNA

XM_015098434.1	STXBP4	mRNA	XR_001024290.1	lncRNA
XM_015098434.1	STXBP4	mRNA	XR_001024290.1	lncRNA
XM_015098434.1	STXBP4	mRNA	XR_001024290.1	lncRNA
XM_015098434.1	STXBP4	mRNA	XR_001024290.1	lncRNA
XM_012102512.2	HEYL	mRNA	XR_001025491.1	lncRNA
XM_012102512.2	HEYL	mRNA	XR_001025491.1	lncRNA
XM_012102512.2	HEYL	mRNA	XR_001025491.1	lncRNA
XM_012102512.2	HEYL	mRNA	XR_001025491.1	lncRNA
XM_012102512.2	HEYL	mRNA	XR_001025491.1	lncRNA
XM_012187546.2	ANKRD45	mRNA	XR_001044126.1	lncRNA
XM_012187546.2	ANKRD45	mRNA	XR_001044126.1	lncRNA
XM_012187546.2	ANKRD45	mRNA	XR_001044126.1	lncRNA
XM_012187546.2	ANKRD45	mRNA	XR_001044126.1	lncRNA
XM_012187546.2	ANKRD45	mRNA	XR_001044126.1	lncRNA
XM_012102512.2	HEYL	mRNA	XR_001024290.1	lncRNA
XM_012102512.2	HEYL	mRNA	XR_001024290.1	lncRNA
XM_012102512.2	HEYL	mRNA	XR_001024290.1	lncRNA
XM_012102512.2	HEYL	mRNA	XR_001024290.1	lncRNA
XM_012102512.2	HEYL	mRNA	XR_001024290.1	lncRNA
XM_012107808.1	NFYC	mRNA	XR_001025491.1	lncRNA
XM_012107808.1	NFYC	mRNA	XR_001025491.1	lncRNA
XM_012107808.1	NFYC	mRNA	XR_001025491.1	lncRNA
XM_012107808.1	NFYC	mRNA	XR_001025491.1	lncRNA
XM_012107808.1	NFYC	mRNA	XR_001025491.1	lncRNA
XM_015098434.1	STXBP4	mRNA	XR_001025491.1	lncRNA
XM_015098434.1	STXBP4	mRNA	XR_001025491.1	lncRNA
XM_015098434.1	STXBP4	mRNA	XR_001025491.1	lncRNA
XM_015098434.1	STXBP4	mRNA	XR_001025491.1	lncRNA
XM_015098434.1	STXBP4	mRNA	XR_001025491.1	lncRNA
XM_012105396.2	C25H10orf11	mRNA	XR_001025491.1	lncRNA
XM_012105396.2	C25H10orf11	mRNA	XR_001025491.1	lncRNA
XM_012105396.2	C25H10orf11	mRNA	XR_001025491.1	lncRNA
XM_012105396.2	C25H10orf11	mRNA	XR_001025491.1	lncRNA
XM_012105396.2	C25H10orf11	mRNA	XR_001025491.1	lncRNA
XM_012112310.2	C2H9orf91	mRNA	TCONS_00075453	lncRNA
XM_012112310.2	C2H9orf91	mRNA	TCONS_00075453	lncRNA
XM_012112310.2	C2H9orf91	mRNA	TCONS_00075453	lncRNA
XM_012112310.2	C2H9orf91	mRNA	TCONS_00075453	lncRNA
XM_012112310.2	C2H9orf91	mRNA	TCONS_00075453	lncRNA
XM_015103117.1	ZBTB44	mRNA	TCONS_00075453	lncRNA
XM_015103117.1	ZBTB44	mRNA	TCONS_00075453	lncRNA
XM_015103117.1	ZBTB44	mRNA	TCONS_00075453	lncRNA
XM_015103117.1	ZBTB44	mRNA	TCONS_00075453	lncRNA
XM_015103117.1	ZBTB44	mRNA	TCONS_00075453	lncRNA
XM_004005653.3	ZBTB26	mRNA	TCONS_00075453	lncRNA
XM_004005653.3	ZBTB26	mRNA	TCONS_00075453	lncRNA
XM_004005653.3	ZBTB26	mRNA	TCONS_00075453	lncRNA
XM_004005653.3	ZBTB26	mRNA	TCONS_00075453	lncRNA
XM_004005653.3	ZBTB26	mRNA	TCONS_00075453	lncRNA
XM_012101919.2	ZBTB44	mRNA	TCONS_00075453	lncRNA
XM_012101919.2	ZBTB44	mRNA	TCONS_00075453	lncRNA
XM_012101919.2	ZBTB44	mRNA	TCONS_00075453	lncRNA
XM_012101919.2	ZBTB44	mRNA	TCONS_00075453	lncRNA
XM_012101919.2	ZBTB44	mRNA	TCONS_00075453	lncRNA

XM_012102658.2	ZNF518A	mRNA	XR_001020707.2	lncRNA
XM_012102658.2	ZNF518A	mRNA	XR_001020707.2	lncRNA
XM_012102658.2	ZNF518A	mRNA	XR_001020707.2	lncRNA
XM_012102658.2	ZNF518A	mRNA	XR_001020707.2	lncRNA
XM_012102658.2	ZNF518A	mRNA	XR_001020707.2	lncRNA
XM_012103119.1	EDRF1	mRNA	XR_001033284.2	lncRNA
XM_012103119.1	EDRF1	mRNA	XR_001033284.2	lncRNA
XM_012103119.1	EDRF1	mRNA	XR_001033284.2	lncRNA
XM_012103119.1	EDRF1	mRNA	XR_001033284.2	lncRNA
XM_012103119.1	EDRF1	mRNA	XR_001033284.2	lncRNA
XM_012179641.2	LOC10112286[illegible]	mRNA	XR_001020707.2	lncRNA
XM_012179641.2	LOC10112286[illegible]	mRNA	XR_001020707.2	lncRNA
XM_012179641.2	LOC10112286[illegible]	mRNA	XR_001020707.2	lncRNA
XM_012179641.2	LOC10112286[illegible]	mRNA	XR_001020707.2	lncRNA
XM_012179641.2	LOC10112286[illegible]	mRNA	XR_001020707.2	lncRNA
XM_012098348.2	MEF2A	mRNA	XR_001020707.2	lncRNA
XM_012098348.2	MEF2A	mRNA	XR_001020707.2	lncRNA
XM_012098348.2	MEF2A	mRNA	XR_001020707.2	lncRNA
XM_012098348.2	MEF2A	mRNA	XR_001020707.2	lncRNA
XM_012098348.2	MEF2A	mRNA	XR_001020707.2	lncRNA
XM_012094704.2	LOC10560198[illegible]	mRNA	XR_001434202.1	lncRNA
XM_012094704.2	LOC10560198[illegible]	mRNA	XR_001434202.1	lncRNA
XM_012094704.2	LOC10560198[illegible]	mRNA	XR_001434202.1	lncRNA
XM_012094704.2	LOC10560198[illegible]	mRNA	XR_001434202.1	lncRNA
XM_012094704.2	LOC10560198[illegible]	mRNA	XR_001434202.1	lncRNA
XM_015095875.1	SAFB	mRNA	XR_001434202.1	lncRNA
XM_015095875.1	SAFB	mRNA	XR_001434202.1	lncRNA
XM_015095875.1	SAFB	mRNA	XR_001434202.1	lncRNA
XM_015095875.1	SAFB	mRNA	XR_001434202.1	lncRNA
XM_015095875.1	SAFB	mRNA	XR_001434202.1	lncRNA
XM_015095875.1	SAFB	mRNA	XR_001034847.2	lncRNA
XM_015095875.1	SAFB	mRNA	XR_001034847.2	lncRNA
XM_015095875.1	SAFB	mRNA	XR_001034847.2	lncRNA
XM_015095875.1	SAFB	mRNA	XR_001034847.2	lncRNA
XM_015095875.1	SAFB	mRNA	XR_001034847.2	lncRNA
XM_012127663.2	IFT74	mRNA	XR_001434202.1	lncRNA
XM_012127663.2	IFT74	mRNA	XR_001434202.1	lncRNA
XM_012127663.2	IFT74	mRNA	XR_001434202.1	lncRNA
XM_012127663.2	IFT74	mRNA	XR_001434202.1	lncRNA
XM_012127663.2	IFT74	mRNA	XR_001434202.1	lncRNA
XM_015099971.1	ZNF181	mRNA	XR_001034847.2	lncRNA
XM_015099971.1	ZNF181	mRNA	XR_001034847.2	lncRNA
XM_015099971.1	ZNF181	mRNA	XR_001034847.2	lncRNA
XM_015099971.1	ZNF181	mRNA	XR_001034847.2	lncRNA
XM_015099971.1	ZNF181	mRNA	XR_001034847.2	lncRNA
XM_012188265.2	CCDC7	mRNA	XR_001034847.2	lncRNA
XM_012188265.2	CCDC7	mRNA	XR_001034847.2	lncRNA
XM_012188265.2	CCDC7	mRNA	XR_001034847.2	lncRNA
XM_012188265.2	CCDC7	mRNA	XR_001034847.2	lncRNA
XM_012188265.2	CCDC7	mRNA	XR_001034847.2	lncRNA
XM_015099971.1	ZNF181	mRNA	XR_001434202.1	lncRNA
XM_015099971.1	ZNF181	mRNA	XR_001434202.1	lncRNA
XM_015099971.1	ZNF181	mRNA	XR_001434202.1	lncRNA
XM_015099971.1	ZNF181	mRNA	XR_001434202.1	lncRNA

XM_015099971.1	ZNF181	mRNA	XR_001434202.1	lncRNA
XM_012127663.2	IFT74	mRNA	XR_001034847.2	lncRNA
XM_012127663.2	IFT74	mRNA	XR_001034847.2	lncRNA
XM_012127663.2	IFT74	mRNA	XR_001034847.2	lncRNA
XM_012127663.2	IFT74	mRNA	XR_001034847.2	lncRNA
XM_012127663.2	IFT74	mRNA	XR_001034847.2	lncRNA
XM_012188265.2	CCDC7	mRNA	XR_001434202.1	lncRNA
XM_012188265.2	CCDC7	mRNA	XR_001434202.1	lncRNA
XM_012188265.2	CCDC7	mRNA	XR_001434202.1	lncRNA
XM_012188265.2	CCDC7	mRNA	XR_001434202.1	lncRNA
XM_012188265.2	CCDC7	mRNA	XR_001434202.1	lncRNA
XM_012094704.2	LOC10560198	mRNA	XR_001034847.2	lncRNA
XM_012094704.2	LOC10560198	mRNA	XR_001034847.2	lncRNA
XM_012094704.2	LOC10560198	mRNA	XR_001034847.2	lncRNA
XM_012094704.2	LOC10560198	mRNA	XR_001034847.2	lncRNA
XM_012094704.2	LOC10560198	mRNA	XR_001034847.2	lncRNA
XM_015104327.1	LOC10111542	mRNA	XR_001034847.2	lncRNA
XM_015104327.1	LOC10111542	mRNA	XR_001034847.2	lncRNA
XM_015104327.1	LOC10111542	mRNA	XR_001034847.2	lncRNA
XM_015104327.1	LOC10111542	mRNA	XR_001034847.2	lncRNA
XM_015104327.1	LOC10111542	mRNA	XR_001034847.2	lncRNA
XM_012186029.1	FBXL20	mRNA	XR_001034847.2	lncRNA
XM_012186029.1	FBXL20	mRNA	XR_001034847.2	lncRNA
XM_012186029.1	FBXL20	mRNA	XR_001034847.2	lncRNA
XM_012186029.1	FBXL20	mRNA	XR_001034847.2	lncRNA
XM_012186029.1	FBXL20	mRNA	XR_001034847.2	lncRNA
XM_012104225.2	DCUN1D3	mRNA	XR_001034847.2	lncRNA
XM_012104225.2	DCUN1D3	mRNA	XR_001034847.2	lncRNA
XM_012104225.2	DCUN1D3	mRNA	XR_001034847.2	lncRNA
XM_012104225.2	DCUN1D3	mRNA	XR_001034847.2	lncRNA
XM_012104225.2	DCUN1D3	mRNA	XR_001034847.2	lncRNA
XM_012170864.2	PAPOLG	mRNA	XR_001434202.1	lncRNA
XM_012170864.2	PAPOLG	mRNA	XR_001434202.1	lncRNA
XM_012170864.2	PAPOLG	mRNA	XR_001434202.1	lncRNA
XM_012170864.2	PAPOLG	mRNA	XR_001434202.1	lncRNA
XM_012170864.2	PAPOLG	mRNA	XR_001434202.1	lncRNA
XM_012104225.2	DCUN1D3	mRNA	XR_001434202.1	lncRNA
XM_012104225.2	DCUN1D3	mRNA	XR_001434202.1	lncRNA
XM_012104225.2	DCUN1D3	mRNA	XR_001434202.1	lncRNA
XM_012104225.2	DCUN1D3	mRNA	XR_001434202.1	lncRNA
XM_012104225.2	DCUN1D3	mRNA	XR_001434202.1	lncRNA
XM_012170864.2	PAPOLG	mRNA	XR_001034847.2	lncRNA
XM_012170864.2	PAPOLG	mRNA	XR_001034847.2	lncRNA
XM_012170864.2	PAPOLG	mRNA	XR_001034847.2	lncRNA
XM_012170864.2	PAPOLG	mRNA	XR_001034847.2	lncRNA
XM_012170864.2	PAPOLG	mRNA	XR_001034847.2	lncRNA
XM_012186029.1	FBXL20	mRNA	XR_001434202.1	lncRNA
XM_012186029.1	FBXL20	mRNA	XR_001434202.1	lncRNA
XM_012186029.1	FBXL20	mRNA	XR_001434202.1	lncRNA
XM_012186029.1	FBXL20	mRNA	XR_001434202.1	lncRNA
XM_012186029.1	FBXL20	mRNA	XR_001434202.1	lncRNA
XM_015104327.1	LOC10111542	mRNA	XR_001434202.1	lncRNA
XM_015104327.1	LOC10111542	mRNA	XR_001434202.1	lncRNA
XM_015104327.1	LOC10111542	mRNA	XR_001434202.1	lncRNA

XM_015104327.1	LOC101115427	mRNA	XR_001434202.1	lncRNA
XM_015104327.1	LOC101115427	mRNA	XR_001434202.1	lncRNA
XM_012134416.2	PPIG	mRNA	TCONS_00022613	lncRNA
XM_012134416.2	PPIG	mRNA	TCONS_00022613	lncRNA
XM_012134416.2	PPIG	mRNA	TCONS_00022613	lncRNA
XM_012134416.2	PPIG	mRNA	TCONS_00022613	lncRNA
XM_012134416.2	PPIG	mRNA	TCONS_00022613	lncRNA
XM_012184885.2	DOCK9	mRNA	TCONS_00022613	lncRNA
XM_012184885.2	DOCK9	mRNA	TCONS_00022613	lncRNA
XM_012184885.2	DOCK9	mRNA	TCONS_00022613	lncRNA
XM_012184885.2	DOCK9	mRNA	TCONS_00022613	lncRNA
XM_012184885.2	DOCK9	mRNA	TCONS_00022613	lncRNA
XM_012135865.2	TANK	mRNA	XR_001025491.1	lncRNA
XM_012135865.2	TANK	mRNA	XR_001025491.1	lncRNA
XM_012135865.2	TANK	mRNA	XR_001025491.1	lncRNA
XM_012135865.2	TANK	mRNA	XR_001025491.1	lncRNA
XM_015098145.1	HSPH1	mRNA	XR_001024290.1	lncRNA
XM_015098145.1	HSPH1	mRNA	XR_001024290.1	lncRNA
XM_015098145.1	HSPH1	mRNA	XR_001024290.1	lncRNA
XM_015098145.1	HSPH1	mRNA	XR_001024290.1	lncRNA
XM_015102007.1	PPP2R3C	mRNA	XR_001024743.2	lncRNA
XM_015102007.1	PPP2R3C	mRNA	XR_001024743.2	lncRNA
XM_015102007.1	PPP2R3C	mRNA	XR_001024743.2	lncRNA
XM_015102007.1	PPP2R3C	mRNA	XR_001024743.2	lncRNA
XM_004011402.3	STXBP5	mRNA	TCONS_00075453	lncRNA
XM_004011402.3	STXBP5	mRNA	TCONS_00075453	lncRNA
XM_004011402.3	STXBP5	mRNA	TCONS_00075453	lncRNA
XM_004011402.3	STXBP5	mRNA	TCONS_00075453	lncRNA
XM_004011402.3	STXBP5	mRNA	TCONS_00075453	lncRNA
XM_015098592.1	RABEP1	mRNA	TCONS_00075453	lncRNA
XM_015098592.1	RABEP1	mRNA	TCONS_00075453	lncRNA
XM_015098592.1	RABEP1	mRNA	TCONS_00075453	lncRNA
XM_015098592.1	RABEP1	mRNA	TCONS_00075453	lncRNA
XM_015098592.1	RABEP1	mRNA	TCONS_00075453	lncRNA
XM_015093911.1	LOC101104054	mRNA	TCONS_00022613	lncRNA
XM_015093911.1	LOC101104054	mRNA	TCONS_00022613	lncRNA
XM_015093911.1	LOC101104054	mRNA	TCONS_00022613	lncRNA
XM_015093911.1	LOC101104054	mRNA	TCONS_00022613	lncRNA
XM_012130346.2	UGGT1	mRNA	TCONS_00054422	lncRNA
XM_012130346.2	UGGT1	mRNA	TCONS_00054422	lncRNA
XM_012130346.2	UGGT1	mRNA	TCONS_00054422	lncRNA
XM_012130346.2	UGGT1	mRNA	TCONS_00054422	lncRNA
XM_012175276.1	PLCM1	mRNA	TCONS_00054422	lncRNA
XM_012175276.1	PLCM1	mRNA	TCONS_00054422	lncRNA
XM_012175276.1	PLCM1	mRNA	TCONS_00054422	lncRNA
XM_012175276.1	PLCM1	mRNA	TCONS_00054422	lncRNA
XM_012104141.2	PDXDC1	mRNA	TCONS_00075051	lncRNA
XM_012104141.2	PDXDC1	mRNA	TCONS_00075051	lncRNA
XM_012104141.2	PDXDC1	mRNA	TCONS_00075051	lncRNA
XM_012104141.2	PDXDC1	mRNA	TCONS_00075051	lncRNA
XM_012096615.2	DLG1	mRNA	TCONS_00054422	lncRNA
XM_012096615.2	DLG1	mRNA	TCONS_00054422	lncRNA
XM_012096615.2	DLG1	mRNA	TCONS_00054422	lncRNA
XM_012096615.2	DLG1	mRNA	TCONS_00054422	lncRNA

XM_012098024.2	THOC5	mRNA	TCONS_00075051	lncRNA
XM_012098024.2	THOC5	mRNA	TCONS_00075051	lncRNA
XM_012098024.2	THOC5	mRNA	TCONS_00075051	lncRNA
XM_012098024.2	THOC5	mRNA	TCONS_00075051	lncRNA
XM_012176829.1	BBS9	mRNA	TCONS_00075051	lncRNA
XM_012176829.1	BBS9	mRNA	TCONS_00075051	lncRNA
XM_012176829.1	BBS9	mRNA	TCONS_00075051	lncRNA
XM_012176829.1	BBS9	mRNA	TCONS_00075051	lncRNA
XM_012094663.2	GRAMD1C	mRNA	TCONS_00075051	lncRNA
XM_012094663.2	GRAMD1C	mRNA	TCONS_00075051	lncRNA
XM_012094663.2	GRAMD1C	mRNA	TCONS_00075051	lncRNA
XM_012094663.2	GRAMD1C	mRNA	TCONS_00075051	lncRNA
XM_012183566.1	FAM49B	mRNA	TCONS_00075051	lncRNA
XM_012183566.1	FAM49B	mRNA	TCONS_00075051	lncRNA
XM_012183566.1	FAM49B	mRNA	TCONS_00075051	lncRNA
XM_012183566.1	FAM49B	mRNA	TCONS_00075051	lncRNA
XM_012101765.1	PRMT3	mRNA	XR_001436931.1	lncRNA
XM_012101765.1	PRMT3	mRNA	XR_001436931.1	lncRNA
XM_012101765.1	PRMT3	mRNA	XR_001436931.1	lncRNA
XM_012101765.1	PRMT3	mRNA	XR_001436931.1	lncRNA
XM_015092714.1	ANKRL18	mRNA	XR_001436931.1	lncRNA
XM_015092714.1	ANKRL18	mRNA	XR_001436931.1	lncRNA
XM_015092714.1	ANKRL18	mRNA	XR_001436931.1	lncRNA
XM_015092714.1	ANKRL18	mRNA	XR_001436931.1	lncRNA
XM_012102669.1	ZNF518A	mRNA	XR_001025491.1	lncRNA
XM_012102669.1	ZNF518A	mRNA	XR_001025491.1	lncRNA
XM_012102669.1	ZNF518A	mRNA	XR_001025491.1	lncRNA
XM_012102669.1	ZNF518A	mRNA	XR_001025491.1	lncRNA
XM_012102669.1	ZNF518A	mRNA	XR_001025491.1	lncRNA
XM_012178109.2	SGTA	mRNA	XR_001025491.1	lncRNA
XM_012178109.2	SGTA	mRNA	XR_001025491.1	lncRNA
XM_012178109.2	SGTA	mRNA	XR_001025491.1	lncRNA
XM_012178109.2	SGTA	mRNA	XR_001025491.1	lncRNA
XM_012178109.2	SGTA	mRNA	XR_001025491.1	lncRNA
XM_015097018.1	ZNF280D	mRNA	XR_001044126.1	lncRNA
XM_015097018.1	ZNF280D	mRNA	XR_001044126.1	lncRNA
XM_015097018.1	ZNF280D	mRNA	XR_001044126.1	lncRNA
XM_015097018.1	ZNF280D	mRNA	XR_001044126.1	lncRNA
XM_015097018.1	ZNF280D	mRNA	XR_001044126.1	lncRNA
XM_012098903.1	PPIP5K1	mRNA	XR_001044126.1	lncRNA
XM_012098903.1	PPIP5K1	mRNA	XR_001044126.1	lncRNA
XM_012098903.1	PPIP5K1	mRNA	XR_001044126.1	lncRNA
XM_012098903.1	PPIP5K1	mRNA	XR_001044126.1	lncRNA
XM_012098903.1	PPIP5K1	mRNA	XR_001044126.1	lncRNA
XM_015104788.1	TBL1X	mRNA	XR_001024290.1	lncRNA
XM_015104788.1	TBL1X	mRNA	XR_001024290.1	lncRNA
XM_015104788.1	TBL1X	mRNA	XR_001024290.1	lncRNA
XM_015104788.1	TBL1X	mRNA	XR_001024290.1	lncRNA
XM_015104788.1	TBL1X	mRNA	XR_001024290.1	lncRNA
XM_012181190.2	EIF2AK4	mRNA	XR_001025491.1	lncRNA
XM_012181190.2	EIF2AK4	mRNA	XR_001025491.1	lncRNA
XM_012181190.2	EIF2AK4	mRNA	XR_001025491.1	lncRNA
XM_012181190.2	EIF2AK4	mRNA	XR_001025491.1	lncRNA
XM_012181190.2	EIF2AK4	mRNA	XR_001025491.1	lncRNA

XM_012140422.2	CLASP1	mRNA	XR_001025491.1	lncRNA
XM_012140422.2	CLASP1	mRNA	XR_001025491.1	lncRNA
XM_012140422.2	CLASP1	mRNA	XR_001025491.1	lncRNA
XM_012140422.2	CLASP1	mRNA	XR_001025491.1	lncRNA
XM_012140422.2	CLASP1	mRNA	XR_001025491.1	lncRNA
XM_012097610.2	ZNF605	mRNA	XR_001020707.2	lncRNA
XM_012097610.2	ZNF605	mRNA	XR_001020707.2	lncRNA
XM_012097610.2	ZNF605	mRNA	XR_001020707.2	lncRNA
XM_012097610.2	ZNF605	mRNA	XR_001020707.2	lncRNA
XM_012097610.2	ZNF605	mRNA	XR_001020707.2	lncRNA
XM_012097254.2	RHOF	mRNA	XR_001020707.2	lncRNA
XM_012097254.2	RHOF	mRNA	XR_001020707.2	lncRNA
XM_012097254.2	RHOF	mRNA	XR_001020707.2	lncRNA
XM_012097254.2	RHOF	mRNA	XR_001020707.2	lncRNA
XM_012097254.2	RHOF	mRNA	XR_001020707.2	lncRNA
XM_012104566.2	ZKSCAN1	mRNA	XR_001436102.1	lncRNA
XM_012104566.2	ZKSCAN1	mRNA	XR_001436102.1	lncRNA
XM_012104566.2	ZKSCAN1	mRNA	XR_001436102.1	lncRNA
XM_012104566.2	ZKSCAN1	mRNA	XR_001436102.1	lncRNA
XM_012104566.2	ZKSCAN1	mRNA	XR_001436102.1	lncRNA
XM_012181827.2	ZBTB1	mRNA	XR_001021689.2	lncRNA
XM_012181827.2	ZBTB1	mRNA	XR_001021689.2	lncRNA
XM_012181827.2	ZBTB1	mRNA	XR_001021689.2	lncRNA
XM_012181827.2	ZBTB1	mRNA	XR_001021689.2	lncRNA
XM_012181827.2	ZBTB1	mRNA	XR_001021689.2	lncRNA
XM_015098136.1	PDS5B	mRNA	XR_001021689.2	lncRNA
XM_015098136.1	PDS5B	mRNA	XR_001021689.2	lncRNA
XM_015098136.1	PDS5B	mRNA	XR_001021689.2	lncRNA
XM_015098136.1	PDS5B	mRNA	XR_001021689.2	lncRNA
XM_015098136.1	PDS5B	mRNA	XR_001021689.2	lncRNA
XM_004004866.2	METTL21A	mRNA	XR_001434892.1	lncRNA
XM_004004866.2	METTL21A	mRNA	XR_001434892.1	lncRNA
XM_004004866.2	METTL21A	mRNA	XR_001434892.1	lncRNA
XM_004004866.2	METTL21A	mRNA	XR_001434892.1	lncRNA
XM_004004866.2	METTL21A	mRNA	XR_001434892.1	lncRNA
XM_004014196.3	ABI1	mRNA	XR_001434892.1	lncRNA
XM_004014196.3	ABI1	mRNA	XR_001434892.1	lncRNA
XM_004014196.3	ABI1	mRNA	XR_001434892.1	lncRNA
XM_004014196.3	ABI1	mRNA	XR_001434892.1	lncRNA
XM_004014196.3	ABI1	mRNA	XR_001434892.1	lncRNA
XM_015097132.1	TMEM229B	mRNA	TCONS_00075453	lncRNA
XM_015097132.1	TMEM229B	mRNA	TCONS_00075453	lncRNA
XM_015097132.1	TMEM229B	mRNA	TCONS_00075453	lncRNA
XM_015097132.1	TMEM229B	mRNA	TCONS_00075453	lncRNA
XM_015097132.1	TMEM229B	mRNA	TCONS_00075453	lncRNA
XM_012094704.2	LOC10560198[illegible]	mRNA	XR_001024743.2	lncRNA
XM_012094704.2	LOC10560198[illegible]	mRNA	XR_001024743.2	lncRNA
XM_012094704.2	LOC10560198[illegible]	mRNA	XR_001024743.2	lncRNA
XM_012094704.2	LOC10560198[illegible]	mRNA	XR_001024743.2	lncRNA
XM_012190370.2	FAM98C	mRNA	XR_001024743.2	lncRNA
XM_012190370.2	FAM98C	mRNA	XR_001024743.2	lncRNA
XM_012190370.2	FAM98C	mRNA	XR_001024743.2	lncRNA
XM_012190370.2	FAM98C	mRNA	XR_001024743.2	lncRNA
XM_015096972.1	L2HGDH	mRNA	XR_001024743.2	lncRNA

XM_015096972.1	L2HGDH	mRNA	XR_001024743.2	lncRNA
XM_015096972.1	L2HGDH	mRNA	XR_001024743.2	lncRNA
XM_015096972.1	L2HGDH	mRNA	XR_001024743.2	lncRNA
XM_015098987.1	ABCA5	mRNA	TCONS_00054422	lncRNA
XM_015098987.1	ABCA5	mRNA	TCONS_00054422	lncRNA
XM_015098987.1	ABCA5	mRNA	TCONS_00054422	lncRNA
XM_015098987.1	ABCA5	mRNA	TCONS_00054422	lncRNA
XM_012184823.2	RBM26	mRNA	XR_001436931.1	lncRNA
XM_012184823.2	RBM26	mRNA	XR_001436931.1	lncRNA
XM_012184823.2	RBM26	mRNA	XR_001436931.1	lncRNA
XM_012184823.2	RBM26	mRNA	XR_001436931.1	lncRNA
XM_012107472.1	NFYC	mRNA	TCONS_00054422	lncRNA
XM_012107472.1	NFYC	mRNA	TCONS_00054422	lncRNA
XM_012107472.1	NFYC	mRNA	TCONS_00054422	lncRNA
XM_012107472.1	NFYC	mRNA	TCONS_00054422	lncRNA
XM_004007700.3	CLDN12	mRNA	TCONS_00054422	lncRNA
XM_004007700.3	CLDN12	mRNA	TCONS_00054422	lncRNA
XM_004007700.3	CLDN12	mRNA	TCONS_00054422	lncRNA
XM_004007700.3	CLDN12	mRNA	TCONS_00054422	lncRNA
XM_012096615.2	DLG1	mRNA	XR_001434202.1	lncRNA
XM_012096615.2	DLG1	mRNA	XR_001434202.1	lncRNA
XM_012096615.2	DLG1	mRNA	XR_001434202.1	lncRNA
XM_012096615.2	DLG1	mRNA	XR_001434202.1	lncRNA
XM_012096615.2	DLG1	mRNA	XR_001434202.1	lncRNA
XM_012096615.2	DLG1	mRNA	XR_001034847.2	lncRNA
XM_012096615.2	DLG1	mRNA	XR_001034847.2	lncRNA
XM_012096615.2	DLG1	mRNA	XR_001034847.2	lncRNA
XM_012096615.2	DLG1	mRNA	XR_001034847.2	lncRNA
XM_012096615.2	DLG1	mRNA	XR_001034847.2	lncRNA
XM_012174536.2	KIF21A	mRNA	XR_001020707.2	lncRNA
XM_012174536.2	KIF21A	mRNA	XR_001020707.2	lncRNA
XM_012174536.2	KIF21A	mRNA	XR_001020707.2	lncRNA
XM_012174536.2	KIF21A	mRNA	XR_001020707.2	lncRNA
XM_012174536.2	KIF21A	mRNA	XR_001020707.2	lncRNA
XM_012104950.1	TTC13	mRNA	XR_001025491.1	lncRNA
XM_012104950.1	TTC13	mRNA	XR_001025491.1	lncRNA
XM_012104950.1	TTC13	mRNA	XR_001025491.1	lncRNA
XM_012104950.1	TTC13	mRNA	XR_001025491.1	lncRNA
XM_012104950.1	TTC13	mRNA	XR_001025491.1	lncRNA
XM_015093216.1	TTC39B	mRNA	XR_001025491.1	lncRNA
XM_015093216.1	TTC39B	mRNA	XR_001025491.1	lncRNA
XM_015093216.1	TTC39B	mRNA	XR_001025491.1	lncRNA
XM_015093216.1	TTC39B	mRNA	XR_001025491.1	lncRNA
XM_015093216.1	TTC39B	mRNA	XR_001025491.1	lncRNA
XM_015101485.1	LOC106991694	mRNA	XR_001044126.1	lncRNA
XM_015101485.1	LOC106991694	mRNA	XR_001044126.1	lncRNA
XM_015101485.1	LOC106991694	mRNA	XR_001044126.1	lncRNA
XM_015101485.1	LOC106991694	mRNA	XR_001044126.1	lncRNA
XM_015101485.1	LOC106991694	mRNA	XR_001044126.1	lncRNA
XM_012180554.2	TARS2	mRNA	XR_001025491.1	lncRNA
XM_012180554.2	TARS2	mRNA	XR_001025491.1	lncRNA
XM_012180554.2	TARS2	mRNA	XR_001025491.1	lncRNA
XM_012180554.2	TARS2	mRNA	XR_001025491.1	lncRNA
XM_012180554.2	TARS2	mRNA	XR_001025491.1	lncRNA

XM_012189207.2	SENP7	mRNA	XR_001436102.1	lncRNA
XM_012189207.2	SENP7	mRNA	XR_001436102.1	lncRNA
XM_012189207.2	SENP7	mRNA	XR_001436102.1	lncRNA
XM_012189207.2	SENP7	mRNA	XR_001436102.1	lncRNA
XM_012183965.2	OXR1	mRNA	XR_001436102.1	lncRNA
XM_012183965.2	OXR1	mRNA	XR_001436102.1	lncRNA
XM_012183965.2	OXR1	mRNA	XR_001436102.1	lncRNA
XM_012183965.2	OXR1	mRNA	XR_001436102.1	lncRNA
XM_012135865.2	TANK	mRNA	XR_001436102.1	lncRNA
XM_012135865.2	TANK	mRNA	XR_001436102.1	lncRNA
XM_012135865.2	TANK	mRNA	XR_001436102.1	lncRNA
XM_012135865.2	TANK	mRNA	XR_001436102.1	lncRNA
XM_012174289.1	ATF1	mRNA	XR_001021689.2	lncRNA
XM_012174289.1	ATF1	mRNA	XR_001021689.2	lncRNA
XM_012174289.1	ATF1	mRNA	XR_001021689.2	lncRNA
XM_012174289.1	ATF1	mRNA	XR_001021689.2	lncRNA
XM_012183145.1	MTRF1L	mRNA	XR_001436102.1	lncRNA
XM_012183145.1	MTRF1L	mRNA	XR_001436102.1	lncRNA
XM_012183145.1	MTRF1L	mRNA	XR_001436102.1	lncRNA
XM_012183145.1	MTRF1L	mRNA	XR_001436102.1	lncRNA
XM_012174289.1	ATF1	mRNA	XR_001436102.1	lncRNA
XM_012174289.1	ATF1	mRNA	XR_001436102.1	lncRNA
XM_012174289.1	ATF1	mRNA	XR_001436102.1	lncRNA
XM_012174289.1	ATF1	mRNA	XR_001436102.1	lncRNA
XM_004002454.2	ECM1	mRNA	XR_001021689.2	lncRNA
XM_004002454.2	ECM1	mRNA	XR_001021689.2	lncRNA
XM_004002454.2	ECM1	mRNA	XR_001021689.2	lncRNA
XM_004002454.2	ECM1	mRNA	XR_001021689.2	lncRNA
XM_015098145.1	HSPH1	mRNA	XR_001436102.1	lncRNA
XM_015098145.1	HSPH1	mRNA	XR_001436102.1	lncRNA
XM_015098145.1	HSPH1	mRNA	XR_001436102.1	lncRNA
XM_015098145.1	HSPH1	mRNA	XR_001436102.1	lncRNA
XM_015104846.1	TAB3	mRNA	XR_001436102.1	lncRNA
XM_015104846.1	TAB3	mRNA	XR_001436102.1	lncRNA
XM_015104846.1	TAB3	mRNA	XR_001436102.1	lncRNA
XM_015104846.1	TAB3	mRNA	XR_001436102.1	lncRNA
XM_015104846.1	TAB3	mRNA	XR_001436102.1	lncRNA
XM_012179696.2	LOC10111286[illegible]	mRNA	TCONS_00075453	lncRNA
XM_012179696.2	LOC10111286[illegible]	mRNA	TCONS_00075453	lncRNA
XM_012179696.2	LOC10111286[illegible]	mRNA	TCONS_00075453	lncRNA
XM_012179696.2	LOC10111286[illegible]	mRNA	TCONS_00075453	lncRNA
XM_012179696.2	LOC10111286[illegible]	mRNA	TCONS_00075453	lncRNA
XM_012134593.2	FASTKD1	mRNA	XR_001033284.2	lncRNA
XM_012134593.2	FASTKD1	mRNA	XR_001033284.2	lncRNA
XM_012134593.2	FASTKD1	mRNA	XR_001033284.2	lncRNA
XM_012134593.2	FASTKD1	mRNA	XR_001033284.2	lncRNA
XM_012181861.1	GPHN	mRNA	TCONS_00075051	lncRNA
XM_012181861.1	GPHN	mRNA	TCONS_00075051	lncRNA
XM_012181861.1	GPHN	mRNA	TCONS_00075051	lncRNA
XM_012181861.1	GPHN	mRNA	TCONS_00075051	lncRNA
XM_012176217.2	EVI5	mRNA	TCONS_00084305	lncRNA
XM_012176217.2	EVI5	mRNA	TCONS_00084305	lncRNA
XM_012176217.2	EVI5	mRNA	TCONS_00084305	lncRNA
XM_012176217.2	EVI5	mRNA	TCONS_00084305	lncRNA

XM_012185217.2	CLTC	mRNA	XR_001436931.1	lncRNA
XM_012185217.2	CLTC	mRNA	XR_001436931.1	lncRNA
XM_012185217.2	CLTC	mRNA	XR_001436931.1	lncRNA
XM_012185217.2	CLTC	mRNA	XR_001436931.1	lncRNA
XM_015104788.1	TBL1X	mRNA	TCONS_00084305	lncRNA
XM_015104788.1	TBL1X	mRNA	TCONS_00084305	lncRNA
XM_015104788.1	TBL1X	mRNA	TCONS_00084305	lncRNA
XM_015104788.1	TBL1X	mRNA	TCONS_00084305	lncRNA
XM_012103119.1	EDRF1	mRNA	TCONS_00075051	lncRNA
XM_012103119.1	EDRF1	mRNA	TCONS_00075051	lncRNA
XM_012103119.1	EDRF1	mRNA	TCONS_00075051	lncRNA
XM_012103119.1	EDRF1	mRNA	TCONS_00075051	lncRNA
NM_001290793.2	PEG10	mRNA	XR_001436931.1	lncRNA
NM_001290793.2	PEG10	mRNA	XR_001436931.1	lncRNA
NM_001290793.2	PEG10	mRNA	XR_001436931.1	lncRNA
NM_001290793.2	PEG10	mRNA	XR_001436931.1	lncRNA
XM_012190370.2	FAM98C	mRNA	XR_001436532.1	lncRNA
XM_012190370.2	FAM98C	mRNA	XR_001436532.1	lncRNA
XM_012190370.2	FAM98C	mRNA	XR_001436532.1	lncRNA
XM_012190370.2	FAM98C	mRNA	XR_001436532.1	lncRNA
XM_012190370.2	FAM98C	mRNA	XR_001436532.1	lncRNA
XM_004014196.3	ABI1	mRNA	XR_001436532.1	lncRNA
XM_004014196.3	ABI1	mRNA	XR_001436532.1	lncRNA
XM_004014196.3	ABI1	mRNA	XR_001436532.1	lncRNA
XM_004014196.3	ABI1	mRNA	XR_001436532.1	lncRNA
XM_004014196.3	ABI1	mRNA	XR_001436532.1	lncRNA
XM_012178788.2	HMGXB3	mRNA	XR_001020707.2	lncRNA
XM_012178788.2	HMGXB3	mRNA	XR_001020707.2	lncRNA
XM_012178788.2	HMGXB3	mRNA	XR_001020707.2	lncRNA
XM_012178788.2	HMGXB3	mRNA	XR_001020707.2	lncRNA
XM_012178788.2	HMGXB3	mRNA	XR_001020707.2	lncRNA
XM_012146543.1	ATG9A	mRNA	XR_001033284.2	lncRNA
XM_012146543.1	ATG9A	mRNA	XR_001033284.2	lncRNA
XM_012146543.1	ATG9A	mRNA	XR_001033284.2	lncRNA
XM_012146543.1	ATG9A	mRNA	XR_001033284.2	lncRNA
XM_012146543.1	ATG9A	mRNA	XR_001033284.2	lncRNA
XM_004002454.2	ECM1	mRNA	XR_001434202.1	lncRNA
XM_004002454.2	ECM1	mRNA	XR_001434202.1	lncRNA
XM_004002454.2	ECM1	mRNA	XR_001434202.1	lncRNA
XM_004002454.2	ECM1	mRNA	XR_001434202.1	lncRNA
XM_012183145.1	MTRF1L	mRNA	XR_001434202.1	lncRNA
XM_012183145.1	MTRF1L	mRNA	XR_001434202.1	lncRNA
XM_012183145.1	MTRF1L	mRNA	XR_001434202.1	lncRNA
XM_012183145.1	MTRF1L	mRNA	XR_001434202.1	lncRNA
XM_012183145.1	MTRF1L	mRNA	XR_001034847.2	lncRNA
XM_012183145.1	MTRF1L	mRNA	XR_001034847.2	lncRNA
XM_012183145.1	MTRF1L	mRNA	XR_001034847.2	lncRNA
XM_012183145.1	MTRF1L	mRNA	XR_001034847.2	lncRNA
XM_004002454.2	ECM1	mRNA	XR_001034847.2	lncRNA
XM_004002454.2	ECM1	mRNA	XR_001034847.2	lncRNA
XM_004002454.2	ECM1	mRNA	XR_001034847.2	lncRNA
XM_004002454.2	ECM1	mRNA	XR_001034847.2	lncRNA
XM_015103387.1	C22H10orf76	mRNA	TCONS_00022613	lncRNA
XM_015103387.1	C22H10orf76	mRNA	TCONS_00022613	lncRNA

XM_015103387.1	C22H10orf76	mRNA	TCONS_00022613	lncRNA
XM_015103387.1	C22H10orf76	mRNA	TCONS_00022613	lncRNA
XM_012096744.2	GFM2	mRNA	TCONS_00022613	lncRNA
XM_012096744.2	GFM2	mRNA	TCONS_00022613	lncRNA
XM_012096744.2	GFM2	mRNA	TCONS_00022613	lncRNA
XM_012096744.2	GFM2	mRNA	TCONS_00022613	lncRNA
XM_012175276.1	PLCM1	mRNA	XR_001024743.2	lncRNA
XM_012175276.1	PLCM1	mRNA	XR_001024743.2	lncRNA
XM_012175276.1	PLCM1	mRNA	XR_001024743.2	lncRNA
XM_012175276.1	PLCM1	mRNA	XR_001024743.2	lncRNA
XM_004011402.3	STXBP5	mRNA	XR_001025491.1	lncRNA
XM_004011402.3	STXBP5	mRNA	XR_001025491.1	lncRNA
XM_004011402.3	STXBP5	mRNA	XR_001025491.1	lncRNA
XM_004011402.3	STXBP5	mRNA	XR_001025491.1	lncRNA
XM_004011402.3	STXBP5	mRNA	XR_001025491.1	lncRNA
XM_004011402.3	STXBP5	mRNA	XR_001044126.1	lncRNA
XM_004011402.3	STXBP5	mRNA	XR_001044126.1	lncRNA
XM_004011402.3	STXBP5	mRNA	XR_001044126.1	lncRNA
XM_004011402.3	STXBP5	mRNA	XR_001044126.1	lncRNA
XM_004011402.3	STXBP5	mRNA	XR_001044126.1	lncRNA
XM_012100080.2	ARIH2	mRNA	XR_001434892.1	lncRNA
XM_012100080.2	ARIH2	mRNA	XR_001434892.1	lncRNA
XM_012100080.2	ARIH2	mRNA	XR_001434892.1	lncRNA
XM_012100080.2	ARIH2	mRNA	XR_001434892.1	lncRNA
XM_012100080.2	ARIH2	mRNA	XR_001434892.1	lncRNA
XM_012184401.1	ZC3H13	mRNA	XR_001434202.1	lncRNA
XM_012184401.1	ZC3H13	mRNA	XR_001434202.1	lncRNA
XM_012184401.1	ZC3H13	mRNA	XR_001434202.1	lncRNA
XM_012184401.1	ZC3H13	mRNA	XR_001434202.1	lncRNA
XM_012184401.1	ZC3H13	mRNA	XR_001434202.1	lncRNA
XM_015099609.1	ARHGAP12	mRNA	XR_001434202.1	lncRNA
XM_015099609.1	ARHGAP12	mRNA	XR_001434202.1	lncRNA
XM_015099609.1	ARHGAP12	mRNA	XR_001434202.1	lncRNA
XM_015099609.1	ARHGAP12	mRNA	XR_001434202.1	lncRNA
XM_015099609.1	ARHGAP12	mRNA	XR_001434202.1	lncRNA
XM_012184401.1	ZC3H13	mRNA	XR_001034847.2	lncRNA
XM_012184401.1	ZC3H13	mRNA	XR_001034847.2	lncRNA
XM_012184401.1	ZC3H13	mRNA	XR_001034847.2	lncRNA
XM_012184401.1	ZC3H13	mRNA	XR_001034847.2	lncRNA
XM_012184401.1	ZC3H13	mRNA	XR_001034847.2	lncRNA
XM_015099609.1	ARHGAP12	mRNA	XR_001034847.2	lncRNA
XM_015099609.1	ARHGAP12	mRNA	XR_001034847.2	lncRNA
XM_015099609.1	ARHGAP12	mRNA	XR_001034847.2	lncRNA
XM_015099609.1	ARHGAP12	mRNA	XR_001034847.2	lncRNA
XM_015099609.1	ARHGAP12	mRNA	XR_001034847.2	lncRNA
XM_012185318.2	RAD51D	mRNA	TCONS_00075453	lncRNA
XM_012185318.2	RAD51D	mRNA	TCONS_00075453	lncRNA
XM_012185318.2	RAD51D	mRNA	TCONS_00075453	lncRNA
XM_012185318.2	RAD51D	mRNA	TCONS_00075453	lncRNA
XM_012185318.2	RAD51D	mRNA	TCONS_00075453	lncRNA
XM_012127655.2	IFT74	mRNA	TCONS_00078440	lncRNA
XM_012127655.2	IFT74	mRNA	TCONS_00078440	lncRNA
XM_012127655.2	IFT74	mRNA	TCONS_00078440	lncRNA
XM_012127655.2	IFT74	mRNA	TCONS_00078440	lncRNA

XM_015094303.1	ACTR2	mRNA	XR_001044126.1	lncRNA
XM_015094303.1	ACTR2	mRNA	XR_001044126.1	lncRNA
XM_015094303.1	ACTR2	mRNA	XR_001044126.1	lncRNA
XM_015094303.1	ACTR2	mRNA	XR_001044126.1	lncRNA
XM_015098003.1	LOC10110962(	mRNA	XR_001025491.1	lncRNA
XM_015098003.1	LOC10110962(	mRNA	XR_001025491.1	lncRNA
XM_015098003.1	LOC10110962(	mRNA	XR_001025491.1	lncRNA
XM_015098003.1	LOC10110962(	mRNA	XR_001025491.1	lncRNA
XM_015098513.1	RHOT1	mRNA	XR_001025491.1	lncRNA
XM_015098513.1	RHOT1	mRNA	XR_001025491.1	lncRNA
XM_015098513.1	RHOT1	mRNA	XR_001025491.1	lncRNA
XM_015098513.1	RHOT1	mRNA	XR_001025491.1	lncRNA
XM_004010712.2	WDR89	mRNA	XR_001025491.1	lncRNA
XM_004010712.2	WDR89	mRNA	XR_001025491.1	lncRNA
XM_004010712.2	WDR89	mRNA	XR_001025491.1	lncRNA
XM_004010712.2	WDR89	mRNA	XR_001025491.1	lncRNA
XM_012098754.1	G2E3	mRNA	XR_001025491.1	lncRNA
XM_012098754.1	G2E3	mRNA	XR_001025491.1	lncRNA
XM_012098754.1	G2E3	mRNA	XR_001025491.1	lncRNA
XM_012098754.1	G2E3	mRNA	XR_001025491.1	lncRNA
XM_012182400.2	SENP6	mRNA	XR_001025491.1	lncRNA
XM_012182400.2	SENP6	mRNA	XR_001025491.1	lncRNA
XM_012182400.2	SENP6	mRNA	XR_001025491.1	lncRNA
XM_012182400.2	SENP6	mRNA	XR_001025491.1	lncRNA
XM_012105643.1	ZFP69	mRNA	XR_001436127.1	lncRNA
XM_012105643.1	ZFP69	mRNA	XR_001436127.1	lncRNA
XM_012105643.1	ZFP69	mRNA	XR_001436127.1	lncRNA
XM_012105643.1	ZFP69	mRNA	XR_001436127.1	lncRNA
XM_015103117.1	ZBTB44	mRNA	XR_001436931.1	lncRNA
XM_015103117.1	ZBTB44	mRNA	XR_001436931.1	lncRNA
XM_015103117.1	ZBTB44	mRNA	XR_001436931.1	lncRNA
XM_015103117.1	ZBTB44	mRNA	XR_001436931.1	lncRNA
XM_012188768.2	UBOX5	mRNA	TCONS_00078442	lncRNA
XM_012188768.2	UBOX5	mRNA	TCONS_00078442	lncRNA
XM_012188768.2	UBOX5	mRNA	TCONS_00078442	lncRNA
XM_012188768.2	UBOX5	mRNA	TCONS_00078442	lncRNA
XM_012102629.2	SORBS1	mRNA	TCONS_00078442	lncRNA
XM_012102629.2	SORBS1	mRNA	TCONS_00078442	lncRNA
XM_012102629.2	SORBS1	mRNA	TCONS_00078442	lncRNA
XM_012102629.2	SORBS1	mRNA	TCONS_00078442	lncRNA
XM_015101485.1	LOC10699169²	mRNA	TCONS_00082638	lncRNA
XM_015101485.1	LOC10699169²	mRNA	TCONS_00082638	lncRNA
XM_015101485.1	LOC10699169²	mRNA	TCONS_00082638	lncRNA
XM_015101485.1	LOC10699169²	mRNA	TCONS_00082638	lncRNA
XM_015098660.1	MAP2K4	mRNA	TCONS_00082638	lncRNA
XM_015098660.1	MAP2K4	mRNA	TCONS_00082638	lncRNA
XM_015098660.1	MAP2K4	mRNA	TCONS_00082638	lncRNA
XM_015098660.1	MAP2K4	mRNA	TCONS_00082638	lncRNA
XM_015092341.1	USP21	mRNA	TCONS_00084305	lncRNA
XM_015092341.1	USP21	mRNA	TCONS_00084305	lncRNA
XM_015092341.1	USP21	mRNA	TCONS_00084305	lncRNA
XM_015092341.1	USP21	mRNA	TCONS_00084305	lncRNA
XM_012101919.2	ZBTB44	mRNA	XR_001436931.1	lncRNA
XM_012101919.2	ZBTB44	mRNA	XR_001436931.1	lncRNA

XM_012101919.2	ZBTB44	mRNA	XR_001436931.1	lncRNA
XM_012101919.2	ZBTB44	mRNA	XR_001436931.1	lncRNA
XM_012097254.2	RHOF	mRNA	TCONS_00054422	lncRNA
XM_012097254.2	RHOF	mRNA	TCONS_00054422	lncRNA
XM_012097254.2	RHOF	mRNA	TCONS_00054422	lncRNA
XM_012097254.2	RHOF	mRNA	TCONS_00054422	lncRNA
XM_015097748.1	PCMTD1	mRNA	XR_001436931.1	lncRNA
XM_015097748.1	PCMTD1	mRNA	XR_001436931.1	lncRNA
XM_015097748.1	PCMTD1	mRNA	XR_001436931.1	lncRNA
XM_015097748.1	PCMTD1	mRNA	XR_001436931.1	lncRNA
XM_015092883.1	PADI1	mRNA	XR_001436102.1	lncRNA
XM_015092883.1	PADI1	mRNA	XR_001436102.1	lncRNA
XM_015092883.1	PADI1	mRNA	XR_001436102.1	lncRNA
XM_015092883.1	PADI1	mRNA	XR_001436102.1	lncRNA
XM_015092883.1	PADI1	mRNA	XR_001436102.1	lncRNA
XM_015094203.1	RC3H2	mRNA	XR_001436127.1	lncRNA
XM_015094203.1	RC3H2	mRNA	XR_001436127.1	lncRNA
XM_015094203.1	RC3H2	mRNA	XR_001436127.1	lncRNA
XM_015094203.1	RC3H2	mRNA	XR_001436127.1	lncRNA
XM_015094203.1	RC3H2	mRNA	XR_001436127.1	lncRNA
XM_012101213.2	LOC105601864	mRNA	XR_001434761.1	lncRNA
XM_012101213.2	LOC105601864	mRNA	XR_001434761.1	lncRNA
XM_012101213.2	LOC105601864	mRNA	XR_001434761.1	lncRNA
XM_012101213.2	LOC105601864	mRNA	XR_001434761.1	lncRNA
XM_015096385.1	TBCK	mRNA	XR_001434761.1	lncRNA
XM_015096385.1	TBCK	mRNA	XR_001434761.1	lncRNA
XM_015096385.1	TBCK	mRNA	XR_001434761.1	lncRNA
XM_015096385.1	TBCK	mRNA	XR_001434761.1	lncRNA
XM_012173546.1	NDUFAF7	mRNA	XR_001434761.1	lncRNA
XM_012173546.1	NDUFAF7	mRNA	XR_001434761.1	lncRNA
XM_012173546.1	NDUFAF7	mRNA	XR_001434761.1	lncRNA
XM_012173546.1	NDUFAF7	mRNA	XR_001434761.1	lncRNA
XM_015098145.1	HSPH1	mRNA	XR_001434761.1	lncRNA
XM_015098145.1	HSPH1	mRNA	XR_001434761.1	lncRNA
XM_015098145.1	HSPH1	mRNA	XR_001434761.1	lncRNA
XM_015098145.1	HSPH1	mRNA	XR_001434761.1	lncRNA
XM_015098144.1	HSPH1	mRNA	XR_001434892.1	lncRNA
XM_015098144.1	HSPH1	mRNA	XR_001434892.1	lncRNA
XM_015098144.1	HSPH1	mRNA	XR_001434892.1	lncRNA
XM_015098144.1	HSPH1	mRNA	XR_001434892.1	lncRNA
XM_012187648.1	LOC101122372	mRNA	XR_001434892.1	lncRNA
XM_012187648.1	LOC101122372	mRNA	XR_001434892.1	lncRNA
XM_012187648.1	LOC101122372	mRNA	XR_001434892.1	lncRNA
XM_012187648.1	LOC101122372	mRNA	XR_001434892.1	lncRNA
XM_004006434.3	PUS7L	mRNA	TCONS_00075453	lncRNA
XM_004006434.3	PUS7L	mRNA	TCONS_00075453	lncRNA
XM_004006434.3	PUS7L	mRNA	TCONS_00075453	lncRNA
XM_004006434.3	PUS7L	mRNA	TCONS_00075453	lncRNA
XM_015098987.1	ABCA5	mRNA	XR_001024743.2	lncRNA
XM_015098987.1	ABCA5	mRNA	XR_001024743.2	lncRNA
XM_015098987.1	ABCA5	mRNA	XR_001024743.2	lncRNA
XM_015098987.1	ABCA5	mRNA	XR_001024743.2	lncRNA
XM_015092486.1	PIGZ	mRNA	TCONS_00082638	lncRNA
XM_015092486.1	PIGZ	mRNA	TCONS_00082638	lncRNA

XM_015092486.1	PIGZ	mRNA	TCONS_00082638	lncRNA
XM_015092486.1	PIGZ	mRNA	TCONS_00082638	lncRNA
XM_015100041.1	ZNF473	mRNA	TCONS_00075051	lncRNA
XM_015100041.1	ZNF473	mRNA	TCONS_00075051	lncRNA
XM_015100041.1	ZNF473	mRNA	TCONS_00075051	lncRNA
XM_015100041.1	ZNF473	mRNA	TCONS_00075051	lncRNA
XM_015100041.1	ZNF473	mRNA	TCONS_00054422	lncRNA
XM_015100041.1	ZNF473	mRNA	TCONS_00054422	lncRNA
XM_015100041.1	ZNF473	mRNA	TCONS_00054422	lncRNA
XM_015100041.1	ZNF473	mRNA	TCONS_00054422	lncRNA
XM_012165732.1	FAM228B	mRNA	TCONS_00078442	lncRNA
XM_012165732.1	FAM228B	mRNA	TCONS_00078442	lncRNA
XM_012165732.1	FAM228B	mRNA	TCONS_00078442	lncRNA
XM_012165732.1	FAM228B	mRNA	TCONS_00078442	lncRNA
XM_012165732.1	FAM228B	mRNA	TCONS_00084305	lncRNA
XM_012165732.1	FAM228B	mRNA	TCONS_00084305	lncRNA
XM_012165732.1	FAM228B	mRNA	TCONS_00084305	lncRNA
XM_012165732.1	FAM228B	mRNA	TCONS_00084305	lncRNA
XM_015092486.1	PIGZ	mRNA	XR_001436931.1	lncRNA
XM_015092486.1	PIGZ	mRNA	XR_001436931.1	lncRNA
XM_015092486.1	PIGZ	mRNA	XR_001436931.1	lncRNA
XM_015092486.1	PIGZ	mRNA	XR_001436931.1	lncRNA
XM_012101211.2	MBNL1	mRNA	TCONS_00054422	lncRNA
XM_012101211.2	MBNL1	mRNA	TCONS_00054422	lncRNA
XM_012101211.2	MBNL1	mRNA	TCONS_00054422	lncRNA
XM_012101211.2	MBNL1	mRNA	TCONS_00054422	lncRNA
XM_015098592.1	RABEP1	mRNA	XR_001436931.1	lncRNA
XM_015098592.1	RABEP1	mRNA	XR_001436931.1	lncRNA
XM_015098592.1	RABEP1	mRNA	XR_001436931.1	lncRNA
XM_015098592.1	RABEP1	mRNA	XR_001436931.1	lncRNA
XM_012186914.2	ZC3H11A	mRNA	TCONS_00022613	lncRNA
XM_012186914.2	ZC3H11A	mRNA	TCONS_00022613	lncRNA
XM_012186914.2	ZC3H11A	mRNA	TCONS_00022613	lncRNA
XM_012186914.2	ZC3H11A	mRNA	TCONS_00022613	lncRNA
XM_012190139.2	ZMYM6	mRNA	XR_001433755.1	lncRNA
XM_012190139.2	ZMYM6	mRNA	XR_001433755.1	lncRNA
XM_012190139.2	ZMYM6	mRNA	XR_001433755.1	lncRNA
XM_012190139.2	ZMYM6	mRNA	XR_001433755.1	lncRNA
XM_015097170.1	NUMB	mRNA	XR_001025491.1	lncRNA
XM_015097170.1	NUMB	mRNA	XR_001025491.1	lncRNA
XM_015097170.1	NUMB	mRNA	XR_001025491.1	lncRNA
XM_015097170.1	NUMB	mRNA	XR_001025491.1	lncRNA
XM_015097170.1	NUMB	mRNA	XR_001025491.1	lncRNA
XM_012102598.2	TBC1D12	mRNA	XR_001436532.1	lncRNA
XM_012102598.2	TBC1D12	mRNA	XR_001436532.1	lncRNA
XM_012102598.2	TBC1D12	mRNA	XR_001436532.1	lncRNA
XM_012102598.2	TBC1D12	mRNA	XR_001436532.1	lncRNA
XM_012100420.2	MAPK14	mRNA	XR_001042381.2	lncRNA
XM_012100420.2	MAPK14	mRNA	XR_001042381.2	lncRNA
XM_012100420.2	MAPK14	mRNA	XR_001042381.2	lncRNA
XM_012100420.2	MAPK14	mRNA	XR_001042381.2	lncRNA
XM_012095725.2	BTBD10	mRNA	XR_001042381.2	lncRNA
XM_012095725.2	BTBD10	mRNA	XR_001042381.2	lncRNA
XM_012095725.2	BTBD10	mRNA	XR_001042381.2	lncRNA

XM_012095725.2	BTBD10	mRNA	XR_001042381.2	lncRNA
XM_015101730.1	ZNF280B	mRNA	XR_001042381.2	lncRNA
XM_015101730.1	ZNF280B	mRNA	XR_001042381.2	lncRNA
XM_015101730.1	ZNF280B	mRNA	XR_001042381.2	lncRNA
XM_015101730.1	ZNF280B	mRNA	XR_001042381.2	lncRNA
XM_012107496.2	PLS3	mRNA	XR_001436532.1	lncRNA
XM_012107496.2	PLS3	mRNA	XR_001436532.1	lncRNA
XM_012107496.2	PLS3	mRNA	XR_001436532.1	lncRNA
XM_012107496.2	PLS3	mRNA	XR_001436532.1	lncRNA
XM_015098144.1	HSPH1	mRNA	XR_001436532.1	lncRNA
XM_015098144.1	HSPH1	mRNA	XR_001436532.1	lncRNA
XM_015098144.1	HSPH1	mRNA	XR_001436532.1	lncRNA
XM_015098144.1	HSPH1	mRNA	XR_001436532.1	lncRNA
XM_012185695.2	RANGRF	mRNA	XR_001436532.1	lncRNA
XM_012185695.2	RANGRF	mRNA	XR_001436532.1	lncRNA
XM_012185695.2	RANGRF	mRNA	XR_001436532.1	lncRNA
XM_012185695.2	RANGRF	mRNA	XR_001436532.1	lncRNA
XM_012177026.2	FAM3C	mRNA	XR_001436532.1	lncRNA
XM_012177026.2	FAM3C	mRNA	XR_001436532.1	lncRNA
XM_012177026.2	FAM3C	mRNA	XR_001436532.1	lncRNA
XM_012177026.2	FAM3C	mRNA	XR_001436532.1	lncRNA
XM_004022454.2	TCEAL8	mRNA	XR_001436532.1	lncRNA
XM_004022454.2	TCEAL8	mRNA	XR_001436532.1	lncRNA
XM_004022454.2	TCEAL8	mRNA	XR_001436532.1	lncRNA
XM_004022454.2	TCEAL8	mRNA	XR_001436532.1	lncRNA
XM_015101080.1	PGM2L1	mRNA	XR_001436532.1	lncRNA
XM_015101080.1	PGM2L1	mRNA	XR_001436532.1	lncRNA
XM_015101080.1	PGM2L1	mRNA	XR_001436532.1	lncRNA
XM_015101080.1	PGM2L1	mRNA	XR_001436532.1	lncRNA
XM_012184216.2	MTBP	mRNA	XR_001033284.2	lncRNA
XM_012184216.2	MTBP	mRNA	XR_001033284.2	lncRNA
XM_012184216.2	MTBP	mRNA	XR_001033284.2	lncRNA
XM_012184216.2	MTBP	mRNA	XR_001033284.2	lncRNA
XM_004003552.3	LOC10112182(	mRNA	XR_001024743.2	lncRNA
XM_004003552.3	LOC10112182(	mRNA	XR_001024743.2	lncRNA
XM_004003552.3	LOC10112182(	mRNA	XR_001024743.2	lncRNA
XM_004003552.3	LOC10112182(	mRNA	XR_001024743.2	lncRNA
XM_012119517.2	SORBS3	mRNA	TCONS_00084305	lncRNA
XM_012119517.2	SORBS3	mRNA	TCONS_00084305	lncRNA
XM_012119517.2	SORBS3	mRNA	TCONS_00084305	lncRNA
XM_012119517.2	SORBS3	mRNA	TCONS_00084305	lncRNA
XM_012102845.2	GBF1	mRNA	TCONS_00078442	lncRNA
XM_012102845.2	GBF1	mRNA	TCONS_00078442	lncRNA
XM_012102845.2	GBF1	mRNA	TCONS_00078442	lncRNA
XM_012102845.2	GBF1	mRNA	TCONS_00078442	lncRNA
XM_015094203.1	RC3H2	mRNA	TCONS_00054422	lncRNA
XM_015094203.1	RC3H2	mRNA	TCONS_00054422	lncRNA
XM_015094203.1	RC3H2	mRNA	TCONS_00054422	lncRNA
XM_015094203.1	RC3H2	mRNA	TCONS_00054422	lncRNA
XM_012134416.2	PPIG	mRNA	TCONS_00078442	lncRNA
XM_012134416.2	PPIG	mRNA	TCONS_00078442	lncRNA
XM_012134416.2	PPIG	mRNA	TCONS_00078442	lncRNA
XM_012134416.2	PPIG	mRNA	TCONS_00078442	lncRNA
XM_012184884.2	DOCK9	mRNA	XR_001436931.1	lncRNA

XM_012184884.2	DOCK9	mRNA	XR_001436931.1	lncRNA
XM_012184884.2	DOCK9	mRNA	XR_001436931.1	lncRNA
XM_012184884.2	DOCK9	mRNA	XR_001436931.1	lncRNA
XM_012100690.1	KLC4	mRNA	XR_001436931.1	lncRNA
XM_012100690.1	KLC4	mRNA	XR_001436931.1	lncRNA
XM_012100690.1	KLC4	mRNA	XR_001436931.1	lncRNA
XM_012100690.1	KLC4	mRNA	XR_001436931.1	lncRNA
XM_012188301.2	NEBL	mRNA	XR_001436931.1	lncRNA
XM_012188301.2	NEBL	mRNA	XR_001436931.1	lncRNA
XM_012188301.2	NEBL	mRNA	XR_001436931.1	lncRNA
XM_012188301.2	NEBL	mRNA	XR_001436931.1	lncRNA
XM_012173780.2	REV1	mRNA	TCONS_00084305	lncRNA
XM_012173780.2	REV1	mRNA	TCONS_00084305	lncRNA
XM_012173780.2	REV1	mRNA	TCONS_00084305	lncRNA
XM_012173780.2	REV1	mRNA	TCONS_00084305	lncRNA
XM_004020074.3	PIK3AP1	mRNA	XR_001436931.1	lncRNA
XM_004020074.3	PIK3AP1	mRNA	XR_001436931.1	lncRNA
XM_004020074.3	PIK3AP1	mRNA	XR_001436931.1	lncRNA
XM_004020074.3	PIK3AP1	mRNA	XR_001436931.1	lncRNA
XM_012105656.2	ZNF596	mRNA	TCONS_00078442	lncRNA
XM_012105656.2	ZNF596	mRNA	TCONS_00078442	lncRNA
XM_012105656.2	ZNF596	mRNA	TCONS_00078442	lncRNA
XM_012105656.2	ZNF596	mRNA	TCONS_00078442	lncRNA
XM_015101031.1	MICAL2	mRNA	TCONS_00054422	lncRNA
XM_015101031.1	MICAL2	mRNA	TCONS_00054422	lncRNA
XM_015101031.1	MICAL2	mRNA	TCONS_00054422	lncRNA
XM_015101031.1	MICAL2	mRNA	TCONS_00054422	lncRNA
XM_012173296.2	CDKL4	mRNA	TCONS_00084305	lncRNA
XM_012173296.2	CDKL4	mRNA	TCONS_00084305	lncRNA
XM_012173296.2	CDKL4	mRNA	TCONS_00084305	lncRNA
XM_012173296.2	CDKL4	mRNA	TCONS_00084305	lncRNA
XM_012184885.2	DOCK9	mRNA	XR_001436931.1	lncRNA
XM_012184885.2	DOCK9	mRNA	XR_001436931.1	lncRNA
XM_012184885.2	DOCK9	mRNA	XR_001436931.1	lncRNA
XM_012184885.2	DOCK9	mRNA	XR_001436931.1	lncRNA
XM_012134416.2	PPIG	mRNA	TCONS_00084305	lncRNA
XM_012134416.2	PPIG	mRNA	TCONS_00084305	lncRNA
XM_012134416.2	PPIG	mRNA	TCONS_00084305	lncRNA
XM_012134416.2	PPIG	mRNA	TCONS_00084305	lncRNA
XM_015098144.1	HSPH1	mRNA	XR_001041211.2	lncRNA
XM_015098144.1	HSPH1	mRNA	XR_001041211.2	lncRNA
XM_015098144.1	HSPH1	mRNA	XR_001041211.2	lncRNA
XM_015098144.1	HSPH1	mRNA	XR_001041211.2	lncRNA
XM_012177026.2	FAM3C	mRNA	XR_001041211.2	lncRNA
XM_012177026.2	FAM3C	mRNA	XR_001041211.2	lncRNA
XM_012177026.2	FAM3C	mRNA	XR_001041211.2	lncRNA
XM_012177026.2	FAM3C	mRNA	XR_001041211.2	lncRNA
XM_004022454.2	TCEAL8	mRNA	XR_001041211.2	lncRNA
XM_004022454.2	TCEAL8	mRNA	XR_001041211.2	lncRNA
XM_004022454.2	TCEAL8	mRNA	XR_001041211.2	lncRNA
XM_004022454.2	TCEAL8	mRNA	XR_001041211.2	lncRNA
XM_012153155.2	HP1BP3	mRNA	TCONS_00039304	lncRNA
XM_012153155.2	HP1BP3	mRNA	TCONS_00039304	lncRNA
XM_012153155.2	HP1BP3	mRNA	TCONS_00039304	lncRNA

XM_004014443.3	RAE1	mRNA	TCONS_00039304	lncRNA
XM_004014443.3	RAE1	mRNA	TCONS_00039304	lncRNA
XM_004014443.3	RAE1	mRNA	TCONS_00039304	lncRNA
XM_004008964.3	LOC101121514	mRNA	XR_001436931.1	lncRNA
XM_004008964.3	LOC101121514	mRNA	XR_001436931.1	lncRNA
XM_004008964.3	LOC101121514	mRNA	XR_001436931.1	lncRNA
XM_012099038.2	SLC25A29	mRNA	TCONS_00084305	lncRNA
XM_012099038.2	SLC25A29	mRNA	TCONS_00084305	lncRNA
XM_012099038.2	SLC25A29	mRNA	TCONS_00084305	lncRNA
XM_012099038.2	SLC25A29	mRNA	TCONS_00084305	lncRNA
XM_012179696.2	LOC101112864	mRNA	XR_001436931.1	lncRNA
XM_012179696.2	LOC101112864	mRNA	XR_001436931.1	lncRNA
XM_012179696.2	LOC101112864	mRNA	XR_001436931.1	lncRNA
XM_012179696.2	LOC101112864	mRNA	XR_001436931.1	lncRNA
XM_012152472.1	LUZP1	mRNA	TCONS_00082638	lncRNA
XM_012152472.1	LUZP1	mRNA	TCONS_00082638	lncRNA
XM_012152472.1	LUZP1	mRNA	TCONS_00082638	lncRNA
XM_012152472.1	LUZP1	mRNA	TCONS_00082638	lncRNA
XM_015098618.1	DLG4	mRNA	TCONS_00082638	lncRNA
XM_015098618.1	DLG4	mRNA	TCONS_00082638	lncRNA
XM_015098618.1	DLG4	mRNA	TCONS_00082638	lncRNA
XM_015098618.1	DLG4	mRNA	TCONS_00082638	lncRNA
XM_012152472.1	LUZP1	mRNA	XR_001436931.1	lncRNA
XM_012152472.1	LUZP1	mRNA	XR_001436931.1	lncRNA
XM_012152472.1	LUZP1	mRNA	XR_001436931.1	lncRNA
XM_012152472.1	LUZP1	mRNA	XR_001436931.1	lncRNA
XM_012134593.2	FASTKD1	mRNA	XR_001436102.1	lncRNA
XM_012134593.2	FASTKD1	mRNA	XR_001436102.1	lncRNA
XM_012134593.2	FASTKD1	mRNA	XR_001436102.1	lncRNA
XM_012134593.2	FASTKD1	mRNA	XR_001436102.1	lncRNA
XM_015098003.1	LOC10111962[illegible]	mRNA	XR_001436102.1	lncRNA
XM_015098003.1	LOC10111962[illegible]	mRNA	XR_001436102.1	lncRNA
XM_015098003.1	LOC10111962[illegible]	mRNA	XR_001436102.1	lncRNA
XM_015098003.1	LOC10111962[illegible]	mRNA	XR_001436102.1	lncRNA
XM_012119403.2	BIN3	mRNA	XR_001436102.1	lncRNA
XM_012119403.2	BIN3	mRNA	XR_001436102.1	lncRNA
XM_012119403.2	BIN3	mRNA	XR_001436102.1	lncRNA
XM_012119403.2	BIN3	mRNA	XR_001436102.1	lncRNA
XM_012098757.1	G2E3	mRNA	XR_001436102.1	lncRNA
XM_012098757.1	G2E3	mRNA	XR_001436102.1	lncRNA
XM_012098757.1	G2E3	mRNA	XR_001436102.1	lncRNA
XM_012098757.1	G2E3	mRNA	XR_001436102.1	lncRNA
XM_012098754.1	G2E3	mRNA	XR_001436102.1	lncRNA
XM_012098754.1	G2E3	mRNA	XR_001436102.1	lncRNA
XM_012098754.1	G2E3	mRNA	XR_001436102.1	lncRNA
XM_012098754.1	G2E3	mRNA	XR_001436102.1	lncRNA
XM_012184823.2	RBM26	mRNA	TCONS_00078440	lncRNA
XM_012184823.2	RBM26	mRNA	TCONS_00078440	lncRNA
XM_012184823.2	RBM26	mRNA	TCONS_00078440	lncRNA
XM_012184823.2	RBM26	mRNA	TCONS_00078440	lncRNA
XM_015100438.1	CYLD	mRNA	TCONS_00078440	lncRNA
XM_015100438.1	CYLD	mRNA	TCONS_00078440	lncRNA
XM_015100438.1	CYLD	mRNA	TCONS_00078440	lncRNA
XM_015100438.1	CYLD	mRNA	TCONS_00078440	lncRNA

XM_015104948.1	RPS6KA6	mRNA	TCONS_00075453	lncRNA
XM_015104948.1	RPS6KA6	mRNA	TCONS_00075453	lncRNA
XM_015104948.1	RPS6KA6	mRNA	TCONS_00075453	lncRNA
XM_015104948.1	RPS6KA6	mRNA	TCONS_00075453	lncRNA
XM_012135865.2	TANK	mRNA	XR_001436817.1	lncRNA
XM_012135865.2	TANK	mRNA	XR_001436817.1	lncRNA
XM_012135865.2	TANK	mRNA	XR_001436817.1	lncRNA
XM_012135865.2	TANK	mRNA	XR_001436817.1	lncRNA
XM_015101730.1	ZNF280B	mRNA	XR_001436817.1	lncRNA
XM_015101730.1	ZNF280B	mRNA	XR_001436817.1	lncRNA
XM_015101730.1	ZNF280B	mRNA	XR_001436817.1	lncRNA
XM_015101730.1	ZNF280B	mRNA	XR_001436817.1	lncRNA
XM_012174040.2	SLC6A15	mRNA	XR_001436817.1	lncRNA
XM_012174040.2	SLC6A15	mRNA	XR_001436817.1	lncRNA
XM_012174040.2	SLC6A15	mRNA	XR_001436817.1	lncRNA
XM_012174040.2	SLC6A15	mRNA	XR_001436817.1	lncRNA
XM_012095725.2	BTBD10	mRNA	XR_001436817.1	lncRNA
XM_012095725.2	BTBD10	mRNA	XR_001436817.1	lncRNA
XM_012095725.2	BTBD10	mRNA	XR_001436817.1	lncRNA
XM_012095725.2	BTBD10	mRNA	XR_001436817.1	lncRNA
XM_012185695.2	RANGRF	mRNA	XR_001436817.1	lncRNA
XM_012185695.2	RANGRF	mRNA	XR_001436817.1	lncRNA
XM_012185695.2	RANGRF	mRNA	XR_001436817.1	lncRNA
XM_012185695.2	RANGRF	mRNA	XR_001436817.1	lncRNA
XM_012107496.2	PLS3	mRNA	XR_001436817.1	lncRNA
XM_012107496.2	PLS3	mRNA	XR_001436817.1	lncRNA
XM_012107496.2	PLS3	mRNA	XR_001436817.1	lncRNA
XM_012107496.2	PLS3	mRNA	XR_001436817.1	lncRNA
XM_012174289.1	ATF1	mRNA	XR_001436817.1	lncRNA
XM_012174289.1	ATF1	mRNA	XR_001436817.1	lncRNA
XM_012174289.1	ATF1	mRNA	XR_001436817.1	lncRNA
XM_012174289.1	ATF1	mRNA	XR_001436817.1	lncRNA
XM_012183145.1	MTRF1L	mRNA	XR_001436817.1	lncRNA
XM_012183145.1	MTRF1L	mRNA	XR_001436817.1	lncRNA
XM_012183145.1	MTRF1L	mRNA	XR_001436817.1	lncRNA
XM_012183145.1	MTRF1L	mRNA	XR_001436817.1	lncRNA
XM_012183965.2	OXR1	mRNA	XR_001436817.1	lncRNA
XM_012183965.2	OXR1	mRNA	XR_001436817.1	lncRNA
XM_012183965.2	OXR1	mRNA	XR_001436817.1	lncRNA
XM_012183965.2	OXR1	mRNA	XR_001436817.1	lncRNA
XM_012174040.2	SLC6A15	mRNA	XR_001434203.1	lncRNA
XM_012174040.2	SLC6A15	mRNA	XR_001434203.1	lncRNA
XM_012174040.2	SLC6A15	mRNA	XR_001434203.1	lncRNA
XM_012174040.2	SLC6A15	mRNA	XR_001434203.1	lncRNA
XM_012183965.2	OXR1	mRNA	XR_001434203.1	lncRNA
XM_012183965.2	OXR1	mRNA	XR_001434203.1	lncRNA
XM_012183965.2	OXR1	mRNA	XR_001434203.1	lncRNA
XM_012183965.2	OXR1	mRNA	XR_001434203.1	lncRNA
XM_012182469.2	ECHDC1	mRNA	XR_001434203.1	lncRNA
XM_012182469.2	ECHDC1	mRNA	XR_001434203.1	lncRNA
XM_012182469.2	ECHDC1	mRNA	XR_001434203.1	lncRNA
XM_012182469.2	ECHDC1	mRNA	XR_001434203.1	lncRNA
XM_004002454.2	ECM1	mRNA	XR_001436817.1	lncRNA
XM_004002454.2	ECM1	mRNA	XR_001436817.1	lncRNA

XM_004002454.2	ECM1	mRNA	XR_001436817.1	lncRNA
XM_004002454.2	ECM1	mRNA	XR_001436817.1	lncRNA
XM_012177026.2	FAM3C	mRNA	XR_001436817.1	lncRNA
XM_012177026.2	FAM3C	mRNA	XR_001436817.1	lncRNA
XM_012177026.2	FAM3C	mRNA	XR_001436817.1	lncRNA
XM_012177026.2	FAM3C	mRNA	XR_001436817.1	lncRNA
XM_015094624.1	PCBP2	mRNA	XR_001436127.1	lncRNA
XM_015094624.1	PCBP2	mRNA	XR_001436127.1	lncRNA
XM_015094624.1	PCBP2	mRNA	XR_001436127.1	lncRNA
XM_015094624.1	PCBP2	mRNA	XR_001436127.1	lncRNA
XM_012148796.2	DIS3L2	mRNA	XR_001025491.1	lncRNA
XM_012148796.2	DIS3L2	mRNA	XR_001025491.1	lncRNA
XM_012148796.2	DIS3L2	mRNA	XR_001025491.1	lncRNA
XM_012148796.2	DIS3L2	mRNA	XR_001025491.1	lncRNA
XM_015100879.1	ARHGAP42	mRNA	XR_001025491.1	lncRNA
XM_015100879.1	ARHGAP42	mRNA	XR_001025491.1	lncRNA
XM_015100879.1	ARHGAP42	mRNA	XR_001025491.1	lncRNA
XM_015100879.1	ARHGAP42	mRNA	XR_001025491.1	lncRNA
XM_015100879.1	ARHGAP42	mRNA	XR_001024290.1	lncRNA
XM_015100879.1	ARHGAP42	mRNA	XR_001024290.1	lncRNA
XM_015100879.1	ARHGAP42	mRNA	XR_001024290.1	lncRNA
XM_015100879.1	ARHGAP42	mRNA	XR_001024290.1	lncRNA
XM_012171189.2	MIER1	mRNA	XR_001024290.1	lncRNA
XM_012171189.2	MIER1	mRNA	XR_001024290.1	lncRNA
XM_012171189.2	MIER1	mRNA	XR_001024290.1	lncRNA
XM_012171189.2	MIER1	mRNA	XR_001024290.1	lncRNA
XM_015103591.1	KIAA1328	mRNA	XR_001024290.1	lncRNA
XM_015103591.1	KIAA1328	mRNA	XR_001024290.1	lncRNA
XM_015103591.1	KIAA1328	mRNA	XR_001024290.1	lncRNA
XM_015103591.1	KIAA1328	mRNA	XR_001024290.1	lncRNA
XM_015099544.1	SEC61A2	mRNA	TCONS_00022613	lncRNA
XM_015099544.1	SEC61A2	mRNA	TCONS_00022613	lncRNA
XM_015099544.1	SEC61A2	mRNA	TCONS_00022613	lncRNA
XM_015099544.1	SEC61A2	mRNA	TCONS_00022613	lncRNA
XM_012105261.2	MCU	mRNA	TCONS_00022613	lncRNA
XM_012105261.2	MCU	mRNA	TCONS_00022613	lncRNA
XM_012105261.2	MCU	mRNA	TCONS_00022613	lncRNA
XM_012105261.2	MCU	mRNA	TCONS_00022613	lncRNA
XM_015098766.1	WIPF2	mRNA	TCONS_00022613	lncRNA
XM_015098766.1	WIPF2	mRNA	TCONS_00022613	lncRNA
XM_015098766.1	WIPF2	mRNA	TCONS_00022613	lncRNA
XM_015098766.1	WIPF2	mRNA	TCONS_00022613	lncRNA
XM_012186029.1	FBXL20	mRNA	TCONS_00022613	lncRNA
XM_012186029.1	FBXL20	mRNA	TCONS_00022613	lncRNA
XM_012186029.1	FBXL20	mRNA	TCONS_00022613	lncRNA
XM_012186029.1	FBXL20	mRNA	TCONS_00022613	lncRNA
XM_012185318.2	RAD51D	mRNA	TCONS_00084305	lncRNA
XM_012185318.2	RAD51D	mRNA	TCONS_00084305	lncRNA
XM_012185318.2	RAD51D	mRNA	TCONS_00084305	lncRNA
XM_012185318.2	RAD51D	mRNA	TCONS_00084305	lncRNA
XM_012177920.2	KANK2	mRNA	TCONS_00054422	lncRNA
XM_012177920.2	KANK2	mRNA	TCONS_00054422	lncRNA
XM_012177920.2	KANK2	mRNA	TCONS_00054422	lncRNA
XM_012177920.2	KANK2	mRNA	TCONS_00054422	lncRNA

XM_012176498.2	RAPGEF5	mRNA	TCONS_00084305	lncRNA
XM_012176498.2	RAPGEF5	mRNA	TCONS_00084305	lncRNA
XM_012176498.2	RAPGEF5	mRNA	TCONS_00084305	lncRNA
XM_012176498.2	RAPGEF5	mRNA	TCONS_00084305	lncRNA
XM_015091997.1	LOC10112163[illegible]	mRNA	TCONS_00082638	lncRNA
XM_015091997.1	LOC10112163[illegible]	mRNA	TCONS_00082638	lncRNA
XM_015091997.1	LOC10112163[illegible]	mRNA	TCONS_00082638	lncRNA
XM_015091997.1	LOC10112163[illegible]	mRNA	TCONS_00082638	lncRNA
XM_015100041.1	ZNF473	mRNA	XR_001024743.2	lncRNA
XM_015100041.1	ZNF473	mRNA	XR_001024743.2	lncRNA
XM_015100041.1	ZNF473	mRNA	XR_001024743.2	lncRNA
XM_015100041.1	ZNF473	mRNA	XR_001024743.2	lncRNA
XM_015104948.1	RPS6KA6	mRNA	XR_001033284.2	lncRNA
XM_015104948.1	RPS6KA6	mRNA	XR_001033284.2	lncRNA
XM_015104948.1	RPS6KA6	mRNA	XR_001033284.2	lncRNA
XM_015104948.1	RPS6KA6	mRNA	XR_001033284.2	lncRNA
XM_015092883.1	PADI1	mRNA	TCONS_00078440	lncRNA
XM_015092883.1	PADI1	mRNA	TCONS_00078440	lncRNA
XM_015092883.1	PADI1	mRNA	TCONS_00078440	lncRNA
XM_015092883.1	PADI1	mRNA	TCONS_00078440	lncRNA
XM_012174160.2	FUBP1	mRNA	TCONS_00078440	lncRNA
XM_012174160.2	FUBP1	mRNA	TCONS_00078440	lncRNA
XM_012174160.2	FUBP1	mRNA	TCONS_00078440	lncRNA
XM_012174160.2	FUBP1	mRNA	TCONS_00078440	lncRNA
XM_012102835.2	PPRC1	mRNA	TCONS_00078440	lncRNA
XM_012102835.2	PPRC1	mRNA	TCONS_00078440	lncRNA
XM_012102835.2	PPRC1	mRNA	TCONS_00078440	lncRNA
XM_012102835.2	PPRC1	mRNA	TCONS_00078440	lncRNA
XM_012095858.2	ZNF214	mRNA	TCONS_00078440	lncRNA
XM_012095858.2	ZNF214	mRNA	TCONS_00078440	lncRNA
XM_012095858.2	ZNF214	mRNA	TCONS_00078440	lncRNA
XM_012095858.2	ZNF214	mRNA	TCONS_00078440	lncRNA
XM_012102835.2	PPRC1	mRNA	XR_001433755.1	lncRNA
XM_012102835.2	PPRC1	mRNA	XR_001433755.1	lncRNA
XM_012102835.2	PPRC1	mRNA	XR_001433755.1	lncRNA
XM_012102835.2	PPRC1	mRNA	XR_001433755.1	lncRNA
XM_012179910.2	ATP10D	mRNA	XR_001433755.1	lncRNA
XM_012179910.2	ATP10D	mRNA	XR_001433755.1	lncRNA
XM_012179910.2	ATP10D	mRNA	XR_001433755.1	lncRNA
XM_012179910.2	ATP10D	mRNA	XR_001433755.1	lncRNA
XM_012095502.1	SIK3	mRNA	TCONS_00078442	lncRNA
XM_012095502.1	SIK3	mRNA	TCONS_00078442	lncRNA
XM_012095502.1	SIK3	mRNA	TCONS_00078442	lncRNA
XM_012095502.1	SIK3	mRNA	TCONS_00078442	lncRNA
XM_012180879.2	MYO9A	mRNA	TCONS_00082638	lncRNA
XM_012180879.2	MYO9A	mRNA	TCONS_00082638	lncRNA
XM_012180879.2	MYO9A	mRNA	TCONS_00082638	lncRNA
XM_012180879.2	MYO9A	mRNA	TCONS_00082638	lncRNA
XM_015092323.1	KIRREL	mRNA	TCONS_00084305	lncRNA
XM_015092323.1	KIRREL	mRNA	TCONS_00084305	lncRNA
XM_015092323.1	KIRREL	mRNA	TCONS_00084305	lncRNA
XM_015092323.1	KIRREL	mRNA	TCONS_00084305	lncRNA
XM_012185285.2	SYNRG	mRNA	TCONS_00054422	lncRNA
XM_012185285.2	SYNRG	mRNA	TCONS_00054422	lncRNA

XM_012185285.2	SYNRG	mRNA	TCONS_00054422	lncRNA
XM_012185285.2	SYNRG	mRNA	TCONS_00054422	lncRNA
XM_012186944.1	DSTYK	mRNA	TCONS_00054422	lncRNA
XM_012186944.1	DSTYK	mRNA	TCONS_00054422	lncRNA
XM_012186944.1	DSTYK	mRNA	TCONS_00054422	lncRNA
XM_012186944.1	DSTYK	mRNA	TCONS_00054422	lncRNA
XM_015104327.1	LOC10111542[illegible]	mRNA	TCONS_00075453	lncRNA
XM_015104327.1	LOC10111542[illegible]	mRNA	TCONS_00075453	lncRNA
XM_015104327.1	LOC10111542[illegible]	mRNA	TCONS_00075453	lncRNA
XM_015104327.1	LOC10111542[illegible]	mRNA	TCONS_00075453	lncRNA
XM_015101031.1	MICAL2	mRNA	XR_001024743.2	lncRNA
XM_015101031.1	MICAL2	mRNA	XR_001024743.2	lncRNA
XM_015101031.1	MICAL2	mRNA	XR_001024743.2	lncRNA
XM_015101031.1	MICAL2	mRNA	XR_001024743.2	lncRNA
XM_015094203.1	RC3H2	mRNA	XR_001024743.2	lncRNA
XM_015094203.1	RC3H2	mRNA	XR_001024743.2	lncRNA
XM_015094203.1	RC3H2	mRNA	XR_001024743.2	lncRNA
XM_015094203.1	RC3H2	mRNA	XR_001024743.2	lncRNA
XM_004010712.2	WDR89	mRNA	XR_001434761.1	lncRNA
XM_004010712.2	WDR89	mRNA	XR_001434761.1	lncRNA
XM_004010712.2	WDR89	mRNA	XR_001434761.1	lncRNA
XM_004010712.2	WDR89	mRNA	XR_001434761.1	lncRNA
XM_012182435.2	C1H1orf43	mRNA	XR_001434892.1	lncRNA
XM_012182435.2	C1H1orf43	mRNA	XR_001434892.1	lncRNA
XM_012182435.2	C1H1orf43	mRNA	XR_001434892.1	lncRNA
XM_012182435.2	C1H1orf43	mRNA	XR_001434892.1	lncRNA
XM_012098754.1	G2E3	mRNA	XR_001434761.1	lncRNA
XM_012098754.1	G2E3	mRNA	XR_001434761.1	lncRNA
XM_012098754.1	G2E3	mRNA	XR_001434761.1	lncRNA
XM_012098754.1	G2E3	mRNA	XR_001434761.1	lncRNA
XM_015096937.1	BMF	mRNA	XR_001436931.1	lncRNA
XM_015096937.1	BMF	mRNA	XR_001436931.1	lncRNA
XM_015096937.1	BMF	mRNA	XR_001436931.1	lncRNA
XM_015096937.1	BMF	mRNA	XR_001436931.1	lncRNA
XM_015096937.1	BMF	mRNA	TCONS_00084305	lncRNA
XM_015096937.1	BMF	mRNA	TCONS_00084305	lncRNA
XM_015096937.1	BMF	mRNA	TCONS_00084305	lncRNA
XM_015096937.1	BMF	mRNA	TCONS_00084305	lncRNA
XM_012137425.2	NEB	mRNA	TCONS_00054422	lncRNA
XM_012137425.2	NEB	mRNA	TCONS_00054422	lncRNA
XM_012137425.2	NEB	mRNA	TCONS_00054422	lncRNA
XM_012137425.2	NEB	mRNA	TCONS_00054422	lncRNA
XM_012179034.2	FER	mRNA	TCONS_00084305	lncRNA
XM_012179034.2	FER	mRNA	TCONS_00084305	lncRNA
XM_012179034.2	FER	mRNA	TCONS_00084305	lncRNA
XM_012179034.2	FER	mRNA	TCONS_00084305	lncRNA
XM_012185926.2	DLX4	mRNA	TCONS_00078442	lncRNA
XM_012185926.2	DLX4	mRNA	TCONS_00078442	lncRNA
XM_012185926.2	DLX4	mRNA	TCONS_00078442	lncRNA
XM_012185926.2	DLX4	mRNA	TCONS_00078442	lncRNA
XM_015103753.1	MBD1	mRNA	TCONS_00022613	lncRNA
XM_015103753.1	MBD1	mRNA	TCONS_00022613	lncRNA
XM_015103753.1	MBD1	mRNA	TCONS_00022613	lncRNA
XM_015103753.1	MBD1	mRNA	TCONS_00022613	lncRNA

XM_012099341.2	CLASP2	mRNA	TCONS_00022613	lncRNA
XM_012099341.2	CLASP2	mRNA	TCONS_00022613	lncRNA
XM_012099341.2	CLASP2	mRNA	TCONS_00022613	lncRNA
XM_012099341.2	CLASP2	mRNA	TCONS_00022613	lncRNA
XM_015091902.1	SCMH1	mRNA	XR_001433755.1	lncRNA
XM_015091902.1	SCMH1	mRNA	XR_001433755.1	lncRNA
XM_015091902.1	SCMH1	mRNA	XR_001433755.1	lncRNA
XM_015091902.1	SCMH1	mRNA	XR_001433755.1	lncRNA
XM_015092421.1	BBX	mRNA	TCONS_00078440	lncRNA
XM_015092421.1	BBX	mRNA	TCONS_00078440	lncRNA
XM_015092421.1	BBX	mRNA	TCONS_00078440	lncRNA
XM_015092421.1	BBX	mRNA	TCONS_00078440	lncRNA
XM_015091991.1	YIPF1	mRNA	XR_001436127.1	lncRNA
XM_015091991.1	YIPF1	mRNA	XR_001436127.1	lncRNA
XM_015091991.1	YIPF1	mRNA	XR_001436127.1	lncRNA
XM_015091991.1	YIPF1	mRNA	XR_001436127.1	lncRNA
XM_012143285.2	ABI2	mRNA	XR_001025491.1	lncRNA
XM_012143285.2	ABI2	mRNA	XR_001025491.1	lncRNA
XM_012143285.2	ABI2	mRNA	XR_001025491.1	lncRNA
XM_012143285.2	ABI2	mRNA	XR_001025491.1	lncRNA
XM_012181234.2	NDUFAF1	mRNA	XR_001436127.1	lncRNA
XM_012181234.2	NDUFAF1	mRNA	XR_001436127.1	lncRNA
XM_012181234.2	NDUFAF1	mRNA	XR_001436127.1	lncRNA
XM_012181234.2	NDUFAF1	mRNA	XR_001436127.1	lncRNA
XM_015103448.1	TACC2	mRNA	TCONS_00084305	lncRNA
XM_015103448.1	TACC2	mRNA	TCONS_00084305	lncRNA
XM_015103448.1	TACC2	mRNA	TCONS_00084305	lncRNA
XM_015103448.1	TACC2	mRNA	TCONS_00084305	lncRNA
XM_015091996.1	LOC10112163[illegible]	mRNA	TCONS_00054422	lncRNA
XM_015091996.1	LOC10112163[illegible]	mRNA	TCONS_00054422	lncRNA
XM_015091996.1	LOC10112163[illegible]	mRNA	TCONS_00054422	lncRNA
XM_015091996.1	LOC10112163[illegible]	mRNA	TCONS_00054422	lncRNA
XM_015098575.1	METTL16	mRNA	XR_001021689.2	lncRNA
XM_015098575.1	METTL16	mRNA	XR_001021689.2	lncRNA
XM_015098575.1	METTL16	mRNA	XR_001021689.2	lncRNA
XM_015098575.1	METTL16	mRNA	XR_001021689.2	lncRNA
XM_004021989.3	DMD	mRNA	XR_001436102.1	lncRNA
XM_004021989.3	DMD	mRNA	XR_001436102.1	lncRNA
XM_004021989.3	DMD	mRNA	XR_001436102.1	lncRNA
XM_004021989.3	DMD	mRNA	XR_001436102.1	lncRNA
XM_012184216.2	MTBP	mRNA	XR_001436102.1	lncRNA
XM_012184216.2	MTBP	mRNA	XR_001436102.1	lncRNA
XM_012184216.2	MTBP	mRNA	XR_001436102.1	lncRNA
XM_012184216.2	MTBP	mRNA	XR_001436102.1	lncRNA
XM_012148796.2	DIS3L2	mRNA	XR_001436102.1	lncRNA
XM_012148796.2	DIS3L2	mRNA	XR_001436102.1	lncRNA
XM_012148796.2	DIS3L2	mRNA	XR_001436102.1	lncRNA
XM_012148796.2	DIS3L2	mRNA	XR_001436102.1	lncRNA
XM_015099256.1	CEP104	mRNA	XR_001024743.2	lncRNA
XM_015099256.1	CEP104	mRNA	XR_001024743.2	lncRNA
XM_015099256.1	CEP104	mRNA	XR_001024743.2	lncRNA
XM_015099256.1	CEP104	mRNA	XR_001024743.2	lncRNA
XM_012100104.2	NME6	mRNA	TCONS_00078440	lncRNA
XM_012100104.2	NME6	mRNA	TCONS_00078440	lncRNA

XM_012100104.2	NME6	mRNA	TCONS_00078440	lncRNA
XM_012170864.2	PAPOLG	mRNA	XR_001020707.2	lncRNA
XM_012170864.2	PAPOLG	mRNA	XR_001020707.2	lncRNA
XM_012170864.2	PAPOLG	mRNA	XR_001020707.2	lncRNA
XM_012170864.2	PAPOLG	mRNA	XR_001020707.2	lncRNA
XM_012186029.1	FBXL20	mRNA	XR_001020707.2	lncRNA
XM_012186029.1	FBXL20	mRNA	XR_001020707.2	lncRNA
XM_012186029.1	FBXL20	mRNA	XR_001020707.2	lncRNA
XM_012186029.1	FBXL20	mRNA	XR_001020707.2	lncRNA
XM_012188945.2	CSTF1	mRNA	XR_001033284.2	lncRNA
XM_012188945.2	CSTF1	mRNA	XR_001033284.2	lncRNA
XM_012188945.2	CSTF1	mRNA	XR_001033284.2	lncRNA
XM_012188945.2	CSTF1	mRNA	XR_001033284.2	lncRNA
XM_012099283.2	NEK10	mRNA	XR_001033284.2	lncRNA
XM_012099283.2	NEK10	mRNA	XR_001033284.2	lncRNA
XM_012099283.2	NEK10	mRNA	XR_001033284.2	lncRNA
XM_012099283.2	NEK10	mRNA	XR_001033284.2	lncRNA
XM_004014209.3	CUL2	mRNA	XR_001033284.2	lncRNA
XM_004014209.3	CUL2	mRNA	XR_001033284.2	lncRNA
XM_004014209.3	CUL2	mRNA	XR_001033284.2	lncRNA
XM_004014209.3	CUL2	mRNA	XR_001033284.2	lncRNA
XM_012107644.2	FAM199X	mRNA	XR_001436532.1	lncRNA
XM_012107644.2	FAM199X	mRNA	XR_001436532.1	lncRNA
XM_012107644.2	FAM199X	mRNA	XR_001436532.1	lncRNA
XM_012107644.2	FAM199X	mRNA	XR_001436532.1	lncRNA
XM_012134593.2	FASTKD1	mRNA	XR_001436532.1	lncRNA
XM_012134593.2	FASTKD1	mRNA	XR_001436532.1	lncRNA
XM_012134593.2	FASTKD1	mRNA	XR_001436532.1	lncRNA
XM_012134593.2	FASTKD1	mRNA	XR_001436532.1	lncRNA
XM_015098592.1	RABEP1	mRNA	TCONS_00078440	lncRNA
XM_015098592.1	RABEP1	mRNA	TCONS_00078440	lncRNA
XM_015098592.1	RABEP1	mRNA	TCONS_00078440	lncRNA
XM_015098592.1	RABEP1	mRNA	TCONS_00078440	lncRNA
XM_015096385.1	TBCK	mRNA	TCONS_00084305	lncRNA
XM_015096385.1	TBCK	mRNA	TCONS_00084305	lncRNA
XM_015096385.1	TBCK	mRNA	TCONS_00084305	lncRNA
XM_012179999.1	AASDH	mRNA	TCONS_00075051	lncRNA
XM_012179999.1	AASDH	mRNA	TCONS_00075051	lncRNA
XM_012179999.1	AASDH	mRNA	TCONS_00075051	lncRNA
XM_012185208.1	YPEL2	mRNA	TCONS_00022613	lncRNA
XM_012185208.1	YPEL2	mRNA	TCONS_00022613	lncRNA
XM_012185208.1	YPEL2	mRNA	TCONS_00022613	lncRNA
XM_012185208.1	YPEL2	mRNA	TCONS_00022613	lncRNA
XM_012184401.1	ZC3H13	mRNA	TCONS_00022613	lncRNA
XM_012184401.1	ZC3H13	mRNA	TCONS_00022613	lncRNA
XM_012184401.1	ZC3H13	mRNA	TCONS_00022613	lncRNA
XM_012184401.1	ZC3H13	mRNA	TCONS_00022613	lncRNA
XM_012107808.1	NFYC	mRNA	TCONS_00022613	lncRNA
XM_012107808.1	NFYC	mRNA	TCONS_00022613	lncRNA
XM_012107808.1	NFYC	mRNA	TCONS_00022613	lncRNA
XM_012107808.1	NFYC	mRNA	TCONS_00022613	lncRNA
XM_012104566.2	ZKSCAN1	mRNA	TCONS_00075453	lncRNA
XM_012104566.2	ZKSCAN1	mRNA	TCONS_00075453	lncRNA
XM_012104566.2	ZKSCAN1	mRNA	TCONS_00075453	lncRNA

XM_012104566.2	ZKSCAN1	mRNA	TCONS_00075453	lncRNA
XM_012101765.1	PRMT3	mRNA	TCONS_00075453	lncRNA
XM_012101765.1	PRMT3	mRNA	TCONS_00075453	lncRNA
XM_012101765.1	PRMT3	mRNA	TCONS_00075453	lncRNA
XM_012101765.1	PRMT3	mRNA	TCONS_00075453	lncRNA
XM_015103387.1	C22H10orf76	mRNA	XR_001034847.2	lncRNA
XM_015103387.1	C22H10orf76	mRNA	XR_001034847.2	lncRNA
XM_015103387.1	C22H10orf76	mRNA	XR_001034847.2	lncRNA
XM_015103387.1	C22H10orf76	mRNA	XR_001034847.2	lncRNA
XM_012096744.2	GFM2	mRNA	XR_001434202.1	lncRNA
XM_012096744.2	GFM2	mRNA	XR_001434202.1	lncRNA
XM_012096744.2	GFM2	mRNA	XR_001434202.1	lncRNA
XM_012096744.2	GFM2	mRNA	XR_001434202.1	lncRNA
XM_004002018.3	OMA1	mRNA	XR_001034847.2	lncRNA
XM_004002018.3	OMA1	mRNA	XR_001034847.2	lncRNA
XM_004002018.3	OMA1	mRNA	XR_001034847.2	lncRNA
XM_004002018.3	OMA1	mRNA	XR_001034847.2	lncRNA
XM_012103465.2	OSBPL1A	mRNA	XR_001434202.1	lncRNA
XM_012103465.2	OSBPL1A	mRNA	XR_001434202.1	lncRNA
XM_012103465.2	OSBPL1A	mRNA	XR_001434202.1	lncRNA
XM_012103465.2	OSBPL1A	mRNA	XR_001434202.1	lncRNA
XM_012096744.2	GFM2	mRNA	XR_001034847.2	lncRNA
XM_012096744.2	GFM2	mRNA	XR_001034847.2	lncRNA
XM_012096744.2	GFM2	mRNA	XR_001034847.2	lncRNA
XM_012096744.2	GFM2	mRNA	XR_001034847.2	lncRNA
XM_015103387.1	C22H10orf76	mRNA	XR_001434202.1	lncRNA
XM_015103387.1	C22H10orf76	mRNA	XR_001434202.1	lncRNA
XM_015103387.1	C22H10orf76	mRNA	XR_001434202.1	lncRNA
XM_015103387.1	C22H10orf76	mRNA	XR_001434202.1	lncRNA
XM_012103465.2	OSBPL1A	mRNA	XR_001034847.2	lncRNA
XM_012103465.2	OSBPL1A	mRNA	XR_001034847.2	lncRNA
XM_012103465.2	OSBPL1A	mRNA	XR_001034847.2	lncRNA
XM_012103465.2	OSBPL1A	mRNA	XR_001034847.2	lncRNA
XM_004021989.3	DMD	mRNA	XR_001034847.2	lncRNA
XM_004021989.3	DMD	mRNA	XR_001034847.2	lncRNA
XM_004021989.3	DMD	mRNA	XR_001034847.2	lncRNA
XM_004021989.3	DMD	mRNA	XR_001034847.2	lncRNA
XM_004021989.3	DMD	mRNA	XR_001434202.1	lncRNA
XM_004021989.3	DMD	mRNA	XR_001434202.1	lncRNA
XM_004021989.3	DMD	mRNA	XR_001434202.1	lncRNA
XM_004021989.3	DMD	mRNA	XR_001434202.1	lncRNA
XM_004002018.3	OMA1	mRNA	XR_001434202.1	lncRNA
XM_004002018.3	OMA1	mRNA	XR_001434202.1	lncRNA
XM_004002018.3	OMA1	mRNA	XR_001434202.1	lncRNA
XM_004002018.3	OMA1	mRNA	XR_001434202.1	lncRNA
XM_012177920.2	KANK2	mRNA	XR_001024743.2	lncRNA
XM_012177920.2	KANK2	mRNA	XR_001024743.2	lncRNA
XM_012177920.2	KANK2	mRNA	XR_001024743.2	lncRNA
XM_012177920.2	KANK2	mRNA	XR_001024743.2	lncRNA
XM_015096972.1	L2HGDH	mRNA	XR_001436127.1	lncRNA
XM_015096972.1	L2HGDH	mRNA	XR_001436127.1	lncRNA
XM_015096972.1	L2HGDH	mRNA	XR_001436127.1	lncRNA
XM_015096972.1	L2HGDH	mRNA	XR_001436127.1	lncRNA
XM_015104471.1	CCSER2	mRNA	XR_001436127.1	lncRNA

XM_015104471.1	CCSER2	mRNA	XR_001436127.1	lncRNA
XM_015104471.1	CCSER2	mRNA	XR_001436127.1	lncRNA
XM_015104471.1	CCSER2	mRNA	XR_001436127.1	lncRNA
XM_012095401.2	ATM	mRNA	XR_001025491.1	lncRNA
XM_012095401.2	ATM	mRNA	XR_001025491.1	lncRNA
XM_012095401.2	ATM	mRNA	XR_001025491.1	lncRNA
XM_012095401.2	ATM	mRNA	XR_001025491.1	lncRNA
XM_012146543.1	ATG9A	mRNA	XR_001433755.1	lncRNA
XM_012146543.1	ATG9A	mRNA	XR_001433755.1	lncRNA
XM_012146543.1	ATG9A	mRNA	XR_001433755.1	lncRNA
XM_012146543.1	ATG9A	mRNA	XR_001433755.1	lncRNA
XM_015099550.1	PARD3	mRNA	TCONS_00078440	lncRNA
XM_015099550.1	PARD3	mRNA	TCONS_00078440	lncRNA
XM_015099550.1	PARD3	mRNA	TCONS_00078440	lncRNA
XM_015099550.1	PARD3	mRNA	TCONS_00078440	lncRNA
XM_015099551.1	PARD3	mRNA	XR_001433755.1	lncRNA
XM_015099551.1	PARD3	mRNA	XR_001433755.1	lncRNA
XM_015099551.1	PARD3	mRNA	XR_001433755.1	lncRNA
XM_015099551.1	PARD3	mRNA	XR_001433755.1	lncRNA
XM_012190075.2	PHLPP2	mRNA	TCONS_00078440	lncRNA
XM_012190075.2	PHLPP2	mRNA	TCONS_00078440	lncRNA
XM_012190075.2	PHLPP2	mRNA	TCONS_00078440	lncRNA
XM_012190075.2	PHLPP2	mRNA	TCONS_00078440	lncRNA
XM_012190075.2	PHLPP2	mRNA	XR_001433755.1	lncRNA
XM_012190075.2	PHLPP2	mRNA	XR_001433755.1	lncRNA
XM_012190075.2	PHLPP2	mRNA	XR_001433755.1	lncRNA
XM_012190075.2	PHLPP2	mRNA	XR_001433755.1	lncRNA
XM_012184148.1	OTUL3B	mRNA	TCONS_00039304	lncRNA
XM_012184148.1	OTUL3B	mRNA	TCONS_00039304	lncRNA
XM_012184148.1	OTUL3B	mRNA	TCONS_00039304	lncRNA
XM_015099971.1	ZNF181	mRNA	TCONS_00039304	lncRNA
XM_015099971.1	ZNF181	mRNA	TCONS_00039304	lncRNA
XM_015099971.1	ZNF181	mRNA	TCONS_00039304	lncRNA
XM_012125926.2	ZDHHC21	mRNA	XR_001436817.1	lncRNA
XM_012125926.2	ZDHHC21	mRNA	XR_001436817.1	lncRNA
XM_012125926.2	ZDHHC21	mRNA	XR_001436817.1	lncRNA
XM_012125926.2	ZDHHC21	mRNA	XR_001436817.1	lncRNA
XM_012134593.2	FASTKD1	mRNA	XR_001436817.1	lncRNA
XM_012134593.2	FASTKD1	mRNA	XR_001436817.1	lncRNA
XM_012134593.2	FASTKD1	mRNA	XR_001436817.1	lncRNA
XM_012134593.2	FASTKD1	mRNA	XR_001436817.1	lncRNA
XM_012098903.1	PPIP5K1	mRNA	TCONS_00022613	lncRNA
XM_012098903.1	PPIP5K1	mRNA	TCONS_00022613	lncRNA
XM_012098903.1	PPIP5K1	mRNA	TCONS_00022613	lncRNA
XM_012098903.1	PPIP5K1	mRNA	TCONS_00022613	lncRNA
XM_012098306.1	UBE3A	mRNA	TCONS_00022613	lncRNA
XM_012098306.1	UBE3A	mRNA	TCONS_00022613	lncRNA
XM_012098306.1	UBE3A	mRNA	TCONS_00022613	lncRNA
XM_012098306.1	UBE3A	mRNA	TCONS_00022613	lncRNA
XM_012181190.2	EIF2AK4	mRNA	TCONS_00022613	lncRNA
XM_012181190.2	EIF2AK4	mRNA	TCONS_00022613	lncRNA
XM_012181190.2	EIF2AK4	mRNA	TCONS_00022613	lncRNA
XM_012181190.2	EIF2AK4	mRNA	TCONS_00022613	lncRNA
XM_012183566.1	FAM49B	mRNA	XR_001033284.2	lncRNA

XM_012183566.1	FAM49B	mRNA	XR_001033284.2	lncRNA
XM_012183566.1	FAM49B	mRNA	XR_001033284.2	lncRNA
XM_012183566.1	FAM49B	mRNA	XR_001033284.2	lncRNA
XM_012102216.2	TFDP2	mRNA	XR_001033284.2	lncRNA
XM_012102216.2	TFDP2	mRNA	XR_001033284.2	lncRNA
XM_012102216.2	TFDP2	mRNA	XR_001033284.2	lncRNA
XM_012102216.2	TFDP2	mRNA	XR_001033284.2	lncRNA
XM_012181326.2	MAP4K5	mRNA	TCONS_00075453	lncRNA
XM_012181326.2	MAP4K5	mRNA	TCONS_00075453	lncRNA
XM_012181326.2	MAP4K5	mRNA	TCONS_00075453	lncRNA
XM_012181326.2	MAP4K5	mRNA	TCONS_00075453	lncRNA
XM_012184823.2	RBM26	mRNA	TCONS_00075453	lncRNA
XM_012184823.2	RBM26	mRNA	TCONS_00075453	lncRNA
XM_012184823.2	RBM26	mRNA	TCONS_00075453	lncRNA
XM_012184823.2	RBM26	mRNA	TCONS_00075453	lncRNA
XM_012171189.2	MIER1	mRNA	XR_001434761.1	lncRNA
XM_012171189.2	MIER1	mRNA	XR_001434761.1	lncRNA
XM_012171189.2	MIER1	mRNA	XR_001434761.1	lncRNA
XM_012171189.2	MIER1	mRNA	XR_001434761.1	lncRNA
XM_012181247.2	MGA	mRNA	XR_001433755.1	lncRNA
XM_012181247.2	MGA	mRNA	XR_001433755.1	lncRNA
XM_012181247.2	MGA	mRNA	XR_001433755.1	lncRNA
XM_012181247.2	MGA	mRNA	XR_001433755.1	lncRNA
XM_015104948.1	RPS6KA6	mRNA	XR_001021689.2	lncRNA
XM_015104948.1	RPS6KA6	mRNA	XR_001021689.2	lncRNA
XM_015104948.1	RPS6KA6	mRNA	XR_001021689.2	lncRNA
XM_015104948.1	RPS6KA6	mRNA	XR_001021689.2	lncRNA
XM_012186312.2	DDX42	mRNA	XR_001021689.2	lncRNA
XM_012186312.2	DDX42	mRNA	XR_001021689.2	lncRNA
XM_012186312.2	DDX42	mRNA	XR_001021689.2	lncRNA
XM_012186312.2	DDX42	mRNA	XR_001021689.2	lncRNA
XM_015098144.1	HSPH1	mRNA	XR_001024743.2	lncRNA
XM_015098144.1	HSPH1	mRNA	XR_001024743.2	lncRNA
XM_015098144.1	HSPH1	mRNA	XR_001024743.2	lncRNA
XM_012104566.2	ZKSCAN1	mRNA	XR_001044126.1	lncRNA
XM_012104566.2	ZKSCAN1	mRNA	XR_001044126.1	lncRNA
XM_012104566.2	ZKSCAN1	mRNA	XR_001044126.1	lncRNA
XM_012104566.2	ZKSCAN1	mRNA	XR_001044126.1	lncRNA
XM_015103753.1	MBD1	mRNA	XR_001024290.1	lncRNA
XM_015103753.1	MBD1	mRNA	XR_001024290.1	lncRNA
XM_015103753.1	MBD1	mRNA	XR_001024290.1	lncRNA
XM_015103753.1	MBD1	mRNA	XR_001024290.1	lncRNA
XM_012143277.2	ABI2	mRNA	XR_001433755.1	lncRNA
XM_012143277.2	ABI2	mRNA	XR_001433755.1	lncRNA
XM_012143277.2	ABI2	mRNA	XR_001433755.1	lncRNA
XM_012143277.2	ABI2	mRNA	XR_001433755.1	lncRNA
XM_012102549.2	CEP70	mRNA	XR_001434202.1	lncRNA
XM_012102549.2	CEP70	mRNA	XR_001434202.1	lncRNA
XM_012102549.2	CEP70	mRNA	XR_001434202.1	lncRNA
XM_012102549.2	CEP70	mRNA	XR_001434202.1	lncRNA
XM_012095387.2	ELMOD1	mRNA	XR_001434202.1	lncRNA
XM_012095387.2	ELMOD1	mRNA	XR_001434202.1	lncRNA
XM_012095387.2	ELMOD1	mRNA	XR_001434202.1	lncRNA
XM_012095387.2	ELMOD1	mRNA	XR_001434202.1	lncRNA

XM_012186914.2	ZC3H11A	mRNA	XR_001434202.1	lncRNA
XM_012186914.2	ZC3H11A	mRNA	XR_001434202.1	lncRNA
XM_012186914.2	ZC3H11A	mRNA	XR_001434202.1	lncRNA
XM_012186914.2	ZC3H11A	mRNA	XR_001434202.1	lncRNA
XM_012188283.2	CCDC7	mRNA	XR_001034847.2	lncRNA
XM_012188283.2	CCDC7	mRNA	XR_001034847.2	lncRNA
XM_012188283.2	CCDC7	mRNA	XR_001034847.2	lncRNA
XM_012188283.2	CCDC7	mRNA	XR_001034847.2	lncRNA
XM_012102549.2	CEP70	mRNA	XR_001034847.2	lncRNA
XM_012102549.2	CEP70	mRNA	XR_001034847.2	lncRNA
XM_012102549.2	CEP70	mRNA	XR_001034847.2	lncRNA
XM_012102549.2	CEP70	mRNA	XR_001034847.2	lncRNA
XM_012095387.2	ELMOD1	mRNA	XR_001034847.2	lncRNA
XM_012095387.2	ELMOD1	mRNA	XR_001034847.2	lncRNA
XM_012095387.2	ELMOD1	mRNA	XR_001034847.2	lncRNA
XM_012095387.2	ELMOD1	mRNA	XR_001034847.2	lncRNA
XM_012186914.2	ZC3H11A	mRNA	XR_001034847.2	lncRNA
XM_012186914.2	ZC3H11A	mRNA	XR_001034847.2	lncRNA
XM_012186914.2	ZC3H11A	mRNA	XR_001034847.2	lncRNA
XM_012186914.2	ZC3H11A	mRNA	XR_001034847.2	lncRNA
XM_015102007.1	PPP2R3C	mRNA	XR_001034847.2	lncRNA
XM_015102007.1	PPP2R3C	mRNA	XR_001034847.2	lncRNA
XM_015102007.1	PPP2R3C	mRNA	XR_001034847.2	lncRNA
XM_015102007.1	PPP2R3C	mRNA	XR_001034847.2	lncRNA
XM_012188283.2	CCDC7	mRNA	XR_001434202.1	lncRNA
XM_012188283.2	CCDC7	mRNA	XR_001434202.1	lncRNA
XM_012188283.2	CCDC7	mRNA	XR_001434202.1	lncRNA
XM_012188283.2	CCDC7	mRNA	XR_001434202.1	lncRNA
XM_015102007.1	PPP2R3C	mRNA	XR_001434202.1	lncRNA
XM_015102007.1	PPP2R3C	mRNA	XR_001434202.1	lncRNA
XM_015102007.1	PPP2R3C	mRNA	XR_001434202.1	lncRNA
XM_015102007.1	PPP2R3C	mRNA	XR_001434202.1	lncRNA
XM_015098003.1	LOC10110962(	mRNA	TCONS_00084305	lncRNA
XM_015098003.1	LOC10110962(	mRNA	TCONS_00084305	lncRNA
XM_015098003.1	LOC10110962(	mRNA	TCONS_00084305	lncRNA
XM_012105643.1	ZFP69	mRNA	TCONS_00054422	lncRNA
XM_012105643.1	ZFP69	mRNA	TCONS_00054422	lncRNA
XM_012105643.1	ZFP69	mRNA	TCONS_00054422	lncRNA
XM_004002178.3	LRRC8B	mRNA	XR_001020707.2	lncRNA
XM_004002178.3	LRRC8B	mRNA	XR_001020707.2	lncRNA
XM_004002178.3	LRRC8B	mRNA	XR_001020707.2	lncRNA
XM_004002178.3	LRRC8B	mRNA	XR_001020707.2	lncRNA
NM_001308600.1	KRT17	mRNA	XR_001020707.2	lncRNA
NM_001308600.1	KRT17	mRNA	XR_001020707.2	lncRNA
NM_001308600.1	KRT17	mRNA	XR_001020707.2	lncRNA
NM_001308600.1	KRT17	mRNA	XR_001020707.2	lncRNA
XM_012095439.2	SIK2	mRNA	XR_001033284.2	lncRNA
XM_012095439.2	SIK2	mRNA	XR_001033284.2	lncRNA
XM_012095439.2	SIK2	mRNA	XR_001033284.2	lncRNA
XM_012095439.2	SIK2	mRNA	XR_001033284.2	lncRNA
XM_004008647.3	RAPGEF6	mRNA	TCONS_00075453	lncRNA
XM_004008647.3	RAPGEF6	mRNA	TCONS_00075453	lncRNA
XM_004008647.3	RAPGEF6	mRNA	TCONS_00075453	lncRNA
XM_004008647.3	RAPGEF6	mRNA	TCONS_00075453	lncRNA

XM_012102658.2	ZNF518A	mRNA	TCONS_00075453	lncRNA
XM_012102658.2	ZNF518A	mRNA	TCONS_00075453	lncRNA
XM_012102658.2	ZNF518A	mRNA	TCONS_00075453	lncRNA
XM_012102658.2	ZNF518A	mRNA	TCONS_00075453	lncRNA
XM_015092883.1	PADI1	mRNA	TCONS_00075453	lncRNA
XM_015092883.1	PADI1	mRNA	TCONS_00075453	lncRNA
XM_015092883.1	PADI1	mRNA	TCONS_00075453	lncRNA
XM_015092883.1	PADI1	mRNA	TCONS_00075453	lncRNA
XM_015098766.1	WIPF2	mRNA	XR_001436102.1	lncRNA
XM_015098766.1	WIPF2	mRNA	XR_001436102.1	lncRNA
XM_015098766.1	WIPF2	mRNA	XR_001436102.1	lncRNA
XM_015098766.1	WIPF2	mRNA	XR_001436102.1	lncRNA
XM_012131583.2	FAM171B	mRNA	XR_001025491.1	lncRNA
XM_012131583.2	FAM171B	mRNA	XR_001025491.1	lncRNA
XM_012131583.2	FAM171B	mRNA	XR_001025491.1	lncRNA
XM_012131583.2	FAM171B	mRNA	XR_001025491.1	lncRNA
XM_012185208.1	YPEL2	mRNA	XR_001025491.1	lncRNA
XM_012185208.1	YPEL2	mRNA	XR_001025491.1	lncRNA
XM_012185208.1	YPEL2	mRNA	XR_001025491.1	lncRNA
XM_012185208.1	YPEL2	mRNA	XR_001025491.1	lncRNA
XM_004018249.3	TRAK1	mRNA	XR_001025491.1	lncRNA
XM_004018249.3	TRAK1	mRNA	XR_001025491.1	lncRNA
XM_004018249.3	TRAK1	mRNA	XR_001025491.1	lncRNA
XM_004018249.3	TRAK1	mRNA	XR_001025491.1	lncRNA
XM_012098709.2	PML	mRNA	TCONS_00075453	lncRNA
XM_012098709.2	PML	mRNA	TCONS_00075453	lncRNA
XM_012098709.2	PML	mRNA	TCONS_00075453	lncRNA
XM_012098709.2	PML	mRNA	TCONS_00075453	lncRNA
XM_012105331.2	USP54	mRNA	TCONS_00075453	lncRNA
XM_012105331.2	USP54	mRNA	TCONS_00075453	lncRNA
XM_012105331.2	USP54	mRNA	TCONS_00075453	lncRNA
XM_012105331.2	USP54	mRNA	TCONS_00075453	lncRNA
XM_012188768.2	UBOX5	mRNA	TCONS_00075453	lncRNA
XM_012188768.2	UBOX5	mRNA	TCONS_00075453	lncRNA
XM_012188768.2	UBOX5	mRNA	TCONS_00075453	lncRNA
XM_012188768.2	UBOX5	mRNA	TCONS_00075453	lncRNA
XM_012143285.2	ABI2	mRNA	XR_001434761.1	lncRNA
XM_012143285.2	ABI2	mRNA	XR_001434761.1	lncRNA
XM_012143285.2	ABI2	mRNA	XR_001434761.1	lncRNA
XM_012143285.2	ABI2	mRNA	XR_001434761.1	lncRNA
XM_004002227.3	SLC35A3	mRNA	XR_001033284.2	lncRNA
XM_004002227.3	SLC35A3	mRNA	XR_001033284.2	lncRNA
XM_004002227.3	SLC35A3	mRNA	XR_001033284.2	lncRNA
XM_012100104.2	NME6	mRNA	XR_001033284.2	lncRNA
XM_012100104.2	NME6	mRNA	XR_001033284.2	lncRNA
XM_012100104.2	NME6	mRNA	XR_001033284.2	lncRNA
XM_012171234.2	CFAP36	mRNA	XR_001020707.2	lncRNA
XM_012171234.2	CFAP36	mRNA	XR_001020707.2	lncRNA
XM_012171234.2	CFAP36	mRNA	XR_001020707.2	lncRNA
XM_012098824.2	BRMS1L	mRNA	XR_001033284.2	lncRNA
XM_012098824.2	BRMS1L	mRNA	XR_001033284.2	lncRNA
XM_012098824.2	BRMS1L	mRNA	XR_001033284.2	lncRNA
XM_012174768.2	RAB5B	mRNA	XR_001020707.2	lncRNA
XM_012174768.2	RAB5B	mRNA	XR_001020707.2	lncRNA

XM_012174768.2	RAB5B	mRNA	XR_001020707.2	lncRNA
XM_012174768.2	RAB5B	mRNA	XR_001020707.2	lncRNA
XM_012102835.2	PPRC1	mRNA	XR_001033284.2	lncRNA
XM_012102835.2	PPRC1	mRNA	XR_001033284.2	lncRNA
XM_012102835.2	PPRC1	mRNA	XR_001033284.2	lncRNA
XM_012102835.2	PPRC1	mRNA	XR_001033284.2	lncRNA
XM_012190630.2	PLEKHA4	mRNA	XR_001033284.2	lncRNA
XM_012190630.2	PLEKHA4	mRNA	XR_001033284.2	lncRNA
XM_012190630.2	PLEKHA4	mRNA	XR_001033284.2	lncRNA
XM_012190630.2	PLEKHA4	mRNA	XR_001033284.2	lncRNA
XM_004016103.2	USP2	mRNA	XR_001033284.2	lncRNA
XM_004016103.2	USP2	mRNA	XR_001033284.2	lncRNA
XM_004016103.2	USP2	mRNA	XR_001033284.2	lncRNA
XM_004016103.2	USP2	mRNA	XR_001033284.2	lncRNA
XM_012101635.2	SYTL2	mRNA	TCONS_00022613	lncRNA
XM_012101635.2	SYTL2	mRNA	TCONS_00022613	lncRNA
XM_012101635.2	SYTL2	mRNA	TCONS_00022613	lncRNA
XM_012101635.2	SYTL2	mRNA	TCONS_00022613	lncRNA
XM_012098820.2	RALGAPA1	mRNA	TCONS_00022613	lncRNA
XM_012098820.2	RALGAPA1	mRNA	TCONS_00022613	lncRNA
XM_012098820.2	RALGAPA1	mRNA	TCONS_00022613	lncRNA
XM_012098820.2	RALGAPA1	mRNA	TCONS_00022613	lncRNA
XM_015097132.1	TMEM229B	mRNA	TCONS_00022613	lncRNA
XM_015097132.1	TMEM229B	mRNA	TCONS_00022613	lncRNA
XM_015097132.1	TMEM229B	mRNA	TCONS_00022613	lncRNA
XM_015097132.1	TMEM229B	mRNA	TCONS_00022613	lncRNA
XM_012105656.2	ZNF596	mRNA	TCONS_00022613	lncRNA
XM_012105656.2	ZNF596	mRNA	TCONS_00022613	lncRNA
XM_012105656.2	ZNF596	mRNA	TCONS_00022613	lncRNA
XM_012105656.2	ZNF596	mRNA	TCONS_00022613	lncRNA
XM_012176680.2	ARHGAP29	mRNA	XR_001434202.1	lncRNA
XM_012176680.2	ARHGAP29	mRNA	XR_001434202.1	lncRNA
XM_012176680.2	ARHGAP29	mRNA	XR_001434202.1	lncRNA
XM_012176680.2	ARHGAP29	mRNA	XR_001434202.1	lncRNA
XM_012176680.2	ARHGAP29	mRNA	XR_001034847.2	lncRNA
XM_012176680.2	ARHGAP29	mRNA	XR_001034847.2	lncRNA
XM_012176680.2	ARHGAP29	mRNA	XR_001034847.2	lncRNA
XM_012176680.2	ARHGAP29	mRNA	XR_001034847.2	lncRNA
XM_015103591.1	KIAA1328	mRNA	TCONS_00084305	lncRNA
XM_015103591.1	KIAA1328	mRNA	TCONS_00084305	lncRNA
XM_015103591.1	KIAA1328	mRNA	TCONS_00084305	lncRNA
XM_004002018.3	OMA1	mRNA	TCONS_00054422	lncRNA
XM_004002018.3	OMA1	mRNA	TCONS_00054422	lncRNA
XM_004002018.3	OMA1	mRNA	TCONS_00054422	lncRNA
XM_012185094.2	STRADA	mRNA	TCONS_00075051	lncRNA
XM_012185094.2	STRADA	mRNA	TCONS_00075051	lncRNA
XM_012185094.2	STRADA	mRNA	TCONS_00075051	lncRNA
XM_012105643.1	ZFP69	mRNA	XR_001024743.2	lncRNA
XM_012105643.1	ZFP69	mRNA	XR_001024743.2	lncRNA
XM_012105643.1	ZFP69	mRNA	XR_001024743.2	lncRNA
XM_012175208.1	ODF2L	mRNA	XR_001025491.1	lncRNA
XM_012175208.1	ODF2L	mRNA	XR_001025491.1	lncRNA
XM_012175208.1	ODF2L	mRNA	XR_001025491.1	lncRNA
XM_012144092.2	METTL21A	mRNA	XR_001436127.1	lncRNA

XM_012144092.2	METTL21A	mRNA	XR_001436127.1	lncRNA
XM_012144092.2	METTL21A	mRNA	XR_001436127.1	lncRNA
XM_012176307.2	KRIT1	mRNA	XR_001025491.1	lncRNA
XM_012176307.2	KRIT1	mRNA	XR_001025491.1	lncRNA
XM_012176307.2	KRIT1	mRNA	XR_001025491.1	lncRNA
XM_012103110.1	METTL10	mRNA	XR_001044126.1	lncRNA
XM_012103110.1	METTL10	mRNA	XR_001044126.1	lncRNA
XM_012103110.1	METTL10	mRNA	XR_001044126.1	lncRNA
XM_012176307.2	KRIT1	mRNA	XR_001044126.1	lncRNA
XM_012176307.2	KRIT1	mRNA	XR_001044126.1	lncRNA
XM_012176307.2	KRIT1	mRNA	XR_001044126.1	lncRNA
XM_012102580.2	FRA10AC1	mRNA	XR_001044126.1	lncRNA
XM_012102580.2	FRA10AC1	mRNA	XR_001044126.1	lncRNA
XM_012102580.2	FRA10AC1	mRNA	XR_001044126.1	lncRNA
XM_012101919.2	ZBTB44	mRNA	TCONS_00039304	lncRNA
XM_012101919.2	ZBTB44	mRNA	TCONS_00039304	lncRNA
XM_012101919.2	ZBTB44	mRNA	TCONS_00039304	lncRNA
XM_015103117.1	ZBTB44	mRNA	TCONS_00039304	lncRNA
XM_015103117.1	ZBTB44	mRNA	TCONS_00039304	lncRNA
XM_015103117.1	ZBTB44	mRNA	TCONS_00039304	lncRNA
XM_015092486.1	PIGZ	mRNA	TCONS_00075453	lncRNA
XM_015092486.1	PIGZ	mRNA	TCONS_00075453	lncRNA
XM_015092486.1	PIGZ	mRNA	TCONS_00075453	lncRNA
XM_015092486.1	PIGZ	mRNA	TCONS_00075453	lncRNA
XM_015104788.1	TBL1X	mRNA	XR_001025491.1	lncRNA
XM_015104788.1	TBL1X	mRNA	XR_001025491.1	lncRNA
XM_015104788.1	TBL1X	mRNA	XR_001025491.1	lncRNA
XM_015104788.1	TBL1X	mRNA	XR_001025491.1	lncRNA
XM_012181190.2	EIF2AK4	mRNA	XR_001044126.1	lncRNA
XM_012181190.2	EIF2AK4	mRNA	XR_001044126.1	lncRNA
XM_012181190.2	EIF2AK4	mRNA	XR_001044126.1	lncRNA
XM_012181190.2	EIF2AK4	mRNA	XR_001044126.1	lncRNA
XM_012140422.2	CLASP1	mRNA	XR_001044126.1	lncRNA
XM_012140422.2	CLASP1	mRNA	XR_001044126.1	lncRNA
XM_012140422.2	CLASP1	mRNA	XR_001044126.1	lncRNA
XM_012140422.2	CLASP1	mRNA	XR_001044126.1	lncRNA
XM_012178138.2	P4HA2	mRNA	XR_001436127.1	lncRNA
XM_012178138.2	P4HA2	mRNA	XR_001436127.1	lncRNA
XM_012178138.2	P4HA2	mRNA	XR_001436127.1	lncRNA
XM_012178138.2	P4HA2	mRNA	XR_001436127.1	lncRNA
XM_015098990.1	GNA13	mRNA	XR_001044126.1	lncRNA
XM_015098990.1	GNA13	mRNA	XR_001044126.1	lncRNA
XM_015098990.1	GNA13	mRNA	XR_001044126.1	lncRNA
XM_015098990.1	GNA13	mRNA	XR_001044126.1	lncRNA
XM_012182238.1	SPG11	mRNA	XR_001033284.2	lncRNA
XM_012182238.1	SPG11	mRNA	XR_001033284.2	lncRNA
XM_012182238.1	SPG11	mRNA	XR_001033284.2	lncRNA
XM_012182238.1	SPG11	mRNA	XR_001033284.2	lncRNA
XM_012101919.2	ZBTB44	mRNA	XR_001033284.2	lncRNA
XM_012101919.2	ZBTB44	mRNA	XR_001033284.2	lncRNA
XM_012101919.2	ZBTB44	mRNA	XR_001033284.2	lncRNA
XM_012101919.2	ZBTB44	mRNA	XR_001033284.2	lncRNA
XM_012182353.2	LRRFIP1	mRNA	XR_001021689.2	lncRNA
XM_012182353.2	LRRFIP1	mRNA	XR_001021689.2	lncRNA

XM_012182353.2	LRRFIP1	mRNA	XR_001021689.2	lncRNA
XM_012182353.2	LRRFIP1	mRNA	XR_001021689.2	lncRNA
XM_004010552.3	VPS13C	mRNA	TCONS_00039304	lncRNA
XM_004010552.3	VPS13C	mRNA	TCONS_00039304	lncRNA
XM_004010552.3	VPS13C	mRNA	TCONS_00039304	lncRNA
XM_015097502.1	SNX9	mRNA	TCONS_00039304	lncRNA
XM_015097502.1	SNX9	mRNA	TCONS_00039304	lncRNA
XM_015097502.1	SNX9	mRNA	TCONS_00039304	lncRNA
XM_012100690.1	KLC4	mRNA	TCONS_00075453	lncRNA
XM_012100690.1	KLC4	mRNA	TCONS_00075453	lncRNA
XM_012100690.1	KLC4	mRNA	TCONS_00075453	lncRNA
XM_012100690.1	KLC4	mRNA	TCONS_00075453	lncRNA
XM_012146543.1	ATG9A	mRNA	TCONS_00075453	lncRNA
XM_012146543.1	ATG9A	mRNA	TCONS_00075453	lncRNA
XM_012146543.1	ATG9A	mRNA	TCONS_00075453	lncRNA
XM_012146543.1	ATG9A	mRNA	TCONS_00075453	lncRNA
XM_012182469.2	ECHDC1	mRNA	TCONS_00022613	lncRNA
XM_012182469.2	ECHDC1	mRNA	TCONS_00022613	lncRNA
XM_012182469.2	ECHDC1	mRNA	TCONS_00022613	lncRNA
XM_012119403.2	BIN3	mRNA	TCONS_00078440	lncRNA
XM_012119403.2	BIN3	mRNA	TCONS_00078440	lncRNA
XM_012119403.2	BIN3	mRNA	TCONS_00078440	lncRNA
XM_004014443.3	RAE1	mRNA	TCONS_00078440	lncRNA
XM_004014443.3	RAE1	mRNA	TCONS_00078440	lncRNA
XM_004014443.3	RAE1	mRNA	TCONS_00078440	lncRNA
XM_015102007.1	PPP2R3C	mRNA	TCONS_00054422	lncRNA
XM_015102007.1	PPP2R3C	mRNA	TCONS_00054422	lncRNA
XM_015102007.1	PPP2R3C	mRNA	TCONS_00054422	lncRNA
XM_015104177.1	PRSS8	mRNA	XR_001436931.1	lncRNA
XM_015104177.1	PRSS8	mRNA	XR_001436931.1	lncRNA
XM_015104177.1	PRSS8	mRNA	XR_001436931.1	lncRNA
XM_012095345.1	CCDC82	mRNA	TCONS_00054422	lncRNA
XM_012095345.1	CCDC82	mRNA	TCONS_00054422	lncRNA
XM_012095345.1	CCDC82	mRNA	TCONS_00054422	lncRNA
XM_015104177.1	PRSS8	mRNA	TCONS_00082638	lncRNA
XM_015104177.1	PRSS8	mRNA	TCONS_00082638	lncRNA
XM_015104177.1	PRSS8	mRNA	TCONS_00082638	lncRNA
XM_012101211.2	MBNL1	mRNA	XR_001020707.2	lncRNA
XM_012101211.2	MBNL1	mRNA	XR_001020707.2	lncRNA
XM_012101211.2	MBNL1	mRNA	XR_001020707.2	lncRNA
XM_012101211.2	MBNL1	mRNA	XR_001020707.2	lncRNA
XM_015102318.1	SETD5	mRNA	TCONS_00039304	lncRNA
XM_015102318.1	SETD5	mRNA	TCONS_00039304	lncRNA
XM_015102318.1	SETD5	mRNA	TCONS_00039304	lncRNA
XM_012098820.2	RALGAPA1	mRNA	TCONS_00039304	lncRNA
XM_012098820.2	RALGAPA1	mRNA	TCONS_00039304	lncRNA
XM_012098820.2	RALGAPA1	mRNA	TCONS_00039304	lncRNA
XM_004010813.3	GSTM1	mRNA	XR_001025491.1	lncRNA
XM_004010813.3	GSTM1	mRNA	XR_001025491.1	lncRNA
XM_004010813.3	GSTM1	mRNA	XR_001025491.1	lncRNA
XM_004010813.3	GSTM1	mRNA	XR_001025491.1	lncRNA
XM_012104950.1	TTC13	mRNA	XR_001044126.1	lncRNA
XM_012104950.1	TTC13	mRNA	XR_001044126.1	lncRNA
XM_012104950.1	TTC13	mRNA	XR_001044126.1	lncRNA

XM_012104950.1	TTC13	mRNA	XR_001044126.1	lncRNA
XM_012103110.1	METTL10	mRNA	XR_001021689.2	lncRNA
XM_012103110.1	METTL10	mRNA	XR_001021689.2	lncRNA
XM_012103110.1	METTL10	mRNA	XR_001021689.2	lncRNA
XM_012171234.2	CFAP36	mRNA	XR_001436102.1	lncRNA
XM_012171234.2	CFAP36	mRNA	XR_001436102.1	lncRNA
XM_012171234.2	CFAP36	mRNA	XR_001436102.1	lncRNA
XM_015104655.1	NKIRAS1	mRNA	XR_001021689.2	lncRNA
XM_015104655.1	NKIRAS1	mRNA	XR_001021689.2	lncRNA
XM_015104655.1	NKIRAS1	mRNA	XR_001021689.2	lncRNA
XM_012175096.2	ERGIC2	mRNA	XR_001021689.2	lncRNA
XM_012175096.2	ERGIC2	mRNA	XR_001021689.2	lncRNA
XM_012175096.2	ERGIC2	mRNA	XR_001021689.2	lncRNA
XM_012098824.2	BRMS1L	mRNA	XR_001436102.1	lncRNA
XM_012098824.2	BRMS1L	mRNA	XR_001436102.1	lncRNA
XM_012098824.2	BRMS1L	mRNA	XR_001436102.1	lncRNA
XM_015104655.1	NKIRAS1	mRNA	XR_001436102.1	lncRNA
XM_015104655.1	NKIRAS1	mRNA	XR_001436102.1	lncRNA
XM_015104655.1	NKIRAS1	mRNA	XR_001436102.1	lncRNA
XM_012103110.1	METTL10	mRNA	XR_001436102.1	lncRNA
XM_012103110.1	METTL10	mRNA	XR_001436102.1	lncRNA
XM_012103110.1	METTL10	mRNA	XR_001436102.1	lncRNA
XM_012135865.2	TANK	mRNA	TCONS_00075453	lncRNA
XM_012135865.2	TANK	mRNA	TCONS_00075453	lncRNA
XM_012135865.2	TANK	mRNA	TCONS_00075453	lncRNA
XM_004021416.3	PBLD	mRNA	TCONS_00075453	lncRNA
XM_004021416.3	PBLD	mRNA	TCONS_00075453	lncRNA
XM_004021416.3	PBLD	mRNA	TCONS_00075453	lncRNA
XM_015102318.1	SETD5	mRNA	XR_001033284.2	lncRNA
XM_015102318.1	SETD5	mRNA	XR_001033284.2	lncRNA
XM_015102318.1	SETD5	mRNA	XR_001033284.2	lncRNA
XM_015102318.1	SETD5	mRNA	XR_001033284.2	lncRNA
XM_015095790.1	CC2D1A	mRNA	XR_001436127.1	lncRNA
XM_015095790.1	CC2D1A	mRNA	XR_001436127.1	lncRNA
XM_015095790.1	CC2D1A	mRNA	XR_001436127.1	lncRNA
XM_015095790.1	CC2D1A	mRNA	XR_001436127.1	lncRNA
XM_015092486.1	PIGZ	mRNA	XR_001044126.1	lncRNA
XM_015092486.1	PIGZ	mRNA	XR_001044126.1	lncRNA
XM_015092486.1	PIGZ	mRNA	XR_001044126.1	lncRNA
XM_015092486.1	PIGZ	mRNA	XR_001044126.1	lncRNA
XM_015104471.1	CCSER2	mRNA	TCONS_00054422	lncRNA
XM_015104471.1	CCSER2	mRNA	TCONS_00054422	lncRNA
XM_015104471.1	CCSER2	mRNA	TCONS_00054422	lncRNA
XM_012188945.2	CSTF1	mRNA	XR_001436931.1	lncRNA
XM_012188945.2	CSTF1	mRNA	XR_001436931.1	lncRNA
XM_012188945.2	CSTF1	mRNA	XR_001436931.1	lncRNA
XM_015095875.1	SAFB	mRNA	TCONS_00054422	lncRNA
XM_015095875.1	SAFB	mRNA	TCONS_00054422	lncRNA
XM_015095875.1	SAFB	mRNA	TCONS_00054422	lncRNA
XM_012188276.2	CCDC7	mRNA	XR_001436931.1	lncRNA
XM_012188276.2	CCDC7	mRNA	XR_001436931.1	lncRNA
XM_012188276.2	CCDC7	mRNA	XR_001436931.1	lncRNA
XM_012186029.1	FBXL20	mRNA	TCONS_00084305	lncRNA
XM_012186029.1	FBXL20	mRNA	TCONS_00084305	lncRNA

XM_012186029.1	FBXL20	mRNA	TCONS_00084305	lncRNA
XM_012170864.2	PAPOLG	mRNA	TCONS_00084305	lncRNA
XM_012170864.2	PAPOLG	mRNA	TCONS_00084305	lncRNA
XM_012170864.2	PAPOLG	mRNA	TCONS_00084305	lncRNA
XM_015103641.1	YES1	mRNA	TCONS_00054422	lncRNA
XM_015103641.1	YES1	mRNA	TCONS_00054422	lncRNA
XM_015103641.1	YES1	mRNA	TCONS_00054422	lncRNA
XM_012152455.2	LUZP1	mRNA	TCONS_00039304	lncRNA
XM_012152455.2	LUZP1	mRNA	TCONS_00039304	lncRNA
XM_012152455.2	LUZP1	mRNA	TCONS_00039304	lncRNA
XM_015091997.1	LOC10112163¢	mRNA	TCONS_00039304	lncRNA
XM_015091997.1	LOC10112163¢	mRNA	TCONS_00039304	lncRNA
XM_015091997.1	LOC10112163¢	mRNA	TCONS_00039304	lncRNA
XM_012187914.1	GIGYF2	mRNA	TCONS_00039304	lncRNA
XM_012187914.1	GIGYF2	mRNA	TCONS_00039304	lncRNA
XM_012187914.1	GIGYF2	mRNA	TCONS_00039304	lncRNA
XM_015102923.1	KCTD14	mRNA	XR_001034847.2	lncRNA
XM_015102923.1	KCTD14	mRNA	XR_001034847.2	lncRNA
XM_015102923.1	KCTD14	mRNA	XR_001034847.2	lncRNA
XM_012095456.1	SDHD	mRNA	XR_001434202.1	lncRNA
XM_012095456.1	SDHD	mRNA	XR_001434202.1	lncRNA
XM_012095456.1	SDHD	mRNA	XR_001434202.1	lncRNA
XM_015102923.1	KCTD14	mRNA	XR_001434202.1	lncRNA
XM_015102923.1	KCTD14	mRNA	XR_001434202.1	lncRNA
XM_015102923.1	KCTD14	mRNA	XR_001434202.1	lncRNA
XM_012175096.2	ERGIC2	mRNA	XR_001434202.1	lncRNA
XM_012175096.2	ERGIC2	mRNA	XR_001434202.1	lncRNA
XM_012175096.2	ERGIC2	mRNA	XR_001434202.1	lncRNA
XM_012175096.2	ERGIC2	mRNA	XR_001034847.2	lncRNA
XM_012175096.2	ERGIC2	mRNA	XR_001034847.2	lncRNA
XM_012175096.2	ERGIC2	mRNA	XR_001034847.2	lncRNA
XM_012095456.1	SDHD	mRNA	XR_001034847.2	lncRNA
XM_012095456.1	SDHD	mRNA	XR_001034847.2	lncRNA
XM_012095456.1	SDHD	mRNA	XR_001034847.2	lncRNA
XM_015101730.1	ZNF280B	mRNA	XR_001033284.2	lncRNA
XM_015101730.1	ZNF280B	mRNA	XR_001033284.2	lncRNA
XM_015101730.1	ZNF280B	mRNA	XR_001033284.2	lncRNA
XM_012189207.2	SENP7	mRNA	XR_001033284.2	lncRNA
XM_012189207.2	SENP7	mRNA	XR_001033284.2	lncRNA
XM_012189207.2	SENP7	mRNA	XR_001033284.2	lncRNA
XM_004021416.3	PBLD	mRNA	XR_001033284.2	lncRNA
XM_004021416.3	PBLD	mRNA	XR_001033284.2	lncRNA
XM_004021416.3	PBLD	mRNA	XR_001033284.2	lncRNA
XM_012180879.2	MYO9A	mRNA	TCONS_00039304	lncRNA
XM_012180879.2	MYO9A	mRNA	TCONS_00039304	lncRNA
XM_012180879.2	MYO9A	mRNA	TCONS_00039304	lncRNA
XM_015091836.1	USP40	mRNA	TCONS_00039304	lncRNA
XM_015091836.1	USP40	mRNA	TCONS_00039304	lncRNA
XM_015091836.1	USP40	mRNA	TCONS_00039304	lncRNA
XM_015092323.1	KIRREL	mRNA	TCONS_00039304	lncRNA
XM_015092323.1	KIRREL	mRNA	TCONS_00039304	lncRNA
XM_015092323.1	KIRREL	mRNA	TCONS_00039304	lncRNA
XM_012178848.2	CNOT8	mRNA	XR_001433755.1	lncRNA
XM_012178848.2	CNOT8	mRNA	XR_001433755.1	lncRNA

XM_012178848.2	CNOT8	mRNA	XR_001433755.1	lncRNA
XM_012178788.2	HMGXB3	mRNA	XR_001025491.1	lncRNA
XM_012178788.2	HMGXB3	mRNA	XR_001025491.1	lncRNA
XM_012178788.2	HMGXB3	mRNA	XR_001025491.1	lncRNA
XM_012178788.2	HMGXB3	mRNA	XR_001025491.1	lncRNA
XM_004015976.3	LOC10112043:	mRNA	XR_001434761.1	lncRNA
XM_004015976.3	LOC10112043:	mRNA	XR_001434761.1	lncRNA
XM_004015976.3	LOC10112043:	mRNA	XR_001434761.1	lncRNA
XM_012174558.1	CNOT2	mRNA	XR_001434892.1	lncRNA
XM_012174558.1	CNOT2	mRNA	XR_001434892.1	lncRNA
XM_012174558.1	CNOT2	mRNA	XR_001434892.1	lncRNA
XM_015102111.1	EIF4E3	mRNA	TCONS_00082638	lncRNA
XM_015102111.1	EIF4E3	mRNA	TCONS_00082638	lncRNA
XM_015102111.1	EIF4E3	mRNA	TCONS_00082638	lncRNA
XM_012118246.2	DCAF12	mRNA	TCONS_00084305	lncRNA
XM_012118246.2	DCAF12	mRNA	TCONS_00084305	lncRNA
XM_012118246.2	DCAF12	mRNA	TCONS_00084305	lncRNA
XM_012183965.2	OXR1	mRNA	XR_001025491.1	lncRNA
XM_012183965.2	OXR1	mRNA	XR_001025491.1	lncRNA
XM_012183965.2	OXR1	mRNA	XR_001025491.1	lncRNA
XM_012177629.2	CLCC1	mRNA	XR_001044126.1	lncRNA
XM_012177629.2	CLCC1	mRNA	XR_001044126.1	lncRNA
XM_012177629.2	CLCC1	mRNA	XR_001044126.1	lncRNA
XM_012174040.2	SLC6A15	mRNA	XR_001044126.1	lncRNA
XM_012174040.2	SLC6A15	mRNA	XR_001044126.1	lncRNA
XM_012174040.2	SLC6A15	mRNA	XR_001044126.1	lncRNA
XM_015098144.1	HSPH1	mRNA	XR_001436127.1	lncRNA
XM_015098144.1	HSPH1	mRNA	XR_001436127.1	lncRNA
XM_015098144.1	HSPH1	mRNA	XR_001436127.1	lncRNA
XM_004022454.2	TCEAL8	mRNA	XR_001436127.1	lncRNA
XM_004022454.2	TCEAL8	mRNA	XR_001436127.1	lncRNA
XM_004022454.2	TCEAL8	mRNA	XR_001436127.1	lncRNA
XM_004002454.2	ECM1	mRNA	XR_001436127.1	lncRNA
XM_004002454.2	ECM1	mRNA	XR_001436127.1	lncRNA
XM_004002454.2	ECM1	mRNA	XR_001436127.1	lncRNA
XM_012183965.2	OXR1	mRNA	XR_001044126.1	lncRNA
XM_012183965.2	OXR1	mRNA	XR_001044126.1	lncRNA
XM_012183965.2	OXR1	mRNA	XR_001044126.1	lncRNA
XM_012173546.1	NDUFAF7	mRNA	XR_001025491.1	lncRNA
XM_012173546.1	NDUFAF7	mRNA	XR_001025491.1	lncRNA
XM_012173546.1	NDUFAF7	mRNA	XR_001025491.1	lncRNA
XM_015103461.1	TACC2	mRNA	TCONS_00039304	lncRNA
XM_015103461.1	TACC2	mRNA	TCONS_00039304	lncRNA
XM_015103461.1	TACC2	mRNA	TCONS_00039304	lncRNA
XM_004004866.2	METTL21A	mRNA	XR_001024743.2	lncRNA
XM_004004866.2	METTL21A	mRNA	XR_001024743.2	lncRNA
XM_004004866.2	METTL21A	mRNA	XR_001024743.2	lncRNA
XM_015104655.1	NKIRAS1	mRNA	XR_001042381.2	lncRNA
XM_015104655.1	NKIRAS1	mRNA	XR_001042381.2	lncRNA
XM_015104655.1	NKIRAS1	mRNA	XR_001042381.2	lncRNA
XM_012144092.2	METTL21A	mRNA	XR_001436532.1	lncRNA
XM_012144092.2	METTL21A	mRNA	XR_001436532.1	lncRNA
XM_012144092.2	METTL21A	mRNA	XR_001436532.1	lncRNA
XM_015102923.1	KCTD14	mRNA	XR_001436532.1	lncRNA

XM_015102923.1	KCTD14	mRNA	XR_001436532.1	lncRNA
XM_015102923.1	KCTD14	mRNA	XR_001436532.1	lncRNA
XM_004002132.3	PRKACB	mRNA	XR_001436532.1	lncRNA
XM_004002132.3	PRKACB	mRNA	XR_001436532.1	lncRNA
XM_004002132.3	PRKACB	mRNA	XR_001436532.1	lncRNA
XM_004021448.3	ASCC1	mRNA	XR_001436532.1	lncRNA
XM_004021448.3	ASCC1	mRNA	XR_001436532.1	lncRNA
XM_004021448.3	ASCC1	mRNA	XR_001436532.1	lncRNA
XM_012102580.2	FRA10AC1	mRNA	XR_001042381.2	lncRNA
XM_012102580.2	FRA10AC1	mRNA	XR_001042381.2	lncRNA
XM_012102580.2	FRA10AC1	mRNA	XR_001042381.2	lncRNA
XM_015103361.1	ZFYVE27	mRNA	XR_001042381.2	lncRNA
XM_015103361.1	ZFYVE27	mRNA	XR_001042381.2	lncRNA
XM_015103361.1	ZFYVE27	mRNA	XR_001042381.2	lncRNA
XM_015094761.1	SLC35E3	mRNA	TCONS_00075453	lncRNA
XM_015094761.1	SLC35E3	mRNA	TCONS_00075453	lncRNA
XM_015094761.1	SLC35E3	mRNA	TCONS_00075453	lncRNA
XM_004014443.3	RAE1	mRNA	TCONS_00075453	lncRNA
XM_004014443.3	RAE1	mRNA	TCONS_00075453	lncRNA
XM_004014443.3	RAE1	mRNA	TCONS_00075453	lncRNA
XM_012177588.2	NR3C1	mRNA	TCONS_00075453	lncRNA
XM_012177588.2	NR3C1	mRNA	TCONS_00075453	lncRNA
XM_012177588.2	NR3C1	mRNA	TCONS_00075453	lncRNA
XM_004009100.3	PAM	mRNA	TCONS_00082638	lncRNA
XM_004009100.3	PAM	mRNA	TCONS_00082638	lncRNA
XM_004009100.3	PAM	mRNA	TCONS_00082638	lncRNA
XM_012181326.2	MAP4K5	mRNA	TCONS_00082638	lncRNA
XM_012181326.2	MAP4K5	mRNA	TCONS_00082638	lncRNA
XM_012181326.2	MAP4K5	mRNA	TCONS_00082638	lncRNA
XM_004023170.3	LOC101108147	mRNA	TCONS_00075051	lncRNA
XM_004023170.3	LOC101108147	mRNA	TCONS_00075051	lncRNA
XM_004023170.3	LOC101108147	mRNA	TCONS_00075051	lncRNA
XM_012187546.2	ANKRD45	mRNA	TCONS_00082638	lncRNA
XM_012187546.2	ANKRD45	mRNA	TCONS_00082638	lncRNA
XM_012187546.2	ANKRD45	mRNA	TCONS_00082638	lncRNA
XM_012102512.2	HEYL	mRNA	TCONS_00084305	lncRNA
XM_012102512.2	HEYL	mRNA	TCONS_00084305	lncRNA
XM_012102512.2	HEYL	mRNA	TCONS_00084305	lncRNA
XM_015098434.1	STXBP4	mRNA	TCONS_00084305	lncRNA
XM_015098434.1	STXBP4	mRNA	TCONS_00084305	lncRNA
XM_015098434.1	STXBP4	mRNA	TCONS_00084305	lncRNA
XM_004006510.3	SLC16A7	mRNA	TCONS_00078442	lncRNA
XM_004006510.3	SLC16A7	mRNA	TCONS_00078442	lncRNA
XM_004006510.3	SLC16A7	mRNA	TCONS_00078442	lncRNA
XM_015092063.1	FUBP1	mRNA	TCONS_00084305	lncRNA
XM_015092063.1	FUBP1	mRNA	TCONS_00084305	lncRNA
XM_015092063.1	FUBP1	mRNA	TCONS_00084305	lncRNA
XM_012184401.1	ZC3H13	mRNA	TCONS_00084305	lncRNA
XM_012184401.1	ZC3H13	mRNA	TCONS_00084305	lncRNA
XM_012184401.1	ZC3H13	mRNA	TCONS_00084305	lncRNA
XM_015104846.1	TAB3	mRNA	TCONS_00078442	lncRNA
XM_015104846.1	TAB3	mRNA	TCONS_00078442	lncRNA
XM_015104846.1	TAB3	mRNA	TCONS_00078442	lncRNA
XM_015104948.1	RPS6KA6	mRNA	TCONS_00078440	lncRNA

XM_015104948.1	RPS6KA6	mRNA	TCONS_00078440	lncRNA
XM_015104948.1	RPS6KA6	mRNA	TCONS_00078440	lncRNA
XM_004002227.3	SLC35A3	mRNA	XR_001041211.2	lncRNA
XM_004002227.3	SLC35A3	mRNA	XR_001041211.2	lncRNA
XM_004002227.3	SLC35A3	mRNA	XR_001041211.2	lncRNA
XM_015102923.1	KCTD14	mRNA	XR_001041211.2	lncRNA
XM_015102923.1	KCTD14	mRNA	XR_001041211.2	lncRNA
XM_015102923.1	KCTD14	mRNA	XR_001041211.2	lncRNA
XM_004002132.3	PRKACB	mRNA	XR_001041211.2	lncRNA
XM_004002132.3	PRKACB	mRNA	XR_001041211.2	lncRNA
XM_004002132.3	PRKACB	mRNA	XR_001041211.2	lncRNA
XM_012174558.1	CNOT2	mRNA	XR_001041211.2	lncRNA
XM_012174558.1	CNOT2	mRNA	XR_001041211.2	lncRNA
XM_012174558.1	CNOT2	mRNA	XR_001041211.2	lncRNA
XM_004021448.3	ASCC1	mRNA	XR_001436817.1	lncRNA
XM_004021448.3	ASCC1	mRNA	XR_001436817.1	lncRNA
XM_004021448.3	ASCC1	mRNA	XR_001436817.1	lncRNA
XM_015104655.1	NKIRAS1	mRNA	XR_001434203.1	lncRNA
XM_015104655.1	NKIRAS1	mRNA	XR_001434203.1	lncRNA
XM_015104655.1	NKIRAS1	mRNA	XR_001434203.1	lncRNA
XM_004002227.3	SLC35A3	mRNA	XR_001436817.1	lncRNA
XM_004002227.3	SLC35A3	mRNA	XR_001436817.1	lncRNA
XM_004002227.3	SLC35A3	mRNA	XR_001436817.1	lncRNA
XM_015104655.1	NKIRAS1	mRNA	XR_001436817.1	lncRNA
XM_015104655.1	NKIRAS1	mRNA	XR_001436817.1	lncRNA
XM_015104655.1	NKIRAS1	mRNA	XR_001436817.1	lncRNA
XM_015103361.1	ZFYVE27	mRNA	XR_001436817.1	lncRNA
XM_015103361.1	ZFYVE27	mRNA	XR_001436817.1	lncRNA
XM_015103361.1	ZFYVE27	mRNA	XR_001436817.1	lncRNA
XM_015102923.1	KCTD14	mRNA	XR_001436817.1	lncRNA
XM_015102923.1	KCTD14	mRNA	XR_001436817.1	lncRNA
XM_015102923.1	KCTD14	mRNA	XR_001436817.1	lncRNA
XM_012144092.2	METTL21A	mRNA	XR_001436817.1	lncRNA
XM_012144092.2	METTL21A	mRNA	XR_001436817.1	lncRNA
XM_012144092.2	METTL21A	mRNA	XR_001436817.1	lncRNA
XM_012175208.1	ODF2L	mRNA	XR_001434203.1	lncRNA
XM_012175208.1	ODF2L	mRNA	XR_001434203.1	lncRNA
XM_012175208.1	ODF2L	mRNA	XR_001434203.1	lncRNA
XM_012102580.2	FRA10AC1	mRNA	XR_001434203.1	lncRNA
XM_012102580.2	FRA10AC1	mRNA	XR_001434203.1	lncRNA
XM_012102580.2	FRA10AC1	mRNA	XR_001434203.1	lncRNA
XM_012102580.2	FRA10AC1	mRNA	XR_001436817.1	lncRNA
XM_012102580.2	FRA10AC1	mRNA	XR_001436817.1	lncRNA
XM_012102580.2	FRA10AC1	mRNA	XR_001436817.1	lncRNA
XM_012171234.2	CFAP36	mRNA	XR_001436817.1	lncRNA
XM_012171234.2	CFAP36	mRNA	XR_001436817.1	lncRNA
XM_012171234.2	CFAP36	mRNA	XR_001436817.1	lncRNA
XM_012153155.2	HP1BP3	mRNA	XR_001033284.2	lncRNA
XM_012153155.2	HP1BP3	mRNA	XR_001033284.2	lncRNA
XM_012153155.2	HP1BP3	mRNA	XR_001033284.2	lncRNA
XM_012119403.2	BIN3	mRNA	XR_001033284.2	lncRNA
XM_012119403.2	BIN3	mRNA	XR_001033284.2	lncRNA
XM_012119403.2	BIN3	mRNA	XR_001033284.2	lncRNA
XM_012098757.1	G2E3	mRNA	XR_001020707.2	lncRNA

XM_012098757.1	G2E3	mRNA	XR_001020707.2	lncRNA
XM_012098757.1	G2E3	mRNA	XR_001020707.2	lncRNA
XM_004014443.3	RAE1	mRNA	XR_001033284.2	lncRNA
XM_004014443.3	RAE1	mRNA	XR_001033284.2	lncRNA
XM_004014443.3	RAE1	mRNA	XR_001033284.2	lncRNA
XM_015092883.1	PADI1	mRNA	TCONS_00084305	lncRNA
XM_015092883.1	PADI1	mRNA	TCONS_00084305	lncRNA
XM_015092883.1	PADI1	mRNA	TCONS_00084305	lncRNA
XM_012174768.2	RAB5B	mRNA	XR_001436931.1	lncRNA
XM_012174768.2	RAB5B	mRNA	XR_001436931.1	lncRNA
XM_012174768.2	RAB5B	mRNA	XR_001436931.1	lncRNA
XM_004003552.3	LOC10112182(	mRNA	TCONS_00054422	lncRNA
XM_004003552.3	LOC10112182(	mRNA	TCONS_00054422	lncRNA
XM_004003552.3	LOC10112182(	mRNA	TCONS_00054422	lncRNA
XM_012190630.2	PLEKHA4	mRNA	TCONS_00078442	lncRNA
XM_012190630.2	PLEKHA4	mRNA	TCONS_00078442	lncRNA
XM_012190630.2	PLEKHA4	mRNA	TCONS_00078442	lncRNA
XM_012174768.2	RAB5B	mRNA	TCONS_00084305	lncRNA
XM_012174768.2	RAB5B	mRNA	TCONS_00084305	lncRNA
XM_012174768.2	RAB5B	mRNA	TCONS_00084305	lncRNA
XM_012098348.2	MEF2A	mRNA	TCONS_00078442	lncRNA
XM_012098348.2	MEF2A	mRNA	TCONS_00078442	lncRNA
XM_012098348.2	MEF2A	mRNA	TCONS_00078442	lncRNA
XM_012102658.2	ZNF518A	mRNA	TCONS_00078442	lncRNA
XM_012102658.2	ZNF518A	mRNA	TCONS_00078442	lncRNA
XM_012102658.2	ZNF518A	mRNA	TCONS_00078442	lncRNA
XM_012098306.1	UBE3A	mRNA	TCONS_00084305	lncRNA
XM_012098306.1	UBE3A	mRNA	TCONS_00084305	lncRNA
XM_012098306.1	UBE3A	mRNA	TCONS_00084305	lncRNA
XM_012106834.1	OPHN1	mRNA	TCONS_00022613	lncRNA
XM_012106834.1	OPHN1	mRNA	TCONS_00022613	lncRNA
XM_012106834.1	OPHN1	mRNA	TCONS_00022613	lncRNA
XM_015096552.1	MOB1B	mRNA	TCONS_00022613	lncRNA
XM_015096552.1	MOB1B	mRNA	TCONS_00022613	lncRNA
XM_015096552.1	MOB1B	mRNA	TCONS_00022613	lncRNA
XM_012177629.2	CLCC1	mRNA	XR_001436102.1	lncRNA
XM_012177629.2	CLCC1	mRNA	XR_001436102.1	lncRNA
XM_012177629.2	CLCC1	mRNA	XR_001436102.1	lncRNA
XM_012174040.2	SLC6A15	mRNA	XR_001021689.2	lncRNA
XM_012174040.2	SLC6A15	mRNA	XR_001021689.2	lncRNA
XM_012174040.2	SLC6A15	mRNA	XR_001021689.2	lncRNA
XM_012107496.2	PLS3	mRNA	XR_001021689.2	lncRNA
XM_012107496.2	PLS3	mRNA	XR_001021689.2	lncRNA
XM_012107496.2	PLS3	mRNA	XR_001021689.2	lncRNA
XM_015096385.1	TBCK	mRNA	XR_001436102.1	lncRNA
XM_015096385.1	TBCK	mRNA	XR_001436102.1	lncRNA
XM_015096385.1	TBCK	mRNA	XR_001436102.1	lncRNA
XM_012183145.1	MTRF1L	mRNA	XR_001021689.2	lncRNA
XM_012183145.1	MTRF1L	mRNA	XR_001021689.2	lncRNA
XM_012183145.1	MTRF1L	mRNA	XR_001021689.2	lncRNA
XM_015092228.1	CIART	mRNA	XR_001433755.1	lncRNA
XM_015092228.1	CIART	mRNA	XR_001433755.1	lncRNA
XM_015092228.1	CIART	mRNA	XR_001433755.1	lncRNA
XM_004004348.2	RFX3	mRNA	TCONS_00078440	lncRNA

XM_004004348.2	RFX3	mRNA	TCONS_00078440	lncRNA
XM_004004348.2	RFX3	mRNA	TCONS_00078440	lncRNA
XM_004014209.3	CUL2	mRNA	TCONS_00078440	lncRNA
XM_004014209.3	CUL2	mRNA	TCONS_00078440	lncRNA
XM_004014209.3	CUL2	mRNA	TCONS_00078440	lncRNA
XM_004014196.3	ABI1	mRNA	XR_001433755.1	lncRNA
XM_004014196.3	ABI1	mRNA	XR_001433755.1	lncRNA
XM_004014196.3	ABI1	mRNA	XR_001433755.1	lncRNA
XM_004004348.2	RFX3	mRNA	XR_001433755.1	lncRNA
XM_004004348.2	RFX3	mRNA	XR_001433755.1	lncRNA
XM_004004348.2	RFX3	mRNA	XR_001433755.1	lncRNA
XM_012182435.2	C1H1orf43	mRNA	XR_001436127.1	lncRNA
XM_012182435.2	C1H1orf43	mRNA	XR_001436127.1	lncRNA
XM_012182435.2	C1H1orf43	mRNA	XR_001436127.1	lncRNA
NM_001161880.1	CDKN1A	mRNA	XR_001436127.1	lncRNA
NM_001161880.1	CDKN1A	mRNA	XR_001436127.1	lncRNA
NM_001161880.1	CDKN1A	mRNA	XR_001436127.1	lncRNA
XM_004011187.3	LOC10110720(	mRNA	XR_001436127.1	lncRNA
XM_004011187.3	LOC10110720(	mRNA	XR_001436127.1	lncRNA
XM_004011187.3	LOC10110720(	mRNA	XR_001436127.1	lncRNA
XM_012098754.1	G2E3	mRNA	XR_001024290.1	lncRNA
XM_012098754.1	G2E3	mRNA	XR_001024290.1	lncRNA
XM_012098754.1	G2E3	mRNA	XR_001024290.1	lncRNA
XM_012107644.2	FAM199X	mRNA	XR_001024290.1	lncRNA
XM_012107644.2	FAM199X	mRNA	XR_001024290.1	lncRNA
XM_012107644.2	FAM199X	mRNA	XR_001024290.1	lncRNA
XM_012182400.2	SENP6	mRNA	XR_001024290.1	lncRNA
XM_012182400.2	SENP6	mRNA	XR_001024290.1	lncRNA
XM_012182400.2	SENP6	mRNA	XR_001024290.1	lncRNA
XM_015098513.1	RHOT1	mRNA	XR_001024290.1	lncRNA
XM_015098513.1	RHOT1	mRNA	XR_001024290.1	lncRNA
XM_015098513.1	RHOT1	mRNA	XR_001024290.1	lncRNA
XM_012105431.2	GHITM	mRNA	XR_001024290.1	lncRNA
XM_012105431.2	GHITM	mRNA	XR_001024290.1	lncRNA
XM_012105431.2	GHITM	mRNA	XR_001024290.1	lncRNA
XM_004010813.3	GSTM1	mRNA	TCONS_00078442	lncRNA
XM_004010813.3	GSTM1	mRNA	TCONS_00078442	lncRNA
XM_004010813.3	GSTM1	mRNA	TCONS_00078442	lncRNA
XM_004006548.3	R3HDM2	mRNA	TCONS_00054422	lncRNA
XM_004006548.3	R3HDM2	mRNA	TCONS_00054422	lncRNA
XM_004006548.3	R3HDM2	mRNA	TCONS_00054422	lncRNA
XM_012180554.2	TARS2	mRNA	TCONS_00084305	lncRNA
XM_012180554.2	TARS2	mRNA	TCONS_00084305	lncRNA
XM_012180554.2	TARS2	mRNA	TCONS_00084305	lncRNA
XM_012105331.2	USP54	mRNA	XR_001436931.1	lncRNA
XM_012105331.2	USP54	mRNA	XR_001436931.1	lncRNA
XM_012105331.2	USP54	mRNA	XR_001436931.1	lncRNA
XM_012182238.1	SPG11	mRNA	TCONS_00075051	lncRNA
XM_012182238.1	SPG11	mRNA	TCONS_00075051	lncRNA
XM_012182238.1	SPG11	mRNA	TCONS_00075051	lncRNA
XM_012102629.2	SORBS1	mRNA	TCONS_00084305	lncRNA
XM_012102629.2	SORBS1	mRNA	TCONS_00084305	lncRNA
XM_012102629.2	SORBS1	mRNA	TCONS_00084305	lncRNA
XM_004005653.3	ZBTB26	mRNA	XR_001436931.1	lncRNA

XM_004005653.3	ZBTB26	mRNA	XR_001436931.1	lncRNA
XM_004005653.3	ZBTB26	mRNA	XR_001436931.1	lncRNA
XM_012135865.2	TANK	mRNA	XR_001034847.2	lncRNA
XM_012135865.2	TANK	mRNA	XR_001034847.2	lncRNA
XM_012135865.2	TANK	mRNA	XR_001034847.2	lncRNA
XM_012135865.2	TANK	mRNA	XR_001434202.1	lncRNA
XM_012135865.2	TANK	mRNA	XR_001434202.1	lncRNA
XM_012135865.2	TANK	mRNA	XR_001434202.1	lncRNA
XM_015097725.1	FAM49B	mRNA	TCONS_00075453	lncRNA
XM_015097725.1	FAM49B	mRNA	TCONS_00075453	lncRNA
XM_015097725.1	FAM49B	mRNA	TCONS_00075453	lncRNA
XM_015098575.1	METTL16	mRNA	TCONS_00075453	lncRNA
XM_015098575.1	METTL16	mRNA	TCONS_00075453	lncRNA
XM_015098575.1	METTL16	mRNA	TCONS_00075453	lncRNA
XM_012106834.1	OPHN1	mRNA	TCONS_00075453	lncRNA
XM_012106834.1	OPHN1	mRNA	TCONS_00075453	lncRNA
XM_012106834.1	OPHN1	mRNA	TCONS_00075453	lncRNA
XM_012148064.2	NDC1	mRNA	TCONS_00075453	lncRNA
XM_012148064.2	NDC1	mRNA	TCONS_00075453	lncRNA
XM_012148064.2	NDC1	mRNA	TCONS_00075453	lncRNA
XM_015092529.1	MCCC1	mRNA	TCONS_00075453	lncRNA
XM_015092529.1	MCCC1	mRNA	TCONS_00075453	lncRNA
XM_015092529.1	MCCC1	mRNA	TCONS_00075453	lncRNA
XM_012182450.2	IBTK	mRNA	XR_001024743.2	lncRNA
XM_012182450.2	IBTK	mRNA	XR_001024743.2	lncRNA
XM_012182450.2	IBTK	mRNA	XR_001024743.2	lncRNA
XM_012104181.2	CCP110	mRNA	TCONS_00084305	lncRNA
XM_012104181.2	CCP110	mRNA	TCONS_00084305	lncRNA
XM_012104181.2	CCP110	mRNA	TCONS_00084305	lncRNA
XM_012105332.2	USP54	mRNA	TCONS_00078442	lncRNA
XM_012105332.2	USP54	mRNA	TCONS_00078442	lncRNA
XM_012105332.2	USP54	mRNA	TCONS_00078442	lncRNA
XM_015097502.1	SNX9	mRNA	TCONS_00082638	lncRNA
XM_015097502.1	SNX9	mRNA	TCONS_00082638	lncRNA
XM_015097502.1	SNX9	mRNA	TCONS_00082638	lncRNA
XM_012105319.2	USP54	mRNA	TCONS_00084305	lncRNA
XM_012105319.2	USP54	mRNA	TCONS_00084305	lncRNA
XM_012105319.2	USP54	mRNA	TCONS_00084305	lncRNA
XM_004011865.3	ENPP2	mRNA	TCONS_00084305	lncRNA
XM_004011865.3	ENPP2	mRNA	TCONS_00084305	lncRNA
XM_004011865.3	ENPP2	mRNA	TCONS_00084305	lncRNA
XM_012105332.2	USP54	mRNA	TCONS_00084305	lncRNA
XM_012105332.2	USP54	mRNA	TCONS_00084305	lncRNA
XM_012105332.2	USP54	mRNA	TCONS_00084305	lncRNA
XM_012189779.2	RBL2	mRNA	TCONS_00054422	lncRNA
XM_012189779.2	RBL2	mRNA	TCONS_00054422	lncRNA
XM_012189779.2	RBL2	mRNA	TCONS_00054422	lncRNA
XM_012190139.2	ZMYM6	mRNA	TCONS_00078440	lncRNA
XM_012190139.2	ZMYM6	mRNA	TCONS_00078440	lncRNA
XM_012190139.2	ZMYM6	mRNA	TCONS_00078440	lncRNA
XM_015104948.1	RPS6KA6	mRNA	TCONS_00022613	lncRNA
XM_015104948.1	RPS6KA6	mRNA	TCONS_00022613	lncRNA
XM_015104948.1	RPS6KA6	mRNA	TCONS_00022613	lncRNA
XM_012102598.2	TBC1D12	mRNA	XR_001434892.1	lncRNA

XM_012102598.2	TBC1D12	mRNA	XR_001434892.1	lncRNA
XM_012102598.2	TBC1D12	mRNA	XR_001434892.1	lncRNA
XM_012135865.2	TANK	mRNA	XR_001434761.1	lncRNA
XM_012135865.2	TANK	mRNA	XR_001434761.1	lncRNA
XM_012135865.2	TANK	mRNA	XR_001434761.1	lncRNA
XM_012186398.2	DUS1L	mRNA	XR_001434761.1	lncRNA
XM_012186398.2	DUS1L	mRNA	XR_001434761.1	lncRNA
XM_012186398.2	DUS1L	mRNA	XR_001434761.1	lncRNA
XM_012148064.2	NDC1	mRNA	XR_001033284.2	lncRNA
XM_012148064.2	NDC1	mRNA	XR_001033284.2	lncRNA
XM_012148064.2	NDC1	mRNA	XR_001033284.2	lncRNA
XM_012096744.2	GFM2	mRNA	XR_001020707.2	lncRNA
XM_012096744.2	GFM2	mRNA	XR_001020707.2	lncRNA
XM_012096744.2	GFM2	mRNA	XR_001020707.2	lncRNA
XM_012103465.2	OSBPL1A	mRNA	XR_001033284.2	lncRNA
XM_012103465.2	OSBPL1A	mRNA	XR_001033284.2	lncRNA
XM_012103465.2	OSBPL1A	mRNA	XR_001033284.2	lncRNA
XM_015102039.1	SYNE3	mRNA	XR_001024743.2	lncRNA
XM_015102039.1	SYNE3	mRNA	XR_001024743.2	lncRNA
XM_015102039.1	SYNE3	mRNA	XR_001024743.2	lncRNA
XM_012098820.2	RALGAPA1	mRNA	TCONS_00082638	lncRNA
XM_012098820.2	RALGAPA1	mRNA	TCONS_00082638	lncRNA
XM_012098820.2	RALGAPA1	mRNA	TCONS_00082638	lncRNA
XM_015096594.1	SEC31A	mRNA	TCONS_00078442	lncRNA
XM_015096594.1	SEC31A	mRNA	TCONS_00078442	lncRNA
XM_015096594.1	SEC31A	mRNA	TCONS_00078442	lncRNA
XM_012184885.2	DOCK9	mRNA	TCONS_00084305	lncRNA
XM_012184885.2	DOCK9	mRNA	TCONS_00084305	lncRNA
XM_012184885.2	DOCK9	mRNA	TCONS_00084305	lncRNA
XM_012119517.2	SORBS3	mRNA	TCONS_00078442	lncRNA
XM_012119517.2	SORBS3	mRNA	TCONS_00078442	lncRNA
XM_012119517.2	SORBS3	mRNA	TCONS_00078442	lncRNA
XM_012178308.2	FAM193B	mRNA	TCONS_00054422	lncRNA
XM_012178308.2	FAM193B	mRNA	TCONS_00054422	lncRNA
XM_012178308.2	FAM193B	mRNA	TCONS_00054422	lncRNA
XM_012182538.2	TRAPPC3L	mRNA	TCONS_00084305	lncRNA
XM_012182538.2	TRAPPC3L	mRNA	TCONS_00084305	lncRNA
XM_012182538.2	TRAPPC3L	mRNA	TCONS_00084305	lncRNA
XM_015097132.1	TMEM229B	mRNA	TCONS_00078442	lncRNA
XM_015097132.1	TMEM229B	mRNA	TCONS_00078442	lncRNA
XM_015097132.1	TMEM229B	mRNA	TCONS_00078442	lncRNA
XM_012100690.1	KLC4	mRNA	TCONS_00078442	lncRNA
XM_012100690.1	KLC4	mRNA	TCONS_00078442	lncRNA
XM_012100690.1	KLC4	mRNA	TCONS_00078442	lncRNA
XM_015096356.1	SEC24D	mRNA	TCONS_00078442	lncRNA
XM_015096356.1	SEC24D	mRNA	TCONS_00078442	lncRNA
XM_015096356.1	SEC24D	mRNA	TCONS_00078442	lncRNA
XM_012094768.2	ZMYM1	mRNA	TCONS_00075453	lncRNA
XM_012094768.2	ZMYM1	mRNA	TCONS_00075453	lncRNA
XM_012094768.2	ZMYM1	mRNA	TCONS_00075453	lncRNA
XM_012189374.1	NXPE3	mRNA	TCONS_00075453	lncRNA
XM_012189374.1	NXPE3	mRNA	TCONS_00075453	lncRNA
XM_012189374.1	NXPE3	mRNA	TCONS_00075453	lncRNA
XM_012095771.2	CTR9	mRNA	TCONS_00075453	lncRNA

XM_012095771.2	CTR9	mRNA	TCONS_00075453	lncRNA
XM_012095771.2	CTR9	mRNA	TCONS_00075453	lncRNA
XM_015094627.1	PCBP2	mRNA	XR_001025491.1	lncRNA
XM_015094627.1	PCBP2	mRNA	XR_001025491.1	lncRNA
XM_015094627.1	PCBP2	mRNA	XR_001025491.1	lncRNA
XM_012148796.2	DIS3L2	mRNA	XR_001044126.1	lncRNA
XM_012148796.2	DIS3L2	mRNA	XR_001044126.1	lncRNA
XM_012148796.2	DIS3L2	mRNA	XR_001044126.1	lncRNA
XM_015099539.1	KIN	mRNA	XR_001436127.1	lncRNA
XM_015099539.1	KIN	mRNA	XR_001436127.1	lncRNA
XM_015099539.1	KIN	mRNA	XR_001436127.1	lncRNA
XM_012106834.1	OPHN1	mRNA	XR_001025491.1	lncRNA
XM_012106834.1	OPHN1	mRNA	XR_001025491.1	lncRNA
XM_012106834.1	OPHN1	mRNA	XR_001025491.1	lncRNA
XM_015096552.1	MOB1B	mRNA	XR_001044126.1	lncRNA
XM_015096552.1	MOB1B	mRNA	XR_001044126.1	lncRNA
XM_015096552.1	MOB1B	mRNA	XR_001044126.1	lncRNA
XM_012152472.1	LUZP1	mRNA	TCONS_00084305	lncRNA
XM_012152472.1	LUZP1	mRNA	TCONS_00084305	lncRNA
XM_012152472.1	LUZP1	mRNA	TCONS_00084305	lncRNA
XM_012184887.2	DOCK9	mRNA	TCONS_00082638	lncRNA
XM_012184887.2	DOCK9	mRNA	TCONS_00082638	lncRNA
XM_012184887.2	DOCK9	mRNA	TCONS_00082638	lncRNA
XM_012182026.2	ENTPD5	mRNA	TCONS_00078442	lncRNA
XM_012182026.2	ENTPD5	mRNA	TCONS_00078442	lncRNA
XM_012182026.2	ENTPD5	mRNA	TCONS_00078442	lncRNA
XM_012097680.2	SCARB1	mRNA	TCONS_00078442	lncRNA
XM_012097680.2	SCARB1	mRNA	TCONS_00078442	lncRNA
XM_012097680.2	SCARB1	mRNA	TCONS_00078442	lncRNA
XM_015099256.1	CEP104	mRNA	TCONS_00054422	lncRNA
XM_015099256.1	CEP104	mRNA	TCONS_00054422	lncRNA
XM_015099256.1	CEP104	mRNA	TCONS_00054422	lncRNA
XM_012097680.2	SCARB1	mRNA	TCONS_00084305	lncRNA
XM_012097680.2	SCARB1	mRNA	TCONS_00084305	lncRNA
XM_012097680.2	SCARB1	mRNA	TCONS_00084305	lncRNA
XM_012097035.1	C16H5orf42	mRNA	TCONS_00084305	lncRNA
XM_012097035.1	C16H5orf42	mRNA	TCONS_00084305	lncRNA
XM_012097035.1	C16H5orf42	mRNA	TCONS_00084305	lncRNA
XM_012177588.2	NR3C1	mRNA	XR_001436102.1	lncRNA
XM_012177588.2	NR3C1	mRNA	XR_001436102.1	lncRNA
XM_012177588.2	NR3C1	mRNA	XR_001436102.1	lncRNA
XM_012125926.2	ZDHHC21	mRNA	XR_001021689.2	lncRNA
XM_012125926.2	ZDHHC21	mRNA	XR_001021689.2	lncRNA
XM_012125926.2	ZDHHC21	mRNA	XR_001021689.2	lncRNA
XM_012181686.2	DLGAP5	mRNA	XR_001436532.1	lncRNA
XM_012181686.2	DLGAP5	mRNA	XR_001436532.1	lncRNA
XM_012181686.2	DLGAP5	mRNA	XR_001436532.1	lncRNA
XM_015092055.1	ZZZ3	mRNA	XR_001436532.1	lncRNA
XM_015092055.1	ZZZ3	mRNA	XR_001436532.1	lncRNA
XM_015092055.1	ZZZ3	mRNA	XR_001436532.1	lncRNA
XM_004002454.2	ECM1	mRNA	XR_001436532.1	lncRNA
XM_004002454.2	ECM1	mRNA	XR_001436532.1	lncRNA
XM_004002454.2	ECM1	mRNA	XR_001436532.1	lncRNA
XM_012176680.2	ARHGAP29	mRNA	TCONS_00022613	lncRNA

XM_012176680.2	ARHGAP29	mRNA	TCONS_00022613	lncRNA
XM_012176680.2	ARHGAP29	mRNA	TCONS_00022613	lncRNA
XM_012181686.2	DLGAP5	mRNA	XR_001041211.2	lncRNA
XM_012181686.2	DLGAP5	mRNA	XR_001041211.2	lncRNA
XM_012181686.2	DLGAP5	mRNA	XR_001041211.2	lncRNA
XM_012103840.2	SERPINB13	mRNA	XR_001041211.2	lncRNA
XM_012103840.2	SERPINB13	mRNA	XR_001041211.2	lncRNA
XM_012103840.2	SERPINB13	mRNA	XR_001041211.2	lncRNA
XM_012181456.2	ADAM10	mRNA	TCONS_00084305	lncRNA
XM_012181456.2	ADAM10	mRNA	TCONS_00084305	lncRNA
XM_012181456.2	ADAM10	mRNA	TCONS_00084305	lncRNA
XM_012176498.2	RAPGEF5	mRNA	TCONS_00078442	lncRNA
XM_012176498.2	RAPGEF5	mRNA	TCONS_00078442	lncRNA
XM_012176498.2	RAPGEF5	mRNA	TCONS_00078442	lncRNA
XM_004003942.3	COL6A6	mRNA	TCONS_00082638	lncRNA
XM_004003942.3	COL6A6	mRNA	TCONS_00082638	lncRNA
XM_004003942.3	COL6A6	mRNA	TCONS_00082638	lncRNA
XM_012144682.2	MAP2	mRNA	TCONS_00084305	lncRNA
XM_012144682.2	MAP2	mRNA	TCONS_00084305	lncRNA
XM_012144682.2	MAP2	mRNA	TCONS_00084305	lncRNA
XM_012098812.2	RALGAPA1	mRNA	TCONS_00078442	lncRNA
XM_012098812.2	RALGAPA1	mRNA	TCONS_00078442	lncRNA
XM_012098812.2	RALGAPA1	mRNA	TCONS_00078442	lncRNA
XM_012181456.2	ADAM10	mRNA	TCONS_00078442	lncRNA
XM_012181456.2	ADAM10	mRNA	TCONS_00078442	lncRNA
XM_012181456.2	ADAM10	mRNA	TCONS_00078442	lncRNA
XM_012144682.2	MAP2	mRNA	TCONS_00078442	lncRNA
XM_012144682.2	MAP2	mRNA	TCONS_00078442	lncRNA
XM_012144682.2	MAP2	mRNA	TCONS_00078442	lncRNA
XM_012177588.2	NR3C1	mRNA	XR_001034847.2	lncRNA
XM_012177588.2	NR3C1	mRNA	XR_001034847.2	lncRNA
XM_012177588.2	NR3C1	mRNA	XR_001034847.2	lncRNA
XM_012177588.2	NR3C1	mRNA	XR_001434202.1	lncRNA
XM_012177588.2	NR3C1	mRNA	XR_001434202.1	lncRNA
XM_012177588.2	NR3C1	mRNA	XR_001434202.1	lncRNA
XM_012094768.2	ZMYM1	mRNA	XR_001020707.2	lncRNA
XM_012094768.2	ZMYM1	mRNA	XR_001020707.2	lncRNA
XM_012094768.2	ZMYM1	mRNA	XR_001020707.2	lncRNA
XM_012095345.1	CCDC82	mRNA	XR_001020707.2	lncRNA
XM_012095345.1	CCDC82	mRNA	XR_001020707.2	lncRNA
XM_012095345.1	CCDC82	mRNA	XR_001020707.2	lncRNA
XM_012186914.2	ZC3H11A	mRNA	XR_001020707.2	lncRNA
XM_012186914.2	ZC3H11A	mRNA	XR_001020707.2	lncRNA
XM_012186914.2	ZC3H11A	mRNA	XR_001020707.2	lncRNA
XM_012094768.2	ZMYM1	mRNA	XR_001033284.2	lncRNA
XM_012094768.2	ZMYM1	mRNA	XR_001033284.2	lncRNA
XM_012094768.2	ZMYM1	mRNA	XR_001033284.2	lncRNA
XM_012187432.1	PRKCZ	mRNA	XR_001433755.1	lncRNA
XM_012187432.1	PRKCZ	mRNA	XR_001433755.1	lncRNA
XM_012187432.1	PRKCZ	mRNA	XR_001433755.1	lncRNA
XM_015091968.1	EPS15	mRNA	TCONS_00075453	lncRNA
XM_015091968.1	EPS15	mRNA	TCONS_00075453	lncRNA
XM_015091968.1	EPS15	mRNA	TCONS_00075453	lncRNA
XM_012188945.2	CSTF1	mRNA	TCONS_00075453	lncRNA

XM_012188945.2	CSTF1	mRNA	TCONS_00075453	lncRNA
XM_012188945.2	CSTF1	mRNA	TCONS_00075453	lncRNA
XM_012188276.2	CCDC7	mRNA	TCONS_00075453	lncRNA
XM_012188276.2	CCDC7	mRNA	TCONS_00075453	lncRNA
XM_012188276.2	CCDC7	mRNA	TCONS_00075453	lncRNA
XM_012099283.2	NEK10	mRNA	TCONS_00075453	lncRNA
XM_012099283.2	NEK10	mRNA	TCONS_00075453	lncRNA
XM_012099283.2	NEK10	mRNA	TCONS_00075453	lncRNA
XM_004004348.2	RFX3	mRNA	TCONS_00075453	lncRNA
XM_004004348.2	RFX3	mRNA	TCONS_00075453	lncRNA
XM_004004348.2	RFX3	mRNA	TCONS_00075453	lncRNA
XM_015099971.1	ZNF181	mRNA	TCONS_00075453	lncRNA
XM_015099971.1	ZNF181	mRNA	TCONS_00075453	lncRNA
XM_015099971.1	ZNF181	mRNA	TCONS_00075453	lncRNA
XM_004014209.3	CUL2	mRNA	TCONS_00075453	lncRNA
XM_004014209.3	CUL2	mRNA	TCONS_00075453	lncRNA
XM_004014209.3	CUL2	mRNA	TCONS_00075453	lncRNA
XM_012181966.2	SIPA1L1	mRNA	TCONS_00084305	lncRNA
XM_012181966.2	SIPA1L1	mRNA	TCONS_00084305	lncRNA
XM_012181966.2	SIPA1L1	mRNA	TCONS_00084305	lncRNA
XM_012096323.1	PHF21A	mRNA	TCONS_00082638	lncRNA
XM_012096323.1	PHF21A	mRNA	TCONS_00082638	lncRNA
XM_012096323.1	PHF21A	mRNA	TCONS_00082638	lncRNA
XM_015096573.1	SOWAHB	mRNA	TCONS_00084305	lncRNA
XM_015096573.1	SOWAHB	mRNA	TCONS_00084305	lncRNA
XM_015096573.1	SOWAHB	mRNA	TCONS_00084305	lncRNA
XM_012181960.2	SIPA1L1	mRNA	TCONS_00084305	lncRNA
XM_012181960.2	SIPA1L1	mRNA	TCONS_00084305	lncRNA
XM_012181960.2	SIPA1L1	mRNA	TCONS_00084305	lncRNA
XM_015102486.1	NR2C2	mRNA	TCONS_00084305	lncRNA
XM_015102486.1	NR2C2	mRNA	TCONS_00084305	lncRNA
XM_015102486.1	NR2C2	mRNA	TCONS_00084305	lncRNA
XM_012118246.2	DCAF12	mRNA	TCONS_00022613	lncRNA
XM_012118246.2	DCAF12	mRNA	TCONS_00022613	lncRNA
XM_012118246.2	DCAF12	mRNA	TCONS_00022613	lncRNA
XM_015104177.1	PRSS8	mRNA	XR_001044126.1	lncRNA
XM_015104177.1	PRSS8	mRNA	XR_001044126.1	lncRNA
XM_015104177.1	PRSS8	mRNA	XR_001044126.1	lncRNA
XM_012190563.2	ZMYM1	mRNA	XR_001025491.1	lncRNA
XM_012190563.2	ZMYM1	mRNA	XR_001025491.1	lncRNA
XM_012190563.2	ZMYM1	mRNA	XR_001025491.1	lncRNA
XM_012179693.1	PIGY	mRNA	XR_001044126.1	lncRNA
XM_012179693.1	PIGY	mRNA	XR_001044126.1	lncRNA
XM_012179693.1	PIGY	mRNA	XR_001044126.1	lncRNA
XM_004017713.3	LINS1	mRNA	XR_001025491.1	lncRNA
XM_004017713.3	LINS1	mRNA	XR_001025491.1	lncRNA
XM_004017713.3	LINS1	mRNA	XR_001025491.1	lncRNA
XM_012186914.2	ZC3H11A	mRNA	XR_001044126.1	lncRNA
XM_012186914.2	ZC3H11A	mRNA	XR_001044126.1	lncRNA
XM_012186914.2	ZC3H11A	mRNA	XR_001044126.1	lncRNA
XM_012101919.2	ZBTB44	mRNA	TCONS_00078440	lncRNA
XM_012101919.2	ZBTB44	mRNA	TCONS_00078440	lncRNA
XM_012101919.2	ZBTB44	mRNA	TCONS_00078440	lncRNA
XM_015097748.1	PCMTD1	mRNA	TCONS_00078440	lncRNA

XM_015097748.1	PCMTD1	mRNA	TCONS_00078440	lncRNA
XM_015097748.1	PCMTD1	mRNA	TCONS_00078440	lncRNA
XM_004005653.3	ZBTB26	mRNA	TCONS_00078440	lncRNA
XM_004005653.3	ZBTB26	mRNA	TCONS_00078440	lncRNA
XM_004005653.3	ZBTB26	mRNA	TCONS_00078440	lncRNA
XM_012105318.2	USP54	mRNA	XR_001433755.1	lncRNA
XM_012105318.2	USP54	mRNA	XR_001433755.1	lncRNA
XM_012105318.2	USP54	mRNA	XR_001433755.1	lncRNA
XM_012184045.2	ZNF706	mRNA	XR_001434761.1	lncRNA
XM_012184045.2	ZNF706	mRNA	XR_001434761.1	lncRNA
XM_012184045.2	ZNF706	mRNA	XR_001434761.1	lncRNA
XM_012174703.2	ZBTB39	mRNA	TCONS_00084305	lncRNA
XM_012174703.2	ZBTB39	mRNA	TCONS_00084305	lncRNA
XM_012174703.2	ZBTB39	mRNA	TCONS_00084305	lncRNA
XM_015096937.1	BMF	mRNA	TCONS_00078442	lncRNA
XM_015096937.1	BMF	mRNA	TCONS_00078442	lncRNA
XM_015096937.1	BMF	mRNA	TCONS_00078442	lncRNA
XM_015103591.1	KIAA1328	mRNA	XR_001436102.1	lncRNA
XM_015103591.1	KIAA1328	mRNA	XR_001436102.1	lncRNA
XM_015103591.1	KIAA1328	mRNA	XR_001436102.1	lncRNA
XM_004010212.3	MTX3	mRNA	XR_001020707.2	lncRNA
XM_004010212.3	MTX3	mRNA	XR_001020707.2	lncRNA
XM_004010212.3	MTX3	mRNA	XR_001020707.2	lncRNA
XM_015095425.1	CUL1	mRNA	XR_001020707.2	lncRNA
XM_015095425.1	CUL1	mRNA	XR_001020707.2	lncRNA
XM_015095425.1	CUL1	mRNA	XR_001020707.2	lncRNA
XM_015091968.1	EPS15	mRNA	XR_001033284.2	lncRNA
XM_015091968.1	EPS15	mRNA	XR_001033284.2	lncRNA
XM_015091968.1	EPS15	mRNA	XR_001033284.2	lncRNA
XM_015099971.1	ZNF181	mRNA	XR_001033284.2	lncRNA
XM_015099971.1	ZNF181	mRNA	XR_001033284.2	lncRNA
XM_015099971.1	ZNF181	mRNA	XR_001033284.2	lncRNA
XM_015092228.1	CIART	mRNA	XR_001033284.2	lncRNA
XM_015092228.1	CIART	mRNA	XR_001033284.2	lncRNA
XM_015092228.1	CIART	mRNA	XR_001033284.2	lncRNA
XM_015094471.1	CCDC142	mRNA	XR_001433755.1	lncRNA
XM_015094471.1	CCDC142	mRNA	XR_001433755.1	lncRNA
XM_015094471.1	CCDC142	mRNA	XR_001433755.1	lncRNA
XM_012100212.2	ATG7	mRNA	TCONS_00078440	lncRNA
XM_012100212.2	ATG7	mRNA	TCONS_00078440	lncRNA
XM_012100212.2	ATG7	mRNA	TCONS_00078440	lncRNA
XM_015092714.1	ANKRL18	mRNA	TCONS_00075453	lncRNA
XM_015092714.1	ANKRL18	mRNA	TCONS_00075453	lncRNA
XM_015092714.1	ANKRL18	mRNA	TCONS_00075453	lncRNA
XM_004009100.3	PAM	mRNA	TCONS_00022613	lncRNA
XM_004009100.3	PAM	mRNA	TCONS_00022613	lncRNA
XM_004009100.3	PAM	mRNA	TCONS_00022613	lncRNA
XM_015100438.1	CYLD	mRNA	TCONS_00022613	lncRNA
XM_015100438.1	CYLD	mRNA	TCONS_00022613	lncRNA
XM_015100438.1	CYLD	mRNA	TCONS_00022613	lncRNA
XM_012170864.2	PAPOLG	mRNA	XR_001024290.1	lncRNA
XM_012170864.2	PAPOLG	mRNA	XR_001024290.1	lncRNA
XM_012170864.2	PAPOLG	mRNA	XR_001024290.1	lncRNA
XM_004004932.2	STK36	mRNA	XR_001025491.1	lncRNA

XM_004004932.2	STK36	mRNA	XR_001025491.1	lncRNA
XM_004004932.2	STK36	mRNA	XR_001025491.1	lncRNA
XM_004020074.3	PIK3AP1	mRNA	TCONS_00078440	lncRNA
XM_004020074.3	PIK3AP1	mRNA	TCONS_00078440	lncRNA
XM_004020074.3	PIK3AP1	mRNA	TCONS_00078440	lncRNA
XM_012188301.2	NEBL	mRNA	TCONS_00078440	lncRNA
XM_012188301.2	NEBL	mRNA	TCONS_00078440	lncRNA
XM_012188301.2	NEBL	mRNA	TCONS_00078440	lncRNA
XM_012130346.2	UGGT1	mRNA	XR_001020707.2	lncRNA
XM_012130346.2	UGGT1	mRNA	XR_001020707.2	lncRNA
XM_012130346.2	UGGT1	mRNA	XR_001020707.2	lncRNA
XM_012096615.2	DLG1	mRNA	XR_001020707.2	lncRNA
XM_012096615.2	DLG1	mRNA	XR_001020707.2	lncRNA
XM_012096615.2	DLG1	mRNA	XR_001020707.2	lncRNA
XM_012101765.1	PRMT3	mRNA	XR_001033284.2	lncRNA
XM_012101765.1	PRMT3	mRNA	XR_001033284.2	lncRNA
XM_012101765.1	PRMT3	mRNA	XR_001033284.2	lncRNA
XM_012180705.1	PAPD4	mRNA	XR_001033284.2	lncRNA
XM_012180705.1	PAPD4	mRNA	XR_001033284.2	lncRNA
XM_012180705.1	PAPD4	mRNA	XR_001033284.2	lncRNA
XM_012094663.2	GRAMD1C	mRNA	XR_001020707.2	lncRNA
XM_012094663.2	GRAMD1C	mRNA	XR_001020707.2	lncRNA
XM_012094663.2	GRAMD1C	mRNA	XR_001020707.2	lncRNA
XM_015104948.1	RPS6KA6	mRNA	XR_001436102.1	lncRNA
XM_015104948.1	RPS6KA6	mRNA	XR_001436102.1	lncRNA
XM_015104948.1	RPS6KA6	mRNA	XR_001436102.1	lncRNA
XM_012189374.1	NXPE3	mRNA	XR_001436102.1	lncRNA
XM_012189374.1	NXPE3	mRNA	XR_001436102.1	lncRNA
XM_012189374.1	NXPE3	mRNA	XR_001436102.1	lncRNA
XM_015094913.1	PLCM1	mRNA	XR_001025491.1	lncRNA
XM_015094913.1	PLCM1	mRNA	XR_001025491.1	lncRNA
XM_015094913.1	PLCM1	mRNA	XR_001025491.1	lncRNA
XM_015096551.1	MOB1B	mRNA	XR_001025491.1	lncRNA
XM_015096551.1	MOB1B	mRNA	XR_001025491.1	lncRNA
XM_015096551.1	MOB1B	mRNA	XR_001025491.1	lncRNA
XM_015103753.1	MBD1	mRNA	XR_001025491.1	lncRNA
XM_015103753.1	MBD1	mRNA	XR_001025491.1	lncRNA
XM_015103753.1	MBD1	mRNA	XR_001025491.1	lncRNA
XM_012104950.1	TTC13	mRNA	TCONS_00022613	lncRNA
XM_012104950.1	TTC13	mRNA	TCONS_00022613	lncRNA
XM_012104950.1	TTC13	mRNA	TCONS_00022613	lncRNA
XM_012181456.2	ADAM10	mRNA	TCONS_00078440	lncRNA
XM_012181456.2	ADAM10	mRNA	TCONS_00078440	lncRNA
XM_012181456.2	ADAM10	mRNA	TCONS_00078440	lncRNA
XM_012104594.2	ZNF789	mRNA	XR_001034847.2	lncRNA
XM_012104594.2	ZNF789	mRNA	XR_001034847.2	lncRNA
XM_012104594.2	ZNF789	mRNA	XR_001034847.2	lncRNA
XM_012104594.2	ZNF789	mRNA	XR_001434202.1	lncRNA
XM_012104594.2	ZNF789	mRNA	XR_001434202.1	lncRNA
XM_012104594.2	ZNF789	mRNA	XR_001434202.1	lncRNA
NM_001290793.2	PEG10	mRNA	TCONS_00075453	lncRNA
NM_001290793.2	PEG10	mRNA	TCONS_00075453	lncRNA
NM_001290793.2	PEG10	mRNA	TCONS_00075453	lncRNA
XM_004023170.3	LOC10110814[illegible]	mRNA	XR_001033284.2	lncRNA

XM_004023170.3	LOC101108147	mRNA	XR_001033284.2	lncRNA
XM_004023170.3	LOC101108147	mRNA	XR_001033284.2	lncRNA
XM_012176510.2	FNBP1L	mRNA	TCONS_00022613	lncRNA
XM_012176510.2	FNBP1L	mRNA	TCONS_00022613	lncRNA
XM_012176510.2	FNBP1L	mRNA	TCONS_00022613	lncRNA
XM_012188214.2	ABI1	mRNA	XR_001021689.2	lncRNA
XM_012188214.2	ABI1	mRNA	XR_001021689.2	lncRNA
XM_012188214.2	ABI1	mRNA	XR_001021689.2	lncRNA
XM_012127663.2	IFT74	mRNA	XR_001436102.1	lncRNA
XM_012127663.2	IFT74	mRNA	XR_001436102.1	lncRNA
XM_012127663.2	IFT74	mRNA	XR_001436102.1	lncRNA
XM_015104327.1	LOC101115427	mRNA	XR_001436102.1	lncRNA
XM_015104327.1	LOC101115427	mRNA	XR_001436102.1	lncRNA
XM_015104327.1	LOC101115427	mRNA	XR_001436102.1	lncRNA
XM_004002178.3	LRRC8B	mRNA	XR_001044126.1	lncRNA
XM_004002178.3	LRRC8B	mRNA	XR_001044126.1	lncRNA
XM_004002178.3	LRRC8B	mRNA	XR_001044126.1	lncRNA
XM_012107808.1	NFYC	mRNA	XR_001044126.1	lncRNA
XM_012107808.1	NFYC	mRNA	XR_001044126.1	lncRNA
XM_012107808.1	NFYC	mRNA	XR_001044126.1	lncRNA
XM_012174768.2	RAB5B	mRNA	XR_001044126.1	lncRNA
XM_012174768.2	RAB5B	mRNA	XR_001044126.1	lncRNA
XM_012174768.2	RAB5B	mRNA	XR_001044126.1	lncRNA
XM_012102669.1	ZNF518A	mRNA	XR_001044126.1	lncRNA
XM_012102669.1	ZNF518A	mRNA	XR_001044126.1	lncRNA
XM_012102669.1	ZNF518A	mRNA	XR_001044126.1	lncRNA
XM_012110487.2	UBQLN1	mRNA	XR_001025491.1	lncRNA
XM_012110487.2	UBQLN1	mRNA	XR_001025491.1	lncRNA
XM_012110487.2	UBQLN1	mRNA	XR_001025491.1	lncRNA
XM_012105331.2	USP54	mRNA	XR_001033284.2	lncRNA
XM_012105331.2	USP54	mRNA	XR_001033284.2	lncRNA
XM_012105331.2	USP54	mRNA	XR_001033284.2	lncRNA
XM_015097502.1	SNX9	mRNA	XR_001044126.1	lncRNA
XM_015097502.1	SNX9	mRNA	XR_001044126.1	lncRNA
XM_015097502.1	SNX9	mRNA	XR_001044126.1	lncRNA

Large and small waves

mRNA	Genesymbol	type	lncRNA	type
XM_004017729.3	CHL1	mRNA	XR_001435107.1	lncRNA
XM_004017729.3	CHL1	mRNA	XR_001435107.1	lncRNA
XM_004017729.3	CHL1	mRNA	XR_001435107.1	lncRNA
XM_004017729.3	CHL1	mRNA	XR_001435107.1	lncRNA
XM_004017729.3	CHL1	mRNA	XR_001435107.1	lncRNA
XM_004017729.3	CHL1	mRNA	XR_001435107.1	lncRNA
XM_004017729.3	CHL1	mRNA	XR_001435107.1	lncRNA
XM_004017729.3	CHL1	mRNA	XR_001435107.1	lncRNA
XM_004017729.3	CHL1	mRNA	XR_001435107.1	lncRNA
XM_004017729.3	CHL1	mRNA	XR_001435107.1	lncRNA
XM_004017729.3	CHL1	mRNA	XR_001435107.1	lncRNA
XM_004017729.3	CHL1	mRNA	XR_001435107.1	lncRNA
XM_012186362.1	PITPNC1	mRNA	XR_001436817.1	lncRNA
XM_012186362.1	PITPNC1	mRNA	XR_001436817.1	lncRNA
XM_012186362.1	PITPNC1	mRNA	XR_001436817.1	lncRNA
XM_012186362.1	PITPNC1	mRNA	XR_001436817.1	lncRNA
XM_012186362.1	PITPNC1	mRNA	XR_001436817.1	lncRNA
XM_012186362.1	PITPNC1	mRNA	XR_001436817.1	lncRNA
XM_012186362.1	PITPNC1	mRNA	XR_001436817.1	lncRNA
XM_012186362.1	PITPNC1	mRNA	XR_001436817.1	lncRNA
XM_012186362.1	PITPNC1	mRNA	XR_001436817.1	lncRNA
XM_012186362.1	PITPNC1	mRNA	XR_001436817.1	lncRNA
XM_012186362.1	PITPNC1	mRNA	XR_001436817.1	lncRNA
XM_012186362.1	PITPNC1	mRNA	XR_001436817.1	lncRNA
XM_015094531.1	ANAPC1	mRNA	XR_001435726.1	lncRNA
XM_015094531.1	ANAPC1	mRNA	XR_001435726.1	lncRNA
XM_015094531.1	ANAPC1	mRNA	XR_001435726.1	lncRNA
XM_015094531.1	ANAPC1	mRNA	XR_001435726.1	lncRNA
XM_015094531.1	ANAPC1	mRNA	XR_001435726.1	lncRNA
XM_015094531.1	ANAPC1	mRNA	XR_001435726.1	lncRNA
XM_015094531.1	ANAPC1	mRNA	XR_001435726.1	lncRNA
XM_015094531.1	ANAPC1	mRNA	XR_001435726.1	lncRNA
XM_015094531.1	ANAPC1	mRNA	XR_001435726.1	lncRNA
XM_015094531.1	ANAPC1	mRNA	XR_001435726.1	lncRNA
XM_015094531.1	ANAPC1	mRNA	XR_001435726.1	lncRNA
XM_012137434.2	NEB	mRNA	XR_001436817.1	lncRNA
XM_012137434.2	NEB	mRNA	XR_001436817.1	lncRNA
XM_012137434.2	NEB	mRNA	XR_001436817.1	lncRNA
XM_012137434.2	NEB	mRNA	XR_001436817.1	lncRNA
XM_012137434.2	NEB	mRNA	XR_001436817.1	lncRNA
XM_012137434.2	NEB	mRNA	XR_001436817.1	lncRNA
XM_012137434.2	NEB	mRNA	XR_001436817.1	lncRNA
XM_012137434.2	NEB	mRNA	XR_001436817.1	lncRNA
XM_012137434.2	NEB	mRNA	XR_001436817.1	lncRNA
XM_012137434.2	NEB	mRNA	XR_001436817.1	lncRNA
XM_012137434.2	NEB	mRNA	XR_001436817.1	lncRNA
XM_015099548.1	PARD3	mRNA	XR_001433856.1	lncRNA
XM_015099548.1	PARD3	mRNA	XR_001433856.1	lncRNA
XM_015099548.1	PARD3	mRNA	XR_001433856.1	lncRNA
XM_015099548.1	PARD3	mRNA	XR_001433856.1	lncRNA
XM_015099548.1	PARD3	mRNA	XR_001433856.1	lncRNA
XM_015099548.1	PARD3	mRNA	XR_001433856.1	lncRNA

XM_015099548.1	PARD3	mRNA	XR_001433856.1	lncRNA
XM_015099548.1	PARD3	mRNA	XR_001433856.1	lncRNA
XM_015099548.1	PARD3	mRNA	XR_001433856.1	lncRNA
XM_015099548.1	PARD3	mRNA	XR_001433856.1	lncRNA
XM_015099548.1	PARD3	mRNA	XR_001433856.1	lncRNA
XM_015094531.1	ANAPC1	mRNA	XR_001433856.1	lncRNA
XM_015094531.1	ANAPC1	mRNA	XR_001433856.1	lncRNA
XM_015094531.1	ANAPC1	mRNA	XR_001433856.1	lncRNA
XM_015094531.1	ANAPC1	mRNA	XR_001433856.1	lncRNA
XM_015094531.1	ANAPC1	mRNA	XR_001433856.1	lncRNA
XM_015094531.1	ANAPC1	mRNA	XR_001433856.1	lncRNA
XM_015094531.1	ANAPC1	mRNA	XR_001433856.1	lncRNA
XM_015094531.1	ANAPC1	mRNA	XR_001433856.1	lncRNA
XM_015094531.1	ANAPC1	mRNA	XR_001433856.1	lncRNA
XM_015094531.1	ANAPC1	mRNA	XR_001433856.1	lncRNA
XM_015094531.1	ANAPC1	mRNA	XR_001433856.1	lncRNA
XM_015094531.1	ANAPC1	mRNA	XR_001436817.1	lncRNA
XM_015094531.1	ANAPC1	mRNA	XR_001436817.1	lncRNA
XM_015094531.1	ANAPC1	mRNA	XR_001436817.1	lncRNA
XM_015094531.1	ANAPC1	mRNA	XR_001436817.1	lncRNA
XM_015094531.1	ANAPC1	mRNA	XR_001436817.1	lncRNA
XM_015094531.1	ANAPC1	mRNA	XR_001436817.1	lncRNA
XM_015094531.1	ANAPC1	mRNA	XR_001436817.1	lncRNA
XM_015094531.1	ANAPC1	mRNA	XR_001436817.1	lncRNA
XM_015094531.1	ANAPC1	mRNA	XR_001436817.1	lncRNA
XM_015094531.1	ANAPC1	mRNA	XR_001436817.1	lncRNA
XM_015094531.1	ANAPC1	mRNA	XR_001436817.1	lncRNA
XM_015094531.1	ANAPC1	mRNA	XR_001435722.1	lncRNA
XM_015094531.1	ANAPC1	mRNA	XR_001435722.1	lncRNA
XM_015094531.1	ANAPC1	mRNA	XR_001435722.1	lncRNA
XM_015094531.1	ANAPC1	mRNA	XR_001435722.1	lncRNA
XM_015094531.1	ANAPC1	mRNA	XR_001435722.1	lncRNA
XM_015094531.1	ANAPC1	mRNA	XR_001435722.1	lncRNA
XM_015094531.1	ANAPC1	mRNA	XR_001435722.1	lncRNA
XM_015094531.1	ANAPC1	mRNA	XR_001435722.1	lncRNA
XM_015094531.1	ANAPC1	mRNA	XR_001435722.1	lncRNA
XM_015094531.1	ANAPC1	mRNA	XR_001435722.1	lncRNA
XM_015094531.1	ANAPC1	mRNA	XR_001435722.1	lncRNA
XM_015094531.1	ANAPC1	mRNA	XR_001434920.1	lncRNA
XM_015094531.1	ANAPC1	mRNA	XR_001434920.1	lncRNA
XM_015094531.1	ANAPC1	mRNA	XR_001434920.1	lncRNA
XM_015094531.1	ANAPC1	mRNA	XR_001434920.1	lncRNA
XM_015094531.1	ANAPC1	mRNA	XR_001434920.1	lncRNA
XM_015094531.1	ANAPC1	mRNA	XR_001434920.1	lncRNA
XM_015094531.1	ANAPC1	mRNA	XR_001434920.1	lncRNA
XM_015094531.1	ANAPC1	mRNA	XR_001434920.1	lncRNA
XM_015094531.1	ANAPC1	mRNA	XR_001434920.1	lncRNA
XM_015094531.1	ANAPC1	mRNA	XR_001434920.1	lncRNA
XM_015094531.1	ANAPC1	mRNA	XR_001434920.1	lncRNA
XM_012186362.1	PITPNC1	mRNA	XR_001435726.1	lncRNA
XM_012186362.1	PITPNC1	mRNA	XR_001435726.1	lncRNA
XM_012186362.1	PITPNC1	mRNA	XR_001435726.1	lncRNA
XM_012186362.1	PITPNC1	mRNA	XR_001435726.1	lncRNA
XM_012186362.1	PITPNC1	mRNA	XR_001435726.1	lncRNA

XM_012186362.1	PITPNC1	mRNA	XR_001435726.1	lncRNA
XM_012186362.1	PITPNC1	mRNA	XR_001435726.1	lncRNA
XM_012186362.1	PITPNC1	mRNA	XR_001435726.1	lncRNA
XM_012186362.1	PITPNC1	mRNA	XR_001435726.1	lncRNA
XM_012186362.1	PITPNC1	mRNA	XR_001435726.1	lncRNA
XM_012186362.1	PITPNC1	mRNA	XR_001435726.1	lncRNA
XM_012186362.1	PITPNC1	mRNA	XR_001024230.1	lncRNA
XM_012186362.1	PITPNC1	mRNA	XR_001024230.1	lncRNA
XM_012186362.1	PITPNC1	mRNA	XR_001024230.1	lncRNA
XM_012186362.1	PITPNC1	mRNA	XR_001024230.1	lncRNA
XM_012186362.1	PITPNC1	mRNA	XR_001024230.1	lncRNA
XM_012186362.1	PITPNC1	mRNA	XR_001024230.1	lncRNA
XM_012186362.1	PITPNC1	mRNA	XR_001024230.1	lncRNA
XM_012186362.1	PITPNC1	mRNA	XR_001024230.1	lncRNA
XM_012186362.1	PITPNC1	mRNA	XR_001024230.1	lncRNA
XM_012186362.1	PITPNC1	mRNA	XR_001024230.1	lncRNA
XM_012186362.1	PITPNC1	mRNA	XR_001024230.1	lncRNA
XM_012186362.1	PITPNC1	mRNA	XR_001435723.1	lncRNA
XM_012186362.1	PITPNC1	mRNA	XR_001435723.1	lncRNA
XM_012186362.1	PITPNC1	mRNA	XR_001435723.1	lncRNA
XM_012186362.1	PITPNC1	mRNA	XR_001435723.1	lncRNA
XM_012186362.1	PITPNC1	mRNA	XR_001435723.1	lncRNA
XM_012186362.1	PITPNC1	mRNA	XR_001435723.1	lncRNA
XM_012186362.1	PITPNC1	mRNA	XR_001435723.1	lncRNA
XM_012186362.1	PITPNC1	mRNA	XR_001435723.1	lncRNA
XM_012186362.1	PITPNC1	mRNA	XR_001435723.1	lncRNA
XM_012186362.1	PITPNC1	mRNA	XR_001435723.1	lncRNA
XM_012186362.1	PITPNC1	mRNA	XR_001435723.1	lncRNA
XM_015094531.1	ANAPC1	mRNA	XR_001436390.1	lncRNA
XM_015094531.1	ANAPC1	mRNA	XR_001436390.1	lncRNA
XM_015094531.1	ANAPC1	mRNA	XR_001436390.1	lncRNA
XM_015094531.1	ANAPC1	mRNA	XR_001436390.1	lncRNA
XM_015094531.1	ANAPC1	mRNA	XR_001436390.1	lncRNA
XM_015094531.1	ANAPC1	mRNA	XR_001436390.1	lncRNA
XM_015094531.1	ANAPC1	mRNA	XR_001436390.1	lncRNA
XM_015094531.1	ANAPC1	mRNA	XR_001436390.1	lncRNA
XM_015094531.1	ANAPC1	mRNA	XR_001436390.1	lncRNA
XM_015094531.1	ANAPC1	mRNA	XR_001436390.1	lncRNA
XM_015099548.1	PARD3	mRNA	XR_001436390.1	lncRNA
XM_015099548.1	PARD3	mRNA	XR_001436390.1	lncRNA
XM_015099548.1	PARD3	mRNA	XR_001436390.1	lncRNA
XM_015099548.1	PARD3	mRNA	XR_001436390.1	lncRNA
XM_015099548.1	PARD3	mRNA	XR_001436390.1	lncRNA
XM_015099548.1	PARD3	mRNA	XR_001436390.1	lncRNA
XM_015099548.1	PARD3	mRNA	XR_001436390.1	lncRNA
XM_015099548.1	PARD3	mRNA	XR_001436390.1	lncRNA
XM_015099548.1	PARD3	mRNA	XR_001436390.1	lncRNA
XM_015099548.1	PARD3	mRNA	XR_001436390.1	lncRNA
XM_012179036.1	FER	mRNA	XR_001436064.1	lncRNA
XM_012179036.1	FER	mRNA	XR_001436064.1	lncRNA
XM_012179036.1	FER	mRNA	XR_001436064.1	lncRNA
XM_012179036.1	FER	mRNA	XR_001436064.1	lncRNA
XM_012179036.1	FER	mRNA	XR_001436064.1	lncRNA
XM_012179036.1	FER	mRNA	XR_001436064.1	lncRNA

XM_012179036.1	FER	mRNA	XR_001436064.1	lncRNA
XM_012179036.1	FER	mRNA	XR_001436064.1	lncRNA
XM_012179036.1	FER	mRNA	XR_001436064.1	lncRNA
XM_012179036.1	FER	mRNA	XR_001436064.1	lncRNA
XM_015101341.1	OTULIN	mRNA	XR_001436064.1	lncRNA
XM_015101341.1	OTULIN	mRNA	XR_001436064.1	lncRNA
XM_015101341.1	OTULIN	mRNA	XR_001436064.1	lncRNA
XM_015101341.1	OTULIN	mRNA	XR_001436064.1	lncRNA
XM_015101341.1	OTULIN	mRNA	XR_001436064.1	lncRNA
XM_015101341.1	OTULIN	mRNA	XR_001436064.1	lncRNA
XM_015101341.1	OTULIN	mRNA	XR_001436064.1	lncRNA
XM_015101341.1	OTULIN	mRNA	XR_001436064.1	lncRNA
XM_015101341.1	OTULIN	mRNA	XR_001436064.1	lncRNA
XM_015101341.1	OTULIN	mRNA	XR_001436064.1	lncRNA
XM_015103687.1	PTPN2	mRNA	XR_001024230.1	lncRNA
XM_015103687.1	PTPN2	mRNA	XR_001024230.1	lncRNA
XM_015103687.1	PTPN2	mRNA	XR_001024230.1	lncRNA
XM_015103687.1	PTPN2	mRNA	XR_001024230.1	lncRNA
XM_015103687.1	PTPN2	mRNA	XR_001024230.1	lncRNA
XM_015103687.1	PTPN2	mRNA	XR_001024230.1	lncRNA
XM_015103687.1	PTPN2	mRNA	XR_001024230.1	lncRNA
XM_015103687.1	PTPN2	mRNA	XR_001024230.1	lncRNA
XM_015103687.1	PTPN2	mRNA	XR_001024230.1	lncRNA
XM_015103687.1	PTPN2	mRNA	XR_001024230.1	lncRNA
XM_015101341.1	OTULIN	mRNA	XR_001024230.1	lncRNA
XM_015101341.1	OTULIN	mRNA	XR_001024230.1	lncRNA
XM_015101341.1	OTULIN	mRNA	XR_001024230.1	lncRNA
XM_015101341.1	OTULIN	mRNA	XR_001024230.1	lncRNA
XM_015101341.1	OTULIN	mRNA	XR_001024230.1	lncRNA
XM_015101341.1	OTULIN	mRNA	XR_001024230.1	lncRNA
XM_015101341.1	OTULIN	mRNA	XR_001024230.1	lncRNA
XM_015101341.1	OTULIN	mRNA	XR_001024230.1	lncRNA
XM_015101341.1	OTULIN	mRNA	XR_001024230.1	lncRNA
XM_015101341.1	OTULIN	mRNA	XR_001024230.1	lncRNA
XM_015099739.1	LOC101122805	mRNA	XR_001435726.1	lncRNA
XM_015099739.1	LOC101122805	mRNA	XR_001435726.1	lncRNA
XM_015099739.1	LOC101122805	mRNA	XR_001435726.1	lncRNA
XM_015099739.1	LOC101122805	mRNA	XR_001435726.1	lncRNA
XM_015099739.1	LOC101122805	mRNA	XR_001435726.1	lncRNA
XM_015099739.1	LOC101122805	mRNA	XR_001435726.1	lncRNA
XM_015099739.1	LOC101122805	mRNA	XR_001435726.1	lncRNA
XM_015099739.1	LOC101122805	mRNA	XR_001435726.1	lncRNA
XM_015099739.1	LOC101122805	mRNA	XR_001435726.1	lncRNA
XM_015099739.1	LOC101122805	mRNA	XR_001435726.1	lncRNA
XM_015099563.1	PARD3	mRNA	XR_001436064.1	lncRNA
XM_015099563.1	PARD3	mRNA	XR_001436064.1	lncRNA
XM_015099563.1	PARD3	mRNA	XR_001436064.1	lncRNA
XM_015099563.1	PARD3	mRNA	XR_001436064.1	lncRNA
XM_015099563.1	PARD3	mRNA	XR_001436064.1	lncRNA
XM_015099563.1	PARD3	mRNA	XR_001436064.1	lncRNA
XM_015099563.1	PARD3	mRNA	XR_001436064.1	lncRNA
XM_015099563.1	PARD3	mRNA	XR_001436064.1	lncRNA
XM_015099563.1	PARD3	mRNA	XR_001436064.1	lncRNA
XM_015099563.1	PARD3	mRNA	XR_001436064.1	lncRNA

XM_015101341.1	OTULIN	mRNA	XR_001435726.1	lncRNA
XM_015101341.1	OTULIN	mRNA	XR_001435726.1	lncRNA
XM_015101341.1	OTULIN	mRNA	XR_001435726.1	lncRNA
XM_015101341.1	OTULIN	mRNA	XR_001435726.1	lncRNA
XM_015101341.1	OTULIN	mRNA	XR_001435726.1	lncRNA
XM_015101341.1	OTULIN	mRNA	XR_001435726.1	lncRNA
XM_015101341.1	OTULIN	mRNA	XR_001435726.1	lncRNA
XM_015101341.1	OTULIN	mRNA	XR_001435726.1	lncRNA
XM_015101341.1	OTULIN	mRNA	XR_001435726.1	lncRNA
XM_015101341.1	OTULIN	mRNA	XR_001435726.1	lncRNA
XM_012096323.1	PHF21A	mRNA	XR_001435726.1	lncRNA
XM_012096323.1	PHF21A	mRNA	XR_001435726.1	lncRNA
XM_012096323.1	PHF21A	mRNA	XR_001435726.1	lncRNA
XM_012096323.1	PHF21A	mRNA	XR_001435726.1	lncRNA
XM_012096323.1	PHF21A	mRNA	XR_001435726.1	lncRNA
XM_012096323.1	PHF21A	mRNA	XR_001435726.1	lncRNA
XM_012096323.1	PHF21A	mRNA	XR_001435726.1	lncRNA
XM_012096323.1	PHF21A	mRNA	XR_001435726.1	lncRNA
XM_012096323.1	PHF21A	mRNA	XR_001435726.1	lncRNA
XM_012096323.1	PHF21A	mRNA	XR_001435726.1	lncRNA
XM_015101341.1	OTULIN	mRNA	XR_001436065.1	lncRNA
XM_015101341.1	OTULIN	mRNA	XR_001436065.1	lncRNA
XM_015101341.1	OTULIN	mRNA	XR_001436065.1	lncRNA
XM_015101341.1	OTULIN	mRNA	XR_001436065.1	lncRNA
XM_015101341.1	OTULIN	mRNA	XR_001436065.1	lncRNA
XM_015101341.1	OTULIN	mRNA	XR_001436065.1	lncRNA
XM_015101341.1	OTULIN	mRNA	XR_001436065.1	lncRNA
XM_015101341.1	OTULIN	mRNA	XR_001436065.1	lncRNA
XM_015101341.1	OTULIN	mRNA	XR_001436065.1	lncRNA
XM_015101341.1	OTULIN	mRNA	XR_001436065.1	lncRNA
XM_015099563.1	PARD3	mRNA	XR_001436065.1	lncRNA
XM_015099563.1	PARD3	mRNA	XR_001436065.1	lncRNA
XM_015099563.1	PARD3	mRNA	XR_001436065.1	lncRNA
XM_015099563.1	PARD3	mRNA	XR_001436065.1	lncRNA
XM_015099563.1	PARD3	mRNA	XR_001436065.1	lncRNA
XM_015099563.1	PARD3	mRNA	XR_001436065.1	lncRNA
XM_015099563.1	PARD3	mRNA	XR_001436065.1	lncRNA
XM_015099563.1	PARD3	mRNA	XR_001436065.1	lncRNA
XM_015099563.1	PARD3	mRNA	XR_001436065.1	lncRNA
XM_015099563.1	PARD3	mRNA	XR_001436065.1	lncRNA
XM_012105324.2	USP54	mRNA	XR_001024230.1	lncRNA
XM_012105324.2	USP54	mRNA	XR_001024230.1	lncRNA
XM_012105324.2	USP54	mRNA	XR_001024230.1	lncRNA
XM_012105324.2	USP54	mRNA	XR_001024230.1	lncRNA
XM_012105324.2	USP54	mRNA	XR_001024230.1	lncRNA
XM_012105324.2	USP54	mRNA	XR_001024230.1	lncRNA
XM_012105324.2	USP54	mRNA	XR_001024230.1	lncRNA
XM_012105324.2	USP54	mRNA	XR_001024230.1	lncRNA
XM_012105324.2	USP54	mRNA	XR_001024230.1	lncRNA
XM_012105324.2	USP54	mRNA	XR_001024230.1	lncRNA
XM_015099739.1	LOC101122805	mRNA	XR_001435723.1	lncRNA
XM_015099739.1	LOC101122805	mRNA	XR_001435723.1	lncRNA
XM_015099739.1	LOC101122805	mRNA	XR_001435723.1	lncRNA
XM_015099739.1	LOC101122805	mRNA	XR_001435723.1	lncRNA

XM_015099739.1	LOC101122805	mRNA	XR_001435723.1	lncRNA
XM_015099739.1	LOC101122805	mRNA	XR_001435723.1	lncRNA
XM_015099739.1	LOC101122805	mRNA	XR_001435723.1	lncRNA
XM_015099739.1	LOC101122805	mRNA	XR_001435723.1	lncRNA
XM_015099739.1	LOC101122805	mRNA	XR_001435723.1	lncRNA
XM_015099739.1	LOC101122805	mRNA	XR_001435723.1	lncRNA
XM_015099563.1	PARD3	mRNA	XR_001435723.1	lncRNA
XM_015099563.1	PARD3	mRNA	XR_001435723.1	lncRNA
XM_015099563.1	PARD3	mRNA	XR_001435723.1	lncRNA
XM_015099563.1	PARD3	mRNA	XR_001435723.1	lncRNA
XM_015099563.1	PARD3	mRNA	XR_001435723.1	lncRNA
XM_015099563.1	PARD3	mRNA	XR_001435723.1	lncRNA
XM_015099563.1	PARD3	mRNA	XR_001435723.1	lncRNA
XM_015099563.1	PARD3	mRNA	XR_001435723.1	lncRNA
XM_015099563.1	PARD3	mRNA	XR_001435723.1	lncRNA
XM_015099563.1	PARD3	mRNA	XR_001435723.1	lncRNA
XM_015101341.1	OTULIN	mRNA	XR_001435723.1	lncRNA
XM_015101341.1	OTULIN	mRNA	XR_001435723.1	lncRNA
XM_015101341.1	OTULIN	mRNA	XR_001435723.1	lncRNA
XM_015101341.1	OTULIN	mRNA	XR_001435723.1	lncRNA
XM_015101341.1	OTULIN	mRNA	XR_001435723.1	lncRNA
XM_015101341.1	OTULIN	mRNA	XR_001435723.1	lncRNA
XM_015101341.1	OTULIN	mRNA	XR_001435723.1	lncRNA
XM_015101341.1	OTULIN	mRNA	XR_001435723.1	lncRNA
XM_015101341.1	OTULIN	mRNA	XR_001435723.1	lncRNA
XM_015101341.1	OTULIN	mRNA	XR_001435723.1	lncRNA
XM_012096323.1	PHF21A	mRNA	XR_001436817.1	lncRNA
XM_012096323.1	PHF21A	mRNA	XR_001436817.1	lncRNA
XM_012096323.1	PHF21A	mRNA	XR_001436817.1	lncRNA
XM_012096323.1	PHF21A	mRNA	XR_001436817.1	lncRNA
XM_012096323.1	PHF21A	mRNA	XR_001436817.1	lncRNA
XM_012096323.1	PHF21A	mRNA	XR_001436817.1	lncRNA
XM_012096323.1	PHF21A	mRNA	XR_001436817.1	lncRNA
XM_012096323.1	PHF21A	mRNA	XR_001436817.1	lncRNA
XM_012096323.1	PHF21A	mRNA	XR_001436817.1	lncRNA
XM_012096323.1	PHF21A	mRNA	XR_001436817.1	lncRNA
XM_012179036.1	FER	mRNA	XR_001435722.1	lncRNA
XM_012179036.1	FER	mRNA	XR_001435722.1	lncRNA
XM_012179036.1	FER	mRNA	XR_001435722.1	lncRNA
XM_012179036.1	FER	mRNA	XR_001435722.1	lncRNA
XM_012179036.1	FER	mRNA	XR_001435722.1	lncRNA
XM_012179036.1	FER	mRNA	XR_001435722.1	lncRNA
XM_012179036.1	FER	mRNA	XR_001435722.1	lncRNA
XM_012179036.1	FER	mRNA	XR_001435722.1	lncRNA
XM_012179036.1	FER	mRNA	XR_001435722.1	lncRNA
XM_012179036.1	FER	mRNA	XR_001435722.1	lncRNA
XM_015099739.1	LOC101122805	mRNA	XR_001435107.1	lncRNA
XM_015099739.1	LOC101122805	mRNA	XR_001435107.1	lncRNA
XM_015099739.1	LOC101122805	mRNA	XR_001435107.1	lncRNA
XM_015099739.1	LOC101122805	mRNA	XR_001435107.1	lncRNA
XM_015099739.1	LOC101122805	mRNA	XR_001435107.1	lncRNA
XM_015099739.1	LOC101122805	mRNA	XR_001435107.1	lncRNA
XM_015099739.1	LOC101122805	mRNA	XR_001435107.1	lncRNA
XM_015099739.1	LOC101122805	mRNA	XR_001435107.1	lncRNA

XM_015099739.1	LOC101122805	mRNA	XR_001435107.1	lncRNA
XM_015099739.1	LOC101122805	mRNA	XR_001435107.1	lncRNA
XM_015103687.1	PTPN2	mRNA	XR_001436817.1	lncRNA
XM_015103687.1	PTPN2	mRNA	XR_001436817.1	lncRNA
XM_015103687.1	PTPN2	mRNA	XR_001436817.1	lncRNA
XM_015103687.1	PTPN2	mRNA	XR_001436817.1	lncRNA
XM_015103687.1	PTPN2	mRNA	XR_001436817.1	lncRNA
XM_015103687.1	PTPN2	mRNA	XR_001436817.1	lncRNA
XM_015103687.1	PTPN2	mRNA	XR_001436817.1	lncRNA
XM_015103687.1	PTPN2	mRNA	XR_001436817.1	lncRNA
XM_015103687.1	PTPN2	mRNA	XR_001436817.1	lncRNA
XM_015103687.1	PTPN2	mRNA	XR_001436817.1	lncRNA
XM_015099739.1	LOC101122805	mRNA	XR_001435722.1	lncRNA
XM_015099739.1	LOC101122805	mRNA	XR_001435722.1	lncRNA
XM_015099739.1	LOC101122805	mRNA	XR_001435722.1	lncRNA
XM_015099739.1	LOC101122805	mRNA	XR_001435722.1	lncRNA
XM_015099739.1	LOC101122805	mRNA	XR_001435722.1	lncRNA
XM_015099739.1	LOC101122805	mRNA	XR_001435722.1	lncRNA
XM_015099739.1	LOC101122805	mRNA	XR_001435722.1	lncRNA
XM_015099739.1	LOC101122805	mRNA	XR_001435722.1	lncRNA
XM_015099739.1	LOC101122805	mRNA	XR_001435722.1	lncRNA
XM_015099739.1	LOC101122805	mRNA	XR_001435722.1	lncRNA
XM_012095733.1	BTBD10	mRNA	XR_001435722.1	lncRNA
XM_012095733.1	BTBD10	mRNA	XR_001435722.1	lncRNA
XM_012095733.1	BTBD10	mRNA	XR_001435722.1	lncRNA
XM_012095733.1	BTBD10	mRNA	XR_001435722.1	lncRNA
XM_012095733.1	BTBD10	mRNA	XR_001435722.1	lncRNA
XM_012095733.1	BTBD10	mRNA	XR_001435722.1	lncRNA
XM_012095733.1	BTBD10	mRNA	XR_001435722.1	lncRNA
XM_012095733.1	BTBD10	mRNA	XR_001435722.1	lncRNA
XM_012095733.1	BTBD10	mRNA	XR_001435722.1	lncRNA
XM_012095733.1	BTBD10	mRNA	XR_001435722.1	lncRNA
XM_012182192.2	EML5	mRNA	XR_001436817.1	lncRNA
XM_012182192.2	EML5	mRNA	XR_001436817.1	lncRNA
XM_012182192.2	EML5	mRNA	XR_001436817.1	lncRNA
XM_012182192.2	EML5	mRNA	XR_001436817.1	lncRNA
XM_012182192.2	EML5	mRNA	XR_001436817.1	lncRNA
XM_012182192.2	EML5	mRNA	XR_001436817.1	lncRNA
XM_012182192.2	EML5	mRNA	XR_001436817.1	lncRNA
XM_012182192.2	EML5	mRNA	XR_001436817.1	lncRNA
XM_012182192.2	EML5	mRNA	XR_001436817.1	lncRNA
XM_012182192.2	EML5	mRNA	XR_001436817.1	lncRNA
XM_012182192.2	EML5	mRNA	XR_001434920.1	lncRNA
XM_012182192.2	EML5	mRNA	XR_001434920.1	lncRNA
XM_012182192.2	EML5	mRNA	XR_001434920.1	lncRNA
XM_012182192.2	EML5	mRNA	XR_001434920.1	lncRNA
XM_012182192.2	EML5	mRNA	XR_001434920.1	lncRNA
XM_012182192.2	EML5	mRNA	XR_001434920.1	lncRNA
XM_012182192.2	EML5	mRNA	XR_001434920.1	lncRNA
XM_012182192.2	EML5	mRNA	XR_001434920.1	lncRNA
XM_012182192.2	EML5	mRNA	XR_001434920.1	lncRNA
XM_012182192.2	EML5	mRNA	XR_001434920.1	lncRNA
XM_012137434.2	NEB	mRNA	XR_001435726.1	lncRNA
XM_012137434.2	NEB	mRNA	XR_001435726.1	lncRNA

XM_012137434.2	NEB	mRNA	XR_001435726.1	lncRNA
XM_012137434.2	NEB	mRNA	XR_001435726.1	lncRNA
XM_012137434.2	NEB	mRNA	XR_001435726.1	lncRNA
XM_012137434.2	NEB	mRNA	XR_001435726.1	lncRNA
XM_012137434.2	NEB	mRNA	XR_001435726.1	lncRNA
XM_012137434.2	NEB	mRNA	XR_001435726.1	lncRNA
XM_012137434.2	NEB	mRNA	XR_001435726.1	lncRNA
XM_012137434.2	NEB	mRNA	XR_001435726.1	lncRNA
XM_012182192.2	EML5	mRNA	XR_001436390.1	lncRNA
XM_012182192.2	EML5	mRNA	XR_001436390.1	lncRNA
XM_012182192.2	EML5	mRNA	XR_001436390.1	lncRNA
XM_012182192.2	EML5	mRNA	XR_001436390.1	lncRNA
XM_012182192.2	EML5	mRNA	XR_001436390.1	lncRNA
XM_012182192.2	EML5	mRNA	XR_001436390.1	lncRNA
XM_012182192.2	EML5	mRNA	XR_001436390.1	lncRNA
XM_012182192.2	EML5	mRNA	XR_001436390.1	lncRNA
XM_012182192.2	EML5	mRNA	XR_001436390.1	lncRNA
XM_012143533.1	INO80D	mRNA	TCONS_00049802	lncRNA
XM_012143533.1	INO80D	mRNA	TCONS_00049802	lncRNA
XM_012143533.1	INO80D	mRNA	TCONS_00049802	lncRNA
XM_012143533.1	INO80D	mRNA	TCONS_00049802	lncRNA
XM_012143533.1	INO80D	mRNA	TCONS_00049802	lncRNA
XM_012143533.1	INO80D	mRNA	TCONS_00049802	lncRNA
XM_012143533.1	INO80D	mRNA	TCONS_00049802	lncRNA
XM_012143533.1	INO80D	mRNA	TCONS_00049802	lncRNA
XM_012143533.1	INO80D	mRNA	TCONS_00049802	lncRNA
XM_015094225.1	ROCK2	mRNA	XR_001024230.1	lncRNA
XM_015094225.1	ROCK2	mRNA	XR_001024230.1	lncRNA
XM_015094225.1	ROCK2	mRNA	XR_001024230.1	lncRNA
XM_015094225.1	ROCK2	mRNA	XR_001024230.1	lncRNA
XM_015094225.1	ROCK2	mRNA	XR_001024230.1	lncRNA
XM_015094225.1	ROCK2	mRNA	XR_001024230.1	lncRNA
XM_015094225.1	ROCK2	mRNA	XR_001024230.1	lncRNA
XM_015094225.1	ROCK2	mRNA	XR_001024230.1	lncRNA
XM_015094225.1	ROCK2	mRNA	XR_001024230.1	lncRNA
XM_015092364.1	GART	mRNA	XR_001024230.1	lncRNA
XM_015092364.1	GART	mRNA	XR_001024230.1	lncRNA
XM_015092364.1	GART	mRNA	XR_001024230.1	lncRNA
XM_015092364.1	GART	mRNA	XR_001024230.1	lncRNA
XM_015092364.1	GART	mRNA	XR_001024230.1	lncRNA
XM_015092364.1	GART	mRNA	XR_001024230.1	lncRNA
XM_015092364.1	GART	mRNA	XR_001024230.1	lncRNA
XM_015092364.1	GART	mRNA	XR_001024230.1	lncRNA
XM_015092364.1	GART	mRNA	XR_001024230.1	lncRNA
XM_012184362.2	AKAP11	mRNA	XR_001024230.1	lncRNA
XM_012184362.2	AKAP11	mRNA	XR_001024230.1	lncRNA
XM_012184362.2	AKAP11	mRNA	XR_001024230.1	lncRNA
XM_012184362.2	AKAP11	mRNA	XR_001024230.1	lncRNA
XM_012184362.2	AKAP11	mRNA	XR_001024230.1	lncRNA
XM_012184362.2	AKAP11	mRNA	XR_001024230.1	lncRNA
XM_012184362.2	AKAP11	mRNA	XR_001024230.1	lncRNA
XM_012184362.2	AKAP11	mRNA	XR_001024230.1	lncRNA
XM_012184362.2	AKAP11	mRNA	XR_001024230.1	lncRNA
XM_012179825.2	N4BP2	mRNA	XR_001024230.1	lncRNA

XM_012179825.2	N4BP2	mRNA	XR_001024230.1	lncRNA
XM_012179825.2	N4BP2	mRNA	XR_001024230.1	lncRNA
XM_012179825.2	N4BP2	mRNA	XR_001024230.1	lncRNA
XM_012179825.2	N4BP2	mRNA	XR_001024230.1	lncRNA
XM_012179825.2	N4BP2	mRNA	XR_001024230.1	lncRNA
XM_012179825.2	N4BP2	mRNA	XR_001024230.1	lncRNA
XM_012179825.2	N4BP2	mRNA	XR_001024230.1	lncRNA
XM_012179825.2	N4BP2	mRNA	XR_001024230.1	lncRNA
XM_012105333.2	USP54	mRNA	XR_001435726.1	lncRNA
XM_012105333.2	USP54	mRNA	XR_001435726.1	lncRNA
XM_012105333.2	USP54	mRNA	XR_001435726.1	lncRNA
XM_012105333.2	USP54	mRNA	XR_001435726.1	lncRNA
XM_012105333.2	USP54	mRNA	XR_001435726.1	lncRNA
XM_012105333.2	USP54	mRNA	XR_001435726.1	lncRNA
XM_012105333.2	USP54	mRNA	XR_001435726.1	lncRNA
XM_012105333.2	USP54	mRNA	XR_001435726.1	lncRNA
XM_012105333.2	USP54	mRNA	XR_001435726.1	lncRNA
XM_015105074.1	LONRF3	mRNA	XR_001024230.1	lncRNA
XM_015105074.1	LONRF3	mRNA	XR_001024230.1	lncRNA
XM_015105074.1	LONRF3	mRNA	XR_001024230.1	lncRNA
XM_015105074.1	LONRF3	mRNA	XR_001024230.1	lncRNA
XM_015105074.1	LONRF3	mRNA	XR_001024230.1	lncRNA
XM_015105074.1	LONRF3	mRNA	XR_001024230.1	lncRNA
XM_015105074.1	LONRF3	mRNA	XR_001024230.1	lncRNA
XM_015105074.1	LONRF3	mRNA	XR_001024230.1	lncRNA
XM_015105074.1	LONRF3	mRNA	XR_001024230.1	lncRNA
XM_015093065.1	FAM219A	mRNA	XR_001024230.1	lncRNA
XM_015093065.1	FAM219A	mRNA	XR_001024230.1	lncRNA
XM_015093065.1	FAM219A	mRNA	XR_001024230.1	lncRNA
XM_015093065.1	FAM219A	mRNA	XR_001024230.1	lncRNA
XM_015093065.1	FAM219A	mRNA	XR_001024230.1	lncRNA
XM_015093065.1	FAM219A	mRNA	XR_001024230.1	lncRNA
XM_015093065.1	FAM219A	mRNA	XR_001024230.1	lncRNA
XM_015093065.1	FAM219A	mRNA	XR_001024230.1	lncRNA
XM_015093065.1	FAM219A	mRNA	XR_001024230.1	lncRNA
XM_015094225.1	ROCK2	mRNA	XR_001436065.1	lncRNA
XM_015094225.1	ROCK2	mRNA	XR_001436065.1	lncRNA
XM_015094225.1	ROCK2	mRNA	XR_001436065.1	lncRNA
XM_015094225.1	ROCK2	mRNA	XR_001436065.1	lncRNA
XM_015094225.1	ROCK2	mRNA	XR_001436065.1	lncRNA
XM_015094225.1	ROCK2	mRNA	XR_001436065.1	lncRNA
XM_015094225.1	ROCK2	mRNA	XR_001436065.1	lncRNA
XM_015094225.1	ROCK2	mRNA	XR_001436065.1	lncRNA
XM_015094225.1	ROCK2	mRNA	XR_001436065.1	lncRNA
XM_015093416.1	RIF1	mRNA	XR_001436065.1	lncRNA
XM_015093416.1	RIF1	mRNA	XR_001436065.1	lncRNA
XM_015093416.1	RIF1	mRNA	XR_001436065.1	lncRNA
XM_015093416.1	RIF1	mRNA	XR_001436065.1	lncRNA
XM_015093416.1	RIF1	mRNA	XR_001436065.1	lncRNA
XM_015093416.1	RIF1	mRNA	XR_001436065.1	lncRNA
XM_015093416.1	RIF1	mRNA	XR_001436065.1	lncRNA
XM_015093416.1	RIF1	mRNA	XR_001436065.1	lncRNA
XM_015093416.1	RIF1	mRNA	XR_001436065.1	lncRNA
XM_015093065.1	FAM219A	mRNA	XR_001435726.1	lncRNA

XM_015093065.1	FAM219A	mRNA	XR_001435726.1	lncRNA
XM_015093065.1	FAM219A	mRNA	XR_001435726.1	lncRNA
XM_015093065.1	FAM219A	mRNA	XR_001435726.1	lncRNA
XM_015093065.1	FAM219A	mRNA	XR_001435726.1	lncRNA
XM_015093065.1	FAM219A	mRNA	XR_001435726.1	lncRNA
XM_015093065.1	FAM219A	mRNA	XR_001435726.1	lncRNA
XM_015093065.1	FAM219A	mRNA	XR_001435726.1	lncRNA
XM_015093065.1	FAM219A	mRNA	XR_001435726.1	lncRNA
XM_012179676.2	CCSER1	mRNA	XR_001024230.1	lncRNA
XM_012179676.2	CCSER1	mRNA	XR_001024230.1	lncRNA
XM_012179676.2	CCSER1	mRNA	XR_001024230.1	lncRNA
XM_012179676.2	CCSER1	mRNA	XR_001024230.1	lncRNA
XM_012179676.2	CCSER1	mRNA	XR_001024230.1	lncRNA
XM_012179676.2	CCSER1	mRNA	XR_001024230.1	lncRNA
XM_012179676.2	CCSER1	mRNA	XR_001024230.1	lncRNA
XM_012179676.2	CCSER1	mRNA	XR_001024230.1	lncRNA
XM_012179676.2	CCSER1	mRNA	XR_001024230.1	lncRNA
XM_004009738.3	PPARGC1A	mRNA	XR_001024230.1	lncRNA
XM_004009738.3	PPARGC1A	mRNA	XR_001024230.1	lncRNA
XM_004009738.3	PPARGC1A	mRNA	XR_001024230.1	lncRNA
XM_004009738.3	PPARGC1A	mRNA	XR_001024230.1	lncRNA
XM_004009738.3	PPARGC1A	mRNA	XR_001024230.1	lncRNA
XM_004009738.3	PPARGC1A	mRNA	XR_001024230.1	lncRNA
XM_004009738.3	PPARGC1A	mRNA	XR_001024230.1	lncRNA
XM_004009738.3	PPARGC1A	mRNA	XR_001024230.1	lncRNA
XM_004009738.3	PPARGC1A	mRNA	XR_001024230.1	lncRNA
XM_015100523.1	NFAT5	mRNA	XR_001436065.1	lncRNA
XM_015100523.1	NFAT5	mRNA	XR_001436065.1	lncRNA
XM_015100523.1	NFAT5	mRNA	XR_001436065.1	lncRNA
XM_015100523.1	NFAT5	mRNA	XR_001436065.1	lncRNA
XM_015100523.1	NFAT5	mRNA	XR_001436065.1	lncRNA
XM_015100523.1	NFAT5	mRNA	XR_001436065.1	lncRNA
XM_015100523.1	NFAT5	mRNA	XR_001436065.1	lncRNA
XM_015100523.1	NFAT5	mRNA	XR_001436065.1	lncRNA
XM_015100523.1	NFAT5	mRNA	XR_001436065.1	lncRNA
XM_012097374.2	LRBA	mRNA	XR_001435726.1	lncRNA
XM_012097374.2	LRBA	mRNA	XR_001435726.1	lncRNA
XM_012097374.2	LRBA	mRNA	XR_001435726.1	lncRNA
XM_012097374.2	LRBA	mRNA	XR_001435726.1	lncRNA
XM_012097374.2	LRBA	mRNA	XR_001435726.1	lncRNA
XM_012097374.2	LRBA	mRNA	XR_001435726.1	lncRNA
XM_012097374.2	LRBA	mRNA	XR_001435726.1	lncRNA
XM_012097374.2	LRBA	mRNA	XR_001435726.1	lncRNA
XM_012097374.2	LRBA	mRNA	XR_001435726.1	lncRNA
XM_012097035.1	C16H5orf42	mRNA	XR_001024230.1	lncRNA
XM_012097035.1	C16H5orf42	mRNA	XR_001024230.1	lncRNA
XM_012097035.1	C16H5orf42	mRNA	XR_001024230.1	lncRNA
XM_012097035.1	C16H5orf42	mRNA	XR_001024230.1	lncRNA
XM_012097035.1	C16H5orf42	mRNA	XR_001024230.1	lncRNA
XM_012097035.1	C16H5orf42	mRNA	XR_001024230.1	lncRNA
XM_012097035.1	C16H5orf42	mRNA	XR_001024230.1	lncRNA
XM_012097035.1	C16H5orf42	mRNA	XR_001024230.1	lncRNA
XM_012097035.1	C16H5orf42	mRNA	XR_001024230.1	lncRNA
XM_015094225.1	ROCK2	mRNA	XR_001436064.1	lncRNA

XM_015094225.1	ROCK2	mRNA	XR_001436064.1	lncRNA
XM_015094225.1	ROCK2	mRNA	XR_001436064.1	lncRNA
XM_015094225.1	ROCK2	mRNA	XR_001436064.1	lncRNA
XM_015094225.1	ROCK2	mRNA	XR_001436064.1	lncRNA
XM_015094225.1	ROCK2	mRNA	XR_001436064.1	lncRNA
XM_015094225.1	ROCK2	mRNA	XR_001436064.1	lncRNA
XM_015094225.1	ROCK2	mRNA	XR_001436064.1	lncRNA
XM_015094225.1	ROCK2	mRNA	XR_001436064.1	lncRNA
XM_012097374.2	LRBA	mRNA	XR_001435723.1	lncRNA
XM_012097374.2	LRBA	mRNA	XR_001435723.1	lncRNA
XM_012097374.2	LRBA	mRNA	XR_001435723.1	lncRNA
XM_012097374.2	LRBA	mRNA	XR_001435723.1	lncRNA
XM_012097374.2	LRBA	mRNA	XR_001435723.1	lncRNA
XM_012097374.2	LRBA	mRNA	XR_001435723.1	lncRNA
XM_012097374.2	LRBA	mRNA	XR_001435723.1	lncRNA
XM_012097374.2	LRBA	mRNA	XR_001435723.1	lncRNA
XM_012097374.2	LRBA	mRNA	XR_001435723.1	lncRNA
XM_015093065.1	FAM219A	mRNA	XR_001435723.1	lncRNA
XM_015093065.1	FAM219A	mRNA	XR_001435723.1	lncRNA
XM_015093065.1	FAM219A	mRNA	XR_001435723.1	lncRNA
XM_015093065.1	FAM219A	mRNA	XR_001435723.1	lncRNA
XM_015093065.1	FAM219A	mRNA	XR_001435723.1	lncRNA
XM_015093065.1	FAM219A	mRNA	XR_001435723.1	lncRNA
XM_015093065.1	FAM219A	mRNA	XR_001435723.1	lncRNA
XM_015093065.1	FAM219A	mRNA	XR_001435723.1	lncRNA
XM_015093065.1	FAM219A	mRNA	XR_001435723.1	lncRNA
XM_012097035.1	C16H5orf42	mRNA	XR_001436064.1	lncRNA
XM_012097035.1	C16H5orf42	mRNA	XR_001436064.1	lncRNA
XM_012097035.1	C16H5orf42	mRNA	XR_001436064.1	lncRNA
XM_012097035.1	C16H5orf42	mRNA	XR_001436064.1	lncRNA
XM_012097035.1	C16H5orf42	mRNA	XR_001436064.1	lncRNA
XM_012097035.1	C16H5orf42	mRNA	XR_001436064.1	lncRNA
XM_012097035.1	C16H5orf42	mRNA	XR_001436064.1	lncRNA
XM_012097035.1	C16H5orf42	mRNA	XR_001436064.1	lncRNA
XM_012097035.1	C16H5orf42	mRNA	XR_001436064.1	lncRNA
XM_004017788.3	ZFANL3	mRNA	XR_001436612.1	lncRNA
XM_004017788.3	ZFANL3	mRNA	XR_001436612.1	lncRNA
XM_004017788.3	ZFANL3	mRNA	XR_001436612.1	lncRNA
XM_004017788.3	ZFANL3	mRNA	XR_001436612.1	lncRNA
XM_004017788.3	ZFANL3	mRNA	XR_001436612.1	lncRNA
XM_004017788.3	ZFANL3	mRNA	XR_001436612.1	lncRNA
XM_004017788.3	ZFANL3	mRNA	XR_001436612.1	lncRNA
XM_004017788.3	ZFANL3	mRNA	XR_001436612.1	lncRNA
XM_004009738.3	PPARGC1A	mRNA	XR_001436817.1	lncRNA
XM_004009738.3	PPARGC1A	mRNA	XR_001436817.1	lncRNA
XM_004009738.3	PPARGC1A	mRNA	XR_001436817.1	lncRNA
XM_004009738.3	PPARGC1A	mRNA	XR_001436817.1	lncRNA
XM_004009738.3	PPARGC1A	mRNA	XR_001436817.1	lncRNA
XM_004009738.3	PPARGC1A	mRNA	XR_001436817.1	lncRNA
XM_004009738.3	PPARGC1A	mRNA	XR_001436817.1	lncRNA
XM_004009738.3	PPARGC1A	mRNA	XR_001436817.1	lncRNA
XM_004009738.3	PPARGC1A	mRNA	XR_001436817.1	lncRNA
XM_012189777.1	CHD9	mRNA	XR_001433856.1	lncRNA
XM_012189777.1	CHD9	mRNA	XR_001433856.1	lncRNA

XM_012189777.1	CHD9	mRNA	XR_001433856.1	lncRNA
XM_012189777.1	CHD9	mRNA	XR_001433856.1	lncRNA
XM_012189777.1	CHD9	mRNA	XR_001433856.1	lncRNA
XM_012189777.1	CHD9	mRNA	XR_001433856.1	lncRNA
XM_012189777.1	CHD9	mRNA	XR_001433856.1	lncRNA
XM_012189777.1	CHD9	mRNA	XR_001433856.1	lncRNA
XM_012189777.1	CHD9	mRNA	XR_001433856.1	lncRNA
XM_004003021.3	DLG1	mRNA	XR_001434920.1	lncRNA
XM_004003021.3	DLG1	mRNA	XR_001434920.1	lncRNA
XM_004003021.3	DLG1	mRNA	XR_001434920.1	lncRNA
XM_004003021.3	DLG1	mRNA	XR_001434920.1	lncRNA
XM_004003021.3	DLG1	mRNA	XR_001434920.1	lncRNA
XM_004003021.3	DLG1	mRNA	XR_001434920.1	lncRNA
XM_004003021.3	DLG1	mRNA	XR_001434920.1	lncRNA
XM_004003021.3	DLG1	mRNA	XR_001434920.1	lncRNA
XM_004003021.3	DLG1	mRNA	XR_001434920.1	lncRNA
XM_004011768.3	SAMD12	mRNA	XR_001435722.1	lncRNA
XM_004011768.3	SAMD12	mRNA	XR_001435722.1	lncRNA
XM_004011768.3	SAMD12	mRNA	XR_001435722.1	lncRNA
XM_004011768.3	SAMD12	mRNA	XR_001435722.1	lncRNA
XM_004011768.3	SAMD12	mRNA	XR_001435722.1	lncRNA
XM_004011768.3	SAMD12	mRNA	XR_001435722.1	lncRNA
XM_004011768.3	SAMD12	mRNA	XR_001435722.1	lncRNA
XM_004011768.3	SAMD12	mRNA	XR_001435722.1	lncRNA
XM_004011768.3	SAMD12	mRNA	XR_001435722.1	lncRNA
XM_012174704.2	ZBTB39	mRNA	XR_001433856.1	lncRNA
XM_012174704.2	ZBTB39	mRNA	XR_001433856.1	lncRNA
XM_012174704.2	ZBTB39	mRNA	XR_001433856.1	lncRNA
XM_012174704.2	ZBTB39	mRNA	XR_001433856.1	lncRNA
XM_012174704.2	ZBTB39	mRNA	XR_001433856.1	lncRNA
XM_012174704.2	ZBTB39	mRNA	XR_001433856.1	lncRNA
XM_012174704.2	ZBTB39	mRNA	XR_001433856.1	lncRNA
XM_012174704.2	ZBTB39	mRNA	XR_001433856.1	lncRNA
XM_012174704.2	ZBTB39	mRNA	XR_001433856.1	lncRNA
XM_012105333.2	USP54	mRNA	XR_001434920.1	lncRNA
XM_012105333.2	USP54	mRNA	XR_001434920.1	lncRNA
XM_012105333.2	USP54	mRNA	XR_001434920.1	lncRNA
XM_012105333.2	USP54	mRNA	XR_001434920.1	lncRNA
XM_012105333.2	USP54	mRNA	XR_001434920.1	lncRNA
XM_012105333.2	USP54	mRNA	XR_001434920.1	lncRNA
XM_012105333.2	USP54	mRNA	XR_001434920.1	lncRNA
XM_012105333.2	USP54	mRNA	XR_001434920.1	lncRNA
XM_012105333.2	USP54	mRNA	XR_001434920.1	lncRNA
XM_015098082.1	RNASEH2B	mRNA	XR_001433856.1	lncRNA
XM_015098082.1	RNASEH2B	mRNA	XR_001433856.1	lncRNA
XM_015098082.1	RNASEH2B	mRNA	XR_001433856.1	lncRNA
XM_015098082.1	RNASEH2B	mRNA	XR_001433856.1	lncRNA
XM_015098082.1	RNASEH2B	mRNA	XR_001433856.1	lncRNA
XM_015098082.1	RNASEH2B	mRNA	XR_001433856.1	lncRNA
XM_015098082.1	RNASEH2B	mRNA	XR_001433856.1	lncRNA
XM_015098082.1	RNASEH2B	mRNA	XR_001433856.1	lncRNA
XM_015098082.1	RNASEH2B	mRNA	XR_001433856.1	lncRNA
XM_012174536.2	KIF21A	mRNA	XR_001435107.1	lncRNA
XM_012174536.2	KIF21A	mRNA	XR_001435107.1	lncRNA

XM_012174536.2	KIF21A	mRNA	XR_001435107.1	lncRNA
XM_012174536.2	KIF21A	mRNA	XR_001435107.1	lncRNA
XM_012174536.2	KIF21A	mRNA	XR_001435107.1	lncRNA
XM_012174536.2	KIF21A	mRNA	XR_001435107.1	lncRNA
XM_012174536.2	KIF21A	mRNA	XR_001435107.1	lncRNA
XM_012174536.2	KIF21A	mRNA	XR_001435107.1	lncRNA
XM_012174536.2	KIF21A	mRNA	XR_001435107.1	lncRNA
XM_004003021.3	DLG1	mRNA	XR_001435107.1	lncRNA
XM_004003021.3	DLG1	mRNA	XR_001435107.1	lncRNA
XM_004003021.3	DLG1	mRNA	XR_001435107.1	lncRNA
XM_004003021.3	DLG1	mRNA	XR_001435107.1	lncRNA
XM_004003021.3	DLG1	mRNA	XR_001435107.1	lncRNA
XM_004003021.3	DLG1	mRNA	XR_001435107.1	lncRNA
XM_004003021.3	DLG1	mRNA	XR_001435107.1	lncRNA
XM_004003021.3	DLG1	mRNA	XR_001435107.1	lncRNA
XM_004003021.3	DLG1	mRNA	XR_001435107.1	lncRNA
XM_012174704.2	ZBTB39	mRNA	XR_001435107.1	lncRNA
XM_012174704.2	ZBTB39	mRNA	XR_001435107.1	lncRNA
XM_012174704.2	ZBTB39	mRNA	XR_001435107.1	lncRNA
XM_012174704.2	ZBTB39	mRNA	XR_001435107.1	lncRNA
XM_012174704.2	ZBTB39	mRNA	XR_001435107.1	lncRNA
XM_012174704.2	ZBTB39	mRNA	XR_001435107.1	lncRNA
XM_012174704.2	ZBTB39	mRNA	XR_001435107.1	lncRNA
XM_012174704.2	ZBTB39	mRNA	XR_001435107.1	lncRNA
XM_012174704.2	ZBTB39	mRNA	XR_001435107.1	lncRNA
XM_012184362.2	AKAP11	mRNA	XR_001436817.1	lncRNA
XM_012184362.2	AKAP11	mRNA	XR_001436817.1	lncRNA
XM_012184362.2	AKAP11	mRNA	XR_001436817.1	lncRNA
XM_012184362.2	AKAP11	mRNA	XR_001436817.1	lncRNA
XM_012184362.2	AKAP11	mRNA	XR_001436817.1	lncRNA
XM_012184362.2	AKAP11	mRNA	XR_001436817.1	lncRNA
XM_012184362.2	AKAP11	mRNA	XR_001436817.1	lncRNA
XM_012184362.2	AKAP11	mRNA	XR_001436817.1	lncRNA
XM_012184362.2	AKAP11	mRNA	XR_001436817.1	lncRNA
XM_012105333.2	USP54	mRNA	XR_001435722.1	lncRNA
XM_012105333.2	USP54	mRNA	XR_001435722.1	lncRNA
XM_012105333.2	USP54	mRNA	XR_001435722.1	lncRNA
XM_012105333.2	USP54	mRNA	XR_001435722.1	lncRNA
XM_012105333.2	USP54	mRNA	XR_001435722.1	lncRNA
XM_012105333.2	USP54	mRNA	XR_001435722.1	lncRNA
XM_012105333.2	USP54	mRNA	XR_001435722.1	lncRNA
XM_012105333.2	USP54	mRNA	XR_001435722.1	lncRNA
XM_012105333.2	USP54	mRNA	XR_001435722.1	lncRNA
XM_012184362.2	AKAP11	mRNA	XR_001435107.1	lncRNA
XM_012184362.2	AKAP11	mRNA	XR_001435107.1	lncRNA
XM_012184362.2	AKAP11	mRNA	XR_001435107.1	lncRNA
XM_012184362.2	AKAP11	mRNA	XR_001435107.1	lncRNA
XM_012184362.2	AKAP11	mRNA	XR_001435107.1	lncRNA
XM_012184362.2	AKAP11	mRNA	XR_001435107.1	lncRNA
XM_012184362.2	AKAP11	mRNA	XR_001435107.1	lncRNA
XM_012184362.2	AKAP11	mRNA	XR_001435107.1	lncRNA
XM_012184362.2	AKAP11	mRNA	XR_001435107.1	lncRNA
XM_004006563.3	ZBTB39	mRNA	XR_001435107.1	lncRNA
XM_004006563.3	ZBTB39	mRNA	XR_001435107.1	lncRNA

XM_004006563.3	ZBTB39	mRNA	XR_001435107.1	lncRNA
XM_004006563.3	ZBTB39	mRNA	XR_001435107.1	lncRNA
XM_004006563.3	ZBTB39	mRNA	XR_001435107.1	lncRNA
XM_004006563.3	ZBTB39	mRNA	XR_001435107.1	lncRNA
XM_004006563.3	ZBTB39	mRNA	XR_001435107.1	lncRNA
XM_004006563.3	ZBTB39	mRNA	XR_001435107.1	lncRNA
XM_004006563.3	ZBTB39	mRNA	XR_001435107.1	lncRNA
XM_012184362.2	AKAP11	mRNA	XR_001435722.1	lncRNA
XM_012184362.2	AKAP11	mRNA	XR_001435722.1	lncRNA
XM_012184362.2	AKAP11	mRNA	XR_001435722.1	lncRNA
XM_012184362.2	AKAP11	mRNA	XR_001435722.1	lncRNA
XM_012184362.2	AKAP11	mRNA	XR_001435722.1	lncRNA
XM_012184362.2	AKAP11	mRNA	XR_001435722.1	lncRNA
XM_012184362.2	AKAP11	mRNA	XR_001435722.1	lncRNA
XM_012184362.2	AKAP11	mRNA	XR_001435722.1	lncRNA
XM_012184362.2	AKAP11	mRNA	XR_001435722.1	lncRNA
XM_012102832.2	PPRC1	mRNA	XR_001433856.1	lncRNA
XM_012102832.2	PPRC1	mRNA	XR_001433856.1	lncRNA
XM_012102832.2	PPRC1	mRNA	XR_001433856.1	lncRNA
XM_012102832.2	PPRC1	mRNA	XR_001433856.1	lncRNA
XM_012102832.2	PPRC1	mRNA	XR_001433856.1	lncRNA
XM_012102832.2	PPRC1	mRNA	XR_001433856.1	lncRNA
XM_012102832.2	PPRC1	mRNA	XR_001433856.1	lncRNA
XM_012102832.2	PPRC1	mRNA	XR_001433856.1	lncRNA
XM_012102832.2	PPRC1	mRNA	XR_001433856.1	lncRNA
XM_012105333.2	USP54	mRNA	XR_001436817.1	lncRNA
XM_012105333.2	USP54	mRNA	XR_001436817.1	lncRNA
XM_012105333.2	USP54	mRNA	XR_001436817.1	lncRNA
XM_012105333.2	USP54	mRNA	XR_001436817.1	lncRNA
XM_012105333.2	USP54	mRNA	XR_001436817.1	lncRNA
XM_012105333.2	USP54	mRNA	XR_001436817.1	lncRNA
XM_012105333.2	USP54	mRNA	XR_001436817.1	lncRNA
XM_012105333.2	USP54	mRNA	XR_001436817.1	lncRNA
XM_012105333.2	USP54	mRNA	XR_001436817.1	lncRNA
XM_012097035.1	C16H5orf42	mRNA	XR_001435722.1	lncRNA
XM_012097035.1	C16H5orf42	mRNA	XR_001435722.1	lncRNA
XM_012097035.1	C16H5orf42	mRNA	XR_001435722.1	lncRNA
XM_012097035.1	C16H5orf42	mRNA	XR_001435722.1	lncRNA
XM_012097035.1	C16H5orf42	mRNA	XR_001435722.1	lncRNA
XM_012097035.1	C16H5orf42	mRNA	XR_001435722.1	lncRNA
XM_012097035.1	C16H5orf42	mRNA	XR_001435722.1	lncRNA
XM_012097035.1	C16H5orf42	mRNA	XR_001435722.1	lncRNA
XM_012097035.1	C16H5orf42	mRNA	XR_001435722.1	lncRNA
XM_012095273.2	ARNTL	mRNA	XR_001433856.1	lncRNA
XM_012095273.2	ARNTL	mRNA	XR_001433856.1	lncRNA
XM_012095273.2	ARNTL	mRNA	XR_001433856.1	lncRNA
XM_012095273.2	ARNTL	mRNA	XR_001433856.1	lncRNA
XM_012095273.2	ARNTL	mRNA	XR_001433856.1	lncRNA
XM_012095273.2	ARNTL	mRNA	XR_001433856.1	lncRNA
XM_012095273.2	ARNTL	mRNA	XR_001433856.1	lncRNA
XM_012095273.2	ARNTL	mRNA	XR_001433856.1	lncRNA
XM_012095273.2	ARNTL	mRNA	XR_001433856.1	lncRNA
XM_004011768.3	SAMD12	mRNA	XR_001433856.1	lncRNA
XM_004011768.3	SAMD12	mRNA	XR_001433856.1	lncRNA

XM_004011768.3	SAMD12	mRNA	XR_001433856.1	lncRNA
XM_004011768.3	SAMD12	mRNA	XR_001433856.1	lncRNA
XM_004011768.3	SAMD12	mRNA	XR_001433856.1	lncRNA
XM_004011768.3	SAMD12	mRNA	XR_001433856.1	lncRNA
XM_004011768.3	SAMD12	mRNA	XR_001433856.1	lncRNA
XM_004011768.3	SAMD12	mRNA	XR_001433856.1	lncRNA
XM_004011768.3	SAMD12	mRNA	XR_001433856.1	lncRNA
XM_012105333.2	USP54	mRNA	XR_001435107.1	lncRNA
XM_012105333.2	USP54	mRNA	XR_001435107.1	lncRNA
XM_012105333.2	USP54	mRNA	XR_001435107.1	lncRNA
XM_012105333.2	USP54	mRNA	XR_001435107.1	lncRNA
XM_012105333.2	USP54	mRNA	XR_001435107.1	lncRNA
XM_012105333.2	USP54	mRNA	XR_001435107.1	lncRNA
XM_012105333.2	USP54	mRNA	XR_001435107.1	lncRNA
XM_012105333.2	USP54	mRNA	XR_001435107.1	lncRNA
XM_012105333.2	USP54	mRNA	XR_001435107.1	lncRNA
XM_004011768.3	SAMD12	mRNA	XR_001435107.1	lncRNA
XM_004011768.3	SAMD12	mRNA	XR_001435107.1	lncRNA
XM_004011768.3	SAMD12	mRNA	XR_001435107.1	lncRNA
XM_004011768.3	SAMD12	mRNA	XR_001435107.1	lncRNA
XM_004011768.3	SAMD12	mRNA	XR_001435107.1	lncRNA
XM_004011768.3	SAMD12	mRNA	XR_001435107.1	lncRNA
XM_004011768.3	SAMD12	mRNA	XR_001435107.1	lncRNA
XM_004011768.3	SAMD12	mRNA	XR_001435107.1	lncRNA
XM_004011768.3	SAMD12	mRNA	XR_001435107.1	lncRNA
XM_012174536.2	KIF21A	mRNA	XR_001435722.1	lncRNA
XM_012174536.2	KIF21A	mRNA	XR_001435722.1	lncRNA
XM_012174536.2	KIF21A	mRNA	XR_001435722.1	lncRNA
XM_012174536.2	KIF21A	mRNA	XR_001435722.1	lncRNA
XM_012174536.2	KIF21A	mRNA	XR_001435722.1	lncRNA
XM_012174536.2	KIF21A	mRNA	XR_001435722.1	lncRNA
XM_012174536.2	KIF21A	mRNA	XR_001435722.1	lncRNA
XM_012174536.2	KIF21A	mRNA	XR_001435722.1	lncRNA
XM_012174536.2	KIF21A	mRNA	XR_001435722.1	lncRNA
XM_012174704.2	ZBTB39	mRNA	XR_001436817.1	lncRNA
XM_012174704.2	ZBTB39	mRNA	XR_001436817.1	lncRNA
XM_012174704.2	ZBTB39	mRNA	XR_001436817.1	lncRNA
XM_012174704.2	ZBTB39	mRNA	XR_001436817.1	lncRNA
XM_012174704.2	ZBTB39	mRNA	XR_001436817.1	lncRNA
XM_012174704.2	ZBTB39	mRNA	XR_001436817.1	lncRNA
XM_012174704.2	ZBTB39	mRNA	XR_001436817.1	lncRNA
XM_012174704.2	ZBTB39	mRNA	XR_001436817.1	lncRNA
XM_012174704.2	ZBTB39	mRNA	XR_001436817.1	lncRNA
XM_012097555.2	RAPGEF2	mRNA	XR_001436065.1	lncRNA
XM_012097555.2	RAPGEF2	mRNA	XR_001436065.1	lncRNA
XM_012097555.2	RAPGEF2	mRNA	XR_001436065.1	lncRNA
XM_012097555.2	RAPGEF2	mRNA	XR_001436065.1	lncRNA
XM_012097555.2	RAPGEF2	mRNA	XR_001436065.1	lncRNA
XM_012097555.2	RAPGEF2	mRNA	XR_001436065.1	lncRNA
XM_012097555.2	RAPGEF2	mRNA	XR_001436065.1	lncRNA
XM_012097555.2	RAPGEF2	mRNA	XR_001436065.1	lncRNA
XM_012097555.2	RAPGEF2	mRNA	XR_001436065.1	lncRNA
XM_012097555.2	RAPGEF2	mRNA	XR_001436064.1	lncRNA
XM_012097555.2	RAPGEF2	mRNA	XR_001436064.1	lncRNA

XM_012097555.2	RAPGEF2	mRNA	XR_001436064.1	lncRNA
XM_012097555.2	RAPGEF2	mRNA	XR_001436064.1	lncRNA
XM_012097555.2	RAPGEF2	mRNA	XR_001436064.1	lncRNA
XM_012097555.2	RAPGEF2	mRNA	XR_001436064.1	lncRNA
XM_012097555.2	RAPGEF2	mRNA	XR_001436064.1	lncRNA
XM_012097555.2	RAPGEF2	mRNA	XR_001436064.1	lncRNA
XM_012097555.2	RAPGEF2	mRNA	XR_001436064.1	lncRNA
XM_004010552.3	VPS13C	mRNA	XR_001435726.1	lncRNA
XM_004010552.3	VPS13C	mRNA	XR_001435726.1	lncRNA
XM_004010552.3	VPS13C	mRNA	XR_001435726.1	lncRNA
XM_004010552.3	VPS13C	mRNA	XR_001435726.1	lncRNA
XM_004010552.3	VPS13C	mRNA	XR_001435726.1	lncRNA
XM_004010552.3	VPS13C	mRNA	XR_001435726.1	lncRNA
XM_004010552.3	VPS13C	mRNA	XR_001435726.1	lncRNA
XM_004010552.3	VPS13C	mRNA	XR_001435726.1	lncRNA
XM_004010552.3	VPS13C	mRNA	XR_001435726.1	lncRNA
XM_004010549.3	VPS13C	mRNA	XR_001436065.1	lncRNA
XM_004010549.3	VPS13C	mRNA	XR_001436065.1	lncRNA
XM_004010549.3	VPS13C	mRNA	XR_001436065.1	lncRNA
XM_004010549.3	VPS13C	mRNA	XR_001436065.1	lncRNA
XM_004010549.3	VPS13C	mRNA	XR_001436065.1	lncRNA
XM_004010549.3	VPS13C	mRNA	XR_001436065.1	lncRNA
XM_004010549.3	VPS13C	mRNA	XR_001436065.1	lncRNA
XM_004010549.3	VPS13C	mRNA	XR_001436065.1	lncRNA
XM_004010549.3	VPS13C	mRNA	XR_001436065.1	lncRNA
XM_012105324.2	USP54	mRNA	XR_001436065.1	lncRNA
XM_012105324.2	USP54	mRNA	XR_001436065.1	lncRNA
XM_012105324.2	USP54	mRNA	XR_001436065.1	lncRNA
XM_012105324.2	USP54	mRNA	XR_001436065.1	lncRNA
XM_012105324.2	USP54	mRNA	XR_001436065.1	lncRNA
XM_012105324.2	USP54	mRNA	XR_001436065.1	lncRNA
XM_012105324.2	USP54	mRNA	XR_001436065.1	lncRNA
XM_012105324.2	USP54	mRNA	XR_001436065.1	lncRNA
XM_012105324.2	USP54	mRNA	XR_001436065.1	lncRNA
XM_012105324.2	USP54	mRNA	XR_001436064.1	lncRNA
XM_012105324.2	USP54	mRNA	XR_001436064.1	lncRNA
XM_012105324.2	USP54	mRNA	XR_001436064.1	lncRNA
XM_012105324.2	USP54	mRNA	XR_001436064.1	lncRNA
XM_012105324.2	USP54	mRNA	XR_001436064.1	lncRNA
XM_012105324.2	USP54	mRNA	XR_001436064.1	lncRNA
XM_012105324.2	USP54	mRNA	XR_001436064.1	lncRNA
XM_012105324.2	USP54	mRNA	XR_001436064.1	lncRNA
XM_012105324.2	USP54	mRNA	XR_001436064.1	lncRNA
XM_012188348.1	KIAA1217	mRNA	XR_001436065.1	lncRNA
XM_012188348.1	KIAA1217	mRNA	XR_001436065.1	lncRNA
XM_012188348.1	KIAA1217	mRNA	XR_001436065.1	lncRNA
XM_012188348.1	KIAA1217	mRNA	XR_001436065.1	lncRNA
XM_012188348.1	KIAA1217	mRNA	XR_001436065.1	lncRNA
XM_012188348.1	KIAA1217	mRNA	XR_001436065.1	lncRNA
XM_012188348.1	KIAA1217	mRNA	XR_001436065.1	lncRNA
XM_012188348.1	KIAA1217	mRNA	XR_001436065.1	lncRNA
XM_012188348.1	KIAA1217	mRNA	XR_001436065.1	lncRNA
XM_012105324.2	USP54	mRNA	XR_001435726.1	lncRNA
XM_012105324.2	USP54	mRNA	XR_001435726.1	lncRNA

XM_012105324.2	USP54	mRNA	XR_001435726.1	lncRNA
XM_012105324.2	USP54	mRNA	XR_001435726.1	lncRNA
XM_012105324.2	USP54	mRNA	XR_001435726.1	lncRNA
XM_012105324.2	USP54	mRNA	XR_001435726.1	lncRNA
XM_012105324.2	USP54	mRNA	XR_001435726.1	lncRNA
XM_012105324.2	USP54	mRNA	XR_001435726.1	lncRNA
XM_012105324.2	USP54	mRNA	XR_001435726.1	lncRNA
XM_015103687.1	PTPN2	mRNA	XR_001435723.1	lncRNA
XM_015103687.1	PTPN2	mRNA	XR_001435723.1	lncRNA
XM_015103687.1	PTPN2	mRNA	XR_001435723.1	lncRNA
XM_015103687.1	PTPN2	mRNA	XR_001435723.1	lncRNA
XM_015103687.1	PTPN2	mRNA	XR_001435723.1	lncRNA
XM_015103687.1	PTPN2	mRNA	XR_001435723.1	lncRNA
XM_015103687.1	PTPN2	mRNA	XR_001435723.1	lncRNA
XM_015103687.1	PTPN2	mRNA	XR_001435723.1	lncRNA
XM_015103687.1	PTPN2	mRNA	XR_001435723.1	lncRNA
XM_012095733.1	BTBD10	mRNA	XR_001435723.1	lncRNA
XM_012095733.1	BTBD10	mRNA	XR_001435723.1	lncRNA
XM_012095733.1	BTBD10	mRNA	XR_001435723.1	lncRNA
XM_012095733.1	BTBD10	mRNA	XR_001435723.1	lncRNA
XM_012095733.1	BTBD10	mRNA	XR_001435723.1	lncRNA
XM_012095733.1	BTBD10	mRNA	XR_001435723.1	lncRNA
XM_012095733.1	BTBD10	mRNA	XR_001435723.1	lncRNA
XM_012095733.1	BTBD10	mRNA	XR_001435723.1	lncRNA
XM_012095733.1	BTBD10	mRNA	XR_001435723.1	lncRNA
XM_012095733.1	BTBD10	mRNA	XR_001436064.1	lncRNA
XM_012095733.1	BTBD10	mRNA	XR_001436064.1	lncRNA
XM_012095733.1	BTBD10	mRNA	XR_001436064.1	lncRNA
XM_012095733.1	BTBD10	mRNA	XR_001436064.1	lncRNA
XM_012095733.1	BTBD10	mRNA	XR_001436064.1	lncRNA
XM_012095733.1	BTBD10	mRNA	XR_001436064.1	lncRNA
XM_012095733.1	BTBD10	mRNA	XR_001436064.1	lncRNA
XM_012095733.1	BTBD10	mRNA	XR_001436064.1	lncRNA
XM_012095733.1	BTBD10	mRNA	XR_001436064.1	lncRNA
XM_012105324.2	USP54	mRNA	XR_001435723.1	lncRNA
XM_012105324.2	USP54	mRNA	XR_001435723.1	lncRNA
XM_012105324.2	USP54	mRNA	XR_001435723.1	lncRNA
XM_012105324.2	USP54	mRNA	XR_001435723.1	lncRNA
XM_012105324.2	USP54	mRNA	XR_001435723.1	lncRNA
XM_012105324.2	USP54	mRNA	XR_001435723.1	lncRNA
XM_012105324.2	USP54	mRNA	XR_001435723.1	lncRNA
XM_012105324.2	USP54	mRNA	XR_001435723.1	lncRNA
XM_012105324.2	USP54	mRNA	XR_001435723.1	lncRNA
XM_004010549.3	VPS13C	mRNA	XR_001436064.1	lncRNA
XM_004010549.3	VPS13C	mRNA	XR_001436064.1	lncRNA
XM_004010549.3	VPS13C	mRNA	XR_001436064.1	lncRNA
XM_004010549.3	VPS13C	mRNA	XR_001436064.1	lncRNA
XM_004010549.3	VPS13C	mRNA	XR_001436064.1	lncRNA
XM_004010549.3	VPS13C	mRNA	XR_001436064.1	lncRNA
XM_004010549.3	VPS13C	mRNA	XR_001436064.1	lncRNA
XM_004010549.3	VPS13C	mRNA	XR_001436064.1	lncRNA
XM_004010549.3	VPS13C	mRNA	XR_001436064.1	lncRNA
XM_015103687.1	PTPN2	mRNA	XR_001435726.1	lncRNA
XM_015103687.1	PTPN2	mRNA	XR_001435726.1	lncRNA

XM_015103687.1	PTPN2	mRNA	XR_001435726.1	lncRNA
XM_015103687.1	PTPN2	mRNA	XR_001435726.1	lncRNA
XM_015103687.1	PTPN2	mRNA	XR_001435726.1	lncRNA
XM_015103687.1	PTPN2	mRNA	XR_001435726.1	lncRNA
XM_015103687.1	PTPN2	mRNA	XR_001435726.1	lncRNA
XM_015103687.1	PTPN2	mRNA	XR_001435726.1	lncRNA
XM_015103687.1	PTPN2	mRNA	XR_001435726.1	lncRNA
XM_015099739.1	LOC101122805	mRNA	XR_001436612.1	lncRNA
XM_015099739.1	LOC101122805	mRNA	XR_001436612.1	lncRNA
XM_015099739.1	LOC101122805	mRNA	XR_001436612.1	lncRNA
XM_015099739.1	LOC101122805	mRNA	XR_001436612.1	lncRNA
XM_015099739.1	LOC101122805	mRNA	XR_001436612.1	lncRNA
XM_015099739.1	LOC101122805	mRNA	XR_001436612.1	lncRNA
XM_015099739.1	LOC101122805	mRNA	XR_001436612.1	lncRNA
XM_015099739.1	LOC101122805	mRNA	XR_001436612.1	lncRNA
XM_004003770.3	IMPG2	mRNA	XR_001436390.1	lncRNA
XM_004003770.3	IMPG2	mRNA	XR_001436390.1	lncRNA
XM_004003770.3	IMPG2	mRNA	XR_001436390.1	lncRNA
XM_004003770.3	IMPG2	mRNA	XR_001436390.1	lncRNA
XM_004003770.3	IMPG2	mRNA	XR_001436390.1	lncRNA
XM_004003770.3	IMPG2	mRNA	XR_001436390.1	lncRNA
XM_004003770.3	IMPG2	mRNA	XR_001436390.1	lncRNA
XM_004003770.3	IMPG2	mRNA	XR_001436390.1	lncRNA
XM_015093065.1	FAM219A	mRNA	TCONS_00049802	lncRNA
XM_015093065.1	FAM219A	mRNA	TCONS_00049802	lncRNA
XM_015093065.1	FAM219A	mRNA	TCONS_00049802	lncRNA
XM_015093065.1	FAM219A	mRNA	TCONS_00049802	lncRNA
XM_015093065.1	FAM219A	mRNA	TCONS_00049802	lncRNA
XM_015093065.1	FAM219A	mRNA	TCONS_00049802	lncRNA
XM_015093065.1	FAM219A	mRNA	TCONS_00049802	lncRNA
XM_015093065.1	FAM219A	mRNA	TCONS_00049802	lncRNA
XM_012184885.2	DOCK9	mRNA	XR_001023627.2	lncRNA
XM_012184885.2	DOCK9	mRNA	XR_001023627.2	lncRNA
XM_012184885.2	DOCK9	mRNA	XR_001023627.2	lncRNA
XM_012184885.2	DOCK9	mRNA	XR_001023627.2	lncRNA
XM_012184885.2	DOCK9	mRNA	XR_001023627.2	lncRNA
XM_012184885.2	DOCK9	mRNA	XR_001023627.2	lncRNA
XM_012184885.2	DOCK9	mRNA	XR_001023627.2	lncRNA
XM_012184885.2	DOCK9	mRNA	XR_001023627.2	lncRNA
XM_015094225.1	ROCK2	mRNA	XR_001434127.1	lncRNA
XM_015094225.1	ROCK2	mRNA	XR_001434127.1	lncRNA
XM_015094225.1	ROCK2	mRNA	XR_001434127.1	lncRNA
XM_015094225.1	ROCK2	mRNA	XR_001434127.1	lncRNA
XM_015094225.1	ROCK2	mRNA	XR_001434127.1	lncRNA
XM_015094225.1	ROCK2	mRNA	XR_001434127.1	lncRNA
XM_015094225.1	ROCK2	mRNA	XR_001434127.1	lncRNA
XM_015094225.1	ROCK2	mRNA	XR_001434127.1	lncRNA
XM_015105074.1	LONRF3	mRNA	XR_001434127.1	lncRNA
XM_015105074.1	LONRF3	mRNA	XR_001434127.1	lncRNA
XM_015105074.1	LONRF3	mRNA	XR_001434127.1	lncRNA
XM_015105074.1	LONRF3	mRNA	XR_001434127.1	lncRNA
XM_015105074.1	LONRF3	mRNA	XR_001434127.1	lncRNA
XM_015105074.1	LONRF3	mRNA	XR_001434127.1	lncRNA
XM_015105074.1	LONRF3	mRNA	XR_001434127.1	lncRNA

XM_015105074.1	LONRF3	mRNA	XR_001434127.1	lncRNA
XM_012137434.2	NEB	mRNA	XR_001436612.1	lncRNA
XM_012137434.2	NEB	mRNA	XR_001436612.1	lncRNA
XM_012137434.2	NEB	mRNA	XR_001436612.1	lncRNA
XM_012137434.2	NEB	mRNA	XR_001436612.1	lncRNA
XM_012137434.2	NEB	mRNA	XR_001436612.1	lncRNA
XM_012137434.2	NEB	mRNA	XR_001436612.1	lncRNA
XM_012137434.2	NEB	mRNA	XR_001436612.1	lncRNA
XM_012137434.2	NEB	mRNA	XR_001436612.1	lncRNA
XM_015094531.1	ANAPC1	mRNA	XR_001436612.1	lncRNA
XM_015094531.1	ANAPC1	mRNA	XR_001436612.1	lncRNA
XM_015094531.1	ANAPC1	mRNA	XR_001436612.1	lncRNA
XM_015094531.1	ANAPC1	mRNA	XR_001436612.1	lncRNA
XM_015094531.1	ANAPC1	mRNA	XR_001436612.1	lncRNA
XM_015094531.1	ANAPC1	mRNA	XR_001436612.1	lncRNA
XM_015094531.1	ANAPC1	mRNA	XR_001436612.1	lncRNA
XM_015094531.1	ANAPC1	mRNA	XR_001436612.1	lncRNA
XM_015101341.1	OTULIN	mRNA	TCONS_00049802	lncRNA
XM_015101341.1	OTULIN	mRNA	TCONS_00049802	lncRNA
XM_015101341.1	OTULIN	mRNA	TCONS_00049802	lncRNA
XM_015101341.1	OTULIN	mRNA	TCONS_00049802	lncRNA
XM_015101341.1	OTULIN	mRNA	TCONS_00049802	lncRNA
XM_015101341.1	OTULIN	mRNA	TCONS_00049802	lncRNA
XM_015101341.1	OTULIN	mRNA	TCONS_00049802	lncRNA
XM_015101341.1	OTULIN	mRNA	TCONS_00049802	lncRNA
XM_015103687.1	PTPN2	mRNA	XR_001023627.2	lncRNA
XM_015103687.1	PTPN2	mRNA	XR_001023627.2	lncRNA
XM_015103687.1	PTPN2	mRNA	XR_001023627.2	lncRNA
XM_015103687.1	PTPN2	mRNA	XR_001023627.2	lncRNA
XM_015103687.1	PTPN2	mRNA	XR_001023627.2	lncRNA
XM_015103687.1	PTPN2	mRNA	XR_001023627.2	lncRNA
XM_015103687.1	PTPN2	mRNA	XR_001023627.2	lncRNA
XM_015103687.1	PTPN2	mRNA	XR_001023627.2	lncRNA
XM_012105324.2	USP54	mRNA	XR_001023627.2	lncRNA
XM_012105324.2	USP54	mRNA	XR_001023627.2	lncRNA
XM_012105324.2	USP54	mRNA	XR_001023627.2	lncRNA
XM_012105324.2	USP54	mRNA	XR_001023627.2	lncRNA
XM_012105324.2	USP54	mRNA	XR_001023627.2	lncRNA
XM_012105324.2	USP54	mRNA	XR_001023627.2	lncRNA
XM_012105324.2	USP54	mRNA	XR_001023627.2	lncRNA
XM_012105324.2	USP54	mRNA	XR_001023627.2	lncRNA
XM_012105324.2	USP54	mRNA	TCONS_00049802	lncRNA
XM_012105324.2	USP54	mRNA	TCONS_00049802	lncRNA
XM_012105324.2	USP54	mRNA	TCONS_00049802	lncRNA
XM_012105324.2	USP54	mRNA	TCONS_00049802	lncRNA
XM_012105324.2	USP54	mRNA	TCONS_00049802	lncRNA
XM_012105324.2	USP54	mRNA	TCONS_00049802	lncRNA
XM_012105324.2	USP54	mRNA	TCONS_00049802	lncRNA
XM_012105324.2	USP54	mRNA	TCONS_00049802	lncRNA
XM_012095733.1	BTBD10	mRNA	XR_001023627.2	lncRNA
XM_012095733.1	BTBD10	mRNA	XR_001023627.2	lncRNA
XM_012095733.1	BTBD10	mRNA	XR_001023627.2	lncRNA
XM_012095733.1	BTBD10	mRNA	XR_001023627.2	lncRNA
XM_012095733.1	BTBD10	mRNA	XR_001023627.2	lncRNA

XM_012095733.1	BTBD10	mRNA	XR_001023627.2	lncRNA
XM_012095733.1	BTBD10	mRNA	XR_001023627.2	lncRNA
XM_012095733.1	BTBD10	mRNA	XR_001023627.2	lncRNA
XM_012095733.1	BTBD10	mRNA	XR_001434127.1	lncRNA
XM_012095733.1	BTBD10	mRNA	XR_001434127.1	lncRNA
XM_012095733.1	BTBD10	mRNA	XR_001434127.1	lncRNA
XM_012095733.1	BTBD10	mRNA	XR_001434127.1	lncRNA
XM_012095733.1	BTBD10	mRNA	XR_001434127.1	lncRNA
XM_012095733.1	BTBD10	mRNA	XR_001434127.1	lncRNA
XM_012095733.1	BTBD10	mRNA	XR_001434127.1	lncRNA
XM_012095733.1	BTBD10	mRNA	XR_001434127.1	lncRNA
XM_004010549.3	VPS13C	mRNA	TCONS_00049802	lncRNA
XM_004010549.3	VPS13C	mRNA	TCONS_00049802	lncRNA
XM_004010549.3	VPS13C	mRNA	TCONS_00049802	lncRNA
XM_004010549.3	VPS13C	mRNA	TCONS_00049802	lncRNA
XM_004010549.3	VPS13C	mRNA	TCONS_00049802	lncRNA
XM_004010549.3	VPS13C	mRNA	TCONS_00049802	lncRNA
XM_004010549.3	VPS13C	mRNA	TCONS_00049802	lncRNA
XM_004010549.3	VPS13C	mRNA	TCONS_00049802	lncRNA
XM_012102832.2	PPRC1	mRNA	XR_001436390.1	lncRNA
XM_012102832.2	PPRC1	mRNA	XR_001436390.1	lncRNA
XM_012102832.2	PPRC1	mRNA	XR_001436390.1	lncRNA
XM_012102832.2	PPRC1	mRNA	XR_001436390.1	lncRNA
XM_012102832.2	PPRC1	mRNA	XR_001436390.1	lncRNA
XM_012102832.2	PPRC1	mRNA	XR_001436390.1	lncRNA
XM_012102832.2	PPRC1	mRNA	XR_001436390.1	lncRNA
XM_012102832.2	PPRC1	mRNA	XR_001436390.1	lncRNA
XM_012174536.2	KIF21A	mRNA	XR_001040147.1	lncRNA
XM_012174536.2	KIF21A	mRNA	XR_001040147.1	lncRNA
XM_012174536.2	KIF21A	mRNA	XR_001040147.1	lncRNA
XM_012174536.2	KIF21A	mRNA	XR_001040147.1	lncRNA
XM_012174536.2	KIF21A	mRNA	XR_001040147.1	lncRNA
XM_012174536.2	KIF21A	mRNA	XR_001040147.1	lncRNA
XM_012174536.2	KIF21A	mRNA	XR_001040147.1	lncRNA
XM_012174536.2	KIF21A	mRNA	XR_001040147.1	lncRNA
XM_012105333.2	USP54	mRNA	XR_001436390.1	lncRNA
XM_012105333.2	USP54	mRNA	XR_001436390.1	lncRNA
XM_012105333.2	USP54	mRNA	XR_001436390.1	lncRNA
XM_012105333.2	USP54	mRNA	XR_001436390.1	lncRNA
XM_012105333.2	USP54	mRNA	XR_001436390.1	lncRNA
XM_012105333.2	USP54	mRNA	XR_001436390.1	lncRNA
XM_012105333.2	USP54	mRNA	XR_001436390.1	lncRNA
XM_012105333.2	USP54	mRNA	XR_001436390.1	lncRNA
XM_012189777.1	CHD9	mRNA	XR_001436390.1	lncRNA
XM_012189777.1	CHD9	mRNA	XR_001436390.1	lncRNA
XM_012189777.1	CHD9	mRNA	XR_001436390.1	lncRNA
XM_012189777.1	CHD9	mRNA	XR_001436390.1	lncRNA
XM_012189777.1	CHD9	mRNA	XR_001436390.1	lncRNA
XM_012189777.1	CHD9	mRNA	XR_001436390.1	lncRNA
XM_012189777.1	CHD9	mRNA	XR_001436390.1	lncRNA
XM_012189777.1	CHD9	mRNA	XR_001436390.1	lncRNA
XM_012095273.2	ARNTL	mRNA	XR_001436390.1	lncRNA
XM_012095273.2	ARNTL	mRNA	XR_001436390.1	lncRNA
XM_012095273.2	ARNTL	mRNA	XR_001436390.1	lncRNA

XM_012095273.2	ARNTL	mRNA	XR_001436390.1	lncRNA
XM_012095273.2	ARNTL	mRNA	XR_001436390.1	lncRNA
XM_012095273.2	ARNTL	mRNA	XR_001436390.1	lncRNA
XM_012095273.2	ARNTL	mRNA	XR_001436390.1	lncRNA
XM_012095273.2	ARNTL	mRNA	XR_001436390.1	lncRNA
XM_012186362.1	PITPNC1	mRNA	XR_001436612.1	lncRNA
XM_012186362.1	PITPNC1	mRNA	XR_001436612.1	lncRNA
XM_012186362.1	PITPNC1	mRNA	XR_001436612.1	lncRNA
XM_012186362.1	PITPNC1	mRNA	XR_001436612.1	lncRNA
XM_012186362.1	PITPNC1	mRNA	XR_001436612.1	lncRNA
XM_012186362.1	PITPNC1	mRNA	XR_001436612.1	lncRNA
XM_012186362.1	PITPNC1	mRNA	XR_001436612.1	lncRNA
XM_012186362.1	PITPNC1	mRNA	XR_001436612.1	lncRNA
XM_012182450.2	IBTK	mRNA	XR_001436064.1	lncRNA
XM_012182450.2	IBTK	mRNA	XR_001436064.1	lncRNA
XM_012182450.2	IBTK	mRNA	XR_001436064.1	lncRNA
XM_012182450.2	IBTK	mRNA	XR_001436064.1	lncRNA
XM_012182450.2	IBTK	mRNA	XR_001436064.1	lncRNA
XM_012182450.2	IBTK	mRNA	XR_001436064.1	lncRNA
XM_012182450.2	IBTK	mRNA	XR_001436064.1	lncRNA
XM_012182450.2	IBTK	mRNA	XR_001436064.1	lncRNA
XM_012186312.2	DDX42	mRNA	XR_001435726.1	lncRNA
XM_012186312.2	DDX42	mRNA	XR_001435726.1	lncRNA
XM_012186312.2	DDX42	mRNA	XR_001435726.1	lncRNA
XM_012186312.2	DDX42	mRNA	XR_001435726.1	lncRNA
XM_012186312.2	DDX42	mRNA	XR_001435726.1	lncRNA
XM_012186312.2	DDX42	mRNA	XR_001435726.1	lncRNA
XM_012186312.2	DDX42	mRNA	XR_001435726.1	lncRNA
XM_012186312.2	DDX42	mRNA	XR_001435726.1	lncRNA
XM_012097365.2	SH3D19	mRNA	XR_001024230.1	lncRNA
XM_012097365.2	SH3D19	mRNA	XR_001024230.1	lncRNA
XM_012097365.2	SH3D19	mRNA	XR_001024230.1	lncRNA
XM_012097365.2	SH3D19	mRNA	XR_001024230.1	lncRNA
XM_012097365.2	SH3D19	mRNA	XR_001024230.1	lncRNA
XM_012097365.2	SH3D19	mRNA	XR_001024230.1	lncRNA
XM_012097365.2	SH3D19	mRNA	XR_001024230.1	lncRNA
XM_012097365.2	SH3D19	mRNA	XR_001024230.1	lncRNA
XM_004017655.3	PRR14L	mRNA	XR_001435726.1	lncRNA
XM_004017655.3	PRR14L	mRNA	XR_001435726.1	lncRNA
XM_004017655.3	PRR14L	mRNA	XR_001435726.1	lncRNA
XM_004017655.3	PRR14L	mRNA	XR_001435726.1	lncRNA
XM_004017655.3	PRR14L	mRNA	XR_001435726.1	lncRNA
XM_004017655.3	PRR14L	mRNA	XR_001435726.1	lncRNA
XM_004017655.3	PRR14L	mRNA	XR_001435726.1	lncRNA
XM_004017655.3	PRR14L	mRNA	XR_001435726.1	lncRNA
XM_012187413.1	NRIP1	mRNA	XR_001024230.1	lncRNA
XM_012187413.1	NRIP1	mRNA	XR_001024230.1	lncRNA
XM_012187413.1	NRIP1	mRNA	XR_001024230.1	lncRNA
XM_012187413.1	NRIP1	mRNA	XR_001024230.1	lncRNA
XM_012187413.1	NRIP1	mRNA	XR_001024230.1	lncRNA
XM_012187413.1	NRIP1	mRNA	XR_001024230.1	lncRNA
XM_012187413.1	NRIP1	mRNA	XR_001024230.1	lncRNA
XM_012187413.1	NRIP1	mRNA	XR_001024230.1	lncRNA
XM_012186312.2	DDX42	mRNA	XR_001435723.1	lncRNA

XM_012186312.2	DDX42	mRNA	XR_001435723.1	lncRNA
XM_012186312.2	DDX42	mRNA	XR_001435723.1	lncRNA
XM_012186312.2	DDX42	mRNA	XR_001435723.1	lncRNA
XM_012186312.2	DDX42	mRNA	XR_001435723.1	lncRNA
XM_012186312.2	DDX42	mRNA	XR_001435723.1	lncRNA
XM_012186312.2	DDX42	mRNA	XR_001435723.1	lncRNA
XM_012186312.2	DDX42	mRNA	XR_001435723.1	lncRNA
XM_004017788.3	ZFANL3	mRNA	XR_001435726.1	lncRNA
XM_004017788.3	ZFANL3	mRNA	XR_001435726.1	lncRNA
XM_004017788.3	ZFANL3	mRNA	XR_001435726.1	lncRNA
XM_004017788.3	ZFANL3	mRNA	XR_001435726.1	lncRNA
XM_004017788.3	ZFANL3	mRNA	XR_001435726.1	lncRNA
XM_004017788.3	ZFANL3	mRNA	XR_001435726.1	lncRNA
XM_004017788.3	ZFANL3	mRNA	XR_001435726.1	lncRNA
XM_004017788.3	ZFANL3	mRNA	XR_001435726.1	lncRNA
XM_012173553.2	STRN	mRNA	XR_001436064.1	lncRNA
XM_012173553.2	STRN	mRNA	XR_001436064.1	lncRNA
XM_012173553.2	STRN	mRNA	XR_001436064.1	lncRNA
XM_012173553.2	STRN	mRNA	XR_001436064.1	lncRNA
XM_012173553.2	STRN	mRNA	XR_001436064.1	lncRNA
XM_012173553.2	STRN	mRNA	XR_001436064.1	lncRNA
XM_012173553.2	STRN	mRNA	XR_001436064.1	lncRNA
XM_012173553.2	STRN	mRNA	XR_001436064.1	lncRNA
XM_015095237.1	DMTF1	mRNA	XR_001024230.1	lncRNA
XM_015095237.1	DMTF1	mRNA	XR_001024230.1	lncRNA
XM_015095237.1	DMTF1	mRNA	XR_001024230.1	lncRNA
XM_015095237.1	DMTF1	mRNA	XR_001024230.1	lncRNA
XM_015095237.1	DMTF1	mRNA	XR_001024230.1	lncRNA
XM_015095237.1	DMTF1	mRNA	XR_001024230.1	lncRNA
XM_015095237.1	DMTF1	mRNA	XR_001024230.1	lncRNA
XM_015095237.1	DMTF1	mRNA	XR_001024230.1	lncRNA
XM_012098812.2	RALGAPA1	mRNA	XR_001435726.1	lncRNA
XM_012098812.2	RALGAPA1	mRNA	XR_001435726.1	lncRNA
XM_012098812.2	RALGAPA1	mRNA	XR_001435726.1	lncRNA
XM_012098812.2	RALGAPA1	mRNA	XR_001435726.1	lncRNA
XM_012098812.2	RALGAPA1	mRNA	XR_001435726.1	lncRNA
XM_012098812.2	RALGAPA1	mRNA	XR_001435726.1	lncRNA
XM_012098812.2	RALGAPA1	mRNA	XR_001435726.1	lncRNA
XM_012098812.2	RALGAPA1	mRNA	XR_001435726.1	lncRNA
XM_004003770.3	IMPG2	mRNA	XR_001435726.1	lncRNA
XM_004003770.3	IMPG2	mRNA	XR_001435726.1	lncRNA
XM_004003770.3	IMPG2	mRNA	XR_001435726.1	lncRNA
XM_004003770.3	IMPG2	mRNA	XR_001435726.1	lncRNA
XM_004003770.3	IMPG2	mRNA	XR_001435726.1	lncRNA
XM_004003770.3	IMPG2	mRNA	XR_001435726.1	lncRNA
XM_004003770.3	IMPG2	mRNA	XR_001435726.1	lncRNA
XM_004003770.3	IMPG2	mRNA	XR_001435726.1	lncRNA
XM_012182450.2	IBTK	mRNA	XR_001436065.1	lncRNA
XM_012182450.2	IBTK	mRNA	XR_001436065.1	lncRNA
XM_012182450.2	IBTK	mRNA	XR_001436065.1	lncRNA
XM_012182450.2	IBTK	mRNA	XR_001436065.1	lncRNA
XM_012182450.2	IBTK	mRNA	XR_001436065.1	lncRNA
XM_012182450.2	IBTK	mRNA	XR_001436065.1	lncRNA
XM_012182450.2	IBTK	mRNA	XR_001436065.1	lncRNA

XM_012182450.2	IBTK	mRNA	XR_001436065.1	lncRNA
XM_004017788.3	ZFANL3	mRNA	XR_001435723.1	lncRNA
XM_004017788.3	ZFANL3	mRNA	XR_001435723.1	lncRNA
XM_004017788.3	ZFANL3	mRNA	XR_001435723.1	lncRNA
XM_004017788.3	ZFANL3	mRNA	XR_001435723.1	lncRNA
XM_004017788.3	ZFANL3	mRNA	XR_001435723.1	lncRNA
XM_004017788.3	ZFANL3	mRNA	XR_001435723.1	lncRNA
XM_004017788.3	ZFANL3	mRNA	XR_001435723.1	lncRNA
XM_004017788.3	ZFANL3	mRNA	XR_001435723.1	lncRNA
XM_004017788.3	ZFANL3	mRNA	XR_001024230.1	lncRNA
XM_004017788.3	ZFANL3	mRNA	XR_001024230.1	lncRNA
XM_004017788.3	ZFANL3	mRNA	XR_001024230.1	lncRNA
XM_004017788.3	ZFANL3	mRNA	XR_001024230.1	lncRNA
XM_004017788.3	ZFANL3	mRNA	XR_001024230.1	lncRNA
XM_004017788.3	ZFANL3	mRNA	XR_001024230.1	lncRNA
XM_004017788.3	ZFANL3	mRNA	XR_001024230.1	lncRNA
XM_004017788.3	ZFANL3	mRNA	XR_001024230.1	lncRNA
XM_015103783.1	WDR7	mRNA	XR_001435726.1	lncRNA
XM_015103783.1	WDR7	mRNA	XR_001435726.1	lncRNA
XM_015103783.1	WDR7	mRNA	XR_001435726.1	lncRNA
XM_015103783.1	WDR7	mRNA	XR_001435726.1	lncRNA
XM_015103783.1	WDR7	mRNA	XR_001435726.1	lncRNA
XM_015103783.1	WDR7	mRNA	XR_001435726.1	lncRNA
XM_015103783.1	WDR7	mRNA	XR_001435726.1	lncRNA
XM_015103783.1	WDR7	mRNA	XR_001435726.1	lncRNA
XM_015096977.1	NIN	mRNA	XR_001435726.1	lncRNA
XM_015096977.1	NIN	mRNA	XR_001435726.1	lncRNA
XM_015096977.1	NIN	mRNA	XR_001435726.1	lncRNA
XM_015096977.1	NIN	mRNA	XR_001435726.1	lncRNA
XM_015096977.1	NIN	mRNA	XR_001435726.1	lncRNA
XM_015096977.1	NIN	mRNA	XR_001435726.1	lncRNA
XM_015096977.1	NIN	mRNA	XR_001435726.1	lncRNA
XM_015096977.1	NIN	mRNA	XR_001435726.1	lncRNA
XM_012180234.2	CNOT6L	mRNA	XR_001024230.1	lncRNA
XM_012180234.2	CNOT6L	mRNA	XR_001024230.1	lncRNA
XM_012180234.2	CNOT6L	mRNA	XR_001024230.1	lncRNA
XM_012180234.2	CNOT6L	mRNA	XR_001024230.1	lncRNA
XM_012180234.2	CNOT6L	mRNA	XR_001024230.1	lncRNA
XM_012180234.2	CNOT6L	mRNA	XR_001024230.1	lncRNA
XM_012180234.2	CNOT6L	mRNA	XR_001024230.1	lncRNA
XM_012180234.2	CNOT6L	mRNA	XR_001024230.1	lncRNA
XM_004003770.3	IMPG2	mRNA	XR_001435723.1	lncRNA
XM_004003770.3	IMPG2	mRNA	XR_001435723.1	lncRNA
XM_004003770.3	IMPG2	mRNA	XR_001435723.1	lncRNA
XM_004003770.3	IMPG2	mRNA	XR_001435723.1	lncRNA
XM_004003770.3	IMPG2	mRNA	XR_001435723.1	lncRNA
XM_004003770.3	IMPG2	mRNA	XR_001435723.1	lncRNA
XM_004003770.3	IMPG2	mRNA	XR_001435723.1	lncRNA
XM_004003770.3	IMPG2	mRNA	XR_001435723.1	lncRNA
XM_012182450.2	IBTK	mRNA	XR_001024230.1	lncRNA
XM_012182450.2	IBTK	mRNA	XR_001024230.1	lncRNA
XM_012182450.2	IBTK	mRNA	XR_001024230.1	lncRNA
XM_012182450.2	IBTK	mRNA	XR_001024230.1	lncRNA
XM_012182450.2	IBTK	mRNA	XR_001024230.1	lncRNA

XM_012182450.2	IBTK	mRNA	XR_001024230.1	lncRNA
XM_012182450.2	IBTK	mRNA	XR_001024230.1	lncRNA
XM_012182450.2	IBTK	mRNA	XR_001024230.1	lncRNA
XM_012097365.2	SH3D19	mRNA	XR_001436065.1	lncRNA
XM_012097365.2	SH3D19	mRNA	XR_001436065.1	lncRNA
XM_012097365.2	SH3D19	mRNA	XR_001436065.1	lncRNA
XM_012097365.2	SH3D19	mRNA	XR_001436065.1	lncRNA
XM_012097365.2	SH3D19	mRNA	XR_001436065.1	lncRNA
XM_012097365.2	SH3D19	mRNA	XR_001436065.1	lncRNA
XM_012097365.2	SH3D19	mRNA	XR_001436065.1	lncRNA
XM_012097365.2	SH3D19	mRNA	XR_001436065.1	lncRNA
XM_012185338.2	ZNF207	mRNA	XR_001436065.1	lncRNA
XM_012185338.2	ZNF207	mRNA	XR_001436065.1	lncRNA
XM_012185338.2	ZNF207	mRNA	XR_001436065.1	lncRNA
XM_012185338.2	ZNF207	mRNA	XR_001436065.1	lncRNA
XM_012185338.2	ZNF207	mRNA	XR_001436065.1	lncRNA
XM_012185338.2	ZNF207	mRNA	XR_001436065.1	lncRNA
XM_012185338.2	ZNF207	mRNA	XR_001436065.1	lncRNA
XM_012185338.2	ZNF207	mRNA	XR_001436065.1	lncRNA
XM_004008056.3	NRF1	mRNA	XR_001436065.1	lncRNA
XM_004008056.3	NRF1	mRNA	XR_001436065.1	lncRNA
XM_004008056.3	NRF1	mRNA	XR_001436065.1	lncRNA
XM_004008056.3	NRF1	mRNA	XR_001436065.1	lncRNA
XM_004008056.3	NRF1	mRNA	XR_001436065.1	lncRNA
XM_004008056.3	NRF1	mRNA	XR_001436065.1	lncRNA
XM_004008056.3	NRF1	mRNA	XR_001436065.1	lncRNA
XM_004008056.3	NRF1	mRNA	XR_001436065.1	lncRNA
XM_012097365.2	SH3D19	mRNA	XR_001436064.1	lncRNA
XM_012097365.2	SH3D19	mRNA	XR_001436064.1	lncRNA
XM_012097365.2	SH3D19	mRNA	XR_001436064.1	lncRNA
XM_012097365.2	SH3D19	mRNA	XR_001436064.1	lncRNA
XM_012097365.2	SH3D19	mRNA	XR_001436064.1	lncRNA
XM_012097365.2	SH3D19	mRNA	XR_001436064.1	lncRNA
XM_012097365.2	SH3D19	mRNA	XR_001436064.1	lncRNA
XM_012097365.2	SH3D19	mRNA	XR_001436064.1	lncRNA
XM_004017655.3	PRR14L	mRNA	XR_001435723.1	lncRNA
XM_004017655.3	PRR14L	mRNA	XR_001435723.1	lncRNA
XM_004017655.3	PRR14L	mRNA	XR_001435723.1	lncRNA
XM_004017655.3	PRR14L	mRNA	XR_001435723.1	lncRNA
XM_004017655.3	PRR14L	mRNA	XR_001435723.1	lncRNA
XM_004017655.3	PRR14L	mRNA	XR_001435723.1	lncRNA
XM_004017655.3	PRR14L	mRNA	XR_001435723.1	lncRNA
XM_004017655.3	PRR14L	mRNA	XR_001435723.1	lncRNA
XM_012185338.2	ZNF207	mRNA	XR_001436064.1	lncRNA
XM_012185338.2	ZNF207	mRNA	XR_001436064.1	lncRNA
XM_012185338.2	ZNF207	mRNA	XR_001436064.1	lncRNA
XM_012185338.2	ZNF207	mRNA	XR_001436064.1	lncRNA
XM_012185338.2	ZNF207	mRNA	XR_001436064.1	lncRNA
XM_012185338.2	ZNF207	mRNA	XR_001436064.1	lncRNA
XM_012185338.2	ZNF207	mRNA	XR_001436064.1	lncRNA
XM_012185338.2	ZNF207	mRNA	XR_001436064.1	lncRNA
XM_004017814.3	DNAJA4	mRNA	XR_001435722.1	lncRNA
XM_004017814.3	DNAJA4	mRNA	XR_001435722.1	lncRNA
XM_004017814.3	DNAJA4	mRNA	XR_001435722.1	lncRNA

XM_004017814.3	DNAJA4	mRNA	XR_001435722.1	lncRNA
XM_004017814.3	DNAJA4	mRNA	XR_001435722.1	lncRNA
XM_004017814.3	DNAJA4	mRNA	XR_001435722.1	lncRNA
XM_004017814.3	DNAJA4	mRNA	XR_001435722.1	lncRNA
XM_004017814.3	DNAJA4	mRNA	XR_001435722.1	lncRNA
XM_015095237.1	DMTF1	mRNA	XR_001435107.1	lncRNA
XM_015095237.1	DMTF1	mRNA	XR_001435107.1	lncRNA
XM_015095237.1	DMTF1	mRNA	XR_001435107.1	lncRNA
XM_015095237.1	DMTF1	mRNA	XR_001435107.1	lncRNA
XM_015095237.1	DMTF1	mRNA	XR_001435107.1	lncRNA
XM_015095237.1	DMTF1	mRNA	XR_001435107.1	lncRNA
XM_015095237.1	DMTF1	mRNA	XR_001435107.1	lncRNA
XM_015095237.1	DMTF1	mRNA	XR_001435107.1	lncRNA
XM_015096977.1	NIN	mRNA	XR_001435107.1	lncRNA
XM_015096977.1	NIN	mRNA	XR_001435107.1	lncRNA
XM_015096977.1	NIN	mRNA	XR_001435107.1	lncRNA
XM_015096977.1	NIN	mRNA	XR_001435107.1	lncRNA
XM_015096977.1	NIN	mRNA	XR_001435107.1	lncRNA
XM_015096977.1	NIN	mRNA	XR_001435107.1	lncRNA
XM_015096977.1	NIN	mRNA	XR_001435107.1	lncRNA
XM_015096977.1	NIN	mRNA	XR_001435107.1	lncRNA
XM_012182353.2	LRRFIP1	mRNA	XR_001436817.1	lncRNA
XM_012182353.2	LRRFIP1	mRNA	XR_001436817.1	lncRNA
XM_012182353.2	LRRFIP1	mRNA	XR_001436817.1	lncRNA
XM_012182353.2	LRRFIP1	mRNA	XR_001436817.1	lncRNA
XM_012182353.2	LRRFIP1	mRNA	XR_001436817.1	lncRNA
XM_012182353.2	LRRFIP1	mRNA	XR_001436817.1	lncRNA
XM_012182353.2	LRRFIP1	mRNA	XR_001436817.1	lncRNA
XM_012182353.2	LRRFIP1	mRNA	XR_001436817.1	lncRNA
XM_012186312.2	DDX42	mRNA	XR_001434920.1	lncRNA
XM_012186312.2	DDX42	mRNA	XR_001434920.1	lncRNA
XM_012186312.2	DDX42	mRNA	XR_001434920.1	lncRNA
XM_012186312.2	DDX42	mRNA	XR_001434920.1	lncRNA
XM_012186312.2	DDX42	mRNA	XR_001434920.1	lncRNA
XM_012186312.2	DDX42	mRNA	XR_001434920.1	lncRNA
XM_012186312.2	DDX42	mRNA	XR_001434920.1	lncRNA
XM_012186312.2	DDX42	mRNA	XR_001434920.1	lncRNA
XM_015094252.1	DNMT3A	mRNA	XR_001435722.1	lncRNA
XM_015094252.1	DNMT3A	mRNA	XR_001435722.1	lncRNA
XM_015094252.1	DNMT3A	mRNA	XR_001435722.1	lncRNA
XM_015094252.1	DNMT3A	mRNA	XR_001435722.1	lncRNA
XM_015094252.1	DNMT3A	mRNA	XR_001435722.1	lncRNA
XM_015094252.1	DNMT3A	mRNA	XR_001435722.1	lncRNA
XM_015094252.1	DNMT3A	mRNA	XR_001435722.1	lncRNA
XM_015094252.1	DNMT3A	mRNA	XR_001435722.1	lncRNA
XM_004017788.3	ZFANL3	mRNA	XR_001436817.1	lncRNA
XM_004017788.3	ZFANL3	mRNA	XR_001436817.1	lncRNA
XM_004017788.3	ZFANL3	mRNA	XR_001436817.1	lncRNA
XM_004017788.3	ZFANL3	mRNA	XR_001436817.1	lncRNA
XM_004017788.3	ZFANL3	mRNA	XR_001436817.1	lncRNA
XM_004017788.3	ZFANL3	mRNA	XR_001436817.1	lncRNA
XM_004017788.3	ZFANL3	mRNA	XR_001436817.1	lncRNA
XM_004017788.3	ZFANL3	mRNA	XR_001436817.1	lncRNA
XM_012182353.2	LRRFIP1	mRNA	XR_001434920.1	lncRNA

XM_012182353.2	LRRFIP1	mRNA	XR_001434920.1	lncRNA
XM_012182353.2	LRRFIP1	mRNA	XR_001434920.1	lncRNA
XM_012182353.2	LRRFIP1	mRNA	XR_001434920.1	lncRNA
XM_012182353.2	LRRFIP1	mRNA	XR_001434920.1	lncRNA
XM_012182353.2	LRRFIP1	mRNA	XR_001434920.1	lncRNA
XM_012182353.2	LRRFIP1	mRNA	XR_001434920.1	lncRNA
XM_012182353.2	LRRFIP1	mRNA	XR_001434920.1	lncRNA
XM_012187161.1	CDC42BPA	mRNA	XR_001434920.1	lncRNA
XM_012187161.1	CDC42BPA	mRNA	XR_001434920.1	lncRNA
XM_012187161.1	CDC42BPA	mRNA	XR_001434920.1	lncRNA
XM_012187161.1	CDC42BPA	mRNA	XR_001434920.1	lncRNA
XM_012187161.1	CDC42BPA	mRNA	XR_001434920.1	lncRNA
XM_012187161.1	CDC42BPA	mRNA	XR_001434920.1	lncRNA
XM_012187161.1	CDC42BPA	mRNA	XR_001434920.1	lncRNA
XM_012187161.1	CDC42BPA	mRNA	XR_001434920.1	lncRNA
XM_012187413.1	NRIP1	mRNA	XR_001435107.1	lncRNA
XM_012187413.1	NRIP1	mRNA	XR_001435107.1	lncRNA
XM_012187413.1	NRIP1	mRNA	XR_001435107.1	lncRNA
XM_012187413.1	NRIP1	mRNA	XR_001435107.1	lncRNA
XM_012187413.1	NRIP1	mRNA	XR_001435107.1	lncRNA
XM_012187413.1	NRIP1	mRNA	XR_001435107.1	lncRNA
XM_012187413.1	NRIP1	mRNA	XR_001435107.1	lncRNA
XM_012187413.1	NRIP1	mRNA	XR_001435107.1	lncRNA
XM_012187161.1	CDC42BPA	mRNA	XR_001435107.1	lncRNA
XM_012187161.1	CDC42BPA	mRNA	XR_001435107.1	lncRNA
XM_012187161.1	CDC42BPA	mRNA	XR_001435107.1	lncRNA
XM_012187161.1	CDC42BPA	mRNA	XR_001435107.1	lncRNA
XM_012187161.1	CDC42BPA	mRNA	XR_001435107.1	lncRNA
XM_012187161.1	CDC42BPA	mRNA	XR_001435107.1	lncRNA
XM_012187161.1	CDC42BPA	mRNA	XR_001435107.1	lncRNA
XM_012187161.1	CDC42BPA	mRNA	XR_001435107.1	lncRNA
XM_015094252.1	DNMT3A	mRNA	XR_001433856.1	lncRNA
XM_015094252.1	DNMT3A	mRNA	XR_001433856.1	lncRNA
XM_015094252.1	DNMT3A	mRNA	XR_001433856.1	lncRNA
XM_015094252.1	DNMT3A	mRNA	XR_001433856.1	lncRNA
XM_015094252.1	DNMT3A	mRNA	XR_001433856.1	lncRNA
XM_015094252.1	DNMT3A	mRNA	XR_001433856.1	lncRNA
XM_015094252.1	DNMT3A	mRNA	XR_001433856.1	lncRNA
XM_015094252.1	DNMT3A	mRNA	XR_001433856.1	lncRNA
XM_012186312.2	DDX42	mRNA	XR_001436817.1	lncRNA
XM_012186312.2	DDX42	mRNA	XR_001436817.1	lncRNA
XM_012186312.2	DDX42	mRNA	XR_001436817.1	lncRNA
XM_012186312.2	DDX42	mRNA	XR_001436817.1	lncRNA
XM_012186312.2	DDX42	mRNA	XR_001436817.1	lncRNA
XM_012186312.2	DDX42	mRNA	XR_001436817.1	lncRNA
XM_012186312.2	DDX42	mRNA	XR_001436817.1	lncRNA
XM_012186312.2	DDX42	mRNA	XR_001436817.1	lncRNA
XM_015096977.1	NIN	mRNA	XR_001433856.1	lncRNA
XM_015096977.1	NIN	mRNA	XR_001433856.1	lncRNA
XM_015096977.1	NIN	mRNA	XR_001433856.1	lncRNA
XM_015096977.1	NIN	mRNA	XR_001433856.1	lncRNA
XM_015096977.1	NIN	mRNA	XR_001433856.1	lncRNA
XM_015096977.1	NIN	mRNA	XR_001433856.1	lncRNA
XM_015096977.1	NIN	mRNA	XR_001433856.1	lncRNA

XM_015096977.1	NIN	mRNA	XR_001433856.1	lncRNA
XM_015095237.1	DMTF1	mRNA	XR_001435722.1	lncRNA
XM_015095237.1	DMTF1	mRNA	XR_001435722.1	lncRNA
XM_015095237.1	DMTF1	mRNA	XR_001435722.1	lncRNA
XM_015095237.1	DMTF1	mRNA	XR_001435722.1	lncRNA
XM_015095237.1	DMTF1	mRNA	XR_001435722.1	lncRNA
XM_015095237.1	DMTF1	mRNA	XR_001435722.1	lncRNA
XM_015095237.1	DMTF1	mRNA	XR_001435722.1	lncRNA
XM_015095237.1	DMTF1	mRNA	XR_001435722.1	lncRNA
XM_012098812.2	RALGAPA1	mRNA	XR_001433856.1	lncRNA
XM_012098812.2	RALGAPA1	mRNA	XR_001433856.1	lncRNA
XM_012098812.2	RALGAPA1	mRNA	XR_001433856.1	lncRNA
XM_012098812.2	RALGAPA1	mRNA	XR_001433856.1	lncRNA
XM_012098812.2	RALGAPA1	mRNA	XR_001433856.1	lncRNA
XM_012098812.2	RALGAPA1	mRNA	XR_001433856.1	lncRNA
XM_012098812.2	RALGAPA1	mRNA	XR_001433856.1	lncRNA
XM_012098812.2	RALGAPA1	mRNA	XR_001433856.1	lncRNA
XM_012185338.2	ZNF207	mRNA	XR_001435722.1	lncRNA
XM_012185338.2	ZNF207	mRNA	XR_001435722.1	lncRNA
XM_012185338.2	ZNF207	mRNA	XR_001435722.1	lncRNA
XM_012185338.2	ZNF207	mRNA	XR_001435722.1	lncRNA
XM_012185338.2	ZNF207	mRNA	XR_001435722.1	lncRNA
XM_012185338.2	ZNF207	mRNA	XR_001435722.1	lncRNA
XM_012185338.2	ZNF207	mRNA	XR_001435722.1	lncRNA
XM_012185338.2	ZNF207	mRNA	XR_001435722.1	lncRNA
XM_012187161.1	CDC42BPA	mRNA	XR_001435722.1	lncRNA
XM_012187161.1	CDC42BPA	mRNA	XR_001435722.1	lncRNA
XM_012187161.1	CDC42BPA	mRNA	XR_001435722.1	lncRNA
XM_012187161.1	CDC42BPA	mRNA	XR_001435722.1	lncRNA
XM_012187161.1	CDC42BPA	mRNA	XR_001435722.1	lncRNA
XM_012187161.1	CDC42BPA	mRNA	XR_001435722.1	lncRNA
XM_012187161.1	CDC42BPA	mRNA	XR_001435722.1	lncRNA
XM_012187161.1	CDC42BPA	mRNA	XR_001435722.1	lncRNA
XM_015094252.1	DNMT3A	mRNA	XR_001434920.1	lncRNA
XM_015094252.1	DNMT3A	mRNA	XR_001434920.1	lncRNA
XM_015094252.1	DNMT3A	mRNA	XR_001434920.1	lncRNA
XM_015094252.1	DNMT3A	mRNA	XR_001434920.1	lncRNA
XM_015094252.1	DNMT3A	mRNA	XR_001434920.1	lncRNA
XM_015094252.1	DNMT3A	mRNA	XR_001434920.1	lncRNA
XM_015094252.1	DNMT3A	mRNA	XR_001434920.1	lncRNA
XM_015094252.1	DNMT3A	mRNA	XR_001434920.1	lncRNA
XM_012187084.2	MIA3	mRNA	XR_001433856.1	lncRNA
XM_012187084.2	MIA3	mRNA	XR_001433856.1	lncRNA
XM_012187084.2	MIA3	mRNA	XR_001433856.1	lncRNA
XM_012187084.2	MIA3	mRNA	XR_001433856.1	lncRNA
XM_012187084.2	MIA3	mRNA	XR_001433856.1	lncRNA
XM_012187084.2	MIA3	mRNA	XR_001433856.1	lncRNA
XM_012187084.2	MIA3	mRNA	XR_001433856.1	lncRNA
XM_012187084.2	MIA3	mRNA	XR_001433856.1	lncRNA
XM_015094252.1	DNMT3A	mRNA	XR_001436817.1	lncRNA
XM_015094252.1	DNMT3A	mRNA	XR_001436817.1	lncRNA
XM_015094252.1	DNMT3A	mRNA	XR_001436817.1	lncRNA
XM_015094252.1	DNMT3A	mRNA	XR_001436817.1	lncRNA
XM_015094252.1	DNMT3A	mRNA	XR_001436817.1	lncRNA

XM_015094252.1	DNMT3A	mRNA	XR_001436817.1	lncRNA
XM_015094252.1	DNMT3A	mRNA	XR_001436817.1	lncRNA
XM_015094252.1	DNMT3A	mRNA	XR_001436817.1	lncRNA
XM_004017655.3	PRR14L	mRNA	XR_001435107.1	lncRNA
XM_004017655.3	PRR14L	mRNA	XR_001435107.1	lncRNA
XM_004017655.3	PRR14L	mRNA	XR_001435107.1	lncRNA
XM_004017655.3	PRR14L	mRNA	XR_001435107.1	lncRNA
XM_004017655.3	PRR14L	mRNA	XR_001435107.1	lncRNA
XM_004017655.3	PRR14L	mRNA	XR_001435107.1	lncRNA
XM_004017655.3	PRR14L	mRNA	XR_001435107.1	lncRNA
XM_004017655.3	PRR14L	mRNA	XR_001435107.1	lncRNA
XM_012095418.2	ZMYM4	mRNA	XR_001434920.1	lncRNA
XM_012095418.2	ZMYM4	mRNA	XR_001434920.1	lncRNA
XM_012095418.2	ZMYM4	mRNA	XR_001434920.1	lncRNA
XM_012095418.2	ZMYM4	mRNA	XR_001434920.1	lncRNA
XM_012095418.2	ZMYM4	mRNA	XR_001434920.1	lncRNA
XM_012095418.2	ZMYM4	mRNA	XR_001434920.1	lncRNA
XM_012095418.2	ZMYM4	mRNA	XR_001434920.1	lncRNA
XM_012095418.2	ZMYM4	mRNA	XR_001434920.1	lncRNA
XM_015095237.1	DMTF1	mRNA	XR_001436817.1	lncRNA
XM_015095237.1	DMTF1	mRNA	XR_001436817.1	lncRNA
XM_015095237.1	DMTF1	mRNA	XR_001436817.1	lncRNA
XM_015095237.1	DMTF1	mRNA	XR_001436817.1	lncRNA
XM_015095237.1	DMTF1	mRNA	XR_001436817.1	lncRNA
XM_015095237.1	DMTF1	mRNA	XR_001436817.1	lncRNA
XM_015095237.1	DMTF1	mRNA	XR_001436817.1	lncRNA
XM_015095237.1	DMTF1	mRNA	XR_001436817.1	lncRNA
XM_015096977.1	NIN	mRNA	XR_001435722.1	lncRNA
XM_015096977.1	NIN	mRNA	XR_001435722.1	lncRNA
XM_015096977.1	NIN	mRNA	XR_001435722.1	lncRNA
XM_015096977.1	NIN	mRNA	XR_001435722.1	lncRNA
XM_015096977.1	NIN	mRNA	XR_001435722.1	lncRNA
XM_015096977.1	NIN	mRNA	XR_001435722.1	lncRNA
XM_015096977.1	NIN	mRNA	XR_001435722.1	lncRNA
XM_015096977.1	NIN	mRNA	XR_001435722.1	lncRNA
XM_015096977.1	NIN	mRNA	XR_001436817.1	lncRNA
XM_015096977.1	NIN	mRNA	XR_001436817.1	lncRNA
XM_015096977.1	NIN	mRNA	XR_001436817.1	lncRNA
XM_015096977.1	NIN	mRNA	XR_001436817.1	lncRNA
XM_015096977.1	NIN	mRNA	XR_001436817.1	lncRNA
XM_015096977.1	NIN	mRNA	XR_001436817.1	lncRNA
XM_015096977.1	NIN	mRNA	XR_001436817.1	lncRNA
XM_015096977.1	NIN	mRNA	XR_001436817.1	lncRNA
XM_012187413.1	NRIP1	mRNA	XR_001435722.1	lncRNA
XM_012187413.1	NRIP1	mRNA	XR_001435722.1	lncRNA
XM_012187413.1	NRIP1	mRNA	XR_001435722.1	lncRNA
XM_012187413.1	NRIP1	mRNA	XR_001435722.1	lncRNA
XM_012187413.1	NRIP1	mRNA	XR_001435722.1	lncRNA
XM_012187413.1	NRIP1	mRNA	XR_001435722.1	lncRNA
XM_012187413.1	NRIP1	mRNA	XR_001435722.1	lncRNA
XM_012187413.1	NRIP1	mRNA	XR_001435722.1	lncRNA
XM_012125769.2	MPDZ	mRNA	XR_001435107.1	lncRNA
XM_012125769.2	MPDZ	mRNA	XR_001435107.1	lncRNA
XM_012125769.2	MPDZ	mRNA	XR_001435107.1	lncRNA

XM_012125769.2	MPDZ	mRNA	XR_001435107.1	lncRNA
XM_012125769.2	MPDZ	mRNA	XR_001435107.1	lncRNA
XM_012125769.2	MPDZ	mRNA	XR_001435107.1	lncRNA
XM_012125769.2	MPDZ	mRNA	XR_001435107.1	lncRNA
XM_012125769.2	MPDZ	mRNA	XR_001435107.1	lncRNA
XM_012186312.2	DDX42	mRNA	XR_001433856.1	lncRNA
XM_012186312.2	DDX42	mRNA	XR_001433856.1	lncRNA
XM_012186312.2	DDX42	mRNA	XR_001433856.1	lncRNA
XM_012186312.2	DDX42	mRNA	XR_001433856.1	lncRNA
XM_012186312.2	DDX42	mRNA	XR_001433856.1	lncRNA
XM_012186312.2	DDX42	mRNA	XR_001433856.1	lncRNA
XM_012186312.2	DDX42	mRNA	XR_001433856.1	lncRNA
XM_012186312.2	DDX42	mRNA	XR_001433856.1	lncRNA
XM_015103466.1	TACC2	mRNA	XR_001435107.1	lncRNA
XM_015103466.1	TACC2	mRNA	XR_001435107.1	lncRNA
XM_015103466.1	TACC2	mRNA	XR_001435107.1	lncRNA
XM_015103466.1	TACC2	mRNA	XR_001435107.1	lncRNA
XM_015103466.1	TACC2	mRNA	XR_001435107.1	lncRNA
XM_015103466.1	TACC2	mRNA	XR_001435107.1	lncRNA
XM_015103466.1	TACC2	mRNA	XR_001435107.1	lncRNA
XM_015103466.1	TACC2	mRNA	XR_001435107.1	lncRNA
XM_012186312.2	DDX42	mRNA	XR_001435722.1	lncRNA
XM_012186312.2	DDX42	mRNA	XR_001435722.1	lncRNA
XM_012186312.2	DDX42	mRNA	XR_001435722.1	lncRNA
XM_012186312.2	DDX42	mRNA	XR_001435722.1	lncRNA
XM_012186312.2	DDX42	mRNA	XR_001435722.1	lncRNA
XM_012186312.2	DDX42	mRNA	XR_001435722.1	lncRNA
XM_012186312.2	DDX42	mRNA	XR_001435722.1	lncRNA
XM_012186312.2	DDX42	mRNA	XR_001435722.1	lncRNA
XM_012098812.2	RALGAPA1	mRNA	XR_001435107.1	lncRNA
XM_012098812.2	RALGAPA1	mRNA	XR_001435107.1	lncRNA
XM_012098812.2	RALGAPA1	mRNA	XR_001435107.1	lncRNA
XM_012098812.2	RALGAPA1	mRNA	XR_001435107.1	lncRNA
XM_012098812.2	RALGAPA1	mRNA	XR_001435107.1	lncRNA
XM_012098812.2	RALGAPA1	mRNA	XR_001435107.1	lncRNA
XM_012098812.2	RALGAPA1	mRNA	XR_001435107.1	lncRNA
XM_012098812.2	RALGAPA1	mRNA	XR_001435107.1	lncRNA
XM_004003770.3	IMPG2	mRNA	XR_001436817.1	lncRNA
XM_004003770.3	IMPG2	mRNA	XR_001436817.1	lncRNA
XM_004003770.3	IMPG2	mRNA	XR_001436817.1	lncRNA
XM_004003770.3	IMPG2	mRNA	XR_001436817.1	lncRNA
XM_004003770.3	IMPG2	mRNA	XR_001436817.1	lncRNA
XM_004003770.3	IMPG2	mRNA	XR_001436817.1	lncRNA
XM_004003770.3	IMPG2	mRNA	XR_001436817.1	lncRNA
XM_004003770.3	IMPG2	mRNA	XR_001436817.1	lncRNA
XM_012173553.2	STRN	mRNA	XR_001435107.1	lncRNA
XM_012173553.2	STRN	mRNA	XR_001435107.1	lncRNA
XM_012173553.2	STRN	mRNA	XR_001435107.1	lncRNA
XM_012173553.2	STRN	mRNA	XR_001435107.1	lncRNA
XM_012173553.2	STRN	mRNA	XR_001435107.1	lncRNA
XM_012173553.2	STRN	mRNA	XR_001435107.1	lncRNA
XM_012173553.2	STRN	mRNA	XR_001435107.1	lncRNA
XM_012173553.2	STRN	mRNA	XR_001435107.1	lncRNA
XM_015096977.1	NIN	mRNA	XR_001434920.1	lncRNA

XM_015096977.1	NIN	mRNA	XR_001434920.1	lncRNA
XM_015096977.1	NIN	mRNA	XR_001434920.1	lncRNA
XM_015096977.1	NIN	mRNA	XR_001434920.1	lncRNA
XM_015096977.1	NIN	mRNA	XR_001434920.1	lncRNA
XM_015096977.1	NIN	mRNA	XR_001434920.1	lncRNA
XM_015096977.1	NIN	mRNA	XR_001434920.1	lncRNA
XM_015096977.1	NIN	mRNA	XR_001434920.1	lncRNA
XM_004010552.3	VPS13C	mRNA	XR_001436390.1	lncRNA
XM_004010552.3	VPS13C	mRNA	XR_001436390.1	lncRNA
XM_004010552.3	VPS13C	mRNA	XR_001436390.1	lncRNA
XM_004010552.3	VPS13C	mRNA	XR_001436390.1	lncRNA
XM_004010552.3	VPS13C	mRNA	XR_001436390.1	lncRNA
XM_004010552.3	VPS13C	mRNA	XR_001436390.1	lncRNA
XM_004010552.3	VPS13C	mRNA	XR_001436390.1	lncRNA
XM_004010552.3	VPS13C	mRNA	XR_001436390.1	lncRNA
XM_015099739.1	LOC101122805	mRNA	XR_001040147.1	lncRNA
XM_015099739.1	LOC101122805	mRNA	XR_001040147.1	lncRNA
XM_015099739.1	LOC101122805	mRNA	XR_001040147.1	lncRNA
XM_015099739.1	LOC101122805	mRNA	XR_001040147.1	lncRNA
XM_015099739.1	LOC101122805	mRNA	XR_001040147.1	lncRNA
XM_015099739.1	LOC101122805	mRNA	XR_001040147.1	lncRNA
XM_015099739.1	LOC101122805	mRNA	XR_001040147.1	lncRNA
XM_015099739.1	LOC101122805	mRNA	XR_001040147.1	lncRNA
XM_012186362.1	PITPNC1	mRNA	XR_001023627.2	lncRNA
XM_012186362.1	PITPNC1	mRNA	XR_001023627.2	lncRNA
XM_012186362.1	PITPNC1	mRNA	XR_001023627.2	lncRNA
XM_012186362.1	PITPNC1	mRNA	XR_001023627.2	lncRNA
XM_012186362.1	PITPNC1	mRNA	XR_001023627.2	lncRNA
XM_012186362.1	PITPNC1	mRNA	XR_001023627.2	lncRNA
XM_012186362.1	PITPNC1	mRNA	XR_001023627.2	lncRNA
XM_012186362.1	PITPNC1	mRNA	XR_001023627.2	lncRNA
XM_012143523.2	INO80D	mRNA	XR_001434127.1	lncRNA
XM_012143523.2	INO80D	mRNA	XR_001434127.1	lncRNA
XM_012143523.2	INO80D	mRNA	XR_001434127.1	lncRNA
XM_012143523.2	INO80D	mRNA	XR_001434127.1	lncRNA
XM_012143523.2	INO80D	mRNA	XR_001434127.1	lncRNA
XM_012143523.2	INO80D	mRNA	XR_001434127.1	lncRNA
XM_012143523.2	INO80D	mRNA	XR_001434127.1	lncRNA
XM_012143523.2	INO80D	mRNA	XR_001434127.1	lncRNA
XM_012184885.2	DOCK9	mRNA	XR_001435723.1	lncRNA
XM_012184885.2	DOCK9	mRNA	XR_001435723.1	lncRNA
XM_012184885.2	DOCK9	mRNA	XR_001435723.1	lncRNA
XM_012184885.2	DOCK9	mRNA	XR_001435723.1	lncRNA
XM_012184885.2	DOCK9	mRNA	XR_001435723.1	lncRNA
XM_012184885.2	DOCK9	mRNA	XR_001435723.1	lncRNA
XM_012184885.2	DOCK9	mRNA	XR_001435723.1	lncRNA
XM_012184885.2	DOCK9	mRNA	XR_001435723.1	lncRNA
XM_015092364.1	GART	mRNA	XR_001435726.1	lncRNA
XM_015092364.1	GART	mRNA	XR_001435726.1	lncRNA
XM_015092364.1	GART	mRNA	XR_001435726.1	lncRNA
XM_015092364.1	GART	mRNA	XR_001435726.1	lncRNA
XM_015092364.1	GART	mRNA	XR_001435726.1	lncRNA
XM_015092364.1	GART	mRNA	XR_001435726.1	lncRNA
XM_015092364.1	GART	mRNA	XR_001435726.1	lncRNA

XM_015092364.1	GART	mRNA	XR_001435726.1	lncRNA
XM_015105074.1	LONRF3	mRNA	XR_001436065.1	lncRNA
XM_015105074.1	LONRF3	mRNA	XR_001436065.1	lncRNA
XM_015105074.1	LONRF3	mRNA	XR_001436065.1	lncRNA
XM_015105074.1	LONRF3	mRNA	XR_001436065.1	lncRNA
XM_015105074.1	LONRF3	mRNA	XR_001436065.1	lncRNA
XM_015105074.1	LONRF3	mRNA	XR_001436065.1	lncRNA
XM_015105074.1	LONRF3	mRNA	XR_001436065.1	lncRNA
XM_015105074.1	LONRF3	mRNA	XR_001436065.1	lncRNA
XM_004009738.3	PPARGC1A	mRNA	XR_001435723.1	lncRNA
XM_004009738.3	PPARGC1A	mRNA	XR_001435723.1	lncRNA
XM_004009738.3	PPARGC1A	mRNA	XR_001435723.1	lncRNA
XM_004009738.3	PPARGC1A	mRNA	XR_001435723.1	lncRNA
XM_004009738.3	PPARGC1A	mRNA	XR_001435723.1	lncRNA
XM_004009738.3	PPARGC1A	mRNA	XR_001435723.1	lncRNA
XM_004009738.3	PPARGC1A	mRNA	XR_001435723.1	lncRNA
XM_004009738.3	PPARGC1A	mRNA	XR_001435723.1	lncRNA
XM_012184885.2	DOCK9	mRNA	XR_001436065.1	lncRNA
XM_012184885.2	DOCK9	mRNA	XR_001436065.1	lncRNA
XM_012184885.2	DOCK9	mRNA	XR_001436065.1	lncRNA
XM_012184885.2	DOCK9	mRNA	XR_001436065.1	lncRNA
XM_012184885.2	DOCK9	mRNA	XR_001436065.1	lncRNA
XM_012184885.2	DOCK9	mRNA	XR_001436065.1	lncRNA
XM_012184885.2	DOCK9	mRNA	XR_001436065.1	lncRNA
XM_012184885.2	DOCK9	mRNA	XR_001436065.1	lncRNA
XM_012097035.1	C16H5orf42	mRNA	XR_001435723.1	lncRNA
XM_012097035.1	C16H5orf42	mRNA	XR_001435723.1	lncRNA
XM_012097035.1	C16H5orf42	mRNA	XR_001435723.1	lncRNA
XM_012097035.1	C16H5orf42	mRNA	XR_001435723.1	lncRNA
XM_012097035.1	C16H5orf42	mRNA	XR_001435723.1	lncRNA
XM_012097035.1	C16H5orf42	mRNA	XR_001435723.1	lncRNA
XM_012097035.1	C16H5orf42	mRNA	XR_001435723.1	lncRNA
XM_012097035.1	C16H5orf42	mRNA	XR_001435723.1	lncRNA
XM_015093065.1	FAM219A	mRNA	XR_001436064.1	lncRNA
XM_015093065.1	FAM219A	mRNA	XR_001436064.1	lncRNA
XM_015093065.1	FAM219A	mRNA	XR_001436064.1	lncRNA
XM_015093065.1	FAM219A	mRNA	XR_001436064.1	lncRNA
XM_015093065.1	FAM219A	mRNA	XR_001436064.1	lncRNA
XM_015093065.1	FAM219A	mRNA	XR_001436064.1	lncRNA
XM_015093065.1	FAM219A	mRNA	XR_001436064.1	lncRNA
XM_015093065.1	FAM219A	mRNA	XR_001436064.1	lncRNA
XM_012179676.2	CCSER1	mRNA	XR_001436065.1	lncRNA
XM_012179676.2	CCSER1	mRNA	XR_001436065.1	lncRNA
XM_012179676.2	CCSER1	mRNA	XR_001436065.1	lncRNA
XM_012179676.2	CCSER1	mRNA	XR_001436065.1	lncRNA
XM_012179676.2	CCSER1	mRNA	XR_001436065.1	lncRNA
XM_012179676.2	CCSER1	mRNA	XR_001436065.1	lncRNA
XM_012179676.2	CCSER1	mRNA	XR_001436065.1	lncRNA
XM_012179676.2	CCSER1	mRNA	XR_001436065.1	lncRNA
XM_012179676.2	CCSER1	mRNA	XR_001435723.1	lncRNA
XM_012179676.2	CCSER1	mRNA	XR_001435723.1	lncRNA
XM_012179676.2	CCSER1	mRNA	XR_001435723.1	lncRNA
XM_012179676.2	CCSER1	mRNA	XR_001435723.1	lncRNA
XM_012179676.2	CCSER1	mRNA	XR_001435723.1	lncRNA

XM_012179676.2	CCSER1	mRNA	XR_001435723.1	lncRNA
XM_012179676.2	CCSER1	mRNA	XR_001435723.1	lncRNA
XM_012179676.2	CCSER1	mRNA	XR_001435723.1	lncRNA
XM_015093065.1	FAM219A	mRNA	XR_001436065.1	lncRNA
XM_015093065.1	FAM219A	mRNA	XR_001436065.1	lncRNA
XM_015093065.1	FAM219A	mRNA	XR_001436065.1	lncRNA
XM_015093065.1	FAM219A	mRNA	XR_001436065.1	lncRNA
XM_015093065.1	FAM219A	mRNA	XR_001436065.1	lncRNA
XM_015093065.1	FAM219A	mRNA	XR_001436065.1	lncRNA
XM_015093065.1	FAM219A	mRNA	XR_001436065.1	lncRNA
XM_015093065.1	FAM219A	mRNA	XR_001436065.1	lncRNA
XM_015094225.1	ROCK2	mRNA	XR_001435723.1	lncRNA
XM_015094225.1	ROCK2	mRNA	XR_001435723.1	lncRNA
XM_015094225.1	ROCK2	mRNA	XR_001435723.1	lncRNA
XM_015094225.1	ROCK2	mRNA	XR_001435723.1	lncRNA
XM_015094225.1	ROCK2	mRNA	XR_001435723.1	lncRNA
XM_015094225.1	ROCK2	mRNA	XR_001435723.1	lncRNA
XM_015094225.1	ROCK2	mRNA	XR_001435723.1	lncRNA
XM_015094225.1	ROCK2	mRNA	XR_001435723.1	lncRNA
XM_015105074.1	LONRF3	mRNA	XR_001436064.1	lncRNA
XM_015105074.1	LONRF3	mRNA	XR_001436064.1	lncRNA
XM_015105074.1	LONRF3	mRNA	XR_001436064.1	lncRNA
XM_015105074.1	LONRF3	mRNA	XR_001436064.1	lncRNA
XM_015105074.1	LONRF3	mRNA	XR_001436064.1	lncRNA
XM_015105074.1	LONRF3	mRNA	XR_001436064.1	lncRNA
XM_015105074.1	LONRF3	mRNA	XR_001436064.1	lncRNA
XM_015105074.1	LONRF3	mRNA	XR_001436064.1	lncRNA
XM_015105074.1	LONRF3	mRNA	XR_001435723.1	lncRNA
XM_015105074.1	LONRF3	mRNA	XR_001435723.1	lncRNA
XM_015105074.1	LONRF3	mRNA	XR_001435723.1	lncRNA
XM_015105074.1	LONRF3	mRNA	XR_001435723.1	lncRNA
XM_015105074.1	LONRF3	mRNA	XR_001435723.1	lncRNA
XM_015105074.1	LONRF3	mRNA	XR_001435723.1	lncRNA
XM_015105074.1	LONRF3	mRNA	XR_001435723.1	lncRNA
XM_015105074.1	LONRF3	mRNA	XR_001435723.1	lncRNA
XM_015101001.1	RRAS2	mRNA	XR_001024230.1	lncRNA
XM_015101001.1	RRAS2	mRNA	XR_001024230.1	lncRNA
XM_015101001.1	RRAS2	mRNA	XR_001024230.1	lncRNA
XM_015101001.1	RRAS2	mRNA	XR_001024230.1	lncRNA
XM_015101001.1	RRAS2	mRNA	XR_001024230.1	lncRNA
XM_015101001.1	RRAS2	mRNA	XR_001024230.1	lncRNA
XM_015101001.1	RRAS2	mRNA	XR_001024230.1	lncRNA
XM_015101001.1	RRAS2	mRNA	XR_001024230.1	lncRNA
XM_012184362.2	AKAP11	mRNA	XR_001435723.1	lncRNA
XM_012184362.2	AKAP11	mRNA	XR_001435723.1	lncRNA
XM_012184362.2	AKAP11	mRNA	XR_001435723.1	lncRNA
XM_012184362.2	AKAP11	mRNA	XR_001435723.1	lncRNA
XM_012184362.2	AKAP11	mRNA	XR_001435723.1	lncRNA
XM_012184362.2	AKAP11	mRNA	XR_001435723.1	lncRNA
XM_012184362.2	AKAP11	mRNA	XR_001435723.1	lncRNA
XM_012184362.2	AKAP11	mRNA	XR_001435723.1	lncRNA
XM_012174704.2	ZBTB39	mRNA	XR_001435726.1	lncRNA
XM_012174704.2	ZBTB39	mRNA	XR_001435726.1	lncRNA
XM_012174704.2	ZBTB39	mRNA	XR_001435726.1	lncRNA

XM_012174704.2	ZBTB39	mRNA	XR_001435726.1	lncRNA
XM_012174704.2	ZBTB39	mRNA	XR_001435726.1	lncRNA
XM_012174704.2	ZBTB39	mRNA	XR_001435726.1	lncRNA
XM_012174704.2	ZBTB39	mRNA	XR_001435726.1	lncRNA
XM_012174704.2	ZBTB39	mRNA	XR_001435726.1	lncRNA
XM_012179676.2	CCSER1	mRNA	XR_001436064.1	lncRNA
XM_012179676.2	CCSER1	mRNA	XR_001436064.1	lncRNA
XM_012179676.2	CCSER1	mRNA	XR_001436064.1	lncRNA
XM_012179676.2	CCSER1	mRNA	XR_001436064.1	lncRNA
XM_012179676.2	CCSER1	mRNA	XR_001436064.1	lncRNA
XM_012179676.2	CCSER1	mRNA	XR_001436064.1	lncRNA
XM_012179676.2	CCSER1	mRNA	XR_001436064.1	lncRNA
XM_012179676.2	CCSER1	mRNA	XR_001436064.1	lncRNA
XM_004009738.3	PPARGC1A	mRNA	XR_001435726.1	lncRNA
XM_004009738.3	PPARGC1A	mRNA	XR_001435726.1	lncRNA
XM_004009738.3	PPARGC1A	mRNA	XR_001435726.1	lncRNA
XM_004009738.3	PPARGC1A	mRNA	XR_001435726.1	lncRNA
XM_004009738.3	PPARGC1A	mRNA	XR_001435726.1	lncRNA
XM_004009738.3	PPARGC1A	mRNA	XR_001435726.1	lncRNA
XM_004009738.3	PPARGC1A	mRNA	XR_001435726.1	lncRNA
XM_004009738.3	PPARGC1A	mRNA	XR_001435726.1	lncRNA
XM_015098082.1	RNASEH2B	mRNA	XR_001435726.1	lncRNA
XM_015098082.1	RNASEH2B	mRNA	XR_001435726.1	lncRNA
XM_015098082.1	RNASEH2B	mRNA	XR_001435726.1	lncRNA
XM_015098082.1	RNASEH2B	mRNA	XR_001435726.1	lncRNA
XM_015098082.1	RNASEH2B	mRNA	XR_001435726.1	lncRNA
XM_015098082.1	RNASEH2B	mRNA	XR_001435726.1	lncRNA
XM_015098082.1	RNASEH2B	mRNA	XR_001435726.1	lncRNA
XM_015098082.1	RNASEH2B	mRNA	XR_001435726.1	lncRNA
XM_015101001.1	RRAS2	mRNA	XR_001436064.1	lncRNA
XM_015101001.1	RRAS2	mRNA	XR_001436064.1	lncRNA
XM_015101001.1	RRAS2	mRNA	XR_001436064.1	lncRNA
XM_015101001.1	RRAS2	mRNA	XR_001436064.1	lncRNA
XM_015101001.1	RRAS2	mRNA	XR_001436064.1	lncRNA
XM_015101001.1	RRAS2	mRNA	XR_001436064.1	lncRNA
XM_015101001.1	RRAS2	mRNA	XR_001436064.1	lncRNA
XM_015101001.1	RRAS2	mRNA	XR_001436064.1	lncRNA
XM_012179825.2	N4BP2	mRNA	XR_001435723.1	lncRNA
XM_012179825.2	N4BP2	mRNA	XR_001435723.1	lncRNA
XM_012179825.2	N4BP2	mRNA	XR_001435723.1	lncRNA
XM_012179825.2	N4BP2	mRNA	XR_001435723.1	lncRNA
XM_012179825.2	N4BP2	mRNA	XR_001435723.1	lncRNA
XM_012179825.2	N4BP2	mRNA	XR_001435723.1	lncRNA
XM_012179825.2	N4BP2	mRNA	XR_001435723.1	lncRNA
XM_012179825.2	N4BP2	mRNA	XR_001435723.1	lncRNA
XM_012184885.2	DOCK9	mRNA	XR_001436064.1	lncRNA
XM_012184885.2	DOCK9	mRNA	XR_001436064.1	lncRNA
XM_012184885.2	DOCK9	mRNA	XR_001436064.1	lncRNA
XM_012184885.2	DOCK9	mRNA	XR_001436064.1	lncRNA
XM_012184885.2	DOCK9	mRNA	XR_001436064.1	lncRNA
XM_012184885.2	DOCK9	mRNA	XR_001436064.1	lncRNA
XM_012184885.2	DOCK9	mRNA	XR_001436064.1	lncRNA
XM_012184885.2	DOCK9	mRNA	XR_001436064.1	lncRNA
XM_015092364.1	GART	mRNA	XR_001435723.1	lncRNA

XM_015092364.1	GART	mRNA	XR_001435723.1	lncRNA
XM_015092364.1	GART	mRNA	XR_001435723.1	lncRNA
XM_015092364.1	GART	mRNA	XR_001435723.1	lncRNA
XM_015092364.1	GART	mRNA	XR_001435723.1	lncRNA
XM_015092364.1	GART	mRNA	XR_001435723.1	lncRNA
XM_015092364.1	GART	mRNA	XR_001435723.1	lncRNA
XM_015092364.1	GART	mRNA	XR_001435723.1	lncRNA
XM_012184362.2	AKAP11	mRNA	XR_001435726.1	lncRNA
XM_012184362.2	AKAP11	mRNA	XR_001435726.1	lncRNA
XM_012184362.2	AKAP11	mRNA	XR_001435726.1	lncRNA
XM_012184362.2	AKAP11	mRNA	XR_001435726.1	lncRNA
XM_012184362.2	AKAP11	mRNA	XR_001435726.1	lncRNA
XM_012184362.2	AKAP11	mRNA	XR_001435726.1	lncRNA
XM_012184362.2	AKAP11	mRNA	XR_001435726.1	lncRNA
XM_012184362.2	AKAP11	mRNA	XR_001435726.1	lncRNA
XM_015098082.1	RNASEH2B	mRNA	XR_001435723.1	lncRNA
XM_015098082.1	RNASEH2B	mRNA	XR_001435723.1	lncRNA
XM_015098082.1	RNASEH2B	mRNA	XR_001435723.1	lncRNA
XM_015098082.1	RNASEH2B	mRNA	XR_001435723.1	lncRNA
XM_015098082.1	RNASEH2B	mRNA	XR_001435723.1	lncRNA
XM_015098082.1	RNASEH2B	mRNA	XR_001435723.1	lncRNA
XM_015098082.1	RNASEH2B	mRNA	XR_001435723.1	lncRNA
XM_015098082.1	RNASEH2B	mRNA	XR_001435723.1	lncRNA
XM_004012101.3	FREM2	mRNA	XR_001436390.1	lncRNA
XM_004012101.3	FREM2	mRNA	XR_001436390.1	lncRNA
XM_004012101.3	FREM2	mRNA	XR_001436390.1	lncRNA
XM_004012101.3	FREM2	mRNA	XR_001436390.1	lncRNA
XM_004012101.3	FREM2	mRNA	XR_001436390.1	lncRNA
XM_004012101.3	FREM2	mRNA	XR_001436390.1	lncRNA
XM_004012101.3	FREM2	mRNA	XR_001436390.1	lncRNA
XM_004012101.3	FREM2	mRNA	XR_001436390.1	lncRNA
XM_015094225.1	ROCK2	mRNA	XR_001433837.1	lncRNA
XM_015094225.1	ROCK2	mRNA	XR_001433837.1	lncRNA
XM_015094225.1	ROCK2	mRNA	XR_001433837.1	lncRNA
XM_015094225.1	ROCK2	mRNA	XR_001433837.1	lncRNA
XM_015094225.1	ROCK2	mRNA	XR_001433837.1	lncRNA
XM_015094225.1	ROCK2	mRNA	XR_001433837.1	lncRNA
XM_015094225.1	ROCK2	mRNA	XR_001433837.1	lncRNA
XM_015105074.1	LONRF3	mRNA	XR_001433837.1	lncRNA
XM_015105074.1	LONRF3	mRNA	XR_001433837.1	lncRNA
XM_015105074.1	LONRF3	mRNA	XR_001433837.1	lncRNA
XM_015105074.1	LONRF3	mRNA	XR_001433837.1	lncRNA
XM_015105074.1	LONRF3	mRNA	XR_001433837.1	lncRNA
XM_015105074.1	LONRF3	mRNA	XR_001433837.1	lncRNA
XM_015105074.1	LONRF3	mRNA	XR_001433837.1	lncRNA
XM_012178157.2	RAPGEF6	mRNA	XR_001023627.2	lncRNA
XM_012178157.2	RAPGEF6	mRNA	XR_001023627.2	lncRNA
XM_012178157.2	RAPGEF6	mRNA	XR_001023627.2	lncRNA
XM_012178157.2	RAPGEF6	mRNA	XR_001023627.2	lncRNA
XM_012178157.2	RAPGEF6	mRNA	XR_001023627.2	lncRNA
XM_012178157.2	RAPGEF6	mRNA	XR_001023627.2	lncRNA
XM_012178157.2	RAPGEF6	mRNA	XR_001023627.2	lncRNA
XM_015103687.1	PTPN2	mRNA	XR_001433837.1	lncRNA
XM_015103687.1	PTPN2	mRNA	XR_001433837.1	lncRNA

XM_015103687.1	PTPN2	mRNA	XR_001433837.1	lncRNA
XM_015103687.1	PTPN2	mRNA	XR_001433837.1	lncRNA
XM_015103687.1	PTPN2	mRNA	XR_001433837.1	lncRNA
XM_015103687.1	PTPN2	mRNA	XR_001433837.1	lncRNA
XM_015103687.1	PTPN2	mRNA	XR_001433837.1	lncRNA
XM_015098148.1	KATNAL1	mRNA	XR_001436390.1	lncRNA
XM_015098148.1	KATNAL1	mRNA	XR_001436390.1	lncRNA
XM_015098148.1	KATNAL1	mRNA	XR_001436390.1	lncRNA
XM_015098148.1	KATNAL1	mRNA	XR_001436390.1	lncRNA
XM_015098148.1	KATNAL1	mRNA	XR_001436390.1	lncRNA
XM_015098148.1	KATNAL1	mRNA	XR_001436390.1	lncRNA
XM_015098148.1	KATNAL1	mRNA	XR_001436390.1	lncRNA
XM_012178221.1	CEP120	mRNA	XR_001436390.1	lncRNA
XM_012178221.1	CEP120	mRNA	XR_001436390.1	lncRNA
XM_012178221.1	CEP120	mRNA	XR_001436390.1	lncRNA
XM_012178221.1	CEP120	mRNA	XR_001436390.1	lncRNA
XM_012178221.1	CEP120	mRNA	XR_001436390.1	lncRNA
XM_012178221.1	CEP120	mRNA	XR_001436390.1	lncRNA
XM_012178221.1	CEP120	mRNA	XR_001436390.1	lncRNA
XM_004003021.3	DLG1	mRNA	XR_001436612.1	lncRNA
XM_004003021.3	DLG1	mRNA	XR_001436612.1	lncRNA
XM_004003021.3	DLG1	mRNA	XR_001436612.1	lncRNA
XM_004003021.3	DLG1	mRNA	XR_001436612.1	lncRNA
XM_004003021.3	DLG1	mRNA	XR_001436612.1	lncRNA
XM_004003021.3	DLG1	mRNA	XR_001436612.1	lncRNA
XM_004003021.3	DLG1	mRNA	XR_001436612.1	lncRNA
XM_012174704.2	ZBTB39	mRNA	XR_001436612.1	lncRNA
XM_012174704.2	ZBTB39	mRNA	XR_001436612.1	lncRNA
XM_012174704.2	ZBTB39	mRNA	XR_001436612.1	lncRNA
XM_012174704.2	ZBTB39	mRNA	XR_001436612.1	lncRNA
XM_012174704.2	ZBTB39	mRNA	XR_001436612.1	lncRNA
XM_012174704.2	ZBTB39	mRNA	XR_001436612.1	lncRNA
XM_012174704.2	ZBTB39	mRNA	XR_001436612.1	lncRNA
XM_012097374.2	LRBA	mRNA	XR_001436612.1	lncRNA
XM_012097374.2	LRBA	mRNA	XR_001436612.1	lncRNA
XM_012097374.2	LRBA	mRNA	XR_001436612.1	lncRNA
XM_012097374.2	LRBA	mRNA	XR_001436612.1	lncRNA
XM_012097374.2	LRBA	mRNA	XR_001436612.1	lncRNA
XM_012097374.2	LRBA	mRNA	XR_001436612.1	lncRNA
XM_012097374.2	LRBA	mRNA	XR_001436612.1	lncRNA
XM_012103799.1	NEDD4L	mRNA	TCONS_00049802	lncRNA
XM_012103799.1	NEDD4L	mRNA	TCONS_00049802	lncRNA
XM_012103799.1	NEDD4L	mRNA	TCONS_00049802	lncRNA
XM_012103799.1	NEDD4L	mRNA	TCONS_00049802	lncRNA
XM_012103799.1	NEDD4L	mRNA	TCONS_00049802	lncRNA
XM_012103799.1	NEDD4L	mRNA	TCONS_00049802	lncRNA
XM_012103799.1	NEDD4L	mRNA	TCONS_00049802	lncRNA
XM_012180234.2	CNOT6L	mRNA	XR_001434127.1	lncRNA
XM_012180234.2	CNOT6L	mRNA	XR_001434127.1	lncRNA
XM_012180234.2	CNOT6L	mRNA	XR_001434127.1	lncRNA
XM_012180234.2	CNOT6L	mRNA	XR_001434127.1	lncRNA
XM_012180234.2	CNOT6L	mRNA	XR_001434127.1	lncRNA
XM_012180234.2	CNOT6L	mRNA	XR_001434127.1	lncRNA
XM_012180234.2	CNOT6L	mRNA	XR_001434127.1	lncRNA

XM_004017814.3	DNAJA4	mRNA	XR_001434127.1	lncRNA
XM_004017814.3	DNAJA4	mRNA	XR_001434127.1	lncRNA
XM_004017814.3	DNAJA4	mRNA	XR_001434127.1	lncRNA
XM_004017814.3	DNAJA4	mRNA	XR_001434127.1	lncRNA
XM_004017814.3	DNAJA4	mRNA	XR_001434127.1	lncRNA
XM_004017814.3	DNAJA4	mRNA	XR_001434127.1	lncRNA
XM_004017814.3	DNAJA4	mRNA	XR_001434127.1	lncRNA
XM_015092063.1	FUBP1	mRNA	XR_001435723.1	lncRNA
XM_015092063.1	FUBP1	mRNA	XR_001435723.1	lncRNA
XM_015092063.1	FUBP1	mRNA	XR_001435723.1	lncRNA
XM_015092063.1	FUBP1	mRNA	XR_001435723.1	lncRNA
XM_015092063.1	FUBP1	mRNA	XR_001435723.1	lncRNA
XM_015092063.1	FUBP1	mRNA	XR_001435723.1	lncRNA
XM_015092063.1	FUBP1	mRNA	XR_001435723.1	lncRNA
XM_015092971.1	SNX30	mRNA	XR_001436064.1	lncRNA
XM_015092971.1	SNX30	mRNA	XR_001436064.1	lncRNA
XM_015092971.1	SNX30	mRNA	XR_001436064.1	lncRNA
XM_015092971.1	SNX30	mRNA	XR_001436064.1	lncRNA
XM_015092971.1	SNX30	mRNA	XR_001436064.1	lncRNA
XM_015092971.1	SNX30	mRNA	XR_001436064.1	lncRNA
XM_015092971.1	SNX30	mRNA	XR_001436064.1	lncRNA
XM_012098903.1	PPIP5K1	mRNA	XR_001024230.1	lncRNA
XM_012098903.1	PPIP5K1	mRNA	XR_001024230.1	lncRNA
XM_012098903.1	PPIP5K1	mRNA	XR_001024230.1	lncRNA
XM_012098903.1	PPIP5K1	mRNA	XR_001024230.1	lncRNA
XM_012098903.1	PPIP5K1	mRNA	XR_001024230.1	lncRNA
XM_012098903.1	PPIP5K1	mRNA	XR_001024230.1	lncRNA
XM_012098903.1	PPIP5K1	mRNA	XR_001024230.1	lncRNA
XM_012099341.2	CLASP2	mRNA	XR_001436065.1	lncRNA
XM_012099341.2	CLASP2	mRNA	XR_001436065.1	lncRNA
XM_012099341.2	CLASP2	mRNA	XR_001436065.1	lncRNA
XM_012099341.2	CLASP2	mRNA	XR_001436065.1	lncRNA
XM_012099341.2	CLASP2	mRNA	XR_001436065.1	lncRNA
XM_012099341.2	CLASP2	mRNA	XR_001436065.1	lncRNA
XM_012099341.2	CLASP2	mRNA	XR_001436065.1	lncRNA
XM_012107808.1	NFYC	mRNA	XR_001436064.1	lncRNA
XM_012107808.1	NFYC	mRNA	XR_001436064.1	lncRNA
XM_012107808.1	NFYC	mRNA	XR_001436064.1	lncRNA
XM_012107808.1	NFYC	mRNA	XR_001436064.1	lncRNA
XM_012107808.1	NFYC	mRNA	XR_001436064.1	lncRNA
XM_012107808.1	NFYC	mRNA	XR_001436064.1	lncRNA
XM_012107808.1	NFYC	mRNA	XR_001436064.1	lncRNA
XM_012181265.2	PLA2G4F	mRNA	XR_001435723.1	lncRNA
XM_012181265.2	PLA2G4F	mRNA	XR_001435723.1	lncRNA
XM_012181265.2	PLA2G4F	mRNA	XR_001435723.1	lncRNA
XM_012181265.2	PLA2G4F	mRNA	XR_001435723.1	lncRNA
XM_012181265.2	PLA2G4F	mRNA	XR_001435723.1	lncRNA
XM_012181265.2	PLA2G4F	mRNA	XR_001435723.1	lncRNA
XM_012181265.2	PLA2G4F	mRNA	XR_001435723.1	lncRNA
XM_012098903.1	PPIP5K1	mRNA	XR_001436064.1	lncRNA
XM_012098903.1	PPIP5K1	mRNA	XR_001436064.1	lncRNA
XM_012098903.1	PPIP5K1	mRNA	XR_001436064.1	lncRNA
XM_012098903.1	PPIP5K1	mRNA	XR_001436064.1	lncRNA
XM_012098903.1	PPIP5K1	mRNA	XR_001436064.1	lncRNA

XM_012098903.1	PPIP5K1	mRNA	XR_001436064.1	lncRNA
XM_012098903.1	PPIP5K1	mRNA	XR_001436064.1	lncRNA
XM_015092971.1	SNX30	mRNA	XR_001436065.1	lncRNA
XM_015092971.1	SNX30	mRNA	XR_001436065.1	lncRNA
XM_015092971.1	SNX30	mRNA	XR_001436065.1	lncRNA
XM_015092971.1	SNX30	mRNA	XR_001436065.1	lncRNA
XM_015092971.1	SNX30	mRNA	XR_001436065.1	lncRNA
XM_015092971.1	SNX30	mRNA	XR_001436065.1	lncRNA
XM_015092971.1	SNX30	mRNA	XR_001436065.1	lncRNA
XM_015104915.1	JADE3	mRNA	XR_001436065.1	lncRNA
XM_015104915.1	JADE3	mRNA	XR_001436065.1	lncRNA
XM_015104915.1	JADE3	mRNA	XR_001436065.1	lncRNA
XM_015104915.1	JADE3	mRNA	XR_001436065.1	lncRNA
XM_015104915.1	JADE3	mRNA	XR_001436065.1	lncRNA
XM_015104915.1	JADE3	mRNA	XR_001436065.1	lncRNA
XM_015104915.1	JADE3	mRNA	XR_001436065.1	lncRNA
XM_012146156.1	USP37	mRNA	XR_001435723.1	lncRNA
XM_012146156.1	USP37	mRNA	XR_001435723.1	lncRNA
XM_012146156.1	USP37	mRNA	XR_001435723.1	lncRNA
XM_012146156.1	USP37	mRNA	XR_001435723.1	lncRNA
XM_012146156.1	USP37	mRNA	XR_001435723.1	lncRNA
XM_012146156.1	USP37	mRNA	XR_001435723.1	lncRNA
XM_012146156.1	USP37	mRNA	XR_001435723.1	lncRNA
XM_015092971.1	SNX30	mRNA	XR_001024230.1	lncRNA
XM_015092971.1	SNX30	mRNA	XR_001024230.1	lncRNA
XM_015092971.1	SNX30	mRNA	XR_001024230.1	lncRNA
XM_015092971.1	SNX30	mRNA	XR_001024230.1	lncRNA
XM_015092971.1	SNX30	mRNA	XR_001024230.1	lncRNA
XM_015092971.1	SNX30	mRNA	XR_001024230.1	lncRNA
XM_015092971.1	SNX30	mRNA	XR_001024230.1	lncRNA
XM_015104915.1	JADE3	mRNA	XR_001024230.1	lncRNA
XM_015104915.1	JADE3	mRNA	XR_001024230.1	lncRNA
XM_015104915.1	JADE3	mRNA	XR_001024230.1	lncRNA
XM_015104915.1	JADE3	mRNA	XR_001024230.1	lncRNA
XM_015104915.1	JADE3	mRNA	XR_001024230.1	lncRNA
XM_015104915.1	JADE3	mRNA	XR_001024230.1	lncRNA
XM_015104915.1	JADE3	mRNA	XR_001024230.1	lncRNA
XM_012146156.1	USP37	mRNA	XR_001436064.1	lncRNA
XM_012146156.1	USP37	mRNA	XR_001436064.1	lncRNA
XM_012146156.1	USP37	mRNA	XR_001436064.1	lncRNA
XM_012146156.1	USP37	mRNA	XR_001436064.1	lncRNA
XM_012146156.1	USP37	mRNA	XR_001436064.1	lncRNA
XM_012146156.1	USP37	mRNA	XR_001436064.1	lncRNA
XM_012146156.1	USP37	mRNA	XR_001436064.1	lncRNA
XM_012184216.2	MTBP	mRNA	XR_001436065.1	lncRNA
XM_012184216.2	MTBP	mRNA	XR_001436065.1	lncRNA
XM_012184216.2	MTBP	mRNA	XR_001436065.1	lncRNA
XM_012184216.2	MTBP	mRNA	XR_001436065.1	lncRNA
XM_012184216.2	MTBP	mRNA	XR_001436065.1	lncRNA
XM_012184216.2	MTBP	mRNA	XR_001436065.1	lncRNA
XM_012184216.2	MTBP	mRNA	XR_001436065.1	lncRNA
XM_012185875.1	LUC7L3	mRNA	XR_001435723.1	lncRNA
XM_012185875.1	LUC7L3	mRNA	XR_001435723.1	lncRNA
XM_012185875.1	LUC7L3	mRNA	XR_001435723.1	lncRNA

XM_012185875.1	LUC7L3	mRNA	XR_001435723.1	lncRNA
XM_012185875.1	LUC7L3	mRNA	XR_001435723.1	lncRNA
XM_012185875.1	LUC7L3	mRNA	XR_001435723.1	lncRNA
XM_012185875.1	LUC7L3	mRNA	XR_001435723.1	lncRNA
XM_012178157.2	RAPGEF6	mRNA	XR_001024230.1	lncRNA
XM_012178157.2	RAPGEF6	mRNA	XR_001024230.1	lncRNA
XM_012178157.2	RAPGEF6	mRNA	XR_001024230.1	lncRNA
XM_012178157.2	RAPGEF6	mRNA	XR_001024230.1	lncRNA
XM_012178157.2	RAPGEF6	mRNA	XR_001024230.1	lncRNA
XM_012178157.2	RAPGEF6	mRNA	XR_001024230.1	lncRNA
XM_012178157.2	RAPGEF6	mRNA	XR_001024230.1	lncRNA
XM_012146156.1	USP37	mRNA	XR_001436065.1	lncRNA
XM_012146156.1	USP37	mRNA	XR_001436065.1	lncRNA
XM_012146156.1	USP37	mRNA	XR_001436065.1	lncRNA
XM_012146156.1	USP37	mRNA	XR_001436065.1	lncRNA
XM_012146156.1	USP37	mRNA	XR_001436065.1	lncRNA
XM_012146156.1	USP37	mRNA	XR_001436065.1	lncRNA
XM_012146156.1	USP37	mRNA	XR_001436065.1	lncRNA
XM_012107808.1	NFYC	mRNA	XR_001435723.1	lncRNA
XM_012107808.1	NFYC	mRNA	XR_001435723.1	lncRNA
XM_012107808.1	NFYC	mRNA	XR_001435723.1	lncRNA
XM_012107808.1	NFYC	mRNA	XR_001435723.1	lncRNA
XM_012107808.1	NFYC	mRNA	XR_001435723.1	lncRNA
XM_012107808.1	NFYC	mRNA	XR_001435723.1	lncRNA
XM_012107808.1	NFYC	mRNA	XR_001435723.1	lncRNA
XM_012185875.1	LUC7L3	mRNA	XR_001436065.1	lncRNA
XM_012185875.1	LUC7L3	mRNA	XR_001436065.1	lncRNA
XM_012185875.1	LUC7L3	mRNA	XR_001436065.1	lncRNA
XM_012185875.1	LUC7L3	mRNA	XR_001436065.1	lncRNA
XM_012185875.1	LUC7L3	mRNA	XR_001436065.1	lncRNA
XM_012185875.1	LUC7L3	mRNA	XR_001436065.1	lncRNA
XM_012185875.1	LUC7L3	mRNA	XR_001436065.1	lncRNA
XM_012181265.2	PLA2G4F	mRNA	XR_001024230.1	lncRNA
XM_012181265.2	PLA2G4F	mRNA	XR_001024230.1	lncRNA
XM_012181265.2	PLA2G4F	mRNA	XR_001024230.1	lncRNA
XM_012181265.2	PLA2G4F	mRNA	XR_001024230.1	lncRNA
XM_012181265.2	PLA2G4F	mRNA	XR_001024230.1	lncRNA
XM_012181265.2	PLA2G4F	mRNA	XR_001024230.1	lncRNA
XM_012181265.2	PLA2G4F	mRNA	XR_001024230.1	lncRNA
XM_012181265.2	PLA2G4F	mRNA	XR_001436065.1	lncRNA
XM_012181265.2	PLA2G4F	mRNA	XR_001436065.1	lncRNA
XM_012181265.2	PLA2G4F	mRNA	XR_001436065.1	lncRNA
XM_012181265.2	PLA2G4F	mRNA	XR_001436065.1	lncRNA
XM_012181265.2	PLA2G4F	mRNA	XR_001436065.1	lncRNA
XM_012181265.2	PLA2G4F	mRNA	XR_001436065.1	lncRNA
XM_012181265.2	PLA2G4F	mRNA	XR_001436065.1	lncRNA
XM_012181265.2	PLA2G4F	mRNA	XR_001436064.1	lncRNA
XM_012181265.2	PLA2G4F	mRNA	XR_001436064.1	lncRNA
XM_012181265.2	PLA2G4F	mRNA	XR_001436064.1	lncRNA
XM_012181265.2	PLA2G4F	mRNA	XR_001436064.1	lncRNA
XM_012181265.2	PLA2G4F	mRNA	XR_001436064.1	lncRNA
XM_012181265.2	PLA2G4F	mRNA	XR_001436064.1	lncRNA
XM_012181265.2	PLA2G4F	mRNA	XR_001436064.1	lncRNA
XM_012188274.2	CCDC7	mRNA	XR_001024230.1	lncRNA

XM_012188274.2	CCDC7	mRNA	XR_001024230.1	lncRNA
XM_012188274.2	CCDC7	mRNA	XR_001024230.1	lncRNA
XM_012188274.2	CCDC7	mRNA	XR_001024230.1	lncRNA
XM_012188274.2	CCDC7	mRNA	XR_001024230.1	lncRNA
XM_012188274.2	CCDC7	mRNA	XR_001024230.1	lncRNA
XM_012188274.2	CCDC7	mRNA	XR_001024230.1	lncRNA
XM_012098903.1	PPIP5K1	mRNA	XR_001435723.1	lncRNA
XM_012098903.1	PPIP5K1	mRNA	XR_001435723.1	lncRNA
XM_012098903.1	PPIP5K1	mRNA	XR_001435723.1	lncRNA
XM_012098903.1	PPIP5K1	mRNA	XR_001435723.1	lncRNA
XM_012098903.1	PPIP5K1	mRNA	XR_001435723.1	lncRNA
XM_012098903.1	PPIP5K1	mRNA	XR_001435723.1	lncRNA
XM_012098903.1	PPIP5K1	mRNA	XR_001435723.1	lncRNA
XM_015099337.1	RALGPS2	mRNA	XR_001024230.1	lncRNA
XM_015099337.1	RALGPS2	mRNA	XR_001024230.1	lncRNA
XM_015099337.1	RALGPS2	mRNA	XR_001024230.1	lncRNA
XM_015099337.1	RALGPS2	mRNA	XR_001024230.1	lncRNA
XM_015099337.1	RALGPS2	mRNA	XR_001024230.1	lncRNA
XM_015099337.1	RALGPS2	mRNA	XR_001024230.1	lncRNA
XM_015099337.1	RALGPS2	mRNA	XR_001024230.1	lncRNA
XM_012146156.1	USP37	mRNA	XR_001024230.1	lncRNA
XM_012146156.1	USP37	mRNA	XR_001024230.1	lncRNA
XM_012146156.1	USP37	mRNA	XR_001024230.1	lncRNA
XM_012146156.1	USP37	mRNA	XR_001024230.1	lncRNA
XM_012146156.1	USP37	mRNA	XR_001024230.1	lncRNA
XM_012146156.1	USP37	mRNA	XR_001024230.1	lncRNA
XM_012146156.1	USP37	mRNA	XR_001024230.1	lncRNA
XM_012107808.1	NFYC	mRNA	XR_001436065.1	lncRNA
XM_012107808.1	NFYC	mRNA	XR_001436065.1	lncRNA
XM_012107808.1	NFYC	mRNA	XR_001436065.1	lncRNA
XM_012107808.1	NFYC	mRNA	XR_001436065.1	lncRNA
XM_012107808.1	NFYC	mRNA	XR_001436065.1	lncRNA
XM_012107808.1	NFYC	mRNA	XR_001436065.1	lncRNA
XM_012107808.1	NFYC	mRNA	XR_001436065.1	lncRNA
XM_012106946.2	ATP7A	mRNA	XR_001435723.1	lncRNA
XM_012106946.2	ATP7A	mRNA	XR_001435723.1	lncRNA
XM_012106946.2	ATP7A	mRNA	XR_001435723.1	lncRNA
XM_012106946.2	ATP7A	mRNA	XR_001435723.1	lncRNA
XM_012106946.2	ATP7A	mRNA	XR_001435723.1	lncRNA
XM_012106946.2	ATP7A	mRNA	XR_001435723.1	lncRNA
XM_012106946.2	ATP7A	mRNA	XR_001435723.1	lncRNA
XM_015099337.1	RALGPS2	mRNA	XR_001436065.1	lncRNA
XM_015099337.1	RALGPS2	mRNA	XR_001436065.1	lncRNA
XM_015099337.1	RALGPS2	mRNA	XR_001436065.1	lncRNA
XM_015099337.1	RALGPS2	mRNA	XR_001436065.1	lncRNA
XM_015099337.1	RALGPS2	mRNA	XR_001436065.1	lncRNA
XM_015099337.1	RALGPS2	mRNA	XR_001436065.1	lncRNA
XM_015099337.1	RALGPS2	mRNA	XR_001436065.1	lncRNA
XM_015099337.1	RALGPS2	mRNA	XR_001436064.1	lncRNA
XM_015099337.1	RALGPS2	mRNA	XR_001436064.1	lncRNA
XM_015099337.1	RALGPS2	mRNA	XR_001436064.1	lncRNA
XM_015099337.1	RALGPS2	mRNA	XR_001436064.1	lncRNA
XM_015099337.1	RALGPS2	mRNA	XR_001436064.1	lncRNA
XM_015099337.1	RALGPS2	mRNA	XR_001436064.1	lncRNA

XM_015099337.1	RALGPS2	mRNA	XR_001436064.1	lncRNA
XM_012106946.2	ATP7A	mRNA	XR_001435726.1	lncRNA
XM_012106946.2	ATP7A	mRNA	XR_001435726.1	lncRNA
XM_012106946.2	ATP7A	mRNA	XR_001435726.1	lncRNA
XM_012106946.2	ATP7A	mRNA	XR_001435726.1	lncRNA
XM_012106946.2	ATP7A	mRNA	XR_001435726.1	lncRNA
XM_012106946.2	ATP7A	mRNA	XR_001435726.1	lncRNA
XM_012106946.2	ATP7A	mRNA	XR_001435726.1	lncRNA
XM_012185875.1	LUC7L3	mRNA	XR_001436064.1	lncRNA
XM_012185875.1	LUC7L3	mRNA	XR_001436064.1	lncRNA
XM_012185875.1	LUC7L3	mRNA	XR_001436064.1	lncRNA
XM_012185875.1	LUC7L3	mRNA	XR_001436064.1	lncRNA
XM_012185875.1	LUC7L3	mRNA	XR_001436064.1	lncRNA
XM_012185875.1	LUC7L3	mRNA	XR_001436064.1	lncRNA
XM_012185875.1	LUC7L3	mRNA	XR_001436064.1	lncRNA
XM_012183298.1	FAM120B	mRNA	XR_001436064.1	lncRNA
XM_012183298.1	FAM120B	mRNA	XR_001436064.1	lncRNA
XM_012183298.1	FAM120B	mRNA	XR_001436064.1	lncRNA
XM_012183298.1	FAM120B	mRNA	XR_001436064.1	lncRNA
XM_012183298.1	FAM120B	mRNA	XR_001436064.1	lncRNA
XM_012183298.1	FAM120B	mRNA	XR_001436064.1	lncRNA
XM_012183298.1	FAM120B	mRNA	XR_001436064.1	lncRNA
XM_012185875.1	LUC7L3	mRNA	XR_001435726.1	lncRNA
XM_012185875.1	LUC7L3	mRNA	XR_001435726.1	lncRNA
XM_012185875.1	LUC7L3	mRNA	XR_001435726.1	lncRNA
XM_012185875.1	LUC7L3	mRNA	XR_001435726.1	lncRNA
XM_012185875.1	LUC7L3	mRNA	XR_001435726.1	lncRNA
XM_012185875.1	LUC7L3	mRNA	XR_001435726.1	lncRNA
XM_012185875.1	LUC7L3	mRNA	XR_001435726.1	lncRNA
XM_012146156.1	USP37	mRNA	XR_001435726.1	lncRNA
XM_012146156.1	USP37	mRNA	XR_001435726.1	lncRNA
XM_012146156.1	USP37	mRNA	XR_001435726.1	lncRNA
XM_012146156.1	USP37	mRNA	XR_001435726.1	lncRNA
XM_012146156.1	USP37	mRNA	XR_001435726.1	lncRNA
XM_012146156.1	USP37	mRNA	XR_001435726.1	lncRNA
XM_012146156.1	USP37	mRNA	XR_001435726.1	lncRNA
XM_015104915.1	JADE3	mRNA	XR_001435723.1	lncRNA
XM_015104915.1	JADE3	mRNA	XR_001435723.1	lncRNA
XM_015104915.1	JADE3	mRNA	XR_001435723.1	lncRNA
XM_015104915.1	JADE3	mRNA	XR_001435723.1	lncRNA
XM_015104915.1	JADE3	mRNA	XR_001435723.1	lncRNA
XM_015104915.1	JADE3	mRNA	XR_001435723.1	lncRNA
XM_015104915.1	JADE3	mRNA	XR_001435723.1	lncRNA
XM_012097627.1	LOC101114390	mRNA	XR_001435726.1	lncRNA
XM_012097627.1	LOC101114390	mRNA	XR_001435726.1	lncRNA
XM_012097627.1	LOC101114390	mRNA	XR_001435726.1	lncRNA
XM_012097627.1	LOC101114390	mRNA	XR_001435726.1	lncRNA
XM_012097627.1	LOC101114390	mRNA	XR_001435726.1	lncRNA
XM_012097627.1	LOC101114390	mRNA	XR_001435726.1	lncRNA
XM_012097627.1	LOC101114390	mRNA	XR_001435726.1	lncRNA
XM_015091887.1	MYCL	mRNA	XR_001435726.1	lncRNA
XM_015091887.1	MYCL	mRNA	XR_001435726.1	lncRNA
XM_015091887.1	MYCL	mRNA	XR_001435726.1	lncRNA
XM_015091887.1	MYCL	mRNA	XR_001435726.1	lncRNA

XM_015091887.1	MYCL	mRNA	XR_001435726.1	lncRNA
XM_015091887.1	MYCL	mRNA	XR_001435726.1	lncRNA
XM_015091887.1	MYCL	mRNA	XR_001435726.1	lncRNA
XM_015104915.1	JADE3	mRNA	XR_001436064.1	lncRNA
XM_015104915.1	JADE3	mRNA	XR_001436064.1	lncRNA
XM_015104915.1	JADE3	mRNA	XR_001436064.1	lncRNA
XM_015104915.1	JADE3	mRNA	XR_001436064.1	lncRNA
XM_015104915.1	JADE3	mRNA	XR_001436064.1	lncRNA
XM_015104915.1	JADE3	mRNA	XR_001436064.1	lncRNA
XM_015104915.1	JADE3	mRNA	XR_001436064.1	lncRNA
XM_012181265.2	PLA2G4F	mRNA	XR_001435726.1	lncRNA
XM_012181265.2	PLA2G4F	mRNA	XR_001435726.1	lncRNA
XM_012181265.2	PLA2G4F	mRNA	XR_001435726.1	lncRNA
XM_012181265.2	PLA2G4F	mRNA	XR_001435726.1	lncRNA
XM_012181265.2	PLA2G4F	mRNA	XR_001435726.1	lncRNA
XM_012181265.2	PLA2G4F	mRNA	XR_001435726.1	lncRNA
XM_012181265.2	PLA2G4F	mRNA	XR_001435726.1	lncRNA
XM_012099341.2	CLASP2	mRNA	XR_001436064.1	lncRNA
XM_012099341.2	CLASP2	mRNA	XR_001436064.1	lncRNA
XM_012099341.2	CLASP2	mRNA	XR_001436064.1	lncRNA
XM_012099341.2	CLASP2	mRNA	XR_001436064.1	lncRNA
XM_012099341.2	CLASP2	mRNA	XR_001436064.1	lncRNA
XM_012099341.2	CLASP2	mRNA	XR_001436064.1	lncRNA
XM_012099341.2	CLASP2	mRNA	XR_001436064.1	lncRNA
XM_015099337.1	RALGPS2	mRNA	XR_001435723.1	lncRNA
XM_015099337.1	RALGPS2	mRNA	XR_001435723.1	lncRNA
XM_015099337.1	RALGPS2	mRNA	XR_001435723.1	lncRNA
XM_015099337.1	RALGPS2	mRNA	XR_001435723.1	lncRNA
XM_015099337.1	RALGPS2	mRNA	XR_001435723.1	lncRNA
XM_015099337.1	RALGPS2	mRNA	XR_001435723.1	lncRNA
XM_015099337.1	RALGPS2	mRNA	XR_001435723.1	lncRNA
XM_012188274.2	CCDC7	mRNA	XR_001435723.1	lncRNA
XM_012188274.2	CCDC7	mRNA	XR_001435723.1	lncRNA
XM_012188274.2	CCDC7	mRNA	XR_001435723.1	lncRNA
XM_012188274.2	CCDC7	mRNA	XR_001435723.1	lncRNA
XM_012188274.2	CCDC7	mRNA	XR_001435723.1	lncRNA
XM_012188274.2	CCDC7	mRNA	XR_001435723.1	lncRNA
XM_012188274.2	CCDC7	mRNA	XR_001435723.1	lncRNA
XM_012142659.2	ALS2	mRNA	XR_001435726.1	lncRNA
XM_012142659.2	ALS2	mRNA	XR_001435726.1	lncRNA
XM_012142659.2	ALS2	mRNA	XR_001435726.1	lncRNA
XM_012142659.2	ALS2	mRNA	XR_001435726.1	lncRNA
XM_012142659.2	ALS2	mRNA	XR_001435726.1	lncRNA
XM_012142659.2	ALS2	mRNA	XR_001435726.1	lncRNA
XM_012142659.2	ALS2	mRNA	XR_001435726.1	lncRNA
XM_012189447.2	ALCAM	mRNA	XR_001435726.1	lncRNA
XM_012189447.2	ALCAM	mRNA	XR_001435726.1	lncRNA
XM_012189447.2	ALCAM	mRNA	XR_001435726.1	lncRNA
XM_012189447.2	ALCAM	mRNA	XR_001435726.1	lncRNA
XM_012189447.2	ALCAM	mRNA	XR_001435726.1	lncRNA
XM_012189447.2	ALCAM	mRNA	XR_001435726.1	lncRNA
XM_012189447.2	ALCAM	mRNA	XR_001435726.1	lncRNA
XM_015092063.1	FUBP1	mRNA	XR_001435726.1	lncRNA
XM_015092063.1	FUBP1	mRNA	XR_001435726.1	lncRNA

XM_015092063.1	FUBP1	mRNA	XR_001435726.1	lncRNA
XM_015092063.1	FUBP1	mRNA	XR_001435726.1	lncRNA
XM_015092063.1	FUBP1	mRNA	XR_001435726.1	lncRNA
XM_015092063.1	FUBP1	mRNA	XR_001435726.1	lncRNA
XM_015092063.1	FUBP1	mRNA	XR_001435726.1	lncRNA
XM_015104915.1	JADE3	mRNA	XR_001435726.1	lncRNA
XM_015104915.1	JADE3	mRNA	XR_001435726.1	lncRNA
XM_015104915.1	JADE3	mRNA	XR_001435726.1	lncRNA
XM_015104915.1	JADE3	mRNA	XR_001435726.1	lncRNA
XM_015104915.1	JADE3	mRNA	XR_001435726.1	lncRNA
XM_015104915.1	JADE3	mRNA	XR_001435726.1	lncRNA
XM_015104915.1	JADE3	mRNA	XR_001435726.1	lncRNA
XM_012107808.1	NFYC	mRNA	XR_001024230.1	lncRNA
XM_012107808.1	NFYC	mRNA	XR_001024230.1	lncRNA
XM_012107808.1	NFYC	mRNA	XR_001024230.1	lncRNA
XM_012107808.1	NFYC	mRNA	XR_001024230.1	lncRNA
XM_012107808.1	NFYC	mRNA	XR_001024230.1	lncRNA
XM_012107808.1	NFYC	mRNA	XR_001024230.1	lncRNA
XM_012107808.1	NFYC	mRNA	XR_001024230.1	lncRNA
XM_012098903.1	PPIP5K1	mRNA	XR_001435726.1	lncRNA
XM_012098903.1	PPIP5K1	mRNA	XR_001435726.1	lncRNA
XM_012098903.1	PPIP5K1	mRNA	XR_001435726.1	lncRNA
XM_012098903.1	PPIP5K1	mRNA	XR_001435726.1	lncRNA
XM_012098903.1	PPIP5K1	mRNA	XR_001435726.1	lncRNA
XM_012098903.1	PPIP5K1	mRNA	XR_001435726.1	lncRNA
XM_012098903.1	PPIP5K1	mRNA	XR_001435726.1	lncRNA
XM_012186362.1	PITPNC1	mRNA	XR_001433837.1	lncRNA
XM_012186362.1	PITPNC1	mRNA	XR_001433837.1	lncRNA
XM_012186362.1	PITPNC1	mRNA	XR_001433837.1	lncRNA
XM_012186362.1	PITPNC1	mRNA	XR_001433837.1	lncRNA
XM_012186362.1	PITPNC1	mRNA	XR_001433837.1	lncRNA
XM_012186362.1	PITPNC1	mRNA	XR_001433837.1	lncRNA
XM_012186362.1	PITPNC1	mRNA	XR_001433837.1	lncRNA
XM_012095418.2	ZMYM4	mRNA	XR_001040147.1	lncRNA
XM_012095418.2	ZMYM4	mRNA	XR_001040147.1	lncRNA
XM_012095418.2	ZMYM4	mRNA	XR_001040147.1	lncRNA
XM_012095418.2	ZMYM4	mRNA	XR_001040147.1	lncRNA
XM_012095418.2	ZMYM4	mRNA	XR_001040147.1	lncRNA
XM_012095418.2	ZMYM4	mRNA	XR_001040147.1	lncRNA
XM_012095418.2	ZMYM4	mRNA	XR_001040147.1	lncRNA
XM_012187084.2	MIA3	mRNA	XR_001436390.1	lncRNA
XM_012187084.2	MIA3	mRNA	XR_001436390.1	lncRNA
XM_012187084.2	MIA3	mRNA	XR_001436390.1	lncRNA
XM_012187084.2	MIA3	mRNA	XR_001436390.1	lncRNA
XM_012187084.2	MIA3	mRNA	XR_001436390.1	lncRNA
XM_012187084.2	MIA3	mRNA	XR_001436390.1	lncRNA
XM_012187084.2	MIA3	mRNA	XR_001436390.1	lncRNA
XM_012098812.2	RALGAPA1	mRNA	XR_001436390.1	lncRNA
XM_012098812.2	RALGAPA1	mRNA	XR_001436390.1	lncRNA
XM_012098812.2	RALGAPA1	mRNA	XR_001436390.1	lncRNA
XM_012098812.2	RALGAPA1	mRNA	XR_001436390.1	lncRNA
XM_012098812.2	RALGAPA1	mRNA	XR_001436390.1	lncRNA
XM_012098812.2	RALGAPA1	mRNA	XR_001436390.1	lncRNA
XM_012098812.2	RALGAPA1	mRNA	XR_001436390.1	lncRNA

XM_012186312.2	DDX42	mRNA	XR_001436390.1	lncRNA
XM_012186312.2	DDX42	mRNA	XR_001436390.1	lncRNA
XM_012186312.2	DDX42	mRNA	XR_001436390.1	lncRNA
XM_012186312.2	DDX42	mRNA	XR_001436390.1	lncRNA
XM_012186312.2	DDX42	mRNA	XR_001436390.1	lncRNA
XM_012186312.2	DDX42	mRNA	XR_001436390.1	lncRNA
XM_012186312.2	DDX42	mRNA	XR_001436390.1	lncRNA
XM_015096977.1	NIN	mRNA	XR_001436390.1	lncRNA
XM_015096977.1	NIN	mRNA	XR_001436390.1	lncRNA
XM_015096977.1	NIN	mRNA	XR_001436390.1	lncRNA
XM_015096977.1	NIN	mRNA	XR_001436390.1	lncRNA
XM_015096977.1	NIN	mRNA	XR_001436390.1	lncRNA
XM_015096977.1	NIN	mRNA	XR_001436390.1	lncRNA
XM_015096977.1	NIN	mRNA	XR_001436390.1	lncRNA
XM_015103687.1	PTPN2	mRNA	XR_001436612.1	lncRNA
XM_015103687.1	PTPN2	mRNA	XR_001436612.1	lncRNA
XM_015103687.1	PTPN2	mRNA	XR_001436612.1	lncRNA
XM_015103687.1	PTPN2	mRNA	XR_001436612.1	lncRNA
XM_015103687.1	PTPN2	mRNA	XR_001436612.1	lncRNA
XM_015103687.1	PTPN2	mRNA	XR_001436612.1	lncRNA
XM_015103687.1	PTPN2	mRNA	XR_001436612.1	lncRNA
XM_012096323.1	PHF21A	mRNA	XR_001436612.1	lncRNA
XM_012096323.1	PHF21A	mRNA	XR_001436612.1	lncRNA
XM_012096323.1	PHF21A	mRNA	XR_001436612.1	lncRNA
XM_012096323.1	PHF21A	mRNA	XR_001436612.1	lncRNA
XM_012096323.1	PHF21A	mRNA	XR_001436612.1	lncRNA
XM_012096323.1	PHF21A	mRNA	XR_001436612.1	lncRNA
XM_012096323.1	PHF21A	mRNA	XR_001436612.1	lncRNA
XM_015105074.1	LONRF3	mRNA	TCONS_00049802	lncRNA
XM_015105074.1	LONRF3	mRNA	TCONS_00049802	lncRNA
XM_015105074.1	LONRF3	mRNA	TCONS_00049802	lncRNA
XM_015105074.1	LONRF3	mRNA	TCONS_00049802	lncRNA
XM_015105074.1	LONRF3	mRNA	TCONS_00049802	lncRNA
XM_015105074.1	LONRF3	mRNA	TCONS_00049802	lncRNA
XM_015105074.1	LONRF3	mRNA	TCONS_00049802	lncRNA
XM_012184885.2	DOCK9	mRNA	XR_001434127.1	lncRNA
XM_012184885.2	DOCK9	mRNA	XR_001434127.1	lncRNA
XM_012184885.2	DOCK9	mRNA	XR_001434127.1	lncRNA
XM_012184885.2	DOCK9	mRNA	XR_001434127.1	lncRNA
XM_012184885.2	DOCK9	mRNA	XR_001434127.1	lncRNA
XM_012184885.2	DOCK9	mRNA	XR_001434127.1	lncRNA
XM_012184885.2	DOCK9	mRNA	XR_001434127.1	lncRNA
XM_012097035.1	C16H5orf42	mRNA	XR_001434127.1	lncRNA
XM_012097035.1	C16H5orf42	mRNA	XR_001434127.1	lncRNA
XM_012097035.1	C16H5orf42	mRNA	XR_001434127.1	lncRNA
XM_012097035.1	C16H5orf42	mRNA	XR_001434127.1	lncRNA
XM_012097035.1	C16H5orf42	mRNA	XR_001434127.1	lncRNA
XM_012097035.1	C16H5orf42	mRNA	XR_001434127.1	lncRNA
XM_012097035.1	C16H5orf42	mRNA	XR_001434127.1	lncRNA
XM_015105074.1	LONRF3	mRNA	XR_001023627.2	lncRNA
XM_015105074.1	LONRF3	mRNA	XR_001023627.2	lncRNA
XM_015105074.1	LONRF3	mRNA	XR_001023627.2	lncRNA
XM_015105074.1	LONRF3	mRNA	XR_001023627.2	lncRNA
XM_015105074.1	LONRF3	mRNA	XR_001023627.2	lncRNA

XM_015105074.1	LONRF3	mRNA	XR_001023627.2	lncRNA
XM_015105074.1	LONRF3	mRNA	XR_001023627.2	lncRNA
XM_004009738.3	PPARGC1A	mRNA	XR_001023627.2	lncRNA
XM_004009738.3	PPARGC1A	mRNA	XR_001023627.2	lncRNA
XM_004009738.3	PPARGC1A	mRNA	XR_001023627.2	lncRNA
XM_004009738.3	PPARGC1A	mRNA	XR_001023627.2	lncRNA
XM_004009738.3	PPARGC1A	mRNA	XR_001023627.2	lncRNA
XM_004009738.3	PPARGC1A	mRNA	XR_001023627.2	lncRNA
XM_004009738.3	PPARGC1A	mRNA	XR_001023627.2	lncRNA
XM_012184885.2	DOCK9	mRNA	TCONS_00049802	lncRNA
XM_012184885.2	DOCK9	mRNA	TCONS_00049802	lncRNA
XM_012184885.2	DOCK9	mRNA	TCONS_00049802	lncRNA
XM_012184885.2	DOCK9	mRNA	TCONS_00049802	lncRNA
XM_012184885.2	DOCK9	mRNA	TCONS_00049802	lncRNA
XM_012184885.2	DOCK9	mRNA	TCONS_00049802	lncRNA
XM_012184885.2	DOCK9	mRNA	TCONS_00049802	lncRNA
XM_012179676.2	CCSER1	mRNA	TCONS_00049802	lncRNA
XM_012179676.2	CCSER1	mRNA	TCONS_00049802	lncRNA
XM_012179676.2	CCSER1	mRNA	TCONS_00049802	lncRNA
XM_012179676.2	CCSER1	mRNA	TCONS_00049802	lncRNA
XM_012179676.2	CCSER1	mRNA	TCONS_00049802	lncRNA
XM_012179676.2	CCSER1	mRNA	TCONS_00049802	lncRNA
XM_012179676.2	CCSER1	mRNA	TCONS_00049802	lncRNA
XM_012179676.2	CCSER1	mRNA	XR_001434127.1	lncRNA
XM_012179676.2	CCSER1	mRNA	XR_001434127.1	lncRNA
XM_012179676.2	CCSER1	mRNA	XR_001434127.1	lncRNA
XM_012179676.2	CCSER1	mRNA	XR_001434127.1	lncRNA
XM_012179676.2	CCSER1	mRNA	XR_001434127.1	lncRNA
XM_012179676.2	CCSER1	mRNA	XR_001434127.1	lncRNA
XM_012179676.2	CCSER1	mRNA	XR_001434127.1	lncRNA
XM_012174536.2	KIF21A	mRNA	XR_001434127.1	lncRNA
XM_012174536.2	KIF21A	mRNA	XR_001434127.1	lncRNA
XM_012174536.2	KIF21A	mRNA	XR_001434127.1	lncRNA
XM_012174536.2	KIF21A	mRNA	XR_001434127.1	lncRNA
XM_012174536.2	KIF21A	mRNA	XR_001434127.1	lncRNA
XM_012174536.2	KIF21A	mRNA	XR_001434127.1	lncRNA
XM_012174536.2	KIF21A	mRNA	XR_001434127.1	lncRNA
XM_015094225.1	ROCK2	mRNA	XR_001023627.2	lncRNA
XM_015094225.1	ROCK2	mRNA	XR_001023627.2	lncRNA
XM_015094225.1	ROCK2	mRNA	XR_001023627.2	lncRNA
XM_015094225.1	ROCK2	mRNA	XR_001023627.2	lncRNA
XM_015094225.1	ROCK2	mRNA	XR_001023627.2	lncRNA
XM_015094225.1	ROCK2	mRNA	XR_001023627.2	lncRNA
XM_015094225.1	ROCK2	mRNA	XR_001023627.2	lncRNA
XM_012184362.2	AKAP11	mRNA	XR_001023627.2	lncRNA
XM_012184362.2	AKAP11	mRNA	XR_001023627.2	lncRNA
XM_012184362.2	AKAP11	mRNA	XR_001023627.2	lncRNA
XM_012184362.2	AKAP11	mRNA	XR_001023627.2	lncRNA
XM_012184362.2	AKAP11	mRNA	XR_001023627.2	lncRNA
XM_012184362.2	AKAP11	mRNA	XR_001023627.2	lncRNA
XM_012184362.2	AKAP11	mRNA	XR_001023627.2	lncRNA
XM_012179676.2	CCSER1	mRNA	XR_001023627.2	lncRNA
XM_012179676.2	CCSER1	mRNA	XR_001023627.2	lncRNA
XM_012179676.2	CCSER1	mRNA	XR_001023627.2	lncRNA

XM_012179676.2	CCSER1	mRNA	XR_001023627.2	lncRNA
XM_012179676.2	CCSER1	mRNA	XR_001023627.2	lncRNA
XM_012179676.2	CCSER1	mRNA	XR_001023627.2	lncRNA
XM_012179676.2	CCSER1	mRNA	XR_001023627.2	lncRNA
XM_015101001.1	RRAS2	mRNA	TCONS_00049802	lncRNA
XM_015101001.1	RRAS2	mRNA	TCONS_00049802	lncRNA
XM_015101001.1	RRAS2	mRNA	TCONS_00049802	lncRNA
XM_015101001.1	RRAS2	mRNA	TCONS_00049802	lncRNA
XM_015101001.1	RRAS2	mRNA	TCONS_00049802	lncRNA
XM_015101001.1	RRAS2	mRNA	TCONS_00049802	lncRNA
XM_015101001.1	RRAS2	mRNA	TCONS_00049802	lncRNA
XM_015091887.1	MYCL	mRNA	XR_001436817.1	lncRNA
XM_015091887.1	MYCL	mRNA	XR_001436817.1	lncRNA
XM_015091887.1	MYCL	mRNA	XR_001436817.1	lncRNA
XM_015091887.1	MYCL	mRNA	XR_001436817.1	lncRNA
XM_015091887.1	MYCL	mRNA	XR_001436817.1	lncRNA
XM_015091887.1	MYCL	mRNA	XR_001436817.1	lncRNA
XM_015091887.1	MYCL	mRNA	XR_001436817.1	lncRNA
XM_012173383.2	DHX57	mRNA	XR_001435722.1	lncRNA
XM_012173383.2	DHX57	mRNA	XR_001435722.1	lncRNA
XM_012173383.2	DHX57	mRNA	XR_001435722.1	lncRNA
XM_012173383.2	DHX57	mRNA	XR_001435722.1	lncRNA
XM_012173383.2	DHX57	mRNA	XR_001435722.1	lncRNA
XM_012173383.2	DHX57	mRNA	XR_001435722.1	lncRNA
XM_012173383.2	DHX57	mRNA	XR_001435722.1	lncRNA
XM_004015120.2	PHLPP2	mRNA	XR_001435107.1	lncRNA
XM_004015120.2	PHLPP2	mRNA	XR_001435107.1	lncRNA
XM_004015120.2	PHLPP2	mRNA	XR_001435107.1	lncRNA
XM_004015120.2	PHLPP2	mRNA	XR_001435107.1	lncRNA
XM_004015120.2	PHLPP2	mRNA	XR_001435107.1	lncRNA
XM_004015120.2	PHLPP2	mRNA	XR_001435107.1	lncRNA
XM_004015120.2	PHLPP2	mRNA	XR_001435107.1	lncRNA
XM_015104323.1	SPRTN	mRNA	XR_001434920.1	lncRNA
XM_015104323.1	SPRTN	mRNA	XR_001434920.1	lncRNA
XM_015104323.1	SPRTN	mRNA	XR_001434920.1	lncRNA
XM_015104323.1	SPRTN	mRNA	XR_001434920.1	lncRNA
XM_015104323.1	SPRTN	mRNA	XR_001434920.1	lncRNA
XM_015104323.1	SPRTN	mRNA	XR_001434920.1	lncRNA
XM_015104323.1	SPRTN	mRNA	XR_001434920.1	lncRNA
XM_015099212.1	MIIP	mRNA	XR_001435722.1	lncRNA
XM_015099212.1	MIIP	mRNA	XR_001435722.1	lncRNA
XM_015099212.1	MIIP	mRNA	XR_001435722.1	lncRNA
XM_015099212.1	MIIP	mRNA	XR_001435722.1	lncRNA
XM_015099212.1	MIIP	mRNA	XR_001435722.1	lncRNA
XM_015099212.1	MIIP	mRNA	XR_001435722.1	lncRNA
XM_015099212.1	MIIP	mRNA	XR_001435722.1	lncRNA
XM_012178221.1	CEP120	mRNA	XR_001435722.1	lncRNA
XM_012178221.1	CEP120	mRNA	XR_001435722.1	lncRNA
XM_012178221.1	CEP120	mRNA	XR_001435722.1	lncRNA
XM_012178221.1	CEP120	mRNA	XR_001435722.1	lncRNA
XM_012178221.1	CEP120	mRNA	XR_001435722.1	lncRNA
XM_012178221.1	CEP120	mRNA	XR_001435722.1	lncRNA
XM_012178221.1	CEP120	mRNA	XR_001435722.1	lncRNA
XM_012173383.2	DHX57	mRNA	XR_001436817.1	lncRNA

XM_012173383.2	DHX57	mRNA	XR_001436817.1	lncRNA
XM_012173383.2	DHX57	mRNA	XR_001436817.1	lncRNA
XM_012173383.2	DHX57	mRNA	XR_001436817.1	lncRNA
XM_012173383.2	DHX57	mRNA	XR_001436817.1	lncRNA
XM_012173383.2	DHX57	mRNA	XR_001436817.1	lncRNA
XM_012173383.2	DHX57	mRNA	XR_001436817.1	lncRNA
XM_004015120.2	PHLPP2	mRNA	XR_001436817.1	lncRNA
XM_004015120.2	PHLPP2	mRNA	XR_001436817.1	lncRNA
XM_004015120.2	PHLPP2	mRNA	XR_001436817.1	lncRNA
XM_004015120.2	PHLPP2	mRNA	XR_001436817.1	lncRNA
XM_004015120.2	PHLPP2	mRNA	XR_001436817.1	lncRNA
XM_004015120.2	PHLPP2	mRNA	XR_001436817.1	lncRNA
XM_004015120.2	PHLPP2	mRNA	XR_001436817.1	lncRNA
XM_015099976.1	ZFP30	mRNA	XR_001433856.1	lncRNA
XM_015099976.1	ZFP30	mRNA	XR_001433856.1	lncRNA
XM_015099976.1	ZFP30	mRNA	XR_001433856.1	lncRNA
XM_015099976.1	ZFP30	mRNA	XR_001433856.1	lncRNA
XM_015099976.1	ZFP30	mRNA	XR_001433856.1	lncRNA
XM_015099976.1	ZFP30	mRNA	XR_001433856.1	lncRNA
XM_015099976.1	ZFP30	mRNA	XR_001433856.1	lncRNA
XM_012189447.2	ALCAM	mRNA	XR_001436817.1	lncRNA
XM_012189447.2	ALCAM	mRNA	XR_001436817.1	lncRNA
XM_012189447.2	ALCAM	mRNA	XR_001436817.1	lncRNA
XM_012189447.2	ALCAM	mRNA	XR_001436817.1	lncRNA
XM_012189447.2	ALCAM	mRNA	XR_001436817.1	lncRNA
XM_012189447.2	ALCAM	mRNA	XR_001436817.1	lncRNA
XM_012189447.2	ALCAM	mRNA	XR_001436817.1	lncRNA
XM_012181265.2	PLA2G4F	mRNA	XR_001436817.1	lncRNA
XM_012181265.2	PLA2G4F	mRNA	XR_001436817.1	lncRNA
XM_012181265.2	PLA2G4F	mRNA	XR_001436817.1	lncRNA
XM_012181265.2	PLA2G4F	mRNA	XR_001436817.1	lncRNA
XM_012181265.2	PLA2G4F	mRNA	XR_001436817.1	lncRNA
XM_012181265.2	PLA2G4F	mRNA	XR_001436817.1	lncRNA
XM_012181265.2	PLA2G4F	mRNA	XR_001436817.1	lncRNA
XM_012106946.2	ATP7A	mRNA	XR_001435722.1	lncRNA
XM_012106946.2	ATP7A	mRNA	XR_001435722.1	lncRNA
XM_012106946.2	ATP7A	mRNA	XR_001435722.1	lncRNA
XM_012106946.2	ATP7A	mRNA	XR_001435722.1	lncRNA
XM_012106946.2	ATP7A	mRNA	XR_001435722.1	lncRNA
XM_012106946.2	ATP7A	mRNA	XR_001435722.1	lncRNA
XM_012106946.2	ATP7A	mRNA	XR_001435722.1	lncRNA
XM_015092063.1	FUBP1	mRNA	XR_001435722.1	lncRNA
XM_015092063.1	FUBP1	mRNA	XR_001435722.1	lncRNA
XM_015092063.1	FUBP1	mRNA	XR_001435722.1	lncRNA
XM_015092063.1	FUBP1	mRNA	XR_001435722.1	lncRNA
XM_015092063.1	FUBP1	mRNA	XR_001435722.1	lncRNA
XM_015092063.1	FUBP1	mRNA	XR_001435722.1	lncRNA
XM_015092063.1	FUBP1	mRNA	XR_001435722.1	lncRNA
XM_004015120.2	PHLPP2	mRNA	XR_001435722.1	lncRNA
XM_004015120.2	PHLPP2	mRNA	XR_001435722.1	lncRNA
XM_004015120.2	PHLPP2	mRNA	XR_001435722.1	lncRNA
XM_004015120.2	PHLPP2	mRNA	XR_001435722.1	lncRNA
XM_004015120.2	PHLPP2	mRNA	XR_001435722.1	lncRNA
XM_004015120.2	PHLPP2	mRNA	XR_001435722.1	lncRNA

XM_004015120.2	PHLPP2	mRNA	XR_001435722.1	lncRNA
XM_015098148.1	KATNAL1	mRNA	XR_001433856.1	lncRNA
XM_015098148.1	KATNAL1	mRNA	XR_001433856.1	lncRNA
XM_015098148.1	KATNAL1	mRNA	XR_001433856.1	lncRNA
XM_015098148.1	KATNAL1	mRNA	XR_001433856.1	lncRNA
XM_015098148.1	KATNAL1	mRNA	XR_001433856.1	lncRNA
XM_015098148.1	KATNAL1	mRNA	XR_001433856.1	lncRNA
XM_015098148.1	KATNAL1	mRNA	XR_001433856.1	lncRNA
XM_012188917.1	STX16	mRNA	XR_001433856.1	lncRNA
XM_012188917.1	STX16	mRNA	XR_001433856.1	lncRNA
XM_012188917.1	STX16	mRNA	XR_001433856.1	lncRNA
XM_012188917.1	STX16	mRNA	XR_001433856.1	lncRNA
XM_012188917.1	STX16	mRNA	XR_001433856.1	lncRNA
XM_012188917.1	STX16	mRNA	XR_001433856.1	lncRNA
XM_012188917.1	STX16	mRNA	XR_001433856.1	lncRNA
XM_012102983.2	ABLIM1	mRNA	XR_001435107.1	lncRNA
XM_012102983.2	ABLIM1	mRNA	XR_001435107.1	lncRNA
XM_012102983.2	ABLIM1	mRNA	XR_001435107.1	lncRNA
XM_012102983.2	ABLIM1	mRNA	XR_001435107.1	lncRNA
XM_012102983.2	ABLIM1	mRNA	XR_001435107.1	lncRNA
XM_012102983.2	ABLIM1	mRNA	XR_001435107.1	lncRNA
XM_012102983.2	ABLIM1	mRNA	XR_001435107.1	lncRNA
XM_012173383.2	DHX57	mRNA	XR_001435107.1	lncRNA
XM_012173383.2	DHX57	mRNA	XR_001435107.1	lncRNA
XM_012173383.2	DHX57	mRNA	XR_001435107.1	lncRNA
XM_012173383.2	DHX57	mRNA	XR_001435107.1	lncRNA
XM_012173383.2	DHX57	mRNA	XR_001435107.1	lncRNA
XM_012173383.2	DHX57	mRNA	XR_001435107.1	lncRNA
XM_012173383.2	DHX57	mRNA	XR_001435107.1	lncRNA
XM_012106946.2	ATP7A	mRNA	XR_001436817.1	lncRNA
XM_012106946.2	ATP7A	mRNA	XR_001436817.1	lncRNA
XM_012106946.2	ATP7A	mRNA	XR_001436817.1	lncRNA
XM_012106946.2	ATP7A	mRNA	XR_001436817.1	lncRNA
XM_012106946.2	ATP7A	mRNA	XR_001436817.1	lncRNA
XM_012106946.2	ATP7A	mRNA	XR_001436817.1	lncRNA
XM_012106946.2	ATP7A	mRNA	XR_001436817.1	lncRNA
XM_015095425.1	CUL1	mRNA	XR_001433856.1	lncRNA
XM_015095425.1	CUL1	mRNA	XR_001433856.1	lncRNA
XM_015095425.1	CUL1	mRNA	XR_001433856.1	lncRNA
XM_015095425.1	CUL1	mRNA	XR_001433856.1	lncRNA
XM_015095425.1	CUL1	mRNA	XR_001433856.1	lncRNA
XM_015095425.1	CUL1	mRNA	XR_001433856.1	lncRNA
XM_015095425.1	CUL1	mRNA	XR_001433856.1	lncRNA
XM_015100436.1	CYLD	mRNA	XR_001435107.1	lncRNA
XM_015100436.1	CYLD	mRNA	XR_001435107.1	lncRNA
XM_015100436.1	CYLD	mRNA	XR_001435107.1	lncRNA
XM_015100436.1	CYLD	mRNA	XR_001435107.1	lncRNA
XM_015100436.1	CYLD	mRNA	XR_001435107.1	lncRNA
XM_015100436.1	CYLD	mRNA	XR_001435107.1	lncRNA
XM_015100436.1	CYLD	mRNA	XR_001435107.1	lncRNA
XM_012173383.2	DHX57	mRNA	XR_001433856.1	lncRNA
XM_012173383.2	DHX57	mRNA	XR_001433856.1	lncRNA
XM_012173383.2	DHX57	mRNA	XR_001433856.1	lncRNA
XM_012173383.2	DHX57	mRNA	XR_001433856.1	lncRNA

XM_012173383.2	DHX57	mRNA	XR_001433856.1	lncRNA
XM_012173383.2	DHX57	mRNA	XR_001433856.1	lncRNA
XM_012173383.2	DHX57	mRNA	XR_001433856.1	lncRNA
XM_012178221.1	CEP120	mRNA	XR_001433856.1	lncRNA
XM_012178221.1	CEP120	mRNA	XR_001433856.1	lncRNA
XM_012178221.1	CEP120	mRNA	XR_001433856.1	lncRNA
XM_012178221.1	CEP120	mRNA	XR_001433856.1	lncRNA
XM_012178221.1	CEP120	mRNA	XR_001433856.1	lncRNA
XM_012178221.1	CEP120	mRNA	XR_001433856.1	lncRNA
XM_012178221.1	CEP120	mRNA	XR_001433856.1	lncRNA
XM_012097468.2	ELF2	mRNA	XR_001435107.1	lncRNA
XM_012097468.2	ELF2	mRNA	XR_001435107.1	lncRNA
XM_012097468.2	ELF2	mRNA	XR_001435107.1	lncRNA
XM_012097468.2	ELF2	mRNA	XR_001435107.1	lncRNA
XM_012097468.2	ELF2	mRNA	XR_001435107.1	lncRNA
XM_012097468.2	ELF2	mRNA	XR_001435107.1	lncRNA
XM_012097468.2	ELF2	mRNA	XR_001435107.1	lncRNA
XM_012099509.2	BRPF1	mRNA	XR_001436817.1	lncRNA
XM_012099509.2	BRPF1	mRNA	XR_001436817.1	lncRNA
XM_012099509.2	BRPF1	mRNA	XR_001436817.1	lncRNA
XM_012099509.2	BRPF1	mRNA	XR_001436817.1	lncRNA
XM_012099509.2	BRPF1	mRNA	XR_001436817.1	lncRNA
XM_012099509.2	BRPF1	mRNA	XR_001436817.1	lncRNA
XM_012099509.2	BRPF1	mRNA	XR_001436817.1	lncRNA
XM_012098756.2	G2E3	mRNA	XR_001434920.1	lncRNA
XM_012098756.2	G2E3	mRNA	XR_001434920.1	lncRNA
XM_012098756.2	G2E3	mRNA	XR_001434920.1	lncRNA
XM_012098756.2	G2E3	mRNA	XR_001434920.1	lncRNA
XM_012098756.2	G2E3	mRNA	XR_001434920.1	lncRNA
XM_012098756.2	G2E3	mRNA	XR_001434920.1	lncRNA
XM_012098756.2	G2E3	mRNA	XR_001434920.1	lncRNA
XM_015095425.1	CUL1	mRNA	XR_001435722.1	lncRNA
XM_015095425.1	CUL1	mRNA	XR_001435722.1	lncRNA
XM_015095425.1	CUL1	mRNA	XR_001435722.1	lncRNA
XM_015095425.1	CUL1	mRNA	XR_001435722.1	lncRNA
XM_015095425.1	CUL1	mRNA	XR_001435722.1	lncRNA
XM_015095425.1	CUL1	mRNA	XR_001435722.1	lncRNA
XM_015095425.1	CUL1	mRNA	XR_001435722.1	lncRNA
XM_012185875.1	LUC7L3	mRNA	XR_001433856.1	lncRNA
XM_012185875.1	LUC7L3	mRNA	XR_001433856.1	lncRNA
XM_012185875.1	LUC7L3	mRNA	XR_001433856.1	lncRNA
XM_012185875.1	LUC7L3	mRNA	XR_001433856.1	lncRNA
XM_012185875.1	LUC7L3	mRNA	XR_001433856.1	lncRNA
XM_012185875.1	LUC7L3	mRNA	XR_001433856.1	lncRNA
XM_012185875.1	LUC7L3	mRNA	XR_001433856.1	lncRNA
XM_015104323.1	SPRTN	mRNA	XR_001436817.1	lncRNA
XM_015104323.1	SPRTN	mRNA	XR_001436817.1	lncRNA
XM_015104323.1	SPRTN	mRNA	XR_001436817.1	lncRNA
XM_015104323.1	SPRTN	mRNA	XR_001436817.1	lncRNA
XM_015104323.1	SPRTN	mRNA	XR_001436817.1	lncRNA
XM_015104323.1	SPRTN	mRNA	XR_001436817.1	lncRNA
XM_015104323.1	SPRTN	mRNA	XR_001436817.1	lncRNA
XM_015100436.1	CYLD	mRNA	XR_001435722.1	lncRNA
XM_015100436.1	CYLD	mRNA	XR_001435722.1	lncRNA

XM_015100436.1	CYLD	mRNA	XR_001435722.1	lncRNA
XM_015100436.1	CYLD	mRNA	XR_001435722.1	lncRNA
XM_015100436.1	CYLD	mRNA	XR_001435722.1	lncRNA
XM_015100436.1	CYLD	mRNA	XR_001435722.1	lncRNA
XM_015100436.1	CYLD	mRNA	XR_001435722.1	lncRNA
XM_012188270.2	CCDC7	mRNA	XR_001434920.1	lncRNA
XM_012188270.2	CCDC7	mRNA	XR_001434920.1	lncRNA
XM_012188270.2	CCDC7	mRNA	XR_001434920.1	lncRNA
XM_012188270.2	CCDC7	mRNA	XR_001434920.1	lncRNA
XM_012188270.2	CCDC7	mRNA	XR_001434920.1	lncRNA
XM_012188270.2	CCDC7	mRNA	XR_001434920.1	lncRNA
XM_012188270.2	CCDC7	mRNA	XR_001434920.1	lncRNA
XM_012181265.2	PLA2G4F	mRNA	XR_001435722.1	lncRNA
XM_012181265.2	PLA2G4F	mRNA	XR_001435722.1	lncRNA
XM_012181265.2	PLA2G4F	mRNA	XR_001435722.1	lncRNA
XM_012181265.2	PLA2G4F	mRNA	XR_001435722.1	lncRNA
XM_012181265.2	PLA2G4F	mRNA	XR_001435722.1	lncRNA
XM_012181265.2	PLA2G4F	mRNA	XR_001435722.1	lncRNA
XM_012181265.2	PLA2G4F	mRNA	XR_001435722.1	lncRNA
XM_012124912.1	KIAA2026	mRNA	XR_001436817.1	lncRNA
XM_012124912.1	KIAA2026	mRNA	XR_001436817.1	lncRNA
XM_012124912.1	KIAA2026	mRNA	XR_001436817.1	lncRNA
XM_012124912.1	KIAA2026	mRNA	XR_001436817.1	lncRNA
XM_012124912.1	KIAA2026	mRNA	XR_001436817.1	lncRNA
XM_012124912.1	KIAA2026	mRNA	XR_001436817.1	lncRNA
XM_012124912.1	KIAA2026	mRNA	XR_001436817.1	lncRNA
XM_012175120.1	PPFIBP1	mRNA	XR_001433856.1	lncRNA
XM_012175120.1	PPFIBP1	mRNA	XR_001433856.1	lncRNA
XM_012175120.1	PPFIBP1	mRNA	XR_001433856.1	lncRNA
XM_012175120.1	PPFIBP1	mRNA	XR_001433856.1	lncRNA
XM_012175120.1	PPFIBP1	mRNA	XR_001433856.1	lncRNA
XM_012175120.1	PPFIBP1	mRNA	XR_001433856.1	lncRNA
XM_012175120.1	PPFIBP1	mRNA	XR_001433856.1	lncRNA
XM_012188270.2	CCDC7	mRNA	XR_001435722.1	lncRNA
XM_012188270.2	CCDC7	mRNA	XR_001435722.1	lncRNA
XM_012188270.2	CCDC7	mRNA	XR_001435722.1	lncRNA
XM_012188270.2	CCDC7	mRNA	XR_001435722.1	lncRNA
XM_012188270.2	CCDC7	mRNA	XR_001435722.1	lncRNA
XM_012188270.2	CCDC7	mRNA	XR_001435722.1	lncRNA
XM_012188270.2	CCDC7	mRNA	XR_001435722.1	lncRNA
XM_012098756.2	G2E3	mRNA	XR_001435722.1	lncRNA
XM_012098756.2	G2E3	mRNA	XR_001435722.1	lncRNA
XM_012098756.2	G2E3	mRNA	XR_001435722.1	lncRNA
XM_012098756.2	G2E3	mRNA	XR_001435722.1	lncRNA
XM_012098756.2	G2E3	mRNA	XR_001435722.1	lncRNA
XM_012098756.2	G2E3	mRNA	XR_001435722.1	lncRNA
XM_012098756.2	G2E3	mRNA	XR_001435722.1	lncRNA
XM_015099212.1	MIIP	mRNA	XR_001434920.1	lncRNA
XM_015099212.1	MIIP	mRNA	XR_001434920.1	lncRNA
XM_015099212.1	MIIP	mRNA	XR_001434920.1	lncRNA
XM_015099212.1	MIIP	mRNA	XR_001434920.1	lncRNA
XM_015099212.1	MIIP	mRNA	XR_001434920.1	lncRNA
XM_015099212.1	MIIP	mRNA	XR_001434920.1	lncRNA
XM_015099212.1	MIIP	mRNA	XR_001434920.1	lncRNA

XM_015092063.1	FUBP1	mRNA	XR_001433856.1	lncRNA
XM_015092063.1	FUBP1	mRNA	XR_001433856.1	lncRNA
XM_015092063.1	FUBP1	mRNA	XR_001433856.1	lncRNA
XM_015092063.1	FUBP1	mRNA	XR_001433856.1	lncRNA
XM_015092063.1	FUBP1	mRNA	XR_001433856.1	lncRNA
XM_015092063.1	FUBP1	mRNA	XR_001433856.1	lncRNA
XM_015092063.1	FUBP1	mRNA	XR_001433856.1	lncRNA
XM_012106946.2	ATP7A	mRNA	XR_001434920.1	lncRNA
XM_012106946.2	ATP7A	mRNA	XR_001434920.1	lncRNA
XM_012106946.2	ATP7A	mRNA	XR_001434920.1	lncRNA
XM_012106946.2	ATP7A	mRNA	XR_001434920.1	lncRNA
XM_012106946.2	ATP7A	mRNA	XR_001434920.1	lncRNA
XM_012106946.2	ATP7A	mRNA	XR_001434920.1	lncRNA
XM_012106946.2	ATP7A	mRNA	XR_001434920.1	lncRNA
XM_015104323.1	SPRTN	mRNA	XR_001435722.1	lncRNA
XM_015104323.1	SPRTN	mRNA	XR_001435722.1	lncRNA
XM_015104323.1	SPRTN	mRNA	XR_001435722.1	lncRNA
XM_015104323.1	SPRTN	mRNA	XR_001435722.1	lncRNA
XM_015104323.1	SPRTN	mRNA	XR_001435722.1	lncRNA
XM_015104323.1	SPRTN	mRNA	XR_001435722.1	lncRNA
XM_015104323.1	SPRTN	mRNA	XR_001435722.1	lncRNA
XM_015091887.1	MYCL	mRNA	XR_001434920.1	lncRNA
XM_015091887.1	MYCL	mRNA	XR_001434920.1	lncRNA
XM_015091887.1	MYCL	mRNA	XR_001434920.1	lncRNA
XM_015091887.1	MYCL	mRNA	XR_001434920.1	lncRNA
XM_015091887.1	MYCL	mRNA	XR_001434920.1	lncRNA
XM_015091887.1	MYCL	mRNA	XR_001434920.1	lncRNA
XM_015091887.1	MYCL	mRNA	XR_001434920.1	lncRNA
XM_015100436.1	CYLD	mRNA	XR_001434920.1	lncRNA
XM_015100436.1	CYLD	mRNA	XR_001434920.1	lncRNA
XM_015100436.1	CYLD	mRNA	XR_001434920.1	lncRNA
XM_015100436.1	CYLD	mRNA	XR_001434920.1	lncRNA
XM_015100436.1	CYLD	mRNA	XR_001434920.1	lncRNA
XM_015100436.1	CYLD	mRNA	XR_001434920.1	lncRNA
XM_015100436.1	CYLD	mRNA	XR_001434920.1	lncRNA
XM_012146540.1	ATG9A	mRNA	XR_001433856.1	lncRNA
XM_012146540.1	ATG9A	mRNA	XR_001433856.1	lncRNA
XM_012146540.1	ATG9A	mRNA	XR_001433856.1	lncRNA
XM_012146540.1	ATG9A	mRNA	XR_001433856.1	lncRNA
XM_012146540.1	ATG9A	mRNA	XR_001433856.1	lncRNA
XM_012146540.1	ATG9A	mRNA	XR_001433856.1	lncRNA
XM_012146540.1	ATG9A	mRNA	XR_001433856.1	lncRNA
XM_012183409.2	PHF3	mRNA	XR_001433856.1	lncRNA
XM_012183409.2	PHF3	mRNA	XR_001433856.1	lncRNA
XM_012183409.2	PHF3	mRNA	XR_001433856.1	lncRNA
XM_012183409.2	PHF3	mRNA	XR_001433856.1	lncRNA
XM_012183409.2	PHF3	mRNA	XR_001433856.1	lncRNA
XM_012183409.2	PHF3	mRNA	XR_001433856.1	lncRNA
XM_012183409.2	PHF3	mRNA	XR_001433856.1	lncRNA
XM_004016880.3	FAM169A	mRNA	XR_001433856.1	lncRNA
XM_004016880.3	FAM169A	mRNA	XR_001433856.1	lncRNA
XM_004016880.3	FAM169A	mRNA	XR_001433856.1	lncRNA
XM_004016880.3	FAM169A	mRNA	XR_001433856.1	lncRNA
XM_004016880.3	FAM169A	mRNA	XR_001433856.1	lncRNA

XM_004016880.3	FAM169A	mRNA	XR_001433856.1	lncRNA
XM_004016880.3	FAM169A	mRNA	XR_001433856.1	lncRNA
XM_015091887.1	MYCL	mRNA	XR_001433856.1	lncRNA
XM_015091887.1	MYCL	mRNA	XR_001433856.1	lncRNA
XM_015091887.1	MYCL	mRNA	XR_001433856.1	lncRNA
XM_015091887.1	MYCL	mRNA	XR_001433856.1	lncRNA
XM_015091887.1	MYCL	mRNA	XR_001433856.1	lncRNA
XM_015091887.1	MYCL	mRNA	XR_001433856.1	lncRNA
XM_015091887.1	MYCL	mRNA	XR_001433856.1	lncRNA
XM_012113353.2	SUSD1	mRNA	XR_001436817.1	lncRNA
XM_012113353.2	SUSD1	mRNA	XR_001436817.1	lncRNA
XM_012113353.2	SUSD1	mRNA	XR_001436817.1	lncRNA
XM_012113353.2	SUSD1	mRNA	XR_001436817.1	lncRNA
XM_012113353.2	SUSD1	mRNA	XR_001436817.1	lncRNA
XM_012113353.2	SUSD1	mRNA	XR_001436817.1	lncRNA
XM_012113353.2	SUSD1	mRNA	XR_001436817.1	lncRNA
XM_015095425.1	CUL1	mRNA	XR_001434920.1	lncRNA
XM_015095425.1	CUL1	mRNA	XR_001434920.1	lncRNA
XM_015095425.1	CUL1	mRNA	XR_001434920.1	lncRNA
XM_015095425.1	CUL1	mRNA	XR_001434920.1	lncRNA
XM_015095425.1	CUL1	mRNA	XR_001434920.1	lncRNA
XM_015095425.1	CUL1	mRNA	XR_001434920.1	lncRNA
XM_015095425.1	CUL1	mRNA	XR_001434920.1	lncRNA
XM_004003021.3	DLG1	mRNA	XR_001040147.1	lncRNA
XM_004003021.3	DLG1	mRNA	XR_001040147.1	lncRNA
XM_004003021.3	DLG1	mRNA	XR_001040147.1	lncRNA
XM_004003021.3	DLG1	mRNA	XR_001040147.1	lncRNA
XM_004003021.3	DLG1	mRNA	XR_001040147.1	lncRNA
XM_004003021.3	DLG1	mRNA	XR_001040147.1	lncRNA
XM_004003021.3	DLG1	mRNA	XR_001040147.1	lncRNA
XM_012184362.2	AKAP11	mRNA	XR_001040147.1	lncRNA
XM_012184362.2	AKAP11	mRNA	XR_001040147.1	lncRNA
XM_012184362.2	AKAP11	mRNA	XR_001040147.1	lncRNA
XM_012184362.2	AKAP11	mRNA	XR_001040147.1	lncRNA
XM_012184362.2	AKAP11	mRNA	XR_001040147.1	lncRNA
XM_012184362.2	AKAP11	mRNA	XR_001040147.1	lncRNA
XM_012184362.2	AKAP11	mRNA	XR_001040147.1	lncRNA
XM_012105333.2	USP54	mRNA	XR_001040147.1	lncRNA
XM_012105333.2	USP54	mRNA	XR_001040147.1	lncRNA
XM_012105333.2	USP54	mRNA	XR_001040147.1	lncRNA
XM_012105333.2	USP54	mRNA	XR_001040147.1	lncRNA
XM_012105333.2	USP54	mRNA	XR_001040147.1	lncRNA
XM_012105333.2	USP54	mRNA	XR_001040147.1	lncRNA
XM_012105333.2	USP54	mRNA	XR_001040147.1	lncRNA
XM_012174704.2	ZBTB39	mRNA	XR_001436390.1	lncRNA
XM_012174704.2	ZBTB39	mRNA	XR_001436390.1	lncRNA
XM_012174704.2	ZBTB39	mRNA	XR_001436390.1	lncRNA
XM_012174704.2	ZBTB39	mRNA	XR_001436390.1	lncRNA
XM_012174704.2	ZBTB39	mRNA	XR_001436390.1	lncRNA
XM_012174704.2	ZBTB39	mRNA	XR_001436390.1	lncRNA
XM_012174704.2	ZBTB39	mRNA	XR_001436390.1	lncRNA
XM_015098082.1	RNASEH2B	mRNA	XR_001436390.1	lncRNA
XM_015098082.1	RNASEH2B	mRNA	XR_001436390.1	lncRNA
XM_015098082.1	RNASEH2B	mRNA	XR_001436390.1	lncRNA

XM_015098082.1	RNASEH2B	mRNA	XR_001436390.1	lncRNA
XM_015098082.1	RNASEH2B	mRNA	XR_001436390.1	lncRNA
XM_015098082.1	RNASEH2B	mRNA	XR_001436390.1	lncRNA
XM_015098082.1	RNASEH2B	mRNA	XR_001436390.1	lncRNA
XM_012105324.2	USP54	mRNA	XR_001434127.1	lncRNA
XM_012105324.2	USP54	mRNA	XR_001434127.1	lncRNA
XM_012105324.2	USP54	mRNA	XR_001434127.1	lncRNA
XM_012105324.2	USP54	mRNA	XR_001434127.1	lncRNA
XM_012105324.2	USP54	mRNA	XR_001434127.1	lncRNA
XM_012105324.2	USP54	mRNA	XR_001434127.1	lncRNA
XM_012105324.2	USP54	mRNA	XR_001434127.1	lncRNA
XM_015101341.1	OTULIN	mRNA	XR_001023627.2	lncRNA
XM_015101341.1	OTULIN	mRNA	XR_001023627.2	lncRNA
XM_015101341.1	OTULIN	mRNA	XR_001023627.2	lncRNA
XM_015101341.1	OTULIN	mRNA	XR_001023627.2	lncRNA
XM_015101341.1	OTULIN	mRNA	XR_001023627.2	lncRNA
XM_015101341.1	OTULIN	mRNA	XR_001023627.2	lncRNA
XM_015101341.1	OTULIN	mRNA	XR_001023627.2	lncRNA
XM_015101341.1	OTULIN	mRNA	XR_001434127.1	lncRNA
XM_015101341.1	OTULIN	mRNA	XR_001434127.1	lncRNA
XM_015101341.1	OTULIN	mRNA	XR_001434127.1	lncRNA
XM_015101341.1	OTULIN	mRNA	XR_001434127.1	lncRNA
XM_015101341.1	OTULIN	mRNA	XR_001434127.1	lncRNA
XM_015101341.1	OTULIN	mRNA	XR_001434127.1	lncRNA
XM_015101341.1	OTULIN	mRNA	XR_001434127.1	lncRNA
XM_012179036.1	FER	mRNA	XR_001023627.2	lncRNA
XM_012179036.1	FER	mRNA	XR_001023627.2	lncRNA
XM_012179036.1	FER	mRNA	XR_001023627.2	lncRNA
XM_012179036.1	FER	mRNA	XR_001023627.2	lncRNA
XM_012179036.1	FER	mRNA	XR_001023627.2	lncRNA
XM_012179036.1	FER	mRNA	XR_001023627.2	lncRNA
XM_012179036.1	FER	mRNA	XR_001023627.2	lncRNA
XM_012097555.2	RAPGEF2	mRNA	XR_001434127.1	lncRNA
XM_012097555.2	RAPGEF2	mRNA	XR_001434127.1	lncRNA
XM_012097555.2	RAPGEF2	mRNA	XR_001434127.1	lncRNA
XM_012097555.2	RAPGEF2	mRNA	XR_001434127.1	lncRNA
XM_012097555.2	RAPGEF2	mRNA	XR_001434127.1	lncRNA
XM_012097555.2	RAPGEF2	mRNA	XR_001434127.1	lncRNA
XM_012097555.2	RAPGEF2	mRNA	XR_001434127.1	lncRNA
XM_015099563.1	PARD3	mRNA	TCONS_00049802	lncRNA
XM_015099563.1	PARD3	mRNA	TCONS_00049802	lncRNA
XM_015099563.1	PARD3	mRNA	TCONS_00049802	lncRNA
XM_015099563.1	PARD3	mRNA	TCONS_00049802	lncRNA
XM_015099563.1	PARD3	mRNA	TCONS_00049802	lncRNA
XM_015099563.1	PARD3	mRNA	TCONS_00049802	lncRNA
XM_015099563.1	PARD3	mRNA	TCONS_00049802	lncRNA
XM_012179036.1	FER	mRNA	XR_001434127.1	lncRNA
XM_012179036.1	FER	mRNA	XR_001434127.1	lncRNA
XM_012179036.1	FER	mRNA	XR_001434127.1	lncRNA
XM_012179036.1	FER	mRNA	XR_001434127.1	lncRNA
XM_012179036.1	FER	mRNA	XR_001434127.1	lncRNA
XM_012179036.1	FER	mRNA	XR_001434127.1	lncRNA
XM_012179036.1	FER	mRNA	XR_001434127.1	lncRNA
XM_012180234.2	CNOT6L	mRNA	XR_001436065.1	lncRNA

XM_012180234.2	CNOT6L	mRNA	XR_001436065.1	lncRNA
XM_012180234.2	CNOT6L	mRNA	XR_001436065.1	lncRNA
XM_012180234.2	CNOT6L	mRNA	XR_001436065.1	lncRNA
XM_012180234.2	CNOT6L	mRNA	XR_001436065.1	lncRNA
XM_012180234.2	CNOT6L	mRNA	XR_001436065.1	lncRNA
XM_012180234.2	CNOT6L	mRNA	XR_001436065.1	lncRNA
XM_004017655.3	PRR14L	mRNA	XR_001024230.1	lncRNA
XM_004017655.3	PRR14L	mRNA	XR_001024230.1	lncRNA
XM_004017655.3	PRR14L	mRNA	XR_001024230.1	lncRNA
XM_004017655.3	PRR14L	mRNA	XR_001024230.1	lncRNA
XM_004017655.3	PRR14L	mRNA	XR_001024230.1	lncRNA
XM_004017655.3	PRR14L	mRNA	XR_001024230.1	lncRNA
XM_004017655.3	PRR14L	mRNA	XR_001024230.1	lncRNA
XM_012097365.2	SH3D19	mRNA	XR_001435726.1	lncRNA
XM_012097365.2	SH3D19	mRNA	XR_001435726.1	lncRNA
XM_012097365.2	SH3D19	mRNA	XR_001435726.1	lncRNA
XM_012097365.2	SH3D19	mRNA	XR_001435726.1	lncRNA
XM_012097365.2	SH3D19	mRNA	XR_001435726.1	lncRNA
XM_012097365.2	SH3D19	mRNA	XR_001435726.1	lncRNA
XM_012097365.2	SH3D19	mRNA	XR_001435726.1	lncRNA
XM_015095237.1	DMTF1	mRNA	XR_001435723.1	lncRNA
XM_015095237.1	DMTF1	mRNA	XR_001435723.1	lncRNA
XM_015095237.1	DMTF1	mRNA	XR_001435723.1	lncRNA
XM_015095237.1	DMTF1	mRNA	XR_001435723.1	lncRNA
XM_015095237.1	DMTF1	mRNA	XR_001435723.1	lncRNA
XM_015095237.1	DMTF1	mRNA	XR_001435723.1	lncRNA
XM_015095237.1	DMTF1	mRNA	XR_001435723.1	lncRNA
XM_012185338.2	ZNF207	mRNA	XR_001024230.1	lncRNA
XM_012185338.2	ZNF207	mRNA	XR_001024230.1	lncRNA
XM_012185338.2	ZNF207	mRNA	XR_001024230.1	lncRNA
XM_012185338.2	ZNF207	mRNA	XR_001024230.1	lncRNA
XM_012185338.2	ZNF207	mRNA	XR_001024230.1	lncRNA
XM_012185338.2	ZNF207	mRNA	XR_001024230.1	lncRNA
XM_012185338.2	ZNF207	mRNA	XR_001024230.1	lncRNA
XM_015095237.1	DMTF1	mRNA	XR_001435726.1	lncRNA
XM_015095237.1	DMTF1	mRNA	XR_001435726.1	lncRNA
XM_015095237.1	DMTF1	mRNA	XR_001435726.1	lncRNA
XM_015095237.1	DMTF1	mRNA	XR_001435726.1	lncRNA
XM_015095237.1	DMTF1	mRNA	XR_001435726.1	lncRNA
XM_015095237.1	DMTF1	mRNA	XR_001435726.1	lncRNA
XM_015095237.1	DMTF1	mRNA	XR_001435726.1	lncRNA
XM_004017814.3	DNAJA4	mRNA	XR_001436064.1	lncRNA
XM_004017814.3	DNAJA4	mRNA	XR_001436064.1	lncRNA
XM_004017814.3	DNAJA4	mRNA	XR_001436064.1	lncRNA
XM_004017814.3	DNAJA4	mRNA	XR_001436064.1	lncRNA
XM_004017814.3	DNAJA4	mRNA	XR_001436064.1	lncRNA
XM_004017814.3	DNAJA4	mRNA	XR_001436064.1	lncRNA
XM_004017814.3	DNAJA4	mRNA	XR_001436064.1	lncRNA
XM_012185338.2	ZNF207	mRNA	XR_001435723.1	lncRNA
XM_012185338.2	ZNF207	mRNA	XR_001435723.1	lncRNA
XM_012185338.2	ZNF207	mRNA	XR_001435723.1	lncRNA
XM_012185338.2	ZNF207	mRNA	XR_001435723.1	lncRNA
XM_012185338.2	ZNF207	mRNA	XR_001435723.1	lncRNA
XM_012185338.2	ZNF207	mRNA	XR_001435723.1	lncRNA

XM_012185338.2	ZNF207	mRNA	XR_001435723.1	lncRNA
XM_012173553.2	STRN	mRNA	XR_001435723.1	lncRNA
XM_012173553.2	STRN	mRNA	XR_001435723.1	lncRNA
XM_012173553.2	STRN	mRNA	XR_001435723.1	lncRNA
XM_012173553.2	STRN	mRNA	XR_001435723.1	lncRNA
XM_012173553.2	STRN	mRNA	XR_001435723.1	lncRNA
XM_012173553.2	STRN	mRNA	XR_001435723.1	lncRNA
XM_012173553.2	STRN	mRNA	XR_001435723.1	lncRNA
XM_012097365.2	SH3D19	mRNA	XR_001435723.1	lncRNA
XM_012097365.2	SH3D19	mRNA	XR_001435723.1	lncRNA
XM_012097365.2	SH3D19	mRNA	XR_001435723.1	lncRNA
XM_012097365.2	SH3D19	mRNA	XR_001435723.1	lncRNA
XM_012097365.2	SH3D19	mRNA	XR_001435723.1	lncRNA
XM_012097365.2	SH3D19	mRNA	XR_001435723.1	lncRNA
XM_012097365.2	SH3D19	mRNA	XR_001435723.1	lncRNA
XM_012180234.2	CNOT6L	mRNA	XR_001436064.1	lncRNA
XM_012180234.2	CNOT6L	mRNA	XR_001436064.1	lncRNA
XM_012180234.2	CNOT6L	mRNA	XR_001436064.1	lncRNA
XM_012180234.2	CNOT6L	mRNA	XR_001436064.1	lncRNA
XM_012180234.2	CNOT6L	mRNA	XR_001436064.1	lncRNA
XM_012180234.2	CNOT6L	mRNA	XR_001436064.1	lncRNA
XM_012180234.2	CNOT6L	mRNA	XR_001436064.1	lncRNA
XM_012187413.1	NRIP1	mRNA	XR_001435723.1	lncRNA
XM_012187413.1	NRIP1	mRNA	XR_001435723.1	lncRNA
XM_012187413.1	NRIP1	mRNA	XR_001435723.1	lncRNA
XM_012187413.1	NRIP1	mRNA	XR_001435723.1	lncRNA
XM_012187413.1	NRIP1	mRNA	XR_001435723.1	lncRNA
XM_012187413.1	NRIP1	mRNA	XR_001435723.1	lncRNA
XM_012187413.1	NRIP1	mRNA	XR_001435723.1	lncRNA
XM_012103799.1	NEDD4L	mRNA	XR_001436064.1	lncRNA
XM_012103799.1	NEDD4L	mRNA	XR_001436064.1	lncRNA
XM_012103799.1	NEDD4L	mRNA	XR_001436064.1	lncRNA
XM_012103799.1	NEDD4L	mRNA	XR_001436064.1	lncRNA
XM_012103799.1	NEDD4L	mRNA	XR_001436064.1	lncRNA
XM_012103799.1	NEDD4L	mRNA	XR_001436064.1	lncRNA
XM_012103799.1	NEDD4L	mRNA	XR_001436064.1	lncRNA
XM_012180234.2	CNOT6L	mRNA	XR_001435723.1	lncRNA
XM_012180234.2	CNOT6L	mRNA	XR_001435723.1	lncRNA
XM_012180234.2	CNOT6L	mRNA	XR_001435723.1	lncRNA
XM_012180234.2	CNOT6L	mRNA	XR_001435723.1	lncRNA
XM_012180234.2	CNOT6L	mRNA	XR_001435723.1	lncRNA
XM_012180234.2	CNOT6L	mRNA	XR_001435723.1	lncRNA
XM_012180234.2	CNOT6L	mRNA	XR_001435723.1	lncRNA
XM_012095510.2	SIDT2	mRNA	XR_001024966.2	lncRNA
XM_012095510.2	SIDT2	mRNA	XR_001024966.2	lncRNA
XM_012095510.2	SIDT2	mRNA	XR_001024966.2	lncRNA
XM_012095510.2	SIDT2	mRNA	XR_001024966.2	lncRNA
XM_012095510.2	SIDT2	mRNA	XR_001024966.2	lncRNA
XM_012095510.2	SIDT2	mRNA	XR_001024966.2	lncRNA
XM_012187827.1	GIGYF2	mRNA	XR_001436612.1	lncRNA
XM_012187827.1	GIGYF2	mRNA	XR_001436612.1	lncRNA
XM_012187827.1	GIGYF2	mRNA	XR_001436612.1	lncRNA
XM_012187827.1	GIGYF2	mRNA	XR_001436612.1	lncRNA
XM_012187827.1	GIGYF2	mRNA	XR_001436612.1	lncRNA

XM_012187827.1	GIGYF2	mRNA	XR_001436612.1	lncRNA
XM_012098616.2	TBC1L1B	mRNA	XR_001436612.1	lncRNA
XM_012098616.2	TBC1L1B	mRNA	XR_001436612.1	lncRNA
XM_012098616.2	TBC1L1B	mRNA	XR_001436612.1	lncRNA
XM_012098616.2	TBC1L1B	mRNA	XR_001436612.1	lncRNA
XM_012098616.2	TBC1L1B	mRNA	XR_001436612.1	lncRNA
XM_012098616.2	TBC1L1B	mRNA	XR_001436612.1	lncRNA
XM_012189777.1	CHD9	mRNA	XR_001023303.2	lncRNA
XM_012189777.1	CHD9	mRNA	XR_001023303.2	lncRNA
XM_012189777.1	CHD9	mRNA	XR_001023303.2	lncRNA
XM_012189777.1	CHD9	mRNA	XR_001023303.2	lncRNA
XM_012189777.1	CHD9	mRNA	XR_001023303.2	lncRNA
XM_012189777.1	CHD9	mRNA	XR_001023303.2	lncRNA
XM_004011768.3	SAMD12	mRNA	XR_001041893.2	lncRNA
XM_004011768.3	SAMD12	mRNA	XR_001041893.2	lncRNA
XM_004011768.3	SAMD12	mRNA	XR_001041893.2	lncRNA
XM_004011768.3	SAMD12	mRNA	XR_001041893.2	lncRNA
XM_004011768.3	SAMD12	mRNA	XR_001041893.2	lncRNA
XM_004011768.3	SAMD12	mRNA	XR_001041893.2	lncRNA
XM_012174704.2	ZBTB39	mRNA	XR_001023303.2	lncRNA
XM_012174704.2	ZBTB39	mRNA	XR_001023303.2	lncRNA
XM_012174704.2	ZBTB39	mRNA	XR_001023303.2	lncRNA
XM_012174704.2	ZBTB39	mRNA	XR_001023303.2	lncRNA
XM_012174704.2	ZBTB39	mRNA	XR_001023303.2	lncRNA
XM_012174704.2	ZBTB39	mRNA	XR_001023303.2	lncRNA
XM_004011768.3	SAMD12	mRNA	XR_001023303.2	lncRNA
XM_004011768.3	SAMD12	mRNA	XR_001023303.2	lncRNA
XM_004011768.3	SAMD12	mRNA	XR_001023303.2	lncRNA
XM_004011768.3	SAMD12	mRNA	XR_001023303.2	lncRNA
XM_004011768.3	SAMD12	mRNA	XR_001023303.2	lncRNA
XM_004011768.3	SAMD12	mRNA	XR_001023303.2	lncRNA
XM_012097035.1	C16H5orf42	mRNA	XR_001041893.2	lncRNA
XM_012097035.1	C16H5orf42	mRNA	XR_001041893.2	lncRNA
XM_012097035.1	C16H5orf42	mRNA	XR_001041893.2	lncRNA
XM_012097035.1	C16H5orf42	mRNA	XR_001041893.2	lncRNA
XM_012097035.1	C16H5orf42	mRNA	XR_001041893.2	lncRNA
XM_012097035.1	C16H5orf42	mRNA	XR_001041893.2	lncRNA
XM_015093416.1	RIF1	mRNA	XR_001023303.2	lncRNA
XM_015093416.1	RIF1	mRNA	XR_001023303.2	lncRNA
XM_015093416.1	RIF1	mRNA	XR_001023303.2	lncRNA
XM_015093416.1	RIF1	mRNA	XR_001023303.2	lncRNA
XM_015093416.1	RIF1	mRNA	XR_001023303.2	lncRNA
XM_015093416.1	RIF1	mRNA	XR_001023303.2	lncRNA
XM_012187413.1	NRIP1	mRNA	XR_001433837.1	lncRNA
XM_012187413.1	NRIP1	mRNA	XR_001433837.1	lncRNA
XM_012187413.1	NRIP1	mRNA	XR_001433837.1	lncRNA
XM_012187413.1	NRIP1	mRNA	XR_001433837.1	lncRNA
XM_012187413.1	NRIP1	mRNA	XR_001433837.1	lncRNA
XM_012187413.1	NRIP1	mRNA	XR_001433837.1	lncRNA
XM_004017788.3	ZFANL3	mRNA	XR_001433837.1	lncRNA
XM_004017788.3	ZFANL3	mRNA	XR_001433837.1	lncRNA
XM_004017788.3	ZFANL3	mRNA	XR_001433837.1	lncRNA
XM_004017788.3	ZFANL3	mRNA	XR_001433837.1	lncRNA
XM_004017788.3	ZFANL3	mRNA	XR_001433837.1	lncRNA

XM_004017788.3	ZFANL3	mRNA	XR_001433837.1	lncRNA
XM_012182450.2	IBTK	mRNA	XR_001433837.1	lncRNA
XM_012182450.2	IBTK	mRNA	XR_001433837.1	lncRNA
XM_012182450.2	IBTK	mRNA	XR_001433837.1	lncRNA
XM_012182450.2	IBTK	mRNA	XR_001433837.1	lncRNA
XM_012182450.2	IBTK	mRNA	XR_001433837.1	lncRNA
XM_012182450.2	IBTK	mRNA	XR_001433837.1	lncRNA
XM_012098903.1	PPIP5K1	mRNA	XR_001436612.1	lncRNA
XM_012098903.1	PPIP5K1	mRNA	XR_001436612.1	lncRNA
XM_012098903.1	PPIP5K1	mRNA	XR_001436612.1	lncRNA
XM_012098903.1	PPIP5K1	mRNA	XR_001436612.1	lncRNA
XM_012098903.1	PPIP5K1	mRNA	XR_001436612.1	lncRNA
XM_012098903.1	PPIP5K1	mRNA	XR_001436612.1	lncRNA
XM_015091887.1	MYCL	mRNA	XR_001436612.1	lncRNA
XM_015091887.1	MYCL	mRNA	XR_001436612.1	lncRNA
XM_015091887.1	MYCL	mRNA	XR_001436612.1	lncRNA
XM_015091887.1	MYCL	mRNA	XR_001436612.1	lncRNA
XM_015091887.1	MYCL	mRNA	XR_001436612.1	lncRNA
XM_015091887.1	MYCL	mRNA	XR_001436612.1	lncRNA
XM_012142659.2	ALS2	mRNA	XR_001436612.1	lncRNA
XM_012142659.2	ALS2	mRNA	XR_001436612.1	lncRNA
XM_012142659.2	ALS2	mRNA	XR_001436612.1	lncRNA
XM_012142659.2	ALS2	mRNA	XR_001436612.1	lncRNA
XM_012142659.2	ALS2	mRNA	XR_001436612.1	lncRNA
XM_012142659.2	ALS2	mRNA	XR_001436612.1	lncRNA
XM_015099563.1	PARD3	mRNA	XR_001041893.2	lncRNA
XM_015099563.1	PARD3	mRNA	XR_001041893.2	lncRNA
XM_015099563.1	PARD3	mRNA	XR_001041893.2	lncRNA
XM_015099563.1	PARD3	mRNA	XR_001041893.2	lncRNA
XM_015099563.1	PARD3	mRNA	XR_001041893.2	lncRNA
XM_015099563.1	PARD3	mRNA	XR_001041893.2	lncRNA
XM_015099738.1	LOC101122805	mRNA	XR_001041893.2	lncRNA
XM_015099738.1	LOC101122805	mRNA	XR_001041893.2	lncRNA
XM_015099738.1	LOC101122805	mRNA	XR_001041893.2	lncRNA
XM_015099738.1	LOC101122805	mRNA	XR_001041893.2	lncRNA
XM_015099738.1	LOC101122805	mRNA	XR_001041893.2	lncRNA
XM_015099738.1	LOC101122805	mRNA	XR_001041893.2	lncRNA
XM_004010552.3	VPS13C	mRNA	XR_001023303.2	lncRNA
XM_004010552.3	VPS13C	mRNA	XR_001023303.2	lncRNA
XM_004010552.3	VPS13C	mRNA	XR_001023303.2	lncRNA
XM_004010552.3	VPS13C	mRNA	XR_001023303.2	lncRNA
XM_004010552.3	VPS13C	mRNA	XR_001023303.2	lncRNA
XM_004010552.3	VPS13C	mRNA	XR_001023303.2	lncRNA
XM_015096383.1	SGMS2	mRNA	XR_001436390.1	lncRNA
XM_015096383.1	SGMS2	mRNA	XR_001436390.1	lncRNA
XM_015096383.1	SGMS2	mRNA	XR_001436390.1	lncRNA
XM_015096383.1	SGMS2	mRNA	XR_001436390.1	lncRNA
XM_015096383.1	SGMS2	mRNA	XR_001436390.1	lncRNA
XM_015096383.1	SGMS2	mRNA	XR_001436390.1	lncRNA
XM_004011835.3	RAD54B	mRNA	XR_001436390.1	lncRNA
XM_004011835.3	RAD54B	mRNA	XR_001436390.1	lncRNA
XM_004011835.3	RAD54B	mRNA	XR_001436390.1	lncRNA
XM_004011835.3	RAD54B	mRNA	XR_001436390.1	lncRNA
XM_004011835.3	RAD54B	mRNA	XR_001436390.1	lncRNA

XM_004011835.3	RAD54B	mRNA	XR_001436390.1	lncRNA
XM_015096932.1	THBS1	mRNA	XR_001436390.1	lncRNA
XM_015096932.1	THBS1	mRNA	XR_001436390.1	lncRNA
XM_015096932.1	THBS1	mRNA	XR_001436390.1	lncRNA
XM_015096932.1	THBS1	mRNA	XR_001436390.1	lncRNA
XM_015096932.1	THBS1	mRNA	XR_001436390.1	lncRNA
XM_015096932.1	THBS1	mRNA	XR_001436390.1	lncRNA
XM_012096817.1	ERBB2IP	mRNA	XR_001436390.1	lncRNA
XM_012096817.1	ERBB2IP	mRNA	XR_001436390.1	lncRNA
XM_012096817.1	ERBB2IP	mRNA	XR_001436390.1	lncRNA
XM_012096817.1	ERBB2IP	mRNA	XR_001436390.1	lncRNA
XM_012096817.1	ERBB2IP	mRNA	XR_001436390.1	lncRNA
XM_012096817.1	ERBB2IP	mRNA	XR_001436390.1	lncRNA
XM_012129647.1	NEK1	mRNA	XR_001436390.1	lncRNA
XM_012129647.1	NEK1	mRNA	XR_001436390.1	lncRNA
XM_012129647.1	NEK1	mRNA	XR_001436390.1	lncRNA
XM_012129647.1	NEK1	mRNA	XR_001436390.1	lncRNA
XM_012129647.1	NEK1	mRNA	XR_001436390.1	lncRNA
XM_012129647.1	NEK1	mRNA	XR_001436390.1	lncRNA
XM_012174509.2	ADGRL2	mRNA	XR_001436390.1	lncRNA
XM_012174509.2	ADGRL2	mRNA	XR_001436390.1	lncRNA
XM_012174509.2	ADGRL2	mRNA	XR_001436390.1	lncRNA
XM_012174509.2	ADGRL2	mRNA	XR_001436390.1	lncRNA
XM_012174509.2	ADGRL2	mRNA	XR_001436390.1	lncRNA
XM_012174509.2	ADGRL2	mRNA	XR_001436390.1	lncRNA
XM_012106889.2	TAF1	mRNA	XR_001436390.1	lncRNA
XM_012106889.2	TAF1	mRNA	XR_001436390.1	lncRNA
XM_012106889.2	TAF1	mRNA	XR_001436390.1	lncRNA
XM_012106889.2	TAF1	mRNA	XR_001436390.1	lncRNA
XM_012106889.2	TAF1	mRNA	XR_001436390.1	lncRNA
XM_012106889.2	TAF1	mRNA	XR_001436390.1	lncRNA
XM_012181265.2	PLA2G4F	mRNA	XR_001023627.2	lncRNA
XM_012181265.2	PLA2G4F	mRNA	XR_001023627.2	lncRNA
XM_012181265.2	PLA2G4F	mRNA	XR_001023627.2	lncRNA
XM_012181265.2	PLA2G4F	mRNA	XR_001023627.2	lncRNA
XM_012181265.2	PLA2G4F	mRNA	XR_001023627.2	lncRNA
XM_012181265.2	PLA2G4F	mRNA	XR_001023627.2	lncRNA
XM_012146156.1	USP37	mRNA	TCONS_00049802	lncRNA
XM_012146156.1	USP37	mRNA	TCONS_00049802	lncRNA
XM_012146156.1	USP37	mRNA	TCONS_00049802	lncRNA
XM_012146156.1	USP37	mRNA	TCONS_00049802	lncRNA
XM_012146156.1	USP37	mRNA	TCONS_00049802	lncRNA
XM_012146156.1	USP37	mRNA	TCONS_00049802	lncRNA
XM_012099341.2	CLASP2	mRNA	TCONS_00049802	lncRNA
XM_012099341.2	CLASP2	mRNA	TCONS_00049802	lncRNA
XM_012099341.2	CLASP2	mRNA	TCONS_00049802	lncRNA
XM_012099341.2	CLASP2	mRNA	TCONS_00049802	lncRNA
XM_012099341.2	CLASP2	mRNA	TCONS_00049802	lncRNA
XM_012099341.2	CLASP2	mRNA	TCONS_00049802	lncRNA
XM_015099337.1	RALGPS2	mRNA	XR_001434127.1	lncRNA
XM_015099337.1	RALGPS2	mRNA	XR_001434127.1	lncRNA
XM_015099337.1	RALGPS2	mRNA	XR_001434127.1	lncRNA
XM_015099337.1	RALGPS2	mRNA	XR_001434127.1	lncRNA
XM_015099337.1	RALGPS2	mRNA	XR_001434127.1	lncRNA

XM_015099337.1	RALGPS2	mRNA	XR_001434127.1	lncRNA
XM_012107808.1	NFYC	mRNA	XR_001023627.2	lncRNA
XM_012107808.1	NFYC	mRNA	XR_001023627.2	lncRNA
XM_012107808.1	NFYC	mRNA	XR_001023627.2	lncRNA
XM_012107808.1	NFYC	mRNA	XR_001023627.2	lncRNA
XM_012107808.1	NFYC	mRNA	XR_001023627.2	lncRNA
XM_012107808.1	NFYC	mRNA	XR_001023627.2	lncRNA
XM_012188274.2	CCDC7	mRNA	XR_001023627.2	lncRNA
XM_012188274.2	CCDC7	mRNA	XR_001023627.2	lncRNA
XM_012188274.2	CCDC7	mRNA	XR_001023627.2	lncRNA
XM_012188274.2	CCDC7	mRNA	XR_001023627.2	lncRNA
XM_012188274.2	CCDC7	mRNA	XR_001023627.2	lncRNA
XM_012188274.2	CCDC7	mRNA	XR_001023627.2	lncRNA
XM_015099337.1	RALGPS2	mRNA	XR_001023627.2	lncRNA
XM_015099337.1	RALGPS2	mRNA	XR_001023627.2	lncRNA
XM_015099337.1	RALGPS2	mRNA	XR_001023627.2	lncRNA
XM_015099337.1	RALGPS2	mRNA	XR_001023627.2	lncRNA
XM_015099337.1	RALGPS2	mRNA	XR_001023627.2	lncRNA
XM_015099337.1	RALGPS2	mRNA	XR_001023627.2	lncRNA
XM_012107808.1	NFYC	mRNA	TCONS_00049802	lncRNA
XM_012107808.1	NFYC	mRNA	TCONS_00049802	lncRNA
XM_012107808.1	NFYC	mRNA	TCONS_00049802	lncRNA
XM_012107808.1	NFYC	mRNA	TCONS_00049802	lncRNA
XM_012107808.1	NFYC	mRNA	TCONS_00049802	lncRNA
XM_012107808.1	NFYC	mRNA	TCONS_00049802	lncRNA
XM_012188274.2	CCDC7	mRNA	XR_001434127.1	lncRNA
XM_012188274.2	CCDC7	mRNA	XR_001434127.1	lncRNA
XM_012188274.2	CCDC7	mRNA	XR_001434127.1	lncRNA
XM_012188274.2	CCDC7	mRNA	XR_001434127.1	lncRNA
XM_012188274.2	CCDC7	mRNA	XR_001434127.1	lncRNA
XM_012188274.2	CCDC7	mRNA	XR_001434127.1	lncRNA
XM_015099337.1	RALGPS2	mRNA	TCONS_00049802	lncRNA
XM_015099337.1	RALGPS2	mRNA	TCONS_00049802	lncRNA
XM_015099337.1	RALGPS2	mRNA	TCONS_00049802	lncRNA
XM_015099337.1	RALGPS2	mRNA	TCONS_00049802	lncRNA
XM_015099337.1	RALGPS2	mRNA	TCONS_00049802	lncRNA
XM_015099337.1	RALGPS2	mRNA	TCONS_00049802	lncRNA
XM_012185875.1	LUC7L3	mRNA	TCONS_00049802	lncRNA
XM_012185875.1	LUC7L3	mRNA	TCONS_00049802	lncRNA
XM_012185875.1	LUC7L3	mRNA	TCONS_00049802	lncRNA
XM_012185875.1	LUC7L3	mRNA	TCONS_00049802	lncRNA
XM_012185875.1	LUC7L3	mRNA	TCONS_00049802	lncRNA
XM_012185875.1	LUC7L3	mRNA	TCONS_00049802	lncRNA
XM_012181265.2	PLA2G4F	mRNA	XR_001434127.1	lncRNA
XM_012181265.2	PLA2G4F	mRNA	XR_001434127.1	lncRNA
XM_012181265.2	PLA2G4F	mRNA	XR_001434127.1	lncRNA
XM_012181265.2	PLA2G4F	mRNA	XR_001434127.1	lncRNA
XM_012181265.2	PLA2G4F	mRNA	XR_001434127.1	lncRNA
XM_012181265.2	PLA2G4F	mRNA	XR_001434127.1	lncRNA
XM_004009738.3	PPARGC1A	mRNA	XR_001433837.1	lncRNA
XM_004009738.3	PPARGC1A	mRNA	XR_001433837.1	lncRNA
XM_004009738.3	PPARGC1A	mRNA	XR_001433837.1	lncRNA
XM_004009738.3	PPARGC1A	mRNA	XR_001433837.1	lncRNA
XM_004009738.3	PPARGC1A	mRNA	XR_001433837.1	lncRNA

XM_004009738.3	PPARGC1A	mRNA	XR_001433837.1	lncRNA
XM_012174536.2	KIF21A	mRNA	XR_001433837.1	lncRNA
XM_012174536.2	KIF21A	mRNA	XR_001433837.1	lncRNA
XM_012174536.2	KIF21A	mRNA	XR_001433837.1	lncRNA
XM_012174536.2	KIF21A	mRNA	XR_001433837.1	lncRNA
XM_012174536.2	KIF21A	mRNA	XR_001433837.1	lncRNA
XM_012174536.2	KIF21A	mRNA	XR_001433837.1	lncRNA
XM_012097035.1	C16H5orf42	mRNA	XR_001433837.1	lncRNA
XM_012097035.1	C16H5orf42	mRNA	XR_001433837.1	lncRNA
XM_012097035.1	C16H5orf42	mRNA	XR_001433837.1	lncRNA
XM_012097035.1	C16H5orf42	mRNA	XR_001433837.1	lncRNA
XM_012097035.1	C16H5orf42	mRNA	XR_001433837.1	lncRNA
XM_012097035.1	C16H5orf42	mRNA	XR_001433837.1	lncRNA
XM_012179825.2	N4BP2	mRNA	XR_001433837.1	lncRNA
XM_012179825.2	N4BP2	mRNA	XR_001433837.1	lncRNA
XM_012179825.2	N4BP2	mRNA	XR_001433837.1	lncRNA
XM_012179825.2	N4BP2	mRNA	XR_001433837.1	lncRNA
XM_012179825.2	N4BP2	mRNA	XR_001433837.1	lncRNA
XM_012179825.2	N4BP2	mRNA	XR_001433837.1	lncRNA
XM_004017655.3	PRR14L	mRNA	XR_001436612.1	lncRNA
XM_004017655.3	PRR14L	mRNA	XR_001436612.1	lncRNA
XM_004017655.3	PRR14L	mRNA	XR_001436612.1	lncRNA
XM_004017655.3	PRR14L	mRNA	XR_001436612.1	lncRNA
XM_004017655.3	PRR14L	mRNA	XR_001436612.1	lncRNA
XM_004017655.3	PRR14L	mRNA	XR_001436612.1	lncRNA
XM_015095237.1	DMTF1	mRNA	XR_001436612.1	lncRNA
XM_015095237.1	DMTF1	mRNA	XR_001436612.1	lncRNA
XM_015095237.1	DMTF1	mRNA	XR_001436612.1	lncRNA
XM_015095237.1	DMTF1	mRNA	XR_001436612.1	lncRNA
XM_015095237.1	DMTF1	mRNA	XR_001436612.1	lncRNA
XM_015095237.1	DMTF1	mRNA	XR_001436612.1	lncRNA
XM_015103466.1	TACC2	mRNA	XR_001436612.1	lncRNA
XM_015103466.1	TACC2	mRNA	XR_001436612.1	lncRNA
XM_015103466.1	TACC2	mRNA	XR_001436612.1	lncRNA
XM_015103466.1	TACC2	mRNA	XR_001436612.1	lncRNA
XM_015103466.1	TACC2	mRNA	XR_001436612.1	lncRNA
XM_015103466.1	TACC2	mRNA	XR_001436612.1	lncRNA
XM_015103783.1	WDR7	mRNA	XR_001436612.1	lncRNA
XM_015103783.1	WDR7	mRNA	XR_001436612.1	lncRNA
XM_015103783.1	WDR7	mRNA	XR_001436612.1	lncRNA
XM_015103783.1	WDR7	mRNA	XR_001436612.1	lncRNA
XM_015103783.1	WDR7	mRNA	XR_001436612.1	lncRNA
XM_015103783.1	WDR7	mRNA	XR_001436612.1	lncRNA
XM_012098812.2	RALGAPA1	mRNA	XR_001436612.1	lncRNA
XM_012098812.2	RALGAPA1	mRNA	XR_001436612.1	lncRNA
XM_012098812.2	RALGAPA1	mRNA	XR_001436612.1	lncRNA
XM_012098812.2	RALGAPA1	mRNA	XR_001436612.1	lncRNA
XM_012098812.2	RALGAPA1	mRNA	XR_001436612.1	lncRNA
XM_012098812.2	RALGAPA1	mRNA	XR_001436612.1	lncRNA
XM_012186362.1	PITPNC1	mRNA	XR_001023303.2	lncRNA
XM_012186362.1	PITPNC1	mRNA	XR_001023303.2	lncRNA
XM_012186362.1	PITPNC1	mRNA	XR_001023303.2	lncRNA
XM_012186362.1	PITPNC1	mRNA	XR_001023303.2	lncRNA
XM_012186362.1	PITPNC1	mRNA	XR_001023303.2	lncRNA

XM_012186362.1	PITPNC1	mRNA	XR_001023303.2	lncRNA
XM_004017729.3	CHL1	mRNA	XR_001024966.2	lncRNA
XM_004017729.3	CHL1	mRNA	XR_001024966.2	lncRNA
XM_004017729.3	CHL1	mRNA	XR_001024966.2	lncRNA
XM_004017729.3	CHL1	mRNA	XR_001024966.2	lncRNA
XM_004017729.3	CHL1	mRNA	XR_001024966.2	lncRNA
XM_004017729.3	CHL1	mRNA	XR_001024966.2	lncRNA
XM_012143523.2	INO80D	mRNA	XR_001041893.2	lncRNA
XM_012143523.2	INO80D	mRNA	XR_001041893.2	lncRNA
XM_012143523.2	INO80D	mRNA	XR_001041893.2	lncRNA
XM_012143523.2	INO80D	mRNA	XR_001041893.2	lncRNA
XM_012143523.2	INO80D	mRNA	XR_001041893.2	lncRNA
XM_012143523.2	INO80D	mRNA	XR_001041893.2	lncRNA
XM_015093216.1	TTC39B	mRNA	XR_001024230.1	lncRNA
XM_015093216.1	TTC39B	mRNA	XR_001024230.1	lncRNA
XM_015093216.1	TTC39B	mRNA	XR_001024230.1	lncRNA
XM_015093216.1	TTC39B	mRNA	XR_001024230.1	lncRNA
XM_015093216.1	TTC39B	mRNA	XR_001024230.1	lncRNA
XM_015093216.1	TTC39B	mRNA	XR_001024230.1	lncRNA
XM_015093216.1	TTC39B	mRNA	XR_001436065.1	lncRNA
XM_015093216.1	TTC39B	mRNA	XR_001436065.1	lncRNA
XM_015093216.1	TTC39B	mRNA	XR_001436065.1	lncRNA
XM_015093216.1	TTC39B	mRNA	XR_001436065.1	lncRNA
XM_015093216.1	TTC39B	mRNA	XR_001436065.1	lncRNA
XM_015093216.1	TTC39B	mRNA	XR_001436065.1	lncRNA
XM_015094671.1	LOC101116975	mRNA	XR_001436065.1	lncRNA
XM_015094671.1	LOC101116975	mRNA	XR_001436065.1	lncRNA
XM_015094671.1	LOC101116975	mRNA	XR_001436065.1	lncRNA
XM_015094671.1	LOC101116975	mRNA	XR_001436065.1	lncRNA
XM_015094671.1	LOC101116975	mRNA	XR_001436065.1	lncRNA
XM_015094671.1	LOC101116975	mRNA	XR_001436065.1	lncRNA
XM_012188097.2	PLCB1	mRNA	XR_001435723.1	lncRNA
XM_012188097.2	PLCB1	mRNA	XR_001435723.1	lncRNA
XM_012188097.2	PLCB1	mRNA	XR_001435723.1	lncRNA
XM_012188097.2	PLCB1	mRNA	XR_001435723.1	lncRNA
XM_012188097.2	PLCB1	mRNA	XR_001435723.1	lncRNA
XM_012188097.2	PLCB1	mRNA	XR_001435723.1	lncRNA
XM_012098820.2	RALGAPA1	mRNA	XR_001435723.1	lncRNA
XM_012098820.2	RALGAPA1	mRNA	XR_001435723.1	lncRNA
XM_012098820.2	RALGAPA1	mRNA	XR_001435723.1	lncRNA
XM_012098820.2	RALGAPA1	mRNA	XR_001435723.1	lncRNA
XM_012098820.2	RALGAPA1	mRNA	XR_001435723.1	lncRNA
XM_012098820.2	RALGAPA1	mRNA	XR_001435723.1	lncRNA
XM_012103723.1	POLI	mRNA	XR_001436065.1	lncRNA
XM_012103723.1	POLI	mRNA	XR_001436065.1	lncRNA
XM_012103723.1	POLI	mRNA	XR_001436065.1	lncRNA
XM_012103723.1	POLI	mRNA	XR_001436065.1	lncRNA
XM_012103723.1	POLI	mRNA	XR_001436065.1	lncRNA
XM_012103723.1	POLI	mRNA	XR_001436065.1	lncRNA
XM_012188097.2	PLCB1	mRNA	XR_001436065.1	lncRNA
XM_012188097.2	PLCB1	mRNA	XR_001436065.1	lncRNA
XM_012188097.2	PLCB1	mRNA	XR_001436065.1	lncRNA
XM_012188097.2	PLCB1	mRNA	XR_001436065.1	lncRNA
XM_012188097.2	PLCB1	mRNA	XR_001436065.1	lncRNA

XM_012188097.2	PLCB1	mRNA	XR_001436065.1	lncRNA
XM_004020402.3	RTTN	mRNA	XR_001435726.1	lncRNA
XM_004020402.3	RTTN	mRNA	XR_001435726.1	lncRNA
XM_004020402.3	RTTN	mRNA	XR_001435726.1	lncRNA
XM_004020402.3	RTTN	mRNA	XR_001435726.1	lncRNA
XM_004020402.3	RTTN	mRNA	XR_001435726.1	lncRNA
XM_004020402.3	RTTN	mRNA	XR_001435726.1	lncRNA
XM_015096383.1	SGMS2	mRNA	XR_001435726.1	lncRNA
XM_015096383.1	SGMS2	mRNA	XR_001435726.1	lncRNA
XM_015096383.1	SGMS2	mRNA	XR_001435726.1	lncRNA
XM_015096383.1	SGMS2	mRNA	XR_001435726.1	lncRNA
XM_015096383.1	SGMS2	mRNA	XR_001435726.1	lncRNA
XM_015096383.1	SGMS2	mRNA	XR_001435726.1	lncRNA
XM_015100095.1	LOC101113523	mRNA	XR_001435726.1	lncRNA
XM_015100095.1	LOC101113523	mRNA	XR_001435726.1	lncRNA
XM_015100095.1	LOC101113523	mRNA	XR_001435726.1	lncRNA
XM_015100095.1	LOC101113523	mRNA	XR_001435726.1	lncRNA
XM_015100095.1	LOC101113523	mRNA	XR_001435726.1	lncRNA
XM_015100095.1	LOC101113523	mRNA	XR_001435726.1	lncRNA
XM_012095602.2	USP2	mRNA	XR_001435726.1	lncRNA
XM_012095602.2	USP2	mRNA	XR_001435726.1	lncRNA
XM_012095602.2	USP2	mRNA	XR_001435726.1	lncRNA
XM_012095602.2	USP2	mRNA	XR_001435726.1	lncRNA
XM_012095602.2	USP2	mRNA	XR_001435726.1	lncRNA
XM_012095602.2	USP2	mRNA	XR_001435726.1	lncRNA
XM_015096518.1	AASDH	mRNA	XR_001024230.1	lncRNA
XM_015096518.1	AASDH	mRNA	XR_001024230.1	lncRNA
XM_015096518.1	AASDH	mRNA	XR_001024230.1	lncRNA
XM_015096518.1	AASDH	mRNA	XR_001024230.1	lncRNA
XM_015096518.1	AASDH	mRNA	XR_001024230.1	lncRNA
XM_015096518.1	AASDH	mRNA	XR_001024230.1	lncRNA
XM_004011835.3	RAD54B	mRNA	XR_001435726.1	lncRNA
XM_004011835.3	RAD54B	mRNA	XR_001435726.1	lncRNA
XM_004011835.3	RAD54B	mRNA	XR_001435726.1	lncRNA
XM_004011835.3	RAD54B	mRNA	XR_001435726.1	lncRNA
XM_004011835.3	RAD54B	mRNA	XR_001435726.1	lncRNA
XM_004011835.3	RAD54B	mRNA	XR_001435726.1	lncRNA
XM_012181434.2	RNF111	mRNA	XR_001024230.1	lncRNA
XM_012181434.2	RNF111	mRNA	XR_001024230.1	lncRNA
XM_012181434.2	RNF111	mRNA	XR_001024230.1	lncRNA
XM_012181434.2	RNF111	mRNA	XR_001024230.1	lncRNA
XM_012181434.2	RNF111	mRNA	XR_001024230.1	lncRNA
XM_012181434.2	RNF111	mRNA	XR_001024230.1	lncRNA
XM_015096383.1	SGMS2	mRNA	XR_001435723.1	lncRNA
XM_015096383.1	SGMS2	mRNA	XR_001435723.1	lncRNA
XM_015096383.1	SGMS2	mRNA	XR_001435723.1	lncRNA
XM_015096383.1	SGMS2	mRNA	XR_001435723.1	lncRNA
XM_015096383.1	SGMS2	mRNA	XR_001435723.1	lncRNA
XM_015096383.1	SGMS2	mRNA	XR_001435723.1	lncRNA
XM_012106889.2	TAF1	mRNA	XR_001435726.1	lncRNA
XM_012106889.2	TAF1	mRNA	XR_001435726.1	lncRNA
XM_012106889.2	TAF1	mRNA	XR_001435726.1	lncRNA
XM_012106889.2	TAF1	mRNA	XR_001435726.1	lncRNA
XM_012106889.2	TAF1	mRNA	XR_001435726.1	lncRNA

XM_012106889.2	TAF1	mRNA	XR_001435726.1	lncRNA
XM_012098820.2	RALGAPA1	mRNA	XR_001024230.1	lncRNA
XM_012098820.2	RALGAPA1	mRNA	XR_001024230.1	lncRNA
XM_012098820.2	RALGAPA1	mRNA	XR_001024230.1	lncRNA
XM_012098820.2	RALGAPA1	mRNA	XR_001024230.1	lncRNA
XM_012098820.2	RALGAPA1	mRNA	XR_001024230.1	lncRNA
XM_012098820.2	RALGAPA1	mRNA	XR_001024230.1	lncRNA
XM_012106889.2	TAF1	mRNA	XR_001435723.1	lncRNA
XM_012106889.2	TAF1	mRNA	XR_001435723.1	lncRNA
XM_012106889.2	TAF1	mRNA	XR_001435723.1	lncRNA
XM_012106889.2	TAF1	mRNA	XR_001435723.1	lncRNA
XM_012106889.2	TAF1	mRNA	XR_001435723.1	lncRNA
XM_012106889.2	TAF1	mRNA	XR_001435723.1	lncRNA
XM_012116543.2	ERCC6L2	mRNA	XR_001436064.1	lncRNA
XM_012116543.2	ERCC6L2	mRNA	XR_001436064.1	lncRNA
XM_012116543.2	ERCC6L2	mRNA	XR_001436064.1	lncRNA
XM_012116543.2	ERCC6L2	mRNA	XR_001436064.1	lncRNA
XM_012116543.2	ERCC6L2	mRNA	XR_001436064.1	lncRNA
XM_012116543.2	ERCC6L2	mRNA	XR_001436064.1	lncRNA
XM_012098820.2	RALGAPA1	mRNA	XR_001435726.1	lncRNA
XM_012098820.2	RALGAPA1	mRNA	XR_001435726.1	lncRNA
XM_012098820.2	RALGAPA1	mRNA	XR_001435726.1	lncRNA
XM_012098820.2	RALGAPA1	mRNA	XR_001435726.1	lncRNA
XM_012098820.2	RALGAPA1	mRNA	XR_001435726.1	lncRNA
XM_012098820.2	RALGAPA1	mRNA	XR_001435726.1	lncRNA
XM_015096931.1	THBS1	mRNA	XR_001436065.1	lncRNA
XM_015096931.1	THBS1	mRNA	XR_001436065.1	lncRNA
XM_015096931.1	THBS1	mRNA	XR_001436065.1	lncRNA
XM_015096931.1	THBS1	mRNA	XR_001436065.1	lncRNA
XM_015096931.1	THBS1	mRNA	XR_001436065.1	lncRNA
XM_015096931.1	THBS1	mRNA	XR_001436065.1	lncRNA
XM_012095602.2	USP2	mRNA	XR_001024230.1	lncRNA
XM_012095602.2	USP2	mRNA	XR_001024230.1	lncRNA
XM_012095602.2	USP2	mRNA	XR_001024230.1	lncRNA
XM_012095602.2	USP2	mRNA	XR_001024230.1	lncRNA
XM_012095602.2	USP2	mRNA	XR_001024230.1	lncRNA
XM_012095602.2	USP2	mRNA	XR_001024230.1	lncRNA
XM_015094671.1	LOC101116975	mRNA	XR_001435726.1	lncRNA
XM_015094671.1	LOC101116975	mRNA	XR_001435726.1	lncRNA
XM_015094671.1	LOC101116975	mRNA	XR_001435726.1	lncRNA
XM_015094671.1	LOC101116975	mRNA	XR_001435726.1	lncRNA
XM_015094671.1	LOC101116975	mRNA	XR_001435726.1	lncRNA
XM_015094671.1	LOC101116975	mRNA	XR_001435726.1	lncRNA
XM_015093216.1	TTC39B	mRNA	XR_001435723.1	lncRNA
XM_015093216.1	TTC39B	mRNA	XR_001435723.1	lncRNA
XM_015093216.1	TTC39B	mRNA	XR_001435723.1	lncRNA
XM_015093216.1	TTC39B	mRNA	XR_001435723.1	lncRNA
XM_015093216.1	TTC39B	mRNA	XR_001435723.1	lncRNA
XM_015093216.1	TTC39B	mRNA	XR_001435723.1	lncRNA
XM_012181434.2	RNF111	mRNA	XR_001435723.1	lncRNA
XM_012181434.2	RNF111	mRNA	XR_001435723.1	lncRNA
XM_012181434.2	RNF111	mRNA	XR_001435723.1	lncRNA
XM_012181434.2	RNF111	mRNA	XR_001435723.1	lncRNA
XM_012181434.2	RNF111	mRNA	XR_001435723.1	lncRNA

XM_012181434.2	RNF111	mRNA	XR_001435723.1	lncRNA
XM_004004584.3	RAPGEF4	mRNA	XR_001436065.1	lncRNA
XM_004004584.3	RAPGEF4	mRNA	XR_001436065.1	lncRNA
XM_004004584.3	RAPGEF4	mRNA	XR_001436065.1	lncRNA
XM_004004584.3	RAPGEF4	mRNA	XR_001436065.1	lncRNA
XM_004004584.3	RAPGEF4	mRNA	XR_001436065.1	lncRNA
XM_004004584.3	RAPGEF4	mRNA	XR_001436065.1	lncRNA
XM_012098820.2	RALGAPA1	mRNA	XR_001436065.1	lncRNA
XM_012098820.2	RALGAPA1	mRNA	XR_001436065.1	lncRNA
XM_012098820.2	RALGAPA1	mRNA	XR_001436065.1	lncRNA
XM_012098820.2	RALGAPA1	mRNA	XR_001436065.1	lncRNA
XM_012098820.2	RALGAPA1	mRNA	XR_001436065.1	lncRNA
XM_012098820.2	RALGAPA1	mRNA	XR_001436065.1	lncRNA
XM_012106889.2	TAF1	mRNA	XR_001436065.1	lncRNA
XM_012106889.2	TAF1	mRNA	XR_001436065.1	lncRNA
XM_012106889.2	TAF1	mRNA	XR_001436065.1	lncRNA
XM_012106889.2	TAF1	mRNA	XR_001436065.1	lncRNA
XM_012106889.2	TAF1	mRNA	XR_001436065.1	lncRNA
XM_012106889.2	TAF1	mRNA	XR_001436065.1	lncRNA
XM_015093216.1	TTC39B	mRNA	XR_001436064.1	lncRNA
XM_015093216.1	TTC39B	mRNA	XR_001436064.1	lncRNA
XM_015093216.1	TTC39B	mRNA	XR_001436064.1	lncRNA
XM_015093216.1	TTC39B	mRNA	XR_001436064.1	lncRNA
XM_015093216.1	TTC39B	mRNA	XR_001436064.1	lncRNA
XM_015093216.1	TTC39B	mRNA	XR_001436064.1	lncRNA
XM_012116543.2	ERCC6L2	mRNA	XR_001436065.1	lncRNA
XM_012116543.2	ERCC6L2	mRNA	XR_001436065.1	lncRNA
XM_012116543.2	ERCC6L2	mRNA	XR_001436065.1	lncRNA
XM_012116543.2	ERCC6L2	mRNA	XR_001436065.1	lncRNA
XM_012116543.2	ERCC6L2	mRNA	XR_001436065.1	lncRNA
XM_012116543.2	ERCC6L2	mRNA	XR_001436065.1	lncRNA
XM_012181434.2	RNF111	mRNA	XR_001436064.1	lncRNA
XM_012181434.2	RNF111	mRNA	XR_001436064.1	lncRNA
XM_012181434.2	RNF111	mRNA	XR_001436064.1	lncRNA
XM_012181434.2	RNF111	mRNA	XR_001436064.1	lncRNA
XM_012181434.2	RNF111	mRNA	XR_001436064.1	lncRNA
XM_012181434.2	RNF111	mRNA	XR_001436064.1	lncRNA
XM_012176761.2	MDFIC	mRNA	XR_001435723.1	lncRNA
XM_012176761.2	MDFIC	mRNA	XR_001435723.1	lncRNA
XM_012176761.2	MDFIC	mRNA	XR_001435723.1	lncRNA
XM_012176761.2	MDFIC	mRNA	XR_001435723.1	lncRNA
XM_012176761.2	MDFIC	mRNA	XR_001435723.1	lncRNA
XM_012176761.2	MDFIC	mRNA	XR_001435723.1	lncRNA
XM_015096931.1	THBS1	mRNA	XR_001435723.1	lncRNA
XM_015096931.1	THBS1	mRNA	XR_001435723.1	lncRNA
XM_015096931.1	THBS1	mRNA	XR_001435723.1	lncRNA
XM_015096931.1	THBS1	mRNA	XR_001435723.1	lncRNA
XM_015096931.1	THBS1	mRNA	XR_001435723.1	lncRNA
XM_015096931.1	THBS1	mRNA	XR_001435723.1	lncRNA
XM_012181434.2	RNF111	mRNA	XR_001436065.1	lncRNA
XM_012181434.2	RNF111	mRNA	XR_001436065.1	lncRNA
XM_012181434.2	RNF111	mRNA	XR_001436065.1	lncRNA
XM_012181434.2	RNF111	mRNA	XR_001436065.1	lncRNA
XM_012181434.2	RNF111	mRNA	XR_001436065.1	lncRNA

XM_012181434.2	RNF111	mRNA	XR_001436065.1	lncRNA
XM_012095602.2	USP2	mRNA	XR_001435723.1	lncRNA
XM_012095602.2	USP2	mRNA	XR_001435723.1	lncRNA
XM_012095602.2	USP2	mRNA	XR_001435723.1	lncRNA
XM_012095602.2	USP2	mRNA	XR_001435723.1	lncRNA
XM_012095602.2	USP2	mRNA	XR_001435723.1	lncRNA
XM_012095602.2	USP2	mRNA	XR_001435723.1	lncRNA
XM_015094671.1	LOC101116975	mRNA	XR_001435723.1	lncRNA
XM_015094671.1	LOC101116975	mRNA	XR_001435723.1	lncRNA
XM_015094671.1	LOC101116975	mRNA	XR_001435723.1	lncRNA
XM_015094671.1	LOC101116975	mRNA	XR_001435723.1	lncRNA
XM_015094671.1	LOC101116975	mRNA	XR_001435723.1	lncRNA
XM_015094671.1	LOC101116975	mRNA	XR_001435723.1	lncRNA
XM_004020402.3	RTTN	mRNA	XR_001435723.1	lncRNA
XM_004020402.3	RTTN	mRNA	XR_001435723.1	lncRNA
XM_004020402.3	RTTN	mRNA	XR_001435723.1	lncRNA
XM_004020402.3	RTTN	mRNA	XR_001435723.1	lncRNA
XM_004020402.3	RTTN	mRNA	XR_001435723.1	lncRNA
XM_004020402.3	RTTN	mRNA	XR_001435723.1	lncRNA
XM_012099944.1	PBRM1	mRNA	XR_001436064.1	lncRNA
XM_012099944.1	PBRM1	mRNA	XR_001436064.1	lncRNA
XM_012099944.1	PBRM1	mRNA	XR_001436064.1	lncRNA
XM_012099944.1	PBRM1	mRNA	XR_001436064.1	lncRNA
XM_012099944.1	PBRM1	mRNA	XR_001436064.1	lncRNA
XM_012099944.1	PBRM1	mRNA	XR_001436064.1	lncRNA
XM_012116543.2	ERCC6L2	mRNA	XR_001435723.1	lncRNA
XM_012116543.2	ERCC6L2	mRNA	XR_001435723.1	lncRNA
XM_012116543.2	ERCC6L2	mRNA	XR_001435723.1	lncRNA
XM_012116543.2	ERCC6L2	mRNA	XR_001435723.1	lncRNA
XM_012116543.2	ERCC6L2	mRNA	XR_001435723.1	lncRNA
XM_012116543.2	ERCC6L2	mRNA	XR_001435723.1	lncRNA
XM_012116543.2	ERCC6L2	mRNA	XR_001024230.1	lncRNA
XM_012116543.2	ERCC6L2	mRNA	XR_001024230.1	lncRNA
XM_012116543.2	ERCC6L2	mRNA	XR_001024230.1	lncRNA
XM_012116543.2	ERCC6L2	mRNA	XR_001024230.1	lncRNA
XM_012116543.2	ERCC6L2	mRNA	XR_001024230.1	lncRNA
XM_012116543.2	ERCC6L2	mRNA	XR_001024230.1	lncRNA
XM_012188097.2	PLCB1	mRNA	XR_001436064.1	lncRNA
XM_012188097.2	PLCB1	mRNA	XR_001436064.1	lncRNA
XM_012188097.2	PLCB1	mRNA	XR_001436064.1	lncRNA
XM_012188097.2	PLCB1	mRNA	XR_001436064.1	lncRNA
XM_012188097.2	PLCB1	mRNA	XR_001436064.1	lncRNA
XM_012188097.2	PLCB1	mRNA	XR_001436064.1	lncRNA
XM_015100095.1	LOC101113523	mRNA	XR_001435723.1	lncRNA
XM_015100095.1	LOC101113523	mRNA	XR_001435723.1	lncRNA
XM_015100095.1	LOC101113523	mRNA	XR_001435723.1	lncRNA
XM_015100095.1	LOC101113523	mRNA	XR_001435723.1	lncRNA
XM_015100095.1	LOC101113523	mRNA	XR_001435723.1	lncRNA
XM_015100095.1	LOC101113523	mRNA	XR_001435723.1	lncRNA
XM_015101341.1	OTULIN	mRNA	XR_001433837.1	lncRNA
XM_015101341.1	OTULIN	mRNA	XR_001433837.1	lncRNA
XM_015101341.1	OTULIN	mRNA	XR_001433837.1	lncRNA
XM_015101341.1	OTULIN	mRNA	XR_001433837.1	lncRNA
XM_015101341.1	OTULIN	mRNA	XR_001433837.1	lncRNA

XM_015101341.1	OTULIN	mRNA	XR_001433837.1	lncRNA
XM_012095733.1	BTBD10	mRNA	XR_001433837.1	lncRNA
XM_012095733.1	BTBD10	mRNA	XR_001433837.1	lncRNA
XM_012095733.1	BTBD10	mRNA	XR_001433837.1	lncRNA
XM_012095733.1	BTBD10	mRNA	XR_001433837.1	lncRNA
XM_012095733.1	BTBD10	mRNA	XR_001433837.1	lncRNA
XM_012095733.1	BTBD10	mRNA	XR_001433837.1	lncRNA
XM_015099739.1	LOC101122805	mRNA	XR_001433837.1	lncRNA
XM_015099739.1	LOC101122805	mRNA	XR_001433837.1	lncRNA
XM_015099739.1	LOC101122805	mRNA	XR_001433837.1	lncRNA
XM_015099739.1	LOC101122805	mRNA	XR_001433837.1	lncRNA
XM_015099739.1	LOC101122805	mRNA	XR_001433837.1	lncRNA
XM_015099739.1	LOC101122805	mRNA	XR_001433837.1	lncRNA
XM_004016880.3	FAM169A	mRNA	XR_001436390.1	lncRNA
XM_004016880.3	FAM169A	mRNA	XR_001436390.1	lncRNA
XM_004016880.3	FAM169A	mRNA	XR_001436390.1	lncRNA
XM_004016880.3	FAM169A	mRNA	XR_001436390.1	lncRNA
XM_004016880.3	FAM169A	mRNA	XR_001436390.1	lncRNA
XM_004016880.3	FAM169A	mRNA	XR_001436390.1	lncRNA
XM_012175120.1	PPFIBP1	mRNA	XR_001436390.1	lncRNA
XM_012175120.1	PPFIBP1	mRNA	XR_001436390.1	lncRNA
XM_012175120.1	PPFIBP1	mRNA	XR_001436390.1	lncRNA
XM_012175120.1	PPFIBP1	mRNA	XR_001436390.1	lncRNA
XM_012175120.1	PPFIBP1	mRNA	XR_001436390.1	lncRNA
XM_012175120.1	PPFIBP1	mRNA	XR_001436390.1	lncRNA
XM_012173383.2	DHX57	mRNA	XR_001436390.1	lncRNA
XM_012173383.2	DHX57	mRNA	XR_001436390.1	lncRNA
XM_012173383.2	DHX57	mRNA	XR_001436390.1	lncRNA
XM_012173383.2	DHX57	mRNA	XR_001436390.1	lncRNA
XM_012173383.2	DHX57	mRNA	XR_001436390.1	lncRNA
XM_012173383.2	DHX57	mRNA	XR_001436390.1	lncRNA
XM_012185875.1	LUC7L3	mRNA	XR_001436390.1	lncRNA
XM_012185875.1	LUC7L3	mRNA	XR_001436390.1	lncRNA
XM_012185875.1	LUC7L3	mRNA	XR_001436390.1	lncRNA
XM_012185875.1	LUC7L3	mRNA	XR_001436390.1	lncRNA
XM_012185875.1	LUC7L3	mRNA	XR_001436390.1	lncRNA
XM_012185875.1	LUC7L3	mRNA	XR_001436390.1	lncRNA
XM_012146540.1	ATG9A	mRNA	XR_001436390.1	lncRNA
XM_012146540.1	ATG9A	mRNA	XR_001436390.1	lncRNA
XM_012146540.1	ATG9A	mRNA	XR_001436390.1	lncRNA
XM_012146540.1	ATG9A	mRNA	XR_001436390.1	lncRNA
XM_012146540.1	ATG9A	mRNA	XR_001436390.1	lncRNA
XM_012146540.1	ATG9A	mRNA	XR_001436390.1	lncRNA
XM_015099976.1	ZFP30	mRNA	XR_001436390.1	lncRNA
XM_015099976.1	ZFP30	mRNA	XR_001436390.1	lncRNA
XM_015099976.1	ZFP30	mRNA	XR_001436390.1	lncRNA
XM_015099976.1	ZFP30	mRNA	XR_001436390.1	lncRNA
XM_015099976.1	ZFP30	mRNA	XR_001436390.1	lncRNA
XM_015099976.1	ZFP30	mRNA	XR_001436390.1	lncRNA
XM_015091887.1	MYCL	mRNA	XR_001436390.1	lncRNA
XM_015091887.1	MYCL	mRNA	XR_001436390.1	lncRNA
XM_015091887.1	MYCL	mRNA	XR_001436390.1	lncRNA
XM_015091887.1	MYCL	mRNA	XR_001436390.1	lncRNA
XM_015091887.1	MYCL	mRNA	XR_001436390.1	lncRNA

XM_015091887.1	MYCL	mRNA	XR_001436390.1	lncRNA
XM_015104323.1	SPRTN	mRNA	XR_001040147.1	lncRNA
XM_015104323.1	SPRTN	mRNA	XR_001040147.1	lncRNA
XM_015104323.1	SPRTN	mRNA	XR_001040147.1	lncRNA
XM_015104323.1	SPRTN	mRNA	XR_001040147.1	lncRNA
XM_015104323.1	SPRTN	mRNA	XR_001040147.1	lncRNA
XM_015104323.1	SPRTN	mRNA	XR_001040147.1	lncRNA
XM_015104417.1	CHST3	mRNA	XR_001435722.1	lncRNA
XM_015104417.1	CHST3	mRNA	XR_001435722.1	lncRNA
XM_015104417.1	CHST3	mRNA	XR_001435722.1	lncRNA
XM_015104417.1	CHST3	mRNA	XR_001435722.1	lncRNA
XM_015104417.1	CHST3	mRNA	XR_001435722.1	lncRNA
XM_015104417.1	CHST3	mRNA	XR_001435722.1	lncRNA
XM_012187727.2	TRMT1L	mRNA	XR_001433856.1	lncRNA
XM_012187727.2	TRMT1L	mRNA	XR_001433856.1	lncRNA
XM_012187727.2	TRMT1L	mRNA	XR_001433856.1	lncRNA
XM_012187727.2	TRMT1L	mRNA	XR_001433856.1	lncRNA
XM_012187727.2	TRMT1L	mRNA	XR_001433856.1	lncRNA
XM_012187727.2	TRMT1L	mRNA	XR_001433856.1	lncRNA
XM_004012073.3	FNDC3A	mRNA	XR_001434920.1	lncRNA
XM_004012073.3	FNDC3A	mRNA	XR_001434920.1	lncRNA
XM_004012073.3	FNDC3A	mRNA	XR_001434920.1	lncRNA
XM_004012073.3	FNDC3A	mRNA	XR_001434920.1	lncRNA
XM_004012073.3	FNDC3A	mRNA	XR_001434920.1	lncRNA
XM_004012073.3	FNDC3A	mRNA	XR_001434920.1	lncRNA
XM_012176761.2	MDFIC	mRNA	XR_001433856.1	lncRNA
XM_012176761.2	MDFIC	mRNA	XR_001433856.1	lncRNA
XM_012176761.2	MDFIC	mRNA	XR_001433856.1	lncRNA
XM_012176761.2	MDFIC	mRNA	XR_001433856.1	lncRNA
XM_012176761.2	MDFIC	mRNA	XR_001433856.1	lncRNA
XM_012176761.2	MDFIC	mRNA	XR_001433856.1	lncRNA
XM_015100095.1	LOC101113523	mRNA	XR_001436817.1	lncRNA
XM_015100095.1	LOC101113523	mRNA	XR_001436817.1	lncRNA
XM_015100095.1	LOC101113523	mRNA	XR_001436817.1	lncRNA
XM_015100095.1	LOC101113523	mRNA	XR_001436817.1	lncRNA
XM_015100095.1	LOC101113523	mRNA	XR_001436817.1	lncRNA
XM_015100095.1	LOC101113523	mRNA	XR_001436817.1	lncRNA
XM_012187727.2	TRMT1L	mRNA	XR_001435107.1	lncRNA
XM_012187727.2	TRMT1L	mRNA	XR_001435107.1	lncRNA
XM_012187727.2	TRMT1L	mRNA	XR_001435107.1	lncRNA
XM_012187727.2	TRMT1L	mRNA	XR_001435107.1	lncRNA
XM_012187727.2	TRMT1L	mRNA	XR_001435107.1	lncRNA
XM_012187727.2	TRMT1L	mRNA	XR_001435107.1	lncRNA
XM_004020402.3	RTTN	mRNA	XR_001433856.1	lncRNA
XM_004020402.3	RTTN	mRNA	XR_001433856.1	lncRNA
XM_004020402.3	RTTN	mRNA	XR_001433856.1	lncRNA
XM_004020402.3	RTTN	mRNA	XR_001433856.1	lncRNA
XM_004020402.3	RTTN	mRNA	XR_001433856.1	lncRNA
XM_004020402.3	RTTN	mRNA	XR_001433856.1	lncRNA
XM_015096518.1	AASDH	mRNA	XR_001436817.1	lncRNA
XM_015096518.1	AASDH	mRNA	XR_001436817.1	lncRNA
XM_015096518.1	AASDH	mRNA	XR_001436817.1	lncRNA
XM_015096518.1	AASDH	mRNA	XR_001436817.1	lncRNA
XM_015096518.1	AASDH	mRNA	XR_001436817.1	lncRNA

XM_015096518.1	AASDH	mRNA	XR_001436817.1	lncRNA
XM_012094885.1	LOC101116597	mRNA	XR_001435722.1	lncRNA
XM_012094885.1	LOC101116597	mRNA	XR_001435722.1	lncRNA
XM_012094885.1	LOC101116597	mRNA	XR_001435722.1	lncRNA
XM_012094885.1	LOC101116597	mRNA	XR_001435722.1	lncRNA
XM_012094885.1	LOC101116597	mRNA	XR_001435722.1	lncRNA
XM_012094885.1	LOC101116597	mRNA	XR_001435722.1	lncRNA
XM_012159182.2	LOC101121820	mRNA	XR_001435722.1	lncRNA
XM_012159182.2	LOC101121820	mRNA	XR_001435722.1	lncRNA
XM_012159182.2	LOC101121820	mRNA	XR_001435722.1	lncRNA
XM_012159182.2	LOC101121820	mRNA	XR_001435722.1	lncRNA
XM_012159182.2	LOC101121820	mRNA	XR_001435722.1	lncRNA
XM_012159182.2	LOC101121820	mRNA	XR_001435722.1	lncRNA
XM_015100095.1	LOC101113523	mRNA	XR_001435722.1	lncRNA
XM_015100095.1	LOC101113523	mRNA	XR_001435722.1	lncRNA
XM_015100095.1	LOC101113523	mRNA	XR_001435722.1	lncRNA
XM_015100095.1	LOC101113523	mRNA	XR_001435722.1	lncRNA
XM_015100095.1	LOC101113523	mRNA	XR_001435722.1	lncRNA
XM_015100095.1	LOC101113523	mRNA	XR_001435722.1	lncRNA
XM_004011835.3	RAD54B	mRNA	XR_001436817.1	lncRNA
XM_004011835.3	RAD54B	mRNA	XR_001436817.1	lncRNA
XM_004011835.3	RAD54B	mRNA	XR_001436817.1	lncRNA
XM_004011835.3	RAD54B	mRNA	XR_001436817.1	lncRNA
XM_004011835.3	RAD54B	mRNA	XR_001436817.1	lncRNA
XM_004011835.3	RAD54B	mRNA	XR_001436817.1	lncRNA
XM_012096602.2	DLG1	mRNA	XR_001435107.1	lncRNA
XM_012096602.2	DLG1	mRNA	XR_001435107.1	lncRNA
XM_012096602.2	DLG1	mRNA	XR_001435107.1	lncRNA
XM_012096602.2	DLG1	mRNA	XR_001435107.1	lncRNA
XM_012096602.2	DLG1	mRNA	XR_001435107.1	lncRNA
XM_012096602.2	DLG1	mRNA	XR_001435107.1	lncRNA
XM_012177522.2	VDAC1	mRNA	XR_001435722.1	lncRNA
XM_012177522.2	VDAC1	mRNA	XR_001435722.1	lncRNA
XM_012177522.2	VDAC1	mRNA	XR_001435722.1	lncRNA
XM_012177522.2	VDAC1	mRNA	XR_001435722.1	lncRNA
XM_012177522.2	VDAC1	mRNA	XR_001435722.1	lncRNA
XM_012177522.2	VDAC1	mRNA	XR_001435722.1	lncRNA
XM_015095516.1	LOC101123551	mRNA	XR_001435722.1	lncRNA
XM_015095516.1	LOC101123551	mRNA	XR_001435722.1	lncRNA
XM_015095516.1	LOC101123551	mRNA	XR_001435722.1	lncRNA
XM_015095516.1	LOC101123551	mRNA	XR_001435722.1	lncRNA
XM_015095516.1	LOC101123551	mRNA	XR_001435722.1	lncRNA
XM_015095516.1	LOC101123551	mRNA	XR_001435722.1	lncRNA
XM_015104417.1	CHST3	mRNA	XR_001435107.1	lncRNA
XM_015104417.1	CHST3	mRNA	XR_001435107.1	lncRNA
XM_015104417.1	CHST3	mRNA	XR_001435107.1	lncRNA
XM_015104417.1	CHST3	mRNA	XR_001435107.1	lncRNA
XM_015104417.1	CHST3	mRNA	XR_001435107.1	lncRNA
XM_015104417.1	CHST3	mRNA	XR_001435107.1	lncRNA
XM_015104417.1	CHST3	mRNA	XR_001436817.1	lncRNA
XM_015104417.1	CHST3	mRNA	XR_001436817.1	lncRNA
XM_015104417.1	CHST3	mRNA	XR_001436817.1	lncRNA
XM_015104417.1	CHST3	mRNA	XR_001436817.1	lncRNA
XM_015104417.1	CHST3	mRNA	XR_001436817.1	lncRNA

XM_015104417.1	CHST3	mRNA	XR_001436817.1	lncRNA
XM_012176414.2	PHF14	mRNA	XR_001435107.1	lncRNA
XM_012176414.2	PHF14	mRNA	XR_001435107.1	lncRNA
XM_012176414.2	PHF14	mRNA	XR_001435107.1	lncRNA
XM_012176414.2	PHF14	mRNA	XR_001435107.1	lncRNA
XM_012176414.2	PHF14	mRNA	XR_001435107.1	lncRNA
XM_012176414.2	PHF14	mRNA	XR_001435107.1	lncRNA
XM_015096518.1	AASDH	mRNA	XR_001435722.1	lncRNA
XM_015096518.1	AASDH	mRNA	XR_001435722.1	lncRNA
XM_015096518.1	AASDH	mRNA	XR_001435722.1	lncRNA
XM_015096518.1	AASDH	mRNA	XR_001435722.1	lncRNA
XM_015096518.1	AASDH	mRNA	XR_001435722.1	lncRNA
XM_015096518.1	AASDH	mRNA	XR_001435722.1	lncRNA
XM_012187727.2	TRMT1L	mRNA	XR_001436817.1	lncRNA
XM_012187727.2	TRMT1L	mRNA	XR_001436817.1	lncRNA
XM_012187727.2	TRMT1L	mRNA	XR_001436817.1	lncRNA
XM_012187727.2	TRMT1L	mRNA	XR_001436817.1	lncRNA
XM_012187727.2	TRMT1L	mRNA	XR_001436817.1	lncRNA
XM_012187727.2	TRMT1L	mRNA	XR_001436817.1	lncRNA
XM_015098592.1	RABEP1	mRNA	XR_001435107.1	lncRNA
XM_015098592.1	RABEP1	mRNA	XR_001435107.1	lncRNA
XM_015098592.1	RABEP1	mRNA	XR_001435107.1	lncRNA
XM_015098592.1	RABEP1	mRNA	XR_001435107.1	lncRNA
XM_015098592.1	RABEP1	mRNA	XR_001435107.1	lncRNA
XM_015098592.1	RABEP1	mRNA	XR_001435107.1	lncRNA
XM_015096383.1	SGMS2	mRNA	XR_001436817.1	lncRNA
XM_015096383.1	SGMS2	mRNA	XR_001436817.1	lncRNA
XM_015096383.1	SGMS2	mRNA	XR_001436817.1	lncRNA
XM_015096383.1	SGMS2	mRNA	XR_001436817.1	lncRNA
XM_015096383.1	SGMS2	mRNA	XR_001436817.1	lncRNA
XM_015096383.1	SGMS2	mRNA	XR_001436817.1	lncRNA
XM_004012073.3	FNDC3A	mRNA	XR_001436817.1	lncRNA
XM_004012073.3	FNDC3A	mRNA	XR_001436817.1	lncRNA
XM_004012073.3	FNDC3A	mRNA	XR_001436817.1	lncRNA
XM_004012073.3	FNDC3A	mRNA	XR_001436817.1	lncRNA
XM_004012073.3	FNDC3A	mRNA	XR_001436817.1	lncRNA
XM_004012073.3	FNDC3A	mRNA	XR_001436817.1	lncRNA
XM_012187827.1	GIGYF2	mRNA	XR_001436817.1	lncRNA
XM_012187827.1	GIGYF2	mRNA	XR_001436817.1	lncRNA
XM_012187827.1	GIGYF2	mRNA	XR_001436817.1	lncRNA
XM_012187827.1	GIGYF2	mRNA	XR_001436817.1	lncRNA
XM_012187827.1	GIGYF2	mRNA	XR_001436817.1	lncRNA
XM_012187827.1	GIGYF2	mRNA	XR_001436817.1	lncRNA
XM_012174509.2	ADGRL2	mRNA	XR_001436817.1	lncRNA
XM_012174509.2	ADGRL2	mRNA	XR_001436817.1	lncRNA
XM_012174509.2	ADGRL2	mRNA	XR_001436817.1	lncRNA
XM_012174509.2	ADGRL2	mRNA	XR_001436817.1	lncRNA
XM_012174509.2	ADGRL2	mRNA	XR_001436817.1	lncRNA
XM_012174509.2	ADGRL2	mRNA	XR_001436817.1	lncRNA
XM_012129647.1	NEK1	mRNA	XR_001436817.1	lncRNA
XM_012129647.1	NEK1	mRNA	XR_001436817.1	lncRNA
XM_012129647.1	NEK1	mRNA	XR_001436817.1	lncRNA
XM_012129647.1	NEK1	mRNA	XR_001436817.1	lncRNA
XM_012129647.1	NEK1	mRNA	XR_001436817.1	lncRNA

XM_012129647.1	NEK1	mRNA	XR_001436817.1	lncRNA
XM_012187727.2	TRMT1L	mRNA	XR_001434920.1	lncRNA
XM_012187727.2	TRMT1L	mRNA	XR_001434920.1	lncRNA
XM_012187727.2	TRMT1L	mRNA	XR_001434920.1	lncRNA
XM_012187727.2	TRMT1L	mRNA	XR_001434920.1	lncRNA
XM_012187727.2	TRMT1L	mRNA	XR_001434920.1	lncRNA
XM_012187727.2	TRMT1L	mRNA	XR_001434920.1	lncRNA
XM_012187630.2	SOAT1	mRNA	XR_001434920.1	lncRNA
XM_012187630.2	SOAT1	mRNA	XR_001434920.1	lncRNA
XM_012187630.2	SOAT1	mRNA	XR_001434920.1	lncRNA
XM_012187630.2	SOAT1	mRNA	XR_001434920.1	lncRNA
XM_012187630.2	SOAT1	mRNA	XR_001434920.1	lncRNA
XM_012187630.2	SOAT1	mRNA	XR_001434920.1	lncRNA
XM_015104417.1	CHST3	mRNA	XR_001434920.1	lncRNA
XM_015104417.1	CHST3	mRNA	XR_001434920.1	lncRNA
XM_015104417.1	CHST3	mRNA	XR_001434920.1	lncRNA
XM_015104417.1	CHST3	mRNA	XR_001434920.1	lncRNA
XM_015104417.1	CHST3	mRNA	XR_001434920.1	lncRNA
XM_015104417.1	CHST3	mRNA	XR_001434920.1	lncRNA
XM_004020402.3	RTTN	mRNA	XR_001435722.1	lncRNA
XM_004020402.3	RTTN	mRNA	XR_001435722.1	lncRNA
XM_004020402.3	RTTN	mRNA	XR_001435722.1	lncRNA
XM_004020402.3	RTTN	mRNA	XR_001435722.1	lncRNA
XM_004020402.3	RTTN	mRNA	XR_001435722.1	lncRNA
XM_004020402.3	RTTN	mRNA	XR_001435722.1	lncRNA
XM_012187827.1	GIGYF2	mRNA	XR_001435107.1	lncRNA
XM_012187827.1	GIGYF2	mRNA	XR_001435107.1	lncRNA
XM_012187827.1	GIGYF2	mRNA	XR_001435107.1	lncRNA
XM_012187827.1	GIGYF2	mRNA	XR_001435107.1	lncRNA
XM_012187827.1	GIGYF2	mRNA	XR_001435107.1	lncRNA
XM_012187827.1	GIGYF2	mRNA	XR_001435107.1	lncRNA
XM_004020074.3	PIK3AP1	mRNA	XR_001435107.1	lncRNA
XM_004020074.3	PIK3AP1	mRNA	XR_001435107.1	lncRNA
XM_004020074.3	PIK3AP1	mRNA	XR_001435107.1	lncRNA
XM_004020074.3	PIK3AP1	mRNA	XR_001435107.1	lncRNA
XM_004020074.3	PIK3AP1	mRNA	XR_001435107.1	lncRNA
XM_004020074.3	PIK3AP1	mRNA	XR_001435107.1	lncRNA
XM_012176414.2	PHF14	mRNA	XR_001433856.1	lncRNA
XM_012176414.2	PHF14	mRNA	XR_001433856.1	lncRNA
XM_012176414.2	PHF14	mRNA	XR_001433856.1	lncRNA
XM_012176414.2	PHF14	mRNA	XR_001433856.1	lncRNA
XM_012176414.2	PHF14	mRNA	XR_001433856.1	lncRNA
XM_012176414.2	PHF14	mRNA	XR_001433856.1	lncRNA
XM_012177522.2	VDAC1	mRNA	XR_001433856.1	lncRNA
XM_012177522.2	VDAC1	mRNA	XR_001433856.1	lncRNA
XM_012177522.2	VDAC1	mRNA	XR_001433856.1	lncRNA
XM_012177522.2	VDAC1	mRNA	XR_001433856.1	lncRNA
XM_012177522.2	VDAC1	mRNA	XR_001433856.1	lncRNA
XM_012177522.2	VDAC1	mRNA	XR_001433856.1	lncRNA
XM_004020402.3	RTTN	mRNA	XR_001435107.1	lncRNA
XM_004020402.3	RTTN	mRNA	XR_001435107.1	lncRNA
XM_004020402.3	RTTN	mRNA	XR_001435107.1	lncRNA
XM_004020402.3	RTTN	mRNA	XR_001435107.1	lncRNA
XM_004020402.3	RTTN	mRNA	XR_001435107.1	lncRNA

XM_004020402.3	RTTN	mRNA	XR_001435107.1	lncRNA
XM_015096518.1	AASDH	mRNA	XR_001433856.1	lncRNA
XM_015096518.1	AASDH	mRNA	XR_001433856.1	lncRNA
XM_015096518.1	AASDH	mRNA	XR_001433856.1	lncRNA
XM_015096518.1	AASDH	mRNA	XR_001433856.1	lncRNA
XM_015096518.1	AASDH	mRNA	XR_001433856.1	lncRNA
XM_015096518.1	AASDH	mRNA	XR_001433856.1	lncRNA
XM_015100095.1	LOC101113523	mRNA	XR_001433856.1	lncRNA
XM_015100095.1	LOC101113523	mRNA	XR_001433856.1	lncRNA
XM_015100095.1	LOC101113523	mRNA	XR_001433856.1	lncRNA
XM_015100095.1	LOC101113523	mRNA	XR_001433856.1	lncRNA
XM_015100095.1	LOC101113523	mRNA	XR_001433856.1	lncRNA
XM_015100095.1	LOC101113523	mRNA	XR_001433856.1	lncRNA
XM_012159182.2	LOC101121820	mRNA	XR_001433856.1	lncRNA
XM_012159182.2	LOC101121820	mRNA	XR_001433856.1	lncRNA
XM_012159182.2	LOC101121820	mRNA	XR_001433856.1	lncRNA
XM_012159182.2	LOC101121820	mRNA	XR_001433856.1	lncRNA
XM_012159182.2	LOC101121820	mRNA	XR_001433856.1	lncRNA
XM_012159182.2	LOC101121820	mRNA	XR_001433856.1	lncRNA
XM_012176761.2	MDFIC	mRNA	XR_001435722.1	lncRNA
XM_012176761.2	MDFIC	mRNA	XR_001435722.1	lncRNA
XM_012176761.2	MDFIC	mRNA	XR_001435722.1	lncRNA
XM_012176761.2	MDFIC	mRNA	XR_001435722.1	lncRNA
XM_012176761.2	MDFIC	mRNA	XR_001435722.1	lncRNA
XM_012176761.2	MDFIC	mRNA	XR_001435722.1	lncRNA
XM_012094885.1	LOC101116597	mRNA	XR_001434920.1	lncRNA
XM_012094885.1	LOC101116597	mRNA	XR_001434920.1	lncRNA
XM_012094885.1	LOC101116597	mRNA	XR_001434920.1	lncRNA
XM_012094885.1	LOC101116597	mRNA	XR_001434920.1	lncRNA
XM_012094885.1	LOC101116597	mRNA	XR_001434920.1	lncRNA
XM_012094885.1	LOC101116597	mRNA	XR_001434920.1	lncRNA
XM_012187727.2	TRMT1L	mRNA	XR_001435722.1	lncRNA
XM_012187727.2	TRMT1L	mRNA	XR_001435722.1	lncRNA
XM_012187727.2	TRMT1L	mRNA	XR_001435722.1	lncRNA
XM_012187727.2	TRMT1L	mRNA	XR_001435722.1	lncRNA
XM_012187727.2	TRMT1L	mRNA	XR_001435722.1	lncRNA
XM_012187727.2	TRMT1L	mRNA	XR_001435722.1	lncRNA
XM_015095516.1	LOC101123551	mRNA	XR_001434920.1	lncRNA
XM_015095516.1	LOC101123551	mRNA	XR_001434920.1	lncRNA
XM_015095516.1	LOC101123551	mRNA	XR_001434920.1	lncRNA
XM_015095516.1	LOC101123551	mRNA	XR_001434920.1	lncRNA
XM_015095516.1	LOC101123551	mRNA	XR_001434920.1	lncRNA
XM_015095516.1	LOC101123551	mRNA	XR_001434920.1	lncRNA
XM_015096518.1	AASDH	mRNA	XR_001435107.1	lncRNA
XM_015096518.1	AASDH	mRNA	XR_001435107.1	lncRNA
XM_015096518.1	AASDH	mRNA	XR_001435107.1	lncRNA
XM_015096518.1	AASDH	mRNA	XR_001435107.1	lncRNA
XM_015096518.1	AASDH	mRNA	XR_001435107.1	lncRNA
XM_015096518.1	AASDH	mRNA	XR_001435107.1	lncRNA
XM_012176414.2	PHF14	mRNA	XR_001436817.1	lncRNA
XM_012176414.2	PHF14	mRNA	XR_001436817.1	lncRNA
XM_012176414.2	PHF14	mRNA	XR_001436817.1	lncRNA
XM_012176414.2	PHF14	mRNA	XR_001436817.1	lncRNA
XM_012176414.2	PHF14	mRNA	XR_001436817.1	lncRNA

XM_012176414.2	PHF14	mRNA	XR_001436817.1	lncRNA
XM_015100095.1	LOC101113523	mRNA	XR_001434920.1	lncRNA
XM_015100095.1	LOC101113523	mRNA	XR_001434920.1	lncRNA
XM_015100095.1	LOC101113523	mRNA	XR_001434920.1	lncRNA
XM_015100095.1	LOC101113523	mRNA	XR_001434920.1	lncRNA
XM_015100095.1	LOC101113523	mRNA	XR_001434920.1	lncRNA
XM_015100095.1	LOC101113523	mRNA	XR_001434920.1	lncRNA
XM_012094885.1	LOC101116597	mRNA	XR_001436817.1	lncRNA
XM_012094885.1	LOC101116597	mRNA	XR_001436817.1	lncRNA
XM_012094885.1	LOC101116597	mRNA	XR_001436817.1	lncRNA
XM_012094885.1	LOC101116597	mRNA	XR_001436817.1	lncRNA
XM_012094885.1	LOC101116597	mRNA	XR_001436817.1	lncRNA
XM_012094885.1	LOC101116597	mRNA	XR_001436817.1	lncRNA
XM_015098592.1	RABEP1	mRNA	XR_001433856.1	lncRNA
XM_015098592.1	RABEP1	mRNA	XR_001433856.1	lncRNA
XM_015098592.1	RABEP1	mRNA	XR_001433856.1	lncRNA
XM_015098592.1	RABEP1	mRNA	XR_001433856.1	lncRNA
XM_015098592.1	RABEP1	mRNA	XR_001433856.1	lncRNA
XM_015098592.1	RABEP1	mRNA	XR_001433856.1	lncRNA
XM_004012073.3	FNDC3A	mRNA	XR_001433856.1	lncRNA
XM_004012073.3	FNDC3A	mRNA	XR_001433856.1	lncRNA
XM_004012073.3	FNDC3A	mRNA	XR_001433856.1	lncRNA
XM_004012073.3	FNDC3A	mRNA	XR_001433856.1	lncRNA
XM_004012073.3	FNDC3A	mRNA	XR_001433856.1	lncRNA
XM_004012073.3	FNDC3A	mRNA	XR_001433856.1	lncRNA
XM_012096817.1	ERBB2IP	mRNA	XR_001433856.1	lncRNA
XM_012096817.1	ERBB2IP	mRNA	XR_001433856.1	lncRNA
XM_012096817.1	ERBB2IP	mRNA	XR_001433856.1	lncRNA
XM_012096817.1	ERBB2IP	mRNA	XR_001433856.1	lncRNA
XM_012096817.1	ERBB2IP	mRNA	XR_001433856.1	lncRNA
XM_012096817.1	ERBB2IP	mRNA	XR_001433856.1	lncRNA
XM_015102459.1	LOC101117971	mRNA	XR_001436817.1	lncRNA
XM_015102459.1	LOC101117971	mRNA	XR_001436817.1	lncRNA
XM_015102459.1	LOC101117971	mRNA	XR_001436817.1	lncRNA
XM_015102459.1	LOC101117971	mRNA	XR_001436817.1	lncRNA
XM_015102459.1	LOC101117971	mRNA	XR_001436817.1	lncRNA
XM_015102459.1	LOC101117971	mRNA	XR_001436817.1	lncRNA
XM_004020402.3	RTTN	mRNA	XR_001436817.1	lncRNA
XM_004020402.3	RTTN	mRNA	XR_001436817.1	lncRNA
XM_004020402.3	RTTN	mRNA	XR_001436817.1	lncRNA
XM_004020402.3	RTTN	mRNA	XR_001436817.1	lncRNA
XM_004020402.3	RTTN	mRNA	XR_001436817.1	lncRNA
XM_004020402.3	RTTN	mRNA	XR_001436817.1	lncRNA
XM_012190083.2	ZMYM6	mRNA	XR_001435107.1	lncRNA
XM_012190083.2	ZMYM6	mRNA	XR_001435107.1	lncRNA
XM_012190083.2	ZMYM6	mRNA	XR_001435107.1	lncRNA
XM_012190083.2	ZMYM6	mRNA	XR_001435107.1	lncRNA
XM_012190083.2	ZMYM6	mRNA	XR_001435107.1	lncRNA
XM_012190083.2	ZMYM6	mRNA	XR_001435107.1	lncRNA
XM_015098434.1	STXBP4	mRNA	XR_001433856.1	lncRNA
XM_015098434.1	STXBP4	mRNA	XR_001433856.1	lncRNA
XM_015098434.1	STXBP4	mRNA	XR_001433856.1	lncRNA
XM_015098434.1	STXBP4	mRNA	XR_001433856.1	lncRNA
XM_015098434.1	STXBP4	mRNA	XR_001433856.1	lncRNA

XM_015098434.1	STXBP4	mRNA	XR_001433856.1	lncRNA
XM_012159182.2	LOC101121820	mRNA	XR_001434920.1	lncRNA
XM_012159182.2	LOC101121820	mRNA	XR_001434920.1	lncRNA
XM_012159182.2	LOC101121820	mRNA	XR_001434920.1	lncRNA
XM_012159182.2	LOC101121820	mRNA	XR_001434920.1	lncRNA
XM_012159182.2	LOC101121820	mRNA	XR_001434920.1	lncRNA
XM_012159182.2	LOC101121820	mRNA	XR_001434920.1	lncRNA
XM_012190083.2	ZMYM6	mRNA	XR_001433856.1	lncRNA
XM_012190083.2	ZMYM6	mRNA	XR_001433856.1	lncRNA
XM_012190083.2	ZMYM6	mRNA	XR_001433856.1	lncRNA
XM_012190083.2	ZMYM6	mRNA	XR_001433856.1	lncRNA
XM_012190083.2	ZMYM6	mRNA	XR_001433856.1	lncRNA
XM_012190083.2	ZMYM6	mRNA	XR_001433856.1	lncRNA
XM_004012073.3	FNDC3A	mRNA	XR_001435107.1	lncRNA
XM_004012073.3	FNDC3A	mRNA	XR_001435107.1	lncRNA
XM_004012073.3	FNDC3A	mRNA	XR_001435107.1	lncRNA
XM_004012073.3	FNDC3A	mRNA	XR_001435107.1	lncRNA
XM_004012073.3	FNDC3A	mRNA	XR_001435107.1	lncRNA
XM_004012073.3	FNDC3A	mRNA	XR_001435107.1	lncRNA
XM_015102009.1	RALGAPA1	mRNA	XR_001433856.1	lncRNA
XM_015102009.1	RALGAPA1	mRNA	XR_001433856.1	lncRNA
XM_015102009.1	RALGAPA1	mRNA	XR_001433856.1	lncRNA
XM_015102009.1	RALGAPA1	mRNA	XR_001433856.1	lncRNA
XM_015102009.1	RALGAPA1	mRNA	XR_001433856.1	lncRNA
XM_015102009.1	RALGAPA1	mRNA	XR_001433856.1	lncRNA
XM_012181728.2	KIAA0586	mRNA	XR_001433856.1	lncRNA
XM_012181728.2	KIAA0586	mRNA	XR_001433856.1	lncRNA
XM_012181728.2	KIAA0586	mRNA	XR_001433856.1	lncRNA
XM_012181728.2	KIAA0586	mRNA	XR_001433856.1	lncRNA
XM_012181728.2	KIAA0586	mRNA	XR_001433856.1	lncRNA
XM_012181728.2	KIAA0586	mRNA	XR_001433856.1	lncRNA
XM_015100095.1	LOC101113523	mRNA	XR_001435107.1	lncRNA
XM_015100095.1	LOC101113523	mRNA	XR_001435107.1	lncRNA
XM_015100095.1	LOC101113523	mRNA	XR_001435107.1	lncRNA
XM_015100095.1	LOC101113523	mRNA	XR_001435107.1	lncRNA
XM_015100095.1	LOC101113523	mRNA	XR_001435107.1	lncRNA
XM_015100095.1	LOC101113523	mRNA	XR_001435107.1	lncRNA
XM_015096383.1	SGMS2	mRNA	XR_001433856.1	lncRNA
XM_015096383.1	SGMS2	mRNA	XR_001433856.1	lncRNA
XM_015096383.1	SGMS2	mRNA	XR_001433856.1	lncRNA
XM_015096383.1	SGMS2	mRNA	XR_001433856.1	lncRNA
XM_015096383.1	SGMS2	mRNA	XR_001433856.1	lncRNA
XM_015096383.1	SGMS2	mRNA	XR_001433856.1	lncRNA
XM_012187630.2	SOAT1	mRNA	XR_001436817.1	lncRNA
XM_012187630.2	SOAT1	mRNA	XR_001436817.1	lncRNA
XM_012187630.2	SOAT1	mRNA	XR_001436817.1	lncRNA
XM_012187630.2	SOAT1	mRNA	XR_001436817.1	lncRNA
XM_012187630.2	SOAT1	mRNA	XR_001436817.1	lncRNA
XM_012187630.2	SOAT1	mRNA	XR_001436817.1	lncRNA
XM_012097365.2	SH3D19	mRNA	TCONS_00049802	lncRNA
XM_012097365.2	SH3D19	mRNA	TCONS_00049802	lncRNA
XM_012097365.2	SH3D19	mRNA	TCONS_00049802	lncRNA
XM_012097365.2	SH3D19	mRNA	TCONS_00049802	lncRNA
XM_012097365.2	SH3D19	mRNA	TCONS_00049802	lncRNA

XM_012097365.2	SH3D19	mRNA	TCONS_00049802	lncRNA
XM_012187413.1	NRIP1	mRNA	XR_001434127.1	lncRNA
XM_012187413.1	NRIP1	mRNA	XR_001434127.1	lncRNA
XM_012187413.1	NRIP1	mRNA	XR_001434127.1	lncRNA
XM_012187413.1	NRIP1	mRNA	XR_001434127.1	lncRNA
XM_012187413.1	NRIP1	mRNA	XR_001434127.1	lncRNA
XM_012187413.1	NRIP1	mRNA	XR_001434127.1	lncRNA
XM_012173553.2	STRN	mRNA	TCONS_00049802	lncRNA
XM_012173553.2	STRN	mRNA	TCONS_00049802	lncRNA
XM_012173553.2	STRN	mRNA	TCONS_00049802	lncRNA
XM_012173553.2	STRN	mRNA	TCONS_00049802	lncRNA
XM_012173553.2	STRN	mRNA	TCONS_00049802	lncRNA
XM_012173553.2	STRN	mRNA	TCONS_00049802	lncRNA
XM_012097365.2	SH3D19	mRNA	XR_001023627.2	lncRNA
XM_012097365.2	SH3D19	mRNA	XR_001023627.2	lncRNA
XM_012097365.2	SH3D19	mRNA	XR_001023627.2	lncRNA
XM_012097365.2	SH3D19	mRNA	XR_001023627.2	lncRNA
XM_012097365.2	SH3D19	mRNA	XR_001023627.2	lncRNA
XM_012097365.2	SH3D19	mRNA	XR_001023627.2	lncRNA
XM_012185338.2	ZNF207	mRNA	XR_001023627.2	lncRNA
XM_012185338.2	ZNF207	mRNA	XR_001023627.2	lncRNA
XM_012185338.2	ZNF207	mRNA	XR_001023627.2	lncRNA
XM_012185338.2	ZNF207	mRNA	XR_001023627.2	lncRNA
XM_012185338.2	ZNF207	mRNA	XR_001023627.2	lncRNA
XM_012185338.2	ZNF207	mRNA	XR_001023627.2	lncRNA
XM_012180234.2	CNOT6L	mRNA	XR_001023627.2	lncRNA
XM_012180234.2	CNOT6L	mRNA	XR_001023627.2	lncRNA
XM_012180234.2	CNOT6L	mRNA	XR_001023627.2	lncRNA
XM_012180234.2	CNOT6L	mRNA	XR_001023627.2	lncRNA
XM_012180234.2	CNOT6L	mRNA	XR_001023627.2	lncRNA
XM_012180234.2	CNOT6L	mRNA	XR_001023627.2	lncRNA
XM_012187413.1	NRIP1	mRNA	XR_001023627.2	lncRNA
XM_012187413.1	NRIP1	mRNA	XR_001023627.2	lncRNA
XM_012187413.1	NRIP1	mRNA	XR_001023627.2	lncRNA
XM_012187413.1	NRIP1	mRNA	XR_001023627.2	lncRNA
XM_012187413.1	NRIP1	mRNA	XR_001023627.2	lncRNA
XM_012187413.1	NRIP1	mRNA	XR_001023627.2	lncRNA
XM_012182450.2	IBTK	mRNA	TCONS_00049802	lncRNA
XM_012182450.2	IBTK	mRNA	TCONS_00049802	lncRNA
XM_012182450.2	IBTK	mRNA	TCONS_00049802	lncRNA
XM_012182450.2	IBTK	mRNA	TCONS_00049802	lncRNA
XM_012182450.2	IBTK	mRNA	TCONS_00049802	lncRNA
XM_012182450.2	IBTK	mRNA	TCONS_00049802	lncRNA
XM_012182450.2	IBTK	mRNA	XR_001434127.1	lncRNA
XM_012182450.2	IBTK	mRNA	XR_001434127.1	lncRNA
XM_012182450.2	IBTK	mRNA	XR_001434127.1	lncRNA
XM_012182450.2	IBTK	mRNA	XR_001434127.1	lncRNA
XM_012182450.2	IBTK	mRNA	XR_001434127.1	lncRNA
XM_012182450.2	IBTK	mRNA	XR_001434127.1	lncRNA
XM_012173553.2	STRN	mRNA	XR_001434127.1	lncRNA
XM_012173553.2	STRN	mRNA	XR_001434127.1	lncRNA
XM_012173553.2	STRN	mRNA	XR_001434127.1	lncRNA
XM_012173553.2	STRN	mRNA	XR_001434127.1	lncRNA
XM_012173553.2	STRN	mRNA	XR_001434127.1	lncRNA

XM_012173553.2	STRN	mRNA	XR_001434127.1	lncRNA
XM_012103799.1	NEDD4L	mRNA	XR_001434127.1	lncRNA
XM_012103799.1	NEDD4L	mRNA	XR_001434127.1	lncRNA
XM_012103799.1	NEDD4L	mRNA	XR_001434127.1	lncRNA
XM_012103799.1	NEDD4L	mRNA	XR_001434127.1	lncRNA
XM_012103799.1	NEDD4L	mRNA	XR_001434127.1	lncRNA
XM_012103799.1	NEDD4L	mRNA	XR_001434127.1	lncRNA
XM_012185338.2	ZNF207	mRNA	XR_001434127.1	lncRNA
XM_012185338.2	ZNF207	mRNA	XR_001434127.1	lncRNA
XM_012185338.2	ZNF207	mRNA	XR_001434127.1	lncRNA
XM_012185338.2	ZNF207	mRNA	XR_001434127.1	lncRNA
XM_012185338.2	ZNF207	mRNA	XR_001434127.1	lncRNA
XM_012185338.2	ZNF207	mRNA	XR_001434127.1	lncRNA
XM_012170869.2	PAPOLG	mRNA	XR_001434127.1	lncRNA
XM_012170869.2	PAPOLG	mRNA	XR_001434127.1	lncRNA
XM_012170869.2	PAPOLG	mRNA	XR_001434127.1	lncRNA
XM_012170869.2	PAPOLG	mRNA	XR_001434127.1	lncRNA
XM_012170869.2	PAPOLG	mRNA	XR_001434127.1	lncRNA
XM_012170869.2	PAPOLG	mRNA	XR_001434127.1	lncRNA
XM_012105333.2	USP54	mRNA	XR_001436612.1	lncRNA
XM_012105333.2	USP54	mRNA	XR_001436612.1	lncRNA
XM_012105333.2	USP54	mRNA	XR_001436612.1	lncRNA
XM_012105333.2	USP54	mRNA	XR_001436612.1	lncRNA
XM_012105333.2	USP54	mRNA	XR_001436612.1	lncRNA
XM_012105333.2	USP54	mRNA	XR_001436612.1	lncRNA
XM_015092364.1	GART	mRNA	XR_001436612.1	lncRNA
XM_015092364.1	GART	mRNA	XR_001436612.1	lncRNA
XM_015092364.1	GART	mRNA	XR_001436612.1	lncRNA
XM_015092364.1	GART	mRNA	XR_001436612.1	lncRNA
XM_015092364.1	GART	mRNA	XR_001436612.1	lncRNA
XM_015092364.1	GART	mRNA	XR_001436612.1	lncRNA
XM_004009738.3	PPARGC1A	mRNA	XR_001436612.1	lncRNA
XM_004009738.3	PPARGC1A	mRNA	XR_001436612.1	lncRNA
XM_004009738.3	PPARGC1A	mRNA	XR_001436612.1	lncRNA
XM_004009738.3	PPARGC1A	mRNA	XR_001436612.1	lncRNA
XM_004009738.3	PPARGC1A	mRNA	XR_001436612.1	lncRNA
XM_004009738.3	PPARGC1A	mRNA	XR_001436612.1	lncRNA
XM_012184362.2	AKAP11	mRNA	XR_001436612.1	lncRNA
XM_012184362.2	AKAP11	mRNA	XR_001436612.1	lncRNA
XM_012184362.2	AKAP11	mRNA	XR_001436612.1	lncRNA
XM_012184362.2	AKAP11	mRNA	XR_001436612.1	lncRNA
XM_012184362.2	AKAP11	mRNA	XR_001436612.1	lncRNA
XM_012184362.2	AKAP11	mRNA	XR_001436612.1	lncRNA
XM_015098186.1	XPO4	mRNA	XR_001023303.2	lncRNA
XM_015098186.1	XPO4	mRNA	XR_001023303.2	lncRNA
XM_015098186.1	XPO4	mRNA	XR_001023303.2	lncRNA
XM_015098186.1	XPO4	mRNA	XR_001023303.2	lncRNA
XM_015098186.1	XPO4	mRNA	XR_001023303.2	lncRNA
XM_015097755.1	TMEM68	mRNA	XR_001041893.2	lncRNA
XM_015097755.1	TMEM68	mRNA	XR_001041893.2	lncRNA
XM_015097755.1	TMEM68	mRNA	XR_001041893.2	lncRNA
XM_015097755.1	TMEM68	mRNA	XR_001041893.2	lncRNA
XM_015097755.1	TMEM68	mRNA	XR_001041893.2	lncRNA
XM_012187413.1	NRIP1	mRNA	XR_001040147.1	lncRNA

XM_012187413.1	NRIP1	mRNA	XR_001040147.1	lncRNA
XM_012187413.1	NRIP1	mRNA	XR_001040147.1	lncRNA
XM_012187413.1	NRIP1	mRNA	XR_001040147.1	lncRNA
XM_012187413.1	NRIP1	mRNA	XR_001040147.1	lncRNA
XM_012187413.1	NRIP1	mRNA	XR_001040147.1	lncRNA
XM_015095237.1	DMTF1	mRNA	XR_001040147.1	lncRNA
XM_015095237.1	DMTF1	mRNA	XR_001040147.1	lncRNA
XM_015095237.1	DMTF1	mRNA	XR_001040147.1	lncRNA
XM_015095237.1	DMTF1	mRNA	XR_001040147.1	lncRNA
XM_015095237.1	DMTF1	mRNA	XR_001040147.1	lncRNA
XM_015095237.1	DMTF1	mRNA	XR_001040147.1	lncRNA
XM_015094252.1	DNMT3A	mRNA	XR_001436390.1	lncRNA
XM_015094252.1	DNMT3A	mRNA	XR_001436390.1	lncRNA
XM_015094252.1	DNMT3A	mRNA	XR_001436390.1	lncRNA
XM_015094252.1	DNMT3A	mRNA	XR_001436390.1	lncRNA
XM_015094252.1	DNMT3A	mRNA	XR_001436390.1	lncRNA
XM_015094252.1	DNMT3A	mRNA	XR_001436390.1	lncRNA
XM_012125769.2	MPDZ	mRNA	XR_001040147.1	lncRNA
XM_012125769.2	MPDZ	mRNA	XR_001040147.1	lncRNA
XM_012125769.2	MPDZ	mRNA	XR_001040147.1	lncRNA
XM_012125769.2	MPDZ	mRNA	XR_001040147.1	lncRNA
XM_012125769.2	MPDZ	mRNA	XR_001040147.1	lncRNA
XM_012125769.2	MPDZ	mRNA	XR_001040147.1	lncRNA
XM_012187161.1	CDC42BPA	mRNA	XR_001040147.1	lncRNA
XM_012187161.1	CDC42BPA	mRNA	XR_001040147.1	lncRNA
XM_012187161.1	CDC42BPA	mRNA	XR_001040147.1	lncRNA
XM_012187161.1	CDC42BPA	mRNA	XR_001040147.1	lncRNA
XM_012187161.1	CDC42BPA	mRNA	XR_001040147.1	lncRNA
XM_012187161.1	CDC42BPA	mRNA	XR_001040147.1	lncRNA
XM_012188274.2	CCDC7	mRNA	XR_001436065.1	lncRNA
XM_012188274.2	CCDC7	mRNA	XR_001436065.1	lncRNA
XM_012188274.2	CCDC7	mRNA	XR_001436065.1	lncRNA
XM_012188274.2	CCDC7	mRNA	XR_001436065.1	lncRNA
XM_012188274.2	CCDC7	mRNA	XR_001436065.1	lncRNA
XM_012188274.2	CCDC7	mRNA	XR_001436065.1	lncRNA
XM_004015120.2	PHLPP2	mRNA	XR_001024230.1	lncRNA
XM_004015120.2	PHLPP2	mRNA	XR_001024230.1	lncRNA
XM_004015120.2	PHLPP2	mRNA	XR_001024230.1	lncRNA
XM_004015120.2	PHLPP2	mRNA	XR_001024230.1	lncRNA
XM_004015120.2	PHLPP2	mRNA	XR_001024230.1	lncRNA
XM_004015120.2	PHLPP2	mRNA	XR_001024230.1	lncRNA
XM_015100438.1	CYLD	mRNA	XR_001436065.1	lncRNA
XM_015100438.1	CYLD	mRNA	XR_001436065.1	lncRNA
XM_015100438.1	CYLD	mRNA	XR_001436065.1	lncRNA
XM_015100438.1	CYLD	mRNA	XR_001436065.1	lncRNA
XM_015100438.1	CYLD	mRNA	XR_001436065.1	lncRNA
XM_015100438.1	CYLD	mRNA	XR_001436065.1	lncRNA
XM_012099509.2	BRPF1	mRNA	XR_001435726.1	lncRNA
XM_012099509.2	BRPF1	mRNA	XR_001435726.1	lncRNA
XM_012099509.2	BRPF1	mRNA	XR_001435726.1	lncRNA
XM_012099509.2	BRPF1	mRNA	XR_001435726.1	lncRNA
XM_012099509.2	BRPF1	mRNA	XR_001435726.1	lncRNA
XM_012099509.2	BRPF1	mRNA	XR_001435726.1	lncRNA
XM_015092063.1	FUBP1	mRNA	XR_001436064.1	lncRNA

XM_015092063.1	FUBP1	mRNA	XR_001436064.1	lncRNA
XM_015092063.1	FUBP1	mRNA	XR_001436064.1	lncRNA
XM_015092063.1	FUBP1	mRNA	XR_001436064.1	lncRNA
XM_015092063.1	FUBP1	mRNA	XR_001436064.1	lncRNA
XM_015092063.1	FUBP1	mRNA	XR_001436064.1	lncRNA
XM_015092063.1	FUBP1	mRNA	XR_001436065.1	lncRNA
XM_015092063.1	FUBP1	mRNA	XR_001436065.1	lncRNA
XM_015092063.1	FUBP1	mRNA	XR_001436065.1	lncRNA
XM_015092063.1	FUBP1	mRNA	XR_001436065.1	lncRNA
XM_015092063.1	FUBP1	mRNA	XR_001436065.1	lncRNA
XM_015092063.1	FUBP1	mRNA	XR_001436065.1	lncRNA
XM_004015120.2	PHLPP2	mRNA	XR_001435726.1	lncRNA
XM_004015120.2	PHLPP2	mRNA	XR_001435726.1	lncRNA
XM_004015120.2	PHLPP2	mRNA	XR_001435726.1	lncRNA
XM_004015120.2	PHLPP2	mRNA	XR_001435726.1	lncRNA
XM_004015120.2	PHLPP2	mRNA	XR_001435726.1	lncRNA
XM_004015120.2	PHLPP2	mRNA	XR_001435726.1	lncRNA
XM_012113353.2	SUSD1	mRNA	XR_001435726.1	lncRNA
XM_012113353.2	SUSD1	mRNA	XR_001435726.1	lncRNA
XM_012113353.2	SUSD1	mRNA	XR_001435726.1	lncRNA
XM_012113353.2	SUSD1	mRNA	XR_001435726.1	lncRNA
XM_012113353.2	SUSD1	mRNA	XR_001435726.1	lncRNA
XM_012113353.2	SUSD1	mRNA	XR_001435726.1	lncRNA
XM_012178157.2	RAPGEF6	mRNA	XR_001435726.1	lncRNA
XM_012178157.2	RAPGEF6	mRNA	XR_001435726.1	lncRNA
XM_012178157.2	RAPGEF6	mRNA	XR_001435726.1	lncRNA
XM_012178157.2	RAPGEF6	mRNA	XR_001435726.1	lncRNA
XM_012178157.2	RAPGEF6	mRNA	XR_001435726.1	lncRNA
XM_012178157.2	RAPGEF6	mRNA	XR_001435726.1	lncRNA
XM_015092971.1	SNX30	mRNA	XR_001435723.1	lncRNA
XM_015092971.1	SNX30	mRNA	XR_001435723.1	lncRNA
XM_015092971.1	SNX30	mRNA	XR_001435723.1	lncRNA
XM_015092971.1	SNX30	mRNA	XR_001435723.1	lncRNA
XM_015092971.1	SNX30	mRNA	XR_001435723.1	lncRNA
XM_015092971.1	SNX30	mRNA	XR_001435723.1	lncRNA
XM_012173383.2	DHX57	mRNA	XR_001435726.1	lncRNA
XM_012173383.2	DHX57	mRNA	XR_001435726.1	lncRNA
XM_012173383.2	DHX57	mRNA	XR_001435726.1	lncRNA
XM_012173383.2	DHX57	mRNA	XR_001435726.1	lncRNA
XM_012173383.2	DHX57	mRNA	XR_001435726.1	lncRNA
XM_012173383.2	DHX57	mRNA	XR_001435726.1	lncRNA
XM_012113353.2	SUSD1	mRNA	XR_001435723.1	lncRNA
XM_012113353.2	SUSD1	mRNA	XR_001435723.1	lncRNA
XM_012113353.2	SUSD1	mRNA	XR_001435723.1	lncRNA
XM_012113353.2	SUSD1	mRNA	XR_001435723.1	lncRNA
XM_012113353.2	SUSD1	mRNA	XR_001435723.1	lncRNA
XM_012113353.2	SUSD1	mRNA	XR_001435723.1	lncRNA
XM_015092063.1	FUBP1	mRNA	XR_001024230.1	lncRNA
XM_015092063.1	FUBP1	mRNA	XR_001024230.1	lncRNA
XM_015092063.1	FUBP1	mRNA	XR_001024230.1	lncRNA
XM_015092063.1	FUBP1	mRNA	XR_001024230.1	lncRNA
XM_015092063.1	FUBP1	mRNA	XR_001024230.1	lncRNA
XM_015092063.1	FUBP1	mRNA	XR_001024230.1	lncRNA
XM_004015120.2	PHLPP2	mRNA	XR_001435723.1	lncRNA

XM_004015120.2	PHLPP2	mRNA	XR_001435723.1	lncRNA
XM_004015120.2	PHLPP2	mRNA	XR_001435723.1	lncRNA
XM_004015120.2	PHLPP2	mRNA	XR_001435723.1	lncRNA
XM_004015120.2	PHLPP2	mRNA	XR_001435723.1	lncRNA
XM_004015120.2	PHLPP2	mRNA	XR_001435723.1	lncRNA
XM_012188274.2	CCDC7	mRNA	XR_001436064.1	lncRNA
XM_012188274.2	CCDC7	mRNA	XR_001436064.1	lncRNA
XM_012188274.2	CCDC7	mRNA	XR_001436064.1	lncRNA
XM_012188274.2	CCDC7	mRNA	XR_001436064.1	lncRNA
XM_012188274.2	CCDC7	mRNA	XR_001436064.1	lncRNA
XM_012188274.2	CCDC7	mRNA	XR_001436064.1	lncRNA
XM_012178157.2	RAPGEF6	mRNA	XR_001435723.1	lncRNA
XM_012178157.2	RAPGEF6	mRNA	XR_001435723.1	lncRNA
XM_012178157.2	RAPGEF6	mRNA	XR_001435723.1	lncRNA
XM_012178157.2	RAPGEF6	mRNA	XR_001435723.1	lncRNA
XM_012178157.2	RAPGEF6	mRNA	XR_001435723.1	lncRNA
XM_012178157.2	RAPGEF6	mRNA	XR_001435723.1	lncRNA
XM_015094225.1	ROCK2	mRNA	TCONS_00049802	lncRNA
XM_015094225.1	ROCK2	mRNA	TCONS_00049802	lncRNA
XM_015094225.1	ROCK2	mRNA	TCONS_00049802	lncRNA
XM_015094225.1	ROCK2	mRNA	TCONS_00049802	lncRNA
XM_015094225.1	ROCK2	mRNA	TCONS_00049802	lncRNA
XM_015094225.1	ROCK2	mRNA	TCONS_00049802	lncRNA
XM_015101001.1	RRAS2	mRNA	XR_001434127.1	lncRNA
XM_015101001.1	RRAS2	mRNA	XR_001434127.1	lncRNA
XM_015101001.1	RRAS2	mRNA	XR_001434127.1	lncRNA
XM_015101001.1	RRAS2	mRNA	XR_001434127.1	lncRNA
XM_015101001.1	RRAS2	mRNA	XR_001434127.1	lncRNA
XM_015101001.1	RRAS2	mRNA	XR_001434127.1	lncRNA
XM_012097035.1	C16H5orf42	mRNA	XR_001023627.2	lncRNA
XM_012097035.1	C16H5orf42	mRNA	XR_001023627.2	lncRNA
XM_012097035.1	C16H5orf42	mRNA	XR_001023627.2	lncRNA
XM_012097035.1	C16H5orf42	mRNA	XR_001023627.2	lncRNA
XM_012097035.1	C16H5orf42	mRNA	XR_001023627.2	lncRNA
XM_012097035.1	C16H5orf42	mRNA	XR_001023627.2	lncRNA
XM_015092364.1	GART	mRNA	XR_001023627.2	lncRNA
XM_015092364.1	GART	mRNA	XR_001023627.2	lncRNA
XM_015092364.1	GART	mRNA	XR_001023627.2	lncRNA
XM_015092364.1	GART	mRNA	XR_001023627.2	lncRNA
XM_015092364.1	GART	mRNA	XR_001023627.2	lncRNA
XM_015092364.1	GART	mRNA	XR_001023627.2	lncRNA
XM_012097035.1	C16H5orf42	mRNA	TCONS_00049802	lncRNA
XM_012097035.1	C16H5orf42	mRNA	TCONS_00049802	lncRNA
XM_012097035.1	C16H5orf42	mRNA	TCONS_00049802	lncRNA
XM_012097035.1	C16H5orf42	mRNA	TCONS_00049802	lncRNA
XM_012097035.1	C16H5orf42	mRNA	TCONS_00049802	lncRNA
XM_012097035.1	C16H5orf42	mRNA	TCONS_00049802	lncRNA
XM_015101001.1	RRAS2	mRNA	XR_001023627.2	lncRNA
XM_015101001.1	RRAS2	mRNA	XR_001023627.2	lncRNA
XM_015101001.1	RRAS2	mRNA	XR_001023627.2	lncRNA
XM_015101001.1	RRAS2	mRNA	XR_001023627.2	lncRNA
XM_015101001.1	RRAS2	mRNA	XR_001023627.2	lncRNA
XM_015101001.1	RRAS2	mRNA	XR_001023627.2	lncRNA
XM_015093065.1	FAM219A	mRNA	XR_001023627.2	lncRNA

XM_015093065.1	FAM219A	mRNA	XR_001023627.2	lncRNA
XM_015093065.1	FAM219A	mRNA	XR_001023627.2	lncRNA
XM_015093065.1	FAM219A	mRNA	XR_001023627.2	lncRNA
XM_015093065.1	FAM219A	mRNA	XR_001023627.2	lncRNA
XM_015093065.1	FAM219A	mRNA	XR_001023627.2	lncRNA
XM_012186029.1	FBXL20	mRNA	XR_001433837.1	lncRNA
XM_012186029.1	FBXL20	mRNA	XR_001433837.1	lncRNA
XM_012186029.1	FBXL20	mRNA	XR_001433837.1	lncRNA
XM_012186029.1	FBXL20	mRNA	XR_001433837.1	lncRNA
XM_012186029.1	FBXL20	mRNA	XR_001433837.1	lncRNA
XM_015092197.1	CD58	mRNA	XR_001433837.1	lncRNA
XM_015092197.1	CD58	mRNA	XR_001433837.1	lncRNA
XM_015092197.1	CD58	mRNA	XR_001433837.1	lncRNA
XM_015092197.1	CD58	mRNA	XR_001433837.1	lncRNA
XM_015092197.1	CD58	mRNA	XR_001433837.1	lncRNA
XM_012099944.1	PBRM1	mRNA	XR_001041893.2	lncRNA
XM_012099944.1	PBRM1	mRNA	XR_001041893.2	lncRNA
XM_012099944.1	PBRM1	mRNA	XR_001041893.2	lncRNA
XM_012099944.1	PBRM1	mRNA	XR_001041893.2	lncRNA
XM_012099944.1	PBRM1	mRNA	XR_001041893.2	lncRNA
XM_012098616.2	TBC1L1B	mRNA	XR_001024966.2	lncRNA
XM_012098616.2	TBC1L1B	mRNA	XR_001024966.2	lncRNA
XM_012098616.2	TBC1L1B	mRNA	XR_001024966.2	lncRNA
XM_012098616.2	TBC1L1B	mRNA	XR_001024966.2	lncRNA
XM_012098616.2	TBC1L1B	mRNA	XR_001024966.2	lncRNA
XM_012177522.2	VDAC1	mRNA	XR_001041893.2	lncRNA
XM_012177522.2	VDAC1	mRNA	XR_001041893.2	lncRNA
XM_012177522.2	VDAC1	mRNA	XR_001041893.2	lncRNA
XM_012177522.2	VDAC1	mRNA	XR_001041893.2	lncRNA
XM_012177522.2	VDAC1	mRNA	XR_001041893.2	lncRNA
XM_004011768.3	SAMD12	mRNA	XR_001040147.1	lncRNA
XM_004011768.3	SAMD12	mRNA	XR_001040147.1	lncRNA
XM_004011768.3	SAMD12	mRNA	XR_001040147.1	lncRNA
XM_004011768.3	SAMD12	mRNA	XR_001040147.1	lncRNA
XM_004011768.3	SAMD12	mRNA	XR_001040147.1	lncRNA
XM_004011768.3	SAMD12	mRNA	XR_001040147.1	lncRNA
XM_015099563.1	PARD3	mRNA	XR_001023627.2	lncRNA
XM_015099563.1	PARD3	mRNA	XR_001023627.2	lncRNA
XM_015099563.1	PARD3	mRNA	XR_001023627.2	lncRNA
XM_015099563.1	PARD3	mRNA	XR_001023627.2	lncRNA
XM_015099563.1	PARD3	mRNA	XR_001023627.2	lncRNA
XM_015099563.1	PARD3	mRNA	XR_001023627.2	lncRNA
XM_012097555.2	RAPGEF2	mRNA	TCONS_00049802	lncRNA
XM_012097555.2	RAPGEF2	mRNA	TCONS_00049802	lncRNA
XM_012097555.2	RAPGEF2	mRNA	TCONS_00049802	lncRNA
XM_012097555.2	RAPGEF2	mRNA	TCONS_00049802	lncRNA
XM_012097555.2	RAPGEF2	mRNA	TCONS_00049802	lncRNA
XM_012097555.2	RAPGEF2	mRNA	TCONS_00049802	lncRNA
XM_015099563.1	PARD3	mRNA	XR_001434127.1	lncRNA
XM_015099563.1	PARD3	mRNA	XR_001434127.1	lncRNA
XM_015099563.1	PARD3	mRNA	XR_001434127.1	lncRNA
XM_015099563.1	PARD3	mRNA	XR_001434127.1	lncRNA
XM_015099563.1	PARD3	mRNA	XR_001434127.1	lncRNA
XM_015099563.1	PARD3	mRNA	XR_001434127.1	lncRNA

XM_015102111.1	EIF4E3	mRNA	XR_001436612.1	lncRNA
XM_015102111.1	EIF4E3	mRNA	XR_001436612.1	lncRNA
XM_015102111.1	EIF4E3	mRNA	XR_001436612.1	lncRNA
XM_015102111.1	EIF4E3	mRNA	XR_001436612.1	lncRNA
XM_015102111.1	EIF4E3	mRNA	XR_001436612.1	lncRNA
XM_015097819.1	STAU2	mRNA	XR_001436612.1	lncRNA
XM_015097819.1	STAU2	mRNA	XR_001436612.1	lncRNA
XM_015097819.1	STAU2	mRNA	XR_001436612.1	lncRNA
XM_015097819.1	STAU2	mRNA	XR_001436612.1	lncRNA
XM_015097819.1	STAU2	mRNA	XR_001436612.1	lncRNA
XM_012143452.2	NRP2	mRNA	XR_001436612.1	lncRNA
XM_012143452.2	NRP2	mRNA	XR_001436612.1	lncRNA
XM_012143452.2	NRP2	mRNA	XR_001436612.1	lncRNA
XM_012143452.2	NRP2	mRNA	XR_001436612.1	lncRNA
XM_012143452.2	NRP2	mRNA	XR_001436612.1	lncRNA
XM_015098148.1	KATNAL1	mRNA	XR_001023303.2	lncRNA
XM_015098148.1	KATNAL1	mRNA	XR_001023303.2	lncRNA
XM_015098148.1	KATNAL1	mRNA	XR_001023303.2	lncRNA
XM_015098148.1	KATNAL1	mRNA	XR_001023303.2	lncRNA
XM_015098148.1	KATNAL1	mRNA	XR_001023303.2	lncRNA
XM_015092971.1	SNX30	mRNA	XR_001023303.2	lncRNA
XM_015092971.1	SNX30	mRNA	XR_001023303.2	lncRNA
XM_015092971.1	SNX30	mRNA	XR_001023303.2	lncRNA
XM_015092971.1	SNX30	mRNA	XR_001023303.2	lncRNA
XM_015092971.1	SNX30	mRNA	XR_001023303.2	lncRNA
XM_012099509.2	BRPF1	mRNA	XR_001024966.2	lncRNA
XM_012099509.2	BRPF1	mRNA	XR_001024966.2	lncRNA
XM_012099509.2	BRPF1	mRNA	XR_001024966.2	lncRNA
XM_012099509.2	BRPF1	mRNA	XR_001024966.2	lncRNA
XM_012099509.2	BRPF1	mRNA	XR_001024966.2	lncRNA
XM_012178221.1	CEP120	mRNA	XR_001041893.2	lncRNA
XM_012178221.1	CEP120	mRNA	XR_001041893.2	lncRNA
XM_012178221.1	CEP120	mRNA	XR_001041893.2	lncRNA
XM_012178221.1	CEP120	mRNA	XR_001041893.2	lncRNA
XM_012178221.1	CEP120	mRNA	XR_001041893.2	lncRNA
XM_015091887.1	MYCL	mRNA	XR_001024966.2	lncRNA
XM_015091887.1	MYCL	mRNA	XR_001024966.2	lncRNA
XM_015091887.1	MYCL	mRNA	XR_001024966.2	lncRNA
XM_015091887.1	MYCL	mRNA	XR_001024966.2	lncRNA
XM_015091887.1	MYCL	mRNA	XR_001024966.2	lncRNA
XM_012181434.2	RNF111	mRNA	XR_001433837.1	lncRNA
XM_012181434.2	RNF111	mRNA	XR_001433837.1	lncRNA
XM_012181434.2	RNF111	mRNA	XR_001433837.1	lncRNA
XM_012181434.2	RNF111	mRNA	XR_001433837.1	lncRNA
XM_012181434.2	RNF111	mRNA	XR_001433837.1	lncRNA
XM_012099944.1	PBRM1	mRNA	XR_001433837.1	lncRNA
XM_012099944.1	PBRM1	mRNA	XR_001433837.1	lncRNA
XM_012099944.1	PBRM1	mRNA	XR_001433837.1	lncRNA
XM_012099944.1	PBRM1	mRNA	XR_001433837.1	lncRNA
XM_012099944.1	PBRM1	mRNA	XR_001433837.1	lncRNA
XM_004009738.3	PPARGC1A	mRNA	XR_001025540.2	lncRNA
XM_004009738.3	PPARGC1A	mRNA	XR_001025540.2	lncRNA
XM_004009738.3	PPARGC1A	mRNA	XR_001025540.2	lncRNA
XM_004009738.3	PPARGC1A	mRNA	XR_001025540.2	lncRNA

XM_004009738.3	PPARGC1A	mRNA	XR_001025540.2	lncRNA
XM_012097555.2	RAPGEF2	mRNA	XR_001025540.2	lncRNA
XM_012097555.2	RAPGEF2	mRNA	XR_001025540.2	lncRNA
XM_012097555.2	RAPGEF2	mRNA	XR_001025540.2	lncRNA
XM_012097555.2	RAPGEF2	mRNA	XR_001025540.2	lncRNA
XM_012097555.2	RAPGEF2	mRNA	XR_001025540.2	lncRNA
XM_015103687.1	PTPN2	mRNA	XR_001025540.2	lncRNA
XM_015103687.1	PTPN2	mRNA	XR_001025540.2	lncRNA
XM_015103687.1	PTPN2	mRNA	XR_001025540.2	lncRNA
XM_015103687.1	PTPN2	mRNA	XR_001025540.2	lncRNA
XM_015103687.1	PTPN2	mRNA	XR_001025540.2	lncRNA
XM_012174737.2	PRKACB	mRNA	XR_001436390.1	lncRNA
XM_012174737.2	PRKACB	mRNA	XR_001436390.1	lncRNA
XM_012174737.2	PRKACB	mRNA	XR_001436390.1	lncRNA
XM_012174737.2	PRKACB	mRNA	XR_001436390.1	lncRNA
XM_012174737.2	PRKACB	mRNA	XR_001436390.1	lncRNA
XM_012174679.2	R3HDM2	mRNA	XR_001436390.1	lncRNA
XM_012174679.2	R3HDM2	mRNA	XR_001436390.1	lncRNA
XM_012174679.2	R3HDM2	mRNA	XR_001436390.1	lncRNA
XM_012174679.2	R3HDM2	mRNA	XR_001436390.1	lncRNA
XM_012174679.2	R3HDM2	mRNA	XR_001436390.1	lncRNA
XM_015092197.1	CD58	mRNA	XR_001436390.1	lncRNA
XM_015092197.1	CD58	mRNA	XR_001436390.1	lncRNA
XM_015092197.1	CD58	mRNA	XR_001436390.1	lncRNA
XM_015092197.1	CD58	mRNA	XR_001436390.1	lncRNA
XM_015092197.1	CD58	mRNA	XR_001436390.1	lncRNA
XM_012175096.2	ERGIC2	mRNA	XR_001436390.1	lncRNA
XM_012175096.2	ERGIC2	mRNA	XR_001436390.1	lncRNA
XM_012175096.2	ERGIC2	mRNA	XR_001436390.1	lncRNA
XM_012175096.2	ERGIC2	mRNA	XR_001436390.1	lncRNA
XM_012175096.2	ERGIC2	mRNA	XR_001436390.1	lncRNA
XM_012174722.2	PRKACB	mRNA	XR_001436390.1	lncRNA
XM_012174722.2	PRKACB	mRNA	XR_001436390.1	lncRNA
XM_012174722.2	PRKACB	mRNA	XR_001436390.1	lncRNA
XM_012174722.2	PRKACB	mRNA	XR_001436390.1	lncRNA
XM_012174722.2	PRKACB	mRNA	XR_001436390.1	lncRNA
XM_012184822.2	RBM26	mRNA	XR_001436390.1	lncRNA
XM_012184822.2	RBM26	mRNA	XR_001436390.1	lncRNA
XM_012184822.2	RBM26	mRNA	XR_001436390.1	lncRNA
XM_012184822.2	RBM26	mRNA	XR_001436390.1	lncRNA
XM_012184822.2	RBM26	mRNA	XR_001436390.1	lncRNA
XM_012143452.2	NRP2	mRNA	XR_001436390.1	lncRNA
XM_012143452.2	NRP2	mRNA	XR_001436390.1	lncRNA
XM_012143452.2	NRP2	mRNA	XR_001436390.1	lncRNA
XM_012143452.2	NRP2	mRNA	XR_001436390.1	lncRNA
XM_012143452.2	NRP2	mRNA	XR_001436390.1	lncRNA
XM_015096456.1	APBB2	mRNA	XR_001436390.1	lncRNA
XM_015096456.1	APBB2	mRNA	XR_001436390.1	lncRNA
XM_015096456.1	APBB2	mRNA	XR_001436390.1	lncRNA
XM_015096456.1	APBB2	mRNA	XR_001436390.1	lncRNA
XM_015096456.1	APBB2	mRNA	XR_001436390.1	lncRNA
XM_004004866.2	METTL21A	mRNA	XR_001436390.1	lncRNA
XM_004004866.2	METTL21A	mRNA	XR_001436390.1	lncRNA
XM_004004866.2	METTL21A	mRNA	XR_001436390.1	lncRNA

XM_004004866.2	METTL21A	mRNA	XR_001436390.1	lncRNA
XM_004004866.2	METTL21A	mRNA	XR_001436390.1	lncRNA
XM_012124921.1	KIAA2026	mRNA	XR_001436390.1	lncRNA
XM_012124921.1	KIAA2026	mRNA	XR_001436390.1	lncRNA
XM_012124921.1	KIAA2026	mRNA	XR_001436390.1	lncRNA
XM_012124921.1	KIAA2026	mRNA	XR_001436390.1	lncRNA
XM_012124921.1	KIAA2026	mRNA	XR_001436390.1	lncRNA
XM_004020402.3	RTTN	mRNA	XR_001436612.1	lncRNA
XM_004020402.3	RTTN	mRNA	XR_001436612.1	lncRNA
XM_004020402.3	RTTN	mRNA	XR_001436612.1	lncRNA
XM_004020402.3	RTTN	mRNA	XR_001436612.1	lncRNA
XM_004020402.3	RTTN	mRNA	XR_001436612.1	lncRNA
XM_015100095.1	LOC101113523	mRNA	XR_001436612.1	lncRNA
XM_015100095.1	LOC101113523	mRNA	XR_001436612.1	lncRNA
XM_015100095.1	LOC101113523	mRNA	XR_001436612.1	lncRNA
XM_015100095.1	LOC101113523	mRNA	XR_001436612.1	lncRNA
XM_015100095.1	LOC101113523	mRNA	XR_001436612.1	lncRNA
XM_015102459.1	LOC101117971	mRNA	XR_001436612.1	lncRNA
XM_015102459.1	LOC101117971	mRNA	XR_001436612.1	lncRNA
XM_015102459.1	LOC101117971	mRNA	XR_001436612.1	lncRNA
XM_015102459.1	LOC101117971	mRNA	XR_001436612.1	lncRNA
XM_015102459.1	LOC101117971	mRNA	XR_001436612.1	lncRNA
XM_004011835.3	RAD54B	mRNA	XR_001436612.1	lncRNA
XM_004011835.3	RAD54B	mRNA	XR_001436612.1	lncRNA
XM_004011835.3	RAD54B	mRNA	XR_001436612.1	lncRNA
XM_004011835.3	RAD54B	mRNA	XR_001436612.1	lncRNA
XM_004011835.3	RAD54B	mRNA	XR_001436612.1	lncRNA
XM_012187413.1	NRIP1	mRNA	XR_001023303.2	lncRNA
XM_012187413.1	NRIP1	mRNA	XR_001023303.2	lncRNA
XM_012187413.1	NRIP1	mRNA	XR_001023303.2	lncRNA
XM_012187413.1	NRIP1	mRNA	XR_001023303.2	lncRNA
XM_012187413.1	NRIP1	mRNA	XR_001023303.2	lncRNA
XM_012173553.2	STRN	mRNA	XR_001023303.2	lncRNA
XM_012173553.2	STRN	mRNA	XR_001023303.2	lncRNA
XM_012173553.2	STRN	mRNA	XR_001023303.2	lncRNA
XM_012173553.2	STRN	mRNA	XR_001023303.2	lncRNA
XM_012173553.2	STRN	mRNA	XR_001023303.2	lncRNA
XM_012098812.2	RALGAPA1	mRNA	XR_001024966.2	lncRNA
XM_012098812.2	RALGAPA1	mRNA	XR_001024966.2	lncRNA
XM_012098812.2	RALGAPA1	mRNA	XR_001024966.2	lncRNA
XM_012098812.2	RALGAPA1	mRNA	XR_001024966.2	lncRNA
XM_012098812.2	RALGAPA1	mRNA	XR_001024966.2	lncRNA
XM_012173553.2	STRN	mRNA	XR_001041893.2	lncRNA
XM_012173553.2	STRN	mRNA	XR_001041893.2	lncRNA
XM_012173553.2	STRN	mRNA	XR_001041893.2	lncRNA
XM_012173553.2	STRN	mRNA	XR_001041893.2	lncRNA
XM_012173553.2	STRN	mRNA	XR_001041893.2	lncRNA
XM_015099337.1	RALGPS2	mRNA	XR_001433837.1	lncRNA
XM_015099337.1	RALGPS2	mRNA	XR_001433837.1	lncRNA
XM_015099337.1	RALGPS2	mRNA	XR_001433837.1	lncRNA
XM_015099337.1	RALGPS2	mRNA	XR_001433837.1	lncRNA
XM_015099337.1	RALGPS2	mRNA	XR_001433837.1	lncRNA
XM_004015120.2	PHLPP2	mRNA	XR_001433837.1	lncRNA
XM_004015120.2	PHLPP2	mRNA	XR_001433837.1	lncRNA

XM_004015120.2	PHLPP2	mRNA	XR_001433837.1	lncRNA
XM_004015120.2	PHLPP2	mRNA	XR_001433837.1	lncRNA
XM_004015120.2	PHLPP2	mRNA	XR_001433837.1	lncRNA
XM_015092971.1	SNX30	mRNA	XR_001433837.1	lncRNA
XM_015092971.1	SNX30	mRNA	XR_001433837.1	lncRNA
XM_015092971.1	SNX30	mRNA	XR_001433837.1	lncRNA
XM_015092971.1	SNX30	mRNA	XR_001433837.1	lncRNA
XM_015092971.1	SNX30	mRNA	XR_001433837.1	lncRNA
XM_012146156.1	USP37	mRNA	XR_001433837.1	lncRNA
XM_012146156.1	USP37	mRNA	XR_001433837.1	lncRNA
XM_012146156.1	USP37	mRNA	XR_001433837.1	lncRNA
XM_012146156.1	USP37	mRNA	XR_001433837.1	lncRNA
XM_012146156.1	USP37	mRNA	XR_001433837.1	lncRNA
XM_012181265.2	PLA2G4F	mRNA	XR_001433837.1	lncRNA
XM_012181265.2	PLA2G4F	mRNA	XR_001433837.1	lncRNA
XM_012181265.2	PLA2G4F	mRNA	XR_001433837.1	lncRNA
XM_012181265.2	PLA2G4F	mRNA	XR_001433837.1	lncRNA
XM_012181265.2	PLA2G4F	mRNA	XR_001433837.1	lncRNA
XM_015094225.1	ROCK2	mRNA	XR_001041893.2	lncRNA
XM_015094225.1	ROCK2	mRNA	XR_001041893.2	lncRNA
XM_015094225.1	ROCK2	mRNA	XR_001041893.2	lncRNA
XM_015094225.1	ROCK2	mRNA	XR_001041893.2	lncRNA
XM_015094225.1	ROCK2	mRNA	XR_001041893.2	lncRNA
XM_012174704.2	ZBTB39	mRNA	XR_001024966.2	lncRNA
XM_012174704.2	ZBTB39	mRNA	XR_001024966.2	lncRNA
XM_012174704.2	ZBTB39	mRNA	XR_001024966.2	lncRNA
XM_012174704.2	ZBTB39	mRNA	XR_001024966.2	lncRNA
XM_012174704.2	ZBTB39	mRNA	XR_001024966.2	lncRNA
XM_012097374.2	LRBA	mRNA	XR_001023303.2	lncRNA
XM_012097374.2	LRBA	mRNA	XR_001023303.2	lncRNA
XM_012097374.2	LRBA	mRNA	XR_001023303.2	lncRNA
XM_012097374.2	LRBA	mRNA	XR_001023303.2	lncRNA
XM_012097374.2	LRBA	mRNA	XR_001023303.2	lncRNA
XM_012097374.2	LRBA	mRNA	XR_001024966.2	lncRNA
XM_012097374.2	LRBA	mRNA	XR_001024966.2	lncRNA
XM_012097374.2	LRBA	mRNA	XR_001024966.2	lncRNA
XM_012097374.2	LRBA	mRNA	XR_001024966.2	lncRNA
XM_012097374.2	LRBA	mRNA	XR_001024966.2	lncRNA
XM_015098434.1	STXBP4	mRNA	XR_001434127.1	lncRNA
XM_015098434.1	STXBP4	mRNA	XR_001434127.1	lncRNA
XM_015098434.1	STXBP4	mRNA	XR_001434127.1	lncRNA
XM_015098434.1	STXBP4	mRNA	XR_001434127.1	lncRNA
XM_015098434.1	STXBP4	mRNA	XR_001434127.1	lncRNA
XM_012188097.2	PLCB1	mRNA	TCONS_00049802	lncRNA
XM_012188097.2	PLCB1	mRNA	TCONS_00049802	lncRNA
XM_012188097.2	PLCB1	mRNA	TCONS_00049802	lncRNA
XM_012188097.2	PLCB1	mRNA	TCONS_00049802	lncRNA
XM_012188097.2	PLCB1	mRNA	TCONS_00049802	lncRNA
XM_012181434.2	RNF111	mRNA	TCONS_00049802	lncRNA
XM_012181434.2	RNF111	mRNA	TCONS_00049802	lncRNA
XM_012181434.2	RNF111	mRNA	TCONS_00049802	lncRNA
XM_012181434.2	RNF111	mRNA	TCONS_00049802	lncRNA
XM_012181434.2	RNF111	mRNA	TCONS_00049802	lncRNA
XM_012181434.2	RNF111	mRNA	XR_001434127.1	lncRNA

XM_012181434.2	RNF111	mRNA	XR_001434127.1	lncRNA
XM_012181434.2	RNF111	mRNA	XR_001434127.1	lncRNA
XM_012181434.2	RNF111	mRNA	XR_001434127.1	lncRNA
XM_012181434.2	RNF111	mRNA	XR_001434127.1	lncRNA
XM_012176761.2	MDFIC	mRNA	TCONS_00049802	lncRNA
XM_012176761.2	MDFIC	mRNA	TCONS_00049802	lncRNA
XM_012176761.2	MDFIC	mRNA	TCONS_00049802	lncRNA
XM_012176761.2	MDFIC	mRNA	TCONS_00049802	lncRNA
XM_012176761.2	MDFIC	mRNA	TCONS_00049802	lncRNA
XM_012176761.2	MDFIC	mRNA	XR_001434127.1	lncRNA
XM_012176761.2	MDFIC	mRNA	XR_001434127.1	lncRNA
XM_012176761.2	MDFIC	mRNA	XR_001434127.1	lncRNA
XM_012176761.2	MDFIC	mRNA	XR_001434127.1	lncRNA
XM_012176761.2	MDFIC	mRNA	XR_001434127.1	lncRNA
XM_012099944.1	PBRM1	mRNA	TCONS_00049802	lncRNA
XM_012099944.1	PBRM1	mRNA	TCONS_00049802	lncRNA
XM_012099944.1	PBRM1	mRNA	TCONS_00049802	lncRNA
XM_012099944.1	PBRM1	mRNA	TCONS_00049802	lncRNA
XM_012099944.1	PBRM1	mRNA	TCONS_00049802	lncRNA
XM_015096518.1	AASDH	mRNA	XR_001023627.2	lncRNA
XM_015096518.1	AASDH	mRNA	XR_001023627.2	lncRNA
XM_015096518.1	AASDH	mRNA	XR_001023627.2	lncRNA
XM_015096518.1	AASDH	mRNA	XR_001023627.2	lncRNA
XM_015096518.1	AASDH	mRNA	XR_001023627.2	lncRNA
XM_012099944.1	PBRM1	mRNA	XR_001434127.1	lncRNA
XM_012099944.1	PBRM1	mRNA	XR_001434127.1	lncRNA
XM_012099944.1	PBRM1	mRNA	XR_001434127.1	lncRNA
XM_012099944.1	PBRM1	mRNA	XR_001434127.1	lncRNA
XM_012099944.1	PBRM1	mRNA	XR_001434127.1	lncRNA
XM_015092197.1	CD58	mRNA	XR_001435723.1	lncRNA
XM_015092197.1	CD58	mRNA	XR_001435723.1	lncRNA
XM_015092197.1	CD58	mRNA	XR_001435723.1	lncRNA
XM_015092197.1	CD58	mRNA	XR_001435723.1	lncRNA
XM_015092197.1	CD58	mRNA	XR_001435723.1	lncRNA
XM_012106892.2	TAF1	mRNA	XR_001436064.1	lncRNA
XM_012106892.2	TAF1	mRNA	XR_001436064.1	lncRNA
XM_012106892.2	TAF1	mRNA	XR_001436064.1	lncRNA
XM_012106892.2	TAF1	mRNA	XR_001436064.1	lncRNA
XM_012106892.2	TAF1	mRNA	XR_001436064.1	lncRNA
XM_015092005.1	MROH7	mRNA	XR_001436064.1	lncRNA
XM_015092005.1	MROH7	mRNA	XR_001436064.1	lncRNA
XM_015092005.1	MROH7	mRNA	XR_001436064.1	lncRNA
XM_015092005.1	MROH7	mRNA	XR_001436064.1	lncRNA
XM_015092005.1	MROH7	mRNA	XR_001436064.1	lncRNA
XM_012174722.2	PRKACB	mRNA	XR_001436064.1	lncRNA
XM_012174722.2	PRKACB	mRNA	XR_001436064.1	lncRNA
XM_012174722.2	PRKACB	mRNA	XR_001436064.1	lncRNA
XM_012174722.2	PRKACB	mRNA	XR_001436064.1	lncRNA
XM_012174722.2	PRKACB	mRNA	XR_001436064.1	lncRNA
XM_015097749.1	PCMTD1	mRNA	XR_001436064.1	lncRNA
XM_015097749.1	PCMTD1	mRNA	XR_001436064.1	lncRNA
XM_015097749.1	PCMTD1	mRNA	XR_001436064.1	lncRNA
XM_015097749.1	PCMTD1	mRNA	XR_001436064.1	lncRNA
XM_015097749.1	PCMTD1	mRNA	XR_001436064.1	lncRNA

XM_012100080.2	ARIH2	mRNA	XR_001435723.1	lncRNA
XM_012100080.2	ARIH2	mRNA	XR_001435723.1	lncRNA
XM_012100080.2	ARIH2	mRNA	XR_001435723.1	lncRNA
XM_012100080.2	ARIH2	mRNA	XR_001435723.1	lncRNA
XM_012100080.2	ARIH2	mRNA	XR_001435723.1	lncRNA
XM_012176647.2	NAPEPLD	mRNA	XR_001435723.1	lncRNA
XM_012176647.2	NAPEPLD	mRNA	XR_001435723.1	lncRNA
XM_012176647.2	NAPEPLD	mRNA	XR_001435723.1	lncRNA
XM_012176647.2	NAPEPLD	mRNA	XR_001435723.1	lncRNA
XM_012176647.2	NAPEPLD	mRNA	XR_001435723.1	lncRNA
XM_012181270.1	VPS39	mRNA	XR_001435723.1	lncRNA
XM_012181270.1	VPS39	mRNA	XR_001435723.1	lncRNA
XM_012181270.1	VPS39	mRNA	XR_001435723.1	lncRNA
XM_012181270.1	VPS39	mRNA	XR_001435723.1	lncRNA
XM_012181270.1	VPS39	mRNA	XR_001435723.1	lncRNA
XM_012174722.2	PRKACB	mRNA	XR_001435723.1	lncRNA
XM_012174722.2	PRKACB	mRNA	XR_001435723.1	lncRNA
XM_012174722.2	PRKACB	mRNA	XR_001435723.1	lncRNA
XM_012174722.2	PRKACB	mRNA	XR_001435723.1	lncRNA
XM_012174722.2	PRKACB	mRNA	XR_001435723.1	lncRNA
XM_012186029.1	FBXL20	mRNA	XR_001436064.1	lncRNA
XM_012186029.1	FBXL20	mRNA	XR_001436064.1	lncRNA
XM_012186029.1	FBXL20	mRNA	XR_001436064.1	lncRNA
XM_012186029.1	FBXL20	mRNA	XR_001436064.1	lncRNA
XM_012186029.1	FBXL20	mRNA	XR_001436064.1	lncRNA
XM_015096221.1	PPIP5K2	mRNA	XR_001435723.1	lncRNA
XM_015096221.1	PPIP5K2	mRNA	XR_001435723.1	lncRNA
XM_015096221.1	PPIP5K2	mRNA	XR_001435723.1	lncRNA
XM_015096221.1	PPIP5K2	mRNA	XR_001435723.1	lncRNA
XM_015096221.1	PPIP5K2	mRNA	XR_001435723.1	lncRNA
XM_012098071.2	RNF185	mRNA	XR_001435723.1	lncRNA
XM_012098071.2	RNF185	mRNA	XR_001435723.1	lncRNA
XM_012098071.2	RNF185	mRNA	XR_001435723.1	lncRNA
XM_012098071.2	RNF185	mRNA	XR_001435723.1	lncRNA
XM_012098071.2	RNF185	mRNA	XR_001435723.1	lncRNA
XM_012101006.2	CCHCR1	mRNA	XR_001435723.1	lncRNA
XM_012101006.2	CCHCR1	mRNA	XR_001435723.1	lncRNA
XM_012101006.2	CCHCR1	mRNA	XR_001435723.1	lncRNA
XM_012101006.2	CCHCR1	mRNA	XR_001435723.1	lncRNA
XM_012101006.2	CCHCR1	mRNA	XR_001435723.1	lncRNA
XM_015103688.1	PTPN2	mRNA	XR_001435723.1	lncRNA
XM_015103688.1	PTPN2	mRNA	XR_001435723.1	lncRNA
XM_015103688.1	PTPN2	mRNA	XR_001435723.1	lncRNA
XM_015103688.1	PTPN2	mRNA	XR_001435723.1	lncRNA
XM_015103688.1	PTPN2	mRNA	XR_001435723.1	lncRNA
XM_015094919.1	WBP11	mRNA	XR_001435723.1	lncRNA
XM_015094919.1	WBP11	mRNA	XR_001435723.1	lncRNA
XM_015094919.1	WBP11	mRNA	XR_001435723.1	lncRNA
XM_015094919.1	WBP11	mRNA	XR_001435723.1	lncRNA
XM_015094919.1	WBP11	mRNA	XR_001435723.1	lncRNA
XM_015102111.1	EIF4E3	mRNA	XR_001435723.1	lncRNA
XM_015102111.1	EIF4E3	mRNA	XR_001435723.1	lncRNA
XM_015102111.1	EIF4E3	mRNA	XR_001435723.1	lncRNA
XM_015102111.1	EIF4E3	mRNA	XR_001435723.1	lncRNA

XM_015102111.1	EIF4E3	mRNA	XR_001435723.1	lncRNA
XM_012106112.2	ARAF	mRNA	XR_001435723.1	lncRNA
XM_012106112.2	ARAF	mRNA	XR_001435723.1	lncRNA
XM_012106112.2	ARAF	mRNA	XR_001435723.1	lncRNA
XM_012106112.2	ARAF	mRNA	XR_001435723.1	lncRNA
XM_012106112.2	ARAF	mRNA	XR_001435723.1	lncRNA
XM_012106112.2	ARAF	mRNA	XR_001436064.1	lncRNA
XM_012106112.2	ARAF	mRNA	XR_001436064.1	lncRNA
XM_012106112.2	ARAF	mRNA	XR_001436064.1	lncRNA
XM_012106112.2	ARAF	mRNA	XR_001436064.1	lncRNA
XM_012106112.2	ARAF	mRNA	XR_001436064.1	lncRNA
XM_012143452.2	NRP2	mRNA	XR_001435723.1	lncRNA
XM_012143452.2	NRP2	mRNA	XR_001435723.1	lncRNA
XM_012143452.2	NRP2	mRNA	XR_001435723.1	lncRNA
XM_012143452.2	NRP2	mRNA	XR_001435723.1	lncRNA
XM_012143452.2	NRP2	mRNA	XR_001435723.1	lncRNA
XM_015094585.1	ATP2B1	mRNA	XR_001435723.1	lncRNA
XM_015094585.1	ATP2B1	mRNA	XR_001435723.1	lncRNA
XM_015094585.1	ATP2B1	mRNA	XR_001435723.1	lncRNA
XM_015094585.1	ATP2B1	mRNA	XR_001435723.1	lncRNA
XM_015094585.1	ATP2B1	mRNA	XR_001435723.1	lncRNA
XM_015094585.1	ATP2B1	mRNA	XR_001435726.1	lncRNA
XM_015094585.1	ATP2B1	mRNA	XR_001435726.1	lncRNA
XM_015094585.1	ATP2B1	mRNA	XR_001435726.1	lncRNA
XM_015094585.1	ATP2B1	mRNA	XR_001435726.1	lncRNA
XM_015094585.1	ATP2B1	mRNA	XR_001435726.1	lncRNA
XM_012176469.2	MACC1	mRNA	XR_001435726.1	lncRNA
XM_012176469.2	MACC1	mRNA	XR_001435726.1	lncRNA
XM_012176469.2	MACC1	mRNA	XR_001435726.1	lncRNA
XM_012176469.2	MACC1	mRNA	XR_001435726.1	lncRNA
XM_012176469.2	MACC1	mRNA	XR_001435726.1	lncRNA
XM_012097252.2	RHOF	mRNA	XR_001435726.1	lncRNA
XM_012097252.2	RHOF	mRNA	XR_001435726.1	lncRNA
XM_012097252.2	RHOF	mRNA	XR_001435726.1	lncRNA
XM_012097252.2	RHOF	mRNA	XR_001435726.1	lncRNA
XM_012097252.2	RHOF	mRNA	XR_001435726.1	lncRNA
XM_015096639.1	CC2L1A	mRNA	XR_001435726.1	lncRNA
XM_015096639.1	CC2L1A	mRNA	XR_001435726.1	lncRNA
XM_015096639.1	CC2L1A	mRNA	XR_001435726.1	lncRNA
XM_015096639.1	CC2L1A	mRNA	XR_001435726.1	lncRNA
XM_015096639.1	CC2L1A	mRNA	XR_001435726.1	lncRNA
XM_015092004.1	MROH7	mRNA	XR_001436065.1	lncRNA
XM_015092004.1	MROH7	mRNA	XR_001436065.1	lncRNA
XM_015092004.1	MROH7	mRNA	XR_001436065.1	lncRNA
XM_015092004.1	MROH7	mRNA	XR_001436065.1	lncRNA
XM_015092004.1	MROH7	mRNA	XR_001436065.1	lncRNA
XM_012098071.2	RNF185	mRNA	XR_001024230.1	lncRNA
XM_012098071.2	RNF185	mRNA	XR_001024230.1	lncRNA
XM_012098071.2	RNF185	mRNA	XR_001024230.1	lncRNA
XM_012098071.2	RNF185	mRNA	XR_001024230.1	lncRNA
XM_012098071.2	RNF185	mRNA	XR_001024230.1	lncRNA
XM_015102111.1	EIF4E3	mRNA	XR_001435726.1	lncRNA
XM_015102111.1	EIF4E3	mRNA	XR_001435726.1	lncRNA
XM_015102111.1	EIF4E3	mRNA	XR_001435726.1	lncRNA

XM_015102111.1	EIF4E3	mRNA	XR_001435726.1	lncRNA
XM_015102111.1	EIF4E3	mRNA	XR_001435726.1	lncRNA
XM_012186029.1	FBXL20	mRNA	XR_001024230.1	lncRNA
XM_012186029.1	FBXL20	mRNA	XR_001024230.1	lncRNA
XM_012186029.1	FBXL20	mRNA	XR_001024230.1	lncRNA
XM_012186029.1	FBXL20	mRNA	XR_001024230.1	lncRNA
XM_012186029.1	FBXL20	mRNA	XR_001024230.1	lncRNA
XM_015103688.1	PTPN2	mRNA	XR_001435726.1	lncRNA
XM_015103688.1	PTPN2	mRNA	XR_001435726.1	lncRNA
XM_015103688.1	PTPN2	mRNA	XR_001435726.1	lncRNA
XM_015103688.1	PTPN2	mRNA	XR_001435726.1	lncRNA
XM_015103688.1	PTPN2	mRNA	XR_001435726.1	lncRNA
XM_012181270.1	VPS39	mRNA	XR_001435726.1	lncRNA
XM_012181270.1	VPS39	mRNA	XR_001435726.1	lncRNA
XM_012181270.1	VPS39	mRNA	XR_001435726.1	lncRNA
XM_012181270.1	VPS39	mRNA	XR_001435726.1	lncRNA
XM_012181270.1	VPS39	mRNA	XR_001435726.1	lncRNA
XM_015103688.1	PTPN2	mRNA	XR_001024230.1	lncRNA
XM_015103688.1	PTPN2	mRNA	XR_001024230.1	lncRNA
XM_015103688.1	PTPN2	mRNA	XR_001024230.1	lncRNA
XM_015103688.1	PTPN2	mRNA	XR_001024230.1	lncRNA
XM_015103688.1	PTPN2	mRNA	XR_001024230.1	lncRNA
XM_015092197.1	CD58	mRNA	XR_001435726.1	lncRNA
XM_015092197.1	CD58	mRNA	XR_001435726.1	lncRNA
XM_015092197.1	CD58	mRNA	XR_001435726.1	lncRNA
XM_015092197.1	CD58	mRNA	XR_001435726.1	lncRNA
XM_015092197.1	CD58	mRNA	XR_001435726.1	lncRNA
XM_012176647.2	NAPEPLD	mRNA	XR_001435726.1	lncRNA
XM_012176647.2	NAPEPLD	mRNA	XR_001435726.1	lncRNA
XM_012176647.2	NAPEPLD	mRNA	XR_001435726.1	lncRNA
XM_012176647.2	NAPEPLD	mRNA	XR_001435726.1	lncRNA
XM_012176647.2	NAPEPLD	mRNA	XR_001435726.1	lncRNA
XM_015092197.1	CD58	mRNA	XR_001436065.1	lncRNA
XM_015092197.1	CD58	mRNA	XR_001436065.1	lncRNA
XM_015092197.1	CD58	mRNA	XR_001436065.1	lncRNA
XM_015092197.1	CD58	mRNA	XR_001436065.1	lncRNA
XM_015092197.1	CD58	mRNA	XR_001436065.1	lncRNA
XM_012106892.2	TAF1	mRNA	XR_001436065.1	lncRNA
XM_012106892.2	TAF1	mRNA	XR_001436065.1	lncRNA
XM_012106892.2	TAF1	mRNA	XR_001436065.1	lncRNA
XM_012106892.2	TAF1	mRNA	XR_001436065.1	lncRNA
XM_012106892.2	TAF1	mRNA	XR_001436065.1	lncRNA
XM_012176469.2	MACC1	mRNA	XR_001024230.1	lncRNA
XM_012176469.2	MACC1	mRNA	XR_001024230.1	lncRNA
XM_012176469.2	MACC1	mRNA	XR_001024230.1	lncRNA
XM_012176469.2	MACC1	mRNA	XR_001024230.1	lncRNA
XM_012176469.2	MACC1	mRNA	XR_001024230.1	lncRNA
XM_012106112.2	ARAF	mRNA	XR_001436065.1	lncRNA
XM_012106112.2	ARAF	mRNA	XR_001436065.1	lncRNA
XM_012106112.2	ARAF	mRNA	XR_001436065.1	lncRNA
XM_012106112.2	ARAF	mRNA	XR_001436065.1	lncRNA
XM_012106112.2	ARAF	mRNA	XR_001436065.1	lncRNA
XM_012174722.2	PRKACB	mRNA	XR_001436065.1	lncRNA
XM_012174722.2	PRKACB	mRNA	XR_001436065.1	lncRNA

XM_012174722.2	PRKACB	mRNA	XR_001436065.1	lncRNA
XM_012174722.2	PRKACB	mRNA	XR_001436065.1	lncRNA
XM_012174722.2	PRKACB	mRNA	XR_001436065.1	lncRNA
XM_015097749.1	PCMTD1	mRNA	XR_001436065.1	lncRNA
XM_015097749.1	PCMTD1	mRNA	XR_001436065.1	lncRNA
XM_015097749.1	PCMTD1	mRNA	XR_001436065.1	lncRNA
XM_015097749.1	PCMTD1	mRNA	XR_001436065.1	lncRNA
XM_015097749.1	PCMTD1	mRNA	XR_001436065.1	lncRNA
XM_012174737.2	PRKACB	mRNA	XR_001436065.1	lncRNA
XM_012174737.2	PRKACB	mRNA	XR_001436065.1	lncRNA
XM_012174737.2	PRKACB	mRNA	XR_001436065.1	lncRNA
XM_012174737.2	PRKACB	mRNA	XR_001436065.1	lncRNA
XM_012174737.2	PRKACB	mRNA	XR_001436065.1	lncRNA
XM_012098071.2	RNF185	mRNA	XR_001436065.1	lncRNA
XM_012098071.2	RNF185	mRNA	XR_001436065.1	lncRNA
XM_012098071.2	RNF185	mRNA	XR_001436065.1	lncRNA
XM_012098071.2	RNF185	mRNA	XR_001436065.1	lncRNA
XM_012098071.2	RNF185	mRNA	XR_001436065.1	lncRNA
XM_012174423.2	SLC38A4	mRNA	XR_001436065.1	lncRNA
XM_012174423.2	SLC38A4	mRNA	XR_001436065.1	lncRNA
XM_012174423.2	SLC38A4	mRNA	XR_001436065.1	lncRNA
XM_012174423.2	SLC38A4	mRNA	XR_001436065.1	lncRNA
XM_012174423.2	SLC38A4	mRNA	XR_001436065.1	lncRNA
XM_015099863.1	DSN1	mRNA	XR_001436065.1	lncRNA
XM_015099863.1	DSN1	mRNA	XR_001436065.1	lncRNA
XM_015099863.1	DSN1	mRNA	XR_001436065.1	lncRNA
XM_015099863.1	DSN1	mRNA	XR_001436065.1	lncRNA
XM_015099863.1	DSN1	mRNA	XR_001436065.1	lncRNA
XM_015103688.1	PTPN2	mRNA	XR_001436065.1	lncRNA
XM_015103688.1	PTPN2	mRNA	XR_001436065.1	lncRNA
XM_015103688.1	PTPN2	mRNA	XR_001436065.1	lncRNA
XM_015103688.1	PTPN2	mRNA	XR_001436065.1	lncRNA
XM_015103688.1	PTPN2	mRNA	XR_001436065.1	lncRNA
XM_012186029.1	FBXL20	mRNA	XR_001436065.1	lncRNA
XM_012186029.1	FBXL20	mRNA	XR_001436065.1	lncRNA
XM_012186029.1	FBXL20	mRNA	XR_001436065.1	lncRNA
XM_012186029.1	FBXL20	mRNA	XR_001436065.1	lncRNA
XM_012186029.1	FBXL20	mRNA	XR_001436065.1	lncRNA
XM_015102542.1	ZNF184	mRNA	XR_001436065.1	lncRNA
XM_015102542.1	ZNF184	mRNA	XR_001436065.1	lncRNA
XM_015102542.1	ZNF184	mRNA	XR_001436065.1	lncRNA
XM_015102542.1	ZNF184	mRNA	XR_001436065.1	lncRNA
XM_015102542.1	ZNF184	mRNA	XR_001436065.1	lncRNA
XM_012101006.2	CCHCR1	mRNA	XR_001024230.1	lncRNA
XM_012101006.2	CCHCR1	mRNA	XR_001024230.1	lncRNA
XM_012101006.2	CCHCR1	mRNA	XR_001024230.1	lncRNA
XM_012101006.2	CCHCR1	mRNA	XR_001024230.1	lncRNA
XM_012101006.2	CCHCR1	mRNA	XR_001024230.1	lncRNA
XM_012101050.1	ZNF311	mRNA	XR_001024230.1	lncRNA
XM_012101050.1	ZNF311	mRNA	XR_001024230.1	lncRNA
XM_012101050.1	ZNF311	mRNA	XR_001024230.1	lncRNA
XM_012101050.1	ZNF311	mRNA	XR_001024230.1	lncRNA
XM_012101050.1	ZNF311	mRNA	XR_001024230.1	lncRNA
XM_015092004.1	MROH7	mRNA	XR_001436064.1	lncRNA

XM_015092004.1	MROH7	mRNA	XR_001436064.1	lncRNA
XM_015092004.1	MROH7	mRNA	XR_001436064.1	lncRNA
XM_015092004.1	MROH7	mRNA	XR_001436064.1	lncRNA
XM_015092004.1	MROH7	mRNA	XR_001436064.1	lncRNA
XM_004014289.3	ZNF133	mRNA	XR_001435726.1	lncRNA
XM_004014289.3	ZNF133	mRNA	XR_001435726.1	lncRNA
XM_004014289.3	ZNF133	mRNA	XR_001435726.1	lncRNA
XM_004014289.3	ZNF133	mRNA	XR_001435726.1	lncRNA
XM_004014289.3	ZNF133	mRNA	XR_001435726.1	lncRNA
XM_012175096.2	ERGIC2	mRNA	XR_001435726.1	lncRNA
XM_012175096.2	ERGIC2	mRNA	XR_001435726.1	lncRNA
XM_012175096.2	ERGIC2	mRNA	XR_001435726.1	lncRNA
XM_012175096.2	ERGIC2	mRNA	XR_001435726.1	lncRNA
XM_012175096.2	ERGIC2	mRNA	XR_001435726.1	lncRNA
XM_012174722.2	PRKACB	mRNA	XR_001435726.1	lncRNA
XM_012174722.2	PRKACB	mRNA	XR_001435726.1	lncRNA
XM_012174722.2	PRKACB	mRNA	XR_001435726.1	lncRNA
XM_012174722.2	PRKACB	mRNA	XR_001435726.1	lncRNA
XM_012174722.2	PRKACB	mRNA	XR_001435726.1	lncRNA
XM_012106112.2	ARAF	mRNA	XR_001435726.1	lncRNA
XM_012106112.2	ARAF	mRNA	XR_001435726.1	lncRNA
XM_012106112.2	ARAF	mRNA	XR_001435726.1	lncRNA
XM_012106112.2	ARAF	mRNA	XR_001435726.1	lncRNA
XM_012106112.2	ARAF	mRNA	XR_001435726.1	lncRNA
XM_012101006.2	CCHCR1	mRNA	XR_001435726.1	lncRNA
XM_012101006.2	CCHCR1	mRNA	XR_001435726.1	lncRNA
XM_012101006.2	CCHCR1	mRNA	XR_001435726.1	lncRNA
XM_012101006.2	CCHCR1	mRNA	XR_001435726.1	lncRNA
XM_012101006.2	CCHCR1	mRNA	XR_001435726.1	lncRNA
XM_015103688.1	PTPN2	mRNA	XR_001436064.1	lncRNA
XM_015103688.1	PTPN2	mRNA	XR_001436064.1	lncRNA
XM_015103688.1	PTPN2	mRNA	XR_001436064.1	lncRNA
XM_015103688.1	PTPN2	mRNA	XR_001436064.1	lncRNA
XM_015103688.1	PTPN2	mRNA	XR_001436064.1	lncRNA
XM_012098071.2	RNF185	mRNA	XR_001436064.1	lncRNA
XM_012098071.2	RNF185	mRNA	XR_001436064.1	lncRNA
XM_012098071.2	RNF185	mRNA	XR_001436064.1	lncRNA
XM_012098071.2	RNF185	mRNA	XR_001436064.1	lncRNA
XM_012098071.2	RNF185	mRNA	XR_001436064.1	lncRNA
XM_012179413.2	PDE5A	mRNA	XR_001435723.1	lncRNA
XM_012179413.2	PDE5A	mRNA	XR_001435723.1	lncRNA
XM_012179413.2	PDE5A	mRNA	XR_001435723.1	lncRNA
XM_012179413.2	PDE5A	mRNA	XR_001435723.1	lncRNA
XM_012179413.2	PDE5A	mRNA	XR_001435723.1	lncRNA
XM_012174423.2	SLC38A4	mRNA	XR_001436064.1	lncRNA
XM_012174423.2	SLC38A4	mRNA	XR_001436064.1	lncRNA
XM_012174423.2	SLC38A4	mRNA	XR_001436064.1	lncRNA
XM_012174423.2	SLC38A4	mRNA	XR_001436064.1	lncRNA
XM_012174423.2	SLC38A4	mRNA	XR_001436064.1	lncRNA
XM_012176469.2	MACC1	mRNA	XR_001435723.1	lncRNA
XM_012176469.2	MACC1	mRNA	XR_001435723.1	lncRNA
XM_012176469.2	MACC1	mRNA	XR_001435723.1	lncRNA
XM_012176469.2	MACC1	mRNA	XR_001435723.1	lncRNA
XM_012176469.2	MACC1	mRNA	XR_001435723.1	lncRNA

XM_012097252.2	RHOF	mRNA	XR_001435723.1	lncRNA
XM_012097252.2	RHOF	mRNA	XR_001435723.1	lncRNA
XM_012097252.2	RHOF	mRNA	XR_001435723.1	lncRNA
XM_012097252.2	RHOF	mRNA	XR_001435723.1	lncRNA
XM_012097252.2	RHOF	mRNA	XR_001435723.1	lncRNA
XM_012100080.2	ARIH2	mRNA	XR_001435726.1	lncRNA
XM_012100080.2	ARIH2	mRNA	XR_001435726.1	lncRNA
XM_012100080.2	ARIH2	mRNA	XR_001435726.1	lncRNA
XM_012100080.2	ARIH2	mRNA	XR_001435726.1	lncRNA
XM_012100080.2	ARIH2	mRNA	XR_001435726.1	lncRNA
XM_012143452.2	NRP2	mRNA	XR_001435726.1	lncRNA
XM_012143452.2	NRP2	mRNA	XR_001435726.1	lncRNA
XM_012143452.2	NRP2	mRNA	XR_001435726.1	lncRNA
XM_012143452.2	NRP2	mRNA	XR_001435726.1	lncRNA
XM_012143452.2	NRP2	mRNA	XR_001435726.1	lncRNA
XM_004004866.2	METTL21A	mRNA	XR_001435726.1	lncRNA
XM_004004866.2	METTL21A	mRNA	XR_001435726.1	lncRNA
XM_004004866.2	METTL21A	mRNA	XR_001435726.1	lncRNA
XM_004004866.2	METTL21A	mRNA	XR_001435726.1	lncRNA
XM_004004866.2	METTL21A	mRNA	XR_001435726.1	lncRNA
XM_012097504.1	MFSD8	mRNA	XR_001435726.1	lncRNA
XM_012097504.1	MFSD8	mRNA	XR_001435726.1	lncRNA
XM_012097504.1	MFSD8	mRNA	XR_001435726.1	lncRNA
XM_012097504.1	MFSD8	mRNA	XR_001435726.1	lncRNA
XM_012097504.1	MFSD8	mRNA	XR_001435726.1	lncRNA
XM_015096221.1	PPIP5K2	mRNA	XR_001435726.1	lncRNA
XM_015096221.1	PPIP5K2	mRNA	XR_001435726.1	lncRNA
XM_015096221.1	PPIP5K2	mRNA	XR_001435726.1	lncRNA
XM_015096221.1	PPIP5K2	mRNA	XR_001435726.1	lncRNA
XM_015096221.1	PPIP5K2	mRNA	XR_001435726.1	lncRNA
XM_012186362.1	PITPNC1	mRNA	XR_001025540.2	lncRNA
XM_012186362.1	PITPNC1	mRNA	XR_001025540.2	lncRNA
XM_012186362.1	PITPNC1	mRNA	XR_001025540.2	lncRNA
XM_012186362.1	PITPNC1	mRNA	XR_001025540.2	lncRNA
XM_012186362.1	PITPNC1	mRNA	XR_001025540.2	lncRNA
XM_012124912.1	KIAA2026	mRNA	XR_001436612.1	lncRNA
XM_012124912.1	KIAA2026	mRNA	XR_001436612.1	lncRNA
XM_012124912.1	KIAA2026	mRNA	XR_001436612.1	lncRNA
XM_012124912.1	KIAA2026	mRNA	XR_001436612.1	lncRNA
XM_012124912.1	KIAA2026	mRNA	XR_001436612.1	lncRNA
XM_004015120.2	PHLPP2	mRNA	XR_001436612.1	lncRNA
XM_004015120.2	PHLPP2	mRNA	XR_001436612.1	lncRNA
XM_004015120.2	PHLPP2	mRNA	XR_001436612.1	lncRNA
XM_004015120.2	PHLPP2	mRNA	XR_001436612.1	lncRNA
XM_004015120.2	PHLPP2	mRNA	XR_001436612.1	lncRNA
XM_012097468.2	ELF2	mRNA	XR_001436612.1	lncRNA
XM_012097468.2	ELF2	mRNA	XR_001436612.1	lncRNA
XM_012097468.2	ELF2	mRNA	XR_001436612.1	lncRNA
XM_012097468.2	ELF2	mRNA	XR_001436612.1	lncRNA
XM_012097468.2	ELF2	mRNA	XR_001436612.1	lncRNA
XM_012173383.2	DHX57	mRNA	XR_001436612.1	lncRNA
XM_012173383.2	DHX57	mRNA	XR_001436612.1	lncRNA
XM_012173383.2	DHX57	mRNA	XR_001436612.1	lncRNA
XM_012173383.2	DHX57	mRNA	XR_001436612.1	lncRNA

XM_012173383.2	DHX57	mRNA	XR_001436612.1	lncRNA
XM_012113353.2	SUSD1	mRNA	XR_001436612.1	lncRNA
XM_012113353.2	SUSD1	mRNA	XR_001436612.1	lncRNA
XM_012113353.2	SUSD1	mRNA	XR_001436612.1	lncRNA
XM_012113353.2	SUSD1	mRNA	XR_001436612.1	lncRNA
XM_012113353.2	SUSD1	mRNA	XR_001436612.1	lncRNA
XM_012185338.2	ZNF207	mRNA	XR_001433837.1	lncRNA
XM_012185338.2	ZNF207	mRNA	XR_001433837.1	lncRNA
XM_012185338.2	ZNF207	mRNA	XR_001433837.1	lncRNA
XM_012185338.2	ZNF207	mRNA	XR_001433837.1	lncRNA
XM_012185338.2	ZNF207	mRNA	XR_001433837.1	lncRNA
XM_012173553.2	STRN	mRNA	XR_001433837.1	lncRNA
XM_012173553.2	STRN	mRNA	XR_001433837.1	lncRNA
XM_012173553.2	STRN	mRNA	XR_001433837.1	lncRNA
XM_012173553.2	STRN	mRNA	XR_001433837.1	lncRNA
XM_012173553.2	STRN	mRNA	XR_001433837.1	lncRNA
XM_012180234.2	CNOT6L	mRNA	XR_001433837.1	lncRNA
XM_012180234.2	CNOT6L	mRNA	XR_001433837.1	lncRNA
XM_012180234.2	CNOT6L	mRNA	XR_001433837.1	lncRNA
XM_012180234.2	CNOT6L	mRNA	XR_001433837.1	lncRNA
XM_012180234.2	CNOT6L	mRNA	XR_001433837.1	lncRNA
XM_015095237.1	DMTF1	mRNA	XR_001433837.1	lncRNA
XM_015095237.1	DMTF1	mRNA	XR_001433837.1	lncRNA
XM_015095237.1	DMTF1	mRNA	XR_001433837.1	lncRNA
XM_015095237.1	DMTF1	mRNA	XR_001433837.1	lncRNA
XM_015095237.1	DMTF1	mRNA	XR_001433837.1	lncRNA
XM_004017655.3	PRR14L	mRNA	XR_001433837.1	lncRNA
XM_004017655.3	PRR14L	mRNA	XR_001433837.1	lncRNA
XM_004017655.3	PRR14L	mRNA	XR_001433837.1	lncRNA
XM_004017655.3	PRR14L	mRNA	XR_001433837.1	lncRNA
XM_004017655.3	PRR14L	mRNA	XR_001433837.1	lncRNA
XM_004017814.3	DNAJA4	mRNA	XR_001433837.1	lncRNA
XM_004017814.3	DNAJA4	mRNA	XR_001433837.1	lncRNA
XM_004017814.3	DNAJA4	mRNA	XR_001433837.1	lncRNA
XM_004017814.3	DNAJA4	mRNA	XR_001433837.1	lncRNA
XM_004017814.3	DNAJA4	mRNA	XR_001433837.1	lncRNA
XM_015096221.1	PPIP5K2	mRNA	XR_001436817.1	lncRNA
XM_015096221.1	PPIP5K2	mRNA	XR_001436817.1	lncRNA
XM_015096221.1	PPIP5K2	mRNA	XR_001436817.1	lncRNA
XM_015096221.1	PPIP5K2	mRNA	XR_001436817.1	lncRNA
XM_015096221.1	PPIP5K2	mRNA	XR_001436817.1	lncRNA
XM_015094919.1	WBP11	mRNA	XR_001435722.1	lncRNA
XM_015094919.1	WBP11	mRNA	XR_001435722.1	lncRNA
XM_015094919.1	WBP11	mRNA	XR_001435722.1	lncRNA
XM_015094919.1	WBP11	mRNA	XR_001435722.1	lncRNA
XM_015094919.1	WBP11	mRNA	XR_001435722.1	lncRNA
XM_004012689.3	MYH10	mRNA	XR_001436817.1	lncRNA
XM_004012689.3	MYH10	mRNA	XR_001436817.1	lncRNA
XM_004012689.3	MYH10	mRNA	XR_001436817.1	lncRNA
XM_004012689.3	MYH10	mRNA	XR_001436817.1	lncRNA
XM_004012689.3	MYH10	mRNA	XR_001436817.1	lncRNA
XM_004014289.3	ZNF133	mRNA	XR_001436817.1	lncRNA
XM_004014289.3	ZNF133	mRNA	XR_001436817.1	lncRNA
XM_004014289.3	ZNF133	mRNA	XR_001436817.1	lncRNA

XM_004014289.3	ZNF133	mRNA	XR_001436817.1	lncRNA
XM_004014289.3	ZNF133	mRNA	XR_001436817.1	lncRNA
XM_012101006.2	CCHCR1	mRNA	XR_001435722.1	lncRNA
XM_012101006.2	CCHCR1	mRNA	XR_001435722.1	lncRNA
XM_012101006.2	CCHCR1	mRNA	XR_001435722.1	lncRNA
XM_012101006.2	CCHCR1	mRNA	XR_001435722.1	lncRNA
XM_012101006.2	CCHCR1	mRNA	XR_001435722.1	lncRNA
XM_012102930.1	GPAM	mRNA	XR_001435722.1	lncRNA
XM_012102930.1	GPAM	mRNA	XR_001435722.1	lncRNA
XM_012102930.1	GPAM	mRNA	XR_001435722.1	lncRNA
XM_012102930.1	GPAM	mRNA	XR_001435722.1	lncRNA
XM_012102930.1	GPAM	mRNA	XR_001435722.1	lncRNA
XM_015103688.1	PTPN2	mRNA	XR_001435722.1	lncRNA
XM_015103688.1	PTPN2	mRNA	XR_001435722.1	lncRNA
XM_015103688.1	PTPN2	mRNA	XR_001435722.1	lncRNA
XM_015103688.1	PTPN2	mRNA	XR_001435722.1	lncRNA
XM_015103688.1	PTPN2	mRNA	XR_001435722.1	lncRNA
XM_012101006.2	CCHCR1	mRNA	XR_001436817.1	lncRNA
XM_012101006.2	CCHCR1	mRNA	XR_001436817.1	lncRNA
XM_012101006.2	CCHCR1	mRNA	XR_001436817.1	lncRNA
XM_012101006.2	CCHCR1	mRNA	XR_001436817.1	lncRNA
XM_012101006.2	CCHCR1	mRNA	XR_001436817.1	lncRNA
XM_015096221.1	PPIP5K2	mRNA	XR_001435722.1	lncRNA
XM_015096221.1	PPIP5K2	mRNA	XR_001435722.1	lncRNA
XM_015096221.1	PPIP5K2	mRNA	XR_001435722.1	lncRNA
XM_015096221.1	PPIP5K2	mRNA	XR_001435722.1	lncRNA
XM_015096221.1	PPIP5K2	mRNA	XR_001435722.1	lncRNA
XM_012100080.2	ARIH2	mRNA	XR_001436817.1	lncRNA
XM_012100080.2	ARIH2	mRNA	XR_001436817.1	lncRNA
XM_012100080.2	ARIH2	mRNA	XR_001436817.1	lncRNA
XM_012100080.2	ARIH2	mRNA	XR_001436817.1	lncRNA
XM_012100080.2	ARIH2	mRNA	XR_001436817.1	lncRNA
XM_015092197.1	CD58	mRNA	XR_001436817.1	lncRNA
XM_015092197.1	CD58	mRNA	XR_001436817.1	lncRNA
XM_015092197.1	CD58	mRNA	XR_001436817.1	lncRNA
XM_015092197.1	CD58	mRNA	XR_001436817.1	lncRNA
XM_015092197.1	CD58	mRNA	XR_001436817.1	lncRNA
XM_012175096.2	ERGIC2	mRNA	XR_001436817.1	lncRNA
XM_012175096.2	ERGIC2	mRNA	XR_001436817.1	lncRNA
XM_012175096.2	ERGIC2	mRNA	XR_001436817.1	lncRNA
XM_012175096.2	ERGIC2	mRNA	XR_001436817.1	lncRNA
XM_012175096.2	ERGIC2	mRNA	XR_001436817.1	lncRNA
XM_015101559.1	DDX51	mRNA	XR_001435722.1	lncRNA
XM_015101559.1	DDX51	mRNA	XR_001435722.1	lncRNA
XM_015101559.1	DDX51	mRNA	XR_001435722.1	lncRNA
XM_015101559.1	DDX51	mRNA	XR_001435722.1	lncRNA
XM_015101559.1	DDX51	mRNA	XR_001435722.1	lncRNA
XM_012097504.1	MFSD8	mRNA	XR_001436817.1	lncRNA
XM_012097504.1	MFSD8	mRNA	XR_001436817.1	lncRNA
XM_012097504.1	MFSD8	mRNA	XR_001436817.1	lncRNA
XM_012097504.1	MFSD8	mRNA	XR_001436817.1	lncRNA
XM_012097504.1	MFSD8	mRNA	XR_001436817.1	lncRNA
XM_012143452.2	NRP2	mRNA	XR_001436817.1	lncRNA
XM_012143452.2	NRP2	mRNA	XR_001436817.1	lncRNA

XM_012143452.2	NRP2	mRNA	XR_001436817.1	lncRNA
XM_012143452.2	NRP2	mRNA	XR_001436817.1	lncRNA
XM_012143452.2	NRP2	mRNA	XR_001436817.1	lncRNA
XM_012184822.2	RBM26	mRNA	XR_001436817.1	lncRNA
XM_012184822.2	RBM26	mRNA	XR_001436817.1	lncRNA
XM_012184822.2	RBM26	mRNA	XR_001436817.1	lncRNA
XM_012184822.2	RBM26	mRNA	XR_001436817.1	lncRNA
XM_012184822.2	RBM26	mRNA	XR_001436817.1	lncRNA
XM_015097819.1	STAU2	mRNA	XR_001436817.1	lncRNA
XM_015097819.1	STAU2	mRNA	XR_001436817.1	lncRNA
XM_015097819.1	STAU2	mRNA	XR_001436817.1	lncRNA
XM_015097819.1	STAU2	mRNA	XR_001436817.1	lncRNA
XM_015097819.1	STAU2	mRNA	XR_001436817.1	lncRNA
XM_012181270.1	VPS39	mRNA	XR_001436817.1	lncRNA
XM_012181270.1	VPS39	mRNA	XR_001436817.1	lncRNA
XM_012181270.1	VPS39	mRNA	XR_001436817.1	lncRNA
XM_012181270.1	VPS39	mRNA	XR_001436817.1	lncRNA
XM_012181270.1	VPS39	mRNA	XR_001436817.1	lncRNA
XM_004012689.3	MYH10	mRNA	XR_001435722.1	lncRNA
XM_004012689.3	MYH10	mRNA	XR_001435722.1	lncRNA
XM_004012689.3	MYH10	mRNA	XR_001435722.1	lncRNA
XM_004012689.3	MYH10	mRNA	XR_001435722.1	lncRNA
XM_004012689.3	MYH10	mRNA	XR_001435722.1	lncRNA
XM_015098186.1	XPO4	mRNA	XR_001435722.1	lncRNA
XM_015098186.1	XPO4	mRNA	XR_001435722.1	lncRNA
XM_015098186.1	XPO4	mRNA	XR_001435722.1	lncRNA
XM_015098186.1	XPO4	mRNA	XR_001435722.1	lncRNA
XM_015098186.1	XPO4	mRNA	XR_001435722.1	lncRNA
XM_015098186.1	XPO4	mRNA	XR_001436817.1	lncRNA
XM_015098186.1	XPO4	mRNA	XR_001436817.1	lncRNA
XM_015098186.1	XPO4	mRNA	XR_001436817.1	lncRNA
XM_015098186.1	XPO4	mRNA	XR_001436817.1	lncRNA
XM_015098186.1	XPO4	mRNA	XR_001436817.1	lncRNA
XM_012102930.1	GPAM	mRNA	XR_001436817.1	lncRNA
XM_012102930.1	GPAM	mRNA	XR_001436817.1	lncRNA
XM_012102930.1	GPAM	mRNA	XR_001436817.1	lncRNA
XM_012102930.1	GPAM	mRNA	XR_001436817.1	lncRNA
XM_012102930.1	GPAM	mRNA	XR_001436817.1	lncRNA
XM_012097252.2	RHOF	mRNA	XR_001435722.1	lncRNA
XM_012097252.2	RHOF	mRNA	XR_001435722.1	lncRNA
XM_012097252.2	RHOF	mRNA	XR_001435722.1	lncRNA
XM_012097252.2	RHOF	mRNA	XR_001435722.1	lncRNA
XM_012097252.2	RHOF	mRNA	XR_001435722.1	lncRNA
XM_012181270.1	VPS39	mRNA	XR_001435722.1	lncRNA
XM_012181270.1	VPS39	mRNA	XR_001435722.1	lncRNA
XM_012181270.1	VPS39	mRNA	XR_001435722.1	lncRNA
XM_012181270.1	VPS39	mRNA	XR_001435722.1	lncRNA
XM_012181270.1	VPS39	mRNA	XR_001435722.1	lncRNA
XM_012179413.2	PDE5A	mRNA	XR_001435722.1	lncRNA
XM_012179413.2	PDE5A	mRNA	XR_001435722.1	lncRNA
XM_012179413.2	PDE5A	mRNA	XR_001435722.1	lncRNA
XM_012179413.2	PDE5A	mRNA	XR_001435722.1	lncRNA
XM_012179413.2	PDE5A	mRNA	XR_001435722.1	lncRNA
XM_015102111.1	EIF4E3	mRNA	XR_001436817.1	lncRNA

XM_015102111.1	EIF4E3	mRNA	XR_001436817.1	lncRNA
XM_015102111.1	EIF4E3	mRNA	XR_001436817.1	lncRNA
XM_015102111.1	EIF4E3	mRNA	XR_001436817.1	lncRNA
XM_015102111.1	EIF4E3	mRNA	XR_001436817.1	lncRNA
XM_004014289.3	ZNF133	mRNA	XR_001435722.1	lncRNA
XM_004014289.3	ZNF133	mRNA	XR_001435722.1	lncRNA
XM_004014289.3	ZNF133	mRNA	XR_001435722.1	lncRNA
XM_004014289.3	ZNF133	mRNA	XR_001435722.1	lncRNA
XM_004014289.3	ZNF133	mRNA	XR_001435722.1	lncRNA
XM_012176469.2	MACC1	mRNA	XR_001436817.1	lncRNA
XM_012176469.2	MACC1	mRNA	XR_001436817.1	lncRNA
XM_012176469.2	MACC1	mRNA	XR_001436817.1	lncRNA
XM_012176469.2	MACC1	mRNA	XR_001436817.1	lncRNA
XM_012176469.2	MACC1	mRNA	XR_001436817.1	lncRNA
XM_015099653.1	LOC101121441	mRNA	XR_001435722.1	lncRNA
XM_015099653.1	LOC101121441	mRNA	XR_001435722.1	lncRNA
XM_015099653.1	LOC101121441	mRNA	XR_001435722.1	lncRNA
XM_015099653.1	LOC101121441	mRNA	XR_001435722.1	lncRNA
XM_015099653.1	LOC101121441	mRNA	XR_001435722.1	lncRNA
XM_012159299.2	LOC101121563	mRNA	XR_001435722.1	lncRNA
XM_012159299.2	LOC101121563	mRNA	XR_001435722.1	lncRNA
XM_012159299.2	LOC101121563	mRNA	XR_001435722.1	lncRNA
XM_012159299.2	LOC101121563	mRNA	XR_001435722.1	lncRNA
XM_012159299.2	LOC101121563	mRNA	XR_001435722.1	lncRNA
XM_004014289.3	ZNF133	mRNA	XR_001434920.1	lncRNA
XM_004014289.3	ZNF133	mRNA	XR_001434920.1	lncRNA
XM_004014289.3	ZNF133	mRNA	XR_001434920.1	lncRNA
XM_004014289.3	ZNF133	mRNA	XR_001434920.1	lncRNA
XM_004014289.3	ZNF133	mRNA	XR_001434920.1	lncRNA
XM_004004866.2	METTL21A	mRNA	XR_001433856.1	lncRNA
XM_004004866.2	METTL21A	mRNA	XR_001433856.1	lncRNA
XM_004004866.2	METTL21A	mRNA	XR_001433856.1	lncRNA
XM_004004866.2	METTL21A	mRNA	XR_001433856.1	lncRNA
XM_004004866.2	METTL21A	mRNA	XR_001433856.1	lncRNA
XM_012189150.1	NDRG3	mRNA	XR_001433856.1	lncRNA
XM_012189150.1	NDRG3	mRNA	XR_001433856.1	lncRNA
XM_012189150.1	NDRG3	mRNA	XR_001433856.1	lncRNA
XM_012189150.1	NDRG3	mRNA	XR_001433856.1	lncRNA
XM_012189150.1	NDRG3	mRNA	XR_001433856.1	lncRNA
XM_012142751.2	ZFYVE9	mRNA	XR_001435107.1	lncRNA
XM_012142751.2	ZFYVE9	mRNA	XR_001435107.1	lncRNA
XM_012142751.2	ZFYVE9	mRNA	XR_001435107.1	lncRNA
XM_012142751.2	ZFYVE9	mRNA	XR_001435107.1	lncRNA
XM_012142751.2	ZFYVE9	mRNA	XR_001435107.1	lncRNA
XM_015099653.1	LOC101121441	mRNA	XR_001434920.1	lncRNA
XM_015099653.1	LOC101121441	mRNA	XR_001434920.1	lncRNA
XM_015099653.1	LOC101121441	mRNA	XR_001434920.1	lncRNA
XM_015099653.1	LOC101121441	mRNA	XR_001434920.1	lncRNA
XM_015099653.1	LOC101121441	mRNA	XR_001434920.1	lncRNA
XM_012174737.2	PRKACB	mRNA	XR_001433856.1	lncRNA
XM_012174737.2	PRKACB	mRNA	XR_001433856.1	lncRNA
XM_012174737.2	PRKACB	mRNA	XR_001433856.1	lncRNA
XM_012174737.2	PRKACB	mRNA	XR_001433856.1	lncRNA
XM_012174737.2	PRKACB	mRNA	XR_001433856.1	lncRNA

XM_015094919.1	WBP11	mRNA	XR_001433856.1	lncRNA
XM_015094919.1	WBP11	mRNA	XR_001433856.1	lncRNA
XM_015094919.1	WBP11	mRNA	XR_001433856.1	lncRNA
XM_015094919.1	WBP11	mRNA	XR_001433856.1	lncRNA
XM_015094919.1	WBP11	mRNA	XR_001433856.1	lncRNA
XM_012102930.1	GPAM	mRNA	XR_001433856.1	lncRNA
XM_012102930.1	GPAM	mRNA	XR_001433856.1	lncRNA
XM_012102930.1	GPAM	mRNA	XR_001433856.1	lncRNA
XM_012102930.1	GPAM	mRNA	XR_001433856.1	lncRNA
XM_012102930.1	GPAM	mRNA	XR_001433856.1	lncRNA
XM_012184822.2	RBM26	mRNA	XR_001433856.1	lncRNA
XM_012184822.2	RBM26	mRNA	XR_001433856.1	lncRNA
XM_012184822.2	RBM26	mRNA	XR_001433856.1	lncRNA
XM_012184822.2	RBM26	mRNA	XR_001433856.1	lncRNA
XM_012184822.2	RBM26	mRNA	XR_001433856.1	lncRNA
XM_012143452.2	NRP2	mRNA	XR_001435107.1	lncRNA
XM_012143452.2	NRP2	mRNA	XR_001435107.1	lncRNA
XM_012143452.2	NRP2	mRNA	XR_001435107.1	lncRNA
XM_012143452.2	NRP2	mRNA	XR_001435107.1	lncRNA
XM_012143452.2	NRP2	mRNA	XR_001435107.1	lncRNA
XM_012177136.1	TMEM209	mRNA	XR_001435107.1	lncRNA
XM_012177136.1	TMEM209	mRNA	XR_001435107.1	lncRNA
XM_012177136.1	TMEM209	mRNA	XR_001435107.1	lncRNA
XM_012177136.1	TMEM209	mRNA	XR_001435107.1	lncRNA
XM_012177136.1	TMEM209	mRNA	XR_001435107.1	lncRNA
XM_012184822.2	RBM26	mRNA	XR_001434920.1	lncRNA
XM_012184822.2	RBM26	mRNA	XR_001434920.1	lncRNA
XM_012184822.2	RBM26	mRNA	XR_001434920.1	lncRNA
XM_012184822.2	RBM26	mRNA	XR_001434920.1	lncRNA
XM_012184822.2	RBM26	mRNA	XR_001434920.1	lncRNA
XM_012159299.2	LOC101121563	mRNA	XR_001434920.1	lncRNA
XM_012159299.2	LOC101121563	mRNA	XR_001434920.1	lncRNA
XM_012159299.2	LOC101121563	mRNA	XR_001434920.1	lncRNA
XM_012159299.2	LOC101121563	mRNA	XR_001434920.1	lncRNA
XM_012159299.2	LOC101121563	mRNA	XR_001434920.1	lncRNA
XM_012124921.1	KIAA2026	mRNA	XR_001433856.1	lncRNA
XM_012124921.1	KIAA2026	mRNA	XR_001433856.1	lncRNA
XM_012124921.1	KIAA2026	mRNA	XR_001433856.1	lncRNA
XM_012124921.1	KIAA2026	mRNA	XR_001433856.1	lncRNA
XM_012124921.1	KIAA2026	mRNA	XR_001433856.1	lncRNA
XM_015096221.1	PPIP5K2	mRNA	XR_001433856.1	lncRNA
XM_015096221.1	PPIP5K2	mRNA	XR_001433856.1	lncRNA
XM_015096221.1	PPIP5K2	mRNA	XR_001433856.1	lncRNA
XM_015096221.1	PPIP5K2	mRNA	XR_001433856.1	lncRNA
XM_015096221.1	PPIP5K2	mRNA	XR_001433856.1	lncRNA
XM_015096456.1	APBB2	mRNA	XR_001433856.1	lncRNA
XM_015096456.1	APBB2	mRNA	XR_001433856.1	lncRNA
XM_015096456.1	APBB2	mRNA	XR_001433856.1	lncRNA
XM_015096456.1	APBB2	mRNA	XR_001433856.1	lncRNA
XM_015096456.1	APBB2	mRNA	XR_001433856.1	lncRNA
XM_015096221.1	PPIP5K2	mRNA	XR_001435107.1	lncRNA
XM_015096221.1	PPIP5K2	mRNA	XR_001435107.1	lncRNA
XM_015096221.1	PPIP5K2	mRNA	XR_001435107.1	lncRNA
XM_015096221.1	PPIP5K2	mRNA	XR_001435107.1	lncRNA

XM_015096221.1	PPIP5K2	mRNA	XR_001435107.1	lncRNA
XM_012187772.1	ANGEL2	mRNA	XR_001433856.1	lncRNA
XM_012187772.1	ANGEL2	mRNA	XR_001433856.1	lncRNA
XM_012187772.1	ANGEL2	mRNA	XR_001433856.1	lncRNA
XM_012187772.1	ANGEL2	mRNA	XR_001433856.1	lncRNA
XM_012187772.1	ANGEL2	mRNA	XR_001433856.1	lncRNA
XM_004008822.3	FAM13B	mRNA	XR_001435107.1	lncRNA
XM_004008822.3	FAM13B	mRNA	XR_001435107.1	lncRNA
XM_004008822.3	FAM13B	mRNA	XR_001435107.1	lncRNA
XM_004008822.3	FAM13B	mRNA	XR_001435107.1	lncRNA
XM_004008822.3	FAM13B	mRNA	XR_001435107.1	lncRNA
XM_004012689.3	MYH10	mRNA	XR_001434920.1	lncRNA
XM_004012689.3	MYH10	mRNA	XR_001434920.1	lncRNA
XM_004012689.3	MYH10	mRNA	XR_001434920.1	lncRNA
XM_004012689.3	MYH10	mRNA	XR_001434920.1	lncRNA
XM_004012689.3	MYH10	mRNA	XR_001434920.1	lncRNA
XM_004008381.3	MAPK9	mRNA	XR_001435107.1	lncRNA
XM_004008381.3	MAPK9	mRNA	XR_001435107.1	lncRNA
XM_004008381.3	MAPK9	mRNA	XR_001435107.1	lncRNA
XM_004008381.3	MAPK9	mRNA	XR_001435107.1	lncRNA
XM_004008381.3	MAPK9	mRNA	XR_001435107.1	lncRNA
XM_012095502.1	SIK3	mRNA	XR_001435107.1	lncRNA
XM_012095502.1	SIK3	mRNA	XR_001435107.1	lncRNA
XM_012095502.1	SIK3	mRNA	XR_001435107.1	lncRNA
XM_012095502.1	SIK3	mRNA	XR_001435107.1	lncRNA
XM_012095502.1	SIK3	mRNA	XR_001435107.1	lncRNA
XM_012174722.2	PRKACB	mRNA	XR_001433856.1	lncRNA
XM_012174722.2	PRKACB	mRNA	XR_001433856.1	lncRNA
XM_012174722.2	PRKACB	mRNA	XR_001433856.1	lncRNA
XM_012174722.2	PRKACB	mRNA	XR_001433856.1	lncRNA
XM_012174722.2	PRKACB	mRNA	XR_001433856.1	lncRNA
XM_012095761.2	HSPBAP1	mRNA	XR_001435107.1	lncRNA
XM_012095761.2	HSPBAP1	mRNA	XR_001435107.1	lncRNA
XM_012095761.2	HSPBAP1	mRNA	XR_001435107.1	lncRNA
XM_012095761.2	HSPBAP1	mRNA	XR_001435107.1	lncRNA
XM_012095761.2	HSPBAP1	mRNA	XR_001435107.1	lncRNA
XM_012181270.1	VPS39	mRNA	XR_001435107.1	lncRNA
XM_012181270.1	VPS39	mRNA	XR_001435107.1	lncRNA
XM_012181270.1	VPS39	mRNA	XR_001435107.1	lncRNA
XM_012181270.1	VPS39	mRNA	XR_001435107.1	lncRNA
XM_012181270.1	VPS39	mRNA	XR_001435107.1	lncRNA
XM_012105610.1	ZFP69	mRNA	XR_001435107.1	lncRNA
XM_012105610.1	ZFP69	mRNA	XR_001435107.1	lncRNA
XM_012105610.1	ZFP69	mRNA	XR_001435107.1	lncRNA
XM_012105610.1	ZFP69	mRNA	XR_001435107.1	lncRNA
XM_012105610.1	ZFP69	mRNA	XR_001435107.1	lncRNA
XM_012176469.2	MACC1	mRNA	XR_001435107.1	lncRNA
XM_012176469.2	MACC1	mRNA	XR_001435107.1	lncRNA
XM_012176469.2	MACC1	mRNA	XR_001435107.1	lncRNA
XM_012176469.2	MACC1	mRNA	XR_001435107.1	lncRNA
XM_012176469.2	MACC1	mRNA	XR_001435107.1	lncRNA
XM_015099653.1	LOC101121441	mRNA	XR_001435107.1	lncRNA
XM_015099653.1	LOC101121441	mRNA	XR_001435107.1	lncRNA
XM_015099653.1	LOC101121441	mRNA	XR_001435107.1	lncRNA

XM_015099653.1	LOC101121441	mRNA	XR_001435107.1	lncRNA
XM_015099653.1	LOC101121441	mRNA	XR_001435107.1	lncRNA
XM_004014289.3	ZNF133	mRNA	XR_001435107.1	lncRNA
XM_004014289.3	ZNF133	mRNA	XR_001435107.1	lncRNA
XM_004014289.3	ZNF133	mRNA	XR_001435107.1	lncRNA
XM_004014289.3	ZNF133	mRNA	XR_001435107.1	lncRNA
XM_004014289.3	ZNF133	mRNA	XR_001435107.1	lncRNA
XM_015094471.1	CCDC142	mRNA	XR_001435107.1	lncRNA
XM_015094471.1	CCDC142	mRNA	XR_001435107.1	lncRNA
XM_015094471.1	CCDC142	mRNA	XR_001435107.1	lncRNA
XM_015094471.1	CCDC142	mRNA	XR_001435107.1	lncRNA
XM_015094471.1	CCDC142	mRNA	XR_001435107.1	lncRNA
XM_012174679.2	R3HDM2	mRNA	XR_001433856.1	lncRNA
XM_012174679.2	R3HDM2	mRNA	XR_001433856.1	lncRNA
XM_012174679.2	R3HDM2	mRNA	XR_001433856.1	lncRNA
XM_012174679.2	R3HDM2	mRNA	XR_001433856.1	lncRNA
XM_012174679.2	R3HDM2	mRNA	XR_001433856.1	lncRNA
XM_004012689.3	MYH10	mRNA	XR_001435107.1	lncRNA
XM_004012689.3	MYH10	mRNA	XR_001435107.1	lncRNA
XM_004012689.3	MYH10	mRNA	XR_001435107.1	lncRNA
XM_004012689.3	MYH10	mRNA	XR_001435107.1	lncRNA
XM_004012689.3	MYH10	mRNA	XR_001435107.1	lncRNA
XM_012175096.2	ERGIC2	mRNA	XR_001435107.1	lncRNA
XM_012175096.2	ERGIC2	mRNA	XR_001435107.1	lncRNA
XM_012175096.2	ERGIC2	mRNA	XR_001435107.1	lncRNA
XM_012175096.2	ERGIC2	mRNA	XR_001435107.1	lncRNA
XM_012175096.2	ERGIC2	mRNA	XR_001435107.1	lncRNA
XM_004014289.3	ZNF133	mRNA	XR_001433856.1	lncRNA
XM_004014289.3	ZNF133	mRNA	XR_001433856.1	lncRNA
XM_004014289.3	ZNF133	mRNA	XR_001433856.1	lncRNA
XM_004014289.3	ZNF133	mRNA	XR_001433856.1	lncRNA
XM_004014289.3	ZNF133	mRNA	XR_001433856.1	lncRNA
XM_012097252.2	RHOF	mRNA	XR_001435107.1	lncRNA
XM_012097252.2	RHOF	mRNA	XR_001435107.1	lncRNA
XM_012097252.2	RHOF	mRNA	XR_001435107.1	lncRNA
XM_012097252.2	RHOF	mRNA	XR_001435107.1	lncRNA
XM_012097252.2	RHOF	mRNA	XR_001435107.1	lncRNA
XM_015098186.1	XPO4	mRNA	XR_001435107.1	lncRNA
XM_015098186.1	XPO4	mRNA	XR_001435107.1	lncRNA
XM_015098186.1	XPO4	mRNA	XR_001435107.1	lncRNA
XM_015098186.1	XPO4	mRNA	XR_001435107.1	lncRNA
XM_015098186.1	XPO4	mRNA	XR_001435107.1	lncRNA
XM_012097252.2	RHOF	mRNA	XR_001433856.1	lncRNA
XM_012097252.2	RHOF	mRNA	XR_001433856.1	lncRNA
XM_012097252.2	RHOF	mRNA	XR_001433856.1	lncRNA
XM_012097252.2	RHOF	mRNA	XR_001433856.1	lncRNA
XM_012097252.2	RHOF	mRNA	XR_001433856.1	lncRNA
XM_015092197.1	CD58	mRNA	XR_001433856.1	lncRNA
XM_015092197.1	CD58	mRNA	XR_001433856.1	lncRNA
XM_015092197.1	CD58	mRNA	XR_001433856.1	lncRNA
XM_015092197.1	CD58	mRNA	XR_001433856.1	lncRNA
XM_015092197.1	CD58	mRNA	XR_001433856.1	lncRNA
XM_015101559.1	DDX51	mRNA	XR_001435107.1	lncRNA
XM_015101559.1	DDX51	mRNA	XR_001435107.1	lncRNA

XM_015101559.1	DDX51	mRNA	XR_001435107.1	lncRNA
XM_015101559.1	DDX51	mRNA	XR_001435107.1	lncRNA
XM_015101559.1	DDX51	mRNA	XR_001435107.1	lncRNA
XM_012189207.2	SENP7	mRNA	XR_001435107.1	lncRNA
XM_012189207.2	SENP7	mRNA	XR_001435107.1	lncRNA
XM_012189207.2	SENP7	mRNA	XR_001435107.1	lncRNA
XM_012189207.2	SENP7	mRNA	XR_001435107.1	lncRNA
XM_012189207.2	SENP7	mRNA	XR_001435107.1	lncRNA
XM_012176656.2	NAPEPLD	mRNA	XR_001433856.1	lncRNA
XM_012176656.2	NAPEPLD	mRNA	XR_001433856.1	lncRNA
XM_012176656.2	NAPEPLD	mRNA	XR_001433856.1	lncRNA
XM_012176656.2	NAPEPLD	mRNA	XR_001433856.1	lncRNA
XM_012176656.2	NAPEPLD	mRNA	XR_001433856.1	lncRNA
XM_012102930.1	GPAM	mRNA	XR_001435107.1	lncRNA
XM_012102930.1	GPAM	mRNA	XR_001435107.1	lncRNA
XM_012102930.1	GPAM	mRNA	XR_001435107.1	lncRNA
XM_012102930.1	GPAM	mRNA	XR_001435107.1	lncRNA
XM_012102930.1	GPAM	mRNA	XR_001435107.1	lncRNA
XM_012179413.2	PDE5A	mRNA	XR_001433856.1	lncRNA
XM_012179413.2	PDE5A	mRNA	XR_001433856.1	lncRNA
XM_012179413.2	PDE5A	mRNA	XR_001433856.1	lncRNA
XM_012179413.2	PDE5A	mRNA	XR_001433856.1	lncRNA
XM_012179413.2	PDE5A	mRNA	XR_001433856.1	lncRNA
XM_015098186.1	XPO4	mRNA	XR_001433856.1	lncRNA
XM_015098186.1	XPO4	mRNA	XR_001433856.1	lncRNA
XM_015098186.1	XPO4	mRNA	XR_001433856.1	lncRNA
XM_015098186.1	XPO4	mRNA	XR_001433856.1	lncRNA
XM_015098186.1	XPO4	mRNA	XR_001433856.1	lncRNA
XM_015099563.1	PARD3	mRNA	XR_001023303.2	lncRNA
XM_015099563.1	PARD3	mRNA	XR_001023303.2	lncRNA
XM_015099563.1	PARD3	mRNA	XR_001023303.2	lncRNA
XM_015099563.1	PARD3	mRNA	XR_001023303.2	lncRNA
XM_015099563.1	PARD3	mRNA	XR_001023303.2	lncRNA
XM_012095733.1	BTBD10	mRNA	XR_001023303.2	lncRNA
XM_012095733.1	BTBD10	mRNA	XR_001023303.2	lncRNA
XM_012095733.1	BTBD10	mRNA	XR_001023303.2	lncRNA
XM_012095733.1	BTBD10	mRNA	XR_001023303.2	lncRNA
XM_012095733.1	BTBD10	mRNA	XR_001023303.2	lncRNA
XM_012188348.1	KIAA1217	mRNA	XR_001023303.2	lncRNA
XM_012188348.1	KIAA1217	mRNA	XR_001023303.2	lncRNA
XM_012188348.1	KIAA1217	mRNA	XR_001023303.2	lncRNA
XM_012188348.1	KIAA1217	mRNA	XR_001023303.2	lncRNA
XM_012188348.1	KIAA1217	mRNA	XR_001023303.2	lncRNA
XM_004010552.3	VPS13C	mRNA	XR_001024966.2	lncRNA
XM_004010552.3	VPS13C	mRNA	XR_001024966.2	lncRNA
XM_004010552.3	VPS13C	mRNA	XR_001024966.2	lncRNA
XM_004010552.3	VPS13C	mRNA	XR_001024966.2	lncRNA
XM_004010552.3	VPS13C	mRNA	XR_001024966.2	lncRNA
XM_012182192.2	EML5	mRNA	XR_001024966.2	lncRNA
XM_012182192.2	EML5	mRNA	XR_001024966.2	lncRNA
XM_012182192.2	EML5	mRNA	XR_001024966.2	lncRNA
XM_012182192.2	EML5	mRNA	XR_001024966.2	lncRNA
XM_012182192.2	EML5	mRNA	XR_001024966.2	lncRNA
XM_012190083.2	ZMYM6	mRNA	XR_001436390.1	lncRNA

XM_012190083.2	ZMYM6	mRNA	XR_001436390.1	lncRNA
XM_012190083.2	ZMYM6	mRNA	XR_001436390.1	lncRNA
XM_012190083.2	ZMYM6	mRNA	XR_001436390.1	lncRNA
XM_012190083.2	ZMYM6	mRNA	XR_001436390.1	lncRNA
XM_012181728.2	KIAA0586	mRNA	XR_001436390.1	lncRNA
XM_012181728.2	KIAA0586	mRNA	XR_001436390.1	lncRNA
XM_012181728.2	KIAA0586	mRNA	XR_001436390.1	lncRNA
XM_012181728.2	KIAA0586	mRNA	XR_001436390.1	lncRNA
XM_012181728.2	KIAA0586	mRNA	XR_001436390.1	lncRNA
XM_012189779.2	RBL2	mRNA	XR_001436390.1	lncRNA
XM_012189779.2	RBL2	mRNA	XR_001436390.1	lncRNA
XM_012189779.2	RBL2	mRNA	XR_001436390.1	lncRNA
XM_012189779.2	RBL2	mRNA	XR_001436390.1	lncRNA
XM_012189779.2	RBL2	mRNA	XR_001436390.1	lncRNA
XM_012159182.2	LOC101121820	mRNA	XR_001436390.1	lncRNA
XM_012159182.2	LOC101121820	mRNA	XR_001436390.1	lncRNA
XM_012159182.2	LOC101121820	mRNA	XR_001436390.1	lncRNA
XM_012159182.2	LOC101121820	mRNA	XR_001436390.1	lncRNA
XM_012159182.2	LOC101121820	mRNA	XR_001436390.1	lncRNA
XM_012098709.2	PML	mRNA	XR_001436390.1	lncRNA
XM_012098709.2	PML	mRNA	XR_001436390.1	lncRNA
XM_012098709.2	PML	mRNA	XR_001436390.1	lncRNA
XM_012098709.2	PML	mRNA	XR_001436390.1	lncRNA
XM_012098709.2	PML	mRNA	XR_001436390.1	lncRNA
XM_004012073.3	FNDC3A	mRNA	XR_001436390.1	lncRNA
XM_004012073.3	FNDC3A	mRNA	XR_001436390.1	lncRNA
XM_004012073.3	FNDC3A	mRNA	XR_001436390.1	lncRNA
XM_004012073.3	FNDC3A	mRNA	XR_001436390.1	lncRNA
XM_004012073.3	FNDC3A	mRNA	XR_001436390.1	lncRNA
XM_015100095.1	LOC101113523	mRNA	XR_001436390.1	lncRNA
XM_015100095.1	LOC101113523	mRNA	XR_001436390.1	lncRNA
XM_015100095.1	LOC101113523	mRNA	XR_001436390.1	lncRNA
XM_015100095.1	LOC101113523	mRNA	XR_001436390.1	lncRNA
XM_015100095.1	LOC101113523	mRNA	XR_001436390.1	lncRNA
XM_015096518.1	AASDH	mRNA	XR_001436390.1	lncRNA
XM_015096518.1	AASDH	mRNA	XR_001436390.1	lncRNA
XM_015096518.1	AASDH	mRNA	XR_001436390.1	lncRNA
XM_015096518.1	AASDH	mRNA	XR_001436390.1	lncRNA
XM_015096518.1	AASDH	mRNA	XR_001436390.1	lncRNA
XM_012176414.2	PHF14	mRNA	XR_001436390.1	lncRNA
XM_012176414.2	PHF14	mRNA	XR_001436390.1	lncRNA
XM_012176414.2	PHF14	mRNA	XR_001436390.1	lncRNA
XM_012176414.2	PHF14	mRNA	XR_001436390.1	lncRNA
XM_012176414.2	PHF14	mRNA	XR_001436390.1	lncRNA
XM_015096518.1	AASDH	mRNA	XR_001040147.1	lncRNA
XM_015096518.1	AASDH	mRNA	XR_001040147.1	lncRNA
XM_015096518.1	AASDH	mRNA	XR_001040147.1	lncRNA
XM_015096518.1	AASDH	mRNA	XR_001040147.1	lncRNA
XM_015096518.1	AASDH	mRNA	XR_001040147.1	lncRNA
XM_012096602.2	DLG1	mRNA	XR_001040147.1	lncRNA
XM_012096602.2	DLG1	mRNA	XR_001040147.1	lncRNA
XM_012096602.2	DLG1	mRNA	XR_001040147.1	lncRNA
XM_012096602.2	DLG1	mRNA	XR_001040147.1	lncRNA
XM_012096602.2	DLG1	mRNA	XR_001040147.1	lncRNA

XM_004012073.3	FNDC3A	mRNA	XR_001040147.1	lncRNA
XM_004012073.3	FNDC3A	mRNA	XR_001040147.1	lncRNA
XM_004012073.3	FNDC3A	mRNA	XR_001040147.1	lncRNA
XM_004012073.3	FNDC3A	mRNA	XR_001040147.1	lncRNA
XM_004012073.3	FNDC3A	mRNA	XR_001040147.1	lncRNA
XM_004020402.3	RTTN	mRNA	XR_001436390.1	lncRNA
XM_004020402.3	RTTN	mRNA	XR_001436390.1	lncRNA
XM_004020402.3	RTTN	mRNA	XR_001436390.1	lncRNA
XM_004020402.3	RTTN	mRNA	XR_001436390.1	lncRNA
XM_004020402.3	RTTN	mRNA	XR_001436390.1	lncRNA
XM_012187727.2	TRMT1L	mRNA	XR_001436390.1	lncRNA
XM_012187727.2	TRMT1L	mRNA	XR_001436390.1	lncRNA
XM_012187727.2	TRMT1L	mRNA	XR_001436390.1	lncRNA
XM_012187727.2	TRMT1L	mRNA	XR_001436390.1	lncRNA
XM_012187727.2	TRMT1L	mRNA	XR_001436390.1	lncRNA
XM_015092971.1	SNX30	mRNA	TCONS_00049802	lncRNA
XM_015092971.1	SNX30	mRNA	TCONS_00049802	lncRNA
XM_015092971.1	SNX30	mRNA	TCONS_00049802	lncRNA
XM_015092971.1	SNX30	mRNA	TCONS_00049802	lncRNA
XM_015092971.1	SNX30	mRNA	TCONS_00049802	lncRNA
XM_015092063.1	FUBP1	mRNA	XR_001023627.2	lncRNA
XM_015092063.1	FUBP1	mRNA	XR_001023627.2	lncRNA
XM_015092063.1	FUBP1	mRNA	XR_001023627.2	lncRNA
XM_015092063.1	FUBP1	mRNA	XR_001023627.2	lncRNA
XM_015092063.1	FUBP1	mRNA	XR_001023627.2	lncRNA
XM_012098903.1	PPIP5K1	mRNA	XR_001023627.2	lncRNA
XM_012098903.1	PPIP5K1	mRNA	XR_001023627.2	lncRNA
XM_012098903.1	PPIP5K1	mRNA	XR_001023627.2	lncRNA
XM_012098903.1	PPIP5K1	mRNA	XR_001023627.2	lncRNA
XM_012098903.1	PPIP5K1	mRNA	XR_001023627.2	lncRNA
XM_015092971.1	SNX30	mRNA	XR_001434127.1	lncRNA
XM_015092971.1	SNX30	mRNA	XR_001434127.1	lncRNA
XM_015092971.1	SNX30	mRNA	XR_001434127.1	lncRNA
XM_015092971.1	SNX30	mRNA	XR_001434127.1	lncRNA
XM_015092971.1	SNX30	mRNA	XR_001434127.1	lncRNA
XM_012183298.1	FAM120B	mRNA	XR_001434127.1	lncRNA
XM_012183298.1	FAM120B	mRNA	XR_001434127.1	lncRNA
XM_012183298.1	FAM120B	mRNA	XR_001434127.1	lncRNA
XM_012183298.1	FAM120B	mRNA	XR_001434127.1	lncRNA
XM_012183298.1	FAM120B	mRNA	XR_001434127.1	lncRNA
XM_012106946.2	ATP7A	mRNA	XR_001023627.2	lncRNA
XM_012106946.2	ATP7A	mRNA	XR_001023627.2	lncRNA
XM_012106946.2	ATP7A	mRNA	XR_001023627.2	lncRNA
XM_012106946.2	ATP7A	mRNA	XR_001023627.2	lncRNA
XM_012106946.2	ATP7A	mRNA	XR_001023627.2	lncRNA
XM_012146156.1	USP37	mRNA	XR_001434127.1	lncRNA
XM_012146156.1	USP37	mRNA	XR_001434127.1	lncRNA
XM_012146156.1	USP37	mRNA	XR_001434127.1	lncRNA
XM_012146156.1	USP37	mRNA	XR_001434127.1	lncRNA
XM_012146156.1	USP37	mRNA	XR_001434127.1	lncRNA
XM_015092971.1	SNX30	mRNA	XR_001023627.2	lncRNA
XM_015092971.1	SNX30	mRNA	XR_001023627.2	lncRNA
XM_015092971.1	SNX30	mRNA	XR_001023627.2	lncRNA
XM_015092971.1	SNX30	mRNA	XR_001023627.2	lncRNA

XM_015092971.1	SNX30	mRNA	XR_001023627.2	lncRNA
XM_012107808.1	NFYC	mRNA	XR_001434127.1	lncRNA
XM_012107808.1	NFYC	mRNA	XR_001434127.1	lncRNA
XM_012107808.1	NFYC	mRNA	XR_001434127.1	lncRNA
XM_012107808.1	NFYC	mRNA	XR_001434127.1	lncRNA
XM_012107808.1	NFYC	mRNA	XR_001434127.1	lncRNA
XM_012146156.1	USP37	mRNA	XR_001023627.2	lncRNA
XM_012146156.1	USP37	mRNA	XR_001023627.2	lncRNA
XM_012146156.1	USP37	mRNA	XR_001023627.2	lncRNA
XM_012146156.1	USP37	mRNA	XR_001023627.2	lncRNA
XM_012146156.1	USP37	mRNA	XR_001023627.2	lncRNA
XM_015092063.1	FUBP1	mRNA	TCONS_00049802	lncRNA
XM_015092063.1	FUBP1	mRNA	TCONS_00049802	lncRNA
XM_015092063.1	FUBP1	mRNA	TCONS_00049802	lncRNA
XM_015092063.1	FUBP1	mRNA	TCONS_00049802	lncRNA
XM_015092063.1	FUBP1	mRNA	TCONS_00049802	lncRNA
XM_012188274.2	CCDC7	mRNA	TCONS_00049802	lncRNA
XM_012188274.2	CCDC7	mRNA	TCONS_00049802	lncRNA
XM_012188274.2	CCDC7	mRNA	TCONS_00049802	lncRNA
XM_012188274.2	CCDC7	mRNA	TCONS_00049802	lncRNA
XM_012188274.2	CCDC7	mRNA	TCONS_00049802	lncRNA
XM_012184362.2	AKAP11	mRNA	XR_001433837.1	lncRNA
XM_012184362.2	AKAP11	mRNA	XR_001433837.1	lncRNA
XM_012184362.2	AKAP11	mRNA	XR_001433837.1	lncRNA
XM_012184362.2	AKAP11	mRNA	XR_001433837.1	lncRNA
XM_012184362.2	AKAP11	mRNA	XR_001433837.1	lncRNA
XM_015093065.1	FAM219A	mRNA	XR_001433837.1	lncRNA
XM_015093065.1	FAM219A	mRNA	XR_001433837.1	lncRNA
XM_015093065.1	FAM219A	mRNA	XR_001433837.1	lncRNA
XM_015093065.1	FAM219A	mRNA	XR_001433837.1	lncRNA
XM_015093065.1	FAM219A	mRNA	XR_001433837.1	lncRNA
XM_012179676.2	CCSER1	mRNA	XR_001433837.1	lncRNA
XM_012179676.2	CCSER1	mRNA	XR_001433837.1	lncRNA
XM_012179676.2	CCSER1	mRNA	XR_001433837.1	lncRNA
XM_012179676.2	CCSER1	mRNA	XR_001433837.1	lncRNA
XM_012179676.2	CCSER1	mRNA	XR_001433837.1	lncRNA
XM_015092364.1	GART	mRNA	XR_001433837.1	lncRNA
XM_015092364.1	GART	mRNA	XR_001433837.1	lncRNA
XM_015092364.1	GART	mRNA	XR_001433837.1	lncRNA
XM_015092364.1	GART	mRNA	XR_001433837.1	lncRNA
XM_015092364.1	GART	mRNA	XR_001433837.1	lncRNA
XM_004011768.3	SAMD12	mRNA	XR_001433837.1	lncRNA
XM_004011768.3	SAMD12	mRNA	XR_001433837.1	lncRNA
XM_004011768.3	SAMD12	mRNA	XR_001433837.1	lncRNA
XM_004011768.3	SAMD12	mRNA	XR_001433837.1	lncRNA
XM_004011768.3	SAMD12	mRNA	XR_001433837.1	lncRNA
XM_015101001.1	RRAS2	mRNA	XR_001433837.1	lncRNA
XM_015101001.1	RRAS2	mRNA	XR_001433837.1	lncRNA
XM_015101001.1	RRAS2	mRNA	XR_001433837.1	lncRNA
XM_015101001.1	RRAS2	mRNA	XR_001433837.1	lncRNA
XM_015101001.1	RRAS2	mRNA	XR_001433837.1	lncRNA
XM_012184885.2	DOCK9	mRNA	XR_001433837.1	lncRNA
XM_012184885.2	DOCK9	mRNA	XR_001433837.1	lncRNA
XM_012184885.2	DOCK9	mRNA	XR_001433837.1	lncRNA

XM_012184885.2	DOCK9	mRNA	XR_001433837.1	lncRNA
XM_012184885.2	DOCK9	mRNA	XR_001433837.1	lncRNA
XM_015099548.1	PARD3	mRNA	XR_001023303.2	lncRNA
XM_015099548.1	PARD3	mRNA	XR_001023303.2	lncRNA
XM_015099548.1	PARD3	mRNA	XR_001023303.2	lncRNA
XM_015099548.1	PARD3	mRNA	XR_001023303.2	lncRNA
XM_015099548.1	PARD3	mRNA	XR_001023303.2	lncRNA
XM_004012101.3	FREM2	mRNA	XR_001023303.2	lncRNA
XM_004012101.3	FREM2	mRNA	XR_001023303.2	lncRNA
XM_004012101.3	FREM2	mRNA	XR_001023303.2	lncRNA
XM_004012101.3	FREM2	mRNA	XR_001023303.2	lncRNA
XM_004012101.3	FREM2	mRNA	XR_001023303.2	lncRNA
XM_012137434.2	NEB	mRNA	XR_001024966.2	lncRNA
XM_012137434.2	NEB	mRNA	XR_001024966.2	lncRNA
XM_012137434.2	NEB	mRNA	XR_001024966.2	lncRNA
XM_012137434.2	NEB	mRNA	XR_001024966.2	lncRNA
XM_012137434.2	NEB	mRNA	XR_001024966.2	lncRNA
XM_015094531.1	ANAPC1	mRNA	XR_001023303.2	lncRNA
XM_015094531.1	ANAPC1	mRNA	XR_001023303.2	lncRNA
XM_015094531.1	ANAPC1	mRNA	XR_001023303.2	lncRNA
XM_015094531.1	ANAPC1	mRNA	XR_001023303.2	lncRNA
XM_015094531.1	ANAPC1	mRNA	XR_001023303.2	lncRNA
XM_015094531.1	ANAPC1	mRNA	XR_001024966.2	lncRNA
XM_015094531.1	ANAPC1	mRNA	XR_001024966.2	lncRNA
XM_015094531.1	ANAPC1	mRNA	XR_001024966.2	lncRNA
XM_015094531.1	ANAPC1	mRNA	XR_001024966.2	lncRNA
XM_015094531.1	ANAPC1	mRNA	XR_001024966.2	lncRNA
XM_015096977.1	NIN	mRNA	XR_001436612.1	lncRNA
XM_015096977.1	NIN	mRNA	XR_001436612.1	lncRNA
XM_015096977.1	NIN	mRNA	XR_001436612.1	lncRNA
XM_015096977.1	NIN	mRNA	XR_001436612.1	lncRNA
XM_015096977.1	NIN	mRNA	XR_001436612.1	lncRNA
XM_012188097.2	PLCB1	mRNA	XR_001024230.1	lncRNA
XM_012188097.2	PLCB1	mRNA	XR_001024230.1	lncRNA
XM_012188097.2	PLCB1	mRNA	XR_001024230.1	lncRNA
XM_012188097.2	PLCB1	mRNA	XR_001024230.1	lncRNA
XM_012188097.2	PLCB1	mRNA	XR_001024230.1	lncRNA
XM_012187727.2	TRMT1L	mRNA	XR_001435726.1	lncRNA
XM_012187727.2	TRMT1L	mRNA	XR_001435726.1	lncRNA
XM_012187727.2	TRMT1L	mRNA	XR_001435726.1	lncRNA
XM_012187727.2	TRMT1L	mRNA	XR_001435726.1	lncRNA
XM_012187727.2	TRMT1L	mRNA	XR_001435726.1	lncRNA
XM_004004584.3	RAPGEF4	mRNA	XR_001435723.1	lncRNA
XM_004004584.3	RAPGEF4	mRNA	XR_001435723.1	lncRNA
XM_004004584.3	RAPGEF4	mRNA	XR_001435723.1	lncRNA
XM_004004584.3	RAPGEF4	mRNA	XR_001435723.1	lncRNA
XM_004004584.3	RAPGEF4	mRNA	XR_001435723.1	lncRNA
XM_015098592.1	RABEP1	mRNA	XR_001435726.1	lncRNA
XM_015098592.1	RABEP1	mRNA	XR_001435726.1	lncRNA
XM_015098592.1	RABEP1	mRNA	XR_001435726.1	lncRNA
XM_015098592.1	RABEP1	mRNA	XR_001435726.1	lncRNA
XM_015098592.1	RABEP1	mRNA	XR_001435726.1	lncRNA
XM_012176761.2	MDFIC	mRNA	XR_001436064.1	lncRNA
XM_012176761.2	MDFIC	mRNA	XR_001436064.1	lncRNA

XM_012176761.2	MDFIC	mRNA	XR_001436064.1	lncRNA
XM_012176761.2	MDFIC	mRNA	XR_001436064.1	lncRNA
XM_012176761.2	MDFIC	mRNA	XR_001436064.1	lncRNA
XM_015096518.1	AASDH	mRNA	XR_001435726.1	lncRNA
XM_015096518.1	AASDH	mRNA	XR_001435726.1	lncRNA
XM_015096518.1	AASDH	mRNA	XR_001435726.1	lncRNA
XM_015096518.1	AASDH	mRNA	XR_001435726.1	lncRNA
XM_015096518.1	AASDH	mRNA	XR_001435726.1	lncRNA
XM_012176414.2	PHF14	mRNA	XR_001435726.1	lncRNA
XM_012176414.2	PHF14	mRNA	XR_001435726.1	lncRNA
XM_012176414.2	PHF14	mRNA	XR_001435726.1	lncRNA
XM_012176414.2	PHF14	mRNA	XR_001435726.1	lncRNA
XM_012176414.2	PHF14	mRNA	XR_001435726.1	lncRNA
XM_015096518.1	AASDH	mRNA	XR_001435723.1	lncRNA
XM_015096518.1	AASDH	mRNA	XR_001435723.1	lncRNA
XM_015096518.1	AASDH	mRNA	XR_001435723.1	lncRNA
XM_015096518.1	AASDH	mRNA	XR_001435723.1	lncRNA
XM_015096518.1	AASDH	mRNA	XR_001435723.1	lncRNA
XM_012176414.2	PHF14	mRNA	XR_001435723.1	lncRNA
XM_012176414.2	PHF14	mRNA	XR_001435723.1	lncRNA
XM_012176414.2	PHF14	mRNA	XR_001435723.1	lncRNA
XM_012176414.2	PHF14	mRNA	XR_001435723.1	lncRNA
XM_012176414.2	PHF14	mRNA	XR_001435723.1	lncRNA
XM_012181827.2	ZBTB1	mRNA	XR_001435726.1	lncRNA
XM_012181827.2	ZBTB1	mRNA	XR_001435726.1	lncRNA
XM_012181827.2	ZBTB1	mRNA	XR_001435726.1	lncRNA
XM_012181827.2	ZBTB1	mRNA	XR_001435726.1	lncRNA
XM_012181827.2	ZBTB1	mRNA	XR_001435726.1	lncRNA
XM_012187727.2	TRMT1L	mRNA	XR_001435723.1	lncRNA
XM_012187727.2	TRMT1L	mRNA	XR_001435723.1	lncRNA
XM_012187727.2	TRMT1L	mRNA	XR_001435723.1	lncRNA
XM_012187727.2	TRMT1L	mRNA	XR_001435723.1	lncRNA
XM_012187727.2	TRMT1L	mRNA	XR_001435723.1	lncRNA
XM_012099509.2	BRPF1	mRNA	XR_001436390.1	lncRNA
XM_012099509.2	BRPF1	mRNA	XR_001436390.1	lncRNA
XM_012099509.2	BRPF1	mRNA	XR_001436390.1	lncRNA
XM_012099509.2	BRPF1	mRNA	XR_001436390.1	lncRNA
XM_012099509.2	BRPF1	mRNA	XR_001436390.1	lncRNA
XM_012173383.2	DHX57	mRNA	XR_001040147.1	lncRNA
XM_012173383.2	DHX57	mRNA	XR_001040147.1	lncRNA
XM_012173383.2	DHX57	mRNA	XR_001040147.1	lncRNA
XM_012173383.2	DHX57	mRNA	XR_001040147.1	lncRNA
XM_012173383.2	DHX57	mRNA	XR_001040147.1	lncRNA
XM_015095425.1	CUL1	mRNA	XR_001436390.1	lncRNA
XM_015095425.1	CUL1	mRNA	XR_001436390.1	lncRNA
XM_015095425.1	CUL1	mRNA	XR_001436390.1	lncRNA
XM_015095425.1	CUL1	mRNA	XR_001436390.1	lncRNA
XM_015095425.1	CUL1	mRNA	XR_001436390.1	lncRNA
XM_012097468.2	ELF2	mRNA	XR_001040147.1	lncRNA
XM_012097468.2	ELF2	mRNA	XR_001040147.1	lncRNA
XM_012097468.2	ELF2	mRNA	XR_001040147.1	lncRNA
XM_012097468.2	ELF2	mRNA	XR_001040147.1	lncRNA
XM_012097468.2	ELF2	mRNA	XR_001040147.1	lncRNA
XM_015100436.1	CYLD	mRNA	XR_001040147.1	lncRNA

XM_015100436.1	CYLD	mRNA	XR_001040147.1	lncRNA
XM_015100436.1	CYLD	mRNA	XR_001040147.1	lncRNA
XM_015100436.1	CYLD	mRNA	XR_001040147.1	lncRNA
XM_015100436.1	CYLD	mRNA	XR_001040147.1	lncRNA
XM_012105324.2	USP54	mRNA	XR_001433837.1	lncRNA
XM_012105324.2	USP54	mRNA	XR_001433837.1	lncRNA
XM_012105324.2	USP54	mRNA	XR_001433837.1	lncRNA
XM_012105324.2	USP54	mRNA	XR_001433837.1	lncRNA
XM_012105324.2	USP54	mRNA	XR_001433837.1	lncRNA
XM_012097555.2	RAPGEF2	mRNA	XR_001433837.1	lncRNA
XM_012097555.2	RAPGEF2	mRNA	XR_001433837.1	lncRNA
XM_012097555.2	RAPGEF2	mRNA	XR_001433837.1	lncRNA
XM_012097555.2	RAPGEF2	mRNA	XR_001433837.1	lncRNA
XM_012097555.2	RAPGEF2	mRNA	XR_001433837.1	lncRNA
XM_015099563.1	PARD3	mRNA	XR_001433837.1	lncRNA
XM_015099563.1	PARD3	mRNA	XR_001433837.1	lncRNA
XM_015099563.1	PARD3	mRNA	XR_001433837.1	lncRNA
XM_015099563.1	PARD3	mRNA	XR_001433837.1	lncRNA
XM_015099563.1	PARD3	mRNA	XR_001433837.1	lncRNA
XM_012097365.2	SH3D19	mRNA	XR_001434127.1	lncRNA
XM_012097365.2	SH3D19	mRNA	XR_001434127.1	lncRNA
XM_012097365.2	SH3D19	mRNA	XR_001434127.1	lncRNA
XM_012097365.2	SH3D19	mRNA	XR_001434127.1	lncRNA
XM_012097365.2	SH3D19	mRNA	XR_001434127.1	lncRNA
XM_012185338.2	ZNF207	mRNA	TCONS_00049802	lncRNA
XM_012185338.2	ZNF207	mRNA	TCONS_00049802	lncRNA
XM_012185338.2	ZNF207	mRNA	TCONS_00049802	lncRNA
XM_012185338.2	ZNF207	mRNA	TCONS_00049802	lncRNA
XM_012185338.2	ZNF207	mRNA	TCONS_00049802	lncRNA
XM_004017788.3	ZFANL3	mRNA	XR_001023627.2	lncRNA
XM_004017788.3	ZFANL3	mRNA	XR_001023627.2	lncRNA
XM_004017788.3	ZFANL3	mRNA	XR_001023627.2	lncRNA
XM_004017788.3	ZFANL3	mRNA	XR_001023627.2	lncRNA
XM_004017788.3	ZFANL3	mRNA	XR_001023627.2	lncRNA
XM_012180234.2	CNOT6L	mRNA	TCONS_00049802	lncRNA
XM_012180234.2	CNOT6L	mRNA	TCONS_00049802	lncRNA
XM_012180234.2	CNOT6L	mRNA	TCONS_00049802	lncRNA
XM_012180234.2	CNOT6L	mRNA	TCONS_00049802	lncRNA
XM_012180234.2	CNOT6L	mRNA	TCONS_00049802	lncRNA
XM_015095237.1	DMTF1	mRNA	XR_001023627.2	lncRNA
XM_015095237.1	DMTF1	mRNA	XR_001023627.2	lncRNA
XM_015095237.1	DMTF1	mRNA	XR_001023627.2	lncRNA
XM_015095237.1	DMTF1	mRNA	XR_001023627.2	lncRNA
XM_015095237.1	DMTF1	mRNA	XR_001023627.2	lncRNA
XM_015101000.1	RRAS2	mRNA	XR_001023303.2	lncRNA
XM_015101000.1	RRAS2	mRNA	XR_001023303.2	lncRNA
XM_015101000.1	RRAS2	mRNA	XR_001023303.2	lncRNA
XM_015101000.1	RRAS2	mRNA	XR_001023303.2	lncRNA
XM_004022228.3	LOC101122577	mRNA	XR_001041893.2	lncRNA
XM_004022228.3	LOC101122577	mRNA	XR_001041893.2	lncRNA
XM_004022228.3	LOC101122577	mRNA	XR_001041893.2	lncRNA
XM_004022228.3	LOC101122577	mRNA	XR_001041893.2	lncRNA
XM_012183459.1	ISG20L2	mRNA	XR_001024966.2	lncRNA
XM_012183459.1	ISG20L2	mRNA	XR_001024966.2	lncRNA

XM_012183459.1	ISG20L2	mRNA	XR_001024966.2	lncRNA
XM_012183459.1	ISG20L2	mRNA	XR_001024966.2	lncRNA
XM_012181992.1	ZFYVE1	mRNA	XR_001041893.2	lncRNA
XM_012181992.1	ZFYVE1	mRNA	XR_001041893.2	lncRNA
XM_012181992.1	ZFYVE1	mRNA	XR_001041893.2	lncRNA
XM_012181992.1	ZFYVE1	mRNA	XR_001041893.2	lncRNA
XM_012098812.2	RALGAPA1	mRNA	XR_001040147.1	lncRNA
XM_012098812.2	RALGAPA1	mRNA	XR_001040147.1	lncRNA
XM_012098812.2	RALGAPA1	mRNA	XR_001040147.1	lncRNA
XM_012098812.2	RALGAPA1	mRNA	XR_001040147.1	lncRNA
XM_012098812.2	RALGAPA1	mRNA	XR_001040147.1	lncRNA
XM_012182353.2	LRRFIP1	mRNA	XR_001436390.1	lncRNA
XM_012182353.2	LRRFIP1	mRNA	XR_001436390.1	lncRNA
XM_012182353.2	LRRFIP1	mRNA	XR_001436390.1	lncRNA
XM_012182353.2	LRRFIP1	mRNA	XR_001436390.1	lncRNA
XM_012182353.2	LRRFIP1	mRNA	XR_001436390.1	lncRNA
XM_015096977.1	NIN	mRNA	XR_001040147.1	lncRNA
XM_015096977.1	NIN	mRNA	XR_001040147.1	lncRNA
XM_015096977.1	NIN	mRNA	XR_001040147.1	lncRNA
XM_015096977.1	NIN	mRNA	XR_001040147.1	lncRNA
XM_015096977.1	NIN	mRNA	XR_001040147.1	lncRNA
XM_015103466.1	TACC2	mRNA	XR_001040147.1	lncRNA
XM_015103466.1	TACC2	mRNA	XR_001040147.1	lncRNA
XM_015103466.1	TACC2	mRNA	XR_001040147.1	lncRNA
XM_015103466.1	TACC2	mRNA	XR_001040147.1	lncRNA
XM_015103466.1	TACC2	mRNA	XR_001040147.1	lncRNA
XM_004011768.3	SAMD12	mRNA	XR_001434127.1	lncRNA
XM_004011768.3	SAMD12	mRNA	XR_001434127.1	lncRNA
XM_004011768.3	SAMD12	mRNA	XR_001434127.1	lncRNA
XM_004011768.3	SAMD12	mRNA	XR_001434127.1	lncRNA
XM_004011768.3	SAMD12	mRNA	XR_001434127.1	lncRNA
XM_012102598.2	TBC1D12	mRNA	XR_001433837.1	lncRNA
XM_012102598.2	TBC1D12	mRNA	XR_001433837.1	lncRNA
XM_012102598.2	TBC1D12	mRNA	XR_001433837.1	lncRNA
XM_012102598.2	TBC1D12	mRNA	XR_001433837.1	lncRNA
XM_015094252.1	DNMT3A	mRNA	TCONS_00068429	lncRNA
XM_015094252.1	DNMT3A	mRNA	TCONS_00068429	lncRNA
XM_015094252.1	DNMT3A	mRNA	TCONS_00068429	lncRNA
XM_015094252.1	DNMT3A	mRNA	TCONS_00068429	lncRNA
XM_012102667.1	ZNF518A	mRNA	XR_001436612.1	lncRNA
XM_012102667.1	ZNF518A	mRNA	XR_001436612.1	lncRNA
XM_012102667.1	ZNF518A	mRNA	XR_001436612.1	lncRNA
XM_012102667.1	ZNF518A	mRNA	XR_001436612.1	lncRNA
XM_015098987.1	ABCA5	mRNA	XR_001436612.1	lncRNA
XM_015098987.1	ABCA5	mRNA	XR_001436612.1	lncRNA
XM_015098987.1	ABCA5	mRNA	XR_001436612.1	lncRNA
XM_015098987.1	ABCA5	mRNA	XR_001436612.1	lncRNA
XM_012183459.1	ISG20L2	mRNA	XR_001436612.1	lncRNA
XM_012183459.1	ISG20L2	mRNA	XR_001436612.1	lncRNA
XM_012183459.1	ISG20L2	mRNA	XR_001436612.1	lncRNA
XM_012183459.1	ISG20L2	mRNA	XR_001436612.1	lncRNA
XM_004014588.3	PKIG	mRNA	XR_001436612.1	lncRNA
XM_004014588.3	PKIG	mRNA	XR_001436612.1	lncRNA
XM_004014588.3	PKIG	mRNA	XR_001436612.1	lncRNA

XM_004014588.3	PKIG	mRNA	XR_001436612.1	lncRNA
XM_012184822.2	RBM26	mRNA	XR_001023303.2	lncRNA
XM_012184822.2	RBM26	mRNA	XR_001023303.2	lncRNA
XM_012184822.2	RBM26	mRNA	XR_001023303.2	lncRNA
XM_012184822.2	RBM26	mRNA	XR_001023303.2	lncRNA
XM_012102930.1	GPAM	mRNA	XR_001024966.2	lncRNA
XM_012102930.1	GPAM	mRNA	XR_001024966.2	lncRNA
XM_012102930.1	GPAM	mRNA	XR_001024966.2	lncRNA
XM_012102930.1	GPAM	mRNA	XR_001024966.2	lncRNA
XM_012176469.2	MACC1	mRNA	XR_001024966.2	lncRNA
XM_012176469.2	MACC1	mRNA	XR_001024966.2	lncRNA
XM_012176469.2	MACC1	mRNA	XR_001024966.2	lncRNA
XM_012176469.2	MACC1	mRNA	XR_001024966.2	lncRNA
XM_012143452.2	NRP2	mRNA	XR_001024966.2	lncRNA
XM_012143452.2	NRP2	mRNA	XR_001024966.2	lncRNA
XM_012143452.2	NRP2	mRNA	XR_001024966.2	lncRNA
XM_012143452.2	NRP2	mRNA	XR_001024966.2	lncRNA
XM_015092197.1	CD58	mRNA	XR_001024966.2	lncRNA
XM_015092197.1	CD58	mRNA	XR_001024966.2	lncRNA
XM_015092197.1	CD58	mRNA	XR_001024966.2	lncRNA
XM_015092197.1	CD58	mRNA	XR_001024966.2	lncRNA
XM_012095502.1	SIK3	mRNA	XR_001024966.2	lncRNA
XM_012095502.1	SIK3	mRNA	XR_001024966.2	lncRNA
XM_012095502.1	SIK3	mRNA	XR_001024966.2	lncRNA
XM_012095502.1	SIK3	mRNA	XR_001024966.2	lncRNA
XM_012184401.1	ZC3H13	mRNA	TCONS_00049802	lncRNA
XM_012184401.1	ZC3H13	mRNA	TCONS_00049802	lncRNA
XM_012184401.1	ZC3H13	mRNA	TCONS_00049802	lncRNA
XM_012184401.1	ZC3H13	mRNA	TCONS_00049802	lncRNA
XM_012110487.2	UBQLN1	mRNA	TCONS_00049802	lncRNA
XM_012110487.2	UBQLN1	mRNA	TCONS_00049802	lncRNA
XM_012110487.2	UBQLN1	mRNA	TCONS_00049802	lncRNA
XM_012110487.2	UBQLN1	mRNA	TCONS_00049802	lncRNA
XM_012187364.1	ENO1	mRNA	TCONS_00049802	lncRNA
XM_012187364.1	ENO1	mRNA	TCONS_00049802	lncRNA
XM_012187364.1	ENO1	mRNA	TCONS_00049802	lncRNA
XM_012187364.1	ENO1	mRNA	TCONS_00049802	lncRNA
XM_012099659.2	FOXP1	mRNA	TCONS_00049802	lncRNA
XM_012099659.2	FOXP1	mRNA	TCONS_00049802	lncRNA
XM_012099659.2	FOXP1	mRNA	TCONS_00049802	lncRNA
XM_012099659.2	FOXP1	mRNA	TCONS_00049802	lncRNA
XM_012175065.2	AMN1	mRNA	TCONS_00049802	lncRNA
XM_012175065.2	AMN1	mRNA	TCONS_00049802	lncRNA
XM_012175065.2	AMN1	mRNA	TCONS_00049802	lncRNA
XM_012175065.2	AMN1	mRNA	TCONS_00049802	lncRNA
XM_015098082.1	RNASEH2B	mRNA	TCONS_00068429	lncRNA
XM_015098082.1	RNASEH2B	mRNA	TCONS_00068429	lncRNA
XM_015098082.1	RNASEH2B	mRNA	TCONS_00068429	lncRNA
XM_015098082.1	RNASEH2B	mRNA	TCONS_00068429	lncRNA
XM_012105331.2	USP54	mRNA	TCONS_00068429	lncRNA
XM_012105331.2	USP54	mRNA	TCONS_00068429	lncRNA
XM_012105331.2	USP54	mRNA	TCONS_00068429	lncRNA
XM_012105331.2	USP54	mRNA	TCONS_00068429	lncRNA
XM_004009738.3	PPARGC1A	mRNA	XR_001041220.2	lncRNA

XM_004009738.3	PPARGC1A	mRNA	XR_001041220.2	lncRNA
XM_004009738.3	PPARGC1A	mRNA	XR_001041220.2	lncRNA
XM_004009738.3	PPARGC1A	mRNA	XR_001041220.2	lncRNA
XM_012105333.2	USP54	mRNA	TCONS_00068429	lncRNA
XM_012105333.2	USP54	mRNA	TCONS_00068429	lncRNA
XM_012105333.2	USP54	mRNA	TCONS_00068429	lncRNA
XM_012105333.2	USP54	mRNA	TCONS_00068429	lncRNA
XM_015103688.1	PTPN2	mRNA	XR_001433837.1	lncRNA
XM_015103688.1	PTPN2	mRNA	XR_001433837.1	lncRNA
XM_015103688.1	PTPN2	mRNA	XR_001433837.1	lncRNA
XM_015103688.1	PTPN2	mRNA	XR_001433837.1	lncRNA
XM_012102930.1	GPAM	mRNA	XR_001433837.1	lncRNA
XM_012102930.1	GPAM	mRNA	XR_001433837.1	lncRNA
XM_012102930.1	GPAM	mRNA	XR_001433837.1	lncRNA
XM_012102930.1	GPAM	mRNA	XR_001433837.1	lncRNA
XM_012188348.1	KIAA1217	mRNA	TCONS_00068429	lncRNA
XM_012188348.1	KIAA1217	mRNA	TCONS_00068429	lncRNA
XM_012188348.1	KIAA1217	mRNA	TCONS_00068429	lncRNA
XM_012188348.1	KIAA1217	mRNA	TCONS_00068429	lncRNA
XM_015103687.1	PTPN2	mRNA	XR_001041220.2	lncRNA
XM_015103687.1	PTPN2	mRNA	XR_001041220.2	lncRNA
XM_015103687.1	PTPN2	mRNA	XR_001041220.2	lncRNA
XM_015103687.1	PTPN2	mRNA	XR_001041220.2	lncRNA
XM_012096323.1	PHF21A	mRNA	XR_001041220.2	lncRNA
XM_012096323.1	PHF21A	mRNA	XR_001041220.2	lncRNA
XM_012096323.1	PHF21A	mRNA	XR_001041220.2	lncRNA
XM_012096323.1	PHF21A	mRNA	XR_001041220.2	lncRNA
XM_015099738.1	LOC101122805	mRNA	XR_001024290.1	lncRNA
XM_015099738.1	LOC101122805	mRNA	XR_001024290.1	lncRNA
XM_015099738.1	LOC101122805	mRNA	XR_001024290.1	lncRNA
XM_015099738.1	LOC101122805	mRNA	XR_001024290.1	lncRNA
XM_004012193.3	RBM26	mRNA	XR_001436390.1	lncRNA
XM_004012193.3	RBM26	mRNA	XR_001436390.1	lncRNA
XM_004012193.3	RBM26	mRNA	XR_001436390.1	lncRNA
XM_004012193.3	RBM26	mRNA	XR_001436390.1	lncRNA
XM_015103487.1	LOC101102356	mRNA	XR_001436390.1	lncRNA
XM_015103487.1	LOC101102356	mRNA	XR_001436390.1	lncRNA
XM_015103487.1	LOC101102356	mRNA	XR_001436390.1	lncRNA
XM_015103487.1	LOC101102356	mRNA	XR_001436390.1	lncRNA
XM_012174289.1	ATF1	mRNA	XR_001436390.1	lncRNA
XM_012174289.1	ATF1	mRNA	XR_001436390.1	lncRNA
XM_012174289.1	ATF1	mRNA	XR_001436390.1	lncRNA
XM_012174289.1	ATF1	mRNA	XR_001436390.1	lncRNA
XM_012182520.2	SLC35F1	mRNA	XR_001436390.1	lncRNA
XM_012182520.2	SLC35F1	mRNA	XR_001436390.1	lncRNA
XM_012182520.2	SLC35F1	mRNA	XR_001436390.1	lncRNA
XM_012182520.2	SLC35F1	mRNA	XR_001436390.1	lncRNA
XM_012146477.2	ABCB6	mRNA	XR_001436390.1	lncRNA
XM_012146477.2	ABCB6	mRNA	XR_001436390.1	lncRNA
XM_012146477.2	ABCB6	mRNA	XR_001436390.1	lncRNA
XM_012146477.2	ABCB6	mRNA	XR_001436390.1	lncRNA
XM_012099221.2	SLC25A26	mRNA	XR_001436390.1	lncRNA
XM_012099221.2	SLC25A26	mRNA	XR_001436390.1	lncRNA
XM_012099221.2	SLC25A26	mRNA	XR_001436390.1	lncRNA

XM_012099221.2	SLC25A26	mRNA	XR_001436390.1	lncRNA
XM_004008085.3	CNOT4	mRNA	XR_001436390.1	lncRNA
XM_004008085.3	CNOT4	mRNA	XR_001436390.1	lncRNA
XM_004008085.3	CNOT4	mRNA	XR_001436390.1	lncRNA
XM_004008085.3	CNOT4	mRNA	XR_001436390.1	lncRNA
XM_012099221.2	SLC25A26	mRNA	XR_001040147.1	lncRNA
XM_012099221.2	SLC25A26	mRNA	XR_001040147.1	lncRNA
XM_012099221.2	SLC25A26	mRNA	XR_001040147.1	lncRNA
XM_012099221.2	SLC25A26	mRNA	XR_001040147.1	lncRNA
XM_012176829.1	BBS9	mRNA	XR_001436390.1	lncRNA
XM_012176829.1	BBS9	mRNA	XR_001436390.1	lncRNA
XM_012176829.1	BBS9	mRNA	XR_001436390.1	lncRNA
XM_012176829.1	BBS9	mRNA	XR_001436390.1	lncRNA
XM_015098987.1	ABCA5	mRNA	XR_001436390.1	lncRNA
XM_015098987.1	ABCA5	mRNA	XR_001436390.1	lncRNA
XM_015098987.1	ABCA5	mRNA	XR_001436390.1	lncRNA
XM_015098987.1	ABCA5	mRNA	XR_001436390.1	lncRNA
XM_015092648.1	ARMC8	mRNA	XR_001436390.1	lncRNA
XM_015092648.1	ARMC8	mRNA	XR_001436390.1	lncRNA
XM_015092648.1	ARMC8	mRNA	XR_001436390.1	lncRNA
XM_015092648.1	ARMC8	mRNA	XR_001436390.1	lncRNA
XM_012183459.1	ISG20L2	mRNA	XR_001436390.1	lncRNA
XM_012183459.1	ISG20L2	mRNA	XR_001436390.1	lncRNA
XM_012183459.1	ISG20L2	mRNA	XR_001436390.1	lncRNA
XM_012183459.1	ISG20L2	mRNA	XR_001436390.1	lncRNA
XM_012174290.1	ATF1	mRNA	XR_001436390.1	lncRNA
XM_012174290.1	ATF1	mRNA	XR_001436390.1	lncRNA
XM_012174290.1	ATF1	mRNA	XR_001436390.1	lncRNA
XM_012174290.1	ATF1	mRNA	XR_001436390.1	lncRNA
XM_012102077.2	LGALS12	mRNA	XR_001436390.1	lncRNA
XM_012102077.2	LGALS12	mRNA	XR_001436390.1	lncRNA
XM_012102077.2	LGALS12	mRNA	XR_001436390.1	lncRNA
XM_012102077.2	LGALS12	mRNA	XR_001436390.1	lncRNA
XM_015095262.1	COG5	mRNA	XR_001436390.1	lncRNA
XM_015095262.1	COG5	mRNA	XR_001436390.1	lncRNA
XM_015095262.1	COG5	mRNA	XR_001436390.1	lncRNA
XM_015095262.1	COG5	mRNA	XR_001436390.1	lncRNA
XM_004020402.3	RTTN	mRNA	XR_001023303.2	lncRNA
XM_004020402.3	RTTN	mRNA	XR_001023303.2	lncRNA
XM_004020402.3	RTTN	mRNA	XR_001023303.2	lncRNA
XM_004020402.3	RTTN	mRNA	XR_001023303.2	lncRNA
XM_015096932.1	THBS1	mRNA	XR_001024966.2	lncRNA
XM_015096932.1	THBS1	mRNA	XR_001024966.2	lncRNA
XM_015096932.1	THBS1	mRNA	XR_001024966.2	lncRNA
XM_015096932.1	THBS1	mRNA	XR_001024966.2	lncRNA
XM_012190083.2	ZMYM6	mRNA	XR_001023303.2	lncRNA
XM_012190083.2	ZMYM6	mRNA	XR_001023303.2	lncRNA
XM_012190083.2	ZMYM6	mRNA	XR_001023303.2	lncRNA
XM_012190083.2	ZMYM6	mRNA	XR_001023303.2	lncRNA
XM_004004584.3	RAPGEF4	mRNA	XR_001023303.2	lncRNA
XM_004004584.3	RAPGEF4	mRNA	XR_001023303.2	lncRNA
XM_004004584.3	RAPGEF4	mRNA	XR_001023303.2	lncRNA
XM_004004584.3	RAPGEF4	mRNA	XR_001023303.2	lncRNA
XM_015093216.1	TTC39B	mRNA	XR_001041893.2	lncRNA

XM_015093216.1	TTC39B	mRNA	XR_001041893.2	lncRNA
XM_015093216.1	TTC39B	mRNA	XR_001041893.2	lncRNA
XM_015093216.1	TTC39B	mRNA	XR_001041893.2	lncRNA
XM_015096383.1	SGMS2	mRNA	XR_001023303.2	lncRNA
XM_015096383.1	SGMS2	mRNA	XR_001023303.2	lncRNA
XM_015096383.1	SGMS2	mRNA	XR_001023303.2	lncRNA
XM_015096383.1	SGMS2	mRNA	XR_001023303.2	lncRNA
XM_015100095.1	LOC101113523	mRNA	XR_001023303.2	lncRNA
XM_015100095.1	LOC101113523	mRNA	XR_001023303.2	lncRNA
XM_015100095.1	LOC101113523	mRNA	XR_001023303.2	lncRNA
XM_015100095.1	LOC101113523	mRNA	XR_001023303.2	lncRNA
XM_004020402.3	RTTN	mRNA	XR_001024966.2	lncRNA
XM_004020402.3	RTTN	mRNA	XR_001024966.2	lncRNA
XM_004020402.3	RTTN	mRNA	XR_001024966.2	lncRNA
XM_004020402.3	RTTN	mRNA	XR_001024966.2	lncRNA
XM_015096383.1	SGMS2	mRNA	XR_001024966.2	lncRNA
XM_015096383.1	SGMS2	mRNA	XR_001024966.2	lncRNA
XM_015096383.1	SGMS2	mRNA	XR_001024966.2	lncRNA
XM_015096383.1	SGMS2	mRNA	XR_001024966.2	lncRNA
XM_012189779.2	RBL2	mRNA	XR_001023303.2	lncRNA
XM_012189779.2	RBL2	mRNA	XR_001023303.2	lncRNA
XM_012189779.2	RBL2	mRNA	XR_001023303.2	lncRNA
XM_012189779.2	RBL2	mRNA	XR_001023303.2	lncRNA
XM_015102009.1	RALGAPA1	mRNA	XR_001041893.2	lncRNA
XM_015102009.1	RALGAPA1	mRNA	XR_001041893.2	lncRNA
XM_015102009.1	RALGAPA1	mRNA	XR_001041893.2	lncRNA
XM_015102009.1	RALGAPA1	mRNA	XR_001041893.2	lncRNA
XM_004020074.3	PIK3AP1	mRNA	XR_001024966.2	lncRNA
XM_004020074.3	PIK3AP1	mRNA	XR_001024966.2	lncRNA
XM_004020074.3	PIK3AP1	mRNA	XR_001024966.2	lncRNA
XM_004020074.3	PIK3AP1	mRNA	XR_001024966.2	lncRNA
XM_012176761.2	MDFIC	mRNA	XR_001041893.2	lncRNA
XM_012176761.2	MDFIC	mRNA	XR_001041893.2	lncRNA
XM_012176761.2	MDFIC	mRNA	XR_001041893.2	lncRNA
XM_012176761.2	MDFIC	mRNA	XR_001041893.2	lncRNA
XM_015098434.1	STXBP4	mRNA	XR_001041893.2	lncRNA
XM_015098434.1	STXBP4	mRNA	XR_001041893.2	lncRNA
XM_015098434.1	STXBP4	mRNA	XR_001041893.2	lncRNA
XM_015098434.1	STXBP4	mRNA	XR_001041893.2	lncRNA
XM_015094531.1	ANAPC1	mRNA	TCONS_00068429	lncRNA
XM_015094531.1	ANAPC1	mRNA	TCONS_00068429	lncRNA
XM_015094531.1	ANAPC1	mRNA	TCONS_00068429	lncRNA
XM_015094531.1	ANAPC1	mRNA	TCONS_00068429	lncRNA
XM_012175096.2	ERGIC2	mRNA	XR_001436612.1	lncRNA
XM_012175096.2	ERGIC2	mRNA	XR_001436612.1	lncRNA
XM_012175096.2	ERGIC2	mRNA	XR_001436612.1	lncRNA
XM_012175096.2	ERGIC2	mRNA	XR_001436612.1	lncRNA
XM_012101050.1	ZNF311	mRNA	XR_001436612.1	lncRNA
XM_012101050.1	ZNF311	mRNA	XR_001436612.1	lncRNA
XM_012101050.1	ZNF311	mRNA	XR_001436612.1	lncRNA
XM_012101050.1	ZNF311	mRNA	XR_001436612.1	lncRNA
XM_015098186.1	XPO4	mRNA	XR_001436612.1	lncRNA
XM_015098186.1	XPO4	mRNA	XR_001436612.1	lncRNA
XM_015098186.1	XPO4	mRNA	XR_001436612.1	lncRNA

XM_015098186.1	XPO4	mRNA	XR_001436612.1	lncRNA
XM_004012689.3	MYH10	mRNA	XR_001436612.1	lncRNA
XM_004012689.3	MYH10	mRNA	XR_001436612.1	lncRNA
XM_004012689.3	MYH10	mRNA	XR_001436612.1	lncRNA
XM_004012689.3	MYH10	mRNA	XR_001436612.1	lncRNA
XM_012176469.2	MACC1	mRNA	XR_001436612.1	lncRNA
XM_012176469.2	MACC1	mRNA	XR_001436612.1	lncRNA
XM_012176469.2	MACC1	mRNA	XR_001436612.1	lncRNA
XM_012176469.2	MACC1	mRNA	XR_001436612.1	lncRNA
XM_015096221.1	PPIP5K2	mRNA	XR_001436612.1	lncRNA
XM_015096221.1	PPIP5K2	mRNA	XR_001436612.1	lncRNA
XM_015096221.1	PPIP5K2	mRNA	XR_001436612.1	lncRNA
XM_015096221.1	PPIP5K2	mRNA	XR_001436612.1	lncRNA
XM_012101006.2	CCHCR1	mRNA	XR_001436612.1	lncRNA
XM_012101006.2	CCHCR1	mRNA	XR_001436612.1	lncRNA
XM_012101006.2	CCHCR1	mRNA	XR_001436612.1	lncRNA
XM_012101006.2	CCHCR1	mRNA	XR_001436612.1	lncRNA
XM_012102930.1	GPAM	mRNA	XR_001436612.1	lncRNA
XM_012102930.1	GPAM	mRNA	XR_001436612.1	lncRNA
XM_012102930.1	GPAM	mRNA	XR_001436612.1	lncRNA
XM_012102930.1	GPAM	mRNA	XR_001436612.1	lncRNA
XM_012104222.2	DCUN1D3	mRNA	XR_001435723.1	lncRNA
XM_012104222.2	DCUN1D3	mRNA	XR_001435723.1	lncRNA
XM_012104222.2	DCUN1D3	mRNA	XR_001435723.1	lncRNA
XM_012104222.2	DCUN1D3	mRNA	XR_001435723.1	lncRNA
XM_015098983.1	C11H17orf80	mRNA	XR_001435726.1	lncRNA
XM_015098983.1	C11H17orf80	mRNA	XR_001435726.1	lncRNA
XM_015098983.1	C11H17orf80	mRNA	XR_001435726.1	lncRNA
XM_015098983.1	C11H17orf80	mRNA	XR_001435726.1	lncRNA
XM_012100082.2	WDR6	mRNA	XR_001435726.1	lncRNA
XM_012100082.2	WDR6	mRNA	XR_001435726.1	lncRNA
XM_012100082.2	WDR6	mRNA	XR_001435726.1	lncRNA
XM_012100082.2	WDR6	mRNA	XR_001435726.1	lncRNA
XM_012183459.1	ISG20L2	mRNA	XR_001435726.1	lncRNA
XM_012183459.1	ISG20L2	mRNA	XR_001435726.1	lncRNA
XM_012183459.1	ISG20L2	mRNA	XR_001435726.1	lncRNA
XM_012183459.1	ISG20L2	mRNA	XR_001435726.1	lncRNA
XM_012187364.1	ENO1	mRNA	XR_001024230.1	lncRNA
XM_012187364.1	ENO1	mRNA	XR_001024230.1	lncRNA
XM_012187364.1	ENO1	mRNA	XR_001024230.1	lncRNA
XM_012187364.1	ENO1	mRNA	XR_001024230.1	lncRNA
XM_012188419.2	LOC101108937	mRNA	XR_001435723.1	lncRNA
XM_012188419.2	LOC101108937	mRNA	XR_001435723.1	lncRNA
XM_012188419.2	LOC101108937	mRNA	XR_001435723.1	lncRNA
XM_012188419.2	LOC101108937	mRNA	XR_001435723.1	lncRNA
XM_012180708.1	PAPD4	mRNA	XR_001435723.1	lncRNA
XM_012180708.1	PAPD4	mRNA	XR_001435723.1	lncRNA
XM_012180708.1	PAPD4	mRNA	XR_001435723.1	lncRNA
XM_012180708.1	PAPD4	mRNA	XR_001435723.1	lncRNA
XM_012174289.1	ATF1	mRNA	XR_001435726.1	lncRNA
XM_012174289.1	ATF1	mRNA	XR_001435726.1	lncRNA
XM_012174289.1	ATF1	mRNA	XR_001435726.1	lncRNA
XM_012174289.1	ATF1	mRNA	XR_001435726.1	lncRNA
XM_015098983.1	C11H17orf80	mRNA	XR_001435723.1	lncRNA

XM_015098983.1	C11H17orf80	mRNA	XR_001435723.1	lncRNA
XM_015098983.1	C11H17orf80	mRNA	XR_001435723.1	lncRNA
XM_015098983.1	C11H17orf80	mRNA	XR_001435723.1	lncRNA
XM_004022228.3	LOC101122577	mRNA	XR_001435723.1	lncRNA
XM_004022228.3	LOC101122577	mRNA	XR_001435723.1	lncRNA
XM_004022228.3	LOC101122577	mRNA	XR_001435723.1	lncRNA
XM_004022228.3	LOC101122577	mRNA	XR_001435723.1	lncRNA
XM_012106815.2	RBM10	mRNA	XR_001435726.1	lncRNA
XM_012106815.2	RBM10	mRNA	XR_001435726.1	lncRNA
XM_012106815.2	RBM10	mRNA	XR_001435726.1	lncRNA
XM_012106815.2	RBM10	mRNA	XR_001435726.1	lncRNA
XM_012159714.2	KITLG	mRNA	XR_001436064.1	lncRNA
XM_012159714.2	KITLG	mRNA	XR_001436064.1	lncRNA
XM_012159714.2	KITLG	mRNA	XR_001436064.1	lncRNA
XM_012159714.2	KITLG	mRNA	XR_001436064.1	lncRNA
XM_004012167.2	GJB6	mRNA	XR_001436065.1	lncRNA
XM_004012167.2	GJB6	mRNA	XR_001436065.1	lncRNA
XM_004012167.2	GJB6	mRNA	XR_001436065.1	lncRNA
XM_004012167.2	GJB6	mRNA	XR_001436065.1	lncRNA
XM_012187364.1	ENO1	mRNA	XR_001435726.1	lncRNA
XM_012187364.1	ENO1	mRNA	XR_001435726.1	lncRNA
XM_012187364.1	ENO1	mRNA	XR_001435726.1	lncRNA
XM_012187364.1	ENO1	mRNA	XR_001435726.1	lncRNA
XM_012106234.2	OFD1	mRNA	XR_001435726.1	lncRNA
XM_012106234.2	OFD1	mRNA	XR_001435726.1	lncRNA
XM_012106234.2	OFD1	mRNA	XR_001435726.1	lncRNA
XM_012106234.2	OFD1	mRNA	XR_001435726.1	lncRNA
XM_004022228.3	LOC101122577	mRNA	XR_001436065.1	lncRNA
XM_004022228.3	LOC101122577	mRNA	XR_001436065.1	lncRNA
XM_004022228.3	LOC101122577	mRNA	XR_001436065.1	lncRNA
XM_004022228.3	LOC101122577	mRNA	XR_001436065.1	lncRNA
XM_015101000.1	RRAS2	mRNA	XR_001435723.1	lncRNA
XM_015101000.1	RRAS2	mRNA	XR_001435723.1	lncRNA
XM_015101000.1	RRAS2	mRNA	XR_001435723.1	lncRNA
XM_015101000.1	RRAS2	mRNA	XR_001435723.1	lncRNA
XM_012110487.2	UBQLN1	mRNA	XR_001436065.1	lncRNA
XM_012110487.2	UBQLN1	mRNA	XR_001436065.1	lncRNA
XM_012110487.2	UBQLN1	mRNA	XR_001436065.1	lncRNA
XM_012110487.2	UBQLN1	mRNA	XR_001436065.1	lncRNA
XM_012146477.2	ABCB6	mRNA	XR_001435726.1	lncRNA
XM_012146477.2	ABCB6	mRNA	XR_001435726.1	lncRNA
XM_012146477.2	ABCB6	mRNA	XR_001435726.1	lncRNA
XM_012146477.2	ABCB6	mRNA	XR_001435726.1	lncRNA
XM_012174290.1	ATF1	mRNA	XR_001435726.1	lncRNA
XM_012174290.1	ATF1	mRNA	XR_001435726.1	lncRNA
XM_012174290.1	ATF1	mRNA	XR_001435726.1	lncRNA
XM_012174290.1	ATF1	mRNA	XR_001435726.1	lncRNA
XM_004014588.3	PKIG	mRNA	XR_001435726.1	lncRNA
XM_004014588.3	PKIG	mRNA	XR_001435726.1	lncRNA
XM_004014588.3	PKIG	mRNA	XR_001435726.1	lncRNA
XM_004014588.3	PKIG	mRNA	XR_001435726.1	lncRNA
XM_012188419.2	LOC101108937	mRNA	XR_001024230.1	lncRNA
XM_012188419.2	LOC101108937	mRNA	XR_001024230.1	lncRNA
XM_012188419.2	LOC101108937	mRNA	XR_001024230.1	lncRNA

XM_012188419.2	LOC101108937	mRNA	XR_001024230.1	lncRNA
XM_012177185.2	CNOT4	mRNA	XR_001435723.1	lncRNA
XM_012177185.2	CNOT4	mRNA	XR_001435723.1	lncRNA
XM_012177185.2	CNOT4	mRNA	XR_001435723.1	lncRNA
XM_012177185.2	CNOT4	mRNA	XR_001435723.1	lncRNA
XM_004012167.2	GJB6	mRNA	XR_001435726.1	lncRNA
XM_004012167.2	GJB6	mRNA	XR_001435726.1	lncRNA
XM_004012167.2	GJB6	mRNA	XR_001435726.1	lncRNA
XM_004012167.2	GJB6	mRNA	XR_001435726.1	lncRNA
XM_012102667.1	ZNF518A	mRNA	XR_001435726.1	lncRNA
XM_012102667.1	ZNF518A	mRNA	XR_001435726.1	lncRNA
XM_012102667.1	ZNF518A	mRNA	XR_001435726.1	lncRNA
XM_012102667.1	ZNF518A	mRNA	XR_001435726.1	lncRNA
XM_012099659.2	FOXP1	mRNA	XR_001435723.1	lncRNA
XM_012099659.2	FOXP1	mRNA	XR_001435723.1	lncRNA
XM_012099659.2	FOXP1	mRNA	XR_001435723.1	lncRNA
XM_012099659.2	FOXP1	mRNA	XR_001435723.1	lncRNA
XM_015098987.1	ABCA5	mRNA	XR_001435726.1	lncRNA
XM_015098987.1	ABCA5	mRNA	XR_001435726.1	lncRNA
XM_015098987.1	ABCA5	mRNA	XR_001435726.1	lncRNA
XM_015098987.1	ABCA5	mRNA	XR_001435726.1	lncRNA
XM_012180708.1	PAPD4	mRNA	XR_001435726.1	lncRNA
XM_012180708.1	PAPD4	mRNA	XR_001435726.1	lncRNA
XM_012180708.1	PAPD4	mRNA	XR_001435726.1	lncRNA
XM_012180708.1	PAPD4	mRNA	XR_001435726.1	lncRNA
XM_012104222.2	DCUN1D3	mRNA	XR_001435726.1	lncRNA
XM_012104222.2	DCUN1D3	mRNA	XR_001435726.1	lncRNA
XM_012104222.2	DCUN1D3	mRNA	XR_001435726.1	lncRNA
XM_012104222.2	DCUN1D3	mRNA	XR_001435726.1	lncRNA
XM_015101000.1	RRAS2	mRNA	XR_001435726.1	lncRNA
XM_015101000.1	RRAS2	mRNA	XR_001435726.1	lncRNA
XM_015101000.1	RRAS2	mRNA	XR_001435726.1	lncRNA
XM_015101000.1	RRAS2	mRNA	XR_001435726.1	lncRNA
XM_012184401.1	ZC3H13	mRNA	XR_001435723.1	lncRNA
XM_012184401.1	ZC3H13	mRNA	XR_001435723.1	lncRNA
XM_012184401.1	ZC3H13	mRNA	XR_001435723.1	lncRNA
XM_012184401.1	ZC3H13	mRNA	XR_001435723.1	lncRNA
XM_015095024.1	JOSD1	mRNA	XR_001435723.1	lncRNA
XM_015095024.1	JOSD1	mRNA	XR_001435723.1	lncRNA
XM_015095024.1	JOSD1	mRNA	XR_001435723.1	lncRNA
XM_015095024.1	JOSD1	mRNA	XR_001435723.1	lncRNA
XM_012180708.1	PAPD4	mRNA	XR_001436065.1	lncRNA
XM_012180708.1	PAPD4	mRNA	XR_001436065.1	lncRNA
XM_012180708.1	PAPD4	mRNA	XR_001436065.1	lncRNA
XM_012180708.1	PAPD4	mRNA	XR_001436065.1	lncRNA
XM_012179573.2	PPA2	mRNA	XR_001436064.1	lncRNA
XM_012179573.2	PPA2	mRNA	XR_001436064.1	lncRNA
XM_012179573.2	PPA2	mRNA	XR_001436064.1	lncRNA
XM_012179573.2	PPA2	mRNA	XR_001436064.1	lncRNA
XM_004012167.2	GJB6	mRNA	XR_001436064.1	lncRNA
XM_004012167.2	GJB6	mRNA	XR_001436064.1	lncRNA
XM_004012167.2	GJB6	mRNA	XR_001436064.1	lncRNA
XM_004012167.2	GJB6	mRNA	XR_001436064.1	lncRNA
XM_004012167.2	GJB6	mRNA	XR_001435723.1	lncRNA

XM_004012167.2	GJB6	mRNA	XR_001435723.1	lncRNA
XM_004012167.2	GJB6	mRNA	XR_001435723.1	lncRNA
XM_004012167.2	GJB6	mRNA	XR_001435723.1	lncRNA
XM_012190349.2	ZNF420	mRNA	XR_001436065.1	lncRNA
XM_012190349.2	ZNF420	mRNA	XR_001436065.1	lncRNA
XM_012190349.2	ZNF420	mRNA	XR_001436065.1	lncRNA
XM_012190349.2	ZNF420	mRNA	XR_001436065.1	lncRNA
XM_012102667.1	ZNF518A	mRNA	XR_001435723.1	lncRNA
XM_012102667.1	ZNF518A	mRNA	XR_001435723.1	lncRNA
XM_012102667.1	ZNF518A	mRNA	XR_001435723.1	lncRNA
XM_012102667.1	ZNF518A	mRNA	XR_001435723.1	lncRNA
XM_012177185.2	CNOT4	mRNA	XR_001436064.1	lncRNA
XM_012177185.2	CNOT4	mRNA	XR_001436064.1	lncRNA
XM_012177185.2	CNOT4	mRNA	XR_001436064.1	lncRNA
XM_012177185.2	CNOT4	mRNA	XR_001436064.1	lncRNA
XM_004020479.3	RBBP8	mRNA	XR_001024230.1	lncRNA
XM_004020479.3	RBBP8	mRNA	XR_001024230.1	lncRNA
XM_004020479.3	RBBP8	mRNA	XR_001024230.1	lncRNA
XM_004020479.3	RBBP8	mRNA	XR_001024230.1	lncRNA
XM_012177185.2	CNOT4	mRNA	XR_001024230.1	lncRNA
XM_012177185.2	CNOT4	mRNA	XR_001024230.1	lncRNA
XM_012177185.2	CNOT4	mRNA	XR_001024230.1	lncRNA
XM_012177185.2	CNOT4	mRNA	XR_001024230.1	lncRNA
XM_012099659.2	FOXP1	mRNA	XR_001024230.1	lncRNA
XM_012099659.2	FOXP1	mRNA	XR_001024230.1	lncRNA
XM_012099659.2	FOXP1	mRNA	XR_001024230.1	lncRNA
XM_012099659.2	FOXP1	mRNA	XR_001024230.1	lncRNA
XM_012102667.1	ZNF518A	mRNA	XR_001436064.1	lncRNA
XM_012102667.1	ZNF518A	mRNA	XR_001436064.1	lncRNA
XM_012102667.1	ZNF518A	mRNA	XR_001436064.1	lncRNA
XM_012102667.1	ZNF518A	mRNA	XR_001436064.1	lncRNA
XM_012110487.2	UBQLN1	mRNA	XR_001436064.1	lncRNA
XM_012110487.2	UBQLN1	mRNA	XR_001436064.1	lncRNA
XM_012110487.2	UBQLN1	mRNA	XR_001436064.1	lncRNA
XM_012110487.2	UBQLN1	mRNA	XR_001436064.1	lncRNA
XM_004013732.3	KIF1B	mRNA	XR_001436065.1	lncRNA
XM_004013732.3	KIF1B	mRNA	XR_001436065.1	lncRNA
XM_004013732.3	KIF1B	mRNA	XR_001436065.1	lncRNA
XM_004013732.3	KIF1B	mRNA	XR_001436065.1	lncRNA
XM_012104507.1	CUX1	mRNA	XR_001436064.1	lncRNA
XM_012104507.1	CUX1	mRNA	XR_001436064.1	lncRNA
XM_012104507.1	CUX1	mRNA	XR_001436064.1	lncRNA
XM_012104507.1	CUX1	mRNA	XR_001436064.1	lncRNA
XM_015096209.1	SPATA9	mRNA	XR_001435726.1	lncRNA
XM_015096209.1	SPATA9	mRNA	XR_001435726.1	lncRNA
XM_015096209.1	SPATA9	mRNA	XR_001435726.1	lncRNA
XM_015096209.1	SPATA9	mRNA	XR_001435726.1	lncRNA
XM_004022228.3	LOC101122577	mRNA	XR_001024230.1	lncRNA
XM_004022228.3	LOC101122577	mRNA	XR_001024230.1	lncRNA
XM_004022228.3	LOC101122577	mRNA	XR_001024230.1	lncRNA
XM_004022228.3	LOC101122577	mRNA	XR_001024230.1	lncRNA
XM_012188419.2	LOC101108937	mRNA	XR_001435726.1	lncRNA
XM_012188419.2	LOC101108937	mRNA	XR_001435726.1	lncRNA
XM_012188419.2	LOC101108937	mRNA	XR_001435726.1	lncRNA

XM_012188419.2	LOC101108937	mRNA	XR_001435726.1	lncRNA
XM_015098987.1	ABCA5	mRNA	XR_001435723.1	lncRNA
XM_015098987.1	ABCA5	mRNA	XR_001435723.1	lncRNA
XM_015098987.1	ABCA5	mRNA	XR_001435723.1	lncRNA
XM_015098987.1	ABCA5	mRNA	XR_001435723.1	lncRNA
XM_004022228.3	LOC101122577	mRNA	XR_001436064.1	lncRNA
XM_004022228.3	LOC101122577	mRNA	XR_001436064.1	lncRNA
XM_004022228.3	LOC101122577	mRNA	XR_001436064.1	lncRNA
XM_004022228.3	LOC101122577	mRNA	XR_001436064.1	lncRNA
XM_012102667.1	ZNF518A	mRNA	XR_001024230.1	lncRNA
XM_012102667.1	ZNF518A	mRNA	XR_001024230.1	lncRNA
XM_012102667.1	ZNF518A	mRNA	XR_001024230.1	lncRNA
XM_012102667.1	ZNF518A	mRNA	XR_001024230.1	lncRNA
XM_012187364.1	ENO1	mRNA	XR_001435723.1	lncRNA
XM_012187364.1	ENO1	mRNA	XR_001435723.1	lncRNA
XM_012187364.1	ENO1	mRNA	XR_001435723.1	lncRNA
XM_012187364.1	ENO1	mRNA	XR_001435723.1	lncRNA
XM_012174876.2	GAS2L3	mRNA	XR_001436065.1	lncRNA
XM_012174876.2	GAS2L3	mRNA	XR_001436065.1	lncRNA
XM_012174876.2	GAS2L3	mRNA	XR_001436065.1	lncRNA
XM_012174876.2	GAS2L3	mRNA	XR_001436065.1	lncRNA
XM_012174289.1	ATF1	mRNA	XR_001435723.1	lncRNA
XM_012174289.1	ATF1	mRNA	XR_001435723.1	lncRNA
XM_012174289.1	ATF1	mRNA	XR_001435723.1	lncRNA
XM_012174289.1	ATF1	mRNA	XR_001435723.1	lncRNA
XM_012106234.2	OFD1	mRNA	XR_001435723.1	lncRNA
XM_012106234.2	OFD1	mRNA	XR_001435723.1	lncRNA
XM_012106234.2	OFD1	mRNA	XR_001435723.1	lncRNA
XM_012106234.2	OFD1	mRNA	XR_001435723.1	lncRNA
XM_012104222.2	DCUN1D3	mRNA	XR_001024230.1	lncRNA
XM_012104222.2	DCUN1D3	mRNA	XR_001024230.1	lncRNA
XM_012104222.2	DCUN1D3	mRNA	XR_001024230.1	lncRNA
XM_012104222.2	DCUN1D3	mRNA	XR_001024230.1	lncRNA
XM_012143523.2	INO80D	mRNA	XR_001024290.1	lncRNA
XM_012143523.2	INO80D	mRNA	XR_001024290.1	lncRNA
XM_012143523.2	INO80D	mRNA	XR_001024290.1	lncRNA
XM_012143523.2	INO80D	mRNA	XR_001024290.1	lncRNA
XM_012176761.2	MDFIC	mRNA	XR_001433837.1	lncRNA
XM_012176761.2	MDFIC	mRNA	XR_001433837.1	lncRNA
XM_012176761.2	MDFIC	mRNA	XR_001433837.1	lncRNA
XM_012176761.2	MDFIC	mRNA	XR_001433837.1	lncRNA
XM_015100095.1	LOC101113523	mRNA	XR_001433837.1	lncRNA
XM_015100095.1	LOC101113523	mRNA	XR_001433837.1	lncRNA
XM_015100095.1	LOC101113523	mRNA	XR_001433837.1	lncRNA
XM_015100095.1	LOC101113523	mRNA	XR_001433837.1	lncRNA
XM_015096931.1	THBS1	mRNA	XR_001433837.1	lncRNA
XM_015096931.1	THBS1	mRNA	XR_001433837.1	lncRNA
XM_015096931.1	THBS1	mRNA	XR_001433837.1	lncRNA
XM_015096931.1	THBS1	mRNA	XR_001433837.1	lncRNA
XM_012173383.2	DHX57	mRNA	XR_001023303.2	lncRNA
XM_012173383.2	DHX57	mRNA	XR_001023303.2	lncRNA
XM_012173383.2	DHX57	mRNA	XR_001023303.2	lncRNA
XM_012173383.2	DHX57	mRNA	XR_001023303.2	lncRNA
XM_012099341.2	CLASP2	mRNA	XR_001041893.2	lncRNA

XM_012099341.2	CLASP2	mRNA	XR_001041893.2	lncRNA
XM_012099341.2	CLASP2	mRNA	XR_001041893.2	lncRNA
XM_012099341.2	CLASP2	mRNA	XR_001041893.2	lncRNA
XM_012146540.1	ATG9A	mRNA	XR_001023303.2	lncRNA
XM_012146540.1	ATG9A	mRNA	XR_001023303.2	lncRNA
XM_012146540.1	ATG9A	mRNA	XR_001023303.2	lncRNA
XM_012146540.1	ATG9A	mRNA	XR_001023303.2	lncRNA
XM_012106946.2	ATP7A	mRNA	XR_001023303.2	lncRNA
XM_012106946.2	ATP7A	mRNA	XR_001023303.2	lncRNA
XM_012106946.2	ATP7A	mRNA	XR_001023303.2	lncRNA
XM_012106946.2	ATP7A	mRNA	XR_001023303.2	lncRNA
XM_012173383.2	DHX57	mRNA	XR_001024966.2	lncRNA
XM_012173383.2	DHX57	mRNA	XR_001024966.2	lncRNA
XM_012173383.2	DHX57	mRNA	XR_001024966.2	lncRNA
XM_012173383.2	DHX57	mRNA	XR_001024966.2	lncRNA
XM_012146540.1	ATG9A	mRNA	XR_001024966.2	lncRNA
XM_012146540.1	ATG9A	mRNA	XR_001024966.2	lncRNA
XM_012146540.1	ATG9A	mRNA	XR_001024966.2	lncRNA
XM_012146540.1	ATG9A	mRNA	XR_001024966.2	lncRNA
XM_012175120.1	PPFIBP1	mRNA	XR_001023303.2	lncRNA
XM_012175120.1	PPFIBP1	mRNA	XR_001023303.2	lncRNA
XM_012175120.1	PPFIBP1	mRNA	XR_001023303.2	lncRNA
XM_012175120.1	PPFIBP1	mRNA	XR_001023303.2	lncRNA
XM_015095425.1	CUL1	mRNA	XR_001023303.2	lncRNA
XM_015095425.1	CUL1	mRNA	XR_001023303.2	lncRNA
XM_015095425.1	CUL1	mRNA	XR_001023303.2	lncRNA
XM_015095425.1	CUL1	mRNA	XR_001023303.2	lncRNA
XM_015099976.1	ZFP30	mRNA	XR_001023303.2	lncRNA
XM_015099976.1	ZFP30	mRNA	XR_001023303.2	lncRNA
XM_015099976.1	ZFP30	mRNA	XR_001023303.2	lncRNA
XM_015099976.1	ZFP30	mRNA	XR_001023303.2	lncRNA
XM_012183409.2	PHF3	mRNA	XR_001023303.2	lncRNA
XM_012183409.2	PHF3	mRNA	XR_001023303.2	lncRNA
XM_012183409.2	PHF3	mRNA	XR_001023303.2	lncRNA
XM_012183409.2	PHF3	mRNA	XR_001023303.2	lncRNA
XM_015091887.1	MYCL	mRNA	XR_001023303.2	lncRNA
XM_015091887.1	MYCL	mRNA	XR_001023303.2	lncRNA
XM_015091887.1	MYCL	mRNA	XR_001023303.2	lncRNA
XM_015091887.1	MYCL	mRNA	XR_001023303.2	lncRNA
XM_012190075.2	PHLPP2	mRNA	XR_001023303.2	lncRNA
XM_012190075.2	PHLPP2	mRNA	XR_001023303.2	lncRNA
XM_012190075.2	PHLPP2	mRNA	XR_001023303.2	lncRNA
XM_012190075.2	PHLPP2	mRNA	XR_001023303.2	lncRNA
XM_012124912.1	KIAA2026	mRNA	XR_001024966.2	lncRNA
XM_012124912.1	KIAA2026	mRNA	XR_001024966.2	lncRNA
XM_012124912.1	KIAA2026	mRNA	XR_001024966.2	lncRNA
XM_012124912.1	KIAA2026	mRNA	XR_001024966.2	lncRNA
XM_012178221.1	CEP120	mRNA	XR_001023303.2	lncRNA
XM_012178221.1	CEP120	mRNA	XR_001023303.2	lncRNA
XM_012178221.1	CEP120	mRNA	XR_001023303.2	lncRNA
XM_012178221.1	CEP120	mRNA	XR_001023303.2	lncRNA
XM_012186029.1	FBXL20	mRNA	XR_001023627.2	lncRNA
XM_012186029.1	FBXL20	mRNA	XR_001023627.2	lncRNA
XM_012186029.1	FBXL20	mRNA	XR_001023627.2	lncRNA

XM_012186029.1	FBXL20	mRNA	XR_001023627.2	lncRNA
XM_012102930.1	GPAM	mRNA	XR_001023627.2	lncRNA
XM_012102930.1	GPAM	mRNA	XR_001023627.2	lncRNA
XM_012102930.1	GPAM	mRNA	XR_001023627.2	lncRNA
XM_012102930.1	GPAM	mRNA	XR_001023627.2	lncRNA
XM_012174423.2	SLC38A4	mRNA	XR_001023627.2	lncRNA
XM_012174423.2	SLC38A4	mRNA	XR_001023627.2	lncRNA
XM_012174423.2	SLC38A4	mRNA	XR_001023627.2	lncRNA
XM_012174423.2	SLC38A4	mRNA	XR_001023627.2	lncRNA
XM_012098071.2	RNF185	mRNA	XR_001434127.1	lncRNA
XM_012098071.2	RNF185	mRNA	XR_001434127.1	lncRNA
XM_012098071.2	RNF185	mRNA	XR_001434127.1	lncRNA
XM_012098071.2	RNF185	mRNA	XR_001434127.1	lncRNA
XM_015097749.1	PCMTD1	mRNA	TCONS_00049802	lncRNA
XM_015097749.1	PCMTD1	mRNA	TCONS_00049802	lncRNA
XM_015097749.1	PCMTD1	mRNA	TCONS_00049802	lncRNA
XM_015097749.1	PCMTD1	mRNA	TCONS_00049802	lncRNA
XM_015097749.1	PCMTD1	mRNA	XR_001434127.1	lncRNA
XM_015097749.1	PCMTD1	mRNA	XR_001434127.1	lncRNA
XM_015097749.1	PCMTD1	mRNA	XR_001434127.1	lncRNA
XM_015097749.1	PCMTD1	mRNA	XR_001434127.1	lncRNA
XM_015103688.1	PTPN2	mRNA	TCONS_00049802	lncRNA
XM_015103688.1	PTPN2	mRNA	TCONS_00049802	lncRNA
XM_015103688.1	PTPN2	mRNA	TCONS_00049802	lncRNA
XM_015103688.1	PTPN2	mRNA	TCONS_00049802	lncRNA
XM_012106112.2	ARAF	mRNA	TCONS_00049802	lncRNA
XM_012106112.2	ARAF	mRNA	TCONS_00049802	lncRNA
XM_012106112.2	ARAF	mRNA	TCONS_00049802	lncRNA
XM_012106112.2	ARAF	mRNA	TCONS_00049802	lncRNA
XM_015103688.1	PTPN2	mRNA	XR_001434127.1	lncRNA
XM_015103688.1	PTPN2	mRNA	XR_001434127.1	lncRNA
XM_015103688.1	PTPN2	mRNA	XR_001434127.1	lncRNA
XM_015103688.1	PTPN2	mRNA	XR_001434127.1	lncRNA
XM_012174423.2	SLC38A4	mRNA	XR_001434127.1	lncRNA
XM_012174423.2	SLC38A4	mRNA	XR_001434127.1	lncRNA
XM_012174423.2	SLC38A4	mRNA	XR_001434127.1	lncRNA
XM_012174423.2	SLC38A4	mRNA	XR_001434127.1	lncRNA
XM_015095675.1	LOC106990607	mRNA	XR_001023627.2	lncRNA
XM_015095675.1	LOC106990607	mRNA	XR_001023627.2	lncRNA
XM_015095675.1	LOC106990607	mRNA	XR_001023627.2	lncRNA
XM_015095675.1	LOC106990607	mRNA	XR_001023627.2	lncRNA
XM_015101559.1	DDX51	mRNA	XR_001434127.1	lncRNA
XM_015101559.1	DDX51	mRNA	XR_001434127.1	lncRNA
XM_015101559.1	DDX51	mRNA	XR_001434127.1	lncRNA
XM_015101559.1	DDX51	mRNA	XR_001434127.1	lncRNA
XM_012179413.2	PDE5A	mRNA	TCONS_00049802	lncRNA
XM_012179413.2	PDE5A	mRNA	TCONS_00049802	lncRNA
XM_012179413.2	PDE5A	mRNA	TCONS_00049802	lncRNA
XM_012179413.2	PDE5A	mRNA	TCONS_00049802	lncRNA
XM_012101050.1	ZNF311	mRNA	XR_001023627.2	lncRNA
XM_012101050.1	ZNF311	mRNA	XR_001023627.2	lncRNA
XM_012101050.1	ZNF311	mRNA	XR_001023627.2	lncRNA
XM_012101050.1	ZNF311	mRNA	XR_001023627.2	lncRNA
XM_012176469.2	MACC1	mRNA	XR_001023627.2	lncRNA

XM_012176469.2	MACC1	mRNA	XR_001023627.2	lncRNA
XM_012176469.2	MACC1	mRNA	XR_001023627.2	lncRNA
XM_012176469.2	MACC1	mRNA	XR_001023627.2	lncRNA
XM_015094919.1	WBP11	mRNA	TCONS_00049802	lncRNA
XM_015094919.1	WBP11	mRNA	TCONS_00049802	lncRNA
XM_015094919.1	WBP11	mRNA	TCONS_00049802	lncRNA
XM_015094919.1	WBP11	mRNA	TCONS_00049802	lncRNA
XM_015092364.1	GART	mRNA	XR_001025540.2	lncRNA
XM_015092364.1	GART	mRNA	XR_001025540.2	lncRNA
XM_015092364.1	GART	mRNA	XR_001025540.2	lncRNA
XM_015092364.1	GART	mRNA	XR_001025540.2	lncRNA
XM_012184885.2	DOCK9	mRNA	XR_001025540.2	lncRNA
XM_012184885.2	DOCK9	mRNA	XR_001025540.2	lncRNA
XM_012184885.2	DOCK9	mRNA	XR_001025540.2	lncRNA
XM_012184885.2	DOCK9	mRNA	XR_001025540.2	lncRNA
XM_015100523.1	NFAT5	mRNA	XR_001025540.2	lncRNA
XM_015100523.1	NFAT5	mRNA	XR_001025540.2	lncRNA
XM_015100523.1	NFAT5	mRNA	XR_001025540.2	lncRNA
XM_015100523.1	NFAT5	mRNA	XR_001025540.2	lncRNA
XM_015093065.1	FAM219A	mRNA	XR_001025540.2	lncRNA
XM_015093065.1	FAM219A	mRNA	XR_001025540.2	lncRNA
XM_015093065.1	FAM219A	mRNA	XR_001025540.2	lncRNA
XM_015093065.1	FAM219A	mRNA	XR_001025540.2	lncRNA
XM_015094225.1	ROCK2	mRNA	XR_001025540.2	lncRNA
XM_015094225.1	ROCK2	mRNA	XR_001025540.2	lncRNA
XM_015094225.1	ROCK2	mRNA	XR_001025540.2	lncRNA
XM_015094225.1	ROCK2	mRNA	XR_001025540.2	lncRNA
XM_004018866.3	LOC101120701	mRNA	XR_001022883.1	lncRNA
XM_004018866.3	LOC101120701	mRNA	XR_001022883.1	lncRNA
XM_004018866.3	LOC101120701	mRNA	XR_001022883.1	lncRNA
XM_004014588.3	PKIG	mRNA	XR_001434920.1	lncRNA
XM_004014588.3	PKIG	mRNA	XR_001434920.1	lncRNA
XM_004014588.3	PKIG	mRNA	XR_001434920.1	lncRNA
XM_004014588.3	PKIG	mRNA	XR_001434920.1	lncRNA
XM_012106234.2	OFD1	mRNA	XR_001435722.1	lncRNA
XM_012106234.2	OFD1	mRNA	XR_001435722.1	lncRNA
XM_012106234.2	OFD1	mRNA	XR_001435722.1	lncRNA
XM_012106234.2	OFD1	mRNA	XR_001435722.1	lncRNA
XM_004008085.3	CNOT4	mRNA	XR_001434920.1	lncRNA
XM_004008085.3	CNOT4	mRNA	XR_001434920.1	lncRNA
XM_004008085.3	CNOT4	mRNA	XR_001434920.1	lncRNA
XM_004008085.3	CNOT4	mRNA	XR_001434920.1	lncRNA
XM_012105014.1	LGALS8	mRNA	XR_001435722.1	lncRNA
XM_012105014.1	LGALS8	mRNA	XR_001435722.1	lncRNA
XM_012105014.1	LGALS8	mRNA	XR_001435722.1	lncRNA
XM_012105014.1	LGALS8	mRNA	XR_001435722.1	lncRNA
XM_012182520.2	SLC35F1	mRNA	XR_001435107.1	lncRNA
XM_012182520.2	SLC35F1	mRNA	XR_001435107.1	lncRNA
XM_012182520.2	SLC35F1	mRNA	XR_001435107.1	lncRNA
XM_012182520.2	SLC35F1	mRNA	XR_001435107.1	lncRNA
XM_012159735.2	KITLG	mRNA	XR_001435107.1	lncRNA
XM_012159735.2	KITLG	mRNA	XR_001435107.1	lncRNA
XM_012159735.2	KITLG	mRNA	XR_001435107.1	lncRNA
XM_012159735.2	KITLG	mRNA	XR_001435107.1	lncRNA

XM_015093726.1	HP1BP3	mRNA	XR_001435722.1	lncRNA
XM_015093726.1	HP1BP3	mRNA	XR_001435722.1	lncRNA
XM_015093726.1	HP1BP3	mRNA	XR_001435722.1	lncRNA
XM_015093726.1	HP1BP3	mRNA	XR_001435722.1	lncRNA
XM_015101000.1	RRAS2	mRNA	XR_001435722.1	lncRNA
XM_015101000.1	RRAS2	mRNA	XR_001435722.1	lncRNA
XM_015101000.1	RRAS2	mRNA	XR_001435722.1	lncRNA
XM_015101000.1	RRAS2	mRNA	XR_001435722.1	lncRNA
XM_015094800.1	RAB5B	mRNA	XR_001435107.1	lncRNA
XM_015094800.1	RAB5B	mRNA	XR_001435107.1	lncRNA
XM_015094800.1	RAB5B	mRNA	XR_001435107.1	lncRNA
XM_015094800.1	RAB5B	mRNA	XR_001435107.1	lncRNA
XM_012159735.2	KITLG	mRNA	XR_001435722.1	lncRNA
XM_012159735.2	KITLG	mRNA	XR_001435722.1	lncRNA
XM_012159735.2	KITLG	mRNA	XR_001435722.1	lncRNA
XM_012159735.2	KITLG	mRNA	XR_001435722.1	lncRNA
XM_012106815.2	RBM10	mRNA	XR_001434920.1	lncRNA
XM_012106815.2	RBM10	mRNA	XR_001434920.1	lncRNA
XM_012106815.2	RBM10	mRNA	XR_001434920.1	lncRNA
XM_012106815.2	RBM10	mRNA	XR_001434920.1	lncRNA
XM_012105556.2	ZFAND4	mRNA	XR_001435107.1	lncRNA
XM_012105556.2	ZFAND4	mRNA	XR_001435107.1	lncRNA
XM_012105556.2	ZFAND4	mRNA	XR_001435107.1	lncRNA
XM_012105556.2	ZFAND4	mRNA	XR_001435107.1	lncRNA
XM_012104232.2	DCUN1D3	mRNA	XR_001435722.1	lncRNA
XM_012104232.2	DCUN1D3	mRNA	XR_001435722.1	lncRNA
XM_012104232.2	DCUN1D3	mRNA	XR_001435722.1	lncRNA
XM_012104232.2	DCUN1D3	mRNA	XR_001435722.1	lncRNA
XM_012183459.1	ISG20L2	mRNA	XR_001435107.1	lncRNA
XM_012183459.1	ISG20L2	mRNA	XR_001435107.1	lncRNA
XM_012183459.1	ISG20L2	mRNA	XR_001435107.1	lncRNA
XM_012183459.1	ISG20L2	mRNA	XR_001435107.1	lncRNA
XM_012104232.2	DCUN1D3	mRNA	XR_001435107.1	lncRNA
XM_012104232.2	DCUN1D3	mRNA	XR_001435107.1	lncRNA
XM_012104232.2	DCUN1D3	mRNA	XR_001435107.1	lncRNA
XM_012104232.2	DCUN1D3	mRNA	XR_001435107.1	lncRNA
XM_015092648.1	ARMC8	mRNA	XR_001435722.1	lncRNA
XM_015092648.1	ARMC8	mRNA	XR_001435722.1	lncRNA
XM_015092648.1	ARMC8	mRNA	XR_001435722.1	lncRNA
XM_015092648.1	ARMC8	mRNA	XR_001435722.1	lncRNA
XM_012183459.1	ISG20L2	mRNA	XR_001435722.1	lncRNA
XM_012183459.1	ISG20L2	mRNA	XR_001435722.1	lncRNA
XM_012183459.1	ISG20L2	mRNA	XR_001435722.1	lncRNA
XM_012183459.1	ISG20L2	mRNA	XR_001435722.1	lncRNA
XM_012104222.2	DCUN1D3	mRNA	XR_001435722.1	lncRNA
XM_012104222.2	DCUN1D3	mRNA	XR_001435722.1	lncRNA
XM_012104222.2	DCUN1D3	mRNA	XR_001435722.1	lncRNA
XM_012104222.2	DCUN1D3	mRNA	XR_001435722.1	lncRNA
XM_012099221.2	SLC25A26	mRNA	XR_001435722.1	lncRNA
XM_012099221.2	SLC25A26	mRNA	XR_001435722.1	lncRNA
XM_012099221.2	SLC25A26	mRNA	XR_001435722.1	lncRNA
XM_012099221.2	SLC25A26	mRNA	XR_001435722.1	lncRNA
XM_012099693.2	EOGT	mRNA	XR_001435107.1	lncRNA
XM_012099693.2	EOGT	mRNA	XR_001435107.1	lncRNA

XM_012099693.2	EOGT	mRNA	XR_001435107.1	lncRNA
XM_012099693.2	EOGT	mRNA	XR_001435107.1	lncRNA
XM_012099221.2	SLC25A26	mRNA	XR_001433856.1	lncRNA
XM_012099221.2	SLC25A26	mRNA	XR_001433856.1	lncRNA
XM_012099221.2	SLC25A26	mRNA	XR_001433856.1	lncRNA
XM_012099221.2	SLC25A26	mRNA	XR_001433856.1	lncRNA
XM_012112438.2	PPIH	mRNA	XR_001433856.1	lncRNA
XM_012112438.2	PPIH	mRNA	XR_001433856.1	lncRNA
XM_012112438.2	PPIH	mRNA	XR_001433856.1	lncRNA
XM_012112438.2	PPIH	mRNA	XR_001433856.1	lncRNA
XM_015098987.1	ABCA5	mRNA	XR_001433856.1	lncRNA
XM_015098987.1	ABCA5	mRNA	XR_001433856.1	lncRNA
XM_015098987.1	ABCA5	mRNA	XR_001433856.1	lncRNA
XM_015098987.1	ABCA5	mRNA	XR_001433856.1	lncRNA
XM_004008085.3	CNOT4	mRNA	XR_001433856.1	lncRNA
XM_004008085.3	CNOT4	mRNA	XR_001433856.1	lncRNA
XM_004008085.3	CNOT4	mRNA	XR_001433856.1	lncRNA
XM_004008085.3	CNOT4	mRNA	XR_001433856.1	lncRNA
XM_012106234.2	OFD1	mRNA	XR_001436817.1	lncRNA
XM_012106234.2	OFD1	mRNA	XR_001436817.1	lncRNA
XM_012106234.2	OFD1	mRNA	XR_001436817.1	lncRNA
XM_012106234.2	OFD1	mRNA	XR_001436817.1	lncRNA
XM_012184401.1	ZC3H13	mRNA	XR_001433856.1	lncRNA
XM_012184401.1	ZC3H13	mRNA	XR_001433856.1	lncRNA
XM_012184401.1	ZC3H13	mRNA	XR_001433856.1	lncRNA
XM_012184401.1	ZC3H13	mRNA	XR_001433856.1	lncRNA
XM_012104507.1	CUX1	mRNA	XR_001433856.1	lncRNA
XM_012104507.1	CUX1	mRNA	XR_001433856.1	lncRNA
XM_012104507.1	CUX1	mRNA	XR_001433856.1	lncRNA
XM_012104507.1	CUX1	mRNA	XR_001433856.1	lncRNA
XM_015095024.1	JOSD1	mRNA	XR_001433856.1	lncRNA
XM_015095024.1	JOSD1	mRNA	XR_001433856.1	lncRNA
XM_015095024.1	JOSD1	mRNA	XR_001433856.1	lncRNA
XM_015095024.1	JOSD1	mRNA	XR_001433856.1	lncRNA
XM_012182520.2	SLC35F1	mRNA	XR_001433856.1	lncRNA
XM_012182520.2	SLC35F1	mRNA	XR_001433856.1	lncRNA
XM_012182520.2	SLC35F1	mRNA	XR_001433856.1	lncRNA
XM_012182520.2	SLC35F1	mRNA	XR_001433856.1	lncRNA
XM_012105556.2	ZFAND4	mRNA	XR_001433856.1	lncRNA
XM_012105556.2	ZFAND4	mRNA	XR_001433856.1	lncRNA
XM_012105556.2	ZFAND4	mRNA	XR_001433856.1	lncRNA
XM_012105556.2	ZFAND4	mRNA	XR_001433856.1	lncRNA
XM_012106815.2	RBM10	mRNA	XR_001435722.1	lncRNA
XM_012106815.2	RBM10	mRNA	XR_001435722.1	lncRNA
XM_012106815.2	RBM10	mRNA	XR_001435722.1	lncRNA
XM_012106815.2	RBM10	mRNA	XR_001435722.1	lncRNA
XM_012106234.2	OFD1	mRNA	XR_001433856.1	lncRNA
XM_012106234.2	OFD1	mRNA	XR_001433856.1	lncRNA
XM_012106234.2	OFD1	mRNA	XR_001433856.1	lncRNA
XM_012106234.2	OFD1	mRNA	XR_001433856.1	lncRNA
XM_012102598.2	TBC1D12	mRNA	XR_001435722.1	lncRNA
XM_012102598.2	TBC1D12	mRNA	XR_001435722.1	lncRNA
XM_012102598.2	TBC1D12	mRNA	XR_001435722.1	lncRNA
XM_012102598.2	TBC1D12	mRNA	XR_001435722.1	lncRNA

XM_004008085.3	CNOT4	mRNA	XR_001436817.1	lncRNA
XM_004008085.3	CNOT4	mRNA	XR_001436817.1	lncRNA
XM_004008085.3	CNOT4	mRNA	XR_001436817.1	lncRNA
XM_004008085.3	CNOT4	mRNA	XR_001436817.1	lncRNA
XM_012106815.2	RBM10	mRNA	XR_001435107.1	lncRNA
XM_012106815.2	RBM10	mRNA	XR_001435107.1	lncRNA
XM_012106815.2	RBM10	mRNA	XR_001435107.1	lncRNA
XM_012106815.2	RBM10	mRNA	XR_001435107.1	lncRNA
XM_004012167.2	GJB6	mRNA	XR_001436817.1	lncRNA
XM_004012167.2	GJB6	mRNA	XR_001436817.1	lncRNA
XM_004012167.2	GJB6	mRNA	XR_001436817.1	lncRNA
XM_004012167.2	GJB6	mRNA	XR_001436817.1	lncRNA
XM_015098987.1	ABCA5	mRNA	XR_001436817.1	lncRNA
XM_015098987.1	ABCA5	mRNA	XR_001436817.1	lncRNA
XM_015098987.1	ABCA5	mRNA	XR_001436817.1	lncRNA
XM_015098987.1	ABCA5	mRNA	XR_001436817.1	lncRNA
XM_012104222.2	DCUN1D3	mRNA	XR_001436817.1	lncRNA
XM_012104222.2	DCUN1D3	mRNA	XR_001436817.1	lncRNA
XM_012104222.2	DCUN1D3	mRNA	XR_001436817.1	lncRNA
XM_012104222.2	DCUN1D3	mRNA	XR_001436817.1	lncRNA
XM_015101000.1	RRAS2	mRNA	XR_001436817.1	lncRNA
XM_015101000.1	RRAS2	mRNA	XR_001436817.1	lncRNA
XM_015101000.1	RRAS2	mRNA	XR_001436817.1	lncRNA
XM_015101000.1	RRAS2	mRNA	XR_001436817.1	lncRNA
XM_012102598.2	TBC1D12	mRNA	XR_001436817.1	lncRNA
XM_012102598.2	TBC1D12	mRNA	XR_001436817.1	lncRNA
XM_012102598.2	TBC1D12	mRNA	XR_001436817.1	lncRNA
XM_012102598.2	TBC1D12	mRNA	XR_001436817.1	lncRNA
XM_004014588.3	PKIG	mRNA	XR_001436817.1	lncRNA
XM_004014588.3	PKIG	mRNA	XR_001436817.1	lncRNA
XM_004014588.3	PKIG	mRNA	XR_001436817.1	lncRNA
XM_004014588.3	PKIG	mRNA	XR_001436817.1	lncRNA
XM_012103323.2	KIAA1328	mRNA	XR_001433856.1	lncRNA
XM_012103323.2	KIAA1328	mRNA	XR_001433856.1	lncRNA
XM_012103323.2	KIAA1328	mRNA	XR_001433856.1	lncRNA
XM_012103323.2	KIAA1328	mRNA	XR_001433856.1	lncRNA
XM_012190351.2	ZNF420	mRNA	XR_001436817.1	lncRNA
XM_012190351.2	ZNF420	mRNA	XR_001436817.1	lncRNA
XM_012190351.2	ZNF420	mRNA	XR_001436817.1	lncRNA
XM_012190351.2	ZNF420	mRNA	XR_001436817.1	lncRNA
XM_012105014.1	LGALS8	mRNA	XR_001436817.1	lncRNA
XM_012105014.1	LGALS8	mRNA	XR_001436817.1	lncRNA
XM_012105014.1	LGALS8	mRNA	XR_001436817.1	lncRNA
XM_012105014.1	LGALS8	mRNA	XR_001436817.1	lncRNA
XM_012161705.2	LRRC8A	mRNA	XR_001433856.1	lncRNA
XM_012161705.2	LRRC8A	mRNA	XR_001433856.1	lncRNA
XM_012161705.2	LRRC8A	mRNA	XR_001433856.1	lncRNA
XM_012161705.2	LRRC8A	mRNA	XR_001433856.1	lncRNA
XM_015095262.1	COG5	mRNA	XR_001433856.1	lncRNA
XM_015095262.1	COG5	mRNA	XR_001433856.1	lncRNA
XM_015095262.1	COG5	mRNA	XR_001433856.1	lncRNA
XM_015095262.1	COG5	mRNA	XR_001433856.1	lncRNA
XM_012159714.2	KITLG	mRNA	XR_001435722.1	lncRNA
XM_012159714.2	KITLG	mRNA	XR_001435722.1	lncRNA

XM_012159714.2	KITLG	mRNA	XR_001435722.1	lncRNA
XM_012159714.2	KITLG	mRNA	XR_001435722.1	lncRNA
XM_012188419.2	LOC101108937	mRNA	XR_001436817.1	lncRNA
XM_012188419.2	LOC101108937	mRNA	XR_001436817.1	lncRNA
XM_012188419.2	LOC101108937	mRNA	XR_001436817.1	lncRNA
XM_012188419.2	LOC101108937	mRNA	XR_001436817.1	lncRNA
XM_015096209.1	SPATA9	mRNA	XR_001436817.1	lncRNA
XM_015096209.1	SPATA9	mRNA	XR_001436817.1	lncRNA
XM_015096209.1	SPATA9	mRNA	XR_001436817.1	lncRNA
XM_015096209.1	SPATA9	mRNA	XR_001436817.1	lncRNA
XM_012184403.1	ZC3H13	mRNA	XR_001436817.1	lncRNA
XM_012184403.1	ZC3H13	mRNA	XR_001436817.1	lncRNA
XM_012184403.1	ZC3H13	mRNA	XR_001436817.1	lncRNA
XM_012184403.1	ZC3H13	mRNA	XR_001436817.1	lncRNA
XM_012100082.2	WDR6	mRNA	XR_001436817.1	lncRNA
XM_012100082.2	WDR6	mRNA	XR_001436817.1	lncRNA
XM_012100082.2	WDR6	mRNA	XR_001436817.1	lncRNA
XM_012100082.2	WDR6	mRNA	XR_001436817.1	lncRNA
XM_012183459.1	ISG20L2	mRNA	XR_001436817.1	lncRNA
XM_012183459.1	ISG20L2	mRNA	XR_001436817.1	lncRNA
XM_012183459.1	ISG20L2	mRNA	XR_001436817.1	lncRNA
XM_012183459.1	ISG20L2	mRNA	XR_001436817.1	lncRNA
XM_015094800.1	RAB5B	mRNA	XR_001433856.1	lncRNA
XM_015094800.1	RAB5B	mRNA	XR_001433856.1	lncRNA
XM_015094800.1	RAB5B	mRNA	XR_001433856.1	lncRNA
XM_015094800.1	RAB5B	mRNA	XR_001433856.1	lncRNA
XM_012146477.2	ABCB6	mRNA	XR_001436817.1	lncRNA
XM_012146477.2	ABCB6	mRNA	XR_001436817.1	lncRNA
XM_012146477.2	ABCB6	mRNA	XR_001436817.1	lncRNA
XM_012146477.2	ABCB6	mRNA	XR_001436817.1	lncRNA
XM_004012193.3	RBM26	mRNA	XR_001433856.1	lncRNA
XM_004012193.3	RBM26	mRNA	XR_001433856.1	lncRNA
XM_004012193.3	RBM26	mRNA	XR_001433856.1	lncRNA
XM_004012193.3	RBM26	mRNA	XR_001433856.1	lncRNA
XM_012190020.2	ZFP90	mRNA	XR_001436817.1	lncRNA
XM_012190020.2	ZFP90	mRNA	XR_001436817.1	lncRNA
XM_012190020.2	ZFP90	mRNA	XR_001436817.1	lncRNA
XM_012190020.2	ZFP90	mRNA	XR_001436817.1	lncRNA
XM_012183459.1	ISG20L2	mRNA	XR_001433856.1	lncRNA
XM_012183459.1	ISG20L2	mRNA	XR_001433856.1	lncRNA
XM_012183459.1	ISG20L2	mRNA	XR_001433856.1	lncRNA
XM_012183459.1	ISG20L2	mRNA	XR_001433856.1	lncRNA
XM_012105014.1	LGALS8	mRNA	XR_001433856.1	lncRNA
XM_012105014.1	LGALS8	mRNA	XR_001433856.1	lncRNA
XM_012105014.1	LGALS8	mRNA	XR_001433856.1	lncRNA
XM_012105014.1	LGALS8	mRNA	XR_001433856.1	lncRNA
XM_004004099.3	GOLM1	mRNA	XR_001433856.1	lncRNA
XM_004004099.3	GOLM1	mRNA	XR_001433856.1	lncRNA
XM_004004099.3	GOLM1	mRNA	XR_001433856.1	lncRNA
XM_004004099.3	GOLM1	mRNA	XR_001433856.1	lncRNA
XM_012174290.1	ATF1	mRNA	XR_001436817.1	lncRNA
XM_012174290.1	ATF1	mRNA	XR_001436817.1	lncRNA
XM_012174290.1	ATF1	mRNA	XR_001436817.1	lncRNA
XM_012174290.1	ATF1	mRNA	XR_001436817.1	lncRNA

XM_012100082.2	WDR6	mRNA	XR_001433856.1	lncRNA
XM_012100082.2	WDR6	mRNA	XR_001433856.1	lncRNA
XM_012100082.2	WDR6	mRNA	XR_001433856.1	lncRNA
XM_012100082.2	WDR6	mRNA	XR_001433856.1	lncRNA
XM_015099384.1	SYT14	mRNA	XR_001436817.1	lncRNA
XM_015099384.1	SYT14	mRNA	XR_001436817.1	lncRNA
XM_015099384.1	SYT14	mRNA	XR_001436817.1	lncRNA
XM_015099384.1	SYT14	mRNA	XR_001436817.1	lncRNA
XM_015093726.1	HP1BP3	mRNA	XR_001433856.1	lncRNA
XM_015093726.1	HP1BP3	mRNA	XR_001433856.1	lncRNA
XM_015093726.1	HP1BP3	mRNA	XR_001433856.1	lncRNA
XM_015093726.1	HP1BP3	mRNA	XR_001433856.1	lncRNA
XM_015095262.1	COG5	mRNA	XR_001436817.1	lncRNA
XM_015095262.1	COG5	mRNA	XR_001436817.1	lncRNA
XM_015095262.1	COG5	mRNA	XR_001436817.1	lncRNA
XM_015095262.1	COG5	mRNA	XR_001436817.1	lncRNA
XM_012173546.1	NDUFAF7	mRNA	XR_001433856.1	lncRNA
XM_012173546.1	NDUFAF7	mRNA	XR_001433856.1	lncRNA
XM_012173546.1	NDUFAF7	mRNA	XR_001433856.1	lncRNA
XM_012173546.1	NDUFAF7	mRNA	XR_001433856.1	lncRNA
XM_012159735.2	KITLG	mRNA	XR_001433856.1	lncRNA
XM_012159735.2	KITLG	mRNA	XR_001433856.1	lncRNA
XM_012159735.2	KITLG	mRNA	XR_001433856.1	lncRNA
XM_012159735.2	KITLG	mRNA	XR_001433856.1	lncRNA
XM_015092648.1	ARMC8	mRNA	XR_001433856.1	lncRNA
XM_015092648.1	ARMC8	mRNA	XR_001433856.1	lncRNA
XM_015092648.1	ARMC8	mRNA	XR_001433856.1	lncRNA
XM_015092648.1	ARMC8	mRNA	XR_001433856.1	lncRNA
XM_012184403.1	ZC3H13	mRNA	XR_001433856.1	lncRNA
XM_012184403.1	ZC3H13	mRNA	XR_001433856.1	lncRNA
XM_012184403.1	ZC3H13	mRNA	XR_001433856.1	lncRNA
XM_012184403.1	ZC3H13	mRNA	XR_001433856.1	lncRNA
XM_012095623.1	TBCEL	mRNA	XR_001433856.1	lncRNA
XM_012095623.1	TBCEL	mRNA	XR_001433856.1	lncRNA
XM_012095623.1	TBCEL	mRNA	XR_001433856.1	lncRNA
XM_012095623.1	TBCEL	mRNA	XR_001433856.1	lncRNA
XM_015098067.1	RCBTB1	mRNA	XR_001433856.1	lncRNA
XM_015098067.1	RCBTB1	mRNA	XR_001433856.1	lncRNA
XM_015098067.1	RCBTB1	mRNA	XR_001433856.1	lncRNA
XM_015098067.1	RCBTB1	mRNA	XR_001433856.1	lncRNA
XM_012174289.1	ATF1	mRNA	XR_001433856.1	lncRNA
XM_012174289.1	ATF1	mRNA	XR_001433856.1	lncRNA
XM_012174289.1	ATF1	mRNA	XR_001433856.1	lncRNA
XM_012174289.1	ATF1	mRNA	XR_001433856.1	lncRNA
XM_004014588.3	PKIG	mRNA	XR_001435107.1	lncRNA
XM_004014588.3	PKIG	mRNA	XR_001435107.1	lncRNA
XM_004014588.3	PKIG	mRNA	XR_001435107.1	lncRNA
XM_004014588.3	PKIG	mRNA	XR_001435107.1	lncRNA
XM_015092648.1	ARMC8	mRNA	XR_001434920.1	lncRNA
XM_015092648.1	ARMC8	mRNA	XR_001434920.1	lncRNA
XM_015092648.1	ARMC8	mRNA	XR_001434920.1	lncRNA
XM_015092648.1	ARMC8	mRNA	XR_001434920.1	lncRNA
XM_015098987.1	ABCA5	mRNA	XR_001435107.1	lncRNA
XM_015098987.1	ABCA5	mRNA	XR_001435107.1	lncRNA

XM_015098987.1	ABCA5	mRNA	XR_001435107.1	lncRNA
XM_015098987.1	ABCA5	mRNA	XR_001435107.1	lncRNA
XM_012159735.2	KITLG	mRNA	XR_001436817.1	lncRNA
XM_012159735.2	KITLG	mRNA	XR_001436817.1	lncRNA
XM_012159735.2	KITLG	mRNA	XR_001436817.1	lncRNA
XM_012159735.2	KITLG	mRNA	XR_001436817.1	lncRNA
XM_012102667.1	ZNF518A	mRNA	XR_001435107.1	lncRNA
XM_012102667.1	ZNF518A	mRNA	XR_001435107.1	lncRNA
XM_012102667.1	ZNF518A	mRNA	XR_001435107.1	lncRNA
XM_012102667.1	ZNF518A	mRNA	XR_001435107.1	lncRNA
XM_012105014.1	LGALS8	mRNA	XR_001434920.1	lncRNA
XM_012105014.1	LGALS8	mRNA	XR_001434920.1	lncRNA
XM_012105014.1	LGALS8	mRNA	XR_001434920.1	lncRNA
XM_012105014.1	LGALS8	mRNA	XR_001434920.1	lncRNA
XM_012104235.2	DCUN1D3	mRNA	XR_001434920.1	lncRNA
XM_012104235.2	DCUN1D3	mRNA	XR_001434920.1	lncRNA
XM_012104235.2	DCUN1D3	mRNA	XR_001434920.1	lncRNA
XM_012104235.2	DCUN1D3	mRNA	XR_001434920.1	lncRNA
XM_012174290.1	ATF1	mRNA	XR_001434920.1	lncRNA
XM_012174290.1	ATF1	mRNA	XR_001434920.1	lncRNA
XM_012174290.1	ATF1	mRNA	XR_001434920.1	lncRNA
XM_012174290.1	ATF1	mRNA	XR_001434920.1	lncRNA
XM_004008085.3	CNOT4	mRNA	XR_001435107.1	lncRNA
XM_004008085.3	CNOT4	mRNA	XR_001435107.1	lncRNA
XM_004008085.3	CNOT4	mRNA	XR_001435107.1	lncRNA
XM_004008085.3	CNOT4	mRNA	XR_001435107.1	lncRNA
XM_012183459.1	ISG20L2	mRNA	XR_001434920.1	lncRNA
XM_012183459.1	ISG20L2	mRNA	XR_001434920.1	lncRNA
XM_012183459.1	ISG20L2	mRNA	XR_001434920.1	lncRNA
XM_012183459.1	ISG20L2	mRNA	XR_001434920.1	lncRNA
XM_012099221.2	SLC25A26	mRNA	XR_001435107.1	lncRNA
XM_012099221.2	SLC25A26	mRNA	XR_001435107.1	lncRNA
XM_012099221.2	SLC25A26	mRNA	XR_001435107.1	lncRNA
XM_012099221.2	SLC25A26	mRNA	XR_001435107.1	lncRNA
XM_015093726.1	HP1BP3	mRNA	XR_001434920.1	lncRNA
XM_015093726.1	HP1BP3	mRNA	XR_001434920.1	lncRNA
XM_015093726.1	HP1BP3	mRNA	XR_001434920.1	lncRNA
XM_015093726.1	HP1BP3	mRNA	XR_001434920.1	lncRNA
XM_012174597.2	NUP107	mRNA	XR_001435107.1	lncRNA
XM_012174597.2	NUP107	mRNA	XR_001435107.1	lncRNA
XM_012174597.2	NUP107	mRNA	XR_001435107.1	lncRNA
XM_012174597.2	NUP107	mRNA	XR_001435107.1	lncRNA
XM_012159735.2	KITLG	mRNA	XR_001434920.1	lncRNA
XM_012159735.2	KITLG	mRNA	XR_001434920.1	lncRNA
XM_012159735.2	KITLG	mRNA	XR_001434920.1	lncRNA
XM_012159735.2	KITLG	mRNA	XR_001434920.1	lncRNA
XM_012184403.1	ZC3H13	mRNA	XR_001434920.1	lncRNA
XM_012184403.1	ZC3H13	mRNA	XR_001434920.1	lncRNA
XM_012184403.1	ZC3H13	mRNA	XR_001434920.1	lncRNA
XM_012184403.1	ZC3H13	mRNA	XR_001434920.1	lncRNA
XM_015095262.1	COG5	mRNA	XR_001434920.1	lncRNA
XM_015095262.1	COG5	mRNA	XR_001434920.1	lncRNA
XM_015095262.1	COG5	mRNA	XR_001434920.1	lncRNA
XM_015095262.1	COG5	mRNA	XR_001434920.1	lncRNA

XM_004008085.3	CNOT4	mRNA	XR_001435722.1	lncRNA
XM_004008085.3	CNOT4	mRNA	XR_001435722.1	lncRNA
XM_004008085.3	CNOT4	mRNA	XR_001435722.1	lncRNA
XM_004008085.3	CNOT4	mRNA	XR_001435722.1	lncRNA
XM_012102598.2	TBC1D12	mRNA	XR_001435107.1	lncRNA
XM_012102598.2	TBC1D12	mRNA	XR_001435107.1	lncRNA
XM_012102598.2	TBC1D12	mRNA	XR_001435107.1	lncRNA
XM_012102598.2	TBC1D12	mRNA	XR_001435107.1	lncRNA
XM_015098987.1	ABCA5	mRNA	XR_001435722.1	lncRNA
XM_015098987.1	ABCA5	mRNA	XR_001435722.1	lncRNA
XM_015098987.1	ABCA5	mRNA	XR_001435722.1	lncRNA
XM_015098987.1	ABCA5	mRNA	XR_001435722.1	lncRNA
XM_012106815.2	RBM10	mRNA	XR_001436817.1	lncRNA
XM_012106815.2	RBM10	mRNA	XR_001436817.1	lncRNA
XM_012106815.2	RBM10	mRNA	XR_001436817.1	lncRNA
XM_012106815.2	RBM10	mRNA	XR_001436817.1	lncRNA
XM_012104232.2	DCUN1D3	mRNA	XR_001434920.1	lncRNA
XM_012104232.2	DCUN1D3	mRNA	XR_001434920.1	lncRNA
XM_012104232.2	DCUN1D3	mRNA	XR_001434920.1	lncRNA
XM_012104232.2	DCUN1D3	mRNA	XR_001434920.1	lncRNA
XM_004014588.3	PKIG	mRNA	XR_001435722.1	lncRNA
XM_004014588.3	PKIG	mRNA	XR_001435722.1	lncRNA
XM_004014588.3	PKIG	mRNA	XR_001435722.1	lncRNA
XM_004014588.3	PKIG	mRNA	XR_001435722.1	lncRNA
XM_012099221.2	SLC25A26	mRNA	XR_001434920.1	lncRNA
XM_012099221.2	SLC25A26	mRNA	XR_001434920.1	lncRNA
XM_012099221.2	SLC25A26	mRNA	XR_001434920.1	lncRNA
XM_012099221.2	SLC25A26	mRNA	XR_001434920.1	lncRNA
XM_015098983.1	C11H17orf80	mRNA	XR_001436817.1	lncRNA
XM_015098983.1	C11H17orf80	mRNA	XR_001436817.1	lncRNA
XM_015098983.1	C11H17orf80	mRNA	XR_001436817.1	lncRNA
XM_015098983.1	C11H17orf80	mRNA	XR_001436817.1	lncRNA
XM_015095024.1	JOSD1	mRNA	XR_001435722.1	lncRNA
XM_015095024.1	JOSD1	mRNA	XR_001435722.1	lncRNA
XM_015095024.1	JOSD1	mRNA	XR_001435722.1	lncRNA
XM_015095024.1	JOSD1	mRNA	XR_001435722.1	lncRNA
XM_015095262.1	COG5	mRNA	XR_001435722.1	lncRNA
XM_015095262.1	COG5	mRNA	XR_001435722.1	lncRNA
XM_015095262.1	COG5	mRNA	XR_001435722.1	lncRNA
XM_015095262.1	COG5	mRNA	XR_001435722.1	lncRNA
XM_004012073.3	FNDC3A	mRNA	XR_001436612.1	lncRNA
XM_004012073.3	FNDC3A	mRNA	XR_001436612.1	lncRNA
XM_004012073.3	FNDC3A	mRNA	XR_001436612.1	lncRNA
XM_004012073.3	FNDC3A	mRNA	XR_001436612.1	lncRNA
XM_012176414.2	PHF14	mRNA	XR_001436612.1	lncRNA
XM_012176414.2	PHF14	mRNA	XR_001436612.1	lncRNA
XM_012176414.2	PHF14	mRNA	XR_001436612.1	lncRNA
XM_012176414.2	PHF14	mRNA	XR_001436612.1	lncRNA
XM_012181827.2	ZBTB1	mRNA	XR_001436612.1	lncRNA
XM_012181827.2	ZBTB1	mRNA	XR_001436612.1	lncRNA
XM_012181827.2	ZBTB1	mRNA	XR_001436612.1	lncRNA
XM_012181827.2	ZBTB1	mRNA	XR_001436612.1	lncRNA
XM_012187630.2	SOAT1	mRNA	XR_001436612.1	lncRNA
XM_012187630.2	SOAT1	mRNA	XR_001436612.1	lncRNA

XM_012187630.2	SOAT1	mRNA	XR_001436612.1	lncRNA
XM_012187630.2	SOAT1	mRNA	XR_001436612.1	lncRNA
XM_012095602.2	USP2	mRNA	XR_001436612.1	lncRNA
XM_012095602.2	USP2	mRNA	XR_001436612.1	lncRNA
XM_012095602.2	USP2	mRNA	XR_001436612.1	lncRNA
XM_012095602.2	USP2	mRNA	XR_001436612.1	lncRNA
XM_012103799.1	NEDD4L	mRNA	XR_001041893.2	lncRNA
XM_012103799.1	NEDD4L	mRNA	XR_001041893.2	lncRNA
XM_012103799.1	NEDD4L	mRNA	XR_001041893.2	lncRNA
XM_012103799.1	NEDD4L	mRNA	XR_001041893.2	lncRNA
XM_015094252.1	DNMT3A	mRNA	XR_001023303.2	lncRNA
XM_015094252.1	DNMT3A	mRNA	XR_001023303.2	lncRNA
XM_015094252.1	DNMT3A	mRNA	XR_001023303.2	lncRNA
XM_015094252.1	DNMT3A	mRNA	XR_001023303.2	lncRNA
XM_012185338.2	ZNF207	mRNA	XR_001023303.2	lncRNA
XM_012185338.2	ZNF207	mRNA	XR_001023303.2	lncRNA
XM_012185338.2	ZNF207	mRNA	XR_001023303.2	lncRNA
XM_012185338.2	ZNF207	mRNA	XR_001023303.2	lncRNA
XM_004017814.3	DNAJA4	mRNA	XR_001041893.2	lncRNA
XM_004017814.3	DNAJA4	mRNA	XR_001041893.2	lncRNA
XM_004017814.3	DNAJA4	mRNA	XR_001041893.2	lncRNA
XM_004017814.3	DNAJA4	mRNA	XR_001041893.2	lncRNA
XM_012187084.2	MIA3	mRNA	XR_001023303.2	lncRNA
XM_012187084.2	MIA3	mRNA	XR_001023303.2	lncRNA
XM_012187084.2	MIA3	mRNA	XR_001023303.2	lncRNA
XM_012187084.2	MIA3	mRNA	XR_001023303.2	lncRNA
XM_012185338.2	ZNF207	mRNA	XR_001041893.2	lncRNA
XM_012185338.2	ZNF207	mRNA	XR_001041893.2	lncRNA
XM_012185338.2	ZNF207	mRNA	XR_001041893.2	lncRNA
XM_012185338.2	ZNF207	mRNA	XR_001041893.2	lncRNA
XM_004003770.3	IMPG2	mRNA	XR_001023303.2	lncRNA
XM_004003770.3	IMPG2	mRNA	XR_001023303.2	lncRNA
XM_004003770.3	IMPG2	mRNA	XR_001023303.2	lncRNA
XM_004003770.3	IMPG2	mRNA	XR_001023303.2	lncRNA
XM_004010549.3	VPS13C	mRNA	XR_001025540.2	lncRNA
XM_004010549.3	VPS13C	mRNA	XR_001025540.2	lncRNA
XM_004010549.3	VPS13C	mRNA	XR_001025540.2	lncRNA
XM_004010549.3	VPS13C	mRNA	XR_001025540.2	lncRNA
XM_015099563.1	PARD3	mRNA	XR_001025540.2	lncRNA
XM_015099563.1	PARD3	mRNA	XR_001025540.2	lncRNA
XM_015099563.1	PARD3	mRNA	XR_001025540.2	lncRNA
XM_015099563.1	PARD3	mRNA	XR_001025540.2	lncRNA
XM_012105324.2	USP54	mRNA	XR_001025540.2	lncRNA
XM_012105324.2	USP54	mRNA	XR_001025540.2	lncRNA
XM_012105324.2	USP54	mRNA	XR_001025540.2	lncRNA
XM_012105324.2	USP54	mRNA	XR_001025540.2	lncRNA
XM_015101341.1	OTULIN	mRNA	XR_001025540.2	lncRNA
XM_015101341.1	OTULIN	mRNA	XR_001025540.2	lncRNA
XM_015101341.1	OTULIN	mRNA	XR_001025540.2	lncRNA
XM_015101341.1	OTULIN	mRNA	XR_001025540.2	lncRNA
XM_012102930.1	GPAM	mRNA	XR_001436390.1	lncRNA
XM_012102930.1	GPAM	mRNA	XR_001436390.1	lncRNA
XM_012102930.1	GPAM	mRNA	XR_001436390.1	lncRNA
XM_012102930.1	GPAM	mRNA	XR_001436390.1	lncRNA

XM_012097252.2	RHOF	mRNA	XR_001040147.1	lncRNA
XM_012097252.2	RHOF	mRNA	XR_001040147.1	lncRNA
XM_012097252.2	RHOF	mRNA	XR_001040147.1	lncRNA
XM_012097252.2	RHOF	mRNA	XR_001040147.1	lncRNA
XM_012106112.2	ARAF	mRNA	XR_001436390.1	lncRNA
XM_012106112.2	ARAF	mRNA	XR_001436390.1	lncRNA
XM_012106112.2	ARAF	mRNA	XR_001436390.1	lncRNA
XM_012106112.2	ARAF	mRNA	XR_001436390.1	lncRNA
XM_015094471.1	CCDC142	mRNA	XR_001040147.1	lncRNA
XM_015094471.1	CCDC142	mRNA	XR_001040147.1	lncRNA
XM_015094471.1	CCDC142	mRNA	XR_001040147.1	lncRNA
XM_015094471.1	CCDC142	mRNA	XR_001040147.1	lncRNA
XM_012180798.1	TIPIN	mRNA	XR_001436390.1	lncRNA
XM_012180798.1	TIPIN	mRNA	XR_001436390.1	lncRNA
XM_012180798.1	TIPIN	mRNA	XR_001436390.1	lncRNA
XM_012180798.1	TIPIN	mRNA	XR_001436390.1	lncRNA
XM_015096221.1	PPIP5K2	mRNA	XR_001436390.1	lncRNA
XM_015096221.1	PPIP5K2	mRNA	XR_001436390.1	lncRNA
XM_015096221.1	PPIP5K2	mRNA	XR_001436390.1	lncRNA
XM_015096221.1	PPIP5K2	mRNA	XR_001436390.1	lncRNA
XM_012100080.2	ARIH2	mRNA	XR_001436390.1	lncRNA
XM_012100080.2	ARIH2	mRNA	XR_001436390.1	lncRNA
XM_012100080.2	ARIH2	mRNA	XR_001436390.1	lncRNA
XM_012100080.2	ARIH2	mRNA	XR_001436390.1	lncRNA
XM_015101559.1	DDX51	mRNA	XR_001040147.1	lncRNA
XM_015101559.1	DDX51	mRNA	XR_001040147.1	lncRNA
XM_015101559.1	DDX51	mRNA	XR_001040147.1	lncRNA
XM_015101559.1	DDX51	mRNA	XR_001040147.1	lncRNA
XM_004008822.3	FAM13B	mRNA	XR_001040147.1	lncRNA
XM_004008822.3	FAM13B	mRNA	XR_001040147.1	lncRNA
XM_004008822.3	FAM13B	mRNA	XR_001040147.1	lncRNA
XM_004008822.3	FAM13B	mRNA	XR_001040147.1	lncRNA
XM_015098186.1	XPO4	mRNA	XR_001436390.1	lncRNA
XM_015098186.1	XPO4	mRNA	XR_001436390.1	lncRNA
XM_015098186.1	XPO4	mRNA	XR_001436390.1	lncRNA
XM_015098186.1	XPO4	mRNA	XR_001436390.1	lncRNA
XM_004008381.3	MAPK9	mRNA	XR_001040147.1	lncRNA
XM_004008381.3	MAPK9	mRNA	XR_001040147.1	lncRNA
XM_004008381.3	MAPK9	mRNA	XR_001040147.1	lncRNA
XM_004008381.3	MAPK9	mRNA	XR_001040147.1	lncRNA
XM_012107808.1	NFYC	mRNA	XR_001433837.1	lncRNA
XM_012107808.1	NFYC	mRNA	XR_001433837.1	lncRNA
XM_012107808.1	NFYC	mRNA	XR_001433837.1	lncRNA
XM_012107808.1	NFYC	mRNA	XR_001433837.1	lncRNA
XM_015104915.1	JADE3	mRNA	XR_001433837.1	lncRNA
XM_015104915.1	JADE3	mRNA	XR_001433837.1	lncRNA
XM_015104915.1	JADE3	mRNA	XR_001433837.1	lncRNA
XM_015104915.1	JADE3	mRNA	XR_001433837.1	lncRNA
XM_012098903.1	PPIP5K1	mRNA	XR_001433837.1	lncRNA
XM_012098903.1	PPIP5K1	mRNA	XR_001433837.1	lncRNA
XM_012098903.1	PPIP5K1	mRNA	XR_001433837.1	lncRNA
XM_012098903.1	PPIP5K1	mRNA	XR_001433837.1	lncRNA
XM_012099341.2	CLASP2	mRNA	XR_001433837.1	lncRNA
XM_012099341.2	CLASP2	mRNA	XR_001433837.1	lncRNA

XM_012099341.2	CLASP2	mRNA	XR_001433837.1	lncRNA
XM_012099341.2	CLASP2	mRNA	XR_001433837.1	lncRNA
XM_012185875.1	LUC7L3	mRNA	XR_001433837.1	lncRNA
XM_012185875.1	LUC7L3	mRNA	XR_001433837.1	lncRNA
XM_012185875.1	LUC7L3	mRNA	XR_001433837.1	lncRNA
XM_012185875.1	LUC7L3	mRNA	XR_001433837.1	lncRNA
XM_012188274.2	CCDC7	mRNA	XR_001433837.1	lncRNA
XM_012188274.2	CCDC7	mRNA	XR_001433837.1	lncRNA
XM_012188274.2	CCDC7	mRNA	XR_001433837.1	lncRNA
XM_012188274.2	CCDC7	mRNA	XR_001433837.1	lncRNA
XM_012176761.2	MDFIC	mRNA	XR_001023627.2	lncRNA
XM_012176761.2	MDFIC	mRNA	XR_001023627.2	lncRNA
XM_012176761.2	MDFIC	mRNA	XR_001023627.2	lncRNA
XM_012176761.2	MDFIC	mRNA	XR_001023627.2	lncRNA
XM_012095602.2	USP2	mRNA	XR_001023627.2	lncRNA
XM_012095602.2	USP2	mRNA	XR_001023627.2	lncRNA
XM_012095602.2	USP2	mRNA	XR_001023627.2	lncRNA
XM_012095602.2	USP2	mRNA	XR_001023627.2	lncRNA
XM_012116543.2	ERCC6L2	mRNA	TCONS_00049802	lncRNA
XM_012116543.2	ERCC6L2	mRNA	TCONS_00049802	lncRNA
XM_012116543.2	ERCC6L2	mRNA	TCONS_00049802	lncRNA
XM_012116543.2	ERCC6L2	mRNA	TCONS_00049802	lncRNA
XM_015093216.1	TTC39B	mRNA	XR_001434127.1	lncRNA
XM_015093216.1	TTC39B	mRNA	XR_001434127.1	lncRNA
XM_015093216.1	TTC39B	mRNA	XR_001434127.1	lncRNA
XM_015093216.1	TTC39B	mRNA	XR_001434127.1	lncRNA
XM_012181434.2	RNF111	mRNA	XR_001023627.2	lncRNA
XM_012181434.2	RNF111	mRNA	XR_001023627.2	lncRNA
XM_012181434.2	RNF111	mRNA	XR_001023627.2	lncRNA
XM_012181434.2	RNF111	mRNA	XR_001023627.2	lncRNA
XM_015093216.1	TTC39B	mRNA	TCONS_00049802	lncRNA
XM_015093216.1	TTC39B	mRNA	TCONS_00049802	lncRNA
XM_015093216.1	TTC39B	mRNA	TCONS_00049802	lncRNA
XM_015093216.1	TTC39B	mRNA	TCONS_00049802	lncRNA
XM_012116543.2	ERCC6L2	mRNA	XR_001434127.1	lncRNA
XM_012116543.2	ERCC6L2	mRNA	XR_001434127.1	lncRNA
XM_012116543.2	ERCC6L2	mRNA	XR_001434127.1	lncRNA
XM_012116543.2	ERCC6L2	mRNA	XR_001434127.1	lncRNA
XM_012116543.2	ERCC6L2	mRNA	XR_001023627.2	lncRNA
XM_012116543.2	ERCC6L2	mRNA	XR_001023627.2	lncRNA
XM_012116543.2	ERCC6L2	mRNA	XR_001023627.2	lncRNA
XM_012116543.2	ERCC6L2	mRNA	XR_001023627.2	lncRNA
XM_012177522.2	VDAC1	mRNA	XR_001434127.1	lncRNA
XM_012177522.2	VDAC1	mRNA	XR_001434127.1	lncRNA
XM_012177522.2	VDAC1	mRNA	XR_001434127.1	lncRNA
XM_012177522.2	VDAC1	mRNA	XR_001434127.1	lncRNA
XM_012095273.2	ARNTL	mRNA	XR_001023303.2	lncRNA
XM_012095273.2	ARNTL	mRNA	XR_001023303.2	lncRNA
XM_012095273.2	ARNTL	mRNA	XR_001023303.2	lncRNA
XM_012095273.2	ARNTL	mRNA	XR_001023303.2	lncRNA
XM_012102832.2	PPRC1	mRNA	XR_001023303.2	lncRNA
XM_012102832.2	PPRC1	mRNA	XR_001023303.2	lncRNA
XM_012102832.2	PPRC1	mRNA	XR_001023303.2	lncRNA
XM_012102832.2	PPRC1	mRNA	XR_001023303.2	lncRNA

XM_015098082.1	RNASEH2B	mRNA	XR_001023303.2	lncRNA
XM_015098082.1	RNASEH2B	mRNA	XR_001023303.2	lncRNA
XM_015098082.1	RNASEH2B	mRNA	XR_001023303.2	lncRNA
XM_015098082.1	RNASEH2B	mRNA	XR_001023303.2	lncRNA
XM_015098082.1	RNASEH2B	mRNA	XR_001024966.2	lncRNA
XM_015098082.1	RNASEH2B	mRNA	XR_001024966.2	lncRNA
XM_015098082.1	RNASEH2B	mRNA	XR_001024966.2	lncRNA
XM_015098082.1	RNASEH2B	mRNA	XR_001024966.2	lncRNA
XM_012105333.2	USP54	mRNA	XR_001024966.2	lncRNA
XM_012105333.2	USP54	mRNA	XR_001024966.2	lncRNA
XM_012105333.2	USP54	mRNA	XR_001024966.2	lncRNA
XM_012105333.2	USP54	mRNA	XR_001024966.2	lncRNA
XM_012097365.2	SH3D19	mRNA	XR_001433837.1	lncRNA
XM_012097365.2	SH3D19	mRNA	XR_001433837.1	lncRNA
XM_012097365.2	SH3D19	mRNA	XR_001433837.1	lncRNA
XM_012097365.2	SH3D19	mRNA	XR_001433837.1	lncRNA
XM_012178157.2	RAPGEF6	mRNA	XR_001436612.1	lncRNA
XM_012178157.2	RAPGEF6	mRNA	XR_001436612.1	lncRNA
XM_012178157.2	RAPGEF6	mRNA	XR_001436612.1	lncRNA
XM_012178157.2	RAPGEF6	mRNA	XR_001436612.1	lncRNA
XM_012189447.2	ALCAM	mRNA	XR_001436612.1	lncRNA
XM_012189447.2	ALCAM	mRNA	XR_001436612.1	lncRNA
XM_012189447.2	ALCAM	mRNA	XR_001436612.1	lncRNA
XM_012189447.2	ALCAM	mRNA	XR_001436612.1	lncRNA
XM_012181270.1	VPS39	mRNA	XR_001024230.1	lncRNA
XM_012181270.1	VPS39	mRNA	XR_001024230.1	lncRNA
XM_012181270.1	VPS39	mRNA	XR_001024230.1	lncRNA
XM_012181270.1	VPS39	mRNA	XR_001024230.1	lncRNA
XM_012179413.2	PDE5A	mRNA	XR_001436064.1	lncRNA
XM_012179413.2	PDE5A	mRNA	XR_001436064.1	lncRNA
XM_012179413.2	PDE5A	mRNA	XR_001436064.1	lncRNA
XM_012179413.2	PDE5A	mRNA	XR_001436064.1	lncRNA
XM_015092004.1	MROH7	mRNA	XR_001024230.1	lncRNA
XM_015092004.1	MROH7	mRNA	XR_001024230.1	lncRNA
XM_015092004.1	MROH7	mRNA	XR_001024230.1	lncRNA
XM_015092004.1	MROH7	mRNA	XR_001024230.1	lncRNA
XM_012186029.1	FBXL20	mRNA	XR_001435723.1	lncRNA
XM_012186029.1	FBXL20	mRNA	XR_001435723.1	lncRNA
XM_012186029.1	FBXL20	mRNA	XR_001435723.1	lncRNA
XM_012186029.1	FBXL20	mRNA	XR_001435723.1	lncRNA
XM_012102930.1	GPAM	mRNA	XR_001435723.1	lncRNA
XM_012102930.1	GPAM	mRNA	XR_001435723.1	lncRNA
XM_012102930.1	GPAM	mRNA	XR_001435723.1	lncRNA
XM_012102930.1	GPAM	mRNA	XR_001435723.1	lncRNA
XM_012101006.2	CCHCR1	mRNA	XR_001436064.1	lncRNA
XM_012101006.2	CCHCR1	mRNA	XR_001436064.1	lncRNA
XM_012101006.2	CCHCR1	mRNA	XR_001436064.1	lncRNA
XM_012101006.2	CCHCR1	mRNA	XR_001436064.1	lncRNA
XM_012101050.1	ZNF311	mRNA	XR_001435723.1	lncRNA
XM_012101050.1	ZNF311	mRNA	XR_001435723.1	lncRNA
XM_012101050.1	ZNF311	mRNA	XR_001435723.1	lncRNA
XM_012101050.1	ZNF311	mRNA	XR_001435723.1	lncRNA
XM_015098186.1	XPO4	mRNA	XR_001435723.1	lncRNA
XM_015098186.1	XPO4	mRNA	XR_001435723.1	lncRNA

XM_015098186.1	XPO4	mRNA	XR_001435723.1	lncRNA
XM_015098186.1	XPO4	mRNA	XR_001435723.1	lncRNA
XM_015092004.1	MROH7	mRNA	XR_001435723.1	lncRNA
XM_015092004.1	MROH7	mRNA	XR_001435723.1	lncRNA
XM_015092004.1	MROH7	mRNA	XR_001435723.1	lncRNA
XM_015092004.1	MROH7	mRNA	XR_001435723.1	lncRNA
XM_015094919.1	WBP11	mRNA	XR_001436064.1	lncRNA
XM_015094919.1	WBP11	mRNA	XR_001436064.1	lncRNA
XM_015094919.1	WBP11	mRNA	XR_001436064.1	lncRNA
XM_015094919.1	WBP11	mRNA	XR_001436064.1	lncRNA
XM_012178866.2	THG1L	mRNA	XR_001435723.1	lncRNA
XM_012178866.2	THG1L	mRNA	XR_001435723.1	lncRNA
XM_012178866.2	THG1L	mRNA	XR_001435723.1	lncRNA
XM_012178866.2	THG1L	mRNA	XR_001435723.1	lncRNA
XM_012106112.2	ARAF	mRNA	XR_001024230.1	lncRNA
XM_012106112.2	ARAF	mRNA	XR_001024230.1	lncRNA
XM_012106112.2	ARAF	mRNA	XR_001024230.1	lncRNA
XM_012106112.2	ARAF	mRNA	XR_001024230.1	lncRNA
XM_015094919.1	WBP11	mRNA	XR_001436065.1	lncRNA
XM_015094919.1	WBP11	mRNA	XR_001436065.1	lncRNA
XM_015094919.1	WBP11	mRNA	XR_001436065.1	lncRNA
XM_015094919.1	WBP11	mRNA	XR_001436065.1	lncRNA
XM_012102930.1	GPAM	mRNA	XR_001435726.1	lncRNA
XM_012102930.1	GPAM	mRNA	XR_001435726.1	lncRNA
XM_012102930.1	GPAM	mRNA	XR_001435726.1	lncRNA
XM_012102930.1	GPAM	mRNA	XR_001435726.1	lncRNA
XM_015098186.1	XPO4	mRNA	XR_001435726.1	lncRNA
XM_015098186.1	XPO4	mRNA	XR_001435726.1	lncRNA
XM_015098186.1	XPO4	mRNA	XR_001435726.1	lncRNA
XM_015098186.1	XPO4	mRNA	XR_001435726.1	lncRNA
XM_012178866.2	THG1L	mRNA	XR_001435726.1	lncRNA
XM_012178866.2	THG1L	mRNA	XR_001435726.1	lncRNA
XM_012178866.2	THG1L	mRNA	XR_001435726.1	lncRNA
XM_012178866.2	THG1L	mRNA	XR_001435726.1	lncRNA
XM_012101050.1	ZNF311	mRNA	XR_001435726.1	lncRNA
XM_012101050.1	ZNF311	mRNA	XR_001435726.1	lncRNA
XM_012101050.1	ZNF311	mRNA	XR_001435726.1	lncRNA
XM_012101050.1	ZNF311	mRNA	XR_001435726.1	lncRNA
XM_012143533.1	INO80D	mRNA	XR_001025540.2	lncRNA
XM_012143533.1	INO80D	mRNA	XR_001025540.2	lncRNA
XM_012143533.1	INO80D	mRNA	XR_001025540.2	lncRNA
XM_012143533.1	INO80D	mRNA	XR_001025540.2	lncRNA
XM_004020479.3	RBBP8	mRNA	TCONS_00039112	lncRNA
XM_004020479.3	RBBP8	mRNA	TCONS_00039112	lncRNA
XM_004020479.3	RBBP8	mRNA	TCONS_00039112	lncRNA
XM_015101000.1	RRAS2	mRNA	TCONS_00056022	lncRNA
XM_015101000.1	RRAS2	mRNA	TCONS_00056022	lncRNA
XM_015101000.1	RRAS2	mRNA	TCONS_00056022	lncRNA
XM_015095262.1	COG5	mRNA	TCONS_00056022	lncRNA
XM_015095262.1	COG5	mRNA	TCONS_00056022	lncRNA
XM_015095262.1	COG5	mRNA	TCONS_00056022	lncRNA
XM_015098592.1	RABEP1	mRNA	XR_001436390.1	lncRNA
XM_015098592.1	RABEP1	mRNA	XR_001436390.1	lncRNA
XM_015098592.1	RABEP1	mRNA	XR_001436390.1	lncRNA

XM_015098592.1	RABEP1	mRNA	XR_001436390.1	lncRNA
XM_015104417.1	CHST3	mRNA	XR_001040147.1	lncRNA
XM_015104417.1	CHST3	mRNA	XR_001040147.1	lncRNA
XM_015104417.1	CHST3	mRNA	XR_001040147.1	lncRNA
XM_015104417.1	CHST3	mRNA	XR_001040147.1	lncRNA
XM_012187827.1	GIGYF2	mRNA	XR_001040147.1	lncRNA
XM_012187827.1	GIGYF2	mRNA	XR_001040147.1	lncRNA
XM_012187827.1	GIGYF2	mRNA	XR_001040147.1	lncRNA
XM_012187827.1	GIGYF2	mRNA	XR_001040147.1	lncRNA
XM_012097555.2	RAPGEF2	mRNA	XR_001041893.2	lncRNA
XM_012097555.2	RAPGEF2	mRNA	XR_001041893.2	lncRNA
XM_012097555.2	RAPGEF2	mRNA	XR_001041893.2	lncRNA
XM_012097555.2	RAPGEF2	mRNA	XR_001041893.2	lncRNA
XM_012105324.2	USP54	mRNA	XR_001023303.2	lncRNA
XM_012105324.2	USP54	mRNA	XR_001023303.2	lncRNA
XM_012105324.2	USP54	mRNA	XR_001023303.2	lncRNA
XM_012105324.2	USP54	mRNA	XR_001023303.2	lncRNA
XM_012095733.1	BTBD10	mRNA	XR_001041893.2	lncRNA
XM_012095733.1	BTBD10	mRNA	XR_001041893.2	lncRNA
XM_012095733.1	BTBD10	mRNA	XR_001041893.2	lncRNA
XM_012095733.1	BTBD10	mRNA	XR_001041893.2	lncRNA
XM_012185875.1	LUC7L3	mRNA	XR_001434127.1	lncRNA
XM_012185875.1	LUC7L3	mRNA	XR_001434127.1	lncRNA
XM_012185875.1	LUC7L3	mRNA	XR_001434127.1	lncRNA
XM_012185875.1	LUC7L3	mRNA	XR_001434127.1	lncRNA
XM_012099341.2	CLASP2	mRNA	XR_001434127.1	lncRNA
XM_012099341.2	CLASP2	mRNA	XR_001434127.1	lncRNA
XM_012099341.2	CLASP2	mRNA	XR_001434127.1	lncRNA
XM_012099341.2	CLASP2	mRNA	XR_001434127.1	lncRNA
XM_015092063.1	FUBP1	mRNA	XR_001434127.1	lncRNA
XM_015092063.1	FUBP1	mRNA	XR_001434127.1	lncRNA
XM_015092063.1	FUBP1	mRNA	XR_001434127.1	lncRNA
XM_015092063.1	FUBP1	mRNA	XR_001434127.1	lncRNA
XM_012183298.1	FAM120B	mRNA	TCONS_00049802	lncRNA
XM_012183298.1	FAM120B	mRNA	TCONS_00049802	lncRNA
XM_012183298.1	FAM120B	mRNA	TCONS_00049802	lncRNA
XM_012183298.1	FAM120B	mRNA	TCONS_00049802	lncRNA
XM_004015120.2	PHLPP2	mRNA	XR_001023627.2	lncRNA
XM_004015120.2	PHLPP2	mRNA	XR_001023627.2	lncRNA
XM_004015120.2	PHLPP2	mRNA	XR_001023627.2	lncRNA
XM_004015120.2	PHLPP2	mRNA	XR_001023627.2	lncRNA
XM_015104915.1	JADE3	mRNA	XR_001023627.2	lncRNA
XM_015104915.1	JADE3	mRNA	XR_001023627.2	lncRNA
XM_015104915.1	JADE3	mRNA	XR_001023627.2	lncRNA
XM_015104915.1	JADE3	mRNA	XR_001023627.2	lncRNA
XM_012099341.2	CLASP2	mRNA	XR_001023627.2	lncRNA
XM_012099341.2	CLASP2	mRNA	XR_001023627.2	lncRNA
XM_012099341.2	CLASP2	mRNA	XR_001023627.2	lncRNA
XM_012099341.2	CLASP2	mRNA	XR_001023627.2	lncRNA
XM_012102544.2	HECTL1	mRNA	XR_001022883.1	lncRNA
XM_012102544.2	HECTL1	mRNA	XR_001022883.1	lncRNA
XM_012102544.2	HECTL1	mRNA	XR_001022883.1	lncRNA
XM_012176821.2	ANLN	mRNA	XR_001022883.1	lncRNA
XM_012176821.2	ANLN	mRNA	XR_001022883.1	lncRNA

XM_012176821.2	ANLN	mRNA	XR_001022883.1	lncRNA
XM_012105014.1	LGALS8	mRNA	TCONS_00068429	lncRNA
XM_012105014.1	LGALS8	mRNA	TCONS_00068429	lncRNA
XM_012105014.1	LGALS8	mRNA	TCONS_00068429	lncRNA
XM_012097473.2	ELF2	mRNA	TCONS_00039112	lncRNA
XM_012097473.2	ELF2	mRNA	TCONS_00039112	lncRNA
XM_012097473.2	ELF2	mRNA	TCONS_00039112	lncRNA
XM_012187630.2	SOAT1	mRNA	TCONS_00056022	lncRNA
XM_012187630.2	SOAT1	mRNA	TCONS_00056022	lncRNA
XM_012187630.2	SOAT1	mRNA	TCONS_00056022	lncRNA
XM_015100095.1	LOC101113523	mRNA	TCONS_00056022	lncRNA
XM_015100095.1	LOC101113523	mRNA	TCONS_00056022	lncRNA
XM_015100095.1	LOC101113523	mRNA	TCONS_00056022	lncRNA
XM_004007703.3	CDK14	mRNA	XR_001041893.2	lncRNA
XM_004007703.3	CDK14	mRNA	XR_001041893.2	lncRNA
XM_004007703.3	CDK14	mRNA	XR_001041893.2	lncRNA
XM_015098807.1	ETV4	mRNA	XR_001024966.2	lncRNA
XM_015098807.1	ETV4	mRNA	XR_001024966.2	lncRNA
XM_015098807.1	ETV4	mRNA	XR_001024966.2	lncRNA
XM_015099976.1	ZFP30	mRNA	TCONS_00039112	lncRNA
XM_015099976.1	ZFP30	mRNA	TCONS_00039112	lncRNA
XM_015099976.1	ZFP30	mRNA	TCONS_00039112	lncRNA
XM_012106946.2	ATP7A	mRNA	TCONS_00056022	lncRNA
XM_012106946.2	ATP7A	mRNA	TCONS_00056022	lncRNA
XM_012106946.2	ATP7A	mRNA	TCONS_00056022	lncRNA
XM_012146540.1	ATG9A	mRNA	XR_001022883.1	lncRNA
XM_012146540.1	ATG9A	mRNA	XR_001022883.1	lncRNA
XM_012146540.1	ATG9A	mRNA	XR_001022883.1	lncRNA
XM_015091887.1	MYCL	mRNA	TCONS_00056022	lncRNA
XM_015091887.1	MYCL	mRNA	TCONS_00056022	lncRNA
XM_015091887.1	MYCL	mRNA	TCONS_00056022	lncRNA
XM_015104577.1	TUSC3	mRNA	XR_001433837.1	lncRNA
XM_015104577.1	TUSC3	mRNA	XR_001433837.1	lncRNA
XM_015104577.1	TUSC3	mRNA	XR_001433837.1	lncRNA
XM_004007914.3	NT5C3A	mRNA	XR_001433837.1	lncRNA
XM_004007914.3	NT5C3A	mRNA	XR_001433837.1	lncRNA
XM_004007914.3	NT5C3A	mRNA	XR_001433837.1	lncRNA
XM_012176839.2	NT5C3A	mRNA	XR_001433837.1	lncRNA
XM_012176839.2	NT5C3A	mRNA	XR_001433837.1	lncRNA
XM_012176839.2	NT5C3A	mRNA	XR_001433837.1	lncRNA
XM_004012186.3	KLF5	mRNA	XR_001433837.1	lncRNA
XM_004012186.3	KLF5	mRNA	XR_001433837.1	lncRNA
XM_004012186.3	KLF5	mRNA	XR_001433837.1	lncRNA
XM_004014289.3	ZNF133	mRNA	TCONS_00068429	lncRNA
XM_004014289.3	ZNF133	mRNA	TCONS_00068429	lncRNA
XM_004014289.3	ZNF133	mRNA	TCONS_00068429	lncRNA
XM_015103672.1	SPIRE1	mRNA	TCONS_00068429	lncRNA
XM_015103672.1	SPIRE1	mRNA	TCONS_00068429	lncRNA
XM_015103672.1	SPIRE1	mRNA	TCONS_00068429	lncRNA
XM_012180798.1	TIPIN	mRNA	TCONS_00068429	lncRNA
XM_012180798.1	TIPIN	mRNA	TCONS_00068429	lncRNA
XM_012180798.1	TIPIN	mRNA	TCONS_00068429	lncRNA
XM_012110487.2	UBQLN1	mRNA	XR_001025540.2	lncRNA
XM_012110487.2	UBQLN1	mRNA	XR_001025540.2	lncRNA

XM_012110487.2	UBQLN1	mRNA	XR_001025540.2	lncRNA
XM_004012186.3	KLF5	mRNA	XR_001436612.1	lncRNA
XM_004012186.3	KLF5	mRNA	XR_001436612.1	lncRNA
XM_004012186.3	KLF5	mRNA	XR_001436612.1	lncRNA
XM_012181772.2	PCNXL4	mRNA	XR_001436612.1	lncRNA
XM_012181772.2	PCNXL4	mRNA	XR_001436612.1	lncRNA
XM_012181772.2	PCNXL4	mRNA	XR_001436612.1	lncRNA
XM_012124491.2	AK3	mRNA	XR_001436612.1	lncRNA
XM_012124491.2	AK3	mRNA	XR_001436612.1	lncRNA
XM_012124491.2	AK3	mRNA	XR_001436612.1	lncRNA
XM_015093485.1	ORC2	mRNA	XR_001436612.1	lncRNA
XM_015093485.1	ORC2	mRNA	XR_001436612.1	lncRNA
XM_015093485.1	ORC2	mRNA	XR_001436612.1	lncRNA
XM_012167069.2	PLB1	mRNA	XR_001436612.1	lncRNA
XM_012167069.2	PLB1	mRNA	XR_001436612.1	lncRNA
XM_012167069.2	PLB1	mRNA	XR_001436612.1	lncRNA
XM_015098807.1	ETV4	mRNA	XR_001436612.1	lncRNA
XM_015098807.1	ETV4	mRNA	XR_001436612.1	lncRNA
XM_015098807.1	ETV4	mRNA	XR_001436612.1	lncRNA
XM_012101269.2	SIRT5	mRNA	XR_001436612.1	lncRNA
XM_012101269.2	SIRT5	mRNA	XR_001436612.1	lncRNA
XM_012101269.2	SIRT5	mRNA	XR_001436612.1	lncRNA
XM_012177522.2	VDAC1	mRNA	XR_001024290.1	lncRNA
XM_012177522.2	VDAC1	mRNA	XR_001024290.1	lncRNA
XM_012177522.2	VDAC1	mRNA	XR_001024290.1	lncRNA
XM_012094885.1	LOC101116597	mRNA	TCONS_00068429	lncRNA
XM_012094885.1	LOC101116597	mRNA	TCONS_00068429	lncRNA
XM_012094885.1	LOC101116597	mRNA	TCONS_00068429	lncRNA
XM_012106889.2	TAF1	mRNA	TCONS_00068429	lncRNA
XM_012106889.2	TAF1	mRNA	TCONS_00068429	lncRNA
XM_012106889.2	TAF1	mRNA	TCONS_00068429	lncRNA
XM_012176829.1	BBS9	mRNA	XR_001024966.2	lncRNA
XM_012176829.1	BBS9	mRNA	XR_001024966.2	lncRNA
XM_012176829.1	BBS9	mRNA	XR_001024966.2	lncRNA
XM_012102077.2	LGALS12	mRNA	XR_001023303.2	lncRNA
XM_012102077.2	LGALS12	mRNA	XR_001023303.2	lncRNA
XM_012102077.2	LGALS12	mRNA	XR_001023303.2	lncRNA
XM_012176829.1	BBS9	mRNA	XR_001023303.2	lncRNA
XM_012176829.1	BBS9	mRNA	XR_001023303.2	lncRNA
XM_012176829.1	BBS9	mRNA	XR_001023303.2	lncRNA
XM_012106234.2	OFD1	mRNA	XR_001023303.2	lncRNA
XM_012106234.2	OFD1	mRNA	XR_001023303.2	lncRNA
XM_012106234.2	OFD1	mRNA	XR_001023303.2	lncRNA
XM_012161705.2	LRRC8A	mRNA	XR_001041893.2	lncRNA
XM_012161705.2	LRRC8A	mRNA	XR_001041893.2	lncRNA
XM_012161705.2	LRRC8A	mRNA	XR_001041893.2	lncRNA
XM_012180708.1	PAPD4	mRNA	XR_001023303.2	lncRNA
XM_012180708.1	PAPD4	mRNA	XR_001023303.2	lncRNA
XM_012180708.1	PAPD4	mRNA	XR_001023303.2	lncRNA
XM_004012193.3	RBM26	mRNA	XR_001023303.2	lncRNA
XM_004012193.3	RBM26	mRNA	XR_001023303.2	lncRNA
XM_004012193.3	RBM26	mRNA	XR_001023303.2	lncRNA
XM_012183459.1	ISG20L2	mRNA	XR_001023303.2	lncRNA
XM_012183459.1	ISG20L2	mRNA	XR_001023303.2	lncRNA

XM_012183459.1	ISG20L2	mRNA	XR_001023303.2	lncRNA
XM_012179573.2	PPA2	mRNA	XR_001041893.2	lncRNA
XM_012179573.2	PPA2	mRNA	XR_001041893.2	lncRNA
XM_012179573.2	PPA2	mRNA	XR_001041893.2	lncRNA
XM_012106234.2	OFD1	mRNA	XR_001024966.2	lncRNA
XM_012106234.2	OFD1	mRNA	XR_001024966.2	lncRNA
XM_012106234.2	OFD1	mRNA	XR_001024966.2	lncRNA
XM_012102636.2	TCTN3	mRNA	XR_001041893.2	lncRNA
XM_012102636.2	TCTN3	mRNA	XR_001041893.2	lncRNA
XM_012102636.2	TCTN3	mRNA	XR_001041893.2	lncRNA
XM_012184401.1	ZC3H13	mRNA	XR_001041893.2	lncRNA
XM_012184401.1	ZC3H13	mRNA	XR_001041893.2	lncRNA
XM_012184401.1	ZC3H13	mRNA	XR_001041893.2	lncRNA
XM_015098987.1	ABCA5	mRNA	XR_001023303.2	lncRNA
XM_015098987.1	ABCA5	mRNA	XR_001023303.2	lncRNA
XM_015098987.1	ABCA5	mRNA	XR_001023303.2	lncRNA
XM_012102598.2	TBC1D12	mRNA	XR_001023303.2	lncRNA
XM_012102598.2	TBC1D12	mRNA	XR_001023303.2	lncRNA
XM_012102598.2	TBC1D12	mRNA	XR_001023303.2	lncRNA
XM_004014588.3	PKIG	mRNA	XR_001024966.2	lncRNA
XM_004014588.3	PKIG	mRNA	XR_001024966.2	lncRNA
XM_004014588.3	PKIG	mRNA	XR_001024966.2	lncRNA
XM_012174597.2	NUP107	mRNA	XR_001023303.2	lncRNA
XM_012174597.2	NUP107	mRNA	XR_001023303.2	lncRNA
XM_012174597.2	NUP107	mRNA	XR_001023303.2	lncRNA
XM_012106815.2	RBM10	mRNA	XR_001024966.2	lncRNA
XM_012106815.2	RBM10	mRNA	XR_001024966.2	lncRNA
XM_012106815.2	RBM10	mRNA	XR_001024966.2	lncRNA
XM_015100523.1	NFAT5	mRNA	XR_001022883.1	lncRNA
XM_015100523.1	NFAT5	mRNA	XR_001022883.1	lncRNA
XM_015100523.1	NFAT5	mRNA	XR_001022883.1	lncRNA
XM_012189777.1	CHD9	mRNA	XR_001022883.1	lncRNA
XM_012189777.1	CHD9	mRNA	XR_001022883.1	lncRNA
XM_012189777.1	CHD9	mRNA	XR_001022883.1	lncRNA
XM_012189777.1	CHD9	mRNA	TCONS_00056022	lncRNA
XM_012189777.1	CHD9	mRNA	TCONS_00056022	lncRNA
XM_012189777.1	CHD9	mRNA	TCONS_00056022	lncRNA
XM_012098983.2	NAALADL2	mRNA	TCONS_00039112	lncRNA
XM_012098983.2	NAALADL2	mRNA	TCONS_00039112	lncRNA
XM_012098983.2	NAALADL2	mRNA	TCONS_00039112	lncRNA
XM_012179825.2	N4BP2	mRNA	TCONS_00039112	lncRNA
XM_012179825.2	N4BP2	mRNA	TCONS_00039112	lncRNA
XM_012179825.2	N4BP2	mRNA	TCONS_00039112	lncRNA
XM_004007703.3	CDK14	mRNA	TCONS_00049802	lncRNA
XM_004007703.3	CDK14	mRNA	TCONS_00049802	lncRNA
XM_004007703.3	CDK14	mRNA	TCONS_00049802	lncRNA
XM_012178486.2	FSTL4	mRNA	XR_001023627.2	lncRNA
XM_012178486.2	FSTL4	mRNA	XR_001023627.2	lncRNA
XM_012178486.2	FSTL4	mRNA	XR_001023627.2	lncRNA
XM_012176839.2	NT5C3A	mRNA	XR_001434127.1	lncRNA
XM_012176839.2	NT5C3A	mRNA	XR_001434127.1	lncRNA
XM_012176839.2	NT5C3A	mRNA	XR_001434127.1	lncRNA
XM_012176839.2	NT5C3A	mRNA	XR_001023627.2	lncRNA
XM_012176839.2	NT5C3A	mRNA	XR_001023627.2	lncRNA

XM_012176839.2	NT5C3A	mRNA	XR_001023627.2	lncRNA
XM_015094627.1	PCBP2	mRNA	XR_001023627.2	lncRNA
XM_015094627.1	PCBP2	mRNA	XR_001023627.2	lncRNA
XM_015094627.1	PCBP2	mRNA	XR_001023627.2	lncRNA
XM_012165482.2	ITGB3BP	mRNA	TCONS_00049802	lncRNA
XM_012165482.2	ITGB3BP	mRNA	TCONS_00049802	lncRNA
XM_012165482.2	ITGB3BP	mRNA	TCONS_00049802	lncRNA
XM_012178486.2	FSTL4	mRNA	XR_001434127.1	lncRNA
XM_012178486.2	FSTL4	mRNA	XR_001434127.1	lncRNA
XM_012178486.2	FSTL4	mRNA	XR_001434127.1	lncRNA
XM_015104577.1	TUSC3	mRNA	XR_001434127.1	lncRNA
XM_015104577.1	TUSC3	mRNA	XR_001434127.1	lncRNA
XM_015104577.1	TUSC3	mRNA	XR_001434127.1	lncRNA
XM_012180559.2	TARS2	mRNA	TCONS_00049802	lncRNA
XM_012180559.2	TARS2	mRNA	TCONS_00049802	lncRNA
XM_012180559.2	TARS2	mRNA	TCONS_00049802	lncRNA
XM_004007914.3	NT5C3A	mRNA	XR_001023627.2	lncRNA
XM_004007914.3	NT5C3A	mRNA	XR_001023627.2	lncRNA
XM_004007914.3	NT5C3A	mRNA	XR_001023627.2	lncRNA
XM_012186029.1	FBXL20	mRNA	XR_001025540.2	lncRNA
XM_012186029.1	FBXL20	mRNA	XR_001025540.2	lncRNA
XM_012186029.1	FBXL20	mRNA	XR_001025540.2	lncRNA
XM_015092004.1	MROH7	mRNA	XR_001025540.2	lncRNA
XM_015092004.1	MROH7	mRNA	XR_001025540.2	lncRNA
XM_015092004.1	MROH7	mRNA	XR_001025540.2	lncRNA
XM_012100861.1	FBXO9	mRNA	XR_001040147.1	lncRNA
XM_012100861.1	FBXO9	mRNA	XR_001040147.1	lncRNA
XM_012100861.1	FBXO9	mRNA	XR_001040147.1	lncRNA
XM_012101269.2	SIRT5	mRNA	XR_001436390.1	lncRNA
XM_012101269.2	SIRT5	mRNA	XR_001436390.1	lncRNA
XM_012101269.2	SIRT5	mRNA	XR_001436390.1	lncRNA
XM_015099603.1	KIF5B	mRNA	XR_001040147.1	lncRNA
XM_015099603.1	KIF5B	mRNA	XR_001040147.1	lncRNA
XM_015099603.1	KIF5B	mRNA	XR_001040147.1	lncRNA
XM_004007914.3	NT5C3A	mRNA	XR_001040147.1	lncRNA
XM_004007914.3	NT5C3A	mRNA	XR_001040147.1	lncRNA
XM_004007914.3	NT5C3A	mRNA	XR_001040147.1	lncRNA
XM_004003396.3	SETD4	mRNA	XR_001436390.1	lncRNA
XM_004003396.3	SETD4	mRNA	XR_001436390.1	lncRNA
XM_004003396.3	SETD4	mRNA	XR_001436390.1	lncRNA
XM_012180559.2	TARS2	mRNA	XR_001436390.1	lncRNA
XM_012180559.2	TARS2	mRNA	XR_001436390.1	lncRNA
XM_012180559.2	TARS2	mRNA	XR_001436390.1	lncRNA
XM_012100861.1	FBXO9	mRNA	XR_001436390.1	lncRNA
XM_012100861.1	FBXO9	mRNA	XR_001436390.1	lncRNA
XM_012100861.1	FBXO9	mRNA	XR_001436390.1	lncRNA
XM_004018866.3	LOC101120701	mRNA	XR_001436390.1	lncRNA
XM_004018866.3	LOC101120701	mRNA	XR_001436390.1	lncRNA
XM_004018866.3	LOC101120701	mRNA	XR_001436390.1	lncRNA
XM_004007913.3	NT5C3A	mRNA	XR_001040147.1	lncRNA
XM_004007913.3	NT5C3A	mRNA	XR_001040147.1	lncRNA
XM_004007913.3	NT5C3A	mRNA	XR_001040147.1	lncRNA
XM_012181094.1	TTC5	mRNA	XR_001436390.1	lncRNA
XM_012181094.1	TTC5	mRNA	XR_001436390.1	lncRNA

XM_012181094.1	TTC5	mRNA	XR_001436390.1	lncRNA
XM_004003396.3	SETD4	mRNA	XR_001040147.1	lncRNA
XM_004003396.3	SETD4	mRNA	XR_001040147.1	lncRNA
XM_004003396.3	SETD4	mRNA	XR_001040147.1	lncRNA
XM_012098852.2	CTAGE5	mRNA	XR_001436390.1	lncRNA
XM_012098852.2	CTAGE5	mRNA	XR_001436390.1	lncRNA
XM_012098852.2	CTAGE5	mRNA	XR_001436390.1	lncRNA
XM_015093485.1	ORC2	mRNA	XR_001436390.1	lncRNA
XM_015093485.1	ORC2	mRNA	XR_001436390.1	lncRNA
XM_015093485.1	ORC2	mRNA	XR_001436390.1	lncRNA
XM_012182192.2	EML5	mRNA	TCONS_00056022	lncRNA
XM_012182192.2	EML5	mRNA	TCONS_00056022	lncRNA
XM_012182192.2	EML5	mRNA	TCONS_00056022	lncRNA
XM_012188348.1	KIAA1217	mRNA	TCONS_00056022	lncRNA
XM_012188348.1	KIAA1217	mRNA	TCONS_00056022	lncRNA
XM_012188348.1	KIAA1217	mRNA	TCONS_00056022	lncRNA
XM_015099563.1	PARD3	mRNA	XR_001022883.1	lncRNA
XM_015099563.1	PARD3	mRNA	XR_001022883.1	lncRNA
XM_015099563.1	PARD3	mRNA	XR_001022883.1	lncRNA
XM_015104838.1	PDK3	mRNA	TCONS_00039112	lncRNA
XM_015104838.1	PDK3	mRNA	TCONS_00039112	lncRNA
XM_015104838.1	PDK3	mRNA	TCONS_00039112	lncRNA
XM_012185875.1	LUC7L3	mRNA	TCONS_00068429	lncRNA
XM_012185875.1	LUC7L3	mRNA	TCONS_00068429	lncRNA
XM_012185875.1	LUC7L3	mRNA	TCONS_00068429	lncRNA
XM_012178221.1	CEP120	mRNA	XR_001024290.1	lncRNA
XM_012178221.1	CEP120	mRNA	XR_001024290.1	lncRNA
XM_012178221.1	CEP120	mRNA	XR_001024290.1	lncRNA
XM_012178157.2	RAPGEF6	mRNA	XR_001041220.2	lncRNA
XM_012178157.2	RAPGEF6	mRNA	XR_001041220.2	lncRNA
XM_012178157.2	RAPGEF6	mRNA	XR_001041220.2	lncRNA
XM_015091887.1	MYCL	mRNA	TCONS_00068429	lncRNA
XM_015091887.1	MYCL	mRNA	TCONS_00068429	lncRNA
XM_015091887.1	MYCL	mRNA	TCONS_00068429	lncRNA
XM_012188917.1	STX16	mRNA	XR_001024290.1	lncRNA
XM_012188917.1	STX16	mRNA	XR_001024290.1	lncRNA
XM_012188917.1	STX16	mRNA	XR_001024290.1	lncRNA
XM_015104323.1	SPRTN	mRNA	TCONS_00068429	lncRNA
XM_015104323.1	SPRTN	mRNA	TCONS_00068429	lncRNA
XM_015104323.1	SPRTN	mRNA	TCONS_00068429	lncRNA
XM_012102667.1	ZNF518A	mRNA	XR_001433837.1	lncRNA
XM_012102667.1	ZNF518A	mRNA	XR_001433837.1	lncRNA
XM_012102667.1	ZNF518A	mRNA	XR_001433837.1	lncRNA
XM_012177185.2	CNOT4	mRNA	XR_001433837.1	lncRNA
XM_012177185.2	CNOT4	mRNA	XR_001433837.1	lncRNA
XM_012177185.2	CNOT4	mRNA	XR_001433837.1	lncRNA
XM_015099548.1	PARD3	mRNA	XR_001022883.1	lncRNA
XM_015099548.1	PARD3	mRNA	XR_001022883.1	lncRNA
XM_015099548.1	PARD3	mRNA	XR_001022883.1	lncRNA
XM_012137599.2	NEB	mRNA	TCONS_00039112	lncRNA
XM_012137599.2	NEB	mRNA	TCONS_00039112	lncRNA
XM_012137599.2	NEB	mRNA	TCONS_00039112	lncRNA
XM_015094531.1	ANAPC1	mRNA	XR_001022883.1	lncRNA
XM_015094531.1	ANAPC1	mRNA	XR_001022883.1	lncRNA

XM_015094531.1	ANAPC1	mRNA	XR_001022883.1	lncRNA
XM_015094531.1	ANAPC1	mRNA	TCONS_00056022	lncRNA
XM_015094531.1	ANAPC1	mRNA	TCONS_00056022	lncRNA
XM_015094531.1	ANAPC1	mRNA	TCONS_00056022	lncRNA
XM_004012101.3	FREM2	mRNA	XR_001022883.1	lncRNA
XM_004012101.3	FREM2	mRNA	XR_001022883.1	lncRNA
XM_004012101.3	FREM2	mRNA	XR_001022883.1	lncRNA
XM_012095602.2	USP2	mRNA	XR_001025540.2	lncRNA
XM_012095602.2	USP2	mRNA	XR_001025540.2	lncRNA
XM_012095602.2	USP2	mRNA	XR_001025540.2	lncRNA
XM_015096518.1	AASDH	mRNA	XR_001025540.2	lncRNA
XM_015096518.1	AASDH	mRNA	XR_001025540.2	lncRNA
XM_015096518.1	AASDH	mRNA	XR_001025540.2	lncRNA
XM_015103688.1	PTPN2	mRNA	XR_001023303.2	lncRNA
XM_015103688.1	PTPN2	mRNA	XR_001023303.2	lncRNA
XM_015103688.1	PTPN2	mRNA	XR_001023303.2	lncRNA
XM_012176469.2	MACC1	mRNA	XR_001023303.2	lncRNA
XM_012176469.2	MACC1	mRNA	XR_001023303.2	lncRNA
XM_012176469.2	MACC1	mRNA	XR_001023303.2	lncRNA
XM_012176656.2	NAPEPLD	mRNA	XR_001041893.2	lncRNA
XM_012176656.2	NAPEPLD	mRNA	XR_001041893.2	lncRNA
XM_012176656.2	NAPEPLD	mRNA	XR_001041893.2	lncRNA
XM_004004866.2	METTL21A	mRNA	XR_001023303.2	lncRNA
XM_004004866.2	METTL21A	mRNA	XR_001023303.2	lncRNA
XM_004004866.2	METTL21A	mRNA	XR_001023303.2	lncRNA
XM_012174737.2	PRKACB	mRNA	XR_001023303.2	lncRNA
XM_012174737.2	PRKACB	mRNA	XR_001023303.2	lncRNA
XM_012174737.2	PRKACB	mRNA	XR_001023303.2	lncRNA
XM_012102930.1	GPAM	mRNA	XR_001023303.2	lncRNA
XM_012102930.1	GPAM	mRNA	XR_001023303.2	lncRNA
XM_012102930.1	GPAM	mRNA	XR_001023303.2	lncRNA
XM_012097252.2	RHOF	mRNA	XR_001024966.2	lncRNA
XM_012097252.2	RHOF	mRNA	XR_001024966.2	lncRNA
XM_012097252.2	RHOF	mRNA	XR_001024966.2	lncRNA
XM_015094585.1	ATP2B1	mRNA	XR_001023303.2	lncRNA
XM_015094585.1	ATP2B1	mRNA	XR_001023303.2	lncRNA
XM_015094585.1	ATP2B1	mRNA	XR_001023303.2	lncRNA
XM_015098186.1	XPO4	mRNA	XR_001024966.2	lncRNA
XM_015098186.1	XPO4	mRNA	XR_001024966.2	lncRNA
XM_015098186.1	XPO4	mRNA	XR_001024966.2	lncRNA
XM_015096221.1	PPIP5K2	mRNA	XR_001024966.2	lncRNA
XM_015096221.1	PPIP5K2	mRNA	XR_001024966.2	lncRNA
XM_015096221.1	PPIP5K2	mRNA	XR_001024966.2	lncRNA
XM_015094919.1	WBP11	mRNA	XR_001041893.2	lncRNA
XM_015094919.1	WBP11	mRNA	XR_001041893.2	lncRNA
XM_015094919.1	WBP11	mRNA	XR_001041893.2	lncRNA
XM_012101006.2	CCHCR1	mRNA	XR_001023303.2	lncRNA
XM_012101006.2	CCHCR1	mRNA	XR_001023303.2	lncRNA
XM_012101006.2	CCHCR1	mRNA	XR_001023303.2	lncRNA
XM_015096456.1	APBB2	mRNA	XR_001041893.2	lncRNA
XM_015096456.1	APBB2	mRNA	XR_001041893.2	lncRNA
XM_015096456.1	APBB2	mRNA	XR_001041893.2	lncRNA
XM_012140245.2	RALB	mRNA	XR_001041893.2	lncRNA
XM_012140245.2	RALB	mRNA	XR_001041893.2	lncRNA

XM_012140245.2	RALB	mRNA	XR_001041893.2	lncRNA
XM_012174722.2	PRKACB	mRNA	XR_001023303.2	lncRNA
XM_012174722.2	PRKACB	mRNA	XR_001023303.2	lncRNA
XM_012174722.2	PRKACB	mRNA	XR_001023303.2	lncRNA
XM_012106112.2	ARAF	mRNA	XR_001023303.2	lncRNA
XM_012106112.2	ARAF	mRNA	XR_001023303.2	lncRNA
XM_012106112.2	ARAF	mRNA	XR_001023303.2	lncRNA
XM_012095761.2	HSPBAP1	mRNA	XR_001024966.2	lncRNA
XM_012095761.2	HSPBAP1	mRNA	XR_001024966.2	lncRNA
XM_012095761.2	HSPBAP1	mRNA	XR_001024966.2	lncRNA
XM_004014289.3	ZNF133	mRNA	XR_001024966.2	lncRNA
XM_004014289.3	ZNF133	mRNA	XR_001024966.2	lncRNA
XM_004014289.3	ZNF133	mRNA	XR_001024966.2	lncRNA
XM_012174423.2	SLC38A4	mRNA	XR_001041893.2	lncRNA
XM_012174423.2	SLC38A4	mRNA	XR_001041893.2	lncRNA
XM_012174423.2	SLC38A4	mRNA	XR_001041893.2	lncRNA
XM_012106892.2	TAF1	mRNA	XR_001023303.2	lncRNA
XM_012106892.2	TAF1	mRNA	XR_001023303.2	lncRNA
XM_012106892.2	TAF1	mRNA	XR_001023303.2	lncRNA
XM_012097504.1	MFSD8	mRNA	XR_001024966.2	lncRNA
XM_012097504.1	MFSD8	mRNA	XR_001024966.2	lncRNA
XM_012097504.1	MFSD8	mRNA	XR_001024966.2	lncRNA
XM_015097819.1	STAU2	mRNA	XR_001024966.2	lncRNA
XM_015097819.1	STAU2	mRNA	XR_001024966.2	lncRNA
XM_015097819.1	STAU2	mRNA	XR_001024966.2	lncRNA
XM_012165482.2	ITGB3BP	mRNA	XR_001024230.1	lncRNA
XM_012165482.2	ITGB3BP	mRNA	XR_001024230.1	lncRNA
XM_012165482.2	ITGB3BP	mRNA	XR_001024230.1	lncRNA
XM_012180559.2	TARS2	mRNA	XR_001436064.1	lncRNA
XM_012180559.2	TARS2	mRNA	XR_001436064.1	lncRNA
XM_012180559.2	TARS2	mRNA	XR_001436064.1	lncRNA
XM_012165482.2	ITGB3BP	mRNA	XR_001436065.1	lncRNA
XM_012165482.2	ITGB3BP	mRNA	XR_001436065.1	lncRNA
XM_012165482.2	ITGB3BP	mRNA	XR_001436065.1	lncRNA
XM_012178486.2	FSTL4	mRNA	XR_001435723.1	lncRNA
XM_012178486.2	FSTL4	mRNA	XR_001435723.1	lncRNA
XM_012178486.2	FSTL4	mRNA	XR_001435723.1	lncRNA
XM_004007703.3	CDK14	mRNA	XR_001436064.1	lncRNA
XM_004007703.3	CDK14	mRNA	XR_001436064.1	lncRNA
XM_004007703.3	CDK14	mRNA	XR_001436064.1	lncRNA
XM_015104577.1	TUSC3	mRNA	XR_001436064.1	lncRNA
XM_015104577.1	TUSC3	mRNA	XR_001436064.1	lncRNA
XM_015104577.1	TUSC3	mRNA	XR_001436064.1	lncRNA
XM_004018866.3	LOC101120701	mRNA	XR_001435723.1	lncRNA
XM_004018866.3	LOC101120701	mRNA	XR_001435723.1	lncRNA
XM_004018866.3	LOC101120701	mRNA	XR_001435723.1	lncRNA
XM_012106701.2	CCNB3	mRNA	XR_001435723.1	lncRNA
XM_012106701.2	CCNB3	mRNA	XR_001435723.1	lncRNA
XM_012106701.2	CCNB3	mRNA	XR_001435723.1	lncRNA
XM_015093485.1	ORC2	mRNA	XR_001435723.1	lncRNA
XM_015093485.1	ORC2	mRNA	XR_001435723.1	lncRNA
XM_015093485.1	ORC2	mRNA	XR_001435723.1	lncRNA
XM_012105261.2	MCU	mRNA	XR_001435723.1	lncRNA
XM_012105261.2	MCU	mRNA	XR_001435723.1	lncRNA

XM_012105261.2	MCU	mRNA	XR_001435723.1	lncRNA
XM_004012186.3	KLF5	mRNA	XR_001024230.1	lncRNA
XM_004012186.3	KLF5	mRNA	XR_001024230.1	lncRNA
XM_004012186.3	KLF5	mRNA	XR_001024230.1	lncRNA
XM_015094627.1	PCBP2	mRNA	XR_001024230.1	lncRNA
XM_015094627.1	PCBP2	mRNA	XR_001024230.1	lncRNA
XM_015094627.1	PCBP2	mRNA	XR_001024230.1	lncRNA
XM_004006568.3	HSD17B6	mRNA	XR_001435723.1	lncRNA
XM_004006568.3	HSD17B6	mRNA	XR_001435723.1	lncRNA
XM_004006568.3	HSD17B6	mRNA	XR_001435723.1	lncRNA
XM_012180559.2	TARS2	mRNA	XR_001024230.1	lncRNA
XM_012180559.2	TARS2	mRNA	XR_001024230.1	lncRNA
XM_012180559.2	TARS2	mRNA	XR_001024230.1	lncRNA
XM_015098807.1	ETV4	mRNA	XR_001435723.1	lncRNA
XM_015098807.1	ETV4	mRNA	XR_001435723.1	lncRNA
XM_015098807.1	ETV4	mRNA	XR_001435723.1	lncRNA
XM_012178486.2	FSTL4	mRNA	XR_001024230.1	lncRNA
XM_012178486.2	FSTL4	mRNA	XR_001024230.1	lncRNA
XM_012178486.2	FSTL4	mRNA	XR_001024230.1	lncRNA
XM_012167069.2	PLB1	mRNA	XR_001435723.1	lncRNA
XM_012167069.2	PLB1	mRNA	XR_001435723.1	lncRNA
XM_012167069.2	PLB1	mRNA	XR_001435723.1	lncRNA
XM_004005618.3	FPGS	mRNA	XR_001435723.1	lncRNA
XM_004005618.3	FPGS	mRNA	XR_001435723.1	lncRNA
XM_004005618.3	FPGS	mRNA	XR_001435723.1	lncRNA
XM_012106701.2	CCNB3	mRNA	XR_001024230.1	lncRNA
XM_012106701.2	CCNB3	mRNA	XR_001024230.1	lncRNA
XM_012106701.2	CCNB3	mRNA	XR_001024230.1	lncRNA
NM_001202421.1	NUDT9	mRNA	XR_001435723.1	lncRNA
NM_001202421.1	NUDT9	mRNA	XR_001435723.1	lncRNA
NM_001202421.1	NUDT9	mRNA	XR_001435723.1	lncRNA
XM_012176839.2	NT5C3A	mRNA	XR_001024230.1	lncRNA
XM_012176839.2	NT5C3A	mRNA	XR_001024230.1	lncRNA
XM_012176839.2	NT5C3A	mRNA	XR_001024230.1	lncRNA
XM_012105261.2	MCU	mRNA	XR_001024230.1	lncRNA
XM_012105261.2	MCU	mRNA	XR_001024230.1	lncRNA
XM_012105261.2	MCU	mRNA	XR_001024230.1	lncRNA
XM_015094627.1	PCBP2	mRNA	XR_001435723.1	lncRNA
XM_015094627.1	PCBP2	mRNA	XR_001435723.1	lncRNA
XM_015094627.1	PCBP2	mRNA	XR_001435723.1	lncRNA
XM_012101269.2	SIRT5	mRNA	XR_001435723.1	lncRNA
XM_012101269.2	SIRT5	mRNA	XR_001435723.1	lncRNA
XM_012101269.2	SIRT5	mRNA	XR_001435723.1	lncRNA
XM_004006568.3	HSD17B6	mRNA	XR_001024230.1	lncRNA
XM_004006568.3	HSD17B6	mRNA	XR_001024230.1	lncRNA
XM_004006568.3	HSD17B6	mRNA	XR_001024230.1	lncRNA
XM_004012186.3	KLF5	mRNA	XR_001435723.1	lncRNA
XM_004012186.3	KLF5	mRNA	XR_001435723.1	lncRNA
XM_004012186.3	KLF5	mRNA	XR_001435723.1	lncRNA
XM_004012600.2	CAMTA2	mRNA	XR_001435723.1	lncRNA
XM_004012600.2	CAMTA2	mRNA	XR_001435723.1	lncRNA
XM_004012600.2	CAMTA2	mRNA	XR_001435723.1	lncRNA
XM_004007914.3	NT5C3A	mRNA	XR_001024230.1	lncRNA
XM_004007914.3	NT5C3A	mRNA	XR_001024230.1	lncRNA

XM_004007914.3	NT5C3A	mRNA	XR_001024230.1	lncRNA
XM_012165482.2	ITGB3BP	mRNA	XR_001435723.1	lncRNA
XM_012165482.2	ITGB3BP	mRNA	XR_001435723.1	lncRNA
XM_012165482.2	ITGB3BP	mRNA	XR_001435723.1	lncRNA
XM_012180559.2	TARS2	mRNA	XR_001435723.1	lncRNA
XM_012180559.2	TARS2	mRNA	XR_001435723.1	lncRNA
XM_012180559.2	TARS2	mRNA	XR_001435723.1	lncRNA
XM_004007703.3	CDK14	mRNA	XR_001436065.1	lncRNA
XM_004007703.3	CDK14	mRNA	XR_001436065.1	lncRNA
XM_004007703.3	CDK14	mRNA	XR_001436065.1	lncRNA
XM_012101269.2	SIRT5	mRNA	XR_001435726.1	lncRNA
XM_012101269.2	SIRT5	mRNA	XR_001435726.1	lncRNA
XM_012101269.2	SIRT5	mRNA	XR_001435726.1	lncRNA
XM_015104577.1	TUSC3	mRNA	XR_001436065.1	lncRNA
XM_015104577.1	TUSC3	mRNA	XR_001436065.1	lncRNA
XM_015104577.1	TUSC3	mRNA	XR_001436065.1	lncRNA
XM_015097727.1	FAM49B	mRNA	XR_001436065.1	lncRNA
XM_015097727.1	FAM49B	mRNA	XR_001436065.1	lncRNA
XM_015097727.1	FAM49B	mRNA	XR_001436065.1	lncRNA
XM_012178937.1	TMEM161B	mRNA	XR_001436065.1	lncRNA
XM_012178937.1	TMEM161B	mRNA	XR_001436065.1	lncRNA
XM_012178937.1	TMEM161B	mRNA	XR_001436065.1	lncRNA
XM_012178486.2	FSTL4	mRNA	XR_001435726.1	lncRNA
XM_012178486.2	FSTL4	mRNA	XR_001435726.1	lncRNA
XM_012178486.2	FSTL4	mRNA	XR_001435726.1	lncRNA
XM_012124491.2	AK3	mRNA	XR_001435726.1	lncRNA
XM_012124491.2	AK3	mRNA	XR_001435726.1	lncRNA
XM_012124491.2	AK3	mRNA	XR_001435726.1	lncRNA
XM_012105261.2	MCU	mRNA	XR_001435726.1	lncRNA
XM_012105261.2	MCU	mRNA	XR_001435726.1	lncRNA
XM_012105261.2	MCU	mRNA	XR_001435726.1	lncRNA
XM_015093485.1	ORC2	mRNA	XR_001435726.1	lncRNA
XM_015093485.1	ORC2	mRNA	XR_001435726.1	lncRNA
XM_015093485.1	ORC2	mRNA	XR_001435726.1	lncRNA
XM_004006568.3	HSD17B6	mRNA	XR_001435726.1	lncRNA
XM_004006568.3	HSD17B6	mRNA	XR_001435726.1	lncRNA
XM_004006568.3	HSD17B6	mRNA	XR_001435726.1	lncRNA
NM_001202421.1	NUDT9	mRNA	XR_001435726.1	lncRNA
NM_001202421.1	NUDT9	mRNA	XR_001435726.1	lncRNA
NM_001202421.1	NUDT9	mRNA	XR_001435726.1	lncRNA
XM_004005618.3	FPGS	mRNA	XR_001435726.1	lncRNA
XM_004005618.3	FPGS	mRNA	XR_001435726.1	lncRNA
XM_004005618.3	FPGS	mRNA	XR_001435726.1	lncRNA
XM_012167069.2	PLB1	mRNA	XR_001435726.1	lncRNA
XM_012167069.2	PLB1	mRNA	XR_001435726.1	lncRNA
XM_012167069.2	PLB1	mRNA	XR_001435726.1	lncRNA
XM_004012186.3	KLF5	mRNA	XR_001435726.1	lncRNA
XM_004012186.3	KLF5	mRNA	XR_001435726.1	lncRNA
XM_004012186.3	KLF5	mRNA	XR_001435726.1	lncRNA
XM_015098807.1	ETV4	mRNA	XR_001435726.1	lncRNA
XM_015098807.1	ETV4	mRNA	XR_001435726.1	lncRNA
XM_015098807.1	ETV4	mRNA	XR_001435726.1	lncRNA
XM_004003396.3	SETD4	mRNA	XR_001435726.1	lncRNA
XM_004003396.3	SETD4	mRNA	XR_001435726.1	lncRNA

XM_004003396.3	SETD4	mRNA	XR_001435726.1	lncRNA
XM_004012600.2	CAMTA2	mRNA	XR_001435726.1	lncRNA
XM_004012600.2	CAMTA2	mRNA	XR_001435726.1	lncRNA
XM_004012600.2	CAMTA2	mRNA	XR_001435726.1	lncRNA
XM_012162993.2	GOLGA1	mRNA	XR_001435726.1	lncRNA
XM_012162993.2	GOLGA1	mRNA	XR_001435726.1	lncRNA
XM_012162993.2	GOLGA1	mRNA	XR_001435726.1	lncRNA
XM_004018866.3	LOC101120701	mRNA	XR_001435726.1	lncRNA
XM_004018866.3	LOC101120701	mRNA	XR_001435726.1	lncRNA
XM_004018866.3	LOC101120701	mRNA	XR_001435726.1	lncRNA
XM_012180559.2	TARS2	mRNA	XR_001436065.1	lncRNA
XM_012180559.2	TARS2	mRNA	XR_001436065.1	lncRNA
XM_012180559.2	TARS2	mRNA	XR_001436065.1	lncRNA
XM_012165482.2	ITGB3BP	mRNA	XR_001436064.1	lncRNA
XM_012165482.2	ITGB3BP	mRNA	XR_001436064.1	lncRNA
XM_012165482.2	ITGB3BP	mRNA	XR_001436064.1	lncRNA
XM_012176839.2	NT5C3A	mRNA	XR_001436064.1	lncRNA
XM_012176839.2	NT5C3A	mRNA	XR_001436064.1	lncRNA
XM_012176839.2	NT5C3A	mRNA	XR_001436064.1	lncRNA
XM_012176839.2	NT5C3A	mRNA	XR_001436065.1	lncRNA
XM_012176839.2	NT5C3A	mRNA	XR_001436065.1	lncRNA
XM_012176839.2	NT5C3A	mRNA	XR_001436065.1	lncRNA
XM_012105261.2	MCU	mRNA	XR_001436064.1	lncRNA
XM_012105261.2	MCU	mRNA	XR_001436064.1	lncRNA
XM_012105261.2	MCU	mRNA	XR_001436064.1	lncRNA
XM_012180559.2	TARS2	mRNA	XR_001435726.1	lncRNA
XM_012180559.2	TARS2	mRNA	XR_001435726.1	lncRNA
XM_012180559.2	TARS2	mRNA	XR_001435726.1	lncRNA
NM_001202421.1	NUDT9	mRNA	XR_001436064.1	lncRNA
NM_001202421.1	NUDT9	mRNA	XR_001436064.1	lncRNA
NM_001202421.1	NUDT9	mRNA	XR_001436064.1	lncRNA
XM_012187926.2	C12H1orf95	mRNA	XR_001436064.1	lncRNA
XM_012187926.2	C12H1orf95	mRNA	XR_001436064.1	lncRNA
XM_012187926.2	C12H1orf95	mRNA	XR_001436064.1	lncRNA
XM_012179472.2	ANK2	mRNA	TCONS_00039112	lncRNA
XM_012179472.2	ANK2	mRNA	TCONS_00039112	lncRNA
XM_012179472.2	ANK2	mRNA	TCONS_00039112	lncRNA
XM_012186362.1	PITPNC1	mRNA	XR_001022883.1	lncRNA
XM_012186362.1	PITPNC1	mRNA	XR_001022883.1	lncRNA
XM_012186362.1	PITPNC1	mRNA	XR_001022883.1	lncRNA
XM_012104222.2	DCUN1D3	mRNA	XR_001436612.1	lncRNA
XM_012104222.2	DCUN1D3	mRNA	XR_001436612.1	lncRNA
XM_012104222.2	DCUN1D3	mRNA	XR_001436612.1	lncRNA
XM_012102598.2	TBC1D12	mRNA	XR_001436612.1	lncRNA
XM_012102598.2	TBC1D12	mRNA	XR_001436612.1	lncRNA
XM_012102598.2	TBC1D12	mRNA	XR_001436612.1	lncRNA
XM_012174597.2	NUP107	mRNA	XR_001436612.1	lncRNA
XM_012174597.2	NUP107	mRNA	XR_001436612.1	lncRNA
XM_012174597.2	NUP107	mRNA	XR_001436612.1	lncRNA
XM_012190351.2	ZNF420	mRNA	XR_001436612.1	lncRNA
XM_012190351.2	ZNF420	mRNA	XR_001436612.1	lncRNA
XM_012190351.2	ZNF420	mRNA	XR_001436612.1	lncRNA
XM_015096209.1	SPATA9	mRNA	XR_001436612.1	lncRNA
XM_015096209.1	SPATA9	mRNA	XR_001436612.1	lncRNA

XM_015096209.1	SPATA9	mRNA	XR_001436612.1	lncRNA
XM_012188419.2	LOC101108937	mRNA	XR_001436612.1	lncRNA
XM_012188419.2	LOC101108937	mRNA	XR_001436612.1	lncRNA
XM_012188419.2	LOC101108937	mRNA	XR_001436612.1	lncRNA
XM_012174290.1	ATF1	mRNA	XR_001436612.1	lncRNA
XM_012174290.1	ATF1	mRNA	XR_001436612.1	lncRNA
XM_012174290.1	ATF1	mRNA	XR_001436612.1	lncRNA
XM_004020479.3	RBBP8	mRNA	XR_001436612.1	lncRNA
XM_004020479.3	RBBP8	mRNA	XR_001436612.1	lncRNA
XM_004020479.3	RBBP8	mRNA	XR_001436612.1	lncRNA
XM_015099384.1	SYT14	mRNA	XR_001436612.1	lncRNA
XM_015099384.1	SYT14	mRNA	XR_001436612.1	lncRNA
XM_015099384.1	SYT14	mRNA	XR_001436612.1	lncRNA
XM_012106234.2	OFD1	mRNA	XR_001436612.1	lncRNA
XM_012106234.2	OFD1	mRNA	XR_001436612.1	lncRNA
XM_012106234.2	OFD1	mRNA	XR_001436612.1	lncRNA
XM_012170869.2	PAPOLG	mRNA	XR_001024290.1	lncRNA
XM_012170869.2	PAPOLG	mRNA	XR_001024290.1	lncRNA
XM_012170869.2	PAPOLG	mRNA	XR_001024290.1	lncRNA
XM_004017814.3	DNAJA4	mRNA	XR_001024290.1	lncRNA
XM_004017814.3	DNAJA4	mRNA	XR_001024290.1	lncRNA
XM_004017814.3	DNAJA4	mRNA	XR_001024290.1	lncRNA
XM_004017788.3	ZFANL3	mRNA	XR_001041220.2	lncRNA
XM_004017788.3	ZFANL3	mRNA	XR_001041220.2	lncRNA
XM_004017788.3	ZFANL3	mRNA	XR_001041220.2	lncRNA
XM_004003770.3	IMPG2	mRNA	TCONS_00068429	lncRNA
XM_004003770.3	IMPG2	mRNA	TCONS_00068429	lncRNA
XM_004003770.3	IMPG2	mRNA	TCONS_00068429	lncRNA
XM_012094911.2	ZNF135	mRNA	XR_001434920.1	lncRNA
XM_012094911.2	ZNF135	mRNA	XR_001434920.1	lncRNA
XM_012094911.2	ZNF135	mRNA	XR_001434920.1	lncRNA
XM_012187091.1	BROX	mRNA	XR_001434920.1	lncRNA
XM_012187091.1	BROX	mRNA	XR_001434920.1	lncRNA
XM_012187091.1	BROX	mRNA	XR_001434920.1	lncRNA
XM_015098807.1	ETV4	mRNA	XR_001435107.1	lncRNA
XM_015098807.1	ETV4	mRNA	XR_001435107.1	lncRNA
XM_015098807.1	ETV4	mRNA	XR_001435107.1	lncRNA
XM_012181772.2	PCNXL4	mRNA	XR_001435107.1	lncRNA
XM_012181772.2	PCNXL4	mRNA	XR_001435107.1	lncRNA
XM_012181772.2	PCNXL4	mRNA	XR_001435107.1	lncRNA
XM_012100861.1	FBXO9	mRNA	XR_001435107.1	lncRNA
XM_012100861.1	FBXO9	mRNA	XR_001435107.1	lncRNA
XM_012100861.1	FBXO9	mRNA	XR_001435107.1	lncRNA
XM_012181772.2	PCNXL4	mRNA	XR_001434920.1	lncRNA
XM_012181772.2	PCNXL4	mRNA	XR_001434920.1	lncRNA
XM_012181772.2	PCNXL4	mRNA	XR_001434920.1	lncRNA
XM_012119403.2	BIN3	mRNA	XR_001434920.1	lncRNA
XM_012119403.2	BIN3	mRNA	XR_001434920.1	lncRNA
XM_012119403.2	BIN3	mRNA	XR_001434920.1	lncRNA
XM_012100861.1	FBXO9	mRNA	XR_001434920.1	lncRNA
XM_012100861.1	FBXO9	mRNA	XR_001434920.1	lncRNA
XM_012100861.1	FBXO9	mRNA	XR_001434920.1	lncRNA
XM_004003396.3	SETD4	mRNA	XR_001434920.1	lncRNA
XM_004003396.3	SETD4	mRNA	XR_001434920.1	lncRNA

XM_004003396.3	SETD4	mRNA	XR_001434920.1	lncRNA
XM_015098878.1	CEP95	mRNA	XR_001433856.1	lncRNA
XM_015098878.1	CEP95	mRNA	XR_001433856.1	lncRNA
XM_015098878.1	CEP95	mRNA	XR_001433856.1	lncRNA
XM_012099283.2	NEK10	mRNA	XR_001433856.1	lncRNA
XM_012099283.2	NEK10	mRNA	XR_001433856.1	lncRNA
XM_012099283.2	NEK10	mRNA	XR_001433856.1	lncRNA
XM_004018866.3	LOC101120701	mRNA	XR_001433856.1	lncRNA
XM_004018866.3	LOC101120701	mRNA	XR_001433856.1	lncRNA
XM_004018866.3	LOC101120701	mRNA	XR_001433856.1	lncRNA
XM_012184143.2	TRIQK	mRNA	XR_001435107.1	lncRNA
XM_012184143.2	TRIQK	mRNA	XR_001435107.1	lncRNA
XM_012184143.2	TRIQK	mRNA	XR_001435107.1	lncRNA
XM_012187091.1	BROX	mRNA	XR_001435107.1	lncRNA
XM_012187091.1	BROX	mRNA	XR_001435107.1	lncRNA
XM_012187091.1	BROX	mRNA	XR_001435107.1	lncRNA
XM_004007913.3	NT5C3A	mRNA	XR_001435107.1	lncRNA
XM_004007913.3	NT5C3A	mRNA	XR_001435107.1	lncRNA
XM_004007913.3	NT5C3A	mRNA	XR_001435107.1	lncRNA
XM_015099603.1	KIF5B	mRNA	XR_001435107.1	lncRNA
XM_015099603.1	KIF5B	mRNA	XR_001435107.1	lncRNA
XM_015099603.1	KIF5B	mRNA	XR_001435107.1	lncRNA
XM_004007914.3	NT5C3A	mRNA	XR_001435107.1	lncRNA
XM_004007914.3	NT5C3A	mRNA	XR_001435107.1	lncRNA
XM_004007914.3	NT5C3A	mRNA	XR_001435107.1	lncRNA
XM_015093485.1	ORC2	mRNA	XR_001435107.1	lncRNA
XM_015093485.1	ORC2	mRNA	XR_001435107.1	lncRNA
XM_015093485.1	ORC2	mRNA	XR_001435107.1	lncRNA
XM_015104507.1	ZNF22	mRNA	XR_001433856.1	lncRNA
XM_015104507.1	ZNF22	mRNA	XR_001433856.1	lncRNA
XM_015104507.1	ZNF22	mRNA	XR_001433856.1	lncRNA
XM_004007913.3	NT5C3A	mRNA	XR_001433856.1	lncRNA
XM_004007913.3	NT5C3A	mRNA	XR_001433856.1	lncRNA
XM_004007913.3	NT5C3A	mRNA	XR_001433856.1	lncRNA
XM_015093485.1	ORC2	mRNA	XR_001434920.1	lncRNA
XM_015093485.1	ORC2	mRNA	XR_001434920.1	lncRNA
XM_015093485.1	ORC2	mRNA	XR_001434920.1	lncRNA
XM_015093485.1	ORC2	mRNA	XR_001433856.1	lncRNA
XM_015093485.1	ORC2	mRNA	XR_001433856.1	lncRNA
XM_015093485.1	ORC2	mRNA	XR_001433856.1	lncRNA
XM_004005618.3	FPGS	mRNA	XR_001433856.1	lncRNA
XM_004005618.3	FPGS	mRNA	XR_001433856.1	lncRNA
XM_004005618.3	FPGS	mRNA	XR_001433856.1	lncRNA
XM_012187098.1	BROX	mRNA	XR_001433856.1	lncRNA
XM_012187098.1	BROX	mRNA	XR_001433856.1	lncRNA
XM_012187098.1	BROX	mRNA	XR_001433856.1	lncRNA
XM_012181094.1	TTC5	mRNA	XR_001433856.1	lncRNA
XM_012181094.1	TTC5	mRNA	XR_001433856.1	lncRNA
XM_012181094.1	TTC5	mRNA	XR_001433856.1	lncRNA
XM_012100861.1	FBXO9	mRNA	XR_001433856.1	lncRNA
XM_012100861.1	FBXO9	mRNA	XR_001433856.1	lncRNA
XM_012100861.1	FBXO9	mRNA	XR_001433856.1	lncRNA
XM_004003396.3	SETD4	mRNA	XR_001435107.1	lncRNA
XM_004003396.3	SETD4	mRNA	XR_001435107.1	lncRNA

XM_004003396.3	SETD4	mRNA	XR_001435107.1	lncRNA
XM_004003396.3	SETD4	mRNA	XR_001433856.1	lncRNA
XM_004003396.3	SETD4	mRNA	XR_001433856.1	lncRNA
XM_004003396.3	SETD4	mRNA	XR_001433856.1	lncRNA
NM_001202421.1	NUDT9	mRNA	XR_001435107.1	lncRNA
NM_001202421.1	NUDT9	mRNA	XR_001435107.1	lncRNA
NM_001202421.1	NUDT9	mRNA	XR_001435107.1	lncRNA
XM_012124491.2	AK3	mRNA	XR_001436817.1	lncRNA
XM_012124491.2	AK3	mRNA	XR_001436817.1	lncRNA
XM_012124491.2	AK3	mRNA	XR_001436817.1	lncRNA
NM_001202421.1	NUDT9	mRNA	XR_001435722.1	lncRNA
NM_001202421.1	NUDT9	mRNA	XR_001435722.1	lncRNA
NM_001202421.1	NUDT9	mRNA	XR_001435722.1	lncRNA
XM_015093485.1	ORC2	mRNA	XR_001435722.1	lncRNA
XM_015093485.1	ORC2	mRNA	XR_001435722.1	lncRNA
XM_015093485.1	ORC2	mRNA	XR_001435722.1	lncRNA
XM_004007914.3	NT5C3A	mRNA	XR_001435722.1	lncRNA
XM_004007914.3	NT5C3A	mRNA	XR_001435722.1	lncRNA
XM_004007914.3	NT5C3A	mRNA	XR_001435722.1	lncRNA
XM_012187091.1	BROX	mRNA	XR_001435722.1	lncRNA
XM_012187091.1	BROX	mRNA	XR_001435722.1	lncRNA
XM_012187091.1	BROX	mRNA	XR_001435722.1	lncRNA
XM_012094911.2	ZNF135	mRNA	XR_001435722.1	lncRNA
XM_012094911.2	ZNF135	mRNA	XR_001435722.1	lncRNA
XM_012094911.2	ZNF135	mRNA	XR_001435722.1	lncRNA
XM_004007914.3	NT5C3A	mRNA	XR_001436817.1	lncRNA
XM_004007914.3	NT5C3A	mRNA	XR_001436817.1	lncRNA
XM_004007914.3	NT5C3A	mRNA	XR_001436817.1	lncRNA
XM_004018866.3	LOC101120701	mRNA	XR_001436817.1	lncRNA
XM_004018866.3	LOC101120701	mRNA	XR_001436817.1	lncRNA
XM_004018866.3	LOC101120701	mRNA	XR_001436817.1	lncRNA
XM_012187091.1	BROX	mRNA	XR_001436817.1	lncRNA
XM_012187091.1	BROX	mRNA	XR_001436817.1	lncRNA
XM_012187091.1	BROX	mRNA	XR_001436817.1	lncRNA
XM_004012186.3	KLF5	mRNA	XR_001436817.1	lncRNA
XM_004012186.3	KLF5	mRNA	XR_001436817.1	lncRNA
XM_004012186.3	KLF5	mRNA	XR_001436817.1	lncRNA
XM_004003396.3	SETD4	mRNA	XR_001436817.1	lncRNA
XM_004003396.3	SETD4	mRNA	XR_001436817.1	lncRNA
XM_004003396.3	SETD4	mRNA	XR_001436817.1	lncRNA
XM_004012600.2	CAMTA2	mRNA	XR_001436817.1	lncRNA
XM_004012600.2	CAMTA2	mRNA	XR_001436817.1	lncRNA
XM_004012600.2	CAMTA2	mRNA	XR_001436817.1	lncRNA
XM_012181772.2	PCNXL4	mRNA	XR_001436817.1	lncRNA
XM_012181772.2	PCNXL4	mRNA	XR_001436817.1	lncRNA
XM_012181772.2	PCNXL4	mRNA	XR_001436817.1	lncRNA
XM_012178486.2	FSTL4	mRNA	XR_001436817.1	lncRNA
XM_012178486.2	FSTL4	mRNA	XR_001436817.1	lncRNA
XM_012178486.2	FSTL4	mRNA	XR_001436817.1	lncRNA
XM_012105261.2	MCU	mRNA	XR_001436817.1	lncRNA
XM_012105261.2	MCU	mRNA	XR_001436817.1	lncRNA
XM_012105261.2	MCU	mRNA	XR_001436817.1	lncRNA
XM_012176839.2	NT5C3A	mRNA	XR_001436817.1	lncRNA
XM_012176839.2	NT5C3A	mRNA	XR_001436817.1	lncRNA

XM_012176839.2	NT5C3A	mRNA	XR_001436817.1	lncRNA
XM_004012186.3	KLF5	mRNA	XR_001435722.1	lncRNA
XM_004012186.3	KLF5	mRNA	XR_001435722.1	lncRNA
XM_004012186.3	KLF5	mRNA	XR_001435722.1	lncRNA
XM_012178486.2	FSTL4	mRNA	XR_001435722.1	lncRNA
XM_012178486.2	FSTL4	mRNA	XR_001435722.1	lncRNA
XM_012178486.2	FSTL4	mRNA	XR_001435722.1	lncRNA
XM_004003396.3	SETD4	mRNA	XR_001435722.1	lncRNA
XM_004003396.3	SETD4	mRNA	XR_001435722.1	lncRNA
XM_004003396.3	SETD4	mRNA	XR_001435722.1	lncRNA
XM_015093485.1	ORC2	mRNA	XR_001436817.1	lncRNA
XM_015093485.1	ORC2	mRNA	XR_001436817.1	lncRNA
XM_015093485.1	ORC2	mRNA	XR_001436817.1	lncRNA
XM_012119403.2	BIN3	mRNA	XR_001435722.1	lncRNA
XM_012119403.2	BIN3	mRNA	XR_001435722.1	lncRNA
XM_012119403.2	BIN3	mRNA	XR_001435722.1	lncRNA
XM_012100861.1	FBXO9	mRNA	XR_001435722.1	lncRNA
XM_012100861.1	FBXO9	mRNA	XR_001435722.1	lncRNA
XM_012100861.1	FBXO9	mRNA	XR_001435722.1	lncRNA
XM_004006568.3	HSD17B6	mRNA	XR_001436817.1	lncRNA
XM_004006568.3	HSD17B6	mRNA	XR_001436817.1	lncRNA
XM_004006568.3	HSD17B6	mRNA	XR_001436817.1	lncRNA
NM_001202421.1	NUDT9	mRNA	XR_001436817.1	lncRNA
NM_001202421.1	NUDT9	mRNA	XR_001436817.1	lncRNA
NM_001202421.1	NUDT9	mRNA	XR_001436817.1	lncRNA
XM_012095273.2	ARNTL	mRNA	XR_001024290.1	lncRNA
XM_012095273.2	ARNTL	mRNA	XR_001024290.1	lncRNA
XM_012095273.2	ARNTL	mRNA	XR_001024290.1	lncRNA
XM_004011768.3	SAMD12	mRNA	XR_001024290.1	lncRNA
XM_004011768.3	SAMD12	mRNA	XR_001024290.1	lncRNA
XM_004011768.3	SAMD12	mRNA	XR_001024290.1	lncRNA
XM_012102832.2	PPRC1	mRNA	TCONS_00068429	lncRNA
XM_012102832.2	PPRC1	mRNA	TCONS_00068429	lncRNA
XM_012102832.2	PPRC1	mRNA	TCONS_00068429	lncRNA
XM_012174704.2	ZBTB39	mRNA	TCONS_00068429	lncRNA
XM_012174704.2	ZBTB39	mRNA	TCONS_00068429	lncRNA
XM_012174704.2	ZBTB39	mRNA	TCONS_00068429	lncRNA
XM_012102667.1	ZNF518A	mRNA	XR_001434127.1	lncRNA
XM_012102667.1	ZNF518A	mRNA	XR_001434127.1	lncRNA
XM_012102667.1	ZNF518A	mRNA	XR_001434127.1	lncRNA
XM_012104507.1	CUX1	mRNA	XR_001434127.1	lncRNA
XM_012104507.1	CUX1	mRNA	XR_001434127.1	lncRNA
XM_012104507.1	CUX1	mRNA	XR_001434127.1	lncRNA
XM_004022228.3	LOC101122577	mRNA	TCONS_00049802	lncRNA
XM_004022228.3	LOC101122577	mRNA	TCONS_00049802	lncRNA
XM_004022228.3	LOC101122577	mRNA	TCONS_00049802	lncRNA
XM_015095024.1	JOSD1	mRNA	TCONS_00049802	lncRNA
XM_015095024.1	JOSD1	mRNA	TCONS_00049802	lncRNA
XM_015095024.1	JOSD1	mRNA	TCONS_00049802	lncRNA
XM_012104507.1	CUX1	mRNA	TCONS_00049802	lncRNA
XM_012104507.1	CUX1	mRNA	TCONS_00049802	lncRNA
XM_012104507.1	CUX1	mRNA	TCONS_00049802	lncRNA
XM_012102667.1	ZNF518A	mRNA	TCONS_00049802	lncRNA
XM_012102667.1	ZNF518A	mRNA	TCONS_00049802	lncRNA

XM_012102667.1	ZNF518A	mRNA	TCONS_00049802	lncRNA
XM_012159714.2	KITLG	mRNA	TCONS_00049802	lncRNA
XM_012159714.2	KITLG	mRNA	TCONS_00049802	lncRNA
XM_012159714.2	KITLG	mRNA	TCONS_00049802	lncRNA
XM_012102636.2	TCTN3	mRNA	XR_001434127.1	lncRNA
XM_012102636.2	TCTN3	mRNA	XR_001434127.1	lncRNA
XM_012102636.2	TCTN3	mRNA	XR_001434127.1	lncRNA
XM_012104222.2	DCUN1D3	mRNA	XR_001023627.2	lncRNA
XM_012104222.2	DCUN1D3	mRNA	XR_001023627.2	lncRNA
XM_012104222.2	DCUN1D3	mRNA	XR_001023627.2	lncRNA
XM_012159714.2	KITLG	mRNA	XR_001023627.2	lncRNA
XM_012159714.2	KITLG	mRNA	XR_001023627.2	lncRNA
XM_012159714.2	KITLG	mRNA	XR_001023627.2	lncRNA
XM_012110487.2	UBQLN1	mRNA	XR_001023627.2	lncRNA
XM_012110487.2	UBQLN1	mRNA	XR_001023627.2	lncRNA
XM_012110487.2	UBQLN1	mRNA	XR_001023627.2	lncRNA
XM_012175065.2	AMN1	mRNA	XR_001023627.2	lncRNA
XM_012175065.2	AMN1	mRNA	XR_001023627.2	lncRNA
XM_012175065.2	AMN1	mRNA	XR_001023627.2	lncRNA
XM_012175065.2	AMN1	mRNA	XR_001434127.1	lncRNA
XM_012175065.2	AMN1	mRNA	XR_001434127.1	lncRNA
XM_012175065.2	AMN1	mRNA	XR_001434127.1	lncRNA
XM_012187364.1	ENO1	mRNA	XR_001434127.1	lncRNA
XM_012187364.1	ENO1	mRNA	XR_001434127.1	lncRNA
XM_012187364.1	ENO1	mRNA	XR_001434127.1	lncRNA
XM_012177185.2	CNOT4	mRNA	XR_001023627.2	lncRNA
XM_012177185.2	CNOT4	mRNA	XR_001023627.2	lncRNA
XM_012177185.2	CNOT4	mRNA	XR_001023627.2	lncRNA
XM_012177185.2	CNOT4	mRNA	XR_001434127.1	lncRNA
XM_012177185.2	CNOT4	mRNA	XR_001434127.1	lncRNA
XM_012177185.2	CNOT4	mRNA	XR_001434127.1	lncRNA
XM_012187364.1	ENO1	mRNA	XR_001023627.2	lncRNA
XM_012187364.1	ENO1	mRNA	XR_001023627.2	lncRNA
XM_012187364.1	ENO1	mRNA	XR_001023627.2	lncRNA
XM_015092004.1	MROH7	mRNA	XR_001433837.1	lncRNA
XM_015092004.1	MROH7	mRNA	XR_001433837.1	lncRNA
XM_015092004.1	MROH7	mRNA	XR_001433837.1	lncRNA
XM_012176469.2	MACC1	mRNA	XR_001433837.1	lncRNA
XM_012176469.2	MACC1	mRNA	XR_001433837.1	lncRNA
XM_012176469.2	MACC1	mRNA	XR_001433837.1	lncRNA
XM_015098186.1	XPO4	mRNA	XR_001433837.1	lncRNA
XM_015098186.1	XPO4	mRNA	XR_001433837.1	lncRNA
XM_015098186.1	XPO4	mRNA	XR_001433837.1	lncRNA
XM_012174423.2	SLC38A4	mRNA	XR_001433837.1	lncRNA
XM_012174423.2	SLC38A4	mRNA	XR_001433837.1	lncRNA
XM_012174423.2	SLC38A4	mRNA	XR_001433837.1	lncRNA
XM_012098071.2	RNF185	mRNA	XR_001433837.1	lncRNA
XM_012098071.2	RNF185	mRNA	XR_001433837.1	lncRNA
XM_012098071.2	RNF185	mRNA	XR_001433837.1	lncRNA
XM_015097749.1	PCMTD1	mRNA	XR_001433837.1	lncRNA
XM_015097749.1	PCMTD1	mRNA	XR_001433837.1	lncRNA
XM_015097749.1	PCMTD1	mRNA	XR_001433837.1	lncRNA
XM_012101006.2	CCHCR1	mRNA	XR_001433837.1	lncRNA
XM_012101006.2	CCHCR1	mRNA	XR_001433837.1	lncRNA

XM_012101006.2	CCHCR1	mRNA	XR_001433837.1	lncRNA
XM_012106112.2	ARAF	mRNA	XR_001433837.1	lncRNA
XM_012106112.2	ARAF	mRNA	XR_001433837.1	lncRNA
XM_012106112.2	ARAF	mRNA	XR_001433837.1	lncRNA
XM_015099337.1	RALGPS2	mRNA	XR_001025540.2	lncRNA
XM_015099337.1	RALGPS2	mRNA	XR_001025540.2	lncRNA
XM_015099337.1	RALGPS2	mRNA	XR_001025540.2	lncRNA
XM_012098903.1	PPIP5K1	mRNA	XR_001025540.2	lncRNA
XM_012098903.1	PPIP5K1	mRNA	XR_001025540.2	lncRNA
XM_012098903.1	PPIP5K1	mRNA	XR_001025540.2	lncRNA
XM_015092971.1	SNX30	mRNA	XR_001025540.2	lncRNA
XM_015092971.1	SNX30	mRNA	XR_001025540.2	lncRNA
XM_015092971.1	SNX30	mRNA	XR_001025540.2	lncRNA
XM_012146156.1	USP37	mRNA	XR_001025540.2	lncRNA
XM_012146156.1	USP37	mRNA	XR_001025540.2	lncRNA
XM_012146156.1	USP37	mRNA	XR_001025540.2	lncRNA
XM_012178157.2	RAPGEF6	mRNA	XR_001025540.2	lncRNA
XM_012178157.2	RAPGEF6	mRNA	XR_001025540.2	lncRNA
XM_012178157.2	RAPGEF6	mRNA	XR_001025540.2	lncRNA
XM_012099341.2	CLASP2	mRNA	XR_001025540.2	lncRNA
XM_012099341.2	CLASP2	mRNA	XR_001025540.2	lncRNA
XM_012099341.2	CLASP2	mRNA	XR_001025540.2	lncRNA
XM_012107808.1	NFYC	mRNA	XR_001025540.2	lncRNA
XM_012107808.1	NFYC	mRNA	XR_001025540.2	lncRNA
XM_012107808.1	NFYC	mRNA	XR_001025540.2	lncRNA
XM_012174509.2	ADGRL2	mRNA	XR_001024966.2	lncRNA
XM_012174509.2	ADGRL2	mRNA	XR_001024966.2	lncRNA
XM_012174509.2	ADGRL2	mRNA	XR_001024966.2	lncRNA
XM_015096518.1	AASDH	mRNA	XR_001023303.2	lncRNA
XM_015096518.1	AASDH	mRNA	XR_001023303.2	lncRNA
XM_015096518.1	AASDH	mRNA	XR_001023303.2	lncRNA
XM_004011835.3	RAD54B	mRNA	XR_001023303.2	lncRNA
XM_004011835.3	RAD54B	mRNA	XR_001023303.2	lncRNA
XM_004011835.3	RAD54B	mRNA	XR_001023303.2	lncRNA
XM_012176414.2	PHF14	mRNA	XR_001023303.2	lncRNA
XM_012176414.2	PHF14	mRNA	XR_001023303.2	lncRNA
XM_012176414.2	PHF14	mRNA	XR_001023303.2	lncRNA
XM_012176761.2	MDFIC	mRNA	XR_001023303.2	lncRNA
XM_012176761.2	MDFIC	mRNA	XR_001023303.2	lncRNA
XM_012176761.2	MDFIC	mRNA	XR_001023303.2	lncRNA
XM_012176414.2	PHF14	mRNA	XR_001024966.2	lncRNA
XM_012176414.2	PHF14	mRNA	XR_001024966.2	lncRNA
XM_012176414.2	PHF14	mRNA	XR_001024966.2	lncRNA
XM_004011835.3	RAD54B	mRNA	XR_001024966.2	lncRNA
XM_004011835.3	RAD54B	mRNA	XR_001024966.2	lncRNA
XM_004011835.3	RAD54B	mRNA	XR_001024966.2	lncRNA
XM_012106889.2	TAF1	mRNA	XR_001023303.2	lncRNA
XM_012106889.2	TAF1	mRNA	XR_001023303.2	lncRNA
XM_012106889.2	TAF1	mRNA	XR_001023303.2	lncRNA
XM_012129647.1	NEK1	mRNA	XR_001023303.2	lncRNA
XM_012129647.1	NEK1	mRNA	XR_001023303.2	lncRNA
XM_012129647.1	NEK1	mRNA	XR_001023303.2	lncRNA
XM_015102459.1	LOC101117971	mRNA	XR_001024966.2	lncRNA
XM_015102459.1	LOC101117971	mRNA	XR_001024966.2	lncRNA

XM_015102459.1	LOC101117971	mRNA	XR_001024966.2	lncRNA
XM_012098709.2	PML	mRNA	XR_001023303.2	lncRNA
XM_012098709.2	PML	mRNA	XR_001023303.2	lncRNA
XM_012098709.2	PML	mRNA	XR_001023303.2	lncRNA
XM_015102009.1	RALGAPA1	mRNA	XR_001023303.2	lncRNA
XM_015102009.1	RALGAPA1	mRNA	XR_001023303.2	lncRNA
XM_015102009.1	RALGAPA1	mRNA	XR_001023303.2	lncRNA
XM_012187827.1	GIGYF2	mRNA	XR_001024966.2	lncRNA
XM_012187827.1	GIGYF2	mRNA	XR_001024966.2	lncRNA
XM_012187827.1	GIGYF2	mRNA	XR_001024966.2	lncRNA
XM_012096759.2	ATP13A3	mRNA	XR_001023303.2	lncRNA
XM_012096759.2	ATP13A3	mRNA	XR_001023303.2	lncRNA
XM_012096759.2	ATP13A3	mRNA	XR_001023303.2	lncRNA
XM_015098592.1	RABEP1	mRNA	XR_001023303.2	lncRNA
XM_015098592.1	RABEP1	mRNA	XR_001023303.2	lncRNA
XM_015098592.1	RABEP1	mRNA	XR_001023303.2	lncRNA
XM_015098592.1	RABEP1	mRNA	XR_001024966.2	lncRNA
XM_015098592.1	RABEP1	mRNA	XR_001024966.2	lncRNA
XM_015098592.1	RABEP1	mRNA	XR_001024966.2	lncRNA
XM_012181827.2	ZBTB1	mRNA	XR_001024966.2	lncRNA
XM_012181827.2	ZBTB1	mRNA	XR_001024966.2	lncRNA
XM_012181827.2	ZBTB1	mRNA	XR_001024966.2	lncRNA
XM_012187630.2	SOAT1	mRNA	XR_001024966.2	lncRNA
XM_012187630.2	SOAT1	mRNA	XR_001024966.2	lncRNA
XM_012187630.2	SOAT1	mRNA	XR_001024966.2	lncRNA
XM_012187727.2	TRMT1L	mRNA	XR_001023303.2	lncRNA
XM_012187727.2	TRMT1L	mRNA	XR_001023303.2	lncRNA
XM_012187727.2	TRMT1L	mRNA	XR_001023303.2	lncRNA
XM_015094671.1	LOC101116975	mRNA	XR_001023303.2	lncRNA
XM_015094671.1	LOC101116975	mRNA	XR_001023303.2	lncRNA
XM_015094671.1	LOC101116975	mRNA	XR_001023303.2	lncRNA
XM_012102598.2	TBC1D12	mRNA	XR_001040147.1	lncRNA
XM_012102598.2	TBC1D12	mRNA	XR_001040147.1	lncRNA
XM_012102598.2	TBC1D12	mRNA	XR_001040147.1	lncRNA
XM_015094800.1	RAB5B	mRNA	XR_001040147.1	lncRNA
XM_015094800.1	RAB5B	mRNA	XR_001040147.1	lncRNA
XM_015094800.1	RAB5B	mRNA	XR_001040147.1	lncRNA
XM_012103323.2	KIAA1328	mRNA	XR_001436390.1	lncRNA
XM_012103323.2	KIAA1328	mRNA	XR_001436390.1	lncRNA
XM_012103323.2	KIAA1328	mRNA	XR_001436390.1	lncRNA
XM_012159735.2	KITLG	mRNA	XR_001040147.1	lncRNA
XM_012159735.2	KITLG	mRNA	XR_001040147.1	lncRNA
XM_012159735.2	KITLG	mRNA	XR_001040147.1	lncRNA
XM_015094800.1	RAB5B	mRNA	XR_001436390.1	lncRNA
XM_015094800.1	RAB5B	mRNA	XR_001436390.1	lncRNA
XM_015094800.1	RAB5B	mRNA	XR_001436390.1	lncRNA
XM_012105556.2	ZFAND4	mRNA	XR_001436390.1	lncRNA
XM_012105556.2	ZFAND4	mRNA	XR_001436390.1	lncRNA
XM_012105556.2	ZFAND4	mRNA	XR_001436390.1	lncRNA
XM_012159735.2	KITLG	mRNA	XR_001436390.1	lncRNA
XM_012159735.2	KITLG	mRNA	XR_001436390.1	lncRNA
XM_012159735.2	KITLG	mRNA	XR_001436390.1	lncRNA
XM_012184403.1	ZC3H13	mRNA	XR_001436390.1	lncRNA
XM_012184403.1	ZC3H13	mRNA	XR_001436390.1	lncRNA

XM_012184403.1	ZC3H13	mRNA	XR_001436390.1	lncRNA
XM_012095623.1	TBCEL	mRNA	XR_001436390.1	lncRNA
XM_012095623.1	TBCEL	mRNA	XR_001436390.1	lncRNA
XM_012095623.1	TBCEL	mRNA	XR_001436390.1	lncRNA
XM_012105014.1	LGALS8	mRNA	XR_001436390.1	lncRNA
XM_012105014.1	LGALS8	mRNA	XR_001436390.1	lncRNA
XM_012105014.1	LGALS8	mRNA	XR_001436390.1	lncRNA
XM_015098983.1	C11H17orf80	mRNA	XR_001436390.1	lncRNA
XM_015098983.1	C11H17orf80	mRNA	XR_001436390.1	lncRNA
XM_015098983.1	C11H17orf80	mRNA	XR_001436390.1	lncRNA
XM_015093726.1	HP1BP3	mRNA	XR_001436390.1	lncRNA
XM_015093726.1	HP1BP3	mRNA	XR_001436390.1	lncRNA
XM_015093726.1	HP1BP3	mRNA	XR_001436390.1	lncRNA
XM_012161705.2	LRRC8A	mRNA	XR_001436390.1	lncRNA
XM_012161705.2	LRRC8A	mRNA	XR_001436390.1	lncRNA
XM_012161705.2	LRRC8A	mRNA	XR_001436390.1	lncRNA
XM_004014588.3	PKIG	mRNA	XR_001040147.1	lncRNA
XM_004014588.3	PKIG	mRNA	XR_001040147.1	lncRNA
XM_004014588.3	PKIG	mRNA	XR_001040147.1	lncRNA
XM_012106234.2	OFD1	mRNA	XR_001436390.1	lncRNA
XM_012106234.2	OFD1	mRNA	XR_001436390.1	lncRNA
XM_012106234.2	OFD1	mRNA	XR_001436390.1	lncRNA
XM_004008085.3	CNOT4	mRNA	XR_001040147.1	lncRNA
XM_004008085.3	CNOT4	mRNA	XR_001040147.1	lncRNA
XM_004008085.3	CNOT4	mRNA	XR_001040147.1	lncRNA
XM_012182192.2	EML5	mRNA	TCONS_00068429	lncRNA
XM_012182192.2	EML5	mRNA	TCONS_00068429	lncRNA
XM_012182192.2	EML5	mRNA	TCONS_00068429	lncRNA
XM_012105324.2	USP54	mRNA	XR_001041220.2	lncRNA
XM_012105324.2	USP54	mRNA	XR_001041220.2	lncRNA
XM_012105324.2	USP54	mRNA	XR_001041220.2	lncRNA
XM_012105610.1	ZFP69	mRNA	XR_001436612.1	lncRNA
XM_012105610.1	ZFP69	mRNA	XR_001436612.1	lncRNA
XM_012105610.1	ZFP69	mRNA	XR_001436612.1	lncRNA
XM_004014289.3	ZNF133	mRNA	XR_001436612.1	lncRNA
XM_004014289.3	ZNF133	mRNA	XR_001436612.1	lncRNA
XM_004014289.3	ZNF133	mRNA	XR_001436612.1	lncRNA
XM_012095761.2	HSPBAP1	mRNA	XR_001436612.1	lncRNA
XM_012095761.2	HSPBAP1	mRNA	XR_001436612.1	lncRNA
XM_012095761.2	HSPBAP1	mRNA	XR_001436612.1	lncRNA
XM_012097504.1	MFSD8	mRNA	XR_001436612.1	lncRNA
XM_012097504.1	MFSD8	mRNA	XR_001436612.1	lncRNA
XM_012097504.1	MFSD8	mRNA	XR_001436612.1	lncRNA
XM_012181270.1	VPS39	mRNA	XR_001436612.1	lncRNA
XM_012181270.1	VPS39	mRNA	XR_001436612.1	lncRNA
XM_012181270.1	VPS39	mRNA	XR_001436612.1	lncRNA
XM_004008056.3	NRF1	mRNA	XR_001025540.2	lncRNA
XM_004008056.3	NRF1	mRNA	XR_001025540.2	lncRNA
XM_004008056.3	NRF1	mRNA	XR_001025540.2	lncRNA
XM_012180234.2	CNOT6L	mRNA	XR_001025540.2	lncRNA
XM_012180234.2	CNOT6L	mRNA	XR_001025540.2	lncRNA
XM_012180234.2	CNOT6L	mRNA	XR_001025540.2	lncRNA
XM_012173553.2	STRN	mRNA	XR_001025540.2	lncRNA
XM_012173553.2	STRN	mRNA	XR_001025540.2	lncRNA

XM_012173553.2	STRN	mRNA	XR_001025540.2	lncRNA
XM_004017788.3	ZFANL3	mRNA	XR_001025540.2	lncRNA
XM_004017788.3	ZFANL3	mRNA	XR_001025540.2	lncRNA
XM_004017788.3	ZFANL3	mRNA	XR_001025540.2	lncRNA
XM_012097365.2	SH3D19	mRNA	XR_001025540.2	lncRNA
XM_012097365.2	SH3D19	mRNA	XR_001025540.2	lncRNA
XM_012097365.2	SH3D19	mRNA	XR_001025540.2	lncRNA
XM_012182450.2	IBTK	mRNA	XR_001025540.2	lncRNA
XM_012182450.2	IBTK	mRNA	XR_001025540.2	lncRNA
XM_012182450.2	IBTK	mRNA	XR_001025540.2	lncRNA
XM_012185338.2	ZNF207	mRNA	XR_001025540.2	lncRNA
XM_012185338.2	ZNF207	mRNA	XR_001025540.2	lncRNA
XM_012185338.2	ZNF207	mRNA	XR_001025540.2	lncRNA
XM_015099548.1	PARD3	mRNA	TCONS_00068429	lncRNA
XM_015099548.1	PARD3	mRNA	TCONS_00068429	lncRNA
XM_015099548.1	PARD3	mRNA	TCONS_00068429	lncRNA
XM_004012101.3	FREM2	mRNA	TCONS_00068429	lncRNA
XM_004012101.3	FREM2	mRNA	TCONS_00068429	lncRNA
XM_004012101.3	FREM2	mRNA	TCONS_00068429	lncRNA
XM_015093216.1	TTC39B	mRNA	XR_001433837.1	lncRNA
XM_015093216.1	TTC39B	mRNA	XR_001433837.1	lncRNA
XM_015093216.1	TTC39B	mRNA	XR_001433837.1	lncRNA
XM_012095602.2	USP2	mRNA	XR_001433837.1	lncRNA
XM_012095602.2	USP2	mRNA	XR_001433837.1	lncRNA
XM_012095602.2	USP2	mRNA	XR_001433837.1	lncRNA
XM_015096518.1	AASDH	mRNA	XR_001433837.1	lncRNA
XM_015096518.1	AASDH	mRNA	XR_001433837.1	lncRNA
XM_015096518.1	AASDH	mRNA	XR_001433837.1	lncRNA
XM_004020402.3	RTTN	mRNA	XR_001433837.1	lncRNA
XM_004020402.3	RTTN	mRNA	XR_001433837.1	lncRNA
XM_004020402.3	RTTN	mRNA	XR_001433837.1	lncRNA
XM_012116543.2	ERCC6L2	mRNA	XR_001433837.1	lncRNA
XM_012116543.2	ERCC6L2	mRNA	XR_001433837.1	lncRNA
XM_012116543.2	ERCC6L2	mRNA	XR_001433837.1	lncRNA
XM_012188097.2	PLCB1	mRNA	XR_001433837.1	lncRNA
XM_012188097.2	PLCB1	mRNA	XR_001433837.1	lncRNA
XM_012188097.2	PLCB1	mRNA	XR_001433837.1	lncRNA
XM_012188917.1	STX16	mRNA	XR_001041893.2	lncRNA
XM_012188917.1	STX16	mRNA	XR_001041893.2	lncRNA
XM_012188917.1	STX16	mRNA	XR_001041893.2	lncRNA
XM_012183298.1	FAM120B	mRNA	XR_001041893.2	lncRNA
XM_012183298.1	FAM120B	mRNA	XR_001041893.2	lncRNA
XM_012183298.1	FAM120B	mRNA	XR_001041893.2	lncRNA
XM_012142659.2	ALS2	mRNA	XR_001024966.2	lncRNA
XM_012142659.2	ALS2	mRNA	XR_001024966.2	lncRNA
XM_012142659.2	ALS2	mRNA	XR_001024966.2	lncRNA
XM_012181265.2	PLA2G4F	mRNA	XR_001023303.2	lncRNA
XM_012181265.2	PLA2G4F	mRNA	XR_001023303.2	lncRNA
XM_012181265.2	PLA2G4F	mRNA	XR_001023303.2	lncRNA
XM_015092063.1	FUBP1	mRNA	XR_001041893.2	lncRNA
XM_015092063.1	FUBP1	mRNA	XR_001041893.2	lncRNA
XM_015092063.1	FUBP1	mRNA	XR_001041893.2	lncRNA
XM_012185875.1	LUC7L3	mRNA	XR_001023303.2	lncRNA
XM_012185875.1	LUC7L3	mRNA	XR_001023303.2	lncRNA

XM_012185875.1	LUC7L3	mRNA	XR_001023303.2	lncRNA
XM_012097468.2	ELF2	mRNA	XR_001024966.2	lncRNA
XM_012097468.2	ELF2	mRNA	XR_001024966.2	lncRNA
XM_012097468.2	ELF2	mRNA	XR_001024966.2	lncRNA
XM_015101001.1	RRAS2	mRNA	XR_001025540.2	lncRNA
XM_015101001.1	RRAS2	mRNA	XR_001025540.2	lncRNA
XM_015101001.1	RRAS2	mRNA	XR_001025540.2	lncRNA
XM_012097374.2	LRBA	mRNA	XR_001025540.2	lncRNA
XM_012097374.2	LRBA	mRNA	XR_001025540.2	lncRNA
XM_012097374.2	LRBA	mRNA	XR_001025540.2	lncRNA
XM_012179676.2	CCSER1	mRNA	XR_001025540.2	lncRNA
XM_012179676.2	CCSER1	mRNA	XR_001025540.2	lncRNA
XM_012179676.2	CCSER1	mRNA	XR_001025540.2	lncRNA
XM_012097035.1	C16H5orf42	mRNA	XR_001025540.2	lncRNA
XM_012097035.1	C16H5orf42	mRNA	XR_001025540.2	lncRNA
XM_012097035.1	C16H5orf42	mRNA	XR_001025540.2	lncRNA
XM_012179825.2	N4BP2	mRNA	XR_001025540.2	lncRNA
XM_012179825.2	N4BP2	mRNA	XR_001025540.2	lncRNA
XM_012179825.2	N4BP2	mRNA	XR_001025540.2	lncRNA
XM_015105074.1	LONRF3	mRNA	XR_001025540.2	lncRNA
XM_015105074.1	LONRF3	mRNA	XR_001025540.2	lncRNA
XM_015105074.1	LONRF3	mRNA	XR_001025540.2	lncRNA
XM_012100080.2	ARIH2	mRNA	XR_001023627.2	lncRNA
XM_012100080.2	ARIH2	mRNA	XR_001023627.2	lncRNA
XM_012100080.2	ARIH2	mRNA	XR_001023627.2	lncRNA
XM_015092004.1	MROH7	mRNA	XR_001023627.2	lncRNA
XM_015092004.1	MROH7	mRNA	XR_001023627.2	lncRNA
XM_015092004.1	MROH7	mRNA	XR_001023627.2	lncRNA
XM_012174423.2	SLC38A4	mRNA	TCONS_00049802	lncRNA
XM_012174423.2	SLC38A4	mRNA	TCONS_00049802	lncRNA
XM_012174423.2	SLC38A4	mRNA	TCONS_00049802	lncRNA
XM_015092004.1	MROH7	mRNA	TCONS_00049802	lncRNA
XM_015092004.1	MROH7	mRNA	TCONS_00049802	lncRNA
XM_015092004.1	MROH7	mRNA	TCONS_00049802	lncRNA
XM_012098071.2	RNF185	mRNA	TCONS_00049802	lncRNA
XM_012098071.2	RNF185	mRNA	TCONS_00049802	lncRNA
XM_012098071.2	RNF185	mRNA	TCONS_00049802	lncRNA
XM_015103688.1	PTPN2	mRNA	XR_001023627.2	lncRNA
XM_015103688.1	PTPN2	mRNA	XR_001023627.2	lncRNA
XM_015103688.1	PTPN2	mRNA	XR_001023627.2	lncRNA
XM_012186029.1	FBXL20	mRNA	TCONS_00049802	lncRNA
XM_012186029.1	FBXL20	mRNA	TCONS_00049802	lncRNA
XM_012186029.1	FBXL20	mRNA	TCONS_00049802	lncRNA
XM_012098071.2	RNF185	mRNA	XR_001023627.2	lncRNA
XM_012098071.2	RNF185	mRNA	XR_001023627.2	lncRNA
XM_012098071.2	RNF185	mRNA	XR_001023627.2	lncRNA
XM_015092005.1	MROH7	mRNA	TCONS_00049802	lncRNA
XM_015092005.1	MROH7	mRNA	TCONS_00049802	lncRNA
XM_015092005.1	MROH7	mRNA	TCONS_00049802	lncRNA
XM_012181270.1	VPS39	mRNA	XR_001023627.2	lncRNA
XM_012181270.1	VPS39	mRNA	XR_001023627.2	lncRNA
XM_012181270.1	VPS39	mRNA	XR_001023627.2	lncRNA
XM_015103475.1	PLEKHA1	mRNA	TCONS_00049802	lncRNA
XM_015103475.1	PLEKHA1	mRNA	TCONS_00049802	lncRNA

XM_015103475.1	PLEKHA1	mRNA	TCONS_00049802	lncRNA
XM_012176656.2	NAPEPLD	mRNA	XR_001434127.1	lncRNA
XM_012176656.2	NAPEPLD	mRNA	XR_001434127.1	lncRNA
XM_012176656.2	NAPEPLD	mRNA	XR_001434127.1	lncRNA
XM_015092005.1	MROH7	mRNA	XR_001434127.1	lncRNA
XM_015092005.1	MROH7	mRNA	XR_001434127.1	lncRNA
XM_015092005.1	MROH7	mRNA	XR_001434127.1	lncRNA
XM_012179413.2	PDE5A	mRNA	XR_001434127.1	lncRNA
XM_012179413.2	PDE5A	mRNA	XR_001434127.1	lncRNA
XM_012179413.2	PDE5A	mRNA	XR_001434127.1	lncRNA
XM_015092004.1	MROH7	mRNA	XR_001434127.1	lncRNA
XM_015092004.1	MROH7	mRNA	XR_001434127.1	lncRNA
XM_015092004.1	MROH7	mRNA	XR_001434127.1	lncRNA
XM_012101006.2	CCHCR1	mRNA	XR_001023627.2	lncRNA
XM_012101006.2	CCHCR1	mRNA	XR_001023627.2	lncRNA
XM_012101006.2	CCHCR1	mRNA	XR_001023627.2	lncRNA
XM_012140245.2	RALB	mRNA	XR_001434127.1	lncRNA
XM_012140245.2	RALB	mRNA	XR_001434127.1	lncRNA
XM_012140245.2	RALB	mRNA	XR_001434127.1	lncRNA
XM_012106112.2	ARAF	mRNA	XR_001023627.2	lncRNA
XM_012106112.2	ARAF	mRNA	XR_001023627.2	lncRNA
XM_012106112.2	ARAF	mRNA	XR_001023627.2	lncRNA
XM_012174597.2	NUP107	mRNA	XR_001435723.1	lncRNA
XM_012174597.2	NUP107	mRNA	XR_001435723.1	lncRNA
XM_012174597.2	NUP107	mRNA	XR_001435723.1	lncRNA
XM_012102598.2	TBC1D12	mRNA	XR_001435723.1	lncRNA
XM_012102598.2	TBC1D12	mRNA	XR_001435723.1	lncRNA
XM_012102598.2	TBC1D12	mRNA	XR_001435723.1	lncRNA
XM_015095024.1	JOSD1	mRNA	XR_001436064.1	lncRNA
XM_015095024.1	JOSD1	mRNA	XR_001436064.1	lncRNA
XM_015095024.1	JOSD1	mRNA	XR_001436064.1	lncRNA
XM_012184401.1	ZC3H13	mRNA	XR_001436064.1	lncRNA
XM_012184401.1	ZC3H13	mRNA	XR_001436064.1	lncRNA
XM_012184401.1	ZC3H13	mRNA	XR_001436064.1	lncRNA
XM_012175065.2	AMN1	mRNA	XR_001436064.1	lncRNA
XM_012175065.2	AMN1	mRNA	XR_001436064.1	lncRNA
XM_012175065.2	AMN1	mRNA	XR_001436064.1	lncRNA
XM_012187364.1	ENO1	mRNA	XR_001436064.1	lncRNA
XM_012187364.1	ENO1	mRNA	XR_001436064.1	lncRNA
XM_012187364.1	ENO1	mRNA	XR_001436064.1	lncRNA
XM_004012167.2	GJB6	mRNA	XR_001024230.1	lncRNA
XM_004012167.2	GJB6	mRNA	XR_001024230.1	lncRNA
XM_004012167.2	GJB6	mRNA	XR_001024230.1	lncRNA
XM_004020479.3	RBBP8	mRNA	XR_001435723.1	lncRNA
XM_004020479.3	RBBP8	mRNA	XR_001435723.1	lncRNA
XM_004020479.3	RBBP8	mRNA	XR_001435723.1	lncRNA
XM_015095024.1	JOSD1	mRNA	XR_001436065.1	lncRNA
XM_015095024.1	JOSD1	mRNA	XR_001436065.1	lncRNA
XM_015095024.1	JOSD1	mRNA	XR_001436065.1	lncRNA
XM_012099659.2	FOXP1	mRNA	XR_001436065.1	lncRNA
XM_012099659.2	FOXP1	mRNA	XR_001436065.1	lncRNA
XM_012099659.2	FOXP1	mRNA	XR_001436065.1	lncRNA
XM_012174597.2	NUP107	mRNA	XR_001435726.1	lncRNA
XM_012174597.2	NUP107	mRNA	XR_001435726.1	lncRNA

XM_012174597.2	NUP107	mRNA	XR_001435726.1	lncRNA
XM_012102598.2	TBC1D12	mRNA	XR_001435726.1	lncRNA
XM_012102598.2	TBC1D12	mRNA	XR_001435726.1	lncRNA
XM_012102598.2	TBC1D12	mRNA	XR_001435726.1	lncRNA
XM_012175065.2	AMN1	mRNA	XR_001436065.1	lncRNA
XM_012175065.2	AMN1	mRNA	XR_001436065.1	lncRNA
XM_012175065.2	AMN1	mRNA	XR_001436065.1	lncRNA
XM_012187364.1	ENO1	mRNA	XR_001436065.1	lncRNA
XM_012187364.1	ENO1	mRNA	XR_001436065.1	lncRNA
XM_012187364.1	ENO1	mRNA	XR_001436065.1	lncRNA
XM_012190351.2	ZNF420	mRNA	XR_001435726.1	lncRNA
XM_012190351.2	ZNF420	mRNA	XR_001435726.1	lncRNA
XM_012190351.2	ZNF420	mRNA	XR_001435726.1	lncRNA
XM_012190020.2	ZFP90	mRNA	XR_001435726.1	lncRNA
XM_012190020.2	ZFP90	mRNA	XR_001435726.1	lncRNA
XM_012190020.2	ZFP90	mRNA	XR_001435726.1	lncRNA
XM_004020479.3	RBBP8	mRNA	XR_001435726.1	lncRNA
XM_004020479.3	RBBP8	mRNA	XR_001435726.1	lncRNA
XM_004020479.3	RBBP8	mRNA	XR_001435726.1	lncRNA
XM_012186362.1	PITPNC1	mRNA	XR_001041220.2	lncRNA
XM_012186362.1	PITPNC1	mRNA	XR_001041220.2	lncRNA
XM_012186362.1	PITPNC1	mRNA	XR_001041220.2	lncRNA
XM_015096518.1	AASDH	mRNA	XR_001436612.1	lncRNA
XM_015096518.1	AASDH	mRNA	XR_001436612.1	lncRNA
XM_015096518.1	AASDH	mRNA	XR_001436612.1	lncRNA
XM_012186312.2	DDX42	mRNA	XR_001023303.2	lncRNA
XM_012186312.2	DDX42	mRNA	XR_001023303.2	lncRNA
XM_012186312.2	DDX42	mRNA	XR_001023303.2	lncRNA
XM_015096977.1	NIN	mRNA	XR_001023303.2	lncRNA
XM_015096977.1	NIN	mRNA	XR_001023303.2	lncRNA
XM_015096977.1	NIN	mRNA	XR_001023303.2	lncRNA
XM_012170869.2	PAPOLG	mRNA	XR_001041893.2	lncRNA
XM_012170869.2	PAPOLG	mRNA	XR_001041893.2	lncRNA
XM_012170869.2	PAPOLG	mRNA	XR_001041893.2	lncRNA
XM_012098812.2	RALGAPA1	mRNA	XR_001023303.2	lncRNA
XM_012098812.2	RALGAPA1	mRNA	XR_001023303.2	lncRNA
XM_012098812.2	RALGAPA1	mRNA	XR_001023303.2	lncRNA
XM_004012689.3	MYH10	mRNA	XR_001040147.1	lncRNA
XM_004012689.3	MYH10	mRNA	XR_001040147.1	lncRNA
XM_004012689.3	MYH10	mRNA	XR_001040147.1	lncRNA
XM_012105610.1	ZFP69	mRNA	XR_001040147.1	lncRNA
XM_012105610.1	ZFP69	mRNA	XR_001040147.1	lncRNA
XM_012105610.1	ZFP69	mRNA	XR_001040147.1	lncRNA
XM_012159299.2	LOC101121563	mRNA	XR_001040147.1	lncRNA
XM_012159299.2	LOC101121563	mRNA	XR_001040147.1	lncRNA
XM_012159299.2	LOC101121563	mRNA	XR_001040147.1	lncRNA
XM_015099653.1	LOC101121441	mRNA	XR_001040147.1	lncRNA
XM_015099653.1	LOC101121441	mRNA	XR_001040147.1	lncRNA
XM_015099653.1	LOC101121441	mRNA	XR_001040147.1	lncRNA
XM_004014289.3	ZNF133	mRNA	XR_001040147.1	lncRNA
XM_004014289.3	ZNF133	mRNA	XR_001040147.1	lncRNA
XM_004014289.3	ZNF133	mRNA	XR_001040147.1	lncRNA
XM_015096221.1	PPIP5K2	mRNA	XR_001040147.1	lncRNA
XM_015096221.1	PPIP5K2	mRNA	XR_001040147.1	lncRNA

XM_015096221.1	PPIP5K2	mRNA	XR_001040147.1	lncRNA
XM_012142751.2	ZFYVE9	mRNA	XR_001040147.1	lncRNA
XM_012142751.2	ZFYVE9	mRNA	XR_001040147.1	lncRNA
XM_012142751.2	ZFYVE9	mRNA	XR_001040147.1	lncRNA
XM_012177136.1	TMEM209	mRNA	XR_001040147.1	lncRNA
XM_012177136.1	TMEM209	mRNA	XR_001040147.1	lncRNA
XM_012177136.1	TMEM209	mRNA	XR_001040147.1	lncRNA
XM_004014289.3	ZNF133	mRNA	XR_001436390.1	lncRNA
XM_004014289.3	ZNF133	mRNA	XR_001436390.1	lncRNA
XM_004014289.3	ZNF133	mRNA	XR_001436390.1	lncRNA
XM_012097252.2	RHOF	mRNA	XR_001436390.1	lncRNA
XM_012097252.2	RHOF	mRNA	XR_001436390.1	lncRNA
XM_012097252.2	RHOF	mRNA	XR_001436390.1	lncRNA
XM_015094919.1	WBP11	mRNA	XR_001436390.1	lncRNA
XM_015094919.1	WBP11	mRNA	XR_001436390.1	lncRNA
XM_015094919.1	WBP11	mRNA	XR_001436390.1	lncRNA
XM_004020402.3	RTTN	mRNA	XR_001023627.2	lncRNA
XM_004020402.3	RTTN	mRNA	XR_001023627.2	lncRNA
XM_004020402.3	RTTN	mRNA	XR_001023627.2	lncRNA
XM_015100095.1	LOC101113523	mRNA	XR_001023627.2	lncRNA
XM_015100095.1	LOC101113523	mRNA	XR_001023627.2	lncRNA
XM_015100095.1	LOC101113523	mRNA	XR_001023627.2	lncRNA
XM_015093216.1	TTC39B	mRNA	XR_001023627.2	lncRNA
XM_015093216.1	TTC39B	mRNA	XR_001023627.2	lncRNA
XM_015093216.1	TTC39B	mRNA	XR_001023627.2	lncRNA
XM_012188097.2	PLCB1	mRNA	XR_001434127.1	lncRNA
XM_012188097.2	PLCB1	mRNA	XR_001434127.1	lncRNA
XM_012188097.2	PLCB1	mRNA	XR_001434127.1	lncRNA
XM_012188097.2	PLCB1	mRNA	XR_001023627.2	lncRNA
XM_012188097.2	PLCB1	mRNA	XR_001023627.2	lncRNA
XM_012188097.2	PLCB1	mRNA	XR_001023627.2	lncRNA
XM_004004584.3	RAPGEF4	mRNA	XR_001023627.2	lncRNA
XM_004004584.3	RAPGEF4	mRNA	XR_001023627.2	lncRNA
XM_004004584.3	RAPGEF4	mRNA	XR_001023627.2	lncRNA
XM_012184885.2	DOCK9	mRNA	XR_001023303.2	lncRNA
XM_012184885.2	DOCK9	mRNA	XR_001023303.2	lncRNA
XM_012184885.2	DOCK9	mRNA	XR_001023303.2	lncRNA
XM_012105333.2	USP54	mRNA	XR_001023303.2	lncRNA
XM_012105333.2	USP54	mRNA	XR_001023303.2	lncRNA
XM_012105333.2	USP54	mRNA	XR_001023303.2	lncRNA
XM_012105331.2	USP54	mRNA	XR_001023303.2	lncRNA
XM_012105331.2	USP54	mRNA	XR_001023303.2	lncRNA
XM_012105331.2	USP54	mRNA	XR_001023303.2	lncRNA
XM_015095516.1	LOC101123551	mRNA	XR_001040147.1	lncRNA
XM_015095516.1	LOC101123551	mRNA	XR_001040147.1	lncRNA
XM_015095516.1	LOC101123551	mRNA	XR_001040147.1	lncRNA

Large and medium waves

mRNA	Genesymbol	type	lncRNA	type
XM_012106710.2	CLCN5	mRNA	XR_001436609.1	lncRNA
XM_012106710.2	CLCN5	mRNA	XR_001436609.1	lncRNA
XM_012106710.2	CLCN5	mRNA	XR_001436609.1	lncRNA
XM_012106710.2	CLCN5	mRNA	XR_001436609.1	lncRNA
XM_012106710.2	CLCN5	mRNA	XR_001436609.1	lncRNA
XM_012106710.2	CLCN5	mRNA	XR_001436609.1	lncRNA
XM_012106710.2	CLCN5	mRNA	XR_001436609.1	lncRNA
XM_012106710.2	CLCN5	mRNA	XR_001436609.1	lncRNA
XM_012106710.2	CLCN5	mRNA	XR_001436609.1	lncRNA
XM_012106710.2	CLCN5	mRNA	XR_001436609.1	lncRNA
XM_012106710.2	CLCN5	mRNA	XR_001436609.1	lncRNA
XM_012106710.2	CLCN5	mRNA	XR_001436609.1	lncRNA
XM_012106710.2	CLCN5	mRNA	XR_001436609.1	lncRNA
XM_012106710.2	CLCN5	mRNA	XR_001436609.1	lncRNA
XM_012106710.2	CLCN5	mRNA	XR_001436531.1	lncRNA
XM_012106710.2	CLCN5	mRNA	XR_001436531.1	lncRNA
XM_012106710.2	CLCN5	mRNA	XR_001436531.1	lncRNA
XM_012106710.2	CLCN5	mRNA	XR_001436531.1	lncRNA
XM_012106710.2	CLCN5	mRNA	XR_001436531.1	lncRNA
XM_012106710.2	CLCN5	mRNA	XR_001436531.1	lncRNA
XM_012106710.2	CLCN5	mRNA	XR_001436531.1	lncRNA
XM_012106710.2	CLCN5	mRNA	XR_001436531.1	lncRNA
XM_012106710.2	CLCN5	mRNA	XR_001436531.1	lncRNA
XM_012106710.2	CLCN5	mRNA	XR_001436531.1	lncRNA
XM_012106710.2	CLCN5	mRNA	XR_001436531.1	lncRNA
XM_012106710.2	CLCN5	mRNA	XR_001436531.1	lncRNA
XM_012106710.2	CLCN5	mRNA	XR_001436531.1	lncRNA
XM_012106710.2	CLCN5	mRNA	XR_001436531.1	lncRNA
XM_012106710.2	CLCN5	mRNA	XR_001435726.1	lncRNA
XM_012106710.2	CLCN5	mRNA	XR_001435726.1	lncRNA
XM_012106710.2	CLCN5	mRNA	XR_001435726.1	lncRNA
XM_012106710.2	CLCN5	mRNA	XR_001435726.1	lncRNA
XM_012106710.2	CLCN5	mRNA	XR_001435726.1	lncRNA
XM_012106710.2	CLCN5	mRNA	XR_001435726.1	lncRNA
XM_012106710.2	CLCN5	mRNA	XR_001435726.1	lncRNA
XM_012106710.2	CLCN5	mRNA	XR_001435726.1	lncRNA
XM_012106710.2	CLCN5	mRNA	XR_001435726.1	lncRNA
XM_012106710.2	CLCN5	mRNA	XR_001435726.1	lncRNA
XM_012106710.2	CLCN5	mRNA	XR_001435726.1	lncRNA
XM_012106710.2	CLCN5	mRNA	XR_001435726.1	lncRNA
XM_012106710.2	CLCN5	mRNA	XR_001435726.1	lncRNA
XM_012106710.2	CLCN5	mRNA	XR_001435726.1	lncRNA
XM_015101011.1	TEAD1	mRNA	XR_001435104.1	lncRNA
XM_015101011.1	TEAD1	mRNA	XR_001435104.1	lncRNA
XM_015101011.1	TEAD1	mRNA	XR_001435104.1	lncRNA
XM_015101011.1	TEAD1	mRNA	XR_001435104.1	lncRNA
XM_015101011.1	TEAD1	mRNA	XR_001435104.1	lncRNA
XM_015101011.1	TEAD1	mRNA	XR_001435104.1	lncRNA
XM_015101011.1	TEAD1	mRNA	XR_001435104.1	lncRNA
XM_015101011.1	TEAD1	mRNA	XR_001435104.1	lncRNA
XM_015101011.1	TEAD1	mRNA	XR_001435104.1	lncRNA
XM_015101011.1	TEAD1	mRNA	XR_001435104.1	lncRNA

XM_015101011.1	TEAD1	mRNA	XR_001435104.1	lncRNA
XM_015101011.1	TEAD1	mRNA	XR_001435104.1	lncRNA
XM_015101011.1	TEAD1	mRNA	XR_001435104.1	lncRNA
XM_015100441.1	CHD9	mRNA	XR_001435723.1	lncRNA
XM_015100441.1	CHD9	mRNA	XR_001435723.1	lncRNA
XM_015100441.1	CHD9	mRNA	XR_001435723.1	lncRNA
XM_015100441.1	CHD9	mRNA	XR_001435723.1	lncRNA
XM_015100441.1	CHD9	mRNA	XR_001435723.1	lncRNA
XM_015100441.1	CHD9	mRNA	XR_001435723.1	lncRNA
XM_015100441.1	CHD9	mRNA	XR_001435723.1	lncRNA
XM_015100441.1	CHD9	mRNA	XR_001435723.1	lncRNA
XM_015100441.1	CHD9	mRNA	XR_001435723.1	lncRNA
XM_015100441.1	CHD9	mRNA	XR_001435723.1	lncRNA
XM_015100441.1	CHD9	mRNA	XR_001435723.1	lncRNA
XM_015100441.1	CHD9	mRNA	XR_001435723.1	lncRNA
XM_015100441.1	CHD9	mRNA	XR_001435723.1	lncRNA
XM_015101011.1	TEAD1	mRNA	XR_001435723.1	lncRNA
XM_015101011.1	TEAD1	mRNA	XR_001435723.1	lncRNA
XM_015101011.1	TEAD1	mRNA	XR_001435723.1	lncRNA
XM_015101011.1	TEAD1	mRNA	XR_001435723.1	lncRNA
XM_015101011.1	TEAD1	mRNA	XR_001435723.1	lncRNA
XM_015101011.1	TEAD1	mRNA	XR_001435723.1	lncRNA
XM_015101011.1	TEAD1	mRNA	XR_001435723.1	lncRNA
XM_015101011.1	TEAD1	mRNA	XR_001435723.1	lncRNA
XM_015101011.1	TEAD1	mRNA	XR_001435723.1	lncRNA
XM_015101011.1	TEAD1	mRNA	XR_001435723.1	lncRNA
XM_015101011.1	TEAD1	mRNA	XR_001435723.1	lncRNA
XM_015101011.1	TEAD1	mRNA	XR_001435723.1	lncRNA
XM_015101011.1	TEAD1	mRNA	XR_001435723.1	lncRNA
XM_004013791.3	PERM1	mRNA	XR_001436609.1	lncRNA
XM_004013791.3	PERM1	mRNA	XR_001436609.1	lncRNA
XM_004013791.3	PERM1	mRNA	XR_001436609.1	lncRNA
XM_004013791.3	PERM1	mRNA	XR_001436609.1	lncRNA
XM_004013791.3	PERM1	mRNA	XR_001436609.1	lncRNA
XM_004013791.3	PERM1	mRNA	XR_001436609.1	lncRNA
XM_004013791.3	PERM1	mRNA	XR_001436609.1	lncRNA
XM_004013791.3	PERM1	mRNA	XR_001436609.1	lncRNA
XM_004013791.3	PERM1	mRNA	XR_001436609.1	lncRNA
XM_004013791.3	PERM1	mRNA	XR_001436609.1	lncRNA
XM_004013791.3	PERM1	mRNA	XR_001436609.1	lncRNA
XM_004013791.3	PERM1	mRNA	XR_001436609.1	lncRNA
XM_004013791.3	PERM1	mRNA	XR_001436609.1	lncRNA
XM_012182190.2	EML5	mRNA	XR_001041211.2	lncRNA
XM_012182190.2	EML5	mRNA	XR_001041211.2	lncRNA
XM_012182190.2	EML5	mRNA	XR_001041211.2	lncRNA
XM_012182190.2	EML5	mRNA	XR_001041211.2	lncRNA
XM_012182190.2	EML5	mRNA	XR_001041211.2	lncRNA
XM_012182190.2	EML5	mRNA	XR_001041211.2	lncRNA
XM_012182190.2	EML5	mRNA	XR_001041211.2	lncRNA
XM_012182190.2	EML5	mRNA	XR_001041211.2	lncRNA
XM_012182190.2	EML5	mRNA	XR_001041211.2	lncRNA
XM_012182190.2	EML5	mRNA	XR_001041211.2	lncRNA
XM_012182190.2	EML5	mRNA	XR_001041211.2	lncRNA
XM_012182190.2	EML5	mRNA	XR_001041211.2	lncRNA

XM_012100587.2	BTBD9	mRNA	XR_001435723.1	lncRNA
XM_012100587.2	BTBD9	mRNA	XR_001435723.1	lncRNA
XM_012100587.2	BTBD9	mRNA	XR_001435723.1	lncRNA
XM_012100587.2	BTBD9	mRNA	XR_001435723.1	lncRNA
XM_012100587.2	BTBD9	mRNA	XR_001435723.1	lncRNA
XM_012100587.2	BTBD9	mRNA	XR_001435723.1	lncRNA
XM_012100587.2	BTBD9	mRNA	XR_001435723.1	lncRNA
XM_012100587.2	BTBD9	mRNA	XR_001435723.1	lncRNA
XM_012100587.2	BTBD9	mRNA	XR_001435723.1	lncRNA
XM_012100587.2	BTBD9	mRNA	XR_001435723.1	lncRNA
XM_012100587.2	BTBD9	mRNA	XR_001435723.1	lncRNA
XM_012100587.2	BTBD9	mRNA	XR_001435723.1	lncRNA
XM_012100587.2	BTBD9	mRNA	XR_001435723.1	lncRNA
XM_012100587.2	BTBD9	mRNA	XR_001435726.1	lncRNA
XM_012100587.2	BTBD9	mRNA	XR_001435726.1	lncRNA
XM_012100587.2	BTBD9	mRNA	XR_001435726.1	lncRNA
XM_012100587.2	BTBD9	mRNA	XR_001435726.1	lncRNA
XM_012100587.2	BTBD9	mRNA	XR_001435726.1	lncRNA
XM_012100587.2	BTBD9	mRNA	XR_001435726.1	lncRNA
XM_012100587.2	BTBD9	mRNA	XR_001435726.1	lncRNA
XM_012100587.2	BTBD9	mRNA	XR_001435726.1	lncRNA
XM_012100587.2	BTBD9	mRNA	XR_001435726.1	lncRNA
XM_012100587.2	BTBD9	mRNA	XR_001435726.1	lncRNA
XM_012100587.2	BTBD9	mRNA	XR_001435726.1	lncRNA
XM_012100587.2	BTBD9	mRNA	XR_001435726.1	lncRNA
XM_012100587.2	BTBD9	mRNA	XR_001435726.1	lncRNA
XM_012095461.2	GOLGB1	mRNA	XR_001436609.1	lncRNA
XM_012095461.2	GOLGB1	mRNA	XR_001436609.1	lncRNA
XM_012095461.2	GOLGB1	mRNA	XR_001436609.1	lncRNA
XM_012095461.2	GOLGB1	mRNA	XR_001436609.1	lncRNA
XM_012095461.2	GOLGB1	mRNA	XR_001436609.1	lncRNA
XM_012095461.2	GOLGB1	mRNA	XR_001436609.1	lncRNA
XM_012095461.2	GOLGB1	mRNA	XR_001436609.1	lncRNA
XM_012095461.2	GOLGB1	mRNA	XR_001436609.1	lncRNA
XM_012095461.2	GOLGB1	mRNA	XR_001436609.1	lncRNA
XM_012095461.2	GOLGB1	mRNA	XR_001436609.1	lncRNA
XM_012095461.2	GOLGB1	mRNA	XR_001436609.1	lncRNA
XM_012095461.2	GOLGB1	mRNA	XR_001436609.1	lncRNA
XM_004004717.2	GTDC1	mRNA	XR_001435104.1	lncRNA
XM_004004717.2	GTDC1	mRNA	XR_001435104.1	lncRNA
XM_004004717.2	GTDC1	mRNA	XR_001435104.1	lncRNA
XM_004004717.2	GTDC1	mRNA	XR_001435104.1	lncRNA
XM_004004717.2	GTDC1	mRNA	XR_001435104.1	lncRNA
XM_004004717.2	GTDC1	mRNA	XR_001435104.1	lncRNA
XM_004004717.2	GTDC1	mRNA	XR_001435104.1	lncRNA
XM_004004717.2	GTDC1	mRNA	XR_001435104.1	lncRNA
XM_004004717.2	GTDC1	mRNA	XR_001435104.1	lncRNA
XM_004004717.2	GTDC1	mRNA	XR_001435104.1	lncRNA
XM_004004717.2	GTDC1	mRNA	XR_001435104.1	lncRNA
XM_004004717.2	GTDC1	mRNA	XR_001435104.1	lncRNA
XM_012189777.1	CHD9	mRNA	XR_001435104.1	lncRNA
XM_012189777.1	CHD9	mRNA	XR_001435104.1	lncRNA
XM_012189777.1	CHD9	mRNA	XR_001435104.1	lncRNA
XM_012189777.1	CHD9	mRNA	XR_001435104.1	lncRNA

XM_012189777.1	CHD9	mRNA	XR_001435104.1	lncRNA
XM_012189777.1	CHD9	mRNA	XR_001435104.1	lncRNA
XM_012189777.1	CHD9	mRNA	XR_001435104.1	lncRNA
XM_012189777.1	CHD9	mRNA	XR_001435104.1	lncRNA
XM_012189777.1	CHD9	mRNA	XR_001435104.1	lncRNA
XM_012189777.1	CHD9	mRNA	XR_001435104.1	lncRNA
XM_012189777.1	CHD9	mRNA	XR_001435104.1	lncRNA
XM_012189777.1	CHD9	mRNA	XR_001435104.1	lncRNA
XM_012189777.1	CHD9	mRNA	XR_001436609.1	lncRNA
XM_012189777.1	CHD9	mRNA	XR_001436609.1	lncRNA
XM_012189777.1	CHD9	mRNA	XR_001436609.1	lncRNA
XM_012189777.1	CHD9	mRNA	XR_001436609.1	lncRNA
XM_012189777.1	CHD9	mRNA	XR_001436609.1	lncRNA
XM_012189777.1	CHD9	mRNA	XR_001436609.1	lncRNA
XM_012189777.1	CHD9	mRNA	XR_001436609.1	lncRNA
XM_012189777.1	CHD9	mRNA	XR_001436609.1	lncRNA
XM_012189777.1	CHD9	mRNA	XR_001436609.1	lncRNA
XM_012189777.1	CHD9	mRNA	XR_001436609.1	lncRNA
XM_012189777.1	CHD9	mRNA	XR_001436609.1	lncRNA
XM_012189777.1	CHD9	mRNA	XR_001436609.1	lncRNA
XM_015102269.1	NKTR	mRNA	XR_001435104.1	lncRNA
XM_015102269.1	NKTR	mRNA	XR_001435104.1	lncRNA
XM_015102269.1	NKTR	mRNA	XR_001435104.1	lncRNA
XM_015102269.1	NKTR	mRNA	XR_001435104.1	lncRNA
XM_015102269.1	NKTR	mRNA	XR_001435104.1	lncRNA
XM_015102269.1	NKTR	mRNA	XR_001435104.1	lncRNA
XM_015102269.1	NKTR	mRNA	XR_001435104.1	lncRNA
XM_015102269.1	NKTR	mRNA	XR_001435104.1	lncRNA
XM_015102269.1	NKTR	mRNA	XR_001435104.1	lncRNA
XM_015102269.1	NKTR	mRNA	XR_001435104.1	lncRNA
XM_015102269.1	NKTR	mRNA	XR_001435104.1	lncRNA
XM_015102269.1	NKTR	mRNA	XR_001435104.1	lncRNA
XM_012189777.1	CHD9	mRNA	XR_001435726.1	lncRNA
XM_012189777.1	CHD9	mRNA	XR_001435726.1	lncRNA
XM_012189777.1	CHD9	mRNA	XR_001435726.1	lncRNA
XM_012189777.1	CHD9	mRNA	XR_001435726.1	lncRNA
XM_012189777.1	CHD9	mRNA	XR_001435726.1	lncRNA
XM_012189777.1	CHD9	mRNA	XR_001435726.1	lncRNA
XM_012189777.1	CHD9	mRNA	XR_001435726.1	lncRNA
XM_012189777.1	CHD9	mRNA	XR_001435726.1	lncRNA
XM_012189777.1	CHD9	mRNA	XR_001435726.1	lncRNA
XM_012189777.1	CHD9	mRNA	XR_001435726.1	lncRNA
XM_012189777.1	CHD9	mRNA	XR_001435726.1	lncRNA
XM_012189777.1	CHD9	mRNA	XR_001435726.1	lncRNA
XM_004004717.2	GTDC1	mRNA	XR_001435723.1	lncRNA
XM_004004717.2	GTDC1	mRNA	XR_001435723.1	lncRNA
XM_004004717.2	GTDC1	mRNA	XR_001435723.1	lncRNA
XM_004004717.2	GTDC1	mRNA	XR_001435723.1	lncRNA
XM_004004717.2	GTDC1	mRNA	XR_001435723.1	lncRNA
XM_004004717.2	GTDC1	mRNA	XR_001435723.1	lncRNA
XM_004004717.2	GTDC1	mRNA	XR_001435723.1	lncRNA
XM_004004717.2	GTDC1	mRNA	XR_001435723.1	lncRNA
XM_004004717.2	GTDC1	mRNA	XR_001435723.1	lncRNA
XM_004004717.2	GTDC1	mRNA	XR_001435723.1	lncRNA

XM_004004717.2	GTDC1	mRNA	XR_001435723.1	lncRNA
XM_004004717.2	GTDC1	mRNA	XR_001435723.1	lncRNA
XM_012095733.1	BTBD10	mRNA	XR_001042381.2	lncRNA
XM_012095733.1	BTBD10	mRNA	XR_001042381.2	lncRNA
XM_012095733.1	BTBD10	mRNA	XR_001042381.2	lncRNA
XM_012095733.1	BTBD10	mRNA	XR_001042381.2	lncRNA
XM_012095733.1	BTBD10	mRNA	XR_001042381.2	lncRNA
XM_012095733.1	BTBD10	mRNA	XR_001042381.2	lncRNA
XM_012095733.1	BTBD10	mRNA	XR_001042381.2	lncRNA
XM_012095733.1	BTBD10	mRNA	XR_001042381.2	lncRNA
XM_012095733.1	BTBD10	mRNA	XR_001042381.2	lncRNA
XM_012095733.1	BTBD10	mRNA	XR_001042381.2	lncRNA
XM_012095733.1	BTBD10	mRNA	XR_001042381.2	lncRNA
XM_012095733.1	BTBD10	mRNA	XR_001042381.2	lncRNA
XM_012137673.2	NEB	mRNA	XR_001435726.1	lncRNA
XM_012137673.2	NEB	mRNA	XR_001435726.1	lncRNA
XM_012137673.2	NEB	mRNA	XR_001435726.1	lncRNA
XM_012137673.2	NEB	mRNA	XR_001435726.1	lncRNA
XM_012137673.2	NEB	mRNA	XR_001435726.1	lncRNA
XM_012137673.2	NEB	mRNA	XR_001435726.1	lncRNA
XM_012137673.2	NEB	mRNA	XR_001435726.1	lncRNA
XM_012137673.2	NEB	mRNA	XR_001435726.1	lncRNA
XM_012137673.2	NEB	mRNA	XR_001435726.1	lncRNA
XM_012137673.2	NEB	mRNA	XR_001435726.1	lncRNA
XM_012137673.2	NEB	mRNA	XR_001435726.1	lncRNA
XM_012137673.2	NEB	mRNA	XR_001435726.1	lncRNA
XM_012102471.1	ASAH2	mRNA	XR_001042381.2	lncRNA
XM_012102471.1	ASAH2	mRNA	XR_001042381.2	lncRNA
XM_012102471.1	ASAH2	mRNA	XR_001042381.2	lncRNA
XM_012102471.1	ASAH2	mRNA	XR_001042381.2	lncRNA
XM_012102471.1	ASAH2	mRNA	XR_001042381.2	lncRNA
XM_012102471.1	ASAH2	mRNA	XR_001042381.2	lncRNA
XM_012102471.1	ASAH2	mRNA	XR_001042381.2	lncRNA
XM_012102471.1	ASAH2	mRNA	XR_001042381.2	lncRNA
XM_012102471.1	ASAH2	mRNA	XR_001042381.2	lncRNA
XM_012102471.1	ASAH2	mRNA	XR_001042381.2	lncRNA
XM_012102471.1	ASAH2	mRNA	XR_001042381.2	lncRNA
XM_012102471.1	ASAH2	mRNA	XR_001042381.2	lncRNA
XM_012144336.2	PIKFYVE	mRNA	XR_001435726.1	lncRNA
XM_012144336.2	PIKFYVE	mRNA	XR_001435726.1	lncRNA
XM_012144336.2	PIKFYVE	mRNA	XR_001435726.1	lncRNA
XM_012144336.2	PIKFYVE	mRNA	XR_001435726.1	lncRNA
XM_012144336.2	PIKFYVE	mRNA	XR_001435726.1	lncRNA
XM_012144336.2	PIKFYVE	mRNA	XR_001435726.1	lncRNA
XM_012144336.2	PIKFYVE	mRNA	XR_001435726.1	lncRNA
XM_012144336.2	PIKFYVE	mRNA	XR_001435726.1	lncRNA
XM_012144336.2	PIKFYVE	mRNA	XR_001435726.1	lncRNA
XM_012144336.2	PIKFYVE	mRNA	XR_001435726.1	lncRNA
XM_012144336.2	PIKFYVE	mRNA	XR_001435726.1	lncRNA
XM_012144336.2	PIKFYVE	mRNA	XR_001435726.1	lncRNA
XM_012144336.2	PIKFYVE	mRNA	XR_001435723.1	lncRNA
XM_012144336.2	PIKFYVE	mRNA	XR_001435723.1	lncRNA
XM_012144336.2	PIKFYVE	mRNA	XR_001435723.1	lncRNA
XM_012144336.2	PIKFYVE	mRNA	XR_001435723.1	lncRNA

XM_012144336.2	PIKFYVE	mRNA	XR_001435723.1	lncRNA
XM_012144336.2	PIKFYVE	mRNA	XR_001435723.1	lncRNA
XM_012144336.2	PIKFYVE	mRNA	XR_001435723.1	lncRNA
XM_012144336.2	PIKFYVE	mRNA	XR_001435723.1	lncRNA
XM_012144336.2	PIKFYVE	mRNA	XR_001435723.1	lncRNA
XM_012144336.2	PIKFYVE	mRNA	XR_001435723.1	lncRNA
XM_012144336.2	PIKFYVE	mRNA	XR_001435723.1	lncRNA
XM_012144336.2	PIKFYVE	mRNA	XR_001435723.1	lncRNA
XM_015102269.1	NKTR	mRNA	XR_001033284.2	lncRNA
XM_015102269.1	NKTR	mRNA	XR_001033284.2	lncRNA
XM_015102269.1	NKTR	mRNA	XR_001033284.2	lncRNA
XM_015102269.1	NKTR	mRNA	XR_001033284.2	lncRNA
XM_015102269.1	NKTR	mRNA	XR_001033284.2	lncRNA
XM_015102269.1	NKTR	mRNA	XR_001033284.2	lncRNA
XM_015102269.1	NKTR	mRNA	XR_001033284.2	lncRNA
XM_015102269.1	NKTR	mRNA	XR_001033284.2	lncRNA
XM_015102269.1	NKTR	mRNA	XR_001033284.2	lncRNA
XM_015102269.1	NKTR	mRNA	XR_001033284.2	lncRNA
XM_015102269.1	NKTR	mRNA	XR_001033284.2	lncRNA
XM_012182190.2	EML5	mRNA	XR_001436609.1	lncRNA
XM_012182190.2	EML5	mRNA	XR_001436609.1	lncRNA
XM_012182190.2	EML5	mRNA	XR_001436609.1	lncRNA
XM_012182190.2	EML5	mRNA	XR_001436609.1	lncRNA
XM_012182190.2	EML5	mRNA	XR_001436609.1	lncRNA
XM_012182190.2	EML5	mRNA	XR_001436609.1	lncRNA
XM_012182190.2	EML5	mRNA	XR_001436609.1	lncRNA
XM_012182190.2	EML5	mRNA	XR_001436609.1	lncRNA
XM_012182190.2	EML5	mRNA	XR_001436609.1	lncRNA
XM_012182190.2	EML5	mRNA	XR_001436609.1	lncRNA
XM_012182190.2	EML5	mRNA	XR_001436609.1	lncRNA
XM_012182190.2	EML5	mRNA	XR_001436609.1	lncRNA
XM_015104838.1	PDK3	mRNA	XR_001436531.1	lncRNA
XM_015104838.1	PDK3	mRNA	XR_001436531.1	lncRNA
XM_015104838.1	PDK3	mRNA	XR_001436531.1	lncRNA
XM_015104838.1	PDK3	mRNA	XR_001436531.1	lncRNA
XM_015104838.1	PDK3	mRNA	XR_001436531.1	lncRNA
XM_015104838.1	PDK3	mRNA	XR_001436531.1	lncRNA
XM_015104838.1	PDK3	mRNA	XR_001436531.1	lncRNA
XM_015104838.1	PDK3	mRNA	XR_001436531.1	lncRNA
XM_015104838.1	PDK3	mRNA	XR_001436531.1	lncRNA
XM_015104838.1	PDK3	mRNA	XR_001436531.1	lncRNA
XM_015104838.1	PDK3	mRNA	XR_001436531.1	lncRNA
XM_015104838.1	PDK3	mRNA	XR_001436531.1	lncRNA
XM_012184887.2	DOCK9	mRNA	XR_001435104.1	lncRNA
XM_012184887.2	DOCK9	mRNA	XR_001435104.1	lncRNA
XM_012184887.2	DOCK9	mRNA	XR_001435104.1	lncRNA
XM_012184887.2	DOCK9	mRNA	XR_001435104.1	lncRNA
XM_012184887.2	DOCK9	mRNA	XR_001435104.1	lncRNA
XM_012184887.2	DOCK9	mRNA	XR_001435104.1	lncRNA
XM_012184887.2	DOCK9	mRNA	XR_001435104.1	lncRNA
XM_012184887.2	DOCK9	mRNA	XR_001435104.1	lncRNA
XM_012184887.2	DOCK9	mRNA	XR_001435104.1	lncRNA
XM_012184887.2	DOCK9	mRNA	XR_001435104.1	lncRNA
XM_012184887.2	DOCK9	mRNA	XR_001435104.1	lncRNA

XM_012184887.2	DOCK9	mRNA	XR_001435104.1	lncRNA
XM_015091887.1	MYCL	mRNA	XR_001435723.1	lncRNA
XM_015091887.1	MYCL	mRNA	XR_001435723.1	lncRNA
XM_015091887.1	MYCL	mRNA	XR_001435723.1	lncRNA
XM_015091887.1	MYCL	mRNA	XR_001435723.1	lncRNA
XM_015091887.1	MYCL	mRNA	XR_001435723.1	lncRNA
XM_015091887.1	MYCL	mRNA	XR_001435723.1	lncRNA
XM_015091887.1	MYCL	mRNA	XR_001435723.1	lncRNA
XM_015091887.1	MYCL	mRNA	XR_001435723.1	lncRNA
XM_015091887.1	MYCL	mRNA	XR_001435723.1	lncRNA
XM_015091887.1	MYCL	mRNA	XR_001435723.1	lncRNA
XM_015091887.1	MYCL	mRNA	XR_001435723.1	lncRNA
XM_015091887.1	MYCL	mRNA	XR_001435723.1	lncRNA
XM_015091887.1	MYCL	mRNA	XR_001435726.1	lncRNA
XM_015091887.1	MYCL	mRNA	XR_001435726.1	lncRNA
XM_015091887.1	MYCL	mRNA	XR_001435726.1	lncRNA
XM_015091887.1	MYCL	mRNA	XR_001435726.1	lncRNA
XM_015091887.1	MYCL	mRNA	XR_001435726.1	lncRNA
XM_015091887.1	MYCL	mRNA	XR_001435726.1	lncRNA
XM_015091887.1	MYCL	mRNA	XR_001435726.1	lncRNA
XM_015091887.1	MYCL	mRNA	XR_001435726.1	lncRNA
XM_015091887.1	MYCL	mRNA	XR_001435726.1	lncRNA
XM_015091887.1	MYCL	mRNA	XR_001435726.1	lncRNA
XM_015091887.1	MYCL	mRNA	XR_001435726.1	lncRNA
XM_015091887.1	MYCL	mRNA	XR_001435726.1	lncRNA
XM_012097252.2	RHOF	mRNA	XR_001436609.1	lncRNA
XM_012097252.2	RHOF	mRNA	XR_001436609.1	lncRNA
XM_012097252.2	RHOF	mRNA	XR_001436609.1	lncRNA
XM_012097252.2	RHOF	mRNA	XR_001436609.1	lncRNA
XM_012097252.2	RHOF	mRNA	XR_001436609.1	lncRNA
XM_012097252.2	RHOF	mRNA	XR_001436609.1	lncRNA
XM_012097252.2	RHOF	mRNA	XR_001436609.1	lncRNA
XM_012097252.2	RHOF	mRNA	XR_001436609.1	lncRNA
XM_012097252.2	RHOF	mRNA	XR_001436609.1	lncRNA
XM_012097252.2	RHOF	mRNA	XR_001436609.1	lncRNA
XM_012097252.2	RHOF	mRNA	XR_001436609.1	lncRNA
XM_012097252.2	RHOF	mRNA	XR_001436609.1	lncRNA
XM_012113242.2	KIAA1958	mRNA	XR_001436531.1	lncRNA
XM_012113242.2	KIAA1958	mRNA	XR_001436531.1	lncRNA
XM_012113242.2	KIAA1958	mRNA	XR_001436531.1	lncRNA
XM_012113242.2	KIAA1958	mRNA	XR_001436531.1	lncRNA
XM_012113242.2	KIAA1958	mRNA	XR_001436531.1	lncRNA
XM_012113242.2	KIAA1958	mRNA	XR_001436531.1	lncRNA
XM_012113242.2	KIAA1958	mRNA	XR_001436531.1	lncRNA
XM_012113242.2	KIAA1958	mRNA	XR_001436531.1	lncRNA
XM_012113242.2	KIAA1958	mRNA	XR_001436531.1	lncRNA
XM_012113242.2	KIAA1958	mRNA	XR_001436531.1	lncRNA
XM_012113242.2	KIAA1958	mRNA	XR_001436531.1	lncRNA
XM_012113242.2	KIAA1958	mRNA	XR_001436531.1	lncRNA
XM_012106710.2	CLCN5	mRNA	XR_001436099.1	lncRNA
XM_012106710.2	CLCN5	mRNA	XR_001436099.1	lncRNA
XM_012106710.2	CLCN5	mRNA	XR_001436099.1	lncRNA
XM_012106710.2	CLCN5	mRNA	XR_001436099.1	lncRNA
XM_012106710.2	CLCN5	mRNA	XR_001436099.1	lncRNA

XM_012106710.2	CLCN5	mRNA	XR_001436099.1	lncRNA
XM_012106710.2	CLCN5	mRNA	XR_001436099.1	lncRNA
XM_012106710.2	CLCN5	mRNA	XR_001436099.1	lncRNA
XM_012106710.2	CLCN5	mRNA	XR_001436099.1	lncRNA
XM_012106710.2	CLCN5	mRNA	XR_001436099.1	lncRNA
XM_012106710.2	CLCN5	mRNA	XR_001436099.1	lncRNA
XM_015101011.1	TEAD1	mRNA	XR_001041209.2	lncRNA
XM_015101011.1	TEAD1	mRNA	XR_001041209.2	lncRNA
XM_015101011.1	TEAD1	mRNA	XR_001041209.2	lncRNA
XM_015101011.1	TEAD1	mRNA	XR_001041209.2	lncRNA
XM_015101011.1	TEAD1	mRNA	XR_001041209.2	lncRNA
XM_015101011.1	TEAD1	mRNA	XR_001041209.2	lncRNA
XM_015101011.1	TEAD1	mRNA	XR_001041209.2	lncRNA
XM_015101011.1	TEAD1	mRNA	XR_001041209.2	lncRNA
XM_015101011.1	TEAD1	mRNA	XR_001041209.2	lncRNA
XM_015101011.1	TEAD1	mRNA	XR_001041209.2	lncRNA
XM_015101011.1	TEAD1	mRNA	XR_001041209.2	lncRNA
XM_015100441.1	CHD9	mRNA	XR_001041209.2	lncRNA
XM_015100441.1	CHD9	mRNA	XR_001041209.2	lncRNA
XM_015100441.1	CHD9	mRNA	XR_001041209.2	lncRNA
XM_015100441.1	CHD9	mRNA	XR_001041209.2	lncRNA
XM_015100441.1	CHD9	mRNA	XR_001041209.2	lncRNA
XM_015100441.1	CHD9	mRNA	XR_001041209.2	lncRNA
XM_015100441.1	CHD9	mRNA	XR_001041209.2	lncRNA
XM_015100441.1	CHD9	mRNA	XR_001041209.2	lncRNA
XM_015100441.1	CHD9	mRNA	XR_001041209.2	lncRNA
XM_015100441.1	CHD9	mRNA	XR_001041209.2	lncRNA
XM_015100441.1	CHD9	mRNA	XR_001041209.2	lncRNA
XM_015102914.1	NAV2	mRNA	XR_001041209.2	lncRNA
XM_015102914.1	NAV2	mRNA	XR_001041209.2	lncRNA
XM_015102914.1	NAV2	mRNA	XR_001041209.2	lncRNA
XM_015102914.1	NAV2	mRNA	XR_001041209.2	lncRNA
XM_015102914.1	NAV2	mRNA	XR_001041209.2	lncRNA
XM_015102914.1	NAV2	mRNA	XR_001041209.2	lncRNA
XM_015102914.1	NAV2	mRNA	XR_001041209.2	lncRNA
XM_015102914.1	NAV2	mRNA	XR_001041209.2	lncRNA
XM_015102914.1	NAV2	mRNA	XR_001041209.2	lncRNA
XM_015102914.1	NAV2	mRNA	XR_001041209.2	lncRNA
XM_015102914.1	NAV2	mRNA	XR_001041209.2	lncRNA
XM_012099994.2	VPRBP	mRNA	XR_001435104.1	lncRNA
XM_012099994.2	VPRBP	mRNA	XR_001435104.1	lncRNA
XM_012099994.2	VPRBP	mRNA	XR_001435104.1	lncRNA
XM_012099994.2	VPRBP	mRNA	XR_001435104.1	lncRNA
XM_012099994.2	VPRBP	mRNA	XR_001435104.1	lncRNA
XM_012099994.2	VPRBP	mRNA	XR_001435104.1	lncRNA
XM_012099994.2	VPRBP	mRNA	XR_001435104.1	lncRNA
XM_012099994.2	VPRBP	mRNA	XR_001435104.1	lncRNA
XM_012099994.2	VPRBP	mRNA	XR_001435104.1	lncRNA
XM_012099994.2	VPRBP	mRNA	XR_001435104.1	lncRNA
XM_012099994.2	VPRBP	mRNA	XR_001435104.1	lncRNA
XM_012098886.2	TP53BP1	mRNA	XR_001436609.1	lncRNA
XM_012098886.2	TP53BP1	mRNA	XR_001436609.1	lncRNA
XM_012098886.2	TP53BP1	mRNA	XR_001436609.1	lncRNA
XM_012098886.2	TP53BP1	mRNA	XR_001436609.1	lncRNA

XM_012098886.2	TP53BP1	mRNA	XR_001436609.1	lncRNA
XM_012098886.2	TP53BP1	mRNA	XR_001436609.1	lncRNA
XM_012098886.2	TP53BP1	mRNA	XR_001436609.1	lncRNA
XM_012098886.2	TP53BP1	mRNA	XR_001436609.1	lncRNA
XM_012098886.2	TP53BP1	mRNA	XR_001436609.1	lncRNA
XM_012098886.2	TP53BP1	mRNA	XR_001436609.1	lncRNA
XM_012098886.2	TP53BP1	mRNA	XR_001436609.1	lncRNA
XM_015096057.1	KDM3B	mRNA	XR_001435104.1	lncRNA
XM_015096057.1	KDM3B	mRNA	XR_001435104.1	lncRNA
XM_015096057.1	KDM3B	mRNA	XR_001435104.1	lncRNA
XM_015096057.1	KDM3B	mRNA	XR_001435104.1	lncRNA
XM_015096057.1	KDM3B	mRNA	XR_001435104.1	lncRNA
XM_015096057.1	KDM3B	mRNA	XR_001435104.1	lncRNA
XM_015096057.1	KDM3B	mRNA	XR_001435104.1	lncRNA
XM_015096057.1	KDM3B	mRNA	XR_001435104.1	lncRNA
XM_015096057.1	KDM3B	mRNA	XR_001435104.1	lncRNA
XM_015096057.1	KDM3B	mRNA	XR_001435104.1	lncRNA
XM_015096057.1	KDM3B	mRNA	XR_001435104.1	lncRNA
XM_012182854.2	PTPRK	mRNA	XR_001435104.1	lncRNA
XM_012182854.2	PTPRK	mRNA	XR_001435104.1	lncRNA
XM_012182854.2	PTPRK	mRNA	XR_001435104.1	lncRNA
XM_012182854.2	PTPRK	mRNA	XR_001435104.1	lncRNA
XM_012182854.2	PTPRK	mRNA	XR_001435104.1	lncRNA
XM_012182854.2	PTPRK	mRNA	XR_001435104.1	lncRNA
XM_012182854.2	PTPRK	mRNA	XR_001435104.1	lncRNA
XM_012182854.2	PTPRK	mRNA	XR_001435104.1	lncRNA
XM_012182854.2	PTPRK	mRNA	XR_001435104.1	lncRNA
XM_012182854.2	PTPRK	mRNA	XR_001435104.1	lncRNA
XM_012182854.2	PTPRK	mRNA	XR_001435104.1	lncRNA
XM_012187548.2	GIGYF2	mRNA	XR_001435104.1	lncRNA
XM_012187548.2	GIGYF2	mRNA	XR_001435104.1	lncRNA
XM_012187548.2	GIGYF2	mRNA	XR_001435104.1	lncRNA
XM_012187548.2	GIGYF2	mRNA	XR_001435104.1	lncRNA
XM_012187548.2	GIGYF2	mRNA	XR_001435104.1	lncRNA
XM_012187548.2	GIGYF2	mRNA	XR_001435104.1	lncRNA
XM_012187548.2	GIGYF2	mRNA	XR_001435104.1	lncRNA
XM_012187548.2	GIGYF2	mRNA	XR_001435104.1	lncRNA
XM_012187548.2	GIGYF2	mRNA	XR_001435104.1	lncRNA
XM_012187548.2	GIGYF2	mRNA	XR_001435104.1	lncRNA
XM_012187548.2	GIGYF2	mRNA	XR_001435104.1	lncRNA
XM_015101107.1	TCP11L1	mRNA	XR_001033284.2	lncRNA
XM_015101107.1	TCP11L1	mRNA	XR_001033284.2	lncRNA
XM_015101107.1	TCP11L1	mRNA	XR_001033284.2	lncRNA
XM_015101107.1	TCP11L1	mRNA	XR_001033284.2	lncRNA
XM_015101107.1	TCP11L1	mRNA	XR_001033284.2	lncRNA
XM_015101107.1	TCP11L1	mRNA	XR_001033284.2	lncRNA
XM_015101107.1	TCP11L1	mRNA	XR_001033284.2	lncRNA
XM_015101107.1	TCP11L1	mRNA	XR_001033284.2	lncRNA
XM_015101107.1	TCP11L1	mRNA	XR_001033284.2	lncRNA
XM_015101107.1	TCP11L1	mRNA	XR_001033284.2	lncRNA
XM_012106710.2	CLCN5	mRNA	XR_001041209.2	lncRNA
XM_012106710.2	CLCN5	mRNA	XR_001041209.2	lncRNA
XM_012106710.2	CLCN5	mRNA	XR_001041209.2	lncRNA
XM_012106710.2	CLCN5	mRNA	XR_001041209.2	lncRNA

XM_012106710.2	CLCN5	mRNA	XR_001041209.2	lncRNA
XM_012106710.2	CLCN5	mRNA	XR_001041209.2	lncRNA
XM_012106710.2	CLCN5	mRNA	XR_001041209.2	lncRNA
XM_012106710.2	CLCN5	mRNA	XR_001041209.2	lncRNA
XM_012106710.2	CLCN5	mRNA	XR_001041209.2	lncRNA
XM_012106710.2	CLCN5	mRNA	XR_001041209.2	lncRNA
XM_012106710.2	CLCN5	mRNA	XR_001041209.2	lncRNA
XM_012100587.2	BTBD9	mRNA	XR_001041209.2	lncRNA
XM_012100587.2	BTBD9	mRNA	XR_001041209.2	lncRNA
XM_012100587.2	BTBD9	mRNA	XR_001041209.2	lncRNA
XM_012100587.2	BTBD9	mRNA	XR_001041209.2	lncRNA
XM_012100587.2	BTBD9	mRNA	XR_001041209.2	lncRNA
XM_012100587.2	BTBD9	mRNA	XR_001041209.2	lncRNA
XM_012100587.2	BTBD9	mRNA	XR_001041209.2	lncRNA
XM_012100587.2	BTBD9	mRNA	XR_001041209.2	lncRNA
XM_012100587.2	BTBD9	mRNA	XR_001041209.2	lncRNA
XM_012100587.2	BTBD9	mRNA	XR_001041209.2	lncRNA
XM_012100587.2	BTBD9	mRNA	XR_001041209.2	lncRNA
XM_015102310.1	SETD5	mRNA	XR_001021939.1	lncRNA
XM_015102310.1	SETD5	mRNA	XR_001021939.1	lncRNA
XM_015102310.1	SETD5	mRNA	XR_001021939.1	lncRNA
XM_015102310.1	SETD5	mRNA	XR_001021939.1	lncRNA
XM_015102310.1	SETD5	mRNA	XR_001021939.1	lncRNA
XM_015102310.1	SETD5	mRNA	XR_001021939.1	lncRNA
XM_015102310.1	SETD5	mRNA	XR_001021939.1	lncRNA
XM_015102310.1	SETD5	mRNA	XR_001021939.1	lncRNA
XM_015102310.1	SETD5	mRNA	XR_001021939.1	lncRNA
XM_015102310.1	SETD5	mRNA	XR_001021939.1	lncRNA
XM_015102310.1	SETD5	mRNA	XR_001021939.1	lncRNA
XM_004009100.3	PAM	mRNA	XR_001042381.2	lncRNA
XM_004009100.3	PAM	mRNA	XR_001042381.2	lncRNA
XM_004009100.3	PAM	mRNA	XR_001042381.2	lncRNA
XM_004009100.3	PAM	mRNA	XR_001042381.2	lncRNA
XM_004009100.3	PAM	mRNA	XR_001042381.2	lncRNA
XM_004009100.3	PAM	mRNA	XR_001042381.2	lncRNA
XM_004009100.3	PAM	mRNA	XR_001042381.2	lncRNA
XM_004009100.3	PAM	mRNA	XR_001042381.2	lncRNA
XM_004009100.3	PAM	mRNA	XR_001042381.2	lncRNA
XM_004009100.3	PAM	mRNA	XR_001042381.2	lncRNA
XM_004009100.3	PAM	mRNA	XR_001042381.2	lncRNA
XM_004003157.3	TNIK	mRNA	XR_001435104.1	lncRNA
XM_004003157.3	TNIK	mRNA	XR_001435104.1	lncRNA
XM_004003157.3	TNIK	mRNA	XR_001435104.1	lncRNA
XM_004003157.3	TNIK	mRNA	XR_001435104.1	lncRNA
XM_004003157.3	TNIK	mRNA	XR_001435104.1	lncRNA
XM_004003157.3	TNIK	mRNA	XR_001435104.1	lncRNA
XM_004003157.3	TNIK	mRNA	XR_001435104.1	lncRNA
XM_004003157.3	TNIK	mRNA	XR_001435104.1	lncRNA
XM_004003157.3	TNIK	mRNA	XR_001435104.1	lncRNA
XM_004003157.3	TNIK	mRNA	XR_001435104.1	lncRNA
XM_004003157.3	TNIK	mRNA	XR_001435104.1	lncRNA
XM_012095733.1	BTBD10	mRNA	XR_001436609.1	lncRNA
XM_012095733.1	BTBD10	mRNA	XR_001436609.1	lncRNA
XM_012095733.1	BTBD10	mRNA	XR_001436609.1	lncRNA

XM_012095733.1	BTBD10	mRNA	XR_001436609.1	lncRNA
XM_012095733.1	BTBD10	mRNA	XR_001436609.1	lncRNA
XM_012095733.1	BTBD10	mRNA	XR_001436609.1	lncRNA
XM_012095733.1	BTBD10	mRNA	XR_001436609.1	lncRNA
XM_012095733.1	BTBD10	mRNA	XR_001436609.1	lncRNA
XM_012095733.1	BTBD10	mRNA	XR_001436609.1	lncRNA
XM_012095733.1	BTBD10	mRNA	XR_001436609.1	lncRNA
XM_012095733.1	BTBD10	mRNA	XR_001436609.1	lncRNA
XM_012095465.2	GOLGB1	mRNA	XR_001435104.1	lncRNA
XM_012095465.2	GOLGB1	mRNA	XR_001435104.1	lncRNA
XM_012095465.2	GOLGB1	mRNA	XR_001435104.1	lncRNA
XM_012095465.2	GOLGB1	mRNA	XR_001435104.1	lncRNA
XM_012095465.2	GOLGB1	mRNA	XR_001435104.1	lncRNA
XM_012095465.2	GOLGB1	mRNA	XR_001435104.1	lncRNA
XM_012095465.2	GOLGB1	mRNA	XR_001435104.1	lncRNA
XM_012095465.2	GOLGB1	mRNA	XR_001435104.1	lncRNA
XM_012095465.2	GOLGB1	mRNA	XR_001435104.1	lncRNA
XM_012095465.2	GOLGB1	mRNA	XR_001435104.1	lncRNA
XM_012095465.2	GOLGB1	mRNA	XR_001435104.1	lncRNA
XM_012137599.2	NEB	mRNA	XR_001436531.1	lncRNA
XM_012137599.2	NEB	mRNA	XR_001436531.1	lncRNA
XM_012137599.2	NEB	mRNA	XR_001436531.1	lncRNA
XM_012137599.2	NEB	mRNA	XR_001436531.1	lncRNA
XM_012137599.2	NEB	mRNA	XR_001436531.1	lncRNA
XM_012137599.2	NEB	mRNA	XR_001436531.1	lncRNA
XM_012137599.2	NEB	mRNA	XR_001436531.1	lncRNA
XM_012137599.2	NEB	mRNA	XR_001436531.1	lncRNA
XM_012137599.2	NEB	mRNA	XR_001436531.1	lncRNA
XM_012137599.2	NEB	mRNA	XR_001436531.1	lncRNA
XM_012137599.2	NEB	mRNA	XR_001436531.1	lncRNA
XM_015102310.1	SETD5	mRNA	XR_001435104.1	lncRNA
XM_015102310.1	SETD5	mRNA	XR_001435104.1	lncRNA
XM_015102310.1	SETD5	mRNA	XR_001435104.1	lncRNA
XM_015102310.1	SETD5	mRNA	XR_001435104.1	lncRNA
XM_015102310.1	SETD5	mRNA	XR_001435104.1	lncRNA
XM_015102310.1	SETD5	mRNA	XR_001435104.1	lncRNA
XM_015102310.1	SETD5	mRNA	XR_001435104.1	lncRNA
XM_015102310.1	SETD5	mRNA	XR_001435104.1	lncRNA
XM_015102310.1	SETD5	mRNA	XR_001435104.1	lncRNA
XM_015102310.1	SETD5	mRNA	XR_001435104.1	lncRNA
XM_015102310.1	SETD5	mRNA	XR_001435104.1	lncRNA
XM_012184887.2	DOCK9	mRNA	XR_001021939.1	lncRNA
XM_012184887.2	DOCK9	mRNA	XR_001021939.1	lncRNA
XM_012184887.2	DOCK9	mRNA	XR_001021939.1	lncRNA
XM_012184887.2	DOCK9	mRNA	XR_001021939.1	lncRNA
XM_012184887.2	DOCK9	mRNA	XR_001021939.1	lncRNA
XM_012184887.2	DOCK9	mRNA	XR_001021939.1	lncRNA
XM_012184887.2	DOCK9	mRNA	XR_001021939.1	lncRNA
XM_012184887.2	DOCK9	mRNA	XR_001021939.1	lncRNA
XM_012184887.2	DOCK9	mRNA	XR_001021939.1	lncRNA
XM_012184887.2	DOCK9	mRNA	XR_001021939.1	lncRNA
XM_012184887.2	DOCK9	mRNA	XR_001021939.1	lncRNA
XM_012104178.2	CCP110	mRNA	XR_001042381.2	lncRNA
XM_012104178.2	CCP110	mRNA	XR_001042381.2	lncRNA

XM_012104178.2	CCP110	mRNA	XR_001042381.2	lncRNA
XM_012104178.2	CCP110	mRNA	XR_001042381.2	lncRNA
XM_012104178.2	CCP110	mRNA	XR_001042381.2	lncRNA
XM_012104178.2	CCP110	mRNA	XR_001042381.2	lncRNA
XM_012104178.2	CCP110	mRNA	XR_001042381.2	lncRNA
XM_012104178.2	CCP110	mRNA	XR_001042381.2	lncRNA
XM_012104178.2	CCP110	mRNA	XR_001042381.2	lncRNA
XM_012104178.2	CCP110	mRNA	XR_001042381.2	lncRNA
XM_012104178.2	CCP110	mRNA	XR_001042381.2	lncRNA
XM_015095780.1	DNAJB1	mRNA	XR_001435726.1	lncRNA
XM_015095780.1	DNAJB1	mRNA	XR_001435726.1	lncRNA
XM_015095780.1	DNAJB1	mRNA	XR_001435726.1	lncRNA
XM_015095780.1	DNAJB1	mRNA	XR_001435726.1	lncRNA
XM_015095780.1	DNAJB1	mRNA	XR_001435726.1	lncRNA
XM_015095780.1	DNAJB1	mRNA	XR_001435726.1	lncRNA
XM_015095780.1	DNAJB1	mRNA	XR_001435726.1	lncRNA
XM_015095780.1	DNAJB1	mRNA	XR_001435726.1	lncRNA
XM_015095780.1	DNAJB1	mRNA	XR_001435726.1	lncRNA
XM_015095780.1	DNAJB1	mRNA	XR_001435726.1	lncRNA
XM_015095780.1	DNAJB1	mRNA	XR_001435726.1	lncRNA
XM_012189186.2	RALGAPB	mRNA	XR_001435104.1	lncRNA
XM_012189186.2	RALGAPB	mRNA	XR_001435104.1	lncRNA
XM_012189186.2	RALGAPB	mRNA	XR_001435104.1	lncRNA
XM_012189186.2	RALGAPB	mRNA	XR_001435104.1	lncRNA
XM_012189186.2	RALGAPB	mRNA	XR_001435104.1	lncRNA
XM_012189186.2	RALGAPB	mRNA	XR_001435104.1	lncRNA
XM_012189186.2	RALGAPB	mRNA	XR_001435104.1	lncRNA
XM_012189186.2	RALGAPB	mRNA	XR_001435104.1	lncRNA
XM_012189186.2	RALGAPB	mRNA	XR_001435104.1	lncRNA
XM_012189186.2	RALGAPB	mRNA	XR_001435104.1	lncRNA
XM_012189186.2	RALGAPB	mRNA	XR_001435104.1	lncRNA
XM_004004717.2	GTDC1	mRNA	XR_001033284.2	lncRNA
XM_004004717.2	GTDC1	mRNA	XR_001033284.2	lncRNA
XM_004004717.2	GTDC1	mRNA	XR_001033284.2	lncRNA
XM_004004717.2	GTDC1	mRNA	XR_001033284.2	lncRNA
XM_004004717.2	GTDC1	mRNA	XR_001033284.2	lncRNA
XM_004004717.2	GTDC1	mRNA	XR_001033284.2	lncRNA
XM_004004717.2	GTDC1	mRNA	XR_001033284.2	lncRNA
XM_004004717.2	GTDC1	mRNA	XR_001033284.2	lncRNA
XM_004004717.2	GTDC1	mRNA	XR_001033284.2	lncRNA
XM_004004717.2	GTDC1	mRNA	XR_001033284.2	lncRNA
XM_015096057.1	KDM3B	mRNA	XR_001041211.2	lncRNA
XM_015096057.1	KDM3B	mRNA	XR_001041211.2	lncRNA
XM_015096057.1	KDM3B	mRNA	XR_001041211.2	lncRNA
XM_015096057.1	KDM3B	mRNA	XR_001041211.2	lncRNA
XM_015096057.1	KDM3B	mRNA	XR_001041211.2	lncRNA
XM_015096057.1	KDM3B	mRNA	XR_001041211.2	lncRNA
XM_015096057.1	KDM3B	mRNA	XR_001041211.2	lncRNA
XM_015096057.1	KDM3B	mRNA	XR_001041211.2	lncRNA
XM_015096057.1	KDM3B	mRNA	XR_001041211.2	lncRNA
XM_015096057.1	KDM3B	mRNA	XR_001041211.2	lncRNA
XM_012187340.2	KIF1B	mRNA	XR_001435726.1	lncRNA
XM_012187340.2	KIF1B	mRNA	XR_001435726.1	lncRNA
XM_012187340.2	KIF1B	mRNA	XR_001435726.1	lncRNA

XM_012187340.2	KIF1B	mRNA	XR_001435726.1	lncRNA
XM_012187340.2	KIF1B	mRNA	XR_001435726.1	lncRNA
XM_012187340.2	KIF1B	mRNA	XR_001435726.1	lncRNA
XM_012187340.2	KIF1B	mRNA	XR_001435726.1	lncRNA
XM_012187340.2	KIF1B	mRNA	XR_001435726.1	lncRNA
XM_012187340.2	KIF1B	mRNA	XR_001435726.1	lncRNA
XM_012187340.2	KIF1B	mRNA	XR_001435726.1	lncRNA
XM_012187340.2	KIF1B	mRNA	XR_001435726.1	lncRNA
XM_012187340.2	KIF1B	mRNA	XR_001435723.1	lncRNA
XM_012187340.2	KIF1B	mRNA	XR_001435723.1	lncRNA
XM_012187340.2	KIF1B	mRNA	XR_001435723.1	lncRNA
XM_012187340.2	KIF1B	mRNA	XR_001435723.1	lncRNA
XM_012187340.2	KIF1B	mRNA	XR_001435723.1	lncRNA
XM_012187340.2	KIF1B	mRNA	XR_001435723.1	lncRNA
XM_012187340.2	KIF1B	mRNA	XR_001435723.1	lncRNA
XM_012187340.2	KIF1B	mRNA	XR_001435723.1	lncRNA
XM_012187340.2	KIF1B	mRNA	XR_001435723.1	lncRNA
XM_012187340.2	KIF1B	mRNA	XR_001435723.1	lncRNA
XM_012187340.2	KIF1B	mRNA	XR_001435723.1	lncRNA
XM_015102914.1	NAV2	mRNA	XR_001435723.1	lncRNA
XM_015102914.1	NAV2	mRNA	XR_001435723.1	lncRNA
XM_015102914.1	NAV2	mRNA	XR_001435723.1	lncRNA
XM_015102914.1	NAV2	mRNA	XR_001435723.1	lncRNA
XM_015102914.1	NAV2	mRNA	XR_001435723.1	lncRNA
XM_015102914.1	NAV2	mRNA	XR_001435723.1	lncRNA
XM_015102914.1	NAV2	mRNA	XR_001435723.1	lncRNA
XM_015102914.1	NAV2	mRNA	XR_001435723.1	lncRNA
XM_015102914.1	NAV2	mRNA	XR_001435723.1	lncRNA
XM_015102914.1	NAV2	mRNA	XR_001435723.1	lncRNA
XM_015102914.1	NAV2	mRNA	XR_001435723.1	lncRNA
XM_012097252.2	RHOF	mRNA	XR_001435104.1	lncRNA
XM_012097252.2	RHOF	mRNA	XR_001435104.1	lncRNA
XM_012097252.2	RHOF	mRNA	XR_001435104.1	lncRNA
XM_012097252.2	RHOF	mRNA	XR_001435104.1	lncRNA
XM_012097252.2	RHOF	mRNA	XR_001435104.1	lncRNA
XM_012097252.2	RHOF	mRNA	XR_001435104.1	lncRNA
XM_012097252.2	RHOF	mRNA	XR_001435104.1	lncRNA
XM_012097252.2	RHOF	mRNA	XR_001435104.1	lncRNA
XM_012097252.2	RHOF	mRNA	XR_001435104.1	lncRNA
XM_012097252.2	RHOF	mRNA	XR_001435104.1	lncRNA
XM_012097252.2	RHOF	mRNA	XR_001435104.1	lncRNA
XM_015101107.1	TCP11L1	mRNA	XR_001435104.1	lncRNA
XM_015101107.1	TCP11L1	mRNA	XR_001435104.1	lncRNA
XM_015101107.1	TCP11L1	mRNA	XR_001435104.1	lncRNA
XM_015101107.1	TCP11L1	mRNA	XR_001435104.1	lncRNA
XM_015101107.1	TCP11L1	mRNA	XR_001435104.1	lncRNA
XM_015101107.1	TCP11L1	mRNA	XR_001435104.1	lncRNA
XM_015101107.1	TCP11L1	mRNA	XR_001435104.1	lncRNA
XM_015101107.1	TCP11L1	mRNA	XR_001435104.1	lncRNA
XM_015101107.1	TCP11L1	mRNA	XR_001435104.1	lncRNA
XM_015101107.1	TCP11L1	mRNA	XR_001435104.1	lncRNA
XM_015102352.1	PXK	mRNA	XR_001436531.1	lncRNA
XM_015102352.1	PXK	mRNA	XR_001436531.1	lncRNA
XM_015102352.1	PXK	mRNA	XR_001436531.1	lncRNA

XM_015102352.1	PXK	mRNA	XR_001436531.1	lncRNA
XM_015102352.1	PXK	mRNA	XR_001436531.1	lncRNA
XM_015102352.1	PXK	mRNA	XR_001436531.1	lncRNA
XM_015102352.1	PXK	mRNA	XR_001436531.1	lncRNA
XM_015102352.1	PXK	mRNA	XR_001436531.1	lncRNA
XM_015102352.1	PXK	mRNA	XR_001436531.1	lncRNA
XM_015102352.1	PXK	mRNA	XR_001436531.1	lncRNA
XM_012182538.2	TRAPPC3L	mRNA	XR_001435104.1	lncRNA
XM_012182538.2	TRAPPC3L	mRNA	XR_001435104.1	lncRNA
XM_012182538.2	TRAPPC3L	mRNA	XR_001435104.1	lncRNA
XM_012182538.2	TRAPPC3L	mRNA	XR_001435104.1	lncRNA
XM_012182538.2	TRAPPC3L	mRNA	XR_001435104.1	lncRNA
XM_012182538.2	TRAPPC3L	mRNA	XR_001435104.1	lncRNA
XM_012182538.2	TRAPPC3L	mRNA	XR_001435104.1	lncRNA
XM_012182538.2	TRAPPC3L	mRNA	XR_001435104.1	lncRNA
XM_012182538.2	TRAPPC3L	mRNA	XR_001435104.1	lncRNA
XM_012182538.2	TRAPPC3L	mRNA	XR_001435104.1	lncRNA
XM_012096185.2	TCP11L1	mRNA	XR_001436609.1	lncRNA
XM_012096185.2	TCP11L1	mRNA	XR_001436609.1	lncRNA
XM_012096185.2	TCP11L1	mRNA	XR_001436609.1	lncRNA
XM_012096185.2	TCP11L1	mRNA	XR_001436609.1	lncRNA
XM_012096185.2	TCP11L1	mRNA	XR_001436609.1	lncRNA
XM_012096185.2	TCP11L1	mRNA	XR_001436609.1	lncRNA
XM_012096185.2	TCP11L1	mRNA	XR_001436609.1	lncRNA
XM_012096185.2	TCP11L1	mRNA	XR_001436609.1	lncRNA
XM_012096185.2	TCP11L1	mRNA	XR_001436609.1	lncRNA
XM_012096185.2	TCP11L1	mRNA	XR_001436609.1	lncRNA
XM_015101107.1	TCP11L1	mRNA	XR_001436609.1	lncRNA
XM_015101107.1	TCP11L1	mRNA	XR_001436609.1	lncRNA
XM_015101107.1	TCP11L1	mRNA	XR_001436609.1	lncRNA
XM_015101107.1	TCP11L1	mRNA	XR_001436609.1	lncRNA
XM_015101107.1	TCP11L1	mRNA	XR_001436609.1	lncRNA
XM_015101107.1	TCP11L1	mRNA	XR_001436609.1	lncRNA
XM_015101107.1	TCP11L1	mRNA	XR_001436609.1	lncRNA
XM_015101107.1	TCP11L1	mRNA	XR_001436609.1	lncRNA
XM_015101107.1	TCP11L1	mRNA	XR_001436609.1	lncRNA
XM_015101107.1	TCP11L1	mRNA	XR_001436609.1	lncRNA
XM_012103019.2	INPP5F	mRNA	XR_001436609.1	lncRNA
XM_012103019.2	INPP5F	mRNA	XR_001436609.1	lncRNA
XM_012103019.2	INPP5F	mRNA	XR_001436609.1	lncRNA
XM_012103019.2	INPP5F	mRNA	XR_001436609.1	lncRNA
XM_012103019.2	INPP5F	mRNA	XR_001436609.1	lncRNA
XM_012103019.2	INPP5F	mRNA	XR_001436609.1	lncRNA
XM_012103019.2	INPP5F	mRNA	XR_001436609.1	lncRNA
XM_012103019.2	INPP5F	mRNA	XR_001436609.1	lncRNA
XM_012103019.2	INPP5F	mRNA	XR_001436609.1	lncRNA
XM_012103019.2	INPP5F	mRNA	XR_001436609.1	lncRNA
XM_012183298.1	FAM120B	mRNA	XR_001435104.1	lncRNA
XM_012183298.1	FAM120B	mRNA	XR_001435104.1	lncRNA
XM_012183298.1	FAM120B	mRNA	XR_001435104.1	lncRNA
XM_012183298.1	FAM120B	mRNA	XR_001435104.1	lncRNA
XM_012183298.1	FAM120B	mRNA	XR_001435104.1	lncRNA
XM_012183298.1	FAM120B	mRNA	XR_001435104.1	lncRNA
XM_012183298.1	FAM120B	mRNA	XR_001435104.1	lncRNA

XM_012183298.1	FAM120B	mRNA	XR_001435104.1	lncRNA
XM_012183298.1	FAM120B	mRNA	XR_001435104.1	lncRNA
XM_012183298.1	FAM120B	mRNA	XR_001435104.1	lncRNA
XM_015100441.1	CHD9	mRNA	XR_001033284.2	lncRNA
XM_015100441.1	CHD9	mRNA	XR_001033284.2	lncRNA
XM_015100441.1	CHD9	mRNA	XR_001033284.2	lncRNA
XM_015100441.1	CHD9	mRNA	XR_001033284.2	lncRNA
XM_015100441.1	CHD9	mRNA	XR_001033284.2	lncRNA
XM_015100441.1	CHD9	mRNA	XR_001033284.2	lncRNA
XM_015100441.1	CHD9	mRNA	XR_001033284.2	lncRNA
XM_015100441.1	CHD9	mRNA	XR_001033284.2	lncRNA
XM_015100441.1	CHD9	mRNA	XR_001033284.2	lncRNA
XM_015100441.1	CHD9	mRNA	XR_001033284.2	lncRNA
XM_012095465.2	GOLGB1	mRNA	XR_001041209.2	lncRNA
XM_012095465.2	GOLGB1	mRNA	XR_001041209.2	lncRNA
XM_012095465.2	GOLGB1	mRNA	XR_001041209.2	lncRNA
XM_012095465.2	GOLGB1	mRNA	XR_001041209.2	lncRNA
XM_012095465.2	GOLGB1	mRNA	XR_001041209.2	lncRNA
XM_012095465.2	GOLGB1	mRNA	XR_001041209.2	lncRNA
XM_012095465.2	GOLGB1	mRNA	XR_001041209.2	lncRNA
XM_012095465.2	GOLGB1	mRNA	XR_001041209.2	lncRNA
XM_012095465.2	GOLGB1	mRNA	XR_001041209.2	lncRNA
XM_012095465.2	GOLGB1	mRNA	XR_001041209.2	lncRNA
XM_012095461.2	GOLGB1	mRNA	XR_001041211.2	lncRNA
XM_012095461.2	GOLGB1	mRNA	XR_001041211.2	lncRNA
XM_012095461.2	GOLGB1	mRNA	XR_001041211.2	lncRNA
XM_012095461.2	GOLGB1	mRNA	XR_001041211.2	lncRNA
XM_012095461.2	GOLGB1	mRNA	XR_001041211.2	lncRNA
XM_012095461.2	GOLGB1	mRNA	XR_001041211.2	lncRNA
XM_012095461.2	GOLGB1	mRNA	XR_001041211.2	lncRNA
XM_012095461.2	GOLGB1	mRNA	XR_001041211.2	lncRNA
XM_012095461.2	GOLGB1	mRNA	XR_001041211.2	lncRNA
XM_012095461.2	GOLGB1	mRNA	XR_001041211.2	lncRNA
XM_004003140.2	NAALADL2	mRNA	XR_001435726.1	lncRNA
XM_004003140.2	NAALADL2	mRNA	XR_001435726.1	lncRNA
XM_004003140.2	NAALADL2	mRNA	XR_001435726.1	lncRNA
XM_004003140.2	NAALADL2	mRNA	XR_001435726.1	lncRNA
XM_004003140.2	NAALADL2	mRNA	XR_001435726.1	lncRNA
XM_004003140.2	NAALADL2	mRNA	XR_001435726.1	lncRNA
XM_004003140.2	NAALADL2	mRNA	XR_001435726.1	lncRNA
XM_004003140.2	NAALADL2	mRNA	XR_001435726.1	lncRNA
XM_004003140.2	NAALADL2	mRNA	XR_001435726.1	lncRNA
XM_004003140.2	NAALADL2	mRNA	XR_001435726.1	lncRNA
XM_012101922.2	ZBTB44	mRNA	XR_001435726.1	lncRNA
XM_012101922.2	ZBTB44	mRNA	XR_001435726.1	lncRNA
XM_012101922.2	ZBTB44	mRNA	XR_001435726.1	lncRNA
XM_012101922.2	ZBTB44	mRNA	XR_001435726.1	lncRNA
XM_012101922.2	ZBTB44	mRNA	XR_001435726.1	lncRNA
XM_012101922.2	ZBTB44	mRNA	XR_001435726.1	lncRNA
XM_012101922.2	ZBTB44	mRNA	XR_001435726.1	lncRNA
XM_012101922.2	ZBTB44	mRNA	XR_001435726.1	lncRNA
XM_012101922.2	ZBTB44	mRNA	XR_001435726.1	lncRNA
XM_012101922.2	ZBTB44	mRNA	XR_001435726.1	lncRNA
XM_004006510.3	SLC16A7	mRNA	XR_001435726.1	lncRNA

XM_004006510.3	SLC16A7	mRNA	XR_001435726.1	lncRNA
XM_004006510.3	SLC16A7	mRNA	XR_001435726.1	lncRNA
XM_004006510.3	SLC16A7	mRNA	XR_001435726.1	lncRNA
XM_004006510.3	SLC16A7	mRNA	XR_001435726.1	lncRNA
XM_004006510.3	SLC16A7	mRNA	XR_001435726.1	lncRNA
XM_004006510.3	SLC16A7	mRNA	XR_001435726.1	lncRNA
XM_004006510.3	SLC16A7	mRNA	XR_001435726.1	lncRNA
XM_004006510.3	SLC16A7	mRNA	XR_001435726.1	lncRNA
XM_004006510.3	SLC16A7	mRNA	XR_001435726.1	lncRNA
XM_015093495.1	NBEAL1	mRNA	XR_001435723.1	lncRNA
XM_015093495.1	NBEAL1	mRNA	XR_001435723.1	lncRNA
XM_015093495.1	NBEAL1	mRNA	XR_001435723.1	lncRNA
XM_015093495.1	NBEAL1	mRNA	XR_001435723.1	lncRNA
XM_015093495.1	NBEAL1	mRNA	XR_001435723.1	lncRNA
XM_015093495.1	NBEAL1	mRNA	XR_001435723.1	lncRNA
XM_015093495.1	NBEAL1	mRNA	XR_001435723.1	lncRNA
XM_015093495.1	NBEAL1	mRNA	XR_001435723.1	lncRNA
XM_015093495.1	NBEAL1	mRNA	XR_001435723.1	lncRNA
XM_015093495.1	NBEAL1	mRNA	XR_001435723.1	lncRNA
XM_004006510.3	SLC16A7	mRNA	XR_001435723.1	lncRNA
XM_004006510.3	SLC16A7	mRNA	XR_001435723.1	lncRNA
XM_004006510.3	SLC16A7	mRNA	XR_001435723.1	lncRNA
XM_004006510.3	SLC16A7	mRNA	XR_001435723.1	lncRNA
XM_004006510.3	SLC16A7	mRNA	XR_001435723.1	lncRNA
XM_004006510.3	SLC16A7	mRNA	XR_001435723.1	lncRNA
XM_004006510.3	SLC16A7	mRNA	XR_001435723.1	lncRNA
XM_004006510.3	SLC16A7	mRNA	XR_001435723.1	lncRNA
XM_004006510.3	SLC16A7	mRNA	XR_001435723.1	lncRNA
XM_004006510.3	SLC16A7	mRNA	XR_001435723.1	lncRNA
XM_004003140.2	NAALADL2	mRNA	XR_001435723.1	lncRNA
XM_004003140.2	NAALADL2	mRNA	XR_001435723.1	lncRNA
XM_004003140.2	NAALADL2	mRNA	XR_001435723.1	lncRNA
XM_004003140.2	NAALADL2	mRNA	XR_001435723.1	lncRNA
XM_004003140.2	NAALADL2	mRNA	XR_001435723.1	lncRNA
XM_004003140.2	NAALADL2	mRNA	XR_001435723.1	lncRNA
XM_004003140.2	NAALADL2	mRNA	XR_001435723.1	lncRNA
XM_004003140.2	NAALADL2	mRNA	XR_001435723.1	lncRNA
XM_004003140.2	NAALADL2	mRNA	XR_001435723.1	lncRNA
XM_004003140.2	NAALADL2	mRNA	XR_001435723.1	lncRNA
XM_012175754.2	PARVB	mRNA	XR_001042381.2	lncRNA
XM_012175754.2	PARVB	mRNA	XR_001042381.2	lncRNA
XM_012175754.2	PARVB	mRNA	XR_001042381.2	lncRNA
XM_012175754.2	PARVB	mRNA	XR_001042381.2	lncRNA
XM_012175754.2	PARVB	mRNA	XR_001042381.2	lncRNA
XM_012175754.2	PARVB	mRNA	XR_001042381.2	lncRNA
XM_012175754.2	PARVB	mRNA	XR_001042381.2	lncRNA
XM_012175754.2	PARVB	mRNA	XR_001042381.2	lncRNA
XM_012175754.2	PARVB	mRNA	XR_001042381.2	lncRNA
XM_012175754.2	PARVB	mRNA	XR_001042381.2	lncRNA
XM_012099994.2	VPRBP	mRNA	XR_001021939.1	lncRNA
XM_012099994.2	VPRBP	mRNA	XR_001021939.1	lncRNA
XM_012099994.2	VPRBP	mRNA	XR_001021939.1	lncRNA
XM_012099994.2	VPRBP	mRNA	XR_001021939.1	lncRNA
XM_012099994.2	VPRBP	mRNA	XR_001021939.1	lncRNA

XM_012099994.2	VPRBP	mRNA	XR_001021939.1	lncRNA
XM_012099994.2	VPRBP	mRNA	XR_001021939.1	lncRNA
XM_012099994.2	VPRBP	mRNA	XR_001021939.1	lncRNA
XM_012099994.2	VPRBP	mRNA	XR_001021939.1	lncRNA
XM_012099994.2	VPRBP	mRNA	XR_001021939.1	lncRNA
XM_012105198.2	TET1	mRNA	XR_001033284.2	lncRNA
XM_012105198.2	TET1	mRNA	XR_001033284.2	lncRNA
XM_012105198.2	TET1	mRNA	XR_001033284.2	lncRNA
XM_012105198.2	TET1	mRNA	XR_001033284.2	lncRNA
XM_012105198.2	TET1	mRNA	XR_001033284.2	lncRNA
XM_012105198.2	TET1	mRNA	XR_001033284.2	lncRNA
XM_012105198.2	TET1	mRNA	XR_001033284.2	lncRNA
XM_012105198.2	TET1	mRNA	XR_001033284.2	lncRNA
XM_012105198.2	TET1	mRNA	XR_001033284.2	lncRNA
XM_012189777.1	CHD9	mRNA	XR_001434081.1	lncRNA
XM_012189777.1	CHD9	mRNA	XR_001434081.1	lncRNA
XM_012189777.1	CHD9	mRNA	XR_001434081.1	lncRNA
XM_012189777.1	CHD9	mRNA	XR_001434081.1	lncRNA
XM_012189777.1	CHD9	mRNA	XR_001434081.1	lncRNA
XM_012189777.1	CHD9	mRNA	XR_001434081.1	lncRNA
XM_012189777.1	CHD9	mRNA	XR_001434081.1	lncRNA
XM_012189777.1	CHD9	mRNA	XR_001434081.1	lncRNA
XM_012189777.1	CHD9	mRNA	XR_001434081.1	lncRNA
XM_012189777.1	CHD9	mRNA	XR_001434081.1	lncRNA
XM_015102914.1	NAV2	mRNA	TCONS_00029399	lncRNA
XM_015102914.1	NAV2	mRNA	TCONS_00029399	lncRNA
XM_015102914.1	NAV2	mRNA	TCONS_00029399	lncRNA
XM_015102914.1	NAV2	mRNA	TCONS_00029399	lncRNA
XM_015102914.1	NAV2	mRNA	TCONS_00029399	lncRNA
XM_015102914.1	NAV2	mRNA	TCONS_00029399	lncRNA
XM_015102914.1	NAV2	mRNA	TCONS_00029399	lncRNA
XM_015102914.1	NAV2	mRNA	TCONS_00029399	lncRNA
XM_015102914.1	NAV2	mRNA	TCONS_00029399	lncRNA
XM_015102914.1	NAV2	mRNA	TCONS_00029399	lncRNA
XM_015101011.1	TEAD1	mRNA	TCONS_00029399	lncRNA
XM_015101011.1	TEAD1	mRNA	TCONS_00029399	lncRNA
XM_015101011.1	TEAD1	mRNA	TCONS_00029399	lncRNA
XM_015101011.1	TEAD1	mRNA	TCONS_00029399	lncRNA
XM_015101011.1	TEAD1	mRNA	TCONS_00029399	lncRNA
XM_015101011.1	TEAD1	mRNA	TCONS_00029399	lncRNA
XM_015101011.1	TEAD1	mRNA	TCONS_00029399	lncRNA
XM_015101011.1	TEAD1	mRNA	TCONS_00029399	lncRNA
XM_015101011.1	TEAD1	mRNA	TCONS_00029399	lncRNA
XM_015101011.1	TEAD1	mRNA	TCONS_00029399	lncRNA
XM_015091887.1	MYCL	mRNA	XR_001041209.2	lncRNA
XM_015091887.1	MYCL	mRNA	XR_001041209.2	lncRNA
XM_015091887.1	MYCL	mRNA	XR_001041209.2	lncRNA
XM_015091887.1	MYCL	mRNA	XR_001041209.2	lncRNA
XM_015091887.1	MYCL	mRNA	XR_001041209.2	lncRNA
XM_015091887.1	MYCL	mRNA	XR_001041209.2	lncRNA
XM_015091887.1	MYCL	mRNA	XR_001041209.2	lncRNA
XM_015091887.1	MYCL	mRNA	XR_001041209.2	lncRNA
XM_015091887.1	MYCL	mRNA	XR_001041209.2	lncRNA
XM_015091887.1	MYCL	mRNA	XR_001041209.2	lncRNA

XM_015100441.1	CHD9	mRNA	TCONS_00029399	lncRNA
XM_015100441.1	CHD9	mRNA	TCONS_00029399	lncRNA
XM_015100441.1	CHD9	mRNA	TCONS_00029399	lncRNA
XM_015100441.1	CHD9	mRNA	TCONS_00029399	lncRNA
XM_015100441.1	CHD9	mRNA	TCONS_00029399	lncRNA
XM_015100441.1	CHD9	mRNA	TCONS_00029399	lncRNA
XM_015100441.1	CHD9	mRNA	TCONS_00029399	lncRNA
XM_015100441.1	CHD9	mRNA	TCONS_00029399	lncRNA
XM_015100441.1	CHD9	mRNA	TCONS_00029399	lncRNA
XM_015100441.1	CHD9	mRNA	TCONS_00029399	lncRNA
XM_004017884.2	AKAP6	mRNA	XR_001041211.2	lncRNA
XM_004017884.2	AKAP6	mRNA	XR_001041211.2	lncRNA
XM_004017884.2	AKAP6	mRNA	XR_001041211.2	lncRNA
XM_004017884.2	AKAP6	mRNA	XR_001041211.2	lncRNA
XM_004017884.2	AKAP6	mRNA	XR_001041211.2	lncRNA
XM_004017884.2	AKAP6	mRNA	XR_001041211.2	lncRNA
XM_004017884.2	AKAP6	mRNA	XR_001041211.2	lncRNA
XM_004017884.2	AKAP6	mRNA	XR_001041211.2	lncRNA
XM_004017884.2	AKAP6	mRNA	XR_001041211.2	lncRNA
XM_004017884.2	AKAP6	mRNA	XR_001041211.2	lncRNA
XM_012159370.2	LOC1011215(	mRNA	XR_001041202.1	lncRNA
XM_012159370.2	LOC1011215(	mRNA	XR_001041202.1	lncRNA
XM_012159370.2	LOC1011215(	mRNA	XR_001041202.1	lncRNA
XM_012159370.2	LOC1011215(	mRNA	XR_001041202.1	lncRNA
XM_012159370.2	LOC1011215(	mRNA	XR_001041202.1	lncRNA
XM_012159370.2	LOC1011215(	mRNA	XR_001041202.1	lncRNA
XM_012159370.2	LOC1011215(	mRNA	XR_001041202.1	lncRNA
XM_012159370.2	LOC1011215(	mRNA	XR_001041202.1	lncRNA
XM_012159370.2	LOC1011215(	mRNA	XR_001041202.1	lncRNA
XM_015102352.1	PXK	mRNA	XR_001041202.1	lncRNA
XM_015102352.1	PXK	mRNA	XR_001041202.1	lncRNA
XM_015102352.1	PXK	mRNA	XR_001041202.1	lncRNA
XM_015102352.1	PXK	mRNA	XR_001041202.1	lncRNA
XM_015102352.1	PXK	mRNA	XR_001041202.1	lncRNA
XM_015102352.1	PXK	mRNA	XR_001041202.1	lncRNA
XM_015102352.1	PXK	mRNA	XR_001041202.1	lncRNA
XM_015102352.1	PXK	mRNA	XR_001041202.1	lncRNA
XM_015102352.1	PXK	mRNA	XR_001041202.1	lncRNA
XM_012096185.2	TCP11L1	mRNA	XR_001041202.1	lncRNA
XM_012096185.2	TCP11L1	mRNA	XR_001041202.1	lncRNA
XM_012096185.2	TCP11L1	mRNA	XR_001041202.1	lncRNA
XM_012096185.2	TCP11L1	mRNA	XR_001041202.1	lncRNA
XM_012096185.2	TCP11L1	mRNA	XR_001041202.1	lncRNA
XM_012096185.2	TCP11L1	mRNA	XR_001041202.1	lncRNA
XM_012096185.2	TCP11L1	mRNA	XR_001041202.1	lncRNA
XM_012096185.2	TCP11L1	mRNA	XR_001041202.1	lncRNA
XM_012096185.2	TCP11L1	mRNA	XR_001041202.1	lncRNA
XM_015097611.1	VPS13B	mRNA	XR_001436609.1	lncRNA
XM_015097611.1	VPS13B	mRNA	XR_001436609.1	lncRNA
XM_015097611.1	VPS13B	mRNA	XR_001436609.1	lncRNA
XM_015097611.1	VPS13B	mRNA	XR_001436609.1	lncRNA
XM_015097611.1	VPS13B	mRNA	XR_001436609.1	lncRNA
XM_015097611.1	VPS13B	mRNA	XR_001436609.1	lncRNA
XM_015097611.1	VPS13B	mRNA	XR_001436609.1	lncRNA

XM_015097611.1	VPS13B	mRNA	XR_001436609.1	lncRNA
XM_015097611.1	VPS13B	mRNA	XR_001436609.1	lncRNA
XM_015097611.1	VPS13B	mRNA	XR_001436609.1	lncRNA
XM_012167258.2	CLIP4	mRNA	XR_001436531.1	lncRNA
XM_012167258.2	CLIP4	mRNA	XR_001436531.1	lncRNA
XM_012167258.2	CLIP4	mRNA	XR_001436531.1	lncRNA
XM_012167258.2	CLIP4	mRNA	XR_001436531.1	lncRNA
XM_012167258.2	CLIP4	mRNA	XR_001436531.1	lncRNA
XM_012167258.2	CLIP4	mRNA	XR_001436531.1	lncRNA
XM_012167258.2	CLIP4	mRNA	XR_001436531.1	lncRNA
XM_012167258.2	CLIP4	mRNA	XR_001436531.1	lncRNA
XM_012167258.2	CLIP4	mRNA	XR_001436531.1	lncRNA
XM_012167258.2	CLIP4	mRNA	XR_001436531.1	lncRNA
XM_015095269.1	NRCAM	mRNA	XR_001436531.1	lncRNA
XM_015095269.1	NRCAM	mRNA	XR_001436531.1	lncRNA
XM_015095269.1	NRCAM	mRNA	XR_001436531.1	lncRNA
XM_015095269.1	NRCAM	mRNA	XR_001436531.1	lncRNA
XM_015095269.1	NRCAM	mRNA	XR_001436531.1	lncRNA
XM_015095269.1	NRCAM	mRNA	XR_001436531.1	lncRNA
XM_015095269.1	NRCAM	mRNA	XR_001436531.1	lncRNA
XM_015095269.1	NRCAM	mRNA	XR_001436531.1	lncRNA
XM_015095269.1	NRCAM	mRNA	XR_001436531.1	lncRNA
XM_015095269.1	NRCAM	mRNA	XR_001436531.1	lncRNA
XM_012152455.2	LUZP1	mRNA	XR_001435104.1	lncRNA
XM_012152455.2	LUZP1	mRNA	XR_001435104.1	lncRNA
XM_012152455.2	LUZP1	mRNA	XR_001435104.1	lncRNA
XM_012152455.2	LUZP1	mRNA	XR_001435104.1	lncRNA
XM_012152455.2	LUZP1	mRNA	XR_001435104.1	lncRNA
XM_012152455.2	LUZP1	mRNA	XR_001435104.1	lncRNA
XM_012152455.2	LUZP1	mRNA	XR_001435104.1	lncRNA
XM_012152455.2	LUZP1	mRNA	XR_001435104.1	lncRNA
XM_012152455.2	LUZP1	mRNA	XR_001435104.1	lncRNA
XM_012152455.2	LUZP1	mRNA	XR_001435104.1	lncRNA
XM_015094919.1	WBP11	mRNA	XR_001435104.1	lncRNA
XM_015094919.1	WBP11	mRNA	XR_001435104.1	lncRNA
XM_015094919.1	WBP11	mRNA	XR_001435104.1	lncRNA
XM_015094919.1	WBP11	mRNA	XR_001435104.1	lncRNA
XM_015094919.1	WBP11	mRNA	XR_001435104.1	lncRNA
XM_015094919.1	WBP11	mRNA	XR_001435104.1	lncRNA
XM_015094919.1	WBP11	mRNA	XR_001435104.1	lncRNA
XM_015094919.1	WBP11	mRNA	XR_001435104.1	lncRNA
XM_015094919.1	WBP11	mRNA	XR_001435104.1	lncRNA
XM_015094919.1	WBP11	mRNA	XR_001435104.1	lncRNA
XM_012122525.2	PRUNE2	mRNA	XR_001436609.1	lncRNA
XM_012122525.2	PRUNE2	mRNA	XR_001436609.1	lncRNA
XM_012122525.2	PRUNE2	mRNA	XR_001436609.1	lncRNA
XM_012122525.2	PRUNE2	mRNA	XR_001436609.1	lncRNA
XM_012122525.2	PRUNE2	mRNA	XR_001436609.1	lncRNA
XM_012122525.2	PRUNE2	mRNA	XR_001436609.1	lncRNA
XM_012122525.2	PRUNE2	mRNA	XR_001436609.1	lncRNA
XM_012122525.2	PRUNE2	mRNA	XR_001436609.1	lncRNA
XM_012122525.2	PRUNE2	mRNA	XR_001436609.1	lncRNA
XM_012122525.2	PRUNE2	mRNA	XR_001436609.1	lncRNA
XM_012098886.2	TP53BP1	mRNA	XR_001435104.1	lncRNA

XM_012098886.2	TP53BP1	mRNA	XR_001435104.1	lncRNA
XM_012098886.2	TP53BP1	mRNA	XR_001435104.1	lncRNA
XM_012098886.2	TP53BP1	mRNA	XR_001435104.1	lncRNA
XM_012098886.2	TP53BP1	mRNA	XR_001435104.1	lncRNA
XM_012098886.2	TP53BP1	mRNA	XR_001435104.1	lncRNA
XM_012098886.2	TP53BP1	mRNA	XR_001435104.1	lncRNA
XM_012098886.2	TP53BP1	mRNA	XR_001435104.1	lncRNA
XM_012098886.2	TP53BP1	mRNA	XR_001435104.1	lncRNA
XM_012098886.2	TP53BP1	mRNA	XR_001435104.1	lncRNA
XM_012096035.1	C2CD3	mRNA	XR_001436531.1	lncRNA
XM_012096035.1	C2CD3	mRNA	XR_001436531.1	lncRNA
XM_012096035.1	C2CD3	mRNA	XR_001436531.1	lncRNA
XM_012096035.1	C2CD3	mRNA	XR_001436531.1	lncRNA
XM_012096035.1	C2CD3	mRNA	XR_001436531.1	lncRNA
XM_012096035.1	C2CD3	mRNA	XR_001436531.1	lncRNA
XM_012096035.1	C2CD3	mRNA	XR_001436531.1	lncRNA
XM_012096035.1	C2CD3	mRNA	XR_001436531.1	lncRNA
XM_012096035.1	C2CD3	mRNA	XR_001436531.1	lncRNA
XM_012096035.1	C2CD3	mRNA	XR_001436531.1	lncRNA
XM_012173782.2	REV1	mRNA	XR_001436609.1	lncRNA
XM_012173782.2	REV1	mRNA	XR_001436609.1	lncRNA
XM_012173782.2	REV1	mRNA	XR_001436609.1	lncRNA
XM_012173782.2	REV1	mRNA	XR_001436609.1	lncRNA
XM_012173782.2	REV1	mRNA	XR_001436609.1	lncRNA
XM_012173782.2	REV1	mRNA	XR_001436609.1	lncRNA
XM_012173782.2	REV1	mRNA	XR_001436609.1	lncRNA
XM_012173782.2	REV1	mRNA	XR_001436609.1	lncRNA
XM_012173782.2	REV1	mRNA	XR_001436609.1	lncRNA
XM_012173782.2	REV1	mRNA	XR_001436609.1	lncRNA
XM_012167258.2	CLIP4	mRNA	XR_001436609.1	lncRNA
XM_012167258.2	CLIP4	mRNA	XR_001436609.1	lncRNA
XM_012167258.2	CLIP4	mRNA	XR_001436609.1	lncRNA
XM_012167258.2	CLIP4	mRNA	XR_001436609.1	lncRNA
XM_012167258.2	CLIP4	mRNA	XR_001436609.1	lncRNA
XM_012167258.2	CLIP4	mRNA	XR_001436609.1	lncRNA
XM_012167258.2	CLIP4	mRNA	XR_001436609.1	lncRNA
XM_012167258.2	CLIP4	mRNA	XR_001436609.1	lncRNA
XM_012167258.2	CLIP4	mRNA	XR_001436609.1	lncRNA
XM_012167258.2	CLIP4	mRNA	XR_001436609.1	lncRNA
XM_012146543.1	ATG9A	mRNA	XR_001021939.1	lncRNA
XM_012146543.1	ATG9A	mRNA	XR_001021939.1	lncRNA
XM_012146543.1	ATG9A	mRNA	XR_001021939.1	lncRNA
XM_012146543.1	ATG9A	mRNA	XR_001021939.1	lncRNA
XM_012146543.1	ATG9A	mRNA	XR_001021939.1	lncRNA
XM_012146543.1	ATG9A	mRNA	XR_001021939.1	lncRNA
XM_012146543.1	ATG9A	mRNA	XR_001021939.1	lncRNA
XM_012146543.1	ATG9A	mRNA	XR_001021939.1	lncRNA
XM_012146543.1	ATG9A	mRNA	XR_001021939.1	lncRNA
XM_012146543.1	ATG9A	mRNA	XR_001021939.1	lncRNA
XM_004013791.3	PERM1	mRNA	XR_001041211.2	lncRNA
XM_004013791.3	PERM1	mRNA	XR_001041211.2	lncRNA
XM_004013791.3	PERM1	mRNA	XR_001041211.2	lncRNA
XM_004013791.3	PERM1	mRNA	XR_001041211.2	lncRNA
XM_004013791.3	PERM1	mRNA	XR_001041211.2	lncRNA

XM_004013791.3	PERM1	mRNA	XR_001041211.2	lncRNA
XM_004013791.3	PERM1	mRNA	XR_001041211.2	lncRNA
XM_004013791.3	PERM1	mRNA	XR_001041211.2	lncRNA
XM_004013791.3	PERM1	mRNA	XR_001041211.2	lncRNA
XM_004013791.3	PERM1	mRNA	XR_001041211.2	lncRNA
XM_012187340.2	KIF1B	mRNA	XR_001434081.1	lncRNA
XM_012187340.2	KIF1B	mRNA	XR_001434081.1	lncRNA
XM_012187340.2	KIF1B	mRNA	XR_001434081.1	lncRNA
XM_012187340.2	KIF1B	mRNA	XR_001434081.1	lncRNA
XM_012187340.2	KIF1B	mRNA	XR_001434081.1	lncRNA
XM_012187340.2	KIF1B	mRNA	XR_001434081.1	lncRNA
XM_012187340.2	KIF1B	mRNA	XR_001434081.1	lncRNA
XM_012187340.2	KIF1B	mRNA	XR_001434081.1	lncRNA
XM_012187340.2	KIF1B	mRNA	XR_001434081.1	lncRNA
XM_012187340.2	KIF1B	mRNA	XR_001434081.1	lncRNA
XM_004015120.2	PHLPP2	mRNA	XR_001042381.2	lncRNA
XM_004015120.2	PHLPP2	mRNA	XR_001042381.2	lncRNA
XM_004015120.2	PHLPP2	mRNA	XR_001042381.2	lncRNA
XM_004015120.2	PHLPP2	mRNA	XR_001042381.2	lncRNA
XM_004015120.2	PHLPP2	mRNA	XR_001042381.2	lncRNA
XM_004015120.2	PHLPP2	mRNA	XR_001042381.2	lncRNA
XM_004015120.2	PHLPP2	mRNA	XR_001042381.2	lncRNA
XM_004015120.2	PHLPP2	mRNA	XR_001042381.2	lncRNA
XM_004015120.2	PHLPP2	mRNA	XR_001042381.2	lncRNA
XM_004015120.2	PHLPP2	mRNA	XR_001042381.2	lncRNA
XM_015096939.1	BMF	mRNA	XR_001042381.2	lncRNA
XM_015096939.1	BMF	mRNA	XR_001042381.2	lncRNA
XM_015096939.1	BMF	mRNA	XR_001042381.2	lncRNA
XM_015096939.1	BMF	mRNA	XR_001042381.2	lncRNA
XM_015096939.1	BMF	mRNA	XR_001042381.2	lncRNA
XM_015096939.1	BMF	mRNA	XR_001042381.2	lncRNA
XM_015096939.1	BMF	mRNA	XR_001042381.2	lncRNA
XM_015096939.1	BMF	mRNA	XR_001042381.2	lncRNA
XM_015096939.1	BMF	mRNA	XR_001042381.2	lncRNA
XM_015096939.1	BMF	mRNA	XR_001042381.2	lncRNA
XM_015096132.1	CSNK1A1	mRNA	XR_001042381.2	lncRNA
XM_015096132.1	CSNK1A1	mRNA	XR_001042381.2	lncRNA
XM_015096132.1	CSNK1A1	mRNA	XR_001042381.2	lncRNA
XM_015096132.1	CSNK1A1	mRNA	XR_001042381.2	lncRNA
XM_015096132.1	CSNK1A1	mRNA	XR_001042381.2	lncRNA
XM_015096132.1	CSNK1A1	mRNA	XR_001042381.2	lncRNA
XM_015096132.1	CSNK1A1	mRNA	XR_001042381.2	lncRNA
XM_015096132.1	CSNK1A1	mRNA	XR_001042381.2	lncRNA
XM_015096132.1	CSNK1A1	mRNA	XR_001042381.2	lncRNA
XM_015096132.1	CSNK1A1	mRNA	XR_001042381.2	lncRNA
XM_015097611.1	VPS13B	mRNA	XR_001435726.1	lncRNA
XM_015097611.1	VPS13B	mRNA	XR_001435726.1	lncRNA
XM_015097611.1	VPS13B	mRNA	XR_001435726.1	lncRNA
XM_015097611.1	VPS13B	mRNA	XR_001435726.1	lncRNA
XM_015097611.1	VPS13B	mRNA	XR_001435726.1	lncRNA
XM_015097611.1	VPS13B	mRNA	XR_001435726.1	lncRNA
XM_015097611.1	VPS13B	mRNA	XR_001435726.1	lncRNA
XM_015097611.1	VPS13B	mRNA	XR_001435726.1	lncRNA
XM_015097611.1	VPS13B	mRNA	XR_001435726.1	lncRNA

XM_015097611.1	VPS13B	mRNA	XR_001435726.1	lncRNA
XM_012101112.2	ABT1	mRNA	XR_001435726.1	lncRNA
XM_012101112.2	ABT1	mRNA	XR_001435726.1	lncRNA
XM_012101112.2	ABT1	mRNA	XR_001435726.1	lncRNA
XM_012101112.2	ABT1	mRNA	XR_001435726.1	lncRNA
XM_012101112.2	ABT1	mRNA	XR_001435726.1	lncRNA
XM_012101112.2	ABT1	mRNA	XR_001435726.1	lncRNA
XM_012101112.2	ABT1	mRNA	XR_001435726.1	lncRNA
XM_012101112.2	ABT1	mRNA	XR_001435726.1	lncRNA
XM_012101112.2	ABT1	mRNA	XR_001435726.1	lncRNA
XM_012101112.2	ABT1	mRNA	XR_001435726.1	lncRNA
XM_015093088.1	CDCA2	mRNA	XR_001042381.2	lncRNA
XM_015093088.1	CDCA2	mRNA	XR_001042381.2	lncRNA
XM_015093088.1	CDCA2	mRNA	XR_001042381.2	lncRNA
XM_015093088.1	CDCA2	mRNA	XR_001042381.2	lncRNA
XM_015093088.1	CDCA2	mRNA	XR_001042381.2	lncRNA
XM_015093088.1	CDCA2	mRNA	XR_001042381.2	lncRNA
XM_015093088.1	CDCA2	mRNA	XR_001042381.2	lncRNA
XM_015093088.1	CDCA2	mRNA	XR_001042381.2	lncRNA
XM_015093088.1	CDCA2	mRNA	XR_001042381.2	lncRNA
XM_015093088.1	CDCA2	mRNA	XR_001042381.2	lncRNA
XM_015101011.1	TEAD1	mRNA	TCONS_00071561	lncRNA
XM_015101011.1	TEAD1	mRNA	TCONS_00071561	lncRNA
XM_015101011.1	TEAD1	mRNA	TCONS_00071561	lncRNA
XM_015101011.1	TEAD1	mRNA	TCONS_00071561	lncRNA
XM_015101011.1	TEAD1	mRNA	TCONS_00071561	lncRNA
XM_015101011.1	TEAD1	mRNA	TCONS_00071561	lncRNA
XM_015101011.1	TEAD1	mRNA	TCONS_00071561	lncRNA
XM_015101011.1	TEAD1	mRNA	TCONS_00071561	lncRNA
XM_015101011.1	TEAD1	mRNA	TCONS_00071561	lncRNA
XM_015100441.1	CHD9	mRNA	TCONS_00071561	lncRNA
XM_015100441.1	CHD9	mRNA	TCONS_00071561	lncRNA
XM_015100441.1	CHD9	mRNA	TCONS_00071561	lncRNA
XM_015100441.1	CHD9	mRNA	TCONS_00071561	lncRNA
XM_015100441.1	CHD9	mRNA	TCONS_00071561	lncRNA
XM_015100441.1	CHD9	mRNA	TCONS_00071561	lncRNA
XM_015100441.1	CHD9	mRNA	TCONS_00071561	lncRNA
XM_015100441.1	CHD9	mRNA	TCONS_00071561	lncRNA
XM_015100441.1	CHD9	mRNA	TCONS_00071561	lncRNA
XM_012188288.2	ARL5B	mRNA	XR_001434081.1	lncRNA
XM_012188288.2	ARL5B	mRNA	XR_001434081.1	lncRNA
XM_012188288.2	ARL5B	mRNA	XR_001434081.1	lncRNA
XM_012188288.2	ARL5B	mRNA	XR_001434081.1	lncRNA
XM_012188288.2	ARL5B	mRNA	XR_001434081.1	lncRNA
XM_012188288.2	ARL5B	mRNA	XR_001434081.1	lncRNA
XM_012188288.2	ARL5B	mRNA	XR_001434081.1	lncRNA
XM_012188288.2	ARL5B	mRNA	XR_001434081.1	lncRNA
XM_012188288.2	ARL5B	mRNA	XR_001434081.1	lncRNA
XM_015095780.1	DNAJB1	mRNA	XR_001436609.1	lncRNA
XM_015095780.1	DNAJB1	mRNA	XR_001436609.1	lncRNA
XM_015095780.1	DNAJB1	mRNA	XR_001436609.1	lncRNA
XM_015095780.1	DNAJB1	mRNA	XR_001436609.1	lncRNA
XM_015095780.1	DNAJB1	mRNA	XR_001436609.1	lncRNA
XM_015095780.1	DNAJB1	mRNA	XR_001436609.1	lncRNA

XM_015095780.1	DNAJB1	mRNA	XR_001436609.1	lncRNA
XM_015095780.1	DNAJB1	mRNA	XR_001436609.1	lncRNA
XM_015095780.1	DNAJB1	mRNA	XR_001436609.1	lncRNA
XM_015095780.1	DNAJB1	mRNA	XR_001436609.1	lncRNA
XM_012189186.2	RALGAPB	mRNA	XR_001021939.1	lncRNA
XM_012189186.2	RALGAPB	mRNA	XR_001021939.1	lncRNA
XM_012189186.2	RALGAPB	mRNA	XR_001021939.1	lncRNA
XM_012189186.2	RALGAPB	mRNA	XR_001021939.1	lncRNA
XM_012189186.2	RALGAPB	mRNA	XR_001021939.1	lncRNA
XM_012189186.2	RALGAPB	mRNA	XR_001021939.1	lncRNA
XM_012189186.2	RALGAPB	mRNA	XR_001021939.1	lncRNA
XM_012189186.2	RALGAPB	mRNA	XR_001021939.1	lncRNA
XM_012189186.2	RALGAPB	mRNA	XR_001021939.1	lncRNA
XM_012189186.2	RALGAPB	mRNA	XR_001021939.1	lncRNA
XM_012175265.1	PLEKHA5	mRNA	XR_001021939.1	lncRNA
XM_012175265.1	PLEKHA5	mRNA	XR_001021939.1	lncRNA
XM_012175265.1	PLEKHA5	mRNA	XR_001021939.1	lncRNA
XM_012175265.1	PLEKHA5	mRNA	XR_001021939.1	lncRNA
XM_012175265.1	PLEKHA5	mRNA	XR_001021939.1	lncRNA
XM_012175265.1	PLEKHA5	mRNA	XR_001021939.1	lncRNA
XM_012175265.1	PLEKHA5	mRNA	XR_001021939.1	lncRNA
XM_012175265.1	PLEKHA5	mRNA	XR_001021939.1	lncRNA
XM_012175265.1	PLEKHA5	mRNA	XR_001021939.1	lncRNA
XM_012101176.2	LRRC16A	mRNA	XR_001021939.1	lncRNA
XM_012101176.2	LRRC16A	mRNA	XR_001021939.1	lncRNA
XM_012101176.2	LRRC16A	mRNA	XR_001021939.1	lncRNA
XM_012101176.2	LRRC16A	mRNA	XR_001021939.1	lncRNA
XM_012101176.2	LRRC16A	mRNA	XR_001021939.1	lncRNA
XM_012101176.2	LRRC16A	mRNA	XR_001021939.1	lncRNA
XM_012101176.2	LRRC16A	mRNA	XR_001021939.1	lncRNA
XM_012101176.2	LRRC16A	mRNA	XR_001021939.1	lncRNA
XM_012101176.2	LRRC16A	mRNA	XR_001021939.1	lncRNA
XM_012149251.2	S100PBP	mRNA	XR_001021939.1	lncRNA
XM_012149251.2	S100PBP	mRNA	XR_001021939.1	lncRNA
XM_012149251.2	S100PBP	mRNA	XR_001021939.1	lncRNA
XM_012149251.2	S100PBP	mRNA	XR_001021939.1	lncRNA
XM_012149251.2	S100PBP	mRNA	XR_001021939.1	lncRNA
XM_012149251.2	S100PBP	mRNA	XR_001021939.1	lncRNA
XM_012149251.2	S100PBP	mRNA	XR_001021939.1	lncRNA
XM_012149251.2	S100PBP	mRNA	XR_001021939.1	lncRNA
XM_012149251.2	S100PBP	mRNA	XR_001021939.1	lncRNA
XM_015096769.1	APC	mRNA	XR_001435726.1	lncRNA
XM_015096769.1	APC	mRNA	XR_001435726.1	lncRNA
XM_015096769.1	APC	mRNA	XR_001435726.1	lncRNA
XM_015096769.1	APC	mRNA	XR_001435726.1	lncRNA
XM_015096769.1	APC	mRNA	XR_001435726.1	lncRNA
XM_015096769.1	APC	mRNA	XR_001435726.1	lncRNA
XM_015096769.1	APC	mRNA	XR_001435726.1	lncRNA
XM_015096769.1	APC	mRNA	XR_001435726.1	lncRNA
XM_015096769.1	APC	mRNA	XR_001435726.1	lncRNA
XM_015096769.1	APC	mRNA	XR_001435726.1	lncRNA
XM_012143382.2	PARD3B	mRNA	XR_001041211.2	lncRNA
XM_012143382.2	PARD3B	mRNA	XR_001041211.2	lncRNA
XM_012143382.2	PARD3B	mRNA	XR_001041211.2	lncRNA

XM_012143382.2	PARD3B	mRNA	XR_001041211.2	lncRNA
XM_012143382.2	PARD3B	mRNA	XR_001041211.2	lncRNA
XM_012143382.2	PARD3B	mRNA	XR_001041211.2	lncRNA
XM_012143382.2	PARD3B	mRNA	XR_001041211.2	lncRNA
XM_012143382.2	PARD3B	mRNA	XR_001041211.2	lncRNA
XM_012143382.2	PARD3B	mRNA	XR_001041211.2	lncRNA
XM_004006510.3	SLC16A7	mRNA	XR_001041209.2	lncRNA
XM_004006510.3	SLC16A7	mRNA	XR_001041209.2	lncRNA
XM_004006510.3	SLC16A7	mRNA	XR_001041209.2	lncRNA
XM_004006510.3	SLC16A7	mRNA	XR_001041209.2	lncRNA
XM_004006510.3	SLC16A7	mRNA	XR_001041209.2	lncRNA
XM_004006510.3	SLC16A7	mRNA	XR_001041209.2	lncRNA
XM_004006510.3	SLC16A7	mRNA	XR_001041209.2	lncRNA
XM_004006510.3	SLC16A7	mRNA	XR_001041209.2	lncRNA
XM_004006510.3	SLC16A7	mRNA	XR_001041209.2	lncRNA
XM_012106629.2	WNK3	mRNA	XR_001041211.2	lncRNA
XM_012106629.2	WNK3	mRNA	XR_001041211.2	lncRNA
XM_012106629.2	WNK3	mRNA	XR_001041211.2	lncRNA
XM_012106629.2	WNK3	mRNA	XR_001041211.2	lncRNA
XM_012106629.2	WNK3	mRNA	XR_001041211.2	lncRNA
XM_012106629.2	WNK3	mRNA	XR_001041211.2	lncRNA
XM_012106629.2	WNK3	mRNA	XR_001041211.2	lncRNA
XM_012106629.2	WNK3	mRNA	XR_001041211.2	lncRNA
XM_012106629.2	WNK3	mRNA	XR_001041211.2	lncRNA
XM_015096814.1	DENND4A	mRNA	TCONS_00029399	lncRNA
XM_015096814.1	DENND4A	mRNA	TCONS_00029399	lncRNA
XM_015096814.1	DENND4A	mRNA	TCONS_00029399	lncRNA
XM_015096814.1	DENND4A	mRNA	TCONS_00029399	lncRNA
XM_015096814.1	DENND4A	mRNA	TCONS_00029399	lncRNA
XM_015096814.1	DENND4A	mRNA	TCONS_00029399	lncRNA
XM_015096814.1	DENND4A	mRNA	TCONS_00029399	lncRNA
XM_015096814.1	DENND4A	mRNA	TCONS_00029399	lncRNA
XM_015096814.1	DENND4A	mRNA	TCONS_00029399	lncRNA
XM_012099994.2	VPRBP	mRNA	XR_001033284.2	lncRNA
XM_012099994.2	VPRBP	mRNA	XR_001033284.2	lncRNA
XM_012099994.2	VPRBP	mRNA	XR_001033284.2	lncRNA
XM_012099994.2	VPRBP	mRNA	XR_001033284.2	lncRNA
XM_012099994.2	VPRBP	mRNA	XR_001033284.2	lncRNA
XM_012099994.2	VPRBP	mRNA	XR_001033284.2	lncRNA
XM_012099994.2	VPRBP	mRNA	XR_001033284.2	lncRNA
XM_012099994.2	VPRBP	mRNA	XR_001033284.2	lncRNA
XM_012099994.2	VPRBP	mRNA	XR_001033284.2	lncRNA
XM_012182854.2	PTPRK	mRNA	XR_001033284.2	lncRNA
XM_012182854.2	PTPRK	mRNA	XR_001033284.2	lncRNA
XM_012182854.2	PTPRK	mRNA	XR_001033284.2	lncRNA
XM_012182854.2	PTPRK	mRNA	XR_001033284.2	lncRNA
XM_012182854.2	PTPRK	mRNA	XR_001033284.2	lncRNA
XM_012182854.2	PTPRK	mRNA	XR_001033284.2	lncRNA
XM_012182854.2	PTPRK	mRNA	XR_001033284.2	lncRNA
XM_012182854.2	PTPRK	mRNA	XR_001033284.2	lncRNA
XM_012182854.2	PTPRK	mRNA	XR_001033284.2	lncRNA
XM_012167258.2	CLIP4	mRNA	XR_001436099.1	lncRNA
XM_012167258.2	CLIP4	mRNA	XR_001436099.1	lncRNA
XM_012167258.2	CLIP4	mRNA	XR_001436099.1	lncRNA

XM_012167258.2	CLIP4	mRNA	XR_001436099.1	lncRNA
XM_012167258.2	CLIP4	mRNA	XR_001436099.1	lncRNA
XM_012167258.2	CLIP4	mRNA	XR_001436099.1	lncRNA
XM_012167258.2	CLIP4	mRNA	XR_001436099.1	lncRNA
XM_012167258.2	CLIP4	mRNA	XR_001436099.1	lncRNA
XM_012167258.2	CLIP4	mRNA	XR_001436099.1	lncRNA
XM_012177560.2	SLC26A2	mRNA	XR_001436609.1	lncRNA
XM_012177560.2	SLC26A2	mRNA	XR_001436609.1	lncRNA
XM_012177560.2	SLC26A2	mRNA	XR_001436609.1	lncRNA
XM_012177560.2	SLC26A2	mRNA	XR_001436609.1	lncRNA
XM_012177560.2	SLC26A2	mRNA	XR_001436609.1	lncRNA
XM_012177560.2	SLC26A2	mRNA	XR_001436609.1	lncRNA
XM_012177560.2	SLC26A2	mRNA	XR_001436609.1	lncRNA
XM_012177560.2	SLC26A2	mRNA	XR_001436609.1	lncRNA
XM_012177560.2	SLC26A2	mRNA	XR_001436609.1	lncRNA
XM_012105198.2	TET1	mRNA	XR_001435104.1	lncRNA
XM_012105198.2	TET1	mRNA	XR_001435104.1	lncRNA
XM_012105198.2	TET1	mRNA	XR_001435104.1	lncRNA
XM_012105198.2	TET1	mRNA	XR_001435104.1	lncRNA
XM_012105198.2	TET1	mRNA	XR_001435104.1	lncRNA
XM_012105198.2	TET1	mRNA	XR_001435104.1	lncRNA
XM_012105198.2	TET1	mRNA	XR_001435104.1	lncRNA
XM_012105198.2	TET1	mRNA	XR_001435104.1	lncRNA
XM_012105198.2	TET1	mRNA	XR_001435104.1	lncRNA
XM_004017788.3	ZFANL3	mRNA	XR_001435104.1	lncRNA
XM_004017788.3	ZFANL3	mRNA	XR_001435104.1	lncRNA
XM_004017788.3	ZFANL3	mRNA	XR_001435104.1	lncRNA
XM_004017788.3	ZFANL3	mRNA	XR_001435104.1	lncRNA
XM_004017788.3	ZFANL3	mRNA	XR_001435104.1	lncRNA
XM_004017788.3	ZFANL3	mRNA	XR_001435104.1	lncRNA
XM_004017788.3	ZFANL3	mRNA	XR_001435104.1	lncRNA
XM_004017788.3	ZFANL3	mRNA	XR_001435104.1	lncRNA
XM_004017788.3	ZFANL3	mRNA	XR_001435104.1	lncRNA
XM_012101176.2	LRRC16A	mRNA	XR_001435104.1	lncRNA
XM_012101176.2	LRRC16A	mRNA	XR_001435104.1	lncRNA
XM_012101176.2	LRRC16A	mRNA	XR_001435104.1	lncRNA
XM_012101176.2	LRRC16A	mRNA	XR_001435104.1	lncRNA
XM_012101176.2	LRRC16A	mRNA	XR_001435104.1	lncRNA
XM_012101176.2	LRRC16A	mRNA	XR_001435104.1	lncRNA
XM_012101176.2	LRRC16A	mRNA	XR_001435104.1	lncRNA
XM_012101176.2	LRRC16A	mRNA	XR_001435104.1	lncRNA
XM_012101176.2	LRRC16A	mRNA	XR_001435104.1	lncRNA
XM_004017788.3	ZFANL3	mRNA	XR_001436609.1	lncRNA
XM_004017788.3	ZFANL3	mRNA	XR_001436609.1	lncRNA
XM_004017788.3	ZFANL3	mRNA	XR_001436609.1	lncRNA
XM_004017788.3	ZFANL3	mRNA	XR_001436609.1	lncRNA
XM_004017788.3	ZFANL3	mRNA	XR_001436609.1	lncRNA
XM_004017788.3	ZFANL3	mRNA	XR_001436609.1	lncRNA
XM_004017788.3	ZFANL3	mRNA	XR_001436609.1	lncRNA
XM_004017788.3	ZFANL3	mRNA	XR_001436609.1	lncRNA
XM_004017788.3	ZFANL3	mRNA	XR_001436609.1	lncRNA
XM_015092207.1	PRKAB2	mRNA	XR_001435104.1	lncRNA
XM_015092207.1	PRKAB2	mRNA	XR_001435104.1	lncRNA
XM_015092207.1	PRKAB2	mRNA	XR_001435104.1	lncRNA

XM_015092207.1	PRKAB2	mRNA	XR_001435104.1	lncRNA
XM_015092207.1	PRKAB2	mRNA	XR_001435104.1	lncRNA
XM_015092207.1	PRKAB2	mRNA	XR_001435104.1	lncRNA
XM_015092207.1	PRKAB2	mRNA	XR_001435104.1	lncRNA
XM_015092207.1	PRKAB2	mRNA	XR_001435104.1	lncRNA
XM_015092207.1	PRKAB2	mRNA	XR_001435104.1	lncRNA
XM_012149251.2	S100PBP	mRNA	XR_001435104.1	lncRNA
XM_012149251.2	S100PBP	mRNA	XR_001435104.1	lncRNA
XM_012149251.2	S100PBP	mRNA	XR_001435104.1	lncRNA
XM_012149251.2	S100PBP	mRNA	XR_001435104.1	lncRNA
XM_012149251.2	S100PBP	mRNA	XR_001435104.1	lncRNA
XM_012149251.2	S100PBP	mRNA	XR_001435104.1	lncRNA
XM_012149251.2	S100PBP	mRNA	XR_001435104.1	lncRNA
XM_012149251.2	S100PBP	mRNA	XR_001435104.1	lncRNA
XM_012149251.2	S100PBP	mRNA	XR_001435104.1	lncRNA
XM_012114904.1	RNF20	mRNA	XR_001436609.1	lncRNA
XM_012114904.1	RNF20	mRNA	XR_001436609.1	lncRNA
XM_012114904.1	RNF20	mRNA	XR_001436609.1	lncRNA
XM_012114904.1	RNF20	mRNA	XR_001436609.1	lncRNA
XM_012114904.1	RNF20	mRNA	XR_001436609.1	lncRNA
XM_012114904.1	RNF20	mRNA	XR_001436609.1	lncRNA
XM_012114904.1	RNF20	mRNA	XR_001436609.1	lncRNA
XM_012114904.1	RNF20	mRNA	XR_001436609.1	lncRNA
XM_012114904.1	RNF20	mRNA	XR_001436609.1	lncRNA
XM_012175265.1	PLEKHA5	mRNA	XR_001435104.1	lncRNA
XM_012175265.1	PLEKHA5	mRNA	XR_001435104.1	lncRNA
XM_012175265.1	PLEKHA5	mRNA	XR_001435104.1	lncRNA
XM_012175265.1	PLEKHA5	mRNA	XR_001435104.1	lncRNA
XM_012175265.1	PLEKHA5	mRNA	XR_001435104.1	lncRNA
XM_012175265.1	PLEKHA5	mRNA	XR_001435104.1	lncRNA
XM_012175265.1	PLEKHA5	mRNA	XR_001435104.1	lncRNA
XM_012175265.1	PLEKHA5	mRNA	XR_001435104.1	lncRNA
XM_012175265.1	PLEKHA5	mRNA	XR_001435104.1	lncRNA
XM_012113353.2	SUSD1	mRNA	XR_001436609.1	lncRNA
XM_012113353.2	SUSD1	mRNA	XR_001436609.1	lncRNA
XM_012113353.2	SUSD1	mRNA	XR_001436609.1	lncRNA
XM_012113353.2	SUSD1	mRNA	XR_001436609.1	lncRNA
XM_012113353.2	SUSD1	mRNA	XR_001436609.1	lncRNA
XM_012113353.2	SUSD1	mRNA	XR_001436609.1	lncRNA
XM_012113353.2	SUSD1	mRNA	XR_001436609.1	lncRNA
XM_012113353.2	SUSD1	mRNA	XR_001436609.1	lncRNA
XM_012113353.2	SUSD1	mRNA	XR_001436609.1	lncRNA
XM_004012194.3	RBM26	mRNA	XR_001435104.1	lncRNA
XM_004012194.3	RBM26	mRNA	XR_001435104.1	lncRNA
XM_004012194.3	RBM26	mRNA	XR_001435104.1	lncRNA
XM_004012194.3	RBM26	mRNA	XR_001435104.1	lncRNA
XM_004012194.3	RBM26	mRNA	XR_001435104.1	lncRNA
XM_004012194.3	RBM26	mRNA	XR_001435104.1	lncRNA
XM_004012194.3	RBM26	mRNA	XR_001435104.1	lncRNA
XM_004012194.3	RBM26	mRNA	XR_001435104.1	lncRNA
XM_004012194.3	RBM26	mRNA	XR_001435104.1	lncRNA
XM_015092393.1	NRIP1	mRNA	XR_001436609.1	lncRNA
XM_015092393.1	NRIP1	mRNA	XR_001436609.1	lncRNA
XM_015092393.1	NRIP1	mRNA	XR_001436609.1	lncRNA

XM_015092393.1	NRIP1	mRNA	XR_001436609.1	lncRNA
XM_015092393.1	NRIP1	mRNA	XR_001436609.1	lncRNA
XM_015092393.1	NRIP1	mRNA	XR_001436609.1	lncRNA
XM_015092393.1	NRIP1	mRNA	XR_001436609.1	lncRNA
XM_015092393.1	NRIP1	mRNA	XR_001436609.1	lncRNA
XM_015092393.1	NRIP1	mRNA	XR_001436609.1	lncRNA
XM_012177560.2	SLC26A2	mRNA	XR_001436531.1	lncRNA
XM_012177560.2	SLC26A2	mRNA	XR_001436531.1	lncRNA
XM_012177560.2	SLC26A2	mRNA	XR_001436531.1	lncRNA
XM_012177560.2	SLC26A2	mRNA	XR_001436531.1	lncRNA
XM_012177560.2	SLC26A2	mRNA	XR_001436531.1	lncRNA
XM_012177560.2	SLC26A2	mRNA	XR_001436531.1	lncRNA
XM_012177560.2	SLC26A2	mRNA	XR_001436531.1	lncRNA
XM_012177560.2	SLC26A2	mRNA	XR_001436531.1	lncRNA
XM_012177560.2	SLC26A2	mRNA	XR_001436531.1	lncRNA
XM_012142889.2	ICA1L	mRNA	XR_001436609.1	lncRNA
XM_012142889.2	ICA1L	mRNA	XR_001436609.1	lncRNA
XM_012142889.2	ICA1L	mRNA	XR_001436609.1	lncRNA
XM_012142889.2	ICA1L	mRNA	XR_001436609.1	lncRNA
XM_012142889.2	ICA1L	mRNA	XR_001436609.1	lncRNA
XM_012142889.2	ICA1L	mRNA	XR_001436609.1	lncRNA
XM_012142889.2	ICA1L	mRNA	XR_001436609.1	lncRNA
XM_012142889.2	ICA1L	mRNA	XR_001436609.1	lncRNA
XM_012142889.2	ICA1L	mRNA	XR_001436609.1	lncRNA
XM_012149251.2	S100PBP	mRNA	XR_001436609.1	lncRNA
XM_012149251.2	S100PBP	mRNA	XR_001436609.1	lncRNA
XM_012149251.2	S100PBP	mRNA	XR_001436609.1	lncRNA
XM_012149251.2	S100PBP	mRNA	XR_001436609.1	lncRNA
XM_012149251.2	S100PBP	mRNA	XR_001436609.1	lncRNA
XM_012149251.2	S100PBP	mRNA	XR_001436609.1	lncRNA
XM_012149251.2	S100PBP	mRNA	XR_001436609.1	lncRNA
XM_012149251.2	S100PBP	mRNA	XR_001436609.1	lncRNA
XM_012149251.2	S100PBP	mRNA	XR_001436609.1	lncRNA
XM_012095713.2	RRAS2	mRNA	XR_001436609.1	lncRNA
XM_012095713.2	RRAS2	mRNA	XR_001436609.1	lncRNA
XM_012095713.2	RRAS2	mRNA	XR_001436609.1	lncRNA
XM_012095713.2	RRAS2	mRNA	XR_001436609.1	lncRNA
XM_012095713.2	RRAS2	mRNA	XR_001436609.1	lncRNA
XM_012095713.2	RRAS2	mRNA	XR_001436609.1	lncRNA
XM_012095713.2	RRAS2	mRNA	XR_001436609.1	lncRNA
XM_012095713.2	RRAS2	mRNA	XR_001436609.1	lncRNA
XM_012095713.2	RRAS2	mRNA	XR_001436609.1	lncRNA
XM_004017884.2	AKAP6	mRNA	XR_001041202.1	lncRNA
XM_004017884.2	AKAP6	mRNA	XR_001041202.1	lncRNA
XM_004017884.2	AKAP6	mRNA	XR_001041202.1	lncRNA
XM_004017884.2	AKAP6	mRNA	XR_001041202.1	lncRNA
XM_004017884.2	AKAP6	mRNA	XR_001041202.1	lncRNA
XM_004017884.2	AKAP6	mRNA	XR_001041202.1	lncRNA
XM_004017884.2	AKAP6	mRNA	XR_001041202.1	lncRNA
XM_004017884.2	AKAP6	mRNA	XR_001041202.1	lncRNA
XM_004017884.2	AKAP6	mRNA	XR_001041202.1	lncRNA
XM_015096814.1	DENND4A	mRNA	XR_001434081.1	lncRNA
XM_015096814.1	DENND4A	mRNA	XR_001434081.1	lncRNA
XM_015096814.1	DENND4A	mRNA	XR_001434081.1	lncRNA

XM_015096814.1	DENND4A	mRNA	XR_001434081.1	lncRNA
XM_015096814.1	DENND4A	mRNA	XR_001434081.1	lncRNA
XM_015096814.1	DENND4A	mRNA	XR_001434081.1	lncRNA
XM_015096814.1	DENND4A	mRNA	XR_001434081.1	lncRNA
XM_015096814.1	DENND4A	mRNA	XR_001434081.1	lncRNA
XM_015096814.1	DENND4A	mRNA	XR_001434081.1	lncRNA
XM_012188288.2	ARL5B	mRNA	XR_001435723.1	lncRNA
XM_012188288.2	ARL5B	mRNA	XR_001435723.1	lncRNA
XM_012188288.2	ARL5B	mRNA	XR_001435723.1	lncRNA
XM_012188288.2	ARL5B	mRNA	XR_001435723.1	lncRNA
XM_012188288.2	ARL5B	mRNA	XR_001435723.1	lncRNA
XM_012188288.2	ARL5B	mRNA	XR_001435723.1	lncRNA
XM_012188288.2	ARL5B	mRNA	XR_001435723.1	lncRNA
XM_012188288.2	ARL5B	mRNA	XR_001435723.1	lncRNA
XM_012188288.2	ARL5B	mRNA	XR_001435723.1	lncRNA
XM_012188288.2	ARL5B	mRNA	XR_001435726.1	lncRNA
XM_012188288.2	ARL5B	mRNA	XR_001435726.1	lncRNA
XM_012188288.2	ARL5B	mRNA	XR_001435726.1	lncRNA
XM_012188288.2	ARL5B	mRNA	XR_001435726.1	lncRNA
XM_012188288.2	ARL5B	mRNA	XR_001435726.1	lncRNA
XM_012188288.2	ARL5B	mRNA	XR_001435726.1	lncRNA
XM_012188288.2	ARL5B	mRNA	XR_001435726.1	lncRNA
XM_012188288.2	ARL5B	mRNA	XR_001435726.1	lncRNA
XM_012188288.2	ARL5B	mRNA	XR_001435726.1	lncRNA
XM_004001781.3	ZSCAN20	mRNA	XR_001435726.1	lncRNA
XM_004001781.3	ZSCAN20	mRNA	XR_001435726.1	lncRNA
XM_004001781.3	ZSCAN20	mRNA	XR_001435726.1	lncRNA
XM_004001781.3	ZSCAN20	mRNA	XR_001435726.1	lncRNA
XM_004001781.3	ZSCAN20	mRNA	XR_001435726.1	lncRNA
XM_004001781.3	ZSCAN20	mRNA	XR_001435726.1	lncRNA
XM_004001781.3	ZSCAN20	mRNA	XR_001435726.1	lncRNA
XM_004001781.3	ZSCAN20	mRNA	XR_001435726.1	lncRNA
XM_004001781.3	ZSCAN20	mRNA	XR_001435726.1	lncRNA
XM_012095713.2	RRAS2	mRNA	XR_001042381.2	lncRNA
XM_012095713.2	RRAS2	mRNA	XR_001042381.2	lncRNA
XM_012095713.2	RRAS2	mRNA	XR_001042381.2	lncRNA
XM_012095713.2	RRAS2	mRNA	XR_001042381.2	lncRNA
XM_012095713.2	RRAS2	mRNA	XR_001042381.2	lncRNA
XM_012095713.2	RRAS2	mRNA	XR_001042381.2	lncRNA
XM_012095713.2	RRAS2	mRNA	XR_001042381.2	lncRNA
XM_012095713.2	RRAS2	mRNA	XR_001042381.2	lncRNA
XM_012095713.2	RRAS2	mRNA	XR_001042381.2	lncRNA
XM_012142889.2	ICA1L	mRNA	XR_001435726.1	lncRNA
XM_012142889.2	ICA1L	mRNA	XR_001435726.1	lncRNA
XM_012142889.2	ICA1L	mRNA	XR_001435726.1	lncRNA
XM_012142889.2	ICA1L	mRNA	XR_001435726.1	lncRNA
XM_012142889.2	ICA1L	mRNA	XR_001435726.1	lncRNA
XM_012142889.2	ICA1L	mRNA	XR_001435726.1	lncRNA
XM_012142889.2	ICA1L	mRNA	XR_001435726.1	lncRNA
XM_012142889.2	ICA1L	mRNA	XR_001435726.1	lncRNA
XM_012142889.2	ICA1L	mRNA	XR_001435726.1	lncRNA
XM_012146156.1	USP37	mRNA	XR_001435723.1	lncRNA
XM_012146156.1	USP37	mRNA	XR_001435723.1	lncRNA
XM_012146156.1	USP37	mRNA	XR_001435723.1	lncRNA

XM_012146156.1	USP37	mRNA	XR_001435723.1	lncRNA
XM_012146156.1	USP37	mRNA	XR_001435723.1	lncRNA
XM_012146156.1	USP37	mRNA	XR_001435723.1	lncRNA
XM_012146156.1	USP37	mRNA	XR_001435723.1	lncRNA
XM_012146156.1	USP37	mRNA	XR_001435723.1	lncRNA
XM_012146156.1	USP37	mRNA	XR_001435723.1	lncRNA
XM_012152004.1	NIPAL3	mRNA	XR_001042381.2	lncRNA
XM_012152004.1	NIPAL3	mRNA	XR_001042381.2	lncRNA
XM_012152004.1	NIPAL3	mRNA	XR_001042381.2	lncRNA
XM_012152004.1	NIPAL3	mRNA	XR_001042381.2	lncRNA
XM_012152004.1	NIPAL3	mRNA	XR_001042381.2	lncRNA
XM_012152004.1	NIPAL3	mRNA	XR_001042381.2	lncRNA
XM_012152004.1	NIPAL3	mRNA	XR_001042381.2	lncRNA
XM_012152004.1	NIPAL3	mRNA	XR_001042381.2	lncRNA
XM_012152004.1	NIPAL3	mRNA	XR_001042381.2	lncRNA
XM_012178641.2	CTTNBP2NL	mRNA	XR_001435723.1	lncRNA
XM_012178641.2	CTTNBP2NL	mRNA	XR_001435723.1	lncRNA
XM_012178641.2	CTTNBP2NL	mRNA	XR_001435723.1	lncRNA
XM_012178641.2	CTTNBP2NL	mRNA	XR_001435723.1	lncRNA
XM_012178641.2	CTTNBP2NL	mRNA	XR_001435723.1	lncRNA
XM_012178641.2	CTTNBP2NL	mRNA	XR_001435723.1	lncRNA
XM_012178641.2	CTTNBP2NL	mRNA	XR_001435723.1	lncRNA
XM_012178641.2	CTTNBP2NL	mRNA	XR_001435723.1	lncRNA
XM_012178641.2	CTTNBP2NL	mRNA	XR_001435723.1	lncRNA
XM_012178641.2	CTTNBP2NL	mRNA	XR_001435726.1	lncRNA
XM_012178641.2	CTTNBP2NL	mRNA	XR_001435726.1	lncRNA
XM_012178641.2	CTTNBP2NL	mRNA	XR_001435726.1	lncRNA
XM_012178641.2	CTTNBP2NL	mRNA	XR_001435726.1	lncRNA
XM_012178641.2	CTTNBP2NL	mRNA	XR_001435726.1	lncRNA
XM_012178641.2	CTTNBP2NL	mRNA	XR_001435726.1	lncRNA
XM_012178641.2	CTTNBP2NL	mRNA	XR_001435726.1	lncRNA
XM_012178641.2	CTTNBP2NL	mRNA	XR_001435726.1	lncRNA
XM_012178641.2	CTTNBP2NL	mRNA	XR_001435726.1	lncRNA
XM_012102834.2	PPRC1	mRNA	XR_001435723.1	lncRNA
XM_012102834.2	PPRC1	mRNA	XR_001435723.1	lncRNA
XM_012102834.2	PPRC1	mRNA	XR_001435723.1	lncRNA
XM_012102834.2	PPRC1	mRNA	XR_001435723.1	lncRNA
XM_012102834.2	PPRC1	mRNA	XR_001435723.1	lncRNA
XM_012102834.2	PPRC1	mRNA	XR_001435723.1	lncRNA
XM_012102834.2	PPRC1	mRNA	XR_001435723.1	lncRNA
XM_012102834.2	PPRC1	mRNA	XR_001435723.1	lncRNA
XM_012102834.2	PPRC1	mRNA	XR_001435723.1	lncRNA
XM_004017788.3	ZFANL3	mRNA	XR_001435726.1	lncRNA
XM_004017788.3	ZFANL3	mRNA	XR_001435726.1	lncRNA
XM_004017788.3	ZFANL3	mRNA	XR_001435726.1	lncRNA
XM_004017788.3	ZFANL3	mRNA	XR_001435726.1	lncRNA
XM_004017788.3	ZFANL3	mRNA	XR_001435726.1	lncRNA
XM_004017788.3	ZFANL3	mRNA	XR_001435726.1	lncRNA
XM_004017788.3	ZFANL3	mRNA	XR_001435726.1	lncRNA
XM_004017788.3	ZFANL3	mRNA	XR_001435726.1	lncRNA
XM_004017788.3	ZFANL3	mRNA	XR_001435726.1	lncRNA
XM_012102834.2	PPRC1	mRNA	XR_001435726.1	lncRNA
XM_012102834.2	PPRC1	mRNA	XR_001435726.1	lncRNA
XM_012102834.2	PPRC1	mRNA	XR_001435726.1	lncRNA

XM_012102834.2	PPRC1	mRNA	XR_001435726.1	lncRNA
XM_012102834.2	PPRC1	mRNA	XR_001435726.1	lncRNA
XM_012102834.2	PPRC1	mRNA	XR_001435726.1	lncRNA
XM_012102834.2	PPRC1	mRNA	XR_001435726.1	lncRNA
XM_012102834.2	PPRC1	mRNA	XR_001435726.1	lncRNA
XM_012102834.2	PPRC1	mRNA	XR_001435726.1	lncRNA
XM_015093350.1	GPR155	mRNA	XR_001435726.1	lncRNA
XM_015093350.1	GPR155	mRNA	XR_001435726.1	lncRNA
XM_015093350.1	GPR155	mRNA	XR_001435726.1	lncRNA
XM_015093350.1	GPR155	mRNA	XR_001435726.1	lncRNA
XM_015093350.1	GPR155	mRNA	XR_001435726.1	lncRNA
XM_015093350.1	GPR155	mRNA	XR_001435726.1	lncRNA
XM_015093350.1	GPR155	mRNA	XR_001435726.1	lncRNA
XM_015093350.1	GPR155	mRNA	XR_001435726.1	lncRNA
XM_015093350.1	GPR155	mRNA	XR_001435726.1	lncRNA
XM_012095713.2	RRAS2	mRNA	XR_001435726.1	lncRNA
XM_012095713.2	RRAS2	mRNA	XR_001435726.1	lncRNA
XM_012095713.2	RRAS2	mRNA	XR_001435726.1	lncRNA
XM_012095713.2	RRAS2	mRNA	XR_001435726.1	lncRNA
XM_012095713.2	RRAS2	mRNA	XR_001435726.1	lncRNA
XM_012095713.2	RRAS2	mRNA	XR_001435726.1	lncRNA
XM_012095713.2	RRAS2	mRNA	XR_001435726.1	lncRNA
XM_012095713.2	RRAS2	mRNA	XR_001435726.1	lncRNA
XM_012095713.2	RRAS2	mRNA	XR_001435726.1	lncRNA
XM_012167258.2	CLIP4	mRNA	XR_001041209.2	lncRNA
XM_012167258.2	CLIP4	mRNA	XR_001041209.2	lncRNA
XM_012167258.2	CLIP4	mRNA	XR_001041209.2	lncRNA
XM_012167258.2	CLIP4	mRNA	XR_001041209.2	lncRNA
XM_012167258.2	CLIP4	mRNA	XR_001041209.2	lncRNA
XM_012167258.2	CLIP4	mRNA	XR_001041209.2	lncRNA
XM_012167258.2	CLIP4	mRNA	XR_001041209.2	lncRNA
XM_012167258.2	CLIP4	mRNA	XR_001041209.2	lncRNA
XM_012167258.2	CLIP4	mRNA	XR_001041209.2	lncRNA
XM_015093088.1	CDCA2	mRNA	TCONS_00029399	lncRNA
XM_015093088.1	CDCA2	mRNA	TCONS_00029399	lncRNA
XM_015093088.1	CDCA2	mRNA	TCONS_00029399	lncRNA
XM_015093088.1	CDCA2	mRNA	TCONS_00029399	lncRNA
XM_015093088.1	CDCA2	mRNA	TCONS_00029399	lncRNA
XM_015093088.1	CDCA2	mRNA	TCONS_00029399	lncRNA
XM_015093088.1	CDCA2	mRNA	TCONS_00029399	lncRNA
XM_015093088.1	CDCA2	mRNA	TCONS_00029399	lncRNA
XM_015093088.1	CDCA2	mRNA	TCONS_00029399	lncRNA
XM_012095465.2	GOLGB1	mRNA	XR_001033284.2	lncRNA
XM_012095465.2	GOLGB1	mRNA	XR_001033284.2	lncRNA
XM_012095465.2	GOLGB1	mRNA	XR_001033284.2	lncRNA
XM_012095465.2	GOLGB1	mRNA	XR_001033284.2	lncRNA
XM_012095465.2	GOLGB1	mRNA	XR_001033284.2	lncRNA
XM_012095465.2	GOLGB1	mRNA	XR_001033284.2	lncRNA
XM_012095465.2	GOLGB1	mRNA	XR_001033284.2	lncRNA
XM_012095465.2	GOLGB1	mRNA	XR_001033284.2	lncRNA
XM_012095465.2	GOLGB1	mRNA	XR_001033284.2	lncRNA
XM_015091899.1	SCMH1	mRNA	XR_001021939.1	lncRNA
XM_015091899.1	SCMH1	mRNA	XR_001021939.1	lncRNA
XM_015091899.1	SCMH1	mRNA	XR_001021939.1	lncRNA

XM_015091899.1	SCMH1	mRNA	XR_001021939.1	lncRNA
XM_015091899.1	SCMH1	mRNA	XR_001021939.1	lncRNA
XM_015091899.1	SCMH1	mRNA	XR_001021939.1	lncRNA
XM_015091899.1	SCMH1	mRNA	XR_001021939.1	lncRNA
XM_015091899.1	SCMH1	mRNA	XR_001021939.1	lncRNA
XM_015091899.1	SCMH1	mRNA	XR_001021939.1	lncRNA
XM_012183298.1	FAM120B	mRNA	XR_001021939.1	lncRNA
XM_012183298.1	FAM120B	mRNA	XR_001021939.1	lncRNA
XM_012183298.1	FAM120B	mRNA	XR_001021939.1	lncRNA
XM_012183298.1	FAM120B	mRNA	XR_001021939.1	lncRNA
XM_012183298.1	FAM120B	mRNA	XR_001021939.1	lncRNA
XM_012183298.1	FAM120B	mRNA	XR_001021939.1	lncRNA
XM_012183298.1	FAM120B	mRNA	XR_001021939.1	lncRNA
XM_012183298.1	FAM120B	mRNA	XR_001021939.1	lncRNA
XM_012183298.1	FAM120B	mRNA	XR_001021939.1	lncRNA
XM_004013791.3	PERM1	mRNA	XR_001041202.1	lncRNA
XM_004013791.3	PERM1	mRNA	XR_001041202.1	lncRNA
XM_004013791.3	PERM1	mRNA	XR_001041202.1	lncRNA
XM_004013791.3	PERM1	mRNA	XR_001041202.1	lncRNA
XM_004013791.3	PERM1	mRNA	XR_001041202.1	lncRNA
XM_004013791.3	PERM1	mRNA	XR_001041202.1	lncRNA
XM_004013791.3	PERM1	mRNA	XR_001041202.1	lncRNA
XM_004013791.3	PERM1	mRNA	XR_001041202.1	lncRNA
XM_004013791.3	PERM1	mRNA	XR_001041202.1	lncRNA
XM_015101011.1	TEAD1	mRNA	XR_001033284.2	lncRNA
XM_015101011.1	TEAD1	mRNA	XR_001033284.2	lncRNA
XM_015101011.1	TEAD1	mRNA	XR_001033284.2	lncRNA
XM_015101011.1	TEAD1	mRNA	XR_001033284.2	lncRNA
XM_015101011.1	TEAD1	mRNA	XR_001033284.2	lncRNA
XM_015101011.1	TEAD1	mRNA	XR_001033284.2	lncRNA
XM_015101011.1	TEAD1	mRNA	XR_001033284.2	lncRNA
XM_015101011.1	TEAD1	mRNA	XR_001033284.2	lncRNA
XM_015101011.1	TEAD1	mRNA	XR_001033284.2	lncRNA
XM_004010549.3	VPS13C	mRNA	XR_001033284.2	lncRNA
XM_004010549.3	VPS13C	mRNA	XR_001033284.2	lncRNA
XM_004010549.3	VPS13C	mRNA	XR_001033284.2	lncRNA
XM_004010549.3	VPS13C	mRNA	XR_001033284.2	lncRNA
XM_004010549.3	VPS13C	mRNA	XR_001033284.2	lncRNA
XM_004010549.3	VPS13C	mRNA	XR_001033284.2	lncRNA
XM_004010549.3	VPS13C	mRNA	XR_001033284.2	lncRNA
XM_004010549.3	VPS13C	mRNA	XR_001033284.2	lncRNA
XM_004010549.3	VPS13C	mRNA	XR_001033284.2	lncRNA
XM_015097611.1	VPS13B	mRNA	XR_001434081.1	lncRNA
XM_015097611.1	VPS13B	mRNA	XR_001434081.1	lncRNA
XM_015097611.1	VPS13B	mRNA	XR_001434081.1	lncRNA
XM_015097611.1	VPS13B	mRNA	XR_001434081.1	lncRNA
XM_015097611.1	VPS13B	mRNA	XR_001434081.1	lncRNA
XM_015097611.1	VPS13B	mRNA	XR_001434081.1	lncRNA
XM_015097611.1	VPS13B	mRNA	XR_001434081.1	lncRNA
XM_015097611.1	VPS13B	mRNA	XR_001434081.1	lncRNA
XM_015097611.1	VPS13B	mRNA	XR_001434081.1	lncRNA
XM_012167258.2	CLIP4	mRNA	XR_001434081.1	lncRNA
XM_012167258.2	CLIP4	mRNA	XR_001434081.1	lncRNA
XM_012167258.2	CLIP4	mRNA	XR_001434081.1	lncRNA

XM_012167258.2	CLIP4	mRNA	XR_001434081.1	lncRNA
XM_012167258.2	CLIP4	mRNA	XR_001434081.1	lncRNA
XM_012167258.2	CLIP4	mRNA	XR_001434081.1	lncRNA
XM_012167258.2	CLIP4	mRNA	XR_001434081.1	lncRNA
XM_012167258.2	CLIP4	mRNA	XR_001434081.1	lncRNA
XM_012167258.2	CLIP4	mRNA	XR_001434081.1	lncRNA
XM_012144336.2	PIKFYVE	mRNA	XR_001041209.2	lncRNA
XM_012144336.2	PIKFYVE	mRNA	XR_001041209.2	lncRNA
XM_012144336.2	PIKFYVE	mRNA	XR_001041209.2	lncRNA
XM_012144336.2	PIKFYVE	mRNA	XR_001041209.2	lncRNA
XM_012144336.2	PIKFYVE	mRNA	XR_001041209.2	lncRNA
XM_012144336.2	PIKFYVE	mRNA	XR_001041209.2	lncRNA
XM_012144336.2	PIKFYVE	mRNA	XR_001041209.2	lncRNA
XM_012144336.2	PIKFYVE	mRNA	XR_001041209.2	lncRNA
XM_012144336.2	PIKFYVE	mRNA	XR_001041209.2	lncRNA
XM_004004717.2	GTDC1	mRNA	TCONS_00029399	lncRNA
XM_004004717.2	GTDC1	mRNA	TCONS_00029399	lncRNA
XM_004004717.2	GTDC1	mRNA	TCONS_00029399	lncRNA
XM_004004717.2	GTDC1	mRNA	TCONS_00029399	lncRNA
XM_004004717.2	GTDC1	mRNA	TCONS_00029399	lncRNA
XM_004004717.2	GTDC1	mRNA	TCONS_00029399	lncRNA
XM_004004717.2	GTDC1	mRNA	TCONS_00029399	lncRNA
XM_004004717.2	GTDC1	mRNA	TCONS_00029399	lncRNA
XM_004004717.2	GTDC1	mRNA	TCONS_00029399	lncRNA
XM_004004717.2	GTDC1	mRNA	XR_001041209.2	lncRNA
XM_004004717.2	GTDC1	mRNA	XR_001041209.2	lncRNA
XM_004004717.2	GTDC1	mRNA	XR_001041209.2	lncRNA
XM_004004717.2	GTDC1	mRNA	XR_001041209.2	lncRNA
XM_004004717.2	GTDC1	mRNA	XR_001041209.2	lncRNA
XM_004004717.2	GTDC1	mRNA	XR_001041209.2	lncRNA
XM_004004717.2	GTDC1	mRNA	XR_001041209.2	lncRNA
XM_004004717.2	GTDC1	mRNA	XR_001041209.2	lncRNA
XM_004004717.2	GTDC1	mRNA	XR_001041209.2	lncRNA
XM_012104178.2	CCP110	mRNA	XR_001041209.2	lncRNA
XM_012104178.2	CCP110	mRNA	XR_001041209.2	lncRNA
XM_012104178.2	CCP110	mRNA	XR_001041209.2	lncRNA
XM_012104178.2	CCP110	mRNA	XR_001041209.2	lncRNA
XM_012104178.2	CCP110	mRNA	XR_001041209.2	lncRNA
XM_012104178.2	CCP110	mRNA	XR_001041209.2	lncRNA
XM_012104178.2	CCP110	mRNA	XR_001041209.2	lncRNA
XM_012104178.2	CCP110	mRNA	XR_001041209.2	lncRNA
XM_012104178.2	CCP110	mRNA	XR_001041209.2	lncRNA
XM_012102669.1	ZNF518A	mRNA	XR_001436531.1	lncRNA
XM_012102669.1	ZNF518A	mRNA	XR_001436531.1	lncRNA
XM_012102669.1	ZNF518A	mRNA	XR_001436531.1	lncRNA
XM_012102669.1	ZNF518A	mRNA	XR_001436531.1	lncRNA
XM_012102669.1	ZNF518A	mRNA	XR_001436531.1	lncRNA
XM_012102669.1	ZNF518A	mRNA	XR_001436531.1	lncRNA
XM_012102669.1	ZNF518A	mRNA	XR_001436531.1	lncRNA
XM_012102669.1	ZNF518A	mRNA	XR_001436531.1	lncRNA
XM_012102669.1	ZNF518A	mRNA	XR_001436531.1	lncRNA
XM_012176498.2	RAPGEF5	mRNA	XR_001436531.1	lncRNA
XM_012176498.2	RAPGEF5	mRNA	XR_001436531.1	lncRNA
XM_012176498.2	RAPGEF5	mRNA	XR_001436531.1	lncRNA

XM_012176498.2	RAPGEF5	mRNA	XR_001436531.1	lncRNA
XM_012176498.2	RAPGEF5	mRNA	XR_001436531.1	lncRNA
XM_012176498.2	RAPGEF5	mRNA	XR_001436531.1	lncRNA
XM_012176498.2	RAPGEF5	mRNA	XR_001436531.1	lncRNA
XM_012176498.2	RAPGEF5	mRNA	XR_001436531.1	lncRNA
XM_012176498.2	RAPGEF5	mRNA	XR_001436531.1	lncRNA
XM_012182215.2	TDP1	mRNA	XR_001435104.1	lncRNA
XM_012182215.2	TDP1	mRNA	XR_001435104.1	lncRNA
XM_012182215.2	TDP1	mRNA	XR_001435104.1	lncRNA
XM_012182215.2	TDP1	mRNA	XR_001435104.1	lncRNA
XM_012182215.2	TDP1	mRNA	XR_001435104.1	lncRNA
XM_012182215.2	TDP1	mRNA	XR_001435104.1	lncRNA
XM_012182215.2	TDP1	mRNA	XR_001435104.1	lncRNA
XM_012182215.2	TDP1	mRNA	XR_001435104.1	lncRNA
XM_012182215.2	TDP1	mRNA	XR_001435104.1	lncRNA
XM_012099659.2	FOXP1	mRNA	XR_001436531.1	lncRNA
XM_012099659.2	FOXP1	mRNA	XR_001436531.1	lncRNA
XM_012099659.2	FOXP1	mRNA	XR_001436531.1	lncRNA
XM_012099659.2	FOXP1	mRNA	XR_001436531.1	lncRNA
XM_012099659.2	FOXP1	mRNA	XR_001436531.1	lncRNA
XM_012099659.2	FOXP1	mRNA	XR_001436531.1	lncRNA
XM_012099659.2	FOXP1	mRNA	XR_001436531.1	lncRNA
XM_012099659.2	FOXP1	mRNA	XR_001436531.1	lncRNA
XM_012099659.2	FOXP1	mRNA	XR_001436531.1	lncRNA
XM_012190696.2	CCDC155	mRNA	XR_001435104.1	lncRNA
XM_012190696.2	CCDC155	mRNA	XR_001435104.1	lncRNA
XM_012190696.2	CCDC155	mRNA	XR_001435104.1	lncRNA
XM_012190696.2	CCDC155	mRNA	XR_001435104.1	lncRNA
XM_012190696.2	CCDC155	mRNA	XR_001435104.1	lncRNA
XM_012190696.2	CCDC155	mRNA	XR_001435104.1	lncRNA
XM_012190696.2	CCDC155	mRNA	XR_001435104.1	lncRNA
XM_012190696.2	CCDC155	mRNA	XR_001435104.1	lncRNA
XM_012190696.2	CCDC155	mRNA	XR_001435104.1	lncRNA
XM_012188917.1	STX16	mRNA	XR_001435104.1	lncRNA
XM_012188917.1	STX16	mRNA	XR_001435104.1	lncRNA
XM_012188917.1	STX16	mRNA	XR_001435104.1	lncRNA
XM_012188917.1	STX16	mRNA	XR_001435104.1	lncRNA
XM_012188917.1	STX16	mRNA	XR_001435104.1	lncRNA
XM_012188917.1	STX16	mRNA	XR_001435104.1	lncRNA
XM_012188917.1	STX16	mRNA	XR_001435104.1	lncRNA
XM_012188917.1	STX16	mRNA	XR_001435104.1	lncRNA
XM_012188917.1	STX16	mRNA	XR_001435104.1	lncRNA
XM_012182215.2	TDP1	mRNA	XR_001436609.1	lncRNA
XM_012182215.2	TDP1	mRNA	XR_001436609.1	lncRNA
XM_012182215.2	TDP1	mRNA	XR_001436609.1	lncRNA
XM_012182215.2	TDP1	mRNA	XR_001436609.1	lncRNA
XM_012182215.2	TDP1	mRNA	XR_001436609.1	lncRNA
XM_012182215.2	TDP1	mRNA	XR_001436609.1	lncRNA
XM_012182215.2	TDP1	mRNA	XR_001436609.1	lncRNA
XM_012182215.2	TDP1	mRNA	XR_001436609.1	lncRNA
XM_012182215.2	TDP1	mRNA	XR_001436609.1	lncRNA
XM_012188917.1	STX16	mRNA	XR_001436609.1	lncRNA
XM_012188917.1	STX16	mRNA	XR_001436609.1	lncRNA
XM_012188917.1	STX16	mRNA	XR_001436609.1	lncRNA

XM_012188917.1	STX16	mRNA	XR_001436609.1	lncRNA
XM_012188917.1	STX16	mRNA	XR_001436609.1	lncRNA
XM_012188917.1	STX16	mRNA	XR_001436609.1	lncRNA
XM_012188917.1	STX16	mRNA	XR_001436609.1	lncRNA
XM_012188917.1	STX16	mRNA	XR_001436609.1	lncRNA
XM_012188917.1	STX16	mRNA	XR_001436609.1	lncRNA
XM_012179952.2	FRYL	mRNA	XR_001436609.1	lncRNA
XM_012179952.2	FRYL	mRNA	XR_001436609.1	lncRNA
XM_012179952.2	FRYL	mRNA	XR_001436609.1	lncRNA
XM_012179952.2	FRYL	mRNA	XR_001436609.1	lncRNA
XM_012179952.2	FRYL	mRNA	XR_001436609.1	lncRNA
XM_012179952.2	FRYL	mRNA	XR_001436609.1	lncRNA
XM_012179952.2	FRYL	mRNA	XR_001436609.1	lncRNA
XM_012179952.2	FRYL	mRNA	XR_001436609.1	lncRNA
XM_012179952.2	FRYL	mRNA	XR_001436609.1	lncRNA
XM_012190696.2	CCDC155	mRNA	XR_001436609.1	lncRNA
XM_012190696.2	CCDC155	mRNA	XR_001436609.1	lncRNA
XM_012190696.2	CCDC155	mRNA	XR_001436609.1	lncRNA
XM_012190696.2	CCDC155	mRNA	XR_001436609.1	lncRNA
XM_012190696.2	CCDC155	mRNA	XR_001436609.1	lncRNA
XM_012190696.2	CCDC155	mRNA	XR_001436609.1	lncRNA
XM_012190696.2	CCDC155	mRNA	XR_001436609.1	lncRNA
XM_012190696.2	CCDC155	mRNA	XR_001436609.1	lncRNA
XM_012190696.2	CCDC155	mRNA	XR_001436609.1	lncRNA
XM_015099551.1	PARD3	mRNA	XR_001436609.1	lncRNA
XM_015099551.1	PARD3	mRNA	XR_001436609.1	lncRNA
XM_015099551.1	PARD3	mRNA	XR_001436609.1	lncRNA
XM_015099551.1	PARD3	mRNA	XR_001436609.1	lncRNA
XM_015099551.1	PARD3	mRNA	XR_001436609.1	lncRNA
XM_015099551.1	PARD3	mRNA	XR_001436609.1	lncRNA
XM_015099551.1	PARD3	mRNA	XR_001436609.1	lncRNA
XM_015099551.1	PARD3	mRNA	XR_001436609.1	lncRNA
XM_015099551.1	PARD3	mRNA	XR_001436609.1	lncRNA
XM_012152455.2	LUZP1	mRNA	XR_001021939.1	lncRNA
XM_012152455.2	LUZP1	mRNA	XR_001021939.1	lncRNA
XM_012152455.2	LUZP1	mRNA	XR_001021939.1	lncRNA
XM_012152455.2	LUZP1	mRNA	XR_001021939.1	lncRNA
XM_012152455.2	LUZP1	mRNA	XR_001021939.1	lncRNA
XM_012152455.2	LUZP1	mRNA	XR_001021939.1	lncRNA
XM_012152455.2	LUZP1	mRNA	XR_001021939.1	lncRNA
XM_012152455.2	LUZP1	mRNA	XR_001021939.1	lncRNA
XM_012152455.2	LUZP1	mRNA	XR_001021939.1	lncRNA
XM_015094919.1	WBP11	mRNA	XR_001021939.1	lncRNA
XM_015094919.1	WBP11	mRNA	XR_001021939.1	lncRNA
XM_015094919.1	WBP11	mRNA	XR_001021939.1	lncRNA
XM_015094919.1	WBP11	mRNA	XR_001021939.1	lncRNA
XM_015094919.1	WBP11	mRNA	XR_001021939.1	lncRNA
XM_015094919.1	WBP11	mRNA	XR_001021939.1	lncRNA
XM_015094919.1	WBP11	mRNA	XR_001021939.1	lncRNA
XM_015094919.1	WBP11	mRNA	XR_001021939.1	lncRNA
XM_015094919.1	WBP11	mRNA	XR_001021939.1	lncRNA
XM_012113242.2	KIAA1958	mRNA	XR_001436099.1	lncRNA
XM_012113242.2	KIAA1958	mRNA	XR_001436099.1	lncRNA
XM_012113242.2	KIAA1958	mRNA	XR_001436099.1	lncRNA

XM_012113242.2	KIAA1958	mRNA	XR_001436099.1	lncRNA
XM_012113242.2	KIAA1958	mRNA	XR_001436099.1	lncRNA
XM_012113242.2	KIAA1958	mRNA	XR_001436099.1	lncRNA
XM_012113242.2	KIAA1958	mRNA	XR_001436099.1	lncRNA
XM_012113242.2	KIAA1958	mRNA	XR_001436099.1	lncRNA
XM_012113242.2	KIAA1958	mRNA	XR_001436099.1	lncRNA
XM_012113242.2	KIAA1958	mRNA	XR_001033284.2	lncRNA
XM_012113242.2	KIAA1958	mRNA	XR_001033284.2	lncRNA
XM_012113242.2	KIAA1958	mRNA	XR_001033284.2	lncRNA
XM_012113242.2	KIAA1958	mRNA	XR_001033284.2	lncRNA
XM_012113242.2	KIAA1958	mRNA	XR_001033284.2	lncRNA
XM_012113242.2	KIAA1958	mRNA	XR_001033284.2	lncRNA
XM_012113242.2	KIAA1958	mRNA	XR_001033284.2	lncRNA
XM_012113242.2	KIAA1958	mRNA	XR_001033284.2	lncRNA
XM_012113242.2	KIAA1958	mRNA	XR_001033284.2	lncRNA
XM_015096814.1	DENND4A	mRNA	XR_001435726.1	lncRNA
XM_015096814.1	DENND4A	mRNA	XR_001435726.1	lncRNA
XM_015096814.1	DENND4A	mRNA	XR_001435726.1	lncRNA
XM_015096814.1	DENND4A	mRNA	XR_001435726.1	lncRNA
XM_015096814.1	DENND4A	mRNA	XR_001435726.1	lncRNA
XM_015096814.1	DENND4A	mRNA	XR_001435726.1	lncRNA
XM_015096814.1	DENND4A	mRNA	XR_001435726.1	lncRNA
XM_015096814.1	DENND4A	mRNA	XR_001435726.1	lncRNA
XM_015096814.1	DENND4A	mRNA	XR_001435726.1	lncRNA
XM_012098990.2	NAALADL2	mRNA	XR_001435723.1	lncRNA
XM_012098990.2	NAALADL2	mRNA	XR_001435723.1	lncRNA
XM_012098990.2	NAALADL2	mRNA	XR_001435723.1	lncRNA
XM_012098990.2	NAALADL2	mRNA	XR_001435723.1	lncRNA
XM_012098990.2	NAALADL2	mRNA	XR_001435723.1	lncRNA
XM_012098990.2	NAALADL2	mRNA	XR_001435723.1	lncRNA
XM_012098990.2	NAALADL2	mRNA	XR_001435723.1	lncRNA
XM_012098990.2	NAALADL2	mRNA	XR_001435723.1	lncRNA
XM_012098990.2	NAALADL2	mRNA	XR_001435723.1	lncRNA
XM_004008381.3	MAPK9	mRNA	XR_001042381.2	lncRNA
XM_004008381.3	MAPK9	mRNA	XR_001042381.2	lncRNA
XM_004008381.3	MAPK9	mRNA	XR_001042381.2	lncRNA
XM_004008381.3	MAPK9	mRNA	XR_001042381.2	lncRNA
XM_004008381.3	MAPK9	mRNA	XR_001042381.2	lncRNA
XM_004008381.3	MAPK9	mRNA	XR_001042381.2	lncRNA
XM_004008381.3	MAPK9	mRNA	XR_001042381.2	lncRNA
XM_004008381.3	MAPK9	mRNA	XR_001042381.2	lncRNA
XM_004008381.3	MAPK9	mRNA	XR_001042381.2	lncRNA
XM_012098990.2	NAALADL2	mRNA	XR_001435726.1	lncRNA
XM_012098990.2	NAALADL2	mRNA	XR_001435726.1	lncRNA
XM_012098990.2	NAALADL2	mRNA	XR_001435726.1	lncRNA
XM_012098990.2	NAALADL2	mRNA	XR_001435726.1	lncRNA
XM_012098990.2	NAALADL2	mRNA	XR_001435726.1	lncRNA
XM_012098990.2	NAALADL2	mRNA	XR_001435726.1	lncRNA
XM_012098990.2	NAALADL2	mRNA	XR_001435726.1	lncRNA
XM_012098990.2	NAALADL2	mRNA	XR_001435726.1	lncRNA
XM_012098990.2	NAALADL2	mRNA	XR_001435726.1	lncRNA
XM_015091899.1	SCMH1	mRNA	TCONS_00071561	lncRNA
XM_015091899.1	SCMH1	mRNA	TCONS_00071561	lncRNA
XM_015091899.1	SCMH1	mRNA	TCONS_00071561	lncRNA

XM_015091899.1	SCMH1	mRNA	TCONS_00071561	lncRNA
XM_015091899.1	SCMH1	mRNA	TCONS_00071561	lncRNA
XM_015091899.1	SCMH1	mRNA	TCONS_00071561	lncRNA
XM_015091899.1	SCMH1	mRNA	TCONS_00071561	lncRNA
XM_015091899.1	SCMH1	mRNA	TCONS_00071561	lncRNA
XM_004017655.3	PRR14L	mRNA	XR_001041209.2	lncRNA
XM_004017655.3	PRR14L	mRNA	XR_001041209.2	lncRNA
XM_004017655.3	PRR14L	mRNA	XR_001041209.2	lncRNA
XM_004017655.3	PRR14L	mRNA	XR_001041209.2	lncRNA
XM_004017655.3	PRR14L	mRNA	XR_001041209.2	lncRNA
XM_004017655.3	PRR14L	mRNA	XR_001041209.2	lncRNA
XM_004017655.3	PRR14L	mRNA	XR_001041209.2	lncRNA
XM_004017655.3	PRR14L	mRNA	XR_001041209.2	lncRNA
XM_004017655.3	PRR14L	mRNA	XR_001041209.2	lncRNA
XM_012187340.2	KIF1B	mRNA	TCONS_00029399	lncRNA
XM_012187340.2	KIF1B	mRNA	TCONS_00029399	lncRNA
XM_012187340.2	KIF1B	mRNA	TCONS_00029399	lncRNA
XM_012187340.2	KIF1B	mRNA	TCONS_00029399	lncRNA
XM_012187340.2	KIF1B	mRNA	TCONS_00029399	lncRNA
XM_012187340.2	KIF1B	mRNA	TCONS_00029399	lncRNA
XM_012187340.2	KIF1B	mRNA	TCONS_00029399	lncRNA
XM_012187340.2	KIF1B	mRNA	TCONS_00029399	lncRNA
XM_012187340.2	KIF1B	mRNA	TCONS_00029399	lncRNA
XM_012095461.2	GOLGB1	mRNA	XR_001434081.1	lncRNA
XM_012095461.2	GOLGB1	mRNA	XR_001434081.1	lncRNA
XM_012095461.2	GOLGB1	mRNA	XR_001434081.1	lncRNA
XM_012095461.2	GOLGB1	mRNA	XR_001434081.1	lncRNA
XM_012095461.2	GOLGB1	mRNA	XR_001434081.1	lncRNA
XM_012095461.2	GOLGB1	mRNA	XR_001434081.1	lncRNA
XM_012095461.2	GOLGB1	mRNA	XR_001434081.1	lncRNA
XM_012095461.2	GOLGB1	mRNA	XR_001434081.1	lncRNA
XM_012095461.2	GOLGB1	mRNA	XR_001434081.1	lncRNA
XM_012137673.2	NEB	mRNA	XR_001434081.1	lncRNA
XM_012137673.2	NEB	mRNA	XR_001434081.1	lncRNA
XM_012137673.2	NEB	mRNA	XR_001434081.1	lncRNA
XM_012137673.2	NEB	mRNA	XR_001434081.1	lncRNA
XM_012137673.2	NEB	mRNA	XR_001434081.1	lncRNA
XM_012137673.2	NEB	mRNA	XR_001434081.1	lncRNA
XM_012137673.2	NEB	mRNA	XR_001434081.1	lncRNA
XM_012137673.2	NEB	mRNA	XR_001434081.1	lncRNA
XM_012137673.2	NEB	mRNA	XR_001434081.1	lncRNA
XM_015095780.1	DNAJB1	mRNA	XR_001434081.1	lncRNA
XM_015095780.1	DNAJB1	mRNA	XR_001434081.1	lncRNA
XM_015095780.1	DNAJB1	mRNA	XR_001434081.1	lncRNA
XM_015095780.1	DNAJB1	mRNA	XR_001434081.1	lncRNA
XM_015095780.1	DNAJB1	mRNA	XR_001434081.1	lncRNA
XM_015095780.1	DNAJB1	mRNA	XR_001434081.1	lncRNA
XM_015095780.1	DNAJB1	mRNA	XR_001434081.1	lncRNA
XM_015095780.1	DNAJB1	mRNA	XR_001434081.1	lncRNA
XM_015095780.1	DNAJB1	mRNA	XR_001434081.1	lncRNA
XM_015096132.1	CSNK1A1	mRNA	XR_001436609.1	lncRNA
XM_015096132.1	CSNK1A1	mRNA	XR_001436609.1	lncRNA
XM_015096132.1	CSNK1A1	mRNA	XR_001436609.1	lncRNA
XM_015096132.1	CSNK1A1	mRNA	XR_001436609.1	lncRNA

XM_015096132.1	CSNK1A1	mRNA	XR_001436609.1	lncRNA
XM_015096132.1	CSNK1A1	mRNA	XR_001436609.1	lncRNA
XM_015096132.1	CSNK1A1	mRNA	XR_001436609.1	lncRNA
XM_015096132.1	CSNK1A1	mRNA	XR_001436609.1	lncRNA
XM_015096132.1	CSNK1A1	mRNA	XR_001436609.1	lncRNA
XM_004017729.3	CHL1	mRNA	XR_001436609.1	lncRNA
XM_004017729.3	CHL1	mRNA	XR_001436609.1	lncRNA
XM_004017729.3	CHL1	mRNA	XR_001436609.1	lncRNA
XM_004017729.3	CHL1	mRNA	XR_001436609.1	lncRNA
XM_004017729.3	CHL1	mRNA	XR_001436609.1	lncRNA
XM_004017729.3	CHL1	mRNA	XR_001436609.1	lncRNA
XM_004017729.3	CHL1	mRNA	XR_001436609.1	lncRNA
XM_004017729.3	CHL1	mRNA	XR_001436609.1	lncRNA
XM_004017729.3	CHL1	mRNA	XR_001436609.1	lncRNA
XM_015093632.1	EPB41	mRNA	XR_001435104.1	lncRNA
XM_015093632.1	EPB41	mRNA	XR_001435104.1	lncRNA
XM_015093632.1	EPB41	mRNA	XR_001435104.1	lncRNA
XM_015093632.1	EPB41	mRNA	XR_001435104.1	lncRNA
XM_015093632.1	EPB41	mRNA	XR_001435104.1	lncRNA
XM_015093632.1	EPB41	mRNA	XR_001435104.1	lncRNA
XM_015093632.1	EPB41	mRNA	XR_001435104.1	lncRNA
XM_015093632.1	EPB41	mRNA	XR_001435104.1	lncRNA
XM_015093632.1	EPB41	mRNA	XR_001435104.1	lncRNA
XM_015093088.1	CDCA2	mRNA	XR_001436609.1	lncRNA
XM_015093088.1	CDCA2	mRNA	XR_001436609.1	lncRNA
XM_015093088.1	CDCA2	mRNA	XR_001436609.1	lncRNA
XM_015093088.1	CDCA2	mRNA	XR_001436609.1	lncRNA
XM_015093088.1	CDCA2	mRNA	XR_001436609.1	lncRNA
XM_015093088.1	CDCA2	mRNA	XR_001436609.1	lncRNA
XM_015093088.1	CDCA2	mRNA	XR_001436609.1	lncRNA
XM_015093088.1	CDCA2	mRNA	XR_001436609.1	lncRNA
XM_015093088.1	CDCA2	mRNA	XR_001436609.1	lncRNA
XM_012187292.1	VPS13D	mRNA	XR_001435104.1	lncRNA
XM_012187292.1	VPS13D	mRNA	XR_001435104.1	lncRNA
XM_012187292.1	VPS13D	mRNA	XR_001435104.1	lncRNA
XM_012187292.1	VPS13D	mRNA	XR_001435104.1	lncRNA
XM_012187292.1	VPS13D	mRNA	XR_001435104.1	lncRNA
XM_012187292.1	VPS13D	mRNA	XR_001435104.1	lncRNA
XM_012187292.1	VPS13D	mRNA	XR_001435104.1	lncRNA
XM_012187292.1	VPS13D	mRNA	XR_001435104.1	lncRNA
XM_012187292.1	VPS13D	mRNA	XR_001435104.1	lncRNA
XM_012101112.2	ABT1	mRNA	XR_001436609.1	lncRNA
XM_012101112.2	ABT1	mRNA	XR_001436609.1	lncRNA
XM_012101112.2	ABT1	mRNA	XR_001436609.1	lncRNA
XM_012101112.2	ABT1	mRNA	XR_001436609.1	lncRNA
XM_012101112.2	ABT1	mRNA	XR_001436609.1	lncRNA
XM_012101112.2	ABT1	mRNA	XR_001436609.1	lncRNA
XM_012101112.2	ABT1	mRNA	XR_001436609.1	lncRNA
XM_012101112.2	ABT1	mRNA	XR_001436609.1	lncRNA
XM_012101112.2	ABT1	mRNA	XR_001436609.1	lncRNA
XM_004017729.3	CHL1	mRNA	XR_001436531.1	lncRNA
XM_004017729.3	CHL1	mRNA	XR_001436531.1	lncRNA
XM_004017729.3	CHL1	mRNA	XR_001436531.1	lncRNA
XM_004017729.3	CHL1	mRNA	XR_001436531.1	lncRNA

XM_004017729.3	CHL1	mRNA	XR_001436531.1	lncRNA
XM_004017729.3	CHL1	mRNA	XR_001436531.1	lncRNA
XM_004017729.3	CHL1	mRNA	XR_001436531.1	lncRNA
XM_004017729.3	CHL1	mRNA	XR_001436531.1	lncRNA
XM_004017729.3	CHL1	mRNA	XR_001436531.1	lncRNA
XM_012098436.1	KLHL25	mRNA	XR_001435104.1	lncRNA
XM_012098436.1	KLHL25	mRNA	XR_001435104.1	lncRNA
XM_012098436.1	KLHL25	mRNA	XR_001435104.1	lncRNA
XM_012098436.1	KLHL25	mRNA	XR_001435104.1	lncRNA
XM_012098436.1	KLHL25	mRNA	XR_001435104.1	lncRNA
XM_012098436.1	KLHL25	mRNA	XR_001435104.1	lncRNA
XM_012098436.1	KLHL25	mRNA	XR_001435104.1	lncRNA
XM_012098436.1	KLHL25	mRNA	XR_001435104.1	lncRNA
XM_012098436.1	KLHL25	mRNA	XR_001435104.1	lncRNA
XM_012098886.2	TP53BP1	mRNA	XR_001021689.2	lncRNA
XM_012098886.2	TP53BP1	mRNA	XR_001021689.2	lncRNA
XM_012098886.2	TP53BP1	mRNA	XR_001021689.2	lncRNA
XM_012098886.2	TP53BP1	mRNA	XR_001021689.2	lncRNA
XM_012098886.2	TP53BP1	mRNA	XR_001021689.2	lncRNA
XM_012098886.2	TP53BP1	mRNA	XR_001021689.2	lncRNA
XM_012098886.2	TP53BP1	mRNA	XR_001021689.2	lncRNA
XM_012098886.2	TP53BP1	mRNA	XR_001021689.2	lncRNA
XM_004012194.3	RBM26	mRNA	XR_001033284.2	lncRNA
XM_004012194.3	RBM26	mRNA	XR_001033284.2	lncRNA
XM_004012194.3	RBM26	mRNA	XR_001033284.2	lncRNA
XM_004012194.3	RBM26	mRNA	XR_001033284.2	lncRNA
XM_004012194.3	RBM26	mRNA	XR_001033284.2	lncRNA
XM_004012194.3	RBM26	mRNA	XR_001033284.2	lncRNA
XM_004012194.3	RBM26	mRNA	XR_001033284.2	lncRNA
XM_004012194.3	RBM26	mRNA	XR_001033284.2	lncRNA
XM_012097252.2	RHOF	mRNA	TCONS_00029399	lncRNA
XM_012097252.2	RHOF	mRNA	TCONS_00029399	lncRNA
XM_012097252.2	RHOF	mRNA	TCONS_00029399	lncRNA
XM_012097252.2	RHOF	mRNA	TCONS_00029399	lncRNA
XM_012097252.2	RHOF	mRNA	TCONS_00029399	lncRNA
XM_012097252.2	RHOF	mRNA	TCONS_00029399	lncRNA
XM_012097252.2	RHOF	mRNA	TCONS_00029399	lncRNA
XM_012097252.2	RHOF	mRNA	TCONS_00029399	lncRNA
XM_012097252.2	RHOF	mRNA	TCONS_00029399	lncRNA
XM_012100587.2	BTBD9	mRNA	TCONS_00029399	lncRNA
XM_012100587.2	BTBD9	mRNA	TCONS_00029399	lncRNA
XM_012100587.2	BTBD9	mRNA	TCONS_00029399	lncRNA
XM_012100587.2	BTBD9	mRNA	TCONS_00029399	lncRNA
XM_012100587.2	BTBD9	mRNA	TCONS_00029399	lncRNA
XM_012100587.2	BTBD9	mRNA	TCONS_00029399	lncRNA
XM_012100587.2	BTBD9	mRNA	TCONS_00029399	lncRNA
XM_012100587.2	BTBD9	mRNA	TCONS_00029399	lncRNA
XM_012100587.2	BTBD9	mRNA	TCONS_00029399	lncRNA
XM_015091887.1	MYCL	mRNA	XR_001434081.1	lncRNA
XM_015091887.1	MYCL	mRNA	XR_001434081.1	lncRNA
XM_015091887.1	MYCL	mRNA	XR_001434081.1	lncRNA
XM_015091887.1	MYCL	mRNA	XR_001434081.1	lncRNA
XM_015091887.1	MYCL	mRNA	XR_001434081.1	lncRNA
XM_015091887.1	MYCL	mRNA	XR_001434081.1	lncRNA

XM_015091887.1	MYCL	mRNA	XR_001434081.1	lncRNA
XM_015091887.1	MYCL	mRNA	XR_001434081.1	lncRNA
XM_015091887.1	MYCL	mRNA	XR_001434081.1	lncRNA
XM_015099551.1	PARD3	mRNA	XR_001041202.1	lncRNA
XM_015099551.1	PARD3	mRNA	XR_001041202.1	lncRNA
XM_015099551.1	PARD3	mRNA	XR_001041202.1	lncRNA
XM_015099551.1	PARD3	mRNA	XR_001041202.1	lncRNA
XM_015099551.1	PARD3	mRNA	XR_001041202.1	lncRNA
XM_015099551.1	PARD3	mRNA	XR_001041202.1	lncRNA
XM_015099551.1	PARD3	mRNA	XR_001041202.1	lncRNA
XM_015099551.1	PARD3	mRNA	XR_001041202.1	lncRNA
XM_004012926.2	ATP6V0A1	mRNA	XR_001042381.2	lncRNA
XM_004012926.2	ATP6V0A1	mRNA	XR_001042381.2	lncRNA
XM_004012926.2	ATP6V0A1	mRNA	XR_001042381.2	lncRNA
XM_004012926.2	ATP6V0A1	mRNA	XR_001042381.2	lncRNA
XM_004012926.2	ATP6V0A1	mRNA	XR_001042381.2	lncRNA
XM_004012926.2	ATP6V0A1	mRNA	XR_001042381.2	lncRNA
XM_004012926.2	ATP6V0A1	mRNA	XR_001042381.2	lncRNA
XM_004012926.2	ATP6V0A1	mRNA	XR_001042381.2	lncRNA
XM_004012926.2	ATP6V0A1	mRNA	XR_001042381.2	lncRNA
XM_012176678.2	KMT2E	mRNA	XR_001021939.1	lncRNA
XM_012176678.2	KMT2E	mRNA	XR_001021939.1	lncRNA
XM_012176678.2	KMT2E	mRNA	XR_001021939.1	lncRNA
XM_012176678.2	KMT2E	mRNA	XR_001021939.1	lncRNA
XM_012176678.2	KMT2E	mRNA	XR_001021939.1	lncRNA
XM_012176678.2	KMT2E	mRNA	XR_001021939.1	lncRNA
XM_012176678.2	KMT2E	mRNA	XR_001021939.1	lncRNA
XM_012176678.2	KMT2E	mRNA	XR_001021939.1	lncRNA
XM_015092483.1	SENP5	mRNA	XR_001021939.1	lncRNA
XM_015092483.1	SENP5	mRNA	XR_001021939.1	lncRNA
XM_015092483.1	SENP5	mRNA	XR_001021939.1	lncRNA
XM_015092483.1	SENP5	mRNA	XR_001021939.1	lncRNA
XM_015092483.1	SENP5	mRNA	XR_001021939.1	lncRNA
XM_015092483.1	SENP5	mRNA	XR_001021939.1	lncRNA
XM_015092483.1	SENP5	mRNA	XR_001021939.1	lncRNA
XM_015092483.1	SENP5	mRNA	XR_001021939.1	lncRNA
XM_012095465.2	GOLGB1	mRNA	TCONS_00071561	lncRNA
XM_012095465.2	GOLGB1	mRNA	TCONS_00071561	lncRNA
XM_012095465.2	GOLGB1	mRNA	TCONS_00071561	lncRNA
XM_012095465.2	GOLGB1	mRNA	TCONS_00071561	lncRNA
XM_012095465.2	GOLGB1	mRNA	TCONS_00071561	lncRNA
XM_012095465.2	GOLGB1	mRNA	TCONS_00071561	lncRNA
XM_012095465.2	GOLGB1	mRNA	TCONS_00071561	lncRNA
XM_012095465.2	GOLGB1	mRNA	TCONS_00071561	lncRNA
XM_012137673.2	NEB	mRNA	XR_001021689.2	lncRNA
XM_012137673.2	NEB	mRNA	XR_001021689.2	lncRNA
XM_012137673.2	NEB	mRNA	XR_001021689.2	lncRNA
XM_012137673.2	NEB	mRNA	XR_001021689.2	lncRNA
XM_012137673.2	NEB	mRNA	XR_001021689.2	lncRNA
XM_012137673.2	NEB	mRNA	XR_001021689.2	lncRNA
XM_012137673.2	NEB	mRNA	XR_001021689.2	lncRNA
XM_012137673.2	NEB	mRNA	XR_001021689.2	lncRNA
XM_012189777.1	CHD9	mRNA	XR_001021689.2	lncRNA
XM_012189777.1	CHD9	mRNA	XR_001021689.2	lncRNA

XM_012189777.1	CHD9	mRNA	XR_001021689.2	lncRNA
XM_012189777.1	CHD9	mRNA	XR_001021689.2	lncRNA
XM_012189777.1	CHD9	mRNA	XR_001021689.2	lncRNA
XM_012189777.1	CHD9	mRNA	XR_001021689.2	lncRNA
XM_012189777.1	CHD9	mRNA	XR_001021689.2	lncRNA
XM_012189777.1	CHD9	mRNA	XR_001021689.2	lncRNA
XM_012095461.2	GOLGB1	mRNA	XR_001021689.2	lncRNA
XM_012095461.2	GOLGB1	mRNA	XR_001021689.2	lncRNA
XM_012095461.2	GOLGB1	mRNA	XR_001021689.2	lncRNA
XM_012095461.2	GOLGB1	mRNA	XR_001021689.2	lncRNA
XM_012095461.2	GOLGB1	mRNA	XR_001021689.2	lncRNA
XM_012095461.2	GOLGB1	mRNA	XR_001021689.2	lncRNA
XM_012095461.2	GOLGB1	mRNA	XR_001021689.2	lncRNA
XM_012095461.2	GOLGB1	mRNA	XR_001021689.2	lncRNA
XM_012104178.2	CCP110	mRNA	XR_001021689.2	lncRNA
XM_012104178.2	CCP110	mRNA	XR_001021689.2	lncRNA
XM_012104178.2	CCP110	mRNA	XR_001021689.2	lncRNA
XM_012104178.2	CCP110	mRNA	XR_001021689.2	lncRNA
XM_012104178.2	CCP110	mRNA	XR_001021689.2	lncRNA
XM_012104178.2	CCP110	mRNA	XR_001021689.2	lncRNA
XM_012104178.2	CCP110	mRNA	XR_001021689.2	lncRNA
XM_012104178.2	CCP110	mRNA	XR_001021689.2	lncRNA
XM_004003157.3	TNIK	mRNA	TCONS_00071561	lncRNA
XM_004003157.3	TNIK	mRNA	TCONS_00071561	lncRNA
XM_004003157.3	TNIK	mRNA	TCONS_00071561	lncRNA
XM_004003157.3	TNIK	mRNA	TCONS_00071561	lncRNA
XM_004003157.3	TNIK	mRNA	TCONS_00071561	lncRNA
XM_004003157.3	TNIK	mRNA	TCONS_00071561	lncRNA
XM_004003157.3	TNIK	mRNA	TCONS_00071561	lncRNA
XM_004003157.3	TNIK	mRNA	TCONS_00071561	lncRNA
XM_015096769.1	APC	mRNA	XR_001021689.2	lncRNA
XM_015096769.1	APC	mRNA	XR_001021689.2	lncRNA
XM_015096769.1	APC	mRNA	XR_001021689.2	lncRNA
XM_015096769.1	APC	mRNA	XR_001021689.2	lncRNA
XM_015096769.1	APC	mRNA	XR_001021689.2	lncRNA
XM_015096769.1	APC	mRNA	XR_001021689.2	lncRNA
XM_015096769.1	APC	mRNA	XR_001021689.2	lncRNA
XM_015096769.1	APC	mRNA	XR_001021689.2	lncRNA
XM_004017788.3	ZFANL3	mRNA	XR_001041209.2	lncRNA
XM_004017788.3	ZFANL3	mRNA	XR_001041209.2	lncRNA
XM_004017788.3	ZFANL3	mRNA	XR_001041209.2	lncRNA
XM_004017788.3	ZFANL3	mRNA	XR_001041209.2	lncRNA
XM_004017788.3	ZFANL3	mRNA	XR_001041209.2	lncRNA
XM_004017788.3	ZFANL3	mRNA	XR_001041209.2	lncRNA
XM_004017788.3	ZFANL3	mRNA	XR_001041209.2	lncRNA
XM_004017788.3	ZFANL3	mRNA	XR_001041209.2	lncRNA
XM_012095713.2	RRAS2	mRNA	XR_001041209.2	lncRNA
XM_012095713.2	RRAS2	mRNA	XR_001041209.2	lncRNA
XM_012095713.2	RRAS2	mRNA	XR_001041209.2	lncRNA
XM_012095713.2	RRAS2	mRNA	XR_001041209.2	lncRNA
XM_012095713.2	RRAS2	mRNA	XR_001041209.2	lncRNA
XM_012095713.2	RRAS2	mRNA	XR_001041209.2	lncRNA
XM_012095713.2	RRAS2	mRNA	XR_001041209.2	lncRNA
XM_012095713.2	RRAS2	mRNA	XR_001041209.2	lncRNA

XM_012178641.2	CTTNBP2NL	mRNA	TCONS_00029399	lncRNA
XM_012178641.2	CTTNBP2NL	mRNA	TCONS_00029399	lncRNA
XM_012178641.2	CTTNBP2NL	mRNA	TCONS_00029399	lncRNA
XM_012178641.2	CTTNBP2NL	mRNA	TCONS_00029399	lncRNA
XM_012178641.2	CTTNBP2NL	mRNA	TCONS_00029399	lncRNA
XM_012178641.2	CTTNBP2NL	mRNA	TCONS_00029399	lncRNA
XM_012178641.2	CTTNBP2NL	mRNA	TCONS_00029399	lncRNA
XM_012178641.2	CTTNBP2NL	mRNA	TCONS_00029399	lncRNA
XM_012178641.2	CTTNBP2NL	mRNA	XR_001041209.2	lncRNA
XM_012178641.2	CTTNBP2NL	mRNA	XR_001041209.2	lncRNA
XM_012178641.2	CTTNBP2NL	mRNA	XR_001041209.2	lncRNA
XM_012178641.2	CTTNBP2NL	mRNA	XR_001041209.2	lncRNA
XM_012178641.2	CTTNBP2NL	mRNA	XR_001041209.2	lncRNA
XM_012178641.2	CTTNBP2NL	mRNA	XR_001041209.2	lncRNA
XM_012178641.2	CTTNBP2NL	mRNA	XR_001041209.2	lncRNA
XM_012178641.2	CTTNBP2NL	mRNA	XR_001041209.2	lncRNA
XM_012146156.1	USP37	mRNA	TCONS_00029399	lncRNA
XM_012146156.1	USP37	mRNA	TCONS_00029399	lncRNA
XM_012146156.1	USP37	mRNA	TCONS_00029399	lncRNA
XM_012146156.1	USP37	mRNA	TCONS_00029399	lncRNA
XM_012146156.1	USP37	mRNA	TCONS_00029399	lncRNA
XM_012146156.1	USP37	mRNA	TCONS_00029399	lncRNA
XM_012146156.1	USP37	mRNA	TCONS_00029399	lncRNA
XM_012146156.1	USP37	mRNA	TCONS_00029399	lncRNA
XM_015092478.1	TFRC	mRNA	XR_001436609.1	lncRNA
XM_015092478.1	TFRC	mRNA	XR_001436609.1	lncRNA
XM_015092478.1	TFRC	mRNA	XR_001436609.1	lncRNA
XM_015092478.1	TFRC	mRNA	XR_001436609.1	lncRNA
XM_015092478.1	TFRC	mRNA	XR_001436609.1	lncRNA
XM_015092478.1	TFRC	mRNA	XR_001436609.1	lncRNA
XM_015092478.1	TFRC	mRNA	XR_001436609.1	lncRNA
XM_015092478.1	TFRC	mRNA	XR_001436609.1	lncRNA
XM_012165221.2	PUM2	mRNA	XR_001436531.1	lncRNA
XM_012165221.2	PUM2	mRNA	XR_001436531.1	lncRNA
XM_012165221.2	PUM2	mRNA	XR_001436531.1	lncRNA
XM_012165221.2	PUM2	mRNA	XR_001436531.1	lncRNA
XM_012165221.2	PUM2	mRNA	XR_001436531.1	lncRNA
XM_012165221.2	PUM2	mRNA	XR_001436531.1	lncRNA
XM_012165221.2	PUM2	mRNA	XR_001436531.1	lncRNA
XM_012165221.2	PUM2	mRNA	XR_001436531.1	lncRNA
XM_012129647.1	NEK1	mRNA	XR_001435104.1	lncRNA
XM_012129647.1	NEK1	mRNA	XR_001435104.1	lncRNA
XM_012129647.1	NEK1	mRNA	XR_001435104.1	lncRNA
XM_012129647.1	NEK1	mRNA	XR_001435104.1	lncRNA
XM_012129647.1	NEK1	mRNA	XR_001435104.1	lncRNA
XM_012129647.1	NEK1	mRNA	XR_001435104.1	lncRNA
XM_012129647.1	NEK1	mRNA	XR_001435104.1	lncRNA
XM_012129647.1	NEK1	mRNA	XR_001435104.1	lncRNA
XM_015100095.1	LOC10111352	mRNA	XR_001435104.1	lncRNA
XM_015100095.1	LOC10111352	mRNA	XR_001435104.1	lncRNA
XM_015100095.1	LOC10111352	mRNA	XR_001435104.1	lncRNA
XM_015100095.1	LOC10111352	mRNA	XR_001435104.1	lncRNA
XM_015100095.1	LOC10111352	mRNA	XR_001435104.1	lncRNA
XM_015100095.1	LOC10111352	mRNA	XR_001435104.1	lncRNA

XM_015100095.1	LOC10111352	mRNA	XR_001435104.1	lncRNA
XM_015100095.1	LOC10111352	mRNA	XR_001435104.1	lncRNA
XM_012176678.2	KMT2E	mRNA	XR_001435104.1	lncRNA
XM_012176678.2	KMT2E	mRNA	XR_001435104.1	lncRNA
XM_012176678.2	KMT2E	mRNA	XR_001435104.1	lncRNA
XM_012176678.2	KMT2E	mRNA	XR_001435104.1	lncRNA
XM_012176678.2	KMT2E	mRNA	XR_001435104.1	lncRNA
XM_012176678.2	KMT2E	mRNA	XR_001435104.1	lncRNA
XM_012176678.2	KMT2E	mRNA	XR_001435104.1	lncRNA
XM_012176678.2	KMT2E	mRNA	XR_001435104.1	lncRNA
XM_015100095.1	LOC10111352	mRNA	XR_001436609.1	lncRNA
XM_015100095.1	LOC10111352	mRNA	XR_001436609.1	lncRNA
XM_015100095.1	LOC10111352	mRNA	XR_001436609.1	lncRNA
XM_015100095.1	LOC10111352	mRNA	XR_001436609.1	lncRNA
XM_015100095.1	LOC10111352	mRNA	XR_001436609.1	lncRNA
XM_015100095.1	LOC10111352	mRNA	XR_001436609.1	lncRNA
XM_015100095.1	LOC10111352	mRNA	XR_001436609.1	lncRNA
XM_015100095.1	LOC10111352	mRNA	XR_001436609.1	lncRNA
XM_012097468.2	ELF2	mRNA	XR_001436609.1	lncRNA
XM_012097468.2	ELF2	mRNA	XR_001436609.1	lncRNA
XM_012097468.2	ELF2	mRNA	XR_001436609.1	lncRNA
XM_012097468.2	ELF2	mRNA	XR_001436609.1	lncRNA
XM_012097468.2	ELF2	mRNA	XR_001436609.1	lncRNA
XM_012097468.2	ELF2	mRNA	XR_001436609.1	lncRNA
XM_012097468.2	ELF2	mRNA	XR_001436609.1	lncRNA
XM_012097468.2	ELF2	mRNA	XR_001436609.1	lncRNA
XM_012178157.2	RAPGEF6	mRNA	XR_001436609.1	lncRNA
XM_012178157.2	RAPGEF6	mRNA	XR_001436609.1	lncRNA
XM_012178157.2	RAPGEF6	mRNA	XR_001436609.1	lncRNA
XM_012178157.2	RAPGEF6	mRNA	XR_001436609.1	lncRNA
XM_012178157.2	RAPGEF6	mRNA	XR_001436609.1	lncRNA
XM_012178157.2	RAPGEF6	mRNA	XR_001436609.1	lncRNA
XM_012178157.2	RAPGEF6	mRNA	XR_001436609.1	lncRNA
XM_012178157.2	RAPGEF6	mRNA	XR_001436609.1	lncRNA
XM_012181854.1	GPHN	mRNA	XR_001436609.1	lncRNA
XM_012181854.1	GPHN	mRNA	XR_001436609.1	lncRNA
XM_012181854.1	GPHN	mRNA	XR_001436609.1	lncRNA
XM_012181854.1	GPHN	mRNA	XR_001436609.1	lncRNA
XM_012181854.1	GPHN	mRNA	XR_001436609.1	lncRNA
XM_012181854.1	GPHN	mRNA	XR_001436609.1	lncRNA
XM_012181854.1	GPHN	mRNA	XR_001436609.1	lncRNA
XM_012181854.1	GPHN	mRNA	XR_001436609.1	lncRNA
XM_012173383.2	DHX57	mRNA	XR_001436609.1	lncRNA
XM_012173383.2	DHX57	mRNA	XR_001436609.1	lncRNA
XM_012173383.2	DHX57	mRNA	XR_001436609.1	lncRNA
XM_012173383.2	DHX57	mRNA	XR_001436609.1	lncRNA
XM_012173383.2	DHX57	mRNA	XR_001436609.1	lncRNA
XM_012173383.2	DHX57	mRNA	XR_001436609.1	lncRNA
XM_012173383.2	DHX57	mRNA	XR_001436609.1	lncRNA
XM_012173383.2	DHX57	mRNA	XR_001436609.1	lncRNA
XM_012099659.2	FOXP1	mRNA	XR_001436099.1	lncRNA
XM_012099659.2	FOXP1	mRNA	XR_001436099.1	lncRNA
XM_012099659.2	FOXP1	mRNA	XR_001436099.1	lncRNA
XM_012099659.2	FOXP1	mRNA	XR_001436099.1	lncRNA

XM_012099659.2	FOXP1	mRNA	XR_001436099.1	lncRNA
XM_012099659.2	FOXP1	mRNA	XR_001436099.1	lncRNA
XM_012099659.2	FOXP1	mRNA	XR_001436099.1	lncRNA
XM_012099659.2	FOXP1	mRNA	XR_001436099.1	lncRNA
XM_012182538.2	TRAPPC3L	mRNA	XR_001033284.2	lncRNA
XM_012182538.2	TRAPPC3L	mRNA	XR_001033284.2	lncRNA
XM_012182538.2	TRAPPC3L	mRNA	XR_001033284.2	lncRNA
XM_012182538.2	TRAPPC3L	mRNA	XR_001033284.2	lncRNA
XM_012182538.2	TRAPPC3L	mRNA	XR_001033284.2	lncRNA
XM_012182538.2	TRAPPC3L	mRNA	XR_001033284.2	lncRNA
XM_012182538.2	TRAPPC3L	mRNA	XR_001033284.2	lncRNA
XM_012182538.2	TRAPPC3L	mRNA	XR_001033284.2	lncRNA
XM_012183298.1	FAM120B	mRNA	XR_001033284.2	lncRNA
XM_012183298.1	FAM120B	mRNA	XR_001033284.2	lncRNA
XM_012183298.1	FAM120B	mRNA	XR_001033284.2	lncRNA
XM_012183298.1	FAM120B	mRNA	XR_001033284.2	lncRNA
XM_012183298.1	FAM120B	mRNA	XR_001033284.2	lncRNA
XM_012183298.1	FAM120B	mRNA	XR_001033284.2	lncRNA
XM_012183298.1	FAM120B	mRNA	XR_001033284.2	lncRNA
XM_012183298.1	FAM120B	mRNA	XR_001033284.2	lncRNA
XM_012122525.2	PRUNE2	mRNA	XR_001041202.1	lncRNA
XM_012122525.2	PRUNE2	mRNA	XR_001041202.1	lncRNA
XM_012122525.2	PRUNE2	mRNA	XR_001041202.1	lncRNA
XM_012122525.2	PRUNE2	mRNA	XR_001041202.1	lncRNA
XM_012122525.2	PRUNE2	mRNA	XR_001041202.1	lncRNA
XM_012122525.2	PRUNE2	mRNA	XR_001041202.1	lncRNA
XM_012122525.2	PRUNE2	mRNA	XR_001041202.1	lncRNA
XM_012122525.2	PRUNE2	mRNA	XR_001041202.1	lncRNA
XM_012167258.2	CLIP4	mRNA	XR_001041202.1	lncRNA
XM_012167258.2	CLIP4	mRNA	XR_001041202.1	lncRNA
XM_012167258.2	CLIP4	mRNA	XR_001041202.1	lncRNA
XM_012167258.2	CLIP4	mRNA	XR_001041202.1	lncRNA
XM_012167258.2	CLIP4	mRNA	XR_001041202.1	lncRNA
XM_012167258.2	CLIP4	mRNA	XR_001041202.1	lncRNA
XM_012167258.2	CLIP4	mRNA	XR_001041202.1	lncRNA
XM_012167258.2	CLIP4	mRNA	XR_001041202.1	lncRNA
XM_012173782.2	REV1	mRNA	XR_001041202.1	lncRNA
XM_012173782.2	REV1	mRNA	XR_001041202.1	lncRNA
XM_012173782.2	REV1	mRNA	XR_001041202.1	lncRNA
XM_012173782.2	REV1	mRNA	XR_001041202.1	lncRNA
XM_012173782.2	REV1	mRNA	XR_001041202.1	lncRNA
XM_012173782.2	REV1	mRNA	XR_001041202.1	lncRNA
XM_012173782.2	REV1	mRNA	XR_001041202.1	lncRNA
XM_012173782.2	REV1	mRNA	XR_001041202.1	lncRNA
XM_012181966.2	SIPA1L1	mRNA	XR_001021689.2	lncRNA
XM_012181966.2	SIPA1L1	mRNA	XR_001021689.2	lncRNA
XM_012181966.2	SIPA1L1	mRNA	XR_001021689.2	lncRNA
XM_012181966.2	SIPA1L1	mRNA	XR_001021689.2	lncRNA
XM_012181966.2	SIPA1L1	mRNA	XR_001021689.2	lncRNA
XM_012181966.2	SIPA1L1	mRNA	XR_001021689.2	lncRNA
XM_012181966.2	SIPA1L1	mRNA	XR_001021689.2	lncRNA
XM_012181966.2	SIPA1L1	mRNA	XR_001021689.2	lncRNA
XM_015102914.1	NAV2	mRNA	TCONS_00071561	lncRNA
XM_015102914.1	NAV2	mRNA	TCONS_00071561	lncRNA

XM_015102914.1	NAV2	mRNA	TCONS_00071561	lncRNA
XM_015102914.1	NAV2	mRNA	TCONS_00071561	lncRNA
XM_015102914.1	NAV2	mRNA	TCONS_00071561	lncRNA
XM_015102914.1	NAV2	mRNA	TCONS_00071561	lncRNA
XM_015102914.1	NAV2	mRNA	TCONS_00071561	lncRNA
XM_015102914.1	NAV2	mRNA	TCONS_00071561	lncRNA
XM_004010549.3	VPS13C	mRNA	TCONS_00071561	lncRNA
XM_004010549.3	VPS13C	mRNA	TCONS_00071561	lncRNA
XM_004010549.3	VPS13C	mRNA	TCONS_00071561	lncRNA
XM_004010549.3	VPS13C	mRNA	TCONS_00071561	lncRNA
XM_004010549.3	VPS13C	mRNA	TCONS_00071561	lncRNA
XM_004010549.3	VPS13C	mRNA	TCONS_00071561	lncRNA
XM_004010549.3	VPS13C	mRNA	TCONS_00071561	lncRNA
XM_004010549.3	VPS13C	mRNA	TCONS_00071561	lncRNA
XM_012187729.2	SWT1	mRNA	XR_001042381.2	lncRNA
XM_012187729.2	SWT1	mRNA	XR_001042381.2	lncRNA
XM_012187729.2	SWT1	mRNA	XR_001042381.2	lncRNA
XM_012187729.2	SWT1	mRNA	XR_001042381.2	lncRNA
XM_012187729.2	SWT1	mRNA	XR_001042381.2	lncRNA
XM_012187729.2	SWT1	mRNA	XR_001042381.2	lncRNA
XM_012187729.2	SWT1	mRNA	XR_001042381.2	lncRNA
XM_012187729.2	SWT1	mRNA	XR_001042381.2	lncRNA
XM_004002409.3	PDZK1	mRNA	XR_001435726.1	lncRNA
XM_004002409.3	PDZK1	mRNA	XR_001435726.1	lncRNA
XM_004002409.3	PDZK1	mRNA	XR_001435726.1	lncRNA
XM_004002409.3	PDZK1	mRNA	XR_001435726.1	lncRNA
XM_004002409.3	PDZK1	mRNA	XR_001435726.1	lncRNA
XM_004002409.3	PDZK1	mRNA	XR_001435726.1	lncRNA
XM_004002409.3	PDZK1	mRNA	XR_001435726.1	lncRNA
XM_004002409.3	PDZK1	mRNA	XR_001435726.1	lncRNA
XM_012099837.2	SLMAP	mRNA	XR_001435723.1	lncRNA
XM_012099837.2	SLMAP	mRNA	XR_001435723.1	lncRNA
XM_012099837.2	SLMAP	mRNA	XR_001435723.1	lncRNA
XM_012099837.2	SLMAP	mRNA	XR_001435723.1	lncRNA
XM_012099837.2	SLMAP	mRNA	XR_001435723.1	lncRNA
XM_012099837.2	SLMAP	mRNA	XR_001435723.1	lncRNA
XM_012099837.2	SLMAP	mRNA	XR_001435723.1	lncRNA
XM_012099837.2	SLMAP	mRNA	XR_001435723.1	lncRNA
XM_012098342.2	FXR1	mRNA	XR_001042381.2	lncRNA
XM_012098342.2	FXR1	mRNA	XR_001042381.2	lncRNA
XM_012098342.2	FXR1	mRNA	XR_001042381.2	lncRNA
XM_012098342.2	FXR1	mRNA	XR_001042381.2	lncRNA
XM_012098342.2	FXR1	mRNA	XR_001042381.2	lncRNA
XM_012098342.2	FXR1	mRNA	XR_001042381.2	lncRNA
XM_012098342.2	FXR1	mRNA	XR_001042381.2	lncRNA
XM_012098342.2	FXR1	mRNA	XR_001042381.2	lncRNA
XM_012174607.2	HELB	mRNA	XR_001435726.1	lncRNA
XM_012174607.2	HELB	mRNA	XR_001435726.1	lncRNA
XM_012174607.2	HELB	mRNA	XR_001435726.1	lncRNA
XM_012174607.2	HELB	mRNA	XR_001435726.1	lncRNA
XM_012174607.2	HELB	mRNA	XR_001435726.1	lncRNA
XM_012174607.2	HELB	mRNA	XR_001435726.1	lncRNA
XM_012174607.2	HELB	mRNA	XR_001435726.1	lncRNA
XM_012174607.2	HELB	mRNA	XR_001435726.1	lncRNA

XM_015100095.1	LOC10111352	mRNA	XR_001435726.1	lncRNA
XM_015100095.1	LOC10111352	mRNA	XR_001435726.1	lncRNA
XM_015100095.1	LOC10111352	mRNA	XR_001435726.1	lncRNA
XM_015100095.1	LOC10111352	mRNA	XR_001435726.1	lncRNA
XM_015100095.1	LOC10111352	mRNA	XR_001435726.1	lncRNA
XM_015100095.1	LOC10111352	mRNA	XR_001435726.1	lncRNA
XM_015100095.1	LOC10111352	mRNA	XR_001435726.1	lncRNA
XM_015100095.1	LOC10111352	mRNA	XR_001435726.1	lncRNA
XM_015099384.1	SYT14	mRNA	XR_001435723.1	lncRNA
XM_015099384.1	SYT14	mRNA	XR_001435723.1	lncRNA
XM_015099384.1	SYT14	mRNA	XR_001435723.1	lncRNA
XM_015099384.1	SYT14	mRNA	XR_001435723.1	lncRNA
XM_015099384.1	SYT14	mRNA	XR_001435723.1	lncRNA
XM_015099384.1	SYT14	mRNA	XR_001435723.1	lncRNA
XM_015099384.1	SYT14	mRNA	XR_001435723.1	lncRNA
XM_015099384.1	SYT14	mRNA	XR_001435723.1	lncRNA
XM_012099837.2	SLMAP	mRNA	XR_001435726.1	lncRNA
XM_012099837.2	SLMAP	mRNA	XR_001435726.1	lncRNA
XM_012099837.2	SLMAP	mRNA	XR_001435726.1	lncRNA
XM_012099837.2	SLMAP	mRNA	XR_001435726.1	lncRNA
XM_012099837.2	SLMAP	mRNA	XR_001435726.1	lncRNA
XM_012099837.2	SLMAP	mRNA	XR_001435726.1	lncRNA
XM_012099837.2	SLMAP	mRNA	XR_001435726.1	lncRNA
XM_012099837.2	SLMAP	mRNA	XR_001435726.1	lncRNA
XM_015099384.1	SYT14	mRNA	XR_001435726.1	lncRNA
XM_015099384.1	SYT14	mRNA	XR_001435726.1	lncRNA
XM_015099384.1	SYT14	mRNA	XR_001435726.1	lncRNA
XM_015099384.1	SYT14	mRNA	XR_001435726.1	lncRNA
XM_015099384.1	SYT14	mRNA	XR_001435726.1	lncRNA
XM_015099384.1	SYT14	mRNA	XR_001435726.1	lncRNA
XM_015099384.1	SYT14	mRNA	XR_001435726.1	lncRNA
XM_015099384.1	SYT14	mRNA	XR_001435726.1	lncRNA
XM_004008093.2	PTN	mRNA	XR_001042381.2	lncRNA
XM_004008093.2	PTN	mRNA	XR_001042381.2	lncRNA
XM_004008093.2	PTN	mRNA	XR_001042381.2	lncRNA
XM_004008093.2	PTN	mRNA	XR_001042381.2	lncRNA
XM_004008093.2	PTN	mRNA	XR_001042381.2	lncRNA
XM_004008093.2	PTN	mRNA	XR_001042381.2	lncRNA
XM_004008093.2	PTN	mRNA	XR_001042381.2	lncRNA
XM_004008093.2	PTN	mRNA	XR_001042381.2	lncRNA
XM_012134593.2	FASTKD1	mRNA	XR_001042381.2	lncRNA
XM_012134593.2	FASTKD1	mRNA	XR_001042381.2	lncRNA
XM_012134593.2	FASTKD1	mRNA	XR_001042381.2	lncRNA
XM_012134593.2	FASTKD1	mRNA	XR_001042381.2	lncRNA
XM_012134593.2	FASTKD1	mRNA	XR_001042381.2	lncRNA
XM_012134593.2	FASTKD1	mRNA	XR_001042381.2	lncRNA
XM_012134593.2	FASTKD1	mRNA	XR_001042381.2	lncRNA
XM_012134593.2	FASTKD1	mRNA	XR_001042381.2	lncRNA
XM_012098458.2	FANCI	mRNA	XR_001435726.1	lncRNA
XM_012098458.2	FANCI	mRNA	XR_001435726.1	lncRNA
XM_012098458.2	FANCI	mRNA	XR_001435726.1	lncRNA
XM_012098458.2	FANCI	mRNA	XR_001435726.1	lncRNA
XM_012098458.2	FANCI	mRNA	XR_001435726.1	lncRNA
XM_012098458.2	FANCI	mRNA	XR_001435726.1	lncRNA

XM_012098458.2	FANCI	mRNA	XR_001435726.1	lncRNA
XM_012098458.2	FANCI	mRNA	XR_001435726.1	lncRNA
XM_012097468.2	ELF2	mRNA	XR_001042381.2	lncRNA
XM_012097468.2	ELF2	mRNA	XR_001042381.2	lncRNA
XM_012097468.2	ELF2	mRNA	XR_001042381.2	lncRNA
XM_012097468.2	ELF2	mRNA	XR_001042381.2	lncRNA
XM_012097468.2	ELF2	mRNA	XR_001042381.2	lncRNA
XM_012097468.2	ELF2	mRNA	XR_001042381.2	lncRNA
XM_012097468.2	ELF2	mRNA	XR_001042381.2	lncRNA
XM_012097468.2	ELF2	mRNA	XR_001042381.2	lncRNA
XM_012181866.1	GPHN	mRNA	XR_001042381.2	lncRNA
XM_012181866.1	GPHN	mRNA	XR_001042381.2	lncRNA
XM_012181866.1	GPHN	mRNA	XR_001042381.2	lncRNA
XM_012181866.1	GPHN	mRNA	XR_001042381.2	lncRNA
XM_012181866.1	GPHN	mRNA	XR_001042381.2	lncRNA
XM_012181866.1	GPHN	mRNA	XR_001042381.2	lncRNA
XM_012181866.1	GPHN	mRNA	XR_001042381.2	lncRNA
XM_012181866.1	GPHN	mRNA	XR_001042381.2	lncRNA
XM_012182215.2	TDP1	mRNA	TCONS_00029399	lncRNA
XM_012182215.2	TDP1	mRNA	TCONS_00029399	lncRNA
XM_012182215.2	TDP1	mRNA	TCONS_00029399	lncRNA
XM_012182215.2	TDP1	mRNA	TCONS_00029399	lncRNA
XM_012182215.2	TDP1	mRNA	TCONS_00029399	lncRNA
XM_012182215.2	TDP1	mRNA	TCONS_00029399	lncRNA
XM_012182215.2	TDP1	mRNA	TCONS_00029399	lncRNA
XM_012182215.2	TDP1	mRNA	TCONS_00029399	lncRNA
XM_012102669.1	ZNF518A	mRNA	XR_001041209.2	lncRNA
XM_012102669.1	ZNF518A	mRNA	XR_001041209.2	lncRNA
XM_012102669.1	ZNF518A	mRNA	XR_001041209.2	lncRNA
XM_012102669.1	ZNF518A	mRNA	XR_001041209.2	lncRNA
XM_012102669.1	ZNF518A	mRNA	XR_001041209.2	lncRNA
XM_012102669.1	ZNF518A	mRNA	XR_001041209.2	lncRNA
XM_012102669.1	ZNF518A	mRNA	XR_001041209.2	lncRNA
XM_012102669.1	ZNF518A	mRNA	XR_001041209.2	lncRNA
XM_012188917.1	STX16	mRNA	XR_001041209.2	lncRNA
XM_012188917.1	STX16	mRNA	XR_001041209.2	lncRNA
XM_012188917.1	STX16	mRNA	XR_001041209.2	lncRNA
XM_012188917.1	STX16	mRNA	XR_001041209.2	lncRNA
XM_012188917.1	STX16	mRNA	XR_001041209.2	lncRNA
XM_012188917.1	STX16	mRNA	XR_001041209.2	lncRNA
XM_012188917.1	STX16	mRNA	XR_001041209.2	lncRNA
XM_012188917.1	STX16	mRNA	XR_001041209.2	lncRNA
XM_004006510.3	SLC16A7	mRNA	TCONS_00029399	lncRNA
XM_004006510.3	SLC16A7	mRNA	TCONS_00029399	lncRNA
XM_004006510.3	SLC16A7	mRNA	TCONS_00029399	lncRNA
XM_004006510.3	SLC16A7	mRNA	TCONS_00029399	lncRNA
XM_004006510.3	SLC16A7	mRNA	TCONS_00029399	lncRNA
XM_004006510.3	SLC16A7	mRNA	TCONS_00029399	lncRNA
XM_004006510.3	SLC16A7	mRNA	TCONS_00029399	lncRNA
XM_004006510.3	SLC16A7	mRNA	TCONS_00029399	lncRNA
XM_012098990.2	NAALADL2	mRNA	XR_001041209.2	lncRNA
XM_012098990.2	NAALADL2	mRNA	XR_001041209.2	lncRNA
XM_012098990.2	NAALADL2	mRNA	XR_001041209.2	lncRNA
XM_012098990.2	NAALADL2	mRNA	XR_001041209.2	lncRNA

XM_012098990.2	NAALADL2	mRNA	XR_001041209.2	lncRNA
XM_012098990.2	NAALADL2	mRNA	XR_001041209.2	lncRNA
XM_012098990.2	NAALADL2	mRNA	XR_001041209.2	lncRNA
XM_012098990.2	NAALADL2	mRNA	XR_001041209.2	lncRNA
XM_012159370.2	LOC1011215(	mRNA	XR_001041211.2	lncRNA
XM_012159370.2	LOC1011215(	mRNA	XR_001041211.2	lncRNA
XM_012159370.2	LOC1011215(	mRNA	XR_001041211.2	lncRNA
XM_012159370.2	LOC1011215(	mRNA	XR_001041211.2	lncRNA
XM_012159370.2	LOC1011215(	mRNA	XR_001041211.2	lncRNA
XM_012159370.2	LOC1011215(	mRNA	XR_001041211.2	lncRNA
XM_012159370.2	LOC1011215(	mRNA	XR_001041211.2	lncRNA
XM_012159370.2	LOC1011215(	mRNA	XR_001041211.2	lncRNA
XM_015093495.1	NBEAL1	mRNA	XR_001041209.2	lncRNA
XM_015093495.1	NBEAL1	mRNA	XR_001041209.2	lncRNA
XM_015093495.1	NBEAL1	mRNA	XR_001041209.2	lncRNA
XM_015093495.1	NBEAL1	mRNA	XR_001041209.2	lncRNA
XM_015093495.1	NBEAL1	mRNA	XR_001041209.2	lncRNA
XM_015093495.1	NBEAL1	mRNA	XR_001041209.2	lncRNA
XM_015093495.1	NBEAL1	mRNA	XR_001041209.2	lncRNA
XM_015093495.1	NBEAL1	mRNA	XR_001041209.2	lncRNA
XM_004003140.2	NAALADL2	mRNA	XR_001041209.2	lncRNA
XM_004003140.2	NAALADL2	mRNA	XR_001041209.2	lncRNA
XM_004003140.2	NAALADL2	mRNA	XR_001041209.2	lncRNA
XM_004003140.2	NAALADL2	mRNA	XR_001041209.2	lncRNA
XM_004003140.2	NAALADL2	mRNA	XR_001041209.2	lncRNA
XM_004003140.2	NAALADL2	mRNA	XR_001041209.2	lncRNA
XM_004003140.2	NAALADL2	mRNA	XR_001041209.2	lncRNA
XM_004003140.2	NAALADL2	mRNA	XR_001041209.2	lncRNA
XM_012181420.2	ICE2	mRNA	TCONS_00029399	lncRNA
XM_012181420.2	ICE2	mRNA	TCONS_00029399	lncRNA
XM_012181420.2	ICE2	mRNA	TCONS_00029399	lncRNA
XM_012181420.2	ICE2	mRNA	TCONS_00029399	lncRNA
XM_012181420.2	ICE2	mRNA	TCONS_00029399	lncRNA
XM_012181420.2	ICE2	mRNA	TCONS_00029399	lncRNA
XM_012181420.2	ICE2	mRNA	TCONS_00029399	lncRNA
XM_012181420.2	ICE2	mRNA	TCONS_00029399	lncRNA
XM_015101107.1	TCP11L1	mRNA	XR_001041211.2	lncRNA
XM_015101107.1	TCP11L1	mRNA	XR_001041211.2	lncRNA
XM_015101107.1	TCP11L1	mRNA	XR_001041211.2	lncRNA
XM_015101107.1	TCP11L1	mRNA	XR_001041211.2	lncRNA
XM_015101107.1	TCP11L1	mRNA	XR_001041211.2	lncRNA
XM_015101107.1	TCP11L1	mRNA	XR_001041211.2	lncRNA
XM_015101107.1	TCP11L1	mRNA	XR_001041211.2	lncRNA
XM_015101107.1	TCP11L1	mRNA	XR_001041211.2	lncRNA
XM_015096814.1	DENND4A	mRNA	XR_001041209.2	lncRNA
XM_015096814.1	DENND4A	mRNA	XR_001041209.2	lncRNA
XM_015096814.1	DENND4A	mRNA	XR_001041209.2	lncRNA
XM_015096814.1	DENND4A	mRNA	XR_001041209.2	lncRNA
XM_015096814.1	DENND4A	mRNA	XR_001041209.2	lncRNA
XM_015096814.1	DENND4A	mRNA	XR_001041209.2	lncRNA
XM_015096814.1	DENND4A	mRNA	XR_001041209.2	lncRNA
XM_015096814.1	DENND4A	mRNA	XR_001041209.2	lncRNA
XM_015098446.1	VEZF1	mRNA	TCONS_00029399	lncRNA
XM_015098446.1	VEZF1	mRNA	TCONS_00029399	lncRNA

XM_015098446.1	VEZF1	mRNA	TCONS_00029399	lncRNA
XM_015098446.1	VEZF1	mRNA	TCONS_00029399	lncRNA
XM_015098446.1	VEZF1	mRNA	TCONS_00029399	lncRNA
XM_015098446.1	VEZF1	mRNA	TCONS_00029399	lncRNA
XM_015098446.1	VEZF1	mRNA	TCONS_00029399	lncRNA
XM_015098446.1	VEZF1	mRNA	TCONS_00029399	lncRNA
XM_012096185.2	TCP11L1	mRNA	XR_001041211.2	lncRNA
XM_012096185.2	TCP11L1	mRNA	XR_001041211.2	lncRNA
XM_012096185.2	TCP11L1	mRNA	XR_001041211.2	lncRNA
XM_012096185.2	TCP11L1	mRNA	XR_001041211.2	lncRNA
XM_012096185.2	TCP11L1	mRNA	XR_001041211.2	lncRNA
XM_012096185.2	TCP11L1	mRNA	XR_001041211.2	lncRNA
XM_012096185.2	TCP11L1	mRNA	XR_001041211.2	lncRNA
XM_012096185.2	TCP11L1	mRNA	XR_001041211.2	lncRNA
XM_012098990.2	NAALADL2	mRNA	TCONS_00029399	lncRNA
XM_012098990.2	NAALADL2	mRNA	TCONS_00029399	lncRNA
XM_012098990.2	NAALADL2	mRNA	TCONS_00029399	lncRNA
XM_012098990.2	NAALADL2	mRNA	TCONS_00029399	lncRNA
XM_012098990.2	NAALADL2	mRNA	TCONS_00029399	lncRNA
XM_012098990.2	NAALADL2	mRNA	TCONS_00029399	lncRNA
XM_012098990.2	NAALADL2	mRNA	TCONS_00029399	lncRNA
XM_012098990.2	NAALADL2	mRNA	TCONS_00029399	lncRNA
XM_015099551.1	PARD3	mRNA	XR_001041211.2	lncRNA
XM_015099551.1	PARD3	mRNA	XR_001041211.2	lncRNA
XM_015099551.1	PARD3	mRNA	XR_001041211.2	lncRNA
XM_015099551.1	PARD3	mRNA	XR_001041211.2	lncRNA
XM_015099551.1	PARD3	mRNA	XR_001041211.2	lncRNA
XM_015099551.1	PARD3	mRNA	XR_001041211.2	lncRNA
XM_015099551.1	PARD3	mRNA	XR_001041211.2	lncRNA
XM_015099551.1	PARD3	mRNA	XR_001041211.2	lncRNA
XM_012101922.2	ZBTB44	mRNA	TCONS_00029399	lncRNA
XM_012101922.2	ZBTB44	mRNA	TCONS_00029399	lncRNA
XM_012101922.2	ZBTB44	mRNA	TCONS_00029399	lncRNA
XM_012101922.2	ZBTB44	mRNA	TCONS_00029399	lncRNA
XM_012101922.2	ZBTB44	mRNA	TCONS_00029399	lncRNA
XM_012101922.2	ZBTB44	mRNA	TCONS_00029399	lncRNA
XM_012101922.2	ZBTB44	mRNA	TCONS_00029399	lncRNA
XM_012101922.2	ZBTB44	mRNA	TCONS_00029399	lncRNA
XM_004003140.2	NAALADL2	mRNA	TCONS_00029399	lncRNA
XM_004003140.2	NAALADL2	mRNA	TCONS_00029399	lncRNA
XM_004003140.2	NAALADL2	mRNA	TCONS_00029399	lncRNA
XM_004003140.2	NAALADL2	mRNA	TCONS_00029399	lncRNA
XM_004003140.2	NAALADL2	mRNA	TCONS_00029399	lncRNA
XM_004003140.2	NAALADL2	mRNA	TCONS_00029399	lncRNA
XM_004003140.2	NAALADL2	mRNA	TCONS_00029399	lncRNA
XM_004003140.2	NAALADL2	mRNA	TCONS_00029399	lncRNA
XM_012182538.2	TRAPPC3L	mRNA	XR_001041209.2	lncRNA
XM_012182538.2	TRAPPC3L	mRNA	XR_001041209.2	lncRNA
XM_012182538.2	TRAPPC3L	mRNA	XR_001041209.2	lncRNA
XM_012182538.2	TRAPPC3L	mRNA	XR_001041209.2	lncRNA
XM_012182538.2	TRAPPC3L	mRNA	XR_001041209.2	lncRNA
XM_012182538.2	TRAPPC3L	mRNA	XR_001041209.2	lncRNA
XM_012182538.2	TRAPPC3L	mRNA	XR_001041209.2	lncRNA
XM_012182538.2	TRAPPC3L	mRNA	XR_001041209.2	lncRNA

XM_004012194.3	RBM26	mRNA	XR_001021939.1	lncRNA
XM_004012194.3	RBM26	mRNA	XR_001021939.1	lncRNA
XM_004012194.3	RBM26	mRNA	XR_001021939.1	lncRNA
XM_004012194.3	RBM26	mRNA	XR_001021939.1	lncRNA
XM_004012194.3	RBM26	mRNA	XR_001021939.1	lncRNA
XM_004012194.3	RBM26	mRNA	XR_001021939.1	lncRNA
XM_004012194.3	RBM26	mRNA	XR_001021939.1	lncRNA
XM_004012194.3	RBM26	mRNA	XR_001021939.1	lncRNA
XM_015092207.1	PRKAB2	mRNA	XR_001021939.1	lncRNA
XM_015092207.1	PRKAB2	mRNA	XR_001021939.1	lncRNA
XM_015092207.1	PRKAB2	mRNA	XR_001021939.1	lncRNA
XM_015092207.1	PRKAB2	mRNA	XR_001021939.1	lncRNA
XM_015092207.1	PRKAB2	mRNA	XR_001021939.1	lncRNA
XM_015092207.1	PRKAB2	mRNA	XR_001021939.1	lncRNA
XM_015092207.1	PRKAB2	mRNA	XR_001021939.1	lncRNA
XM_015092207.1	PRKAB2	mRNA	XR_001021939.1	lncRNA
XM_012106710.2	CLCN5	mRNA	XR_001021689.2	lncRNA
XM_012106710.2	CLCN5	mRNA	XR_001021689.2	lncRNA
XM_012106710.2	CLCN5	mRNA	XR_001021689.2	lncRNA
XM_012106710.2	CLCN5	mRNA	XR_001021689.2	lncRNA
XM_012106710.2	CLCN5	mRNA	XR_001021689.2	lncRNA
XM_012106710.2	CLCN5	mRNA	XR_001021689.2	lncRNA
XM_012106710.2	CLCN5	mRNA	XR_001021689.2	lncRNA
XM_012106710.2	CLCN5	mRNA	XR_001021689.2	lncRNA
XM_012097252.2	RHOF	mRNA	XR_001021689.2	lncRNA
XM_012097252.2	RHOF	mRNA	XR_001021689.2	lncRNA
XM_012097252.2	RHOF	mRNA	XR_001021689.2	lncRNA
XM_012097252.2	RHOF	mRNA	XR_001021689.2	lncRNA
XM_012097252.2	RHOF	mRNA	XR_001021689.2	lncRNA
XM_012097252.2	RHOF	mRNA	XR_001021689.2	lncRNA
XM_012097252.2	RHOF	mRNA	XR_001021689.2	lncRNA
XM_012097252.2	RHOF	mRNA	XR_001021689.2	lncRNA
XM_012096035.1	C2CD3	mRNA	XR_001436099.1	lncRNA
XM_012096035.1	C2CD3	mRNA	XR_001436099.1	lncRNA
XM_012096035.1	C2CD3	mRNA	XR_001436099.1	lncRNA
XM_012096035.1	C2CD3	mRNA	XR_001436099.1	lncRNA
XM_012096035.1	C2CD3	mRNA	XR_001436099.1	lncRNA
XM_012096035.1	C2CD3	mRNA	XR_001436099.1	lncRNA
XM_012096035.1	C2CD3	mRNA	XR_001436099.1	lncRNA
XM_012096035.1	C2CD3	mRNA	XR_001436099.1	lncRNA
XM_012187548.2	GIGYF2	mRNA	XR_001033284.2	lncRNA
XM_012187548.2	GIGYF2	mRNA	XR_001033284.2	lncRNA
XM_012187548.2	GIGYF2	mRNA	XR_001033284.2	lncRNA
XM_012187548.2	GIGYF2	mRNA	XR_001033284.2	lncRNA
XM_012187548.2	GIGYF2	mRNA	XR_001033284.2	lncRNA
XM_012187548.2	GIGYF2	mRNA	XR_001033284.2	lncRNA
XM_012187548.2	GIGYF2	mRNA	XR_001033284.2	lncRNA
XM_012187548.2	GIGYF2	mRNA	XR_001033284.2	lncRNA
XM_012190105.1	ANKRL17	mRNA	XR_001041202.1	lncRNA
XM_012190105.1	ANKRL17	mRNA	XR_001041202.1	lncRNA
XM_012190105.1	ANKRL17	mRNA	XR_001041202.1	lncRNA
XM_012190105.1	ANKRL17	mRNA	XR_001041202.1	lncRNA
XM_012190105.1	ANKRL17	mRNA	XR_001041202.1	lncRNA
XM_012190105.1	ANKRL17	mRNA	XR_001041202.1	lncRNA

XM_012190105.1	ANKRL17	mRNA	XR_001041202.1	lncRNA
XM_012176656.2	NAPEPLD	mRNA	XR_001041202.1	lncRNA
XM_012176656.2	NAPEPLD	mRNA	XR_001041202.1	lncRNA
XM_012176656.2	NAPEPLD	mRNA	XR_001041202.1	lncRNA
XM_012176656.2	NAPEPLD	mRNA	XR_001041202.1	lncRNA
XM_012176656.2	NAPEPLD	mRNA	XR_001041202.1	lncRNA
XM_012176656.2	NAPEPLD	mRNA	XR_001041202.1	lncRNA
XM_012176656.2	NAPEPLD	mRNA	XR_001041202.1	lncRNA
XM_004006510.3	SLC16A7	mRNA	XR_001434081.1	lncRNA
XM_004006510.3	SLC16A7	mRNA	XR_001434081.1	lncRNA
XM_004006510.3	SLC16A7	mRNA	XR_001434081.1	lncRNA
XM_004006510.3	SLC16A7	mRNA	XR_001434081.1	lncRNA
XM_004006510.3	SLC16A7	mRNA	XR_001434081.1	lncRNA
XM_004006510.3	SLC16A7	mRNA	XR_001434081.1	lncRNA
XM_004006510.3	SLC16A7	mRNA	XR_001434081.1	lncRNA
XM_004006510.3	SLC16A7	mRNA	XR_001434081.1	lncRNA
XM_012181829.2	ZBTB1	mRNA	XR_001434081.1	lncRNA
XM_012181829.2	ZBTB1	mRNA	XR_001434081.1	lncRNA
XM_012181829.2	ZBTB1	mRNA	XR_001434081.1	lncRNA
XM_012181829.2	ZBTB1	mRNA	XR_001434081.1	lncRNA
XM_012181829.2	ZBTB1	mRNA	XR_001434081.1	lncRNA
XM_012181829.2	ZBTB1	mRNA	XR_001434081.1	lncRNA
XM_012181829.2	ZBTB1	mRNA	XR_001434081.1	lncRNA
XM_012181829.2	ZBTB1	mRNA	XR_001434081.1	lncRNA
XM_012103019.2	INPP5F	mRNA	XR_001434081.1	lncRNA
XM_012103019.2	INPP5F	mRNA	XR_001434081.1	lncRNA
XM_012103019.2	INPP5F	mRNA	XR_001434081.1	lncRNA
XM_012103019.2	INPP5F	mRNA	XR_001434081.1	lncRNA
XM_012103019.2	INPP5F	mRNA	XR_001434081.1	lncRNA
XM_012103019.2	INPP5F	mRNA	XR_001434081.1	lncRNA
XM_012103019.2	INPP5F	mRNA	XR_001434081.1	lncRNA
XM_012103019.2	INPP5F	mRNA	XR_001434081.1	lncRNA
XM_012179952.2	FRYL	mRNA	XR_001434081.1	lncRNA
XM_012179952.2	FRYL	mRNA	XR_001434081.1	lncRNA
XM_012179952.2	FRYL	mRNA	XR_001434081.1	lncRNA
XM_012179952.2	FRYL	mRNA	XR_001434081.1	lncRNA
XM_012179952.2	FRYL	mRNA	XR_001434081.1	lncRNA
XM_012179952.2	FRYL	mRNA	XR_001434081.1	lncRNA
XM_012179952.2	FRYL	mRNA	XR_001434081.1	lncRNA
XM_012179952.2	FRYL	mRNA	XR_001434081.1	lncRNA
XM_012105316.2	USP54	mRNA	XR_001436609.1	lncRNA
XM_012105316.2	USP54	mRNA	XR_001436609.1	lncRNA
XM_012105316.2	USP54	mRNA	XR_001436609.1	lncRNA
XM_012105316.2	USP54	mRNA	XR_001436609.1	lncRNA
XM_012105316.2	USP54	mRNA	XR_001436609.1	lncRNA
XM_012105316.2	USP54	mRNA	XR_001436609.1	lncRNA
XM_012105316.2	USP54	mRNA	XR_001436609.1	lncRNA
XM_012105316.2	USP54	mRNA	XR_001436609.1	lncRNA
XM_012190630.2	PLEKHA4	mRNA	XR_001435104.1	lncRNA
XM_012190630.2	PLEKHA4	mRNA	XR_001435104.1	lncRNA
XM_012190630.2	PLEKHA4	mRNA	XR_001435104.1	lncRNA
XM_012190630.2	PLEKHA4	mRNA	XR_001435104.1	lncRNA
XM_012190630.2	PLEKHA4	mRNA	XR_001435104.1	lncRNA
XM_012190630.2	PLEKHA4	mRNA	XR_001435104.1	lncRNA

XM_012190630.2	PLEKHA4	mRNA	XR_001435104.1	lncRNA
XM_012190630.2	PLEKHA4	mRNA	XR_001435104.1	lncRNA
XM_012186340.1	POLG2	mRNA	XR_001436609.1	lncRNA
XM_012186340.1	POLG2	mRNA	XR_001436609.1	lncRNA
XM_012186340.1	POLG2	mRNA	XR_001436609.1	lncRNA
XM_012186340.1	POLG2	mRNA	XR_001436609.1	lncRNA
XM_012186340.1	POLG2	mRNA	XR_001436609.1	lncRNA
XM_012186340.1	POLG2	mRNA	XR_001436609.1	lncRNA
XM_012186340.1	POLG2	mRNA	XR_001436609.1	lncRNA
XM_012186340.1	POLG2	mRNA	XR_001436609.1	lncRNA
XM_012186340.1	POLG2	mRNA	XR_001436531.1	lncRNA
XM_012186340.1	POLG2	mRNA	XR_001436531.1	lncRNA
XM_012186340.1	POLG2	mRNA	XR_001436531.1	lncRNA
XM_012186340.1	POLG2	mRNA	XR_001436531.1	lncRNA
XM_012186340.1	POLG2	mRNA	XR_001436531.1	lncRNA
XM_012186340.1	POLG2	mRNA	XR_001436531.1	lncRNA
XM_012186340.1	POLG2	mRNA	XR_001436531.1	lncRNA
XM_012186340.1	POLG2	mRNA	XR_001436531.1	lncRNA
XM_012142889.2	ICA1L	mRNA	XR_001435104.1	lncRNA
XM_012142889.2	ICA1L	mRNA	XR_001435104.1	lncRNA
XM_012142889.2	ICA1L	mRNA	XR_001435104.1	lncRNA
XM_012142889.2	ICA1L	mRNA	XR_001435104.1	lncRNA
XM_012142889.2	ICA1L	mRNA	XR_001435104.1	lncRNA
XM_012142889.2	ICA1L	mRNA	XR_001435104.1	lncRNA
XM_012142889.2	ICA1L	mRNA	XR_001435104.1	lncRNA
XM_012142889.2	ICA1L	mRNA	XR_001435104.1	lncRNA
XM_004012194.3	RBM26	mRNA	XR_001436609.1	lncRNA
XM_004012194.3	RBM26	mRNA	XR_001436609.1	lncRNA
XM_004012194.3	RBM26	mRNA	XR_001436609.1	lncRNA
XM_004012194.3	RBM26	mRNA	XR_001436609.1	lncRNA
XM_004012194.3	RBM26	mRNA	XR_001436609.1	lncRNA
XM_004012194.3	RBM26	mRNA	XR_001436609.1	lncRNA
XM_004012194.3	RBM26	mRNA	XR_001436609.1	lncRNA
XM_004012194.3	RBM26	mRNA	XR_001436609.1	lncRNA
XM_012180332.2	GSK3B	mRNA	XR_001436609.1	lncRNA
XM_012180332.2	GSK3B	mRNA	XR_001436609.1	lncRNA
XM_012180332.2	GSK3B	mRNA	XR_001436609.1	lncRNA
XM_012180332.2	GSK3B	mRNA	XR_001436609.1	lncRNA
XM_012180332.2	GSK3B	mRNA	XR_001436609.1	lncRNA
XM_012180332.2	GSK3B	mRNA	XR_001436609.1	lncRNA
XM_012180332.2	GSK3B	mRNA	XR_001436609.1	lncRNA
XM_012180332.2	GSK3B	mRNA	XR_001436609.1	lncRNA
XM_012190630.2	PLEKHA4	mRNA	XR_001436609.1	lncRNA
XM_012190630.2	PLEKHA4	mRNA	XR_001436609.1	lncRNA
XM_012190630.2	PLEKHA4	mRNA	XR_001436609.1	lncRNA
XM_012190630.2	PLEKHA4	mRNA	XR_001436609.1	lncRNA
XM_012190630.2	PLEKHA4	mRNA	XR_001436609.1	lncRNA
XM_012190630.2	PLEKHA4	mRNA	XR_001436609.1	lncRNA
XM_012190630.2	PLEKHA4	mRNA	XR_001436609.1	lncRNA
XM_012190630.2	PLEKHA4	mRNA	XR_001436609.1	lncRNA
XM_012140422.2	CLASP1	mRNA	XR_001033284.2	lncRNA
XM_012140422.2	CLASP1	mRNA	XR_001033284.2	lncRNA
XM_012140422.2	CLASP1	mRNA	XR_001033284.2	lncRNA
XM_012140422.2	CLASP1	mRNA	XR_001033284.2	lncRNA

XM_012140422.2	CLASP1	mRNA	XR_001033284.2	lncRNA
XM_012140422.2	CLASP1	mRNA	XR_001033284.2	lncRNA
XM_012140422.2	CLASP1	mRNA	XR_001033284.2	lncRNA
XM_012140422.2	CLASP1	mRNA	XR_001033284.2	lncRNA
XM_015102310.1	SETD5	mRNA	XR_001033284.2	lncRNA
XM_015102310.1	SETD5	mRNA	XR_001033284.2	lncRNA
XM_015102310.1	SETD5	mRNA	XR_001033284.2	lncRNA
XM_015102310.1	SETD5	mRNA	XR_001033284.2	lncRNA
XM_015102310.1	SETD5	mRNA	XR_001033284.2	lncRNA
XM_015102310.1	SETD5	mRNA	XR_001033284.2	lncRNA
XM_015102310.1	SETD5	mRNA	XR_001033284.2	lncRNA
XM_015102310.1	SETD5	mRNA	XR_001033284.2	lncRNA
XM_012101112.2	ABT1	mRNA	TCONS_00029399	lncRNA
XM_012101112.2	ABT1	mRNA	TCONS_00029399	lncRNA
XM_012101112.2	ABT1	mRNA	TCONS_00029399	lncRNA
XM_012101112.2	ABT1	mRNA	TCONS_00029399	lncRNA
XM_012101112.2	ABT1	mRNA	TCONS_00029399	lncRNA
XM_012101112.2	ABT1	mRNA	TCONS_00029399	lncRNA
XM_012101112.2	ABT1	mRNA	TCONS_00029399	lncRNA
XM_012101112.2	ABT1	mRNA	TCONS_00029399	lncRNA
XM_012122525.2	PRUNE2	mRNA	XR_001041211.2	lncRNA
XM_012122525.2	PRUNE2	mRNA	XR_001041211.2	lncRNA
XM_012122525.2	PRUNE2	mRNA	XR_001041211.2	lncRNA
XM_012122525.2	PRUNE2	mRNA	XR_001041211.2	lncRNA
XM_012122525.2	PRUNE2	mRNA	XR_001041211.2	lncRNA
XM_012122525.2	PRUNE2	mRNA	XR_001041211.2	lncRNA
XM_012122525.2	PRUNE2	mRNA	XR_001041211.2	lncRNA
XM_012122525.2	PRUNE2	mRNA	XR_001041211.2	lncRNA
XM_012182854.2	PTPRK	mRNA	XR_001041209.2	lncRNA
XM_012182854.2	PTPRK	mRNA	XR_001041209.2	lncRNA
XM_012182854.2	PTPRK	mRNA	XR_001041209.2	lncRNA
XM_012182854.2	PTPRK	mRNA	XR_001041209.2	lncRNA
XM_012182854.2	PTPRK	mRNA	XR_001041209.2	lncRNA
XM_012182854.2	PTPRK	mRNA	XR_001041209.2	lncRNA
XM_012182854.2	PTPRK	mRNA	XR_001041209.2	lncRNA
XM_012182854.2	PTPRK	mRNA	XR_001041209.2	lncRNA
XM_012173782.2	REV1	mRNA	XR_001041211.2	lncRNA
XM_012173782.2	REV1	mRNA	XR_001041211.2	lncRNA
XM_012173782.2	REV1	mRNA	XR_001041211.2	lncRNA
XM_012173782.2	REV1	mRNA	XR_001041211.2	lncRNA
XM_012173782.2	REV1	mRNA	XR_001041211.2	lncRNA
XM_012173782.2	REV1	mRNA	XR_001041211.2	lncRNA
XM_012173782.2	REV1	mRNA	XR_001041211.2	lncRNA
XM_012173782.2	REV1	mRNA	XR_001041211.2	lncRNA
XM_012187548.2	GIGYF2	mRNA	TCONS_00029399	lncRNA
XM_012187548.2	GIGYF2	mRNA	TCONS_00029399	lncRNA
XM_012187548.2	GIGYF2	mRNA	TCONS_00029399	lncRNA
XM_012187548.2	GIGYF2	mRNA	TCONS_00029399	lncRNA
XM_012187548.2	GIGYF2	mRNA	TCONS_00029399	lncRNA
XM_012187548.2	GIGYF2	mRNA	TCONS_00029399	lncRNA
XM_012187548.2	GIGYF2	mRNA	TCONS_00029399	lncRNA
XM_012187548.2	GIGYF2	mRNA	TCONS_00029399	lncRNA
XM_012187063.2	MARK1	mRNA	XR_001042381.2	lncRNA
XM_012187063.2	MARK1	mRNA	XR_001042381.2	lncRNA

XM_012187063.2	MARK1	mRNA	XR_001042381.2	lncRNA
XM_012187063.2	MARK1	mRNA	XR_001042381.2	lncRNA
XM_012187063.2	MARK1	mRNA	XR_001042381.2	lncRNA
XM_012187063.2	MARK1	mRNA	XR_001042381.2	lncRNA
XM_012187063.2	MARK1	mRNA	XR_001042381.2	lncRNA
XM_012187063.2	MARK1	mRNA	XR_001042381.2	lncRNA
XM_012174897.2	UTP20	mRNA	XR_001021939.1	lncRNA
XM_012174897.2	UTP20	mRNA	XR_001021939.1	lncRNA
XM_012174897.2	UTP20	mRNA	XR_001021939.1	lncRNA
XM_012174897.2	UTP20	mRNA	XR_001021939.1	lncRNA
XM_012174897.2	UTP20	mRNA	XR_001021939.1	lncRNA
XM_012174897.2	UTP20	mRNA	XR_001021939.1	lncRNA
XM_012174897.2	UTP20	mRNA	XR_001021939.1	lncRNA
XM_012174897.2	UTP20	mRNA	XR_001021939.1	lncRNA
XM_015102914.1	NAV2	mRNA	XR_001033284.2	lncRNA
XM_015102914.1	NAV2	mRNA	XR_001033284.2	lncRNA
XM_015102914.1	NAV2	mRNA	XR_001033284.2	lncRNA
XM_015102914.1	NAV2	mRNA	XR_001033284.2	lncRNA
XM_015102914.1	NAV2	mRNA	XR_001033284.2	lncRNA
XM_015102914.1	NAV2	mRNA	XR_001033284.2	lncRNA
XM_015102914.1	NAV2	mRNA	XR_001033284.2	lncRNA
XM_015102914.1	NAV2	mRNA	XR_001033284.2	lncRNA
XM_012173782.2	REV1	mRNA	XR_001434081.1	lncRNA
XM_012173782.2	REV1	mRNA	XR_001434081.1	lncRNA
XM_012173782.2	REV1	mRNA	XR_001434081.1	lncRNA
XM_012173782.2	REV1	mRNA	XR_001434081.1	lncRNA
XM_012173782.2	REV1	mRNA	XR_001434081.1	lncRNA
XM_012173782.2	REV1	mRNA	XR_001434081.1	lncRNA
XM_012173782.2	REV1	mRNA	XR_001434081.1	lncRNA
XM_012173782.2	REV1	mRNA	XR_001434081.1	lncRNA
XM_012101112.2	ABT1	mRNA	XR_001434081.1	lncRNA
XM_012101112.2	ABT1	mRNA	XR_001434081.1	lncRNA
XM_012101112.2	ABT1	mRNA	XR_001434081.1	lncRNA
XM_012101112.2	ABT1	mRNA	XR_001434081.1	lncRNA
XM_012101112.2	ABT1	mRNA	XR_001434081.1	lncRNA
XM_012101112.2	ABT1	mRNA	XR_001434081.1	lncRNA
XM_012101112.2	ABT1	mRNA	XR_001434081.1	lncRNA
XM_012101112.2	ABT1	mRNA	XR_001434081.1	lncRNA
XM_012137673.2	NEB	mRNA	XR_001041209.2	lncRNA
XM_012137673.2	NEB	mRNA	XR_001041209.2	lncRNA
XM_012137673.2	NEB	mRNA	XR_001041209.2	lncRNA
XM_012137673.2	NEB	mRNA	XR_001041209.2	lncRNA
XM_012137673.2	NEB	mRNA	XR_001041209.2	lncRNA
XM_012137673.2	NEB	mRNA	XR_001041209.2	lncRNA
XM_012137673.2	NEB	mRNA	XR_001041209.2	lncRNA
XM_012137673.2	NEB	mRNA	XR_001041209.2	lncRNA
XM_012144336.2	PIKFYVE	mRNA	TCONS_00029399	lncRNA
XM_012144336.2	PIKFYVE	mRNA	TCONS_00029399	lncRNA
XM_012144336.2	PIKFYVE	mRNA	TCONS_00029399	lncRNA
XM_012144336.2	PIKFYVE	mRNA	TCONS_00029399	lncRNA
XM_012144336.2	PIKFYVE	mRNA	TCONS_00029399	lncRNA
XM_012144336.2	PIKFYVE	mRNA	TCONS_00029399	lncRNA
XM_012144336.2	PIKFYVE	mRNA	TCONS_00029399	lncRNA
XM_012144336.2	PIKFYVE	mRNA	TCONS_00029399	lncRNA

XM_012104178.2	CCP110	mRNA	TCONS_00029399	lncRNA
XM_012104178.2	CCP110	mRNA	TCONS_00029399	lncRNA
XM_012104178.2	CCP110	mRNA	TCONS_00029399	lncRNA
XM_012104178.2	CCP110	mRNA	TCONS_00029399	lncRNA
XM_012104178.2	CCP110	mRNA	TCONS_00029399	lncRNA
XM_012104178.2	CCP110	mRNA	TCONS_00029399	lncRNA
XM_012104178.2	CCP110	mRNA	TCONS_00029399	lncRNA
XM_012104178.2	CCP110	mRNA	TCONS_00029399	lncRNA
XM_012179641.2	LOC1011228(	mRNA	XR_001041209.2	lncRNA
XM_012179641.2	LOC1011228(	mRNA	XR_001041209.2	lncRNA
XM_012179641.2	LOC1011228(	mRNA	XR_001041209.2	lncRNA
XM_012179641.2	LOC1011228(	mRNA	XR_001041209.2	lncRNA
XM_012179641.2	LOC1011228(	mRNA	XR_001041209.2	lncRNA
XM_012179641.2	LOC1011228(	mRNA	XR_001041209.2	lncRNA
XM_012179641.2	LOC1011228(	mRNA	XR_001041209.2	lncRNA
XM_012179641.2	LOC1011228(	mRNA	TCONS_00029399	lncRNA
XM_012179641.2	LOC1011228(	mRNA	TCONS_00029399	lncRNA
XM_012179641.2	LOC1011228(	mRNA	TCONS_00029399	lncRNA
XM_012179641.2	LOC1011228(	mRNA	TCONS_00029399	lncRNA
XM_012179641.2	LOC1011228(	mRNA	TCONS_00029399	lncRNA
XM_012179641.2	LOC1011228(	mRNA	TCONS_00029399	lncRNA
XM_012179641.2	LOC1011228(	mRNA	TCONS_00029399	lncRNA
XM_004022347.2	TMEM255A	mRNA	XR_001041211.2	lncRNA
XM_004022347.2	TMEM255A	mRNA	XR_001041211.2	lncRNA
XM_004022347.2	TMEM255A	mRNA	XR_001041211.2	lncRNA
XM_004022347.2	TMEM255A	mRNA	XR_001041211.2	lncRNA
XM_004022347.2	TMEM255A	mRNA	XR_001041211.2	lncRNA
XM_004022347.2	TMEM255A	mRNA	XR_001041211.2	lncRNA
XM_004022347.2	TMEM255A	mRNA	XR_001041211.2	lncRNA
XM_015098446.1	VEZF1	mRNA	XR_001435104.1	lncRNA
XM_015098446.1	VEZF1	mRNA	XR_001435104.1	lncRNA
XM_015098446.1	VEZF1	mRNA	XR_001435104.1	lncRNA
XM_015098446.1	VEZF1	mRNA	XR_001435104.1	lncRNA
XM_015098446.1	VEZF1	mRNA	XR_001435104.1	lncRNA
XM_015098446.1	VEZF1	mRNA	XR_001435104.1	lncRNA
XM_015098446.1	VEZF1	mRNA	XR_001435104.1	lncRNA
XM_015098446.1	VEZF1	mRNA	XR_001435104.1	lncRNA
XM_012181829.2	ZBTB1	mRNA	XR_001436609.1	lncRNA
XM_012181829.2	ZBTB1	mRNA	XR_001436609.1	lncRNA
XM_012181829.2	ZBTB1	mRNA	XR_001436609.1	lncRNA
XM_012181829.2	ZBTB1	mRNA	XR_001436609.1	lncRNA
XM_012181829.2	ZBTB1	mRNA	XR_001436609.1	lncRNA
XM_012181829.2	ZBTB1	mRNA	XR_001436609.1	lncRNA
XM_012181829.2	ZBTB1	mRNA	XR_001436609.1	lncRNA
XM_012181829.2	ZBTB1	mRNA	XR_001436609.1	lncRNA
XM_012182238.1	SPG11	mRNA	XR_001435104.1	lncRNA
XM_012182238.1	SPG11	mRNA	XR_001435104.1	lncRNA
XM_012182238.1	SPG11	mRNA	XR_001435104.1	lncRNA
XM_012182238.1	SPG11	mRNA	XR_001435104.1	lncRNA
XM_012182238.1	SPG11	mRNA	XR_001435104.1	lncRNA
XM_012182238.1	SPG11	mRNA	XR_001435104.1	lncRNA
XM_012182238.1	SPG11	mRNA	XR_001435104.1	lncRNA
XM_012182238.1	SPG11	mRNA	XR_001435104.1	lncRNA
XM_015096814.1	DENND4A	mRNA	XR_001436609.1	lncRNA

XM_015096814.1	DENND4A	mRNA	XR_001436609.1	lncRNA
XM_015096814.1	DENND4A	mRNA	XR_001436609.1	lncRNA
XM_015096814.1	DENND4A	mRNA	XR_001436609.1	lncRNA
XM_015096814.1	DENND4A	mRNA	XR_001436609.1	lncRNA
XM_015096814.1	DENND4A	mRNA	XR_001436609.1	lncRNA
XM_015096814.1	DENND4A	mRNA	XR_001436609.1	lncRNA
XM_015096814.1	DENND4A	mRNA	XR_001436609.1	lncRNA
XM_015101485.1	LOC1069916	mRNA	XR_001436531.1	lncRNA
XM_015101485.1	LOC1069916	mRNA	XR_001436531.1	lncRNA
XM_015101485.1	LOC1069916	mRNA	XR_001436531.1	lncRNA
XM_015101485.1	LOC1069916	mRNA	XR_001436531.1	lncRNA
XM_015101485.1	LOC1069916	mRNA	XR_001436531.1	lncRNA
XM_015101485.1	LOC1069916	mRNA	XR_001436531.1	lncRNA
XM_015101485.1	LOC1069916	mRNA	XR_001436531.1	lncRNA
XM_015101485.1	LOC1069916	mRNA	XR_001436531.1	lncRNA
XM_012099916.2	PRKCD	mRNA	XR_001435104.1	lncRNA
XM_012099916.2	PRKCD	mRNA	XR_001435104.1	lncRNA
XM_012099916.2	PRKCD	mRNA	XR_001435104.1	lncRNA
XM_012099916.2	PRKCD	mRNA	XR_001435104.1	lncRNA
XM_012099916.2	PRKCD	mRNA	XR_001435104.1	lncRNA
XM_012099916.2	PRKCD	mRNA	XR_001435104.1	lncRNA
XM_012099916.2	PRKCD	mRNA	XR_001435104.1	lncRNA
XM_012099916.2	PRKCD	mRNA	XR_001435104.1	lncRNA
XM_012099916.2	PRKCD	mRNA	XR_001436609.1	lncRNA
XM_012099916.2	PRKCD	mRNA	XR_001436609.1	lncRNA
XM_012099916.2	PRKCD	mRNA	XR_001436609.1	lncRNA
XM_012099916.2	PRKCD	mRNA	XR_001436609.1	lncRNA
XM_012099916.2	PRKCD	mRNA	XR_001436609.1	lncRNA
XM_012099916.2	PRKCD	mRNA	XR_001436609.1	lncRNA
XM_012099916.2	PRKCD	mRNA	XR_001436609.1	lncRNA
XM_012099916.2	PRKCD	mRNA	XR_001436609.1	lncRNA
XM_012102077.2	LGALS12	mRNA	XR_001435104.1	lncRNA
XM_012102077.2	LGALS12	mRNA	XR_001435104.1	lncRNA
XM_012102077.2	LGALS12	mRNA	XR_001435104.1	lncRNA
XM_012102077.2	LGALS12	mRNA	XR_001435104.1	lncRNA
XM_012102077.2	LGALS12	mRNA	XR_001435104.1	lncRNA
XM_012102077.2	LGALS12	mRNA	XR_001435104.1	lncRNA
XM_012102077.2	LGALS12	mRNA	XR_001435104.1	lncRNA
XM_012102077.2	LGALS12	mRNA	XR_001435104.1	lncRNA
XM_012181854.1	GPHN	mRNA	XR_001041202.1	lncRNA
XM_012181854.1	GPHN	mRNA	XR_001041202.1	lncRNA
XM_012181854.1	GPHN	mRNA	XR_001041202.1	lncRNA
XM_012181854.1	GPHN	mRNA	XR_001041202.1	lncRNA
XM_012181854.1	GPHN	mRNA	XR_001041202.1	lncRNA
XM_012181854.1	GPHN	mRNA	XR_001041202.1	lncRNA
XM_012181854.1	GPHN	mRNA	XR_001041202.1	lncRNA
XM_015098446.1	VEZF1	mRNA	TCONS_00029398	lncRNA
XM_015098446.1	VEZF1	mRNA	TCONS_00029398	lncRNA
XM_015098446.1	VEZF1	mRNA	TCONS_00029398	lncRNA
XM_015098446.1	VEZF1	mRNA	TCONS_00029398	lncRNA
XM_015098446.1	VEZF1	mRNA	TCONS_00029398	lncRNA
XM_015098446.1	VEZF1	mRNA	TCONS_00029398	lncRNA
XM_015098446.1	VEZF1	mRNA	TCONS_00029398	lncRNA
XM_012177560.2	SLC26A2	mRNA	XR_001021689.2	lncRNA

XM_012177560.2	SLC26A2	mRNA	XR_001021689.2	lncRNA
XM_012177560.2	SLC26A2	mRNA	XR_001021689.2	lncRNA
XM_012177560.2	SLC26A2	mRNA	XR_001021689.2	lncRNA
XM_012177560.2	SLC26A2	mRNA	XR_001021689.2	lncRNA
XM_012177560.2	SLC26A2	mRNA	XR_001021689.2	lncRNA
XM_012177560.2	SLC26A2	mRNA	XR_001021689.2	lncRNA
XM_012180332.2	GSK3B	mRNA	XR_001021689.2	lncRNA
XM_012180332.2	GSK3B	mRNA	XR_001021689.2	lncRNA
XM_012180332.2	GSK3B	mRNA	XR_001021689.2	lncRNA
XM_012180332.2	GSK3B	mRNA	XR_001021689.2	lncRNA
XM_012180332.2	GSK3B	mRNA	XR_001021689.2	lncRNA
XM_012180332.2	GSK3B	mRNA	XR_001021689.2	lncRNA
XM_012180332.2	GSK3B	mRNA	XR_001021689.2	lncRNA
XM_012178641.2	CTTNBP2NL	mRNA	TCONS_00071561	lncRNA
XM_012178641.2	CTTNBP2NL	mRNA	TCONS_00071561	lncRNA
XM_012178641.2	CTTNBP2NL	mRNA	TCONS_00071561	lncRNA
XM_012178641.2	CTTNBP2NL	mRNA	TCONS_00071561	lncRNA
XM_012178641.2	CTTNBP2NL	mRNA	TCONS_00071561	lncRNA
XM_012178641.2	CTTNBP2NL	mRNA	TCONS_00071561	lncRNA
XM_012178641.2	CTTNBP2NL	mRNA	TCONS_00071561	lncRNA
XM_012176365.2	SLC25A13	mRNA	XR_001021939.1	lncRNA
XM_012176365.2	SLC25A13	mRNA	XR_001021939.1	lncRNA
XM_012176365.2	SLC25A13	mRNA	XR_001021939.1	lncRNA
XM_012176365.2	SLC25A13	mRNA	XR_001021939.1	lncRNA
XM_012176365.2	SLC25A13	mRNA	XR_001021939.1	lncRNA
XM_012176365.2	SLC25A13	mRNA	XR_001021939.1	lncRNA
XM_012176365.2	SLC25A13	mRNA	XR_001021939.1	lncRNA
XM_012176365.2	SLC25A13	mRNA	XR_001021939.1	lncRNA
XM_012188917.1	STX16	mRNA	XR_001042381.2	lncRNA
XM_012188917.1	STX16	mRNA	XR_001042381.2	lncRNA
XM_012188917.1	STX16	mRNA	XR_001042381.2	lncRNA
XM_012188917.1	STX16	mRNA	XR_001042381.2	lncRNA
XM_012188917.1	STX16	mRNA	XR_001042381.2	lncRNA
XM_012188917.1	STX16	mRNA	XR_001042381.2	lncRNA
XM_012188917.1	STX16	mRNA	XR_001042381.2	lncRNA
XM_012188917.1	STX16	mRNA	XR_001042381.2	lncRNA
XM_012181829.2	ZBTB1	mRNA	XR_001435726.1	lncRNA
XM_012181829.2	ZBTB1	mRNA	XR_001435726.1	lncRNA
XM_012181829.2	ZBTB1	mRNA	XR_001435726.1	lncRNA
XM_012181829.2	ZBTB1	mRNA	XR_001435726.1	lncRNA
XM_012181829.2	ZBTB1	mRNA	XR_001435726.1	lncRNA
XM_012181829.2	ZBTB1	mRNA	XR_001435726.1	lncRNA
XM_012181829.2	ZBTB1	mRNA	XR_001435726.1	lncRNA
XM_012181829.2	ZBTB1	mRNA	XR_001435726.1	lncRNA
XM_012181420.2	ICE2	mRNA	XR_001435723.1	lncRNA
XM_012181420.2	ICE2	mRNA	XR_001435723.1	lncRNA
XM_012181420.2	ICE2	mRNA	XR_001435723.1	lncRNA
XM_012181420.2	ICE2	mRNA	XR_001435723.1	lncRNA
XM_012181420.2	ICE2	mRNA	XR_001435723.1	lncRNA
XM_012181420.2	ICE2	mRNA	XR_001435723.1	lncRNA
XM_012181420.2	ICE2	mRNA	XR_001435723.1	lncRNA
XM_012181420.2	ICE2	mRNA	XR_001435723.1	lncRNA
XM_015091887.1	MYCL	mRNA	TCONS_00029399	lncRNA
XM_015091887.1	MYCL	mRNA	TCONS_00029399	lncRNA

XM_015091887.1	MYCL	mRNA	TCONS_00029399	lncRNA
XM_015091887.1	MYCL	mRNA	TCONS_00029399	lncRNA
XM_015091887.1	MYCL	mRNA	TCONS_00029399	lncRNA
XM_015091887.1	MYCL	mRNA	TCONS_00029399	lncRNA
XM_015091887.1	MYCL	mRNA	TCONS_00029399	lncRNA
XM_015091887.1	MYCL	mRNA	TCONS_00029399	lncRNA
XM_012187340.2	KIF1B	mRNA	XR_001041209.2	lncRNA
XM_012187340.2	KIF1B	mRNA	XR_001041209.2	lncRNA
XM_012187340.2	KIF1B	mRNA	XR_001041209.2	lncRNA
XM_012187340.2	KIF1B	mRNA	XR_001041209.2	lncRNA
XM_012187340.2	KIF1B	mRNA	XR_001041209.2	lncRNA
XM_012187340.2	KIF1B	mRNA	XR_001041209.2	lncRNA
XM_012187340.2	KIF1B	mRNA	XR_001041209.2	lncRNA
XM_012187340.2	KIF1B	mRNA	XR_001041209.2	lncRNA
XM_015093632.1	EPB41	mRNA	TCONS_00029398	lncRNA
XM_015093632.1	EPB41	mRNA	TCONS_00029398	lncRNA
XM_015093632.1	EPB41	mRNA	TCONS_00029398	lncRNA
XM_015093632.1	EPB41	mRNA	TCONS_00029398	lncRNA
XM_015093632.1	EPB41	mRNA	TCONS_00029398	lncRNA
XM_015093632.1	EPB41	mRNA	TCONS_00029398	lncRNA
XM_015093632.1	EPB41	mRNA	TCONS_00029398	lncRNA
XM_012098142.2	YEATS2	mRNA	XR_001436099.1	lncRNA
XM_012098142.2	YEATS2	mRNA	XR_001436099.1	lncRNA
XM_012098142.2	YEATS2	mRNA	XR_001436099.1	lncRNA
XM_012098142.2	YEATS2	mRNA	XR_001436099.1	lncRNA
XM_012098142.2	YEATS2	mRNA	XR_001436099.1	lncRNA
XM_012098142.2	YEATS2	mRNA	XR_001436099.1	lncRNA
XM_012098142.2	YEATS2	mRNA	XR_001436099.1	lncRNA
XM_012181720.2	ARID4A	mRNA	XR_001033284.2	lncRNA
XM_012181720.2	ARID4A	mRNA	XR_001033284.2	lncRNA
XM_012181720.2	ARID4A	mRNA	XR_001033284.2	lncRNA
XM_012181720.2	ARID4A	mRNA	XR_001033284.2	lncRNA
XM_012181720.2	ARID4A	mRNA	XR_001033284.2	lncRNA
XM_012181720.2	ARID4A	mRNA	XR_001033284.2	lncRNA
XM_012181720.2	ARID4A	mRNA	XR_001033284.2	lncRNA
XM_012103723.1	POLI	mRNA	XR_001033284.2	lncRNA
XM_012103723.1	POLI	mRNA	XR_001033284.2	lncRNA
XM_012103723.1	POLI	mRNA	XR_001033284.2	lncRNA
XM_012103723.1	POLI	mRNA	XR_001033284.2	lncRNA
XM_012103723.1	POLI	mRNA	XR_001033284.2	lncRNA
XM_012103723.1	POLI	mRNA	XR_001033284.2	lncRNA
XM_012103723.1	POLI	mRNA	XR_001033284.2	lncRNA
XM_012165221.2	PUM2	mRNA	XR_001436099.1	lncRNA
XM_012165221.2	PUM2	mRNA	XR_001436099.1	lncRNA
XM_012165221.2	PUM2	mRNA	XR_001436099.1	lncRNA
XM_012165221.2	PUM2	mRNA	XR_001436099.1	lncRNA
XM_012165221.2	PUM2	mRNA	XR_001436099.1	lncRNA
XM_012165221.2	PUM2	mRNA	XR_001436099.1	lncRNA
XM_012165221.2	PUM2	mRNA	XR_001436099.1	lncRNA
XM_015093088.1	CDCA2	mRNA	XR_001435104.1	lncRNA
XM_015093088.1	CDCA2	mRNA	XR_001435104.1	lncRNA
XM_015093088.1	CDCA2	mRNA	XR_001435104.1	lncRNA
XM_015093088.1	CDCA2	mRNA	XR_001435104.1	lncRNA
XM_015093088.1	CDCA2	mRNA	XR_001435104.1	lncRNA

XM_015093088.1	CDCA2	mRNA	XR_001435104.1	lncRNA
XM_015093088.1	CDCA2	mRNA	XR_001435104.1	lncRNA
XM_015093088.1	CDCA2	mRNA	XR_001435104.1	lncRNA
XM_012176365.2	SLC25A13	mRNA	XR_001435104.1	lncRNA
XM_012176365.2	SLC25A13	mRNA	XR_001435104.1	lncRNA
XM_012176365.2	SLC25A13	mRNA	XR_001435104.1	lncRNA
XM_012176365.2	SLC25A13	mRNA	XR_001435104.1	lncRNA
XM_012176365.2	SLC25A13	mRNA	XR_001435104.1	lncRNA
XM_012176365.2	SLC25A13	mRNA	XR_001435104.1	lncRNA
XM_012176365.2	SLC25A13	mRNA	XR_001435104.1	lncRNA
XM_012176365.2	SLC25A13	mRNA	XR_001435104.1	lncRNA
XM_012187575.2	RABGAP1L	mRNA	XR_001021939.1	lncRNA
XM_012187575.2	RABGAP1L	mRNA	XR_001021939.1	lncRNA
XM_012187575.2	RABGAP1L	mRNA	XR_001021939.1	lncRNA
XM_012187575.2	RABGAP1L	mRNA	XR_001021939.1	lncRNA
XM_012187575.2	RABGAP1L	mRNA	XR_001021939.1	lncRNA
XM_012187575.2	RABGAP1L	mRNA	XR_001021939.1	lncRNA
XM_012187575.2	RABGAP1L	mRNA	XR_001021939.1	lncRNA
XM_012177560.2	SLC26A2	mRNA	XR_001041202.1	lncRNA
XM_012177560.2	SLC26A2	mRNA	XR_001041202.1	lncRNA
XM_012177560.2	SLC26A2	mRNA	XR_001041202.1	lncRNA
XM_012177560.2	SLC26A2	mRNA	XR_001041202.1	lncRNA
XM_012177560.2	SLC26A2	mRNA	XR_001041202.1	lncRNA
XM_012177560.2	SLC26A2	mRNA	XR_001041202.1	lncRNA
XM_012177560.2	SLC26A2	mRNA	XR_001041202.1	lncRNA
XM_012186340.1	POLG2	mRNA	XR_001041202.1	lncRNA
XM_012186340.1	POLG2	mRNA	XR_001041202.1	lncRNA
XM_012186340.1	POLG2	mRNA	XR_001041202.1	lncRNA
XM_012186340.1	POLG2	mRNA	XR_001041202.1	lncRNA
XM_012186340.1	POLG2	mRNA	XR_001041202.1	lncRNA
XM_012186340.1	POLG2	mRNA	XR_001041202.1	lncRNA
XM_012186340.1	POLG2	mRNA	XR_001041202.1	lncRNA
XM_012113353.2	SUSD1	mRNA	XR_001041202.1	lncRNA
XM_012113353.2	SUSD1	mRNA	XR_001041202.1	lncRNA
XM_012113353.2	SUSD1	mRNA	XR_001041202.1	lncRNA
XM_012113353.2	SUSD1	mRNA	XR_001041202.1	lncRNA
XM_012113353.2	SUSD1	mRNA	XR_001041202.1	lncRNA
XM_012113353.2	SUSD1	mRNA	XR_001041202.1	lncRNA
XM_012113353.2	SUSD1	mRNA	XR_001041202.1	lncRNA
XM_004003140.2	NAALADL2	mRNA	XR_001021689.2	lncRNA
XM_004003140.2	NAALADL2	mRNA	XR_001021689.2	lncRNA
XM_004003140.2	NAALADL2	mRNA	XR_001021689.2	lncRNA
XM_004003140.2	NAALADL2	mRNA	XR_001021689.2	lncRNA
XM_004003140.2	NAALADL2	mRNA	XR_001021689.2	lncRNA
XM_004003140.2	NAALADL2	mRNA	XR_001021689.2	lncRNA
XM_004003140.2	NAALADL2	mRNA	XR_001021689.2	lncRNA
XM_012182538.2	TRAPPC3L	mRNA	TCONS_00071561	lncRNA
XM_012182538.2	TRAPPC3L	mRNA	TCONS_00071561	lncRNA
XM_012182538.2	TRAPPC3L	mRNA	TCONS_00071561	lncRNA
XM_012182538.2	TRAPPC3L	mRNA	TCONS_00071561	lncRNA
XM_012182538.2	TRAPPC3L	mRNA	TCONS_00071561	lncRNA
XM_012182538.2	TRAPPC3L	mRNA	TCONS_00071561	lncRNA
XM_012182538.2	TRAPPC3L	mRNA	TCONS_00071561	lncRNA
XM_012182238.1	SPG11	mRNA	XR_001021689.2	lncRNA

XM_012182238.1	SPG11	mRNA	XR_001021689.2	lncRNA
XM_012182238.1	SPG11	mRNA	XR_001021689.2	lncRNA
XM_012182238.1	SPG11	mRNA	XR_001021689.2	lncRNA
XM_012182238.1	SPG11	mRNA	XR_001021689.2	lncRNA
XM_012182238.1	SPG11	mRNA	XR_001021689.2	lncRNA
XM_012182238.1	SPG11	mRNA	XR_001021689.2	lncRNA
XM_012097252.2	RHOF	mRNA	XR_001041209.2	lncRNA
XM_012097252.2	RHOF	mRNA	XR_001041209.2	lncRNA
XM_012097252.2	RHOF	mRNA	XR_001041209.2	lncRNA
XM_012097252.2	RHOF	mRNA	XR_001041209.2	lncRNA
XM_012097252.2	RHOF	mRNA	XR_001041209.2	lncRNA
XM_012097252.2	RHOF	mRNA	XR_001041209.2	lncRNA
XM_012097252.2	RHOF	mRNA	XR_001041209.2	lncRNA
XM_012097252.2	RHOF	mRNA	XR_001041209.2	lncRNA
XM_015100095.1	LOC10111352	mRNA	XR_001041209.2	lncRNA
XM_015100095.1	LOC10111352	mRNA	XR_001041209.2	lncRNA
XM_015100095.1	LOC10111352	mRNA	XR_001041209.2	lncRNA
XM_015100095.1	LOC10111352	mRNA	XR_001041209.2	lncRNA
XM_015100095.1	LOC10111352	mRNA	XR_001041209.2	lncRNA
XM_015100095.1	LOC10111352	mRNA	XR_001041209.2	lncRNA
XM_015100095.1	LOC10111352	mRNA	XR_001041209.2	lncRNA
XM_012099837.2	SLMAP	mRNA	XR_001041209.2	lncRNA
XM_012099837.2	SLMAP	mRNA	XR_001041209.2	lncRNA
XM_012099837.2	SLMAP	mRNA	XR_001041209.2	lncRNA
XM_012099837.2	SLMAP	mRNA	XR_001041209.2	lncRNA
XM_012099837.2	SLMAP	mRNA	XR_001041209.2	lncRNA
XM_012099837.2	SLMAP	mRNA	XR_001041209.2	lncRNA
XM_012099837.2	SLMAP	mRNA	XR_001041209.2	lncRNA
XM_015099384.1	SYT14	mRNA	XR_001041209.2	lncRNA
XM_015099384.1	SYT14	mRNA	XR_001041209.2	lncRNA
XM_015099384.1	SYT14	mRNA	XR_001041209.2	lncRNA
XM_015099384.1	SYT14	mRNA	XR_001041209.2	lncRNA
XM_015099384.1	SYT14	mRNA	XR_001041209.2	lncRNA
XM_015099384.1	SYT14	mRNA	XR_001041209.2	lncRNA
XM_015099384.1	SYT14	mRNA	XR_001041209.2	lncRNA
XM_012181854.1	GPHN	mRNA	XR_001041211.2	lncRNA
XM_012181854.1	GPHN	mRNA	XR_001041211.2	lncRNA
XM_012181854.1	GPHN	mRNA	XR_001041211.2	lncRNA
XM_012181854.1	GPHN	mRNA	XR_001041211.2	lncRNA
XM_012181854.1	GPHN	mRNA	XR_001041211.2	lncRNA
XM_012181854.1	GPHN	mRNA	XR_001041211.2	lncRNA
XM_012181854.1	GPHN	mRNA	XR_001041211.2	lncRNA
XM_004008093.2	PTN	mRNA	TCONS_00029399	lncRNA
XM_004008093.2	PTN	mRNA	TCONS_00029399	lncRNA
XM_004008093.2	PTN	mRNA	TCONS_00029399	lncRNA
XM_004008093.2	PTN	mRNA	TCONS_00029399	lncRNA
XM_004008093.2	PTN	mRNA	TCONS_00029399	lncRNA
XM_004008093.2	PTN	mRNA	TCONS_00029399	lncRNA
XM_004008093.2	PTN	mRNA	TCONS_00029399	lncRNA
XM_012105225.2	SGPL1	mRNA	XR_001041209.2	lncRNA
XM_012105225.2	SGPL1	mRNA	XR_001041209.2	lncRNA
XM_012105225.2	SGPL1	mRNA	XR_001041209.2	lncRNA
XM_012105225.2	SGPL1	mRNA	XR_001041209.2	lncRNA
XM_012105225.2	SGPL1	mRNA	XR_001041209.2	lncRNA

XM_012105225.2	SGPL1	mRNA	XR_001041209.2	lncRNA
XM_012105225.2	SGPL1	mRNA	XR_001041209.2	lncRNA
XM_012098342.2	FXR1	mRNA	XR_001041209.2	lncRNA
XM_012098342.2	FXR1	mRNA	XR_001041209.2	lncRNA
XM_012098342.2	FXR1	mRNA	XR_001041209.2	lncRNA
XM_012098342.2	FXR1	mRNA	XR_001041209.2	lncRNA
XM_012098342.2	FXR1	mRNA	XR_001041209.2	lncRNA
XM_012098342.2	FXR1	mRNA	XR_001041209.2	lncRNA
XM_012098342.2	FXR1	mRNA	XR_001041209.2	lncRNA
XM_015099384.1	SYT14	mRNA	TCONS_00029399	lncRNA
XM_015099384.1	SYT14	mRNA	TCONS_00029399	lncRNA
XM_015099384.1	SYT14	mRNA	TCONS_00029399	lncRNA
XM_015099384.1	SYT14	mRNA	TCONS_00029399	lncRNA
XM_015099384.1	SYT14	mRNA	TCONS_00029399	lncRNA
XM_015099384.1	SYT14	mRNA	TCONS_00029399	lncRNA
XM_015099384.1	SYT14	mRNA	TCONS_00029399	lncRNA
XM_015096769.1	APC	mRNA	TCONS_00029398	lncRNA
XM_015096769.1	APC	mRNA	TCONS_00029398	lncRNA
XM_015096769.1	APC	mRNA	TCONS_00029398	lncRNA
XM_015096769.1	APC	mRNA	TCONS_00029398	lncRNA
XM_015096769.1	APC	mRNA	TCONS_00029398	lncRNA
XM_015096769.1	APC	mRNA	TCONS_00029398	lncRNA
XM_015096769.1	APC	mRNA	TCONS_00029398	lncRNA
XM_015104131.1	TNRC6A	mRNA	XR_001436609.1	lncRNA
XM_015104131.1	TNRC6A	mRNA	XR_001436609.1	lncRNA
XM_015104131.1	TNRC6A	mRNA	XR_001436609.1	lncRNA
XM_015104131.1	TNRC6A	mRNA	XR_001436609.1	lncRNA
XM_015104131.1	TNRC6A	mRNA	XR_001436609.1	lncRNA
XM_015104131.1	TNRC6A	mRNA	XR_001436609.1	lncRNA
XM_015104131.1	TNRC6A	mRNA	XR_001436609.1	lncRNA
XM_004022347.2	TMEM255A	mRNA	XR_001436609.1	lncRNA
XM_004022347.2	TMEM255A	mRNA	XR_001436609.1	lncRNA
XM_004022347.2	TMEM255A	mRNA	XR_001436609.1	lncRNA
XM_004022347.2	TMEM255A	mRNA	XR_001436609.1	lncRNA
XM_004022347.2	TMEM255A	mRNA	XR_001436609.1	lncRNA
XM_004022347.2	TMEM255A	mRNA	XR_001436609.1	lncRNA
XM_004022347.2	TMEM255A	mRNA	XR_001436609.1	lncRNA
XM_012106466.2	DMD	mRNA	XR_001436609.1	lncRNA
XM_012106466.2	DMD	mRNA	XR_001436609.1	lncRNA
XM_012106466.2	DMD	mRNA	XR_001436609.1	lncRNA
XM_012106466.2	DMD	mRNA	XR_001436609.1	lncRNA
XM_012106466.2	DMD	mRNA	XR_001436609.1	lncRNA
XM_012106466.2	DMD	mRNA	XR_001436609.1	lncRNA
XM_012106466.2	DMD	mRNA	XR_001436609.1	lncRNA
XM_012180800.1	TIPIN	mRNA	XR_001436609.1	lncRNA
XM_012180800.1	TIPIN	mRNA	XR_001436609.1	lncRNA
XM_012180800.1	TIPIN	mRNA	XR_001436609.1	lncRNA
XM_012180800.1	TIPIN	mRNA	XR_001436609.1	lncRNA
XM_012180800.1	TIPIN	mRNA	XR_001436609.1	lncRNA
XM_012180800.1	TIPIN	mRNA	XR_001436609.1	lncRNA
XM_012180800.1	TIPIN	mRNA	XR_001436609.1	lncRNA
XM_012190105.1	ANKRL17	mRNA	XR_001436609.1	lncRNA
XM_012190105.1	ANKRL17	mRNA	XR_001436609.1	lncRNA
XM_012190105.1	ANKRL17	mRNA	XR_001436609.1	lncRNA

XM_012190105.1	ANKRL17	mRNA	XR_001436609.1	lncRNA
XM_012190105.1	ANKRL17	mRNA	XR_001436609.1	lncRNA
XM_012190105.1	ANKRL17	mRNA	XR_001436609.1	lncRNA
XM_012190105.1	ANKRL17	mRNA	XR_001436609.1	lncRNA
XM_012176656.2	NAPEPLD	mRNA	XR_001436609.1	lncRNA
XM_012176656.2	NAPEPLD	mRNA	XR_001436609.1	lncRNA
XM_012176656.2	NAPEPLD	mRNA	XR_001436609.1	lncRNA
XM_012176656.2	NAPEPLD	mRNA	XR_001436609.1	lncRNA
XM_012176656.2	NAPEPLD	mRNA	XR_001436609.1	lncRNA
XM_012176656.2	NAPEPLD	mRNA	XR_001436609.1	lncRNA
XM_012176656.2	NAPEPLD	mRNA	XR_001436609.1	lncRNA
XM_012187575.2	RABGAP1L	mRNA	XR_001436609.1	lncRNA
XM_012187575.2	RABGAP1L	mRNA	XR_001436609.1	lncRNA
XM_012187575.2	RABGAP1L	mRNA	XR_001436609.1	lncRNA
XM_012187575.2	RABGAP1L	mRNA	XR_001436609.1	lncRNA
XM_012187575.2	RABGAP1L	mRNA	XR_001436609.1	lncRNA
XM_012187575.2	RABGAP1L	mRNA	XR_001436609.1	lncRNA
XM_012187575.2	RABGAP1L	mRNA	XR_001436609.1	lncRNA
XM_004008647.3	RAPGEF6	mRNA	XR_001436609.1	lncRNA
XM_004008647.3	RAPGEF6	mRNA	XR_001436609.1	lncRNA
XM_004008647.3	RAPGEF6	mRNA	XR_001436609.1	lncRNA
XM_004008647.3	RAPGEF6	mRNA	XR_001436609.1	lncRNA
XM_004008647.3	RAPGEF6	mRNA	XR_001436609.1	lncRNA
XM_004008647.3	RAPGEF6	mRNA	XR_001436609.1	lncRNA
XM_004008647.3	RAPGEF6	mRNA	XR_001436609.1	lncRNA
XM_012105800.2	PCM1	mRNA	XR_001436609.1	lncRNA
XM_012105800.2	PCM1	mRNA	XR_001436609.1	lncRNA
XM_012105800.2	PCM1	mRNA	XR_001436609.1	lncRNA
XM_012105800.2	PCM1	mRNA	XR_001436609.1	lncRNA
XM_012105800.2	PCM1	mRNA	XR_001436609.1	lncRNA
XM_012105800.2	PCM1	mRNA	XR_001436609.1	lncRNA
XM_012105800.2	PCM1	mRNA	XR_001436609.1	lncRNA
XM_012187575.2	RABGAP1L	mRNA	XR_001435104.1	lncRNA
XM_012187575.2	RABGAP1L	mRNA	XR_001435104.1	lncRNA
XM_012187575.2	RABGAP1L	mRNA	XR_001435104.1	lncRNA
XM_012187575.2	RABGAP1L	mRNA	XR_001435104.1	lncRNA
XM_012187575.2	RABGAP1L	mRNA	XR_001435104.1	lncRNA
XM_012187575.2	RABGAP1L	mRNA	XR_001435104.1	lncRNA
XM_012187575.2	RABGAP1L	mRNA	XR_001435104.1	lncRNA
XM_012174787.1	LOC1011137	mRNA	XR_001436609.1	lncRNA
XM_012174787.1	LOC1011137	mRNA	XR_001436609.1	lncRNA
XM_012174787.1	LOC1011137	mRNA	XR_001436609.1	lncRNA
XM_012174787.1	LOC1011137	mRNA	XR_001436609.1	lncRNA
XM_012174787.1	LOC1011137	mRNA	XR_001436609.1	lncRNA
XM_012174787.1	LOC1011137	mRNA	XR_001436609.1	lncRNA
XM_012174787.1	LOC1011137	mRNA	XR_001436609.1	lncRNA
XM_012180800.1	TIPIN	mRNA	XR_001435104.1	lncRNA
XM_012180800.1	TIPIN	mRNA	XR_001435104.1	lncRNA
XM_012180800.1	TIPIN	mRNA	XR_001435104.1	lncRNA
XM_012180800.1	TIPIN	mRNA	XR_001435104.1	lncRNA
XM_012180800.1	TIPIN	mRNA	XR_001435104.1	lncRNA
XM_012180800.1	TIPIN	mRNA	XR_001435104.1	lncRNA
XM_012180800.1	TIPIN	mRNA	XR_001435104.1	lncRNA
XM_012116202.1	IARS	mRNA	XR_001436531.1	lncRNA

XM_012116202.1	IARS	mRNA	XR_001436531.1	lncRNA
XM_012116202.1	IARS	mRNA	XR_001436531.1	lncRNA
XM_012116202.1	IARS	mRNA	XR_001436531.1	lncRNA
XM_012116202.1	IARS	mRNA	XR_001436531.1	lncRNA
XM_012116202.1	IARS	mRNA	XR_001436531.1	lncRNA
XM_012116202.1	IARS	mRNA	XR_001436531.1	lncRNA
XM_012095858.2	ZNF214	mRNA	XR_001435104.1	lncRNA
XM_012095858.2	ZNF214	mRNA	XR_001435104.1	lncRNA
XM_012095858.2	ZNF214	mRNA	XR_001435104.1	lncRNA
XM_012095858.2	ZNF214	mRNA	XR_001435104.1	lncRNA
XM_012095858.2	ZNF214	mRNA	XR_001435104.1	lncRNA
XM_012095858.2	ZNF214	mRNA	XR_001435104.1	lncRNA
XM_012095858.2	ZNF214	mRNA	XR_001435104.1	lncRNA
XM_004022347.2	TMEM255A	mRNA	XR_001435104.1	lncRNA
XM_004022347.2	TMEM255A	mRNA	XR_001435104.1	lncRNA
XM_004022347.2	TMEM255A	mRNA	XR_001435104.1	lncRNA
XM_004022347.2	TMEM255A	mRNA	XR_001435104.1	lncRNA
XM_004022347.2	TMEM255A	mRNA	XR_001435104.1	lncRNA
XM_004022347.2	TMEM255A	mRNA	XR_001435104.1	lncRNA
XM_004022347.2	TMEM255A	mRNA	XR_001435104.1	lncRNA
XM_004008647.3	RAPGEF6	mRNA	XR_001435104.1	lncRNA
XM_004008647.3	RAPGEF6	mRNA	XR_001435104.1	lncRNA
XM_004008647.3	RAPGEF6	mRNA	XR_001435104.1	lncRNA
XM_004008647.3	RAPGEF6	mRNA	XR_001435104.1	lncRNA
XM_004008647.3	RAPGEF6	mRNA	XR_001435104.1	lncRNA
XM_004008647.3	RAPGEF6	mRNA	XR_001435104.1	lncRNA
XM_004008647.3	RAPGEF6	mRNA	XR_001435104.1	lncRNA
XM_012105800.2	PCM1	mRNA	XR_001435104.1	lncRNA
XM_012105800.2	PCM1	mRNA	XR_001435104.1	lncRNA
XM_012105800.2	PCM1	mRNA	XR_001435104.1	lncRNA
XM_012105800.2	PCM1	mRNA	XR_001435104.1	lncRNA
XM_012105800.2	PCM1	mRNA	XR_001435104.1	lncRNA
XM_012105800.2	PCM1	mRNA	XR_001435104.1	lncRNA
XM_012105800.2	PCM1	mRNA	XR_001435104.1	lncRNA
XM_015097252.1	SLC17A5	mRNA	XR_001435104.1	lncRNA
XM_015097252.1	SLC17A5	mRNA	XR_001435104.1	lncRNA
XM_015097252.1	SLC17A5	mRNA	XR_001435104.1	lncRNA
XM_015097252.1	SLC17A5	mRNA	XR_001435104.1	lncRNA
XM_015097252.1	SLC17A5	mRNA	XR_001435104.1	lncRNA
XM_015097252.1	SLC17A5	mRNA	XR_001435104.1	lncRNA
XM_015097252.1	SLC17A5	mRNA	XR_001435104.1	lncRNA
XM_012133845.1	DYNC1I2	mRNA	XR_001435104.1	lncRNA
XM_012133845.1	DYNC1I2	mRNA	XR_001435104.1	lncRNA
XM_012133845.1	DYNC1I2	mRNA	XR_001435104.1	lncRNA
XM_012133845.1	DYNC1I2	mRNA	XR_001435104.1	lncRNA
XM_012133845.1	DYNC1I2	mRNA	XR_001435104.1	lncRNA
XM_012133845.1	DYNC1I2	mRNA	XR_001435104.1	lncRNA
XM_012133845.1	DYNC1I2	mRNA	XR_001435104.1	lncRNA
XM_012188017.2	ZNF654	mRNA	XR_001436531.1	lncRNA
XM_012188017.2	ZNF654	mRNA	XR_001436531.1	lncRNA
XM_012188017.2	ZNF654	mRNA	XR_001436531.1	lncRNA
XM_012188017.2	ZNF654	mRNA	XR_001436531.1	lncRNA
XM_012188017.2	ZNF654	mRNA	XR_001436531.1	lncRNA
XM_012188017.2	ZNF654	mRNA	XR_001436531.1	lncRNA

XM_012188017.2	ZNF654	mRNA	XR_001436531.1	lncRNA
XM_012190105.1	ANKRL17	mRNA	XR_001435104.1	lncRNA
XM_012190105.1	ANKRL17	mRNA	XR_001435104.1	lncRNA
XM_012190105.1	ANKRL17	mRNA	XR_001435104.1	lncRNA
XM_012190105.1	ANKRL17	mRNA	XR_001435104.1	lncRNA
XM_012190105.1	ANKRL17	mRNA	XR_001435104.1	lncRNA
XM_012190105.1	ANKRL17	mRNA	XR_001435104.1	lncRNA
XM_012190105.1	ANKRL17	mRNA	XR_001435104.1	lncRNA
XM_012180800.1	TIPIN	mRNA	XR_001435726.1	lncRNA
XM_012180800.1	TIPIN	mRNA	XR_001435726.1	lncRNA
XM_012180800.1	TIPIN	mRNA	XR_001435726.1	lncRNA
XM_012180800.1	TIPIN	mRNA	XR_001435726.1	lncRNA
XM_012180800.1	TIPIN	mRNA	XR_001435726.1	lncRNA
XM_012180800.1	TIPIN	mRNA	XR_001435726.1	lncRNA
XM_012180800.1	TIPIN	mRNA	XR_001435726.1	lncRNA
XM_012184103.1	SDC2	mRNA	XR_001042381.2	lncRNA
XM_012184103.1	SDC2	mRNA	XR_001042381.2	lncRNA
XM_012184103.1	SDC2	mRNA	XR_001042381.2	lncRNA
XM_012184103.1	SDC2	mRNA	XR_001042381.2	lncRNA
XM_012184103.1	SDC2	mRNA	XR_001042381.2	lncRNA
XM_012184103.1	SDC2	mRNA	XR_001042381.2	lncRNA
XM_012184103.1	SDC2	mRNA	XR_001042381.2	lncRNA
XM_012174787.1	LOC1011137	mRNA	XR_001435726.1	lncRNA
XM_012174787.1	LOC1011137	mRNA	XR_001435726.1	lncRNA
XM_012174787.1	LOC1011137	mRNA	XR_001435726.1	lncRNA
XM_012174787.1	LOC1011137	mRNA	XR_001435726.1	lncRNA
XM_012174787.1	LOC1011137	mRNA	XR_001435726.1	lncRNA
XM_012174787.1	LOC1011137	mRNA	XR_001435726.1	lncRNA
XM_012174787.1	LOC1011137	mRNA	XR_001435726.1	lncRNA
XM_012105564.2	ZFAND4	mRNA	XR_001042381.2	lncRNA
XM_012105564.2	ZFAND4	mRNA	XR_001042381.2	lncRNA
XM_012105564.2	ZFAND4	mRNA	XR_001042381.2	lncRNA
XM_012105564.2	ZFAND4	mRNA	XR_001042381.2	lncRNA
XM_012105564.2	ZFAND4	mRNA	XR_001042381.2	lncRNA
XM_012105564.2	ZFAND4	mRNA	XR_001042381.2	lncRNA
XM_012105564.2	ZFAND4	mRNA	XR_001042381.2	lncRNA
XM_015099706.1	ZMYND11	mRNA	XR_001042381.2	lncRNA
XM_015099706.1	ZMYND11	mRNA	XR_001042381.2	lncRNA
XM_015099706.1	ZMYND11	mRNA	XR_001042381.2	lncRNA
XM_015099706.1	ZMYND11	mRNA	XR_001042381.2	lncRNA
XM_015099706.1	ZMYND11	mRNA	XR_001042381.2	lncRNA
XM_015099706.1	ZMYND11	mRNA	XR_001042381.2	lncRNA
XM_015099706.1	ZMYND11	mRNA	XR_001042381.2	lncRNA
XM_012179641.2	LOC1011228	mRNA	XR_001435723.1	lncRNA
XM_012179641.2	LOC1011228	mRNA	XR_001435723.1	lncRNA
XM_012179641.2	LOC1011228	mRNA	XR_001435723.1	lncRNA
XM_012179641.2	LOC1011228	mRNA	XR_001435723.1	lncRNA
XM_012179641.2	LOC1011228	mRNA	XR_001435723.1	lncRNA
XM_012179641.2	LOC1011228	mRNA	XR_001435723.1	lncRNA
XM_012179641.2	LOC1011228	mRNA	XR_001435723.1	lncRNA
XM_012174754.2	RNF41	mRNA	XR_001435723.1	lncRNA
XM_012174754.2	RNF41	mRNA	XR_001435723.1	lncRNA
XM_012174754.2	RNF41	mRNA	XR_001435723.1	lncRNA
XM_012174754.2	RNF41	mRNA	XR_001435723.1	lncRNA

XM_012174754.2	RNF41	mRNA	XR_001435723.1	lncRNA
XM_012174754.2	RNF41	mRNA	XR_001435723.1	lncRNA
XM_012174754.2	RNF41	mRNA	XR_001435723.1	lncRNA
XM_004008647.3	RAPGEF6	mRNA	XR_001435726.1	lncRNA
XM_004008647.3	RAPGEF6	mRNA	XR_001435726.1	lncRNA
XM_004008647.3	RAPGEF6	mRNA	XR_001435726.1	lncRNA
XM_004008647.3	RAPGEF6	mRNA	XR_001435726.1	lncRNA
XM_004008647.3	RAPGEF6	mRNA	XR_001435726.1	lncRNA
XM_004008647.3	RAPGEF6	mRNA	XR_001435726.1	lncRNA
XM_004008647.3	RAPGEF6	mRNA	XR_001435726.1	lncRNA
XM_015097323.1	PKIB	mRNA	XR_001435726.1	lncRNA
XM_015097323.1	PKIB	mRNA	XR_001435726.1	lncRNA
XM_015097323.1	PKIB	mRNA	XR_001435726.1	lncRNA
XM_015097323.1	PKIB	mRNA	XR_001435726.1	lncRNA
XM_015097323.1	PKIB	mRNA	XR_001435726.1	lncRNA
XM_015097323.1	PKIB	mRNA	XR_001435726.1	lncRNA
XM_015097323.1	PKIB	mRNA	XR_001435726.1	lncRNA
XM_012174754.2	RNF41	mRNA	XR_001435726.1	lncRNA
XM_012174754.2	RNF41	mRNA	XR_001435726.1	lncRNA
XM_012174754.2	RNF41	mRNA	XR_001435726.1	lncRNA
XM_012174754.2	RNF41	mRNA	XR_001435726.1	lncRNA
XM_012174754.2	RNF41	mRNA	XR_001435726.1	lncRNA
XM_012174754.2	RNF41	mRNA	XR_001435726.1	lncRNA
XM_012174754.2	RNF41	mRNA	XR_001435726.1	lncRNA
XM_012118246.2	DCAF12	mRNA	XR_001042381.2	lncRNA
XM_012118246.2	DCAF12	mRNA	XR_001042381.2	lncRNA
XM_012118246.2	DCAF12	mRNA	XR_001042381.2	lncRNA
XM_012118246.2	DCAF12	mRNA	XR_001042381.2	lncRNA
XM_012118246.2	DCAF12	mRNA	XR_001042381.2	lncRNA
XM_012118246.2	DCAF12	mRNA	XR_001042381.2	lncRNA
XM_012118246.2	DCAF12	mRNA	XR_001042381.2	lncRNA
XM_015097323.1	PKIB	mRNA	XR_001435723.1	lncRNA
XM_015097323.1	PKIB	mRNA	XR_001435723.1	lncRNA
XM_015097323.1	PKIB	mRNA	XR_001435723.1	lncRNA
XM_015097323.1	PKIB	mRNA	XR_001435723.1	lncRNA
XM_015097323.1	PKIB	mRNA	XR_001435723.1	lncRNA
XM_015097323.1	PKIB	mRNA	XR_001435723.1	lncRNA
XM_015097323.1	PKIB	mRNA	XR_001435723.1	lncRNA
XM_012164777.2	NBAS	mRNA	XR_001042381.2	lncRNA
XM_012164777.2	NBAS	mRNA	XR_001042381.2	lncRNA
XM_012164777.2	NBAS	mRNA	XR_001042381.2	lncRNA
XM_012164777.2	NBAS	mRNA	XR_001042381.2	lncRNA
XM_012164777.2	NBAS	mRNA	XR_001042381.2	lncRNA
XM_012164777.2	NBAS	mRNA	XR_001042381.2	lncRNA
XM_012164777.2	NBAS	mRNA	XR_001042381.2	lncRNA
XM_012174754.2	RNF41	mRNA	XR_001042381.2	lncRNA
XM_012174754.2	RNF41	mRNA	XR_001042381.2	lncRNA
XM_012174754.2	RNF41	mRNA	XR_001042381.2	lncRNA
XM_012174754.2	RNF41	mRNA	XR_001042381.2	lncRNA
XM_012174754.2	RNF41	mRNA	XR_001042381.2	lncRNA
XM_012174754.2	RNF41	mRNA	XR_001042381.2	lncRNA
XM_012174754.2	RNF41	mRNA	XR_001042381.2	lncRNA
XM_012179641.2	LOC1011228(	mRNA	XR_001435726.1	lncRNA
XM_012179641.2	LOC1011228(	mRNA	XR_001435726.1	lncRNA

XM_012179641.2	LOC1011228(	mRNA	XR_001435726.1	lncRNA
XM_012179641.2	LOC1011228(	mRNA	XR_001435726.1	lncRNA
XM_012179641.2	LOC1011228(	mRNA	XR_001435726.1	lncRNA
XM_012179641.2	LOC1011228(	mRNA	XR_001435726.1	lncRNA
XM_012179641.2	LOC1011228(	mRNA	XR_001435726.1	lncRNA
XM_012095398.2	NPAT	mRNA	XR_001042381.2	lncRNA
XM_012095398.2	NPAT	mRNA	XR_001042381.2	lncRNA
XM_012095398.2	NPAT	mRNA	XR_001042381.2	lncRNA
XM_012095398.2	NPAT	mRNA	XR_001042381.2	lncRNA
XM_012095398.2	NPAT	mRNA	XR_001042381.2	lncRNA
XM_012095398.2	NPAT	mRNA	XR_001042381.2	lncRNA
XM_012095398.2	NPAT	mRNA	XR_001042381.2	lncRNA
XM_015096057.1	KDM3B	mRNA	TCONS_00071561	lncRNA
XM_015096057.1	KDM3B	mRNA	TCONS_00071561	lncRNA
XM_015096057.1	KDM3B	mRNA	TCONS_00071561	lncRNA
XM_015096057.1	KDM3B	mRNA	TCONS_00071561	lncRNA
XM_015096057.1	KDM3B	mRNA	TCONS_00071561	lncRNA
XM_015096057.1	KDM3B	mRNA	TCONS_00071561	lncRNA
XM_015096057.1	KDM3B	mRNA	TCONS_00071561	lncRNA
XM_012182854.2	PTPRK	mRNA	TCONS_00071561	lncRNA
XM_012182854.2	PTPRK	mRNA	TCONS_00071561	lncRNA
XM_012182854.2	PTPRK	mRNA	TCONS_00071561	lncRNA
XM_012182854.2	PTPRK	mRNA	TCONS_00071561	lncRNA
XM_012182854.2	PTPRK	mRNA	TCONS_00071561	lncRNA
XM_012182854.2	PTPRK	mRNA	TCONS_00071561	lncRNA
XM_012182854.2	PTPRK	mRNA	TCONS_00071561	lncRNA
XM_015097611.1	VPS13B	mRNA	XR_001021689.2	lncRNA
XM_015097611.1	VPS13B	mRNA	XR_001021689.2	lncRNA
XM_015097611.1	VPS13B	mRNA	XR_001021689.2	lncRNA
XM_015097611.1	VPS13B	mRNA	XR_001021689.2	lncRNA
XM_015097611.1	VPS13B	mRNA	XR_001021689.2	lncRNA
XM_015097611.1	VPS13B	mRNA	XR_001021689.2	lncRNA
XM_015097611.1	VPS13B	mRNA	XR_001021689.2	lncRNA
XM_012173782.2	REV1	mRNA	XR_001021689.2	lncRNA
XM_012173782.2	REV1	mRNA	XR_001021689.2	lncRNA
XM_012173782.2	REV1	mRNA	XR_001021689.2	lncRNA
XM_012173782.2	REV1	mRNA	XR_001021689.2	lncRNA
XM_012173782.2	REV1	mRNA	XR_001021689.2	lncRNA
XM_012173782.2	REV1	mRNA	XR_001021689.2	lncRNA
XM_012173782.2	REV1	mRNA	XR_001021689.2	lncRNA
XM_012099994.2	VPRBP	mRNA	TCONS_00071561	lncRNA
XM_012099994.2	VPRBP	mRNA	TCONS_00071561	lncRNA
XM_012099994.2	VPRBP	mRNA	TCONS_00071561	lncRNA
XM_012099994.2	VPRBP	mRNA	TCONS_00071561	lncRNA
XM_012099994.2	VPRBP	mRNA	TCONS_00071561	lncRNA
XM_012099994.2	VPRBP	mRNA	TCONS_00071561	lncRNA
XM_012099994.2	VPRBP	mRNA	TCONS_00071561	lncRNA
XM_004009100.3	PAM	mRNA	XR_001021689.2	lncRNA
XM_004009100.3	PAM	mRNA	XR_001021689.2	lncRNA
XM_004009100.3	PAM	mRNA	XR_001021689.2	lncRNA
XM_004009100.3	PAM	mRNA	XR_001021689.2	lncRNA
XM_004009100.3	PAM	mRNA	XR_001021689.2	lncRNA
XM_004009100.3	PAM	mRNA	XR_001021689.2	lncRNA
XM_004009100.3	PAM	mRNA	XR_001021689.2	lncRNA

XM_012187548.2	GIGYF2	mRNA	TCONS_00071561	lncRNA
XM_012187548.2	GIGYF2	mRNA	TCONS_00071561	lncRNA
XM_012187548.2	GIGYF2	mRNA	TCONS_00071561	lncRNA
XM_012187548.2	GIGYF2	mRNA	TCONS_00071561	lncRNA
XM_012187548.2	GIGYF2	mRNA	TCONS_00071561	lncRNA
XM_012187548.2	GIGYF2	mRNA	TCONS_00071561	lncRNA
XM_012187548.2	GIGYF2	mRNA	TCONS_00071561	lncRNA
XM_012177560.2	SLC26A2	mRNA	XR_001436099.1	lncRNA
XM_012177560.2	SLC26A2	mRNA	XR_001436099.1	lncRNA
XM_012177560.2	SLC26A2	mRNA	XR_001436099.1	lncRNA
XM_012177560.2	SLC26A2	mRNA	XR_001436099.1	lncRNA
XM_012177560.2	SLC26A2	mRNA	XR_001436099.1	lncRNA
XM_012177560.2	SLC26A2	mRNA	XR_001436099.1	lncRNA
XM_012177560.2	SLC26A2	mRNA	XR_001436099.1	lncRNA
XM_012175265.1	PLEKHA5	mRNA	XR_001033284.2	lncRNA
XM_012175265.1	PLEKHA5	mRNA	XR_001033284.2	lncRNA
XM_012175265.1	PLEKHA5	mRNA	XR_001033284.2	lncRNA
XM_012175265.1	PLEKHA5	mRNA	XR_001033284.2	lncRNA
XM_012175265.1	PLEKHA5	mRNA	XR_001033284.2	lncRNA
XM_012175265.1	PLEKHA5	mRNA	XR_001033284.2	lncRNA
XM_012175265.1	PLEKHA5	mRNA	XR_001033284.2	lncRNA
XM_012174607.2	HELB	mRNA	XR_001434081.1	lncRNA
XM_012174607.2	HELB	mRNA	XR_001434081.1	lncRNA
XM_012174607.2	HELB	mRNA	XR_001434081.1	lncRNA
XM_012174607.2	HELB	mRNA	XR_001434081.1	lncRNA
XM_012174607.2	HELB	mRNA	XR_001434081.1	lncRNA
XM_012174607.2	HELB	mRNA	XR_001434081.1	lncRNA
XM_012174607.2	HELB	mRNA	XR_001434081.1	lncRNA
XM_012178157.2	RAPGEF6	mRNA	XR_001434081.1	lncRNA
XM_012178157.2	RAPGEF6	mRNA	XR_001434081.1	lncRNA
XM_012178157.2	RAPGEF6	mRNA	XR_001434081.1	lncRNA
XM_012178157.2	RAPGEF6	mRNA	XR_001434081.1	lncRNA
XM_012178157.2	RAPGEF6	mRNA	XR_001434081.1	lncRNA
XM_012178157.2	RAPGEF6	mRNA	XR_001434081.1	lncRNA
XM_012178157.2	RAPGEF6	mRNA	XR_001434081.1	lncRNA
XM_015092478.1	TFRC	mRNA	XR_001434081.1	lncRNA
XM_015092478.1	TFRC	mRNA	XR_001434081.1	lncRNA
XM_015092478.1	TFRC	mRNA	XR_001434081.1	lncRNA
XM_015092478.1	TFRC	mRNA	XR_001434081.1	lncRNA
XM_015092478.1	TFRC	mRNA	XR_001434081.1	lncRNA
XM_015092478.1	TFRC	mRNA	XR_001434081.1	lncRNA
XM_015092478.1	TFRC	mRNA	XR_001434081.1	lncRNA
XM_012190192.2	PHLDB2	mRNA	TCONS_00029398	lncRNA
XM_012190192.2	PHLDB2	mRNA	TCONS_00029398	lncRNA
XM_012190192.2	PHLDB2	mRNA	TCONS_00029398	lncRNA
XM_012190192.2	PHLDB2	mRNA	TCONS_00029398	lncRNA
XM_012190192.2	PHLDB2	mRNA	TCONS_00029398	lncRNA
XM_012190192.2	PHLDB2	mRNA	TCONS_00029398	lncRNA
XM_012190192.2	PHLDB2	mRNA	TCONS_00029398	lncRNA
XM_012179952.2	FRYL	mRNA	XR_001041202.1	lncRNA
XM_012179952.2	FRYL	mRNA	XR_001041202.1	lncRNA
XM_012179952.2	FRYL	mRNA	XR_001041202.1	lncRNA
XM_012179952.2	FRYL	mRNA	XR_001041202.1	lncRNA
XM_012179952.2	FRYL	mRNA	XR_001041202.1	lncRNA

XM_012179952.2	FRYL	mRNA	XR_001041202.1	lncRNA
XM_012179952.2	FRYL	mRNA	XR_001041202.1	lncRNA
XM_012106629.2	WNK3	mRNA	XR_001041202.1	lncRNA
XM_012106629.2	WNK3	mRNA	XR_001041202.1	lncRNA
XM_012106629.2	WNK3	mRNA	XR_001041202.1	lncRNA
XM_012106629.2	WNK3	mRNA	XR_001041202.1	lncRNA
XM_012106629.2	WNK3	mRNA	XR_001041202.1	lncRNA
XM_012106629.2	WNK3	mRNA	XR_001041202.1	lncRNA
XM_012106629.2	WNK3	mRNA	XR_001041202.1	lncRNA
XM_012103019.2	INPP5F	mRNA	XR_001041202.1	lncRNA
XM_012103019.2	INPP5F	mRNA	XR_001041202.1	lncRNA
XM_012103019.2	INPP5F	mRNA	XR_001041202.1	lncRNA
XM_012103019.2	INPP5F	mRNA	XR_001041202.1	lncRNA
XM_012103019.2	INPP5F	mRNA	XR_001041202.1	lncRNA
XM_012103019.2	INPP5F	mRNA	XR_001041202.1	lncRNA
XM_012103019.2	INPP5F	mRNA	XR_001041202.1	lncRNA
XM_012181720.2	ARID4A	mRNA	XR_001021939.1	lncRNA
XM_012181720.2	ARID4A	mRNA	XR_001021939.1	lncRNA
XM_012181720.2	ARID4A	mRNA	XR_001021939.1	lncRNA
XM_012181720.2	ARID4A	mRNA	XR_001021939.1	lncRNA
XM_012181720.2	ARID4A	mRNA	XR_001021939.1	lncRNA
XM_012181720.2	ARID4A	mRNA	XR_001021939.1	lncRNA
XM_012181720.2	ARID4A	mRNA	XR_001021939.1	lncRNA
XM_004006434.3	PUS7L	mRNA	XR_001021939.1	lncRNA
XM_004006434.3	PUS7L	mRNA	XR_001021939.1	lncRNA
XM_004006434.3	PUS7L	mRNA	XR_001021939.1	lncRNA
XM_004006434.3	PUS7L	mRNA	XR_001021939.1	lncRNA
XM_004006434.3	PUS7L	mRNA	XR_001021939.1	lncRNA
XM_004006434.3	PUS7L	mRNA	XR_001021939.1	lncRNA
XM_004006434.3	PUS7L	mRNA	XR_001021939.1	lncRNA
XM_012114904.1	RNF20	mRNA	XR_001041211.2	lncRNA
XM_012114904.1	RNF20	mRNA	XR_001041211.2	lncRNA
XM_012114904.1	RNF20	mRNA	XR_001041211.2	lncRNA
XM_012114904.1	RNF20	mRNA	XR_001041211.2	lncRNA
XM_012114904.1	RNF20	mRNA	XR_001041211.2	lncRNA
XM_012114904.1	RNF20	mRNA	XR_001041211.2	lncRNA
XM_012114904.1	RNF20	mRNA	XR_001041211.2	lncRNA
XM_012188154.2	FBXO18	mRNA	XR_001041211.2	lncRNA
XM_012188154.2	FBXO18	mRNA	XR_001041211.2	lncRNA
XM_012188154.2	FBXO18	mRNA	XR_001041211.2	lncRNA
XM_012188154.2	FBXO18	mRNA	XR_001041211.2	lncRNA
XM_012188154.2	FBXO18	mRNA	XR_001041211.2	lncRNA
XM_012188154.2	FBXO18	mRNA	XR_001041211.2	lncRNA
XM_012188154.2	FBXO18	mRNA	XR_001041211.2	lncRNA
XM_004017788.3	ZFANL3	mRNA	TCONS_00029399	lncRNA
XM_004017788.3	ZFANL3	mRNA	TCONS_00029399	lncRNA
XM_004017788.3	ZFANL3	mRNA	TCONS_00029399	lncRNA
XM_004017788.3	ZFANL3	mRNA	TCONS_00029399	lncRNA
XM_004017788.3	ZFANL3	mRNA	TCONS_00029399	lncRNA
XM_004017788.3	ZFANL3	mRNA	TCONS_00029399	lncRNA
XM_004017788.3	ZFANL3	mRNA	TCONS_00029399	lncRNA
XM_012146156.1	USP37	mRNA	XR_001041209.2	lncRNA
XM_012146156.1	USP37	mRNA	XR_001041209.2	lncRNA
XM_012146156.1	USP37	mRNA	XR_001041209.2	lncRNA

XM_012146156.1	USP37	mRNA	XR_001041209.2	lncRNA
XM_012146156.1	USP37	mRNA	XR_001041209.2	lncRNA
XM_012146156.1	USP37	mRNA	XR_001041209.2	lncRNA
XM_012146156.1	USP37	mRNA	XR_001041209.2	lncRNA
XM_015093350.1	GPR155	mRNA	XR_001041209.2	lncRNA
XM_015093350.1	GPR155	mRNA	XR_001041209.2	lncRNA
XM_015093350.1	GPR155	mRNA	XR_001041209.2	lncRNA
XM_015093350.1	GPR155	mRNA	XR_001041209.2	lncRNA
XM_015093350.1	GPR155	mRNA	XR_001041209.2	lncRNA
XM_015093350.1	GPR155	mRNA	XR_001041209.2	lncRNA
XM_015093350.1	GPR155	mRNA	XR_001041209.2	lncRNA
XM_004012194.3	RBM26	mRNA	XR_001041211.2	lncRNA
XM_004012194.3	RBM26	mRNA	XR_001041211.2	lncRNA
XM_004012194.3	RBM26	mRNA	XR_001041211.2	lncRNA
XM_004012194.3	RBM26	mRNA	XR_001041211.2	lncRNA
XM_004012194.3	RBM26	mRNA	XR_001041211.2	lncRNA
XM_004012194.3	RBM26	mRNA	XR_001041211.2	lncRNA
XM_004012194.3	RBM26	mRNA	XR_001041211.2	lncRNA
XM_012149251.2	S100PBP	mRNA	XR_001041209.2	lncRNA
XM_012149251.2	S100PBP	mRNA	XR_001041209.2	lncRNA
XM_012149251.2	S100PBP	mRNA	XR_001041209.2	lncRNA
XM_012149251.2	S100PBP	mRNA	XR_001041209.2	lncRNA
XM_012149251.2	S100PBP	mRNA	XR_001041209.2	lncRNA
XM_012149251.2	S100PBP	mRNA	XR_001041209.2	lncRNA
XM_012149251.2	S100PBP	mRNA	XR_001041209.2	lncRNA
XM_015092207.1	PRKAB2	mRNA	XR_001041211.2	lncRNA
XM_015092207.1	PRKAB2	mRNA	XR_001041211.2	lncRNA
XM_015092207.1	PRKAB2	mRNA	XR_001041211.2	lncRNA
XM_015092207.1	PRKAB2	mRNA	XR_001041211.2	lncRNA
XM_015092207.1	PRKAB2	mRNA	XR_001041211.2	lncRNA
XM_015092207.1	PRKAB2	mRNA	XR_001041211.2	lncRNA
XM_015092207.1	PRKAB2	mRNA	XR_001041211.2	lncRNA
XM_004001781.3	ZSCAN20	mRNA	XR_001041209.2	lncRNA
XM_004001781.3	ZSCAN20	mRNA	XR_001041209.2	lncRNA
XM_004001781.3	ZSCAN20	mRNA	XR_001041209.2	lncRNA
XM_004001781.3	ZSCAN20	mRNA	XR_001041209.2	lncRNA
XM_004001781.3	ZSCAN20	mRNA	XR_001041209.2	lncRNA
XM_004001781.3	ZSCAN20	mRNA	XR_001041209.2	lncRNA
XM_004001781.3	ZSCAN20	mRNA	XR_001041209.2	lncRNA
XM_012095713.2	RRAS2	mRNA	TCONS_00029399	lncRNA
XM_012095713.2	RRAS2	mRNA	TCONS_00029399	lncRNA
XM_012095713.2	RRAS2	mRNA	TCONS_00029399	lncRNA
XM_012095713.2	RRAS2	mRNA	TCONS_00029399	lncRNA
XM_012095713.2	RRAS2	mRNA	TCONS_00029399	lncRNA
XM_012095713.2	RRAS2	mRNA	TCONS_00029399	lncRNA
XM_012095713.2	RRAS2	mRNA	TCONS_00029399	lncRNA
XM_004004717.2	GTDC1	mRNA	TCONS_00071561	lncRNA
XM_004004717.2	GTDC1	mRNA	TCONS_00071561	lncRNA
XM_004004717.2	GTDC1	mRNA	TCONS_00071561	lncRNA
XM_004004717.2	GTDC1	mRNA	TCONS_00071561	lncRNA
XM_004004717.2	GTDC1	mRNA	TCONS_00071561	lncRNA
XM_004004717.2	GTDC1	mRNA	TCONS_00071561	lncRNA
XM_004004717.2	GTDC1	mRNA	TCONS_00071561	lncRNA
XM_015095780.1	DNAJB1	mRNA	XR_001021689.2	lncRNA

XM_015095780.1	DNAJB1	mRNA	XR_001021689.2	lncRNA
XM_015095780.1	DNAJB1	mRNA	XR_001021689.2	lncRNA
XM_015095780.1	DNAJB1	mRNA	XR_001021689.2	lncRNA
XM_015095780.1	DNAJB1	mRNA	XR_001021689.2	lncRNA
XM_015095780.1	DNAJB1	mRNA	XR_001021689.2	lncRNA
XM_015095780.1	DNAJB1	mRNA	XR_001021689.2	lncRNA
XM_004003157.3	TNIK	mRNA	XR_001021689.2	lncRNA
XM_004003157.3	TNIK	mRNA	XR_001021689.2	lncRNA
XM_004003157.3	TNIK	mRNA	XR_001021689.2	lncRNA
XM_004003157.3	TNIK	mRNA	XR_001021689.2	lncRNA
XM_004003157.3	TNIK	mRNA	XR_001021689.2	lncRNA
XM_004003157.3	TNIK	mRNA	XR_001021689.2	lncRNA
XM_004003157.3	TNIK	mRNA	XR_001021689.2	lncRNA
XM_012102471.1	ASAH2	mRNA	XR_001021689.2	lncRNA
XM_012102471.1	ASAH2	mRNA	XR_001021689.2	lncRNA
XM_012102471.1	ASAH2	mRNA	XR_001021689.2	lncRNA
XM_012102471.1	ASAH2	mRNA	XR_001021689.2	lncRNA
XM_012102471.1	ASAH2	mRNA	XR_001021689.2	lncRNA
XM_012102471.1	ASAH2	mRNA	XR_001021689.2	lncRNA
XM_012102471.1	ASAH2	mRNA	XR_001021689.2	lncRNA
XM_015102269.1	NKTR	mRNA	TCONS_00071561	lncRNA
XM_015102269.1	NKTR	mRNA	TCONS_00071561	lncRNA
XM_015102269.1	NKTR	mRNA	TCONS_00071561	lncRNA
XM_015102269.1	NKTR	mRNA	TCONS_00071561	lncRNA
XM_015102269.1	NKTR	mRNA	TCONS_00071561	lncRNA
XM_015102269.1	NKTR	mRNA	TCONS_00071561	lncRNA
XM_015102269.1	NKTR	mRNA	TCONS_00071561	lncRNA
XM_012095418.2	ZMYM4	mRNA	XR_001021689.2	lncRNA
XM_012095418.2	ZMYM4	mRNA	XR_001021689.2	lncRNA
XM_012095418.2	ZMYM4	mRNA	XR_001021689.2	lncRNA
XM_012095418.2	ZMYM4	mRNA	XR_001021689.2	lncRNA
XM_012095418.2	ZMYM4	mRNA	XR_001021689.2	lncRNA
XM_012095418.2	ZMYM4	mRNA	XR_001021689.2	lncRNA
XM_012174540.1	CPNE8	mRNA	XR_001021689.2	lncRNA
XM_012174540.1	CPNE8	mRNA	XR_001021689.2	lncRNA
XM_012174540.1	CPNE8	mRNA	XR_001021689.2	lncRNA
XM_012174540.1	CPNE8	mRNA	XR_001021689.2	lncRNA
XM_012174540.1	CPNE8	mRNA	XR_001021689.2	lncRNA
XM_012174540.1	CPNE8	mRNA	XR_001021689.2	lncRNA
XM_012182988.2	REPS1	mRNA	XR_001021689.2	lncRNA
XM_012182988.2	REPS1	mRNA	XR_001021689.2	lncRNA
XM_012182988.2	REPS1	mRNA	XR_001021689.2	lncRNA
XM_012182988.2	REPS1	mRNA	XR_001021689.2	lncRNA
XM_012182988.2	REPS1	mRNA	XR_001021689.2	lncRNA
XM_012182988.2	REPS1	mRNA	XR_001021689.2	lncRNA
XM_015101485.1	LOC1069916[illegible]	mRNA	XR_001436099.1	lncRNA
XM_015101485.1	LOC1069916[illegible]	mRNA	XR_001436099.1	lncRNA
XM_015101485.1	LOC1069916[illegible]	mRNA	XR_001436099.1	lncRNA
XM_015101485.1	LOC1069916[illegible]	mRNA	XR_001436099.1	lncRNA
XM_015101485.1	LOC1069916[illegible]	mRNA	XR_001436099.1	lncRNA
XM_015101485.1	LOC1069916[illegible]	mRNA	XR_001436099.1	lncRNA
XM_015101485.1	LOC1069916[illegible]	mRNA	XR_001436099.1	lncRNA
XM_015091899.1	SCMH1	mRNA	XR_001033284.2	lncRNA
XM_015091899.1	SCMH1	mRNA	XR_001033284.2	lncRNA

XM_015091899.1	SCMH1	mRNA	XR_001033284.2	lncRNA
XM_015091899.1	SCMH1	mRNA	XR_001033284.2	lncRNA
XM_015091899.1	SCMH1	mRNA	XR_001033284.2	lncRNA
XM_015091899.1	SCMH1	mRNA	XR_001033284.2	lncRNA
XM_015091899.1	SCMH1	mRNA	XR_001033284.2	lncRNA
XM_012102669.1	ZNF518A	mRNA	XR_001436099.1	lncRNA
XM_012102669.1	ZNF518A	mRNA	XR_001436099.1	lncRNA
XM_012102669.1	ZNF518A	mRNA	XR_001436099.1	lncRNA
XM_012102669.1	ZNF518A	mRNA	XR_001436099.1	lncRNA
XM_012102669.1	ZNF518A	mRNA	XR_001436099.1	lncRNA
XM_012102669.1	ZNF518A	mRNA	XR_001436099.1	lncRNA
XM_012102669.1	ZNF518A	mRNA	XR_001436099.1	lncRNA
XM_012174897.2	UTP20	mRNA	XR_001033284.2	lncRNA
XM_012174897.2	UTP20	mRNA	XR_001033284.2	lncRNA
XM_012174897.2	UTP20	mRNA	XR_001033284.2	lncRNA
XM_012174897.2	UTP20	mRNA	XR_001033284.2	lncRNA
XM_012174897.2	UTP20	mRNA	XR_001033284.2	lncRNA
XM_012174897.2	UTP20	mRNA	XR_001033284.2	lncRNA
XM_012174897.2	UTP20	mRNA	XR_001033284.2	lncRNA
XM_012190696.2	CCDC155	mRNA	XR_001033284.2	lncRNA
XM_012190696.2	CCDC155	mRNA	XR_001033284.2	lncRNA
XM_012190696.2	CCDC155	mRNA	XR_001033284.2	lncRNA
XM_012190696.2	CCDC155	mRNA	XR_001033284.2	lncRNA
XM_012190696.2	CCDC155	mRNA	XR_001033284.2	lncRNA
XM_012190696.2	CCDC155	mRNA	XR_001033284.2	lncRNA
XM_012190696.2	CCDC155	mRNA	XR_001033284.2	lncRNA
XM_004006434.3	PUS7L	mRNA	XR_001435104.1	lncRNA
XM_004006434.3	PUS7L	mRNA	XR_001435104.1	lncRNA
XM_004006434.3	PUS7L	mRNA	XR_001435104.1	lncRNA
XM_004006434.3	PUS7L	mRNA	XR_001435104.1	lncRNA
XM_004006434.3	PUS7L	mRNA	XR_001435104.1	lncRNA
XM_004006434.3	PUS7L	mRNA	XR_001435104.1	lncRNA
XM_004006434.3	PUS7L	mRNA	XR_001435104.1	lncRNA
XM_012181720.2	ARID4A	mRNA	XR_001435104.1	lncRNA
XM_012181720.2	ARID4A	mRNA	XR_001435104.1	lncRNA
XM_012181720.2	ARID4A	mRNA	XR_001435104.1	lncRNA
XM_012181720.2	ARID4A	mRNA	XR_001435104.1	lncRNA
XM_012181720.2	ARID4A	mRNA	XR_001435104.1	lncRNA
XM_012181720.2	ARID4A	mRNA	XR_001435104.1	lncRNA
XM_012181720.2	ARID4A	mRNA	XR_001435104.1	lncRNA
XM_012173383.2	DHX57	mRNA	XR_001435104.1	lncRNA
XM_012173383.2	DHX57	mRNA	XR_001435104.1	lncRNA
XM_012173383.2	DHX57	mRNA	XR_001435104.1	lncRNA
XM_012173383.2	DHX57	mRNA	XR_001435104.1	lncRNA
XM_012173383.2	DHX57	mRNA	XR_001435104.1	lncRNA
XM_012173383.2	DHX57	mRNA	XR_001435104.1	lncRNA
XM_012173383.2	DHX57	mRNA	XR_001435104.1	lncRNA
XM_012098142.2	YEATS2	mRNA	XR_001436531.1	lncRNA
XM_012098142.2	YEATS2	mRNA	XR_001436531.1	lncRNA
XM_012098142.2	YEATS2	mRNA	XR_001436531.1	lncRNA
XM_012098142.2	YEATS2	mRNA	XR_001436531.1	lncRNA
XM_012098142.2	YEATS2	mRNA	XR_001436531.1	lncRNA
XM_012098142.2	YEATS2	mRNA	XR_001436531.1	lncRNA
XM_012098142.2	YEATS2	mRNA	XR_001436531.1	lncRNA

XM_012174607.2	HELB	mRNA	XR_001436609.1	lncRNA
XM_012174607.2	HELB	mRNA	XR_001436609.1	lncRNA
XM_012174607.2	HELB	mRNA	XR_001436609.1	lncRNA
XM_012174607.2	HELB	mRNA	XR_001436609.1	lncRNA
XM_012174607.2	HELB	mRNA	XR_001436609.1	lncRNA
XM_012174607.2	HELB	mRNA	XR_001436609.1	lncRNA
XM_012174607.2	HELB	mRNA	XR_001436609.1	lncRNA
XM_012098017.2	KREMEN1	mRNA	XR_001436531.1	lncRNA
XM_012098017.2	KREMEN1	mRNA	XR_001436531.1	lncRNA
XM_012098017.2	KREMEN1	mRNA	XR_001436531.1	lncRNA
XM_012098017.2	KREMEN1	mRNA	XR_001436531.1	lncRNA
XM_012098017.2	KREMEN1	mRNA	XR_001436531.1	lncRNA
XM_012098017.2	KREMEN1	mRNA	XR_001436531.1	lncRNA
XM_012098017.2	KREMEN1	mRNA	XR_001436531.1	lncRNA
XM_012134593.2	FASTKD1	mRNA	XR_001435104.1	lncRNA
XM_012134593.2	FASTKD1	mRNA	XR_001435104.1	lncRNA
XM_012134593.2	FASTKD1	mRNA	XR_001435104.1	lncRNA
XM_012134593.2	FASTKD1	mRNA	XR_001435104.1	lncRNA
XM_012134593.2	FASTKD1	mRNA	XR_001435104.1	lncRNA
XM_012134593.2	FASTKD1	mRNA	XR_001435104.1	lncRNA
XM_012134593.2	FASTKD1	mRNA	XR_001435104.1	lncRNA
XM_012186944.1	DSTYK	mRNA	XR_001436609.1	lncRNA
XM_012186944.1	DSTYK	mRNA	XR_001436609.1	lncRNA
XM_012186944.1	DSTYK	mRNA	XR_001436609.1	lncRNA
XM_012186944.1	DSTYK	mRNA	XR_001436609.1	lncRNA
XM_012186944.1	DSTYK	mRNA	XR_001436609.1	lncRNA
XM_012186944.1	DSTYK	mRNA	XR_001436609.1	lncRNA
XM_012186944.1	DSTYK	mRNA	XR_001436609.1	lncRNA
XM_015103117.1	ZBTB44	mRNA	XR_001435104.1	lncRNA
XM_015103117.1	ZBTB44	mRNA	XR_001435104.1	lncRNA
XM_015103117.1	ZBTB44	mRNA	XR_001435104.1	lncRNA
XM_015103117.1	ZBTB44	mRNA	XR_001435104.1	lncRNA
XM_015103117.1	ZBTB44	mRNA	XR_001435104.1	lncRNA
XM_015103117.1	ZBTB44	mRNA	XR_001435104.1	lncRNA
XM_015103117.1	ZBTB44	mRNA	XR_001435104.1	lncRNA
XM_012174607.2	HELB	mRNA	XR_001436531.1	lncRNA
XM_012174607.2	HELB	mRNA	XR_001436531.1	lncRNA
XM_012174607.2	HELB	mRNA	XR_001436531.1	lncRNA
XM_012174607.2	HELB	mRNA	XR_001436531.1	lncRNA
XM_012174607.2	HELB	mRNA	XR_001436531.1	lncRNA
XM_012174607.2	HELB	mRNA	XR_001436531.1	lncRNA
XM_012174607.2	HELB	mRNA	XR_001436531.1	lncRNA
XM_012134593.2	FASTKD1	mRNA	XR_001436609.1	lncRNA
XM_012134593.2	FASTKD1	mRNA	XR_001436609.1	lncRNA
XM_012134593.2	FASTKD1	mRNA	XR_001436609.1	lncRNA
XM_012134593.2	FASTKD1	mRNA	XR_001436609.1	lncRNA
XM_012134593.2	FASTKD1	mRNA	XR_001436609.1	lncRNA
XM_012134593.2	FASTKD1	mRNA	XR_001436609.1	lncRNA
XM_012134593.2	FASTKD1	mRNA	XR_001436609.1	lncRNA
XM_012100126.2	MAP4	mRNA	XR_001435104.1	lncRNA
XM_012100126.2	MAP4	mRNA	XR_001435104.1	lncRNA
XM_012100126.2	MAP4	mRNA	XR_001435104.1	lncRNA
XM_012100126.2	MAP4	mRNA	XR_001435104.1	lncRNA
XM_012100126.2	MAP4	mRNA	XR_001435104.1	lncRNA

XM_012100126.2	MAP4	mRNA	XR_001435104.1	lncRNA
XM_012100126.2	MAP4	mRNA	XR_001435104.1	lncRNA
XM_015094782.1	MARS	mRNA	XR_001435104.1	lncRNA
XM_015094782.1	MARS	mRNA	XR_001435104.1	lncRNA
XM_015094782.1	MARS	mRNA	XR_001435104.1	lncRNA
XM_015094782.1	MARS	mRNA	XR_001435104.1	lncRNA
XM_015094782.1	MARS	mRNA	XR_001435104.1	lncRNA
XM_015094782.1	MARS	mRNA	XR_001435104.1	lncRNA
XM_015094782.1	MARS	mRNA	XR_001435104.1	lncRNA
XM_015093350.1	GPR155	mRNA	XR_001434081.1	lncRNA
XM_015093350.1	GPR155	mRNA	XR_001434081.1	lncRNA
XM_015093350.1	GPR155	mRNA	XR_001434081.1	lncRNA
XM_015093350.1	GPR155	mRNA	XR_001434081.1	lncRNA
XM_015093350.1	GPR155	mRNA	XR_001434081.1	lncRNA
XM_015093350.1	GPR155	mRNA	XR_001434081.1	lncRNA
XM_015093350.1	GPR155	mRNA	XR_001434081.1	lncRNA
XM_012095713.2	RRAS2	mRNA	XR_001434081.1	lncRNA
XM_012095713.2	RRAS2	mRNA	XR_001434081.1	lncRNA
XM_012095713.2	RRAS2	mRNA	XR_001434081.1	lncRNA
XM_012095713.2	RRAS2	mRNA	XR_001434081.1	lncRNA
XM_012095713.2	RRAS2	mRNA	XR_001434081.1	lncRNA
XM_012095713.2	RRAS2	mRNA	XR_001434081.1	lncRNA
XM_012095713.2	RRAS2	mRNA	XR_001434081.1	lncRNA
XM_012142889.2	ICA1L	mRNA	XR_001434081.1	lncRNA
XM_012142889.2	ICA1L	mRNA	XR_001434081.1	lncRNA
XM_012142889.2	ICA1L	mRNA	XR_001434081.1	lncRNA
XM_012142889.2	ICA1L	mRNA	XR_001434081.1	lncRNA
XM_012142889.2	ICA1L	mRNA	XR_001434081.1	lncRNA
XM_012142889.2	ICA1L	mRNA	XR_001434081.1	lncRNA
XM_012142889.2	ICA1L	mRNA	XR_001434081.1	lncRNA
XM_012186340.1	POLG2	mRNA	XR_001434081.1	lncRNA
XM_012186340.1	POLG2	mRNA	XR_001434081.1	lncRNA
XM_012186340.1	POLG2	mRNA	XR_001434081.1	lncRNA
XM_012186340.1	POLG2	mRNA	XR_001434081.1	lncRNA
XM_012186340.1	POLG2	mRNA	XR_001434081.1	lncRNA
XM_012186340.1	POLG2	mRNA	XR_001434081.1	lncRNA
XM_012186340.1	POLG2	mRNA	XR_001434081.1	lncRNA
XM_004017788.3	ZFANL3	mRNA	XR_001434081.1	lncRNA
XM_004017788.3	ZFANL3	mRNA	XR_001434081.1	lncRNA
XM_004017788.3	ZFANL3	mRNA	XR_001434081.1	lncRNA
XM_004017788.3	ZFANL3	mRNA	XR_001434081.1	lncRNA
XM_004017788.3	ZFANL3	mRNA	XR_001434081.1	lncRNA
XM_004017788.3	ZFANL3	mRNA	XR_001434081.1	lncRNA
XM_004017788.3	ZFANL3	mRNA	XR_001434081.1	lncRNA
XM_012177175.2	CALD1	mRNA	XR_001434081.1	lncRNA
XM_012177175.2	CALD1	mRNA	XR_001434081.1	lncRNA
XM_012177175.2	CALD1	mRNA	XR_001434081.1	lncRNA
XM_012177175.2	CALD1	mRNA	XR_001434081.1	lncRNA
XM_012177175.2	CALD1	mRNA	XR_001434081.1	lncRNA
XM_012177175.2	CALD1	mRNA	XR_001434081.1	lncRNA
XM_012177175.2	CALD1	mRNA	XR_001434081.1	lncRNA
XM_012184887.2	DOCK9	mRNA	XR_001021689.2	lncRNA
XM_012184887.2	DOCK9	mRNA	XR_001021689.2	lncRNA
XM_012184887.2	DOCK9	mRNA	XR_001021689.2	lncRNA

XM_012184887.2	DOCK9	mRNA	XR_001021689.2	lncRNA
XM_012184887.2	DOCK9	mRNA	XR_001021689.2	lncRNA
XM_012184887.2	DOCK9	mRNA	XR_001021689.2	lncRNA
XM_012184887.2	DOCK9	mRNA	XR_001021689.2	lncRNA
XM_015101011.1	TEAD1	mRNA	XR_001021689.2	lncRNA
XM_015101011.1	TEAD1	mRNA	XR_001021689.2	lncRNA
XM_015101011.1	TEAD1	mRNA	XR_001021689.2	lncRNA
XM_015101011.1	TEAD1	mRNA	XR_001021689.2	lncRNA
XM_015101011.1	TEAD1	mRNA	XR_001021689.2	lncRNA
XM_015101011.1	TEAD1	mRNA	XR_001021689.2	lncRNA
XM_015101011.1	TEAD1	mRNA	XR_001021689.2	lncRNA
XM_012187340.2	KIF1B	mRNA	XR_001021689.2	lncRNA
XM_012187340.2	KIF1B	mRNA	XR_001021689.2	lncRNA
XM_012187340.2	KIF1B	mRNA	XR_001021689.2	lncRNA
XM_012187340.2	KIF1B	mRNA	XR_001021689.2	lncRNA
XM_012187340.2	KIF1B	mRNA	XR_001021689.2	lncRNA
XM_012187340.2	KIF1B	mRNA	XR_001021689.2	lncRNA
XM_012187340.2	KIF1B	mRNA	XR_001021689.2	lncRNA
XM_012190192.2	PHLDB2	mRNA	TCONS_00071561	lncRNA
XM_012190192.2	PHLDB2	mRNA	TCONS_00071561	lncRNA
XM_012190192.2	PHLDB2	mRNA	TCONS_00071561	lncRNA
XM_012190192.2	PHLDB2	mRNA	TCONS_00071561	lncRNA
XM_012190192.2	PHLDB2	mRNA	TCONS_00071561	lncRNA
XM_012190192.2	PHLDB2	mRNA	TCONS_00071561	lncRNA
XM_012190192.2	PHLDB2	mRNA	TCONS_00071561	lncRNA
XM_012190083.2	ZMYM6	mRNA	XR_001041202.1	lncRNA
XM_012190083.2	ZMYM6	mRNA	XR_001041202.1	lncRNA
XM_012190083.2	ZMYM6	mRNA	XR_001041202.1	lncRNA
XM_012190083.2	ZMYM6	mRNA	XR_001041202.1	lncRNA
XM_012190083.2	ZMYM6	mRNA	XR_001041202.1	lncRNA
XM_012190083.2	ZMYM6	mRNA	XR_001041202.1	lncRNA
XM_012145933.2	CXCR2	mRNA	XR_001041202.1	lncRNA
XM_012145933.2	CXCR2	mRNA	XR_001041202.1	lncRNA
XM_012145933.2	CXCR2	mRNA	XR_001041202.1	lncRNA
XM_012145933.2	CXCR2	mRNA	XR_001041202.1	lncRNA
XM_012145933.2	CXCR2	mRNA	XR_001041202.1	lncRNA
XM_012145933.2	CXCR2	mRNA	XR_001041202.1	lncRNA
XM_015103117.1	ZBTB44	mRNA	XR_001042381.2	lncRNA
XM_015103117.1	ZBTB44	mRNA	XR_001042381.2	lncRNA
XM_015103117.1	ZBTB44	mRNA	XR_001042381.2	lncRNA
XM_015103117.1	ZBTB44	mRNA	XR_001042381.2	lncRNA
XM_015103117.1	ZBTB44	mRNA	XR_001042381.2	lncRNA
XM_015103117.1	ZBTB44	mRNA	XR_001042381.2	lncRNA
XM_015103117.1	ZBTB44	mRNA	XR_001042381.2	lncRNA
XM_012177139.2	TMEM209	mRNA	XR_001042381.2	lncRNA
XM_012177139.2	TMEM209	mRNA	XR_001042381.2	lncRNA
XM_012177139.2	TMEM209	mRNA	XR_001042381.2	lncRNA
XM_012177139.2	TMEM209	mRNA	XR_001042381.2	lncRNA
XM_012177139.2	TMEM209	mRNA	XR_001042381.2	lncRNA
XM_012177139.2	TMEM209	mRNA	XR_001042381.2	lncRNA
XM_012177139.2	TMEM209	mRNA	XR_001042381.2	lncRNA
XM_012173383.2	DHX57	mRNA	XR_001042381.2	lncRNA
XM_012173383.2	DHX57	mRNA	XR_001042381.2	lncRNA
XM_012173383.2	DHX57	mRNA	XR_001042381.2	lncRNA

XM_012173383.2	DHX57	mRNA	XR_001042381.2	lncRNA
XM_012173383.2	DHX57	mRNA	XR_001042381.2	lncRNA
XM_012173383.2	DHX57	mRNA	XR_001042381.2	lncRNA
XM_012173383.2	DHX57	mRNA	XR_001042381.2	lncRNA
XM_012105225.2	SGPL1	mRNA	XR_001042381.2	lncRNA
XM_012105225.2	SGPL1	mRNA	XR_001042381.2	lncRNA
XM_012105225.2	SGPL1	mRNA	XR_001042381.2	lncRNA
XM_012105225.2	SGPL1	mRNA	XR_001042381.2	lncRNA
XM_012105225.2	SGPL1	mRNA	XR_001042381.2	lncRNA
XM_012105225.2	SGPL1	mRNA	XR_001042381.2	lncRNA
XM_012105225.2	SGPL1	mRNA	XR_001042381.2	lncRNA
XM_015093495.1	NBEAL1	mRNA	TCONS_00029399	lncRNA
XM_015093495.1	NBEAL1	mRNA	TCONS_00029399	lncRNA
XM_015093495.1	NBEAL1	mRNA	TCONS_00029399	lncRNA
XM_015093495.1	NBEAL1	mRNA	TCONS_00029399	lncRNA
XM_015093495.1	NBEAL1	mRNA	TCONS_00029399	lncRNA
XM_015093495.1	NBEAL1	mRNA	TCONS_00029399	lncRNA
XM_015093495.1	NBEAL1	mRNA	TCONS_00029399	lncRNA
XM_012181829.2	ZBTB1	mRNA	XR_001041209.2	lncRNA
XM_012181829.2	ZBTB1	mRNA	XR_001041209.2	lncRNA
XM_012181829.2	ZBTB1	mRNA	XR_001041209.2	lncRNA
XM_012181829.2	ZBTB1	mRNA	XR_001041209.2	lncRNA
XM_012181829.2	ZBTB1	mRNA	XR_001041209.2	lncRNA
XM_012181829.2	ZBTB1	mRNA	XR_001041209.2	lncRNA
XM_012181829.2	ZBTB1	mRNA	XR_001041209.2	lncRNA
XM_012182215.2	TDP1	mRNA	XR_001041209.2	lncRNA
XM_012182215.2	TDP1	mRNA	XR_001041209.2	lncRNA
XM_012182215.2	TDP1	mRNA	XR_001041209.2	lncRNA
XM_012182215.2	TDP1	mRNA	XR_001041209.2	lncRNA
XM_012182215.2	TDP1	mRNA	XR_001041209.2	lncRNA
XM_012182215.2	TDP1	mRNA	XR_001041209.2	lncRNA
XM_012182215.2	TDP1	mRNA	XR_001041209.2	lncRNA
XM_012182238.1	SPG11	mRNA	XR_001041211.2	lncRNA
XM_012182238.1	SPG11	mRNA	XR_001041211.2	lncRNA
XM_012182238.1	SPG11	mRNA	XR_001041211.2	lncRNA
XM_012182238.1	SPG11	mRNA	XR_001041211.2	lncRNA
XM_012182238.1	SPG11	mRNA	XR_001041211.2	lncRNA
XM_012182238.1	SPG11	mRNA	XR_001041211.2	lncRNA
XM_012182238.1	SPG11	mRNA	XR_001041211.2	lncRNA
XM_012181829.2	ZBTB1	mRNA	TCONS_00029399	lncRNA
XM_012181829.2	ZBTB1	mRNA	TCONS_00029399	lncRNA
XM_012181829.2	ZBTB1	mRNA	TCONS_00029399	lncRNA
XM_012181829.2	ZBTB1	mRNA	TCONS_00029399	lncRNA
XM_012181829.2	ZBTB1	mRNA	TCONS_00029399	lncRNA
XM_012181829.2	ZBTB1	mRNA	TCONS_00029399	lncRNA
XM_012181829.2	ZBTB1	mRNA	TCONS_00029399	lncRNA
XM_004006165.3	ITPRIPL1	mRNA	XR_001041211.2	lncRNA
XM_004006165.3	ITPRIPL1	mRNA	XR_001041211.2	lncRNA
XM_004006165.3	ITPRIPL1	mRNA	XR_001041211.2	lncRNA
XM_004006165.3	ITPRIPL1	mRNA	XR_001041211.2	lncRNA
XM_004006165.3	ITPRIPL1	mRNA	XR_001041211.2	lncRNA
XM_004006165.3	ITPRIPL1	mRNA	XR_001041211.2	lncRNA
XM_004006165.3	ITPRIPL1	mRNA	XR_001041211.2	lncRNA
XM_012188917.1	STX16	mRNA	TCONS_00029399	lncRNA

XM_012188917.1	STX16	mRNA	TCONS_00029399	lncRNA
XM_012188917.1	STX16	mRNA	TCONS_00029399	lncRNA
XM_012188917.1	STX16	mRNA	TCONS_00029399	lncRNA
XM_012188917.1	STX16	mRNA	TCONS_00029399	lncRNA
XM_012188917.1	STX16	mRNA	TCONS_00029399	lncRNA
XM_012188917.1	STX16	mRNA	TCONS_00029399	lncRNA
XM_012181420.2	ICE2	mRNA	XR_001041209.2	lncRNA
XM_012181420.2	ICE2	mRNA	XR_001041209.2	lncRNA
XM_012181420.2	ICE2	mRNA	XR_001041209.2	lncRNA
XM_012181420.2	ICE2	mRNA	XR_001041209.2	lncRNA
XM_012181420.2	ICE2	mRNA	XR_001041209.2	lncRNA
XM_012181420.2	ICE2	mRNA	XR_001041209.2	lncRNA
XM_012181420.2	ICE2	mRNA	XR_001041209.2	lncRNA
XM_015096057.1	KDM3B	mRNA	XR_001033284.2	lncRNA
XM_015096057.1	KDM3B	mRNA	XR_001033284.2	lncRNA
XM_015096057.1	KDM3B	mRNA	XR_001033284.2	lncRNA
XM_015096057.1	KDM3B	mRNA	XR_001033284.2	lncRNA
XM_015096057.1	KDM3B	mRNA	XR_001033284.2	lncRNA
XM_015096057.1	KDM3B	mRNA	XR_001033284.2	lncRNA
XM_015096057.1	KDM3B	mRNA	XR_001033284.2	lncRNA
XM_012152455.2	LUZP1	mRNA	XR_001033284.2	lncRNA
XM_012152455.2	LUZP1	mRNA	XR_001033284.2	lncRNA
XM_012152455.2	LUZP1	mRNA	XR_001033284.2	lncRNA
XM_012152455.2	LUZP1	mRNA	XR_001033284.2	lncRNA
XM_012152455.2	LUZP1	mRNA	XR_001033284.2	lncRNA
XM_012152455.2	LUZP1	mRNA	XR_001033284.2	lncRNA
XM_012152455.2	LUZP1	mRNA	XR_001033284.2	lncRNA
XM_015095269.1	NRCAM	mRNA	XR_001436099.1	lncRNA
XM_015095269.1	NRCAM	mRNA	XR_001436099.1	lncRNA
XM_015095269.1	NRCAM	mRNA	XR_001436099.1	lncRNA
XM_015095269.1	NRCAM	mRNA	XR_001436099.1	lncRNA
XM_015095269.1	NRCAM	mRNA	XR_001436099.1	lncRNA
XM_015095269.1	NRCAM	mRNA	XR_001436099.1	lncRNA
XM_015095269.1	NRCAM	mRNA	XR_001436099.1	lncRNA
XM_012190515.2	ZMYM1	mRNA	XR_001436099.1	lncRNA
XM_012190515.2	ZMYM1	mRNA	XR_001436099.1	lncRNA
XM_012190515.2	ZMYM1	mRNA	XR_001436099.1	lncRNA
XM_012190515.2	ZMYM1	mRNA	XR_001436099.1	lncRNA
XM_012190515.2	ZMYM1	mRNA	XR_001436099.1	lncRNA
XM_012190515.2	ZMYM1	mRNA	XR_001436099.1	lncRNA
XM_012190515.2	ZMYM1	mRNA	XR_001033284.2	lncRNA
XM_012190515.2	ZMYM1	mRNA	XR_001033284.2	lncRNA
XM_012190515.2	ZMYM1	mRNA	XR_001033284.2	lncRNA
XM_012190515.2	ZMYM1	mRNA	XR_001033284.2	lncRNA
XM_012190515.2	ZMYM1	mRNA	XR_001033284.2	lncRNA
XM_012190515.2	ZMYM1	mRNA	XR_001033284.2	lncRNA
XM_012182988.2	REPS1	mRNA	XR_001033284.2	lncRNA
XM_012182988.2	REPS1	mRNA	XR_001033284.2	lncRNA
XM_012182988.2	REPS1	mRNA	XR_001033284.2	lncRNA
XM_012182988.2	REPS1	mRNA	XR_001033284.2	lncRNA
XM_012182988.2	REPS1	mRNA	XR_001033284.2	lncRNA
XM_012182988.2	REPS1	mRNA	XR_001033284.2	lncRNA
XM_012104222.2	DCUN1D3	mRNA	XR_001033284.2	lncRNA
XM_012104222.2	DCUN1D3	mRNA	XR_001033284.2	lncRNA

XM_012104222.2	DCUN1D3	mRNA	XR_001033284.2	lncRNA
XM_012104222.2	DCUN1D3	mRNA	XR_001033284.2	lncRNA
XM_012104222.2	DCUN1D3	mRNA	XR_001033284.2	lncRNA
XM_012104222.2	DCUN1D3	mRNA	XR_001033284.2	lncRNA
XM_012176469.2	MACC1	mRNA	XR_001033284.2	lncRNA
XM_012176469.2	MACC1	mRNA	XR_001033284.2	lncRNA
XM_012176469.2	MACC1	mRNA	XR_001033284.2	lncRNA
XM_012176469.2	MACC1	mRNA	XR_001033284.2	lncRNA
XM_012176469.2	MACC1	mRNA	XR_001033284.2	lncRNA
XM_012176469.2	MACC1	mRNA	XR_001033284.2	lncRNA
XM_012102560.2	CEP70	mRNA	XR_001033284.2	lncRNA
XM_012102560.2	CEP70	mRNA	XR_001033284.2	lncRNA
XM_012102560.2	CEP70	mRNA	XR_001033284.2	lncRNA
XM_012102560.2	CEP70	mRNA	XR_001033284.2	lncRNA
XM_012102560.2	CEP70	mRNA	XR_001033284.2	lncRNA
XM_012102560.2	CEP70	mRNA	XR_001033284.2	lncRNA
XM_012105802.2	PCM1	mRNA	XR_001021689.2	lncRNA
XM_012105802.2	PCM1	mRNA	XR_001021689.2	lncRNA
XM_012105802.2	PCM1	mRNA	XR_001021689.2	lncRNA
XM_012105802.2	PCM1	mRNA	XR_001021689.2	lncRNA
XM_012105802.2	PCM1	mRNA	XR_001021689.2	lncRNA
XM_012105802.2	PCM1	mRNA	XR_001021689.2	lncRNA
XM_012105800.2	PCM1	mRNA	XR_001021689.2	lncRNA
XM_012105800.2	PCM1	mRNA	XR_001021689.2	lncRNA
XM_012105800.2	PCM1	mRNA	XR_001021689.2	lncRNA
XM_012105800.2	PCM1	mRNA	XR_001021689.2	lncRNA
XM_012105800.2	PCM1	mRNA	XR_001021689.2	lncRNA
XM_012105800.2	PCM1	mRNA	XR_001021689.2	lncRNA
XM_012101112.2	ABT1	mRNA	XR_001041209.2	lncRNA
XM_012101112.2	ABT1	mRNA	XR_001041209.2	lncRNA
XM_012101112.2	ABT1	mRNA	XR_001041209.2	lncRNA
XM_012101112.2	ABT1	mRNA	XR_001041209.2	lncRNA
XM_012101112.2	ABT1	mRNA	XR_001041209.2	lncRNA
XM_012101112.2	ABT1	mRNA	XR_001041209.2	lncRNA
XM_012101112.2	ABT1	mRNA	XR_001041209.2	lncRNA
XM_012176365.2	SLC25A13	mRNA	XR_001041209.2	lncRNA
XM_012176365.2	SLC25A13	mRNA	XR_001041209.2	lncRNA
XM_012176365.2	SLC25A13	mRNA	XR_001041209.2	lncRNA
XM_012176365.2	SLC25A13	mRNA	XR_001041209.2	lncRNA
XM_012176365.2	SLC25A13	mRNA	XR_001041209.2	lncRNA
XM_012176365.2	SLC25A13	mRNA	XR_001041209.2	lncRNA
XM_012176365.2	SLC25A13	mRNA	XR_001041209.2	lncRNA
XM_012098886.2	TP53BP1	mRNA	XR_001041209.2	lncRNA
XM_012098886.2	TP53BP1	mRNA	XR_001041209.2	lncRNA
XM_012098886.2	TP53BP1	mRNA	XR_001041209.2	lncRNA
XM_012098886.2	TP53BP1	mRNA	XR_001041209.2	lncRNA
XM_012098886.2	TP53BP1	mRNA	XR_001041209.2	lncRNA
XM_012098886.2	TP53BP1	mRNA	XR_001041209.2	lncRNA
XM_012098886.2	TP53BP1	mRNA	XR_001041209.2	lncRNA
XM_012187292.1	VPS13D	mRNA	XR_001041211.2	lncRNA
XM_012187292.1	VPS13D	mRNA	XR_001041211.2	lncRNA
XM_012187292.1	VPS13D	mRNA	XR_001041211.2	lncRNA
XM_012187292.1	VPS13D	mRNA	XR_001041211.2	lncRNA
XM_012187292.1	VPS13D	mRNA	XR_001041211.2	lncRNA

XM_012187292.1	VPS13D	mRNA	XR_001041211.2	lncRNA
XM_012187292.1	VPS13D	mRNA	XR_001041211.2	lncRNA
XM_012137599.2	NEB	mRNA	XR_001436099.1	lncRNA
XM_012137599.2	NEB	mRNA	XR_001436099.1	lncRNA
XM_012137599.2	NEB	mRNA	XR_001436099.1	lncRNA
XM_012137599.2	NEB	mRNA	XR_001436099.1	lncRNA
XM_012137599.2	NEB	mRNA	XR_001436099.1	lncRNA
XM_012137599.2	NEB	mRNA	XR_001436099.1	lncRNA
XM_012137599.2	NEB	mRNA	XR_001436099.1	lncRNA
XM_012096744.2	GFM2	mRNA	XR_001041209.2	lncRNA
XM_012096744.2	GFM2	mRNA	XR_001041209.2	lncRNA
XM_012096744.2	GFM2	mRNA	XR_001041209.2	lncRNA
XM_012096744.2	GFM2	mRNA	XR_001041209.2	lncRNA
XM_012096744.2	GFM2	mRNA	XR_001041209.2	lncRNA
XM_012096744.2	GFM2	mRNA	XR_001041209.2	lncRNA
XM_015097359.1	ARMC2	mRNA	XR_001041209.2	lncRNA
XM_015097359.1	ARMC2	mRNA	XR_001041209.2	lncRNA
XM_015097359.1	ARMC2	mRNA	XR_001041209.2	lncRNA
XM_015097359.1	ARMC2	mRNA	XR_001041209.2	lncRNA
XM_015097359.1	ARMC2	mRNA	XR_001041209.2	lncRNA
XM_015097359.1	ARMC2	mRNA	XR_001041209.2	lncRNA
XM_012181536.2	FAM214A	mRNA	XR_001041209.2	lncRNA
XM_012181536.2	FAM214A	mRNA	XR_001041209.2	lncRNA
XM_012181536.2	FAM214A	mRNA	XR_001041209.2	lncRNA
XM_012181536.2	FAM214A	mRNA	XR_001041209.2	lncRNA
XM_012181536.2	FAM214A	mRNA	XR_001041209.2	lncRNA
XM_012181536.2	FAM214A	mRNA	XR_001041209.2	lncRNA
XM_012188664.2	RASSF2	mRNA	XR_001041209.2	lncRNA
XM_012188664.2	RASSF2	mRNA	XR_001041209.2	lncRNA
XM_012188664.2	RASSF2	mRNA	XR_001041209.2	lncRNA
XM_012188664.2	RASSF2	mRNA	XR_001041209.2	lncRNA
XM_012188664.2	RASSF2	mRNA	XR_001041209.2	lncRNA
XM_012188664.2	RASSF2	mRNA	XR_001041209.2	lncRNA
XM_012096744.2	GFM2	mRNA	TCONS_00029399	lncRNA
XM_012096744.2	GFM2	mRNA	TCONS_00029399	lncRNA
XM_012096744.2	GFM2	mRNA	TCONS_00029399	lncRNA
XM_012096744.2	GFM2	mRNA	TCONS_00029399	lncRNA
XM_012096744.2	GFM2	mRNA	TCONS_00029399	lncRNA
XM_012096744.2	GFM2	mRNA	TCONS_00029399	lncRNA
XM_012145933.2	CXCR2	mRNA	XR_001041211.2	lncRNA
XM_012145933.2	CXCR2	mRNA	XR_001041211.2	lncRNA
XM_012145933.2	CXCR2	mRNA	XR_001041211.2	lncRNA
XM_012145933.2	CXCR2	mRNA	XR_001041211.2	lncRNA
XM_012145933.2	CXCR2	mRNA	XR_001041211.2	lncRNA
XM_012145933.2	CXCR2	mRNA	XR_001041211.2	lncRNA
XM_015104393.1	RUFY2	mRNA	XR_001435726.1	lncRNA
XM_015104393.1	RUFY2	mRNA	XR_001435726.1	lncRNA
XM_015104393.1	RUFY2	mRNA	XR_001435726.1	lncRNA
XM_015104393.1	RUFY2	mRNA	XR_001435726.1	lncRNA
XM_015104393.1	RUFY2	mRNA	XR_001435726.1	lncRNA
XM_015104393.1	RUFY2	mRNA	XR_001435726.1	lncRNA
XM_015104393.1	RUFY2	mRNA	XR_001435726.1	lncRNA
XM_015104393.1	RUFY2	mRNA	XR_001435723.1	lncRNA
XM_015104393.1	RUFY2	mRNA	XR_001435723.1	lncRNA

XM_015104393.1	RUFY2	mRNA	XR_001435723.1	lncRNA
XM_015104393.1	RUFY2	mRNA	XR_001435723.1	lncRNA
XM_015104393.1	RUFY2	mRNA	XR_001435723.1	lncRNA
XM_015104393.1	RUFY2	mRNA	XR_001435723.1	lncRNA
XM_015104393.1	RUFY2	mRNA	XR_001435723.1	lncRNA
XM_012190192.2	PHLDB2	mRNA	XR_001033284.2	lncRNA
XM_012190192.2	PHLDB2	mRNA	XR_001033284.2	lncRNA
XM_012190192.2	PHLDB2	mRNA	XR_001033284.2	lncRNA
XM_012190192.2	PHLDB2	mRNA	XR_001033284.2	lncRNA
XM_012190192.2	PHLDB2	mRNA	XR_001033284.2	lncRNA
XM_012190192.2	PHLDB2	mRNA	XR_001033284.2	lncRNA
XM_012190192.2	PHLDB2	mRNA	XR_001033284.2	lncRNA
XM_012137673.2	NEB	mRNA	TCONS_00029399	lncRNA
XM_012137673.2	NEB	mRNA	TCONS_00029399	lncRNA
XM_012137673.2	NEB	mRNA	TCONS_00029399	lncRNA
XM_012137673.2	NEB	mRNA	TCONS_00029399	lncRNA
XM_012137673.2	NEB	mRNA	TCONS_00029399	lncRNA
XM_012137673.2	NEB	mRNA	TCONS_00029399	lncRNA
XM_012137673.2	NEB	mRNA	TCONS_00029399	lncRNA
XM_015096769.1	APC	mRNA	TCONS_00029399	lncRNA
XM_015096769.1	APC	mRNA	TCONS_00029399	lncRNA
XM_015096769.1	APC	mRNA	TCONS_00029399	lncRNA
XM_015096769.1	APC	mRNA	TCONS_00029399	lncRNA
XM_015096769.1	APC	mRNA	TCONS_00029399	lncRNA
XM_015096769.1	APC	mRNA	TCONS_00029399	lncRNA
XM_015096769.1	APC	mRNA	TCONS_00029399	lncRNA
XM_015095780.1	DNAJB1	mRNA	XR_001041209.2	lncRNA
XM_015095780.1	DNAJB1	mRNA	XR_001041209.2	lncRNA
XM_015095780.1	DNAJB1	mRNA	XR_001041209.2	lncRNA
XM_015095780.1	DNAJB1	mRNA	XR_001041209.2	lncRNA
XM_015095780.1	DNAJB1	mRNA	XR_001041209.2	lncRNA
XM_015095780.1	DNAJB1	mRNA	XR_001041209.2	lncRNA
XM_015095780.1	DNAJB1	mRNA	XR_001041209.2	lncRNA
XM_015095780.1	DNAJB1	mRNA	TCONS_00029399	lncRNA
XM_015095780.1	DNAJB1	mRNA	TCONS_00029399	lncRNA
XM_015095780.1	DNAJB1	mRNA	TCONS_00029399	lncRNA
XM_015095780.1	DNAJB1	mRNA	TCONS_00029399	lncRNA
XM_015095780.1	DNAJB1	mRNA	TCONS_00029399	lncRNA
XM_015095780.1	DNAJB1	mRNA	TCONS_00029399	lncRNA
XM_015095780.1	DNAJB1	mRNA	TCONS_00029399	lncRNA
XM_015092197.1	CD58	mRNA	XR_001434081.1	lncRNA
XM_015092197.1	CD58	mRNA	XR_001434081.1	lncRNA
XM_015092197.1	CD58	mRNA	XR_001434081.1	lncRNA
XM_015092197.1	CD58	mRNA	XR_001434081.1	lncRNA
XM_015092197.1	CD58	mRNA	XR_001434081.1	lncRNA
XM_015092197.1	CD58	mRNA	XR_001434081.1	lncRNA
XM_012095272.2	ARNTL	mRNA	XR_001434081.1	lncRNA
XM_012095272.2	ARNTL	mRNA	XR_001434081.1	lncRNA
XM_012095272.2	ARNTL	mRNA	XR_001434081.1	lncRNA
XM_012095272.2	ARNTL	mRNA	XR_001434081.1	lncRNA
XM_012095272.2	ARNTL	mRNA	XR_001434081.1	lncRNA
XM_012095272.2	ARNTL	mRNA	XR_001434081.1	lncRNA
XM_012176296.1	AKAP9	mRNA	XR_001039281.1	lncRNA
XM_012176296.1	AKAP9	mRNA	XR_001039281.1	lncRNA

XM_012176296.1	AKAP9	mRNA	XR_001039281.1	lncRNA
XM_012176296.1	AKAP9	mRNA	XR_001039281.1	lncRNA
XM_012176296.1	AKAP9	mRNA	XR_001039281.1	lncRNA
XM_012176296.1	AKAP9	mRNA	XR_001039281.1	lncRNA
XM_012187340.2	KIF1B	mRNA	TCONS_00072289	lncRNA
XM_012187340.2	KIF1B	mRNA	TCONS_00072289	lncRNA
XM_012187340.2	KIF1B	mRNA	TCONS_00072289	lncRNA
XM_012187340.2	KIF1B	mRNA	TCONS_00072289	lncRNA
XM_012187340.2	KIF1B	mRNA	TCONS_00072289	lncRNA
XM_012187340.2	KIF1B	mRNA	TCONS_00072289	lncRNA
XM_015091887.1	MYCL	mRNA	TCONS_00072289	lncRNA
XM_015091887.1	MYCL	mRNA	TCONS_00072289	lncRNA
XM_015091887.1	MYCL	mRNA	TCONS_00072289	lncRNA
XM_015091887.1	MYCL	mRNA	TCONS_00072289	lncRNA
XM_015091887.1	MYCL	mRNA	TCONS_00072289	lncRNA
XM_015091887.1	MYCL	mRNA	TCONS_00072289	lncRNA
XM_012100126.2	MAP4	mRNA	XR_001021689.2	lncRNA
XM_012100126.2	MAP4	mRNA	XR_001021689.2	lncRNA
XM_012100126.2	MAP4	mRNA	XR_001021689.2	lncRNA
XM_012100126.2	MAP4	mRNA	XR_001021689.2	lncRNA
XM_012100126.2	MAP4	mRNA	XR_001021689.2	lncRNA
XM_012100126.2	MAP4	mRNA	XR_001021689.2	lncRNA
XM_012173383.2	DHX57	mRNA	XR_001021689.2	lncRNA
XM_012173383.2	DHX57	mRNA	XR_001021689.2	lncRNA
XM_012173383.2	DHX57	mRNA	XR_001021689.2	lncRNA
XM_012173383.2	DHX57	mRNA	XR_001021689.2	lncRNA
XM_012173383.2	DHX57	mRNA	XR_001021689.2	lncRNA
XM_012173383.2	DHX57	mRNA	XR_001021689.2	lncRNA
XM_012181866.1	GPHN	mRNA	XR_001021689.2	lncRNA
XM_012181866.1	GPHN	mRNA	XR_001021689.2	lncRNA
XM_012181866.1	GPHN	mRNA	XR_001021689.2	lncRNA
XM_012181866.1	GPHN	mRNA	XR_001021689.2	lncRNA
XM_012181866.1	GPHN	mRNA	XR_001021689.2	lncRNA
XM_012181866.1	GPHN	mRNA	XR_001021689.2	lncRNA
XM_012097468.2	ELF2	mRNA	XR_001021689.2	lncRNA
XM_012097468.2	ELF2	mRNA	XR_001021689.2	lncRNA
XM_012097468.2	ELF2	mRNA	XR_001021689.2	lncRNA
XM_012097468.2	ELF2	mRNA	XR_001021689.2	lncRNA
XM_012097468.2	ELF2	mRNA	XR_001021689.2	lncRNA
XM_012097468.2	ELF2	mRNA	XR_001021689.2	lncRNA
XM_012176678.2	KMT2E	mRNA	TCONS_00071561	lncRNA
XM_012176678.2	KMT2E	mRNA	TCONS_00071561	lncRNA
XM_012176678.2	KMT2E	mRNA	TCONS_00071561	lncRNA
XM_012176678.2	KMT2E	mRNA	TCONS_00071561	lncRNA
XM_012176678.2	KMT2E	mRNA	TCONS_00071561	lncRNA
XM_012176678.2	KMT2E	mRNA	TCONS_00071561	lncRNA
XM_012104222.2	DCUN1D3	mRNA	XR_001021939.1	lncRNA
XM_012104222.2	DCUN1D3	mRNA	XR_001021939.1	lncRNA
XM_012104222.2	DCUN1D3	mRNA	XR_001021939.1	lncRNA
XM_012104222.2	DCUN1D3	mRNA	XR_001021939.1	lncRNA
XM_012104222.2	DCUN1D3	mRNA	XR_001021939.1	lncRNA
XM_012104222.2	DCUN1D3	mRNA	XR_001021939.1	lncRNA
XM_012184485.2	RNASEH2B	mRNA	XR_001021939.1	lncRNA
XM_012184485.2	RNASEH2B	mRNA	XR_001021939.1	lncRNA

XM_012184485.2	RNASEH2B	mRNA	XR_001021939.1	lncRNA
XM_012184485.2	RNASEH2B	mRNA	XR_001021939.1	lncRNA
XM_012184485.2	RNASEH2B	mRNA	XR_001021939.1	lncRNA
XM_012184485.2	RNASEH2B	mRNA	XR_001021939.1	lncRNA
XM_012176469.2	MACC1	mRNA	XR_001021939.1	lncRNA
XM_012176469.2	MACC1	mRNA	XR_001021939.1	lncRNA
XM_012176469.2	MACC1	mRNA	XR_001021939.1	lncRNA
XM_012176469.2	MACC1	mRNA	XR_001021939.1	lncRNA
XM_012176469.2	MACC1	mRNA	XR_001021939.1	lncRNA
XM_012176469.2	MACC1	mRNA	XR_001021939.1	lncRNA
XM_012189374.1	NXPE3	mRNA	XR_001021939.1	lncRNA
XM_012189374.1	NXPE3	mRNA	XR_001021939.1	lncRNA
XM_012189374.1	NXPE3	mRNA	XR_001021939.1	lncRNA
XM_012189374.1	NXPE3	mRNA	XR_001021939.1	lncRNA
XM_012189374.1	NXPE3	mRNA	XR_001021939.1	lncRNA
XM_012189374.1	NXPE3	mRNA	XR_001021939.1	lncRNA
XM_012184629.2	USPL1	mRNA	XR_001021939.1	lncRNA
XM_012184629.2	USPL1	mRNA	XR_001021939.1	lncRNA
XM_012184629.2	USPL1	mRNA	XR_001021939.1	lncRNA
XM_012184629.2	USPL1	mRNA	XR_001021939.1	lncRNA
XM_012184629.2	USPL1	mRNA	XR_001021939.1	lncRNA
XM_012184629.2	USPL1	mRNA	XR_001021939.1	lncRNA
XM_012107777.1	NFYC	mRNA	XR_001021939.1	lncRNA
XM_012107777.1	NFYC	mRNA	XR_001021939.1	lncRNA
XM_012107777.1	NFYC	mRNA	XR_001021939.1	lncRNA
XM_012107777.1	NFYC	mRNA	XR_001021939.1	lncRNA
XM_012107777.1	NFYC	mRNA	XR_001021939.1	lncRNA
XM_012107777.1	NFYC	mRNA	XR_001021939.1	lncRNA
XM_015092641.1	SLC25A36	mRNA	XR_001436099.1	lncRNA
XM_015092641.1	SLC25A36	mRNA	XR_001436099.1	lncRNA
XM_015092641.1	SLC25A36	mRNA	XR_001436099.1	lncRNA
XM_015092641.1	SLC25A36	mRNA	XR_001436099.1	lncRNA
XM_015092641.1	SLC25A36	mRNA	XR_001436099.1	lncRNA
XM_015092641.1	SLC25A36	mRNA	XR_001436099.1	lncRNA
XM_012188017.2	ZNF654	mRNA	XR_001436099.1	lncRNA
XM_012188017.2	ZNF654	mRNA	XR_001436099.1	lncRNA
XM_012188017.2	ZNF654	mRNA	XR_001436099.1	lncRNA
XM_012188017.2	ZNF654	mRNA	XR_001436099.1	lncRNA
XM_012188017.2	ZNF654	mRNA	XR_001436099.1	lncRNA
XM_012188017.2	ZNF654	mRNA	XR_001436099.1	lncRNA
XM_012095858.2	ZNF214	mRNA	XR_001033284.2	lncRNA
XM_012095858.2	ZNF214	mRNA	XR_001033284.2	lncRNA
XM_012095858.2	ZNF214	mRNA	XR_001033284.2	lncRNA
XM_012095858.2	ZNF214	mRNA	XR_001033284.2	lncRNA
XM_012095858.2	ZNF214	mRNA	XR_001033284.2	lncRNA
XM_012095858.2	ZNF214	mRNA	XR_001033284.2	lncRNA
XM_012188017.2	ZNF654	mRNA	XR_001033284.2	lncRNA
XM_012188017.2	ZNF654	mRNA	XR_001033284.2	lncRNA
XM_012188017.2	ZNF654	mRNA	XR_001033284.2	lncRNA
XM_012188017.2	ZNF654	mRNA	XR_001033284.2	lncRNA
XM_012188017.2	ZNF654	mRNA	XR_001033284.2	lncRNA
XM_012188017.2	ZNF654	mRNA	XR_001033284.2	lncRNA
XM_015097252.1	SLC17A5	mRNA	XR_001033284.2	lncRNA
XM_015097252.1	SLC17A5	mRNA	XR_001033284.2	lncRNA

XM_015097252.1	SLC17A5	mRNA	XR_001033284.2	lncRNA
XM_015097252.1	SLC17A5	mRNA	XR_001033284.2	lncRNA
XM_015097252.1	SLC17A5	mRNA	XR_001033284.2	lncRNA
XM_015097252.1	SLC17A5	mRNA	XR_001033284.2	lncRNA
XM_015096551.1	MOB1B	mRNA	XR_001033284.2	lncRNA
XM_015096551.1	MOB1B	mRNA	XR_001033284.2	lncRNA
XM_015096551.1	MOB1B	mRNA	XR_001033284.2	lncRNA
XM_015096551.1	MOB1B	mRNA	XR_001033284.2	lncRNA
XM_015096551.1	MOB1B	mRNA	XR_001033284.2	lncRNA
XM_015096551.1	MOB1B	mRNA	XR_001033284.2	lncRNA
XM_012124912.1	KIAA2026	mRNA	XR_001436609.1	lncRNA
XM_012124912.1	KIAA2026	mRNA	XR_001436609.1	lncRNA
XM_012124912.1	KIAA2026	mRNA	XR_001436609.1	lncRNA
XM_012124912.1	KIAA2026	mRNA	XR_001436609.1	lncRNA
XM_012124912.1	KIAA2026	mRNA	XR_001436609.1	lncRNA
XM_012124912.1	KIAA2026	mRNA	XR_001436609.1	lncRNA
XM_012188664.2	RASSF2	mRNA	XR_001436609.1	lncRNA
XM_012188664.2	RASSF2	mRNA	XR_001436609.1	lncRNA
XM_012188664.2	RASSF2	mRNA	XR_001436609.1	lncRNA
XM_012188664.2	RASSF2	mRNA	XR_001436609.1	lncRNA
XM_012188664.2	RASSF2	mRNA	XR_001436609.1	lncRNA
XM_012188664.2	RASSF2	mRNA	XR_001436609.1	lncRNA
XM_012107777.1	NFYC	mRNA	XR_001435104.1	lncRNA
XM_012107777.1	NFYC	mRNA	XR_001435104.1	lncRNA
XM_012107777.1	NFYC	mRNA	XR_001435104.1	lncRNA
XM_012107777.1	NFYC	mRNA	XR_001435104.1	lncRNA
XM_012107777.1	NFYC	mRNA	XR_001435104.1	lncRNA
XM_012107777.1	NFYC	mRNA	XR_001435104.1	lncRNA
XM_012189374.1	NXPE3	mRNA	XR_001435104.1	lncRNA
XM_012189374.1	NXPE3	mRNA	XR_001435104.1	lncRNA
XM_012189374.1	NXPE3	mRNA	XR_001435104.1	lncRNA
XM_012189374.1	NXPE3	mRNA	XR_001435104.1	lncRNA
XM_012189374.1	NXPE3	mRNA	XR_001435104.1	lncRNA
XM_012189374.1	NXPE3	mRNA	XR_001435104.1	lncRNA
XM_012094768.2	ZMYM1	mRNA	XR_001435104.1	lncRNA
XM_012094768.2	ZMYM1	mRNA	XR_001435104.1	lncRNA
XM_012094768.2	ZMYM1	mRNA	XR_001435104.1	lncRNA
XM_012094768.2	ZMYM1	mRNA	XR_001435104.1	lncRNA
XM_012094768.2	ZMYM1	mRNA	XR_001435104.1	lncRNA
XM_012094768.2	ZMYM1	mRNA	XR_001435104.1	lncRNA
XM_012176469.2	MACC1	mRNA	XR_001435104.1	lncRNA
XM_012176469.2	MACC1	mRNA	XR_001435104.1	lncRNA
XM_012176469.2	MACC1	mRNA	XR_001435104.1	lncRNA
XM_012176469.2	MACC1	mRNA	XR_001435104.1	lncRNA
XM_012176469.2	MACC1	mRNA	XR_001435104.1	lncRNA
XM_012176469.2	MACC1	mRNA	XR_001435104.1	lncRNA
XM_012106040.2	LOC10112088	mRNA	XR_001435104.1	lncRNA
XM_012106040.2	LOC10112088	mRNA	XR_001435104.1	lncRNA
XM_012106040.2	LOC10112088	mRNA	XR_001435104.1	lncRNA
XM_012106040.2	LOC10112088	mRNA	XR_001435104.1	lncRNA
XM_012106040.2	LOC10112088	mRNA	XR_001435104.1	lncRNA
XM_012106040.2	LOC10112088	mRNA	XR_001435104.1	lncRNA
XM_012104222.2	DCUN1D3	mRNA	XR_001435104.1	lncRNA
XM_012104222.2	DCUN1D3	mRNA	XR_001435104.1	lncRNA

XM_012104222.2	DCUN1D3	mRNA	XR_001435104.1	lncRNA
XM_012104222.2	DCUN1D3	mRNA	XR_001435104.1	lncRNA
XM_012104222.2	DCUN1D3	mRNA	XR_001435104.1	lncRNA
XM_012104222.2	DCUN1D3	mRNA	XR_001435104.1	lncRNA
XM_012145933.2	CXCR2	mRNA	XR_001435104.1	lncRNA
XM_012145933.2	CXCR2	mRNA	XR_001435104.1	lncRNA
XM_012145933.2	CXCR2	mRNA	XR_001435104.1	lncRNA
XM_012145933.2	CXCR2	mRNA	XR_001435104.1	lncRNA
XM_012145933.2	CXCR2	mRNA	XR_001435104.1	lncRNA
XM_012145933.2	CXCR2	mRNA	XR_001435104.1	lncRNA
XM_012190083.2	ZMYM6	mRNA	XR_001436609.1	lncRNA
XM_012190083.2	ZMYM6	mRNA	XR_001436609.1	lncRNA
XM_012190083.2	ZMYM6	mRNA	XR_001436609.1	lncRNA
XM_012190083.2	ZMYM6	mRNA	XR_001436609.1	lncRNA
XM_012190083.2	ZMYM6	mRNA	XR_001436609.1	lncRNA
XM_012190083.2	ZMYM6	mRNA	XR_001436609.1	lncRNA
XM_012174540.1	CPNE8	mRNA	XR_001436609.1	lncRNA
XM_012174540.1	CPNE8	mRNA	XR_001436609.1	lncRNA
XM_012174540.1	CPNE8	mRNA	XR_001436609.1	lncRNA
XM_012174540.1	CPNE8	mRNA	XR_001436609.1	lncRNA
XM_012174540.1	CPNE8	mRNA	XR_001436609.1	lncRNA
XM_012174540.1	CPNE8	mRNA	XR_001436609.1	lncRNA
XM_012133854.2	DYNC1I2	mRNA	XR_001436609.1	lncRNA
XM_012133854.2	DYNC1I2	mRNA	XR_001436609.1	lncRNA
XM_012133854.2	DYNC1I2	mRNA	XR_001436609.1	lncRNA
XM_012133854.2	DYNC1I2	mRNA	XR_001436609.1	lncRNA
XM_012133854.2	DYNC1I2	mRNA	XR_001436609.1	lncRNA
XM_012133854.2	DYNC1I2	mRNA	XR_001436609.1	lncRNA
XM_012182988.2	REPS1	mRNA	XR_001436609.1	lncRNA
XM_012182988.2	REPS1	mRNA	XR_001436609.1	lncRNA
XM_012182988.2	REPS1	mRNA	XR_001436609.1	lncRNA
XM_012182988.2	REPS1	mRNA	XR_001436609.1	lncRNA
XM_012182988.2	REPS1	mRNA	XR_001436609.1	lncRNA
XM_012182988.2	REPS1	mRNA	XR_001436609.1	lncRNA
XM_015097359.1	ARMC2	mRNA	XR_001436609.1	lncRNA
XM_015097359.1	ARMC2	mRNA	XR_001436609.1	lncRNA
XM_015097359.1	ARMC2	mRNA	XR_001436609.1	lncRNA
XM_015097359.1	ARMC2	mRNA	XR_001436609.1	lncRNA
XM_015097359.1	ARMC2	mRNA	XR_001436609.1	lncRNA
XM_015097359.1	ARMC2	mRNA	XR_001436609.1	lncRNA
XM_012184485.2	RNASEH2B	mRNA	XR_001436609.1	lncRNA
XM_012184485.2	RNASEH2B	mRNA	XR_001436609.1	lncRNA
XM_012184485.2	RNASEH2B	mRNA	XR_001436609.1	lncRNA
XM_012184485.2	RNASEH2B	mRNA	XR_001436609.1	lncRNA
XM_012184485.2	RNASEH2B	mRNA	XR_001436609.1	lncRNA
XM_012184485.2	RNASEH2B	mRNA	XR_001436609.1	lncRNA
XM_012094768.2	ZMYM1	mRNA	XR_001436609.1	lncRNA
XM_012094768.2	ZMYM1	mRNA	XR_001436609.1	lncRNA
XM_012094768.2	ZMYM1	mRNA	XR_001436609.1	lncRNA
XM_012094768.2	ZMYM1	mRNA	XR_001436609.1	lncRNA
XM_012094768.2	ZMYM1	mRNA	XR_001436609.1	lncRNA
XM_012094768.2	ZMYM1	mRNA	XR_001436609.1	lncRNA
XM_012145933.2	CXCR2	mRNA	XR_001436609.1	lncRNA
XM_012145933.2	CXCR2	mRNA	XR_001436609.1	lncRNA

XM_012145933.2	CXCR2	mRNA	XR_001436609.1	lncRNA
XM_012145933.2	CXCR2	mRNA	XR_001436609.1	lncRNA
XM_012145933.2	CXCR2	mRNA	XR_001436609.1	lncRNA
XM_012145933.2	CXCR2	mRNA	XR_001436609.1	lncRNA
XM_015102735.1	SUPT3H	mRNA	XR_001435104.1	lncRNA
XM_015102735.1	SUPT3H	mRNA	XR_001435104.1	lncRNA
XM_015102735.1	SUPT3H	mRNA	XR_001435104.1	lncRNA
XM_015102735.1	SUPT3H	mRNA	XR_001435104.1	lncRNA
XM_015102735.1	SUPT3H	mRNA	XR_001435104.1	lncRNA
XM_015102735.1	SUPT3H	mRNA	XR_001435104.1	lncRNA
XM_012133854.2	DYNC1I2	mRNA	XR_001435104.1	lncRNA
XM_012133854.2	DYNC1I2	mRNA	XR_001435104.1	lncRNA
XM_012133854.2	DYNC1I2	mRNA	XR_001435104.1	lncRNA
XM_012133854.2	DYNC1I2	mRNA	XR_001435104.1	lncRNA
XM_012133854.2	DYNC1I2	mRNA	XR_001435104.1	lncRNA
XM_012133854.2	DYNC1I2	mRNA	XR_001435104.1	lncRNA
XM_015093098.1	PPP3CC	mRNA	XR_001435104.1	lncRNA
XM_015093098.1	PPP3CC	mRNA	XR_001435104.1	lncRNA
XM_015093098.1	PPP3CC	mRNA	XR_001435104.1	lncRNA
XM_015093098.1	PPP3CC	mRNA	XR_001435104.1	lncRNA
XM_015093098.1	PPP3CC	mRNA	XR_001435104.1	lncRNA
XM_015093098.1	PPP3CC	mRNA	XR_001435104.1	lncRNA
XM_015093101.1	PPP3CC	mRNA	XR_001435104.1	lncRNA
XM_015093101.1	PPP3CC	mRNA	XR_001435104.1	lncRNA
XM_015093101.1	PPP3CC	mRNA	XR_001435104.1	lncRNA
XM_015093101.1	PPP3CC	mRNA	XR_001435104.1	lncRNA
XM_015093101.1	PPP3CC	mRNA	XR_001435104.1	lncRNA
XM_015093101.1	PPP3CC	mRNA	XR_001435104.1	lncRNA
XM_015092197.1	CD58	mRNA	XR_001435104.1	lncRNA
XM_015092197.1	CD58	mRNA	XR_001435104.1	lncRNA
XM_015092197.1	CD58	mRNA	XR_001435104.1	lncRNA
XM_015092197.1	CD58	mRNA	XR_001435104.1	lncRNA
XM_015092197.1	CD58	mRNA	XR_001435104.1	lncRNA
XM_015092197.1	CD58	mRNA	XR_001435104.1	lncRNA
XM_012102560.2	CEP70	mRNA	XR_001436531.1	lncRNA
XM_012102560.2	CEP70	mRNA	XR_001436531.1	lncRNA
XM_012102560.2	CEP70	mRNA	XR_001436531.1	lncRNA
XM_012102560.2	CEP70	mRNA	XR_001436531.1	lncRNA
XM_012102560.2	CEP70	mRNA	XR_001436531.1	lncRNA
XM_012102560.2	CEP70	mRNA	XR_001436531.1	lncRNA
XM_012184485.2	RNASEH2B	mRNA	XR_001435104.1	lncRNA
XM_012184485.2	RNASEH2B	mRNA	XR_001435104.1	lncRNA
XM_012184485.2	RNASEH2B	mRNA	XR_001435104.1	lncRNA
XM_012184485.2	RNASEH2B	mRNA	XR_001435104.1	lncRNA
XM_012184485.2	RNASEH2B	mRNA	XR_001435104.1	lncRNA
XM_012184485.2	RNASEH2B	mRNA	XR_001435104.1	lncRNA
XM_012179604.2	UBE2D3	mRNA	XR_001435104.1	lncRNA
XM_012179604.2	UBE2D3	mRNA	XR_001435104.1	lncRNA
XM_012179604.2	UBE2D3	mRNA	XR_001435104.1	lncRNA
XM_012179604.2	UBE2D3	mRNA	XR_001435104.1	lncRNA
XM_012179604.2	UBE2D3	mRNA	XR_001435104.1	lncRNA
XM_012179604.2	UBE2D3	mRNA	XR_001435104.1	lncRNA
XM_015097359.1	ARMC2	mRNA	XR_001435104.1	lncRNA
XM_015097359.1	ARMC2	mRNA	XR_001435104.1	lncRNA

XM_015097359.1	ARMC2	mRNA	XR_001435104.1	lncRNA
XM_015097359.1	ARMC2	mRNA	XR_001435104.1	lncRNA
XM_015097359.1	ARMC2	mRNA	XR_001435104.1	lncRNA
XM_015097359.1	ARMC2	mRNA	XR_001435104.1	lncRNA
XM_012182988.2	REPS1	mRNA	XR_001435104.1	lncRNA
XM_012182988.2	REPS1	mRNA	XR_001435104.1	lncRNA
XM_012182988.2	REPS1	mRNA	XR_001435104.1	lncRNA
XM_012182988.2	REPS1	mRNA	XR_001435104.1	lncRNA
XM_012182988.2	REPS1	mRNA	XR_001435104.1	lncRNA
XM_012182988.2	REPS1	mRNA	XR_001435104.1	lncRNA
XM_012104225.2	DCUN1D3	mRNA	XR_001436531.1	lncRNA
XM_012104225.2	DCUN1D3	mRNA	XR_001436531.1	lncRNA
XM_012104225.2	DCUN1D3	mRNA	XR_001436531.1	lncRNA
XM_012104225.2	DCUN1D3	mRNA	XR_001436531.1	lncRNA
XM_012104225.2	DCUN1D3	mRNA	XR_001436531.1	lncRNA
XM_012104225.2	DCUN1D3	mRNA	XR_001436531.1	lncRNA
XM_012188664.2	RASSF2	mRNA	XR_001435104.1	lncRNA
XM_012188664.2	RASSF2	mRNA	XR_001435104.1	lncRNA
XM_012188664.2	RASSF2	mRNA	XR_001435104.1	lncRNA
XM_012188664.2	RASSF2	mRNA	XR_001435104.1	lncRNA
XM_012188664.2	RASSF2	mRNA	XR_001435104.1	lncRNA
XM_012188664.2	RASSF2	mRNA	XR_001435104.1	lncRNA
XM_012190515.2	ZMYM1	mRNA	XR_001436531.1	lncRNA
XM_012190515.2	ZMYM1	mRNA	XR_001436531.1	lncRNA
XM_012190515.2	ZMYM1	mRNA	XR_001436531.1	lncRNA
XM_012190515.2	ZMYM1	mRNA	XR_001436531.1	lncRNA
XM_012190515.2	ZMYM1	mRNA	XR_001436531.1	lncRNA
XM_012190515.2	ZMYM1	mRNA	XR_001436531.1	lncRNA
XM_012187630.2	SOAT1	mRNA	XR_001435104.1	lncRNA
XM_012187630.2	SOAT1	mRNA	XR_001435104.1	lncRNA
XM_012187630.2	SOAT1	mRNA	XR_001435104.1	lncRNA
XM_012187630.2	SOAT1	mRNA	XR_001435104.1	lncRNA
XM_012187630.2	SOAT1	mRNA	XR_001435104.1	lncRNA
XM_012187630.2	SOAT1	mRNA	XR_001435104.1	lncRNA
XM_012178157.2	RAPGEF6	mRNA	XR_001041202.1	lncRNA
XM_012178157.2	RAPGEF6	mRNA	XR_001041202.1	lncRNA
XM_012178157.2	RAPGEF6	mRNA	XR_001041202.1	lncRNA
XM_012178157.2	RAPGEF6	mRNA	XR_001041202.1	lncRNA
XM_012178157.2	RAPGEF6	mRNA	XR_001041202.1	lncRNA
XM_012178157.2	RAPGEF6	mRNA	XR_001041202.1	lncRNA
XM_015097252.1	SLC17A5	mRNA	XR_001041211.2	lncRNA
XM_015097252.1	SLC17A5	mRNA	XR_001041211.2	lncRNA
XM_015097252.1	SLC17A5	mRNA	XR_001041211.2	lncRNA
XM_015097252.1	SLC17A5	mRNA	XR_001041211.2	lncRNA
XM_015097252.1	SLC17A5	mRNA	XR_001041211.2	lncRNA
XM_015097252.1	SLC17A5	mRNA	XR_001041211.2	lncRNA
XM_012176656.2	NAPEPLD	mRNA	XR_001041211.2	lncRNA
XM_012176656.2	NAPEPLD	mRNA	XR_001041211.2	lncRNA
XM_012176656.2	NAPEPLD	mRNA	XR_001041211.2	lncRNA
XM_012176656.2	NAPEPLD	mRNA	XR_001041211.2	lncRNA
XM_012176656.2	NAPEPLD	mRNA	XR_001041211.2	lncRNA
XM_012176656.2	NAPEPLD	mRNA	XR_001041211.2	lncRNA
XM_004008647.3	RAPGEF6	mRNA	XR_001041209.2	lncRNA
XM_004008647.3	RAPGEF6	mRNA	XR_001041209.2	lncRNA

XM_004008647.3	RAPGEF6	mRNA	XR_001041209.2	lncRNA
XM_004008647.3	RAPGEF6	mRNA	XR_001041209.2	lncRNA
XM_004008647.3	RAPGEF6	mRNA	XR_001041209.2	lncRNA
XM_004008647.3	RAPGEF6	mRNA	XR_001041209.2	lncRNA
XM_012180800.1	TIPIN	mRNA	XR_001041209.2	lncRNA
XM_012180800.1	TIPIN	mRNA	XR_001041209.2	lncRNA
XM_012180800.1	TIPIN	mRNA	XR_001041209.2	lncRNA
XM_012180800.1	TIPIN	mRNA	XR_001041209.2	lncRNA
XM_012180800.1	TIPIN	mRNA	XR_001041209.2	lncRNA
XM_012180800.1	TIPIN	mRNA	XR_001041209.2	lncRNA
XM_012187575.2	RABGAP1L	mRNA	XR_001041211.2	lncRNA
XM_012187575.2	RABGAP1L	mRNA	XR_001041211.2	lncRNA
XM_012187575.2	RABGAP1L	mRNA	XR_001041211.2	lncRNA
XM_012187575.2	RABGAP1L	mRNA	XR_001041211.2	lncRNA
XM_012187575.2	RABGAP1L	mRNA	XR_001041211.2	lncRNA
XM_012187575.2	RABGAP1L	mRNA	XR_001041211.2	lncRNA
XM_012095858.2	ZNF214	mRNA	TCONS_00029399	lncRNA
XM_012095858.2	ZNF214	mRNA	TCONS_00029399	lncRNA
XM_012095858.2	ZNF214	mRNA	TCONS_00029399	lncRNA
XM_012095858.2	ZNF214	mRNA	TCONS_00029399	lncRNA
XM_012095858.2	ZNF214	mRNA	TCONS_00029399	lncRNA
XM_012095858.2	ZNF214	mRNA	TCONS_00029399	lncRNA
XM_012174754.2	RNF41	mRNA	XR_001041209.2	lncRNA
XM_012174754.2	RNF41	mRNA	XR_001041209.2	lncRNA
XM_012174754.2	RNF41	mRNA	XR_001041209.2	lncRNA
XM_012174754.2	RNF41	mRNA	XR_001041209.2	lncRNA
XM_012174754.2	RNF41	mRNA	XR_001041209.2	lncRNA
XM_012174754.2	RNF41	mRNA	XR_001041209.2	lncRNA
XM_012174787.1	LOC1011137[illegible]	mRNA	XR_001041209.2	lncRNA
XM_012174787.1	LOC1011137[illegible]	mRNA	XR_001041209.2	lncRNA
XM_012174787.1	LOC1011137[illegible]	mRNA	XR_001041209.2	lncRNA
XM_012174787.1	LOC1011137[illegible]	mRNA	XR_001041209.2	lncRNA
XM_012174787.1	LOC1011137[illegible]	mRNA	XR_001041209.2	lncRNA
XM_012174787.1	LOC1011137[illegible]	mRNA	XR_001041209.2	lncRNA
XM_004017788.3	ZFANL3	mRNA	XR_001021689.2	lncRNA
XM_004017788.3	ZFANL3	mRNA	XR_001021689.2	lncRNA
XM_004017788.3	ZFANL3	mRNA	XR_001021689.2	lncRNA
XM_004017788.3	ZFANL3	mRNA	XR_001021689.2	lncRNA
XM_004017788.3	ZFANL3	mRNA	XR_001021689.2	lncRNA
XM_004017788.3	ZFANL3	mRNA	XR_001021689.2	lncRNA
XM_012095713.2	RRAS2	mRNA	XR_001021689.2	lncRNA
XM_012095713.2	RRAS2	mRNA	XR_001021689.2	lncRNA
XM_012095713.2	RRAS2	mRNA	XR_001021689.2	lncRNA
XM_012095713.2	RRAS2	mRNA	XR_001021689.2	lncRNA
XM_012095713.2	RRAS2	mRNA	XR_001021689.2	lncRNA
XM_012095713.2	RRAS2	mRNA	XR_001021689.2	lncRNA
XM_012175265.1	PLEKHA5	mRNA	TCONS_00071561	lncRNA
XM_012175265.1	PLEKHA5	mRNA	TCONS_00071561	lncRNA
XM_012175265.1	PLEKHA5	mRNA	TCONS_00071561	lncRNA
XM_012175265.1	PLEKHA5	mRNA	TCONS_00071561	lncRNA
XM_012175265.1	PLEKHA5	mRNA	TCONS_00071561	lncRNA
XM_012175265.1	PLEKHA5	mRNA	TCONS_00071561	lncRNA
XM_012105198.2	TET1	mRNA	TCONS_00071561	lncRNA
XM_012105198.2	TET1	mRNA	TCONS_00071561	lncRNA

XM_012105198.2	TET1	mRNA	TCONS_00071561	lncRNA
XM_012105198.2	TET1	mRNA	TCONS_00071561	lncRNA
XM_012105198.2	TET1	mRNA	TCONS_00071561	lncRNA
XM_012105198.2	TET1	mRNA	TCONS_00071561	lncRNA
XM_012133854.2	DYNC1I2	mRNA	XR_001435726.1	lncRNA
XM_012133854.2	DYNC1I2	mRNA	XR_001435726.1	lncRNA
XM_012133854.2	DYNC1I2	mRNA	XR_001435726.1	lncRNA
XM_012133854.2	DYNC1I2	mRNA	XR_001435726.1	lncRNA
XM_012133854.2	DYNC1I2	mRNA	XR_001435726.1	lncRNA
XM_012133854.2	DYNC1I2	mRNA	XR_001435726.1	lncRNA
XM_012095418.2	ZMYM4	mRNA	XR_001042381.2	lncRNA
XM_012095418.2	ZMYM4	mRNA	XR_001042381.2	lncRNA
XM_012095418.2	ZMYM4	mRNA	XR_001042381.2	lncRNA
XM_012095418.2	ZMYM4	mRNA	XR_001042381.2	lncRNA
XM_012095418.2	ZMYM4	mRNA	XR_001042381.2	lncRNA
XM_012095418.2	ZMYM4	mRNA	XR_001042381.2	lncRNA
XM_015093101.1	PPP3CC	mRNA	XR_001435726.1	lncRNA
XM_015093101.1	PPP3CC	mRNA	XR_001435726.1	lncRNA
XM_015093101.1	PPP3CC	mRNA	XR_001435726.1	lncRNA
XM_015093101.1	PPP3CC	mRNA	XR_001435726.1	lncRNA
XM_015093101.1	PPP3CC	mRNA	XR_001435726.1	lncRNA
XM_015093101.1	PPP3CC	mRNA	XR_001435726.1	lncRNA
XM_012096744.2	GFM2	mRNA	XR_001435723.1	lncRNA
XM_012096744.2	GFM2	mRNA	XR_001435723.1	lncRNA
XM_012096744.2	GFM2	mRNA	XR_001435723.1	lncRNA
XM_012096744.2	GFM2	mRNA	XR_001435723.1	lncRNA
XM_012096744.2	GFM2	mRNA	XR_001435723.1	lncRNA
XM_012096744.2	GFM2	mRNA	XR_001435723.1	lncRNA
XM_012104232.2	DCUN1D3	mRNA	XR_001042381.2	lncRNA
XM_012104232.2	DCUN1D3	mRNA	XR_001042381.2	lncRNA
XM_012104232.2	DCUN1D3	mRNA	XR_001042381.2	lncRNA
XM_012104232.2	DCUN1D3	mRNA	XR_001042381.2	lncRNA
XM_012104232.2	DCUN1D3	mRNA	XR_001042381.2	lncRNA
XM_012104232.2	DCUN1D3	mRNA	XR_001042381.2	lncRNA
XM_012124912.1	KIAA2026	mRNA	XR_001435726.1	lncRNA
XM_012124912.1	KIAA2026	mRNA	XR_001435726.1	lncRNA
XM_012124912.1	KIAA2026	mRNA	XR_001435726.1	lncRNA
XM_012124912.1	KIAA2026	mRNA	XR_001435726.1	lncRNA
XM_012124912.1	KIAA2026	mRNA	XR_001435726.1	lncRNA
XM_012124912.1	KIAA2026	mRNA	XR_001435726.1	lncRNA
XM_012188226.2	CUL2	mRNA	XR_001435723.1	lncRNA
XM_012188226.2	CUL2	mRNA	XR_001435723.1	lncRNA
XM_012188226.2	CUL2	mRNA	XR_001435723.1	lncRNA
XM_012188226.2	CUL2	mRNA	XR_001435723.1	lncRNA
XM_012188226.2	CUL2	mRNA	XR_001435723.1	lncRNA
XM_012188226.2	CUL2	mRNA	XR_001435723.1	lncRNA
XM_012096744.2	GFM2	mRNA	XR_001435726.1	lncRNA
XM_012096744.2	GFM2	mRNA	XR_001435726.1	lncRNA
XM_012096744.2	GFM2	mRNA	XR_001435726.1	lncRNA
XM_012096744.2	GFM2	mRNA	XR_001435726.1	lncRNA
XM_012096744.2	GFM2	mRNA	XR_001435726.1	lncRNA
XM_012096744.2	GFM2	mRNA	XR_001435726.1	lncRNA
XM_012190083.2	ZMYM6	mRNA	XR_001435726.1	lncRNA
XM_012190083.2	ZMYM6	mRNA	XR_001435726.1	lncRNA

XM_012190083.2	ZMYM6	mRNA	XR_001435726.1	lncRNA
XM_012190083.2	ZMYM6	mRNA	XR_001435726.1	lncRNA
XM_012190083.2	ZMYM6	mRNA	XR_001435726.1	lncRNA
XM_012190083.2	ZMYM6	mRNA	XR_001435726.1	lncRNA
XM_012104225.2	DCUN1D3	mRNA	XR_001435726.1	lncRNA
XM_012104225.2	DCUN1D3	mRNA	XR_001435726.1	lncRNA
XM_012104225.2	DCUN1D3	mRNA	XR_001435726.1	lncRNA
XM_012104225.2	DCUN1D3	mRNA	XR_001435726.1	lncRNA
XM_012104225.2	DCUN1D3	mRNA	XR_001435726.1	lncRNA
XM_012104225.2	DCUN1D3	mRNA	XR_001435726.1	lncRNA
XM_012187919.2	LOC1056166	mRNA	XR_001435726.1	lncRNA
XM_012187919.2	LOC1056166	mRNA	XR_001435726.1	lncRNA
XM_012187919.2	LOC1056166	mRNA	XR_001435726.1	lncRNA
XM_012187919.2	LOC1056166	mRNA	XR_001435726.1	lncRNA
XM_012187919.2	LOC1056166	mRNA	XR_001435726.1	lncRNA
XM_012187919.2	LOC1056166	mRNA	XR_001435726.1	lncRNA
XM_012124912.1	KIAA2026	mRNA	XR_001435723.1	lncRNA
XM_012124912.1	KIAA2026	mRNA	XR_001435723.1	lncRNA
XM_012124912.1	KIAA2026	mRNA	XR_001435723.1	lncRNA
XM_012124912.1	KIAA2026	mRNA	XR_001435723.1	lncRNA
XM_012124912.1	KIAA2026	mRNA	XR_001435723.1	lncRNA
XM_012124912.1	KIAA2026	mRNA	XR_001435723.1	lncRNA
XM_012102549.2	CEP70	mRNA	XR_001435723.1	lncRNA
XM_012102549.2	CEP70	mRNA	XR_001435723.1	lncRNA
XM_012102549.2	CEP70	mRNA	XR_001435723.1	lncRNA
XM_012102549.2	CEP70	mRNA	XR_001435723.1	lncRNA
XM_012102549.2	CEP70	mRNA	XR_001435723.1	lncRNA
XM_012102549.2	CEP70	mRNA	XR_001435723.1	lncRNA
XM_012094768.2	ZMYM1	mRNA	XR_001435726.1	lncRNA
XM_012094768.2	ZMYM1	mRNA	XR_001435726.1	lncRNA
XM_012094768.2	ZMYM1	mRNA	XR_001435726.1	lncRNA
XM_012094768.2	ZMYM1	mRNA	XR_001435726.1	lncRNA
XM_012094768.2	ZMYM1	mRNA	XR_001435726.1	lncRNA
XM_012094768.2	ZMYM1	mRNA	XR_001435726.1	lncRNA
XM_012181536.2	FAM214A	mRNA	XR_001042381.2	lncRNA
XM_012181536.2	FAM214A	mRNA	XR_001042381.2	lncRNA
XM_012181536.2	FAM214A	mRNA	XR_001042381.2	lncRNA
XM_012181536.2	FAM214A	mRNA	XR_001042381.2	lncRNA
XM_012181536.2	FAM214A	mRNA	XR_001042381.2	lncRNA
XM_012181536.2	FAM214A	mRNA	XR_001042381.2	lncRNA
XM_015102735.1	SUPT3H	mRNA	XR_001435723.1	lncRNA
XM_015102735.1	SUPT3H	mRNA	XR_001435723.1	lncRNA
XM_015102735.1	SUPT3H	mRNA	XR_001435723.1	lncRNA
XM_015102735.1	SUPT3H	mRNA	XR_001435723.1	lncRNA
XM_015102735.1	SUPT3H	mRNA	XR_001435723.1	lncRNA
XM_015102735.1	SUPT3H	mRNA	XR_001435723.1	lncRNA
XM_015093101.1	PPP3CC	mRNA	XR_001435723.1	lncRNA
XM_015093101.1	PPP3CC	mRNA	XR_001435723.1	lncRNA
XM_015093101.1	PPP3CC	mRNA	XR_001435723.1	lncRNA
XM_015093101.1	PPP3CC	mRNA	XR_001435723.1	lncRNA
XM_015093101.1	PPP3CC	mRNA	XR_001435723.1	lncRNA
XM_015093101.1	PPP3CC	mRNA	XR_001435723.1	lncRNA
XM_012102549.2	CEP70	mRNA	XR_001435726.1	lncRNA
XM_012102549.2	CEP70	mRNA	XR_001435726.1	lncRNA

XM_012102549.2	CEP70	mRNA	XR_001435726.1	lncRNA
XM_012102549.2	CEP70	mRNA	XR_001435726.1	lncRNA
XM_012102549.2	CEP70	mRNA	XR_001435726.1	lncRNA
XM_012102549.2	CEP70	mRNA	XR_001435726.1	lncRNA
XM_012095272.2	ARNTL	mRNA	XR_001435726.1	lncRNA
XM_012095272.2	ARNTL	mRNA	XR_001435726.1	lncRNA
XM_012095272.2	ARNTL	mRNA	XR_001435726.1	lncRNA
XM_012095272.2	ARNTL	mRNA	XR_001435726.1	lncRNA
XM_012095272.2	ARNTL	mRNA	XR_001435726.1	lncRNA
XM_012095272.2	ARNTL	mRNA	XR_001435726.1	lncRNA
XM_004008647.3	RAPGEF6	mRNA	XR_001434081.1	lncRNA
XM_004008647.3	RAPGEF6	mRNA	XR_001434081.1	lncRNA
XM_004008647.3	RAPGEF6	mRNA	XR_001434081.1	lncRNA
XM_004008647.3	RAPGEF6	mRNA	XR_001434081.1	lncRNA
XM_004008647.3	RAPGEF6	mRNA	XR_001434081.1	lncRNA
XM_004008647.3	RAPGEF6	mRNA	XR_001434081.1	lncRNA
XM_012190105.1	ANKRL17	mRNA	XR_001434081.1	lncRNA
XM_012190105.1	ANKRL17	mRNA	XR_001434081.1	lncRNA
XM_012190105.1	ANKRL17	mRNA	XR_001434081.1	lncRNA
XM_012190105.1	ANKRL17	mRNA	XR_001434081.1	lncRNA
XM_012190105.1	ANKRL17	mRNA	XR_001434081.1	lncRNA
XM_012190105.1	ANKRL17	mRNA	XR_001434081.1	lncRNA
XM_012105564.2	ZFAND4	mRNA	XR_001434081.1	lncRNA
XM_012105564.2	ZFAND4	mRNA	XR_001434081.1	lncRNA
XM_012105564.2	ZFAND4	mRNA	XR_001434081.1	lncRNA
XM_012105564.2	ZFAND4	mRNA	XR_001434081.1	lncRNA
XM_012105564.2	ZFAND4	mRNA	XR_001434081.1	lncRNA
XM_012105564.2	ZFAND4	mRNA	XR_001434081.1	lncRNA
XM_012176656.2	NAPEPLD	mRNA	XR_001434081.1	lncRNA
XM_012176656.2	NAPEPLD	mRNA	XR_001434081.1	lncRNA
XM_012176656.2	NAPEPLD	mRNA	XR_001434081.1	lncRNA
XM_012176656.2	NAPEPLD	mRNA	XR_001434081.1	lncRNA
XM_012176656.2	NAPEPLD	mRNA	XR_001434081.1	lncRNA
XM_012176656.2	NAPEPLD	mRNA	XR_001434081.1	lncRNA
XM_012106466.2	DMD	mRNA	XR_001434081.1	lncRNA
XM_012106466.2	DMD	mRNA	XR_001434081.1	lncRNA
XM_012106466.2	DMD	mRNA	XR_001434081.1	lncRNA
XM_012106466.2	DMD	mRNA	XR_001434081.1	lncRNA
XM_012106466.2	DMD	mRNA	XR_001434081.1	lncRNA
XM_012106466.2	DMD	mRNA	XR_001434081.1	lncRNA
XM_004006434.3	PUS7L	mRNA	XR_001033284.2	lncRNA
XM_004006434.3	PUS7L	mRNA	XR_001033284.2	lncRNA
XM_004006434.3	PUS7L	mRNA	XR_001033284.2	lncRNA
XM_004006434.3	PUS7L	mRNA	XR_001033284.2	lncRNA
XM_004006434.3	PUS7L	mRNA	XR_001033284.2	lncRNA
XM_004006434.3	PUS7L	mRNA	XR_001033284.2	lncRNA
XM_015094782.1	MARS	mRNA	XR_001033284.2	lncRNA
XM_015094782.1	MARS	mRNA	XR_001033284.2	lncRNA
XM_015094782.1	MARS	mRNA	XR_001033284.2	lncRNA
XM_015094782.1	MARS	mRNA	XR_001033284.2	lncRNA
XM_015094782.1	MARS	mRNA	XR_001033284.2	lncRNA
XM_015094782.1	MARS	mRNA	XR_001033284.2	lncRNA
XM_012098017.2	KREMEN1	mRNA	XR_001436099.1	lncRNA
XM_012098017.2	KREMEN1	mRNA	XR_001436099.1	lncRNA

XM_012098017.2	KREMEN1	mRNA	XR_001436099.1	lncRNA
XM_012098017.2	KREMEN1	mRNA	XR_001436099.1	lncRNA
XM_012098017.2	KREMEN1	mRNA	XR_001436099.1	lncRNA
XM_012098017.2	KREMEN1	mRNA	XR_001436099.1	lncRNA
XM_012176678.2	KMT2E	mRNA	XR_001033284.2	lncRNA
XM_012176678.2	KMT2E	mRNA	XR_001033284.2	lncRNA
XM_012176678.2	KMT2E	mRNA	XR_001033284.2	lncRNA
XM_012176678.2	KMT2E	mRNA	XR_001033284.2	lncRNA
XM_012176678.2	KMT2E	mRNA	XR_001033284.2	lncRNA
XM_012176678.2	KMT2E	mRNA	XR_001033284.2	lncRNA
XM_012177175.2	CALD1	mRNA	XR_001041202.1	lncRNA
XM_012177175.2	CALD1	mRNA	XR_001041202.1	lncRNA
XM_012177175.2	CALD1	mRNA	XR_001041202.1	lncRNA
XM_012177175.2	CALD1	mRNA	XR_001041202.1	lncRNA
XM_012177175.2	CALD1	mRNA	XR_001041202.1	lncRNA
XM_012177175.2	CALD1	mRNA	XR_001041202.1	lncRNA
XM_012098990.2	NAALADL2	mRNA	XR_001021689.2	lncRNA
XM_012098990.2	NAALADL2	mRNA	XR_001021689.2	lncRNA
XM_012098990.2	NAALADL2	mRNA	XR_001021689.2	lncRNA
XM_012098990.2	NAALADL2	mRNA	XR_001021689.2	lncRNA
XM_012098990.2	NAALADL2	mRNA	XR_001021689.2	lncRNA
XM_012098990.2	NAALADL2	mRNA	XR_001021689.2	lncRNA
XM_012183298.1	FAM120B	mRNA	TCONS_00071561	lncRNA
XM_012183298.1	FAM120B	mRNA	TCONS_00071561	lncRNA
XM_012183298.1	FAM120B	mRNA	TCONS_00071561	lncRNA
XM_012183298.1	FAM120B	mRNA	TCONS_00071561	lncRNA
XM_012183298.1	FAM120B	mRNA	TCONS_00071561	lncRNA
XM_012183298.1	FAM120B	mRNA	TCONS_00071561	lncRNA
XM_012190696.2	CCDC155	mRNA	TCONS_00071561	lncRNA
XM_012190696.2	CCDC155	mRNA	TCONS_00071561	lncRNA
XM_012190696.2	CCDC155	mRNA	TCONS_00071561	lncRNA
XM_012190696.2	CCDC155	mRNA	TCONS_00071561	lncRNA
XM_012190696.2	CCDC155	mRNA	TCONS_00071561	lncRNA
XM_012190696.2	CCDC155	mRNA	TCONS_00071561	lncRNA
XM_015101107.1	TCP11L1	mRNA	TCONS_00071561	lncRNA
XM_015101107.1	TCP11L1	mRNA	TCONS_00071561	lncRNA
XM_015101107.1	TCP11L1	mRNA	TCONS_00071561	lncRNA
XM_015101107.1	TCP11L1	mRNA	TCONS_00071561	lncRNA
XM_015101107.1	TCP11L1	mRNA	TCONS_00071561	lncRNA
XM_015101107.1	TCP11L1	mRNA	TCONS_00071561	lncRNA
XM_012099916.2	PRKCD	mRNA	TCONS_00071561	lncRNA
XM_012099916.2	PRKCD	mRNA	TCONS_00071561	lncRNA
XM_012099916.2	PRKCD	mRNA	TCONS_00071561	lncRNA
XM_012099916.2	PRKCD	mRNA	TCONS_00071561	lncRNA
XM_012099916.2	PRKCD	mRNA	TCONS_00071561	lncRNA
XM_012099916.2	PRKCD	mRNA	TCONS_00071561	lncRNA
XM_012095858.2	ZNF214	mRNA	XR_001021939.1	lncRNA
XM_012095858.2	ZNF214	mRNA	XR_001021939.1	lncRNA
XM_012095858.2	ZNF214	mRNA	XR_001021939.1	lncRNA
XM_012095858.2	ZNF214	mRNA	XR_001021939.1	lncRNA
XM_012095858.2	ZNF214	mRNA	XR_001021939.1	lncRNA
XM_012095858.2	ZNF214	mRNA	XR_001021939.1	lncRNA
XM_012187729.2	SWT1	mRNA	TCONS_00029399	lncRNA
XM_012187729.2	SWT1	mRNA	TCONS_00029399	lncRNA

XM_012187729.2	SWT1	mRNA	TCONS_00029399	lncRNA
XM_012187729.2	SWT1	mRNA	TCONS_00029399	lncRNA
XM_012187729.2	SWT1	mRNA	TCONS_00029399	lncRNA
XM_012187729.2	SWT1	mRNA	TCONS_00029399	lncRNA
XM_012181720.2	ARID4A	mRNA	XR_001041209.2	lncRNA
XM_012181720.2	ARID4A	mRNA	XR_001041209.2	lncRNA
XM_012181720.2	ARID4A	mRNA	XR_001041209.2	lncRNA
XM_012181720.2	ARID4A	mRNA	XR_001041209.2	lncRNA
XM_012181720.2	ARID4A	mRNA	XR_001041209.2	lncRNA
XM_012181720.2	ARID4A	mRNA	XR_001041209.2	lncRNA
XM_012100126.2	MAP4	mRNA	XR_001041209.2	lncRNA
XM_012100126.2	MAP4	mRNA	XR_001041209.2	lncRNA
XM_012100126.2	MAP4	mRNA	XR_001041209.2	lncRNA
XM_012100126.2	MAP4	mRNA	XR_001041209.2	lncRNA
XM_012100126.2	MAP4	mRNA	XR_001041209.2	lncRNA
XM_012100126.2	MAP4	mRNA	XR_001041209.2	lncRNA
XM_012129647.1	NEK1	mRNA	XR_001041211.2	lncRNA
XM_012129647.1	NEK1	mRNA	XR_001041211.2	lncRNA
XM_012129647.1	NEK1	mRNA	XR_001041211.2	lncRNA
XM_012129647.1	NEK1	mRNA	XR_001041211.2	lncRNA
XM_012129647.1	NEK1	mRNA	XR_001041211.2	lncRNA
XM_012129647.1	NEK1	mRNA	XR_001041211.2	lncRNA
XM_015100095.1	LOC10111352	mRNA	TCONS_00029399	lncRNA
XM_015100095.1	LOC10111352	mRNA	TCONS_00029399	lncRNA
XM_015100095.1	LOC10111352	mRNA	TCONS_00029399	lncRNA
XM_015100095.1	LOC10111352	mRNA	TCONS_00029399	lncRNA
XM_015100095.1	LOC10111352	mRNA	TCONS_00029399	lncRNA
XM_015100095.1	LOC10111352	mRNA	TCONS_00029399	lncRNA
XM_012186944.1	DSTYK	mRNA	XR_001041211.2	lncRNA
XM_012186944.1	DSTYK	mRNA	XR_001041211.2	lncRNA
XM_012186944.1	DSTYK	mRNA	XR_001041211.2	lncRNA
XM_012186944.1	DSTYK	mRNA	XR_001041211.2	lncRNA
XM_012186944.1	DSTYK	mRNA	XR_001041211.2	lncRNA
XM_012186944.1	DSTYK	mRNA	XR_001041211.2	lncRNA
XM_015105099.1	PAK3	mRNA	XR_001041211.2	lncRNA
XM_015105099.1	PAK3	mRNA	XR_001041211.2	lncRNA
XM_015105099.1	PAK3	mRNA	XR_001041211.2	lncRNA
XM_015105099.1	PAK3	mRNA	XR_001041211.2	lncRNA
XM_015105099.1	PAK3	mRNA	XR_001041211.2	lncRNA
XM_015105099.1	PAK3	mRNA	XR_001041211.2	lncRNA
XM_012181866.1	GPHN	mRNA	TCONS_00029399	lncRNA
XM_012181866.1	GPHN	mRNA	TCONS_00029399	lncRNA
XM_012181866.1	GPHN	mRNA	TCONS_00029399	lncRNA
XM_012181866.1	GPHN	mRNA	TCONS_00029399	lncRNA
XM_012181866.1	GPHN	mRNA	TCONS_00029399	lncRNA
XM_012181866.1	GPHN	mRNA	TCONS_00029399	lncRNA
XM_012099837.2	SLMAP	mRNA	TCONS_00029399	lncRNA
XM_012099837.2	SLMAP	mRNA	TCONS_00029399	lncRNA
XM_012099837.2	SLMAP	mRNA	TCONS_00029399	lncRNA
XM_012099837.2	SLMAP	mRNA	TCONS_00029399	lncRNA
XM_012099837.2	SLMAP	mRNA	TCONS_00029399	lncRNA
XM_012099837.2	SLMAP	mRNA	TCONS_00029399	lncRNA
XM_004006434.3	PUS7L	mRNA	XR_001041211.2	lncRNA
XM_004006434.3	PUS7L	mRNA	XR_001041211.2	lncRNA

XM_004006434.3	PUS7L	mRNA	XR_001041211.2	lncRNA
XM_004006434.3	PUS7L	mRNA	XR_001041211.2	lncRNA
XM_004006434.3	PUS7L	mRNA	XR_001041211.2	lncRNA
XM_004006434.3	PUS7L	mRNA	XR_001041211.2	lncRNA
XM_015103117.1	ZBTB44	mRNA	TCONS_00029399	lncRNA
XM_015103117.1	ZBTB44	mRNA	TCONS_00029399	lncRNA
XM_015103117.1	ZBTB44	mRNA	TCONS_00029399	lncRNA
XM_015103117.1	ZBTB44	mRNA	TCONS_00029399	lncRNA
XM_015103117.1	ZBTB44	mRNA	TCONS_00029399	lncRNA
XM_015103117.1	ZBTB44	mRNA	TCONS_00029399	lncRNA
XM_012173383.2	DHX57	mRNA	TCONS_00029399	lncRNA
XM_012173383.2	DHX57	mRNA	TCONS_00029399	lncRNA
XM_012173383.2	DHX57	mRNA	TCONS_00029399	lncRNA
XM_012173383.2	DHX57	mRNA	TCONS_00029399	lncRNA
XM_012173383.2	DHX57	mRNA	TCONS_00029399	lncRNA
XM_012173383.2	DHX57	mRNA	TCONS_00029399	lncRNA
XM_015103117.1	ZBTB44	mRNA	XR_001041209.2	lncRNA
XM_015103117.1	ZBTB44	mRNA	XR_001041209.2	lncRNA
XM_015103117.1	ZBTB44	mRNA	XR_001041209.2	lncRNA
XM_015103117.1	ZBTB44	mRNA	XR_001041209.2	lncRNA
XM_015103117.1	ZBTB44	mRNA	XR_001041209.2	lncRNA
XM_015103117.1	ZBTB44	mRNA	XR_001041209.2	lncRNA
XM_012173383.2	DHX57	mRNA	XR_001041209.2	lncRNA
XM_012173383.2	DHX57	mRNA	XR_001041209.2	lncRNA
XM_012173383.2	DHX57	mRNA	XR_001041209.2	lncRNA
XM_012173383.2	DHX57	mRNA	XR_001041209.2	lncRNA
XM_012173383.2	DHX57	mRNA	XR_001041209.2	lncRNA
XM_012173383.2	DHX57	mRNA	XR_001041209.2	lncRNA
XM_012177139.2	TMEM209	mRNA	TCONS_00029399	lncRNA
XM_012177139.2	TMEM209	mRNA	TCONS_00029399	lncRNA
XM_012177139.2	TMEM209	mRNA	TCONS_00029399	lncRNA
XM_012177139.2	TMEM209	mRNA	TCONS_00029399	lncRNA
XM_012177139.2	TMEM209	mRNA	TCONS_00029399	lncRNA
XM_012177139.2	TMEM209	mRNA	TCONS_00029399	lncRNA
XM_012098342.2	FXR1	mRNA	TCONS_00029399	lncRNA
XM_012098342.2	FXR1	mRNA	TCONS_00029399	lncRNA
XM_012098342.2	FXR1	mRNA	TCONS_00029399	lncRNA
XM_012098342.2	FXR1	mRNA	TCONS_00029399	lncRNA
XM_012098342.2	FXR1	mRNA	TCONS_00029399	lncRNA
XM_012098342.2	FXR1	mRNA	TCONS_00029399	lncRNA
XM_012189777.1	CHD9	mRNA	TCONS_00029398	lncRNA
XM_012189777.1	CHD9	mRNA	TCONS_00029398	lncRNA
XM_012189777.1	CHD9	mRNA	TCONS_00029398	lncRNA
XM_012189777.1	CHD9	mRNA	TCONS_00029398	lncRNA
XM_012189777.1	CHD9	mRNA	TCONS_00029398	lncRNA
XM_012189777.1	CHD9	mRNA	TCONS_00029398	lncRNA
XM_015097018.1	ZNF280D	mRNA	XR_001436531.1	lncRNA
XM_015097018.1	ZNF280D	mRNA	XR_001436531.1	lncRNA
XM_015097018.1	ZNF280D	mRNA	XR_001436531.1	lncRNA
XM_015097018.1	ZNF280D	mRNA	XR_001436531.1	lncRNA
XM_015097018.1	ZNF280D	mRNA	XR_001436531.1	lncRNA
XM_015097018.1	ZNF280D	mRNA	XR_001436531.1	lncRNA
XM_015104131.1	TNRC6A	mRNA	XR_001435104.1	lncRNA
XM_015104131.1	TNRC6A	mRNA	XR_001435104.1	lncRNA

XM_015104131.1	TNRC6A	mRNA	XR_001435104.1	lncRNA
XM_015104131.1	TNRC6A	mRNA	XR_001435104.1	lncRNA
XM_015104131.1	TNRC6A	mRNA	XR_001435104.1	lncRNA
XM_015104131.1	TNRC6A	mRNA	XR_001435104.1	lncRNA
XM_012178308.2	FAM193B	mRNA	XR_001436609.1	lncRNA
XM_012178308.2	FAM193B	mRNA	XR_001436609.1	lncRNA
XM_012178308.2	FAM193B	mRNA	XR_001436609.1	lncRNA
XM_012178308.2	FAM193B	mRNA	XR_001436609.1	lncRNA
XM_012178308.2	FAM193B	mRNA	XR_001436609.1	lncRNA
XM_012178308.2	FAM193B	mRNA	XR_001436609.1	lncRNA
XM_012174295.2	ATF1	mRNA	XR_001436531.1	lncRNA
XM_012174295.2	ATF1	mRNA	XR_001436531.1	lncRNA
XM_012174295.2	ATF1	mRNA	XR_001436531.1	lncRNA
XM_012174295.2	ATF1	mRNA	XR_001436531.1	lncRNA
XM_012174295.2	ATF1	mRNA	XR_001436531.1	lncRNA
XM_012174295.2	ATF1	mRNA	XR_001436531.1	lncRNA
XM_015096551.1	MOB1B	mRNA	XR_001435104.1	lncRNA
XM_015096551.1	MOB1B	mRNA	XR_001435104.1	lncRNA
XM_015096551.1	MOB1B	mRNA	XR_001435104.1	lncRNA
XM_015096551.1	MOB1B	mRNA	XR_001435104.1	lncRNA
XM_015096551.1	MOB1B	mRNA	XR_001435104.1	lncRNA
XM_015096551.1	MOB1B	mRNA	XR_001435104.1	lncRNA
XM_015093088.1	CDCA2	mRNA	XR_001021689.2	lncRNA
XM_015093088.1	CDCA2	mRNA	XR_001021689.2	lncRNA
XM_015093088.1	CDCA2	mRNA	XR_001021689.2	lncRNA
XM_015093088.1	CDCA2	mRNA	XR_001021689.2	lncRNA
XM_015093088.1	CDCA2	mRNA	XR_001021689.2	lncRNA
XM_015093088.1	CDCA2	mRNA	XR_001021689.2	lncRNA
XM_012167258.2	CLIP4	mRNA	XR_001021689.2	lncRNA
XM_012167258.2	CLIP4	mRNA	XR_001021689.2	lncRNA
XM_012167258.2	CLIP4	mRNA	XR_001021689.2	lncRNA
XM_012167258.2	CLIP4	mRNA	XR_001021689.2	lncRNA
XM_012167258.2	CLIP4	mRNA	XR_001021689.2	lncRNA
XM_012167258.2	CLIP4	mRNA	XR_001021689.2	lncRNA
XM_012098436.1	KLHL25	mRNA	TCONS_00071561	lncRNA
XM_012098436.1	KLHL25	mRNA	TCONS_00071561	lncRNA
XM_012098436.1	KLHL25	mRNA	TCONS_00071561	lncRNA
XM_012098436.1	KLHL25	mRNA	TCONS_00071561	lncRNA
XM_012098436.1	KLHL25	mRNA	TCONS_00071561	lncRNA
XM_012098436.1	KLHL25	mRNA	TCONS_00071561	lncRNA
XM_012143382.2	PARD3B	mRNA	XR_001041202.1	lncRNA
XM_012143382.2	PARD3B	mRNA	XR_001041202.1	lncRNA
XM_012143382.2	PARD3B	mRNA	XR_001041202.1	lncRNA
XM_012143382.2	PARD3B	mRNA	XR_001041202.1	lncRNA
XM_012143382.2	PARD3B	mRNA	XR_001041202.1	lncRNA
XM_012143382.2	PARD3B	mRNA	XR_001041202.1	lncRNA
XM_004008093.2	PTN	mRNA	XR_001434081.1	lncRNA
XM_004008093.2	PTN	mRNA	XR_001434081.1	lncRNA
XM_004008093.2	PTN	mRNA	XR_001434081.1	lncRNA
XM_004008093.2	PTN	mRNA	XR_001434081.1	lncRNA
XM_004008093.2	PTN	mRNA	XR_001434081.1	lncRNA
XM_004008093.2	PTN	mRNA	XR_001434081.1	lncRNA
XM_012098458.2	FANCI	mRNA	XR_001434081.1	lncRNA
XM_012098458.2	FANCI	mRNA	XR_001434081.1	lncRNA

XM_012098458.2	FANCI	mRNA	XR_001434081.1	lncRNA
XM_012098458.2	FANCI	mRNA	XR_001434081.1	lncRNA
XM_012098458.2	FANCI	mRNA	XR_001434081.1	lncRNA
XM_012098458.2	FANCI	mRNA	XR_001434081.1	lncRNA
XM_012182190.2	EML5	mRNA	TCONS_00029398	lncRNA
XM_012182190.2	EML5	mRNA	TCONS_00029398	lncRNA
XM_012182190.2	EML5	mRNA	TCONS_00029398	lncRNA
XM_012182190.2	EML5	mRNA	TCONS_00029398	lncRNA
XM_012182190.2	EML5	mRNA	TCONS_00029398	lncRNA
XM_012182190.2	EML5	mRNA	TCONS_00029398	lncRNA
XM_015101011.1	TEAD1	mRNA	TCONS_00029398	lncRNA
XM_015101011.1	TEAD1	mRNA	TCONS_00029398	lncRNA
XM_015101011.1	TEAD1	mRNA	TCONS_00029398	lncRNA
XM_015101011.1	TEAD1	mRNA	TCONS_00029398	lncRNA
XM_015101011.1	TEAD1	mRNA	TCONS_00029398	lncRNA
XM_015101011.1	TEAD1	mRNA	TCONS_00029398	lncRNA
XM_012181966.2	SIPA1L1	mRNA	TCONS_00029398	lncRNA
XM_012181966.2	SIPA1L1	mRNA	TCONS_00029398	lncRNA
XM_012181966.2	SIPA1L1	mRNA	TCONS_00029398	lncRNA
XM_012181966.2	SIPA1L1	mRNA	TCONS_00029398	lncRNA
XM_012181966.2	SIPA1L1	mRNA	TCONS_00029398	lncRNA
XM_012181966.2	SIPA1L1	mRNA	TCONS_00029398	lncRNA
XM_012142889.2	ICA1L	mRNA	TCONS_00052150	lncRNA
XM_012142889.2	ICA1L	mRNA	TCONS_00052150	lncRNA
XM_012142889.2	ICA1L	mRNA	TCONS_00052150	lncRNA
XM_012142889.2	ICA1L	mRNA	TCONS_00052150	lncRNA
XM_012142889.2	ICA1L	mRNA	TCONS_00052150	lncRNA
XM_012096215.2	EHF	mRNA	XR_001021689.2	lncRNA
XM_012096215.2	EHF	mRNA	XR_001021689.2	lncRNA
XM_012096215.2	EHF	mRNA	XR_001021689.2	lncRNA
XM_012096215.2	EHF	mRNA	XR_001021689.2	lncRNA
XM_012096215.2	EHF	mRNA	XR_001021689.2	lncRNA
XM_012178872.2	AP4B1	mRNA	TCONS_00071561	lncRNA
XM_012178872.2	AP4B1	mRNA	TCONS_00071561	lncRNA
XM_012178872.2	AP4B1	mRNA	TCONS_00071561	lncRNA
XM_012178872.2	AP4B1	mRNA	TCONS_00071561	lncRNA
XM_012178872.2	AP4B1	mRNA	TCONS_00071561	lncRNA
XM_004012898.3	KRT34	mRNA	XR_001021689.2	lncRNA
XM_004012898.3	KRT34	mRNA	XR_001021689.2	lncRNA
XM_004012898.3	KRT34	mRNA	XR_001021689.2	lncRNA
XM_004012898.3	KRT34	mRNA	XR_001021689.2	lncRNA
XM_004012898.3	KRT34	mRNA	XR_001021689.2	lncRNA
XM_015103687.1	PTPN2	mRNA	XR_001435726.1	lncRNA
XM_015103687.1	PTPN2	mRNA	XR_001435726.1	lncRNA
XM_015103687.1	PTPN2	mRNA	XR_001435726.1	lncRNA
XM_015103687.1	PTPN2	mRNA	XR_001435726.1	lncRNA
XM_015103687.1	PTPN2	mRNA	XR_001435726.1	lncRNA
XM_015103687.1	PTPN2	mRNA	XR_001435726.1	lncRNA
XM_012133845.1	DYNC1I2	mRNA	XR_001042381.2	lncRNA
XM_012133845.1	DYNC1I2	mRNA	XR_001042381.2	lncRNA
XM_012133845.1	DYNC1I2	mRNA	XR_001042381.2	lncRNA
XM_012133845.1	DYNC1I2	mRNA	XR_001042381.2	lncRNA
XM_012133845.1	DYNC1I2	mRNA	XR_001042381.2	lncRNA
XM_012133845.1	DYNC1I2	mRNA	XR_001042381.2	lncRNA

XM_015103658.1	VAPA	mRNA	XR_001042381.2	lncRNA
XM_015103658.1	VAPA	mRNA	XR_001042381.2	lncRNA
XM_015103658.1	VAPA	mRNA	XR_001042381.2	lncRNA
XM_015103658.1	VAPA	mRNA	XR_001042381.2	lncRNA
XM_015103658.1	VAPA	mRNA	XR_001042381.2	lncRNA
XM_015103658.1	VAPA	mRNA	XR_001042381.2	lncRNA
XM_015103687.1	PTPN2	mRNA	XR_001435723.1	lncRNA
XM_015103687.1	PTPN2	mRNA	XR_001435723.1	lncRNA
XM_015103687.1	PTPN2	mRNA	XR_001435723.1	lncRNA
XM_015103687.1	PTPN2	mRNA	XR_001435723.1	lncRNA
XM_015103687.1	PTPN2	mRNA	XR_001435723.1	lncRNA
XM_015103687.1	PTPN2	mRNA	XR_001435723.1	lncRNA
XM_015102310.1	SETD5	mRNA	TCONS_00071561	lncRNA
XM_015102310.1	SETD5	mRNA	TCONS_00071561	lncRNA
XM_015102310.1	SETD5	mRNA	TCONS_00071561	lncRNA
XM_015102310.1	SETD5	mRNA	TCONS_00071561	lncRNA
XM_015102310.1	SETD5	mRNA	TCONS_00071561	lncRNA
XM_015102310.1	SETD5	mRNA	TCONS_00071561	lncRNA
XM_012140422.2	CLASP1	mRNA	TCONS_00071561	lncRNA
XM_012140422.2	CLASP1	mRNA	TCONS_00071561	lncRNA
XM_012140422.2	CLASP1	mRNA	TCONS_00071561	lncRNA
XM_012140422.2	CLASP1	mRNA	TCONS_00071561	lncRNA
XM_012140422.2	CLASP1	mRNA	TCONS_00071561	lncRNA
XM_012140422.2	CLASP1	mRNA	TCONS_00071561	lncRNA
XM_012188288.2	ARL5B	mRNA	XR_001041209.2	lncRNA
XM_012188288.2	ARL5B	mRNA	XR_001041209.2	lncRNA
XM_012188288.2	ARL5B	mRNA	XR_001041209.2	lncRNA
XM_012188288.2	ARL5B	mRNA	XR_001041209.2	lncRNA
XM_012188288.2	ARL5B	mRNA	XR_001041209.2	lncRNA
XM_012188288.2	ARL5B	mRNA	XR_001041209.2	lncRNA
XM_012113353.2	SUSD1	mRNA	XR_001041211.2	lncRNA
XM_012113353.2	SUSD1	mRNA	XR_001041211.2	lncRNA
XM_012113353.2	SUSD1	mRNA	XR_001041211.2	lncRNA
XM_012113353.2	SUSD1	mRNA	XR_001041211.2	lncRNA
XM_012113353.2	SUSD1	mRNA	XR_001041211.2	lncRNA
XM_012113353.2	SUSD1	mRNA	XR_001041211.2	lncRNA
XM_012180332.2	GSK3B	mRNA	XR_001041211.2	lncRNA
XM_012180332.2	GSK3B	mRNA	XR_001041211.2	lncRNA
XM_012180332.2	GSK3B	mRNA	XR_001041211.2	lncRNA
XM_012180332.2	GSK3B	mRNA	XR_001041211.2	lncRNA
XM_012180332.2	GSK3B	mRNA	XR_001041211.2	lncRNA
XM_012180332.2	GSK3B	mRNA	XR_001041211.2	lncRNA
XM_012142889.2	ICA1L	mRNA	XR_001041209.2	lncRNA
XM_012142889.2	ICA1L	mRNA	XR_001041209.2	lncRNA
XM_012142889.2	ICA1L	mRNA	XR_001041209.2	lncRNA
XM_012142889.2	ICA1L	mRNA	XR_001041209.2	lncRNA
XM_012142889.2	ICA1L	mRNA	XR_001041209.2	lncRNA
XM_012142889.2	ICA1L	mRNA	XR_001041209.2	lncRNA
XM_012187063.2	MARK1	mRNA	XR_001041209.2	lncRNA
XM_012187063.2	MARK1	mRNA	XR_001041209.2	lncRNA
XM_012187063.2	MARK1	mRNA	XR_001041209.2	lncRNA
XM_012187063.2	MARK1	mRNA	XR_001041209.2	lncRNA
XM_012187063.2	MARK1	mRNA	XR_001041209.2	lncRNA
XM_012187063.2	MARK1	mRNA	XR_001041209.2	lncRNA

XM_012188288.2	ARL5B	mRNA	TCONS_00029399	lncRNA
XM_012188288.2	ARL5B	mRNA	TCONS_00029399	lncRNA
XM_012188288.2	ARL5B	mRNA	TCONS_00029399	lncRNA
XM_012188288.2	ARL5B	mRNA	TCONS_00029399	lncRNA
XM_012188288.2	ARL5B	mRNA	TCONS_00029399	lncRNA
XM_012188288.2	ARL5B	mRNA	TCONS_00029399	lncRNA
XM_012102834.2	PPRC1	mRNA	XR_001041209.2	lncRNA
XM_012102834.2	PPRC1	mRNA	XR_001041209.2	lncRNA
XM_012102834.2	PPRC1	mRNA	XR_001041209.2	lncRNA
XM_012102834.2	PPRC1	mRNA	XR_001041209.2	lncRNA
XM_012102834.2	PPRC1	mRNA	XR_001041209.2	lncRNA
XM_012102834.2	PPRC1	mRNA	XR_001041209.2	lncRNA
XM_012190630.2	PLEKHA4	mRNA	TCONS_00029399	lncRNA
XM_012190630.2	PLEKHA4	mRNA	TCONS_00029399	lncRNA
XM_012190630.2	PLEKHA4	mRNA	TCONS_00029399	lncRNA
XM_012190630.2	PLEKHA4	mRNA	TCONS_00029399	lncRNA
XM_012190630.2	PLEKHA4	mRNA	TCONS_00029399	lncRNA
XM_012190630.2	PLEKHA4	mRNA	TCONS_00029399	lncRNA
XM_012187063.2	MARK1	mRNA	TCONS_00029399	lncRNA
XM_012187063.2	MARK1	mRNA	TCONS_00029399	lncRNA
XM_012187063.2	MARK1	mRNA	TCONS_00029399	lncRNA
XM_012187063.2	MARK1	mRNA	TCONS_00029399	lncRNA
XM_012187063.2	MARK1	mRNA	TCONS_00029399	lncRNA
XM_012187063.2	MARK1	mRNA	TCONS_00029399	lncRNA
XM_012190630.2	PLEKHA4	mRNA	XR_001041209.2	lncRNA
XM_012190630.2	PLEKHA4	mRNA	XR_001041209.2	lncRNA
XM_012190630.2	PLEKHA4	mRNA	XR_001041209.2	lncRNA
XM_012190630.2	PLEKHA4	mRNA	XR_001041209.2	lncRNA
XM_012190630.2	PLEKHA4	mRNA	XR_001041209.2	lncRNA
XM_012190630.2	PLEKHA4	mRNA	XR_001041209.2	lncRNA
XM_012177174.2	CALD1	mRNA	XR_001041211.2	lncRNA
XM_012177174.2	CALD1	mRNA	XR_001041211.2	lncRNA
XM_012177174.2	CALD1	mRNA	XR_001041211.2	lncRNA
XM_012177174.2	CALD1	mRNA	XR_001041211.2	lncRNA
XM_012177174.2	CALD1	mRNA	XR_001041211.2	lncRNA
XM_012177174.2	CALD1	mRNA	XR_001041211.2	lncRNA
XM_015105096.1	PAK3	mRNA	XR_001041211.2	lncRNA
XM_015105096.1	PAK3	mRNA	XR_001041211.2	lncRNA
XM_015105096.1	PAK3	mRNA	XR_001041211.2	lncRNA
XM_015105096.1	PAK3	mRNA	XR_001041211.2	lncRNA
XM_015105096.1	PAK3	mRNA	XR_001041211.2	lncRNA
XM_015105096.1	PAK3	mRNA	XR_001041211.2	lncRNA
XM_012102834.2	PPRC1	mRNA	TCONS_00029399	lncRNA
XM_012102834.2	PPRC1	mRNA	TCONS_00029399	lncRNA
XM_012102834.2	PPRC1	mRNA	TCONS_00029399	lncRNA
XM_012102834.2	PPRC1	mRNA	TCONS_00029399	lncRNA
XM_012102834.2	PPRC1	mRNA	TCONS_00029399	lncRNA
XM_012102834.2	PPRC1	mRNA	TCONS_00029399	lncRNA
XM_015092393.1	NRIP1	mRNA	XR_001041211.2	lncRNA
XM_015092393.1	NRIP1	mRNA	XR_001041211.2	lncRNA
XM_015092393.1	NRIP1	mRNA	XR_001041211.2	lncRNA
XM_015092393.1	NRIP1	mRNA	XR_001041211.2	lncRNA
XM_015092393.1	NRIP1	mRNA	XR_001041211.2	lncRNA
XM_015092393.1	NRIP1	mRNA	XR_001041211.2	lncRNA

XM_012177175.2	CALD1	mRNA	XR_001041211.2	lncRNA
XM_012177175.2	CALD1	mRNA	XR_001041211.2	lncRNA
XM_012177175.2	CALD1	mRNA	XR_001041211.2	lncRNA
XM_012177175.2	CALD1	mRNA	XR_001041211.2	lncRNA
XM_012177175.2	CALD1	mRNA	XR_001041211.2	lncRNA
XM_012177175.2	CALD1	mRNA	XR_001041211.2	lncRNA
XM_015104393.1	RUFY2	mRNA	TCONS_00029399	lncRNA
XM_015104393.1	RUFY2	mRNA	TCONS_00029399	lncRNA
XM_015104393.1	RUFY2	mRNA	TCONS_00029399	lncRNA
XM_015104393.1	RUFY2	mRNA	TCONS_00029399	lncRNA
XM_015104393.1	RUFY2	mRNA	TCONS_00029399	lncRNA
XM_015104393.1	RUFY2	mRNA	TCONS_00029399	lncRNA
XM_004001781.3	ZSCAN20	mRNA	TCONS_00029399	lncRNA
XM_004001781.3	ZSCAN20	mRNA	TCONS_00029399	lncRNA
XM_004001781.3	ZSCAN20	mRNA	TCONS_00029399	lncRNA
XM_004001781.3	ZSCAN20	mRNA	TCONS_00029399	lncRNA
XM_004001781.3	ZSCAN20	mRNA	TCONS_00029399	lncRNA
XM_004001781.3	ZSCAN20	mRNA	TCONS_00029399	lncRNA
XM_015094782.1	MARS	mRNA	XR_001021939.1	lncRNA
XM_015094782.1	MARS	mRNA	XR_001021939.1	lncRNA
XM_015094782.1	MARS	mRNA	XR_001021939.1	lncRNA
XM_015094782.1	MARS	mRNA	XR_001021939.1	lncRNA
XM_015094782.1	MARS	mRNA	XR_001021939.1	lncRNA
XM_015094782.1	MARS	mRNA	XR_001021939.1	lncRNA
XM_004017729.3	CHL1	mRNA	XR_001041202.1	lncRNA
XM_004017729.3	CHL1	mRNA	XR_001041202.1	lncRNA
XM_004017729.3	CHL1	mRNA	XR_001041202.1	lncRNA
XM_004017729.3	CHL1	mRNA	XR_001041202.1	lncRNA
XM_004017729.3	CHL1	mRNA	XR_001041202.1	lncRNA
XM_004017729.3	CHL1	mRNA	XR_001041202.1	lncRNA
XM_012179952.2	FRYL	mRNA	TCONS_00052150	lncRNA
XM_012179952.2	FRYL	mRNA	TCONS_00052150	lncRNA
XM_012179952.2	FRYL	mRNA	TCONS_00052150	lncRNA
XM_012179952.2	FRYL	mRNA	TCONS_00052150	lncRNA
XM_012179952.2	FRYL	mRNA	TCONS_00052150	lncRNA
XM_012103019.2	INPP5F	mRNA	TCONS_00052150	lncRNA
XM_012103019.2	INPP5F	mRNA	TCONS_00052150	lncRNA
XM_012103019.2	INPP5F	mRNA	TCONS_00052150	lncRNA
XM_012103019.2	INPP5F	mRNA	TCONS_00052150	lncRNA
XM_012103019.2	INPP5F	mRNA	TCONS_00052150	lncRNA
XM_015098446.1	VEZF1	mRNA	TCONS_00078442	lncRNA
XM_015098446.1	VEZF1	mRNA	TCONS_00078442	lncRNA
XM_015098446.1	VEZF1	mRNA	TCONS_00078442	lncRNA
XM_015098446.1	VEZF1	mRNA	TCONS_00078442	lncRNA
XM_015098446.1	VEZF1	mRNA	TCONS_00078442	lncRNA
XM_015096569.1	CDKL2	mRNA	XR_001041202.1	lncRNA
XM_015096569.1	CDKL2	mRNA	XR_001041202.1	lncRNA
XM_015096569.1	CDKL2	mRNA	XR_001041202.1	lncRNA
XM_015096569.1	CDKL2	mRNA	XR_001041202.1	lncRNA
XM_015096569.1	CDKL2	mRNA	XR_001041202.1	lncRNA
XM_012189447.2	ALCAM	mRNA	XR_001436609.1	lncRNA
XM_012189447.2	ALCAM	mRNA	XR_001436609.1	lncRNA
XM_012189447.2	ALCAM	mRNA	XR_001436609.1	lncRNA
XM_012189447.2	ALCAM	mRNA	XR_001436609.1	lncRNA

XM_012189447.2	ALCAM	mRNA	XR_001436609.1	lncRNA
XM_012189447.2	ALCAM	mRNA	XR_001436609.1	lncRNA
XM_012105225.2	SGPL1	mRNA	XR_001436609.1	lncRNA
XM_012105225.2	SGPL1	mRNA	XR_001436609.1	lncRNA
XM_012105225.2	SGPL1	mRNA	XR_001436609.1	lncRNA
XM_012105225.2	SGPL1	mRNA	XR_001436609.1	lncRNA
XM_012105225.2	SGPL1	mRNA	XR_001436609.1	lncRNA
XM_012105225.2	SGPL1	mRNA	XR_001436609.1	lncRNA
XM_004008093.2	PTN	mRNA	XR_001436609.1	lncRNA
XM_004008093.2	PTN	mRNA	XR_001436609.1	lncRNA
XM_004008093.2	PTN	mRNA	XR_001436609.1	lncRNA
XM_004008093.2	PTN	mRNA	XR_001436609.1	lncRNA
XM_004008093.2	PTN	mRNA	XR_001436609.1	lncRNA
XM_004008093.2	PTN	mRNA	XR_001436609.1	lncRNA
XM_012105333.2	USP54	mRNA	XR_001434081.1	lncRNA
XM_012105333.2	USP54	mRNA	XR_001434081.1	lncRNA
XM_012105333.2	USP54	mRNA	XR_001434081.1	lncRNA
XM_012105333.2	USP54	mRNA	XR_001434081.1	lncRNA
XM_012105333.2	USP54	mRNA	XR_001434081.1	lncRNA
XM_012105333.2	USP54	mRNA	XR_001434081.1	lncRNA
XM_015099256.1	CEP104	mRNA	XR_001434081.1	lncRNA
XM_015099256.1	CEP104	mRNA	XR_001434081.1	lncRNA
XM_015099256.1	CEP104	mRNA	XR_001434081.1	lncRNA
XM_015099256.1	CEP104	mRNA	XR_001434081.1	lncRNA
XM_015099256.1	CEP104	mRNA	XR_001434081.1	lncRNA
XM_015099256.1	CEP104	mRNA	XR_001434081.1	lncRNA
XM_015091887.1	MYCL	mRNA	XR_001021689.2	lncRNA
XM_015091887.1	MYCL	mRNA	XR_001021689.2	lncRNA
XM_015091887.1	MYCL	mRNA	XR_001021689.2	lncRNA
XM_015091887.1	MYCL	mRNA	XR_001021689.2	lncRNA
XM_015091887.1	MYCL	mRNA	XR_001021689.2	lncRNA
XM_015091887.1	MYCL	mRNA	XR_001021689.2	lncRNA
XM_012180278.2	LIN54	mRNA	XR_001039281.1	lncRNA
XM_012180278.2	LIN54	mRNA	XR_001039281.1	lncRNA
XM_012180278.2	LIN54	mRNA	XR_001039281.1	lncRNA
XM_012180278.2	LIN54	mRNA	XR_001039281.1	lncRNA
XM_012180278.2	LIN54	mRNA	XR_001039281.1	lncRNA
XM_012103723.1	POLI	mRNA	TCONS_00072289	lncRNA
XM_012103723.1	POLI	mRNA	TCONS_00072289	lncRNA
XM_012103723.1	POLI	mRNA	TCONS_00072289	lncRNA
XM_012103723.1	POLI	mRNA	TCONS_00072289	lncRNA
XM_012103723.1	POLI	mRNA	TCONS_00072289	lncRNA
XM_012099916.2	PRKCD	mRNA	XR_001041211.2	lncRNA
XM_012099916.2	PRKCD	mRNA	XR_001041211.2	lncRNA
XM_012099916.2	PRKCD	mRNA	XR_001041211.2	lncRNA
XM_012099916.2	PRKCD	mRNA	XR_001041211.2	lncRNA
XM_012099916.2	PRKCD	mRNA	XR_001041211.2	lncRNA
XM_012099916.2	PRKCD	mRNA	XR_001041211.2	lncRNA
XM_012134516.1	PPIG	mRNA	XR_001436099.1	lncRNA
XM_012134516.1	PPIG	mRNA	XR_001436099.1	lncRNA
XM_012134516.1	PPIG	mRNA	XR_001436099.1	lncRNA
XM_012134516.1	PPIG	mRNA	XR_001436099.1	lncRNA
XM_012134516.1	PPIG	mRNA	XR_001436099.1	lncRNA
XM_012178872.2	AP4B1	mRNA	XR_001033284.2	lncRNA

XM_012178872.2	AP4B1	mRNA	XR_001033284.2	lncRNA
XM_012178872.2	AP4B1	mRNA	XR_001033284.2	lncRNA
XM_012178872.2	AP4B1	mRNA	XR_001033284.2	lncRNA
XM_012178872.2	AP4B1	mRNA	XR_001033284.2	lncRNA
XM_012096215.2	EHF	mRNA	XR_001033284.2	lncRNA
XM_012096215.2	EHF	mRNA	XR_001033284.2	lncRNA
XM_012096215.2	EHF	mRNA	XR_001033284.2	lncRNA
XM_012096215.2	EHF	mRNA	XR_001033284.2	lncRNA
XM_012096215.2	EHF	mRNA	XR_001033284.2	lncRNA
XM_012134451.2	PPIG	mRNA	XR_001436099.1	lncRNA
XM_012134451.2	PPIG	mRNA	XR_001436099.1	lncRNA
XM_012134451.2	PPIG	mRNA	XR_001436099.1	lncRNA
XM_012134451.2	PPIG	mRNA	XR_001436099.1	lncRNA
XM_012134451.2	PPIG	mRNA	XR_001436099.1	lncRNA
XM_015097545.1	PHF10	mRNA	XR_001436099.1	lncRNA
XM_015097545.1	PHF10	mRNA	XR_001436099.1	lncRNA
XM_015097545.1	PHF10	mRNA	XR_001436099.1	lncRNA
XM_015097545.1	PHF10	mRNA	XR_001436099.1	lncRNA
XM_015097545.1	PHF10	mRNA	XR_001436099.1	lncRNA
XM_012101112.2	ABT1	mRNA	TCONS_00052150	lncRNA
XM_012101112.2	ABT1	mRNA	TCONS_00052150	lncRNA
XM_012101112.2	ABT1	mRNA	TCONS_00052150	lncRNA
XM_012101112.2	ABT1	mRNA	TCONS_00052150	lncRNA
XM_012101112.2	ABT1	mRNA	TCONS_00052150	lncRNA
XM_012173782.2	REV1	mRNA	TCONS_00052150	lncRNA
XM_012173782.2	REV1	mRNA	TCONS_00052150	lncRNA
XM_012173782.2	REV1	mRNA	TCONS_00052150	lncRNA
XM_012173782.2	REV1	mRNA	TCONS_00052150	lncRNA
XM_012173782.2	REV1	mRNA	TCONS_00052150	lncRNA
XM_012098436.1	KLHL25	mRNA	XR_001033284.2	lncRNA
XM_012098436.1	KLHL25	mRNA	XR_001033284.2	lncRNA
XM_012098436.1	KLHL25	mRNA	XR_001033284.2	lncRNA
XM_012098436.1	KLHL25	mRNA	XR_001033284.2	lncRNA
XM_012098436.1	KLHL25	mRNA	XR_001033284.2	lncRNA
XM_012098436.1	KLHL25	mRNA	XR_001033284.2	lncRNA
XM_012176365.2	SLC25A13	mRNA	XR_001033284.2	lncRNA
XM_012176365.2	SLC25A13	mRNA	XR_001033284.2	lncRNA
XM_012176365.2	SLC25A13	mRNA	XR_001033284.2	lncRNA
XM_012176365.2	SLC25A13	mRNA	XR_001033284.2	lncRNA
XM_012176365.2	SLC25A13	mRNA	XR_001033284.2	lncRNA
XM_012176365.2	SLC25A13	mRNA	XR_001033284.2	lncRNA
XM_004017729.3	CHL1	mRNA	XR_001436099.1	lncRNA
XM_004017729.3	CHL1	mRNA	XR_001436099.1	lncRNA
XM_004017729.3	CHL1	mRNA	XR_001436099.1	lncRNA
XM_004017729.3	CHL1	mRNA	XR_001436099.1	lncRNA
XM_004017729.3	CHL1	mRNA	XR_001436099.1	lncRNA
XM_004017729.3	CHL1	mRNA	XR_001436099.1	lncRNA
XM_004008093.2	PTN	mRNA	XR_001435726.1	lncRNA
XM_004008093.2	PTN	mRNA	XR_001435726.1	lncRNA
XM_004008093.2	PTN	mRNA	XR_001435726.1	lncRNA
XM_004008093.2	PTN	mRNA	XR_001435726.1	lncRNA
XM_004008093.2	PTN	mRNA	XR_001435726.1	lncRNA
XM_004008093.2	PTN	mRNA	XR_001435726.1	lncRNA
XM_004012689.3	MYH10	mRNA	XR_001042381.2	lncRNA

XM_004012689.3	MYH10	mRNA	XR_001042381.2	lncRNA
XM_004012689.3	MYH10	mRNA	XR_001042381.2	lncRNA
XM_004012689.3	MYH10	mRNA	XR_001042381.2	lncRNA
XM_004012689.3	MYH10	mRNA	XR_001042381.2	lncRNA
XM_004012689.3	MYH10	mRNA	XR_001042381.2	lncRNA
XM_012178138.2	P4HA2	mRNA	XR_001435726.1	lncRNA
XM_012178138.2	P4HA2	mRNA	XR_001435726.1	lncRNA
XM_012178138.2	P4HA2	mRNA	XR_001435726.1	lncRNA
XM_012178138.2	P4HA2	mRNA	XR_001435726.1	lncRNA
XM_012178138.2	P4HA2	mRNA	XR_001435726.1	lncRNA
XM_012178138.2	P4HA2	mRNA	XR_001435726.1	lncRNA
XM_012098348.2	MEF2A	mRNA	XR_001435726.1	lncRNA
XM_012098348.2	MEF2A	mRNA	XR_001435726.1	lncRNA
XM_012098348.2	MEF2A	mRNA	XR_001435726.1	lncRNA
XM_012098348.2	MEF2A	mRNA	XR_001435726.1	lncRNA
XM_012098348.2	MEF2A	mRNA	XR_001435726.1	lncRNA
XM_012098348.2	MEF2A	mRNA	XR_001435726.1	lncRNA
XM_012098348.2	MEF2A	mRNA	XR_001435723.1	lncRNA
XM_012098348.2	MEF2A	mRNA	XR_001435723.1	lncRNA
XM_012098348.2	MEF2A	mRNA	XR_001435723.1	lncRNA
XM_012098348.2	MEF2A	mRNA	XR_001435723.1	lncRNA
XM_012098348.2	MEF2A	mRNA	XR_001435723.1	lncRNA
XM_012098348.2	MEF2A	mRNA	XR_001435723.1	lncRNA
XM_012100126.2	MAP4	mRNA	XR_001042381.2	lncRNA
XM_012100126.2	MAP4	mRNA	XR_001042381.2	lncRNA
XM_012100126.2	MAP4	mRNA	XR_001042381.2	lncRNA
XM_012100126.2	MAP4	mRNA	XR_001042381.2	lncRNA
XM_012100126.2	MAP4	mRNA	XR_001042381.2	lncRNA
XM_012100126.2	MAP4	mRNA	XR_001042381.2	lncRNA
XM_015093350.1	GPR155	mRNA	TCONS_00072289	lncRNA
XM_015093350.1	GPR155	mRNA	TCONS_00072289	lncRNA
XM_015093350.1	GPR155	mRNA	TCONS_00072289	lncRNA
XM_015093350.1	GPR155	mRNA	TCONS_00072289	lncRNA
XM_015093350.1	GPR155	mRNA	TCONS_00072289	lncRNA
XM_004012194.3	RBM26	mRNA	XR_001039281.1	lncRNA
XM_004012194.3	RBM26	mRNA	XR_001039281.1	lncRNA
XM_004012194.3	RBM26	mRNA	XR_001039281.1	lncRNA
XM_004012194.3	RBM26	mRNA	XR_001039281.1	lncRNA
XM_004012194.3	RBM26	mRNA	XR_001039281.1	lncRNA
XM_012114904.1	RNF20	mRNA	TCONS_00072289	lncRNA
XM_012114904.1	RNF20	mRNA	TCONS_00072289	lncRNA
XM_012114904.1	RNF20	mRNA	TCONS_00072289	lncRNA
XM_012114904.1	RNF20	mRNA	TCONS_00072289	lncRNA
XM_012114904.1	RNF20	mRNA	TCONS_00072289	lncRNA
XM_012187630.2	SOAT1	mRNA	XR_001021689.2	lncRNA
XM_012187630.2	SOAT1	mRNA	XR_001021689.2	lncRNA
XM_012187630.2	SOAT1	mRNA	XR_001021689.2	lncRNA
XM_012187630.2	SOAT1	mRNA	XR_001021689.2	lncRNA
XM_012187630.2	SOAT1	mRNA	XR_001021689.2	lncRNA
XM_015093101.1	PPP3CC	mRNA	TCONS_00071561	lncRNA
XM_015093101.1	PPP3CC	mRNA	TCONS_00071561	lncRNA
XM_015093101.1	PPP3CC	mRNA	TCONS_00071561	lncRNA
XM_015093101.1	PPP3CC	mRNA	TCONS_00071561	lncRNA
XM_015093101.1	PPP3CC	mRNA	TCONS_00071561	lncRNA

XM_015097359.1	ARMC2	mRNA	XR_001021689.2	lncRNA
XM_015097359.1	ARMC2	mRNA	XR_001021689.2	lncRNA
XM_015097359.1	ARMC2	mRNA	XR_001021689.2	lncRNA
XM_015097359.1	ARMC2	mRNA	XR_001021689.2	lncRNA
XM_015097359.1	ARMC2	mRNA	XR_001021689.2	lncRNA
XM_012179604.2	UBE2D3	mRNA	TCONS_00071561	lncRNA
XM_012179604.2	UBE2D3	mRNA	TCONS_00071561	lncRNA
XM_012179604.2	UBE2D3	mRNA	TCONS_00071561	lncRNA
XM_012179604.2	UBE2D3	mRNA	TCONS_00071561	lncRNA
XM_012179604.2	UBE2D3	mRNA	TCONS_00071561	lncRNA
XM_012187919.2	LOC1056166	mRNA	XR_001021689.2	lncRNA
XM_012187919.2	LOC1056166	mRNA	XR_001021689.2	lncRNA
XM_012187919.2	LOC1056166	mRNA	XR_001021689.2	lncRNA
XM_012187919.2	LOC1056166	mRNA	XR_001021689.2	lncRNA
XM_012187919.2	LOC1056166	mRNA	XR_001021689.2	lncRNA
XM_012106040.2	LOC1011208	mRNA	XR_001021689.2	lncRNA
XM_012106040.2	LOC1011208	mRNA	XR_001021689.2	lncRNA
XM_012106040.2	LOC1011208	mRNA	XR_001021689.2	lncRNA
XM_012106040.2	LOC1011208	mRNA	XR_001021689.2	lncRNA
XM_012106040.2	LOC1011208	mRNA	XR_001021689.2	lncRNA
XM_015099863.1	DSN1	mRNA	TCONS_00071561	lncRNA
XM_015099863.1	DSN1	mRNA	TCONS_00071561	lncRNA
XM_015099863.1	DSN1	mRNA	TCONS_00071561	lncRNA
XM_015099863.1	DSN1	mRNA	TCONS_00071561	lncRNA
XM_015099863.1	DSN1	mRNA	TCONS_00071561	lncRNA
XM_012124912.1	KIAA2026	mRNA	XR_001021689.2	lncRNA
XM_012124912.1	KIAA2026	mRNA	XR_001021689.2	lncRNA
XM_012124912.1	KIAA2026	mRNA	XR_001021689.2	lncRNA
XM_012124912.1	KIAA2026	mRNA	XR_001021689.2	lncRNA
XM_012124912.1	KIAA2026	mRNA	XR_001021689.2	lncRNA
XM_012187240.2	SLC19A2	mRNA	XR_001041209.2	lncRNA
XM_012187240.2	SLC19A2	mRNA	XR_001041209.2	lncRNA
XM_012187240.2	SLC19A2	mRNA	XR_001041209.2	lncRNA
XM_012187240.2	SLC19A2	mRNA	XR_001041209.2	lncRNA
XM_012187240.2	SLC19A2	mRNA	XR_001041209.2	lncRNA
XM_012180229.1	CCNG2	mRNA	XR_001041209.2	lncRNA
XM_012180229.1	CCNG2	mRNA	XR_001041209.2	lncRNA
XM_012180229.1	CCNG2	mRNA	XR_001041209.2	lncRNA
XM_012180229.1	CCNG2	mRNA	XR_001041209.2	lncRNA
XM_012180229.1	CCNG2	mRNA	XR_001041209.2	lncRNA
XM_012178872.2	AP4B1	mRNA	TCONS_00029399	lncRNA
XM_012178872.2	AP4B1	mRNA	TCONS_00029399	lncRNA
XM_012178872.2	AP4B1	mRNA	TCONS_00029399	lncRNA
XM_012178872.2	AP4B1	mRNA	TCONS_00029399	lncRNA
XM_012178872.2	AP4B1	mRNA	TCONS_00029399	lncRNA
XM_012187727.2	TRMT1L	mRNA	XR_001041211.2	lncRNA
XM_012187727.2	TRMT1L	mRNA	XR_001041211.2	lncRNA
XM_012187727.2	TRMT1L	mRNA	XR_001041211.2	lncRNA
XM_012187727.2	TRMT1L	mRNA	XR_001041211.2	lncRNA
XM_012187727.2	TRMT1L	mRNA	XR_001041211.2	lncRNA
XM_012119517.2	SORBS3	mRNA	TCONS_00029399	lncRNA
XM_012119517.2	SORBS3	mRNA	TCONS_00029399	lncRNA
XM_012119517.2	SORBS3	mRNA	TCONS_00029399	lncRNA
XM_012119517.2	SORBS3	mRNA	TCONS_00029399	lncRNA

XM_012119517.2	SORBS3	mRNA	TCONS_00029399	lncRNA
XM_012178872.2	AP4B1	mRNA	XR_001041209.2	lncRNA
XM_012178872.2	AP4B1	mRNA	XR_001041209.2	lncRNA
XM_012178872.2	AP4B1	mRNA	XR_001041209.2	lncRNA
XM_012178872.2	AP4B1	mRNA	XR_001041209.2	lncRNA
XM_012178872.2	AP4B1	mRNA	XR_001041209.2	lncRNA
XM_012104035.2	CLUAP1	mRNA	TCONS_00029399	lncRNA
XM_012104035.2	CLUAP1	mRNA	TCONS_00029399	lncRNA
XM_012104035.2	CLUAP1	mRNA	TCONS_00029399	lncRNA
XM_012104035.2	CLUAP1	mRNA	TCONS_00029399	lncRNA
XM_012104035.2	CLUAP1	mRNA	TCONS_00029399	lncRNA
XM_012095461.2	GOLGB1	mRNA	TCONS_00052150	lncRNA
XM_012095461.2	GOLGB1	mRNA	TCONS_00052150	lncRNA
XM_012095461.2	GOLGB1	mRNA	TCONS_00052150	lncRNA
XM_012095461.2	GOLGB1	mRNA	TCONS_00052150	lncRNA
XM_012095461.2	GOLGB1	mRNA	TCONS_00052150	lncRNA
XM_012177174.2	CALD1	mRNA	XR_001436609.1	lncRNA
XM_012177174.2	CALD1	mRNA	XR_001436609.1	lncRNA
XM_012177174.2	CALD1	mRNA	XR_001436609.1	lncRNA
XM_012177174.2	CALD1	mRNA	XR_001436609.1	lncRNA
XM_012177174.2	CALD1	mRNA	XR_001436609.1	lncRNA
XM_012177174.2	CALD1	mRNA	XR_001436609.1	lncRNA
XM_012177175.2	CALD1	mRNA	XR_001436609.1	lncRNA
XM_012177175.2	CALD1	mRNA	XR_001436609.1	lncRNA
XM_012177175.2	CALD1	mRNA	XR_001436609.1	lncRNA
XM_012177175.2	CALD1	mRNA	XR_001436609.1	lncRNA
XM_012177175.2	CALD1	mRNA	XR_001436609.1	lncRNA
XM_012177175.2	CALD1	mRNA	XR_001436609.1	lncRNA
XM_015099256.1	CEP104	mRNA	XR_001436609.1	lncRNA
XM_015099256.1	CEP104	mRNA	XR_001436609.1	lncRNA
XM_015099256.1	CEP104	mRNA	XR_001436609.1	lncRNA
XM_015099256.1	CEP104	mRNA	XR_001436609.1	lncRNA
XM_015099256.1	CEP104	mRNA	XR_001436609.1	lncRNA
XM_015099256.1	CEP104	mRNA	XR_001436609.1	lncRNA
XM_015093632.1	EPB41	mRNA	XR_001041211.2	lncRNA
XM_015093632.1	EPB41	mRNA	XR_001041211.2	lncRNA
XM_015093632.1	EPB41	mRNA	XR_001041211.2	lncRNA
XM_015093632.1	EPB41	mRNA	XR_001041211.2	lncRNA
XM_015093632.1	EPB41	mRNA	XR_001041211.2	lncRNA
XM_015093632.1	EPB41	mRNA	XR_001041211.2	lncRNA
XM_015096216.1	PAM	mRNA	XR_001041211.2	lncRNA
XM_015096216.1	PAM	mRNA	XR_001041211.2	lncRNA
XM_015096216.1	PAM	mRNA	XR_001041211.2	lncRNA
XM_015096216.1	PAM	mRNA	XR_001041211.2	lncRNA
XM_015096216.1	PAM	mRNA	XR_001041211.2	lncRNA
XM_015096216.1	PAM	mRNA	XR_001041211.2	lncRNA
XM_015101011.1	TEAD1	mRNA	TCONS_00078442	lncRNA
XM_015101011.1	TEAD1	mRNA	TCONS_00078442	lncRNA
XM_015101011.1	TEAD1	mRNA	TCONS_00078442	lncRNA
XM_015101011.1	TEAD1	mRNA	TCONS_00078442	lncRNA
XM_015101011.1	TEAD1	mRNA	TCONS_00078442	lncRNA
XM_012182190.2	EML5	mRNA	TCONS_00052150	lncRNA
XM_012182190.2	EML5	mRNA	TCONS_00052150	lncRNA
XM_012182190.2	EML5	mRNA	TCONS_00052150	lncRNA

XM_012182190.2	EML5	mRNA	TCONS_00052150	lncRNA
XM_012182190.2	EML5	mRNA	TCONS_00052150	lncRNA
XM_004012037.3	TDRD3	mRNA	XR_001434081.1	lncRNA
XM_004012037.3	TDRD3	mRNA	XR_001434081.1	lncRNA
XM_004012037.3	TDRD3	mRNA	XR_001434081.1	lncRNA
XM_004012037.3	TDRD3	mRNA	XR_001434081.1	lncRNA
XM_004012037.3	TDRD3	mRNA	XR_001434081.1	lncRNA
XM_012183409.2	PHF3	mRNA	TCONS_00072289	lncRNA
XM_012183409.2	PHF3	mRNA	TCONS_00072289	lncRNA
XM_012183409.2	PHF3	mRNA	TCONS_00072289	lncRNA
XM_012183409.2	PHF3	mRNA	TCONS_00072289	lncRNA
XM_012183409.2	PHF3	mRNA	TCONS_00072289	lncRNA
XM_012099659.2	FOXP1	mRNA	XR_001039281.1	lncRNA
XM_012099659.2	FOXP1	mRNA	XR_001039281.1	lncRNA
XM_012099659.2	FOXP1	mRNA	XR_001039281.1	lncRNA
XM_012099659.2	FOXP1	mRNA	XR_001039281.1	lncRNA
XM_012099659.2	FOXP1	mRNA	XR_001039281.1	lncRNA
XM_004006165.3	ITPRIPL1	mRNA	TCONS_00072289	lncRNA
XM_004006165.3	ITPRIPL1	mRNA	TCONS_00072289	lncRNA
XM_004006165.3	ITPRIPL1	mRNA	TCONS_00072289	lncRNA
XM_004006165.3	ITPRIPL1	mRNA	TCONS_00072289	lncRNA
XM_004006165.3	ITPRIPL1	mRNA	TCONS_00072289	lncRNA
XM_012174897.2	UTP20	mRNA	XR_001039281.1	lncRNA
XM_012174897.2	UTP20	mRNA	XR_001039281.1	lncRNA
XM_012174897.2	UTP20	mRNA	XR_001039281.1	lncRNA
XM_012174897.2	UTP20	mRNA	XR_001039281.1	lncRNA
XM_012174897.2	UTP20	mRNA	XR_001039281.1	lncRNA
XM_015101107.1	TCP11L1	mRNA	TCONS_00072289	lncRNA
XM_015101107.1	TCP11L1	mRNA	TCONS_00072289	lncRNA
XM_015101107.1	TCP11L1	mRNA	TCONS_00072289	lncRNA
XM_015101107.1	TCP11L1	mRNA	TCONS_00072289	lncRNA
XM_015101107.1	TCP11L1	mRNA	TCONS_00072289	lncRNA
XM_012182538.2	TRAPPC3L	mRNA	XR_001039281.1	lncRNA
XM_012182538.2	TRAPPC3L	mRNA	XR_001039281.1	lncRNA
XM_012182538.2	TRAPPC3L	mRNA	XR_001039281.1	lncRNA
XM_012182538.2	TRAPPC3L	mRNA	XR_001039281.1	lncRNA
XM_012182538.2	TRAPPC3L	mRNA	XR_001039281.1	lncRNA
XM_015096769.1	APC	mRNA	XR_001041209.2	lncRNA
XM_015096769.1	APC	mRNA	XR_001041209.2	lncRNA
XM_015096769.1	APC	mRNA	XR_001041209.2	lncRNA
XM_015096769.1	APC	mRNA	XR_001041209.2	lncRNA
XM_015096769.1	APC	mRNA	XR_001041209.2	lncRNA
XM_015096769.1	APC	mRNA	XR_001041209.2	lncRNA
XM_012099956.2	BAP1	mRNA	XR_001021939.1	lncRNA
XM_012099956.2	BAP1	mRNA	XR_001021939.1	lncRNA
XM_012099956.2	BAP1	mRNA	XR_001021939.1	lncRNA
XM_012099956.2	BAP1	mRNA	XR_001021939.1	lncRNA
XM_012099956.2	BAP1	mRNA	XR_001021939.1	lncRNA
XM_012096215.2	EHF	mRNA	XR_001021939.1	lncRNA
XM_012096215.2	EHF	mRNA	XR_001021939.1	lncRNA
XM_012096215.2	EHF	mRNA	XR_001021939.1	lncRNA
XM_012096215.2	EHF	mRNA	XR_001021939.1	lncRNA
XM_012096215.2	EHF	mRNA	XR_001021939.1	lncRNA
XM_012187727.2	TRMT1L	mRNA	XR_001021939.1	lncRNA

XM_012187727.2	TRMT1L	mRNA	XR_001021939.1	lncRNA
XM_012187727.2	TRMT1L	mRNA	XR_001021939.1	lncRNA
XM_012187727.2	TRMT1L	mRNA	XR_001021939.1	lncRNA
XM_012187727.2	TRMT1L	mRNA	XR_001021939.1	lncRNA
XM_012106710.2	CLCN5	mRNA	TCONS_00078442	lncRNA
XM_012106710.2	CLCN5	mRNA	TCONS_00078442	lncRNA
XM_012106710.2	CLCN5	mRNA	TCONS_00078442	lncRNA
XM_012106710.2	CLCN5	mRNA	TCONS_00078442	lncRNA
XM_012106710.2	CLCN5	mRNA	TCONS_00078442	lncRNA
XM_012097252.2	RHOF	mRNA	TCONS_00078442	lncRNA
XM_012097252.2	RHOF	mRNA	TCONS_00078442	lncRNA
XM_012097252.2	RHOF	mRNA	TCONS_00078442	lncRNA
XM_012097252.2	RHOF	mRNA	TCONS_00078442	lncRNA
XM_012097252.2	RHOF	mRNA	TCONS_00078442	lncRNA
XM_012095858.2	ZNF214	mRNA	XR_001021689.2	lncRNA
XM_012095858.2	ZNF214	mRNA	XR_001021689.2	lncRNA
XM_012095858.2	ZNF214	mRNA	XR_001021689.2	lncRNA
XM_012095858.2	ZNF214	mRNA	XR_001021689.2	lncRNA
XM_012095858.2	ZNF214	mRNA	XR_001021689.2	lncRNA
XM_012164777.2	NBAS	mRNA	XR_001021689.2	lncRNA
XM_012164777.2	NBAS	mRNA	XR_001021689.2	lncRNA
XM_012164777.2	NBAS	mRNA	XR_001021689.2	lncRNA
XM_012164777.2	NBAS	mRNA	XR_001021689.2	lncRNA
XM_012164777.2	NBAS	mRNA	XR_001021689.2	lncRNA
XM_012095858.2	ZNF214	mRNA	TCONS_00071561	lncRNA
XM_012095858.2	ZNF214	mRNA	TCONS_00071561	lncRNA
XM_012095858.2	ZNF214	mRNA	TCONS_00071561	lncRNA
XM_012095858.2	ZNF214	mRNA	TCONS_00071561	lncRNA
XM_012095858.2	ZNF214	mRNA	TCONS_00071561	lncRNA
XM_012180800.1	TIPIN	mRNA	XR_001021689.2	lncRNA
XM_012180800.1	TIPIN	mRNA	XR_001021689.2	lncRNA
XM_012180800.1	TIPIN	mRNA	XR_001021689.2	lncRNA
XM_012180800.1	TIPIN	mRNA	XR_001021689.2	lncRNA
XM_012180800.1	TIPIN	mRNA	XR_001021689.2	lncRNA
XM_012152455.2	LUZP1	mRNA	XR_001039281.1	lncRNA
XM_012152455.2	LUZP1	mRNA	XR_001039281.1	lncRNA
XM_012152455.2	LUZP1	mRNA	XR_001039281.1	lncRNA
XM_012152455.2	LUZP1	mRNA	XR_001039281.1	lncRNA
XM_012152455.2	LUZP1	mRNA	XR_001039281.1	lncRNA
XM_015096057.1	KDM3B	mRNA	XR_001039281.1	lncRNA
XM_015096057.1	KDM3B	mRNA	XR_001039281.1	lncRNA
XM_015096057.1	KDM3B	mRNA	XR_001039281.1	lncRNA
XM_015096057.1	KDM3B	mRNA	XR_001039281.1	lncRNA
XM_015096057.1	KDM3B	mRNA	XR_001039281.1	lncRNA
XM_012173782.2	REV1	mRNA	TCONS_00072289	lncRNA
XM_012173782.2	REV1	mRNA	TCONS_00072289	lncRNA
XM_012173782.2	REV1	mRNA	TCONS_00072289	lncRNA
XM_012173782.2	REV1	mRNA	TCONS_00072289	lncRNA
XM_012173782.2	REV1	mRNA	TCONS_00072289	lncRNA
XM_012182854.2	PTPRK	mRNA	XR_001039281.1	lncRNA
XM_012182854.2	PTPRK	mRNA	XR_001039281.1	lncRNA
XM_012182854.2	PTPRK	mRNA	XR_001039281.1	lncRNA
XM_012182854.2	PTPRK	mRNA	XR_001039281.1	lncRNA
XM_012182854.2	PTPRK	mRNA	XR_001039281.1	lncRNA

XM_012188301.2	NEBL	mRNA	XR_001033284.2	lncRNA
XM_012188301.2	NEBL	mRNA	XR_001033284.2	lncRNA
XM_012188301.2	NEBL	mRNA	XR_001033284.2	lncRNA
XM_012188301.2	NEBL	mRNA	XR_001033284.2	lncRNA
XM_012188301.2	NEBL	mRNA	XR_001033284.2	lncRNA
XM_012106040.2	LOC1011208[illegible]	mRNA	XR_001033284.2	lncRNA
XM_012106040.2	LOC1011208[illegible]	mRNA	XR_001033284.2	lncRNA
XM_012106040.2	LOC1011208[illegible]	mRNA	XR_001033284.2	lncRNA
XM_012106040.2	LOC1011208[illegible]	mRNA	XR_001033284.2	lncRNA
XM_012106040.2	LOC1011208[illegible]	mRNA	XR_001033284.2	lncRNA
XM_012184485.2	RNASEH2B	mRNA	XR_001033284.2	lncRNA
XM_012184485.2	RNASEH2B	mRNA	XR_001033284.2	lncRNA
XM_012184485.2	RNASEH2B	mRNA	XR_001033284.2	lncRNA
XM_012184485.2	RNASEH2B	mRNA	XR_001033284.2	lncRNA
XM_012184485.2	RNASEH2B	mRNA	XR_001033284.2	lncRNA
XM_012104225.2	DCUN1D3	mRNA	XR_001436099.1	lncRNA
XM_012104225.2	DCUN1D3	mRNA	XR_001436099.1	lncRNA
XM_012104225.2	DCUN1D3	mRNA	XR_001436099.1	lncRNA
XM_012104225.2	DCUN1D3	mRNA	XR_001436099.1	lncRNA
XM_012104225.2	DCUN1D3	mRNA	XR_001436099.1	lncRNA
XM_012179421.1	METTL14	mRNA	XR_001033284.2	lncRNA
XM_012179421.1	METTL14	mRNA	XR_001033284.2	lncRNA
XM_012179421.1	METTL14	mRNA	XR_001033284.2	lncRNA
XM_012179421.1	METTL14	mRNA	XR_001033284.2	lncRNA
XM_012179421.1	METTL14	mRNA	XR_001033284.2	lncRNA
XM_012102560.2	CEP70	mRNA	XR_001436099.1	lncRNA
XM_012102560.2	CEP70	mRNA	XR_001436099.1	lncRNA
XM_012102560.2	CEP70	mRNA	XR_001436099.1	lncRNA
XM_012102560.2	CEP70	mRNA	XR_001436099.1	lncRNA
XM_012102560.2	CEP70	mRNA	XR_001436099.1	lncRNA
XM_012189374.1	NXPE3	mRNA	XR_001033284.2	lncRNA
XM_012189374.1	NXPE3	mRNA	XR_001033284.2	lncRNA
XM_012189374.1	NXPE3	mRNA	XR_001033284.2	lncRNA
XM_012189374.1	NXPE3	mRNA	XR_001033284.2	lncRNA
XM_012189374.1	NXPE3	mRNA	XR_001033284.2	lncRNA
XM_012188226.2	CUL2	mRNA	XR_001033284.2	lncRNA
XM_012188226.2	CUL2	mRNA	XR_001033284.2	lncRNA
XM_012188226.2	CUL2	mRNA	XR_001033284.2	lncRNA
XM_012188226.2	CUL2	mRNA	XR_001033284.2	lncRNA
XM_012188226.2	CUL2	mRNA	XR_001033284.2	lncRNA
XM_015097359.1	ARMC2	mRNA	XR_001033284.2	lncRNA
XM_015097359.1	ARMC2	mRNA	XR_001033284.2	lncRNA
XM_015097359.1	ARMC2	mRNA	XR_001033284.2	lncRNA
XM_015097359.1	ARMC2	mRNA	XR_001033284.2	lncRNA
XM_015097359.1	ARMC2	mRNA	XR_001033284.2	lncRNA
XM_004004099.3	GOLM1	mRNA	XR_001436609.1	lncRNA
XM_004004099.3	GOLM1	mRNA	XR_001436609.1	lncRNA
XM_004004099.3	GOLM1	mRNA	XR_001436609.1	lncRNA
XM_004004099.3	GOLM1	mRNA	XR_001436609.1	lncRNA
XM_004004099.3	GOLM1	mRNA	XR_001436609.1	lncRNA
XM_004012037.3	TDRD3	mRNA	XR_001436531.1	lncRNA
XM_004012037.3	TDRD3	mRNA	XR_001436531.1	lncRNA
XM_004012037.3	TDRD3	mRNA	XR_001436531.1	lncRNA
XM_004012037.3	TDRD3	mRNA	XR_001436531.1	lncRNA

XM_004012037.3	TDRD3	mRNA	XR_001436531.1	lncRNA
XM_012180229.1	CCNG2	mRNA	XR_001436609.1	lncRNA
XM_012180229.1	CCNG2	mRNA	XR_001436609.1	lncRNA
XM_012180229.1	CCNG2	mRNA	XR_001436609.1	lncRNA
XM_012180229.1	CCNG2	mRNA	XR_001436609.1	lncRNA
XM_012180229.1	CCNG2	mRNA	XR_001436609.1	lncRNA
XM_004002227.3	SLC35A3	mRNA	XR_001435104.1	lncRNA
XM_004002227.3	SLC35A3	mRNA	XR_001435104.1	lncRNA
XM_004002227.3	SLC35A3	mRNA	XR_001435104.1	lncRNA
XM_004002227.3	SLC35A3	mRNA	XR_001435104.1	lncRNA
XM_004002227.3	SLC35A3	mRNA	XR_001435104.1	lncRNA
XM_004004723.3	SPOPL	mRNA	XR_001436609.1	lncRNA
XM_004004723.3	SPOPL	mRNA	XR_001436609.1	lncRNA
XM_004004723.3	SPOPL	mRNA	XR_001436609.1	lncRNA
XM_004004723.3	SPOPL	mRNA	XR_001436609.1	lncRNA
XM_004004723.3	SPOPL	mRNA	XR_001436609.1	lncRNA
XM_012096215.2	EHF	mRNA	XR_001435104.1	lncRNA
XM_012096215.2	EHF	mRNA	XR_001435104.1	lncRNA
XM_012096215.2	EHF	mRNA	XR_001435104.1	lncRNA
XM_012096215.2	EHF	mRNA	XR_001435104.1	lncRNA
XM_012096215.2	EHF	mRNA	XR_001435104.1	lncRNA
XM_012104035.2	CLUAP1	mRNA	XR_001435104.1	lncRNA
XM_012104035.2	CLUAP1	mRNA	XR_001435104.1	lncRNA
XM_012104035.2	CLUAP1	mRNA	XR_001435104.1	lncRNA
XM_012104035.2	CLUAP1	mRNA	XR_001435104.1	lncRNA
XM_012104035.2	CLUAP1	mRNA	XR_001435104.1	lncRNA
XM_015103641.1	YES1	mRNA	XR_001436609.1	lncRNA
XM_015103641.1	YES1	mRNA	XR_001436609.1	lncRNA
XM_015103641.1	YES1	mRNA	XR_001436609.1	lncRNA
XM_015103641.1	YES1	mRNA	XR_001436609.1	lncRNA
XM_015103641.1	YES1	mRNA	XR_001436609.1	lncRNA
XM_004012037.3	TDRD3	mRNA	XR_001436609.1	lncRNA
XM_004012037.3	TDRD3	mRNA	XR_001436609.1	lncRNA
XM_004012037.3	TDRD3	mRNA	XR_001436609.1	lncRNA
XM_004012037.3	TDRD3	mRNA	XR_001436609.1	lncRNA
XM_004012037.3	TDRD3	mRNA	XR_001436609.1	lncRNA
XM_015096569.1	CDKL2	mRNA	XR_001436609.1	lncRNA
XM_015096569.1	CDKL2	mRNA	XR_001436609.1	lncRNA
XM_015096569.1	CDKL2	mRNA	XR_001436609.1	lncRNA
XM_015096569.1	CDKL2	mRNA	XR_001436609.1	lncRNA
XM_015096569.1	CDKL2	mRNA	XR_001436609.1	lncRNA
XM_012187727.2	TRMT1L	mRNA	XR_001436609.1	lncRNA
XM_012187727.2	TRMT1L	mRNA	XR_001436609.1	lncRNA
XM_012187727.2	TRMT1L	mRNA	XR_001436609.1	lncRNA
XM_012187727.2	TRMT1L	mRNA	XR_001436609.1	lncRNA
XM_012187727.2	TRMT1L	mRNA	XR_001436609.1	lncRNA
XM_015093291.1	NIPA2	mRNA	XR_001436609.1	lncRNA
XM_015093291.1	NIPA2	mRNA	XR_001436609.1	lncRNA
XM_015093291.1	NIPA2	mRNA	XR_001436609.1	lncRNA
XM_015093291.1	NIPA2	mRNA	XR_001436609.1	lncRNA
XM_015093291.1	NIPA2	mRNA	XR_001436609.1	lncRNA
XM_012134451.2	PPIG	mRNA	XR_001436531.1	lncRNA
XM_012134451.2	PPIG	mRNA	XR_001436531.1	lncRNA
XM_012134451.2	PPIG	mRNA	XR_001436531.1	lncRNA

XM_012134451.2	PPIG	mRNA	XR_001436531.1	lncRNA
XM_012134451.2	PPIG	mRNA	XR_001436531.1	lncRNA
XM_015103643.1	YES1	mRNA	XR_001436609.1	lncRNA
XM_015103643.1	YES1	mRNA	XR_001436609.1	lncRNA
XM_015103643.1	YES1	mRNA	XR_001436609.1	lncRNA
XM_015103643.1	YES1	mRNA	XR_001436609.1	lncRNA
XM_015103643.1	YES1	mRNA	XR_001436609.1	lncRNA
XM_012175343.2	LRP6	mRNA	XR_001436531.1	lncRNA
XM_012175343.2	LRP6	mRNA	XR_001436531.1	lncRNA
XM_012175343.2	LRP6	mRNA	XR_001436531.1	lncRNA
XM_012175343.2	LRP6	mRNA	XR_001436531.1	lncRNA
XM_012175343.2	LRP6	mRNA	XR_001436531.1	lncRNA
XM_012134516.1	PPIG	mRNA	XR_001436531.1	lncRNA
XM_012134516.1	PPIG	mRNA	XR_001436531.1	lncRNA
XM_012134516.1	PPIG	mRNA	XR_001436531.1	lncRNA
XM_012134516.1	PPIG	mRNA	XR_001436531.1	lncRNA
XM_012134516.1	PPIG	mRNA	XR_001436531.1	lncRNA
XM_015097545.1	PHF10	mRNA	XR_001436531.1	lncRNA
XM_015097545.1	PHF10	mRNA	XR_001436531.1	lncRNA
XM_015097545.1	PHF10	mRNA	XR_001436531.1	lncRNA
XM_015097545.1	PHF10	mRNA	XR_001436531.1	lncRNA
XM_015097545.1	PHF10	mRNA	XR_001436531.1	lncRNA
XM_012166941.1	BRE	mRNA	XR_001435104.1	lncRNA
XM_012166941.1	BRE	mRNA	XR_001435104.1	lncRNA
XM_012166941.1	BRE	mRNA	XR_001435104.1	lncRNA
XM_012166941.1	BRE	mRNA	XR_001435104.1	lncRNA
XM_012166941.1	BRE	mRNA	XR_001435104.1	lncRNA
XM_015093291.1	NIPA2	mRNA	XR_001436531.1	lncRNA
XM_015093291.1	NIPA2	mRNA	XR_001436531.1	lncRNA
XM_015093291.1	NIPA2	mRNA	XR_001436531.1	lncRNA
XM_015093291.1	NIPA2	mRNA	XR_001436531.1	lncRNA
XM_015093291.1	NIPA2	mRNA	XR_001436531.1	lncRNA
XM_012187727.2	TRMT1L	mRNA	XR_001435104.1	lncRNA
XM_012187727.2	TRMT1L	mRNA	XR_001435104.1	lncRNA
XM_012187727.2	TRMT1L	mRNA	XR_001435104.1	lncRNA
XM_012187727.2	TRMT1L	mRNA	XR_001435104.1	lncRNA
XM_012187727.2	TRMT1L	mRNA	XR_001435104.1	lncRNA
XM_012180229.1	CCNG2	mRNA	XR_001435104.1	lncRNA
XM_012180229.1	CCNG2	mRNA	XR_001435104.1	lncRNA
XM_012180229.1	CCNG2	mRNA	XR_001435104.1	lncRNA
XM_012180229.1	CCNG2	mRNA	XR_001435104.1	lncRNA
XM_012180229.1	CCNG2	mRNA	XR_001435104.1	lncRNA
XM_012096215.2	EHF	mRNA	XR_001436609.1	lncRNA
XM_012096215.2	EHF	mRNA	XR_001436609.1	lncRNA
XM_012096215.2	EHF	mRNA	XR_001436609.1	lncRNA
XM_012096215.2	EHF	mRNA	XR_001436609.1	lncRNA
XM_012096215.2	EHF	mRNA	XR_001436609.1	lncRNA
XM_012129647.1	NEK1	mRNA	TCONS_00029398	lncRNA
XM_012129647.1	NEK1	mRNA	TCONS_00029398	lncRNA
XM_012129647.1	NEK1	mRNA	TCONS_00029398	lncRNA
XM_012129647.1	NEK1	mRNA	TCONS_00029398	lncRNA
XM_012129647.1	NEK1	mRNA	TCONS_00029398	lncRNA
XM_015093291.1	NIPA2	mRNA	XR_001435726.1	lncRNA
XM_015093291.1	NIPA2	mRNA	XR_001435726.1	lncRNA

XM_015093291.1	NIPA2	mRNA	XR_001435726.1	lncRNA
XM_015093291.1	NIPA2	mRNA	XR_001435726.1	lncRNA
XM_015093291.1	NIPA2	mRNA	XR_001435726.1	lncRNA
XM_012187240.2	SLC19A2	mRNA	XR_001042381.2	lncRNA
XM_012187240.2	SLC19A2	mRNA	XR_001042381.2	lncRNA
XM_012187240.2	SLC19A2	mRNA	XR_001042381.2	lncRNA
XM_012187240.2	SLC19A2	mRNA	XR_001042381.2	lncRNA
XM_012187240.2	SLC19A2	mRNA	XR_001042381.2	lncRNA
XM_012180229.1	CCNG2	mRNA	XR_001042381.2	lncRNA
XM_012180229.1	CCNG2	mRNA	XR_001042381.2	lncRNA
XM_012180229.1	CCNG2	mRNA	XR_001042381.2	lncRNA
XM_012180229.1	CCNG2	mRNA	XR_001042381.2	lncRNA
XM_012180229.1	CCNG2	mRNA	XR_001042381.2	lncRNA
XM_015101857.1	LINS1	mRNA	XR_001042381.2	lncRNA
XM_015101857.1	LINS1	mRNA	XR_001042381.2	lncRNA
XM_015101857.1	LINS1	mRNA	XR_001042381.2	lncRNA
XM_015101857.1	LINS1	mRNA	XR_001042381.2	lncRNA
XM_015101857.1	LINS1	mRNA	XR_001042381.2	lncRNA
XM_012095725.2	BTBD10	mRNA	XR_001042381.2	lncRNA
XM_012095725.2	BTBD10	mRNA	XR_001042381.2	lncRNA
XM_012095725.2	BTBD10	mRNA	XR_001042381.2	lncRNA
XM_012095725.2	BTBD10	mRNA	XR_001042381.2	lncRNA
XM_012095725.2	BTBD10	mRNA	XR_001042381.2	lncRNA
XM_004004723.3	SPOPL	mRNA	XR_001435726.1	lncRNA
XM_004004723.3	SPOPL	mRNA	XR_001435726.1	lncRNA
XM_004004723.3	SPOPL	mRNA	XR_001435726.1	lncRNA
XM_004004723.3	SPOPL	mRNA	XR_001435726.1	lncRNA
XM_004004723.3	SPOPL	mRNA	XR_001435726.1	lncRNA
XM_012181771.2	PCNXL4	mRNA	XR_001042381.2	lncRNA
XM_012181771.2	PCNXL4	mRNA	XR_001042381.2	lncRNA
XM_012181771.2	PCNXL4	mRNA	XR_001042381.2	lncRNA
XM_012181771.2	PCNXL4	mRNA	XR_001042381.2	lncRNA
XM_012181771.2	PCNXL4	mRNA	XR_001042381.2	lncRNA
XM_012100861.1	FBXO9	mRNA	XR_001042381.2	lncRNA
XM_012100861.1	FBXO9	mRNA	XR_001042381.2	lncRNA
XM_012100861.1	FBXO9	mRNA	XR_001042381.2	lncRNA
XM_012100861.1	FBXO9	mRNA	XR_001042381.2	lncRNA
XM_012100861.1	FBXO9	mRNA	XR_001042381.2	lncRNA
XM_004012898.3	KRT34	mRNA	XR_001042381.2	lncRNA
XM_004012898.3	KRT34	mRNA	XR_001042381.2	lncRNA
XM_004012898.3	KRT34	mRNA	XR_001042381.2	lncRNA
XM_004012898.3	KRT34	mRNA	XR_001042381.2	lncRNA
XM_004012898.3	KRT34	mRNA	XR_001042381.2	lncRNA
XM_012178872.2	AP4B1	mRNA	XR_001435723.1	lncRNA
XM_012178872.2	AP4B1	mRNA	XR_001435723.1	lncRNA
XM_012178872.2	AP4B1	mRNA	XR_001435723.1	lncRNA
XM_012178872.2	AP4B1	mRNA	XR_001435723.1	lncRNA
XM_012178872.2	AP4B1	mRNA	XR_001435723.1	lncRNA
XM_004012898.3	KRT34	mRNA	XR_001435726.1	lncRNA
XM_004012898.3	KRT34	mRNA	XR_001435726.1	lncRNA
XM_004012898.3	KRT34	mRNA	XR_001435726.1	lncRNA
XM_004012898.3	KRT34	mRNA	XR_001435726.1	lncRNA
XM_004012898.3	KRT34	mRNA	XR_001435726.1	lncRNA
XM_012101717.1	PAK1	mRNA	XR_001042381.2	lncRNA

XM_012101717.1	PAK1	mRNA	XR_001042381.2	lncRNA
XM_012101717.1	PAK1	mRNA	XR_001042381.2	lncRNA
XM_012101717.1	PAK1	mRNA	XR_001042381.2	lncRNA
XM_012101717.1	PAK1	mRNA	XR_001042381.2	lncRNA
XM_015098611.1	DLG4	mRNA	XR_001435726.1	lncRNA
XM_015098611.1	DLG4	mRNA	XR_001435726.1	lncRNA
XM_015098611.1	DLG4	mRNA	XR_001435726.1	lncRNA
XM_015098611.1	DLG4	mRNA	XR_001435726.1	lncRNA
XM_015098611.1	DLG4	mRNA	XR_001435726.1	lncRNA
XM_012137599.2	NEB	mRNA	XR_001039281.1	lncRNA
XM_012137599.2	NEB	mRNA	XR_001039281.1	lncRNA
XM_012137599.2	NEB	mRNA	XR_001039281.1	lncRNA
XM_012137599.2	NEB	mRNA	XR_001039281.1	lncRNA
XM_012137599.2	NEB	mRNA	XR_001039281.1	lncRNA
XM_012095465.2	GOLGB1	mRNA	XR_001039281.1	lncRNA
XM_012095465.2	GOLGB1	mRNA	XR_001039281.1	lncRNA
XM_012095465.2	GOLGB1	mRNA	XR_001039281.1	lncRNA
XM_012095465.2	GOLGB1	mRNA	XR_001039281.1	lncRNA
XM_012095465.2	GOLGB1	mRNA	XR_001039281.1	lncRNA
XM_012189777.1	CHD9	mRNA	TCONS_00072289	lncRNA
XM_012189777.1	CHD9	mRNA	TCONS_00072289	lncRNA
XM_012189777.1	CHD9	mRNA	TCONS_00072289	lncRNA
XM_012189777.1	CHD9	mRNA	TCONS_00072289	lncRNA
XM_012189777.1	CHD9	mRNA	TCONS_00072289	lncRNA
XM_012095461.2	GOLGB1	mRNA	TCONS_00072289	lncRNA
XM_012095461.2	GOLGB1	mRNA	TCONS_00072289	lncRNA
XM_012095461.2	GOLGB1	mRNA	TCONS_00072289	lncRNA
XM_012095461.2	GOLGB1	mRNA	TCONS_00072289	lncRNA
XM_012095461.2	GOLGB1	mRNA	TCONS_00072289	lncRNA
XM_015092197.1	CD58	mRNA	XR_001041209.2	lncRNA
XM_015092197.1	CD58	mRNA	XR_001041209.2	lncRNA
XM_015092197.1	CD58	mRNA	XR_001041209.2	lncRNA
XM_015092197.1	CD58	mRNA	XR_001041209.2	lncRNA
XM_015092197.1	CD58	mRNA	XR_001041209.2	lncRNA
XM_015093101.1	PPP3CC	mRNA	XR_001041209.2	lncRNA
XM_015093101.1	PPP3CC	mRNA	XR_001041209.2	lncRNA
XM_015093101.1	PPP3CC	mRNA	XR_001041209.2	lncRNA
XM_015093101.1	PPP3CC	mRNA	XR_001041209.2	lncRNA
XM_015093101.1	PPP3CC	mRNA	XR_001041209.2	lncRNA
XM_012104222.2	DCUN1D3	mRNA	TCONS_00029399	lncRNA
XM_012104222.2	DCUN1D3	mRNA	TCONS_00029399	lncRNA
XM_012104222.2	DCUN1D3	mRNA	TCONS_00029399	lncRNA
XM_012104222.2	DCUN1D3	mRNA	TCONS_00029399	lncRNA
XM_012104222.2	DCUN1D3	mRNA	TCONS_00029399	lncRNA
XM_015094750.1	RAB3IP	mRNA	XR_001041211.2	lncRNA
XM_015094750.1	RAB3IP	mRNA	XR_001041211.2	lncRNA
XM_015094750.1	RAB3IP	mRNA	XR_001041211.2	lncRNA
XM_015094750.1	RAB3IP	mRNA	XR_001041211.2	lncRNA
XM_015094750.1	RAB3IP	mRNA	XR_001041211.2	lncRNA
XM_012190083.2	ZMYM6	mRNA	XR_001041209.2	lncRNA
XM_012190083.2	ZMYM6	mRNA	XR_001041209.2	lncRNA
XM_012190083.2	ZMYM6	mRNA	XR_001041209.2	lncRNA
XM_012190083.2	ZMYM6	mRNA	XR_001041209.2	lncRNA
XM_012190083.2	ZMYM6	mRNA	XR_001041209.2	lncRNA

XM_012187630.2	SOAT1	mRNA	TCONS_00029399	lncRNA
XM_012187630.2	SOAT1	mRNA	TCONS_00029399	lncRNA
XM_012187630.2	SOAT1	mRNA	TCONS_00029399	lncRNA
XM_012187630.2	SOAT1	mRNA	TCONS_00029399	lncRNA
XM_012187630.2	SOAT1	mRNA	TCONS_00029399	lncRNA
XM_015102735.1	SUPT3H	mRNA	XR_001041209.2	lncRNA
XM_015102735.1	SUPT3H	mRNA	XR_001041209.2	lncRNA
XM_015102735.1	SUPT3H	mRNA	XR_001041209.2	lncRNA
XM_015102735.1	SUPT3H	mRNA	XR_001041209.2	lncRNA
XM_015102735.1	SUPT3H	mRNA	XR_001041209.2	lncRNA
XM_012184485.2	RNASEH2B	mRNA	XR_001041209.2	lncRNA
XM_012184485.2	RNASEH2B	mRNA	XR_001041209.2	lncRNA
XM_012184485.2	RNASEH2B	mRNA	XR_001041209.2	lncRNA
XM_012184485.2	RNASEH2B	mRNA	XR_001041209.2	lncRNA
XM_012184485.2	RNASEH2B	mRNA	XR_001041209.2	lncRNA
XM_012181536.2	FAM214A	mRNA	TCONS_00029399	lncRNA
XM_012181536.2	FAM214A	mRNA	TCONS_00029399	lncRNA
XM_012181536.2	FAM214A	mRNA	TCONS_00029399	lncRNA
XM_012181536.2	FAM214A	mRNA	TCONS_00029399	lncRNA
XM_012181536.2	FAM214A	mRNA	TCONS_00029399	lncRNA
XM_012189374.1	NXPE3	mRNA	XR_001041209.2	lncRNA
XM_012189374.1	NXPE3	mRNA	XR_001041209.2	lncRNA
XM_012189374.1	NXPE3	mRNA	XR_001041209.2	lncRNA
XM_012189374.1	NXPE3	mRNA	XR_001041209.2	lncRNA
XM_012189374.1	NXPE3	mRNA	XR_001041209.2	lncRNA
XM_012094768.2	ZMYM1	mRNA	XR_001041209.2	lncRNA
XM_012094768.2	ZMYM1	mRNA	XR_001041209.2	lncRNA
XM_012094768.2	ZMYM1	mRNA	XR_001041209.2	lncRNA
XM_012094768.2	ZMYM1	mRNA	XR_001041209.2	lncRNA
XM_012094768.2	ZMYM1	mRNA	XR_001041209.2	lncRNA
XM_012106040.2	LOC1011208	mRNA	XR_001041209.2	lncRNA
XM_012106040.2	LOC1011208	mRNA	XR_001041209.2	lncRNA
XM_012106040.2	LOC1011208	mRNA	XR_001041209.2	lncRNA
XM_012106040.2	LOC1011208	mRNA	XR_001041209.2	lncRNA
XM_012106040.2	LOC1011208	mRNA	XR_001041209.2	lncRNA
XM_012102549.2	CEP70	mRNA	XR_001041209.2	lncRNA
XM_012102549.2	CEP70	mRNA	XR_001041209.2	lncRNA
XM_012102549.2	CEP70	mRNA	XR_001041209.2	lncRNA
XM_012102549.2	CEP70	mRNA	XR_001041209.2	lncRNA
XM_012102549.2	CEP70	mRNA	XR_001041209.2	lncRNA
XM_012104222.2	DCUN1D3	mRNA	XR_001041209.2	lncRNA
XM_012104222.2	DCUN1D3	mRNA	XR_001041209.2	lncRNA
XM_012104222.2	DCUN1D3	mRNA	XR_001041209.2	lncRNA
XM_012104222.2	DCUN1D3	mRNA	XR_001041209.2	lncRNA
XM_012104222.2	DCUN1D3	mRNA	XR_001041209.2	lncRNA
XM_012188664.2	RASSF2	mRNA	TCONS_00029399	lncRNA
XM_012188664.2	RASSF2	mRNA	TCONS_00029399	lncRNA
XM_012188664.2	RASSF2	mRNA	TCONS_00029399	lncRNA
XM_012188664.2	RASSF2	mRNA	TCONS_00029399	lncRNA
XM_012188664.2	RASSF2	mRNA	TCONS_00029399	lncRNA
XM_012094768.2	ZMYM1	mRNA	TCONS_00029399	lncRNA
XM_012094768.2	ZMYM1	mRNA	TCONS_00029399	lncRNA
XM_012094768.2	ZMYM1	mRNA	TCONS_00029399	lncRNA
XM_012094768.2	ZMYM1	mRNA	TCONS_00029399	lncRNA

XM_012094768.2	ZMYM1	mRNA	TCONS_00029399	lncRNA
XM_012104225.2	DCUN1D3	mRNA	XR_001041209.2	lncRNA
XM_012104225.2	DCUN1D3	mRNA	XR_001041209.2	lncRNA
XM_012104225.2	DCUN1D3	mRNA	XR_001041209.2	lncRNA
XM_012104225.2	DCUN1D3	mRNA	XR_001041209.2	lncRNA
XM_012104225.2	DCUN1D3	mRNA	XR_001041209.2	lncRNA
XM_015092786.1	LOC1069908[illegible]	mRNA	XR_001041211.2	lncRNA
XM_015092786.1	LOC1069908[illegible]	mRNA	XR_001041211.2	lncRNA
XM_015092786.1	LOC1069908[illegible]	mRNA	XR_001041211.2	lncRNA
XM_015092786.1	LOC1069908[illegible]	mRNA	XR_001041211.2	lncRNA
XM_015092786.1	LOC1069908[illegible]	mRNA	XR_001041211.2	lncRNA
XM_015104027.1	PDPK1	mRNA	TCONS_00029399	lncRNA
XM_015104027.1	PDPK1	mRNA	TCONS_00029399	lncRNA
XM_015104027.1	PDPK1	mRNA	TCONS_00029399	lncRNA
XM_015104027.1	PDPK1	mRNA	TCONS_00029399	lncRNA
XM_015104027.1	PDPK1	mRNA	TCONS_00029399	lncRNA
XM_012124912.1	KIAA2026	mRNA	TCONS_00029399	lncRNA
XM_012124912.1	KIAA2026	mRNA	TCONS_00029399	lncRNA
XM_012124912.1	KIAA2026	mRNA	TCONS_00029399	lncRNA
XM_012124912.1	KIAA2026	mRNA	TCONS_00029399	lncRNA
XM_012124912.1	KIAA2026	mRNA	TCONS_00029399	lncRNA
XM_015092197.1	CD58	mRNA	XR_001041211.2	lncRNA
XM_015092197.1	CD58	mRNA	XR_001041211.2	lncRNA
XM_015092197.1	CD58	mRNA	XR_001041211.2	lncRNA
XM_015092197.1	CD58	mRNA	XR_001041211.2	lncRNA
XM_015092197.1	CD58	mRNA	XR_001041211.2	lncRNA
XM_015102735.1	SUPT3H	mRNA	TCONS_00029399	lncRNA
XM_015102735.1	SUPT3H	mRNA	TCONS_00029399	lncRNA
XM_015102735.1	SUPT3H	mRNA	TCONS_00029399	lncRNA
XM_015102735.1	SUPT3H	mRNA	TCONS_00029399	lncRNA
XM_015102735.1	SUPT3H	mRNA	TCONS_00029399	lncRNA
XM_012102549.2	CEP70	mRNA	TCONS_00029399	lncRNA
XM_012102549.2	CEP70	mRNA	TCONS_00029399	lncRNA
XM_012102549.2	CEP70	mRNA	TCONS_00029399	lncRNA
XM_012102549.2	CEP70	mRNA	TCONS_00029399	lncRNA
XM_012102549.2	CEP70	mRNA	TCONS_00029399	lncRNA
XM_012106040.2	LOC1011208[illegible]	mRNA	TCONS_00029399	lncRNA
XM_012106040.2	LOC1011208[illegible]	mRNA	TCONS_00029399	lncRNA
XM_012106040.2	LOC1011208[illegible]	mRNA	TCONS_00029399	lncRNA
XM_012106040.2	LOC1011208[illegible]	mRNA	TCONS_00029399	lncRNA
XM_012106040.2	LOC1011208[illegible]	mRNA	TCONS_00029399	lncRNA
XM_015093098.1	PPP3CC	mRNA	XR_001041211.2	lncRNA
XM_015093098.1	PPP3CC	mRNA	XR_001041211.2	lncRNA
XM_015093098.1	PPP3CC	mRNA	XR_001041211.2	lncRNA
XM_015093098.1	PPP3CC	mRNA	XR_001041211.2	lncRNA
XM_015093098.1	PPP3CC	mRNA	XR_001041211.2	lncRNA
XM_012105198.2	TET1	mRNA	TCONS_00029398	lncRNA
XM_012105198.2	TET1	mRNA	TCONS_00029398	lncRNA
XM_012105198.2	TET1	mRNA	TCONS_00029398	lncRNA
XM_012105198.2	TET1	mRNA	TCONS_00029398	lncRNA
XM_012105198.2	TET1	mRNA	TCONS_00029398	lncRNA
XM_012187729.2	SWT1	mRNA	XR_001021689.2	lncRNA
XM_012187729.2	SWT1	mRNA	XR_001021689.2	lncRNA
XM_012187729.2	SWT1	mRNA	XR_001021689.2	lncRNA

XM_012187729.2	SWT1	mRNA	XR_001021689.2	lncRNA
XM_012187729.2	SWT1	mRNA	XR_001021689.2	lncRNA
XM_012186944.1	DSTYK	mRNA	XR_001021689.2	lncRNA
XM_012186944.1	DSTYK	mRNA	XR_001021689.2	lncRNA
XM_012186944.1	DSTYK	mRNA	XR_001021689.2	lncRNA
XM_012186944.1	DSTYK	mRNA	XR_001021689.2	lncRNA
XM_012186944.1	DSTYK	mRNA	XR_001021689.2	lncRNA
XM_015094782.1	MARS	mRNA	TCONS_00071561	lncRNA
XM_015094782.1	MARS	mRNA	TCONS_00071561	lncRNA
XM_015094782.1	MARS	mRNA	TCONS_00071561	lncRNA
XM_015094782.1	MARS	mRNA	TCONS_00071561	lncRNA
XM_015094782.1	MARS	mRNA	TCONS_00071561	lncRNA
XM_004012689.3	MYH10	mRNA	XR_001021689.2	lncRNA
XM_004012689.3	MYH10	mRNA	XR_001021689.2	lncRNA
XM_004012689.3	MYH10	mRNA	XR_001021689.2	lncRNA
XM_004012689.3	MYH10	mRNA	XR_001021689.2	lncRNA
XM_004012689.3	MYH10	mRNA	XR_001021689.2	lncRNA
XM_012146843.1	EPHA4	mRNA	TCONS_00071561	lncRNA
XM_012146843.1	EPHA4	mRNA	TCONS_00071561	lncRNA
XM_012146843.1	EPHA4	mRNA	TCONS_00071561	lncRNA
XM_012146843.1	EPHA4	mRNA	TCONS_00071561	lncRNA
XM_012146843.1	EPHA4	mRNA	TCONS_00071561	lncRNA
XM_004010549.3	VPS13C	mRNA	XR_001039281.1	lncRNA
XM_004010549.3	VPS13C	mRNA	XR_001039281.1	lncRNA
XM_004010549.3	VPS13C	mRNA	XR_001039281.1	lncRNA
XM_004010549.3	VPS13C	mRNA	XR_001039281.1	lncRNA
XM_004010549.3	VPS13C	mRNA	XR_001039281.1	lncRNA
XM_012182190.2	EML5	mRNA	TCONS_00072289	lncRNA
XM_012182190.2	EML5	mRNA	TCONS_00072289	lncRNA
XM_012182190.2	EML5	mRNA	TCONS_00072289	lncRNA
XM_012182190.2	EML5	mRNA	TCONS_00072289	lncRNA
XM_012182190.2	EML5	mRNA	TCONS_00072289	lncRNA
XM_012190192.2	PHLDB2	mRNA	XR_001039281.1	lncRNA
XM_012190192.2	PHLDB2	mRNA	XR_001039281.1	lncRNA
XM_012190192.2	PHLDB2	mRNA	XR_001039281.1	lncRNA
XM_012190192.2	PHLDB2	mRNA	XR_001039281.1	lncRNA
XM_012190192.2	PHLDB2	mRNA	XR_001039281.1	lncRNA
XM_012169683.2	LOC1011072(	mRNA	XR_001434081.1	lncRNA
XM_012169683.2	LOC1011072(	mRNA	XR_001434081.1	lncRNA
XM_012169683.2	LOC1011072(	mRNA	XR_001434081.1	lncRNA
XM_012169683.2	LOC1011072(	mRNA	XR_001434081.1	lncRNA
XM_012169683.2	LOC1011072(	mRNA	XR_001434081.1	lncRNA
XM_012124912.1	KIAA2026	mRNA	XR_001434081.1	lncRNA
XM_012124912.1	KIAA2026	mRNA	XR_001434081.1	lncRNA
XM_012124912.1	KIAA2026	mRNA	XR_001434081.1	lncRNA
XM_012124912.1	KIAA2026	mRNA	XR_001434081.1	lncRNA
XM_012124912.1	KIAA2026	mRNA	XR_001434081.1	lncRNA
XM_015093098.1	PPP3CC	mRNA	XR_001434081.1	lncRNA
XM_015093098.1	PPP3CC	mRNA	XR_001434081.1	lncRNA
XM_015093098.1	PPP3CC	mRNA	XR_001434081.1	lncRNA
XM_015093098.1	PPP3CC	mRNA	XR_001434081.1	lncRNA
XM_015093098.1	PPP3CC	mRNA	XR_001434081.1	lncRNA
XM_012104225.2	DCUN1D3	mRNA	XR_001434081.1	lncRNA
XM_012104225.2	DCUN1D3	mRNA	XR_001434081.1	lncRNA

XM_012104225.2	DCUN1D3	mRNA	XR_001434081.1	lncRNA
XM_012104225.2	DCUN1D3	mRNA	XR_001434081.1	lncRNA
XM_012104225.2	DCUN1D3	mRNA	XR_001434081.1	lncRNA
XM_012094768.2	ZMYM1	mRNA	XR_001434081.1	lncRNA
XM_012094768.2	ZMYM1	mRNA	XR_001434081.1	lncRNA
XM_012094768.2	ZMYM1	mRNA	XR_001434081.1	lncRNA
XM_012094768.2	ZMYM1	mRNA	XR_001434081.1	lncRNA
XM_012094768.2	ZMYM1	mRNA	XR_001434081.1	lncRNA
XM_012190083.2	ZMYM6	mRNA	XR_001434081.1	lncRNA
XM_012190083.2	ZMYM6	mRNA	XR_001434081.1	lncRNA
XM_012190083.2	ZMYM6	mRNA	XR_001434081.1	lncRNA
XM_012190083.2	ZMYM6	mRNA	XR_001434081.1	lncRNA
XM_012190083.2	ZMYM6	mRNA	XR_001434081.1	lncRNA
XM_012145933.2	CXCR2	mRNA	XR_001434081.1	lncRNA
XM_012145933.2	CXCR2	mRNA	XR_001434081.1	lncRNA
XM_012145933.2	CXCR2	mRNA	XR_001434081.1	lncRNA
XM_012145933.2	CXCR2	mRNA	XR_001434081.1	lncRNA
XM_012145933.2	CXCR2	mRNA	XR_001434081.1	lncRNA
XM_015102528.1	FLOT1	mRNA	TCONS_00063226	lncRNA
XM_015102528.1	FLOT1	mRNA	TCONS_00063226	lncRNA
XM_015102528.1	FLOT1	mRNA	TCONS_00063226	lncRNA
XM_015102528.1	FLOT1	mRNA	TCONS_00063226	lncRNA
XM_015093007.1	TMEM246	mRNA	XR_001039281.1	lncRNA
XM_015093007.1	TMEM246	mRNA	XR_001039281.1	lncRNA
XM_015093007.1	TMEM246	mRNA	XR_001039281.1	lncRNA
XM_015093007.1	TMEM246	mRNA	XR_001039281.1	lncRNA
XM_012187575.2	RABGAP1L	mRNA	XR_001033284.2	lncRNA
XM_012187575.2	RABGAP1L	mRNA	XR_001033284.2	lncRNA
XM_012187575.2	RABGAP1L	mRNA	XR_001033284.2	lncRNA
XM_012187575.2	RABGAP1L	mRNA	XR_001033284.2	lncRNA
XM_012187575.2	RABGAP1L	mRNA	XR_001033284.2	lncRNA
XM_012174295.2	ATF1	mRNA	XR_001436099.1	lncRNA
XM_012174295.2	ATF1	mRNA	XR_001436099.1	lncRNA
XM_012174295.2	ATF1	mRNA	XR_001436099.1	lncRNA
XM_012174295.2	ATF1	mRNA	XR_001436099.1	lncRNA
XM_012174295.2	ATF1	mRNA	XR_001436099.1	lncRNA
XM_015097018.1	ZNF280D	mRNA	XR_001436099.1	lncRNA
XM_015097018.1	ZNF280D	mRNA	XR_001436099.1	lncRNA
XM_015097018.1	ZNF280D	mRNA	XR_001436099.1	lncRNA
XM_015097018.1	ZNF280D	mRNA	XR_001436099.1	lncRNA
XM_015097018.1	ZNF280D	mRNA	XR_001436099.1	lncRNA
XM_012113242.2	KIAA1958	mRNA	XR_001039281.1	lncRNA
XM_012113242.2	KIAA1958	mRNA	XR_001039281.1	lncRNA
XM_012113242.2	KIAA1958	mRNA	XR_001039281.1	lncRNA
XM_012113242.2	KIAA1958	mRNA	XR_001039281.1	lncRNA
XM_012113242.2	KIAA1958	mRNA	XR_001039281.1	lncRNA
XM_015097809.1	NCOA2	mRNA	TCONS_00072289	lncRNA
XM_015097809.1	NCOA2	mRNA	TCONS_00072289	lncRNA
XM_015097809.1	NCOA2	mRNA	TCONS_00072289	lncRNA
XM_015097809.1	NCOA2	mRNA	TCONS_00072289	lncRNA
XM_015097809.1	NCOA2	mRNA	TCONS_00072289	lncRNA
XM_012106040.2	LOC1011208[illegible]	mRNA	XR_001021939.1	lncRNA
XM_012106040.2	LOC1011208[illegible]	mRNA	XR_001021939.1	lncRNA
XM_012106040.2	LOC1011208[illegible]	mRNA	XR_001021939.1	lncRNA

XM_012106040.2	LOC1011208{	mRNA	XR_001021939.1	lncRNA
XM_012106040.2	LOC1011208{	mRNA	XR_001021939.1	lncRNA
XM_012188301.2	NEBL	mRNA	XR_001021939.1	lncRNA
XM_012188301.2	NEBL	mRNA	XR_001021939.1	lncRNA
XM_012188301.2	NEBL	mRNA	XR_001021939.1	lncRNA
XM_012188301.2	NEBL	mRNA	XR_001021939.1	lncRNA
XM_012188301.2	NEBL	mRNA	XR_001021939.1	lncRNA
XM_012179604.2	UBE2D3	mRNA	XR_001021939.1	lncRNA
XM_012179604.2	UBE2D3	mRNA	XR_001021939.1	lncRNA
XM_012179604.2	UBE2D3	mRNA	XR_001021939.1	lncRNA
XM_012179604.2	UBE2D3	mRNA	XR_001021939.1	lncRNA
XM_012179604.2	UBE2D3	mRNA	XR_001021939.1	lncRNA
XM_012106112.2	ARAF	mRNA	XR_001021939.1	lncRNA
XM_012106112.2	ARAF	mRNA	XR_001021939.1	lncRNA
XM_012106112.2	ARAF	mRNA	XR_001021939.1	lncRNA
XM_012106112.2	ARAF	mRNA	XR_001021939.1	lncRNA
XM_012106112.2	ARAF	mRNA	XR_001021939.1	lncRNA
XM_012176399.2	ICA1	mRNA	TCONS_00052150	lncRNA
XM_012176399.2	ICA1	mRNA	TCONS_00052150	lncRNA
XM_012176399.2	ICA1	mRNA	TCONS_00052150	lncRNA
XM_012176399.2	ICA1	mRNA	TCONS_00052150	lncRNA
XM_012189447.2	ALCAM	mRNA	XR_001041202.1	lncRNA
XM_012189447.2	ALCAM	mRNA	XR_001041202.1	lncRNA
XM_012189447.2	ALCAM	mRNA	XR_001041202.1	lncRNA
XM_012189447.2	ALCAM	mRNA	XR_001041202.1	lncRNA
XM_012189447.2	ALCAM	mRNA	XR_001041202.1	lncRNA
XM_012182238.1	SPG11	mRNA	TCONS_00029398	lncRNA
XM_012182238.1	SPG11	mRNA	TCONS_00029398	lncRNA
XM_012182238.1	SPG11	mRNA	TCONS_00029398	lncRNA
XM_012182238.1	SPG11	mRNA	TCONS_00029398	lncRNA
XM_012182238.1	SPG11	mRNA	TCONS_00029398	lncRNA
XM_015101107.1	TCP11L1	mRNA	TCONS_00029398	lncRNA
XM_015101107.1	TCP11L1	mRNA	TCONS_00029398	lncRNA
XM_015101107.1	TCP11L1	mRNA	TCONS_00029398	lncRNA
XM_015101107.1	TCP11L1	mRNA	TCONS_00029398	lncRNA
XM_015101107.1	TCP11L1	mRNA	TCONS_00029398	lncRNA
XM_012099916.2	PRKCD	mRNA	TCONS_00029398	lncRNA
XM_012099916.2	PRKCD	mRNA	TCONS_00029398	lncRNA
XM_012099916.2	PRKCD	mRNA	TCONS_00029398	lncRNA
XM_012099916.2	PRKCD	mRNA	TCONS_00029398	lncRNA
XM_012099916.2	PRKCD	mRNA	TCONS_00029398	lncRNA
XM_012102077.2	LGALS12	mRNA	TCONS_00029398	lncRNA
XM_012102077.2	LGALS12	mRNA	TCONS_00029398	lncRNA
XM_012102077.2	LGALS12	mRNA	TCONS_00029398	lncRNA
XM_012102077.2	LGALS12	mRNA	TCONS_00029398	lncRNA
XM_012102077.2	LGALS12	mRNA	TCONS_00029398	lncRNA
XM_004012194.3	RBM26	mRNA	XR_001021689.2	lncRNA
XM_004012194.3	RBM26	mRNA	XR_001021689.2	lncRNA
XM_004012194.3	RBM26	mRNA	XR_001021689.2	lncRNA
XM_004012194.3	RBM26	mRNA	XR_001021689.2	lncRNA
XM_004012194.3	RBM26	mRNA	XR_001021689.2	lncRNA
XM_012149251.2	S100PBP	mRNA	XR_001021689.2	lncRNA
XM_012149251.2	S100PBP	mRNA	XR_001021689.2	lncRNA
XM_012149251.2	S100PBP	mRNA	XR_001021689.2	lncRNA

XM_012149251.2	S100PBP	mRNA	XR_001021689.2	lncRNA
XM_012149251.2	S100PBP	mRNA	XR_001021689.2	lncRNA
XM_012142889.2	ICA1L	mRNA	XR_001021689.2	lncRNA
XM_012142889.2	ICA1L	mRNA	XR_001021689.2	lncRNA
XM_012142889.2	ICA1L	mRNA	XR_001021689.2	lncRNA
XM_012142889.2	ICA1L	mRNA	XR_001021689.2	lncRNA
XM_012142889.2	ICA1L	mRNA	XR_001021689.2	lncRNA
XM_004001781.3	ZSCAN20	mRNA	XR_001021689.2	lncRNA
XM_004001781.3	ZSCAN20	mRNA	XR_001021689.2	lncRNA
XM_004001781.3	ZSCAN20	mRNA	XR_001021689.2	lncRNA
XM_004001781.3	ZSCAN20	mRNA	XR_001021689.2	lncRNA
XM_004001781.3	ZSCAN20	mRNA	XR_001021689.2	lncRNA
XM_012187063.2	MARK1	mRNA	XR_001021689.2	lncRNA
XM_012187063.2	MARK1	mRNA	XR_001021689.2	lncRNA
XM_012187063.2	MARK1	mRNA	XR_001021689.2	lncRNA
XM_012187063.2	MARK1	mRNA	XR_001021689.2	lncRNA
XM_012187063.2	MARK1	mRNA	XR_001021689.2	lncRNA
XM_012133845.1	DYNC1I2	mRNA	XR_001041209.2	lncRNA
XM_012133845.1	DYNC1I2	mRNA	XR_001041209.2	lncRNA
XM_012133845.1	DYNC1I2	mRNA	XR_001041209.2	lncRNA
XM_012133845.1	DYNC1I2	mRNA	XR_001041209.2	lncRNA
XM_012133845.1	DYNC1I2	mRNA	XR_001041209.2	lncRNA
XM_015097323.1	PKIB	mRNA	TCONS_00029399	lncRNA
XM_015097323.1	PKIB	mRNA	TCONS_00029399	lncRNA
XM_015097323.1	PKIB	mRNA	TCONS_00029399	lncRNA
XM_015097323.1	PKIB	mRNA	TCONS_00029399	lncRNA
XM_015097323.1	PKIB	mRNA	TCONS_00029399	lncRNA
XM_012105802.2	PCM1	mRNA	TCONS_00029399	lncRNA
XM_012105802.2	PCM1	mRNA	TCONS_00029399	lncRNA
XM_012105802.2	PCM1	mRNA	TCONS_00029399	lncRNA
XM_012105802.2	PCM1	mRNA	TCONS_00029399	lncRNA
XM_012105802.2	PCM1	mRNA	TCONS_00029399	lncRNA
XM_015097323.1	PKIB	mRNA	XR_001041209.2	lncRNA
XM_015097323.1	PKIB	mRNA	XR_001041209.2	lncRNA
XM_015097323.1	PKIB	mRNA	XR_001041209.2	lncRNA
XM_015097323.1	PKIB	mRNA	XR_001041209.2	lncRNA
XM_015097323.1	PKIB	mRNA	XR_001041209.2	lncRNA
XM_012174754.2	RNF41	mRNA	TCONS_00029399	lncRNA
XM_012174754.2	RNF41	mRNA	TCONS_00029399	lncRNA
XM_012174754.2	RNF41	mRNA	TCONS_00029399	lncRNA
XM_012174754.2	RNF41	mRNA	TCONS_00029399	lncRNA
XM_012174754.2	RNF41	mRNA	TCONS_00029399	lncRNA
XM_004008647.3	RAPGEF6	mRNA	TCONS_00029399	lncRNA
XM_004008647.3	RAPGEF6	mRNA	TCONS_00029399	lncRNA
XM_004008647.3	RAPGEF6	mRNA	TCONS_00029399	lncRNA
XM_004008647.3	RAPGEF6	mRNA	TCONS_00029399	lncRNA
XM_004008647.3	RAPGEF6	mRNA	TCONS_00029399	lncRNA
XM_012180800.1	TIPIN	mRNA	TCONS_00029399	lncRNA
XM_012180800.1	TIPIN	mRNA	TCONS_00029399	lncRNA
XM_012180800.1	TIPIN	mRNA	TCONS_00029399	lncRNA
XM_012180800.1	TIPIN	mRNA	TCONS_00029399	lncRNA
XM_012180800.1	TIPIN	mRNA	TCONS_00029399	lncRNA
XM_012190105.1	ANKRL17	mRNA	XR_001041211.2	lncRNA
XM_012190105.1	ANKRL17	mRNA	XR_001041211.2	lncRNA

XM_012190105.1	ANKRL17	mRNA	XR_001041211.2	lncRNA
XM_012190105.1	ANKRL17	mRNA	XR_001041211.2	lncRNA
XM_012190105.1	ANKRL17	mRNA	XR_001041211.2	lncRNA
XM_015104131.1	TNRC6A	mRNA	XR_001041211.2	lncRNA
XM_015104131.1	TNRC6A	mRNA	XR_001041211.2	lncRNA
XM_015104131.1	TNRC6A	mRNA	XR_001041211.2	lncRNA
XM_015104131.1	TNRC6A	mRNA	XR_001041211.2	lncRNA
XM_015104131.1	TNRC6A	mRNA	XR_001041211.2	lncRNA
XM_015103687.1	PTPN2	mRNA	TCONS_00029399	lncRNA
XM_015103687.1	PTPN2	mRNA	TCONS_00029399	lncRNA
XM_015103687.1	PTPN2	mRNA	TCONS_00029399	lncRNA
XM_015103687.1	PTPN2	mRNA	TCONS_00029399	lncRNA
XM_015103687.1	PTPN2	mRNA	TCONS_00029399	lncRNA
XM_012105800.2	PCM1	mRNA	XR_001041211.2	lncRNA
XM_012105800.2	PCM1	mRNA	XR_001041211.2	lncRNA
XM_012105800.2	PCM1	mRNA	XR_001041211.2	lncRNA
XM_012105800.2	PCM1	mRNA	XR_001041211.2	lncRNA
XM_012105800.2	PCM1	mRNA	XR_001041211.2	lncRNA
XM_012173983.2	PPP1R12A	mRNA	XR_001041209.2	lncRNA
XM_012173983.2	PPP1R12A	mRNA	XR_001041209.2	lncRNA
XM_012173983.2	PPP1R12A	mRNA	XR_001041209.2	lncRNA
XM_012173983.2	PPP1R12A	mRNA	XR_001041209.2	lncRNA
XM_012173983.2	PPP1R12A	mRNA	XR_001041209.2	lncRNA
XM_015093098.1	PPP3CC	mRNA	XR_001436609.1	lncRNA
XM_015093098.1	PPP3CC	mRNA	XR_001436609.1	lncRNA
XM_015093098.1	PPP3CC	mRNA	XR_001436609.1	lncRNA
XM_015093098.1	PPP3CC	mRNA	XR_001436609.1	lncRNA
XM_015093098.1	PPP3CC	mRNA	XR_001436609.1	lncRNA
XM_012179421.1	METTL14	mRNA	XR_001435104.1	lncRNA
XM_012179421.1	METTL14	mRNA	XR_001435104.1	lncRNA
XM_012179421.1	METTL14	mRNA	XR_001435104.1	lncRNA
XM_012179421.1	METTL14	mRNA	XR_001435104.1	lncRNA
XM_012179421.1	METTL14	mRNA	XR_001435104.1	lncRNA
XM_012169683.2	LOC1011072(	mRNA	XR_001436609.1	lncRNA
XM_012169683.2	LOC1011072(	mRNA	XR_001436609.1	lncRNA
XM_012169683.2	LOC1011072(	mRNA	XR_001436609.1	lncRNA
XM_012169683.2	LOC1011072(	mRNA	XR_001436609.1	lncRNA
XM_012169683.2	LOC1011072(	mRNA	XR_001436609.1	lncRNA
XM_012187919.2	LOC1056166	mRNA	XR_001436609.1	lncRNA
XM_012187919.2	LOC1056166	mRNA	XR_001436609.1	lncRNA
XM_012187919.2	LOC1056166	mRNA	XR_001436609.1	lncRNA
XM_012187919.2	LOC1056166	mRNA	XR_001436609.1	lncRNA
XM_012187919.2	LOC1056166	mRNA	XR_001436609.1	lncRNA
XM_015092197.1	CD58	mRNA	XR_001436609.1	lncRNA
XM_015092197.1	CD58	mRNA	XR_001436609.1	lncRNA
XM_015092197.1	CD58	mRNA	XR_001436609.1	lncRNA
XM_015092197.1	CD58	mRNA	XR_001436609.1	lncRNA
XM_015092197.1	CD58	mRNA	XR_001436609.1	lncRNA
XM_012106112.2	ARAF	mRNA	XR_001435104.1	lncRNA
XM_012106112.2	ARAF	mRNA	XR_001435104.1	lncRNA
XM_012106112.2	ARAF	mRNA	XR_001435104.1	lncRNA
XM_012106112.2	ARAF	mRNA	XR_001435104.1	lncRNA
XM_012106112.2	ARAF	mRNA	XR_001435104.1	lncRNA
XM_015099863.1	DSN1	mRNA	XR_001435104.1	lncRNA

XM_015099863.1	DSN1	mRNA	XR_001435104.1	lncRNA
XM_015099863.1	DSN1	mRNA	XR_001435104.1	lncRNA
XM_015099863.1	DSN1	mRNA	XR_001435104.1	lncRNA
XM_015099863.1	DSN1	mRNA	XR_001435104.1	lncRNA
XM_015094750.1	RAB3IP	mRNA	XR_001435104.1	lncRNA
XM_015094750.1	RAB3IP	mRNA	XR_001435104.1	lncRNA
XM_015094750.1	RAB3IP	mRNA	XR_001435104.1	lncRNA
XM_015094750.1	RAB3IP	mRNA	XR_001435104.1	lncRNA
XM_015094750.1	RAB3IP	mRNA	XR_001435104.1	lncRNA
XM_015104027.1	PDPK1	mRNA	XR_001435104.1	lncRNA
XM_015104027.1	PDPK1	mRNA	XR_001435104.1	lncRNA
XM_015104027.1	PDPK1	mRNA	XR_001435104.1	lncRNA
XM_015104027.1	PDPK1	mRNA	XR_001435104.1	lncRNA
XM_015104027.1	PDPK1	mRNA	XR_001435104.1	lncRNA
XM_012188301.2	NEBL	mRNA	XR_001435104.1	lncRNA
XM_012188301.2	NEBL	mRNA	XR_001435104.1	lncRNA
XM_012188301.2	NEBL	mRNA	XR_001435104.1	lncRNA
XM_012188301.2	NEBL	mRNA	XR_001435104.1	lncRNA
XM_012188301.2	NEBL	mRNA	XR_001435104.1	lncRNA
XM_012098017.2	KREMEN1	mRNA	XR_001033284.2	lncRNA
XM_012098017.2	KREMEN1	mRNA	XR_001033284.2	lncRNA
XM_012098017.2	KREMEN1	mRNA	XR_001033284.2	lncRNA
XM_012098017.2	KREMEN1	mRNA	XR_001033284.2	lncRNA
XM_012098017.2	KREMEN1	mRNA	XR_001033284.2	lncRNA
XM_012187292.1	VPS13D	mRNA	TCONS_00029398	lncRNA
XM_012187292.1	VPS13D	mRNA	TCONS_00029398	lncRNA
XM_012187292.1	VPS13D	mRNA	TCONS_00029398	lncRNA
XM_012187292.1	VPS13D	mRNA	TCONS_00029398	lncRNA
XM_012187292.1	VPS13D	mRNA	TCONS_00029398	lncRNA
XM_015102528.1	FLOT1	mRNA	XR_001435726.1	lncRNA
XM_015102528.1	FLOT1	mRNA	XR_001435726.1	lncRNA
XM_015102528.1	FLOT1	mRNA	XR_001435726.1	lncRNA
XM_015102528.1	FLOT1	mRNA	XR_001435726.1	lncRNA
XM_015102528.1	FLOT1	mRNA	XR_001435726.1	lncRNA
XM_012184148.1	OTUL3B	mRNA	XR_001042381.2	lncRNA
XM_012184148.1	OTUL3B	mRNA	XR_001042381.2	lncRNA
XM_012184148.1	OTUL3B	mRNA	XR_001042381.2	lncRNA
XM_012184148.1	OTUL3B	mRNA	XR_001042381.2	lncRNA
XM_012184148.1	OTUL3B	mRNA	XR_001042381.2	lncRNA
XM_012174540.1	CPNE8	mRNA	XR_001042381.2	lncRNA
XM_012174540.1	CPNE8	mRNA	XR_001042381.2	lncRNA
XM_012174540.1	CPNE8	mRNA	XR_001042381.2	lncRNA
XM_012174540.1	CPNE8	mRNA	XR_001042381.2	lncRNA
XM_012174540.1	CPNE8	mRNA	XR_001042381.2	lncRNA
XM_015105047.1	XIAP	mRNA	XR_001435723.1	lncRNA
XM_015105047.1	XIAP	mRNA	XR_001435723.1	lncRNA
XM_015105047.1	XIAP	mRNA	XR_001435723.1	lncRNA
XM_015105047.1	XIAP	mRNA	XR_001435723.1	lncRNA
XM_015105047.1	XIAP	mRNA	XR_001435723.1	lncRNA
XM_015104027.1	PDPK1	mRNA	XR_001435723.1	lncRNA
XM_015104027.1	PDPK1	mRNA	XR_001435723.1	lncRNA
XM_015104027.1	PDPK1	mRNA	XR_001435723.1	lncRNA
XM_015104027.1	PDPK1	mRNA	XR_001435723.1	lncRNA
XM_015104027.1	PDPK1	mRNA	XR_001435723.1	lncRNA

XM_015105047.1	XIAP	mRNA	XR_001435726.1	lncRNA
XM_015105047.1	XIAP	mRNA	XR_001435726.1	lncRNA
XM_015105047.1	XIAP	mRNA	XR_001435726.1	lncRNA
XM_015105047.1	XIAP	mRNA	XR_001435726.1	lncRNA
XM_015105047.1	XIAP	mRNA	XR_001435726.1	lncRNA
XM_015097323.1	PKIB	mRNA	XR_001434081.1	lncRNA
XM_015097323.1	PKIB	mRNA	XR_001434081.1	lncRNA
XM_015097323.1	PKIB	mRNA	XR_001434081.1	lncRNA
XM_015097323.1	PKIB	mRNA	XR_001434081.1	lncRNA
XM_015097323.1	PKIB	mRNA	XR_001434081.1	lncRNA
XM_012174787.1	LOC1011137	mRNA	XR_001434081.1	lncRNA
XM_012174787.1	LOC1011137	mRNA	XR_001434081.1	lncRNA
XM_012174787.1	LOC1011137	mRNA	XR_001434081.1	lncRNA
XM_012174787.1	LOC1011137	mRNA	XR_001434081.1	lncRNA
XM_012174787.1	LOC1011137	mRNA	XR_001434081.1	lncRNA
XM_004022347.2	TMEM255A	mRNA	XR_001434081.1	lncRNA
XM_004022347.2	TMEM255A	mRNA	XR_001434081.1	lncRNA
XM_004022347.2	TMEM255A	mRNA	XR_001434081.1	lncRNA
XM_004022347.2	TMEM255A	mRNA	XR_001434081.1	lncRNA
XM_004022347.2	TMEM255A	mRNA	XR_001434081.1	lncRNA
XM_012101922.2	ZBTB44	mRNA	XR_001021689.2	lncRNA
XM_012101922.2	ZBTB44	mRNA	XR_001021689.2	lncRNA
XM_012101922.2	ZBTB44	mRNA	XR_001021689.2	lncRNA
XM_012101922.2	ZBTB44	mRNA	XR_001021689.2	lncRNA
XM_012101922.2	ZBTB44	mRNA	XR_001021689.2	lncRNA
XM_015098446.1	VEZF1	mRNA	XR_001021689.2	lncRNA
XM_015098446.1	VEZF1	mRNA	XR_001021689.2	lncRNA
XM_015098446.1	VEZF1	mRNA	XR_001021689.2	lncRNA
XM_015098446.1	VEZF1	mRNA	XR_001021689.2	lncRNA
XM_015098446.1	VEZF1	mRNA	XR_001021689.2	lncRNA
XM_012102077.2	LGALS12	mRNA	TCONS_00071561	lncRNA
XM_012102077.2	LGALS12	mRNA	TCONS_00071561	lncRNA
XM_012102077.2	LGALS12	mRNA	TCONS_00071561	lncRNA
XM_012102077.2	LGALS12	mRNA	TCONS_00071561	lncRNA
XM_012102077.2	LGALS12	mRNA	TCONS_00071561	lncRNA
XM_012099916.2	PRKCD	mRNA	XR_001021689.2	lncRNA
XM_012099916.2	PRKCD	mRNA	XR_001021689.2	lncRNA
XM_012099916.2	PRKCD	mRNA	XR_001021689.2	lncRNA
XM_012099916.2	PRKCD	mRNA	XR_001021689.2	lncRNA
XM_012099916.2	PRKCD	mRNA	XR_001021689.2	lncRNA
XM_012188917.1	STX16	mRNA	XR_001021689.2	lncRNA
XM_012188917.1	STX16	mRNA	XR_001021689.2	lncRNA
XM_012188917.1	STX16	mRNA	XR_001021689.2	lncRNA
XM_012188917.1	STX16	mRNA	XR_001021689.2	lncRNA
XM_012188917.1	STX16	mRNA	XR_001021689.2	lncRNA
XM_015102735.1	SUPT3H	mRNA	TCONS_00078442	lncRNA
XM_015102735.1	SUPT3H	mRNA	TCONS_00078442	lncRNA
XM_015102735.1	SUPT3H	mRNA	TCONS_00078442	lncRNA
XM_015102735.1	SUPT3H	mRNA	TCONS_00078442	lncRNA
XM_012190083.2	ZMYM6	mRNA	TCONS_00052150	lncRNA
XM_012190083.2	ZMYM6	mRNA	TCONS_00052150	lncRNA
XM_012190083.2	ZMYM6	mRNA	TCONS_00052150	lncRNA
XM_012190083.2	ZMYM6	mRNA	TCONS_00052150	lncRNA
XM_012145933.2	CXCR2	mRNA	TCONS_00052150	lncRNA

XM_012145933.2	CXCR2	mRNA	TCONS_00052150	lncRNA
XM_012145933.2	CXCR2	mRNA	TCONS_00052150	lncRNA
XM_012145933.2	CXCR2	mRNA	TCONS_00052150	lncRNA
XM_012098348.2	MEF2A	mRNA	TCONS_00029399	lncRNA
XM_012098348.2	MEF2A	mRNA	TCONS_00029399	lncRNA
XM_012098348.2	MEF2A	mRNA	TCONS_00029399	lncRNA
XM_012098348.2	MEF2A	mRNA	TCONS_00029399	lncRNA
XM_012098348.2	MEF2A	mRNA	TCONS_00029399	lncRNA
XM_012097468.2	ELF2	mRNA	XR_001041209.2	lncRNA
XM_012097468.2	ELF2	mRNA	XR_001041209.2	lncRNA
XM_012097468.2	ELF2	mRNA	XR_001041209.2	lncRNA
XM_012097468.2	ELF2	mRNA	XR_001041209.2	lncRNA
XM_012097468.2	ELF2	mRNA	XR_001041209.2	lncRNA
XM_012098348.2	MEF2A	mRNA	XR_001041209.2	lncRNA
XM_012098348.2	MEF2A	mRNA	XR_001041209.2	lncRNA
XM_012098348.2	MEF2A	mRNA	XR_001041209.2	lncRNA
XM_012098348.2	MEF2A	mRNA	XR_001041209.2	lncRNA
XM_012098348.2	MEF2A	mRNA	XR_001041209.2	lncRNA
XM_012189447.2	ALCAM	mRNA	XR_001041211.2	lncRNA
XM_012189447.2	ALCAM	mRNA	XR_001041211.2	lncRNA
XM_012189447.2	ALCAM	mRNA	XR_001041211.2	lncRNA
XM_012189447.2	ALCAM	mRNA	XR_001041211.2	lncRNA
XM_012189447.2	ALCAM	mRNA	XR_001041211.2	lncRNA
XM_012100126.2	MAP4	mRNA	TCONS_00029399	lncRNA
XM_012100126.2	MAP4	mRNA	TCONS_00029399	lncRNA
XM_012100126.2	MAP4	mRNA	TCONS_00029399	lncRNA
XM_012100126.2	MAP4	mRNA	TCONS_00029399	lncRNA
XM_012100126.2	MAP4	mRNA	TCONS_00029399	lncRNA
XM_004008093.2	PTN	mRNA	XR_001041209.2	lncRNA
XM_004008093.2	PTN	mRNA	XR_001041209.2	lncRNA
XM_004008093.2	PTN	mRNA	XR_001041209.2	lncRNA
XM_004008093.2	PTN	mRNA	XR_001041209.2	lncRNA
XM_004008093.2	PTN	mRNA	XR_001041209.2	lncRNA
XM_015102269.1	NKTR	mRNA	TCONS_00029398	lncRNA
XM_015102269.1	NKTR	mRNA	TCONS_00029398	lncRNA
XM_015102269.1	NKTR	mRNA	TCONS_00029398	lncRNA
XM_015102269.1	NKTR	mRNA	TCONS_00029398	lncRNA
XM_015102269.1	NKTR	mRNA	TCONS_00029398	lncRNA
XM_012095461.2	GOLGB1	mRNA	TCONS_00029398	lncRNA
XM_012095461.2	GOLGB1	mRNA	TCONS_00029398	lncRNA
XM_012095461.2	GOLGB1	mRNA	TCONS_00029398	lncRNA
XM_012095461.2	GOLGB1	mRNA	TCONS_00029398	lncRNA
XM_012095461.2	GOLGB1	mRNA	TCONS_00029398	lncRNA
XM_012095465.2	GOLGB1	mRNA	TCONS_00029398	lncRNA
XM_012095465.2	GOLGB1	mRNA	TCONS_00029398	lncRNA
XM_012095465.2	GOLGB1	mRNA	TCONS_00029398	lncRNA
XM_012095465.2	GOLGB1	mRNA	TCONS_00029398	lncRNA
XM_012095465.2	GOLGB1	mRNA	TCONS_00029398	lncRNA
XM_015096132.1	CSNK1A1	mRNA	XR_001021689.2	lncRNA
XM_015096132.1	CSNK1A1	mRNA	XR_001021689.2	lncRNA
XM_015096132.1	CSNK1A1	mRNA	XR_001021689.2	lncRNA
XM_015096132.1	CSNK1A1	mRNA	XR_001021689.2	lncRNA
XM_015096132.1	CSNK1A1	mRNA	XR_001021689.2	lncRNA
XM_012101112.2	ABT1	mRNA	XR_001021689.2	lncRNA

XM_012101112.2	ABT1	mRNA	XR_001021689.2	lncRNA
XM_012101112.2	ABT1	mRNA	XR_001021689.2	lncRNA
XM_012101112.2	ABT1	mRNA	XR_001021689.2	lncRNA
XM_012101112.2	ABT1	mRNA	XR_001021689.2	lncRNA
XM_012105564.2	ZFAND4	mRNA	XR_001436609.1	lncRNA
XM_012105564.2	ZFAND4	mRNA	XR_001436609.1	lncRNA
XM_012105564.2	ZFAND4	mRNA	XR_001436609.1	lncRNA
XM_012105564.2	ZFAND4	mRNA	XR_001436609.1	lncRNA
XM_012105564.2	ZFAND4	mRNA	XR_001436609.1	lncRNA
XM_012189279.2	DNTTIP1	mRNA	XR_001436609.1	lncRNA
XM_012189279.2	DNTTIP1	mRNA	XR_001436609.1	lncRNA
XM_012189279.2	DNTTIP1	mRNA	XR_001436609.1	lncRNA
XM_012189279.2	DNTTIP1	mRNA	XR_001436609.1	lncRNA
XM_012189279.2	DNTTIP1	mRNA	XR_001436609.1	lncRNA
XM_012175064.2	KIAA1551	mRNA	XR_001021689.2	lncRNA
XM_012175064.2	KIAA1551	mRNA	XR_001021689.2	lncRNA
XM_012175064.2	KIAA1551	mRNA	XR_001021689.2	lncRNA
XM_012175064.2	KIAA1551	mRNA	XR_001021689.2	lncRNA
XM_004006746.3	KIAA1551	mRNA	XR_001021689.2	lncRNA
XM_004006746.3	KIAA1551	mRNA	XR_001021689.2	lncRNA
XM_004006746.3	KIAA1551	mRNA	XR_001021689.2	lncRNA
XM_004006746.3	KIAA1551	mRNA	XR_001021689.2	lncRNA
XM_012184819.1	RNF219	mRNA	XR_001021689.2	lncRNA
XM_012184819.1	RNF219	mRNA	XR_001021689.2	lncRNA
XM_012184819.1	RNF219	mRNA	XR_001021689.2	lncRNA
XM_012184819.1	RNF219	mRNA	XR_001021689.2	lncRNA
XM_012178138.2	P4HA2	mRNA	XR_001434081.1	lncRNA
XM_012178138.2	P4HA2	mRNA	XR_001434081.1	lncRNA
XM_012178138.2	P4HA2	mRNA	XR_001434081.1	lncRNA
XM_012178138.2	P4HA2	mRNA	XR_001434081.1	lncRNA
XM_012178138.2	P4HA2	mRNA	XR_001434081.1	lncRNA
XM_012176656.2	NAPEPLD	mRNA	TCONS_00052150	lncRNA
XM_012176656.2	NAPEPLD	mRNA	TCONS_00052150	lncRNA
XM_012176656.2	NAPEPLD	mRNA	TCONS_00052150	lncRNA
XM_012176656.2	NAPEPLD	mRNA	TCONS_00052150	lncRNA
XM_012190105.1	ANKRL17	mRNA	TCONS_00052150	lncRNA
XM_012190105.1	ANKRL17	mRNA	TCONS_00052150	lncRNA
XM_012190105.1	ANKRL17	mRNA	TCONS_00052150	lncRNA
XM_012190105.1	ANKRL17	mRNA	TCONS_00052150	lncRNA
XM_012106466.2	DMD	mRNA	TCONS_00052150	lncRNA
XM_012106466.2	DMD	mRNA	TCONS_00052150	lncRNA
XM_012106466.2	DMD	mRNA	TCONS_00052150	lncRNA
XM_012106466.2	DMD	mRNA	TCONS_00052150	lncRNA
XM_004022347.2	TMEM255A	mRNA	TCONS_00052150	lncRNA
XM_004022347.2	TMEM255A	mRNA	TCONS_00052150	lncRNA
XM_004022347.2	TMEM255A	mRNA	TCONS_00052150	lncRNA
XM_004022347.2	TMEM255A	mRNA	TCONS_00052150	lncRNA
XM_012105564.2	ZFAND4	mRNA	XR_001435726.1	lncRNA
XM_012105564.2	ZFAND4	mRNA	XR_001435726.1	lncRNA
XM_012105564.2	ZFAND4	mRNA	XR_001435726.1	lncRNA
XM_012105564.2	ZFAND4	mRNA	XR_001435726.1	lncRNA
XM_012105564.2	ZFAND4	mRNA	XR_001435726.1	lncRNA
XM_012105316.2	USP54	mRNA	XR_001041211.2	lncRNA
XM_012105316.2	USP54	mRNA	XR_001041211.2	lncRNA

XM_012105316.2	USP54	mRNA	XR_001041211.2	lncRNA
XM_012105316.2	USP54	mRNA	XR_001041211.2	lncRNA
XM_012105316.2	USP54	mRNA	XR_001041211.2	lncRNA
XM_015104393.1	RUFY2	mRNA	XR_001041209.2	lncRNA
XM_015104393.1	RUFY2	mRNA	XR_001041209.2	lncRNA
XM_015104393.1	RUFY2	mRNA	XR_001041209.2	lncRNA
XM_015104393.1	RUFY2	mRNA	XR_001041209.2	lncRNA
XM_015104393.1	RUFY2	mRNA	XR_001041209.2	lncRNA
XM_012095733.1	BTBD10	mRNA	XR_001021689.2	lncRNA
XM_012095733.1	BTBD10	mRNA	XR_001021689.2	lncRNA
XM_012095733.1	BTBD10	mRNA	XR_001021689.2	lncRNA
XM_012095733.1	BTBD10	mRNA	XR_001021689.2	lncRNA
XM_012095733.1	BTBD10	mRNA	XR_001021689.2	lncRNA
XM_015091991.1	YIPF1	mRNA	XR_001041202.1	lncRNA
XM_015091991.1	YIPF1	mRNA	XR_001041202.1	lncRNA
XM_015091991.1	YIPF1	mRNA	XR_001041202.1	lncRNA
XM_015091991.1	YIPF1	mRNA	XR_001041202.1	lncRNA
XM_012176498.2	RAPGEF5	mRNA	XR_001436099.1	lncRNA
XM_012176498.2	RAPGEF5	mRNA	XR_001436099.1	lncRNA
XM_012176498.2	RAPGEF5	mRNA	XR_001436099.1	lncRNA
XM_012176498.2	RAPGEF5	mRNA	XR_001436099.1	lncRNA
XM_012176498.2	RAPGEF5	mRNA	XR_001436099.1	lncRNA
XM_012169683.2	LOC1011072(	mRNA	XR_001039281.1	lncRNA
XM_012169683.2	LOC1011072(	mRNA	XR_001039281.1	lncRNA
XM_012169683.2	LOC1011072(	mRNA	XR_001039281.1	lncRNA
XM_012169683.2	LOC1011072(	mRNA	XR_001039281.1	lncRNA
XM_012145933.2	CXCR2	mRNA	TCONS_00072289	lncRNA
XM_012145933.2	CXCR2	mRNA	TCONS_00072289	lncRNA
XM_012145933.2	CXCR2	mRNA	TCONS_00072289	lncRNA
XM_012145933.2	CXCR2	mRNA	TCONS_00072289	lncRNA
XM_012095272.2	ARNTL	mRNA	TCONS_00072289	lncRNA
XM_012095272.2	ARNTL	mRNA	TCONS_00072289	lncRNA
XM_012095272.2	ARNTL	mRNA	TCONS_00072289	lncRNA
XM_012095272.2	ARNTL	mRNA	TCONS_00072289	lncRNA
XM_004014209.3	CUL2	mRNA	XR_001033284.2	lncRNA
XM_004014209.3	CUL2	mRNA	XR_001033284.2	lncRNA
XM_004014209.3	CUL2	mRNA	XR_001033284.2	lncRNA
XM_004014209.3	CUL2	mRNA	XR_001033284.2	lncRNA
XM_012103008.2	FAM45A	mRNA	XR_001033284.2	lncRNA
XM_012103008.2	FAM45A	mRNA	XR_001033284.2	lncRNA
XM_012103008.2	FAM45A	mRNA	XR_001033284.2	lncRNA
XM_012103008.2	FAM45A	mRNA	XR_001033284.2	lncRNA
XM_012190349.2	ZNF420	mRNA	XR_001033284.2	lncRNA
XM_012190349.2	ZNF420	mRNA	XR_001033284.2	lncRNA
XM_012190349.2	ZNF420	mRNA	XR_001033284.2	lncRNA
XM_012190349.2	ZNF420	mRNA	XR_001033284.2	lncRNA
XM_012096375.2	PSMB2	mRNA	XR_001033284.2	lncRNA
XM_012096375.2	PSMB2	mRNA	XR_001033284.2	lncRNA
XM_012096375.2	PSMB2	mRNA	XR_001033284.2	lncRNA
XM_012096375.2	PSMB2	mRNA	XR_001033284.2	lncRNA
XM_012098342.2	FXR1	mRNA	TCONS_00078442	lncRNA
XM_012098342.2	FXR1	mRNA	TCONS_00078442	lncRNA
XM_012098342.2	FXR1	mRNA	TCONS_00078442	lncRNA
XM_012098342.2	FXR1	mRNA	TCONS_00078442	lncRNA

XM_012100126.2	MAP4	mRNA	TCONS_00078442	lncRNA
XM_012100126.2	MAP4	mRNA	TCONS_00078442	lncRNA
XM_012100126.2	MAP4	mRNA	TCONS_00078442	lncRNA
XM_012100126.2	MAP4	mRNA	TCONS_00078442	lncRNA
XM_012098458.2	FANCI	mRNA	TCONS_00052150	lncRNA
XM_012098458.2	FANCI	mRNA	TCONS_00052150	lncRNA
XM_012098458.2	FANCI	mRNA	TCONS_00052150	lncRNA
XM_012098458.2	FANCI	mRNA	TCONS_00052150	lncRNA
XM_012181854.1	GPHN	mRNA	TCONS_00052150	lncRNA
XM_012181854.1	GPHN	mRNA	TCONS_00052150	lncRNA
XM_012181854.1	GPHN	mRNA	TCONS_00052150	lncRNA
XM_012181854.1	GPHN	mRNA	TCONS_00052150	lncRNA
XM_015100095.1	LOC10111352	mRNA	TCONS_00078442	lncRNA
XM_015100095.1	LOC10111352	mRNA	TCONS_00078442	lncRNA
XM_015100095.1	LOC10111352	mRNA	TCONS_00078442	lncRNA
XM_015100095.1	LOC10111352	mRNA	TCONS_00078442	lncRNA
XM_012119517.2	SORBS3	mRNA	TCONS_00029398	lncRNA
XM_012119517.2	SORBS3	mRNA	TCONS_00029398	lncRNA
XM_012119517.2	SORBS3	mRNA	TCONS_00029398	lncRNA
XM_012119517.2	SORBS3	mRNA	TCONS_00029398	lncRNA
XM_012134498.2	PPIG	mRNA	TCONS_00029398	lncRNA
XM_012134498.2	PPIG	mRNA	TCONS_00029398	lncRNA
XM_012134498.2	PPIG	mRNA	TCONS_00029398	lncRNA
XM_012134498.2	PPIG	mRNA	TCONS_00029398	lncRNA
XM_004001901.2	ERI3	mRNA	XR_001041211.2	lncRNA
XM_004001901.2	ERI3	mRNA	XR_001041211.2	lncRNA
XM_004001901.2	ERI3	mRNA	XR_001041211.2	lncRNA
XM_004001901.2	ERI3	mRNA	XR_001041211.2	lncRNA
XM_012096375.2	PSMB2	mRNA	XR_001041209.2	lncRNA
XM_012096375.2	PSMB2	mRNA	XR_001041209.2	lncRNA
XM_012096375.2	PSMB2	mRNA	XR_001041209.2	lncRNA
XM_012096375.2	PSMB2	mRNA	XR_001041209.2	lncRNA
XM_012170748.1	MIER1	mRNA	XR_001041209.2	lncRNA
XM_012170748.1	MIER1	mRNA	XR_001041209.2	lncRNA
XM_012170748.1	MIER1	mRNA	XR_001041209.2	lncRNA
XM_012170748.1	MIER1	mRNA	XR_001041209.2	lncRNA
XM_015097326.1	TBC1D32	mRNA	XR_001041209.2	lncRNA
XM_015097326.1	TBC1D32	mRNA	XR_001041209.2	lncRNA
XM_015097326.1	TBC1D32	mRNA	XR_001041209.2	lncRNA
XM_015097326.1	TBC1D32	mRNA	XR_001041209.2	lncRNA
XM_012096375.2	PSMB2	mRNA	TCONS_00029399	lncRNA
XM_012096375.2	PSMB2	mRNA	TCONS_00029399	lncRNA
XM_012096375.2	PSMB2	mRNA	TCONS_00029399	lncRNA
XM_012096375.2	PSMB2	mRNA	TCONS_00029399	lncRNA
XM_012137599.2	NEB	mRNA	TCONS_00063226	lncRNA
XM_012137599.2	NEB	mRNA	TCONS_00063226	lncRNA
XM_012137599.2	NEB	mRNA	TCONS_00063226	lncRNA
XM_012137599.2	NEB	mRNA	TCONS_00063226	lncRNA
XM_012174787.1	LOC10111374	mRNA	TCONS_00072289	lncRNA
XM_012174787.1	LOC10111374	mRNA	TCONS_00072289	lncRNA
XM_012174787.1	LOC10111374	mRNA	TCONS_00072289	lncRNA
XM_012174787.1	LOC10111374	mRNA	TCONS_00072289	lncRNA
XM_012190105.1	ANKRL17	mRNA	TCONS_00072289	lncRNA
XM_012190105.1	ANKRL17	mRNA	TCONS_00072289	lncRNA

XM_012190105.1	ANKRL17	mRNA	TCONS_00072289	lncRNA
XM_012190105.1	ANKRL17	mRNA	TCONS_00072289	lncRNA
XM_012188017.2	ZNF654	mRNA	XR_001039281.1	lncRNA
XM_012188017.2	ZNF654	mRNA	XR_001039281.1	lncRNA
XM_012188017.2	ZNF654	mRNA	XR_001039281.1	lncRNA
XM_012188017.2	ZNF654	mRNA	XR_001039281.1	lncRNA
XM_015103643.1	YES1	mRNA	XR_001021689.2	lncRNA
XM_015103643.1	YES1	mRNA	XR_001021689.2	lncRNA
XM_015103643.1	YES1	mRNA	XR_001021689.2	lncRNA
XM_015103643.1	YES1	mRNA	XR_001021689.2	lncRNA
XM_012180229.1	CCNG2	mRNA	XR_001021689.2	lncRNA
XM_012180229.1	CCNG2	mRNA	XR_001021689.2	lncRNA
XM_012180229.1	CCNG2	mRNA	XR_001021689.2	lncRNA
XM_012180229.1	CCNG2	mRNA	XR_001021689.2	lncRNA
XM_012166941.1	BRE	mRNA	XR_001021689.2	lncRNA
XM_012166941.1	BRE	mRNA	XR_001021689.2	lncRNA
XM_012166941.1	BRE	mRNA	XR_001021689.2	lncRNA
XM_012166941.1	BRE	mRNA	XR_001021689.2	lncRNA
XM_004012037.3	TDRD3	mRNA	XR_001021689.2	lncRNA
XM_004012037.3	TDRD3	mRNA	XR_001021689.2	lncRNA
XM_004012037.3	TDRD3	mRNA	XR_001021689.2	lncRNA
XM_004012037.3	TDRD3	mRNA	XR_001021689.2	lncRNA
XM_012104035.2	CLUAP1	mRNA	XR_001021689.2	lncRNA
XM_012104035.2	CLUAP1	mRNA	XR_001021689.2	lncRNA
XM_012104035.2	CLUAP1	mRNA	XR_001021689.2	lncRNA
XM_012104035.2	CLUAP1	mRNA	XR_001021689.2	lncRNA
XM_012188288.2	ARL5B	mRNA	TCONS_00078442	lncRNA
XM_012188288.2	ARL5B	mRNA	TCONS_00078442	lncRNA
XM_012188288.2	ARL5B	mRNA	TCONS_00078442	lncRNA
XM_012188288.2	ARL5B	mRNA	TCONS_00078442	lncRNA
XM_012113353.2	SUSD1	mRNA	TCONS_00052150	lncRNA
XM_012113353.2	SUSD1	mRNA	TCONS_00052150	lncRNA
XM_012113353.2	SUSD1	mRNA	TCONS_00052150	lncRNA
XM_012113353.2	SUSD1	mRNA	TCONS_00052150	lncRNA
XM_012114904.1	RNF20	mRNA	TCONS_00052150	lncRNA
XM_012114904.1	RNF20	mRNA	TCONS_00052150	lncRNA
XM_012114904.1	RNF20	mRNA	TCONS_00052150	lncRNA
XM_012114904.1	RNF20	mRNA	TCONS_00052150	lncRNA
XM_004001781.3	ZSCAN20	mRNA	TCONS_00078442	lncRNA
XM_004001781.3	ZSCAN20	mRNA	TCONS_00078442	lncRNA
XM_004001781.3	ZSCAN20	mRNA	TCONS_00078442	lncRNA
XM_004001781.3	ZSCAN20	mRNA	TCONS_00078442	lncRNA
XM_012146156.1	USP37	mRNA	TCONS_00078442	lncRNA
XM_012146156.1	USP37	mRNA	TCONS_00078442	lncRNA
XM_012146156.1	USP37	mRNA	TCONS_00078442	lncRNA
XM_012146156.1	USP37	mRNA	TCONS_00078442	lncRNA
XM_012177560.2	SLC26A2	mRNA	TCONS_00052150	lncRNA
XM_012177560.2	SLC26A2	mRNA	TCONS_00052150	lncRNA
XM_012177560.2	SLC26A2	mRNA	TCONS_00052150	lncRNA
XM_012177560.2	SLC26A2	mRNA	TCONS_00052150	lncRNA
XM_012186340.1	POLG2	mRNA	TCONS_00052150	lncRNA
XM_012186340.1	POLG2	mRNA	TCONS_00052150	lncRNA
XM_012186340.1	POLG2	mRNA	TCONS_00052150	lncRNA
XM_012186340.1	POLG2	mRNA	TCONS_00052150	lncRNA

XM_012179621.1	DNAJB14	mRNA	XR_001434081.1	lncRNA
XM_012179621.1	DNAJB14	mRNA	XR_001434081.1	lncRNA
XM_012179621.1	DNAJB14	mRNA	XR_001434081.1	lncRNA
XM_012179621.1	DNAJB14	mRNA	XR_001434081.1	lncRNA
XM_015091991.1	YIPF1	mRNA	XR_001434081.1	lncRNA
XM_015091991.1	YIPF1	mRNA	XR_001434081.1	lncRNA
XM_015091991.1	YIPF1	mRNA	XR_001434081.1	lncRNA
XM_015091991.1	YIPF1	mRNA	XR_001434081.1	lncRNA
XM_015097326.1	TBC1D32	mRNA	XR_001434081.1	lncRNA
XM_015097326.1	TBC1D32	mRNA	XR_001434081.1	lncRNA
XM_015097326.1	TBC1D32	mRNA	XR_001434081.1	lncRNA
XM_015097326.1	TBC1D32	mRNA	XR_001434081.1	lncRNA
XM_004016017.3	C15H11orf1	mRNA	XR_001021939.1	lncRNA
XM_004016017.3	C15H11orf1	mRNA	XR_001021939.1	lncRNA
XM_004016017.3	C15H11orf1	mRNA	XR_001021939.1	lncRNA
XM_004016017.3	C15H11orf1	mRNA	XR_001021939.1	lncRNA
XM_015100510.1	ZFP90	mRNA	TCONS_00029398	lncRNA
XM_015100510.1	ZFP90	mRNA	TCONS_00029398	lncRNA
XM_015100510.1	ZFP90	mRNA	TCONS_00029398	lncRNA
XM_015100510.1	ZFP90	mRNA	TCONS_00029398	lncRNA
XM_015102735.1	SUPT3H	mRNA	TCONS_00029398	lncRNA
XM_015102735.1	SUPT3H	mRNA	TCONS_00029398	lncRNA
XM_015102735.1	SUPT3H	mRNA	TCONS_00029398	lncRNA
XM_015102735.1	SUPT3H	mRNA	TCONS_00029398	lncRNA
XM_012178138.2	P4HA2	mRNA	TCONS_00072289	lncRNA
XM_012178138.2	P4HA2	mRNA	TCONS_00072289	lncRNA
XM_012178138.2	P4HA2	mRNA	TCONS_00072289	lncRNA
XM_012178138.2	P4HA2	mRNA	TCONS_00072289	lncRNA
XM_012098458.2	FANCI	mRNA	TCONS_00072289	lncRNA
XM_012098458.2	FANCI	mRNA	TCONS_00072289	lncRNA
XM_012098458.2	FANCI	mRNA	TCONS_00072289	lncRNA
XM_012098458.2	FANCI	mRNA	TCONS_00072289	lncRNA
XM_012178157.2	RAPGEF6	mRNA	TCONS_00072289	lncRNA
XM_012178157.2	RAPGEF6	mRNA	TCONS_00072289	lncRNA
XM_012178157.2	RAPGEF6	mRNA	TCONS_00072289	lncRNA
XM_012178157.2	RAPGEF6	mRNA	TCONS_00072289	lncRNA
XM_012146843.1	EPHA4	mRNA	TCONS_00072289	lncRNA
XM_012146843.1	EPHA4	mRNA	TCONS_00072289	lncRNA
XM_012146843.1	EPHA4	mRNA	TCONS_00072289	lncRNA
XM_012146843.1	EPHA4	mRNA	TCONS_00072289	lncRNA
XM_012188917.1	STX16	mRNA	TCONS_00078442	lncRNA
XM_012188917.1	STX16	mRNA	TCONS_00078442	lncRNA
XM_012188917.1	STX16	mRNA	TCONS_00078442	lncRNA
XM_012188917.1	STX16	mRNA	TCONS_00078442	lncRNA
XM_004003140.2	NAALADL2	mRNA	TCONS_00078442	lncRNA
XM_004003140.2	NAALADL2	mRNA	TCONS_00078442	lncRNA
XM_004003140.2	NAALADL2	mRNA	TCONS_00078442	lncRNA
XM_004003140.2	NAALADL2	mRNA	TCONS_00078442	lncRNA
XM_015096814.1	DENND4A	mRNA	TCONS_00052150	lncRNA
XM_015096814.1	DENND4A	mRNA	TCONS_00052150	lncRNA
XM_015096814.1	DENND4A	mRNA	TCONS_00052150	lncRNA
XM_015096814.1	DENND4A	mRNA	TCONS_00052150	lncRNA
XM_012096185.2	TCP11L1	mRNA	TCONS_00052150	lncRNA
XM_012096185.2	TCP11L1	mRNA	TCONS_00052150	lncRNA

XM_012096185.2	TCP11L1	mRNA	TCONS_00052150	lncRNA
XM_012096185.2	TCP11L1	mRNA	TCONS_00052150	lncRNA
XM_012102669.1	ZNF518A	mRNA	TCONS_00078442	lncRNA
XM_012102669.1	ZNF518A	mRNA	TCONS_00078442	lncRNA
XM_012102669.1	ZNF518A	mRNA	TCONS_00078442	lncRNA
XM_012102669.1	ZNF518A	mRNA	TCONS_00078442	lncRNA
XM_012101922.2	ZBTB44	mRNA	TCONS_00078442	lncRNA
XM_012101922.2	ZBTB44	mRNA	TCONS_00078442	lncRNA
XM_012101922.2	ZBTB44	mRNA	TCONS_00078442	lncRNA
XM_012101922.2	ZBTB44	mRNA	TCONS_00078442	lncRNA
XM_015103643.1	YES1	mRNA	XR_001041202.1	lncRNA
XM_015103643.1	YES1	mRNA	XR_001041202.1	lncRNA
XM_015103643.1	YES1	mRNA	XR_001041202.1	lncRNA
XM_015103643.1	YES1	mRNA	XR_001041202.1	lncRNA
XM_004012037.3	TDRD3	mRNA	XR_001041202.1	lncRNA
XM_004012037.3	TDRD3	mRNA	XR_001041202.1	lncRNA
XM_004012037.3	TDRD3	mRNA	XR_001041202.1	lncRNA
XM_004012037.3	TDRD3	mRNA	XR_001041202.1	lncRNA
XM_015103641.1	YES1	mRNA	XR_001041202.1	lncRNA
XM_015103641.1	YES1	mRNA	XR_001041202.1	lncRNA
XM_015103641.1	YES1	mRNA	XR_001041202.1	lncRNA
XM_015103641.1	YES1	mRNA	XR_001041202.1	lncRNA
XM_004004099.3	GOLM1	mRNA	XR_001041202.1	lncRNA
XM_004004099.3	GOLM1	mRNA	XR_001041202.1	lncRNA
XM_004004099.3	GOLM1	mRNA	XR_001041202.1	lncRNA
XM_004004099.3	GOLM1	mRNA	XR_001041202.1	lncRNA
XM_012115168.2	LOC1011236(	mRNA	XR_001041202.1	lncRNA
XM_012115168.2	LOC1011236(	mRNA	XR_001041202.1	lncRNA
XM_012115168.2	LOC1011236(	mRNA	XR_001041202.1	lncRNA
XM_012115168.2	LOC1011236(	mRNA	XR_001041202.1	lncRNA
XM_015105106.1	ATG4A	mRNA	XR_001041202.1	lncRNA
XM_015105106.1	ATG4A	mRNA	XR_001041202.1	lncRNA
XM_015105106.1	ATG4A	mRNA	XR_001041202.1	lncRNA
XM_015105106.1	ATG4A	mRNA	XR_001041202.1	lncRNA
XM_012103008.2	FAM45A	mRNA	XR_001435104.1	lncRNA
XM_012103008.2	FAM45A	mRNA	XR_001435104.1	lncRNA
XM_012103008.2	FAM45A	mRNA	XR_001435104.1	lncRNA
XM_012103008.2	FAM45A	mRNA	XR_001435104.1	lncRNA
XM_015103487.1	LOC1011023!	mRNA	XR_001435104.1	lncRNA
XM_015103487.1	LOC1011023!	mRNA	XR_001435104.1	lncRNA
XM_015103487.1	LOC1011023!	mRNA	XR_001435104.1	lncRNA
XM_015103487.1	LOC1011023!	mRNA	XR_001435104.1	lncRNA
XM_012106742.2	PQBP1	mRNA	XR_001436531.1	lncRNA
XM_012106742.2	PQBP1	mRNA	XR_001436531.1	lncRNA
XM_012106742.2	PQBP1	mRNA	XR_001436531.1	lncRNA
XM_012106742.2	PQBP1	mRNA	XR_001436531.1	lncRNA
XM_012099058.2	PPP2R5C	mRNA	XR_001435104.1	lncRNA
XM_012099058.2	PPP2R5C	mRNA	XR_001435104.1	lncRNA
XM_012099058.2	PPP2R5C	mRNA	XR_001435104.1	lncRNA
XM_012099058.2	PPP2R5C	mRNA	XR_001435104.1	lncRNA
XM_012170567.2	CCDC138	mRNA	XR_001436531.1	lncRNA
XM_012170567.2	CCDC138	mRNA	XR_001436531.1	lncRNA
XM_012170567.2	CCDC138	mRNA	XR_001436531.1	lncRNA
XM_012170567.2	CCDC138	mRNA	XR_001436531.1	lncRNA

XM_004014209.3	CUL2	mRNA	XR_001436531.1	lncRNA
XM_004014209.3	CUL2	mRNA	XR_001436531.1	lncRNA
XM_004014209.3	CUL2	mRNA	XR_001436531.1	lncRNA
XM_004014209.3	CUL2	mRNA	XR_001436531.1	lncRNA
XM_012184819.1	RNF219	mRNA	XR_001436609.1	lncRNA
XM_012184819.1	RNF219	mRNA	XR_001436609.1	lncRNA
XM_012184819.1	RNF219	mRNA	XR_001436609.1	lncRNA
XM_012184819.1	RNF219	mRNA	XR_001436609.1	lncRNA
XM_004006746.3	KIAA1551	mRNA	XR_001435104.1	lncRNA
XM_004006746.3	KIAA1551	mRNA	XR_001435104.1	lncRNA
XM_004006746.3	KIAA1551	mRNA	XR_001435104.1	lncRNA
XM_004006746.3	KIAA1551	mRNA	XR_001435104.1	lncRNA
XM_015091991.1	YIPF1	mRNA	XR_001436609.1	lncRNA
XM_015091991.1	YIPF1	mRNA	XR_001436609.1	lncRNA
XM_015091991.1	YIPF1	mRNA	XR_001436609.1	lncRNA
XM_015091991.1	YIPF1	mRNA	XR_001436609.1	lncRNA
XM_012190349.2	ZNF420	mRNA	XR_001436609.1	lncRNA
XM_012190349.2	ZNF420	mRNA	XR_001436609.1	lncRNA
XM_012190349.2	ZNF420	mRNA	XR_001436609.1	lncRNA
XM_012190349.2	ZNF420	mRNA	XR_001436609.1	lncRNA
XM_012103822.2	KIAA1468	mRNA	XR_001436609.1	lncRNA
XM_012103822.2	KIAA1468	mRNA	XR_001436609.1	lncRNA
XM_012103822.2	KIAA1468	mRNA	XR_001436609.1	lncRNA
XM_012103822.2	KIAA1468	mRNA	XR_001436609.1	lncRNA
XM_015097223.1	ZC3H14	mRNA	XR_001436609.1	lncRNA
XM_015097223.1	ZC3H14	mRNA	XR_001436609.1	lncRNA
XM_015097223.1	ZC3H14	mRNA	XR_001436609.1	lncRNA
XM_015097223.1	ZC3H14	mRNA	XR_001436609.1	lncRNA
XM_004016017.3	C15H11orf1	mRNA	XR_001435104.1	lncRNA
XM_004016017.3	C15H11orf1	mRNA	XR_001435104.1	lncRNA
XM_004016017.3	C15H11orf1	mRNA	XR_001435104.1	lncRNA
XM_004016017.3	C15H11orf1	mRNA	XR_001435104.1	lncRNA
XM_012182520.2	SLC35F1	mRNA	XR_001436609.1	lncRNA
XM_012182520.2	SLC35F1	mRNA	XR_001436609.1	lncRNA
XM_012182520.2	SLC35F1	mRNA	XR_001436609.1	lncRNA
XM_012182520.2	SLC35F1	mRNA	XR_001436609.1	lncRNA
XM_012184453.2	FNDC3A	mRNA	XR_001436609.1	lncRNA
XM_012184453.2	FNDC3A	mRNA	XR_001436609.1	lncRNA
XM_012184453.2	FNDC3A	mRNA	XR_001436609.1	lncRNA
XM_012184453.2	FNDC3A	mRNA	XR_001436609.1	lncRNA
XM_012179621.1	DNAJB14	mRNA	XR_001436609.1	lncRNA
XM_012179621.1	DNAJB14	mRNA	XR_001436609.1	lncRNA
XM_012179621.1	DNAJB14	mRNA	XR_001436609.1	lncRNA
XM_012179621.1	DNAJB14	mRNA	XR_001436609.1	lncRNA
XM_012099058.2	PPP2R5C	mRNA	XR_001436609.1	lncRNA
XM_012099058.2	PPP2R5C	mRNA	XR_001436609.1	lncRNA
XM_012099058.2	PPP2R5C	mRNA	XR_001436609.1	lncRNA
XM_012099058.2	PPP2R5C	mRNA	XR_001436609.1	lncRNA
XM_004001901.2	ERI3	mRNA	XR_001436609.1	lncRNA
XM_004001901.2	ERI3	mRNA	XR_001436609.1	lncRNA
XM_004001901.2	ERI3	mRNA	XR_001436609.1	lncRNA
XM_004001901.2	ERI3	mRNA	XR_001436609.1	lncRNA
XM_012105248.1	ASCC1	mRNA	XR_001436609.1	lncRNA
XM_012105248.1	ASCC1	mRNA	XR_001436609.1	lncRNA

XM_012105248.1	ASCC1	mRNA	XR_001436609.1	lncRNA
XM_012105248.1	ASCC1	mRNA	XR_001436609.1	lncRNA
XM_012106742.2	PQBP1	mRNA	XR_001436609.1	lncRNA
XM_012106742.2	PQBP1	mRNA	XR_001436609.1	lncRNA
XM_012106742.2	PQBP1	mRNA	XR_001436609.1	lncRNA
XM_012106742.2	PQBP1	mRNA	XR_001436609.1	lncRNA
XM_004016017.3	C15H11orf1	mRNA	XR_001436609.1	lncRNA
XM_004016017.3	C15H11orf1	mRNA	XR_001436609.1	lncRNA
XM_004016017.3	C15H11orf1	mRNA	XR_001436609.1	lncRNA
XM_004016017.3	C15H11orf1	mRNA	XR_001436609.1	lncRNA
XM_012187727.2	TRMT1L	mRNA	XR_001033284.2	lncRNA
XM_012187727.2	TRMT1L	mRNA	XR_001033284.2	lncRNA
XM_012187727.2	TRMT1L	mRNA	XR_001033284.2	lncRNA
XM_012187727.2	TRMT1L	mRNA	XR_001033284.2	lncRNA
XM_012177369.2	PRKAG2	mRNA	XR_001033284.2	lncRNA
XM_012177369.2	PRKAG2	mRNA	XR_001033284.2	lncRNA
XM_012177369.2	PRKAG2	mRNA	XR_001033284.2	lncRNA
XM_012177369.2	PRKAG2	mRNA	XR_001033284.2	lncRNA
XM_012175343.2	LRP6	mRNA	XR_001436099.1	lncRNA
XM_012175343.2	LRP6	mRNA	XR_001436099.1	lncRNA
XM_012175343.2	LRP6	mRNA	XR_001436099.1	lncRNA
XM_012175343.2	LRP6	mRNA	XR_001436099.1	lncRNA
XM_015093291.1	NIPA2	mRNA	XR_001436099.1	lncRNA
XM_015093291.1	NIPA2	mRNA	XR_001436099.1	lncRNA
XM_015093291.1	NIPA2	mRNA	XR_001436099.1	lncRNA
XM_015093291.1	NIPA2	mRNA	XR_001436099.1	lncRNA
XM_015097611.1	VPS13B	mRNA	TCONS_00052150	lncRNA
XM_015097611.1	VPS13B	mRNA	TCONS_00052150	lncRNA
XM_015097611.1	VPS13B	mRNA	TCONS_00052150	lncRNA
XM_015097611.1	VPS13B	mRNA	TCONS_00052150	lncRNA
XM_012122525.2	PRUNE2	mRNA	TCONS_00052150	lncRNA
XM_012122525.2	PRUNE2	mRNA	TCONS_00052150	lncRNA
XM_012122525.2	PRUNE2	mRNA	TCONS_00052150	lncRNA
XM_012122525.2	PRUNE2	mRNA	TCONS_00052150	lncRNA
XM_012101112.2	ABT1	mRNA	TCONS_00078442	lncRNA
XM_012101112.2	ABT1	mRNA	TCONS_00078442	lncRNA
XM_012101112.2	ABT1	mRNA	TCONS_00078442	lncRNA
XM_012101112.2	ABT1	mRNA	TCONS_00078442	lncRNA
XM_012100729.1	POLH	mRNA	XR_001435723.1	lncRNA
XM_012100729.1	POLH	mRNA	XR_001435723.1	lncRNA
XM_012100729.1	POLH	mRNA	XR_001435723.1	lncRNA
XM_012100729.1	POLH	mRNA	XR_001435723.1	lncRNA
XM_012096375.2	PSMB2	mRNA	XR_001435723.1	lncRNA
XM_012096375.2	PSMB2	mRNA	XR_001435723.1	lncRNA
XM_012096375.2	PSMB2	mRNA	XR_001435723.1	lncRNA
XM_012096375.2	PSMB2	mRNA	XR_001435723.1	lncRNA
XM_012179621.1	DNAJB14	mRNA	XR_001435726.1	lncRNA
XM_012179621.1	DNAJB14	mRNA	XR_001435726.1	lncRNA
XM_012179621.1	DNAJB14	mRNA	XR_001435726.1	lncRNA
XM_012179621.1	DNAJB14	mRNA	XR_001435726.1	lncRNA
XM_015097223.1	ZC3H14	mRNA	XR_001435726.1	lncRNA
XM_015097223.1	ZC3H14	mRNA	XR_001435726.1	lncRNA
XM_015097223.1	ZC3H14	mRNA	XR_001435726.1	lncRNA
XM_015097223.1	ZC3H14	mRNA	XR_001435726.1	lncRNA

XM_015097326.1	TBC1D32	mRNA	XR_001435726.1	lncRNA
XM_015097326.1	TBC1D32	mRNA	XR_001435726.1	lncRNA
XM_015097326.1	TBC1D32	mRNA	XR_001435726.1	lncRNA
XM_015097326.1	TBC1D32	mRNA	XR_001435726.1	lncRNA
XM_012170748.1	MIER1	mRNA	XR_001042381.2	lncRNA
XM_012170748.1	MIER1	mRNA	XR_001042381.2	lncRNA
XM_012170748.1	MIER1	mRNA	XR_001042381.2	lncRNA
XM_012170748.1	MIER1	mRNA	XR_001042381.2	lncRNA
XM_012175064.2	KIAA1551	mRNA	XR_001042381.2	lncRNA
XM_012175064.2	KIAA1551	mRNA	XR_001042381.2	lncRNA
XM_012175064.2	KIAA1551	mRNA	XR_001042381.2	lncRNA
XM_012175064.2	KIAA1551	mRNA	XR_001042381.2	lncRNA
XM_015099653.1	LOC10112144	mRNA	XR_001042381.2	lncRNA
XM_015099653.1	LOC10112144	mRNA	XR_001042381.2	lncRNA
XM_015099653.1	LOC10112144	mRNA	XR_001042381.2	lncRNA
XM_015099653.1	LOC10112144	mRNA	XR_001042381.2	lncRNA
XM_012179621.1	DNAJB14	mRNA	XR_001042381.2	lncRNA
XM_012179621.1	DNAJB14	mRNA	XR_001042381.2	lncRNA
XM_012179621.1	DNAJB14	mRNA	XR_001042381.2	lncRNA
XM_012179621.1	DNAJB14	mRNA	XR_001042381.2	lncRNA
XM_012184045.2	ZNF706	mRNA	XR_001042381.2	lncRNA
XM_012184045.2	ZNF706	mRNA	XR_001042381.2	lncRNA
XM_012184045.2	ZNF706	mRNA	XR_001042381.2	lncRNA
XM_012184045.2	ZNF706	mRNA	XR_001042381.2	lncRNA
XM_012184819.1	RNF219	mRNA	XR_001042381.2	lncRNA
XM_012184819.1	RNF219	mRNA	XR_001042381.2	lncRNA
XM_012184819.1	RNF219	mRNA	XR_001042381.2	lncRNA
XM_012184819.1	RNF219	mRNA	XR_001042381.2	lncRNA
XM_012186398.2	DUS1L	mRNA	XR_001042381.2	lncRNA
XM_012186398.2	DUS1L	mRNA	XR_001042381.2	lncRNA
XM_012186398.2	DUS1L	mRNA	XR_001042381.2	lncRNA
XM_012186398.2	DUS1L	mRNA	XR_001042381.2	lncRNA
XM_015102413.1	RBM6	mRNA	XR_001435726.1	lncRNA
XM_015102413.1	RBM6	mRNA	XR_001435726.1	lncRNA
XM_015102413.1	RBM6	mRNA	XR_001435726.1	lncRNA
XM_015102413.1	RBM6	mRNA	XR_001435726.1	lncRNA
XM_004008822.3	FAM13B	mRNA	XR_001042381.2	lncRNA
XM_004008822.3	FAM13B	mRNA	XR_001042381.2	lncRNA
XM_004008822.3	FAM13B	mRNA	XR_001042381.2	lncRNA
XM_004008822.3	FAM13B	mRNA	XR_001042381.2	lncRNA
XM_012104222.2	DCUN1D3	mRNA	TCONS_00071561	lncRNA
XM_012104222.2	DCUN1D3	mRNA	TCONS_00071561	lncRNA
XM_012104222.2	DCUN1D3	mRNA	TCONS_00071561	lncRNA
XM_012104222.2	DCUN1D3	mRNA	TCONS_00071561	lncRNA
XM_012188301.2	NEBL	mRNA	TCONS_00071561	lncRNA
XM_012188301.2	NEBL	mRNA	TCONS_00071561	lncRNA
XM_012188301.2	NEBL	mRNA	TCONS_00071561	lncRNA
XM_012188301.2	NEBL	mRNA	TCONS_00071561	lncRNA
XM_012188226.2	CUL2	mRNA	TCONS_00071561	lncRNA
XM_012188226.2	CUL2	mRNA	TCONS_00071561	lncRNA
XM_012188226.2	CUL2	mRNA	TCONS_00071561	lncRNA
XM_012188226.2	CUL2	mRNA	TCONS_00071561	lncRNA
XM_012182988.2	REPS1	mRNA	TCONS_00071561	lncRNA
XM_012182988.2	REPS1	mRNA	TCONS_00071561	lncRNA

XM_012182988.2	REPS1	mRNA	TCONS_00071561	lncRNA
XM_012182988.2	REPS1	mRNA	TCONS_00071561	lncRNA
XM_012179421.1	METTL14	mRNA	TCONS_00071561	lncRNA
XM_012179421.1	METTL14	mRNA	TCONS_00071561	lncRNA
XM_012179421.1	METTL14	mRNA	TCONS_00071561	lncRNA
XM_012179421.1	METTL14	mRNA	TCONS_00071561	lncRNA
XM_012181536.2	FAM214A	mRNA	XR_001021689.2	lncRNA
XM_012181536.2	FAM214A	mRNA	XR_001021689.2	lncRNA
XM_012181536.2	FAM214A	mRNA	XR_001021689.2	lncRNA
XM_012181536.2	FAM214A	mRNA	XR_001021689.2	lncRNA
XM_015102528.1	FLOT1	mRNA	XR_001021689.2	lncRNA
XM_015102528.1	FLOT1	mRNA	XR_001021689.2	lncRNA
XM_015102528.1	FLOT1	mRNA	XR_001021689.2	lncRNA
XM_015102528.1	FLOT1	mRNA	XR_001021689.2	lncRNA
XM_012188664.2	RASSF2	mRNA	XR_001021689.2	lncRNA
XM_012188664.2	RASSF2	mRNA	XR_001021689.2	lncRNA
XM_012188664.2	RASSF2	mRNA	XR_001021689.2	lncRNA
XM_012188664.2	RASSF2	mRNA	XR_001021689.2	lncRNA
XM_012190083.2	ZMYM6	mRNA	XR_001021689.2	lncRNA
XM_012190083.2	ZMYM6	mRNA	XR_001021689.2	lncRNA
XM_012190083.2	ZMYM6	mRNA	XR_001021689.2	lncRNA
XM_012190083.2	ZMYM6	mRNA	XR_001021689.2	lncRNA
XM_012094768.2	ZMYM1	mRNA	XR_001021689.2	lncRNA
XM_012094768.2	ZMYM1	mRNA	XR_001021689.2	lncRNA
XM_012094768.2	ZMYM1	mRNA	XR_001021689.2	lncRNA
XM_012094768.2	ZMYM1	mRNA	XR_001021689.2	lncRNA
XM_012133854.2	DYNC1I2	mRNA	XR_001021689.2	lncRNA
XM_012133854.2	DYNC1I2	mRNA	XR_001021689.2	lncRNA
XM_012133854.2	DYNC1I2	mRNA	XR_001021689.2	lncRNA
XM_012133854.2	DYNC1I2	mRNA	XR_001021689.2	lncRNA
XM_015104027.1	PDPK1	mRNA	XR_001021689.2	lncRNA
XM_015104027.1	PDPK1	mRNA	XR_001021689.2	lncRNA
XM_015104027.1	PDPK1	mRNA	XR_001021689.2	lncRNA
XM_015104027.1	PDPK1	mRNA	XR_001021689.2	lncRNA
XM_004017788.3	ZFANL3	mRNA	TCONS_00072289	lncRNA
XM_004017788.3	ZFANL3	mRNA	TCONS_00072289	lncRNA
XM_004017788.3	ZFANL3	mRNA	TCONS_00072289	lncRNA
XM_004017788.3	ZFANL3	mRNA	TCONS_00072289	lncRNA
XM_012177175.2	CALD1	mRNA	TCONS_00072289	lncRNA
XM_012177175.2	CALD1	mRNA	TCONS_00072289	lncRNA
XM_012177175.2	CALD1	mRNA	TCONS_00072289	lncRNA
XM_012177175.2	CALD1	mRNA	TCONS_00072289	lncRNA
XM_012142889.2	ICA1L	mRNA	TCONS_00072289	lncRNA
XM_012142889.2	ICA1L	mRNA	TCONS_00072289	lncRNA
XM_012142889.2	ICA1L	mRNA	TCONS_00072289	lncRNA
XM_012142889.2	ICA1L	mRNA	TCONS_00072289	lncRNA
XM_012177174.2	CALD1	mRNA	TCONS_00072289	lncRNA
XM_012177174.2	CALD1	mRNA	TCONS_00072289	lncRNA
XM_012177174.2	CALD1	mRNA	TCONS_00072289	lncRNA
XM_012177174.2	CALD1	mRNA	TCONS_00072289	lncRNA
XM_012175265.1	PLEKHA5	mRNA	XR_001039281.1	lncRNA
XM_012175265.1	PLEKHA5	mRNA	XR_001039281.1	lncRNA
XM_012175265.1	PLEKHA5	mRNA	XR_001039281.1	lncRNA
XM_012175265.1	PLEKHA5	mRNA	XR_001039281.1	lncRNA

XM_012095858.2	ZNF214	mRNA	TCONS_00029398	lncRNA
XM_012095858.2	ZNF214	mRNA	TCONS_00029398	lncRNA
XM_012095858.2	ZNF214	mRNA	TCONS_00029398	lncRNA
XM_012095858.2	ZNF214	mRNA	TCONS_00029398	lncRNA
XM_012180800.1	TIPIN	mRNA	TCONS_00029398	lncRNA
XM_012180800.1	TIPIN	mRNA	TCONS_00029398	lncRNA
XM_012180800.1	TIPIN	mRNA	TCONS_00029398	lncRNA
XM_012180800.1	TIPIN	mRNA	TCONS_00029398	lncRNA
XM_004022347.2	TMEM255A	mRNA	TCONS_00029398	lncRNA
XM_004022347.2	TMEM255A	mRNA	TCONS_00029398	lncRNA
XM_004022347.2	TMEM255A	mRNA	TCONS_00029398	lncRNA
XM_004022347.2	TMEM255A	mRNA	TCONS_00029398	lncRNA
XM_015104131.1	TNRC6A	mRNA	TCONS_00029398	lncRNA
XM_015104131.1	TNRC6A	mRNA	TCONS_00029398	lncRNA
XM_015104131.1	TNRC6A	mRNA	TCONS_00029398	lncRNA
XM_015104131.1	TNRC6A	mRNA	TCONS_00029398	lncRNA
XM_015093291.1	NIPA2	mRNA	XR_001041209.2	lncRNA
XM_015093291.1	NIPA2	mRNA	XR_001041209.2	lncRNA
XM_015093291.1	NIPA2	mRNA	XR_001041209.2	lncRNA
XM_015093291.1	NIPA2	mRNA	XR_001041209.2	lncRNA
XM_015092793.1	LOC10699053	mRNA	XR_001041211.2	lncRNA
XM_015092793.1	LOC10699053	mRNA	XR_001041211.2	lncRNA
XM_015092793.1	LOC10699053	mRNA	XR_001041211.2	lncRNA
XM_015092793.1	LOC10699053	mRNA	XR_001041211.2	lncRNA
XM_015103641.1	YES1	mRNA	XR_001041211.2	lncRNA
XM_015103641.1	YES1	mRNA	XR_001041211.2	lncRNA
XM_015103641.1	YES1	mRNA	XR_001041211.2	lncRNA
XM_015103641.1	YES1	mRNA	XR_001041211.2	lncRNA
XM_012180229.1	CCNG2	mRNA	TCONS_00029399	lncRNA
XM_012180229.1	CCNG2	mRNA	TCONS_00029399	lncRNA
XM_012180229.1	CCNG2	mRNA	TCONS_00029399	lncRNA
XM_012180229.1	CCNG2	mRNA	TCONS_00029399	lncRNA
XM_015103643.1	YES1	mRNA	XR_001041211.2	lncRNA
XM_015103643.1	YES1	mRNA	XR_001041211.2	lncRNA
XM_015103643.1	YES1	mRNA	XR_001041211.2	lncRNA
XM_015103643.1	YES1	mRNA	XR_001041211.2	lncRNA
XM_012134516.1	PPIG	mRNA	XR_001041209.2	lncRNA
XM_012134516.1	PPIG	mRNA	XR_001041209.2	lncRNA
XM_012134516.1	PPIG	mRNA	XR_001041209.2	lncRNA
XM_012134516.1	PPIG	mRNA	XR_001041209.2	lncRNA
XM_012100861.1	FBXO9	mRNA	TCONS_00029399	lncRNA
XM_012100861.1	FBXO9	mRNA	TCONS_00029399	lncRNA
XM_012100861.1	FBXO9	mRNA	TCONS_00029399	lncRNA
XM_012100861.1	FBXO9	mRNA	TCONS_00029399	lncRNA
XM_004004723.3	SPOPL	mRNA	XR_001041209.2	lncRNA
XM_004004723.3	SPOPL	mRNA	XR_001041209.2	lncRNA
XM_004004723.3	SPOPL	mRNA	XR_001041209.2	lncRNA
XM_004004723.3	SPOPL	mRNA	XR_001041209.2	lncRNA
XM_004012898.3	KRT34	mRNA	XR_001041209.2	lncRNA
XM_004012898.3	KRT34	mRNA	XR_001041209.2	lncRNA
XM_004012898.3	KRT34	mRNA	XR_001041209.2	lncRNA
XM_004012898.3	KRT34	mRNA	XR_001041209.2	lncRNA
XM_012166941.1	BRE	mRNA	XR_001041209.2	lncRNA
XM_012166941.1	BRE	mRNA	XR_001041209.2	lncRNA

XM_012166941.1	BRE	mRNA	XR_001041209.2	lncRNA
XM_012166941.1	BRE	mRNA	XR_001041209.2	lncRNA
XM_012095522.2	JAML	mRNA	XR_001041211.2	lncRNA
XM_012095522.2	JAML	mRNA	XR_001041211.2	lncRNA
XM_012095522.2	JAML	mRNA	XR_001041211.2	lncRNA
XM_012095522.2	JAML	mRNA	XR_001041211.2	lncRNA
XM_004004717.2	GTDC1	mRNA	TCONS_00078442	lncRNA
XM_004004717.2	GTDC1	mRNA	TCONS_00078442	lncRNA
XM_004004717.2	GTDC1	mRNA	TCONS_00078442	lncRNA
XM_004004717.2	GTDC1	mRNA	TCONS_00078442	lncRNA
XM_012144336.2	PIKFYVE	mRNA	TCONS_00078442	lncRNA
XM_012144336.2	PIKFYVE	mRNA	TCONS_00078442	lncRNA
XM_012144336.2	PIKFYVE	mRNA	TCONS_00078442	lncRNA
XM_012144336.2	PIKFYVE	mRNA	TCONS_00078442	lncRNA
XM_012189777.1	CHD9	mRNA	TCONS_00078442	lncRNA
XM_012189777.1	CHD9	mRNA	TCONS_00078442	lncRNA
XM_012189777.1	CHD9	mRNA	TCONS_00078442	lncRNA
XM_012189777.1	CHD9	mRNA	TCONS_00078442	lncRNA
XM_015095780.1	DNAJB1	mRNA	TCONS_00078442	lncRNA
XM_015095780.1	DNAJB1	mRNA	TCONS_00078442	lncRNA
XM_015095780.1	DNAJB1	mRNA	TCONS_00078442	lncRNA
XM_015095780.1	DNAJB1	mRNA	TCONS_00078442	lncRNA
XM_015092531.1	DCUN1D1	mRNA	XR_001041202.1	lncRNA
XM_015092531.1	DCUN1D1	mRNA	XR_001041202.1	lncRNA
XM_015092531.1	DCUN1D1	mRNA	XR_001041202.1	lncRNA
XM_015092531.1	DCUN1D1	mRNA	XR_001041202.1	lncRNA
XM_012183298.1	FAM120B	mRNA	XR_001039281.1	lncRNA
XM_012183298.1	FAM120B	mRNA	XR_001039281.1	lncRNA
XM_012183298.1	FAM120B	mRNA	XR_001039281.1	lncRNA
XM_012183298.1	FAM120B	mRNA	XR_001039281.1	lncRNA
XM_012179952.2	FRYL	mRNA	TCONS_00072289	lncRNA
XM_012179952.2	FRYL	mRNA	TCONS_00072289	lncRNA
XM_012179952.2	FRYL	mRNA	TCONS_00072289	lncRNA
XM_012179952.2	FRYL	mRNA	TCONS_00072289	lncRNA
XM_012099916.2	PRKCD	mRNA	XR_001039281.1	lncRNA
XM_012099916.2	PRKCD	mRNA	XR_001039281.1	lncRNA
XM_012099916.2	PRKCD	mRNA	XR_001039281.1	lncRNA
XM_012099916.2	PRKCD	mRNA	XR_001039281.1	lncRNA
XM_015100441.1	CHD9	mRNA	TCONS_00078442	lncRNA
XM_015100441.1	CHD9	mRNA	TCONS_00078442	lncRNA
XM_015100441.1	CHD9	mRNA	TCONS_00078442	lncRNA
XM_015100441.1	CHD9	mRNA	TCONS_00078442	lncRNA
XM_012095522.2	JAML	mRNA	XR_001434081.1	lncRNA
XM_012095522.2	JAML	mRNA	XR_001434081.1	lncRNA
XM_012095522.2	JAML	mRNA	XR_001434081.1	lncRNA
XM_012095522.2	JAML	mRNA	XR_001434081.1	lncRNA
XM_004004723.3	SPOPL	mRNA	XR_001434081.1	lncRNA
XM_004004723.3	SPOPL	mRNA	XR_001434081.1	lncRNA
XM_004004723.3	SPOPL	mRNA	XR_001434081.1	lncRNA
XM_004004723.3	SPOPL	mRNA	XR_001434081.1	lncRNA
XM_012176399.2	ICA1	mRNA	XR_001434081.1	lncRNA
XM_012176399.2	ICA1	mRNA	XR_001434081.1	lncRNA
XM_012176399.2	ICA1	mRNA	XR_001434081.1	lncRNA
XM_012176399.2	ICA1	mRNA	XR_001434081.1	lncRNA

XM_015096569.1	CDKL2	mRNA	XR_001434081.1	lncRNA
XM_015096569.1	CDKL2	mRNA	XR_001434081.1	lncRNA
XM_015096569.1	CDKL2	mRNA	XR_001434081.1	lncRNA
XM_015096569.1	CDKL2	mRNA	XR_001434081.1	lncRNA
XM_012119517.2	SORBS3	mRNA	XR_001434081.1	lncRNA
XM_012119517.2	SORBS3	mRNA	XR_001434081.1	lncRNA
XM_012119517.2	SORBS3	mRNA	XR_001434081.1	lncRNA
XM_012119517.2	SORBS3	mRNA	XR_001434081.1	lncRNA
XM_012182825.2	SNX14	mRNA	XR_001434081.1	lncRNA
XM_012182825.2	SNX14	mRNA	XR_001434081.1	lncRNA
XM_012182825.2	SNX14	mRNA	XR_001434081.1	lncRNA
XM_012182825.2	SNX14	mRNA	XR_001434081.1	lncRNA
XM_004008647.3	RAPGEF6	mRNA	XR_001021689.2	lncRNA
XM_004008647.3	RAPGEF6	mRNA	XR_001021689.2	lncRNA
XM_004008647.3	RAPGEF6	mRNA	XR_001021689.2	lncRNA
XM_004008647.3	RAPGEF6	mRNA	XR_001021689.2	lncRNA
XM_012174754.2	RNF41	mRNA	XR_001021689.2	lncRNA
XM_012174754.2	RNF41	mRNA	XR_001021689.2	lncRNA
XM_012174754.2	RNF41	mRNA	XR_001021689.2	lncRNA
XM_012174754.2	RNF41	mRNA	XR_001021689.2	lncRNA
XM_015096551.1	MOB1B	mRNA	TCONS_00071561	lncRNA
XM_015096551.1	MOB1B	mRNA	TCONS_00071561	lncRNA
XM_015096551.1	MOB1B	mRNA	TCONS_00071561	lncRNA
XM_015096551.1	MOB1B	mRNA	TCONS_00071561	lncRNA
XM_012173983.2	PPP1R12A	mRNA	XR_001021689.2	lncRNA
XM_012173983.2	PPP1R12A	mRNA	XR_001021689.2	lncRNA
XM_012173983.2	PPP1R12A	mRNA	XR_001021689.2	lncRNA
XM_012173983.2	PPP1R12A	mRNA	XR_001021689.2	lncRNA
XM_015096551.1	MOB1B	mRNA	XR_001021689.2	lncRNA
XM_015096551.1	MOB1B	mRNA	XR_001021689.2	lncRNA
XM_015096551.1	MOB1B	mRNA	XR_001021689.2	lncRNA
XM_015096551.1	MOB1B	mRNA	XR_001021689.2	lncRNA
XM_012189279.2	DNTTIP1	mRNA	XR_001021689.2	lncRNA
XM_012189279.2	DNTTIP1	mRNA	XR_001021689.2	lncRNA
XM_012189279.2	DNTTIP1	mRNA	XR_001021689.2	lncRNA
XM_012189279.2	DNTTIP1	mRNA	XR_001021689.2	lncRNA
XM_012174787.1	LOC1011137[illegible]	mRNA	XR_001021689.2	lncRNA
XM_012174787.1	LOC1011137[illegible]	mRNA	XR_001021689.2	lncRNA
XM_012174787.1	LOC1011137[illegible]	mRNA	XR_001021689.2	lncRNA
XM_012174787.1	LOC1011137[illegible]	mRNA	XR_001021689.2	lncRNA
XM_012182446.2	TTK	mRNA	TCONS_00071561	lncRNA
XM_012182446.2	TTK	mRNA	TCONS_00071561	lncRNA
XM_012182446.2	TTK	mRNA	TCONS_00071561	lncRNA
XM_012182446.2	TTK	mRNA	TCONS_00071561	lncRNA
XM_012190105.1	ANKRL17	mRNA	XR_001021689.2	lncRNA
XM_012190105.1	ANKRL17	mRNA	XR_001021689.2	lncRNA
XM_012190105.1	ANKRL17	mRNA	XR_001021689.2	lncRNA
XM_012190105.1	ANKRL17	mRNA	XR_001021689.2	lncRNA
XM_012133845.1	DYNC1I2	mRNA	XR_001021689.2	lncRNA
XM_012133845.1	DYNC1I2	mRNA	XR_001021689.2	lncRNA
XM_012133845.1	DYNC1I2	mRNA	XR_001021689.2	lncRNA
XM_012133845.1	DYNC1I2	mRNA	XR_001021689.2	lncRNA
XM_012118246.2	DCAF12	mRNA	XR_001021689.2	lncRNA
XM_012118246.2	DCAF12	mRNA	XR_001021689.2	lncRNA

XM_012118246.2	DCAF12	mRNA	XR_001021689.2	lncRNA
XM_012118246.2	DCAF12	mRNA	XR_001021689.2	lncRNA
XM_012103723.1	POLI	mRNA	TCONS_00029398	lncRNA
XM_012103723.1	POLI	mRNA	TCONS_00029398	lncRNA
XM_012103723.1	POLI	mRNA	TCONS_00029398	lncRNA
XM_012103723.1	POLI	mRNA	TCONS_00029398	lncRNA
XM_012182301.2	LRRFIP1	mRNA	TCONS_00029398	lncRNA
XM_012182301.2	LRRFIP1	mRNA	TCONS_00029398	lncRNA
XM_012182301.2	LRRFIP1	mRNA	TCONS_00029398	lncRNA
XM_012182301.2	LRRFIP1	mRNA	TCONS_00029398	lncRNA
XM_012095771.2	CTR9	mRNA	TCONS_00029398	lncRNA
XM_012095771.2	CTR9	mRNA	TCONS_00029398	lncRNA
XM_012095771.2	CTR9	mRNA	TCONS_00029398	lncRNA
XM_012095771.2	CTR9	mRNA	TCONS_00029398	lncRNA
XM_012190140.2	LSM14A	mRNA	XR_001436099.1	lncRNA
XM_012190140.2	LSM14A	mRNA	XR_001436099.1	lncRNA
XM_012190140.2	LSM14A	mRNA	XR_001436099.1	lncRNA
XM_012190140.2	LSM14A	mRNA	XR_001436099.1	lncRNA
XM_015093101.1	PPP3CC	mRNA	XR_001033284.2	lncRNA
XM_015093101.1	PPP3CC	mRNA	XR_001033284.2	lncRNA
XM_015093101.1	PPP3CC	mRNA	XR_001033284.2	lncRNA
XM_015093101.1	PPP3CC	mRNA	XR_001033284.2	lncRNA
XM_012106112.2	ARAF	mRNA	XR_001033284.2	lncRNA
XM_012106112.2	ARAF	mRNA	XR_001033284.2	lncRNA
XM_012106112.2	ARAF	mRNA	XR_001033284.2	lncRNA
XM_012106112.2	ARAF	mRNA	XR_001033284.2	lncRNA
XM_015099863.1	DSN1	mRNA	XR_001033284.2	lncRNA
XM_015099863.1	DSN1	mRNA	XR_001033284.2	lncRNA
XM_015099863.1	DSN1	mRNA	XR_001033284.2	lncRNA
XM_015099863.1	DSN1	mRNA	XR_001033284.2	lncRNA
XM_012173780.2	REV1	mRNA	XR_001039281.1	lncRNA
XM_012173780.2	REV1	mRNA	XR_001039281.1	lncRNA
XM_012173780.2	REV1	mRNA	XR_001039281.1	lncRNA
XM_012173780.2	REV1	mRNA	XR_001039281.1	lncRNA
XM_015097611.1	VPS13B	mRNA	TCONS_00072289	lncRNA
XM_015097611.1	VPS13B	mRNA	TCONS_00072289	lncRNA
XM_015097611.1	VPS13B	mRNA	TCONS_00072289	lncRNA
XM_015097611.1	VPS13B	mRNA	TCONS_00072289	lncRNA
XM_012098436.1	KLHL25	mRNA	XR_001039281.1	lncRNA
XM_012098436.1	KLHL25	mRNA	XR_001039281.1	lncRNA
XM_012098436.1	KLHL25	mRNA	XR_001039281.1	lncRNA
XM_012098436.1	KLHL25	mRNA	XR_001039281.1	lncRNA
XM_012177369.2	PRKAG2	mRNA	XR_001021939.1	lncRNA
XM_012177369.2	PRKAG2	mRNA	XR_001021939.1	lncRNA
XM_012177369.2	PRKAG2	mRNA	XR_001021939.1	lncRNA
XM_012177369.2	PRKAG2	mRNA	XR_001021939.1	lncRNA
XM_012099723.2	PSML3	mRNA	TCONS_00078442	lncRNA
XM_012099723.2	PSML3	mRNA	TCONS_00078442	lncRNA
XM_012099723.2	PSML3	mRNA	TCONS_00078442	lncRNA
XM_012124491.2	AK3	mRNA	TCONS_00078442	lncRNA
XM_012124491.2	AK3	mRNA	TCONS_00078442	lncRNA
XM_012124491.2	AK3	mRNA	TCONS_00078442	lncRNA
XM_012166941.1	BRE	mRNA	XR_001436609.1	lncRNA
XM_012166941.1	BRE	mRNA	XR_001436609.1	lncRNA

XM_012166941.1	BRE	mRNA	XR_001436609.1	lncRNA
XM_012166941.1	BRE	mRNA	XR_001436609.1	lncRNA
XM_015103753.1	MBD1	mRNA	XR_001436609.1	lncRNA
XM_015103753.1	MBD1	mRNA	XR_001436609.1	lncRNA
XM_015103753.1	MBD1	mRNA	XR_001436609.1	lncRNA
XM_015103753.1	MBD1	mRNA	XR_001436609.1	lncRNA
XM_012181771.2	PCNXL4	mRNA	XR_001436609.1	lncRNA
XM_012181771.2	PCNXL4	mRNA	XR_001436609.1	lncRNA
XM_012181771.2	PCNXL4	mRNA	XR_001436609.1	lncRNA
XM_012181771.2	PCNXL4	mRNA	XR_001436609.1	lncRNA
XM_012115168.2	LOC1011236(	mRNA	XR_001436609.1	lncRNA
XM_012115168.2	LOC1011236(	mRNA	XR_001436609.1	lncRNA
XM_012115168.2	LOC1011236(	mRNA	XR_001436609.1	lncRNA
XM_012115168.2	LOC1011236(	mRNA	XR_001436609.1	lncRNA
XM_015092793.1	LOC10699053	mRNA	XR_001436609.1	lncRNA
XM_015092793.1	LOC10699053	mRNA	XR_001436609.1	lncRNA
XM_015092793.1	LOC10699053	mRNA	XR_001436609.1	lncRNA
XM_015092793.1	LOC10699053	mRNA	XR_001436609.1	lncRNA
XM_012095725.2	BTBD10	mRNA	XR_001436609.1	lncRNA
XM_012095725.2	BTBD10	mRNA	XR_001436609.1	lncRNA
XM_012095725.2	BTBD10	mRNA	XR_001436609.1	lncRNA
XM_012095725.2	BTBD10	mRNA	XR_001436609.1	lncRNA
XM_012177369.2	PRKAG2	mRNA	XR_001435104.1	lncRNA
XM_012177369.2	PRKAG2	mRNA	XR_001435104.1	lncRNA
XM_012177369.2	PRKAG2	mRNA	XR_001435104.1	lncRNA
XM_012177369.2	PRKAG2	mRNA	XR_001435104.1	lncRNA
XM_012095522.2	JAML	mRNA	XR_001436609.1	lncRNA
XM_012095522.2	JAML	mRNA	XR_001436609.1	lncRNA
XM_012095522.2	JAML	mRNA	XR_001436609.1	lncRNA
XM_012095522.2	JAML	mRNA	XR_001436609.1	lncRNA
XM_012119517.2	SORBS3	mRNA	XR_001435104.1	lncRNA
XM_012119517.2	SORBS3	mRNA	XR_001435104.1	lncRNA
XM_012119517.2	SORBS3	mRNA	XR_001435104.1	lncRNA
XM_012119517.2	SORBS3	mRNA	XR_001435104.1	lncRNA
XM_012182825.2	SNX14	mRNA	XR_001436609.1	lncRNA
XM_012182825.2	SNX14	mRNA	XR_001436609.1	lncRNA
XM_012182825.2	SNX14	mRNA	XR_001436609.1	lncRNA
XM_012182825.2	SNX14	mRNA	XR_001436609.1	lncRNA
XM_015103753.1	MBD1	mRNA	XR_001436531.1	lncRNA
XM_015103753.1	MBD1	mRNA	XR_001436531.1	lncRNA
XM_015103753.1	MBD1	mRNA	XR_001436531.1	lncRNA
XM_015103753.1	MBD1	mRNA	XR_001436531.1	lncRNA
XM_012105800.2	PCM1	mRNA	XR_001041202.1	lncRNA
XM_012105800.2	PCM1	mRNA	XR_001041202.1	lncRNA
XM_012105800.2	PCM1	mRNA	XR_001041202.1	lncRNA
XM_012105800.2	PCM1	mRNA	XR_001041202.1	lncRNA
XM_012178308.2	FAM193B	mRNA	XR_001041202.1	lncRNA
XM_012178308.2	FAM193B	mRNA	XR_001041202.1	lncRNA
XM_012178308.2	FAM193B	mRNA	XR_001041202.1	lncRNA
XM_012178308.2	FAM193B	mRNA	XR_001041202.1	lncRNA
XM_012133854.2	DYNC1I2	mRNA	XR_001041209.2	lncRNA
XM_012133854.2	DYNC1I2	mRNA	XR_001041209.2	lncRNA
XM_012133854.2	DYNC1I2	mRNA	XR_001041209.2	lncRNA
XM_012133854.2	DYNC1I2	mRNA	XR_001041209.2	lncRNA

XM_012182988.2	REPS1	mRNA	XR_001041209.2	lncRNA
XM_012182988.2	REPS1	mRNA	XR_001041209.2	lncRNA
XM_012182988.2	REPS1	mRNA	XR_001041209.2	lncRNA
XM_012182988.2	REPS1	mRNA	XR_001041209.2	lncRNA
XM_015093101.1	PPP3CC	mRNA	TCONS_00029399	lncRNA
XM_015093101.1	PPP3CC	mRNA	TCONS_00029399	lncRNA
XM_015093101.1	PPP3CC	mRNA	TCONS_00029399	lncRNA
XM_015093101.1	PPP3CC	mRNA	TCONS_00029399	lncRNA
XM_012187630.2	SOAT1	mRNA	XR_001041209.2	lncRNA
XM_012187630.2	SOAT1	mRNA	XR_001041209.2	lncRNA
XM_012187630.2	SOAT1	mRNA	XR_001041209.2	lncRNA
XM_012187630.2	SOAT1	mRNA	XR_001041209.2	lncRNA
XM_012106024.2	HOOK3	mRNA	XR_001041211.2	lncRNA
XM_012106024.2	HOOK3	mRNA	XR_001041211.2	lncRNA
XM_012106024.2	HOOK3	mRNA	XR_001041211.2	lncRNA
XM_012106024.2	HOOK3	mRNA	XR_001041211.2	lncRNA
XM_012124912.1	KIAA2026	mRNA	XR_001041209.2	lncRNA
XM_012124912.1	KIAA2026	mRNA	XR_001041209.2	lncRNA
XM_012124912.1	KIAA2026	mRNA	XR_001041209.2	lncRNA
XM_012124912.1	KIAA2026	mRNA	XR_001041209.2	lncRNA
XM_015104027.1	PDPK1	mRNA	XR_001041209.2	lncRNA
XM_015104027.1	PDPK1	mRNA	XR_001041209.2	lncRNA
XM_015104027.1	PDPK1	mRNA	XR_001041209.2	lncRNA
XM_015104027.1	PDPK1	mRNA	XR_001041209.2	lncRNA
XM_012184485.2	RNASEH2B	mRNA	TCONS_00029399	lncRNA
XM_012184485.2	RNASEH2B	mRNA	TCONS_00029399	lncRNA
XM_012184485.2	RNASEH2B	mRNA	TCONS_00029399	lncRNA
XM_012184485.2	RNASEH2B	mRNA	TCONS_00029399	lncRNA
XM_012133854.2	DYNC1I2	mRNA	TCONS_00029399	lncRNA
XM_012133854.2	DYNC1I2	mRNA	TCONS_00029399	lncRNA
XM_012133854.2	DYNC1I2	mRNA	TCONS_00029399	lncRNA
XM_012133854.2	DYNC1I2	mRNA	TCONS_00029399	lncRNA
XM_012095418.2	ZMYM4	mRNA	TCONS_00029399	lncRNA
XM_012095418.2	ZMYM4	mRNA	TCONS_00029399	lncRNA
XM_012095418.2	ZMYM4	mRNA	TCONS_00029399	lncRNA
XM_012095418.2	ZMYM4	mRNA	TCONS_00029399	lncRNA
XM_015092531.1	DCUN1D1	mRNA	XR_001041211.2	lncRNA
XM_015092531.1	DCUN1D1	mRNA	XR_001041211.2	lncRNA
XM_015092531.1	DCUN1D1	mRNA	XR_001041211.2	lncRNA
XM_015092531.1	DCUN1D1	mRNA	XR_001041211.2	lncRNA
XM_012187919.2	LOC10561661	mRNA	XR_001041209.2	lncRNA
XM_012187919.2	LOC10561661	mRNA	XR_001041209.2	lncRNA
XM_012187919.2	LOC10561661	mRNA	XR_001041209.2	lncRNA
XM_012187919.2	LOC10561661	mRNA	XR_001041209.2	lncRNA
XM_012095387.2	ELMOD1	mRNA	TCONS_00029399	lncRNA
XM_012095387.2	ELMOD1	mRNA	TCONS_00029399	lncRNA
XM_012095387.2	ELMOD1	mRNA	TCONS_00029399	lncRNA
XM_012095387.2	ELMOD1	mRNA	TCONS_00029399	lncRNA
XM_015102310.1	SETD5	mRNA	XR_001039281.1	lncRNA
XM_015102310.1	SETD5	mRNA	XR_001039281.1	lncRNA
XM_015102310.1	SETD5	mRNA	XR_001039281.1	lncRNA
XM_015102310.1	SETD5	mRNA	XR_001039281.1	lncRNA
XM_015102269.1	NKTR	mRNA	XR_001039281.1	lncRNA
XM_015102269.1	NKTR	mRNA	XR_001039281.1	lncRNA

XM_015102269.1	NKTR	mRNA	XR_001039281.1	lncRNA
XM_015102269.1	NKTR	mRNA	XR_001039281.1	lncRNA
XM_015095780.1	DNAJB1	mRNA	TCONS_00072289	lncRNA
XM_015095780.1	DNAJB1	mRNA	TCONS_00072289	lncRNA
XM_015095780.1	DNAJB1	mRNA	TCONS_00072289	lncRNA
XM_015095780.1	DNAJB1	mRNA	TCONS_00072289	lncRNA
XM_012181720.2	ARID4A	mRNA	TCONS_00071561	lncRNA
XM_012181720.2	ARID4A	mRNA	TCONS_00071561	lncRNA
XM_012181720.2	ARID4A	mRNA	TCONS_00071561	lncRNA
XM_012181720.2	ARID4A	mRNA	TCONS_00071561	lncRNA
XM_015100095.1	LOC10111352	mRNA	XR_001021689.2	lncRNA
XM_015100095.1	LOC10111352	mRNA	XR_001021689.2	lncRNA
XM_015100095.1	LOC10111352	mRNA	XR_001021689.2	lncRNA
XM_015100095.1	LOC10111352	mRNA	XR_001021689.2	lncRNA
XM_012134593.2	FASTKD1	mRNA	XR_001021689.2	lncRNA
XM_012134593.2	FASTKD1	mRNA	XR_001021689.2	lncRNA
XM_012134593.2	FASTKD1	mRNA	XR_001021689.2	lncRNA
XM_012134593.2	FASTKD1	mRNA	XR_001021689.2	lncRNA
XM_012103723.1	POLI	mRNA	TCONS_00071561	lncRNA
XM_012103723.1	POLI	mRNA	TCONS_00071561	lncRNA
XM_012103723.1	POLI	mRNA	TCONS_00071561	lncRNA
XM_012103723.1	POLI	mRNA	TCONS_00071561	lncRNA
XM_015103117.1	ZBTB44	mRNA	XR_001021689.2	lncRNA
XM_015103117.1	ZBTB44	mRNA	XR_001021689.2	lncRNA
XM_015103117.1	ZBTB44	mRNA	XR_001021689.2	lncRNA
XM_015103117.1	ZBTB44	mRNA	XR_001021689.2	lncRNA
XM_012098342.2	FXR1	mRNA	XR_001021689.2	lncRNA
XM_012098342.2	FXR1	mRNA	XR_001021689.2	lncRNA
XM_012098342.2	FXR1	mRNA	XR_001021689.2	lncRNA
XM_012098342.2	FXR1	mRNA	XR_001021689.2	lncRNA
XM_012177139.2	TMEM209	mRNA	XR_001021689.2	lncRNA
XM_012177139.2	TMEM209	mRNA	XR_001021689.2	lncRNA
XM_012177139.2	TMEM209	mRNA	XR_001021689.2	lncRNA
XM_012177139.2	TMEM209	mRNA	XR_001021689.2	lncRNA
XM_004017788.3	ZFANL3	mRNA	TCONS_00029398	lncRNA
XM_004017788.3	ZFANL3	mRNA	TCONS_00029398	lncRNA
XM_004017788.3	ZFANL3	mRNA	TCONS_00029398	lncRNA
XM_004017788.3	ZFANL3	mRNA	TCONS_00029398	lncRNA
XM_012177174.2	CALD1	mRNA	TCONS_00029398	lncRNA
XM_012177174.2	CALD1	mRNA	TCONS_00029398	lncRNA
XM_012177174.2	CALD1	mRNA	TCONS_00029398	lncRNA
XM_012177174.2	CALD1	mRNA	TCONS_00029398	lncRNA
XM_004001781.3	ZSCAN20	mRNA	TCONS_00029398	lncRNA
XM_004001781.3	ZSCAN20	mRNA	TCONS_00029398	lncRNA
XM_004001781.3	ZSCAN20	mRNA	TCONS_00029398	lncRNA
XM_004001781.3	ZSCAN20	mRNA	TCONS_00029398	lncRNA
XM_012100861.1	FBXO9	mRNA	XR_001435726.1	lncRNA
XM_012100861.1	FBXO9	mRNA	XR_001435726.1	lncRNA
XM_012100861.1	FBXO9	mRNA	XR_001435726.1	lncRNA
XM_012100861.1	FBXO9	mRNA	XR_001435726.1	lncRNA
XM_012119517.2	SORBS3	mRNA	XR_001435726.1	lncRNA
XM_012119517.2	SORBS3	mRNA	XR_001435726.1	lncRNA
XM_012119517.2	SORBS3	mRNA	XR_001435726.1	lncRNA
XM_012119517.2	SORBS3	mRNA	XR_001435726.1	lncRNA

XM_012100555.2	PNPLA1	mRNA	XR_001435723.1	lncRNA
XM_012100555.2	PNPLA1	mRNA	XR_001435723.1	lncRNA
XM_012100555.2	PNPLA1	mRNA	XR_001435723.1	lncRNA
XM_012100555.2	PNPLA1	mRNA	XR_001435723.1	lncRNA
XM_012106574.2	ARHGEF9	mRNA	XR_001042381.2	lncRNA
XM_012106574.2	ARHGEF9	mRNA	XR_001042381.2	lncRNA
XM_012106574.2	ARHGEF9	mRNA	XR_001042381.2	lncRNA
XM_012106574.2	ARHGEF9	mRNA	XR_001042381.2	lncRNA
XM_012119517.2	SORBS3	mRNA	XR_001435723.1	lncRNA
XM_012119517.2	SORBS3	mRNA	XR_001435723.1	lncRNA
XM_012119517.2	SORBS3	mRNA	XR_001435723.1	lncRNA
XM_012119517.2	SORBS3	mRNA	XR_001435723.1	lncRNA
XM_012187919.2	LOC10561661	mRNA	XR_001434081.1	lncRNA
XM_012187919.2	LOC10561661	mRNA	XR_001434081.1	lncRNA
XM_012187919.2	LOC10561661	mRNA	XR_001434081.1	lncRNA
XM_012187919.2	LOC10561661	mRNA	XR_001434081.1	lncRNA
XM_015102528.1	FLOT1	mRNA	XR_001434081.1	lncRNA
XM_015102528.1	FLOT1	mRNA	XR_001434081.1	lncRNA
XM_015102528.1	FLOT1	mRNA	XR_001434081.1	lncRNA
XM_015102528.1	FLOT1	mRNA	XR_001434081.1	lncRNA
XM_012116202.1	IARS	mRNA	XR_001436099.1	lncRNA
XM_012116202.1	IARS	mRNA	XR_001436099.1	lncRNA
XM_012116202.1	IARS	mRNA	XR_001436099.1	lncRNA
XM_012116202.1	IARS	mRNA	XR_001436099.1	lncRNA
XM_012187630.2	SOAT1	mRNA	TCONS_00056022	lncRNA
XM_012187630.2	SOAT1	mRNA	TCONS_00056022	lncRNA
XM_012187630.2	SOAT1	mRNA	TCONS_00056022	lncRNA
XM_012179604.2	UBE2D3	mRNA	TCONS_00056022	lncRNA
XM_012179604.2	UBE2D3	mRNA	TCONS_00056022	lncRNA
XM_012179604.2	UBE2D3	mRNA	TCONS_00056022	lncRNA
XM_015094750.1	RAB3IP	mRNA	XR_001021939.1	lncRNA
XM_015094750.1	RAB3IP	mRNA	XR_001021939.1	lncRNA
XM_015094750.1	RAB3IP	mRNA	XR_001021939.1	lncRNA
XM_015094750.1	RAB3IP	mRNA	XR_001021939.1	lncRNA
XM_012179421.1	METTL14	mRNA	XR_001021939.1	lncRNA
XM_012179421.1	METTL14	mRNA	XR_001021939.1	lncRNA
XM_012179421.1	METTL14	mRNA	XR_001021939.1	lncRNA
XM_012179421.1	METTL14	mRNA	XR_001021939.1	lncRNA
XM_012178641.2	CTTNBP2NL	mRNA	XR_001021689.2	lncRNA
XM_012178641.2	CTTNBP2NL	mRNA	XR_001021689.2	lncRNA
XM_012178641.2	CTTNBP2NL	mRNA	XR_001021689.2	lncRNA
XM_012178641.2	CTTNBP2NL	mRNA	XR_001021689.2	lncRNA
XM_012190630.2	PLEKHA4	mRNA	XR_001021689.2	lncRNA
XM_012190630.2	PLEKHA4	mRNA	XR_001021689.2	lncRNA
XM_012190630.2	PLEKHA4	mRNA	XR_001021689.2	lncRNA
XM_012190630.2	PLEKHA4	mRNA	XR_001021689.2	lncRNA
XM_012133845.1	DYNC1I2	mRNA	TCONS_00029399	lncRNA
XM_012133845.1	DYNC1I2	mRNA	TCONS_00029399	lncRNA
XM_012133845.1	DYNC1I2	mRNA	TCONS_00029399	lncRNA
XM_012133845.1	DYNC1I2	mRNA	TCONS_00029399	lncRNA
XM_012173983.2	PPP1R12A	mRNA	TCONS_00029399	lncRNA
XM_012173983.2	PPP1R12A	mRNA	TCONS_00029399	lncRNA
XM_012173983.2	PPP1R12A	mRNA	TCONS_00029399	lncRNA
XM_012173983.2	PPP1R12A	mRNA	TCONS_00029399	lncRNA

XM_015103687.1	PTPN2	mRNA	XR_001041209.2	lncRNA
XM_015103687.1	PTPN2	mRNA	XR_001041209.2	lncRNA
XM_015103687.1	PTPN2	mRNA	XR_001041209.2	lncRNA
XM_015103687.1	PTPN2	mRNA	XR_001041209.2	lncRNA
XM_012189279.2	DNTTIP1	mRNA	XR_001041211.2	lncRNA
XM_012189279.2	DNTTIP1	mRNA	XR_001041211.2	lncRNA
XM_012189279.2	DNTTIP1	mRNA	XR_001041211.2	lncRNA
XM_012189279.2	DNTTIP1	mRNA	XR_001041211.2	lncRNA
XM_012161685.1	LRRC8A	mRNA	XR_001041211.2	lncRNA
XM_012161685.1	LRRC8A	mRNA	XR_001041211.2	lncRNA
XM_012161685.1	LRRC8A	mRNA	XR_001041211.2	lncRNA
XM_012161685.1	LRRC8A	mRNA	XR_001041211.2	lncRNA
XM_012190140.2	LSM14A	mRNA	XR_001436531.1	lncRNA
XM_012190140.2	LSM14A	mRNA	XR_001436531.1	lncRNA
XM_012190140.2	LSM14A	mRNA	XR_001436531.1	lncRNA
XM_012190140.2	LSM14A	mRNA	XR_001436531.1	lncRNA
XM_012190140.2	LSM14A	mRNA	XR_001436609.1	lncRNA
XM_012190140.2	LSM14A	mRNA	XR_001436609.1	lncRNA
XM_012190140.2	LSM14A	mRNA	XR_001436609.1	lncRNA
XM_012190140.2	LSM14A	mRNA	XR_001436609.1	lncRNA
XM_012106024.2	HOOK3	mRNA	XR_001436609.1	lncRNA
XM_012106024.2	HOOK3	mRNA	XR_001436609.1	lncRNA
XM_012106024.2	HOOK3	mRNA	XR_001436609.1	lncRNA
XM_012106024.2	HOOK3	mRNA	XR_001436609.1	lncRNA
XM_015102528.1	FLOT1	mRNA	XR_001436609.1	lncRNA
XM_015102528.1	FLOT1	mRNA	XR_001436609.1	lncRNA
XM_015102528.1	FLOT1	mRNA	XR_001436609.1	lncRNA
XM_015102528.1	FLOT1	mRNA	XR_001436609.1	lncRNA
XM_012095858.2	ZNF214	mRNA	TCONS_00056022	lncRNA
XM_012095858.2	ZNF214	mRNA	TCONS_00056022	lncRNA
XM_012095858.2	ZNF214	mRNA	TCONS_00056022	lncRNA
XM_015095288.1	NRCAM	mRNA	TCONS_00029398	lncRNA
XM_015095288.1	NRCAM	mRNA	TCONS_00029398	lncRNA
XM_015095288.1	NRCAM	mRNA	TCONS_00029398	lncRNA
XM_015095288.1	NRCAM	mRNA	TCONS_00029398	lncRNA
XM_012189279.2	DNTTIP1	mRNA	XR_001434081.1	lncRNA
XM_012189279.2	DNTTIP1	mRNA	XR_001434081.1	lncRNA
XM_012189279.2	DNTTIP1	mRNA	XR_001434081.1	lncRNA
XM_012189279.2	DNTTIP1	mRNA	XR_001434081.1	lncRNA
XM_015102413.1	RBM6	mRNA	TCONS_00078442	lncRNA
XM_015102413.1	RBM6	mRNA	TCONS_00078442	lncRNA
XM_015102413.1	RBM6	mRNA	TCONS_00078442	lncRNA
XM_012100729.1	POLH	mRNA	TCONS_00078442	lncRNA
XM_012100729.1	POLH	mRNA	TCONS_00078442	lncRNA
XM_012100729.1	POLH	mRNA	TCONS_00078442	lncRNA
XM_012179621.1	DNAJB14	mRNA	TCONS_00078442	lncRNA
XM_012179621.1	DNAJB14	mRNA	TCONS_00078442	lncRNA
XM_012179621.1	DNAJB14	mRNA	TCONS_00078442	lncRNA
XM_012097771.2	ORAI1	mRNA	TCONS_00078442	lncRNA
XM_012097771.2	ORAI1	mRNA	TCONS_00078442	lncRNA
XM_012097771.2	ORAI1	mRNA	TCONS_00078442	lncRNA
XM_012182238.1	SPG11	mRNA	TCONS_00071561	lncRNA
XM_012182238.1	SPG11	mRNA	TCONS_00071561	lncRNA
XM_012182238.1	SPG11	mRNA	TCONS_00071561	lncRNA

XM_012182238.1	SPG11	mRNA	TCONS_00071561	lncRNA
XM_012182215.2	TDP1	mRNA	XR_001021689.2	lncRNA
XM_012182215.2	TDP1	mRNA	XR_001021689.2	lncRNA
XM_012182215.2	TDP1	mRNA	XR_001021689.2	lncRNA
XM_012182215.2	TDP1	mRNA	XR_001021689.2	lncRNA
XM_015096814.1	DENND4A	mRNA	XR_001021689.2	lncRNA
XM_015096814.1	DENND4A	mRNA	XR_001021689.2	lncRNA
XM_015096814.1	DENND4A	mRNA	XR_001021689.2	lncRNA
XM_015096814.1	DENND4A	mRNA	XR_001021689.2	lncRNA
XM_012181829.2	ZBTB1	mRNA	XR_001021689.2	lncRNA
XM_012181829.2	ZBTB1	mRNA	XR_001021689.2	lncRNA
XM_012181829.2	ZBTB1	mRNA	XR_001021689.2	lncRNA
XM_012181829.2	ZBTB1	mRNA	XR_001021689.2	lncRNA
XM_012181420.2	ICE2	mRNA	XR_001021689.2	lncRNA
XM_012181420.2	ICE2	mRNA	XR_001021689.2	lncRNA
XM_012181420.2	ICE2	mRNA	XR_001021689.2	lncRNA
XM_012181420.2	ICE2	mRNA	XR_001021689.2	lncRNA
XM_015099256.1	CEP104	mRNA	XR_001041202.1	lncRNA
XM_015099256.1	CEP104	mRNA	XR_001041202.1	lncRNA
XM_015099256.1	CEP104	mRNA	XR_001041202.1	lncRNA
XM_015099256.1	CEP104	mRNA	XR_001041202.1	lncRNA
XM_012105316.2	USP54	mRNA	XR_001041202.1	lncRNA
XM_012105316.2	USP54	mRNA	XR_001041202.1	lncRNA
XM_012105316.2	USP54	mRNA	XR_001041202.1	lncRNA
XM_012105316.2	USP54	mRNA	XR_001041202.1	lncRNA
XM_012095387.2	ELMOD1	mRNA	XR_001435726.1	lncRNA
XM_012095387.2	ELMOD1	mRNA	XR_001435726.1	lncRNA
XM_012095387.2	ELMOD1	mRNA	XR_001435726.1	lncRNA
XM_012095387.2	ELMOD1	mRNA	XR_001435726.1	lncRNA
XM_012182301.2	LRRFIP1	mRNA	XR_001041211.2	lncRNA
XM_012182301.2	LRRFIP1	mRNA	XR_001041211.2	lncRNA
XM_012182301.2	LRRFIP1	mRNA	XR_001041211.2	lncRNA
XM_012182301.2	LRRFIP1	mRNA	XR_001041211.2	lncRNA
XM_004003157.3	TNIK	mRNA	TCONS_00029398	lncRNA
XM_004003157.3	TNIK	mRNA	TCONS_00029398	lncRNA
XM_004003157.3	TNIK	mRNA	TCONS_00029398	lncRNA
XM_004003157.3	TNIK	mRNA	TCONS_00029398	lncRNA
XM_012101213.2	LOC1056018	mRNA	TCONS_00029398	lncRNA
XM_012101213.2	LOC1056018	mRNA	TCONS_00029398	lncRNA
XM_012101213.2	LOC1056018	mRNA	TCONS_00029398	lncRNA
XM_012099723.2	PSML3	mRNA	TCONS_00029398	lncRNA
XM_012099723.2	PSML3	mRNA	TCONS_00029398	lncRNA
XM_012099723.2	PSML3	mRNA	TCONS_00029398	lncRNA
XM_012186944.1	DSTYK	mRNA	XR_001434081.1	lncRNA
XM_012186944.1	DSTYK	mRNA	XR_001434081.1	lncRNA
XM_012186944.1	DSTYK	mRNA	XR_001434081.1	lncRNA
XM_012186944.1	DSTYK	mRNA	XR_001434081.1	lncRNA
XM_004002409.3	PDZK1	mRNA	XR_001434081.1	lncRNA
XM_004002409.3	PDZK1	mRNA	XR_001434081.1	lncRNA
XM_004002409.3	PDZK1	mRNA	XR_001434081.1	lncRNA
XM_004002409.3	PDZK1	mRNA	XR_001434081.1	lncRNA
XM_015091887.1	MYCL	mRNA	TCONS_00029398	lncRNA
XM_015091887.1	MYCL	mRNA	TCONS_00029398	lncRNA
XM_015091887.1	MYCL	mRNA	TCONS_00029398	lncRNA

XM_015091887.1	MYCL	mRNA	TCONS_00029398	lncRNA
XM_012175201.2	C2CD5	mRNA	XR_001039281.1	lncRNA
XM_012175201.2	C2CD5	mRNA	XR_001039281.1	lncRNA
XM_012175201.2	C2CD5	mRNA	XR_001039281.1	lncRNA
XM_004014209.3	CUL2	mRNA	XR_001039281.1	lncRNA
XM_004014209.3	CUL2	mRNA	XR_001039281.1	lncRNA
XM_004014209.3	CUL2	mRNA	XR_001039281.1	lncRNA
XM_012190349.2	ZNF420	mRNA	XR_001039281.1	lncRNA
XM_012190349.2	ZNF420	mRNA	XR_001039281.1	lncRNA
XM_012190349.2	ZNF420	mRNA	XR_001039281.1	lncRNA
XM_015097326.1	TBC1D32	mRNA	TCONS_00072289	lncRNA
XM_015097326.1	TBC1D32	mRNA	TCONS_00072289	lncRNA
XM_015097326.1	TBC1D32	mRNA	TCONS_00072289	lncRNA
XM_012101391.1	GMDS	mRNA	TCONS_00072289	lncRNA
XM_012101391.1	GMDS	mRNA	TCONS_00072289	lncRNA
XM_012101391.1	GMDS	mRNA	TCONS_00072289	lncRNA
XM_012105198.2	TET1	mRNA	TCONS_00056022	lncRNA
XM_012105198.2	TET1	mRNA	TCONS_00056022	lncRNA
XM_012105198.2	TET1	mRNA	TCONS_00056022	lncRNA
XM_012187063.2	MARK1	mRNA	TCONS_00018945	lncRNA
XM_012187063.2	MARK1	mRNA	TCONS_00018945	lncRNA
XM_012187063.2	MARK1	mRNA	TCONS_00018945	lncRNA
XM_012100861.1	FBXO9	mRNA	TCONS_00078442	lncRNA
XM_012100861.1	FBXO9	mRNA	TCONS_00078442	lncRNA
XM_012100861.1	FBXO9	mRNA	TCONS_00078442	lncRNA
XM_015103753.1	MBD1	mRNA	TCONS_00078442	lncRNA
XM_015103753.1	MBD1	mRNA	TCONS_00078442	lncRNA
XM_015103753.1	MBD1	mRNA	TCONS_00078442	lncRNA
XM_012115168.2	LOC1011236(	mRNA	TCONS_00052150	lncRNA
XM_012115168.2	LOC1011236(	mRNA	TCONS_00052150	lncRNA
XM_012115168.2	LOC1011236(	mRNA	TCONS_00052150	lncRNA
XM_004004723.3	SPOPL	mRNA	TCONS_00052150	lncRNA
XM_004004723.3	SPOPL	mRNA	TCONS_00052150	lncRNA
XM_004004723.3	SPOPL	mRNA	TCONS_00052150	lncRNA
XM_012174558.1	CNOT2	mRNA	XR_001041202.1	lncRNA
XM_012174558.1	CNOT2	mRNA	XR_001041202.1	lncRNA
XM_012174558.1	CNOT2	mRNA	XR_001041202.1	lncRNA
XM_012189279.2	DNTTIP1	mRNA	TCONS_00063226	lncRNA
XM_012189279.2	DNTTIP1	mRNA	TCONS_00063226	lncRNA
XM_012189279.2	DNTTIP1	mRNA	TCONS_00063226	lncRNA
XM_012098436.1	KLHL25	mRNA	TCONS_00082638	lncRNA
XM_012098436.1	KLHL25	mRNA	TCONS_00082638	lncRNA
XM_012098436.1	KLHL25	mRNA	TCONS_00082638	lncRNA
XM_012188664.2	RASSF2	mRNA	TCONS_00078442	lncRNA
XM_012188664.2	RASSF2	mRNA	TCONS_00078442	lncRNA
XM_012188664.2	RASSF2	mRNA	TCONS_00078442	lncRNA
XM_012096744.2	GFM2	mRNA	TCONS_00078442	lncRNA
XM_012096744.2	GFM2	mRNA	TCONS_00078442	lncRNA
XM_012096744.2	GFM2	mRNA	TCONS_00078442	lncRNA
XM_012187919.2	LOC10561661	mRNA	TCONS_00078442	lncRNA
XM_012187919.2	LOC10561661	mRNA	TCONS_00078442	lncRNA
XM_012187919.2	LOC10561661	mRNA	TCONS_00078442	lncRNA
XM_015092197.1	CD58	mRNA	TCONS_00078442	lncRNA
XM_015092197.1	CD58	mRNA	TCONS_00078442	lncRNA

XM_015092197.1	CD58	mRNA	TCONS_00078442	lncRNA
XM_012133854.2	DYNC1I2	mRNA	TCONS_00078442	lncRNA
XM_012133854.2	DYNC1I2	mRNA	TCONS_00078442	lncRNA
XM_012133854.2	DYNC1I2	mRNA	TCONS_00078442	lncRNA
XM_012187630.2	SOAT1	mRNA	TCONS_00078442	lncRNA
XM_012187630.2	SOAT1	mRNA	TCONS_00078442	lncRNA
XM_012187630.2	SOAT1	mRNA	TCONS_00078442	lncRNA
XM_015097359.1	ARMC2	mRNA	TCONS_00078442	lncRNA
XM_015097359.1	ARMC2	mRNA	TCONS_00078442	lncRNA
XM_015097359.1	ARMC2	mRNA	TCONS_00078442	lncRNA
XM_015093098.1	PPP3CC	mRNA	TCONS_00052150	lncRNA
XM_015093098.1	PPP3CC	mRNA	TCONS_00052150	lncRNA
XM_015093098.1	PPP3CC	mRNA	TCONS_00052150	lncRNA
XM_012102549.2	CEP70	mRNA	TCONS_00078442	lncRNA
XM_012102549.2	CEP70	mRNA	TCONS_00078442	lncRNA
XM_012102549.2	CEP70	mRNA	TCONS_00078442	lncRNA
XM_012106040.2	LOC1011208[illegible]	mRNA	TCONS_00078442	lncRNA
XM_012106040.2	LOC1011208[illegible]	mRNA	TCONS_00078442	lncRNA
XM_012106040.2	LOC1011208[illegible]	mRNA	TCONS_00078442	lncRNA
XM_012124912.1	KIAA2026	mRNA	TCONS_00078442	lncRNA
XM_012124912.1	KIAA2026	mRNA	TCONS_00078442	lncRNA
XM_012124912.1	KIAA2026	mRNA	TCONS_00078442	lncRNA
XM_015105106.1	ATG4A	mRNA	TCONS_00072289	lncRNA
XM_015105106.1	ATG4A	mRNA	TCONS_00072289	lncRNA
XM_015105106.1	ATG4A	mRNA	TCONS_00072289	lncRNA
XM_012186040.2	GRB7	mRNA	XR_001039281.1	lncRNA
XM_012186040.2	GRB7	mRNA	XR_001039281.1	lncRNA
XM_012186040.2	GRB7	mRNA	XR_001039281.1	lncRNA
XM_012115168.2	LOC1011236[illegible]	mRNA	TCONS_00072289	lncRNA
XM_012115168.2	LOC1011236[illegible]	mRNA	TCONS_00072289	lncRNA
XM_012115168.2	LOC1011236[illegible]	mRNA	TCONS_00072289	lncRNA
XM_012107383.2	XIAP	mRNA	XR_001039281.1	lncRNA
XM_012107383.2	XIAP	mRNA	XR_001039281.1	lncRNA
XM_012107383.2	XIAP	mRNA	XR_001039281.1	lncRNA
XM_004004723.3	SPOPL	mRNA	TCONS_00072289	lncRNA
XM_004004723.3	SPOPL	mRNA	TCONS_00072289	lncRNA
XM_004004723.3	SPOPL	mRNA	TCONS_00072289	lncRNA
XM_015092793.1	LOC1069905[illegible]	mRNA	XR_001039281.1	lncRNA
XM_015092793.1	LOC1069905[illegible]	mRNA	XR_001039281.1	lncRNA
XM_015092793.1	LOC1069905[illegible]	mRNA	XR_001039281.1	lncRNA
XM_012096215.2	EHF	mRNA	XR_001039281.1	lncRNA
XM_012096215.2	EHF	mRNA	XR_001039281.1	lncRNA
XM_012096215.2	EHF	mRNA	XR_001039281.1	lncRNA
XM_004002018.3	OMA1	mRNA	XR_001041211.2	lncRNA
XM_004002018.3	OMA1	mRNA	XR_001041211.2	lncRNA
XM_004002018.3	OMA1	mRNA	XR_001041211.2	lncRNA
XM_012174558.1	CNOT2	mRNA	XR_001041211.2	lncRNA
XM_012174558.1	CNOT2	mRNA	XR_001041211.2	lncRNA
XM_012174558.1	CNOT2	mRNA	XR_001041211.2	lncRNA
XM_012099723.2	PSML3	mRNA	TCONS_00029399	lncRNA
XM_012099723.2	PSML3	mRNA	TCONS_00029399	lncRNA
XM_012099723.2	PSML3	mRNA	TCONS_00029399	lncRNA
XM_012102908.2	IL20RB	mRNA	TCONS_00029399	lncRNA
XM_012102908.2	IL20RB	mRNA	TCONS_00029399	lncRNA

XM_012102908.2	IL20RB	mRNA	TCONS_00029399	lncRNA
XM_012124491.2	AK3	mRNA	XR_001041209.2	lncRNA
XM_012124491.2	AK3	mRNA	XR_001041209.2	lncRNA
XM_012124491.2	AK3	mRNA	XR_001041209.2	lncRNA
XM_012102908.2	IL20RB	mRNA	XR_001041209.2	lncRNA
XM_012102908.2	IL20RB	mRNA	XR_001041209.2	lncRNA
XM_012102908.2	IL20RB	mRNA	XR_001041209.2	lncRNA
XM_012187090.2	BROX	mRNA	XR_001041209.2	lncRNA
XM_012187090.2	BROX	mRNA	XR_001041209.2	lncRNA
XM_012187090.2	BROX	mRNA	XR_001041209.2	lncRNA
XM_012176644.2	DNAJC2	mRNA	XR_001041209.2	lncRNA
XM_012176644.2	DNAJC2	mRNA	XR_001041209.2	lncRNA
XM_012176644.2	DNAJC2	mRNA	XR_001041209.2	lncRNA
XM_015098203.1	LOC10111931	mRNA	XR_001041211.2	lncRNA
XM_015098203.1	LOC10111931	mRNA	XR_001041211.2	lncRNA
XM_015098203.1	LOC10111931	mRNA	XR_001041211.2	lncRNA
XM_012124491.2	AK3	mRNA	TCONS_00029399	lncRNA
XM_012124491.2	AK3	mRNA	TCONS_00029399	lncRNA
XM_012124491.2	AK3	mRNA	TCONS_00029399	lncRNA
XM_015100042.1	ZNF473	mRNA	XR_001041209.2	lncRNA
XM_015100042.1	ZNF473	mRNA	XR_001041209.2	lncRNA
XM_015100042.1	ZNF473	mRNA	XR_001041209.2	lncRNA
XM_012176644.2	DNAJC2	mRNA	TCONS_00029399	lncRNA
XM_012176644.2	DNAJC2	mRNA	TCONS_00029399	lncRNA
XM_012176644.2	DNAJC2	mRNA	TCONS_00029399	lncRNA
XM_012187090.2	BROX	mRNA	TCONS_00029399	lncRNA
XM_012187090.2	BROX	mRNA	TCONS_00029399	lncRNA
XM_012187090.2	BROX	mRNA	TCONS_00029399	lncRNA
XM_015102269.1	NKTR	mRNA	TCONS_00056022	lncRNA
XM_015102269.1	NKTR	mRNA	TCONS_00056022	lncRNA
XM_015102269.1	NKTR	mRNA	TCONS_00056022	lncRNA
XM_012189777.1	CHD9	mRNA	TCONS_00056022	lncRNA
XM_012189777.1	CHD9	mRNA	TCONS_00056022	lncRNA
XM_012189777.1	CHD9	mRNA	TCONS_00056022	lncRNA
XM_004003157.3	TNIK	mRNA	TCONS_00056022	lncRNA
XM_004003157.3	TNIK	mRNA	TCONS_00056022	lncRNA
XM_004003157.3	TNIK	mRNA	TCONS_00056022	lncRNA
XM_012175201.2	C2CD5	mRNA	TCONS_00029398	lncRNA
XM_012175201.2	C2CD5	mRNA	TCONS_00029398	lncRNA
XM_012175201.2	C2CD5	mRNA	TCONS_00029398	lncRNA
XM_004001901.2	ERI3	mRNA	TCONS_00029398	lncRNA
XM_004001901.2	ERI3	mRNA	TCONS_00029398	lncRNA
XM_004001901.2	ERI3	mRNA	TCONS_00029398	lncRNA
XM_004002018.3	OMA1	mRNA	XR_001434081.1	lncRNA
XM_004002018.3	OMA1	mRNA	XR_001434081.1	lncRNA
XM_004002018.3	OMA1	mRNA	XR_001434081.1	lncRNA
XM_012105672.2	LOC1011063[illegible]	mRNA	XR_001434081.1	lncRNA
XM_012105672.2	LOC1011063[illegible]	mRNA	XR_001434081.1	lncRNA
XM_012105672.2	LOC1011063[illegible]	mRNA	XR_001434081.1	lncRNA
XM_012182190.2	EML5	mRNA	TCONS_00056022	lncRNA
XM_012182190.2	EML5	mRNA	TCONS_00056022	lncRNA
XM_012182190.2	EML5	mRNA	TCONS_00056022	lncRNA
XM_015104838.1	PDK3	mRNA	TCONS_00082638	lncRNA
XM_015104838.1	PDK3	mRNA	TCONS_00082638	lncRNA

XM_015104838.1	PDK3	mRNA	TCONS_00082638	lncRNA
XM_012179621.1	DNAJB14	mRNA	XR_001021689.2	lncRNA
XM_012179621.1	DNAJB14	mRNA	XR_001021689.2	lncRNA
XM_012179621.1	DNAJB14	mRNA	XR_001021689.2	lncRNA
XM_004016017.3	C15H11orf1	mRNA	TCONS_00071561	lncRNA
XM_004016017.3	C15H11orf1	mRNA	TCONS_00071561	lncRNA
XM_004016017.3	C15H11orf1	mRNA	TCONS_00071561	lncRNA
XM_012182520.2	SLC35F1	mRNA	XR_001021689.2	lncRNA
XM_012182520.2	SLC35F1	mRNA	XR_001021689.2	lncRNA
XM_012182520.2	SLC35F1	mRNA	XR_001021689.2	lncRNA
XM_015097223.1	ZC3H14	mRNA	XR_001021689.2	lncRNA
XM_015097223.1	ZC3H14	mRNA	XR_001021689.2	lncRNA
XM_015097223.1	ZC3H14	mRNA	XR_001021689.2	lncRNA
XM_012096375.2	PSMB2	mRNA	TCONS_00071561	lncRNA
XM_012096375.2	PSMB2	mRNA	TCONS_00071561	lncRNA
XM_012096375.2	PSMB2	mRNA	TCONS_00071561	lncRNA
XM_012100729.1	POLH	mRNA	TCONS_00071561	lncRNA
XM_012100729.1	POLH	mRNA	TCONS_00071561	lncRNA
XM_012100729.1	POLH	mRNA	TCONS_00071561	lncRNA
XM_015093007.1	TMEM246	mRNA	TCONS_00071561	lncRNA
XM_015093007.1	TMEM246	mRNA	TCONS_00071561	lncRNA
XM_015093007.1	TMEM246	mRNA	TCONS_00071561	lncRNA
XM_012174787.1	LOC1011137	mRNA	TCONS_00052150	lncRNA
XM_012174787.1	LOC1011137	mRNA	TCONS_00052150	lncRNA
XM_012174787.1	LOC1011137	mRNA	TCONS_00052150	lncRNA
XM_012174787.1	LOC1011137	mRNA	TCONS_00078442	lncRNA
XM_012174787.1	LOC1011137	mRNA	TCONS_00078442	lncRNA
XM_012174787.1	LOC1011137	mRNA	TCONS_00078442	lncRNA
XM_012133845.1	DYNC1I2	mRNA	TCONS_00078442	lncRNA
XM_012133845.1	DYNC1I2	mRNA	TCONS_00078442	lncRNA
XM_012133845.1	DYNC1I2	mRNA	TCONS_00078442	lncRNA
XM_012180800.1	TIPIN	mRNA	TCONS_00078442	lncRNA
XM_012180800.1	TIPIN	mRNA	TCONS_00078442	lncRNA
XM_012180800.1	TIPIN	mRNA	TCONS_00078442	lncRNA
XM_015103687.1	PTPN2	mRNA	TCONS_00078442	lncRNA
XM_015103687.1	PTPN2	mRNA	TCONS_00078442	lncRNA
XM_015103687.1	PTPN2	mRNA	TCONS_00078442	lncRNA
XM_012106710.2	CLCN5	mRNA	TCONS_00082638	lncRNA
XM_012106710.2	CLCN5	mRNA	TCONS_00082638	lncRNA
XM_012106710.2	CLCN5	mRNA	TCONS_00082638	lncRNA
XM_012190293.2	ZNF568	mRNA	XR_001021939.1	lncRNA
XM_012190293.2	ZNF568	mRNA	XR_001021939.1	lncRNA
XM_012190293.2	ZNF568	mRNA	XR_001021939.1	lncRNA
XM_012185629.2	ELP5	mRNA	XR_001021939.1	lncRNA
XM_012185629.2	ELP5	mRNA	XR_001021939.1	lncRNA
XM_012185629.2	ELP5	mRNA	XR_001021939.1	lncRNA
XM_012190515.2	ZMYM1	mRNA	XR_001039281.1	lncRNA
XM_012190515.2	ZMYM1	mRNA	XR_001039281.1	lncRNA
XM_012190515.2	ZMYM1	mRNA	XR_001039281.1	lncRNA
XM_012182988.2	REPS1	mRNA	XR_001039281.1	lncRNA
XM_012182988.2	REPS1	mRNA	XR_001039281.1	lncRNA
XM_012182988.2	REPS1	mRNA	XR_001039281.1	lncRNA
XM_012094768.2	ZMYM1	mRNA	TCONS_00072289	lncRNA
XM_012094768.2	ZMYM1	mRNA	TCONS_00072289	lncRNA

XM_012094768.2	ZMYM1	mRNA	TCONS_00072289	lncRNA
XM_012190083.2	ZMYM6	mRNA	TCONS_00072289	lncRNA
XM_012190083.2	ZMYM6	mRNA	TCONS_00072289	lncRNA
XM_012190083.2	ZMYM6	mRNA	TCONS_00072289	lncRNA
XM_015097359.1	ARMC2	mRNA	XR_001039281.1	lncRNA
XM_015097359.1	ARMC2	mRNA	XR_001039281.1	lncRNA
XM_015097359.1	ARMC2	mRNA	XR_001039281.1	lncRNA
XM_015093098.1	PPP3CC	mRNA	TCONS_00072289	lncRNA
XM_015093098.1	PPP3CC	mRNA	TCONS_00072289	lncRNA
XM_015093098.1	PPP3CC	mRNA	TCONS_00072289	lncRNA
XM_015092531.1	DCUN1D1	mRNA	XR_001039281.1	lncRNA
XM_015092531.1	DCUN1D1	mRNA	XR_001039281.1	lncRNA
XM_015092531.1	DCUN1D1	mRNA	XR_001039281.1	lncRNA
XM_012101213.2	LOC1056018(	mRNA	XR_001436609.1	lncRNA
XM_012101213.2	LOC1056018(	mRNA	XR_001436609.1	lncRNA
XM_012101213.2	LOC1056018(	mRNA	XR_001436609.1	lncRNA
XM_012101213.2	LOC1056018(	mRNA	XR_001435104.1	lncRNA
XM_012101213.2	LOC1056018(	mRNA	XR_001435104.1	lncRNA
XM_012101213.2	LOC1056018(	mRNA	XR_001435104.1	lncRNA
XM_012102908.2	IL20RB	mRNA	XR_001436609.1	lncRNA
XM_012102908.2	IL20RB	mRNA	XR_001436609.1	lncRNA
XM_012102908.2	IL20RB	mRNA	XR_001436609.1	lncRNA
XM_012185629.2	ELP5	mRNA	XR_001435104.1	lncRNA
XM_012185629.2	ELP5	mRNA	XR_001435104.1	lncRNA
XM_012185629.2	ELP5	mRNA	XR_001435104.1	lncRNA
XM_004002018.3	OMA1	mRNA	XR_001435104.1	lncRNA
XM_004002018.3	OMA1	mRNA	XR_001435104.1	lncRNA
XM_004002018.3	OMA1	mRNA	XR_001435104.1	lncRNA
XM_012099723.2	PSML3	mRNA	XR_001435104.1	lncRNA
XM_012099723.2	PSML3	mRNA	XR_001435104.1	lncRNA
XM_012099723.2	PSML3	mRNA	XR_001435104.1	lncRNA
XM_012190293.2	ZNF568	mRNA	XR_001435104.1	lncRNA
XM_012190293.2	ZNF568	mRNA	XR_001435104.1	lncRNA
XM_012190293.2	ZNF568	mRNA	XR_001435104.1	lncRNA
XM_004004614.3	PHOSPHO2	mRNA	XR_001436531.1	lncRNA
XM_004004614.3	PHOSPHO2	mRNA	XR_001436531.1	lncRNA
XM_004004614.3	PHOSPHO2	mRNA	XR_001436531.1	lncRNA
XM_015095069.1	LOC10561082	mRNA	XR_001435104.1	lncRNA
XM_015095069.1	LOC10561082	mRNA	XR_001435104.1	lncRNA
XM_015095069.1	LOC10561082	mRNA	XR_001435104.1	lncRNA
XM_015100042.1	ZNF473	mRNA	XR_001435104.1	lncRNA
XM_015100042.1	ZNF473	mRNA	XR_001435104.1	lncRNA
XM_015100042.1	ZNF473	mRNA	XR_001435104.1	lncRNA
XM_004002018.3	OMA1	mRNA	XR_001436609.1	lncRNA
XM_004002018.3	OMA1	mRNA	XR_001436609.1	lncRNA
XM_004002018.3	OMA1	mRNA	XR_001436609.1	lncRNA
XM_012188279.1	CCDC7	mRNA	XR_001436609.1	lncRNA
XM_012188279.1	CCDC7	mRNA	XR_001436609.1	lncRNA
XM_012188279.1	CCDC7	mRNA	XR_001436609.1	lncRNA
XM_015104365.1	CSGALNACT	mRNA	XR_001436531.1	lncRNA
XM_015104365.1	CSGALNACT	mRNA	XR_001436531.1	lncRNA
XM_015104365.1	CSGALNACT	mRNA	XR_001436531.1	lncRNA
XM_012095761.2	HSPBAP1	mRNA	XR_001436609.1	lncRNA
XM_012095761.2	HSPBAP1	mRNA	XR_001436609.1	lncRNA

XM_012095761.2	HSPBAP1	mRNA	XR_001436609.1	lncRNA
XM_012105643.1	ZFP69	mRNA	XR_001436609.1	lncRNA
XM_012105643.1	ZFP69	mRNA	XR_001436609.1	lncRNA
XM_012105643.1	ZFP69	mRNA	XR_001436609.1	lncRNA
XM_012099223.2	SLC25A26	mRNA	XR_001436609.1	lncRNA
XM_012099223.2	SLC25A26	mRNA	XR_001436609.1	lncRNA
XM_012099223.2	SLC25A26	mRNA	XR_001436609.1	lncRNA
XM_012188279.1	CCDC7	mRNA	XR_001436531.1	lncRNA
XM_012188279.1	CCDC7	mRNA	XR_001436531.1	lncRNA
XM_012188279.1	CCDC7	mRNA	XR_001436531.1	lncRNA
XM_012174138.2	FAM73A	mRNA	XR_001436609.1	lncRNA
XM_012174138.2	FAM73A	mRNA	XR_001436609.1	lncRNA
XM_012174138.2	FAM73A	mRNA	XR_001436609.1	lncRNA
XM_012099723.2	PSML3	mRNA	XR_001436609.1	lncRNA
XM_012099723.2	PSML3	mRNA	XR_001436609.1	lncRNA
XM_012099723.2	PSML3	mRNA	XR_001436609.1	lncRNA
XM_012099223.2	SLC25A26	mRNA	XR_001435104.1	lncRNA
XM_012099223.2	SLC25A26	mRNA	XR_001435104.1	lncRNA
XM_012099223.2	SLC25A26	mRNA	XR_001435104.1	lncRNA
XM_012100296.2	MGLL	mRNA	XR_001436609.1	lncRNA
XM_012100296.2	MGLL	mRNA	XR_001436609.1	lncRNA
XM_012100296.2	MGLL	mRNA	XR_001436609.1	lncRNA
XM_012174558.1	CNOT2	mRNA	XR_001436609.1	lncRNA
XM_012174558.1	CNOT2	mRNA	XR_001436609.1	lncRNA
XM_012174558.1	CNOT2	mRNA	XR_001436609.1	lncRNA
XM_012103822.2	KIAA1468	mRNA	XR_001041202.1	lncRNA
XM_012103822.2	KIAA1468	mRNA	XR_001041202.1	lncRNA
XM_012103822.2	KIAA1468	mRNA	XR_001041202.1	lncRNA
XM_012184453.2	FNDC3A	mRNA	XR_001041202.1	lncRNA
XM_012184453.2	FNDC3A	mRNA	XR_001041202.1	lncRNA
XM_012184453.2	FNDC3A	mRNA	XR_001041202.1	lncRNA
XM_012182520.2	SLC35F1	mRNA	XR_001041202.1	lncRNA
XM_012182520.2	SLC35F1	mRNA	XR_001041202.1	lncRNA
XM_012182520.2	SLC35F1	mRNA	XR_001041202.1	lncRNA
XM_012100993.2	FLOT1	mRNA	XR_001041202.1	lncRNA
XM_012100993.2	FLOT1	mRNA	XR_001041202.1	lncRNA
XM_012100993.2	FLOT1	mRNA	XR_001041202.1	lncRNA
XM_012181686.2	DLGAP5	mRNA	XR_001041202.1	lncRNA
XM_012181686.2	DLGAP5	mRNA	XR_001041202.1	lncRNA
XM_012181686.2	DLGAP5	mRNA	XR_001041202.1	lncRNA
XM_012176498.2	RAPGEF5	mRNA	TCONS_00063226	lncRNA
XM_012176498.2	RAPGEF5	mRNA	TCONS_00063226	lncRNA
XM_012176498.2	RAPGEF5	mRNA	TCONS_00063226	lncRNA
XM_012102669.1	ZNF518A	mRNA	TCONS_00063226	lncRNA
XM_012102669.1	ZNF518A	mRNA	TCONS_00063226	lncRNA
XM_012102669.1	ZNF518A	mRNA	TCONS_00063226	lncRNA
XM_012095522.2	JAML	mRNA	TCONS_00029398	lncRNA
XM_012095522.2	JAML	mRNA	TCONS_00029398	lncRNA
XM_012095522.2	JAML	mRNA	TCONS_00029398	lncRNA
XM_012184609.2	N4BP2L2	mRNA	TCONS_00029398	lncRNA
XM_012184609.2	N4BP2L2	mRNA	TCONS_00029398	lncRNA
XM_012184609.2	N4BP2L2	mRNA	TCONS_00029398	lncRNA
XM_004002227.3	SLC35A3	mRNA	TCONS_00029398	lncRNA
XM_004002227.3	SLC35A3	mRNA	TCONS_00029398	lncRNA

XM_004002227.3	SLC35A3	mRNA	TCONS_00029398	lncRNA
XM_012188283.2	CCDC7	mRNA	TCONS_00029398	lncRNA
XM_012188283.2	CCDC7	mRNA	TCONS_00029398	lncRNA
XM_012188283.2	CCDC7	mRNA	TCONS_00029398	lncRNA
XM_012188286.2	CCDC7	mRNA	TCONS_00029398	lncRNA
XM_012188286.2	CCDC7	mRNA	TCONS_00029398	lncRNA
XM_012188286.2	CCDC7	mRNA	TCONS_00029398	lncRNA
XM_015092478.1	TFRC	mRNA	TCONS_00052150	lncRNA
XM_015092478.1	TFRC	mRNA	TCONS_00052150	lncRNA
XM_015092478.1	TFRC	mRNA	TCONS_00052150	lncRNA
XM_012098348.2	MEF2A	mRNA	TCONS_00078442	lncRNA
XM_012098348.2	MEF2A	mRNA	TCONS_00078442	lncRNA
XM_012098348.2	MEF2A	mRNA	TCONS_00078442	lncRNA
XM_012097468.2	ELF2	mRNA	TCONS_00078442	lncRNA
XM_012097468.2	ELF2	mRNA	TCONS_00078442	lncRNA
XM_012097468.2	ELF2	mRNA	TCONS_00078442	lncRNA
XM_015099384.1	SYT14	mRNA	TCONS_00078442	lncRNA
XM_015099384.1	SYT14	mRNA	TCONS_00078442	lncRNA
XM_015099384.1	SYT14	mRNA	TCONS_00078442	lncRNA
XM_012189447.2	ALCAM	mRNA	TCONS_00052150	lncRNA
XM_012189447.2	ALCAM	mRNA	TCONS_00052150	lncRNA
XM_012189447.2	ALCAM	mRNA	TCONS_00052150	lncRNA
XM_015103117.1	ZBTB44	mRNA	TCONS_00078442	lncRNA
XM_015103117.1	ZBTB44	mRNA	TCONS_00078442	lncRNA
XM_015103117.1	ZBTB44	mRNA	TCONS_00078442	lncRNA
XM_012181720.2	ARID4A	mRNA	TCONS_00078442	lncRNA
XM_012181720.2	ARID4A	mRNA	TCONS_00078442	lncRNA
XM_012181720.2	ARID4A	mRNA	TCONS_00078442	lncRNA
XM_004008093.2	PTN	mRNA	TCONS_00078442	lncRNA
XM_004008093.2	PTN	mRNA	TCONS_00078442	lncRNA
XM_004008093.2	PTN	mRNA	TCONS_00078442	lncRNA
XM_012180905.2	NPTN	mRNA	XR_001435726.1	lncRNA
XM_012180905.2	NPTN	mRNA	XR_001435726.1	lncRNA
XM_012180905.2	NPTN	mRNA	XR_001435726.1	lncRNA
XM_012176644.2	DNAJC2	mRNA	XR_001435726.1	lncRNA
XM_012176644.2	DNAJC2	mRNA	XR_001435726.1	lncRNA
XM_012176644.2	DNAJC2	mRNA	XR_001435726.1	lncRNA
XM_012174138.2	FAM73A	mRNA	XR_001435726.1	lncRNA
XM_012174138.2	FAM73A	mRNA	XR_001435726.1	lncRNA
XM_012174138.2	FAM73A	mRNA	XR_001435726.1	lncRNA
XM_012124491.2	AK3	mRNA	XR_001435723.1	lncRNA
XM_012124491.2	AK3	mRNA	XR_001435723.1	lncRNA
XM_012124491.2	AK3	mRNA	XR_001435723.1	lncRNA
XM_012187090.2	BROX	mRNA	XR_001435723.1	lncRNA
XM_012187090.2	BROX	mRNA	XR_001435723.1	lncRNA
XM_012187090.2	BROX	mRNA	XR_001435723.1	lncRNA
XM_012180905.2	NPTN	mRNA	XR_001435723.1	lncRNA
XM_012180905.2	NPTN	mRNA	XR_001435723.1	lncRNA
XM_012180905.2	NPTN	mRNA	XR_001435723.1	lncRNA
XM_012176644.2	DNAJC2	mRNA	XR_001435723.1	lncRNA
XM_012176644.2	DNAJC2	mRNA	XR_001435723.1	lncRNA
XM_012176644.2	DNAJC2	mRNA	XR_001435723.1	lncRNA
XM_012100431.2	VEGFA	mRNA	XR_001042381.2	lncRNA
XM_012100431.2	VEGFA	mRNA	XR_001042381.2	lncRNA

XM_012100431.2	VEGFA	mRNA	XR_001042381.2	lncRNA
XM_015100042.1	ZNF473	mRNA	XR_001435726.1	lncRNA
XM_015100042.1	ZNF473	mRNA	XR_001435726.1	lncRNA
XM_015100042.1	ZNF473	mRNA	XR_001435726.1	lncRNA
XM_012124491.2	AK3	mRNA	XR_001435726.1	lncRNA
XM_012124491.2	AK3	mRNA	XR_001435726.1	lncRNA
XM_012124491.2	AK3	mRNA	XR_001435726.1	lncRNA
XM_015099773.1	TPD52L2	mRNA	XR_001042381.2	lncRNA
XM_015099773.1	TPD52L2	mRNA	XR_001042381.2	lncRNA
XM_015099773.1	TPD52L2	mRNA	XR_001042381.2	lncRNA
XM_012105668.2	LOC1011063[illegible]	mRNA	XR_001042381.2	lncRNA
XM_012105668.2	LOC1011063[illegible]	mRNA	XR_001042381.2	lncRNA
XM_012105668.2	LOC1011063[illegible]	mRNA	XR_001042381.2	lncRNA
NM_001161880.1	CDKN1A	mRNA	XR_001042381.2	lncRNA
NM_001161880.1	CDKN1A	mRNA	XR_001042381.2	lncRNA
NM_001161880.1	CDKN1A	mRNA	XR_001042381.2	lncRNA
XM_015101282.1	KIF2A	mRNA	XR_001042381.2	lncRNA
XM_015101282.1	KIF2A	mRNA	XR_001042381.2	lncRNA
XM_015101282.1	KIF2A	mRNA	XR_001042381.2	lncRNA
XM_012183965.2	OXR1	mRNA	XR_001042381.2	lncRNA
XM_012183965.2	OXR1	mRNA	XR_001042381.2	lncRNA
XM_012183965.2	OXR1	mRNA	XR_001042381.2	lncRNA
XM_012095761.2	HSPBAP1	mRNA	XR_001042381.2	lncRNA
XM_012095761.2	HSPBAP1	mRNA	XR_001042381.2	lncRNA
XM_012095761.2	HSPBAP1	mRNA	XR_001042381.2	lncRNA
XM_012102908.2	IL20RB	mRNA	XR_001435726.1	lncRNA
XM_012102908.2	IL20RB	mRNA	XR_001435726.1	lncRNA
XM_012102908.2	IL20RB	mRNA	XR_001435726.1	lncRNA
XM_015093007.1	TMEM246	mRNA	XR_001033284.2	lncRNA
XM_015093007.1	TMEM246	mRNA	XR_001033284.2	lncRNA
XM_015093007.1	TMEM246	mRNA	XR_001033284.2	lncRNA
XM_004016017.3	C15H11orf1	mRNA	XR_001033284.2	lncRNA
XM_004016017.3	C15H11orf1	mRNA	XR_001033284.2	lncRNA
XM_004016017.3	C15H11orf1	mRNA	XR_001033284.2	lncRNA
XM_004014209.3	CUL2	mRNA	XR_001436099.1	lncRNA
XM_004014209.3	CUL2	mRNA	XR_001436099.1	lncRNA
XM_004014209.3	CUL2	mRNA	XR_001436099.1	lncRNA
XM_004017729.3	CHL1	mRNA	TCONS_00063226	lncRNA
XM_004017729.3	CHL1	mRNA	TCONS_00063226	lncRNA
XM_004017729.3	CHL1	mRNA	TCONS_00063226	lncRNA
XM_012180800.1	TIPIN	mRNA	TCONS_00072289	lncRNA
XM_012180800.1	TIPIN	mRNA	TCONS_00072289	lncRNA
XM_012180800.1	TIPIN	mRNA	TCONS_00072289	lncRNA
XM_004022347.2	TMEM255A	mRNA	TCONS_00072289	lncRNA
XM_004022347.2	TMEM255A	mRNA	TCONS_00072289	lncRNA
XM_004022347.2	TMEM255A	mRNA	TCONS_00072289	lncRNA
XM_012106466.2	DMD	mRNA	TCONS_00072289	lncRNA
XM_012106466.2	DMD	mRNA	TCONS_00072289	lncRNA
XM_012106466.2	DMD	mRNA	TCONS_00072289	lncRNA
XM_012182446.2	TTK	mRNA	XR_001039281.1	lncRNA
XM_012182446.2	TTK	mRNA	XR_001039281.1	lncRNA
XM_012182446.2	TTK	mRNA	XR_001039281.1	lncRNA
XM_015104131.1	TNRC6A	mRNA	XR_001039281.1	lncRNA
XM_015104131.1	TNRC6A	mRNA	XR_001039281.1	lncRNA

XM_015104131.1	TNRC6A	mRNA	XR_001039281.1	lncRNA
XM_012176656.2	NAPEPLD	mRNA	TCONS_00072289	lncRNA
XM_012176656.2	NAPEPLD	mRNA	TCONS_00072289	lncRNA
XM_012176656.2	NAPEPLD	mRNA	TCONS_00072289	lncRNA
XM_004008647.3	RAPGEF6	mRNA	TCONS_00072289	lncRNA
XM_004008647.3	RAPGEF6	mRNA	TCONS_00072289	lncRNA
XM_004008647.3	RAPGEF6	mRNA	TCONS_00072289	lncRNA
XM_012182445.2	TTK	mRNA	XR_001039281.1	lncRNA
XM_012182445.2	TTK	mRNA	XR_001039281.1	lncRNA
XM_012182445.2	TTK	mRNA	XR_001039281.1	lncRNA
XM_012095713.2	RRAS2	mRNA	TCONS_00078442	lncRNA
XM_012095713.2	RRAS2	mRNA	TCONS_00078442	lncRNA
XM_012095713.2	RRAS2	mRNA	TCONS_00078442	lncRNA
XM_012105333.2	USP54	mRNA	TCONS_00052150	lncRNA
XM_012105333.2	USP54	mRNA	TCONS_00052150	lncRNA
XM_012105333.2	USP54	mRNA	TCONS_00052150	lncRNA
XM_015099256.1	CEP104	mRNA	TCONS_00052150	lncRNA
XM_015099256.1	CEP104	mRNA	TCONS_00052150	lncRNA
XM_015099256.1	CEP104	mRNA	TCONS_00052150	lncRNA
XM_012178641.2	CTTNBP2NL	mRNA	TCONS_00078442	lncRNA
XM_012178641.2	CTTNBP2NL	mRNA	TCONS_00078442	lncRNA
XM_012178641.2	CTTNBP2NL	mRNA	TCONS_00078442	lncRNA
XM_012177174.2	CALD1	mRNA	TCONS_00052150	lncRNA
XM_012177174.2	CALD1	mRNA	TCONS_00052150	lncRNA
XM_012177174.2	CALD1	mRNA	TCONS_00052150	lncRNA
XM_012187063.2	MARK1	mRNA	TCONS_00078442	lncRNA
XM_012187063.2	MARK1	mRNA	TCONS_00078442	lncRNA
XM_012187063.2	MARK1	mRNA	TCONS_00078442	lncRNA
XM_012105316.2	USP54	mRNA	TCONS_00052150	lncRNA
XM_012105316.2	USP54	mRNA	TCONS_00052150	lncRNA
XM_012105316.2	USP54	mRNA	TCONS_00052150	lncRNA
XM_012177175.2	CALD1	mRNA	TCONS_00052150	lncRNA
XM_012177175.2	CALD1	mRNA	TCONS_00052150	lncRNA
XM_012177175.2	CALD1	mRNA	TCONS_00052150	lncRNA
XM_015093350.1	GPR155	mRNA	TCONS_00078442	lncRNA
XM_015093350.1	GPR155	mRNA	TCONS_00078442	lncRNA
XM_015093350.1	GPR155	mRNA	TCONS_00078442	lncRNA
XM_012142889.2	ICA1L	mRNA	TCONS_00078442	lncRNA
XM_012142889.2	ICA1L	mRNA	TCONS_00078442	lncRNA
XM_012142889.2	ICA1L	mRNA	TCONS_00078442	lncRNA
XM_004017788.3	ZFANL3	mRNA	TCONS_00078442	lncRNA
XM_004017788.3	ZFANL3	mRNA	TCONS_00078442	lncRNA
XM_004017788.3	ZFANL3	mRNA	TCONS_00078442	lncRNA
XM_012190630.2	PLEKHA4	mRNA	TCONS_00078442	lncRNA
XM_012190630.2	PLEKHA4	mRNA	TCONS_00078442	lncRNA
XM_012190630.2	PLEKHA4	mRNA	TCONS_00078442	lncRNA
XM_012102834.2	PPRC1	mRNA	TCONS_00078442	lncRNA
XM_012102834.2	PPRC1	mRNA	TCONS_00078442	lncRNA
XM_012102834.2	PPRC1	mRNA	TCONS_00078442	lncRNA
XM_012177369.2	PRKAG2	mRNA	TCONS_00071561	lncRNA
XM_012177369.2	PRKAG2	mRNA	TCONS_00071561	lncRNA
XM_012177369.2	PRKAG2	mRNA	TCONS_00071561	lncRNA
XM_012181771.2	PCNXL4	mRNA	XR_001021689.2	lncRNA
XM_012181771.2	PCNXL4	mRNA	XR_001021689.2	lncRNA

XM_012181771.2	PCNXL4	mRNA	XR_001021689.2	lncRNA
XM_012187727.2	TRMT1L	mRNA	XR_001021689.2	lncRNA
XM_012187727.2	TRMT1L	mRNA	XR_001021689.2	lncRNA
XM_012187727.2	TRMT1L	mRNA	XR_001021689.2	lncRNA
XM_012188286.2	CCDC7	mRNA	XR_001021689.2	lncRNA
XM_012188286.2	CCDC7	mRNA	XR_001021689.2	lncRNA
XM_012188286.2	CCDC7	mRNA	XR_001021689.2	lncRNA
XM_004002227.3	SLC35A3	mRNA	XR_001021689.2	lncRNA
XM_004002227.3	SLC35A3	mRNA	XR_001021689.2	lncRNA
XM_004002227.3	SLC35A3	mRNA	XR_001021689.2	lncRNA
XM_004004723.3	SPOPL	mRNA	XR_001021689.2	lncRNA
XM_004004723.3	SPOPL	mRNA	XR_001021689.2	lncRNA
XM_004004723.3	SPOPL	mRNA	XR_001021689.2	lncRNA
XM_012100861.1	FBXO9	mRNA	XR_001021689.2	lncRNA
XM_012100861.1	FBXO9	mRNA	XR_001021689.2	lncRNA
XM_012100861.1	FBXO9	mRNA	XR_001021689.2	lncRNA
XM_015093291.1	NIPA2	mRNA	XR_001021689.2	lncRNA
XM_015093291.1	NIPA2	mRNA	XR_001021689.2	lncRNA
XM_015093291.1	NIPA2	mRNA	XR_001021689.2	lncRNA
XM_012178872.2	AP4B1	mRNA	XR_001021689.2	lncRNA
XM_012178872.2	AP4B1	mRNA	XR_001021689.2	lncRNA
XM_012178872.2	AP4B1	mRNA	XR_001021689.2	lncRNA
XM_012187240.2	SLC19A2	mRNA	XR_001021689.2	lncRNA
XM_012187240.2	SLC19A2	mRNA	XR_001021689.2	lncRNA
XM_012187240.2	SLC19A2	mRNA	XR_001021689.2	lncRNA
XM_012099058.2	PPP2R5C	mRNA	XR_001041211.2	lncRNA
XM_012099058.2	PPP2R5C	mRNA	XR_001041211.2	lncRNA
XM_012099058.2	PPP2R5C	mRNA	XR_001041211.2	lncRNA
XM_012100729.1	POLH	mRNA	TCONS_00029399	lncRNA
XM_012100729.1	POLH	mRNA	TCONS_00029399	lncRNA
XM_012100729.1	POLH	mRNA	TCONS_00029399	lncRNA
XM_015097223.1	ZC3H14	mRNA	XR_001041209.2	lncRNA
XM_015097223.1	ZC3H14	mRNA	XR_001041209.2	lncRNA
XM_015097223.1	ZC3H14	mRNA	XR_001041209.2	lncRNA
XM_012179621.1	DNAJB14	mRNA	TCONS_00029399	lncRNA
XM_012179621.1	DNAJB14	mRNA	TCONS_00029399	lncRNA
XM_012179621.1	DNAJB14	mRNA	TCONS_00029399	lncRNA
XM_012179621.1	DNAJB14	mRNA	XR_001041209.2	lncRNA
XM_012179621.1	DNAJB14	mRNA	XR_001041209.2	lncRNA
XM_012179621.1	DNAJB14	mRNA	XR_001041209.2	lncRNA
XM_012184453.2	FNDC3A	mRNA	XR_001041211.2	lncRNA
XM_012184453.2	FNDC3A	mRNA	XR_001041211.2	lncRNA
XM_012184453.2	FNDC3A	mRNA	XR_001041211.2	lncRNA
XM_012100729.1	POLH	mRNA	XR_001041209.2	lncRNA
XM_012100729.1	POLH	mRNA	XR_001041209.2	lncRNA
XM_012100729.1	POLH	mRNA	XR_001041209.2	lncRNA
XM_015097326.1	TBC1D32	mRNA	TCONS_00029399	lncRNA
XM_015097326.1	TBC1D32	mRNA	TCONS_00029399	lncRNA
XM_015097326.1	TBC1D32	mRNA	TCONS_00029399	lncRNA
XM_012097771.2	ORAI1	mRNA	XR_001041209.2	lncRNA
XM_012097771.2	ORAI1	mRNA	XR_001041209.2	lncRNA
XM_012097771.2	ORAI1	mRNA	XR_001041209.2	lncRNA
XM_015102413.1	RBM6	mRNA	XR_001041209.2	lncRNA
XM_015102413.1	RBM6	mRNA	XR_001041209.2	lncRNA

XM_015102413.1	RBM6	mRNA	XR_001041209.2	lncRNA
XM_004016017.3	C15H11orf1	mRNA	XR_001041211.2	lncRNA
XM_004016017.3	C15H11orf1	mRNA	XR_001041211.2	lncRNA
XM_004016017.3	C15H11orf1	mRNA	XR_001041211.2	lncRNA
XM_012175064.2	KIAA1551	mRNA	XR_001041209.2	lncRNA
XM_012175064.2	KIAA1551	mRNA	XR_001041209.2	lncRNA
XM_012175064.2	KIAA1551	mRNA	XR_001041209.2	lncRNA
XM_012106614.1	RRAGB	mRNA	TCONS_00029398	lncRNA
XM_012106614.1	RRAGB	mRNA	TCONS_00029398	lncRNA
XM_012106614.1	RRAGB	mRNA	TCONS_00029398	lncRNA
XM_015092786.1	LOC1069908[illegible]	mRNA	TCONS_00029398	lncRNA
XM_015092786.1	LOC1069908[illegible]	mRNA	TCONS_00029398	lncRNA
XM_015092786.1	LOC1069908[illegible]	mRNA	TCONS_00029398	lncRNA
XM_012095272.2	ARNTL	mRNA	TCONS_00029398	lncRNA
XM_012095272.2	ARNTL	mRNA	TCONS_00029398	lncRNA
XM_012095272.2	ARNTL	mRNA	TCONS_00029398	lncRNA
XM_012187919.2	LOC10561661	mRNA	TCONS_00029398	lncRNA
XM_012187919.2	LOC10561661	mRNA	TCONS_00029398	lncRNA
XM_012187919.2	LOC10561661	mRNA	TCONS_00029398	lncRNA
XM_012182301.2	LRRFIP1	mRNA	XR_001039281.1	lncRNA
XM_012182301.2	LRRFIP1	mRNA	XR_001039281.1	lncRNA
XM_012182301.2	LRRFIP1	mRNA	XR_001039281.1	lncRNA
XM_012129647.1	NEK1	mRNA	TCONS_00072289	lncRNA
XM_012129647.1	NEK1	mRNA	TCONS_00072289	lncRNA
XM_012129647.1	NEK1	mRNA	TCONS_00072289	lncRNA
XM_004002409.3	PDZK1	mRNA	TCONS_00072289	lncRNA
XM_004002409.3	PDZK1	mRNA	TCONS_00072289	lncRNA
XM_004002409.3	PDZK1	mRNA	TCONS_00072289	lncRNA
XM_012182301.2	LRRFIP1	mRNA	TCONS_00072289	lncRNA
XM_012182301.2	LRRFIP1	mRNA	TCONS_00072289	lncRNA
XM_012182301.2	LRRFIP1	mRNA	TCONS_00072289	lncRNA
XM_012176678.2	KMT2E	mRNA	XR_001039281.1	lncRNA
XM_012176678.2	KMT2E	mRNA	XR_001039281.1	lncRNA
XM_012176678.2	KMT2E	mRNA	XR_001039281.1	lncRNA
XM_012103723.1	POLI	mRNA	XR_001039281.1	lncRNA
XM_012103723.1	POLI	mRNA	XR_001039281.1	lncRNA
XM_012103723.1	POLI	mRNA	XR_001039281.1	lncRNA
XM_012174607.2	HELB	mRNA	TCONS_00072289	lncRNA
XM_012174607.2	HELB	mRNA	TCONS_00072289	lncRNA
XM_012174607.2	HELB	mRNA	TCONS_00072289	lncRNA
XM_015102413.1	RBM6	mRNA	XR_001434081.1	lncRNA
XM_015102413.1	RBM6	mRNA	XR_001434081.1	lncRNA
XM_015102413.1	RBM6	mRNA	XR_001434081.1	lncRNA
XM_004001901.2	ERI3	mRNA	XR_001434081.1	lncRNA
XM_004001901.2	ERI3	mRNA	XR_001434081.1	lncRNA
XM_004001901.2	ERI3	mRNA	XR_001434081.1	lncRNA
XM_012106742.2	PQBP1	mRNA	XR_001434081.1	lncRNA
XM_012106742.2	PQBP1	mRNA	XR_001434081.1	lncRNA
XM_012106742.2	PQBP1	mRNA	XR_001434081.1	lncRNA
XM_012182520.2	SLC35F1	mRNA	XR_001434081.1	lncRNA
XM_012182520.2	SLC35F1	mRNA	XR_001434081.1	lncRNA
XM_012182520.2	SLC35F1	mRNA	XR_001434081.1	lncRNA
XM_012105248.1	ASCC1	mRNA	XR_001434081.1	lncRNA
XM_012105248.1	ASCC1	mRNA	XR_001434081.1	lncRNA

XM_012105248.1	ASCC1	mRNA	XR_001434081.1	lncRNA
XM_012182825.2	SNX14	mRNA	XR_001041202.1	lncRNA
XM_012182825.2	SNX14	mRNA	XR_001041202.1	lncRNA
XM_012182825.2	SNX14	mRNA	XR_001041202.1	lncRNA
XM_012181420.2	ICE2	mRNA	TCONS_00078442	lncRNA
XM_012181420.2	ICE2	mRNA	TCONS_00078442	lncRNA
XM_012181420.2	ICE2	mRNA	TCONS_00078442	lncRNA
XM_004006510.3	SLC16A7	mRNA	TCONS_00078442	lncRNA
XM_004006510.3	SLC16A7	mRNA	TCONS_00078442	lncRNA
XM_004006510.3	SLC16A7	mRNA	TCONS_00078442	lncRNA
XM_015096814.1	DENND4A	mRNA	TCONS_00078442	lncRNA
XM_015096814.1	DENND4A	mRNA	TCONS_00078442	lncRNA
XM_015096814.1	DENND4A	mRNA	TCONS_00078442	lncRNA
XM_015093495.1	NBEAL1	mRNA	TCONS_00078442	lncRNA
XM_015093495.1	NBEAL1	mRNA	TCONS_00078442	lncRNA
XM_015093495.1	NBEAL1	mRNA	TCONS_00078442	lncRNA
XM_012098990.2	NAALADL2	mRNA	TCONS_00078442	lncRNA
XM_012098990.2	NAALADL2	mRNA	TCONS_00078442	lncRNA
XM_012098990.2	NAALADL2	mRNA	TCONS_00078442	lncRNA
XM_012182215.2	TDP1	mRNA	TCONS_00078442	lncRNA
XM_012182215.2	TDP1	mRNA	TCONS_00078442	lncRNA
XM_012182215.2	TDP1	mRNA	TCONS_00078442	lncRNA
XM_012188685.2	NIT2	mRNA	XR_001021939.1	lncRNA
XM_012188685.2	NIT2	mRNA	XR_001021939.1	lncRNA
XM_012188685.2	NIT2	mRNA	XR_001021939.1	lncRNA
XM_012103008.2	FAM45A	mRNA	XR_001021939.1	lncRNA
XM_012103008.2	FAM45A	mRNA	XR_001021939.1	lncRNA
XM_012103008.2	FAM45A	mRNA	XR_001021939.1	lncRNA
XM_012107383.2	XIAP	mRNA	XR_001033284.2	lncRNA
XM_012107383.2	XIAP	mRNA	XR_001033284.2	lncRNA
XM_012107383.2	XIAP	mRNA	XR_001033284.2	lncRNA
XM_015098766.1	WIPF2	mRNA	XR_001033284.2	lncRNA
XM_015098766.1	WIPF2	mRNA	XR_001033284.2	lncRNA
XM_015098766.1	WIPF2	mRNA	XR_001033284.2	lncRNA
XM_015104027.1	PDPK1	mRNA	TCONS_00071561	lncRNA
XM_015104027.1	PDPK1	mRNA	TCONS_00071561	lncRNA
XM_015104027.1	PDPK1	mRNA	TCONS_00071561	lncRNA
XM_012096744.2	GFM2	mRNA	XR_001021689.2	lncRNA
XM_012096744.2	GFM2	mRNA	XR_001021689.2	lncRNA
XM_012096744.2	GFM2	mRNA	XR_001021689.2	lncRNA
XM_012145933.2	CXCR2	mRNA	XR_001021689.2	lncRNA
XM_012145933.2	CXCR2	mRNA	XR_001021689.2	lncRNA
XM_012145933.2	CXCR2	mRNA	XR_001021689.2	lncRNA
XM_012184485.2	RNASEH2B	mRNA	TCONS_00071561	lncRNA
XM_012184485.2	RNASEH2B	mRNA	TCONS_00071561	lncRNA
XM_012184485.2	RNASEH2B	mRNA	TCONS_00071561	lncRNA
XM_015102735.1	SUPT3H	mRNA	TCONS_00071561	lncRNA
XM_015102735.1	SUPT3H	mRNA	TCONS_00071561	lncRNA
XM_015102735.1	SUPT3H	mRNA	TCONS_00071561	lncRNA
XM_012189374.1	NXPE3	mRNA	TCONS_00071561	lncRNA
XM_012189374.1	NXPE3	mRNA	TCONS_00071561	lncRNA
XM_012189374.1	NXPE3	mRNA	TCONS_00071561	lncRNA
XM_015097359.1	ARMC2	mRNA	TCONS_00071561	lncRNA
XM_015097359.1	ARMC2	mRNA	TCONS_00071561	lncRNA

XM_015097359.1	ARMC2	mRNA	TCONS_00071561	lncRNA
XM_012185602.2	ALOX12	mRNA	TCONS_00071561	lncRNA
XM_012185602.2	ALOX12	mRNA	TCONS_00071561	lncRNA
XM_012185602.2	ALOX12	mRNA	TCONS_00071561	lncRNA
XM_012104222.2	DCUN1D3	mRNA	XR_001021689.2	lncRNA
XM_012104222.2	DCUN1D3	mRNA	XR_001021689.2	lncRNA
XM_012104222.2	DCUN1D3	mRNA	XR_001021689.2	lncRNA
XM_012184485.2	RNASEH2B	mRNA	XR_001021689.2	lncRNA
XM_012184485.2	RNASEH2B	mRNA	XR_001021689.2	lncRNA
XM_012184485.2	RNASEH2B	mRNA	XR_001021689.2	lncRNA
XM_012188288.2	ARL5B	mRNA	TCONS_00072289	lncRNA
XM_012188288.2	ARL5B	mRNA	TCONS_00072289	lncRNA
XM_012188288.2	ARL5B	mRNA	TCONS_00072289	lncRNA
XM_015099256.1	CEP104	mRNA	TCONS_00072289	lncRNA
XM_015099256.1	CEP104	mRNA	TCONS_00072289	lncRNA
XM_015099256.1	CEP104	mRNA	TCONS_00072289	lncRNA
XM_015092207.1	PRKAB2	mRNA	XR_001039281.1	lncRNA
XM_015092207.1	PRKAB2	mRNA	XR_001039281.1	lncRNA
XM_015092207.1	PRKAB2	mRNA	XR_001039281.1	lncRNA
XM_015097326.1	TBC1D32	mRNA	XR_001436609.1	lncRNA
XM_015097326.1	TBC1D32	mRNA	XR_001436609.1	lncRNA
XM_015097326.1	TBC1D32	mRNA	XR_001436609.1	lncRNA
XM_012100993.2	FLOT1	mRNA	XR_001436531.1	lncRNA
XM_012100993.2	FLOT1	mRNA	XR_001436531.1	lncRNA
XM_012100993.2	FLOT1	mRNA	XR_001436531.1	lncRNA
XM_012097771.2	ORAI1	mRNA	XR_001435104.1	lncRNA
XM_012097771.2	ORAI1	mRNA	XR_001435104.1	lncRNA
XM_012097771.2	ORAI1	mRNA	XR_001435104.1	lncRNA
XM_015097223.1	ZC3H14	mRNA	XR_001435104.1	lncRNA
XM_015097223.1	ZC3H14	mRNA	XR_001435104.1	lncRNA
XM_015097223.1	ZC3H14	mRNA	XR_001435104.1	lncRNA
XM_012097771.2	ORAI1	mRNA	XR_001436609.1	lncRNA
XM_012097771.2	ORAI1	mRNA	XR_001436609.1	lncRNA
XM_012097771.2	ORAI1	mRNA	XR_001436609.1	lncRNA
XM_012115851.2	PTPDC1	mRNA	XR_001435104.1	lncRNA
XM_012115851.2	PTPDC1	mRNA	XR_001435104.1	lncRNA
XM_012115851.2	PTPDC1	mRNA	XR_001435104.1	lncRNA
XM_015097252.1	SLC17A5	mRNA	TCONS_00029398	lncRNA
XM_015097252.1	SLC17A5	mRNA	TCONS_00029398	lncRNA
XM_015097252.1	SLC17A5	mRNA	TCONS_00029398	lncRNA
XM_015104785.1	TBL1X	mRNA	TCONS_00029398	lncRNA
XM_015104785.1	TBL1X	mRNA	TCONS_00029398	lncRNA
XM_015104785.1	TBL1X	mRNA	TCONS_00029398	lncRNA
XM_015096551.1	MOB1B	mRNA	TCONS_00029398	lncRNA
XM_015096551.1	MOB1B	mRNA	TCONS_00029398	lncRNA
XM_015096551.1	MOB1B	mRNA	TCONS_00029398	lncRNA
XM_004012898.3	KRT34	mRNA	TCONS_00029399	lncRNA
XM_004012898.3	KRT34	mRNA	TCONS_00029399	lncRNA
XM_004012898.3	KRT34	mRNA	TCONS_00029399	lncRNA
XM_012100555.2	PNPLA1	mRNA	XR_001041209.2	lncRNA
XM_012100555.2	PNPLA1	mRNA	XR_001041209.2	lncRNA
XM_012100555.2	PNPLA1	mRNA	XR_001041209.2	lncRNA
XM_012096215.2	EHF	mRNA	XR_001041211.2	lncRNA
XM_012096215.2	EHF	mRNA	XR_001041211.2	lncRNA

XM_012096215.2	EHF	mRNA	XR_001041211.2	lncRNA
XM_015103753.1	MBD1	mRNA	XR_001041209.2	lncRNA
XM_015103753.1	MBD1	mRNA	XR_001041209.2	lncRNA
XM_015103753.1	MBD1	mRNA	XR_001041209.2	lncRNA
XM_012119517.2	SORBS3	mRNA	XR_001041209.2	lncRNA
XM_012119517.2	SORBS3	mRNA	XR_001041209.2	lncRNA
XM_012119517.2	SORBS3	mRNA	XR_001041209.2	lncRNA
XM_012188286.2	CCDC7	mRNA	TCONS_00029399	lncRNA
XM_012188286.2	CCDC7	mRNA	TCONS_00029399	lncRNA
XM_012188286.2	CCDC7	mRNA	TCONS_00029399	lncRNA
XM_012188286.2	CCDC7	mRNA	XR_001041209.2	lncRNA
XM_012188286.2	CCDC7	mRNA	XR_001041209.2	lncRNA
XM_012188286.2	CCDC7	mRNA	XR_001041209.2	lncRNA
XM_012095465.2	GOLGB1	mRNA	TCONS_00078442	lncRNA
XM_012095465.2	GOLGB1	mRNA	TCONS_00078442	lncRNA
XM_012095465.2	GOLGB1	mRNA	TCONS_00078442	lncRNA
XM_012137673.2	NEB	mRNA	TCONS_00078442	lncRNA
XM_012137673.2	NEB	mRNA	TCONS_00078442	lncRNA
XM_012137673.2	NEB	mRNA	TCONS_00078442	lncRNA
XM_015096769.1	APC	mRNA	TCONS_00078442	lncRNA
XM_015096769.1	APC	mRNA	TCONS_00078442	lncRNA
XM_015096769.1	APC	mRNA	TCONS_00078442	lncRNA
XM_012104178.2	CCP110	mRNA	TCONS_00078442	lncRNA
XM_012104178.2	CCP110	mRNA	TCONS_00078442	lncRNA
XM_012104178.2	CCP110	mRNA	TCONS_00078442	lncRNA
XM_012097771.2	ORAI1	mRNA	XR_001435726.1	lncRNA
XM_012097771.2	ORAI1	mRNA	XR_001435726.1	lncRNA
XM_012097771.2	ORAI1	mRNA	XR_001435726.1	lncRNA
XM_015097223.1	ZC3H14	mRNA	XR_001042381.2	lncRNA
XM_015097223.1	ZC3H14	mRNA	XR_001042381.2	lncRNA
XM_015097223.1	ZC3H14	mRNA	XR_001042381.2	lncRNA
XM_015102528.1	FLOT1	mRNA	XR_001041202.1	lncRNA
XM_015102528.1	FLOT1	mRNA	XR_001041202.1	lncRNA
XM_015102528.1	FLOT1	mRNA	XR_001041202.1	lncRNA
XM_012106024.2	HOOK3	mRNA	XR_001041202.1	lncRNA
XM_012106024.2	HOOK3	mRNA	XR_001041202.1	lncRNA
XM_012106024.2	HOOK3	mRNA	XR_001041202.1	lncRNA
XM_015101107.1	TCP11L1	mRNA	XR_001039281.1	lncRNA
XM_015101107.1	TCP11L1	mRNA	XR_001039281.1	lncRNA
XM_015101107.1	TCP11L1	mRNA	XR_001039281.1	lncRNA
XM_012181829.2	ZBTB1	mRNA	TCONS_00072289	lncRNA
XM_012181829.2	ZBTB1	mRNA	TCONS_00072289	lncRNA
XM_012181829.2	ZBTB1	mRNA	TCONS_00072289	lncRNA
XM_004004099.3	GOLM1	mRNA	XR_001434081.1	lncRNA
XM_004004099.3	GOLM1	mRNA	XR_001434081.1	lncRNA
XM_004004099.3	GOLM1	mRNA	XR_001434081.1	lncRNA
XM_015098611.1	DLG4	mRNA	XR_001434081.1	lncRNA
XM_015098611.1	DLG4	mRNA	XR_001434081.1	lncRNA
XM_015098611.1	DLG4	mRNA	XR_001434081.1	lncRNA
XM_012115168.2	LOC1011236(	mRNA	XR_001434081.1	lncRNA
XM_012115168.2	LOC1011236(	mRNA	XR_001434081.1	lncRNA
XM_012115168.2	LOC1011236(	mRNA	XR_001434081.1	lncRNA
XM_012187340.2	KIF1B	mRNA	TCONS_00078442	lncRNA
XM_012187340.2	KIF1B	mRNA	TCONS_00078442	lncRNA

XM_012187340.2	KIF1B	mRNA	TCONS_00078442	lncRNA
XM_015091887.1	MYCL	mRNA	TCONS_00078442	lncRNA
XM_015091887.1	MYCL	mRNA	TCONS_00078442	lncRNA
XM_015091887.1	MYCL	mRNA	TCONS_00078442	lncRNA
XM_015102914.1	NAV2	mRNA	TCONS_00078442	lncRNA
XM_015102914.1	NAV2	mRNA	TCONS_00078442	lncRNA
XM_015102914.1	NAV2	mRNA	TCONS_00078442	lncRNA
XM_015097252.1	SLC17A5	mRNA	TCONS_00071561	lncRNA
XM_015097252.1	SLC17A5	mRNA	TCONS_00071561	lncRNA
XM_015097252.1	SLC17A5	mRNA	TCONS_00071561	lncRNA
XM_015104131.1	TNRC6A	mRNA	TCONS_00071561	lncRNA
XM_015104131.1	TNRC6A	mRNA	TCONS_00071561	lncRNA
XM_015104131.1	TNRC6A	mRNA	TCONS_00071561	lncRNA
XM_012105564.2	ZFAND4	mRNA	XR_001021689.2	lncRNA
XM_012105564.2	ZFAND4	mRNA	XR_001021689.2	lncRNA
XM_012105564.2	ZFAND4	mRNA	XR_001021689.2	lncRNA
XM_012161674.1	LRRC8A	mRNA	TCONS_00071561	lncRNA
XM_012161674.1	LRRC8A	mRNA	TCONS_00071561	lncRNA
XM_012161674.1	LRRC8A	mRNA	TCONS_00071561	lncRNA
XM_012107777.1	NFYC	mRNA	XR_001033284.2	lncRNA
XM_012107777.1	NFYC	mRNA	XR_001033284.2	lncRNA
XM_012107777.1	NFYC	mRNA	XR_001033284.2	lncRNA
XM_012188664.2	RASSF2	mRNA	XR_001033284.2	lncRNA
XM_012188664.2	RASSF2	mRNA	XR_001033284.2	lncRNA
XM_012188664.2	RASSF2	mRNA	XR_001033284.2	lncRNA
XM_015093632.1	EPB41	mRNA	TCONS_00072289	lncRNA
XM_015093632.1	EPB41	mRNA	TCONS_00072289	lncRNA
XM_015093632.1	EPB41	mRNA	TCONS_00072289	lncRNA
XM_012105556.2	ZFAND4	mRNA	XR_001021939.1	lncRNA
XM_012105556.2	ZFAND4	mRNA	XR_001021939.1	lncRNA
XM_012105556.2	ZFAND4	mRNA	XR_001021939.1	lncRNA
XM_012100587.2	BTBD9	mRNA	TCONS_00078442	lncRNA
XM_012100587.2	BTBD9	mRNA	TCONS_00078442	lncRNA
XM_012100587.2	BTBD9	mRNA	TCONS_00078442	lncRNA
XM_015105047.1	XIAP	mRNA	TCONS_00029399	lncRNA
XM_015105047.1	XIAP	mRNA	TCONS_00029399	lncRNA
XM_015105047.1	XIAP	mRNA	TCONS_00029399	lncRNA
XM_012095387.2	ELMOD1	mRNA	XR_001041209.2	lncRNA
XM_012095387.2	ELMOD1	mRNA	XR_001041209.2	lncRNA
XM_012095387.2	ELMOD1	mRNA	XR_001041209.2	lncRNA
XM_012140422.2	CLASP1	mRNA	XR_001039281.1	lncRNA
XM_012140422.2	CLASP1	mRNA	XR_001039281.1	lncRNA
XM_012140422.2	CLASP1	mRNA	XR_001039281.1	lncRNA
XM_012188286.2	CCDC7	mRNA	XR_001435104.1	lncRNA
XM_012188286.2	CCDC7	mRNA	XR_001435104.1	lncRNA
XM_012188286.2	CCDC7	mRNA	XR_001435104.1	lncRNA
XM_012188068.2	ARFRP1	mRNA	XR_001436531.1	lncRNA
XM_012188068.2	ARFRP1	mRNA	XR_001436531.1	lncRNA
XM_012188068.2	ARFRP1	mRNA	XR_001436531.1	lncRNA
XM_012105556.2	ZFAND4	mRNA	XR_001436609.1	lncRNA
XM_012105556.2	ZFAND4	mRNA	XR_001436609.1	lncRNA
XM_012105556.2	ZFAND4	mRNA	XR_001436609.1	lncRNA
XM_012107383.2	XIAP	mRNA	XR_001435104.1	lncRNA
XM_012107383.2	XIAP	mRNA	XR_001435104.1	lncRNA

XM_012107383.2	XIAP	mRNA	XR_001435104.1	lncRNA
XM_012105556.2	ZFAND4	mRNA	XR_001435104.1	lncRNA
XM_012105556.2	ZFAND4	mRNA	XR_001435104.1	lncRNA
XM_012105556.2	ZFAND4	mRNA	XR_001435104.1	lncRNA
XM_004006434.3	PUS7L	mRNA	TCONS_00071561	lncRNA
XM_004006434.3	PUS7L	mRNA	TCONS_00071561	lncRNA
XM_004006434.3	PUS7L	mRNA	TCONS_00071561	lncRNA
XM_004008093.2	PTN	mRNA	XR_001021689.2	lncRNA
XM_004008093.2	PTN	mRNA	XR_001021689.2	lncRNA
XM_004008093.2	PTN	mRNA	XR_001021689.2	lncRNA
XM_012188286.2	CCDC7	mRNA	XR_001435723.1	lncRNA
XM_012188286.2	CCDC7	mRNA	XR_001435723.1	lncRNA
XM_012188286.2	CCDC7	mRNA	XR_001435723.1	lncRNA
XM_012095387.2	ELMOD1	mRNA	XR_001434081.1	lncRNA
XM_012095387.2	ELMOD1	mRNA	XR_001434081.1	lncRNA
XM_012095387.2	ELMOD1	mRNA	XR_001434081.1	lncRNA
XM_015104131.1	TNRC6A	mRNA	XR_001033284.2	lncRNA
XM_015104131.1	TNRC6A	mRNA	XR_001033284.2	lncRNA
XM_015104131.1	TNRC6A	mRNA	XR_001033284.2	lncRNA
XM_004006165.3	ITPRIPL1	mRNA	TCONS_00029398	lncRNA
XM_004006165.3	ITPRIPL1	mRNA	TCONS_00029398	lncRNA
XM_004006165.3	ITPRIPL1	mRNA	TCONS_00029398	lncRNA
XM_012105333.2	USP54	mRNA	XR_001021689.2	lncRNA
XM_012105333.2	USP54	mRNA	XR_001021689.2	lncRNA
XM_012105333.2	USP54	mRNA	XR_001021689.2	lncRNA
XM_004012689.3	MYH10	mRNA	TCONS_00029399	lncRNA
XM_004012689.3	MYH10	mRNA	TCONS_00029399	lncRNA
XM_004012689.3	MYH10	mRNA	TCONS_00029399	lncRNA
